# Supplementary material for: A genome-wide CRISPR knockout screen identified host genes essential for Brucella invasion and intracellular survival
Source: Emerg Microbes Infect. 2026 Aug 19;15(1):2713322. doi: 10.1080/22221751.2026.2713322 (PMC13491901; doi:10.1080/22221751.2026.2713322)
Supplement: Supplementary file 1 [file TEMI_A_2713322_SM6411.pdf]

| LOC100505679 | HGLibA_26852 | 57 | -0.619108944 | LOC100505679 | - |
| SMEK1 | HGLibA_45692 | 57 | -0.619108944 | SMEK1 | - |
| XAGE1D | HGLibA_54474 | 57 | -0.619108944 | XAGE1D | - |
| ATP5J2 | HGLibA_03672 | 56 | -0.628154287 | ATP5J2 | - |
| C22orf29 | HGLibA_06069 | 56 | -0.628154287 | C22orf29 | - |
| C3orf43 | HGLibA_06244 | 56 | -0.628154287 | C3orf43 | - |
| EMR2 | HGLibA_15085 | 56 | -0.628154287 | EMR2 | - |
| ENSG00000004939 | HGLibA_45201 | 56 | -0.628154287 | SLC4A1 | solute carrier family 4 member 1 (Diego blood group) [Source:HGNC Symbol;Acc:HGNC:11027] |
| ENSG00000005007 | HGLibA_53055 | 56 | -0.628154287 | UPF1 | RNA helicase and ATPase [Source:HGNC Symbol;Acc:HGNC:9962]" |
| ENSG00000005700 | HGLibA_22486 | 56 | -0.628154287 | IBTK | inhibitor of Bruton tyrosine kinase [Source:HGNC Symbol;Acc:HGNC:17853] |
| ENSG00000006607 | HGLibA_16878 | 56 | -0.628154287 | FARP2 | ARH/RhoGEF and pleckstrin domain protein 2 [Source:HGNC Symbol;Acc:HGNC:16460]" |
| ENSG00000006607 | HGLibA_16877 | 56 | -0.628154287 | FARP2 | ARH/RhoGEF and pleckstrin domain protein 2 [Source:HGNC Symbol;Acc:HGNC:16460]" |
| ENSG00000010322 | HGLibA_31979 | 56 | -0.628154287 | NISCH | nischarin [Source:HGNC Symbol;Acc:HGNC:18006] |
| ENSG00000010803 | HGLibA_42955 | 56 | -0.628154287 | SCMH1 | Scm polycomb group protein homolog 1 [Source:HGNC Symbol;Acc:HGNC:19003] |
| ENSG00000013503 | HGLibA_37612 | 56 | -0.628154287 | POLR3B | RNA polymerase III subunit B [Source:HGNC Symbol;Acc:HGNC:30348] |
| ENSG00000023902 | HGLibA_37150 | 56 | -0.628154287 | PLEKHO1 | pleckstrin homology domain containing O1 [Source:HGNC Symbol;Acc:HGNC:24310] |
| ENSG00000024048 | HGLibA_52749 | 56 | -0.628154287 | UBR2 | ubiquitin protein ligase E3 component n-recognin 2 [Source:HGNC Symbol;Acc:HGNC:21289] |
| ENSG00000029364 | HGLibA_45127 | 56 | -0.628154287 | SLC39A9 | solute carrier family 39 member 9 [Source:HGNC Symbol;Acc:HGNC:20182] |
| ENSG00000056097 | HGLibA_55259 | 56 | -0.628154287 | ZFR | zinc finger RNA binding protein [Source:HGNC Symbol;Acc:HGNC:17277] |
| ENSG00000059758 | HGLibA_08746 | 56 | -0.628154287 | CDK17 | cyclin dependent kinase 17 [Source:HGNC Symbol;Acc:HGNC:8750] |
| ENSG00000065621 | HGLibA_20490 | 56 | -0.628154287 | GSTO2 | glutathione S-transferase omega 2 [Source:HGNC Symbol;Acc:HGNC:23064] |
| ENSG00000065883 | HGLibA_08735 | 56 | -0.628154287 | CDK13 | cyclin dependent kinase 13 [Source:HGNC Symbol;Acc:HGNC:1733] |
| ENSG00000066933 | HGLibA_30869 | 56 | -0.628154287 | MYO9A | myosin IXA [Source:HGNC Symbol;Acc:HGNC:7608] |
| ENSG00000067221 | HGLibA_47534 | 56 | -0.628154287 | STOML1 | stomatin like 1 [Source:HGNC Symbol;Acc:HGNC:14560] |
| ENSG00000069188 | HGLibA_43153 | 56 | -0.628154287 | SDK2 | sidekick cell adhesion molecule 2 [Source:HGNC Symbol;Acc:HGNC:19308] |
| ENSG00000070423 | HGLibA_41495 | 56 | -0.628154287 | RNF126 | ring finger protein 126 [Source:HGNC Symbol;Acc:HGNC:21151] |
| ENSG00000071127 | HGLibA_54022 | 56 | -0.628154287 | WDR1 | WD repeat domain 1 [Source:HGNC Symbol;Acc:HGNC:12754] |
| ENSG00000073969 | HGLibA_32752 | 56 | -0.628154287 | NSF | vesicle fusing ATPase [Source:HGNC Symbol;Acc:HGNC:8016]" |
| ENSG00000082068 | HGLibA_54164 | 56 | -0.628154287 | WDR70 | WD repeat domain 70 [Source:HGNC Symbol;Acc:HGNC:25495] |
| ENSG00000083845 | HGLibA_42151 | 56 | -0.628154287 | RPS5 | ribosomal protein S5 [Source:HGNC Symbol;Acc:HGNC:10426] |
| ENSG00000085760 | HGLibA_30397 | 56 | -0.628154287 | MTIF2 | mitochondrial translational initiation factor 2 [Source:HGNC Symbol;Acc:HGNC:7441] |
| ENSG00000086205 | HGLibA_17780 | 56 | -0.628154287 | FOLH1 | folate hydrolase 1 [Source:HGNC Symbol;Acc:HGNC:3788] |
| ENSG00000089250 | HGLibA_32323 | 56 | -0.628154287 | NOS1 | nitric oxide synthase 1 [Source:HGNC Symbol;Acc:HGNC:7872] |
| ENSG00000090776 | HGLibA_14596 | 56 | -0.628154287 | EFNB1 | ephrin B1 [Source:HGNC Symbol;Acc:HGNC:3226] |
| ENSG00000091164 | HGLibA_52434 | 56 | -0.628154287 | TXNL1 | thioredoxin like 1 [Source:HGNC Symbol;Acc:HGNC:12436] |
| ENSG00000099341 | HGLibA_39184 | 56 | -0.628154287 | PSMD8 | non-ATPase 8 [Source:HGNC Symbol;Acc:HGNC:9566]" |
| ENSG00000099622 | HGLibA_09721 | 56 | -0.628154287 | CIRBP | cold inducible RNA binding protein [Source:HGNC Symbol;Acc:HGNC:1982] |
| ENSG00000100106 | HGLibA_51631 | 56 | -0.628154287 | TRIOBP | TRIO and F-actin binding protein [Source:HGNC Symbol;Acc:HGNC:17009] |
| ENSG00000101000 | HGLibA_38655 | 56 | -0.628154287 | PROCR | protein C receptor [Source:HGNC Symbol;Acc:HGNC:9452] |
| ENSG00000101255 | HGLibA_51397 | 56 | -0.628154287 | TRIB3 | tribbles pseudokinase 3 [Source:HGNC Symbol;Acc:HGNC:16228] |
| ENSG00000101882 | HGLibA_32002 | 56 | -0.628154287 | NKAP | NFKB activating protein [Source:HGNC Symbol;Acc:HGNC:29873] |
| ENSG00000101977 | HGLibA_28577 | 56 | -0.628154287 | MCF2 | MCF.2 cell line derived transforming sequence [Source:HGNC Symbol;Acc:HGNC:6940] |
| ENSG00000102312 | HGLibA_37708 | 56 | -0.628154287 | PORCN | porcupine O-acyltransferase [Source:HGNC Symbol;Acc:HGNC:17652] |
| ENSG00000103512 | HGLibA_32289 | 56 | -0.628154287 | NOMO1 | NODAL modulator 1 [Source:HGNC Symbol;Acc:HGNC:30060] |
| ENSG00000104324 | HGLibA_10987 | 56 | -0.628154287 | CPQ | carboxypeptidase Q [Source:HGNC Symbol;Acc:HGNC:16910] |
| ENSG00000104419 | HGLibA_31412 | 56 | -0.628154287 | NDRG1 | N-myc downstream regulated 1 [Source:HGNC Symbol;Acc:HGNC:7679] |
| ENSG00000104884 | HGLibA_15435 | 56 | -0.628154287 | ERCC2 | TFIIH core complex helicase subunit [Source:HGNC Symbol;Acc:HGNC:3434]" |
| ENSG00000106052 | HGLibA_48449 | 56 | -0.628154287 | TAX1BP1 | Tax1 binding protein 1 [Source:HGNC Symbol;Acc:HGNC:11575] |
| ENSG00000106245 | HGLibA_05001 | 56 | -0.628154287 | BUD31 | BUD31 homolog [Source:HGNC Symbol;Acc:HGNC:29629] |
| ENSG00000108379 | HGLibA_54363 | 56 | -0.628154287 | WNT3 | Wnt family member 3 [Source:HGNC Symbol;Acc:HGNC:12782] |
| ENSG00000109163 | HGLibA_19585 | 56 | -0.628154287 | GNRHR | gonadotropin releasing hormone receptor [Source:HGNC Symbol;Acc:HGNC:4421] |
| ENSG00000110048 | HGLibA_34636 | 56 | -0.628154287 | OSBP | oxysterol binding protein [Source:HGNC Symbol;Acc:HGNC:8503] |
| ENSG00000110245 | HGLibA_02509 | 56 | -0.628154287 | APOC3 | apolipoprotein C3 [Source:HGNC Symbol;Acc:HGNC:610] |
| ENSG00000110344 | HGLibA_52691 | 56 | -0.628154287 | UBE4A | ubiquitination factor E4A [Source:HGNC Symbol;Acc:HGNC:12499] |
| ENSG00000110911 | HGLibA_44353 | 56 | -0.628154287 | SLC11A2 | solute carrier family 11 member 2 [Source:HGNC Symbol;Acc:HGNC:10908] |
| ENSG00000112038 | HGLibA_33451 | 56 | -0.628154287 | OPRM1 | opioid receptor mu 1 [Source:HGNC Symbol;Acc:HGNC:8156] |
| ENSG00000112561 | HGLibA_49164 | 56 | -0.628154287 | TFEB | transcription factor EB [Source:HGNC Symbol;Acc:HGNC:11753] |
| ENSG00000113312 | HGLibA_52046 | 56 | -0.628154287 | TTC1 | tetratricopeptide repeat domain 1 [Source:HGNC Symbol;Acc:HGNC:12391] |
| ENSG00000114346 | HGLibA_14425 | 56 | -0.628154287 | ECT2 | epithelial cell transforming 2 [Source:HGNC Symbol;Acc:HGNC:3155] |
| ENSG00000114383 | HGLibA_52344 | 56 | -0.628154287 | TUSC2 | mitochondrial calcium regulator [Source:HGNC Symbol;Acc:HGNC:17034]" |
| ENSG00000114978 | HGLibA_29586 | 56 | -0.628154287 | MOB1A | MOB kinase activator 1A [Source:HGNC Symbol;Acc:HGNC:16015] |
| ENSG00000115085 | HGLibA_54774 | 56 | -0.628154287 | ZAP70 | zeta chain of T cell receptor associated protein kinase 70 [Source:HGNC Symbol;Acc:HGNC:12858] |
| ENSG00000115286 | HGLibA_31563 | 56 | -0.628154287 | NDUFS7 | NADH:ubiquinone oxidoreductase core subunit S7 [Source:HGNC Symbol;Acc:HGNC:7714] |
| ENSG00000115325 | HGLibA_13768 | 56 | -0.628154287 | DOK1 | docking protein 1 [Source:HGNC Symbol;Acc:HGNC:2990] |
| ENSG00000115457 | HGLibA_22818 | 56 | -0.628154287 | IGFBP2 | insulin like growth factor binding protein 2 [Source:HGNC Symbol;Acc:HGNC:5471] |
| ENSG00000115594 | HGLibA_23045 | 56 | -0.628154287 | IL1R1 | interleukin 1 receptor type 1 [Source:HGNC Symbol;Acc:HGNC:5993] |
| ENSG00000116809 | HGLibA_54828 | 56 | -0.628154287 | ZBTB17 | zinc finger and BTB domain containing 17 [Source:HGNC Symbol;Acc:HGNC:12936] |
| ENSG00000116852 | HGLibA_24901 | 56 | -0.628154287 | KIF21B | kinesin family member 21B [Source:HGNC Symbol;Acc:HGNC:29442] |
| ENSG00000117153 | HGLibA_25128 | 56 | -0.628154287 | KLHL12 | kelch like family member 12 [Source:HGNC Symbol;Acc:HGNC:19360] |

| | | | | | |
|---|---|---|---|---|---|
| ENSG00000119283 | HGLibA_51588 | 56 | -0.628154287 | TRIM67 | tripartite motif containing 67 [Source:HGNC Symbol;Acc:HGNC:31859] |
| ENSG00000119541 | HGLibA_53799 | 56 | -0.628154287 | VPS4B | vacuolar protein sorting 4 homolog B [Source:HGNC Symbol;Acc:HGNC:10895] |
| ENSG00000119599 | HGLibA_12439 | 56 | -0.628154287 | DCAF4 | DDB1 and CUL4 associated factor 4 [Source:HGNC Symbol;Acc:HGNC:20229] |
| ENSG00000121797 | HGLibA_08178 | 56 | -0.628154287 | CCRL2 | C-C motif chemokine receptor like 2 [Source:HGNC Symbol;Acc:HGNC:1612] |
| ENSG00000125457 | HGLibA_29264 | 56 | -0.628154287 | MIF4GD | MIF4G domain containing [Source:HGNC Symbol;Acc:HGNC:24030] |
| ENSG00000125482 | HGLibA_52167 | 56 | -0.628154287 | TTF1 | transcription termination factor 1 [Source:HGNC Symbol;Acc:HGNC:12397] |
| ENSG00000127946 | HGLibA_21333 | 56 | -0.628154287 | HIP1 | huntingtin interacting protein 1 [Source:HGNC Symbol;Acc:HGNC:4913] |
| ENSG00000129596 | HGLibA_08853 | 56 | -0.628154287 | CDO1 | cysteine dioxygenase type 1 [Source:HGNC Symbol;Acc:HGNC:1795] |
| ENSG00000130385 | HGLibA_04596 | 56 | -0.628154287 | BMP15 | bone morphogenetic protein 15 [Source:HGNC Symbol;Acc:HGNC:1068] |
| ENSG00000130725 | HGLibA_52634 | 56 | -0.628154287 | UBE2M | ubiquitin conjugating enzyme E2 M [Source:HGNC Symbol;Acc:HGNC:12491] |
| ENSG00000132254 | HGLibA_02678 | 56 | -0.628154287 | ARFIP2 | ADP ribosylation factor interacting protein 2 [Source:HGNC Symbol;Acc:HGNC:17160] |
| ENSG00000132406 | HGLibA_49918 | 56 | -0.628154287 | TMEM128 | transmembrane protein 128 [Source:HGNC Symbol;Acc:HGNC:28201] |
| ENSG00000132718 | HGLibA_48050 | 56 | -0.628154287 | SYT11 | synaptotagmin 11 [Source:HGNC Symbol;Acc:HGNC:19239] |
| ENSG00000132718 | HGLibA_48051 | 56 | -0.628154287 | SYT11 | synaptotagmin 11 [Source:HGNC Symbol;Acc:HGNC:19239] |
| ENSG00000133107 | HGLibA_51734 | 56 | -0.628154287 | TRPC4 | transient receptor potential cation channel subfamily C member 4 [Source:HGNC Symbol;Acc:HGNC:12336] |
| ENSG00000134108 | HGLibA_03000 | 56 | -0.628154287 | ARL8B | ADP ribosylation factor like GTPase 8B [Source:HGNC Symbol;Acc:HGNC:25564] |
| ENSG00000134323 | HGLibA_30680 | 56 | -0.628154287 | MYCN | bHLH transcription factor [Source:HGNC Symbol;Acc:HGNC:7559]" |
| ENSG00000134508 | HGLibA_06841 | 56 | -0.628154287 | CABLES1 | Cdk5 and Abl enzyme substrate 1 [Source:HGNC Symbol;Acc:HGNC:25097] |
| ENSG00000135211 | HGLibA_50464 | 56 | -0.628154287 | TMEM60 | transmembrane protein 60 [Source:HGNC Symbol;Acc:HGNC:21754] |
| ENSG00000135333 | HGLibA_15306 | 56 | -0.628154287 | EPHA7 | EPH receptor A7 [Source:HGNC Symbol;Acc:HGNC:3390] |
| ENSG00000135677 | HGLibA_19590 | 56 | -0.628154287 | GNS | glucosamine (N-acetyl)-6-sulfatase [Source:HGNC Symbol;Acc:HGNC:4422] |
| ENSG00000136488 | HGLibA_11351 | 56 | -0.628154287 | CSH1 | chorionic somatomammotropin hormone 1 [Source:HGNC Symbol;Acc:HGNC:2440] |
| ENSG00000137478 | HGLibA_17214 | 56 | -0.628154287 | FCHSD2 | FCH and double SH3 domains 2 [Source:HGNC Symbol;Acc:HGNC:29114] |
| ENSG00000137642 | HGLibA_46194 | 56 | -0.628154287 | SORL1 | sortilin related receptor 1 [Source:HGNC Symbol;Acc:HGNC:11185] |
| ENSG00000138207 | HGLibA_40638 | 56 | -0.628154287 | RBP4 | retinol binding protein 4 [Source:HGNC Symbol;Acc:HGNC:9922] |
| ENSG00000138379 | HGLibA_30265 | 56 | -0.628154287 | MSTN | myostatin [Source:HGNC Symbol;Acc:HGNC:4223] |
| ENSG00000138483 | HGLibA_07802 | 56 | -0.628154287 | CCDC54 | coiled-coil domain containing 54 [Source:HGNC Symbol;Acc:HGNC:30703] |
| ENSG00000139117 | HGLibA_10974 | 56 | -0.628154287 | CPNE8 | copine 8 [Source:HGNC Symbol;Acc:HGNC:23498] |
| ENSG00000140650 | HGLibA_37318 | 56 | -0.628154287 | PMM2 | phosphomannomutase 2 [Source:HGNC Symbol;Acc:HGNC:9115] |
| ENSG00000140798 | HGLibA_00167 | 56 | -0.628154287 | ABCC12 | ATP binding cassette subfamily C member 12 [Source:HGNC Symbol;Acc:HGNC:14640] |
| ENSG00000140931 | HGLibA_10214 | 56 | -0.628154287 | CMTM3 | CKLF like MARVEL transmembrane domain containing 3 [Source:HGNC Symbol;Acc:HGNC:19174] |
| ENSG00000141480 | HGLibA_03111 | 56 | -0.628154287 | ARRB2 | arrestin beta 2 [Source:HGNC Symbol;Acc:HGNC:712] |
| ENSG00000141580 | HGLibA_54099 | 56 | -0.628154287 | WDR45B | WD repeat domain 45B [Source:HGNC Symbol;Acc:HGNC:25072] |
| ENSG00000142751 | HGLibA_19828 | 56 | -0.628154287 | GPN2 | GPN-loop GTPase 2 [Source:HGNC Symbol;Acc:HGNC:25513] |
| ENSG00000143149 | HGLibA_01589 | 56 | -0.628154287 | ALDH9A1 | aldehyde dehydrogenase 9 family member A1 [Source:HGNC Symbol;Acc:HGNC:412] |
| ENSG00000143183 | HGLibA_49781 | 56 | -0.628154287 | TMCO1 | transmembrane and coiled-coil domains 1 [Source:HGNC Symbol;Acc:HGNC:18188] |
| ENSG00000143450 | HGLibA_33219 | 56 | -0.628154287 | OAZ3 | ornithine decarboxylase antizyme 3 [Source:HGNC Symbol;Acc:HGNC:8097] |
| ENSG00000146433 | HGLibA_50098 | 56 | -0.628154287 | TMEM181 | transmembrane protein 181 [Source:HGNC Symbol;Acc:HGNC:20958] |
| ENSG00000146576 | HGLibA_06528 | 56 | -0.628154287 | C7orf26 | chromosome 7 open reading frame 26 [Source:HGNC Symbol;Acc:HGNC:21702] |
| ENSG00000147604 | HGLibA_41993 | 56 | -0.628154287 | RPL7 | ribosomal protein L7 [Source:HGNC Symbol;Acc:HGNC:10363] |
| ENSG00000147905 | HGLibA_55044 | 56 | -0.628154287 | ZCCHC7 | zinc finger CCHC-type containing 7 [Source:HGNC Symbol;Acc:HGNC:26209] |
| ENSG00000148090 | HGLibA_03863 | 56 | -0.628154287 | AUH | AU RNA binding methylglutaconyl-CoA hydratase [Source:HGNC Symbol;Acc:HGNC:890] |
| ENSG00000149016 | HGLibA_52354 | 56 | -0.628154287 | TUT1 | U6 snRNA-specific [Source:HGNC Symbol;Acc:HGNC:26184]" |
| ENSG00000149021 | HGLibA_42908 | 56 | -0.628154287 | SCGB1A1 | secretoglobin family 1A member 1 [Source:HGNC Symbol;Acc:HGNC:12523] |
| ENSG00000149212 | HGLibA_43626 | 56 | -0.628154287 | SESN3 | sestrin 3 [Source:HGNC Symbol;Acc:HGNC:23060] |
| ENSG00000149380 | HGLibA_34906 | 56 | -0.628154287 | P4HA3 | prolyl 4-hydroxylase subunit alpha 3 [Source:HGNC Symbol;Acc:HGNC:30135] |
| ENSG00000149679 | HGLibA_06844 | 56 | -0.628154287 | CABLES2 | Cdk5 and Abl enzyme substrate 2 [Source:HGNC Symbol;Acc:HGNC:16143] |
| ENSG00000150722 | HGLibA_38070 | 56 | -0.628154287 | PPP1R1C | protein phosphatase 1 regulatory inhibitor subunit 1C [Source:HGNC Symbol;Acc:HGNC:14940] |
| ENSG00000151229 | HGLibA_44863 | 56 | -0.628154287 | SLC2A13 | solute carrier family 2 member 13 [Source:HGNC Symbol;Acc:HGNC:15956] |
| ENSG00000152778 | HGLibA_22636 | 56 | -0.628154287 | IFIT5 | interferon induced protein with tetratricopeptide repeats 5 [Source:HGNC Symbol;Acc:HGNC:13328] |
| ENSG00000153721 | HGLibA_10302 | 56 | -0.628154287 | CNKSR3 | CNKSR family member 3 [Source:HGNC Symbol;Acc:HGNC:23034] |
| ENSG00000153823 | HGLibA_36529 | 56 | -0.628154287 | PID1 | phosphotyrosine interaction domain containing 1 [Source:HGNC Symbol;Acc:HGNC:26084] |
| ENSG00000154620 | HGLibA_50667 | 56 | -0.628154287 | TMSB4Y | thymosin beta 4 Y-linked [Source:HGNC Symbol;Acc:HGNC:11882] |
| ENSG00000155313 | HGLibA_53312 | 56 | -0.628154287 | USP25 | ubiquitin specific peptidase 25 [Source:HGNC Symbol;Acc:HGNC:12624] |
| ENSG00000155876 | HGLibA_42223 | 56 | -0.628154287 | RRAGA | Ras related GTP binding A [Source:HGNC Symbol;Acc:HGNC:16963] |
| ENSG00000155961 | HGLibA_39905 | 56 | -0.628154287 | RAB39B | member RAS oncogene family [Source:HGNC Symbol;Acc:HGNC:16499]" |
| ENSG00000156218 | HGLibA_00853 | 56 | -0.628154287 | ADAMTSL3 | ADAMTS like 3 [Source:HGNC Symbol;Acc:HGNC:14633] |
| ENSG00000157110 | HGLibA_40652 | 56 | -0.628154287 | RBPMS | mRNA processing factor [Source:HGNC Symbol;Acc:HGNC:19097]" |
| ENSG00000157600 | HGLibA_50034 | 56 | -0.628154287 | TMEM164 | transmembrane protein 164 [Source:HGNC Symbol;Acc:HGNC:26217] |
| ENSG00000157884 | HGLibA_09692 | 56 | -0.628154287 | CIB4 | calcium and integrin binding family member 4 [Source:HGNC Symbol;Acc:HGNC:33703] |
| ENSG00000158321 | HGLibA_03886 | 56 | -0.628154287 | AUTS2 | activator of transcription and developmental regulator [Source:HGNC Symbol;Acc:HGNC:14262]" |
| ENSG00000158796 | HGLibA_12779 | 56 | -0.628154287 | DEDD | death effector domain containing [Source:HGNC Symbol;Acc:HGNC:2755] |
| ENSG00000159128 | HGLibA_22712 | 56 | -0.628154287 | IFNGR2 | interferon gamma receptor 2 [Source:HGNC Symbol;Acc:HGNC:5440] |
| ENSG00000159915 | HGLibA_55643 | 56 | -0.628154287 | ZNF233 | zinc finger protein 233 [Source:HGNC Symbol;Acc:HGNC:30946] |
| ENSG00000162460 | HGLibA_50523 | 56 | -0.628154287 | TMEM82 | transmembrane protein 82 [Source:HGNC Symbol;Acc:HGNC:32350] |
| ENSG00000162882 | HGLibA_20791 | 56 | -0.628154287 | HAAO | 4-dioxygenase [Source:HGNC Symbol;Acc:HGNC:4796]" |
| ENSG00000163207 | HGLibA_23880 | 56 | -0.628154287 | IVL | involucrin [Source:HGNC Symbol;Acc:HGNC:6187] |
| ENSG00000163354 | HGLibA_12531 | 56 | -0.628154287 | DCST2 | DC-STAMP domain containing 2 [Source:HGNC Symbol;Acc:HGNC:26562] |
| ENSG00000163466 | HGLibA_03079 | 56 | -0.628154287 | ARPC2 | actin related protein 2/3 complex subunit 2 [Source:HGNC Symbol;Acc:HGNC:705] |
| ENSG00000163644 | HGLibA_37987 | 56 | -0.628154287 | PPM1K | Mg2+/Mn2+ dependent 1K [Source:HGNC Symbol;Acc:HGNC:25415]" |
| ENSG00000164128 | HGLibA_32551 | 56 | -0.628154287 | NPY1R | neuropeptide Y receptor Y1 [Source:HGNC Symbol;Acc:HGNC:7956] |
| ENSG00000164867 | HGLibA_32332 | 56 | -0.628154287 | NOS3 | nitric oxide synthase 3 [Source:HGNC Symbol;Acc:HGNC:7876] |
| ENSG00000165934 | HGLibA_10996 | 56 | -0.628154287 | CPSF2 | cleavage and polyadenylation specific factor 2 [Source:HGNC Symbol;Acc:HGNC:2325] |
| ENSG00000166068 | HGLibA_46791 | 56 | -0.628154287 | SPRED1 | sprouty related EVH1 domain containing 1 [Source:HGNC Symbol;Acc:HGNC:20249] |
| ENSG00000166862 | HGLibA_06931 | 56 | -0.628154287 | CACNG2 | calcium voltage-gated channel auxiliary subunit gamma 2 [Source:HGNC Symbol;Acc:HGNC:1406] |
| ENSG00000167394 | HGLibA_56493 | 56 | -0.628154287 | ZNF668 | zinc finger protein 668 [Source:HGNC Symbol;Acc:HGNC:25821] |
| ENSG00000167625 | HGLibA_56163 | 56 | -0.628154287 | ZNF526 | zinc finger protein 526 [Source:HGNC Symbol;Acc:HGNC:29415] |
| ENSG00000167671 | HGLibA_52791 | 56 | -0.628154287 | UBXN6 | UBX domain protein 6 [Source:HGNC Symbol;Acc:HGNC:14928] |
| ENSG00000168077 | HGLibA_42864 | 56 | -0.628154287 | SCARA3 | scavenger receptor class A member 3 [Source:HGNC Symbol;Acc:HGNC:19000] |
| ENSG00000168418 | HGLibA_24208 | 56 | -0.628154287 | KCNG4 | potassium voltage-gated channel modifier subfamily G member 4 [Source:HGNC Symbol;Acc:HGNC:19697] |
| ENSG00000168743 | HGLibA_32495 | 56 | -0.628154287 | NPNT | nephronectin [Source:HGNC Symbol;Acc:HGNC:27405] |
| ENSG00000168758 | HGLibA_43345 | 56 | -0.628154287 | SEMA4C | semaphorin 4C [Source:HGNC Symbol;Acc:HGNC:10731] |
| ENSG00000169064 | HGLibA_54785 | 56 | -0.628154287 | ZBBX | zinc finger B-box domain containing [Source:HGNC Symbol;Acc:HGNC:26245] |
| ENSG00000169252 | HGLibA_01058 | 56 | -0.628154287 | ADRB2 | adrenoceptor beta 2 [Source:HGNC Symbol;Acc:HGNC:286] |
| ENSG00000169851 | HGLibA_35415 | 56 | -0.628154287 | PCDH7 | protocadherin 7 [Source:HGNC Symbol;Acc:HGNC:8659] |
| ENSG00000170088 | HGLibA_50151 | 56 | -0.628154287 | TMEM192 | transmembrane protein 192 [Source:HGNC Symbol;Acc:HGNC:26775] |
| ENSG00000170579 | HGLibA_13340 | 56 | -0.628154287 | DLGAP1 | DLG associated protein 1 [Source:HGNC Symbol;Acc:HGNC:2905] |
| ENSG00000171989 | HGLibA_26226 | 56 | -0.628154287 | LDHAL6B | lactate dehydrogenase A like 6B [Source:HGNC Symbol;Acc:HGNC:21481] |
| ENSG00000172466 | HGLibA_55658 | 56 | -0.628154287 | ZNF24 | zinc finger protein 24 [Source:HGNC Symbol;Acc:HGNC:13032] |
| ENSG00000173376 | HGLibA_31400 | 56 | -0.628154287 | NDNF | neuron derived neurotrophic factor [Source:HGNC Symbol;Acc:HGNC:26256] |
| ENSG00000173401 | HGLibA_19261 | 56 | -0.628154287 | GLIPR1L1 | GLIPR1 like 1 [Source:HGNC Symbol;Acc:HGNC:28392] |
| ENSG00000174013 | HGLibA_17109 | 56 | -0.628154287 | FBXO45 | F-box protein 45 [Source:HGNC Symbol;Acc:HGNC:29148] |
| ENSG00000175398 | HGLibA_33535 | 56 | -0.628154287 | OR10P1 | olfactory receptor family 10 subfamily P member 1 [Source:HGNC Symbol;Acc:HGNC:15378] |
| ENSG00000175426 | HGLibA_35668 | 56 | -0.628154287 | PCSK1 | proprotein convertase subtilisin/kexin type 1 [Source:HGNC Symbol;Acc:HGNC:8743] |
| ENSG00000176406 | HGLibA_41280 | 56 | -0.628154287 | RIMS2 | regulating synaptic membrane exocytosis 2 [Source:HGNC Symbol;Acc:HGNC:17283] |
| ENSG00000177174 | HGLibA_33631 | 56 | -0.628154287 | OR14C36 | olfactory receptor family 14 subfamily C member 36 [Source:HGNC Symbol;Acc:HGNC:15026] |
| ENSG00000179008 | HGLibA_05366 | 56 | -0.628154287 | C14orf39 | chromosome 14 open reading frame 39 [Source:HGNC Symbol;Acc:HGNC:19849] |
| ENSG00000179044 | HGLibA_15739 | 56 | -0.628154287 | EXOC3L1 | exocyst complex component 3 like 1 [Source:HGNC Symbol;Acc:HGNC:27540] |
| ENSG00000179626 | HGLibA_34391 | 56 | -0.628154287 | OR6C4 | olfactory receptor family 6 subfamily C member 4 [Source:HGNC Symbol;Acc:HGNC:19632] |
| ENSG00000179988 | HGLibA_39236 | 56 | -0.628154287 | PSTK | phosphoseryl-tRNA kinase [Source:HGNC Symbol;Acc:HGNC:28578] |
| ENSG00000180011 | HGLibA_54770 | 56 | -0.628154287 | ZADH2 | zinc binding alcohol dehydrogenase domain containing 2 [Source:HGNC Symbol;Acc:HGNC:28697] |
| ENSG00000180098 | HGLibA_51708 | 56 | -0.628154287 | TRNAU1AP | tRNA selenocysteine 1 associated protein 1 [Source:HGNC Symbol;Acc:HGNC:30813] |
| ENSG00000180543 | HGLibA_51988 | 56 | -0.628154287 | TSPYL5 | TSPY like 5 [Source:HGNC Symbol;Acc:HGNC:29367] |
| ENSG00000180644 | HGLibA_38448 | 56 | -0.628154287 | PRF1 | perforin 1 [Source:HGNC Symbol;Acc:HGNC:9360] |
| ENSG00000180767 | HGLibA_09622 | 56 | -0.628154287 | CHST13 | carbohydrate sulfotransferase 13 [Source:HGNC Symbol;Acc:HGNC:21755] |
| ENSG00000180917 | HGLibA_10235 | 56 | -0.628154287 | CMTR2 | cap methyltransferase 2 [Source:HGNC Symbol;Acc:HGNC:25635] |
| ENSG00000181924 | HGLibA_10450 | 56 | -0.628154287 | COA4 | cytochrome c oxidase assembly factor 4 homolog [Source:HGNC Symbol;Acc:HGNC:24604] |
| ENSG00000182247 | HGLibA_52597 | 56 | -0.628154287 | UBE2E2 | ubiquitin conjugating enzyme E2 E2 [Source:HGNC Symbol;Acc:HGNC:12478] |
| ENSG00000182333 | HGLibA_26609 | 56 | -0.628154287 | LIPF | gastric type [Source:HGNC Symbol;Acc:HGNC:6622]" |
| ENSG00000183401 | HGLibA_07667 | 56 | -0.628154287 | CCDC159 | coiled-coil domain containing 159 [Source:HGNC Symbol;Acc:HGNC:26996] |
| ENSG00000183648 | HGLibA_31502 | 56 | -0.628154287 | NDUFB1 | NADH:ubiquinone oxidoreductase subunit B1 [Source:HGNC Symbol;Acc:HGNC:7695] |
| ENSG00000183943 | HGLibA_38594 | 56 | -0.628154287 | PRKX | protein kinase X-linked [Source:HGNC Symbol;Acc:HGNC:9441] |
| ENSG00000185475 | HGLibA_50090 | 56 | -0.628154287 | TMEM179B | transmembrane protein 179B [Source:HGNC Symbol;Acc:HGNC:33744] |
| ENSG00000185946 | HGLibA_41725 | 56 | -0.628154287 | RNPC3 | RRM) containing 3 [Source:HGNC Symbol;Acc:HGNC:18666]" |
| ENSG00000186281 | HGLibA_19731 | 56 | -0.628154287 | GPAT2 | mitochondrial [Source:HGNC Symbol;Acc:HGNC:27168]" |
| ENSG00000186364 | HGLibA_32958 | 56 | -0.628154287 | NUDT17 | nudix hydrolase 17 [Source:HGNC Symbol;Acc:HGNC:26618] |
| ENSG00000187098 | HGLibA_29319 | 56 | -0.628154287 | MITF | melanocyte inducing transcription factor [Source:HGNC Symbol;Acc:HGNC:7105] |
| ENSG00000187796 | HGLibA_07240 | 56 | -0.628154287 | CARD9 | caspase recruitment domain family member 9 [Source:HGNC Symbol;Acc:HGNC:16391] |
| ENSG00000188026 | HGLibA_41253 | 56 | -0.628154287 | RILPL1 | Rab interacting lysosomal protein like 1 [Source:HGNC Symbol;Acc:HGNC:26814] |
| ENSG00000188076 | HGLibA_42910 | 56 | -0.628154287 | SCGB1C1 | secretoglobin family 1C member 1 [Source:HGNC Symbol;Acc:HGNC:18394] |
| ENSG00000188909 | HGLibA_04888 | 56 | -0.628154287 | BSX | brain specific homeobox [Source:HGNC Symbol;Acc:HGNC:20450] |
| ENSG00000189339 | HGLibA_44993 | 56 | -0.628154287 | SLC35E2B | solute carrier family 35 member E2B [Source:HGNC Symbol;Acc:HGNC:33941] |
| ENSG00000196071 | HGLibA_33819 | 56 | -0.628154287 | OR2L13 | olfactory receptor family 2 subfamily L member 13 [Source:HGNC Symbol;Acc:HGNC:19578] |
| ENSG00000196767 | HGLibA_37775 | 56 | -0.628154287 | POU3F4 | POU class 3 homeobox 4 [Source:HGNC Symbol;Acc:HGNC:9217] |
| ENSG00000197121 | HGLibA_36208 | 56 | -0.628154287 | PGAP1 | post-GPI attachment to proteins 1 [Source:HGNC Symbol;Acc:HGNC:25712] |
| ENSG00000197410 | HGLibA_12492 | 56 | -0.628154287 | DCHS2 | dachsous cadherin-related 2 [Source:HGNC Symbol;Acc:HGNC:23111] |
| ENSG00000197520 | HGLibA_16298 | 56 | -0.628154287 | FAM177B | family with sequence similarity 177 member B [Source:HGNC Symbol;Acc:HGNC:34395] |
| ENSG00000197683 | HGLibA_25730 | 56 | -0.628154287 | KRTAP26-1 | keratin associated protein 26-1 [Source:HGNC Symbol;Acc:HGNC:33760] |
| ENSG00000198429 | HGLibA_56547 | 56 | -0.628154287 | ZNF69 | zinc finger protein 69 [Source:HGNC Symbol;Acc:HGNC:13138] |
| ENSG00000198535 | HGLibA_06095 | 56 | -0.628154287 | C2CD4A | C2 calcium dependent domain containing 4A [Source:HGNC Symbol;Acc:HGNC:33627] |
| ENSG00000198929 | HGLibA_32326 | 56 | -0.628154287 | NOS1AP | nitric oxide synthase 1 adaptor protein [Source:HGNC Symbol;Acc:HGNC:16859] |
| ENSG00000203880 | HGLibA_35632 | 56 | -0.628154287 | PCMTD2 | protein-L-isoaspartate (D-aspartate) O-methyltransferase domain containing 2 [Source:HGNC Symbol;Acc:HGNC:15882] |
| ENSG00000204010 | HGLibA_22626 | 56 | -0.628154287 | IFIT1B | interferon induced protein with tetratricopeptide repeats 1B [Source:HGNC Symbol;Acc:HGNC:23442] |
| ENSG00000204138 | HGLibA_36306 | 56 | -0.628154287 | PHACTR4 | phosphatase and actin regulator 4 [Source:HGNC Symbol;Acc:HGNC:25793] |
| ENSG00000204576 | HGLibA_38837 | 56 | -0.628154287 | PRR3 | proline rich 3 [Source:HGNC Symbol;Acc:HGNC:21149] |

| | | | | | |
|---|---|---|---|---|---|
| ENSG00000205084 | HGLibA_50258 | 56 | -0.628154287 | TMEM231 | transmembrane protein 231 [Source:HGNC Symbol;Acc:HGNC:37234] |
| ENSG00000206106 | HGLibA_25718 | 56 | -0.628154287 | KRTAP22-2 | keratin associated protein 22-2 [Source:HGNC Symbol;Acc:HGNC:37091] |
| ENSG00000206107 | HGLibA_25733 | 56 | -0.628154287 | KRTAP27-1 | keratin associated protein 27-1 [Source:HGNC Symbol;Acc:HGNC:33864] |
| ENSG00000213022 | HGLibA_25273 | 56 | -0.628154287 | KLK9 | kallikrein related peptidase 9 [Source:HGNC Symbol;Acc:HGNC:6370] |
| ENSG00000214688 | HGLibA_05022 | 56 | -0.628154287 | C10orf105 | chromosome 10 open reading frame 105 [Source:HGNC Symbol;Acc:HGNC:20304] |
| ENSG00000221837 | HGLibA_25624 | 56 | -0.628154287 | KRTAP10-9 | keratin associated protein 10-9 [Source:HGNC Symbol;Acc:HGNC:22971] |
| ENSG00000221923 | HGLibA_56894 | 56 | -0.628154287 | ZNF880 | zinc finger protein 880 [Source:HGNC Symbol;Acc:HGNC:37249] |
| ENSG00000227151 | HGLibA_38808 | 56 | -0.628154287 | PRR20D | proline rich 20D [Source:HGNC Symbol;Acc:HGNC:37222] |
| ENSG00000227500 | HGLibA_42845 | 56 | -0.628154287 | SCAMP4 | secretory carrier membrane protein 4 [Source:HGNC Symbol;Acc:HGNC:30385] |
| ENSG00000227551 | HGLibA_53222 | 56 | -0.628154287 | USP17L12 | ubiquitin specific peptidase 17-like family member 12 [Source:HGNC Symbol;Acc:HGNC:44440] |
| ENSG00000236761 | HGLibA_11582 | 56 | -0.628154287 | CTAGE9 | CTAGE family member 9 [Source:HGNC Symbol;Acc:HGNC:37275] |
| ENSG00000237247 | HGLibA_28485 | 56 | -0.628154287 | MBD3L5 | methyl-CpG binding domain protein 3 like 5 [Source:HGNC Symbol;Acc:HGNC:37204] |
| ENSG00000240747 | HGLibA_25392 | 56 | -0.628154287 | KRBOX1 | KRAB box domain containing 1 [Source:HGNC Symbol;Acc:HGNC:38708] |
| ENSG00000242950 | HGLibA_15551 | 56 | -0.628154287 | ERVW-1 | envelope [Source:HGNC Symbol;Acc:HGNC:13525]" |
| ENSG00000243955 | HGLibA_20452 | 56 | -0.628154287 | GSTA1 | glutathione S-transferase alpha 1 [Source:HGNC Symbol;Acc:HGNC:4626] |
| ENSG00000244274 | HGLibA_12395 | 56 | -0.628154287 | DBNDD2 | dysbindin domain containing 2 [Source:HGNC Symbol;Acc:HGNC:15881] |
| ENSG00000249158 | HGLibA_35431 | 56 | -0.628154287 | PCDHA11 | protocadherin alpha 11 [Source:HGNC Symbol;Acc:HGNC:8665] |
| ENSG00000249811 | HGLibA_53249 | 56 | -0.628154287 | USP17L21 | ubiquitin specific peptidase 17-like family member 21 [Source:HGNC Symbol;Acc:HGNC:44449] |
| ENSG00000254221 | HGLibA_35549 | 56 | -0.628154287 | PCDHGB1 | 1 [Source:HGNC Symbol;Acc:HGNC:8708]" |
| ENSG00000254996 | HGLibA_01966 | 56 | -0.628154287 | ANKHD1-EIF4EBP3 | ANKHD1-EIF4EBP3 readthrough [Source:HGNC Symbol;Acc:HGNC:33530] |
| ENSG00000263001 | HGLibA_20565 | 56 | -0.628154287 | GTF2I | general transcription factor IIi [Source:HGNC Symbol;Acc:HGNC:4659] |
| ENSG00000272395 | HGLibA_22729 | 56 | -0.628154287 | IFNL4 | interferon lambda 4 (gene/pseudogene) [Source:HGNC Symbol;Acc:HGNC:44480] |
| ENSG00000275074 | HGLibA_32962 | 56 | -0.628154287 | NUDT18 | nudix hydrolase 18 [Source:HGNC Symbol;Acc:HGNC:26194] |
| ENSG00000277399 | HGLibA_19979 | 56 | -0.628154287 | GPR179 | G protein-coupled receptor 179 [Source:HGNC Symbol;Acc:HGNC:31371] |
| ENSG00000280090 | HGLibA_34503 | 56 | -0.628154287 | OR8B4 | olfactory receptor family 8 subfamily B member 4 (gene/pseudogene) [Source:HGNC Symbol;Acc:HGNC:8473] |
| ERO1LB | HGLibA_15522 | 56 | -0.628154287 | ERO1LB | - |
| FAM105A | HGLibA_15995 | 56 | -0.628154287 | FAM105A | - |
| FAM132A | HGLibA_16113 | 56 | -0.628154287 | FAM132A | - |
| FAM212A | HGLibA_16465 | 56 | -0.628154287 | FAM212A | - |
| GRAMD2 | HGLibA_20203 | 56 | -0.628154287 | GRAMD2 | - |
| HIST1H3H | HGLibA_21477 | 56 | -0.628154287 | HIST1H3H | - |
| HIST1H4B | HGLibA_21489 | 56 | -0.628154287 | HIST1H4B | - |
| hsa-mir-1-1 | HGLibA_57140 | 56 | -0.628154287 | hsa-mir-1-1 | - |
| hsa-mir-1249 | HGLibA_57393 | 56 | -0.628154287 | hsa-mir-1249 | - |
| hsa-mir-1275 | HGLibA_57557 | 56 | -0.628154287 | hsa-mir-1275 | - |
| hsa-mir-134 | HGLibA_57785 | 56 | -0.628154287 | hsa-mir-134 | - |
| hsa-mir-2053 | HGLibA_58183 | 56 | -0.628154287 | hsa-mir-2053 | - |
| hsa-mir-2110 | HGLibA_58219 | 56 | -0.628154287 | hsa-mir-2110 | - |
| hsa-mir-29c | HGLibA_58422 | 56 | -0.628154287 | hsa-mir-29c | - |
| hsa-mir-3161 | HGLibA_58725 | 56 | -0.628154287 | hsa-mir-3161 | - |
| hsa-mir-3529 | HGLibA_59048 | 56 | -0.628154287 | hsa-mir-3529 | - |
| hsa-mir-378j | HGLibA_59464 | 56 | -0.628154287 | hsa-mir-378j | - |
| hsa-mir-3911 | HGLibA_59514 | 56 | -0.628154287 | hsa-mir-3911 | - |
| hsa-mir-411 | HGLibA_59683 | 56 | -0.628154287 | hsa-mir-411 | - |
| hsa-mir-4313 | HGLibA_59968 | 56 | -0.628154287 | hsa-mir-4313 | - |
| hsa-mir-4489 | HGLibA_60376 | 56 | -0.628154287 | hsa-mir-4489 | - |
| hsa-mir-4668 | HGLibA_60789 | 56 | -0.628154287 | hsa-mir-4668 | - |
| hsa-mir-4720 | HGLibA_60977 | 56 | -0.628154287 | hsa-mir-4720 | - |
| hsa-mir-4788 | HGLibA_61259 | 56 | -0.628154287 | hsa-mir-4788 | - |
| hsa-mir-548av | HGLibA_61931 | 56 | -0.628154287 | hsa-mir-548av | - |
| hsa-mir-550a-2 | HGLibA_62102 | 56 | -0.628154287 | hsa-mir-550a-2 | - |
| hsa-mir-5692a-2 | HGLibA_62290 | 56 | -0.628154287 | hsa-mir-5692a-2 | - |
| hsa-mir-579 | HGLibA_62413 | 56 | -0.628154287 | hsa-mir-579 | - |
| hsa-mir-6089-2 | HGLibA_62619 | 56 | -0.628154287 | hsa-mir-6089-2 | - |
| hsa-mir-6738 | HGLibA_63107 | 56 | -0.628154287 | hsa-mir-6738 | - |
| hsa-mir-6790 | HGLibA_63339 | 56 | -0.628154287 | hsa-mir-6790 | - |
| hsa-mir-6804 | HGLibA_63394 | 56 | -0.628154287 | hsa-mir-6804 | - |
| hsa-mir-6843 | HGLibA_63549 | 56 | -0.628154287 | hsa-mir-6843 | - |
| hsa-mir-6893 | HGLibA_63757 | 56 | -0.628154287 | hsa-mir-6893 | - |
| hsa-mir-766 | HGLibA_63926 | 56 | -0.628154287 | hsa-mir-766 | - |
| hsa-mir-7852 | HGLibA_63996 | 56 | -0.628154287 | hsa-mir-7852 | - |
| KIAA1731 | HGLibA_24810 | 56 | -0.628154287 | KIAA1731 | - |
| LRRC16B | HGLibA_27267 | 56 | -0.628154287 | LRRC16B | - |
| MEF2BNB-MEF2B | HGLibA_28834 | 56 | -0.628154287 | MEF2BNB-MEF2B | - |
| MKL2 | HGLibA_29337 | 56 | -0.628154287 | MKL2 | - |
| VWA9 | HGLibA_53919 | 56 | -0.628154287 | VWA9 | - |
| ZFP106 | HGLibA_55182 | 56 | -0.628154287 | ZFP106 | - |
| C12orf74 | HGLibA_05295 | 55 | -0.63719963 | C12orf74 | - |
| C17orf102 | HGLibA_05518 | 55 | -0.63719963 | C17orf102 | - |
| C17orf96 | HGLibA_05593 | 55 | -0.63719963 | C17orf96 | - |
| C20orf24 | HGLibA_06016 | 55 | -0.63719963 | C20orf24 | - |
| C2orf53 | HGLibA_06146 | 55 | -0.63719963 | C2orf53 | - |
| C4B_2 | HGLibA_06287 | 55 | -0.63719963 | C4B_2 | - |
| DARC | HGLibA_12337 | 55 | -0.63719963 | DARC | - |
| ELMSAN1 | HGLibA_14948 | 55 | -0.63719963 | ELMSAN1 | - |
| ENSG00000008853 | HGLibA_41176 | 55 | -0.63719963 | RHOBTB2 | Rho related BTB domain containing 2 [Source:HGNC Symbol;Acc:HGNC:18756] |
| ENSG00000011052 | HGLibA_32153 | 55 | -0.63719963 | NME1-NME2 | NME1-NME2 readthrough [Source:HGNC Symbol;Acc:HGNC:33531] |
| ENSG00000011243 | HGLibA_01449 | 55 | -0.63719963 | AKAP8L | A-kinase anchoring protein 8 like [Source:HGNC Symbol;Acc:HGNC:29857] |
| ENSG00000065328 | HGLibA_28600 | 55 | -0.63719963 | MCM10 | minichromosome maintenance 10 replication initiation factor [Source:HGNC Symbol;Acc:HGNC:18043] |
| ENSG00000068650 | HGLibA_03555 | 55 | -0.63719963 | ATP11A | ATPase phospholipid transporting 11A [Source:HGNC Symbol;Acc:HGNC:13552] |
| ENSG00000073150 | HGLibA_35111 | 55 | -0.63719963 | PANX2 | pannexin 2 [Source:HGNC Symbol;Acc:HGNC:8600] |
| ENSG00000074181 | HGLibA_32348 | 55 | -0.63719963 | NOTCH3 | notch 3 [Source:HGNC Symbol;Acc:HGNC:7883] |
| ENSG00000075651 | HGLibA_37035 | 55 | -0.63719963 | PLD1 | phospholipase D1 [Source:HGNC Symbol;Acc:HGNC:9067] |
| ENSG00000077713 | HGLibA_44745 | 55 | -0.63719963 | SLC25A43 | solute carrier family 25 member 43 [Source:HGNC Symbol;Acc:HGNC:30557] |
| ENSG00000078967 | HGLibA_52590 | 55 | -0.63719963 | UBE2D4 | ubiquitin conjugating enzyme E2 D4 (putative) [Source:HGNC Symbol;Acc:HGNC:21647] |
| ENSG00000080511 | HGLibA_40752 | 55 | -0.63719963 | RDH8 | retinol dehydrogenase 8 [Source:HGNC Symbol;Acc:HGNC:14423] |
| ENSG00000082269 | HGLibA_16133 | 55 | -0.63719963 | FAM135A | family with sequence similarity 135 member A [Source:HGNC Symbol;Acc:HGNC:21084] |
| ENSG00000085832 | HGLibA_15368 | 55 | -0.63719963 | EPS15 | epidermal growth factor receptor pathway substrate 15 [Source:HGNC Symbol;Acc:HGNC:3419] |
| ENSG00000089486 | HGLibA_08712 | 55 | -0.63719963 | CDIP1 | cell death inducing p53 target 1 [Source:HGNC Symbol;Acc:HGNC:13234] |
| ENSG00000095752 | HGLibA_22952 | 55 | -0.63719963 | IL11 | interleukin 11 [Source:HGNC Symbol;Acc:HGNC:5966] |
| ENSG00000099622 | HGLibA_09722 | 55 | -0.63719963 | CIRBP | cold inducible RNA binding protein [Source:HGNC Symbol;Acc:HGNC:1982] |
| ENSG00000099942 | HGLibA_11184 | 55 | -0.63719963 | CRKL | adaptor protein [Source:HGNC Symbol;Acc:HGNC:2363]" |
| ENSG00000099994 | HGLibA_47847 | 55 | -0.63719963 | SUSD2 | sushi domain containing 2 [Source:HGNC Symbol;Acc:HGNC:30667] |
| ENSG00000100034 | HGLibA_37976 | 55 | -0.63719963 | PPM1F | Mg2+/Mn2+ dependent 1F [Source:HGNC Symbol;Acc:HGNC:19388]" |
| ENSG00000100554 | HGLibA_03744 | 55 | -0.63719963 | ATP6V1D | ATPase H+ transporting V1 subunit D [Source:HGNC Symbol;Acc:HGNC:13527] |
| ENSG00000101577 | HGLibA_27098 | 55 | -0.63719963 | LPIN2 | lipin 2 [Source:HGNC Symbol;Acc:HGNC:14450] |
| ENSG00000101752 | HGLibA_29191 | 55 | -0.63719963 | MIB1 | mindbomb E3 ubiquitin protein ligase 1 [Source:HGNC Symbol;Acc:HGNC:21086] |
| ENSG00000102104 | HGLibA_42292 | 55 | -0.63719963 | RS1 | retinoschisin 1 [Source:HGNC Symbol;Acc:HGNC:10457] |
| ENSG00000103061 | HGLibA_45377 | 55 | -0.63719963 | SLC7A6OS | solute carrier family 7 member 6 opposite strand [Source:HGNC Symbol;Acc:HGNC:25807] |
| ENSG00000103313 | HGLibA_28844 | 55 | -0.63719963 | MEFV | pyrin innate immunity regulator [Source:HGNC Symbol;Acc:HGNC:6998]" |
| ENSG00000103494 | HGLibA_41841 | 55 | -0.63719963 | RPGRIP1L | RPGRIP1 like [Source:HGNC Symbol;Acc:HGNC:29168] |
| ENSG00000103495 | HGLibA_28450 | 55 | -0.63719963 | MAZ | MYC associated zinc finger protein [Source:HGNC Symbol;Acc:HGNC:6914] |
| ENSG00000104774 | HGLibA_28077 | 55 | -0.63719963 | MAN2B1 | mannosidase alpha class 2B member 1 [Source:HGNC Symbol;Acc:HGNC:6826] |
| ENSG00000104805 | HGLibA_32910 | 55 | -0.63719963 | NUCB1 | nucleobindin 1 [Source:HGNC Symbol;Acc:HGNC:8043] |
| ENSG00000105085 | HGLibA_28791 | 55 | -0.63719963 | MED26 | mediator complex subunit 26 [Source:HGNC Symbol;Acc:HGNC:2376] |
| ENSG00000105245 | HGLibA_33016 | 55 | -0.63719963 | NUMBL | endocytic adaptor protein [Source:HGNC Symbol;Acc:HGNC:8061]" |
| ENSG00000105287 | HGLibA_38568 | 55 | -0.63719963 | PRKD2 | protein kinase D2 [Source:HGNC Symbol;Acc:HGNC:17293] |
| ENSG00000105808 | HGLibA_40305 | 55 | -0.63719963 | RASA4 | RAS p21 protein activator 4 [Source:HGNC Symbol;Acc:HGNC:23181] |
| ENSG00000106991 | HGLibA_15126 | 55 | -0.63719963 | ENG | endoglin [Source:HGNC Symbol;Acc:HGNC:3349] |
| ENSG00000107338 | HGLibA_43994 | 55 | -0.63719963 | SHB | SH2 domain containing adaptor protein B [Source:HGNC Symbol;Acc:HGNC:10838] |
| ENSG00000108443 | HGLibA_42175 | 55 | -0.63719963 | RPS6KB1 | ribosomal protein S6 kinase B1 [Source:HGNC Symbol;Acc:HGNC:10436] |
| ENSG00000108578 | HGLibA_04552 | 55 | -0.63719963 | BLMH | bleomycin hydrolase [Source:HGNC Symbol;Acc:HGNC:1059] |
| ENSG00000109472 | HGLibA_10913 | 55 | -0.63719963 | CPE | carboxypeptidase E [Source:HGNC Symbol;Acc:HGNC:2303] |
| ENSG00000110536 | HGLibA_39427 | 55 | -0.63719963 | PTPMT1 | mitochondrial 1 [Source:HGNC Symbol;Acc:HGNC:26965]" |
| ENSG00000110844 | HGLibA_38736 | 55 | -0.63719963 | PRPF40B | pre-mRNA processing factor 40 homolog B [Source:HGNC Symbol;Acc:HGNC:25031] |
| ENSG00000111911 | HGLibA_21330 | 55 | -0.63719963 | HINT3 | histidine triad nucleotide binding protein 3 [Source:HGNC Symbol;Acc:HGNC:18468] |
| ENSG00000112378 | HGLibA_36062 | 55 | -0.63719963 | PERP | TP53 apoptosis effector [Source:HGNC Symbol;Acc:HGNC:17637]" |
| ENSG00000113716 | HGLibA_21732 | 55 | -0.63719963 | HMGXB3 | HMG-box containing 3 [Source:HGNC Symbol;Acc:HGNC:28982] |
| ENSG00000114423 | HGLibA_07398 | 55 | -0.63719963 | CBLB | Cbl proto-oncogene B [Source:HGNC Symbol;Acc:HGNC:1542] |
| ENSG00000114503 | HGLibA_31284 | 55 | -0.63719963 | NCBP2 | nuclear cap binding protein subunit 2 [Source:HGNC Symbol;Acc:HGNC:7659] |
| ENSG00000114771 | HGLibA_00029 | 55 | -0.63719963 | AADAC | arylacetamide deacetylase [Source:HGNC Symbol;Acc:HGNC:17] |
| ENSG00000115275 | HGLibA_29633 | 55 | -0.63719963 | MOGS | mannosyl-oligosaccharide glucosidase [Source:HGNC Symbol;Acc:HGNC:24862] |
| ENSG00000116525 | HGLibA_51570 | 55 | -0.63719963 | TRIM62 | tripartite motif containing 62 [Source:HGNC Symbol;Acc:HGNC:25574] |
| ENSG00000116793 | HGLibA_36461 | 55 | -0.63719963 | PHTF1 | putative homeodomain transcription factor 1 [Source:HGNC Symbol;Acc:HGNC:8939] |
| ENSG00000116809 | HGLibA_54830 | 55 | -0.63719963 | ZBTB17 | zinc finger and BTB domain containing 17 [Source:HGNC Symbol;Acc:HGNC:12936] |
| ENSG00000116898 | HGLibA_30042 | 55 | -0.63719963 | MRPS15 | mitochondrial ribosomal protein S15 [Source:HGNC Symbol;Acc:HGNC:14504] |
| ENSG00000117090 | HGLibA_44313 | 55 | -0.63719963 | SLAMF1 | signaling lymphocytic activation molecule family member 1 [Source:HGNC Symbol;Acc:HGNC:10903] |
| ENSG00000117139 | HGLibA_24523 | 55 | -0.63719963 | KDM5B | lysine demethylase 5B [Source:HGNC Symbol;Acc:HGNC:18039] |
| ENSG00000117395 | HGLibA_14371 | 55 | -0.63719963 | EBNA1BP2 | EBNA1 binding protein 2 [Source:HGNC Symbol;Acc:HGNC:15531] |
| ENSG00000117595 | HGLibA_23595 | 55 | -0.63719963 | IRF6 | interferon regulatory factor 6 [Source:HGNC Symbol;Acc:HGNC:6121] |
| ENSG00000119314 | HGLibA_39255 | 55 | -0.63719963 | PTBP3 | polypyrimidine tract binding protein 3 [Source:HGNC Symbol;Acc:HGNC:10253] |
| ENSG00000119541 | HGLibA_53801 | 55 | -0.63719963 | VPS4B | vacuolar protein sorting 4 homolog B [Source:HGNC Symbol;Acc:HGNC:10895] |
| ENSG00000120289 | HGLibA_27951 | 55 | -0.63719963 | MAGEB4 | MAGE family member B4 [Source:HGNC Symbol;Acc:HGNC:6811] |
| ENSG00000120329 | HGLibA_44665 | 55 | -0.63719963 | SLC25A2 | solute carrier family 25 member 2 [Source:HGNC Symbol;Acc:HGNC:22921] |
| ENSG00000121068 | HGLibA_48656 | 55 | -0.63719963 | TBX2 | T-box 2 [Source:HGNC Symbol;Acc:HGNC:11597] |

| | | | | | |
|---|---|---|---|---|---|
| ENSG00000121895 | HGLibA_50013 | 55 | -0.63719963 | TMEM156 | transmembrane protein 156 [Source:HGNC Symbol;Acc:HGNC:26260] |
| ENSG00000122420 | HGLibA_39341 | 55 | -0.63719963 | PTGFR | prostaglandin F receptor [Source:HGNC Symbol;Acc:HGNC:9600] |
| ENSG00000122644 | HGLibA_02966 | 55 | -0.63719963 | ARL4A | ADP ribosylation factor like GTPase 4A [Source:HGNC Symbol;Acc:HGNC:695] |
| ENSG00000124191 | HGLibA_51038 | 55 | -0.63719963 | TOX2 | TOX high mobility group box family member 2 [Source:HGNC Symbol;Acc:HGNC:16095] |
| ENSG00000124507 | HGLibA_34961 | 55 | -0.63719963 | PACSIN1 | protein kinase C and casein kinase substrate in neurons 1 [Source:HGNC Symbol;Acc:HGNC:8570] |
| ENSG00000125414 | HGLibA_30721 | 55 | -0.63719963 | MYH2 | myosin heavy chain 2 [Source:HGNC Symbol;Acc:HGNC:7572] |
| ENSG00000125726 | HGLibA_08438 | 55 | -0.63719963 | CD70 | CD70 molecule [Source:HGNC Symbol;Acc:HGNC:11937] |
| ENSG00000125971 | HGLibA_14259 | 55 | -0.63719963 | DYNLRB1 | dynein light chain roadblock-type 1 [Source:HGNC Symbol;Acc:HGNC:15468] |
| ENSG00000126456 | HGLibA_23585 | 55 | -0.63719963 | IRF3 | interferon regulatory factor 3 [Source:HGNC Symbol;Acc:HGNC:6118] |
| ENSG00000128242 | HGLibA_18496 | 55 | -0.63719963 | GAL3ST1 | galactose-3-O-sulfotransferase 1 [Source:HGNC Symbol;Acc:HGNC:24240] |
| ENSG00000128271 | HGLibA_01012 | 55 | -0.63719963 | ADORA2A | adenosine A2a receptor [Source:HGNC Symbol;Acc:HGNC:263] |
| ENSG00000128482 | HGLibA_41468 | 55 | -0.63719963 | RNF112 | ring finger protein 112 [Source:HGNC Symbol;Acc:HGNC:12968] |
| ENSG00000129515 | HGLibA_46101 | 55 | -0.63719963 | SNX6 | sorting nexin 6 [Source:HGNC Symbol;Acc:HGNC:14970] |
| ENSG00000130066 | HGLibA_42771 | 55 | -0.63719963 | SAT1 | spermidine/spermine N1-acetyltransferase 1 [Source:HGNC Symbol;Acc:HGNC:10540] |
| ENSG00000130300 | HGLibA_37248 | 55 | -0.63719963 | PLVAP | plasmalemma vesicle associated protein [Source:HGNC Symbol;Acc:HGNC:13635] |
| ENSG00000130477 | HGLibA_52993 | 55 | -0.63719963 | UNC13A | unc-13 homolog A [Source:HGNC Symbol;Acc:HGNC:23150] |
| ENSG00000130638 | HGLibA_03832 | 55 | -0.63719963 | ATXN10 | ataxin 10 [Source:HGNC Symbol;Acc:HGNC:10549] |
| ENSG00000130787 | HGLibA_21337 | 55 | -0.63719963 | HIP1R | huntingtin interacting protein 1 related [Source:HGNC Symbol;Acc:HGNC:18415] |
| ENSG00000131725 | HGLibA_54093 | 55 | -0.63719963 | WDR44 | WD repeat domain 44 [Source:HGNC Symbol;Acc:HGNC:30512] |
| ENSG00000132386 | HGLibA_43585 | 55 | -0.63719963 | SERPINF1 | serpin family F member 1 [Source:HGNC Symbol;Acc:HGNC:8824] |
| ENSG00000133056 | HGLibA_36624 | 55 | -0.63719963 | PIK3C2B | phosphatidylinositol-4-phosphate 3-kinase catalytic subunit type 2 beta [Source:HGNC Symbol;Acc:HGNC:8972] |
| ENSG00000134308 | HGLibA_54751 | 55 | -0.63719963 | YWHAQ | tyrosine 3-monooxygenase/tryptophan 5-monooxygenase activation protein theta [Source:HGNC Symbol;Acc:HGNC:12854] |
| ENSG00000134321 | HGLibA_42300 | 55 | -0.63719963 | RSAD2 | radical S-adenosyl methionine domain containing 2 [Source:HGNC Symbol;Acc:HGNC:30908] |
| ENSG00000135317 | HGLibA_46038 | 55 | -0.63719963 | SNX14 | sorting nexin 14 [Source:HGNC Symbol;Acc:HGNC:14977] |
| ENSG00000136144 | HGLibA_40677 | 55 | -0.63719963 | RCBTB1 | RCC1 and BTB domain containing protein 1 [Source:HGNC Symbol;Acc:HGNC:18243] |
| ENSG00000136819 | HGLibA_06760 | 55 | -0.63719963 | C9orf78 | chromosome 9 open reading frame 78 [Source:HGNC Symbol;Acc:HGNC:24932] |
| ENSG00000136929 | HGLibA_21128 | 55 | -0.63719963 | HEMGN | hemogen [Source:HGNC Symbol;Acc:HGNC:17509] |
| ENSG00000137824 | HGLibA_41369 | 55 | -0.63719963 | RMDN3 | regulator of microtubule dynamics 3 [Source:HGNC Symbol;Acc:HGNC:25550] |
| ENSG00000137869 | HGLibA_12064 | 55 | -0.63719963 | CYP19A1 | cytochrome P450 family 19 subfamily A member 1 [Source:HGNC Symbol;Acc:HGNC:2594] |
| ENSG00000137996 | HGLibA_42372 | 55 | -0.63719963 | RTCA | RNA 3'-terminal phosphate cyclase [Source:HGNC Symbol;Acc:HGNC:17981] |
| ENSG00000138069 | HGLibA_39829 | 55 | -0.63719963 | RAB1A | member RAS oncogene family [Source:HGNC Symbol;Acc:HGNC:9758]" |
| ENSG00000143502 | HGLibA_47852 | 55 | -0.63719963 | SUSD4 | sushi domain containing 4 [Source:HGNC Symbol;Acc:HGNC:25470] |
| ENSG00000143947 | HGLibA_42124 | 55 | -0.63719963 | RPS27A | ribosomal protein S27a [Source:HGNC Symbol;Acc:HGNC:10417] |
| ENSG00000144118 | HGLibA_40149 | 55 | -0.63719963 | RALB | RAS like proto-oncogene B [Source:HGNC Symbol;Acc:HGNC:9840] |
| ENSG00000144369 | HGLibA_16269 | 55 | -0.63719963 | FAM171B | family with sequence similarity 171 member B [Source:HGNC Symbol;Acc:HGNC:29412] |
| ENSG00000144840 | HGLibA_40033 | 55 | -0.63719963 | RABL3 | member of RAS oncogene family like 3 [Source:HGNC Symbol;Acc:HGNC:18072]" |
| ENSG00000144909 | HGLibA_34645 | 55 | -0.63719963 | OSBPL11 | oxysterol binding protein like 11 [Source:HGNC Symbol;Acc:HGNC:16397] |
| ENSG00000144909 | HGLibA_34646 | 55 | -0.63719963 | OSBPL11 | oxysterol binding protein like 11 [Source:HGNC Symbol;Acc:HGNC:16397] |
| ENSG00000145779 | HGLibA_50718 | 55 | -0.63719963 | TNFAIP8 | TNF alpha induced protein 8 [Source:HGNC Symbol;Acc:HGNC:17260] |
| ENSG00000145780 | HGLibA_17278 | 55 | -0.63719963 | FEM1C | fem-1 homolog C [Source:HGNC Symbol;Acc:HGNC:16933] |
| ENSG00000145920 | HGLibA_10936 | 55 | -0.63719963 | CPLX2 | complexin 2 [Source:HGNC Symbol;Acc:HGNC:2310] |
| ENSG00000146054 | HGLibA_51597 | 55 | -0.63719963 | TRIM7 | tripartite motif containing 7 [Source:HGNC Symbol;Acc:HGNC:16278] |
| ENSG00000146802 | HGLibA_50045 | 55 | -0.63719963 | TMEM168 | transmembrane protein 168 [Source:HGNC Symbol;Acc:HGNC:25826] |
| ENSG00000147434 | HGLibA_09581 | 55 | -0.63719963 | CHRNA6 | cholinergic receptor nicotinic alpha 6 subunit [Source:HGNC Symbol;Acc:HGNC:15963] |
| ENSG00000147570 | HGLibA_13628 | 55 | -0.63719963 | DNAJC5B | DnaJ heat shock protein family (Hsp40) member C5 beta [Source:HGNC Symbol;Acc:HGNC:24138] |
| ENSG00000147697 | HGLibA_20411 | 55 | -0.63719963 | GSDMC | gasdermin C [Source:HGNC Symbol;Acc:HGNC:7151] |
| ENSG00000148153 | HGLibA_23313 | 55 | -0.63719963 | INIP | INTS3 and NABP interacting protein [Source:HGNC Symbol;Acc:HGNC:24994] |
| ENSG00000149289 | HGLibA_54959 | 55 | -0.63719963 | ZC3H12C | zinc finger CCCH-type containing 12C [Source:HGNC Symbol;Acc:HGNC:29362] |
| ENSG00000151465 | HGLibA_08508 | 55 | -0.63719963 | CDC123 | cell division cycle 123 [Source:HGNC Symbol;Acc:HGNC:16827] |
| ENSG00000151575 | HGLibA_49111 | 55 | -0.63719963 | TEX9 | testis expressed 9 [Source:HGNC Symbol;Acc:HGNC:29585] |
| ENSG00000152127 | HGLibA_29151 | 55 | -0.63719963 | MGAT5 | 6-mannosylglycoprotein 6-beta-N-acetylglucosaminyltransferase [Source:HGNC Symbol;Acc:HGNC:7049]" |
| ENSG00000153234 | HGLibA_32628 | 55 | -0.63719963 | NR4A2 | nuclear receptor subfamily 4 group A member 2 [Source:HGNC Symbol;Acc:HGNC:7981] |
| ENSG00000154721 | HGLibA_23930 | 55 | -0.63719963 | JAM2 | junctional adhesion molecule 2 [Source:HGNC Symbol;Acc:HGNC:14686] |
| ENSG00000155115 | HGLibA_20594 | 55 | -0.63719963 | GTF3C6 | general transcription factor IIIC subunit 6 [Source:HGNC Symbol;Acc:HGNC:20872] |
| ENSG00000158062 | HGLibA_52781 | 55 | -0.63719963 | UBXN11 | UBX domain protein 11 [Source:HGNC Symbol;Acc:HGNC:30600] |
| ENSG00000159202 | HGLibA_52675 | 55 | -0.63719963 | UBE2Z | ubiquitin conjugating enzyme E2 Z [Source:HGNC Symbol;Acc:HGNC:25847] |
| ENSG00000159267 | HGLibA_21650 | 55 | -0.63719963 | HLCS | holocarboxylase synthetase [Source:HGNC Symbol;Acc:HGNC:4976] |
| ENSG00000163515 | HGLibA_40878 | 55 | -0.63719963 | RETNLB | resistin like beta [Source:HGNC Symbol;Acc:HGNC:20388] |
| ENSG00000163884 | HGLibA_25058 | 55 | -0.63719963 | KLF15 | Kruppel like factor 15 [Source:HGNC Symbol;Acc:HGNC:14536] |
| ENSG00000164010 | HGLibA_15501 | 55 | -0.63719963 | ERMAP | erythroblast membrane associated protein (Scianna blood group) [Source:HGNC Symbol;Acc:HGNC:15743] |
| ENSG00000164082 | HGLibA_20344 | 55 | -0.63719963 | GRM2 | glutamate metabotropic receptor 2 [Source:HGNC Symbol;Acc:HGNC:4594] |
| ENSG00000165156 | HGLibA_55310 | 55 | -0.63719963 | ZHX1 | zinc fingers and homeoboxes 1 [Source:HGNC Symbol;Acc:HGNC:12871] |
| ENSG00000165478 | HGLibA_21138 | 55 | -0.63719963 | HEPACAM | hepatic and glial cell adhesion molecule [Source:HGNC Symbol;Acc:HGNC:26361] |
| ENSG00000165509 | HGLibA_27964 | 55 | -0.63719963 | MAGEC3 | MAGE family member C3 [Source:HGNC Symbol;Acc:HGNC:23798] |
| ENSG00000165527 | HGLibA_02657 | 55 | -0.63719963 | ARF6 | ADP ribosylation factor 6 [Source:HGNC Symbol;Acc:HGNC:659] |
| ENSG00000165702 | HGLibA_18943 | 55 | -0.63719963 | GFI1B | growth factor independent 1B transcriptional repressor [Source:HGNC Symbol;Acc:HGNC:4238] |
| ENSG00000165799 | HGLibA_41415 | 55 | -0.63719963 | RNASE7 | ribonuclease A family member 7 [Source:HGNC Symbol;Acc:HGNC:19278] |
| ENSG00000165861 | HGLibA_55270 | 55 | -0.63719963 | ZFYVE1 | zinc finger FYVE-type containing 1 [Source:HGNC Symbol;Acc:HGNC:13180] |
| ENSG00000165973 | HGLibA_31682 | 55 | -0.63719963 | NELL1 | neural EGFL like 1 [Source:HGNC Symbol;Acc:HGNC:7750] |
| ENSG00000167173 | HGLibA_05392 | 55 | -0.63719963 | C15orf39 | chromosome 15 open reading frame 39 [Source:HGNC Symbol;Acc:HGNC:24497] |
| ENSG00000168056 | HGLibA_27596 | 55 | -0.63719963 | LTBP3 | latent transforming growth factor beta binding protein 3 [Source:HGNC Symbol;Acc:HGNC:6716] |
| ENSG00000168067 | HGLibA_28213 | 55 | -0.63719963 | MAP4K2 | mitogen-activated protein kinase kinase kinase kinase 2 [Source:HGNC Symbol;Acc:HGNC:6864] |
| ENSG00000169067 | HGLibA_00605 | 55 | -0.63719963 | ACTBL2 | beta like 2 [Source:HGNC Symbol;Acc:HGNC:17780]" |
| ENSG00000169715 | HGLibA_30283 | 55 | -0.63719963 | MT1E | metallothionein 1E [Source:HGNC Symbol;Acc:HGNC:7397] |
| ENSG00000169715 | HGLibA_30284 | 55 | -0.63719963 | MT1E | metallothionein 1E [Source:HGNC Symbol;Acc:HGNC:7397] |
| ENSG00000169855 | HGLibA_41733 | 55 | -0.63719963 | ROBO1 | roundabout guidance receptor 1 [Source:HGNC Symbol;Acc:HGNC:10249] |
| ENSG00000171004 | HGLibA_22144 | 55 | -0.63719963 | HS6ST2 | heparan sulfate 6-O-sulfotransferase 2 [Source:HGNC Symbol;Acc:HGNC:19133] |
| ENSG00000171557 | HGLibA_17455 | 55 | -0.63719963 | FGG | fibrinogen gamma chain [Source:HGNC Symbol;Acc:HGNC:3694] |
| ENSG00000171747 | HGLibA_26369 | 55 | -0.63719963 | LGALS4 | galectin 4 [Source:HGNC Symbol;Acc:HGNC:6565] |
| ENSG00000172020 | HGLibA_18612 | 55 | -0.63719963 | GAP43 | growth associated protein 43 [Source:HGNC Symbol;Acc:HGNC:4140] |
| ENSG00000172156 | HGLibA_07964 | 55 | -0.63719963 | CCL11 | C-C motif chemokine ligand 11 [Source:HGNC Symbol;Acc:HGNC:10610] |
| ENSG00000173464 | HGLibA_41393 | 55 | -0.63719963 | RNASE11 | ribonuclease A family member 11 (inactive) [Source:HGNC Symbol;Acc:HGNC:19269] |
| ENSG00000174327 | HGLibA_44428 | 55 | -0.63719963 | SLC16A13 | solute carrier family 16 member 13 [Source:HGNC Symbol;Acc:HGNC:31037] |
| ENSG00000174796 | HGLibA_49306 | 55 | -0.63719963 | THAP6 | THAP domain containing 6 [Source:HGNC Symbol;Acc:HGNC:23189] |
| ENSG00000174799 | HGLibA_09106 | 55 | -0.63719963 | CEP135 | centrosomal protein 135 [Source:HGNC Symbol;Acc:HGNC:29086] |
| ENSG00000174970 | HGLibA_33482 | 55 | -0.63719963 | OR10AG1 | olfactory receptor family 10 subfamily AG member 1 [Source:HGNC Symbol;Acc:HGNC:19607] |
| ENSG00000175215 | HGLibA_11612 | 55 | -0.63719963 | CTDSP2 | CTD small phosphatase 2 [Source:HGNC Symbol;Acc:HGNC:17077] |
| ENSG00000175482 | HGLibA_37502 | 55 | -0.63719963 | POLD4 | accessory subunit [Source:HGNC Symbol;Acc:HGNC:14106]" |
| ENSG00000175787 | HGLibA_55519 | 55 | -0.63719963 | ZNF169 | zinc finger protein 169 [Source:HGNC Symbol;Acc:HGNC:12957] |
| ENSG00000176444 | HGLibA_10058 | 55 | -0.63719963 | CLK2 | CDC like kinase 2 [Source:HGNC Symbol;Acc:HGNC:2069] |
| ENSG00000176871 | HGLibA_54418 | 55 | -0.63719963 | WSB2 | WD repeat and SOCS box containing 2 [Source:HGNC Symbol;Acc:HGNC:19222] |
| ENSG00000179456 | HGLibA_54833 | 55 | -0.63719963 | ZBTB18 | zinc finger and BTB domain containing 18 [Source:HGNC Symbol;Acc:HGNC:13030] |
| ENSG00000179902 | HGLibA_05817 | 55 | -0.63719963 | C1orf194 | chromosome 1 open reading frame 194 [Source:HGNC Symbol;Acc:HGNC:32331] |
| ENSG00000181499 | HGLibA_34446 | 55 | -0.63719963 | OR6T1 | olfactory receptor family 6 subfamily T member 1 [Source:HGNC Symbol;Acc:HGNC:14848] |
| ENSG00000182400 | HGLibA_51333 | 55 | -0.63719963 | TRAPPC6B | trafficking protein particle complex 6B [Source:HGNC Symbol;Acc:HGNC:23066] |
| ENSG00000182670 | HGLibA_52102 | 55 | -0.63719963 | TTC3 | tetratricopeptide repeat domain 3 [Source:HGNC Symbol;Acc:HGNC:12393] |
| ENSG00000183018 | HGLibA_46736 | 55 | -0.63719963 | SPNS2 | sphingolipid transporter 2 [Source:HGNC Symbol;Acc:HGNC:26992] |
| ENSG00000183054 | HGLibA_41041 | 55 | -0.63719963 | RGPD6 | RANBP2-like and GRIP domain containing 6 [Source:HGNC Symbol;Acc:HGNC:32419] |
| ENSG00000183695 | HGLibA_29848 | 55 | -0.63719963 | MRGPRX2 | MAS related GPR family member X2 [Source:HGNC Symbol;Acc:HGNC:17983] |
| ENSG00000183734 | HGLibA_03270 | 55 | -0.63719963 | ASCL2 | achaete-scute family bHLH transcription factor 2 [Source:HGNC Symbol;Acc:HGNC:739] |
| ENSG00000184343 | HGLibA_47023 | 55 | -0.63719963 | SRPK3 | SRSF protein kinase 3 [Source:HGNC Symbol;Acc:HGNC:11402] |
| ENSG00000184601 | HGLibA_05348 | 55 | -0.63719963 | C14orf180 | chromosome 14 open reading frame 180 [Source:HGNC Symbol;Acc:HGNC:33795] |
| ENSG00000184650 | HGLibA_33296 | 55 | -0.63719963 | ODF4 | outer dense fiber of sperm tails 4 [Source:HGNC Symbol;Acc:HGNC:19056] |
| ENSG00000184828 | HGLibA_54921 | 55 | -0.63719963 | ZBTB7C | zinc finger and BTB domain containing 7C [Source:HGNC Symbol;Acc:HGNC:31700] |
| ENSG00000184933 | HGLibA_34371 | 55 | -0.63719963 | OR6A2 | olfactory receptor family 6 subfamily A member 2 [Source:HGNC Symbol;Acc:HGNC:15301] |
| ENSG00000185305 | HGLibA_02945 | 55 | -0.63719963 | ARL15 | ADP ribosylation factor like GTPase 15 [Source:HGNC Symbol;Acc:HGNC:25945] |
| ENSG00000185739 | HGLibA_46991 | 55 | -0.63719963 | SRL | sarcalumenin [Source:HGNC Symbol;Acc:HGNC:11295] |
| ENSG00000186416 | HGLibA_32022 | 55 | -0.63719963 | NKRF | NFKB repressing factor [Source:HGNC Symbol;Acc:HGNC:19374] |
| ENSG00000186453 | HGLibA_16534 | 55 | -0.63719963 | FAM228A | family with sequence similarity 228 member A [Source:HGNC Symbol;Acc:HGNC:34418] |
| ENSG00000187554 | HGLibA_49655 | 55 | -0.63719963 | TLR5 | toll like receptor 5 [Source:HGNC Symbol;Acc:HGNC:11851] |
| ENSG00000187626 | HGLibA_55354 | 55 | -0.63719963 | ZKSCAN4 | zinc finger with KRAB and SCAN domains 4 [Source:HGNC Symbol;Acc:HGNC:13854] |
| ENSG00000187701 | HGLibA_33867 | 55 | -0.63719963 | OR2T27 | olfactory receptor family 2 subfamily T member 27 [Source:HGNC Symbol;Acc:HGNC:31252] |
| ENSG00000188649 | HGLibA_07485 | 55 | -0.63719963 | CC2D2B | coiled-coil and C2 domain containing 2B [Source:HGNC Symbol;Acc:HGNC:31666] |
| ENSG00000188828 | HGLibA_19306 | 55 | -0.63719963 | GLRA4 | glycine receptor alpha 4 [Source:HGNC Symbol;Acc:HGNC:31715] |
| ENSG00000188895 | HGLibA_30210 | 55 | -0.63719963 | MSL1 | male specific lethal 1 homolog [Source:HGNC Symbol;Acc:HGNC:27905] |
| ENSG00000189410 | HGLibA_43903 | 55 | -0.63719963 | SH2D5 | SH2 domain containing 5 [Source:HGNC Symbol;Acc:HGNC:28819] |
| ENSG00000196242 | HGLibA_33778 | 55 | -0.63719963 | OR2C3 | olfactory receptor family 2 subfamily C member 3 [Source:HGNC Symbol;Acc:HGNC:15005] |
| ENSG00000196357 | HGLibA_56256 | 55 | -0.63719963 | ZNF565 | zinc finger protein 565 [Source:HGNC Symbol;Acc:HGNC:26726] |
| ENSG00000196368 | HGLibA_32936 | 55 | -0.63719963 | NUDT11 | nudix hydrolase 11 [Source:HGNC Symbol;Acc:HGNC:18011] |
| ENSG00000197429 | HGLibA_23482 | 55 | -0.63719963 | IPP | intracisternal A particle-promoted polypeptide [Source:HGNC Symbol;Acc:HGNC:6108] |
| ENSG00000197919 | HGLibA_22655 | 55 | -0.63719963 | IFNA1 | interferon alpha 1 [Source:HGNC Symbol;Acc:HGNC:5417] |
| ENSG00000197961 | HGLibA_55452 | 55 | -0.63719963 | ZNF121 | zinc finger protein 121 [Source:HGNC Symbol;Acc:HGNC:12904] |
| ENSG00000197977 | HGLibA_14960 | 55 | -0.63719963 | ELOVL2 | ELOVL fatty acid elongase 2 [Source:HGNC Symbol;Acc:HGNC:14416] |
| ENSG00000198142 | HGLibA_46219 | 55 | -0.63719963 | SOWAHC | sosondowah ankyrin repeat domain family member C [Source:HGNC Symbol;Acc:HGNC:26149] |
| ENSG00000204314 | HGLibA_38880 | 55 | -0.63719963 | PRRT1 | proline rich transmembrane protein 1 [Source:HGNC Symbol;Acc:HGNC:13943] |
| ENSG00000204560 | HGLibA_13170 | 55 | -0.63719963 | DHX16 | DEAH-box helicase 16 [Source:HGNC Symbol;Acc:HGNC:2739] |
| ENSG00000204560 | HGLibA_13169 | 55 | -0.63719963 | DHX16 | DEAH-box helicase 16 [Source:HGNC Symbol;Acc:HGNC:2739] |
| ENSG00000204659 | HGLibA_07471 | 55 | -0.63719963 | CBY3 | chibby family member 3 [Source:HGNC Symbol;Acc:HGNC:33278] |
| ENSG00000204695 | HGLibA_33639 | 55 | -0.63719963 | OR14J1 | olfactory receptor family 14 subfamily J member 1 [Source:HGNC Symbol;Acc:HGNC:13971] |
| ENSG00000211452 | HGLibA_13237 | 55 | -0.63719963 | DIO1 | iodothyronine deiodinase 1 [Source:HGNC Symbol;Acc:HGNC:2883] |
| ENSG00000212933 | HGLibA_25638 | 55 | -0.63719963 | KRTAP12-4 | keratin associated protein 12-4 [Source:HGNC Symbol;Acc:HGNC:20532] |
| ENSG00000213398 | HGLibA_26097 | 55 | -0.63719963 | LCAT | lecithin-cholesterol acyltransferase [Source:HGNC Symbol;Acc:HGNC:6522] |
| ENSG00000213889 | HGLibA_37995 | 55 | -0.63719963 | PPM1N | Mg2+/Mn2+ dependent 1N (putative) [Source:HGNC Symbol;Acc:HGNC:26845]" |
| ENSG00000214140 | HGLibA_38355 | 55 | -0.63719963 | PRCD | photoreceptor disc component [Source:HGNC Symbol;Acc:HGNC:32528] |
| ENSG00000215029 | HGLibA_48850 | 55 | -0.63719963 | TCP11X2 | X-linked 2 [Source:HGNC Symbol;Acc:HGNC:48335]" |

| | | | | | |
|---|---|---|---|---|---|
| ENSG00000215131 | HGLibA_05497 | 55 | -0.63719963 | C16orf90 | chromosome 16 open reading frame 90 [Source:HGNC Symbol;Acc:HGNC:34455] |
| ENSG00000228075 | HGLibA_04675 | 55 | -0.63719963 | BOD1L2 | biorientation of chromosomes in cell division 1 like 2 [Source:HGNC Symbol;Acc:HGNC:28505] |
| ENSG00000230937 | HGLibA_29300 | 55 | -0.63719963 | MIR205HG | MIR205 host gene [Source:HGNC Symbol;Acc:HGNC:43562] |
| ENSG00000234278 | HGLibA_38813 | 55 | -0.63719963 | PRR20E | proline rich 20E [Source:HGNC Symbol;Acc:HGNC:37223] |
| ENSG00000237957 | HGLibA_11537 | 55 | -0.63719963 | CT47A5 | member A5 [Source:HGNC Symbol;Acc:HGNC:33286]" |
| ENSG00000239779 | HGLibA_53968 | 55 | -0.63719963 | WBP1 | WW domain binding protein 1 [Source:HGNC Symbol;Acc:HGNC:12737] |
| ENSG00000240682 | HGLibA_23693 | 55 | -0.63719963 | ISY1 | ISY1 splicing factor homolog [Source:HGNC Symbol;Acc:HGNC:29201] |
| ENSG00000242362 | HGLibA_11528 | 55 | -0.63719963 | CT47A2 | member A2 [Source:HGNC Symbol;Acc:HGNC:33283]" |
| ENSG00000242441 | HGLibA_20522 | 55 | -0.63719963 | GTF2A1L | general transcription factor IIA subunit 1 like [Source:HGNC Symbol;Acc:HGNC:30727] |
| ENSG00000243480 | HGLibA_01849 | 55 | -0.63719963 | AMY2A | alpha 2A (pancreatic) [Source:HGNC Symbol;Acc:HGNC:477]" |
| ENSG00000257949 | HGLibA_48988 | 55 | -0.63719963 | TEN1 | CST complex subunit [Source:HGNC Symbol;Acc:HGNC:37242]" |
| ENSG00000264424 | HGLibA_30726 | 55 | -0.63719963 | MYH4 | myosin heavy chain 4 [Source:HGNC Symbol;Acc:HGNC:7574] |
| ENSG00000277481 | HGLibA_36795 | 55 | -0.63719963 | PKD1L3 | transient receptor potential channel interacting [Source:HGNC Symbol;Acc:HGNC:21716]" |
| ENSG00000278540 | HGLibA_00346 | 55 | -0.63719963 | ACACA | acetyl-CoA carboxylase alpha [Source:HGNC Symbol;Acc:HGNC:84] |
| ENSG00000279000 | HGLibA_33474 | 55 | -0.63719963 | OR10A6 | olfactory receptor family 10 subfamily A member 6 (gene/pseudogene) [Source:HGNC Symbol;Acc:HGNC:15132] |
| FAM159B | HGLibA_16200 | 55 | -0.63719963 | FAM159B | - |
| FAM63A | HGLibA_16658 | 55 | -0.63719963 | FAM63A | - |
| HIATL1 | HGLibA_21280 | 55 | -0.63719963 | HIATL1 | - |
| HIST1H4F | HGLibA_21502 | 55 | -0.63719963 | HIST1H4F | - |
| hsa-mir-1229 | HGLibA_57313 | 55 | -0.63719963 | hsa-mir-1229 | - |
| hsa-mir-1231 | HGLibA_57320 | 55 | -0.63719963 | hsa-mir-1231 | - |
| hsa-mir-1233-2 | HGLibA_57325 | 55 | -0.63719963 | hsa-mir-1233-2 | - |
| hsa-mir-1909 | HGLibA_58032 | 55 | -0.63719963 | hsa-mir-1909 | - |
| hsa-mir-190a | HGLibA_58036 | 55 | -0.63719963 | hsa-mir-190a | - |
| hsa-mir-215 | HGLibA_58252 | 55 | -0.63719963 | hsa-mir-215 | - |
| hsa-mir-374b | HGLibA_59395 | 55 | -0.63719963 | hsa-mir-374b | - |
| hsa-mir-3973 | HGLibA_59652 | 55 | -0.63719963 | hsa-mir-3973 | - |
| hsa-mir-4262 | HGLibA_59755 | 55 | -0.63719963 | hsa-mir-4262 | - |
| hsa-mir-4421 | HGLibA_60072 | 55 | -0.63719963 | hsa-mir-4421 | - |
| hsa-mir-4452 | HGLibA_60214 | 55 | -0.63719963 | hsa-mir-4452 | - |
| hsa-mir-450a-2 | HGLibA_60476 | 55 | -0.63719963 | hsa-mir-450a-2 | - |
| hsa-mir-4514 | HGLibA_60500 | 55 | -0.63719963 | hsa-mir-4514 | - |
| hsa-mir-4536-1 | HGLibA_60603 | 55 | -0.63719963 | hsa-mir-4536-1 | - |
| hsa-mir-4722 | HGLibA_60984 | 55 | -0.63719963 | hsa-mir-4722 | - |
| hsa-mir-4801 | HGLibA_61309 | 55 | -0.63719963 | hsa-mir-4801 | - |
| hsa-mir-516a-1 | HGLibA_61607 | 55 | -0.63719963 | hsa-mir-516a-1 | - |
| hsa-mir-516b-1 | HGLibA_61615 | 55 | -0.63719963 | hsa-mir-516b-1 | - |
| hsa-mir-548i-4 | HGLibA_62025 | 55 | -0.63719963 | hsa-mir-548i-4 | - |
| hsa-mir-550b-2 | HGLibA_62114 | 55 | -0.63719963 | hsa-mir-550b-2 | - |
| hsa-mir-555 | HGLibA_62138 | 55 | -0.63719963 | hsa-mir-555 | - |
| hsa-mir-5692a-1 | HGLibA_62288 | 55 | -0.63719963 | hsa-mir-5692a-1 | - |
| hsa-mir-636 | HGLibA_62782 | 55 | -0.63719963 | hsa-mir-636 | - |
| hsa-mir-6505 | HGLibA_62869 | 55 | -0.63719963 | hsa-mir-6505 | - |
| hsa-mir-6717 | HGLibA_63028 | 55 | -0.63719963 | hsa-mir-6717 | - |
| hsa-mir-6799 | HGLibA_63373 | 55 | -0.63719963 | hsa-mir-6799 | - |
| hsa-mir-6806 | HGLibA_63403 | 55 | -0.63719963 | hsa-mir-6806 | - |
| hsa-mir-6868 | HGLibA_63656 | 55 | -0.63719963 | hsa-mir-6868 | - |
| hsa-mir-7152 | HGLibA_63836 | 55 | -0.63719963 | hsa-mir-7152 | - |
| hsa-mir-8068 | HGLibA_64111 | 55 | -0.63719963 | hsa-mir-8068 | - |
| hsa-mir-933 | HGLibA_64306 | 55 | -0.63719963 | hsa-mir-933 | - |
| IMPAD1 | HGLibA_23253 | 55 | -0.63719963 | IMPAD1 | - |
| LOC286238 | HGLibA_26918 | 55 | -0.63719963 | LOC286238 | - |
| MESDC1 | HGLibA_28911 | 55 | -0.63719963 | MESDC1 | - |
| METTL21D | HGLibA_28985 | 55 | -0.63719963 | METTL21D | - |
| PROSC | HGLibA_38693 | 55 | -0.63719963 | PROSC | - |
| SCXB | HGLibA_43083 | 55 | -0.63719963 | SCXB | - |
| XAGE1E | HGLibA_54477 | 55 | -0.63719963 | XAGE1E | - |
| ANXA8L2 | HGLibA_02257 | 54 | -0.646244973 | ANXA8L2 | - |
| ATP5S | HGLibA_03688 | 54 | -0.646244973 | ATP5S | - |
| C14orf80 | HGLibA_05373 | 54 | -0.646244973 | C14orf80 | - |
| C15orf57 | HGLibA_05419 | 54 | -0.646244973 | C15orf57 | - |
| C16orf13 | HGLibA_05441 | 54 | -0.646244973 | C16orf13 | - |
| C2orf62 | HGLibA_06158 | 54 | -0.646244973 | C2orf62 | - |
| C8orf47 | HGLibA_06633 | 54 | -0.646244973 | C8orf47 | - |
| ENSG00000000003 | HGLibA_51943 | 54 | -0.646244973 | TSPAN6 | tetraspanin 6 [Source:HGNC Symbol;Acc:HGNC:11858] |
| ENSG00000008282 | HGLibA_48036 | 54 | -0.646244973 | SYPL1 | synaptophysin like 1 [Source:HGNC Symbol;Acc:HGNC:11507] |
| ENSG00000021826 | HGLibA_10990 | 54 | -0.646244973 | CPS1 | carbamoyl-phosphate synthase 1 [Source:HGNC Symbol;Acc:HGNC:2323] |
| ENSG00000033122 | HGLibA_27412 | 54 | -0.646244973 | LRRC7 | leucine rich repeat containing 7 [Source:HGNC Symbol;Acc:HGNC:18531] |
| ENSG00000048162 | HGLibA_32307 | 54 | -0.646244973 | NOP16 | NOP16 nucleolar protein [Source:HGNC Symbol;Acc:HGNC:26934] |
| ENSG00000053108 | HGLibA_18106 | 54 | -0.646244973 | FSTL4 | follistatin like 4 [Source:HGNC Symbol;Acc:HGNC:21389] |
| ENSG00000054392 | HGLibA_21251 | 54 | -0.646244973 | HHAT | hedgehog acyltransferase [Source:HGNC Symbol;Acc:HGNC:18270] |
| ENSG00000057294 | HGLibA_36856 | 54 | -0.646244973 | PKP2 | plakophilin 2 [Source:HGNC Symbol;Acc:HGNC:9024] |
| ENSG00000062096 | HGLibA_03141 | 54 | -0.646244973 | ARSF | arylsulfatase F [Source:HGNC Symbol;Acc:HGNC:721] |
| ENSG00000063046 | HGLibA_14814 | 54 | -0.646244973 | EIF4B | eukaryotic translation initiation factor 4B [Source:HGNC Symbol;Acc:HGNC:3285] |
| ENSG00000065717 | HGLibA_49609 | 54 | -0.646244973 | TLE2 | transducin like enhancer of split 2 [Source:HGNC Symbol;Acc:HGNC:11838] |
| ENSG00000065809 | HGLibA_16004 | 54 | -0.646244973 | FAM107B | family with sequence similarity 107 member B [Source:HGNC Symbol;Acc:HGNC:23726] |
| ENSG00000066322 | HGLibA_14957 | 54 | -0.646244973 | ELOVL1 | ELOVL fatty acid elongase 1 [Source:HGNC Symbol;Acc:HGNC:14418] |
| ENSG00000075673 | HGLibA_03563 | 54 | -0.646244973 | ATP12A | ATPase H+/K+ transporting non-gastric alpha2 subunit [Source:HGNC Symbol;Acc:HGNC:13816] |
| ENSG00000081014 | HGLibA_02343 | 54 | -0.646244973 | AP4E1 | adaptor related protein complex 4 subunit epsilon 1 [Source:HGNC Symbol;Acc:HGNC:573] |
| ENSG00000082146 | HGLibA_47576 | 54 | -0.646244973 | STRADB | STE20-related kinase adaptor beta [Source:HGNC Symbol;Acc:HGNC:13205] |
| ENSG00000085224 | HGLibA_03826 | 54 | -0.646244973 | ATRX | chromatin remodeler [Source:HGNC Symbol;Acc:HGNC:886]" |
| ENSG00000089597 | HGLibA_18605 | 54 | -0.646244973 | GANAB | glucosidase II alpha subunit [Source:HGNC Symbol;Acc:HGNC:4138] |
| ENSG00000092470 | HGLibA_54178 | 54 | -0.646244973 | WDR76 | WD repeat domain 76 [Source:HGNC Symbol;Acc:HGNC:25773] |
| ENSG00000099204 | HGLibA_00309 | 54 | -0.646244973 | ABLIM1 | actin binding LIM protein 1 [Source:HGNC Symbol;Acc:HGNC:78] |
| ENSG00000099617 | HGLibA_14585 | 54 | -0.646244973 | EFNA2 | ephrin A2 [Source:HGNC Symbol;Acc:HGNC:3222] |
| ENSG00000099769 | HGLibA_22812 | 54 | -0.646244973 | IGFALS | insulin like growth factor binding protein acid labile subunit [Source:HGNC Symbol;Acc:HGNC:5468] |
| ENSG00000100092 | HGLibA_43925 | 54 | -0.646244973 | SH3BP1 | SH3 domain binding protein 1 [Source:HGNC Symbol;Acc:HGNC:10824] |
| ENSG00000100412 | HGLibA_00465 | 54 | -0.646244973 | ACO2 | aconitase 2 [Source:HGNC Symbol;Acc:HGNC:118] |
| ENSG00000100897 | HGLibA_12414 | 54 | -0.646244973 | DCAF11 | DDB1 and CUL4 associated factor 11 [Source:HGNC Symbol;Acc:HGNC:20258] |
| ENSG00000100916 | HGLibA_04833 | 54 | -0.646244973 | BRMS1L | BRMS1 like transcriptional repressor [Source:HGNC Symbol;Acc:HGNC:20512] |
| ENSG00000102172 | HGLibA_45829 | 54 | -0.646244973 | SMS | spermine synthase [Source:HGNC Symbol;Acc:HGNC:11123] |
| ENSG00000102781 | HGLibA_24076 | 54 | -0.646244973 | KATNAL1 | katanin catalytic subunit A1 like 1 [Source:HGNC Symbol;Acc:HGNC:28361] |
| ENSG00000102878 | HGLibA_22230 | 54 | -0.646244973 | HSF4 | heat shock transcription factor 4 [Source:HGNC Symbol;Acc:HGNC:5227] |
| ENSG00000103005 | HGLibA_53162 | 54 | -0.646244973 | USB1 | U6 snRNA biogenesis phosphodiesterase 1 [Source:HGNC Symbol;Acc:HGNC:25792] |
| ENSG00000103479 | HGLibA_40469 | 54 | -0.646244973 | RBL2 | RB transcriptional corepressor like 2 [Source:HGNC Symbol;Acc:HGNC:9894] |
| ENSG00000103657 | HGLibA_21152 | 54 | -0.646244973 | HERC1 | HECT and RLD domain containing E3 ubiquitin protein ligase family member 1 [Source:HGNC Symbol;Acc:HGNC:4867] |
| ENSG00000105197 | HGLibA_49520 | 54 | -0.646244973 | TIMM50 | translocase of inner mitochondrial membrane 50 [Source:HGNC Symbol;Acc:HGNC:23656] |
| ENSG00000105492 | HGLibA_44144 | 54 | -0.646244973 | SIGLEC6 | sialic acid binding Ig like lectin 6 [Source:HGNC Symbol;Acc:HGNC:10875] |
| ENSG00000105856 | HGLibA_20935 | 54 | -0.646244973 | HBP1 | HMG-box transcription factor 1 [Source:HGNC Symbol;Acc:HGNC:23200] |
| ENSG00000107779 | HGLibA_04629 | 54 | -0.646244973 | BMPR1A | bone morphogenetic protein receptor type 1A [Source:HGNC Symbol;Acc:HGNC:1076] |
| ENSG00000108963 | HGLibA_13836 | 54 | -0.646244973 | DPH1 | diphthamide biosynthesis 1 [Source:HGNC Symbol;Acc:HGNC:3003] |
| ENSG00000109321 | HGLibA_02639 | 54 | -0.646244973 | AREG | amphiregulin [Source:HGNC Symbol;Acc:HGNC:651] |
| ENSG00000109814 | HGLibA_52864 | 54 | -0.646244973 | UGDH | UDP-glucose 6-dehydrogenase [Source:HGNC Symbol;Acc:HGNC:12525] |
| ENSG00000109919 | HGLibA_30328 | 54 | -0.646244973 | MTCH2 | mitochondrial carrier 2 [Source:HGNC Symbol;Acc:HGNC:17587] |
| ENSG00000110651 | HGLibA_08456 | 54 | -0.646244973 | CD81 | CD81 molecule [Source:HGNC Symbol;Acc:HGNC:1701] |
| ENSG00000111319 | HGLibA_43013 | 54 | -0.646244973 | SCNN1A | sodium channel epithelial 1 alpha subunit [Source:HGNC Symbol;Acc:HGNC:10599] |
| ENSG00000112294 | HGLibA_01577 | 54 | -0.646244973 | ALDH5A1 | aldehyde dehydrogenase 5 family member A1 [Source:HGNC Symbol;Acc:HGNC:408] |
| ENSG00000113141 | HGLibA_22910 | 54 | -0.646244973 | IK | IK cytokine [Source:HGNC Symbol;Acc:HGNC:5958] |
| ENSG00000114480 | HGLibA_18719 | 54 | -0.646244973 | GBE1 | 4-alpha-glucan branching enzyme 1 [Source:HGNC Symbol;Acc:HGNC:4180]" |
| ENSG00000117411 | HGLibA_04027 | 54 | -0.646244973 | B4GALT2 | 4-galactosyltransferase 2 [Source:HGNC Symbol;Acc:HGNC:925]" |
| ENSG00000119777 | HGLibA_50216 | 54 | -0.646244973 | TMEM214 | transmembrane protein 214 [Source:HGNC Symbol;Acc:HGNC:25983] |
| ENSG00000120094 | HGLibA_21926 | 54 | -0.646244973 | HOXB1 | homeobox B1 [Source:HGNC Symbol;Acc:HGNC:5111] |
| ENSG00000123143 | HGLibA_36838 | 54 | -0.646244973 | PKN1 | protein kinase N1 [Source:HGNC Symbol;Acc:HGNC:9405] |
| ENSG00000124596 | HGLibA_33194 | 54 | -0.646244973 | OARD1 | O-acyl-ADP-ribose deacylase 1 [Source:HGNC Symbol;Acc:HGNC:21257] |
| ENSG00000125968 | HGLibA_22525 | 54 | -0.646244973 | ID1 | HLH protein [Source:HGNC Symbol;Acc:HGNC:5360]" |
| ENSG00000126500 | HGLibA_17654 | 54 | -0.646244973 | FLRT1 | fibronectin leucine rich transmembrane protein 1 [Source:HGNC Symbol;Acc:HGNC:3760] |
| ENSG00000130714 | HGLibA_37669 | 54 | -0.646244973 | POMT1 | protein O-mannosyltransferase 1 [Source:HGNC Symbol;Acc:HGNC:9202] |
| ENSG00000131153 | HGLibA_19107 | 54 | -0.646244973 | GINS2 | GINS complex subunit 2 [Source:HGNC Symbol;Acc:HGNC:24575] |
| ENSG00000132514 | HGLibA_09923 | 54 | -0.646244973 | CLEC10A | C-type lectin domain containing 10A [Source:HGNC Symbol;Acc:HGNC:16916] |
| ENSG00000133138 | HGLibA_48568 | 54 | -0.646244973 | TBC1D8B | TBC1 domain family member 8B [Source:HGNC Symbol;Acc:HGNC:24715] |
| ENSG00000134278 | HGLibA_46723 | 54 | -0.646244973 | SPIRE1 | spire type actin nucleation factor 1 [Source:HGNC Symbol;Acc:HGNC:30622] |
| ENSG00000135862 | HGLibA_25967 | 54 | -0.646244973 | LAMC1 | laminin subunit gamma 1 [Source:HGNC Symbol;Acc:HGNC:6492] |
| ENSG00000136518 | HGLibA_00621 | 54 | -0.646244973 | ACTL6A | actin like 6A [Source:HGNC Symbol;Acc:HGNC:24124] |
| ENSG00000136715 | HGLibA_42713 | 54 | -0.646244973 | SAP130 | Sin3A associated protein 130 [Source:HGNC Symbol;Acc:HGNC:29813] |
| ENSG00000136869 | HGLibA_49652 | 54 | -0.646244973 | TLR4 | toll like receptor 4 [Source:HGNC Symbol;Acc:HGNC:11850] |
| ENSG00000136883 | HGLibA_24852 | 54 | -0.646244973 | KIF12 | kinesin family member 12 [Source:HGNC Symbol;Acc:HGNC:21495] |
| ENSG00000136960 | HGLibA_15171 | 54 | -0.646244973 | ENPP2 | ectonucleotide pyrophosphatase/phosphodiesterase 2 [Source:HGNC Symbol;Acc:HGNC:3357] |
| ENSG00000137460 | HGLibA_17473 | 54 | -0.646244973 | FHDC1 | FH2 domain containing 1 [Source:HGNC Symbol;Acc:HGNC:29363] |
| ENSG00000137674 | HGLibA_29509 | 54 | -0.646244973 | MMP20 | matrix metallopeptidase 20 [Source:HGNC Symbol;Acc:HGNC:7167] |
| ENSG00000138028 | HGLibA_09325 | 54 | -0.646244973 | CGREF1 | cell growth regulator with EF-hand domain 1 [Source:HGNC Symbol;Acc:HGNC:16962] |
| ENSG00000138092 | HGLibA_09076 | 54 | -0.646244973 | CENPO | centromere protein O [Source:HGNC Symbol;Acc:HGNC:28152] |
| ENSG00000139445 | HGLibA_17917 | 54 | -0.646244973 | FOXN4 | forkhead box N4 [Source:HGNC Symbol;Acc:HGNC:21399] |
| ENSG00000139797 | HGLibA_41475 | 54 | -0.646244973 | RNF113B | ring finger protein 113B [Source:HGNC Symbol;Acc:HGNC:17267] |
| ENSG00000140044 | HGLibA_23942 | 54 | -0.646244973 | JDP2 | Jun dimerization protein 2 [Source:HGNC Symbol;Acc:HGNC:17546] |
| ENSG00000140403 | HGLibA_13519 | 54 | -0.646244973 | DNAJA4 | DnaJ heat shock protein family (Hsp40) member A4 [Source:HGNC Symbol;Acc:HGNC:14885] |

| | | | | | |
|---|---|---|---|---|---|
| ENSG00000140443 | HGLibA_22795 | 54 | -0.646244973 | IGF1R | insulin like growth factor 1 receptor [Source:HGNC Symbol;Acc:HGNC:5465] |
| ENSG00000140749 | HGLibA_22899 | 54 | -0.646244973 | IGSF6 | immunoglobulin superfamily member 6 [Source:HGNC Symbol;Acc:HGNC:5953] |
| ENSG00000141447 | HGLibA_34648 | 54 | -0.646244973 | OSBPL1A | oxysterol binding protein like 1A [Source:HGNC Symbol;Acc:HGNC:16398] |
| ENSG00000141458 | HGLibA_32419 | 54 | -0.646244973 | NPC1 | NPC intracellular cholesterol transporter 1 [Source:HGNC Symbol;Acc:HGNC:7897] |
| ENSG00000141959 | HGLibA_36164 | 54 | -0.646244973 | PFKL | liver type [Source:HGNC Symbol;Acc:HGNC:8876]" |
| ENSG00000142235 | HGLibA_26730 | 54 | -0.646244973 | LMTK3 | lemur tyrosine kinase 3 [Source:HGNC Symbol;Acc:HGNC:19295] |
| ENSG00000142623 | HGLibA_34970 | 54 | -0.646244973 | PADI1 | peptidyl arginine deiminase 1 [Source:HGNC Symbol;Acc:HGNC:18367] |
| ENSG00000142937 | HGLibA_42190 | 54 | -0.646244973 | RPS8 | ribosomal protein S8 [Source:HGNC Symbol;Acc:HGNC:10441] |
| ENSG00000143156 | HGLibA_32172 | 54 | -0.646244973 | NME7 | NME/NM23 family member 7 [Source:HGNC Symbol;Acc:HGNC:20461] |
| ENSG00000143486 | HGLibA_14749 | 54 | -0.646244973 | EIF2D | eukaryotic translation initiation factor 2D [Source:HGNC Symbol;Acc:HGNC:6583] |
| ENSG00000143603 | HGLibA_24359 | 54 | -0.646244973 | KCNN3 | potassium calcium-activated channel subfamily N member 3 [Source:HGNC Symbol;Acc:HGNC:6292] |
| ENSG00000144227 | HGLibA_33165 | 54 | -0.646244973 | NXPH2 | neurexophilin 2 [Source:HGNC Symbol;Acc:HGNC:8076] |
| ENSG00000146066 | HGLibA_21316 | 54 | -0.646244973 | HIGD2A | HIG1 hypoxia inducible domain family member 2A [Source:HGNC Symbol;Acc:HGNC:28311] |
| ENSG00000148634 | HGLibA_21161 | 54 | -0.646244973 | HERC4 | HECT and RLD domain containing E3 ubiquitin protein ligase 4 [Source:HGNC Symbol;Acc:HGNC:24521] |
| ENSG00000148842 | HGLibA_10317 | 54 | -0.646244973 | CNNM2 | cyclin and CBS domain divalent metal cation transport mediator 2 [Source:HGNC Symbol;Acc:HGNC:103] |
| ENSG00000149084 | HGLibA_22177 | 54 | -0.646244973 | HSD17B12 | hydroxysteroid 17-beta dehydrogenase 12 [Source:HGNC Symbol;Acc:HGNC:18646] |
| ENSG00000149554 | HGLibA_09420 | 54 | -0.646244973 | CHEK1 | checkpoint kinase 1 [Source:HGNC Symbol;Acc:HGNC:1925] |
| ENSG00000149782 | HGLibA_36983 | 54 | -0.646244973 | PLCB3 | phospholipase C beta 3 [Source:HGNC Symbol;Acc:HGNC:9056] |
| ENSG00000150593 | HGLibA_35733 | 54 | -0.646244973 | PDCD4 | programmed cell death 4 [Source:HGNC Symbol;Acc:HGNC:8763] |
| ENSG00000150753 | HGLibA_08204 | 54 | -0.646244973 | CCT5 | chaperonin containing TCP1 subunit 5 [Source:HGNC Symbol;Acc:HGNC:1618] |
| ENSG00000151224 | HGLibA_28416 | 54 | -0.646244973 | MAT1A | methionine adenosyltransferase 1A [Source:HGNC Symbol;Acc:HGNC:6903] |
| ENSG00000151748 | HGLibA_42784 | 54 | -0.646244973 | SAV1 | salvador family WW domain containing protein 1 [Source:HGNC Symbol;Acc:HGNC:17795] |
| ENSG00000151846 | HGLibA_34934 | 54 | -0.646244973 | PABPC3 | poly(A) binding protein cytoplasmic 3 [Source:HGNC Symbol;Acc:HGNC:8556] |
| ENSG00000152413 | HGLibA_21865 | 54 | -0.646244973 | HOMER1 | homer scaffold protein 1 [Source:HGNC Symbol;Acc:HGNC:17512] |
| ENSG00000152518 | HGLibA_55210 | 54 | -0.646244973 | ZFP36L2 | ZFP36 ring finger protein like 2 [Source:HGNC Symbol;Acc:HGNC:1108] |
| ENSG00000152611 | HGLibA_07203 | 54 | -0.646244973 | CAPSL | calcyphosine like [Source:HGNC Symbol;Acc:HGNC:28375] |
| ENSG00000153113 | HGLibA_07346 | 54 | -0.646244973 | CAST | calpastatin [Source:HGNC Symbol;Acc:HGNC:1515] |
| ENSG00000153130 | HGLibA_43030 | 54 | -0.646244973 | SCOC | short coiled-coil protein [Source:HGNC Symbol;Acc:HGNC:20335] |
| ENSG00000154473 | HGLibA_04995 | 54 | -0.646244973 | BUB3 | mitotic checkpoint protein [Source:HGNC Symbol;Acc:HGNC:1151]" |
| ENSG00000156256 | HGLibA_53211 | 54 | -0.646244973 | USP16 | ubiquitin specific peptidase 16 [Source:HGNC Symbol;Acc:HGNC:12614] |
| ENSG00000158286 | HGLibA_41613 | 54 | -0.646244973 | RNF207 | ring finger protein 207 [Source:HGNC Symbol;Acc:HGNC:32947] |
| ENSG00000159958 | HGLibA_50756 | 54 | -0.646244973 | TNFRSF13C | TNF receptor superfamily member 13C [Source:HGNC Symbol;Acc:HGNC:17755] |
| ENSG00000160294 | HGLibA_28611 | 54 | -0.646244973 | MCM3AP | minichromosome maintenance complex component 3 associated protein [Source:HGNC Symbol;Acc:HGNC:6946] |
| ENSG00000160606 | HGLibA_49594 | 54 | -0.646244973 | TLCD1 | TLC domain containing 1 [Source:HGNC Symbol;Acc:HGNC:25177] |
| ENSG00000161642 | HGLibA_55898 | 54 | -0.646244973 | ZNF385A | zinc finger protein 385A [Source:HGNC Symbol;Acc:HGNC:17521] |
| ENSG00000161849 | HGLibA_25566 | 54 | -0.646244973 | KRT84 | keratin 84 [Source:HGNC Symbol;Acc:HGNC:6461] |
| ENSG00000161904 | HGLibA_26288 | 54 | -0.646244973 | LEMD2 | LEM domain containing 2 [Source:HGNC Symbol;Acc:HGNC:21244] |
| ENSG00000162244 | HGLibA_41929 | 54 | -0.646244973 | RPL29 | ribosomal protein L29 [Source:HGNC Symbol;Acc:HGNC:10331] |
| ENSG00000162747 | HGLibA_17198 | 54 | -0.646244973 | FCGR3B | Fc fragment of IgG receptor IIIb [Source:HGNC Symbol;Acc:HGNC:3620] |
| ENSG00000162813 | HGLibA_04737 | 54 | -0.646244973 | BPNT1 | 5'-bisphosphate nucleotidase 1 [Source:HGNC Symbol;Acc:HGNC:1096]" |
| ENSG00000162869 | HGLibA_38076 | 54 | -0.646244973 | PPP1R21 | protein phosphatase 1 regulatory subunit 21 [Source:HGNC Symbol;Acc:HGNC:30595] |
| ENSG00000162946 | HGLibA_13280 | 54 | -0.646244973 | DISC1 | DISC1 scaffold protein [Source:HGNC Symbol;Acc:HGNC:2888] |
| ENSG00000163145 | HGLibA_05953 | 54 | -0.646244973 | C1QTNF7 | C1q and TNF related 7 [Source:HGNC Symbol;Acc:HGNC:14342] |
| ENSG00000163605 | HGLibA_38205 | 54 | -0.646244973 | PPP4R2 | protein phosphatase 4 regulatory subunit 2 [Source:HGNC Symbol;Acc:HGNC:18296] |
| ENSG00000163638 | HGLibA_00844 | 54 | -0.646244973 | ADAMTS9 | ADAM metallopeptidase with thrombospondin type 1 motif 9 [Source:HGNC Symbol;Acc:HGNC:13202] |
| ENSG00000163788 | HGLibA_45938 | 54 | -0.646244973 | SNRK | SNF related kinase [Source:HGNC Symbol;Acc:HGNC:30598] |
| ENSG00000163933 | HGLibA_40948 | 54 | -0.646244973 | RFT1 | RFT1 homolog [Source:HGNC Symbol;Acc:HGNC:30220] |
| ENSG00000164068 | HGLibA_41491 | 54 | -0.646244973 | RNF123 | ring finger protein 123 [Source:HGNC Symbol;Acc:HGNC:21148] |
| ENSG00000164136 | HGLibA_22981 | 54 | -0.646244973 | IL15 | interleukin 15 [Source:HGNC Symbol;Acc:HGNC:5977] |
| ENSG00000164654 | HGLibA_29284 | 54 | -0.646244973 | MIOS | meiosis regulator for oocyte development [Source:HGNC Symbol;Acc:HGNC:21905] |
| ENSG00000164933 | HGLibA_44708 | 54 | -0.646244973 | SLC25A32 | solute carrier family 25 member 32 [Source:HGNC Symbol;Acc:HGNC:29683] |
| ENSG00000166828 | HGLibA_43022 | 54 | -0.646244973 | SCNN1G | sodium channel epithelial 1 gamma subunit [Source:HGNC Symbol;Acc:HGNC:10602] |
| ENSG00000166847 | HGLibA_12556 | 54 | -0.646244973 | DCTN5 | dynactin subunit 5 [Source:HGNC Symbol;Acc:HGNC:24594] |
| ENSG00000166971 | HGLibA_01518 | 54 | -0.646244973 | AKTIP | AKT interacting protein [Source:HGNC Symbol;Acc:HGNC:16710] |
| ENSG00000167193 | HGLibA_11180 | 54 | -0.646244973 | CRK | adaptor protein [Source:HGNC Symbol;Acc:HGNC:2362]" |
| ENSG00000167468 | HGLibA_20179 | 54 | -0.646244973 | GPX4 | glutathione peroxidase 4 [Source:HGNC Symbol;Acc:HGNC:4556] |
| ENSG00000167646 | HGLibA_13465 | 54 | -0.646244973 | DNAAF3 | dynein axonemal assembly factor 3 [Source:HGNC Symbol;Acc:HGNC:30492] |
| ENSG00000167992 | HGLibA_53929 | 54 | -0.646244973 | VWCE | von Willebrand factor C and EGF domains [Source:HGNC Symbol;Acc:HGNC:26487] |
| ENSG00000168883 | HGLibA_53355 | 54 | -0.646244973 | USP39 | ubiquitin specific peptidase 39 [Source:HGNC Symbol;Acc:HGNC:20071] |
| ENSG00000168918 | HGLibA_23349 | 54 | -0.646244973 | INPP5D | inositol polyphosphate-5-phosphatase D [Source:HGNC Symbol;Acc:HGNC:6079] |
| ENSG00000168994 | HGLibA_39646 | 54 | -0.646244973 | PXDC1 | PX domain containing 1 [Source:HGNC Symbol;Acc:HGNC:21361] |
| ENSG00000169469 | HGLibA_46806 | 54 | -0.646244973 | SPRR1B | small proline rich protein 1B [Source:HGNC Symbol;Acc:HGNC:11260] |
| ENSG00000170006 | HGLibA_50007 | 54 | -0.646244973 | TMEM154 | transmembrane protein 154 [Source:HGNC Symbol;Acc:HGNC:26489] |
| ENSG00000170027 | HGLibA_54746 | 54 | -0.646244973 | YWHAG | tyrosine 3-monooxygenase/tryptophan 5-monooxygenase activation protein gamma [Source:HGNC Symbol;Acc:HGNC:12852] |
| ENSG00000170381 | HGLibA_43330 | 54 | -0.646244973 | SEMA3E | semaphorin 3E [Source:HGNC Symbol;Acc:HGNC:10727] |
| ENSG00000170454 | HGLibA_25536 | 54 | -0.646244973 | KRT75 | keratin 75 [Source:HGNC Symbol;Acc:HGNC:24431] |
| ENSG00000170745 | HGLibA_24389 | 54 | -0.646244973 | KCNS3 | potassium voltage-gated channel modifier subfamily S member 3 [Source:HGNC Symbol;Acc:HGNC:6302] |
| ENSG00000170893 | HGLibA_51378 | 54 | -0.646244973 | TRH | thyrotropin releasing hormone [Source:HGNC Symbol;Acc:HGNC:12298] |
| ENSG00000170920 | HGLibA_34486 | 54 | -0.646244973 | OR7G3 | olfactory receptor family 7 subfamily G member 3 [Source:HGNC Symbol;Acc:HGNC:8467] |
| ENSG00000171121 | HGLibA_24349 | 54 | -0.646244973 | KCNMB3 | potassium calcium-activated channel subfamily M regulatory beta subunit 3 [Source:HGNC Symbol;Acc:HGNC:6287] |
| ENSG00000171345 | HGLibA_25441 | 54 | -0.646244973 | KRT19 | keratin 19 [Source:HGNC Symbol;Acc:HGNC:6436] |
| ENSG00000171793 | HGLibA_11667 | 54 | -0.646244973 | CTPS1 | CTP synthase 1 [Source:HGNC Symbol;Acc:HGNC:2519] |
| ENSG00000172020 | HGLibA_18610 | 54 | -0.646244973 | GAP43 | growth associated protein 43 [Source:HGNC Symbol;Acc:HGNC:4140] |
| ENSG00000173250 | HGLibA_19933 | 54 | -0.646244973 | GPR151 | G protein-coupled receptor 151 [Source:HGNC Symbol;Acc:HGNC:23624] |
| ENSG00000173264 | HGLibA_19896 | 54 | -0.646244973 | GPR137 | G protein-coupled receptor 137 [Source:HGNC Symbol;Acc:HGNC:24300] |
| ENSG00000173452 | HGLibA_50160 | 54 | -0.646244973 | TMEM196 | transmembrane protein 196 [Source:HGNC Symbol;Acc:HGNC:22431] |
| ENSG00000173531 | HGLibA_30253 | 54 | -0.646244973 | MST1 | macrophage stimulating 1 [Source:HGNC Symbol;Acc:HGNC:7380] |
| ENSG00000173597 | HGLibA_47742 | 54 | -0.646244973 | SULT1B1 | sulfotransferase family 1B member 1 [Source:HGNC Symbol;Acc:HGNC:17845] |
| ENSG00000174236 | HGLibA_40843 | 54 | -0.646244973 | REP15 | RAB15 effector protein [Source:HGNC Symbol;Acc:HGNC:33748] |
| ENSG00000174744 | HGLibA_04831 | 54 | -0.646244973 | BRMS1 | transcriptional repressor and anoikis regulator [Source:HGNC Symbol;Acc:HGNC:17262]" |
| ENSG00000175054 | HGLibA_03810 | 54 | -0.646244973 | ATR | ATR serine/threonine kinase [Source:HGNC Symbol;Acc:HGNC:882] |
| ENSG00000175206 | HGLibA_32500 | 54 | -0.646244973 | NPPA | natriuretic peptide A [Source:HGNC Symbol;Acc:HGNC:7939] |
| ENSG00000175215 | HGLibA_11610 | 54 | -0.646244973 | CTDSP2 | CTD small phosphatase 2 [Source:HGNC Symbol;Acc:HGNC:17077] |
| ENSG00000175535 | HGLibA_37355 | 54 | -0.646244973 | PNLIP | pancreatic lipase [Source:HGNC Symbol;Acc:HGNC:9155] |
| ENSG00000175595 | HGLibA_15441 | 54 | -0.646244973 | ERCC4 | endonuclease catalytic subunit [Source:HGNC Symbol;Acc:HGNC:3436]" |
| ENSG00000175595 | HGLibA_15440 | 54 | -0.646244973 | ERCC4 | endonuclease catalytic subunit [Source:HGNC Symbol;Acc:HGNC:3436]" |
| ENSG00000175766 | HGLibA_14819 | 54 | -0.646244973 | EIF4E1B | eukaryotic translation initiation factor 4E family member 1B [Source:HGNC Symbol;Acc:HGNC:33179] |
| ENSG00000176142 | HGLibA_50383 | 54 | -0.646244973 | TMEM39A | transmembrane protein 39A [Source:HGNC Symbol;Acc:HGNC:25600] |
| ENSG00000176732 | HGLibA_36183 | 54 | -0.646244973 | PFN4 | profilin family member 4 [Source:HGNC Symbol;Acc:HGNC:31103] |
| ENSG00000177257 | HGLibA_12918 | 54 | -0.646244973 | DEFB4B | defensin beta 4B [Source:HGNC Symbol;Acc:HGNC:30193] |
| ENSG00000177600 | HGLibA_42015 | 54 | -0.646244973 | RPLP2 | ribosomal protein lateral stalk subunit P2 [Source:HGNC Symbol;Acc:HGNC:10377] |
| ENSG00000178585 | HGLibA_11653 | 54 | -0.646244973 | CTNNBIP1 | catenin beta interacting protein 1 [Source:HGNC Symbol;Acc:HGNC:16913] |
| ENSG00000179456 | HGLibA_54831 | 54 | -0.646244973 | ZBTB18 | zinc finger and BTB domain containing 18 [Source:HGNC Symbol;Acc:HGNC:13030] |
| ENSG00000179820 | HGLibA_30632 | 54 | -0.646244973 | MYADM | myeloid associated differentiation marker [Source:HGNC Symbol;Acc:HGNC:7544] |
| ENSG00000180035 | HGLibA_56069 | 54 | -0.646244973 | ZNF48 | zinc finger protein 48 [Source:HGNC Symbol;Acc:HGNC:13114] |
| ENSG00000180447 | HGLibA_18642 | 54 | -0.646244973 | GAS1 | growth arrest specific 1 [Source:HGNC Symbol;Acc:HGNC:4165] |
| ENSG00000181616 | HGLibA_34168 | 54 | -0.646244973 | OR52H1 | olfactory receptor family 52 subfamily H member 1 [Source:HGNC Symbol;Acc:HGNC:15218] |
| ENSG00000181625 | HGLibA_45560 | 54 | -0.646244973 | SLX1B | structure-specific endonuclease subunit [Source:HGNC Symbol;Acc:HGNC:28748]" |
| ENSG00000182782 | HGLibA_20950 | 54 | -0.646244973 | HCAR2 | hydroxycarboxylic acid receptor 2 [Source:HGNC Symbol;Acc:HGNC:24827] |
| ENSG00000183035 | HGLibA_12044 | 54 | -0.646244973 | CYLC1 | cylicin 1 [Source:HGNC Symbol;Acc:HGNC:2582] |
| ENSG00000183463 | HGLibA_53132 | 54 | -0.646244973 | URAD | ureidoimidazoline (2-oxo-4-hydroxy-4-carboxy-5-) decarboxylase [Source:HGNC Symbol;Acc:HGNC:17785] |
| ENSG00000184575 | HGLibA_54576 | 54 | -0.646244973 | XPOT | exportin for tRNA [Source:HGNC Symbol;Acc:HGNC:12826] |
| ENSG00000185104 | HGLibA_15945 | 54 | -0.646244973 | FAF1 | Fas associated factor 1 [Source:HGNC Symbol;Acc:HGNC:3578] |
| ENSG00000185198 | HGLibA_38967 | 54 | -0.646244973 | PRSS57 | serine protease 57 [Source:HGNC Symbol;Acc:HGNC:31397] |
| ENSG00000185247 | HGLibA_27900 | 54 | -0.646244973 | MAGEA11 | MAGE family member A11 [Source:HGNC Symbol;Acc:HGNC:6798] |
| ENSG00000185386 | HGLibA_28253 | 54 | -0.646244973 | MAPK11 | mitogen-activated protein kinase 11 [Source:HGNC Symbol;Acc:HGNC:6873] |
| ENSG00000185559 | HGLibA_13353 | 54 | -0.646244973 | DLK1 | delta like non-canonical Notch ligand 1 [Source:HGNC Symbol;Acc:HGNC:2907] |
| ENSG00000185989 | HGLibA_40303 | 54 | -0.646244973 | RASA3 | RAS p21 protein activator 3 [Source:HGNC Symbol;Acc:HGNC:20331] |
| ENSG00000186104 | HGLibA_12140 | 54 | -0.646244973 | CYP2R1 | cytochrome P450 family 2 subfamily R member 1 [Source:HGNC Symbol;Acc:HGNC:20580] |
| ENSG00000186298 | HGLibA_38011 | 54 | -0.646244973 | PPP1CC | protein phosphatase 1 catalytic subunit gamma [Source:HGNC Symbol;Acc:HGNC:9283] |
| ENSG00000186335 | HGLibA_45033 | 54 | -0.646244973 | SLC36A2 | solute carrier family 36 member 2 [Source:HGNC Symbol;Acc:HGNC:18762] |
| ENSG00000186628 | HGLibA_18082 | 54 | -0.646244973 | FSD2 | fibronectin type III and SPRY domain containing 2 [Source:HGNC Symbol;Acc:HGNC:18024] |
| ENSG00000186912 | HGLibA_34889 | 54 | -0.646244973 | P2RY4 | pyrimidinergic receptor P2Y4 [Source:HGNC Symbol;Acc:HGNC:8542] |
| ENSG00000187257 | HGLibA_42305 | 54 | -0.646244973 | RSBN1L | round spermatid basic protein 1 like [Source:HGNC Symbol;Acc:HGNC:24765] |
| ENSG00000188038 | HGLibA_32712 | 54 | -0.646244973 | NRN1L | neuritin 1 like [Source:HGNC Symbol;Acc:HGNC:29811] |
| ENSG00000188425 | HGLibA_31098 | 54 | -0.646244973 | NANOS2 | nanos C2HC-type zinc finger 2 [Source:HGNC Symbol;Acc:HGNC:23292] |
| ENSG00000188729 | HGLibA_34716 | 54 | -0.646244973 | OSTN | osteocrin [Source:HGNC Symbol;Acc:HGNC:29961] |
| ENSG00000196072 | HGLibA_04560 | 54 | -0.646244973 | BLOC1S2 | biogenesis of lysosomal organelles complex 1 subunit 2 [Source:HGNC Symbol;Acc:HGNC:20984] |
| ENSG00000196323 | HGLibA_54893 | 54 | -0.646244973 | ZBTB44 | zinc finger and BTB domain containing 44 [Source:HGNC Symbol;Acc:HGNC:25001] |
| ENSG00000196420 | HGLibA_42559 | 54 | -0.646244973 | S100A5 | S100 calcium binding protein A5 [Source:HGNC Symbol;Acc:HGNC:10495] |
| ENSG00000197102 | HGLibA_14231 | 54 | -0.646244973 | DYNC1H1 | dynein cytoplasmic 1 heavy chain 1 [Source:HGNC Symbol;Acc:HGNC:2961] |
| ENSG00000197256 | HGLibA_24028 | 54 | -0.646244973 | KANK2 | KN motif and ankyrin repeat domains 2 [Source:HGNC Symbol;Acc:HGNC:29300] |
| ENSG00000197296 | HGLibA_17542 | 54 | -0.646244973 | FITM2 | fat storage inducing transmembrane protein 2 [Source:HGNC Symbol;Acc:HGNC:16135] |
| ENSG00000197405 | HGLibA_06354 | 54 | -0.646244973 | C5AR1 | complement C5a receptor 1 [Source:HGNC Symbol;Acc:HGNC:1338] |
| ENSG00000197912 | HGLibA_46638 | 54 | -0.646244973 | SPG7 | paraplegin matrix AAA peptidase subunit [Source:HGNC Symbol;Acc:HGNC:11237]" |
| ENSG00000198019 | HGLibA_17185 | 54 | -0.646244973 | FCGR1B | Fc fragment of IgG receptor Ib [Source:HGNC Symbol;Acc:HGNC:3614] |
| ENSG00000198752 | HGLibA_08560 | 54 | -0.646244973 | CDC42BPB | CDC42 binding protein kinase beta [Source:HGNC Symbol;Acc:HGNC:1738] |
| ENSG00000198879 | HGLibA_43724 | 54 | -0.646244973 | SFMBT2 | Scm like with four mbt domains 2 [Source:HGNC Symbol;Acc:HGNC:20256] |
| ENSG00000204681 | HGLibA_18341 | 54 | -0.646244973 | GABBR1 | gamma-aminobutyric acid type B receptor subunit 1 [Source:HGNC Symbol;Acc:HGNC:4070] |
| ENSG00000204859 | HGLibA_54905 | 54 | -0.646244973 | ZBTB48 | zinc finger and BTB domain containing 48 [Source:HGNC Symbol;Acc:HGNC:4930] |
| ENSG00000206418 | HGLibA_39809 | 54 | -0.646244973 | RAB12 | member RAS oncogene family [Source:HGNC Symbol;Acc:HGNC:31332]" |
| ENSG00000212710 | HGLibA_11568 | 54 | -0.646244973 | CTAGE1 | cutaneous T cell lymphoma-associated antigen 1 [Source:HGNC Symbol;Acc:HGNC:24346] |
| ENSG00000213445 | HGLibA_44188 | 54 | -0.646244973 | SIPA1 | signal-induced proliferation-associated 1 [Source:HGNC Symbol;Acc:HGNC:10885] |
| ENSG00000214140 | HGLibA_38357 | 54 | -0.646244973 | PRCD | photoreceptor disc component [Source:HGNC Symbol;Acc:HGNC:32528] |
| ENSG00000215845 | HGLibA_52029 | 54 | -0.646244973 | TSTD1 | thiosulfate sulfurtransferase like domain containing 1 [Source:HGNC Symbol;Acc:HGNC:35410] |

| | | | | | |
|---|---|---|---|---|---|
| ENSG00000221955 | HGLibA_44379 | 54 | -0.646244973 | SLC12A8 | solute carrier family 12 member 8 [Source:HGNC Symbol;Acc:HGNC:15595] |
| ENSG00000226650 | HGLibA_24946 | 54 | -0.646244973 | KIF4B | kinesin family member 4B [Source:HGNC Symbol;Acc:HGNC:6322] |
| ENSG00000227729 | HGLibA_40728 | 54 | -0.646244973 | RD3L | retinal degeneration 3 like [Source:HGNC Symbol;Acc:HGNC:40912] |
| ENSG00000230453 | HGLibA_02020 | 54 | -0.646244973 | ANKRD18B | ankyrin repeat domain 18B [Source:HGNC Symbol;Acc:HGNC:23644] |
| ENSG00000231051 | HGLibA_53267 | 54 | -0.646244973 | USP17L28 | ubiquitin specific peptidase 17-like family member 28 [Source:HGNC Symbol;Acc:HGNC:44456] |
| ENSG00000233412 | HGLibA_34295 | 54 | -0.646244973 | OR5H15 | olfactory receptor family 5 subfamily H member 15 [Source:HGNC Symbol;Acc:HGNC:31287] |
| ENSG00000233803 | HGLibA_51974 | 54 | -0.646244973 | TSPY4 | testis specific protein Y-linked 4 [Source:HGNC Symbol;Acc:HGNC:37287] |
| ENSG00000234278 | HGLibA_38811 | 54 | -0.646244973 | PRR20E | proline rich 20E [Source:HGNC Symbol;Acc:HGNC:37223] |
| ENSG00000240505 | HGLibA_50754 | 54 | -0.646244973 | TNFRSF13B | TNF receptor superfamily member 13B [Source:HGNC Symbol;Acc:HGNC:18153] |
| ENSG00000242114 | HGLibA_30357 | 54 | -0.646244973 | MTFP1 | mitochondrial fission process 1 [Source:HGNC Symbol;Acc:HGNC:26945] |
| ENSG00000242389 | HGLibA_40621 | 54 | -0.646244973 | RBMY1E | member E [Source:HGNC Symbol;Acc:HGNC:23916]" |
| ENSG00000242852 | HGLibA_56606 | 54 | -0.646244973 | ZNF709 | zinc finger protein 709 [Source:HGNC Symbol;Acc:HGNC:20629] |
| ENSG00000244588 | HGLibA_40066 | 54 | -0.646244973 | RAD21L1 | RAD21 cohesin complex component like 1 [Source:HGNC Symbol;Acc:HGNC:16271] |
| ENSG00000254505 | HGLibA_09488 | 54 | -0.646244973 | CHMP4A | charged multivesicular body protein 4A [Source:HGNC Symbol;Acc:HGNC:20274] |
| ENSG00000257218 | HGLibA_18693 | 54 | -0.646244973 | GATC | glutamyl-tRNA amidotransferase subunit C [Source:HGNC Symbol;Acc:HGNC:25068] |
| ENSG00000260027 | HGLibA_21946 | 54 | -0.646244973 | HOXB7 | homeobox B7 [Source:HGNC Symbol;Acc:HGNC:5118] |
| ENSG00000263002 | HGLibA_55646 | 54 | -0.646244973 | ZNF234 | zinc finger protein 234 [Source:HGNC Symbol;Acc:HGNC:13027] |
| ENSG00000269556 | HGLibA_50121 | 54 | -0.646244973 | TMEM185A | transmembrane protein 185A [Source:HGNC Symbol;Acc:HGNC:17125] |
| ENSG00000273045 | HGLibA_06110 | 54 | -0.646244973 | C2orf15 | chromosome 2 open reading frame 15 [Source:HGNC Symbol;Acc:HGNC:28436] |
| ENSG00000275722 | HGLibA_27793 | 54 | -0.646244973 | LYZL6 | lysozyme like 6 [Source:HGNC Symbol;Acc:HGNC:29614] |
| FTH1P18 | HGLibA_18120 | 54 | -0.646244973 | FTH1P18 | - |
| hsa-mir-1286 | HGLibA_57609 | 54 | -0.646244973 | hsa-mir-1286 | - |
| hsa-mir-1286 | HGLibA_57610 | 54 | -0.646244973 | hsa-mir-1286 | - |
| hsa-mir-1321 | HGLibA_57758 | 54 | -0.646244973 | hsa-mir-1321 | - |
| hsa-mir-4418 | HGLibA_60056 | 54 | -0.646244973 | hsa-mir-4418 | - |
| hsa-mir-4473 | HGLibA_60304 | 54 | -0.646244973 | hsa-mir-4473 | - |
| hsa-mir-4525 | HGLibA_60561 | 54 | -0.646244973 | hsa-mir-4525 | - |
| hsa-mir-4670 | HGLibA_60796 | 54 | -0.646244973 | hsa-mir-4670 | - |
| hsa-mir-4689 | HGLibA_60867 | 54 | -0.646244973 | hsa-mir-4689 | - |
| hsa-mir-4768 | HGLibA_61167 | 54 | -0.646244973 | hsa-mir-4768 | - |
| hsa-mir-4798 | HGLibA_61296 | 54 | -0.646244973 | hsa-mir-4798 | - |
| hsa-mir-5192 | HGLibA_61688 | 54 | -0.646244973 | hsa-mir-5192 | - |
| hsa-mir-563 | HGLibA_62215 | 54 | -0.646244973 | hsa-mir-563 | - |
| hsa-mir-5692c-2 | HGLibA_62301 | 54 | -0.646244973 | hsa-mir-5692c-2 | - |
| hsa-mir-6133 | HGLibA_62685 | 54 | -0.646244973 | hsa-mir-6133 | - |
| hsa-mir-6877 | HGLibA_63691 | 54 | -0.646244973 | hsa-mir-6877 | - |
| hsa-mir-7850 | HGLibA_63990 | 54 | -0.646244973 | hsa-mir-7850 | - |
| hsa-mir-7855 | HGLibA_64009 | 54 | -0.646244973 | hsa-mir-7855 | - |
| MARS | HGLibA_28376 | 54 | -0.646244973 | MARS | - |
| MEF2BNB-MEF2B | HGLibA_28835 | 54 | -0.646244973 | MEF2BNB-MEF2B | - |
| METTL21B | HGLibA_28980 | 54 | -0.646244973 | METTL21B | - |
| PLA2G16 | HGLibA_36878 | 54 | -0.646244973 | PLA2G16 | - |
| PRAC | HGLibA_38261 | 54 | -0.646244973 | PRAC | - |
| PRSS42 | HGLibA_38942 | 54 | -0.646244973 | PRSS42 | - |
| RQCD1 | HGLibA_42219 | 54 | -0.646244973 | RQCD1 | - |
| SOLH | HGLibA_46169 | 54 | -0.646244973 | SOLH | - |
| WBSCR16 | HGLibA_53989 | 54 | -0.646244973 | WBSCR16 | - |
| APOPT1 | HGLibA_02558 | 53 | -0.655290316 | APOPT1 | - |
| ATP5O | HGLibA_03685 | 53 | -0.655290316 | ATP5O | - |
| C12orf65 | HGLibA_05279 | 53 | -0.655290316 | C12orf65 | - |
| C17orf47 | HGLibA_05533 | 53 | -0.655290316 | C17orf47 | - |
| C17orf66 | HGLibA_05561 | 53 | -0.655290316 | C17orf66 | - |
| C1orf151-NBL1 | HGLibA_05777 | 53 | -0.655290316 | C1orf151-NBL1 | - |
| C1orf65 | HGLibA_05888 | 53 | -0.655290316 | C1orf65 | - |
| CCDC101 | HGLibA_07504 | 53 | -0.655290316 | CCDC101 | - |
| ENSG00000000460 | HGLibA_05749 | 53 | -0.655290316 | C1orf112 | chromosome 1 open reading frame 112 [Source:HGNC Symbol;Acc:HGNC:25565] |
| ENSG00000001561 | HGLibA_15175 | 53 | -0.655290316 | ENPP4 | ectonucleotide pyrophosphatase/phosphodiesterase 4 [Source:HGNC Symbol;Acc:HGNC:3359] |
| ENSG00000009307 | HGLibA_11314 | 53 | -0.655290316 | CSDE1 | cold shock domain containing E1 [Source:HGNC Symbol;Acc:HGNC:29905] |
| ENSG00000012232 | HGLibA_15814 | 53 | -0.655290316 | EXTL3 | exostosin like glycosyltransferase 3 [Source:HGNC Symbol;Acc:HGNC:3518] |
| ENSG00000024526 | HGLibA_12974 | 53 | -0.655290316 | DEPDC1 | DEP domain containing 1 [Source:HGNC Symbol;Acc:HGNC:22949] |
| ENSG00000048052 | HGLibA_21027 | 53 | -0.655290316 | HDAC9 | histone deacetylase 9 [Source:HGNC Symbol;Acc:HGNC:14065] |
| ENSG00000059122 | HGLibA_17682 | 53 | -0.655290316 | FLYWCH1 | FLYWCH-type zinc finger 1 [Source:HGNC Symbol;Acc:HGNC:25404] |
| ENSG00000061676 | HGLibA_31310 | 53 | -0.655290316 | NCKAP1 | NCK associated protein 1 [Source:HGNC Symbol;Acc:HGNC:7666] |
| ENSG00000063127 | HGLibA_45307 | 53 | -0.655290316 | SLC6A16 | solute carrier family 6 member 16 [Source:HGNC Symbol;Acc:HGNC:13622] |
| ENSG00000065548 | HGLibA_54967 | 53 | -0.655290316 | ZC3H15 | zinc finger CCCH-type containing 15 [Source:HGNC Symbol;Acc:HGNC:29528] |
| ENSG00000066405 | HGLibA_09870 | 53 | -0.655290316 | CLDN18 | claudin 18 [Source:HGNC Symbol;Acc:HGNC:2039] |
| ENSG00000070476 | HGLibA_57080 | 53 | -0.655290316 | ZXDC | ZXD family zinc finger C [Source:HGNC Symbol;Acc:HGNC:28160] |
| ENSG00000071537 | HGLibA_43276 | 53 | -0.655290316 | SEL1L | ERAD E3 ligase adaptor subunit [Source:HGNC Symbol;Acc:HGNC:10717]" |
| ENSG00000071564 | HGLibA_48784 | 53 | -0.655290316 | TCF3 | transcription factor 3 [Source:HGNC Symbol;Acc:HGNC:11633] |
| ENSG00000074660 | HGLibA_42876 | 53 | -0.655290316 | SCARF1 | scavenger receptor class F member 1 [Source:HGNC Symbol;Acc:HGNC:16820] |
| ENSG00000078295 | HGLibA_00912 | 53 | -0.655290316 | ADCY2 | adenylate cyclase 2 [Source:HGNC Symbol;Acc:HGNC:233] |
| ENSG00000078304 | HGLibA_38175 | 53 | -0.655290316 | PPP2R5C | protein phosphatase 2 regulatory subunit B'gamma [Source:HGNC Symbol;Acc:HGNC:9311] |
| ENSG00000079805 | HGLibA_13689 | 53 | -0.655290316 | DNM2 | dynamin 2 [Source:HGNC Symbol;Acc:HGNC:2974] |
| ENSG00000081154 | HGLibA_35637 | 53 | -0.655290316 | PCNP | PEST proteolytic signal containing nuclear protein [Source:HGNC Symbol;Acc:HGNC:30023] |
| ENSG00000081307 | HGLibA_52523 | 53 | -0.655290316 | UBA5 | ubiquitin like modifier activating enzyme 5 [Source:HGNC Symbol;Acc:HGNC:23230] |
| ENSG00000085514 | HGLibA_36669 | 53 | -0.655290316 | PILRA | paired immunoglobin like type 2 receptor alpha [Source:HGNC Symbol;Acc:HGNC:20396] |
| ENSG00000091009 | HGLibA_40524 | 53 | -0.655290316 | RBM27 | RNA binding motif protein 27 [Source:HGNC Symbol;Acc:HGNC:29243] |
| ENSG00000095209 | HGLibA_50380 | 53 | -0.655290316 | TMEM38B | transmembrane protein 38B [Source:HGNC Symbol;Acc:HGNC:25535] |
| ENSG00000095713 | HGLibA_11218 | 53 | -0.655290316 | CRTAC1 | cartilage acidic protein 1 [Source:HGNC Symbol;Acc:HGNC:14882] |
| ENSG00000100095 | HGLibA_43683 | 53 | -0.655290316 | SEZ6L | seizure related 6 homolog like [Source:HGNC Symbol;Acc:HGNC:10763] |
| ENSG00000100320 | HGLibA_40458 | 53 | -0.655290316 | RBFOX2 | RNA binding fox-1 homolog 2 [Source:HGNC Symbol;Acc:HGNC:9906] |
| ENSG00000100650 | HGLibA_47080 | 53 | -0.655290316 | SRSF5 | serine and arginine rich splicing factor 5 [Source:HGNC Symbol;Acc:HGNC:10787] |
| ENSG00000100867 | HGLibA_13131 | 53 | -0.655290316 | DHRS2 | dehydrogenase/reductase 2 [Source:HGNC Symbol;Acc:HGNC:18349] |
| ENSG00000102349 | HGLibA_25085 | 53 | -0.655290316 | KLF8 | Kruppel like factor 8 [Source:HGNC Symbol;Acc:HGNC:6351] |
| ENSG00000102401 | HGLibA_03043 | 53 | -0.655290316 | ARMCX3 | armadillo repeat containing X-linked 3 [Source:HGNC Symbol;Acc:HGNC:24065] |
| ENSG00000102683 | HGLibA_43808 | 53 | -0.655290316 | SGCG | sarcoglycan gamma [Source:HGNC Symbol;Acc:HGNC:10809] |
| ENSG00000103005 | HGLibA_53161 | 53 | -0.655290316 | USB1 | U6 snRNA biogenesis phosphodiesterase 1 [Source:HGNC Symbol;Acc:HGNC:25792] |
| ENSG00000103056 | HGLibA_45808 | 53 | -0.655290316 | SMPD3 | sphingomyelin phosphodiesterase 3 [Source:HGNC Symbol;Acc:HGNC:14240] |
| ENSG00000105011 | HGLibA_03285 | 53 | -0.655290316 | ASF1B | anti-silencing function 1B histone chaperone [Source:HGNC Symbol;Acc:HGNC:20996] |
| ENSG00000105856 | HGLibA_20934 | 53 | -0.655290316 | HBP1 | HMG-box transcription factor 1 [Source:HGNC Symbol;Acc:HGNC:23200] |
| ENSG00000107864 | HGLibA_10923 | 53 | -0.655290316 | CPEB3 | cytoplasmic polyadenylation element binding protein 3 [Source:HGNC Symbol;Acc:HGNC:21746] |
| ENSG00000108797 | HGLibA_10419 | 53 | -0.655290316 | CNTNAP1 | contactin associated protein 1 [Source:HGNC Symbol;Acc:HGNC:8011] |
| ENSG00000108861 | HGLibA_14179 | 53 | -0.655290316 | DUSP3 | dual specificity phosphatase 3 [Source:HGNC Symbol;Acc:HGNC:3069] |
| ENSG00000108950 | HGLibA_16443 | 53 | -0.655290316 | FAM20A | golgi associated secretory pathway pseudokinase [Source:HGNC Symbol;Acc:HGNC:23015]" |
| ENSG00000109320 | HGLibA_31815 | 53 | -0.655290316 | NFKB1 | nuclear factor kappa B subunit 1 [Source:HGNC Symbol;Acc:HGNC:7794] |
| ENSG00000110243 | HGLibA_02460 | 53 | -0.655290316 | APOA5 | apolipoprotein A5 [Source:HGNC Symbol;Acc:HGNC:17288] |
| ENSG00000110719 | HGLibA_48813 | 53 | -0.655290316 | TCIRG1 | ATPase H+ transporting V0 subunit a3 [Source:HGNC Symbol;Acc:HGNC:11647]" |
| ENSG00000112159 | HGLibA_28704 | 53 | -0.655290316 | MDN1 | midasin AAA ATPase 1 [Source:HGNC Symbol;Acc:HGNC:18302] |
| ENSG00000113119 | HGLibA_49795 | 53 | -0.655290316 | TMCO6 | transmembrane and coiled-coil domains 6 [Source:HGNC Symbol;Acc:HGNC:28814] |
| ENSG00000113319 | HGLibA_40340 | 53 | -0.655290316 | RASGRF2 | Ras protein specific guanine nucleotide releasing factor 2 [Source:HGNC Symbol;Acc:HGNC:9876] |
| ENSG00000114013 | HGLibA_08467 | 53 | -0.655290316 | CD86 | CD86 molecule [Source:HGNC Symbol;Acc:HGNC:1705] |
| ENSG00000114098 | HGLibA_03032 | 53 | -0.655290316 | ARMC8 | armadillo repeat containing 8 [Source:HGNC Symbol;Acc:HGNC:24999] |
| ENSG00000114416 | HGLibA_18217 | 53 | -0.655290316 | FXR1 | FMR1 autosomal homolog 1 [Source:HGNC Symbol;Acc:HGNC:4023] |
| ENSG00000115073 | HGLibA_00654 | 53 | -0.655290316 | ACTR1B | ARP1 actin related protein 1 homolog B [Source:HGNC Symbol;Acc:HGNC:168] |
| ENSG00000117543 | HGLibA_13849 | 53 | -0.655290316 | DPH5 | diphthamide biosynthesis 5 [Source:HGNC Symbol;Acc:HGNC:24270] |
| ENSG00000118420 | HGLibA_52688 | 53 | -0.655290316 | UBE3D | ubiquitin protein ligase E3D [Source:HGNC Symbol;Acc:HGNC:21381] |
| ENSG00000118900 | HGLibA_52720 | 53 | -0.655290316 | UBN1 | ubinuclein 1 [Source:HGNC Symbol;Acc:HGNC:12506] |
| ENSG00000119913 | HGLibA_48955 | 53 | -0.655290316 | TECTB | tectorin beta [Source:HGNC Symbol;Acc:HGNC:11721] |
| ENSG00000120519 | HGLibA_44347 | 53 | -0.655290316 | SLC10A7 | solute carrier family 10 member 7 [Source:HGNC Symbol;Acc:HGNC:23088] |
| ENSG00000121769 | HGLibA_15916 | 53 | -0.655290316 | FABP3 | fatty acid binding protein 3 [Source:HGNC Symbol;Acc:HGNC:3557] |
| ENSG00000122674 | HGLibA_08222 | 53 | -0.655290316 | CCZ1 | vacuolar protein trafficking and biogenesis associated [Source:HGNC Symbol;Acc:HGNC:21691]" |
| ENSG00000123136 | HGLibA_12695 | 53 | -0.655290316 | DDX39A | DExD-box helicase 39A [Source:HGNC Symbol;Acc:HGNC:17821] |
| ENSG00000123342 | HGLibA_29504 | 53 | -0.655290316 | MMP19 | matrix metallopeptidase 19 [Source:HGNC Symbol;Acc:HGNC:7165] |
| ENSG00000123572 | HGLibA_32699 | 53 | -0.655290316 | NRK | Nik related kinase [Source:HGNC Symbol;Acc:HGNC:25391] |
| ENSG00000123836 | HGLibA_36157 | 53 | -0.655290316 | PFKFB2 | 6-biphosphatase 2 [Source:HGNC Symbol;Acc:HGNC:8873]" |
| ENSG00000123892 | HGLibA_39897 | 53 | -0.655290316 | RAB38 | member RAS oncogene family [Source:HGNC Symbol;Acc:HGNC:9776]" |
| ENSG00000124839 | HGLibA_39820 | 53 | -0.655290316 | RAB17 | member RAS oncogene family [Source:HGNC Symbol;Acc:HGNC:16523]" |
| ENSG00000125823 | HGLibA_11495 | 53 | -0.655290316 | CSTL1 | cystatin like 1 [Source:HGNC Symbol;Acc:HGNC:15958] |
| ENSG00000126091 | HGLibA_47242 | 53 | -0.655290316 | ST3GAL3 | 3-sialyltransferase 3 [Source:HGNC Symbol;Acc:HGNC:10866]" |
| ENSG00000126456 | HGLibA_23587 | 53 | -0.655290316 | IRF3 | interferon regulatory factor 3 [Source:HGNC Symbol;Acc:HGNC:6118] |
| ENSG00000126562 | HGLibA_54339 | 53 | -0.655290316 | WNK4 | WNK lysine deficient protein kinase 4 [Source:HGNC Symbol;Acc:HGNC:14544] |
| ENSG00000128272 | HGLibA_03439 | 53 | -0.655290316 | ATF4 | activating transcription factor 4 [Source:HGNC Symbol;Acc:HGNC:786] |
| ENSG00000128298 | HGLibA_04128 | 53 | -0.655290316 | BAIAP2L2 | BAI1 associated protein 2 like 2 [Source:HGNC Symbol;Acc:HGNC:26203] |
| ENSG00000128340 | HGLibA_40046 | 53 | -0.655290316 | RAC2 | Rac family small GTPase 2 [Source:HGNC Symbol;Acc:HGNC:9802] |
| ENSG00000128596 | HGLibA_07605 | 53 | -0.655290316 | CCDC136 | coiled-coil domain containing 136 [Source:HGNC Symbol;Acc:HGNC:22225] |
| ENSG00000129170 | HGLibA_11442 | 53 | -0.655290316 | CSRP3 | cysteine and glycine rich protein 3 [Source:HGNC Symbol;Acc:HGNC:2472] |
| ENSG00000130935 | HGLibA_32258 | 53 | -0.655290316 | NOL11 | nucleolar protein 11 [Source:HGNC Symbol;Acc:HGNC:24557] |
| ENSG00000132205 | HGLibA_15050 | 53 | -0.655290316 | EMILIN2 | elastin microfibril interfacer 2 [Source:HGNC Symbol;Acc:HGNC:19881] |
| ENSG00000132341 | HGLibA_40190 | 53 | -0.655290316 | RAN | member RAS oncogene family [Source:HGNC Symbol;Acc:HGNC:9846]" |
| ENSG00000132437 | HGLibA_12599 | 53 | -0.655290316 | DDC | dopa decarboxylase [Source:HGNC Symbol;Acc:HGNC:2719] |
| ENSG00000134014 | HGLibA_14980 | 53 | -0.655290316 | ELP3 | elongator acetyltransferase complex subunit 3 [Source:HGNC Symbol;Acc:HGNC:20696] |
| ENSG00000134595 | HGLibA_46259 | 53 | -0.655290316 | SOX3 | SRY-box 3 [Source:HGNC Symbol;Acc:HGNC:11199] |
| ENSG00000134905 | HGLibA_07262 | 53 | -0.655290316 | CARS2 | mitochondrial [Source:HGNC Symbol;Acc:HGNC:25695]" |
| ENSG00000135114 | HGLibA_33207 | 53 | -0.655290316 | OASL | 2'-5'-oligoadenylate synthetase like [Source:HGNC Symbol;Acc:HGNC:8090] |
| ENSG00000135392 | HGLibA_13577 | 53 | -0.655290316 | DNAJC14 | DnaJ heat shock protein family (Hsp40) member C14 [Source:HGNC Symbol;Acc:HGNC:24581] |

| | | | | | |
|---|---|---|---|---|---|
| ENSG00000135413 | HGLibA_25913 | 53 | -0.655290316 | LACRT | lacritin [Source:HGNC Symbol;Acc:HGNC:16430] |
| ENSG00000136875 | HGLibA_38731 | 53 | -0.655290316 | PRPF4 | pre-mRNA processing factor 4 [Source:HGNC Symbol;Acc:HGNC:17349] |
| ENSG00000137076 | HGLibA_49633 | 53 | -0.655290316 | TLN1 | talin 1 [Source:HGNC Symbol;Acc:HGNC:11845] |
| ENSG00000137275 | HGLibA_41316 | 53 | -0.655290316 | RIPK1 | receptor interacting serine/threonine kinase 1 [Source:HGNC Symbol;Acc:HGNC:10019] |
| ENSG00000138002 | HGLibA_22750 | 53 | -0.655290316 | IFT172 | intraflagellar transport 172 [Source:HGNC Symbol;Acc:HGNC:30391] |
| ENSG00000138160 | HGLibA_24849 | 53 | -0.655290316 | KIF11 | kinesin family member 11 [Source:HGNC Symbol;Acc:HGNC:6388] |
| ENSG00000138439 | HGLibA_16045 | 53 | -0.655290316 | FAM117B | family with sequence similarity 117 member B [Source:HGNC Symbol;Acc:HGNC:14440] |
| ENSG00000138496 | HGLibA_35246 | 53 | -0.655290316 | PARP9 | poly(ADP-ribose) polymerase family member 9 [Source:HGNC Symbol;Acc:HGNC:24118] |
| ENSG00000139351 | HGLibA_47938 | 53 | -0.655290316 | SYCP3 | synaptonemal complex protein 3 [Source:HGNC Symbol;Acc:HGNC:18130] |
| ENSG00000140545 | HGLibA_29059 | 53 | -0.655290316 | MFGE8 | milk fat globule-EGF factor 8 protein [Source:HGNC Symbol;Acc:HGNC:7036] |
| ENSG00000140553 | HGLibA_53006 | 53 | -0.655290316 | UNC45A | unc-45 myosin chaperone A [Source:HGNC Symbol;Acc:HGNC:30594] |
| ENSG00000140691 | HGLibA_03024 | 53 | -0.655290316 | ARMC5 | armadillo repeat containing 5 [Source:HGNC Symbol;Acc:HGNC:25781] |
| ENSG00000140995 | HGLibA_12788 | 53 | -0.655290316 | DEF8 | differentially expressed in FDCP 8 homolog [Source:HGNC Symbol;Acc:HGNC:25969] |
| ENSG00000141434 | HGLibA_28898 | 53 | -0.655290316 | MEP1B | meprin A subunit beta [Source:HGNC Symbol;Acc:HGNC:7020] |
| ENSG00000144559 | HGLibA_48287 | 53 | -0.655290316 | TAMM41 | TAM41 mitochondrial translocator assembly and maintenance homolog [Source:HGNC Symbol;Acc:HGNC:25187] |
| ENSG00000144736 | HGLibA_44083 | 53 | -0.655290316 | SHQ1 | H/ACA ribonucleoprotein assembly factor [Source:HGNC Symbol;Acc:HGNC:25543]" |
| ENSG00000145730 | HGLibA_35083 | 53 | -0.655290316 | PAM | peptidylglycine alpha-amidating monooxygenase [Source:HGNC Symbol;Acc:HGNC:8596] |
| ENSG00000145782 | HGLibA_03465 | 53 | -0.655290316 | ATG12 | autophagy related 12 [Source:HGNC Symbol;Acc:HGNC:588] |
| ENSG00000146910 | HGLibA_10361 | 53 | -0.655290316 | CNPY1 | canopy FGF signaling regulator 1 [Source:HGNC Symbol;Acc:HGNC:27786] |
| ENSG00000147475 | HGLibA_15497 | 53 | -0.655290316 | ERLIN2 | ER lipid raft associated 2 [Source:HGNC Symbol;Acc:HGNC:1356] |
| ENSG00000148965 | HGLibA_42618 | 53 | -0.655290316 | SAA4 | constitutive [Source:HGNC Symbol;Acc:HGNC:10516]" |
| ENSG00000151612 | HGLibA_56824 | 53 | -0.655290316 | ZNF827 | zinc finger protein 827 [Source:HGNC Symbol;Acc:HGNC:27193] |
| ENSG00000152359 | HGLibA_37449 | 53 | -0.655290316 | POC5 | POC5 centriolar protein [Source:HGNC Symbol;Acc:HGNC:26658] |
| ENSG00000152767 | HGLibA_16874 | 53 | -0.655290316 | FARP1 | ARH/RhoGEF and pleckstrin domain protein 1 [Source:HGNC Symbol;Acc:HGNC:3591]" |
| ENSG00000153165 | HGLibA_41031 | 53 | -0.655290316 | RGPD3 | RANBP2-like and GRIP domain containing 3 [Source:HGNC Symbol;Acc:HGNC:32416] |
| ENSG00000154370 | HGLibA_51405 | 53 | -0.655290316 | TRIM11 | tripartite motif containing 11 [Source:HGNC Symbol;Acc:HGNC:16281] |
| ENSG00000154767 | HGLibA_54551 | 53 | -0.655290316 | XPC | DNA damage recognition and repair factor [Source:HGNC Symbol;Acc:HGNC:12816]" |
| ENSG00000155329 | HGLibA_55002 | 53 | -0.655290316 | ZCCHC10 | zinc finger CCHC-type containing 10 [Source:HGNC Symbol;Acc:HGNC:25954] |
| ENSG00000155330 | HGLibA_05493 | 53 | -0.655290316 | C16orf87 | chromosome 16 open reading frame 87 [Source:HGNC Symbol;Acc:HGNC:33754] |
| ENSG00000155530 | HGLibA_27171 | 53 | -0.655290316 | LRGUK | leucine rich repeats and guanylate kinase domain containing [Source:HGNC Symbol;Acc:HGNC:21964] |
| ENSG00000155850 | HGLibA_44790 | 53 | -0.655290316 | SLC26A2 | solute carrier family 26 member 2 [Source:HGNC Symbol;Acc:HGNC:10994] |
| ENSG00000157111 | HGLibA_50061 | 53 | -0.655290316 | TMEM171 | transmembrane protein 171 [Source:HGNC Symbol;Acc:HGNC:27031] |
| ENSG00000157450 | HGLibA_41467 | 53 | -0.655290316 | RNF111 | ring finger protein 111 [Source:HGNC Symbol;Acc:HGNC:17384] |
| ENSG00000158352 | HGLibA_44095 | 53 | -0.655290316 | SHROOM4 | shroom family member 4 [Source:HGNC Symbol;Acc:HGNC:29215] |
| ENSG00000158623 | HGLibA_10708 | 53 | -0.655290316 | COPG2 | coatomer protein complex subunit gamma 2 [Source:HGNC Symbol;Acc:HGNC:2237] |
| ENSG00000158941 | HGLibA_07491 | 53 | -0.655290316 | CCAR2 | cell cycle and apoptosis regulator 2 [Source:HGNC Symbol;Acc:HGNC:23360] |
| ENSG00000158941 | HGLibA_07489 | 53 | -0.655290316 | CCAR2 | cell cycle and apoptosis regulator 2 [Source:HGNC Symbol;Acc:HGNC:23360] |
| ENSG00000160179 | HGLibA_00215 | 53 | -0.655290316 | ABCG1 | ATP binding cassette subfamily G member 1 [Source:HGNC Symbol;Acc:HGNC:73] |
| ENSG00000160867 | HGLibA_17451 | 53 | -0.655290316 | FGFR4 | fibroblast growth factor receptor 4 [Source:HGNC Symbol;Acc:HGNC:3691] |
| ENSG00000162813 | HGLibA_04736 | 53 | -0.655290316 | BPNT1 | 5'-bisphosphate nucleotidase 1 [Source:HGNC Symbol;Acc:HGNC:1096]" |
| ENSG00000163462 | HGLibA_51512 | 53 | -0.655290316 | TRIM46 | tripartite motif containing 46 [Source:HGNC Symbol;Acc:HGNC:19019] |
| ENSG00000163517 | HGLibA_21002 | 53 | -0.655290316 | HDAC11 | histone deacetylase 11 [Source:HGNC Symbol;Acc:HGNC:19086] |
| ENSG00000165025 | HGLibA_47950 | 53 | -0.655290316 | SYK | spleen associated tyrosine kinase [Source:HGNC Symbol;Acc:HGNC:11491] |
| ENSG00000167969 | HGLibA_14406 | 53 | -0.655290316 | ECI1 | enoyl-CoA delta isomerase 1 [Source:HGNC Symbol;Acc:HGNC:2703] |
| ENSG00000168487 | HGLibA_04591 | 53 | -0.655290316 | BMP1 | bone morphogenetic protein 1 [Source:HGNC Symbol;Acc:HGNC:1067] |
| ENSG00000169045 | HGLibA_21821 | 53 | -0.655290316 | HNRNPH1 | heterogeneous nuclear ribonucleoprotein H1 [Source:HGNC Symbol;Acc:HGNC:5041] |
| ENSG00000169224 | HGLibA_18830 | 53 | -0.655290316 | GCSAML | germinal center associated signaling and motility like [Source:HGNC Symbol;Acc:HGNC:29583] |
| ENSG00000171402 | HGLibA_54483 | 53 | -0.655290316 | XAGE3 | X antigen family member 3 [Source:HGNC Symbol;Acc:HGNC:14618] |
| ENSG00000171487 | HGLibA_32125 | 53 | -0.655290316 | NLRP5 | NLR family pyrin domain containing 5 [Source:HGNC Symbol;Acc:HGNC:21269] |
| ENSG00000171916 | HGLibA_26388 | 53 | -0.655290316 | LGALS9C | galectin 9C [Source:HGNC Symbol;Acc:HGNC:33874] |
| ENSG00000172073 | HGLibA_49107 | 53 | -0.655290316 | TEX37 | testis expressed 37 [Source:HGNC Symbol;Acc:HGNC:26341] |
| ENSG00000172264 | HGLibA_27839 | 53 | -0.655290316 | MACROD2 | MACRO domain containing 2 [Source:HGNC Symbol;Acc:HGNC:16126] |
| ENSG00000172867 | HGLibA_25443 | 53 | -0.655290316 | KRT2 | keratin 2 [Source:HGNC Symbol;Acc:HGNC:6439] |
| ENSG00000173272 | HGLibA_30942 | 53 | -0.655290316 | MZT2A | mitotic spindle organizing protein 2A [Source:HGNC Symbol;Acc:HGNC:33187] |
| ENSG00000173825 | HGLibA_49468 | 53 | -0.655290316 | TIGD3 | tigger transposable element derived 3 [Source:HGNC Symbol;Acc:HGNC:18334] |
| ENSG00000174453 | HGLibA_53926 | 53 | -0.655290316 | VWC2L | von Willebrand factor C domain containing 2 like [Source:HGNC Symbol;Acc:HGNC:37203] |
| ENSG00000175029 | HGLibA_11586 | 53 | -0.655290316 | CTBP2 | C-terminal binding protein 2 [Source:HGNC Symbol;Acc:HGNC:2495] |
| ENSG00000176438 | HGLibA_47982 | 53 | -0.655290316 | SYNE3 | spectrin repeat containing nuclear envelope family member 3 [Source:HGNC Symbol;Acc:HGNC:19861] |
| ENSG00000177238 | HGLibA_51602 | 53 | -0.655290316 | TRIM72 | tripartite motif containing 72 [Source:HGNC Symbol;Acc:HGNC:32671] |
| ENSG00000180061 | HGLibA_49994 | 53 | -0.655290316 | TMEM150B | transmembrane protein 150B [Source:HGNC Symbol;Acc:HGNC:34415] |
| ENSG00000180432 | HGLibA_12218 | 53 | -0.655290316 | CYP8B1 | cytochrome P450 family 8 subfamily B member 1 [Source:HGNC Symbol;Acc:HGNC:2653] |
| ENSG00000181195 | HGLibA_36047 | 53 | -0.655290316 | PENK | proenkephalin [Source:HGNC Symbol;Acc:HGNC:8831] |
| ENSG00000181374 | HGLibA_07968 | 53 | -0.655290316 | CCL13 | C-C motif chemokine ligand 13 [Source:HGNC Symbol;Acc:HGNC:10611] |
| ENSG00000181698 | HGLibA_34356 | 53 | -0.655290316 | OR5T1 | olfactory receptor family 5 subfamily T member 1 [Source:HGNC Symbol;Acc:HGNC:14821] |
| ENSG00000182950 | HGLibA_33290 | 53 | -0.655290316 | ODF3L1 | outer dense fiber of sperm tails 3 like 1 [Source:HGNC Symbol;Acc:HGNC:28735] |
| ENSG00000183166 | HGLibA_07044 | 53 | -0.655290316 | CALN1 | calneuron 1 [Source:HGNC Symbol;Acc:HGNC:13248] |
| ENSG00000183625 | HGLibA_08153 | 53 | -0.655290316 | CCR3 | C-C motif chemokine receptor 3 [Source:HGNC Symbol;Acc:HGNC:1604] |
| ENSG00000184155 | HGLibA_33527 | 53 | -0.655290316 | OR10J5 | olfactory receptor family 10 subfamily J member 5 [Source:HGNC Symbol;Acc:HGNC:14993] |
| ENSG00000184939 | HGLibA_55241 | 53 | -0.655290316 | ZFP90 | ZFP90 zinc finger protein [Source:HGNC Symbol;Acc:HGNC:23329] |
| ENSG00000185436 | HGLibA_22732 | 53 | -0.655290316 | IFNLR1 | interferon lambda receptor 1 [Source:HGNC Symbol;Acc:HGNC:18584] |
| ENSG00000185467 | HGLibA_25371 | 53 | -0.655290316 | KPNA7 | karyopherin subunit alpha 7 [Source:HGNC Symbol;Acc:HGNC:21839] |
| ENSG00000185621 | HGLibA_26690 | 53 | -0.655290316 | LMLN | leishmanolysin like peptidase [Source:HGNC Symbol;Acc:HGNC:15991] |
| ENSG00000185958 | HGLibA_16335 | 53 | -0.655290316 | FAM186A | family with sequence similarity 186 member A [Source:HGNC Symbol;Acc:HGNC:26980] |
| ENSG00000186334 | HGLibA_45038 | 53 | -0.655290316 | SLC36A3 | solute carrier family 36 member 3 [Source:HGNC Symbol;Acc:HGNC:19659] |
| ENSG00000186352 | HGLibA_02089 | 53 | -0.655290316 | ANKRD37 | ankyrin repeat domain 37 [Source:HGNC Symbol;Acc:HGNC:29593] |
| ENSG00000186567 | HGLibA_08930 | 53 | -0.655290316 | CEACAM19 | carcinoembryonic antigen related cell adhesion molecule 19 [Source:HGNC Symbol;Acc:HGNC:31951] |
| ENSG00000186654 | HGLibA_38841 | 53 | -0.655290316 | PRR5 | proline rich 5 [Source:HGNC Symbol;Acc:HGNC:31682] |
| ENSG00000187037 | HGLibA_19909 | 53 | -0.655290316 | GPR141 | G protein-coupled receptor 141 [Source:HGNC Symbol;Acc:HGNC:19997] |
| ENSG00000188227 | HGLibA_56774 | 53 | -0.655290316 | ZNF793 | zinc finger protein 793 [Source:HGNC Symbol;Acc:HGNC:33115] |
| ENSG00000188313 | HGLibA_37230 | 53 | -0.655290316 | PLSCR1 | phospholipid scramblase 1 [Source:HGNC Symbol;Acc:HGNC:9092] |
| ENSG00000188517 | HGLibA_10550 | 53 | -0.655290316 | COL25A1 | collagen type XXV alpha 1 chain [Source:HGNC Symbol;Acc:HGNC:18603] |
| ENSG00000188620 | HGLibA_21766 | 53 | -0.655290316 | HMX3 | H6 family homeobox 3 [Source:HGNC Symbol;Acc:HGNC:5019] |
| ENSG00000189266 | HGLibA_37437 | 53 | -0.655290316 | PNRC2 | proline rich nuclear receptor coactivator 2 [Source:HGNC Symbol;Acc:HGNC:23158] |
| ENSG00000196169 | HGLibA_24879 | 53 | -0.655290316 | KIF19 | kinesin family member 19 [Source:HGNC Symbol;Acc:HGNC:26735] |
| ENSG00000196378 | HGLibA_55837 | 53 | -0.655290316 | ZNF34 | zinc finger protein 34 [Source:HGNC Symbol;Acc:HGNC:13098] |
| ENSG00000196419 | HGLibA_54597 | 53 | -0.655290316 | XRCC6 | X-ray repair cross complementing 6 [Source:HGNC Symbol;Acc:HGNC:4055] |
| ENSG00000196502 | HGLibA_47730 | 53 | -0.655290316 | SULT1A1 | sulfotransferase family 1A member 1 [Source:HGNC Symbol;Acc:HGNC:11453] |
| ENSG00000196743 | HGLibA_19387 | 53 | -0.655290316 | GM2A | GM2 ganglioside activator [Source:HGNC Symbol;Acc:HGNC:4367] |
| ENSG00000197165 | HGLibA_47733 | 53 | -0.655290316 | SULT1A2 | sulfotransferase family 1A member 2 [Source:HGNC Symbol;Acc:HGNC:11454] |
| ENSG00000197208 | HGLibA_44587 | 53 | -0.655290316 | SLC22A4 | solute carrier family 22 member 4 [Source:HGNC Symbol;Acc:HGNC:10968] |
| ENSG00000197249 | HGLibA_43506 | 53 | -0.655290316 | SERPINA1 | serpin family A member 1 [Source:HGNC Symbol;Acc:HGNC:8941] |
| ENSG00000197302 | HGLibA_56626 | 53 | -0.655290316 | ZNF720 | zinc finger protein 720 [Source:HGNC Symbol;Acc:HGNC:26987] |
| ENSG00000197361 | HGLibA_17002 | 53 | -0.655290316 | FBXL22 | F-box and leucine rich repeat protein 22 [Source:HGNC Symbol;Acc:HGNC:27537] |
| ENSG00000197467 | HGLibA_10509 | 53 | -0.655290316 | COL13A1 | collagen type XIII alpha 1 chain [Source:HGNC Symbol;Acc:HGNC:2190] |
| ENSG00000198791 | HGLibA_10349 | 53 | -0.655290316 | CNOT7 | CCR4-NOT transcription complex subunit 7 [Source:HGNC Symbol;Acc:HGNC:14101] |
| ENSG00000198848 | HGLibA_09218 | 53 | -0.655290316 | CES1 | carboxylesterase 1 [Source:HGNC Symbol;Acc:HGNC:1863] |
| ENSG00000203783 | HGLibA_38855 | 53 | -0.655290316 | PRR9 | proline rich 9 [Source:HGNC Symbol;Acc:HGNC:32057] |
| ENSG00000203909 | HGLibA_13896 | 53 | -0.655290316 | DPPA5 | developmental pluripotency associated 5 [Source:HGNC Symbol;Acc:HGNC:19201] |
| ENSG00000204397 | HGLibA_07226 | 53 | -0.655290316 | CARD16 | caspase recruitment domain family member 16 [Source:HGNC Symbol;Acc:HGNC:33701] |
| ENSG00000204701 | HGLibA_33812 | 53 | -0.655290316 | OR2J3 | olfactory receptor family 2 subfamily J member 3 [Source:HGNC Symbol;Acc:HGNC:8261] |
| ENSG00000205542 | HGLibA_50665 | 53 | -0.655290316 | TMSB4X | thymosin beta 4 X-linked [Source:HGNC Symbol;Acc:HGNC:11881] |
| ENSG00000205609 | HGLibA_14771 | 53 | -0.655290316 | EIF3CL | eukaryotic translation initiation factor 3 subunit C like [Source:HGNC Symbol;Acc:HGNC:26347] |
| ENSG00000214360 | HGLibA_14556 | 53 | -0.655290316 | EFCAB9 | EF-hand calcium binding domain 9 [Source:HGNC Symbol;Acc:HGNC:34530] |
| ENSG00000217236 | HGLibA_46319 | 53 | -0.655290316 | SP9 | Sp9 transcription factor [Source:HGNC Symbol;Acc:HGNC:30690] |
| ENSG00000221932 | HGLibA_21151 | 53 | -0.655290316 | HEPN1 | down-regulated 1 [Source:HGNC Symbol;Acc:HGNC:34400]" |
| ENSG00000222036 | HGLibA_37746 | 53 | -0.655290316 | POTEM | POTE ankyrin domain family member M [Source:HGNC Symbol;Acc:HGNC:37096] |
| ENSG00000236981 | HGLibA_33503 | 53 | -0.655290316 | OR10G9 | olfactory receptor family 10 subfamily G member 9 [Source:HGNC Symbol;Acc:HGNC:15129] |
| ENSG00000243414 | HGLibA_49448 | 53 | -0.655290316 | TICAM2 | toll like receptor adaptor molecule 2 [Source:HGNC Symbol;Acc:HGNC:21354] |
| ENSG00000251201 | HGLibA_49823 | 53 | -0.655290316 | TMED7-TICAM2 | TMED7-TICAM2 readthrough [Source:HGNC Symbol;Acc:HGNC:33945] |
| ENSG00000253293 | HGLibA_21895 | 53 | -0.655290316 | HOXA10 | homeobox A10 [Source:HGNC Symbol;Acc:HGNC:5100] |
| ENSG00000256436 | HGLibA_48391 | 53 | -0.655290316 | TAS2R31 | taste 2 receptor member 31 [Source:HGNC Symbol;Acc:HGNC:19113] |
| ENSG00000262664 | HGLibA_34788 | 53 | -0.655290316 | OVCA2 | serine hydrolase domain containing [Source:HGNC Symbol;Acc:HGNC:24203]" |
| ENSG00000264230 | HGLibA_02254 | 53 | -0.655290316 | ANXA8L1 | annexin A8 like 1 [Source:HGNC Symbol;Acc:HGNC:23334] |
| ENSG00000268629 | HGLibA_49059 | 53 | -0.655290316 | TEX13A | testis expressed 13A [Source:HGNC Symbol;Acc:HGNC:11735] |
| ENSG00000269791 | HGLibA_47212 | 53 | -0.655290316 | SSX4B | SSX family member 4B [Source:HGNC Symbol;Acc:HGNC:16880] |
| ENSG00000275066 | HGLibA_48029 | 53 | -0.655290316 | SYNRG | synergin gamma [Source:HGNC Symbol;Acc:HGNC:557] |
| ENSG00000275896 | HGLibA_38908 | 53 | -0.655290316 | PRSS2 | serine protease 2 [Source:HGNC Symbol;Acc:HGNC:9483] |
| FAM19A5 | HGLibA_16409 | 53 | -0.655290316 | FAM19A5 | - |
| hsa-let-7e | HGLibA_57122 | 53 | -0.655290316 | hsa-let-7e | - |
| hsa-mir-10a | HGLibA_57197 | 53 | -0.655290316 | hsa-mir-10a | - |
| hsa-mir-1180 | HGLibA_57213 | 53 | -0.655290316 | hsa-mir-1180 | - |
| hsa-mir-218-2 | HGLibA_58273 | 53 | -0.655290316 | hsa-mir-218-2 | - |
| hsa-mir-3119-1 | HGLibA_58526 | 53 | -0.655290316 | hsa-mir-3119-1 | - |
| hsa-mir-320c-2 | HGLibA_58940 | 53 | -0.655290316 | hsa-mir-320c-2 | - |
| hsa-mir-331 | HGLibA_58990 | 53 | -0.655290316 | hsa-mir-331 | - |
| hsa-mir-338 | HGLibA_59004 | 53 | -0.655290316 | hsa-mir-338 | - |
| hsa-mir-3919 | HGLibA_59553 | 53 | -0.655290316 | hsa-mir-3919 | - |
| hsa-mir-421 | HGLibA_59692 | 53 | -0.655290316 | hsa-mir-421 | - |
| hsa-mir-4438 | HGLibA_60153 | 53 | -0.655290316 | hsa-mir-4438 | - |
| hsa-mir-4490 | HGLibA_60379 | 53 | -0.655290316 | hsa-mir-4490 | - |
| hsa-mir-4653 | HGLibA_60718 | 53 | -0.655290316 | hsa-mir-4653 | - |
| hsa-mir-4706 | HGLibA_60926 | 53 | -0.655290316 | hsa-mir-4706 | - |
| hsa-mir-4709 | HGLibA_60935 | 53 | -0.655290316 | hsa-mir-4709 | - |
| hsa-mir-4739 | HGLibA_61055 | 53 | -0.655290316 | hsa-mir-4739 | - |
| hsa-mir-548ah | HGLibA_61882 | 53 | -0.655290316 | hsa-mir-548ah | - |

| | | | | | |
|---|---|---|---|---|---|
| hsa-mir-601 | HGLibA_62496 | 53 | -0.655290316 | hsa-mir-601 | - |
| hsa-mir-6075 | HGLibA_62552 | 53 | -0.655290316 | hsa-mir-6075 | - |
| hsa-mir-623 | HGLibA_62732 | 53 | -0.655290316 | hsa-mir-623 | - |
| hsa-mir-6510 | HGLibA_62892 | 53 | -0.655290316 | hsa-mir-6510 | - |
| hsa-mir-6805 | HGLibA_63399 | 53 | -0.655290316 | hsa-mir-6805 | - |
| hsa-mir-8059 | HGLibA_64075 | 53 | -0.655290316 | hsa-mir-8059 | - |
| hsa-mir-877 | HGLibA_64217 | 53 | -0.655290316 | hsa-mir-877 | - |
| hsa-mir-924 | HGLibA_64288 | 53 | -0.655290316 | hsa-mir-924 | - |
| LOC100129520 | HGLibA_26764 | 53 | -0.655290316 | LOC100129520 | - |
| LOC100505679 | HGLibA_26853 | 53 | -0.655290316 | LOC100505679 | - |
| PRHOXNB | HGLibA_38468 | 53 | -0.655290316 | PRHOXNB | - |
| TMEM183B | HGLibA_50107 | 53 | -0.655290316 | TMEM183B | - |
| 5-Mar | HGLibA_28340 | 52 | -0.664335659 | 5-Mar | - |
| ADSS | HGLibA_01077 | 52 | -0.664335659 | ADSS | - |
| ASMT_Y | HGLibA_03322 | 52 | -0.664335659 | ASMT_Y | - |
| C15orf60 | HGLibA_05425 | 52 | -0.664335659 | C15orf60 | - |
| C1orf106 | HGLibA_05739 | 52 | -0.664335659 | C1orf106 | - |
| C6orf211 | HGLibA_06472 | 52 | -0.664335659 | C6orf211 | - |
| CCDC23 | HGLibA_07732 | 52 | -0.664335659 | CCDC23 | - |
| DIEXF | HGLibA_13233 | 52 | -0.664335659 | DIEXF | - |
| ENSG00000002933 | HGLibA_50073 | 52 | -0.664335659 | TMEM176A | transmembrane protein 176A [Source:HGNC Symbol;Acc:HGNC:24930] |
| ENSG00000006837 | HGLibA_08813 | 52 | -0.664335659 | CDKL3 | cyclin dependent kinase like 3 [Source:HGNC Symbol;Acc:HGNC:15483] |
| ENSG00000010704 | HGLibA_21225 | 52 | -0.664335659 | HFE | homeostatic iron regulator [Source:HGNC Symbol;Acc:HGNC:4886] |
| ENSG00000011304 | HGLibA_39251 | 52 | -0.664335659 | PTBP1 | polypyrimidine tract binding protein 1 [Source:HGNC Symbol;Acc:HGNC:9583] |
| ENSG00000025800 | HGLibA_25368 | 52 | -0.664335659 | KPNA6 | karyopherin subunit alpha 6 [Source:HGNC Symbol;Acc:HGNC:6399] |
| ENSG00000033030 | HGLibA_55048 | 52 | -0.664335659 | ZCCHC8 | zinc finger CCHC-type containing 8 [Source:HGNC Symbol;Acc:HGNC:25265] |
| ENSG00000035681 | HGLibA_32762 | 52 | -0.664335659 | NSMAF | neutral sphingomyelinase activation associated factor [Source:HGNC Symbol;Acc:HGNC:8017] |
| ENSG00000048740 | HGLibA_09007 | 52 | -0.664335659 | CELF2 | CUGBP Elav-like family member 2 [Source:HGNC Symbol;Acc:HGNC:2550] |
| ENSG00000065183 | HGLibA_54060 | 52 | -0.664335659 | WDR3 | WD repeat domain 3 [Source:HGNC Symbol;Acc:HGNC:12755] |
| ENSG00000068323 | HGLibA_49160 | 52 | -0.664335659 | TFE3 | transcription factor binding to IGHM enhancer 3 [Source:HGNC Symbol;Acc:HGNC:11752] |
| ENSG00000068912 | HGLibA_15490 | 52 | -0.664335659 | ERLEC1 | endoplasmic reticulum lectin 1 [Source:HGNC Symbol;Acc:HGNC:25222] |
| ENSG00000070367 | HGLibA_15751 | 52 | -0.664335659 | EXOC5 | exocyst complex component 5 [Source:HGNC Symbol;Acc:HGNC:10696] |
| ENSG00000078589 | HGLibA_34870 | 52 | -0.664335659 | P2RY10 | P2Y receptor family member 10 [Source:HGNC Symbol;Acc:HGNC:19906] |
| ENSG00000086848 | HGLibA_01641 | 52 | -0.664335659 | ALG9 | 2-mannosyltransferase [Source:HGNC Symbol;Acc:HGNC:15672]" |
| ENSG00000088827 | HGLibA_44122 | 52 | -0.664335659 | SIGLEC1 | sialic acid binding Ig like lectin 1 [Source:HGNC Symbol;Acc:HGNC:11127] |
| ENSG00000100024 | HGLibA_53054 | 52 | -0.664335659 | UPB1 | beta-ureidopropionase 1 [Source:HGNC Symbol;Acc:HGNC:16297] |
| ENSG00000100433 | HGLibA_24298 | 52 | -0.664335659 | KCNK10 | potassium two pore domain channel subfamily K member 10 [Source:HGNC Symbol;Acc:HGNC:6273] |
| ENSG00000100889 | HGLibA_35615 | 52 | -0.664335659 | PCK2 | mitochondrial [Source:HGNC Symbol;Acc:HGNC:8725]" |
| ENSG00000101251 | HGLibA_43279 | 52 | -0.664335659 | SEL1L2 | ERAD E3 ligase adaptor subunit [Source:HGNC Symbol;Acc:HGNC:15897]" |
| ENSG00000101265 | HGLibA_40376 | 52 | -0.664335659 | RASSF2 | Ras association domain family member 2 [Source:HGNC Symbol;Acc:HGNC:9883] |
| ENSG00000102021 | HGLibA_27645 | 52 | -0.664335659 | LUZP4 | leucine zipper protein 4 [Source:HGNC Symbol;Acc:HGNC:24971] |
| ENSG00000102103 | HGLibA_38248 | 52 | -0.664335659 | PQBP1 | polyglutamine binding protein 1 [Source:HGNC Symbol;Acc:HGNC:9330] |
| ENSG00000102471 | HGLibA_31396 | 52 | -0.664335659 | NDFIP2 | Nedd4 family interacting protein 2 [Source:HGNC Symbol;Acc:HGNC:18537] |
| ENSG00000103429 | HGLibA_04453 | 52 | -0.664335659 | BFAR | bifunctional apoptosis regulator [Source:HGNC Symbol;Acc:HGNC:17613] |
| ENSG00000103995 | HGLibA_09111 | 52 | -0.664335659 | CEP152 | centrosomal protein 152 [Source:HGNC Symbol;Acc:HGNC:29298] |
| ENSG00000104112 | HGLibA_42903 | 52 | -0.664335659 | SCG3 | secretogranin III [Source:HGNC Symbol;Acc:HGNC:13707] |
| ENSG00000105997 | HGLibA_21907 | 52 | -0.664335659 | HOXA3 | homeobox A3 [Source:HGNC Symbol;Acc:HGNC:5104] |
| ENSG00000106078 | HGLibA_10460 | 52 | -0.664335659 | COBL | cordon-bleu WH2 repeat protein [Source:HGNC Symbol;Acc:HGNC:22199] |
| ENSG00000106733 | HGLibA_32197 | 52 | -0.664335659 | NMRK1 | nicotinamide riboside kinase 1 [Source:HGNC Symbol;Acc:HGNC:26057] |
| ENSG00000106803 | HGLibA_43252 | 52 | -0.664335659 | SEC61B | Sec61 translocon beta subunit [Source:HGNC Symbol;Acc:HGNC:16993] |
| ENSG00000107672 | HGLibA_32773 | 52 | -0.664335659 | NSMCE4A | SMC5-SMC6 complex component [Source:HGNC Symbol;Acc:HGNC:25935]" |
| ENSG00000108878 | HGLibA_06929 | 52 | -0.664335659 | CACNG1 | calcium voltage-gated channel auxiliary subunit gamma 1 [Source:HGNC Symbol;Acc:HGNC:1405] |
| ENSG00000109103 | HGLibA_52988 | 52 | -0.664335659 | UNC119 | unc-119 lipid binding chaperone [Source:HGNC Symbol;Acc:HGNC:12565] |
| ENSG00000111335 | HGLibA_33202 | 52 | -0.664335659 | OAS2 | 2'-5'-oligoadenylate synthetase 2 [Source:HGNC Symbol;Acc:HGNC:8087] |
| ENSG00000111704 | HGLibA_31089 | 52 | -0.664335659 | NANOG | Nanog homeobox [Source:HGNC Symbol;Acc:HGNC:20857] |
| ENSG00000111713 | HGLibA_20712 | 52 | -0.664335659 | GYS2 | glycogen synthase 2 [Source:HGNC Symbol;Acc:HGNC:4707] |
| ENSG00000111728 | HGLibA_47289 | 52 | -0.664335659 | ST8SIA1 | 8-sialyltransferase 1 [Source:HGNC Symbol;Acc:HGNC:10869]" |
| ENSG00000111859 | HGLibA_31613 | 52 | -0.664335659 | NEDD9 | developmentally down-regulated 9 [Source:HGNC Symbol;Acc:HGNC:7733]" |
| ENSG00000112584 | HGLibA_16060 | 52 | -0.664335659 | FAM120B | family with sequence similarity 120B [Source:HGNC Symbol;Acc:HGNC:21109] |
| ENSG00000113396 | HGLibA_44827 | 52 | -0.664335659 | SLC27A6 | solute carrier family 27 member 6 [Source:HGNC Symbol;Acc:HGNC:11000] |
| ENSG00000114107 | HGLibA_09158 | 52 | -0.664335659 | CEP70 | centrosomal protein 70 [Source:HGNC Symbol;Acc:HGNC:29972] |
| ENSG00000114125 | HGLibA_41697 | 52 | -0.664335659 | RNF7 | ring finger protein 7 [Source:HGNC Symbol;Acc:HGNC:10070] |
| ENSG00000114999 | HGLibA_52182 | 52 | -0.664335659 | TTL | tubulin tyrosine ligase [Source:HGNC Symbol;Acc:HGNC:21586] |
| ENSG00000115361 | HGLibA_00364 | 52 | -0.664335659 | ACADL | acyl-CoA dehydrogenase long chain [Source:HGNC Symbol;Acc:HGNC:88] |
| ENSG00000115474 | HGLibA_24258 | 52 | -0.664335659 | KCNJ13 | potassium voltage-gated channel subfamily J member 13 [Source:HGNC Symbol;Acc:HGNC:6259] |
| ENSG00000115525 | HGLibA_47249 | 52 | -0.664335659 | ST3GAL5 | 3-sialyltransferase 5 [Source:HGNC Symbol;Acc:HGNC:10872]" |
| ENSG00000116199 | HGLibA_16447 | 52 | -0.664335659 | FAM20B | glycosaminoglycan xylosylkinase [Source:HGNC Symbol;Acc:HGNC:23017]" |
| ENSG00000117410 | HGLibA_03713 | 52 | -0.664335659 | ATP6V0B | ATPase H+ transporting V0 subunit b [Source:HGNC Symbol;Acc:HGNC:861] |
| ENSG00000117500 | HGLibA_49815 | 52 | -0.664335659 | TMED5 | transmembrane p24 trafficking protein 5 [Source:HGNC Symbol;Acc:HGNC:24251] |
| ENSG00000118007 | HGLibA_47322 | 52 | -0.664335659 | STAG1 | stromal antigen 1 [Source:HGNC Symbol;Acc:HGNC:11354] |
| ENSG00000118271 | HGLibA_52228 | 52 | -0.664335659 | TTR | transthyretin [Source:HGNC Symbol;Acc:HGNC:12405] |
| ENSG00000118473 | HGLibA_43814 | 52 | -0.664335659 | SGIP1 | SH3 domain GRB2 like endophilin interacting protein 1 [Source:HGNC Symbol;Acc:HGNC:25412] |
| ENSG00000118526 | HGLibA_48773 | 52 | -0.664335659 | TCF21 | transcription factor 21 [Source:HGNC Symbol;Acc:HGNC:11632] |
| ENSG00000119231 | HGLibA_43393 | 52 | -0.664335659 | SENP5 | SUMO specific peptidase 5 [Source:HGNC Symbol;Acc:HGNC:28407] |
| ENSG00000119559 | HGLibA_05643 | 52 | -0.664335659 | C19orf25 | chromosome 19 open reading frame 25 [Source:HGNC Symbol;Acc:HGNC:26711] |
| ENSG00000120265 | HGLibA_35624 | 52 | -0.664335659 | PCMT1 | protein-L-isoaspartate (D-aspartate) O-methyltransferase [Source:HGNC Symbol;Acc:HGNC:8728] |
| ENSG00000120306 | HGLibA_12233 | 52 | -0.664335659 | CYSTM1 | cysteine rich transmembrane module containing 1 [Source:HGNC Symbol;Acc:HGNC:30239] |
| ENSG00000122188 | HGLibA_26069 | 52 | -0.664335659 | LAX1 | lymphocyte transmembrane adaptor 1 [Source:HGNC Symbol;Acc:HGNC:26005] |
| ENSG00000123096 | HGLibA_47148 | 52 | -0.664335659 | SSPN | sarcospan [Source:HGNC Symbol;Acc:HGNC:11322] |
| ENSG00000123572 | HGLibA_32700 | 52 | -0.664335659 | NRK | Nik related kinase [Source:HGNC Symbol;Acc:HGNC:25391] |
| ENSG00000124116 | HGLibA_54265 | 52 | -0.664335659 | WFDC3 | WAP four-disulfide core domain 3 [Source:HGNC Symbol;Acc:HGNC:15957] |
| ENSG00000124659 | HGLibA_48585 | 52 | -0.664335659 | TBCC | tubulin folding cofactor C [Source:HGNC Symbol;Acc:HGNC:11580] |
| ENSG00000125538 | HGLibA_23040 | 52 | -0.664335659 | IL1B | interleukin 1 beta [Source:HGNC Symbol;Acc:HGNC:5992] |
| ENSG00000126001 | HGLibA_09127 | 52 | -0.664335659 | CEP250 | centrosomal protein 250 [Source:HGNC Symbol;Acc:HGNC:1859] |
| ENSG00000129128 | HGLibA_46565 | 52 | -0.664335659 | SPCS3 | signal peptidase complex subunit 3 [Source:HGNC Symbol;Acc:HGNC:26212] |
| ENSG00000130024 | HGLibA_36333 | 52 | -0.664335659 | PHF10 | PHD finger protein 10 [Source:HGNC Symbol;Acc:HGNC:18250] |
| ENSG00000130560 | HGLibA_52533 | 52 | -0.664335659 | UBAC1 | UBA domain containing 1 [Source:HGNC Symbol;Acc:HGNC:30221] |
| ENSG00000130935 | HGLibA_32259 | 52 | -0.664335659 | NOL11 | nucleolar protein 11 [Source:HGNC Symbol;Acc:HGNC:24557] |
| ENSG00000131653 | HGLibA_51266 | 52 | -0.664335659 | TRAF7 | TNF receptor associated factor 7 [Source:HGNC Symbol;Acc:HGNC:20456] |
| ENSG00000131738 | HGLibA_25484 | 52 | -0.664335659 | KRT33B | keratin 33B [Source:HGNC Symbol;Acc:HGNC:6451] |
| ENSG00000132170 | HGLibA_37844 | 52 | -0.664335659 | PPARG | peroxisome proliferator activated receptor gamma [Source:HGNC Symbol;Acc:HGNC:9236] |
| ENSG00000132688 | HGLibA_31697 | 52 | -0.664335659 | NES | nestin [Source:HGNC Symbol;Acc:HGNC:7756] |
| ENSG00000132716 | HGLibA_12458 | 52 | -0.664335659 | DCAF8 | DDB1 and CUL4 associated factor 8 [Source:HGNC Symbol;Acc:HGNC:24891] |
| ENSG00000133112 | HGLibA_51210 | 52 | -0.664335659 | TPT1 | translationally-controlled 1 [Source:HGNC Symbol;Acc:HGNC:12022]" |
| ENSG00000133134 | HGLibA_04443 | 52 | -0.664335659 | BEX2 | brain expressed X-linked 2 [Source:HGNC Symbol;Acc:HGNC:30933] |
| ENSG00000135334 | HGLibA_01461 | 52 | -0.664335659 | AKIRIN2 | akirin 2 [Source:HGNC Symbol;Acc:HGNC:21407] |
| ENSG00000135722 | HGLibA_17022 | 52 | -0.664335659 | FBXL8 | F-box and leucine rich repeat protein 8 [Source:HGNC Symbol;Acc:HGNC:17875] |
| ENSG00000136153 | HGLibA_26714 | 52 | -0.664335659 | LMO7 | LIM domain 7 [Source:HGNC Symbol;Acc:HGNC:6646] |
| ENSG00000136240 | HGLibA_24483 | 52 | -0.664335659 | KDELR2 | KDEL endoplasmic reticulum protein retention receptor 2 [Source:HGNC Symbol;Acc:HGNC:6305] |
| ENSG00000136758 | HGLibA_54701 | 52 | -0.664335659 | YME1L1 | YME1 like 1 ATPase [Source:HGNC Symbol;Acc:HGNC:12843] |
| ENSG00000136877 | HGLibA_17959 | 52 | -0.664335659 | FPGS | folylpolyglutamate synthase [Source:HGNC Symbol;Acc:HGNC:3824] |
| ENSG00000136908 | HGLibA_13860 | 52 | -0.664335659 | DPM2 | regulatory [Source:HGNC Symbol;Acc:HGNC:3006]" |
| ENSG00000137491 | HGLibA_45462 | 52 | -0.664335659 | SLCO2B1 | solute carrier organic anion transporter family member 2B1 [Source:HGNC Symbol;Acc:HGNC:10962] |
| ENSG00000137513 | HGLibA_31158 | 52 | -0.664335659 | NARS2 | mitochondrial [Source:HGNC Symbol;Acc:HGNC:26274]" |
| ENSG00000138629 | HGLibA_52713 | 52 | -0.664335659 | UBL7 | ubiquitin like 7 [Source:HGNC Symbol;Acc:HGNC:28221] |
| ENSG00000139220 | HGLibA_37877 | 52 | -0.664335659 | PPFIA2 | PTPRF interacting protein alpha 2 [Source:HGNC Symbol;Acc:HGNC:9246] |
| ENSG00000139645 | HGLibA_02117 | 52 | -0.664335659 | ANKRD52 | ankyrin repeat domain 52 [Source:HGNC Symbol;Acc:HGNC:26614] |
| ENSG00000139826 | HGLibA_00245 | 52 | -0.664335659 | ABHD13 | abhydrolase domain containing 13 [Source:HGNC Symbol;Acc:HGNC:20293] |
| ENSG00000139874 | HGLibA_47173 | 52 | -0.664335659 | SSTR1 | somatostatin receptor 1 [Source:HGNC Symbol;Acc:HGNC:11330] |
| ENSG00000141096 | HGLibA_13824 | 52 | -0.664335659 | DPEP3 | dipeptidase 3 [Source:HGNC Symbol;Acc:HGNC:23029] |
| ENSG00000141504 | HGLibA_42773 | 52 | -0.664335659 | SAT2 | spermidine/spermine N1-acetyltransferase family member 2 [Source:HGNC Symbol;Acc:HGNC:23160] |
| ENSG00000143815 | HGLibA_26082 | 52 | -0.664335659 | LBR | lamin B receptor [Source:HGNC Symbol;Acc:HGNC:6518] |
| ENSG00000143870 | HGLibA_35886 | 52 | -0.664335659 | PDIA6 | protein disulfide isomerase family A member 6 [Source:HGNC Symbol;Acc:HGNC:30168] |
| ENSG00000144043 | HGLibA_49081 | 52 | -0.664335659 | TEX261 | testis expressed 261 [Source:HGNC Symbol;Acc:HGNC:30712] |
| ENSG00000147274 | HGLibA_40597 | 52 | -0.664335659 | RBMX | RNA binding motif protein X-linked [Source:HGNC Symbol;Acc:HGNC:9910] |
| ENSG00000148300 | HGLibA_40900 | 52 | -0.664335659 | REXO4 | 3'-5' exonuclease [Source:HGNC Symbol;Acc:HGNC:12820]" |
| ENSG00000148826 | HGLibA_32057 | 52 | -0.664335659 | NKX6-2 | NK6 homeobox 2 [Source:HGNC Symbol;Acc:HGNC:19321] |
| ENSG00000149124 | HGLibA_19369 | 52 | -0.664335659 | GLYAT | glycine-N-acyltransferase [Source:HGNC Symbol;Acc:HGNC:13734] |
| ENSG00000150990 | HGLibA_13192 | 52 | -0.664335659 | DHX37 | DEAH-box helicase 37 [Source:HGNC Symbol;Acc:HGNC:17210] |
| ENSG00000152520 | HGLibA_35093 | 52 | -0.664335659 | PAN3 | poly(A) specific ribonuclease subunit PAN3 [Source:HGNC Symbol;Acc:HGNC:29991] |
| ENSG00000155011 | HGLibA_13298 | 52 | -0.664335659 | DKK2 | dickkopf WNT signaling pathway inhibitor 2 [Source:HGNC Symbol;Acc:HGNC:2892] |
| ENSG00000155158 | HGLibA_52135 | 52 | -0.664335659 | TTC39B | tetratricopeptide repeat domain 39B [Source:HGNC Symbol;Acc:HGNC:23704] |
| ENSG00000155330 | HGLibA_05492 | 52 | -0.664335659 | C16orf87 | chromosome 16 open reading frame 87 [Source:HGNC Symbol;Acc:HGNC:33754] |
| ENSG00000156232 | HGLibA_54290 | 52 | -0.664335659 | WHAMM | golgi membranes and microtubules [Source:HGNC Symbol;Acc:HGNC:30493]" |
| ENSG00000156298 | HGLibA_51947 | 52 | -0.664335659 | TSPAN7 | tetraspanin 7 [Source:HGNC Symbol;Acc:HGNC:11854] |
| ENSG00000156587 | HGLibA_52630 | 52 | -0.664335659 | UBE2L6 | ubiquitin conjugating enzyme E2 L6 [Source:HGNC Symbol;Acc:HGNC:12490] |
| ENSG00000158497 | HGLibA_21740 | 52 | -0.664335659 | HMHB1 | histocompatibility minor HB-1 [Source:HGNC Symbol;Acc:HGNC:29677] |
| ENSG00000158528 | HGLibA_38127 | 52 | -0.664335659 | PPP1R9A | protein phosphatase 1 regulatory subunit 9A [Source:HGNC Symbol;Acc:HGNC:14946] |
| ENSG00000160305 | HGLibA_13248 | 52 | -0.664335659 | DIP2A | disco interacting protein 2 homolog A [Source:HGNC Symbol;Acc:HGNC:17217] |
| ENSG00000160796 | HGLibA_31212 | 52 | -0.664335659 | NBEAL2 | neurobeachin like 2 [Source:HGNC Symbol;Acc:HGNC:31928] |
| ENSG00000160883 | HGLibA_21586 | 52 | -0.664335659 | HK3 | hexokinase 3 [Source:HGNC Symbol;Acc:HGNC:4925] |
| ENSG00000162063 | HGLibA_08091 | 52 | -0.664335659 | CCNF | cyclin F [Source:HGNC Symbol;Acc:HGNC:1591] |
| ENSG00000162078 | HGLibA_55299 | 52 | -0.664335659 | ZG16B | zymogen granule protein 16B [Source:HGNC Symbol;Acc:HGNC:30456] |
| ENSG00000162374 | HGLibA_14889 | 52 | -0.664335659 | ELAVL4 | ELAV like RNA binding protein 4 [Source:HGNC Symbol;Acc:HGNC:3315] |
| ENSG00000162769 | HGLibA_17674 | 52 | -0.664335659 | FLVCR1 | feline leukemia virus subgroup C cellular receptor 1 [Source:HGNC Symbol;Acc:HGNC:24682] |

| | | | | | |
|---|---|---|---|---|---|
| ENSG00000163705 | HGLibA_16841 | 52 | -0.664335659 | FANCD2OS | FANCD2 opposite strand [Source:HGNC Symbol;Acc:HGNC:28623] |
| ENSG00000163811 | HGLibA_54091 | 52 | -0.664335659 | WDR43 | WD repeat domain 43 [Source:HGNC Symbol;Acc:HGNC:28945] |
| ENSG00000163872 | HGLibA_54655 | 52 | -0.664335659 | YEATS2 | YEATS domain containing 2 [Source:HGNC Symbol;Acc:HGNC:25489] |
| ENSG00000164080 | HGLibA_40105 | 52 | -0.664335659 | RAD54L2 | RAD54 like 2 [Source:HGNC Symbol;Acc:HGNC:29123] |
| ENSG00000164087 | HGLibA_37441 | 52 | -0.664335659 | POC1A | POC1 centriolar protein A [Source:HGNC Symbol;Acc:HGNC:24488] |
| ENSG00000164167 | HGLibA_27547 | 52 | -0.664335659 | LSM6 | U6 small nuclear RNA and mRNA degradation associated [Source:HGNC Symbol;Acc:HGNC:17017]" |
| ENSG00000164651 | HGLibA_46316 | 52 | -0.664335659 | SP8 | Sp8 transcription factor [Source:HGNC Symbol;Acc:HGNC:19196] |
| ENSG00000164751 | HGLibA_36107 | 52 | -0.664335659 | PEX2 | peroxisomal biogenesis factor 2 [Source:HGNC Symbol;Acc:HGNC:9717] |
| ENSG00000164930 | HGLibA_18277 | 52 | -0.664335659 | FZD6 | frizzled class receptor 6 [Source:HGNC Symbol;Acc:HGNC:4044] |
| ENSG00000164941 | HGLibA_23433 | 52 | -0.664335659 | INTS8 | integrator complex subunit 8 [Source:HGNC Symbol;Acc:HGNC:26048] |
| ENSG00000164989 | HGLibA_07694 | 52 | -0.664335659 | CCDC171 | coiled-coil domain containing 171 [Source:HGNC Symbol;Acc:HGNC:29828] |
| ENSG00000165443 | HGLibA_36476 | 52 | -0.664335659 | PHYHIPL | phytanoyl-CoA 2-hydroxylase interacting protein like [Source:HGNC Symbol;Acc:HGNC:29378] |
| ENSG00000165526 | HGLibA_42216 | 52 | -0.664335659 | RPUSD4 | RNA pseudouridine synthase D4 [Source:HGNC Symbol;Acc:HGNC:25898] |
| ENSG00000166750 | HGLibA_45497 | 52 | -0.664335659 | SLFN5 | schlafen family member 5 [Source:HGNC Symbol;Acc:HGNC:28286] |
| ENSG00000167775 | HGLibA_08351 | 52 | -0.664335659 | CD320 | CD320 molecule [Source:HGNC Symbol;Acc:HGNC:16692] |
| ENSG00000169439 | HGLibA_43099 | 52 | -0.664335659 | SDC2 | syndecan 2 [Source:HGNC Symbol;Acc:HGNC:10659] |
| ENSG00000169548 | HGLibA_55722 | 52 | -0.664335659 | ZNF280A | zinc finger protein 280A [Source:HGNC Symbol;Acc:HGNC:18597] |
| ENSG00000169710 | HGLibA_16895 | 52 | -0.664335659 | FASN | fatty acid synthase [Source:HGNC Symbol;Acc:HGNC:3594] |
| ENSG00000169905 | HGLibA_51021 | 52 | -0.664335659 | TOR1AIP2 | torsin 1A interacting protein 2 [Source:HGNC Symbol;Acc:HGNC:24055] |
| ENSG00000169972 | HGLibA_39609 | 52 | -0.664335659 | PUSL1 | pseudouridine synthase like 1 [Source:HGNC Symbol;Acc:HGNC:26914] |
| ENSG00000170185 | HGLibA_53353 | 52 | -0.664335659 | USP38 | ubiquitin specific peptidase 38 [Source:HGNC Symbol;Acc:HGNC:20067] |
| ENSG00000170485 | HGLibA_32396 | 52 | -0.664335659 | NPAS2 | neuronal PAS domain protein 2 [Source:HGNC Symbol;Acc:HGNC:7895] |
| ENSG00000170703 | HGLibA_52211 | 52 | -0.664335659 | TTLL6 | tubulin tyrosine ligase like 6 [Source:HGNC Symbol;Acc:HGNC:26664] |
| ENSG00000170835 | HGLibA_08985 | 52 | -0.664335659 | CEL | carboxyl ester lipase [Source:HGNC Symbol;Acc:HGNC:1848] |
| ENSG00000170915 | HGLibA_35172 | 52 | -0.664335659 | PAQR8 | progestin and adipoQ receptor family member 8 [Source:HGNC Symbol;Acc:HGNC:15708] |
| ENSG00000171160 | HGLibA_29674 | 52 | -0.664335659 | MORN4 | MORN repeat containing 4 [Source:HGNC Symbol;Acc:HGNC:24001] |
| ENSG00000172819 | HGLibA_40277 | 52 | -0.664335659 | RARG | retinoic acid receptor gamma [Source:HGNC Symbol;Acc:HGNC:9866] |
| ENSG00000172828 | HGLibA_09224 | 52 | -0.664335659 | CES3 | carboxylesterase 3 [Source:HGNC Symbol;Acc:HGNC:1865] |
| ENSG00000173114 | HGLibA_27475 | 52 | -0.664335659 | LRRN3 | leucine rich repeat neuronal 3 [Source:HGNC Symbol;Acc:HGNC:17200] |
| ENSG00000173349 | HGLibA_43758 | 52 | -0.664335659 | SFT2D3 | SFT2 domain containing 3 [Source:HGNC Symbol;Acc:HGNC:28767] |
| ENSG00000173846 | HGLibA_37193 | 52 | -0.664335659 | PLK3 | polo like kinase 3 [Source:HGNC Symbol;Acc:HGNC:2154] |
| ENSG00000175137 | HGLibA_43936 | 52 | -0.664335659 | SH3BP5L | SH3 binding domain protein 5 like [Source:HGNC Symbol;Acc:HGNC:29360] |
| ENSG00000175161 | HGLibA_06967 | 52 | -0.664335659 | CADM2 | cell adhesion molecule 2 [Source:HGNC Symbol;Acc:HGNC:29849] |
| ENSG00000175309 | HGLibA_36481 | 52 | -0.664335659 | PHYKPL | 5-phosphohydroxy-L-lysine phospho-lyase [Source:HGNC Symbol;Acc:HGNC:28249] |
| ENSG00000176624 | HGLibA_29033 | 52 | -0.664335659 | MEX3C | mex-3 RNA binding family member C [Source:HGNC Symbol;Acc:HGNC:28040] |
| ENSG00000176809 | HGLibA_27333 | 52 | -0.664335659 | LRRC37A3 | leucine rich repeat containing 37 member A3 [Source:HGNC Symbol;Acc:HGNC:32427] |
| ENSG00000178125 | HGLibA_38118 | 52 | -0.664335659 | PPP1R42 | protein phosphatase 1 regulatory subunit 42 [Source:HGNC Symbol;Acc:HGNC:33732] |
| ENSG00000178125 | HGLibA_38119 | 52 | -0.664335659 | PPP1R42 | protein phosphatase 1 regulatory subunit 42 [Source:HGNC Symbol;Acc:HGNC:33732] |
| ENSG00000178363 | HGLibA_07032 | 52 | -0.664335659 | CALML3 | calmodulin like 3 [Source:HGNC Symbol;Acc:HGNC:1452] |
| ENSG00000178531 | HGLibA_11755 | 52 | -0.664335659 | CTXN1 | cortexin 1 [Source:HGNC Symbol;Acc:HGNC:31108] |
| ENSG00000178922 | HGLibA_22453 | 52 | -0.664335659 | HYI | hydroxypyruvate isomerase (putative) [Source:HGNC Symbol;Acc:HGNC:26948] |
| ENSG00000179256 | HGLibA_45667 | 52 | -0.664335659 | SMCO3 | single-pass membrane protein with coiled-coil domains 3 [Source:HGNC Symbol;Acc:HGNC:34401] |
| ENSG00000181104 | HGLibA_15862 | 52 | -0.664335659 | F2R | coagulation factor II thrombin receptor [Source:HGNC Symbol;Acc:HGNC:3537] |
| ENSG00000181192 | HGLibA_13162 | 52 | -0.664335659 | DHTKD1 | dehydrogenase E1 and transketolase domain containing 1 [Source:HGNC Symbol;Acc:HGNC:23537] |
| ENSG00000181781 | HGLibA_33293 | 52 | -0.664335659 | ODF3L2 | outer dense fiber of sperm tails 3 like 2 [Source:HGNC Symbol;Acc:HGNC:26841] |
| ENSG00000182103 | HGLibA_16321 | 52 | -0.664335659 | FAM181B | family with sequence similarity 181 member B [Source:HGNC Symbol;Acc:HGNC:28512] |
| ENSG00000183542 | HGLibA_25294 | 52 | -0.664335659 | KLRC4 | killer cell lectin like receptor C4 [Source:HGNC Symbol;Acc:HGNC:6377] |
| ENSG00000184481 | HGLibA_17926 | 52 | -0.664335659 | FOXO4 | forkhead box O4 [Source:HGNC Symbol;Acc:HGNC:7139] |
| ENSG00000184863 | HGLibA_40534 | 52 | -0.664335659 | RBM33 | RNA binding motif protein 33 [Source:HGNC Symbol;Acc:HGNC:27223] |
| ENSG00000184986 | HGLibA_49902 | 52 | -0.664335659 | TMEM121 | transmembrane protein 121 [Source:HGNC Symbol;Acc:HGNC:20511] |
| ENSG00000185002 | HGLibA_40978 | 52 | -0.664335659 | RFX6 | regulatory factor X6 [Source:HGNC Symbol;Acc:HGNC:21478] |
| ENSG00000185189 | HGLibA_32657 | 52 | -0.664335659 | NRBP2 | nuclear receptor binding protein 2 [Source:HGNC Symbol;Acc:HGNC:19339] |
| ENSG00000185372 | HGLibA_33896 | 52 | -0.664335659 | OR2V1 | olfactory receptor family 2 subfamily V member 1 [Source:HGNC Symbol;Acc:HGNC:8280] |
| ENSG00000185658 | HGLibA_04855 | 52 | -0.664335659 | BRWD1 | bromodomain and WD repeat domain containing 1 [Source:HGNC Symbol;Acc:HGNC:12760] |
| ENSG00000185774 | HGLibA_24243 | 52 | -0.664335659 | KCNIP4 | potassium voltage-gated channel interacting protein 4 [Source:HGNC Symbol;Acc:HGNC:30083] |
| ENSG00000185803 | HGLibA_45247 | 52 | -0.664335659 | SLC52A2 | solute carrier family 52 member 2 [Source:HGNC Symbol;Acc:HGNC:30224] |
| ENSG00000186063 | HGLibA_01327 | 52 | -0.664335659 | AIDA | dorsalization associated [Source:HGNC Symbol;Acc:HGNC:25761]" |
| ENSG00000186393 | HGLibA_25463 | 52 | -0.664335659 | KRT26 | keratin 26 [Source:HGNC Symbol;Acc:HGNC:30840] |
| ENSG00000186965 | HGLibA_25666 | 52 | -0.664335659 | KRTAP19-2 | keratin associated protein 19-2 [Source:HGNC Symbol;Acc:HGNC:18937] |
| ENSG00000188021 | HGLibA_52735 | 52 | -0.664335659 | UBQLN2 | ubiquilin 2 [Source:HGNC Symbol;Acc:HGNC:12509] |
| ENSG00000188522 | HGLibA_16757 | 52 | -0.664335659 | FAM83G | family with sequence similarity 83 member G [Source:HGNC Symbol;Acc:HGNC:32554] |
| ENSG00000188690 | HGLibA_53157 | 52 | -0.664335659 | UROS | uroporphyrinogen III synthase [Source:HGNC Symbol;Acc:HGNC:12592] |
| ENSG00000189050 | HGLibA_41703 | 52 | -0.664335659 | RNFT1 | transmembrane 1 [Source:HGNC Symbol;Acc:HGNC:30206]" |
| ENSG00000189056 | HGLibA_40823 | 52 | -0.664335659 | RELN | reelin [Source:HGNC Symbol;Acc:HGNC:9957] |
| ENSG00000189280 | HGLibA_19168 | 52 | -0.664335659 | GJB5 | gap junction protein beta 5 [Source:HGNC Symbol;Acc:HGNC:4287] |
| ENSG00000196109 | HGLibA_56515 | 52 | -0.664335659 | ZNF676 | zinc finger protein 676 [Source:HGNC Symbol;Acc:HGNC:20429] |
| ENSG00000196341 | HGLibA_34509 | 52 | -0.664335659 | OR8D1 | olfactory receptor family 8 subfamily D member 1 [Source:HGNC Symbol;Acc:HGNC:8481] |
| ENSG00000197416 | HGLibA_15911 | 52 | -0.664335659 | FABP12 | fatty acid binding protein 12 [Source:HGNC Symbol;Acc:HGNC:34524] |
| ENSG00000197859 | HGLibA_00850 | 52 | -0.664335659 | ADAMTSL2 | ADAMTS like 2 [Source:HGNC Symbol;Acc:HGNC:14631] |
| ENSG00000198034 | HGLibA_42143 | 52 | -0.664335659 | RPS4X | ribosomal protein S4 X-linked [Source:HGNC Symbol;Acc:HGNC:10424] |
| ENSG00000198355 | HGLibA_36682 | 52 | -0.664335659 | PIM3 | serine/threonine kinase [Source:HGNC Symbol;Acc:HGNC:19310]" |
| ENSG00000198586 | HGLibA_49621 | 52 | -0.664335659 | TLK1 | tousled like kinase 1 [Source:HGNC Symbol;Acc:HGNC:11841] |
| ENSG00000198796 | HGLibA_01702 | 52 | -0.664335659 | ALPK2 | alpha kinase 2 [Source:HGNC Symbol;Acc:HGNC:20565] |
| ENSG00000198805 | HGLibA_37401 | 52 | -0.664335659 | PNP | purine nucleoside phosphorylase [Source:HGNC Symbol;Acc:HGNC:7892] |
| ENSG00000198824 | HGLibA_09353 | 52 | -0.664335659 | CHAMP1 | chromosome alignment maintaining phosphoprotein 1 [Source:HGNC Symbol;Acc:HGNC:20311] |
| ENSG00000198924 | HGLibA_12507 | 52 | -0.664335659 | DCLRE1A | DNA cross-link repair 1A [Source:HGNC Symbol;Acc:HGNC:17660] |
| ENSG00000203923 | HGLibA_46403 | 52 | -0.664335659 | SPANXN1 | SPANX family member N1 [Source:HGNC Symbol;Acc:HGNC:33174] |
| ENSG00000204568 | HGLibA_30052 | 52 | -0.664335659 | MRPS18B | mitochondrial ribosomal protein S18B [Source:HGNC Symbol;Acc:HGNC:14516] |
| ENSG00000204872 | HGLibA_31184 | 52 | -0.664335659 | NAT8B | gene/pseudogene) [Source:HGNC Symbol;Acc:HGNC:30235]" |
| ENSG00000204918 | HGLibA_38802 | 52 | -0.664335659 | PRR20B | proline rich 20B [Source:HGNC Symbol;Acc:HGNC:37220] |
| ENSG00000205111 | HGLibA_08815 | 52 | -0.664335659 | CDKL4 | cyclin dependent kinase like 4 [Source:HGNC Symbol;Acc:HGNC:19287] |
| ENSG00000205177 | HGLibA_05210 | 52 | -0.664335659 | C11orf91 | chromosome 11 open reading frame 91 [Source:HGNC Symbol;Acc:HGNC:34444] |
| ENSG00000205693 | HGLibA_28104 | 52 | -0.664335659 | MANSC4 | MANSC domain containing 4 [Source:HGNC Symbol;Acc:HGNC:40023] |
| ENSG00000205809 | HGLibA_25288 | 52 | -0.664335659 | KLRC2 | killer cell lectin like receptor C2 [Source:HGNC Symbol;Acc:HGNC:6375] |
| ENSG00000213648 | HGLibA_47740 | 52 | -0.664335659 | SULT1A4 | sulfotransferase family 1A member 4 [Source:HGNC Symbol;Acc:HGNC:30004] |
| ENSG00000215113 | HGLibA_11936 | 52 | -0.664335659 | CXorf49B | chromosome X open reading frame 49B [Source:HGNC Symbol;Acc:HGNC:34229] |
| ENSG00000215547 | HGLibA_12858 | 52 | -0.664335659 | DEFB115 | defensin beta 115 [Source:HGNC Symbol;Acc:HGNC:18096] |
| ENSG00000217128 | HGLibA_17764 | 52 | -0.664335659 | FNIP1 | folliculin interacting protein 1 [Source:HGNC Symbol;Acc:HGNC:29418] |
| ENSG00000221867 | HGLibA_27911 | 52 | -0.664335659 | MAGEA3 | MAGE family member A3 [Source:HGNC Symbol;Acc:HGNC:6801] |
| ENSG00000221914 | HGLibA_38146 | 52 | -0.664335659 | PPP2R2A | protein phosphatase 2 regulatory subunit Balpha [Source:HGNC Symbol;Acc:HGNC:9304] |
| ENSG00000227057 | HGLibA_54103 | 52 | -0.664335659 | WDR46 | WD repeat domain 46 [Source:HGNC Symbol;Acc:HGNC:13923] |
| ENSG00000227140 | HGLibA_53280 | 52 | -0.664335659 | USP17L5 | ubiquitin specific peptidase 17-like family member 5 [Source:HGNC Symbol;Acc:HGNC:37177] |
| ENSG00000233816 | HGLibA_22662 | 52 | -0.664335659 | IFNA13 | interferon alpha 13 [Source:HGNC Symbol;Acc:HGNC:5419] |
| ENSG00000234511 | HGLibA_06413 | 52 | -0.664335659 | C5orf58 | chromosome 5 open reading frame 58 [Source:HGNC Symbol;Acc:HGNC:37272] |
| ENSG00000237441 | HGLibA_41003 | 52 | -0.664335659 | RGL2 | ral guanine nucleotide dissociation stimulator like 2 [Source:HGNC Symbol;Acc:HGNC:9769] |
| ENSG00000241598 | HGLibA_25798 | 52 | -0.664335659 | KRTAP5-4 | keratin associated protein 5-4 [Source:HGNC Symbol;Acc:HGNC:23599] |
| ENSG00000250067 | HGLibA_54691 | 52 | -0.664335659 | YJEFN3 | YjeF N-terminal domain containing 3 [Source:HGNC Symbol;Acc:HGNC:24785] |
| ENSG00000255071 | HGLibA_42613 | 52 | -0.664335659 | SAA2-SAA4 | SAA2-SAA4 readthrough [Source:HGNC Symbol;Acc:HGNC:39550] |
| ENSG00000258873 | HGLibA_14206 | 52 | -0.664335659 | DUXA | double homeobox A [Source:HGNC Symbol;Acc:HGNC:32179] |
| ENSG00000266028 | HGLibA_46971 | 52 | -0.664335659 | SRGAP2 | SLIT-ROBO Rho GTPase activating protein 2 [Source:HGNC Symbol;Acc:HGNC:19751] |
| ENSG00000267631 | HGLibA_09302 | 52 | -0.664335659 | CGB1 | chorionic gonadotropin subunit beta 1 [Source:HGNC Symbol;Acc:HGNC:16721] |
| ENSG00000268009 | HGLibA_47210 | 52 | -0.664335659 | SSX4 | SSX family member 4 [Source:HGNC Symbol;Acc:HGNC:11338] |
| ENSG00000268964 | HGLibA_15547 | 52 | -0.664335659 | ERVV-2 | envelope [Source:HGNC Symbol;Acc:HGNC:39051]" |
| ENSG00000277632 | HGLibA_08019 | 52 | -0.664335659 | CCL3 | C-C motif chemokine ligand 3 [Source:HGNC Symbol;Acc:HGNC:10627] |
| FAM21A | HGLibA_16503 | 52 | -0.664335659 | FAM21A | - |
| FGFR1OP | HGLibA_17438 | 52 | -0.664335659 | FGFR1OP | - |
| hsa-mir-1182 | HGLibA_57221 | 52 | -0.664335659 | hsa-mir-1182 | - |
| hsa-mir-127 | HGLibA_57512 | 52 | -0.664335659 | hsa-mir-127 | - |
| hsa-mir-141 | HGLibA_57830 | 52 | -0.664335659 | hsa-mir-141 | - |
| hsa-mir-1909 | HGLibA_58030 | 52 | -0.664335659 | hsa-mir-1909 | - |
| hsa-mir-1913 | HGLibA_58057 | 52 | -0.664335659 | hsa-mir-1913 | - |
| hsa-mir-3158-1 | HGLibA_58702 | 52 | -0.664335659 | hsa-mir-3158-1 | - |
| hsa-mir-3656 | HGLibA_59169 | 52 | -0.664335659 | hsa-mir-3656 | - |
| hsa-mir-376b | HGLibA_59415 | 52 | -0.664335659 | hsa-mir-376b | - |
| hsa-mir-4315-2 | HGLibA_59981 | 52 | -0.664335659 | hsa-mir-4315-2 | - |
| hsa-mir-4434 | HGLibA_60126 | 52 | -0.664335659 | hsa-mir-4434 | - |
| hsa-mir-4443 | HGLibA_60175 | 52 | -0.664335659 | hsa-mir-4443 | - |
| hsa-mir-4498 | HGLibA_60410 | 52 | -0.664335659 | hsa-mir-4498 | - |
| hsa-mir-4530 | HGLibA_60580 | 52 | -0.664335659 | hsa-mir-4530 | - |
| hsa-mir-4673 | HGLibA_60808 | 52 | -0.664335659 | hsa-mir-4673 | - |
| hsa-mir-4687 | HGLibA_60859 | 52 | -0.664335659 | hsa-mir-4687 | - |
| hsa-mir-4766 | HGLibA_61162 | 52 | -0.664335659 | hsa-mir-4766 | - |
| hsa-mir-5047 | HGLibA_61477 | 52 | -0.664335659 | hsa-mir-5047 | - |
| hsa-mir-548ag-2 | HGLibA_61878 | 52 | -0.664335659 | hsa-mir-548ag-2 | - |
| hsa-mir-548ay | HGLibA_61942 | 52 | -0.664335659 | hsa-mir-548ay | - |
| hsa-mir-606 | HGLibA_62516 | 52 | -0.664335659 | hsa-mir-606 | - |
| hsa-mir-6132 | HGLibA_62681 | 52 | -0.664335659 | hsa-mir-6132 | - |
| hsa-mir-635 | HGLibA_62780 | 52 | -0.664335659 | hsa-mir-635 | - |
| hsa-mir-6739 | HGLibA_63108 | 52 | -0.664335659 | hsa-mir-6739 | - |
| hsa-mir-6791 | HGLibA_63340 | 52 | -0.664335659 | hsa-mir-6791 | - |
| hsa-mir-6836 | HGLibA_63518 | 52 | -0.664335659 | hsa-mir-6836 | - |
| hsa-mir-6839 | HGLibA_63530 | 52 | -0.664335659 | hsa-mir-6839 | - |
| hsa-mir-7161 | HGLibA_63874 | 52 | -0.664335659 | hsa-mir-7161 | - |
| KIAA1239 | HGLibA_24725 | 52 | -0.664335659 | KIAA1239 | - |
| KIAA1430 | HGLibA_24753 | 52 | -0.664335659 | KIAA1430 | - |

| | | | | | |
|---|---|---|---|---|---|
| KIAA1967 | HGLibA_24833 | 52 | -0.664335659 | KIAA1967 | - |
| LOC100506688 | HGLibA_26865 | 52 | -0.664335659 | LOC100506688 | - |
| MST4 | HGLibA_30262 | 52 | -0.664335659 | MST4 | - |
| SPRY3_X | HGLibA_46841 | 52 | -0.664335659 | SPRY3_X | - |
| T | HGLibA_48121 | 52 | -0.664335659 | T | - |
| 5-Mar | HGLibA_28341 | 51 | -0.673381002 | 5-Mar | - |
| 6-Mar | HGLibA_28344 | 51 | -0.673381002 | 6-Mar | - |
| C10orf2 | HGLibA_05062 | 51 | -0.673381002 | C10orf2 | - |
| C14orf166 | HGLibA_05333 | 51 | -0.673381002 | C14orf166 | - |
| C19orf10 | HGLibA_05626 | 51 | -0.673381002 | C19orf10 | - |
| C1orf63 | HGLibA_05882 | 51 | -0.673381002 | C1orf63 | - |
| C2orf61 | HGLibA_06154 | 51 | -0.673381002 | C2orf61 | - |
| CD99_X | HGLibA_08489 | 51 | -0.673381002 | CD99_X | - |
| ENSG00000007516 | HGLibA_04130 | 51 | -0.673381002 | BAIAP3 | BAI1 associated protein 3 [Source:HGNC Symbol;Acc:HGNC:948] |
| ENSG00000008438 | HGLibA_36248 | 51 | -0.673381002 | PGLYRP1 | peptidoglycan recognition protein 1 [Source:HGNC Symbol;Acc:HGNC:8904] |
| ENSG00000010256 | HGLibA_53114 | 51 | -0.673381002 | UQCRC1 | ubiquinol-cytochrome c reductase core protein 1 [Source:HGNC Symbol;Acc:HGNC:12585] |
| ENSG00000012124 | HGLibA_08297 | 51 | -0.673381002 | CD22 | CD22 molecule [Source:HGNC Symbol;Acc:HGNC:1643] |
| ENSG00000015568 | HGLibA_41038 | 51 | -0.673381002 | RGPD5 | RANBP2-like and GRIP domain containing 5 [Source:HGNC Symbol;Acc:HGNC:32418] |
| ENSG00000017260 | HGLibA_03625 | 51 | -0.673381002 | ATP2C1 | ATPase secretory pathway Ca2+ transporting 1 [Source:HGNC Symbol;Acc:HGNC:13211] |
| ENSG00000026950 | HGLibA_04968 | 51 | -0.673381002 | BTN3A1 | butyrophilin subfamily 3 member A1 [Source:HGNC Symbol;Acc:HGNC:1138] |
| ENSG00000033170 | HGLibA_18205 | 51 | -0.673381002 | FUT8 | fucosyltransferase 8 [Source:HGNC Symbol;Acc:HGNC:4019] |
| ENSG00000041353 | HGLibA_39860 | 51 | -0.673381002 | RAB27B | member RAS oncogene family [Source:HGNC Symbol;Acc:HGNC:9767]" |
| ENSG00000042429 | HGLibA_28762 | 51 | -0.673381002 | MED17 | mediator complex subunit 17 [Source:HGNC Symbol;Acc:HGNC:2375] |
| ENSG00000060971 | HGLibA_00342 | 51 | -0.673381002 | ACAA1 | acetyl-CoA acyltransferase 1 [Source:HGNC Symbol;Acc:HGNC:82] |
| ENSG00000064787 | HGLibA_04252 | 51 | -0.673381002 | BCAS1 | breast carcinoma amplified sequence 1 [Source:HGNC Symbol;Acc:HGNC:974] |
| ENSG00000065548 | HGLibA_54968 | 51 | -0.673381002 | ZC3H15 | zinc finger CCCH-type containing 15 [Source:HGNC Symbol;Acc:HGNC:29528] |
| ENSG00000066382 | HGLibA_29775 | 51 | -0.673381002 | MPPED2 | metallophosphoesterase domain containing 2 [Source:HGNC Symbol;Acc:HGNC:1180] |
| ENSG00000066382 | HGLibA_29774 | 51 | -0.673381002 | MPPED2 | metallophosphoesterase domain containing 2 [Source:HGNC Symbol;Acc:HGNC:1180] |
| ENSG00000067606 | HGLibA_38564 | 51 | -0.673381002 | PRKCZ | protein kinase C zeta [Source:HGNC Symbol;Acc:HGNC:9412] |
| ENSG00000071073 | HGLibA_29140 | 51 | -0.673381002 | MGAT4A | 3-mannosyl-glycoprotein 4-beta-N-acetylglucosaminyltransferase A [Source:HGNC Symbol;Acc:HGNC:7047]" |
| ENSG00000075790 | HGLibA_04238 | 51 | -0.673381002 | BCAP29 | B cell receptor associated protein 29 [Source:HGNC Symbol;Acc:HGNC:24131] |
| ENSG00000076554 | HGLibA_51111 | 51 | -0.673381002 | TPD52 | tumor protein D52 [Source:HGNC Symbol;Acc:HGNC:12005] |
| ENSG00000077721 | HGLibA_52572 | 51 | -0.673381002 | UBE2A | ubiquitin conjugating enzyme E2 A [Source:HGNC Symbol;Acc:HGNC:12472] |
| ENSG00000079616 | HGLibA_24904 | 51 | -0.673381002 | KIF22 | kinesin family member 22 [Source:HGNC Symbol;Acc:HGNC:6391] |
| ENSG00000086200 | HGLibA_23460 | 51 | -0.673381002 | IPO11 | importin 11 [Source:HGNC Symbol;Acc:HGNC:20628] |
| ENSG00000088543 | HGLibA_06215 | 51 | -0.673381002 | C3orf18 | chromosome 3 open reading frame 18 [Source:HGNC Symbol;Acc:HGNC:24837] |
| ENSG00000090339 | HGLibA_22494 | 51 | -0.673381002 | ICAM1 | intercellular adhesion molecule 1 [Source:HGNC Symbol;Acc:HGNC:5344] |
| ENSG00000094841 | HGLibA_53093 | 51 | -0.673381002 | UPRT | uracil phosphoribosyltransferase homolog [Source:HGNC Symbol;Acc:HGNC:28334] |
| ENSG00000099326 | HGLibA_30938 | 51 | -0.673381002 | MZF1 | myeloid zinc finger 1 [Source:HGNC Symbol;Acc:HGNC:13108] |
| ENSG00000100030 | HGLibA_28247 | 51 | -0.673381002 | MAPK1 | mitogen-activated protein kinase 1 [Source:HGNC Symbol;Acc:HGNC:6871] |
| ENSG00000100031 | HGLibA_19010 | 51 | -0.673381002 | GGT1 | gamma-glutamyltransferase 1 [Source:HGNC Symbol;Acc:HGNC:4250] |
| ENSG00000100319 | HGLibA_55379 | 51 | -0.673381002 | ZMAT5 | zinc finger matrin-type 5 [Source:HGNC Symbol;Acc:HGNC:28046] |
| ENSG00000101160 | HGLibA_11737 | 51 | -0.673381002 | CTSZ | cathepsin Z [Source:HGNC Symbol;Acc:HGNC:2547] |
| ENSG00000101856 | HGLibA_36288 | 51 | -0.673381002 | PGRMC1 | progesterone receptor membrane component 1 [Source:HGNC Symbol;Acc:HGNC:16090] |
| ENSG00000104941 | HGLibA_42337 | 51 | -0.673381002 | RSPH6A | radial spoke head 6 homolog A [Source:HGNC Symbol;Acc:HGNC:14241] |
| ENSG00000104983 | HGLibA_07817 | 51 | -0.673381002 | CCDC61 | coiled-coil domain containing 61 [Source:HGNC Symbol;Acc:HGNC:33629] |
| ENSG00000106688 | HGLibA_44506 | 51 | -0.673381002 | SLC1A1 | solute carrier family 1 member 1 [Source:HGNC Symbol;Acc:HGNC:10939] |
| ENSG00000107223 | HGLibA_14463 | 51 | -0.673381002 | EDF1 | endothelial differentiation related factor 1 [Source:HGNC Symbol;Acc:HGNC:3164] |
| ENSG00000107242 | HGLibA_36718 | 51 | -0.673381002 | PIP5K1B | phosphatidylinositol-4-phosphate 5-kinase type 1 beta [Source:HGNC Symbol;Acc:HGNC:8995] |
| ENSG00000108654 | HGLibA_12729 | 51 | -0.673381002 | DDX5 | DEAD-box helicase 5 [Source:HGNC Symbol;Acc:HGNC:2746] |
| ENSG00000108733 | HGLibA_36093 | 51 | -0.673381002 | PEX12 | peroxisomal biogenesis factor 12 [Source:HGNC Symbol;Acc:HGNC:8854] |
| ENSG00000108852 | HGLibA_29750 | 51 | -0.673381002 | MPP2 | membrane palmitoylated protein 2 [Source:HGNC Symbol;Acc:HGNC:7220] |
| ENSG00000109471 | HGLibA_23071 | 51 | -0.673381002 | IL2 | interleukin 2 [Source:HGNC Symbol;Acc:HGNC:6001] |
| ENSG00000110934 | HGLibA_04510 | 51 | -0.673381002 | BIN2 | bridging integrator 2 [Source:HGNC Symbol;Acc:HGNC:1053] |
| ENSG00000111262 | HGLibA_24124 | 51 | -0.673381002 | KCNA1 | potassium voltage-gated channel subfamily A member 1 [Source:HGNC Symbol;Acc:HGNC:6218] |
| ENSG00000111845 | HGLibA_35041 | 51 | -0.673381002 | PAK1IP1 | PAK1 interacting protein 1 [Source:HGNC Symbol;Acc:HGNC:20882] |
| ENSG00000113946 | HGLibA_09864 | 51 | -0.673381002 | CLDN16 | claudin 16 [Source:HGNC Symbol;Acc:HGNC:2037] |
| ENSG00000115598 | HGLibA_23064 | 51 | -0.673381002 | IL1RL2 | interleukin 1 receptor like 2 [Source:HGNC Symbol;Acc:HGNC:5999] |
| ENSG00000115875 | HGLibA_47086 | 51 | -0.673381002 | SRSF7 | serine and arginine rich splicing factor 7 [Source:HGNC Symbol;Acc:HGNC:10789] |
| ENSG00000115904 | HGLibA_46202 | 51 | -0.673381002 | SOS1 | SOS Ras/Rac guanine nucleotide exchange factor 1 [Source:HGNC Symbol;Acc:HGNC:11187] |
| ENSG00000117305 | HGLibA_21702 | 51 | -0.673381002 | HMGCL | 3-hydroxy-3-methylglutaryl-CoA lyase [Source:HGNC Symbol;Acc:HGNC:5005] |
| ENSG00000119866 | HGLibA_04290 | 51 | -0.673381002 | BCL11A | B cell CLL/lymphoma 11A [Source:HGNC Symbol;Acc:HGNC:13221] |
| ENSG00000119938 | HGLibA_38104 | 51 | -0.673381002 | PPP1R3C | protein phosphatase 1 regulatory subunit 3C [Source:HGNC Symbol;Acc:HGNC:9293] |
| ENSG00000120451 | HGLibA_46054 | 51 | -0.673381002 | SNX19 | sorting nexin 19 [Source:HGNC Symbol;Acc:HGNC:21532] |
| ENSG00000124067 | HGLibA_44365 | 51 | -0.673381002 | SLC12A4 | solute carrier family 12 member 4 [Source:HGNC Symbol;Acc:HGNC:10913] |
| ENSG00000124181 | HGLibA_37001 | 51 | -0.673381002 | PLCG1 | phospholipase C gamma 1 [Source:HGNC Symbol;Acc:HGNC:9065] |
| ENSG00000124701 | HGLibA_02471 | 51 | -0.673381002 | APOBEC2 | apolipoprotein B mRNA editing enzyme catalytic subunit 2 [Source:HGNC Symbol;Acc:HGNC:605] |
| ENSG00000125746 | HGLibA_15060 | 51 | -0.673381002 | EML2 | echinoderm microtubule associated protein like 2 [Source:HGNC Symbol;Acc:HGNC:18035] |
| ENSG00000125835 | HGLibA_45969 | 51 | -0.673381002 | SNRPB | small nuclear ribonucleoprotein polypeptides B and B1 [Source:HGNC Symbol;Acc:HGNC:11153] |
| ENSG00000125910 | HGLibA_42601 | 51 | -0.673381002 | S1PR4 | sphingosine-1-phosphate receptor 4 [Source:HGNC Symbol;Acc:HGNC:3170] |
| ENSG00000127124 | HGLibA_21572 | 51 | -0.673381002 | HIVEP3 | human immunodeficiency virus type I enhancer binding protein 3 [Source:HGNC Symbol;Acc:HGNC:13561] |
| ENSG00000127337 | HGLibA_54659 | 51 | -0.673381002 | YEATS4 | YEATS domain containing 4 [Source:HGNC Symbol;Acc:HGNC:24859] |
| ENSG00000129071 | HGLibA_28488 | 51 | -0.673381002 | MBD4 | DNA glycosylase [Source:HGNC Symbol;Acc:HGNC:6919]" |
| ENSG00000129250 | HGLibA_24889 | 51 | -0.673381002 | KIF1C | kinesin family member 1C [Source:HGNC Symbol;Acc:HGNC:6317] |
| ENSG00000130045 | HGLibA_33147 | 51 | -0.673381002 | NXNL2 | nucleoredoxin like 2 [Source:HGNC Symbol;Acc:HGNC:30482] |
| ENSG00000130748 | HGLibA_50020 | 51 | -0.673381002 | TMEM160 | transmembrane protein 160 [Source:HGNC Symbol;Acc:HGNC:26042] |
| ENSG00000130844 | HGLibA_55817 | 51 | -0.673381002 | ZNF331 | zinc finger protein 331 [Source:HGNC Symbol;Acc:HGNC:15489] |
| ENSG00000131019 | HGLibA_52965 | 51 | -0.673381002 | ULBP3 | UL16 binding protein 3 [Source:HGNC Symbol;Acc:HGNC:14895] |
| ENSG00000131459 | HGLibA_18962 | 51 | -0.673381002 | GFPT2 | glutamine-fructose-6-phosphate transaminase 2 [Source:HGNC Symbol;Acc:HGNC:4242] |
| ENSG00000131931 | HGLibA_49287 | 51 | -0.673381002 | THAP1 | THAP domain containing 1 [Source:HGNC Symbol;Acc:HGNC:20856] |
| ENSG00000132467 | HGLibA_53452 | 51 | -0.673381002 | UTP3 | small subunit processome component [Source:HGNC Symbol;Acc:HGNC:24477]" |
| ENSG00000132612 | HGLibA_53797 | 51 | -0.673381002 | VPS4A | vacuolar protein sorting 4 homolog A [Source:HGNC Symbol;Acc:HGNC:13488] |
| ENSG00000132669 | HGLibA_41291 | 51 | -0.673381002 | RIN2 | Ras and Rab interactor 2 [Source:HGNC Symbol;Acc:HGNC:18750] |
| ENSG00000133703 | HGLibA_25383 | 51 | -0.673381002 | KRAS | GTPase [Source:HGNC Symbol;Acc:HGNC:6407]" |
| ENSG00000133863 | HGLibA_49068 | 51 | -0.673381002 | TEX15 | meiosis and synapsis associated [Source:HGNC Symbol;Acc:HGNC:11738]" |
| ENSG00000134153 | HGLibA_15020 | 51 | -0.673381002 | EMC7 | ER membrane protein complex subunit 7 [Source:HGNC Symbol;Acc:HGNC:24301] |
| ENSG00000134215 | HGLibA_53554 | 51 | -0.673381002 | VAV3 | vav guanine nucleotide exchange factor 3 [Source:HGNC Symbol;Acc:HGNC:12659] |
| ENSG00000134326 | HGLibA_10203 | 51 | -0.673381002 | CMPK2 | cytidine/uridine monophosphate kinase 2 [Source:HGNC Symbol;Acc:HGNC:27015] |
| ENSG00000134698 | HGLibA_01214 | 51 | -0.673381002 | AGO4 | RISC catalytic component [Source:HGNC Symbol;Acc:HGNC:18424]" |
| ENSG00000135220 | HGLibA_52909 | 51 | -0.673381002 | UGT2A3 | UDP glucuronosyltransferase family 2 member A3 [Source:HGNC Symbol;Acc:HGNC:28528] |
| ENSG00000135447 | HGLibA_38064 | 51 | -0.673381002 | PPP1R1A | protein phosphatase 1 regulatory inhibitor subunit 1A [Source:HGNC Symbol;Acc:HGNC:9286] |
| ENSG00000136152 | HGLibA_10477 | 51 | -0.673381002 | COG3 | component of oligomeric golgi complex 3 [Source:HGNC Symbol;Acc:HGNC:18619] |
| ENSG00000136938 | HGLibA_02200 | 51 | -0.673381002 | ANP32B | acidic nuclear phosphoprotein 32 family member B [Source:HGNC Symbol;Acc:HGNC:16677] |
| ENSG00000137992 | HGLibA_12405 | 51 | -0.673381002 | DBT | dihydrolipoamide branched chain transacylase E2 [Source:HGNC Symbol;Acc:HGNC:2698] |
| ENSG00000138375 | HGLibA_45618 | 51 | -0.673381002 | SMARCAL1 | subfamily a like 1 [Source:HGNC Symbol;Acc:HGNC:11102]" |
| ENSG00000139540 | HGLibA_45114 | 51 | -0.673381002 | SLC39A5 | solute carrier family 39 member 5 [Source:HGNC Symbol;Acc:HGNC:20502] |
| ENSG00000139977 | HGLibA_30988 | 51 | -0.673381002 | NAA30 | NatC catalytic subunit [Source:HGNC Symbol;Acc:HGNC:19844]" |
| ENSG00000140153 | HGLibA_54045 | 51 | -0.673381002 | WDR20 | WD repeat domain 20 [Source:HGNC Symbol;Acc:HGNC:19667] |
| ENSG00000141030 | HGLibA_10717 | 51 | -0.673381002 | COPS3 | COP9 signalosome subunit 3 [Source:HGNC Symbol;Acc:HGNC:2239] |
| ENSG00000144134 | HGLibA_40027 | 51 | -0.673381002 | RABL2A | member of RAS oncogene family like 2A [Source:HGNC Symbol;Acc:HGNC:9799]" |
| ENSG00000144191 | HGLibA_10269 | 51 | -0.673381002 | CNGA3 | cyclic nucleotide gated channel alpha 3 [Source:HGNC Symbol;Acc:HGNC:2150] |
| ENSG00000144524 | HGLibA_10732 | 51 | -0.673381002 | COPS7B | COP9 signalosome subunit 7B [Source:HGNC Symbol;Acc:HGNC:16760] |
| ENSG00000144736 | HGLibA_44084 | 51 | -0.673381002 | SHQ1 | H/ACA ribonucleoprotein assembly factor [Source:HGNC Symbol;Acc:HGNC:25543]" |
| ENSG00000145191 | HGLibA_14747 | 51 | -0.673381002 | EIF2B5 | eukaryotic translation initiation factor 2B subunit epsilon [Source:HGNC Symbol;Acc:HGNC:3261] |
| ENSG00000145721 | HGLibA_26642 | 51 | -0.673381002 | LIX1 | limb and CNS expressed 1 [Source:HGNC Symbol;Acc:HGNC:18581] |
| ENSG00000145919 | HGLibA_04667 | 51 | -0.673381002 | BOD1 | biorientation of chromosomes in cell division 1 [Source:HGNC Symbol;Acc:HGNC:25114] |
| ENSG00000146618 | HGLibA_17292 | 51 | -0.673381002 | FERD3L | Fer3 like bHLH transcription factor [Source:HGNC Symbol;Acc:HGNC:16660] |
| ENSG00000146856 | HGLibA_01171 | 51 | -0.673381002 | AGBL3 | ATP/GTP binding protein like 3 [Source:HGNC Symbol;Acc:HGNC:27981] |
| ENSG00000147588 | HGLibA_37322 | 51 | -0.673381002 | PMP2 | peripheral myelin protein 2 [Source:HGNC Symbol;Acc:HGNC:9117] |
| ENSG00000148308 | HGLibA_20593 | 51 | -0.673381002 | GTF3C5 | general transcription factor IIIC subunit 5 [Source:HGNC Symbol;Acc:HGNC:4668] |
| ENSG00000148719 | HGLibA_13529 | 51 | -0.673381002 | DNAJB12 | DnaJ heat shock protein family (Hsp40) member B12 [Source:HGNC Symbol;Acc:HGNC:14891] |
| ENSG00000148965 | HGLibA_42617 | 51 | -0.673381002 | SAA4 | constitutive [Source:HGNC Symbol;Acc:HGNC:10516]" |
| ENSG00000149577 | HGLibA_44117 | 51 | -0.673381002 | SIDT2 | SID1 transmembrane family member 2 [Source:HGNC Symbol;Acc:HGNC:24272] |
| ENSG00000149781 | HGLibA_17301 | 51 | -0.673381002 | FERMT3 | fermitin family member 3 [Source:HGNC Symbol;Acc:HGNC:23151] |
| ENSG00000149970 | HGLibA_10298 | 51 | -0.673381002 | CNKSR2 | connector enhancer of kinase suppressor of Ras 2 [Source:HGNC Symbol;Acc:HGNC:19701] |
| ENSG00000150540 | HGLibA_21785 | 51 | -0.673381002 | HNMT | histamine N-methyltransferase [Source:HGNC Symbol;Acc:HGNC:5028] |
| ENSG00000150656 | HGLibA_10250 | 51 | -0.673381002 | CNDP1 | carnosine dipeptidase 1 [Source:HGNC Symbol;Acc:HGNC:20675] |
| ENSG00000151617 | HGLibA_14479 | 51 | -0.673381002 | EDNRA | endothelin receptor type A [Source:HGNC Symbol;Acc:HGNC:3179] |
| ENSG00000151834 | HGLibA_18358 | 51 | -0.673381002 | GABRA2 | gamma-aminobutyric acid type A receptor alpha2 subunit [Source:HGNC Symbol;Acc:HGNC:4076] |
| ENSG00000152061 | HGLibA_40013 | 51 | -0.673381002 | RABGAP1L | RAB GTPase activating protein 1 like [Source:HGNC Symbol;Acc:HGNC:24663] |
| ENSG00000152409 | HGLibA_23971 | 51 | -0.673381002 | JMY | p53 cofactor [Source:HGNC Symbol;Acc:HGNC:28916]" |
| ENSG00000155846 | HGLibA_37849 | 51 | -0.673381002 | PPARGC1B | PPARG coactivator 1 beta [Source:HGNC Symbol;Acc:HGNC:30022] |
| ENSG00000158161 | HGLibA_15822 | 51 | -0.673381002 | EYA3 | EYA transcriptional coactivator and phosphatase 3 [Source:HGNC Symbol;Acc:HGNC:3521] |
| ENSG00000158417 | HGLibA_14865 | 51 | -0.673381002 | EIF5B | eukaryotic translation initiation factor 5B [Source:HGNC Symbol;Acc:HGNC:30793] |
| ENSG00000160051 | HGLibA_23494 | 51 | -0.673381002 | IQCC | IQ motif containing C [Source:HGNC Symbol;Acc:HGNC:25545] |
| ENSG00000160282 | HGLibA_18112 | 51 | -0.673381002 | FTCD | formimidoyltransferase cyclodeaminase [Source:HGNC Symbol;Acc:HGNC:3974] |
| ENSG00000160352 | HGLibA_56620 | 51 | -0.673381002 | ZNF714 | zinc finger protein 714 [Source:HGNC Symbol;Acc:HGNC:27124] |
| ENSG00000160703 | HGLibA_32139 | 51 | -0.673381002 | NLRX1 | NLR family member X1 [Source:HGNC Symbol;Acc:HGNC:29890] |
| ENSG00000160868 | HGLibA_12153 | 51 | -0.673381002 | CYP3A4 | cytochrome P450 family 3 subfamily A member 4 [Source:HGNC Symbol;Acc:HGNC:2637] |
| ENSG00000161013 | HGLibA_29144 | 51 | -0.673381002 | MGAT4B | 3-mannosyl-glycoprotein 4-beta-N-acetylglucosaminyltransferase B [Source:HGNC Symbol;Acc:HGNC:7048]" |
| ENSG00000161405 | HGLibA_22934 | 51 | -0.673381002 | IKZF3 | IKAROS family zinc finger 3 [Source:HGNC Symbol;Acc:HGNC:13178] |
| ENSG00000162267 | HGLibA_23810 | 51 | -0.673381002 | ITIH3 | inter-alpha-trypsin inhibitor heavy chain 3 [Source:HGNC Symbol;Acc:HGNC:6168] |
| ENSG00000162433 | HGLibA_01385 | 51 | -0.673381002 | AK4 | adenylate kinase 4 [Source:HGNC Symbol;Acc:HGNC:363] |
| ENSG00000162494 | HGLibA_27338 | 51 | -0.673381002 | LRRC38 | leucine rich repeat containing 38 [Source:HGNC Symbol;Acc:HGNC:27005] |
| ENSG00000163060 | HGLibA_48980 | 51 | -0.673381002 | TEKT4 | tektin 4 [Source:HGNC Symbol;Acc:HGNC:31012] |

| | | | | | |
|---|---|---|---|---|---|
| ENSG00000163428 | HGLibA_27399 | 51 | -0.673381002 | LRRC58 | leucine rich repeat containing 58 [Source:HGNC Symbol;Acc:HGNC:26968] |
| ENSG00000163554 | HGLibA_46870 | 51 | -0.673381002 | SPTA1 | erythrocytic 1 [Source:HGNC Symbol;Acc:HGNC:11272]" |
| ENSG00000163788 | HGLibA_45939 | 51 | -0.673381002 | SNRK | SNF related kinase [Source:HGNC Symbol;Acc:HGNC:30598] |
| ENSG00000164070 | HGLibA_22287 | 51 | -0.673381002 | HSPA4L | heat shock protein family A (Hsp70) member 4 like [Source:HGNC Symbol;Acc:HGNC:17041] |
| ENSG00000165209 | HGLibA_47582 | 51 | -0.673381002 | STRBP | spermatid perinuclear RNA binding protein [Source:HGNC Symbol;Acc:HGNC:16462] |
| ENSG00000165475 | HGLibA_11289 | 51 | -0.673381002 | CRYL1 | crystallin lambda 1 [Source:HGNC Symbol;Acc:HGNC:18246] |
| ENSG00000165606 | HGLibA_13979 | 51 | -0.673381002 | DRGX | dorsal root ganglia homeobox [Source:HGNC Symbol;Acc:HGNC:21536] |
| ENSG00000165914 | HGLibA_52154 | 51 | -0.673381002 | TTC7B | tetratricopeptide repeat domain 7B [Source:HGNC Symbol;Acc:HGNC:19858] |
| ENSG00000166106 | HGLibA_00805 | 51 | -0.673381002 | ADAMTS15 | ADAM metallopeptidase with thrombospondin type 1 motif 15 [Source:HGNC Symbol;Acc:HGNC:16305] |
| ENSG00000166508 | HGLibA_28622 | 51 | -0.673381002 | MCM7 | minichromosome maintenance complex component 7 [Source:HGNC Symbol;Acc:HGNC:6950] |
| ENSG00000166603 | HGLibA_28551 | 51 | -0.673381002 | MC4R | melanocortin 4 receptor [Source:HGNC Symbol;Acc:HGNC:6932] |
| ENSG00000167195 | HGLibA_19614 | 51 | -0.673381002 | GOLGA6C | golgin A6 family member C [Source:HGNC Symbol;Acc:HGNC:32206] |
| ENSG00000167617 | HGLibA_08578 | 51 | -0.673381002 | CDC42EP5 | CDC42 effector protein 5 [Source:HGNC Symbol;Acc:HGNC:17408] |
| ENSG00000167925 | HGLibA_19035 | 51 | -0.673381002 | GHDC | GH3 domain containing [Source:HGNC Symbol;Acc:HGNC:24438] |
| ENSG00000167984 | HGLibA_32088 | 51 | -0.673381002 | NLRC3 | NLR family CARD domain containing 3 [Source:HGNC Symbol;Acc:HGNC:29889] |
| ENSG00000168101 | HGLibA_32955 | 51 | -0.673381002 | NUDT16L1 | nudix hydrolase 16 like 1 [Source:HGNC Symbol;Acc:HGNC:28154] |
| ENSG00000168143 | HGLibA_16744 | 51 | -0.673381002 | FAM83B | family with sequence similarity 83 member B [Source:HGNC Symbol;Acc:HGNC:21357] |
| ENSG00000168724 | HGLibA_13598 | 51 | -0.673381002 | DNAJC21 | DnaJ heat shock protein family (Hsp40) member C21 [Source:HGNC Symbol;Acc:HGNC:27030] |
| ENSG00000168907 | HGLibA_36916 | 51 | -0.673381002 | PLA2G4F | phospholipase A2 group IVF [Source:HGNC Symbol;Acc:HGNC:27396] |
| ENSG00000168961 | HGLibA_26380 | 51 | -0.673381002 | LGALS9 | galectin 9 [Source:HGNC Symbol;Acc:HGNC:6570] |
| ENSG00000169906 | HGLibA_42581 | 51 | -0.673381002 | S100G | S100 calcium binding protein G [Source:HGNC Symbol;Acc:HGNC:1436] |
| ENSG00000169953 | HGLibA_22244 | 51 | -0.673381002 | HSFY2 | heat shock transcription factor Y-linked 2 [Source:HGNC Symbol;Acc:HGNC:23950] |
| ENSG00000171916 | HGLibA_26387 | 51 | -0.673381002 | LGALS9C | galectin 9C [Source:HGNC Symbol;Acc:HGNC:33874] |
| ENSG00000172375 | HGLibA_06089 | 51 | -0.673381002 | C2CD2L | C2CD2 like [Source:HGNC Symbol;Acc:HGNC:29000] |
| ENSG00000172772 | HGLibA_33554 | 51 | -0.673381002 | OR10W1 | olfactory receptor family 10 subfamily W member 1 [Source:HGNC Symbol;Acc:HGNC:15139] |
| ENSG00000173431 | HGLibA_41418 | 51 | -0.673381002 | RNASE8 | ribonuclease A family member 8 [Source:HGNC Symbol;Acc:HGNC:19277] |
| ENSG00000173805 | HGLibA_20839 | 51 | -0.673381002 | HAP1 | huntingtin associated protein 1 [Source:HGNC Symbol;Acc:HGNC:4812] |
| ENSG00000175387 | HGLibA_45573 | 51 | -0.673381002 | SMAD2 | SMAD family member 2 [Source:HGNC Symbol;Acc:HGNC:6768] |
| ENSG00000175395 | HGLibA_55662 | 51 | -0.673381002 | ZNF25 | zinc finger protein 25 [Source:HGNC Symbol;Acc:HGNC:13043] |
| ENSG00000176055 | HGLibA_28504 | 51 | -0.673381002 | MBLAC2 | metallo-beta-lactamase domain containing 2 [Source:HGNC Symbol;Acc:HGNC:33711] |
| ENSG00000176900 | HGLibA_34133 | 51 | -0.673381002 | OR51T1 | olfactory receptor family 51 subfamily T member 1 [Source:HGNC Symbol;Acc:HGNC:15205] |
| ENSG00000177143 | HGLibA_09234 | 51 | -0.673381002 | CETN1 | centrin 1 [Source:HGNC Symbol;Acc:HGNC:1866] |
| ENSG00000177954 | HGLibA_42121 | 51 | -0.673381002 | RPS27 | ribosomal protein S27 [Source:HGNC Symbol;Acc:HGNC:10416] |
| ENSG00000178093 | HGLibA_52020 | 51 | -0.673381002 | TSSK6 | testis specific serine kinase 6 [Source:HGNC Symbol;Acc:HGNC:30410] |
| ENSG00000178287 | HGLibA_46346 | 51 | -0.673381002 | SPAG11A | sperm associated antigen 11A [Source:HGNC Symbol;Acc:HGNC:33342] |
| ENSG00000178287 | HGLibA_46345 | 51 | -0.673381002 | SPAG11A | sperm associated antigen 11A [Source:HGNC Symbol;Acc:HGNC:33342] |
| ENSG00000179626 | HGLibA_34392 | 51 | -0.673381002 | OR6C4 | olfactory receptor family 6 subfamily C member 4 [Source:HGNC Symbol;Acc:HGNC:19632] |
| ENSG00000180257 | HGLibA_56811 | 51 | -0.673381002 | ZNF816 | zinc finger protein 816 [Source:HGNC Symbol;Acc:HGNC:26995] |
| ENSG00000180509 | HGLibA_24182 | 51 | -0.673381002 | KCNE1 | potassium voltage-gated channel subfamily E regulatory subunit 1 [Source:HGNC Symbol;Acc:HGNC:6240] |
| ENSG00000180613 | HGLibA_20510 | 51 | -0.673381002 | GSX2 | GS homeobox 2 [Source:HGNC Symbol;Acc:HGNC:24959] |
| ENSG00000180879 | HGLibA_47163 | 51 | -0.673381002 | SSR4 | signal sequence receptor subunit 4 [Source:HGNC Symbol;Acc:HGNC:11326] |
| ENSG00000182054 | HGLibA_22541 | 51 | -0.673381002 | IDH2 | mitochondrial [Source:HGNC Symbol;Acc:HGNC:5383]" |
| ENSG00000182512 | HGLibA_19323 | 51 | -0.673381002 | GLRX5 | glutaredoxin 5 [Source:HGNC Symbol;Acc:HGNC:20134] |
| ENSG00000182583 | HGLibA_53581 | 51 | -0.673381002 | VCX | variable charge X-linked [Source:HGNC Symbol;Acc:HGNC:12667] |
| ENSG00000183090 | HGLibA_17989 | 51 | -0.673381002 | FREM3 | FRAS1 related extracellular matrix 3 [Source:HGNC Symbol;Acc:HGNC:25172] |
| ENSG00000183527 | HGLibA_39205 | 51 | -0.673381002 | PSMG1 | proteasome assembly chaperone 1 [Source:HGNC Symbol;Acc:HGNC:3043] |
| ENSG00000183773 | HGLibA_01343 | 51 | -0.673381002 | AIFM3 | mitochondria associated 3 [Source:HGNC Symbol;Acc:HGNC:26398]" |
| ENSG00000184937 | HGLibA_54427 | 51 | -0.673381002 | WT1 | Wilms tumor 1 [Source:HGNC Symbol;Acc:HGNC:12796] |
| ENSG00000184995 | HGLibA_22705 | 51 | -0.673381002 | IFNE | interferon epsilon [Source:HGNC Symbol;Acc:HGNC:18163] |
| ENSG00000185467 | HGLibA_25372 | 51 | -0.673381002 | KPNA7 | karyopherin subunit alpha 7 [Source:HGNC Symbol;Acc:HGNC:21839] |
| ENSG00000185988 | HGLibA_37201 | 51 | -0.673381002 | PLK5 | polo like kinase 5 [Source:HGNC Symbol;Acc:HGNC:27001] |
| ENSG00000186965 | HGLibA_25667 | 51 | -0.673381002 | KRTAP19-2 | keratin associated protein 19-2 [Source:HGNC Symbol;Acc:HGNC:18937] |
| ENSG00000187987 | HGLibA_57008 | 51 | -0.673381002 | ZSCAN23 | zinc finger and SCAN domain containing 23 [Source:HGNC Symbol;Acc:HGNC:21193] |
| ENSG00000188064 | HGLibA_54386 | 51 | -0.673381002 | WNT7B | Wnt family member 7B [Source:HGNC Symbol;Acc:HGNC:12787] |
| ENSG00000188338 | HGLibA_45068 | 51 | -0.673381002 | SLC38A3 | solute carrier family 38 member 3 [Source:HGNC Symbol;Acc:HGNC:18044] |
| ENSG00000189227 | HGLibA_05428 | 51 | -0.673381002 | C15orf61 | chromosome 15 open reading frame 61 [Source:HGNC Symbol;Acc:HGNC:34453] |
| ENSG00000189401 | HGLibA_34771 | 51 | -0.673381002 | OTUD6A | OTU deubiquitinase 6A [Source:HGNC Symbol;Acc:HGNC:32312] |
| ENSG00000196323 | HGLibA_54891 | 51 | -0.673381002 | ZBTB44 | zinc finger and BTB domain containing 44 [Source:HGNC Symbol;Acc:HGNC:25001] |
| ENSG00000196329 | HGLibA_19083 | 51 | -0.673381002 | GIMAP5 | IMAP family member 5 [Source:HGNC Symbol;Acc:HGNC:18005]" |
| ENSG00000196569 | HGLibA_25944 | 51 | -0.673381002 | LAMA2 | laminin subunit alpha 2 [Source:HGNC Symbol;Acc:HGNC:6482] |
| ENSG00000198121 | HGLibA_27054 | 51 | -0.673381002 | LPAR1 | lysophosphatidic acid receptor 1 [Source:HGNC Symbol;Acc:HGNC:3166] |
| ENSG00000198873 | HGLibA_20332 | 51 | -0.673381002 | GRK5 | G protein-coupled receptor kinase 5 [Source:HGNC Symbol;Acc:HGNC:4544] |
| ENSG00000198944 | HGLibA_46212 | 51 | -0.673381002 | SOWAHA | sosondowah ankyrin repeat domain family member A [Source:HGNC Symbol;Acc:HGNC:27033] |
| ENSG00000204291 | HGLibA_10515 | 51 | -0.673381002 | COL15A1 | collagen type XV alpha 1 chain [Source:HGNC Symbol;Acc:HGNC:2192] |
| ENSG00000204304 | HGLibA_35345 | 51 | -0.673381002 | PBX2 | PBX homeobox 2 [Source:HGNC Symbol;Acc:HGNC:8633] |
| ENSG00000204345 | HGLibA_08338 | 51 | -0.673381002 | CD300LD | CD300 molecule like family member d [Source:HGNC Symbol;Acc:HGNC:16848] |
| ENSG00000204347 | HGLibA_04904 | 51 | -0.673381002 | BTBD17 | BTB domain containing 17 [Source:HGNC Symbol;Acc:HGNC:33758] |
| ENSG00000204644 | HGLibA_55222 | 51 | -0.673381002 | ZFP57 | ZFP57 zinc finger protein [Source:HGNC Symbol;Acc:HGNC:18791] |
| ENSG00000205126 | HGLibA_00416 | 51 | -0.673381002 | ACCSL | 1-aminocyclopropane-1-carboxylate synthase homolog (inactive) like [Source:HGNC Symbol;Acc:HGNC:34391] |
| ENSG00000205129 | HGLibA_06339 | 51 | -0.673381002 | C4orf47 | chromosome 4 open reading frame 47 [Source:HGNC Symbol;Acc:HGNC:34346] |
| ENSG00000205517 | HGLibA_41006 | 51 | -0.673381002 | RGL3 | ral guanine nucleotide dissociation stimulator like 3 [Source:HGNC Symbol;Acc:HGNC:30282] |
| ENSG00000205730 | HGLibA_23868 | 51 | -0.673381002 | ITPRIPL2 | ITPRIP like 2 [Source:HGNC Symbol;Acc:HGNC:27257] |
| ENSG00000214694 | HGLibA_02849 | 51 | -0.673381002 | ARHGEF33 | Rho guanine nucleotide exchange factor 33 [Source:HGNC Symbol;Acc:HGNC:37252] |
| ENSG00000215421 | HGLibA_55927 | 51 | -0.673381002 | ZNF407 | zinc finger protein 407 [Source:HGNC Symbol;Acc:HGNC:19904] |
| ENSG00000215475 | HGLibA_44111 | 51 | -0.673381002 | SIAH3 | siah E3 ubiquitin protein ligase family member 3 [Source:HGNC Symbol;Acc:HGNC:30553] |
| ENSG00000215644 | HGLibA_18778 | 51 | -0.673381002 | GCGR | glucagon receptor [Source:HGNC Symbol;Acc:HGNC:4192] |
| ENSG00000247077 | HGLibA_36205 | 51 | -0.673381002 | PGAM5 | mitochondrial serine/threonine protein phosphatase [Source:HGNC Symbol;Acc:HGNC:28763]" |
| ENSG00000254647 | HGLibA_23368 | 51 | -0.673381002 | INS | insulin [Source:HGNC Symbol;Acc:HGNC:6081] |
| ENSG00000255150 | HGLibA_14702 | 51 | -0.673381002 | EID3 | EP300 interacting inhibitor of differentiation 3 [Source:HGNC Symbol;Acc:HGNC:32961] |
| ENSG00000261011 | HGLibA_01138 | 51 | -0.673381002 | AGAP11 | ankyrin repeat and PH domain family (AGAP) pseudogene" |
| ENSG00000268651 | HGLibA_11557 | 51 | -0.673381002 | CTAG1A | cancer/testis antigen 1A [Source:HGNC Symbol;Acc:HGNC:24198] |
| ENSG00000275700 | HGLibA_00087 | 51 | -0.673381002 | AATF | apoptosis antagonizing transcription factor [Source:HGNC Symbol;Acc:HGNC:19235] |
| ENSG00000279116 | HGLibA_34510 | 51 | -0.673381002 | OR8D2 | olfactory receptor family 8 subfamily D member 2 (gene/pseudogene) [Source:HGNC Symbol;Acc:HGNC:8482] |
| FAM150A | HGLibA_16156 | 51 | -0.673381002 | FAM150A | - |
| FLJ44313 | HGLibA_17631 | 51 | -0.673381002 | FLJ44313 | - |
| GPR126 | HGLibA_19883 | 51 | -0.673381002 | GPR126 | - |
| HIST1H2BN | HGLibA_21451 | 51 | -0.673381002 | HIST1H2BN | - |
| hsa-mir-1180 | HGLibA_57215 | 51 | -0.673381002 | hsa-mir-1180 | - |
| hsa-mir-29b-1 | HGLibA_58413 | 51 | -0.673381002 | hsa-mir-29b-1 | - |
| hsa-mir-3128 | HGLibA_58564 | 51 | -0.673381002 | hsa-mir-3128 | - |
| hsa-mir-320c-1 | HGLibA_58934 | 51 | -0.673381002 | hsa-mir-320c-1 | - |
| hsa-mir-325 | HGLibA_58968 | 51 | -0.673381002 | hsa-mir-325 | - |
| hsa-mir-367 | HGLibA_59228 | 51 | -0.673381002 | hsa-mir-367 | - |
| hsa-mir-3688-2 | HGLibA_59308 | 51 | -0.673381002 | hsa-mir-3688-2 | - |
| hsa-mir-3691 | HGLibA_59354 | 51 | -0.673381002 | hsa-mir-3691 | - |
| hsa-mir-4310 | HGLibA_59957 | 51 | -0.673381002 | hsa-mir-4310 | - |
| hsa-mir-4433b | HGLibA_60123 | 51 | -0.673381002 | hsa-mir-4433b | - |
| hsa-mir-4493 | HGLibA_60391 | 51 | -0.673381002 | hsa-mir-4493 | - |
| hsa-mir-4649 | HGLibA_60698 | 51 | -0.673381002 | hsa-mir-4649 | - |
| hsa-mir-4709 | HGLibA_60937 | 51 | -0.673381002 | hsa-mir-4709 | - |
| hsa-mir-504 | HGLibA_61473 | 51 | -0.673381002 | hsa-mir-504 | - |
| hsa-mir-517c | HGLibA_61631 | 51 | -0.673381002 | hsa-mir-517c | - |
| hsa-mir-518d | HGLibA_61669 | 51 | -0.673381002 | hsa-mir-518d | - |
| hsa-mir-521-1 | HGLibA_61767 | 51 | -0.673381002 | hsa-mir-521-1 | - |
| hsa-mir-524 | HGLibA_61783 | 51 | -0.673381002 | hsa-mir-524 | - |
| hsa-mir-548aw | HGLibA_61934 | 51 | -0.673381002 | hsa-mir-548aw | - |
| hsa-mir-548g | HGLibA_61990 | 51 | -0.673381002 | hsa-mir-548g | - |
| hsa-mir-548x-2 | HGLibA_62084 | 51 | -0.673381002 | hsa-mir-548x-2 | - |
| hsa-mir-602 | HGLibA_62502 | 51 | -0.673381002 | hsa-mir-602 | - |
| hsa-mir-6082 | HGLibA_62587 | 51 | -0.673381002 | hsa-mir-6082 | - |
| hsa-mir-6130 | HGLibA_62671 | 51 | -0.673381002 | hsa-mir-6130 | - |
| hsa-mir-642b | HGLibA_62812 | 51 | -0.673381002 | hsa-mir-642b | - |
| hsa-mir-6719 | HGLibA_63036 | 51 | -0.673381002 | hsa-mir-6719 | - |
| hsa-mir-6759 | HGLibA_63194 | 51 | -0.673381002 | hsa-mir-6759 | - |
| hsa-mir-6812 | HGLibA_63426 | 51 | -0.673381002 | hsa-mir-6812 | - |
| hsa-mir-7705 | HGLibA_63954 | 51 | -0.673381002 | hsa-mir-7705 | - |
| hsa-mir-921 | HGLibA_64277 | 51 | -0.673381002 | hsa-mir-921 | - |
| LOC339862 | HGLibA_26920 | 51 | -0.673381002 | LOC339862 | - |
| LOC729020 | HGLibA_26997 | 51 | -0.673381002 | LOC729020 | - |
| TGIF2-C20orf24 | HGLibA_49244 | 51 | -0.673381002 | TGIF2-C20orf24 | - |
| C17orf85 | HGLibA_05588 | 50 | -0.682426345 | C17orf85 | - |
| C19orf60 | HGLibA_05691 | 50 | -0.682426345 | C19orf60 | - |
| C2orf27B | HGLibA_06118 | 50 | -0.682426345 | C2orf27B | - |
| C4orf22 | HGLibA_06305 | 50 | -0.682426345 | C4orf22 | - |
| CTSL2 | HGLibA_11721 | 50 | -0.682426345 | CTSL2 | - |
| ENSG00000004939 | HGLibA_45203 | 50 | -0.682426345 | SLC4A1 | solute carrier family 4 member 1 (Diego blood group) [Source:HGNC Symbol;Acc:HGNC:11027] |
| ENSG00000007168 | HGLibA_34990 | 50 | -0.682426345 | PAFAH1B1 | platelet activating factor acetylhydrolase 1b regulatory subunit 1 [Source:HGNC Symbol;Acc:HGNC:8574] |
| ENSG00000015171 | HGLibA_55415 | 50 | -0.682426345 | ZMYND11 | zinc finger MYND-type containing 11 [Source:HGNC Symbol;Acc:HGNC:16966] |
| ENSG00000026751 | HGLibA_44317 | 50 | -0.682426345 | SLAMF7 | SLAM family member 7 [Source:HGNC Symbol;Acc:HGNC:21394] |
| ENSG00000033327 | HGLibA_18324 | 50 | -0.682426345 | GAB2 | GRB2 associated binding protein 2 [Source:HGNC Symbol;Acc:HGNC:14458] |
| ENSG00000053371 | HGLibA_01500 | 50 | -0.682426345 | AKR7A2 | aldo-keto reductase family 7 member A2 [Source:HGNC Symbol;Acc:HGNC:389] |
| ENSG00000053747 | HGLibA_25948 | 50 | -0.682426345 | LAMA3 | laminin subunit alpha 3 [Source:HGNC Symbol;Acc:HGNC:6483] |
| ENSG00000055070 | HGLibA_48114 | 50 | -0.682426345 | SZRD1 | SUZ RNA binding domain containing 1 [Source:HGNC Symbol;Acc:HGNC:30232] |
| ENSG00000069011 | HGLibA_36763 | 50 | -0.682426345 | PITX1 | paired like homeodomain 1 [Source:HGNC Symbol;Acc:HGNC:9004] |
| ENSG00000069206 | HGLibA_00779 | 50 | -0.682426345 | ADAM7 | ADAM metallopeptidase domain 7 [Source:HGNC Symbol;Acc:HGNC:214] |

| | | | | | |
|---|---|---|---|---|---|
| ENSG00000072501 | HGLibA_45640 | 50 | -0.682426345 | SMC1A | structural maintenance of chromosomes 1A [Source:HGNC Symbol;Acc:HGNC:11111] |
| ENSG00000074527 | HGLibA_32860 | 50 | -0.682426345 | NTN4 | netrin 4 [Source:HGNC Symbol;Acc:HGNC:13658] |
| ENSG00000077150 | HGLibA_31819 | 50 | -0.682426345 | NFKB2 | nuclear factor kappa B subunit 2 [Source:HGNC Symbol;Acc:HGNC:7795] |
| ENSG00000077232 | HGLibA_13565 | 50 | -0.682426345 | DNAJC10 | DnaJ heat shock protein family (Hsp40) member C10 [Source:HGNC Symbol;Acc:HGNC:24637] |
| ENSG00000082458 | HGLibA_13330 | 50 | -0.682426345 | DLG3 | discs large MAGUK scaffold protein 3 [Source:HGNC Symbol;Acc:HGNC:2902] |
| ENSG00000083444 | HGLibA_37207 | 50 | -0.682426345 | PLOD1 | 2-oxoglutarate 5-dioxygenase 1 [Source:HGNC Symbol;Acc:HGNC:9081]" |
| ENSG00000085832 | HGLibA_15367 | 50 | -0.682426345 | EPS15 | epidermal growth factor receptor pathway substrate 15 [Source:HGNC Symbol;Acc:HGNC:3419] |
| ENSG00000085998 | HGLibA_37657 | 50 | -0.682426345 | POMGNT1 | 2-) [Source:HGNC Symbol;Acc:HGNC:19139]" |
| ENSG00000086589 | HGLibA_40509 | 50 | -0.682426345 | RBM22 | RNA binding motif protein 22 [Source:HGNC Symbol;Acc:HGNC:25503] |
| ENSG00000088205 | HGLibA_12661 | 50 | -0.682426345 | DDX18 | DEAD-box helicase 18 [Source:HGNC Symbol;Acc:HGNC:2741] |
| ENSG00000088256 | HGLibA_19435 | 50 | -0.682426345 | GNA11 | G protein subunit alpha 11 [Source:HGNC Symbol;Acc:HGNC:4379] |
| ENSG00000089157 | HGLibA_42009 | 50 | -0.682426345 | RPLP0 | ribosomal protein lateral stalk subunit P0 [Source:HGNC Symbol;Acc:HGNC:10371] |
| ENSG00000091483 | HGLibA_17470 | 50 | -0.682426345 | FH | fumarate hydratase [Source:HGNC Symbol;Acc:HGNC:3700] |
| ENSG00000094916 | HGLibA_07456 | 50 | -0.682426345 | CBX5 | chromobox 5 [Source:HGNC Symbol;Acc:HGNC:1555] |
| ENSG00000099365 | HGLibA_47640 | 50 | -0.682426345 | STX1B | syntaxin 1B [Source:HGNC Symbol;Acc:HGNC:18539] |
| ENSG00000100242 | HGLibA_47792 | 50 | -0.682426345 | SUN2 | Sad1 and UNC84 domain containing 2 [Source:HGNC Symbol;Acc:HGNC:14210] |
| ENSG00000100413 | HGLibA_37633 | 50 | -0.682426345 | POLR3H | RNA polymerase III subunit H [Source:HGNC Symbol;Acc:HGNC:30349] |
| ENSG00000100811 | HGLibA_54757 | 50 | -0.682426345 | YY1 | YY1 transcription factor [Source:HGNC Symbol;Acc:HGNC:12856] |
| ENSG00000100815 | HGLibA_51636 | 50 | -0.682426345 | TRIP11 | thyroid hormone receptor interactor 11 [Source:HGNC Symbol;Acc:HGNC:12305] |
| ENSG00000100908 | HGLibA_15027 | 50 | -0.682426345 | EMC9 | ER membrane protein complex subunit 9 [Source:HGNC Symbol;Acc:HGNC:20273] |
| ENSG00000100918 | HGLibA_40762 | 50 | -0.682426345 | REC8 | REC8 meiotic recombination protein [Source:HGNC Symbol;Acc:HGNC:16879] |
| ENSG00000102145 | HGLibA_18664 | 50 | -0.682426345 | GATA1 | GATA binding protein 1 [Source:HGNC Symbol;Acc:HGNC:4170] |
| ENSG00000103061 | HGLibA_45375 | 50 | -0.682426345 | SLC7A6OS | solute carrier family 7 member 6 opposite strand [Source:HGNC Symbol;Acc:HGNC:25807] |
| ENSG00000103740 | HGLibA_00543 | 50 | -0.682426345 | ACSBG1 | acyl-CoA synthetase bubblegum family member 1 [Source:HGNC Symbol;Acc:HGNC:29567] |
| ENSG00000103994 | HGLibA_55437 | 50 | -0.682426345 | ZNF106 | zinc finger protein 106 [Source:HGNC Symbol;Acc:HGNC:12886] |
| ENSG00000105011 | HGLibA_03284 | 50 | -0.682426345 | ASF1B | anti-silencing function 1B histone chaperone [Source:HGNC Symbol;Acc:HGNC:20996] |
| ENSG00000105355 | HGLibA_37176 | 50 | -0.682426345 | PLIN3 | perilipin 3 [Source:HGNC Symbol;Acc:HGNC:16893] |
| ENSG00000105639 | HGLibA_23920 | 50 | -0.682426345 | JAK3 | Janus kinase 3 [Source:HGNC Symbol;Acc:HGNC:6193] |
| ENSG00000107798 | HGLibA_26600 | 50 | -0.682426345 | LIPA | lysosomal acid type [Source:HGNC Symbol;Acc:HGNC:6617]" |
| ENSG00000109189 | HGLibA_53377 | 50 | -0.682426345 | USP46 | ubiquitin specific peptidase 46 [Source:HGNC Symbol;Acc:HGNC:20075] |
| ENSG00000109686 | HGLibA_43941 | 50 | -0.682426345 | SH3D19 | SH3 domain containing 19 [Source:HGNC Symbol;Acc:HGNC:30418] |
| ENSG00000109814 | HGLibA_52865 | 50 | -0.682426345 | UGDH | UDP-glucose 6-dehydrogenase [Source:HGNC Symbol;Acc:HGNC:12525] |
| ENSG00000110721 | HGLibA_09456 | 50 | -0.682426345 | CHKA | choline kinase alpha [Source:HGNC Symbol;Acc:HGNC:1937] |
| ENSG00000111602 | HGLibA_49491 | 50 | -0.682426345 | TIMELESS | timeless circadian regulator [Source:HGNC Symbol;Acc:HGNC:11813] |
| ENSG00000111897 | HGLibA_43484 | 50 | -0.682426345 | SERINC1 | serine incorporator 1 [Source:HGNC Symbol;Acc:HGNC:13464] |
| ENSG00000112769 | HGLibA_25949 | 50 | -0.682426345 | LAMA4 | laminin subunit alpha 4 [Source:HGNC Symbol;Acc:HGNC:6484] |
| ENSG00000115310 | HGLibA_42406 | 50 | -0.682426345 | RTN4 | reticulon 4 [Source:HGNC Symbol;Acc:HGNC:14085] |
| ENSG00000117834 | HGLibA_45286 | 50 | -0.682426345 | SLC5A9 | solute carrier family 5 member 9 [Source:HGNC Symbol;Acc:HGNC:22146] |
| ENSG00000118263 | HGLibA_25080 | 50 | -0.682426345 | KLF7 | Kruppel like factor 7 [Source:HGNC Symbol;Acc:HGNC:6350] |
| ENSG00000118939 | HGLibA_52804 | 50 | -0.682426345 | UCHL3 | ubiquitin C-terminal hydrolase L3 [Source:HGNC Symbol;Acc:HGNC:12515] |
| ENSG00000119314 | HGLibA_39257 | 50 | -0.682426345 | PTBP3 | polypyrimidine tract binding protein 3 [Source:HGNC Symbol;Acc:HGNC:10253] |
| ENSG00000119965 | HGLibA_05101 | 50 | -0.682426345 | C10orf88 | chromosome 10 open reading frame 88 [Source:HGNC Symbol;Acc:HGNC:25822] |
| ENSG00000120075 | HGLibA_21941 | 50 | -0.682426345 | HOXB5 | homeobox B5 [Source:HGNC Symbol;Acc:HGNC:5116] |
| ENSG00000121310 | HGLibA_14397 | 50 | -0.682426345 | ECHDC2 | enoyl-CoA hydratase domain containing 2 [Source:HGNC Symbol;Acc:HGNC:23408] |
| ENSG00000121691 | HGLibA_07353 | 50 | -0.682426345 | CAT | catalase [Source:HGNC Symbol;Acc:HGNC:1516] |
| ENSG00000121905 | HGLibA_22017 | 50 | -0.682426345 | HPCA | hippocalcin [Source:HGNC Symbol;Acc:HGNC:5144] |
| ENSG00000124157 | HGLibA_43382 | 50 | -0.682426345 | SEMG2 | semenogelin 2 [Source:HGNC Symbol;Acc:HGNC:10743] |
| ENSG00000124596 | HGLibA_33196 | 50 | -0.682426345 | OARD1 | O-acyl-ADP-ribose deacylase 1 [Source:HGNC Symbol;Acc:HGNC:21257] |
| ENSG00000124615 | HGLibA_29611 | 50 | -0.682426345 | MOCS1 | molybdenum cofactor synthesis 1 [Source:HGNC Symbol;Acc:HGNC:7190] |
| ENSG00000126790 | HGLibA_25894 | 50 | -0.682426345 | L3HYPDH | trans-L-3-hydroxyproline dehydratase [Source:HGNC Symbol;Acc:HGNC:20488] |
| ENSG00000127412 | HGLibA_51793 | 50 | -0.682426345 | TRPV5 | transient receptor potential cation channel subfamily V member 5 [Source:HGNC Symbol;Acc:HGNC:3145] |
| ENSG00000128487 | HGLibA_46602 | 50 | -0.682426345 | SPECC1 | sperm antigen with calponin homology and coiled-coil domains 1 [Source:HGNC Symbol;Acc:HGNC:30615] |
| ENSG00000128607 | HGLibA_25094 | 50 | -0.682426345 | KLHDC10 | kelch domain containing 10 [Source:HGNC Symbol;Acc:HGNC:22194] |
| ENSG00000128908 | HGLibA_23319 | 50 | -0.682426345 | INO80 | INO80 complex subunit [Source:HGNC Symbol;Acc:HGNC:26956] |
| ENSG00000130203 | HGLibA_02519 | 50 | -0.682426345 | APOE | apolipoprotein E [Source:HGNC Symbol;Acc:HGNC:613] |
| ENSG00000132434 | HGLibA_26006 | 50 | -0.682426345 | LANCL2 | LanC like 2 [Source:HGNC Symbol;Acc:HGNC:6509] |
| ENSG00000132471 | HGLibA_53976 | 50 | -0.682426345 | WBP2 | WW domain binding protein 2 [Source:HGNC Symbol;Acc:HGNC:12738] |
| ENSG00000133105 | HGLibA_42498 | 50 | -0.682426345 | RXFP2 | relaxin family peptide receptor 2 [Source:HGNC Symbol;Acc:HGNC:17318] |
| ENSG00000133250 | HGLibA_55940 | 50 | -0.682426345 | ZNF414 | zinc finger protein 414 [Source:HGNC Symbol;Acc:HGNC:20630] |
| ENSG00000133835 | HGLibA_22193 | 50 | -0.682426345 | HSD17B4 | hydroxysteroid 17-beta dehydrogenase 4 [Source:HGNC Symbol;Acc:HGNC:5213] |
| ENSG00000134153 | HGLibA_15019 | 50 | -0.682426345 | EMC7 | ER membrane protein complex subunit 7 [Source:HGNC Symbol;Acc:HGNC:24301] |
| ENSG00000134594 | HGLibA_39881 | 50 | -0.682426345 | RAB33A | member RAS oncogene family [Source:HGNC Symbol;Acc:HGNC:9773]" |
| ENSG00000134884 | HGLibA_02693 | 50 | -0.682426345 | ARGLU1 | arginine and glutamate rich 1 [Source:HGNC Symbol;Acc:HGNC:25482] |
| ENSG00000135148 | HGLibA_51270 | 50 | -0.682426345 | TRAFD1 | TRAF-type zinc finger domain containing 1 [Source:HGNC Symbol;Acc:HGNC:24808] |
| ENSG00000135372 | HGLibA_31167 | 50 | -0.682426345 | NAT10 | N-acetyltransferase 10 [Source:HGNC Symbol;Acc:HGNC:29830] |
| ENSG00000135636 | HGLibA_14287 | 50 | -0.682426345 | DYSF | dysferlin [Source:HGNC Symbol;Acc:HGNC:3097] |
| ENSG00000135903 | HGLibA_35300 | 50 | -0.682426345 | PAX3 | paired box 3 [Source:HGNC Symbol;Acc:HGNC:8617] |
| ENSG00000136935 | HGLibA_19593 | 50 | -0.682426345 | GOLGA1 | golgin A1 [Source:HGNC Symbol;Acc:HGNC:4424] |
| ENSG00000137080 | HGLibA_22678 | 50 | -0.682426345 | IFNA21 | interferon alpha 21 [Source:HGNC Symbol;Acc:HGNC:5424] |
| ENSG00000137251 | HGLibA_49546 | 50 | -0.682426345 | TINAG | tubulointerstitial nephritis antigen [Source:HGNC Symbol;Acc:HGNC:14599] |
| ENSG00000140067 | HGLibA_16318 | 50 | -0.682426345 | FAM181A | family with sequence similarity 181 member A [Source:HGNC Symbol;Acc:HGNC:20491] |
| ENSG00000140511 | HGLibA_20848 | 50 | -0.682426345 | HAPLN3 | hyaluronan and proteoglycan link protein 3 [Source:HGNC Symbol;Acc:HGNC:21446] |
| ENSG00000140905 | HGLibA_18833 | 50 | -0.682426345 | GCSH | glycine cleavage system protein H [Source:HGNC Symbol;Acc:HGNC:4208] |
| ENSG00000141956 | HGLibA_38380 | 50 | -0.682426345 | PRDM15 | PR/SET domain 15 [Source:HGNC Symbol;Acc:HGNC:13999] |
| ENSG00000143194 | HGLibA_27864 | 50 | -0.682426345 | MAEL | maelstrom spermatogenic transposon silencer [Source:HGNC Symbol;Acc:HGNC:25929] |
| ENSG00000144339 | HGLibA_49835 | 50 | -0.682426345 | TMEFF2 | transmembrane protein with EGF like and two follistatin like domains 2 [Source:HGNC Symbol;Acc:HGNC:11867] |
| ENSG00000144802 | HGLibA_31836 | 50 | -0.682426345 | NFKBIZ | NFKB inhibitor zeta [Source:HGNC Symbol;Acc:HGNC:29805] |
| ENSG00000145780 | HGLibA_17279 | 50 | -0.682426345 | FEM1C | fem-1 homolog C [Source:HGNC Symbol;Acc:HGNC:16933] |
| ENSG00000145868 | HGLibA_17084 | 50 | -0.682426345 | FBXO38 | F-box protein 38 [Source:HGNC Symbol;Acc:HGNC:28844] |
| ENSG00000146143 | HGLibA_38485 | 50 | -0.682426345 | PRIM2 | DNA primase subunit 2 [Source:HGNC Symbol;Acc:HGNC:9370] |
| ENSG00000146535 | HGLibA_19440 | 50 | -0.682426345 | GNA12 | G protein subunit alpha 12 [Source:HGNC Symbol;Acc:HGNC:4380] |
| ENSG00000146938 | HGLibA_32075 | 50 | -0.682426345 | NLGN4X | neuroligin 4 X-linked [Source:HGNC Symbol;Acc:HGNC:14287] |
| ENSG00000147488 | HGLibA_47229 | 50 | -0.682426345 | ST18 | C2H2C-type zinc finger [Source:HGNC Symbol;Acc:HGNC:18695]" |
| ENSG00000147996 | HGLibA_07439 | 50 | -0.682426345 | CBWD5 | COBW domain containing 5 [Source:HGNC Symbol;Acc:HGNC:24584] |
| ENSG00000149100 | HGLibA_14801 | 50 | -0.682426345 | EIF3M | eukaryotic translation initiation factor 3 subunit M [Source:HGNC Symbol;Acc:HGNC:24460] |
| ENSG00000149596 | HGLibA_23982 | 50 | -0.682426345 | JPH2 | junctophilin 2 [Source:HGNC Symbol;Acc:HGNC:14202] |
| ENSG00000149639 | HGLibA_46152 | 50 | -0.682426345 | SOGA1 | autophagy associated 1 [Source:HGNC Symbol;Acc:HGNC:16111]" |
| ENSG00000149646 | HGLibA_10244 | 50 | -0.682426345 | CNBD2 | cyclic nucleotide binding domain containing 2 [Source:HGNC Symbol;Acc:HGNC:16145] |
| ENSG00000151502 | HGLibA_53755 | 50 | -0.682426345 | VPS26B | retromer complex component B [Source:HGNC Symbol;Acc:HGNC:28119]" |
| ENSG00000152102 | HGLibA_16248 | 50 | -0.682426345 | FAM168B | family with sequence similarity 168 member B [Source:HGNC Symbol;Acc:HGNC:27016] |
| ENSG00000153094 | HGLibA_04307 | 50 | -0.682426345 | BCL2L11 | BCL2 like 11 [Source:HGNC Symbol;Acc:HGNC:994] |
| ENSG00000155367 | HGLibA_37985 | 50 | -0.682426345 | PPM1J | Mg2+/Mn2+ dependent 1J [Source:HGNC Symbol;Acc:HGNC:20785]" |
| ENSG00000156103 | HGLibA_29498 | 50 | -0.682426345 | MMP16 | matrix metallopeptidase 16 [Source:HGNC Symbol;Acc:HGNC:7162] |
| ENSG00000158470 | HGLibA_04034 | 50 | -0.682426345 | B4GALT5 | 4-galactosyltransferase 5 [Source:HGNC Symbol;Acc:HGNC:928]" |
| ENSG00000158865 | HGLibA_45260 | 50 | -0.682426345 | SLC5A11 | solute carrier family 5 member 11 [Source:HGNC Symbol;Acc:HGNC:23091] |
| ENSG00000159182 | HGLibA_38264 | 50 | -0.682426345 | PRAC1 | PRAC1 small nuclear protein [Source:HGNC Symbol;Acc:HGNC:30591] |
| ENSG00000160271 | HGLibA_40165 | 50 | -0.682426345 | RALGDS | ral guanine nucleotide dissociation stimulator [Source:HGNC Symbol;Acc:HGNC:9842] |
| ENSG00000160813 | HGLibA_38090 | 50 | -0.682426345 | PPP1R35 | protein phosphatase 1 regulatory subunit 35 [Source:HGNC Symbol;Acc:HGNC:28320] |
| ENSG00000162482 | HGLibA_01503 | 50 | -0.682426345 | AKR7A3 | aldo-keto reductase family 7 member A3 [Source:HGNC Symbol;Acc:HGNC:390] |
| ENSG00000162493 | HGLibA_35933 | 50 | -0.682426345 | PDPN | podoplanin [Source:HGNC Symbol;Acc:HGNC:29602] |
| ENSG00000163166 | HGLibA_23886 | 50 | -0.682426345 | IWS1 | SUPT6H interacting protein [Source:HGNC Symbol;Acc:HGNC:25467]" |
| ENSG00000163395 | HGLibA_22853 | 50 | -0.682426345 | IGFN1 | immunoglobulin-like and fibronectin type III domain containing 1 [Source:HGNC Symbol;Acc:HGNC:24607] |
| ENSG00000163399 | HGLibA_03580 | 50 | -0.682426345 | ATP1A1 | ATPase Na+/K+ transporting subunit alpha 1 [Source:HGNC Symbol;Acc:HGNC:799] |
| ENSG00000163576 | HGLibA_14568 | 50 | -0.682426345 | EFHB | EF-hand domain family member B [Source:HGNC Symbol;Acc:HGNC:26330] |
| ENSG00000163840 | HGLibA_14088 | 50 | -0.682426345 | DTX3L | deltex E3 ubiquitin ligase 3L [Source:HGNC Symbol;Acc:HGNC:30323] |
| ENSG00000163882 | HGLibA_37584 | 50 | -0.682426345 | POLR2H | RNA polymerase II subunit H [Source:HGNC Symbol;Acc:HGNC:9195] |
| ENSG00000163923 | HGLibA_41975 | 50 | -0.682426345 | RPL39L | ribosomal protein L39 like [Source:HGNC Symbol;Acc:HGNC:17094] |
| ENSG00000164434 | HGLibA_15928 | 50 | -0.682426345 | FABP7 | fatty acid binding protein 7 [Source:HGNC Symbol;Acc:HGNC:3562] |
| ENSG00000164466 | HGLibA_43780 | 50 | -0.682426345 | SFXN1 | sideroflexin 1 [Source:HGNC Symbol;Acc:HGNC:16085] |
| ENSG00000166016 | HGLibA_00338 | 50 | -0.682426345 | ABTB2 | ankyrin repeat and BTB domain containing 2 [Source:HGNC Symbol;Acc:HGNC:23842] |
| ENSG00000167123 | HGLibA_09190 | 50 | -0.682426345 | CERCAM | cerebral endothelial cell adhesion molecule [Source:HGNC Symbol;Acc:HGNC:23723] |
| ENSG00000167508 | HGLibA_30594 | 50 | -0.682426345 | MVD | mevalonate diphosphate decarboxylase [Source:HGNC Symbol;Acc:HGNC:7529] |
| ENSG00000167720 | HGLibA_47040 | 50 | -0.682426345 | SRR | serine racemase [Source:HGNC Symbol;Acc:HGNC:14398] |
| ENSG00000169231 | HGLibA_49328 | 50 | -0.682426345 | THBS3 | thrombospondin 3 [Source:HGNC Symbol;Acc:HGNC:11787] |
| ENSG00000169515 | HGLibA_07875 | 50 | -0.682426345 | CCDC8 | coiled-coil domain containing 8 [Source:HGNC Symbol;Acc:HGNC:25367] |
| ENSG00000169515 | HGLibA_07874 | 50 | -0.682426345 | CCDC8 | coiled-coil domain containing 8 [Source:HGNC Symbol;Acc:HGNC:25367] |
| ENSG00000170160 | HGLibA_07623 | 50 | -0.682426345 | CCDC144A | coiled-coil domain containing 144A [Source:HGNC Symbol;Acc:HGNC:29072] |
| ENSG00000170613 | HGLibA_16688 | 50 | -0.682426345 | FAM71B | family with sequence similarity 71 member B [Source:HGNC Symbol;Acc:HGNC:28397] |
| ENSG00000171291 | HGLibA_55996 | 50 | -0.682426345 | ZNF439 | zinc finger protein 439 [Source:HGNC Symbol;Acc:HGNC:20873] |
| ENSG00000172139 | HGLibA_45440 | 50 | -0.682426345 | SLC9C1 | solute carrier family 9 member C1 [Source:HGNC Symbol;Acc:HGNC:31401] |
| ENSG00000172216 | HGLibA_08959 | 50 | -0.682426345 | CEBPB | CCAAT enhancer binding protein beta [Source:HGNC Symbol;Acc:HGNC:1834] |
| ENSG00000172456 | HGLibA_17459 | 50 | -0.682426345 | FGGY | FGGY carbohydrate kinase domain containing [Source:HGNC Symbol;Acc:HGNC:25610] |
| ENSG00000173349 | HGLibA_43756 | 50 | -0.682426345 | SFT2D3 | SFT2 domain containing 3 [Source:HGNC Symbol;Acc:HGNC:28767] |
| ENSG00000173809 | HGLibA_48901 | 50 | -0.682426345 | TDRD12 | tudor domain containing 12 [Source:HGNC Symbol;Acc:HGNC:25044] |
| ENSG00000173930 | HGLibA_45471 | 50 | -0.682426345 | SLCO4C1 | solute carrier organic anion transporter family member 4C1 [Source:HGNC Symbol;Acc:HGNC:23612] |
| ENSG00000175513 | HGLibA_51850 | 50 | -0.682426345 | TSGA10IP | testis specific 10 interacting protein [Source:HGNC Symbol;Acc:HGNC:26555] |
| ENSG00000175764 | HGLibA_52192 | 50 | -0.682426345 | TTLL11 | tubulin tyrosine ligase like 11 [Source:HGNC Symbol;Acc:HGNC:18113] |
| ENSG00000175874 | HGLibA_11124 | 50 | -0.682426345 | CREG2 | cellular repressor of E1A stimulated genes 2 [Source:HGNC Symbol;Acc:HGNC:14272] |
| ENSG00000175984 | HGLibA_12940 | 50 | -0.682426345 | DENND2C | DENN domain containing 2C [Source:HGNC Symbol;Acc:HGNC:24748] |
| ENSG00000178201 | HGLibA_53699 | 50 | -0.682426345 | VN1R1 | vomeronasal 1 receptor 1 [Source:HGNC Symbol;Acc:HGNC:13548] |
| ENSG00000179615 | HGLibA_33758 | 50 | -0.682426345 | OR2AP1 | olfactory receptor family 2 subfamily AP member 1 [Source:HGNC Symbol;Acc:HGNC:15335] |
| ENSG00000179918 | HGLibA_43414 | 50 | -0.682426345 | SEPHS2 | selenophosphate synthetase 2 [Source:HGNC Symbol;Acc:HGNC:19686] |
| ENSG00000180822 | HGLibA_39214 | 50 | -0.682426345 | PSMG4 | proteasome assembly chaperone 4 [Source:HGNC Symbol;Acc:HGNC:21108] |
| ENSG00000181392 | HGLibA_47986 | 50 | -0.682426345 | SYNE4 | spectrin repeat containing nuclear envelope family member 4 [Source:HGNC Symbol;Acc:HGNC:26703] |
| ENSG00000181616 | HGLibA_34169 | 50 | -0.682426345 | OR52H1 | olfactory receptor family 52 subfamily H member 1 [Source:HGNC Symbol;Acc:HGNC:15218] |

| | | | | | |
|---|---|---|---|---|---|
| ENSG00000181778 | HGLibA_50322 | 50 | -0.682426345 | TMEM252 | transmembrane protein 252 [Source:HGNC Symbol;Acc:HGNC:28537] |
| ENSG00000182223 | HGLibA_54778 | 50 | -0.682426345 | ZAR1 | zygote arrest 1 [Source:HGNC Symbol;Acc:HGNC:20436] |
| ENSG00000182521 | HGLibA_48629 | 50 | -0.682426345 | TBPL2 | TATA-box binding protein like 2 [Source:HGNC Symbol;Acc:HGNC:19841] |
| ENSG00000182613 | HGLibA_33898 | 50 | -0.682426345 | OR2V2 | olfactory receptor family 2 subfamily V member 2 [Source:HGNC Symbol;Acc:HGNC:15341] |
| ENSG00000182652 | HGLibA_34058 | 50 | -0.682426345 | OR4Q3 | olfactory receptor family 4 subfamily Q member 3 [Source:HGNC Symbol;Acc:HGNC:15426] |
| ENSG00000184208 | HGLibA_06082 | 50 | -0.682426345 | C22orf46 | chromosome 22 open reading frame 46 [Source:HGNC Symbol;Acc:HGNC:26294] |
| ENSG00000184459 | HGLibA_04735 | 50 | -0.682426345 | BPIFC | BPI fold containing family C [Source:HGNC Symbol;Acc:HGNC:16503] |
| ENSG00000184898 | HGLibA_40554 | 50 | -0.682426345 | RBM43 | RNA binding motif protein 43 [Source:HGNC Symbol;Acc:HGNC:24790] |
| ENSG00000185046 | HGLibA_02153 | 50 | -0.682426345 | ANKS1B | ankyrin repeat and sterile alpha motif domain containing 1B [Source:HGNC Symbol;Acc:HGNC:24600] |
| ENSG00000185088 | HGLibA_42128 | 50 | -0.682426345 | RPS27L | ribosomal protein S27 like [Source:HGNC Symbol;Acc:HGNC:18476] |
| ENSG00000187266 | HGLibA_15352 | 50 | -0.682426345 | EPOR | erythropoietin receptor [Source:HGNC Symbol;Acc:HGNC:3416] |
| ENSG00000187372 | HGLibA_35481 | 50 | -0.682426345 | PCDHB13 | protocadherin beta 13 [Source:HGNC Symbol;Acc:HGNC:8684] |
| ENSG00000187650 | HGLibA_53688 | 50 | -0.682426345 | VMAC | vimentin type intermediate filament associated coiled-coil protein [Source:HGNC Symbol;Acc:HGNC:33803] |
| ENSG00000188060 | HGLibA_39945 | 50 | -0.682426345 | RAB42 | member RAS oncogene family [Source:HGNC Symbol;Acc:HGNC:28702]" |
| ENSG00000188290 | HGLibA_21186 | 50 | -0.682426345 | HES4 | hes family bHLH transcription factor 4 [Source:HGNC Symbol;Acc:HGNC:24149] |
| ENSG00000188324 | HGLibA_34394 | 50 | -0.682426345 | OR6C6 | olfactory receptor family 6 subfamily C member 6 [Source:HGNC Symbol;Acc:HGNC:31293] |
| ENSG00000196139 | HGLibA_01488 | 50 | -0.682426345 | AKR1C3 | aldo-keto reductase family 1 member C3 [Source:HGNC Symbol;Acc:HGNC:386] |
| ENSG00000196539 | HGLibA_33873 | 50 | -0.682426345 | OR2T3 | olfactory receptor family 2 subfamily T member 3 [Source:HGNC Symbol;Acc:HGNC:14727] |
| ENSG00000197889 | HGLibA_28865 | 50 | -0.682426345 | MEIG1 | meiosis/spermiogenesis associated 1 [Source:HGNC Symbol;Acc:HGNC:23429] |
| ENSG00000197928 | HGLibA_56518 | 50 | -0.682426345 | ZNF677 | zinc finger protein 677 [Source:HGNC Symbol;Acc:HGNC:28730] |
| ENSG00000198128 | HGLibA_33823 | 50 | -0.682426345 | OR2L3 | olfactory receptor family 2 subfamily L member 3 [Source:HGNC Symbol;Acc:HGNC:15009] |
| ENSG00000198160 | HGLibA_29251 | 50 | -0.682426345 | MIER1 | MIER1 transcriptional regulator [Source:HGNC Symbol;Acc:HGNC:29657] |
| ENSG00000198681 | HGLibA_27890 | 50 | -0.682426345 | MAGEA1 | MAGE family member A1 [Source:HGNC Symbol;Acc:HGNC:6796] |
| ENSG00000198719 | HGLibA_13360 | 50 | -0.682426345 | DLL1 | delta like canonical Notch ligand 1 [Source:HGNC Symbol;Acc:HGNC:2908] |
| ENSG00000198728 | HGLibA_26212 | 50 | -0.682426345 | LDB1 | LIM domain binding 1 [Source:HGNC Symbol;Acc:HGNC:6532] |
| ENSG00000203756 | HGLibA_50296 | 50 | -0.682426345 | TMEM244 | transmembrane protein 244 [Source:HGNC Symbol;Acc:HGNC:21571] |
| ENSG00000203837 | HGLibA_37362 | 50 | -0.682426345 | PNLIPRP3 | pancreatic lipase related protein 3 [Source:HGNC Symbol;Acc:HGNC:23492] |
| ENSG00000203943 | HGLibA_42671 | 50 | -0.682426345 | SAMD13 | sterile alpha motif domain containing 13 [Source:HGNC Symbol;Acc:HGNC:24582] |
| ENSG00000204599 | HGLibA_51483 | 50 | -0.682426345 | TRIM39 | tripartite motif containing 39 [Source:HGNC Symbol;Acc:HGNC:10065] |
| ENSG00000204634 | HGLibA_48567 | 50 | -0.682426345 | TBC1D8 | TBC1 domain family member 8 [Source:HGNC Symbol;Acc:HGNC:17791] |
| ENSG00000205089 | HGLibA_08104 | 50 | -0.682426345 | CCNI2 | cyclin I family member 2 [Source:HGNC Symbol;Acc:HGNC:33869] |
| ENSG00000205097 | HGLibA_17996 | 50 | -0.682426345 | FRG2 | FSHD region gene 2 [Source:HGNC Symbol;Acc:HGNC:19136] |
| ENSG00000206203 | HGLibA_52012 | 50 | -0.682426345 | TSSK2 | testis specific serine kinase 2 [Source:HGNC Symbol;Acc:HGNC:11401] |
| ENSG00000213186 | HGLibA_51555 | 50 | -0.682426345 | TRIM59 | tripartite motif containing 59 [Source:HGNC Symbol;Acc:HGNC:30834] |
| ENSG00000214842 | HGLibA_40083 | 50 | -0.682426345 | RAD51AP2 | RAD51 associated protein 2 [Source:HGNC Symbol;Acc:HGNC:34417] |
| ENSG00000215115 | HGLibA_11933 | 50 | -0.682426345 | CXorf49 | chromosome X open reading frame 49 [Source:HGNC Symbol;Acc:HGNC:30891] |
| ENSG00000221826 | HGLibA_39042 | 50 | -0.682426345 | PSG3 | pregnancy specific beta-1-glycoprotein 3 [Source:HGNC Symbol;Acc:HGNC:9520] |
| ENSG00000221867 | HGLibA_27910 | 50 | -0.682426345 | MAGEA3 | MAGE family member A3 [Source:HGNC Symbol;Acc:HGNC:6801] |
| ENSG00000225697 | HGLibA_44800 | 50 | -0.682426345 | SLC26A6 | solute carrier family 26 member 6 [Source:HGNC Symbol;Acc:HGNC:14472] |
| ENSG00000232382 | HGLibA_34309 | 50 | -0.682426345 | OR5K1 | olfactory receptor family 5 subfamily K member 1 [Source:HGNC Symbol;Acc:HGNC:8349] |
| ENSG00000240771 | HGLibA_02836 | 50 | -0.682426345 | ARHGEF25 | Rho guanine nucleotide exchange factor 25 [Source:HGNC Symbol;Acc:HGNC:30275] |
| ENSG00000254122 | HGLibA_35569 | 50 | -0.682426345 | PCDHGB7 | 7 [Source:HGNC Symbol;Acc:HGNC:8714]" |
| ENSG00000254585 | HGLibA_27992 | 50 | -0.682426345 | MAGEL2 | MAGE family member L2 [Source:HGNC Symbol;Acc:HGNC:6814] |
| ENSG00000255833 | HGLibA_49461 | 50 | -0.682426345 | TIFAB | TIFA inhibitor [Source:HGNC Symbol;Acc:HGNC:34024] |
| ENSG00000257335 | HGLibA_29127 | 50 | -0.682426345 | MGAM | maltase-glucoamylase [Source:HGNC Symbol;Acc:HGNC:7043] |
| ENSG00000261052 | HGLibA_47736 | 50 | -0.682426345 | SULT1A3 | sulfotransferase family 1A member 3 [Source:HGNC Symbol;Acc:HGNC:11455] |
| ENSG00000261796 | HGLibA_23696 | 50 | -0.682426345 | ISY1-RAB43 | ISY1-RAB43 readthrough [Source:HGNC Symbol;Acc:HGNC:42969] |
| ENSG00000271449 | HGLibA_11502 | 50 | -0.682426345 | CT45A2 | cancer/testis antigen family 45 member A2 [Source:HGNC Symbol;Acc:HGNC:28400] |
| ENSG00000284922 | HGLibA_27507 | 50 | -0.682426345 | LRTOMT | novel protein |
| FAM21C | HGLibA_16511 | 50 | -0.682426345 | FAM21C | - |
| FLJ44635 | HGLibA_17633 | 50 | -0.682426345 | FLJ44635 | - |
| FOPNL | HGLibA_17792 | 50 | -0.682426345 | FOPNL | - |
| FTSJ2 | HGLibA_18138 | 50 | -0.682426345 | FTSJ2 | - |
| HIST1H2BB | HGLibA_21413 | 50 | -0.682426345 | HIST1H2BB | - |
| hsa-mir-1181 | HGLibA_57217 | 50 | -0.682426345 | hsa-mir-1181 | - |
| hsa-mir-1281 | HGLibA_57581 | 50 | -0.682426345 | hsa-mir-1281 | - |
| hsa-mir-1289-2 | HGLibA_57626 | 50 | -0.682426345 | hsa-mir-1289-2 | - |
| hsa-mir-1293 | HGLibA_57649 | 50 | -0.682426345 | hsa-mir-1293 | - |
| hsa-mir-149 | HGLibA_57887 | 50 | -0.682426345 | hsa-mir-149 | - |
| hsa-mir-1911 | HGLibA_58049 | 50 | -0.682426345 | hsa-mir-1911 | - |
| hsa-mir-203a | HGLibA_58168 | 50 | -0.682426345 | hsa-mir-203a | - |
| hsa-mir-302a | HGLibA_58435 | 50 | -0.682426345 | hsa-mir-302a | - |
| hsa-mir-3117 | HGLibA_58512 | 50 | -0.682426345 | hsa-mir-3117 | - |
| hsa-mir-3120 | HGLibA_58531 | 50 | -0.682426345 | hsa-mir-3120 | - |
| hsa-mir-3147 | HGLibA_58645 | 50 | -0.682426345 | hsa-mir-3147 | - |
| hsa-mir-3198-1 | HGLibA_58888 | 50 | -0.682426345 | hsa-mir-3198-1 | - |
| hsa-mir-3198-2 | HGLibA_58895 | 50 | -0.682426345 | hsa-mir-3198-2 | - |
| hsa-mir-369 | HGLibA_59341 | 50 | -0.682426345 | hsa-mir-369 | - |
| hsa-mir-411 | HGLibA_59684 | 50 | -0.682426345 | hsa-mir-411 | - |
| hsa-mir-4320 | HGLibA_60003 | 50 | -0.682426345 | hsa-mir-4320 | - |
| hsa-mir-4423 | HGLibA_60078 | 50 | -0.682426345 | hsa-mir-4423 | - |
| hsa-mir-4520b | HGLibA_60537 | 50 | -0.682426345 | hsa-mir-4520b | - |
| hsa-mir-4648 | HGLibA_60694 | 50 | -0.682426345 | hsa-mir-4648 | - |
| hsa-mir-4762 | HGLibA_61146 | 50 | -0.682426345 | hsa-mir-4762 | - |
| hsa-mir-4769 | HGLibA_61173 | 50 | -0.682426345 | hsa-mir-4769 | - |
| hsa-mir-4776-2 | HGLibA_61211 | 50 | -0.682426345 | hsa-mir-4776-2 | - |
| hsa-mir-513a-1 | HGLibA_61571 | 50 | -0.682426345 | hsa-mir-513a-1 | - |
| hsa-mir-5188 | HGLibA_61643 | 50 | -0.682426345 | hsa-mir-5188 | - |
| hsa-mir-520g | HGLibA_61760 | 50 | -0.682426345 | hsa-mir-520g | - |
| hsa-mir-548ab | HGLibA_61857 | 50 | -0.682426345 | hsa-mir-548ab | - |
| hsa-mir-548h-5 | HGLibA_62008 | 50 | -0.682426345 | hsa-mir-548h-5 | - |
| hsa-mir-548q | HGLibA_62060 | 50 | -0.682426345 | hsa-mir-548q | - |
| hsa-mir-561 | HGLibA_62206 | 50 | -0.682426345 | hsa-mir-561 | - |
| hsa-mir-613 | HGLibA_62667 | 50 | -0.682426345 | hsa-mir-613 | - |
| hsa-mir-6789 | HGLibA_63334 | 50 | -0.682426345 | hsa-mir-6789 | - |
| hsa-mir-6802 | HGLibA_63385 | 50 | -0.682426345 | hsa-mir-6802 | - |
| hsa-mir-6861 | HGLibA_63625 | 50 | -0.682426345 | hsa-mir-6861 | - |
| hsa-mir-6886 | HGLibA_63730 | 50 | -0.682426345 | hsa-mir-6886 | - |
| hsa-mir-7844 | HGLibA_63966 | 50 | -0.682426345 | hsa-mir-7844 | - |
| hsa-mir-7852 | HGLibA_63997 | 50 | -0.682426345 | hsa-mir-7852 | - |
| hsa-mir-8054 | HGLibA_64054 | 50 | -0.682426345 | hsa-mir-8054 | - |
| hsa-mir-8083 | HGLibA_64176 | 50 | -0.682426345 | hsa-mir-8083 | - |
| hsa-mir-941-2 | HGLibA_64343 | 50 | -0.682426345 | hsa-mir-941-2 | - |
| KIAA0430 | HGLibA_24638 | 50 | -0.682426345 | KIAA0430 | - |
| KIAA0947 | HGLibA_24674 | 50 | -0.682426345 | KIAA0947 | - |
| KIAA1324 | HGLibA_24736 | 50 | -0.682426345 | KIAA1324 | - |
| LOC643037 | HGLibA_26964 | 50 | -0.682426345 | LOC643037 | - |
| PPAPDC1B | HGLibA_37827 | 50 | -0.682426345 | PPAPDC1B | - |
| SLMO1 | HGLibA_45536 | 50 | -0.682426345 | SLMO1 | - |
| TMEM159 | HGLibA_50019 | 50 | -0.682426345 | TMEM159 | - |
| UPK3BL | HGLibA_53082 | 50 | -0.682426345 | UPK3BL | - |
| C11orf48 | HGLibA_05144 | 49 | -0.691471687 | C11orf48 | - |
| C2orf44 | HGLibA_06130 | 49 | -0.691471687 | C2orf44 | - |
| C5orf48 | HGLibA_06396 | 49 | -0.691471687 | C5orf48 | - |
| CARKD | HGLibA_07249 | 49 | -0.691471687 | CARKD | - |
| DUSP27 | HGLibA_14173 | 49 | -0.691471687 | DUSP27 | - |
| ENSG00000004776 | HGLibA_22313 | 49 | -0.691471687 | HSPB6 | heat shock protein family B (small) member 6 [Source:HGNC Symbol;Acc:HGNC:26511] |
| ENSG00000005471 | HGLibA_00139 | 49 | -0.691471687 | ABCB4 | ATP binding cassette subfamily B member 4 [Source:HGNC Symbol;Acc:HGNC:45] |
| ENSG00000006210 | HGLibA_11833 | 49 | -0.691471687 | CX3CL1 | C-X3-C motif chemokine ligand 1 [Source:HGNC Symbol;Acc:HGNC:10647] |
| ENSG00000007376 | HGLibA_42207 | 49 | -0.691471687 | RPUSD1 | RNA pseudouridine synthase domain containing 1 [Source:HGNC Symbol;Acc:HGNC:14173] |
| ENSG00000009780 | HGLibA_16721 | 49 | -0.691471687 | FAM76A | family with sequence similarity 76 member A [Source:HGNC Symbol;Acc:HGNC:28530] |
| ENSG00000010295 | HGLibA_22588 | 49 | -0.691471687 | IFFO1 | intermediate filament family orphan 1 [Source:HGNC Symbol;Acc:HGNC:24970] |
| ENSG00000011600 | HGLibA_52472 | 49 | -0.691471687 | TYROBP | TYRO protein tyrosine kinase binding protein [Source:HGNC Symbol;Acc:HGNC:12449] |
| ENSG00000015520 | HGLibA_32420 | 49 | -0.691471687 | NPC1L1 | NPC1 like intracellular cholesterol transporter 1 [Source:HGNC Symbol;Acc:HGNC:7898] |
| ENSG00000023892 | HGLibA_12786 | 49 | -0.691471687 | DEF6 | guanine nucleotide exchange factor [Source:HGNC Symbol;Acc:HGNC:2760]" |
| ENSG00000033011 | HGLibA_01601 | 49 | -0.691471687 | ALG1 | chitobiosyldiphosphodolichol beta-mannosyltransferase [Source:HGNC Symbol;Acc:HGNC:18294]" |
| ENSG00000042813 | HGLibA_56961 | 49 | -0.691471687 | ZPBP | zona pellucida binding protein [Source:HGNC Symbol;Acc:HGNC:15662] |
| ENSG00000054523 | HGLibA_24885 | 49 | -0.691471687 | KIF1B | kinesin family member 1B [Source:HGNC Symbol;Acc:HGNC:16636] |
| ENSG00000054965 | HGLibA_16247 | 49 | -0.691471687 | FAM168A | family with sequence similarity 168 member A [Source:HGNC Symbol;Acc:HGNC:28999] |
| ENSG00000066735 | HGLibA_24915 | 49 | -0.691471687 | KIF26A | kinesin family member 26A [Source:HGNC Symbol;Acc:HGNC:20226] |
| ENSG00000068305 | HGLibA_28827 | 49 | -0.691471687 | MEF2A | myocyte enhancer factor 2A [Source:HGNC Symbol;Acc:HGNC:6993] |
| ENSG00000068885 | HGLibA_22772 | 49 | -0.691471687 | IFT80 | intraflagellar transport 80 [Source:HGNC Symbol;Acc:HGNC:29262] |
| ENSG00000069812 | HGLibA_21181 | 49 | -0.691471687 | HES2 | hes family bHLH transcription factor 2 [Source:HGNC Symbol;Acc:HGNC:16005] |
| ENSG00000073614 | HGLibA_24520 | 49 | -0.691471687 | KDM5A | lysine demethylase 5A [Source:HGNC Symbol;Acc:HGNC:9886] |
| ENSG00000074219 | HGLibA_48930 | 49 | -0.691471687 | TEAD2 | TEA domain transcription factor 2 [Source:HGNC Symbol;Acc:HGNC:11715] |
| ENSG00000074319 | HGLibA_51845 | 49 | -0.691471687 | TSG101 | tumor susceptibility 101 [Source:HGNC Symbol;Acc:HGNC:15971] |
| ENSG00000075826 | HGLibA_43243 | 49 | -0.691471687 | SEC31B | COPII coat complex component [Source:HGNC Symbol;Acc:HGNC:23197]" |
| ENSG00000083817 | HGLibA_55946 | 49 | -0.691471687 | ZNF416 | zinc finger protein 416 [Source:HGNC Symbol;Acc:HGNC:20645] |
| ENSG00000084652 | HGLibA_52390 | 49 | -0.691471687 | TXLNA | taxilin alpha [Source:HGNC Symbol;Acc:HGNC:30685] |
| ENSG00000088053 | HGLibA_19712 | 49 | -0.691471687 | GP6 | glycoprotein VI platelet [Source:HGNC Symbol;Acc:HGNC:14388] |
| ENSG00000091513 | HGLibA_49115 | 49 | -0.691471687 | TF | transferrin [Source:HGNC Symbol;Acc:HGNC:11740] |
| ENSG00000095139 | HGLibA_02638 | 49 | -0.691471687 | ARCN1 | archain 1 [Source:HGNC Symbol;Acc:HGNC:649] |
| ENSG00000099889 | HGLibA_03176 | 49 | -0.691471687 | ARVCF | delta catenin family member [Source:HGNC Symbol;Acc:HGNC:728]" |
| ENSG00000100129 | HGLibA_14798 | 49 | -0.691471687 | EIF3L | eukaryotic translation initiation factor 3 subunit L [Source:HGNC Symbol;Acc:HGNC:18138] |
| ENSG00000100365 | HGLibA_31303 | 49 | -0.691471687 | NCF4 | neutrophil cytosolic factor 4 [Source:HGNC Symbol;Acc:HGNC:7662] |

| | | | | | |
|---|---|---|---|---|---|
| ENSG00000101255 | HGLibA_51396 | 49 | -0.691471687 | TRIB3 | tribbles pseudokinase 3 [Source:HGNC Symbol;Acc:HGNC:16228] |
| ENSG00000101292 | HGLibA_38673 | 49 | -0.691471687 | PROKR2 | prokineticin receptor 2 [Source:HGNC Symbol;Acc:HGNC:15836] |
| ENSG00000102908 | HGLibA_31770 | 49 | -0.691471687 | NFAT5 | nuclear factor of activated T cells 5 [Source:HGNC Symbol;Acc:HGNC:7774] |
| ENSG00000103199 | HGLibA_56108 | 49 | -0.691471687 | ZNF500 | zinc finger protein 500 [Source:HGNC Symbol;Acc:HGNC:23716] |
| ENSG00000103740 | HGLibA_00541 | 49 | -0.691471687 | ACSBG1 | acyl-CoA synthetase bubblegum family member 1 [Source:HGNC Symbol;Acc:HGNC:29567] |
| ENSG00000104140 | HGLibA_41212 | 49 | -0.691471687 | RHOV | ras homolog family member V [Source:HGNC Symbol;Acc:HGNC:18313] |
| ENSG00000104205 | HGLibA_43832 | 49 | -0.691471687 | SGK3 | serum/glucocorticoid regulated kinase family member 3 [Source:HGNC Symbol;Acc:HGNC:10812] |
| ENSG00000104219 | HGLibA_55096 | 49 | -0.691471687 | ZDHHC2 | zinc finger DHHC-type containing 2 [Source:HGNC Symbol;Acc:HGNC:18469] |
| ENSG00000105357 | HGLibA_30716 | 49 | -0.691471687 | MYH14 | myosin heavy chain 14 [Source:HGNC Symbol;Acc:HGNC:23212] |
| ENSG00000105642 | HGLibA_24355 | 49 | -0.691471687 | KCNN1 | potassium calcium-activated channel subfamily N member 1 [Source:HGNC Symbol;Acc:HGNC:6290] |
| ENSG00000105852 | HGLibA_37685 | 49 | -0.691471687 | PON3 | paraoxonase 3 [Source:HGNC Symbol;Acc:HGNC:9206] |
| ENSG00000105880 | HGLibA_13385 | 49 | -0.691471687 | DLX5 | distal-less homeobox 5 [Source:HGNC Symbol;Acc:HGNC:2918] |
| ENSG00000106459 | HGLibA_32672 | 49 | -0.691471687 | NRF1 | nuclear respiratory factor 1 [Source:HGNC Symbol;Acc:HGNC:7996] |
| ENSG00000106511 | HGLibA_28891 | 49 | -0.691471687 | MEOX2 | mesenchyme homeobox 2 [Source:HGNC Symbol;Acc:HGNC:7014] |
| ENSG00000108639 | HGLibA_47995 | 49 | -0.691471687 | SYNGR2 | synaptogyrin 2 [Source:HGNC Symbol;Acc:HGNC:11499] |
| ENSG00000109805 | HGLibA_31270 | 49 | -0.691471687 | NCAPG | non-SMC condensin I complex subunit G [Source:HGNC Symbol;Acc:HGNC:24304] |
| ENSG00000110042 | HGLibA_14090 | 49 | -0.691471687 | DTX4 | deltex E3 ubiquitin ligase 4 [Source:HGNC Symbol;Acc:HGNC:29151] |
| ENSG00000111110 | HGLibA_37980 | 49 | -0.691471687 | PPM1H | Mg2+/Mn2+ dependent 1H [Source:HGNC Symbol;Acc:HGNC:18583]" |
| ENSG00000112309 | HGLibA_03976 | 49 | -0.691471687 | B3GAT2 | 3-glucuronyltransferase 2 [Source:HGNC Symbol;Acc:HGNC:922]" |
| ENSG00000112365 | HGLibA_54847 | 49 | -0.691471687 | ZBTB24 | zinc finger and BTB domain containing 24 [Source:HGNC Symbol;Acc:HGNC:21143] |
| ENSG00000113108 | HGLibA_02385 | 49 | -0.691471687 | APBB3 | amyloid beta precursor protein binding family B member 3 [Source:HGNC Symbol;Acc:HGNC:20708] |
| ENSG00000113209 | HGLibA_35503 | 49 | -0.691471687 | PCDHB5 | protocadherin beta 5 [Source:HGNC Symbol;Acc:HGNC:8690] |
| ENSG00000116793 | HGLibA_36463 | 49 | -0.691471687 | PHTF1 | putative homeodomain transcription factor 1 [Source:HGNC Symbol;Acc:HGNC:8939] |
| ENSG00000117477 | HGLibA_07723 | 49 | -0.691471687 | CCDC181 | coiled-coil domain containing 181 [Source:HGNC Symbol;Acc:HGNC:28051] |
| ENSG00000118137 | HGLibA_02448 | 49 | -0.691471687 | APOA1 | apolipoprotein A1 [Source:HGNC Symbol;Acc:HGNC:600] |
| ENSG00000118518 | HGLibA_41536 | 49 | -0.691471687 | RNF146 | ring finger protein 146 [Source:HGNC Symbol;Acc:HGNC:21336] |
| ENSG00000119698 | HGLibA_38209 | 49 | -0.691471687 | PPP4R4 | protein phosphatase 4 regulatory subunit 4 [Source:HGNC Symbol;Acc:HGNC:23788] |
| ENSG00000119711 | HGLibA_01581 | 49 | -0.691471687 | ALDH6A1 | aldehyde dehydrogenase 6 family member A1 [Source:HGNC Symbol;Acc:HGNC:7179] |
| ENSG00000120647 | HGLibA_07868 | 49 | -0.691471687 | CCDC77 | coiled-coil domain containing 77 [Source:HGNC Symbol;Acc:HGNC:28203] |
| ENSG00000120925 | HGLibA_41573 | 49 | -0.691471687 | RNF170 | ring finger protein 170 [Source:HGNC Symbol;Acc:HGNC:25358] |
| ENSG00000121716 | HGLibA_36671 | 49 | -0.691471687 | PILRB | paired immunoglobin-like type 2 receptor beta [Source:HGNC Symbol;Acc:HGNC:18297] |
| ENSG00000124444 | HGLibA_56292 | 49 | -0.691471687 | ZNF576 | zinc finger protein 576 [Source:HGNC Symbol;Acc:HGNC:28357] |
| ENSG00000124939 | HGLibA_42924 | 49 | -0.691471687 | SCGB2A1 | secretoglobin family 2A member 1 [Source:HGNC Symbol;Acc:HGNC:7051] |
| ENSG00000125107 | HGLibA_10327 | 49 | -0.691471687 | CNOT1 | CCR4-NOT transcription complex subunit 1 [Source:HGNC Symbol;Acc:HGNC:7877] |
| ENSG00000125741 | HGLibA_33407 | 49 | -0.691471687 | OPA3 | outer mitochondrial membrane lipid metabolism regulator [Source:HGNC Symbol;Acc:HGNC:8142]" |
| ENSG00000126803 | HGLibA_22280 | 49 | -0.691471687 | HSPA2 | heat shock protein family A (Hsp70) member 2 [Source:HGNC Symbol;Acc:HGNC:5235] |
| ENSG00000126947 | HGLibA_03039 | 49 | -0.691471687 | ARMCX1 | armadillo repeat containing X-linked 1 [Source:HGNC Symbol;Acc:HGNC:18073] |
| ENSG00000128383 | HGLibA_02473 | 49 | -0.691471687 | APOBEC3A | apolipoprotein B mRNA editing enzyme catalytic subunit 3A [Source:HGNC Symbol;Acc:HGNC:17343] |
| ENSG00000128886 | HGLibA_14926 | 49 | -0.691471687 | ELL3 | elongation factor for RNA polymerase II 3 [Source:HGNC Symbol;Acc:HGNC:23113] |
| ENSG00000128989 | HGLibA_03098 | 49 | -0.691471687 | ARPP19 | cAMP regulated phosphoprotein 19 [Source:HGNC Symbol;Acc:HGNC:16967] |
| ENSG00000128989 | HGLibA_03097 | 49 | -0.691471687 | ARPP19 | cAMP regulated phosphoprotein 19 [Source:HGNC Symbol;Acc:HGNC:16967] |
| ENSG00000129422 | HGLibA_30504 | 49 | -0.691471687 | MTUS1 | microtubule associated scaffold protein 1 [Source:HGNC Symbol;Acc:HGNC:29789] |
| ENSG00000129910 | HGLibA_08645 | 49 | -0.691471687 | CDH15 | cadherin 15 [Source:HGNC Symbol;Acc:HGNC:1754] |
| ENSG00000130055 | HGLibA_18894 | 49 | -0.691471687 | GDPD2 | glycerophosphodiester phosphodiesterase domain containing 2 [Source:HGNC Symbol;Acc:HGNC:25974] |
| ENSG00000130592 | HGLibA_27564 | 49 | -0.691471687 | LSP1 | lymphocyte specific protein 1 [Source:HGNC Symbol;Acc:HGNC:6707] |
| ENSG00000131652 | HGLibA_49376 | 49 | -0.691471687 | THOC6 | THO complex 6 [Source:HGNC Symbol;Acc:HGNC:28369] |
| ENSG00000132432 | HGLibA_43256 | 49 | -0.691471687 | SEC61G | Sec61 translocon gamma subunit [Source:HGNC Symbol;Acc:HGNC:18277] |
| ENSG00000132773 | HGLibA_50939 | 49 | -0.691471687 | TOE1 | exonuclease [Source:HGNC Symbol;Acc:HGNC:15954]" |
| ENSG00000132819 | HGLibA_40538 | 49 | -0.691471687 | RBM38 | RNA binding motif protein 38 [Source:HGNC Symbol;Acc:HGNC:15818] |
| ENSG00000133863 | HGLibA_49066 | 49 | -0.691471687 | TEX15 | meiosis and synapsis associated [Source:HGNC Symbol;Acc:HGNC:11738]" |
| ENSG00000134240 | HGLibA_21715 | 49 | -0.691471687 | HMGCS2 | 3-hydroxy-3-methylglutaryl-CoA synthase 2 [Source:HGNC Symbol;Acc:HGNC:5008] |
| ENSG00000134250 | HGLibA_32343 | 49 | -0.691471687 | NOTCH2 | notch 2 [Source:HGNC Symbol;Acc:HGNC:7882] |
| ENSG00000134253 | HGLibA_51509 | 49 | -0.691471687 | TRIM45 | tripartite motif containing 45 [Source:HGNC Symbol;Acc:HGNC:19018] |
| ENSG00000134262 | HGLibA_02341 | 49 | -0.691471687 | AP4B1 | adaptor related protein complex 4 subunit beta 1 [Source:HGNC Symbol;Acc:HGNC:572] |
| ENSG00000135111 | HGLibA_48669 | 49 | -0.691471687 | TBX3 | T-box 3 [Source:HGNC Symbol;Acc:HGNC:11602] |
| ENSG00000135404 | HGLibA_08426 | 49 | -0.691471687 | CD63 | CD63 molecule [Source:HGNC Symbol;Acc:HGNC:1692] |
| ENSG00000135577 | HGLibA_32144 | 49 | -0.691471687 | NMBR | neuromedin B receptor [Source:HGNC Symbol;Acc:HGNC:7843] |
| ENSG00000135973 | HGLibA_20044 | 49 | -0.691471687 | GPR45 | G protein-coupled receptor 45 [Source:HGNC Symbol;Acc:HGNC:4503] |
| ENSG00000137656 | HGLibA_04997 | 49 | -0.691471687 | BUD13 | BUD13 homolog [Source:HGNC Symbol;Acc:HGNC:28199] |
| ENSG00000137699 | HGLibA_51452 | 49 | -0.691471687 | TRIM29 | tripartite motif containing 29 [Source:HGNC Symbol;Acc:HGNC:17274] |
| ENSG00000138035 | HGLibA_37433 | 49 | -0.691471687 | PNPT1 | polyribonucleotide nucleotidyltransferase 1 [Source:HGNC Symbol;Acc:HGNC:23166] |
| ENSG00000138380 | HGLibA_07243 | 49 | -0.691471687 | CARF | calcium responsive transcription factor [Source:HGNC Symbol;Acc:HGNC:14435] |
| ENSG00000138443 | HGLibA_00293 | 49 | -0.691471687 | ABI2 | abl interactor 2 [Source:HGNC Symbol;Acc:HGNC:24011] |
| ENSG00000138448 | HGLibA_23763 | 49 | -0.691471687 | ITGAV | integrin subunit alpha V [Source:HGNC Symbol;Acc:HGNC:6150] |
| ENSG00000138785 | HGLibA_23412 | 49 | -0.691471687 | INTS12 | integrator complex subunit 12 [Source:HGNC Symbol;Acc:HGNC:25067] |
| ENSG00000139173 | HGLibA_49888 | 49 | -0.691471687 | TMEM117 | transmembrane protein 117 [Source:HGNC Symbol;Acc:HGNC:25308] |
| ENSG00000139629 | HGLibA_18565 | 49 | -0.691471687 | GALNT6 | polypeptide N-acetylgalactosaminyltransferase 6 [Source:HGNC Symbol;Acc:HGNC:4128] |
| ENSG00000141068 | HGLibA_25864 | 49 | -0.691471687 | KSR1 | kinase suppressor of ras 1 [Source:HGNC Symbol;Acc:HGNC:6465] |
| ENSG00000142449 | HGLibA_16962 | 49 | -0.691471687 | FBN3 | fibrillin 3 [Source:HGNC Symbol;Acc:HGNC:18794] |
| ENSG00000142945 | HGLibA_24931 | 49 | -0.691471687 | KIF2C | kinesin family member 2C [Source:HGNC Symbol;Acc:HGNC:6393] |
| ENSG00000143369 | HGLibA_14413 | 49 | -0.691471687 | ECM1 | extracellular matrix protein 1 [Source:HGNC Symbol;Acc:HGNC:3153] |
| ENSG00000144118 | HGLibA_40151 | 49 | -0.691471687 | RALB | RAS like proto-oncogene B [Source:HGNC Symbol;Acc:HGNC:9840] |
| ENSG00000144354 | HGLibA_08613 | 49 | -0.691471687 | CDCA7 | cell division cycle associated 7 [Source:HGNC Symbol;Acc:HGNC:14628] |
| ENSG00000144460 | HGLibA_33183 | 49 | -0.691471687 | NYAP2 | neuronal tyrosine-phosphorylated phosphoinositide-3-kinase adaptor 2 [Source:HGNC Symbol;Acc:HGNC:29291] |
| ENSG00000144771 | HGLibA_27499 | 49 | -0.691471687 | LRTM1 | leucine rich repeats and transmembrane domains 1 [Source:HGNC Symbol;Acc:HGNC:25023] |
| ENSG00000145354 | HGLibA_09731 | 49 | -0.691471687 | CISD2 | CDGSH iron sulfur domain 2 [Source:HGNC Symbol;Acc:HGNC:24212] |
| ENSG00000146360 | HGLibA_20059 | 49 | -0.691471687 | GPR6 | G protein-coupled receptor 6 [Source:HGNC Symbol;Acc:HGNC:4515] |
| ENSG00000146904 | HGLibA_15284 | 49 | -0.691471687 | EPHA1 | EPH receptor A1 [Source:HGNC Symbol;Acc:HGNC:3385] |
| ENSG00000147364 | HGLibA_17053 | 49 | -0.691471687 | FBXO25 | F-box protein 25 [Source:HGNC Symbol;Acc:HGNC:13596] |
| ENSG00000147649 | HGLibA_30333 | 49 | -0.691471687 | MTDH | metadherin [Source:HGNC Symbol;Acc:HGNC:29608] |
| ENSG00000148143 | HGLibA_56040 | 49 | -0.691471687 | ZNF462 | zinc finger protein 462 [Source:HGNC Symbol;Acc:HGNC:21684] |
| ENSG00000148225 | HGLibA_54064 | 49 | -0.691471687 | WDR31 | WD repeat domain 31 [Source:HGNC Symbol;Acc:HGNC:21421] |
| ENSG00000149328 | HGLibA_19223 | 49 | -0.691471687 | GLB1L2 | galactosidase beta 1 like 2 [Source:HGNC Symbol;Acc:HGNC:25129] |
| ENSG00000149357 | HGLibA_25988 | 49 | -0.691471687 | LAMTOR1 | MAPK and MTOR activator 1 [Source:HGNC Symbol;Acc:HGNC:26068]" |
| ENSG00000149657 | HGLibA_27533 | 49 | -0.691471687 | LSM14B | LSM family member 14B [Source:HGNC Symbol;Acc:HGNC:15887] |
| ENSG00000151320 | HGLibA_01438 | 49 | -0.691471687 | AKAP6 | A-kinase anchoring protein 6 [Source:HGNC Symbol;Acc:HGNC:376] |
| ENSG00000151692 | HGLibA_41525 | 49 | -0.691471687 | RNF144A | ring finger protein 144A [Source:HGNC Symbol;Acc:HGNC:20457] |
| ENSG00000152700 | HGLibA_42740 | 49 | -0.691471687 | SAR1B | secretion associated Ras related GTPase 1B [Source:HGNC Symbol;Acc:HGNC:10535] |
| ENSG00000153037 | HGLibA_47002 | 49 | -0.691471687 | SRP19 | signal recognition particle 19 [Source:HGNC Symbol;Acc:HGNC:11300] |
| ENSG00000154240 | HGLibA_09099 | 49 | -0.691471687 | CEP112 | centrosomal protein 112 [Source:HGNC Symbol;Acc:HGNC:28514] |
| ENSG00000156509 | HGLibA_17103 | 49 | -0.691471687 | FBXO43 | F-box protein 43 [Source:HGNC Symbol;Acc:HGNC:28521] |
| ENSG00000156599 | HGLibA_55121 | 49 | -0.691471687 | ZDHHC5 | zinc finger DHHC-type containing 5 [Source:HGNC Symbol;Acc:HGNC:18472] |
| ENSG00000156650 | HGLibA_24062 | 49 | -0.691471687 | KAT6B | lysine acetyltransferase 6B [Source:HGNC Symbol;Acc:HGNC:17582] |
| ENSG00000157227 | HGLibA_29493 | 49 | -0.691471687 | MMP14 | matrix metallopeptidase 14 [Source:HGNC Symbol;Acc:HGNC:7160] |
| ENSG00000157933 | HGLibA_44275 | 49 | -0.691471687 | SKI | SKI proto-oncogene [Source:HGNC Symbol;Acc:HGNC:10896] |
| ENSG00000158473 | HGLibA_08274 | 49 | -0.691471687 | CD1D | CD1d molecule [Source:HGNC Symbol;Acc:HGNC:1637] |
| ENSG00000158747 | HGLibA_31216 | 49 | -0.691471687 | NBL1 | DAN family BMP antagonist [Source:HGNC Symbol;Acc:HGNC:7650]" |
| ENSG00000158773 | HGLibA_53168 | 49 | -0.691471687 | USF1 | upstream transcription factor 1 [Source:HGNC Symbol;Acc:HGNC:12593] |
| ENSG00000159556 | HGLibA_23666 | 49 | -0.691471687 | ISL2 | ISL LIM homeobox 2 [Source:HGNC Symbol;Acc:HGNC:18524] |
| ENSG00000159685 | HGLibA_09376 | 49 | -0.691471687 | CHCHD6 | coiled-coil-helix-coiled-coil-helix domain containing 6 [Source:HGNC Symbol;Acc:HGNC:28184] |
| ENSG00000160446 | HGLibA_55071 | 49 | -0.691471687 | ZDHHC12 | zinc finger DHHC-type containing 12 [Source:HGNC Symbol;Acc:HGNC:19159] |
| ENSG00000160678 | HGLibA_42530 | 49 | -0.691471687 | S100A1 | S100 calcium binding protein A1 [Source:HGNC Symbol;Acc:HGNC:10486] |
| ENSG00000160741 | HGLibA_11231 | 49 | -0.691471687 | CRTC2 | CREB regulated transcription coactivator 2 [Source:HGNC Symbol;Acc:HGNC:27301] |
| ENSG00000160808 | HGLibA_30761 | 49 | -0.691471687 | MYL3 | myosin light chain 3 [Source:HGNC Symbol;Acc:HGNC:7584] |
| ENSG00000161036 | HGLibA_27509 | 49 | -0.691471687 | LRWD1 | leucine rich repeats and WD repeat domain containing 1 [Source:HGNC Symbol;Acc:HGNC:21769] |
| ENSG00000161526 | HGLibA_42726 | 49 | -0.691471687 | SAP30BP | SAP30 binding protein [Source:HGNC Symbol;Acc:HGNC:30785] |
| ENSG00000162595 | HGLibA_13263 | 49 | -0.691471687 | DIRAS3 | DIRAS family GTPase 3 [Source:HGNC Symbol;Acc:HGNC:687] |
| ENSG00000162723 | HGLibA_44323 | 49 | -0.691471687 | SLAMF9 | SLAM family member 9 [Source:HGNC Symbol;Acc:HGNC:18430] |
| ENSG00000162736 | HGLibA_31377 | 49 | -0.691471687 | NCSTN | nicastrin [Source:HGNC Symbol;Acc:HGNC:17091] |
| ENSG00000163002 | HGLibA_33047 | 49 | -0.691471687 | NUP35 | nucleoporin 35 [Source:HGNC Symbol;Acc:HGNC:29797] |
| ENSG00000163081 | HGLibA_07616 | 49 | -0.691471687 | CCDC140 | coiled-coil domain containing 140 [Source:HGNC Symbol;Acc:HGNC:26514] |
| ENSG00000163159 | HGLibA_53814 | 49 | -0.691471687 | VPS72 | vacuolar protein sorting 72 homolog [Source:HGNC Symbol;Acc:HGNC:11644] |
| ENSG00000163312 | HGLibA_21116 | 49 | -0.691471687 | HELQ | POLQ like [Source:HGNC Symbol;Acc:HGNC:18536]" |
| ENSG00000163430 | HGLibA_12530 | 49 | -0.691471687 | DCST1 | DC-STAMP domain containing 1 [Source:HGNC Symbol;Acc:HGNC:26539] |
| ENSG00000163435 | HGLibA_14898 | 49 | -0.691471687 | ELF3 | E74 like ETS transcription factor 3 [Source:HGNC Symbol;Acc:HGNC:3318] |
| ENSG00000163453 | HGLibA_22834 | 49 | -0.691471687 | IGFBP7 | insulin like growth factor binding protein 7 [Source:HGNC Symbol;Acc:HGNC:5476] |
| ENSG00000163479 | HGLibA_47156 | 49 | -0.691471687 | SSR2 | signal sequence receptor subunit 2 [Source:HGNC Symbol;Acc:HGNC:11324] |
| ENSG00000163879 | HGLibA_13651 | 49 | -0.691471687 | DNALI1 | dynein axonemal light intermediate chain 1 [Source:HGNC Symbol;Acc:HGNC:14353] |
| ENSG00000163960 | HGLibA_52795 | 49 | -0.691471687 | UBXN7 | UBX domain protein 7 [Source:HGNC Symbol;Acc:HGNC:29119] |
| ENSG00000164104 | HGLibA_21694 | 49 | -0.691471687 | HMGB2 | high mobility group box 2 [Source:HGNC Symbol;Acc:HGNC:5000] |
| ENSG00000164129 | HGLibA_32559 | 49 | -0.691471687 | NPY5R | neuropeptide Y receptor Y5 [Source:HGNC Symbol;Acc:HGNC:7958] |
| ENSG00000164171 | HGLibA_23723 | 49 | -0.691471687 | ITGA2 | integrin subunit alpha 2 [Source:HGNC Symbol;Acc:HGNC:6137] |
| ENSG00000164615 | HGLibA_07101 | 49 | -0.691471687 | CAMLG | calcium modulating ligand [Source:HGNC Symbol;Acc:HGNC:1471] |
| ENSG00000165060 | HGLibA_18215 | 49 | -0.691471687 | FXN | frataxin [Source:HGNC Symbol;Acc:HGNC:3951] |
| ENSG00000167118 | HGLibA_53148 | 49 | -0.691471687 | URM1 | ubiquitin related modifier 1 [Source:HGNC Symbol;Acc:HGNC:28378] |
| ENSG00000167965 | HGLibA_29436 | 49 | -0.691471687 | MLST8 | LST8 homolog [Source:HGNC Symbol;Acc:HGNC:24825]" |
| ENSG00000167968 | HGLibA_13659 | 49 | -0.691471687 | DNASE1L2 | deoxyribonuclease 1 like 2 [Source:HGNC Symbol;Acc:HGNC:2958] |
| ENSG00000167985 | HGLibA_43140 | 49 | -0.691471687 | SDHAF2 | succinate dehydrogenase complex assembly factor 2 [Source:HGNC Symbol;Acc:HGNC:26034] |
| ENSG00000168143 | HGLibA_16743 | 49 | -0.691471687 | FAM83B | family with sequence similarity 83 member B [Source:HGNC Symbol;Acc:HGNC:21357] |
| ENSG00000168806 | HGLibA_26162 | 49 | -0.691471687 | LCMT2 | leucine carboxyl methyltransferase 2 [Source:HGNC Symbol;Acc:HGNC:17558] |
| ENSG00000168917 | HGLibA_45020 | 49 | -0.691471687 | SLC35G2 | solute carrier family 35 member G2 [Source:HGNC Symbol;Acc:HGNC:28480] |
| ENSG00000169682 | HGLibA_46734 | 49 | -0.691471687 | SPNS1 | sphingolipid transporter 1 (putative) [Source:HGNC Symbol;Acc:HGNC:30621] |
| ENSG00000169914 | HGLibA_34763 | 49 | -0.691471687 | OTUD3 | OTU deubiquitinase 3 [Source:HGNC Symbol;Acc:HGNC:29038] |
| ENSG00000170454 | HGLibA_25538 | 49 | -0.691471687 | KRT75 | keratin 75 [Source:HGNC Symbol;Acc:HGNC:24431] |
| ENSG00000171368 | HGLibA_51165 | 49 | -0.691471687 | TPPP | tubulin polymerization promoting protein [Source:HGNC Symbol;Acc:HGNC:24164] |

| | | | | | |
|---|---|---|---|---|---|
| ENSG00000171396 | HGLibA_25764 | 49 | -0.691471687 | KRTAP4-4 | keratin associated protein 4-4 [Source:HGNC Symbol;Acc:HGNC:16928] |
| ENSG00000171944 | HGLibA_34143 | 49 | -0.691471687 | OR52A5 | olfactory receptor family 52 subfamily A member 5 [Source:HGNC Symbol;Acc:HGNC:19580] |
| ENSG00000172296 | HGLibA_46898 | 49 | -0.691471687 | SPTLC3 | serine palmitoyltransferase long chain base subunit 3 [Source:HGNC Symbol;Acc:HGNC:16253] |
| ENSG00000172362 | HGLibA_34257 | 49 | -0.691471687 | OR5B12 | olfactory receptor family 5 subfamily B member 12 [Source:HGNC Symbol;Acc:HGNC:15432] |
| ENSG00000173545 | HGLibA_56410 | 49 | -0.691471687 | ZNF622 | zinc finger protein 622 [Source:HGNC Symbol;Acc:HGNC:30958] |
| ENSG00000174804 | HGLibA_18272 | 49 | -0.691471687 | FZD4 | frizzled class receptor 4 [Source:HGNC Symbol;Acc:HGNC:4042] |
| ENSG00000174844 | HGLibA_13477 | 49 | -0.691471687 | DNAH12 | dynein axonemal heavy chain 12 [Source:HGNC Symbol;Acc:HGNC:2943] |
| ENSG00000174891 | HGLibA_42359 | 49 | -0.691471687 | RSRC1 | arginine and serine rich coiled-coil 1 [Source:HGNC Symbol;Acc:HGNC:24152] |
| ENSG00000175906 | HGLibA_02971 | 49 | -0.691471687 | ARL4D | ADP ribosylation factor like GTPase 4D [Source:HGNC Symbol;Acc:HGNC:656] |
| ENSG00000176444 | HGLibA_10056 | 49 | -0.691471687 | CLK2 | CDC like kinase 2 [Source:HGNC Symbol;Acc:HGNC:2069] |
| ENSG00000177084 | HGLibA_37511 | 49 | -0.691471687 | POLE | catalytic subunit [Source:HGNC Symbol;Acc:HGNC:9177]" |
| ENSG00000178878 | HGLibA_02545 | 49 | -0.691471687 | APOLD1 | apolipoprotein L domain containing 1 [Source:HGNC Symbol;Acc:HGNC:25268] |
| ENSG00000180263 | HGLibA_17358 | 49 | -0.691471687 | FGD6 | RhoGEF and PH domain containing 6 [Source:HGNC Symbol;Acc:HGNC:21740]" |
| ENSG00000181631 | HGLibA_34880 | 49 | -0.691471687 | P2RY13 | purinergic receptor P2Y13 [Source:HGNC Symbol;Acc:HGNC:4537] |
| ENSG00000182447 | HGLibA_34733 | 49 | -0.691471687 | OTOL1 | otolin 1 [Source:HGNC Symbol;Acc:HGNC:34071] |
| ENSG00000182698 | HGLibA_40866 | 49 | -0.691471687 | RESP18 | regulated endocrine specific protein 18 [Source:HGNC Symbol;Acc:HGNC:33762] |
| ENSG00000182827 | HGLibA_00399 | 49 | -0.691471687 | ACBD3 | acyl-CoA binding domain containing 3 [Source:HGNC Symbol;Acc:HGNC:15453] |
| ENSG00000183479 | HGLibA_51375 | 49 | -0.691471687 | TREX2 | three prime repair exonuclease 2 [Source:HGNC Symbol;Acc:HGNC:12270] |
| ENSG00000183617 | HGLibA_30021 | 49 | -0.691471687 | MRPL54 | mitochondrial ribosomal protein L54 [Source:HGNC Symbol;Acc:HGNC:16685] |
| ENSG00000183747 | HGLibA_00573 | 49 | -0.691471687 | ACSM2A | acyl-CoA synthetase medium chain family member 2A [Source:HGNC Symbol;Acc:HGNC:32017] |
| ENSG00000184032 | HGLibA_25703 | 49 | -0.691471687 | KRTAP20-2 | keratin associated protein 20-2 [Source:HGNC Symbol;Acc:HGNC:18944] |
| ENSG00000184033 | HGLibA_11561 | 49 | -0.691471687 | CTAG1B | cancer/testis antigen 1B [Source:HGNC Symbol;Acc:HGNC:2491] |
| ENSG00000184166 | HGLibA_33654 | 49 | -0.691471687 | OR1D2 | olfactory receptor family 1 subfamily D member 2 [Source:HGNC Symbol;Acc:HGNC:8183] |
| ENSG00000184220 | HGLibA_10206 | 49 | -0.691471687 | CMSS1 | cms1 ribosomal small subunit homolog [Source:HGNC Symbol;Acc:HGNC:28666] |
| ENSG00000184351 | HGLibA_25663 | 49 | -0.691471687 | KRTAP19-1 | keratin associated protein 19-1 [Source:HGNC Symbol;Acc:HGNC:18936] |
| ENSG00000184465 | HGLibA_54059 | 49 | -0.691471687 | WDR27 | WD repeat domain 27 [Source:HGNC Symbol;Acc:HGNC:21248] |
| ENSG00000184489 | HGLibA_39408 | 49 | -0.691471687 | PTP4A3 | member 3 [Source:HGNC Symbol;Acc:HGNC:9636]" |
| ENSG00000184967 | HGLibA_32241 | 49 | -0.691471687 | NOC4L | nucleolar complex associated 4 homolog [Source:HGNC Symbol;Acc:HGNC:28461] |
| ENSG00000185189 | HGLibA_32658 | 49 | -0.691471687 | NRBP2 | nuclear receptor binding protein 2 [Source:HGNC Symbol;Acc:HGNC:19339] |
| ENSG00000185761 | HGLibA_00861 | 49 | -0.691471687 | ADAMTSL5 | ADAMTS like 5 [Source:HGNC Symbol;Acc:HGNC:27912] |
| ENSG00000186281 | HGLibA_19732 | 49 | -0.691471687 | GPAT2 | mitochondrial [Source:HGNC Symbol;Acc:HGNC:27168]" |
| ENSG00000186496 | HGLibA_55914 | 49 | -0.691471687 | ZNF396 | zinc finger protein 396 [Source:HGNC Symbol;Acc:HGNC:18824] |
| ENSG00000187672 | HGLibA_15428 | 49 | -0.691471687 | ERC2 | ELKS/RAB6-interacting/CAST family member 2 [Source:HGNC Symbol;Acc:HGNC:31922] |
| ENSG00000187778 | HGLibA_28649 | 49 | -0.691471687 | MCRS1 | microspherule protein 1 [Source:HGNC Symbol;Acc:HGNC:6960] |
| ENSG00000188157 | HGLibA_01249 | 49 | -0.691471687 | AGRN | agrin [Source:HGNC Symbol;Acc:HGNC:329] |
| ENSG00000189001 | HGLibA_42811 | 49 | -0.691471687 | SBSN | suprabasin [Source:HGNC Symbol;Acc:HGNC:24950] |
| ENSG00000189050 | HGLibA_41702 | 49 | -0.691471687 | RNFT1 | transmembrane 1 [Source:HGNC Symbol;Acc:HGNC:30206]" |
| ENSG00000196453 | HGLibA_56724 | 49 | -0.691471687 | ZNF777 | zinc finger protein 777 [Source:HGNC Symbol;Acc:HGNC:22213] |
| ENSG00000196468 | HGLibA_17377 | 49 | -0.691471687 | FGF16 | fibroblast growth factor 16 [Source:HGNC Symbol;Acc:HGNC:3672] |
| ENSG00000196776 | HGLibA_08397 | 49 | -0.691471687 | CD47 | CD47 molecule [Source:HGNC Symbol;Acc:HGNC:1682] |
| ENSG00000196976 | HGLibA_25930 | 49 | -0.691471687 | LAGE3 | L antigen family member 3 [Source:HGNC Symbol;Acc:HGNC:26058] |
| ENSG00000198471 | HGLibA_42426 | 49 | -0.691471687 | RTP2 | receptor transporter protein 2 [Source:HGNC Symbol;Acc:HGNC:32486] |
| ENSG00000198780 | HGLibA_16252 | 49 | -0.691471687 | FAM169A | family with sequence similarity 169 member A [Source:HGNC Symbol;Acc:HGNC:29138] |
| ENSG00000198874 | HGLibA_52480 | 49 | -0.691471687 | TYW1 | tRNA-yW synthesizing protein 1 homolog [Source:HGNC Symbol;Acc:HGNC:25598] |
| ENSG00000204315 | HGLibA_17594 | 49 | -0.691471687 | FKBPL | FK506 binding protein like [Source:HGNC Symbol;Acc:HGNC:13949] |
| ENSG00000204632 | HGLibA_21647 | 49 | -0.691471687 | HLA-G | G [Source:HGNC Symbol;Acc:HGNC:4964]" |
| ENSG00000204640 | HGLibA_32202 | 49 | -0.691471687 | NMS | neuromedin S [Source:HGNC Symbol;Acc:HGNC:32203] |
| ENSG00000205022 | HGLibA_34946 | 49 | -0.691471687 | PABPN1L | cytoplasmic [Source:HGNC Symbol;Acc:HGNC:37237]" |
| ENSG00000205497 | HGLibA_34075 | 49 | -0.691471687 | OR51A4 | olfactory receptor family 51 subfamily A member 4 [Source:HGNC Symbol;Acc:HGNC:14795] |
| ENSG00000205549 | HGLibA_06779 | 49 | -0.691471687 | C9orf92 | chromosome 9 open reading frame 92 [Source:HGNC Symbol;Acc:HGNC:19054] |
| ENSG00000205659 | HGLibA_26567 | 49 | -0.691471687 | LIN52 | lin-52 DREAM MuvB core complex component [Source:HGNC Symbol;Acc:HGNC:19856] |
| ENSG00000205882 | HGLibA_12904 | 49 | -0.691471687 | DEFB134 | defensin beta 134 [Source:HGNC Symbol;Acc:HGNC:32399] |
| ENSG00000213762 | HGLibA_55467 | 49 | -0.691471687 | ZNF134 | zinc finger protein 134 [Source:HGNC Symbol;Acc:HGNC:12918] |
| ENSG00000214042 | HGLibA_22690 | 49 | -0.691471687 | IFNA7 | interferon alpha 7 [Source:HGNC Symbol;Acc:HGNC:5428] |
| ENSG00000214402 | HGLibA_26190 | 49 | -0.691471687 | LCNL1 | lipocalin like 1 [Source:HGNC Symbol;Acc:HGNC:34436] |
| ENSG00000215717 | HGLibA_50042 | 49 | -0.691471687 | TMEM167B | transmembrane protein 167B [Source:HGNC Symbol;Acc:HGNC:30187] |
| ENSG00000229544 | HGLibA_32028 | 49 | -0.691471687 | NKX1-2 | NK1 homeobox 2 [Source:HGNC Symbol;Acc:HGNC:31652] |
| ENSG00000230989 | HGLibA_22150 | 49 | -0.691471687 | HSBP1 | heat shock factor binding protein 1 [Source:HGNC Symbol;Acc:HGNC:5203] |
| ENSG00000236637 | HGLibA_22679 | 49 | -0.691471687 | IFNA4 | interferon alpha 4 [Source:HGNC Symbol;Acc:HGNC:5425] |
| ENSG00000239672 | HGLibA_32150 | 49 | -0.691471687 | NME1 | NME/NM23 nucleoside diphosphate kinase 1 [Source:HGNC Symbol;Acc:HGNC:7849] |
| ENSG00000240403 | HGLibA_24993 | 49 | -0.691471687 | KIR3DL2 | three Ig domains and long cytoplasmic tail 2 [Source:HGNC Symbol;Acc:HGNC:6339]" |
| ENSG00000247746 | HGLibA_53396 | 49 | -0.691471687 | USP51 | ubiquitin specific peptidase 51 [Source:HGNC Symbol;Acc:HGNC:23086] |
| ENSG00000251258 | HGLibA_40945 | 49 | -0.691471687 | RFPL4B | ret finger protein like 4B [Source:HGNC Symbol;Acc:HGNC:33264] |
| ENSG00000259571 | HGLibA_04541 | 49 | -0.691471687 | BLID | cell death inducer [Source:HGNC Symbol;Acc:HGNC:33495]" |
| ENSG00000260001 | HGLibA_49233 | 49 | -0.691471687 | TGFBR3L | transforming growth factor beta receptor 3 like [Source:HGNC Symbol;Acc:HGNC:44152] |
| ENSG00000260458 | HGLibA_24270 | 49 | -0.691471687 | KCNJ18 | potassium voltage-gated channel subfamily J member 18 [Source:HGNC Symbol;Acc:HGNC:39080] |
| ENSG00000268606 | HGLibA_27906 | 49 | -0.691471687 | MAGEA2 | MAGE family member A2 [Source:HGNC Symbol;Acc:HGNC:6800] |
| ENSG00000272573 | HGLibA_30579 | 49 | -0.691471687 | MUSTN1 | embryonic nuclear protein 1 [Source:HGNC Symbol;Acc:HGNC:22144]" |
| ENSG00000276043 | HGLibA_52946 | 49 | -0.691471687 | UHRF1 | ubiquitin like with PHD and ring finger domains 1 [Source:HGNC Symbol;Acc:HGNC:12556] |
| ENSG00000284723 | HGLibA_34553 | 49 | -0.691471687 | OR8S1 | olfactory receptor family 8 subfamily S member 1 [Source:HGNC Symbol;Acc:HGNC:19628] |
| ENSG00000288722 | HGLibA_15887 | 49 | -0.691471687 | F8A1 | - |
| EPB49 | HGLibA_15263 | 49 | -0.691471687 | EPB49 | - |
| FAM198B | HGLibA_16389 | 49 | -0.691471687 | FAM198B | - |
| GLTSCR1L | HGLibA_19354 | 49 | -0.691471687 | GLTSCR1L | - |
| GTPBP6_Y | HGLibA_20619 | 49 | -0.691471687 | GTPBP6_Y | - |
| HIST1H2BE | HGLibA_21424 | 49 | -0.691471687 | HIST1H2BE | - |
| HIST1H2BF | HGLibA_21426 | 49 | -0.691471687 | HIST1H2BF | - |
| hsa-mir-1282 | HGLibA_57584 | 49 | -0.691471687 | hsa-mir-1282 | - |
| hsa-mir-135a-2 | HGLibA_57797 | 49 | -0.691471687 | hsa-mir-135a-2 | - |
| hsa-mir-198 | HGLibA_58123 | 49 | -0.691471687 | hsa-mir-198 | - |
| hsa-mir-206 | HGLibA_58192 | 49 | -0.691471687 | hsa-mir-206 | - |
| hsa-mir-23b | HGLibA_58333 | 49 | -0.691471687 | hsa-mir-23b | - |
| hsa-mir-24-2 | HGLibA_58343 | 49 | -0.691471687 | hsa-mir-24-2 | - |
| hsa-mir-302c | HGLibA_58443 | 49 | -0.691471687 | hsa-mir-302c | - |
| hsa-mir-3158-1 | HGLibA_58703 | 49 | -0.691471687 | hsa-mir-3158-1 | - |
| hsa-mir-33a | HGLibA_59012 | 49 | -0.691471687 | hsa-mir-33a | - |
| hsa-mir-3614 | HGLibA_59086 | 49 | -0.691471687 | hsa-mir-3614 | - |
| hsa-mir-376b | HGLibA_59414 | 49 | -0.691471687 | hsa-mir-376b | - |
| hsa-mir-3909 | HGLibA_59501 | 49 | -0.691471687 | hsa-mir-3909 | - |
| hsa-mir-4490 | HGLibA_60377 | 49 | -0.691471687 | hsa-mir-4490 | - |
| hsa-mir-4504 | HGLibA_60441 | 49 | -0.691471687 | hsa-mir-4504 | - |
| hsa-mir-4639 | HGLibA_60661 | 49 | -0.691471687 | hsa-mir-4639 | - |
| hsa-mir-4646 | HGLibA_60686 | 49 | -0.691471687 | hsa-mir-4646 | - |
| hsa-mir-4671 | HGLibA_60801 | 49 | -0.691471687 | hsa-mir-4671 | - |
| hsa-mir-4673 | HGLibA_60809 | 49 | -0.691471687 | hsa-mir-4673 | - |
| hsa-mir-4741 | HGLibA_61063 | 49 | -0.691471687 | hsa-mir-4741 | - |
| hsa-mir-519b | HGLibA_61720 | 49 | -0.691471687 | hsa-mir-519b | - |
| hsa-mir-5701-2 | HGLibA_62343 | 49 | -0.691471687 | hsa-mir-5701-2 | - |
| hsa-mir-581 | HGLibA_62422 | 49 | -0.691471687 | hsa-mir-581 | - |
| hsa-mir-616 | HGLibA_62699 | 49 | -0.691471687 | hsa-mir-616 | - |
| hsa-mir-633 | HGLibA_62770 | 49 | -0.691471687 | hsa-mir-633 | - |
| hsa-mir-649 | HGLibA_62838 | 49 | -0.691471687 | hsa-mir-649 | - |
| hsa-mir-6753 | HGLibA_63170 | 49 | -0.691471687 | hsa-mir-6753 | - |
| hsa-mir-760 | HGLibA_63900 | 49 | -0.691471687 | hsa-mir-760 | - |
| hsa-mir-8062 | HGLibA_64086 | 49 | -0.691471687 | hsa-mir-8062 | - |
| hsa-mir-944 | HGLibA_64361 | 49 | -0.691471687 | hsa-mir-944 | - |
| LOC100129924 | HGLibA_26772 | 49 | -0.691471687 | LOC100129924 | - |
| LOC100289561 | HGLibA_26844 | 49 | -0.691471687 | LOC100289561 | - |
| LOC642778 | HGLibA_26956 | 49 | -0.691471687 | LOC642778 | - |
| SETD8 | HGLibA_43661 | 49 | -0.691471687 | SETD8 | - |
| SLC25A6_X | HGLibA_44773 | 49 | -0.691471687 | SLC25A6_X | - |
| ASMT_X | HGLibA_03321 | 48 | -0.70051703 | ASMT_X | - |
| ASNA1 | HGLibA_03332 | 48 | -0.70051703 | ASNA1 | - |
| C12orf44 | HGLibA_05247 | 48 | -0.70051703 | C12orf44 | - |
| C4orf29 | HGLibA_06314 | 48 | -0.70051703 | C4orf29 | - |
| CXorf56 | HGLibA_11945 | 48 | -0.70051703 | CXorf56 | - |
| ENSG00000004059 | HGLibA_02656 | 48 | -0.70051703 | ARF5 | ADP ribosylation factor 5 [Source:HGNC Symbol;Acc:HGNC:658] |
| ENSG00000010318 | HGLibA_36386 | 48 | -0.70051703 | PHF7 | PHD finger protein 7 [Source:HGNC Symbol;Acc:HGNC:18458] |
| ENSG00000013588 | HGLibA_20124 | 48 | -0.70051703 | GPRC5A | G protein-coupled receptor class C group 5 member A [Source:HGNC Symbol;Acc:HGNC:9836] |
| ENSG00000016082 | HGLibA_23664 | 48 | -0.70051703 | ISL1 | ISL LIM homeobox 1 [Source:HGNC Symbol;Acc:HGNC:6132] |
| ENSG00000023191 | HGLibA_41712 | 48 | -0.70051703 | RNH1 | ribonuclease/angiogenin inhibitor 1 [Source:HGNC Symbol;Acc:HGNC:10074] |
| ENSG00000026297 | HGLibA_41441 | 48 | -0.70051703 | RNASET2 | ribonuclease T2 [Source:HGNC Symbol;Acc:HGNC:21686] |
| ENSG00000035928 | HGLibA_40901 | 48 | -0.70051703 | RFC1 | replication factor C subunit 1 [Source:HGNC Symbol;Acc:HGNC:9969] |
| ENSG00000041802 | HGLibA_27514 | 48 | -0.70051703 | LSG1 | large 60S subunit nuclear export GTPase 1 [Source:HGNC Symbol;Acc:HGNC:25652] |
| ENSG00000048828 | HGLibA_16054 | 48 | -0.70051703 | FAM120A | family with sequence similarity 120A [Source:HGNC Symbol;Acc:HGNC:13247] |
| ENSG00000054654 | HGLibA_47978 | 48 | -0.70051703 | SYNE2 | spectrin repeat containing nuclear envelope protein 2 [Source:HGNC Symbol;Acc:HGNC:17084] |
| ENSG00000055483 | HGLibA_53348 | 48 | -0.70051703 | USP36 | ubiquitin specific peptidase 36 [Source:HGNC Symbol;Acc:HGNC:20062] |
| ENSG00000065978 | HGLibA_54642 | 48 | -0.70051703 | YBX1 | Y-box binding protein 1 [Source:HGNC Symbol;Acc:HGNC:8014] |
| ENSG00000066135 | HGLibA_24507 | 48 | -0.70051703 | KDM4A | lysine demethylase 4A [Source:HGNC Symbol;Acc:HGNC:22978] |
| ENSG00000066583 | HGLibA_23682 | 48 | -0.70051703 | ISOC1 | isochorismatase domain containing 1 [Source:HGNC Symbol;Acc:HGNC:24254] |
| ENSG00000067057 | HGLibA_36172 | 48 | -0.70051703 | PFKP | platelet [Source:HGNC Symbol;Acc:HGNC:8878]" |
| ENSG00000068305 | HGLibA_28826 | 48 | -0.70051703 | MEF2A | myocyte enhancer factor 2A [Source:HGNC Symbol;Acc:HGNC:6993] |
| ENSG00000070950 | HGLibA_40060 | 48 | -0.70051703 | RAD18 | E3 ubiquitin protein ligase [Source:HGNC Symbol;Acc:HGNC:18278]" |
| ENSG00000071677 | HGLibA_38598 | 48 | -0.70051703 | PRLH | prolactin releasing hormone [Source:HGNC Symbol;Acc:HGNC:17945] |
| ENSG00000072274 | HGLibA_49197 | 48 | -0.70051703 | TFRC | transferrin receptor [Source:HGNC Symbol;Acc:HGNC:11763] |

| | | | | | |
|---|---|---|---|---|---|
| ENSG00000074771 | HGLibA_32374 | 48 | -0.70051703 | NOX3 | NADPH oxidase 3 [Source:HGNC Symbol;Acc:HGNC:7890] |
| ENSG00000079150 | HGLibA_17584 | 48 | -0.70051703 | FKBP7 | FK506 binding protein 7 [Source:HGNC Symbol;Acc:HGNC:3723] |
| ENSG00000080618 | HGLibA_10909 | 48 | -0.70051703 | CPB2 | carboxypeptidase B2 [Source:HGNC Symbol;Acc:HGNC:2300] |
| ENSG00000080824 | HGLibA_22250 | 48 | -0.70051703 | HSP90AA1 | heat shock protein 90 alpha family class A member 1 [Source:HGNC Symbol;Acc:HGNC:5253] |
| ENSG00000081665 | HGLibA_56119 | 48 | -0.70051703 | ZNF506 | zinc finger protein 506 [Source:HGNC Symbol;Acc:HGNC:23780] |
| ENSG00000084112 | HGLibA_47132 | 48 | -0.70051703 | SSH1 | slingshot protein phosphatase 1 [Source:HGNC Symbol;Acc:HGNC:30579] |
| ENSG00000084754 | HGLibA_20807 | 48 | -0.70051703 | HADHA | hydroxyacyl-CoA dehydrogenase trifunctional multienzyme complex subunit alpha [Source:HGNC Symbol;Acc:HGNC:4801] |
| ENSG00000089053 | HGLibA_01885 | 48 | -0.70051703 | ANAPC5 | anaphase promoting complex subunit 5 [Source:HGNC Symbol;Acc:HGNC:15713] |
| ENSG00000092820 | HGLibA_15839 | 48 | -0.70051703 | EZR | ezrin [Source:HGNC Symbol;Acc:HGNC:12691] |
| ENSG00000096264 | HGLibA_31365 | 48 | -0.70051703 | NCR2 | natural cytotoxicity triggering receptor 2 [Source:HGNC Symbol;Acc:HGNC:6732] |
| ENSG00000099377 | HGLibA_22211 | 48 | -0.70051703 | HSD3B7 | 3 beta- and steroid delta-isomerase 7 [Source:HGNC Symbol;Acc:HGNC:18324]" |
| ENSG00000099715 | HGLibA_35395 | 48 | -0.70051703 | PCDH11Y | protocadherin 11 Y-linked [Source:HGNC Symbol;Acc:HGNC:15813] |
| ENSG00000100299 | HGLibA_03128 | 48 | -0.70051703 | ARSA | arylsulfatase A [Source:HGNC Symbol;Acc:HGNC:713] |
| ENSG00000101955 | HGLibA_47032 | 48 | -0.70051703 | SRPX | sushi repeat containing protein X-linked [Source:HGNC Symbol;Acc:HGNC:11309] |
| ENSG00000102781 | HGLibA_24074 | 48 | -0.70051703 | KATNAL1 | katanin catalytic subunit A1 like 1 [Source:HGNC Symbol;Acc:HGNC:28361] |
| ENSG00000103266 | HGLibA_47611 | 48 | -0.70051703 | STUB1 | STIP1 homology and U-box containing protein 1 [Source:HGNC Symbol;Acc:HGNC:11427] |
| ENSG00000104738 | HGLibA_28614 | 48 | -0.70051703 | MCM4 | minichromosome maintenance complex component 4 [Source:HGNC Symbol;Acc:HGNC:6947] |
| ENSG00000104818 | HGLibA_09305 | 48 | -0.70051703 | CGB2 | chorionic gonadotropin subunit beta 2 [Source:HGNC Symbol;Acc:HGNC:16722] |
| ENSG00000104883 | HGLibA_36090 | 48 | -0.70051703 | PEX11G | peroxisomal biogenesis factor 11 gamma [Source:HGNC Symbol;Acc:HGNC:20208] |
| ENSG00000104953 | HGLibA_49619 | 48 | -0.70051703 | TLE6 | transducin like enhancer of split 6 [Source:HGNC Symbol;Acc:HGNC:30788] |
| ENSG00000105137 | HGLibA_47940 | 48 | -0.70051703 | SYDE1 | synapse defective Rho GTPase homolog 1 [Source:HGNC Symbol;Acc:HGNC:25824] |
| ENSG00000105379 | HGLibA_15623 | 48 | -0.70051703 | ETFB | electron transfer flavoprotein subunit beta [Source:HGNC Symbol;Acc:HGNC:3482] |
| ENSG00000106128 | HGLibA_19046 | 48 | -0.70051703 | GHRHR | growth hormone releasing hormone receptor [Source:HGNC Symbol;Acc:HGNC:4266] |
| ENSG00000106153 | HGLibA_09364 | 48 | -0.70051703 | CHCHD2 | coiled-coil-helix-coiled-coil-helix domain containing 2 [Source:HGNC Symbol;Acc:HGNC:21645] |
| ENSG00000106636 | HGLibA_54695 | 48 | -0.70051703 | YKT6 | YKT6 v-SNARE homolog [Source:HGNC Symbol;Acc:HGNC:16959] |
| ENSG00000107186 | HGLibA_29714 | 48 | -0.70051703 | MPDZ | multiple PDZ domain crumbs cell polarity complex component [Source:HGNC Symbol;Acc:HGNC:7208] |
| ENSG00000107625 | HGLibA_12732 | 48 | -0.70051703 | DDX50 | DExD-box helicase 50 [Source:HGNC Symbol;Acc:HGNC:17906] |
| ENSG00000107902 | HGLibA_26443 | 48 | -0.70051703 | LHPP | phospholysine phosphohistidine inorganic pyrophosphate phosphatase [Source:HGNC Symbol;Acc:HGNC:30042] |
| ENSG00000109061 | HGLibA_30702 | 48 | -0.70051703 | MYH1 | myosin heavy chain 1 [Source:HGNC Symbol;Acc:HGNC:7567] |
| ENSG00000109466 | HGLibA_25146 | 48 | -0.70051703 | KLHL2 | kelch like family member 2 [Source:HGNC Symbol;Acc:HGNC:6353] |
| ENSG00000109471 | HGLibA_23070 | 48 | -0.70051703 | IL2 | interleukin 2 [Source:HGNC Symbol;Acc:HGNC:6001] |
| ENSG00000109618 | HGLibA_43424 | 48 | -0.70051703 | SEPSECS | Sep (O-phosphoserine) tRNA:Sec (selenocysteine) tRNA synthase [Source:HGNC Symbol;Acc:HGNC:30605] |
| ENSG00000111364 | HGLibA_12748 | 48 | -0.70051703 | DDX55 | DEAD-box helicase 55 [Source:HGNC Symbol;Acc:HGNC:20085] |
| ENSG00000111639 | HGLibA_30011 | 48 | -0.70051703 | MRPL51 | mitochondrial ribosomal protein L51 [Source:HGNC Symbol;Acc:HGNC:14044] |
| ENSG00000112079 | HGLibA_47501 | 48 | -0.70051703 | STK38 | serine/threonine kinase 38 [Source:HGNC Symbol;Acc:HGNC:17847] |
| ENSG00000112139 | HGLibA_28675 | 48 | -0.70051703 | MDGA1 | MAM domain containing glycosylphosphatidylinositol anchor 1 [Source:HGNC Symbol;Acc:HGNC:19267] |
| ENSG00000112651 | HGLibA_29921 | 48 | -0.70051703 | MRPL2 | mitochondrial ribosomal protein L2 [Source:HGNC Symbol;Acc:HGNC:14056] |
| ENSG00000113575 | HGLibA_38134 | 48 | -0.70051703 | PPP2CA | protein phosphatase 2 catalytic subunit alpha [Source:HGNC Symbol;Acc:HGNC:9299] |
| ENSG00000113812 | HGLibA_00674 | 48 | -0.70051703 | ACTR8 | ARP8 actin related protein 8 homolog [Source:HGNC Symbol;Acc:HGNC:14672] |
| ENSG00000115226 | HGLibA_17752 | 48 | -0.70051703 | FNDC4 | fibronectin type III domain containing 4 [Source:HGNC Symbol;Acc:HGNC:20239] |
| ENSG00000116005 | HGLibA_35693 | 48 | -0.70051703 | PCYOX1 | prenylcysteine oxidase 1 [Source:HGNC Symbol;Acc:HGNC:20588] |
| ENSG00000116690 | HGLibA_38457 | 48 | -0.70051703 | PRG4 | proteoglycan 4 [Source:HGNC Symbol;Acc:HGNC:9364] |
| ENSG00000117862 | HGLibA_52408 | 48 | -0.70051703 | TXNDC12 | thioredoxin domain containing 12 [Source:HGNC Symbol;Acc:HGNC:24626] |
| ENSG00000119661 | HGLibA_13644 | 48 | -0.70051703 | DNAL1 | dynein axonemal light chain 1 [Source:HGNC Symbol;Acc:HGNC:23247] |
| ENSG00000121101 | HGLibA_49065 | 48 | -0.70051703 | TEX14 | intercellular bridge forming factor [Source:HGNC Symbol;Acc:HGNC:11737]" |
| ENSG00000121380 | HGLibA_04317 | 48 | -0.70051703 | BCL2L14 | BCL2 like 14 [Source:HGNC Symbol;Acc:HGNC:16657] |
| ENSG00000121741 | HGLibA_55393 | 48 | -0.70051703 | ZMYM2 | zinc finger MYM-type containing 2 [Source:HGNC Symbol;Acc:HGNC:12989] |
| ENSG00000122550 | HGLibA_25223 | 48 | -0.70051703 | KLHL7 | kelch like family member 7 [Source:HGNC Symbol;Acc:HGNC:15646] |
| ENSG00000122870 | HGLibA_04491 | 48 | -0.70051703 | BICC1 | BicC family RNA binding protein 1 [Source:HGNC Symbol;Acc:HGNC:19351] |
| ENSG00000123810 | HGLibA_04046 | 48 | -0.70051703 | B9D2 | B9 domain containing 2 [Source:HGNC Symbol;Acc:HGNC:28636] |
| ENSG00000124177 | HGLibA_09402 | 48 | -0.70051703 | CHD6 | chromodomain helicase DNA binding protein 6 [Source:HGNC Symbol;Acc:HGNC:19057] |
| ENSG00000124493 | HGLibA_20350 | 48 | -0.70051703 | GRM4 | glutamate metabotropic receptor 4 [Source:HGNC Symbol;Acc:HGNC:4596] |
| ENSG00000124535 | HGLibA_54412 | 48 | -0.70051703 | WRNIP1 | Werner helicase interacting protein 1 [Source:HGNC Symbol;Acc:HGNC:20876] |
| ENSG00000124541 | HGLibA_42279 | 48 | -0.70051703 | RRP36 | ribosomal RNA processing 36 [Source:HGNC Symbol;Acc:HGNC:21374] |
| ENSG00000125257 | HGLibA_00175 | 48 | -0.70051703 | ABCC4 | ATP binding cassette subfamily C member 4 [Source:HGNC Symbol;Acc:HGNC:55] |
| ENSG00000125651 | HGLibA_20538 | 48 | -0.70051703 | GTF2F1 | general transcription factor IIF subunit 1 [Source:HGNC Symbol;Acc:HGNC:4652] |
| ENSG00000125810 | HGLibA_08480 | 48 | -0.70051703 | CD93 | CD93 molecule [Source:HGNC Symbol;Acc:HGNC:15855] |
| ENSG00000125995 | HGLibA_41758 | 48 | -0.70051703 | ROMO1 | reactive oxygen species modulator 1 [Source:HGNC Symbol;Acc:HGNC:16185] |
| ENSG00000126550 | HGLibA_22351 | 48 | -0.70051703 | HTN1 | histatin 1 [Source:HGNC Symbol;Acc:HGNC:5283] |
| ENSG00000127990 | HGLibA_43805 | 48 | -0.70051703 | SGCE | sarcoglycan epsilon [Source:HGNC Symbol;Acc:HGNC:10808] |
| ENSG00000128016 | HGLibA_55205 | 48 | -0.70051703 | ZFP36 | ZFP36 ring finger protein [Source:HGNC Symbol;Acc:HGNC:12862] |
| ENSG00000128519 | HGLibA_48375 | 48 | -0.70051703 | TAS2R16 | taste 2 receptor member 16 [Source:HGNC Symbol;Acc:HGNC:14921] |
| ENSG00000128655 | HGLibA_35763 | 48 | -0.70051703 | PDE11A | phosphodiesterase 11A [Source:HGNC Symbol;Acc:HGNC:8773] |
| ENSG00000128708 | HGLibA_20872 | 48 | -0.70051703 | HAT1 | histone acetyltransferase 1 [Source:HGNC Symbol;Acc:HGNC:4821] |
| ENSG00000130226 | HGLibA_13876 | 48 | -0.70051703 | DPP6 | dipeptidyl peptidase like 6 [Source:HGNC Symbol;Acc:HGNC:3010] |
| ENSG00000131061 | HGLibA_55840 | 48 | -0.70051703 | ZNF341 | zinc finger protein 341 [Source:HGNC Symbol;Acc:HGNC:15992] |
| ENSG00000133640 | HGLibA_27456 | 48 | -0.70051703 | LRRIQ1 | leucine rich repeats and IQ motif containing 1 [Source:HGNC Symbol;Acc:HGNC:25708] |
| ENSG00000133678 | HGLibA_50327 | 48 | -0.70051703 | TMEM254 | transmembrane protein 254 [Source:HGNC Symbol;Acc:HGNC:25804] |
| ENSG00000133710 | HGLibA_46696 | 48 | -0.70051703 | SPINK5 | Kazal type 5 [Source:HGNC Symbol;Acc:HGNC:15464]" |
| ENSG00000134014 | HGLibA_14982 | 48 | -0.70051703 | ELP3 | elongator acetyltransferase complex subunit 3 [Source:HGNC Symbol;Acc:HGNC:20696] |
| ENSG00000137857 | HGLibA_14098 | 48 | -0.70051703 | DUOX1 | dual oxidase 1 [Source:HGNC Symbol;Acc:HGNC:3062] |
| ENSG00000138639 | HGLibA_02735 | 48 | -0.70051703 | ARHGAP24 | Rho GTPase activating protein 24 [Source:HGNC Symbol;Acc:HGNC:25361] |
| ENSG00000140396 | HGLibA_31337 | 48 | -0.70051703 | NCOA2 | nuclear receptor coactivator 2 [Source:HGNC Symbol;Acc:HGNC:7669] |
| ENSG00000142185 | HGLibA_51755 | 48 | -0.70051703 | TRPM2 | transient receptor potential cation channel subfamily M member 2 [Source:HGNC Symbol;Acc:HGNC:12339] |
| ENSG00000143190 | HGLibA_37756 | 48 | -0.70051703 | POU2F1 | POU class 2 homeobox 1 [Source:HGNC Symbol;Acc:HGNC:9212] |
| ENSG00000143627 | HGLibA_36829 | 48 | -0.70051703 | PKLR | pyruvate kinase L/R [Source:HGNC Symbol;Acc:HGNC:9020] |
| ENSG00000144010 | HGLibA_51503 | 48 | -0.70051703 | TRIM43B | tripartite motif containing 43B [Source:HGNC Symbol;Acc:HGNC:37146] |
| ENSG00000144407 | HGLibA_39372 | 48 | -0.70051703 | PTH2R | parathyroid hormone 2 receptor [Source:HGNC Symbol;Acc:HGNC:9609] |
| ENSG00000145248 | HGLibA_44338 | 48 | -0.70051703 | SLC10A4 | solute carrier family 10 member 4 [Source:HGNC Symbol;Acc:HGNC:22980] |
| ENSG00000145476 | HGLibA_12198 | 48 | -0.70051703 | CYP4V2 | cytochrome P450 family 4 subfamily V member 2 [Source:HGNC Symbol;Acc:HGNC:23198] |
| ENSG00000145864 | HGLibA_18377 | 48 | -0.70051703 | GABRB2 | gamma-aminobutyric acid type A receptor beta2 subunit [Source:HGNC Symbol;Acc:HGNC:4082] |
| ENSG00000146039 | HGLibA_44469 | 48 | -0.70051703 | SLC17A4 | solute carrier family 17 member 4 [Source:HGNC Symbol;Acc:HGNC:10932] |
| ENSG00000146373 | HGLibA_41634 | 48 | -0.70051703 | RNF217 | ring finger protein 217 [Source:HGNC Symbol;Acc:HGNC:21487] |
| ENSG00000148308 | HGLibA_20591 | 48 | -0.70051703 | GTF3C5 | general transcription factor IIIC subunit 5 [Source:HGNC Symbol;Acc:HGNC:4668] |
| ENSG00000148719 | HGLibA_13528 | 48 | -0.70051703 | DNAJB12 | DnaJ heat shock protein family (Hsp40) member B12 [Source:HGNC Symbol;Acc:HGNC:14891] |
| ENSG00000149050 | HGLibA_55594 | 48 | -0.70051703 | ZNF214 | zinc finger protein 214 [Source:HGNC Symbol;Acc:HGNC:13006] |
| ENSG00000149089 | HGLibA_02422 | 48 | -0.70051703 | APIP | APAF1 interacting protein [Source:HGNC Symbol;Acc:HGNC:17581] |
| ENSG00000149927 | HGLibA_13725 | 48 | -0.70051703 | DOC2A | double C2 domain alpha [Source:HGNC Symbol;Acc:HGNC:2985] |
| ENSG00000149929 | HGLibA_21353 | 48 | -0.70051703 | HIRIP3 | HIRA interacting protein 3 [Source:HGNC Symbol;Acc:HGNC:4917] |
| ENSG00000150712 | HGLibA_30419 | 48 | -0.70051703 | MTMR12 | myotubularin related protein 12 [Source:HGNC Symbol;Acc:HGNC:18191] |
| ENSG00000151304 | HGLibA_46963 | 48 | -0.70051703 | SRFBP1 | serum response factor binding protein 1 [Source:HGNC Symbol;Acc:HGNC:26333] |
| ENSG00000151704 | HGLibA_24246 | 48 | -0.70051703 | KCNJ1 | potassium voltage-gated channel subfamily J member 1 [Source:HGNC Symbol;Acc:HGNC:6255] |
| ENSG00000152503 | HGLibA_51474 | 48 | -0.70051703 | TRIM36 | tripartite motif containing 36 [Source:HGNC Symbol;Acc:HGNC:16280] |
| ENSG00000153086 | HGLibA_00454 | 48 | -0.70051703 | ACMSD | aminocarboxymuconate semialdehyde decarboxylase [Source:HGNC Symbol;Acc:HGNC:19288] |
| ENSG00000154146 | HGLibA_32687 | 48 | -0.70051703 | NRGN | neurogranin [Source:HGNC Symbol;Acc:HGNC:8000] |
| ENSG00000154217 | HGLibA_36748 | 48 | -0.70051703 | PITPNC1 | phosphatidylinositol transfer protein cytoplasmic 1 [Source:HGNC Symbol;Acc:HGNC:21045] |
| ENSG00000155085 | HGLibA_01400 | 48 | -0.70051703 | AK9 | adenylate kinase 9 [Source:HGNC Symbol;Acc:HGNC:33814] |
| ENSG00000155761 | HGLibA_46355 | 48 | -0.70051703 | SPAG17 | sperm associated antigen 17 [Source:HGNC Symbol;Acc:HGNC:26620] |
| ENSG00000156564 | HGLibA_27154 | 48 | -0.70051703 | LRFN2 | leucine rich repeat and fibronectin type III domain containing 2 [Source:HGNC Symbol;Acc:HGNC:21226] |
| ENSG00000157500 | HGLibA_02567 | 48 | -0.70051703 | APPL1 | phosphotyrosine interacting with PH domain and leucine zipper 1 [Source:HGNC Symbol;Acc:HGNC:24035]" |
| ENSG00000158201 | HGLibA_00276 | 48 | -0.70051703 | ABHD3 | abhydrolase domain containing 3 [Source:HGNC Symbol;Acc:HGNC:18718] |
| ENSG00000158352 | HGLibA_44096 | 48 | -0.70051703 | SHROOM4 | shroom family member 4 [Source:HGNC Symbol;Acc:HGNC:29215] |
| ENSG00000158560 | HGLibA_14236 | 48 | -0.70051703 | DYNC1I1 | dynein cytoplasmic 1 intermediate chain 1 [Source:HGNC Symbol;Acc:HGNC:2963] |
| ENSG00000159450 | HGLibA_48804 | 48 | -0.70051703 | TCHH | trichohyalin [Source:HGNC Symbol;Acc:HGNC:11791] |
| ENSG00000161203 | HGLibA_02313 | 48 | -0.70051703 | AP2M1 | adaptor related protein complex 2 subunit mu 1 [Source:HGNC Symbol;Acc:HGNC:564] |
| ENSG00000161970 | HGLibA_41913 | 48 | -0.70051703 | RPL26 | ribosomal protein L26 [Source:HGNC Symbol;Acc:HGNC:10327] |
| ENSG00000162104 | HGLibA_00932 | 48 | -0.70051703 | ADCY9 | adenylate cyclase 9 [Source:HGNC Symbol;Acc:HGNC:240] |
| ENSG00000162300 | HGLibA_55249 | 48 | -0.70051703 | ZFPL1 | zinc finger protein like 1 [Source:HGNC Symbol;Acc:HGNC:12868] |
| ENSG00000162517 | HGLibA_36024 | 48 | -0.70051703 | PEF1 | penta-EF-hand domain containing 1 [Source:HGNC Symbol;Acc:HGNC:30009] |
| ENSG00000162572 | HGLibA_43020 | 48 | -0.70051703 | SCNN1D | sodium channel epithelial 1 delta subunit [Source:HGNC Symbol;Acc:HGNC:10601] |
| ENSG00000162706 | HGLibA_06972 | 48 | -0.70051703 | CADM3 | cell adhesion molecule 3 [Source:HGNC Symbol;Acc:HGNC:17601] |
| ENSG00000162736 | HGLibA_31378 | 48 | -0.70051703 | NCSTN | nicastrin [Source:HGNC Symbol;Acc:HGNC:17091] |
| ENSG00000163170 | HGLibA_04689 | 48 | -0.70051703 | BOLA3 | bolA family member 3 [Source:HGNC Symbol;Acc:HGNC:24415] |
| ENSG00000163406 | HGLibA_44407 | 48 | -0.70051703 | SLC15A2 | solute carrier family 15 member 2 [Source:HGNC Symbol;Acc:HGNC:10921] |
| ENSG00000163464 | HGLibA_11881 | 48 | -0.70051703 | CXCR1 | C-X-C motif chemokine receptor 1 [Source:HGNC Symbol;Acc:HGNC:6026] |
| ENSG00000164283 | HGLibA_15570 | 48 | -0.70051703 | ESM1 | endothelial cell specific molecule 1 [Source:HGNC Symbol;Acc:HGNC:3466] |
| ENSG00000164604 | HGLibA_20097 | 48 | -0.70051703 | GPR85 | G protein-coupled receptor 85 [Source:HGNC Symbol;Acc:HGNC:4536] |
| ENSG00000165215 | HGLibA_09894 | 48 | -0.70051703 | CLDN3 | claudin 3 [Source:HGNC Symbol;Acc:HGNC:2045] |
| ENSG00000165309 | HGLibA_03016 | 48 | -0.70051703 | ARMC3 | armadillo repeat containing 3 [Source:HGNC Symbol;Acc:HGNC:30964] |
| ENSG00000165917 | HGLibA_40268 | 48 | -0.70051703 | RAPSN | receptor associated protein of the synapse [Source:HGNC Symbol;Acc:HGNC:9863] |
| ENSG00000166261 | HGLibA_55574 | 48 | -0.70051703 | ZNF202 | zinc finger protein 202 [Source:HGNC Symbol;Acc:HGNC:12994] |
| ENSG00000166454 | HGLibA_03527 | 48 | -0.70051703 | ATMIN | ATM interactor [Source:HGNC Symbol;Acc:HGNC:29034] |
| ENSG00000166478 | HGLibA_55491 | 48 | -0.70051703 | ZNF143 | zinc finger protein 143 [Source:HGNC Symbol;Acc:HGNC:12928] |
| ENSG00000166526 | HGLibA_55765 | 48 | -0.70051703 | ZNF3 | zinc finger protein 3 [Source:HGNC Symbol;Acc:HGNC:13089] |
| ENSG00000167105 | HGLibA_50560 | 48 | -0.70051703 | TMEM92 | transmembrane protein 92 [Source:HGNC Symbol;Acc:HGNC:26579] |
| ENSG00000167107 | HGLibA_00548 | 48 | -0.70051703 | ACSF2 | acyl-CoA synthetase family member 2 [Source:HGNC Symbol;Acc:HGNC:26101] |
| ENSG00000167553 | HGLibA_52249 | 48 | -0.70051703 | TUBA1C | tubulin alpha 1c [Source:HGNC Symbol;Acc:HGNC:20768] |
| ENSG00000167705 | HGLibA_41249 | 48 | -0.70051703 | RILP | Rab interacting lysosomal protein [Source:HGNC Symbol;Acc:HGNC:30266] |
| ENSG00000168314 | HGLibA_29605 | 48 | -0.70051703 | MOBP | myelin-associated oligodendrocyte basic protein [Source:HGNC Symbol;Acc:HGNC:7189] |
| ENSG00000168411 | HGLibA_40959 | 48 | -0.70051703 | RFWD3 | ring finger and WD repeat domain 3 [Source:HGNC Symbol;Acc:HGNC:25539] |
| ENSG00000168772 | HGLibA_11972 | 48 | -0.70051703 | CXXC4 | CXXC finger protein 4 [Source:HGNC Symbol;Acc:HGNC:24593] |
| ENSG00000170848 | HGLibA_39052 | 48 | -0.70051703 | PSG6 | pregnancy specific beta-1-glycoprotein 6 [Source:HGNC Symbol;Acc:HGNC:9523] |
| ENSG00000171425 | HGLibA_56308 | 48 | -0.70051703 | ZNF581 | zinc finger protein 581 [Source:HGNC Symbol;Acc:HGNC:25017] |
| ENSG00000171448 | HGLibA_54853 | 48 | -0.70051703 | ZBTB26 | zinc finger and BTB domain containing 26 [Source:HGNC Symbol;Acc:HGNC:23383] |
| ENSG00000171806 | HGLibA_28970 | 48 | -0.70051703 | METTL18 | methyltransferase like 18 [Source:HGNC Symbol;Acc:HGNC:28793] |

| | | | | | |
|---|---|---|---|---|---|
| ENSG00000171936 | HGLibA_33512 | 48 | -0.70051703 | OR10H3 | olfactory receptor family 10 subfamily H member 3 [Source:HGNC Symbol;Acc:HGNC:8174] |
| ENSG00000172404 | HGLibA_13552 | 48 | -0.70051703 | DNAJB7 | DnaJ heat shock protein family (Hsp40) member B7 [Source:HGNC Symbol;Acc:HGNC:24986] |
| ENSG00000172671 | HGLibA_55157 | 48 | -0.70051703 | ZFAND4 | zinc finger AN1-type containing 4 [Source:HGNC Symbol;Acc:HGNC:23504] |
| ENSG00000172738 | HGLibA_50226 | 48 | -0.70051703 | TMEM217 | transmembrane protein 217 [Source:HGNC Symbol;Acc:HGNC:21238] |
| ENSG00000173404 | HGLibA_23395 | 48 | -0.70051703 | INSM1 | INSM transcriptional repressor 1 [Source:HGNC Symbol;Acc:HGNC:6090] |
| ENSG00000173626 | HGLibA_51320 | 48 | -0.70051703 | TRAPPC3L | trafficking protein particle complex 3 like [Source:HGNC Symbol;Acc:HGNC:21090] |
| ENSG00000173762 | HGLibA_08435 | 48 | -0.70051703 | CD7 | CD7 molecule [Source:HGNC Symbol;Acc:HGNC:1695] |
| ENSG00000174460 | HGLibA_55008 | 48 | -0.70051703 | ZCCHC12 | zinc finger CCHC-type containing 12 [Source:HGNC Symbol;Acc:HGNC:27273] |
| ENSG00000174527 | HGLibA_30840 | 48 | -0.70051703 | MYO1H | myosin IH [Source:HGNC Symbol;Acc:HGNC:13879] |
| ENSG00000175110 | HGLibA_30065 | 48 | -0.70051703 | MRPS22 | mitochondrial ribosomal protein S22 [Source:HGNC Symbol;Acc:HGNC:14508] |
| ENSG00000176256 | HGLibA_21700 | 48 | -0.70051703 | HMGB4 | high mobility group box 4 [Source:HGNC Symbol;Acc:HGNC:24954] |
| ENSG00000176294 | HGLibA_34045 | 48 | -0.70051703 | OR4N2 | olfactory receptor family 4 subfamily N member 2 [Source:HGNC Symbol;Acc:HGNC:14742] |
| ENSG00000177302 | HGLibA_50999 | 48 | -0.70051703 | TOP3A | DNA topoisomerase III alpha [Source:HGNC Symbol;Acc:HGNC:11992] |
| ENSG00000177414 | HGLibA_52663 | 48 | -0.70051703 | UBE2U | ubiquitin conjugating enzyme E2 U [Source:HGNC Symbol;Acc:HGNC:28559] |
| ENSG00000177414 | HGLibA_52664 | 48 | -0.70051703 | UBE2U | ubiquitin conjugating enzyme E2 U [Source:HGNC Symbol;Acc:HGNC:28559] |
| ENSG00000177675 | HGLibA_08245 | 48 | -0.70051703 | CD163L1 | CD163 molecule like 1 [Source:HGNC Symbol;Acc:HGNC:30375] |
| ENSG00000179023 | HGLibA_25106 | 48 | -0.70051703 | KLHDC7A | kelch domain containing 7A [Source:HGNC Symbol;Acc:HGNC:26791] |
| ENSG00000179564 | HGLibA_27561 | 48 | -0.70051703 | LSMEM2 | leucine rich single-pass membrane protein 2 [Source:HGNC Symbol;Acc:HGNC:26781] |
| ENSG00000179930 | HGLibA_56456 | 48 | -0.70051703 | ZNF648 | zinc finger protein 648 [Source:HGNC Symbol;Acc:HGNC:18190] |
| ENSG00000180447 | HGLibA_18641 | 48 | -0.70051703 | GAS1 | growth arrest specific 1 [Source:HGNC Symbol;Acc:HGNC:4165] |
| ENSG00000182271 | HGLibA_50586 | 48 | -0.70051703 | TMIGD1 | transmembrane and immunoglobulin domain containing 1 [Source:HGNC Symbol;Acc:HGNC:32431] |
| ENSG00000183943 | HGLibA_38593 | 48 | -0.70051703 | PRKX | protein kinase X-linked [Source:HGNC Symbol;Acc:HGNC:9441] |
| ENSG00000186432 | HGLibA_25364 | 48 | -0.70051703 | KPNA4 | karyopherin subunit alpha 4 [Source:HGNC Symbol;Acc:HGNC:6397] |
| ENSG00000186496 | HGLibA_55916 | 48 | -0.70051703 | ZNF396 | zinc finger protein 396 [Source:HGNC Symbol;Acc:HGNC:18824] |
| ENSG00000187550 | HGLibA_42803 | 48 | -0.70051703 | SBK2 | SH3 domain binding kinase family member 2 [Source:HGNC Symbol;Acc:HGNC:34416] |
| ENSG00000188321 | HGLibA_56236 | 48 | -0.70051703 | ZNF559 | zinc finger protein 559 [Source:HGNC Symbol;Acc:HGNC:28197] |
| ENSG00000188763 | HGLibA_18288 | 48 | -0.70051703 | FZD9 | frizzled class receptor 9 [Source:HGNC Symbol;Acc:HGNC:4047] |
| ENSG00000189068 | HGLibA_53856 | 48 | -0.70051703 | VSTM1 | V-set and transmembrane domain containing 1 [Source:HGNC Symbol;Acc:HGNC:29455] |
| ENSG00000196455 | HGLibA_36656 | 48 | -0.70051703 | PIK3R4 | phosphoinositide-3-kinase regulatory subunit 4 [Source:HGNC Symbol;Acc:HGNC:8982] |
| ENSG00000196754 | HGLibA_42552 | 48 | -0.70051703 | S100A2 | S100 calcium binding protein A2 [Source:HGNC Symbol;Acc:HGNC:10492] |
| ENSG00000197323 | HGLibA_51464 | 48 | -0.70051703 | TRIM33 | tripartite motif containing 33 [Source:HGNC Symbol;Acc:HGNC:16290] |
| ENSG00000198466 | HGLibA_56330 | 48 | -0.70051703 | ZNF587 | zinc finger protein 587 [Source:HGNC Symbol;Acc:HGNC:30955] |
| ENSG00000203786 | HGLibA_25379 | 48 | -0.70051703 | KPRP | keratinocyte proline rich protein [Source:HGNC Symbol;Acc:HGNC:31823] |
| ENSG00000204104 | HGLibA_51249 | 48 | -0.70051703 | TRAF3IP1 | TRAF3 interacting protein 1 [Source:HGNC Symbol;Acc:HGNC:17861] |
| ENSG00000204175 | HGLibA_20144 | 48 | -0.70051703 | GPRIN2 | G protein regulated inducer of neurite outgrowth 2 [Source:HGNC Symbol;Acc:HGNC:23730] |
| ENSG00000204308 | HGLibA_41691 | 48 | -0.70051703 | RNF5 | ring finger protein 5 [Source:HGNC Symbol;Acc:HGNC:10068] |
| ENSG00000204382 | HGLibA_54469 | 48 | -0.70051703 | XAGE1B | X antigen family member 1B [Source:HGNC Symbol;Acc:HGNC:25400] |
| ENSG00000204614 | HGLibA_51492 | 48 | -0.70051703 | TRIM40 | tripartite motif containing 40 [Source:HGNC Symbol;Acc:HGNC:18736] |
| ENSG00000204710 | HGLibA_46577 | 48 | -0.70051703 | SPDYC | speedy/RINGO cell cycle regulator family member C [Source:HGNC Symbol;Acc:HGNC:32681] |
| ENSG00000205129 | HGLibA_06337 | 48 | -0.70051703 | C4orf47 | chromosome 4 open reading frame 47 [Source:HGNC Symbol;Acc:HGNC:34346] |
| ENSG00000205643 | HGLibA_08860 | 48 | -0.70051703 | CDPF1 | cysteine rich DPF motif domain containing 1 [Source:HGNC Symbol;Acc:HGNC:33710] |
| ENSG00000205883 | HGLibA_12907 | 48 | -0.70051703 | DEFB135 | defensin beta 135 [Source:HGNC Symbol;Acc:HGNC:32400] |
| ENSG00000206140 | HGLibA_50147 | 48 | -0.70051703 | TMEM191C | transmembrane protein 191C [Source:HGNC Symbol;Acc:HGNC:33601] |
| ENSG00000206260 | HGLibA_38822 | 48 | -0.70051703 | PRR23A | proline rich 23A [Source:HGNC Symbol;Acc:HGNC:37172] |
| ENSG00000206531 | HGLibA_08289 | 48 | -0.70051703 | CD200R1L | CD200 receptor 1 like [Source:HGNC Symbol;Acc:HGNC:24665] |
| ENSG00000213214 | HGLibA_02851 | 48 | -0.70051703 | ARHGEF35 | Rho guanine nucleotide exchange factor 35 [Source:HGNC Symbol;Acc:HGNC:33846] |
| ENSG00000213658 | HGLibA_26058 | 48 | -0.70051703 | LAT | linker for activation of T cells [Source:HGNC Symbol;Acc:HGNC:18874] |
| ENSG00000214107 | HGLibA_27928 | 48 | -0.70051703 | MAGEB1 | MAGE family member B1 [Source:HGNC Symbol;Acc:HGNC:6808] |
| ENSG00000215262 | HGLibA_24398 | 48 | -0.70051703 | KCNU1 | potassium calcium-activated channel subfamily U member 1 [Source:HGNC Symbol;Acc:HGNC:18867] |
| ENSG00000221909 | HGLibA_16411 | 48 | -0.70051703 | FAM200A | family with sequence similarity 200 member A [Source:HGNC Symbol;Acc:HGNC:25401] |
| ENSG00000229453 | HGLibA_46706 | 48 | -0.70051703 | SPINK8 | Kazal type 8 (putative) [Source:HGNC Symbol;Acc:HGNC:33160]" |
| ENSG00000240857 | HGLibA_40742 | 48 | -0.70051703 | RDH14 | retinol dehydrogenase 14 [Source:HGNC Symbol;Acc:HGNC:19979] |
| ENSG00000241553 | HGLibA_03086 | 48 | -0.70051703 | ARPC4 | actin related protein 2/3 complex subunit 4 [Source:HGNC Symbol;Acc:HGNC:707] |
| ENSG00000243729 | HGLibA_34365 | 48 | -0.70051703 | OR5V1 | olfactory receptor family 5 subfamily V member 1 [Source:HGNC Symbol;Acc:HGNC:13972] |
| ENSG00000251247 | HGLibA_55847 | 48 | -0.70051703 | ZNF345 | zinc finger protein 345 [Source:HGNC Symbol;Acc:HGNC:16367] |
| ENSG00000253368 | HGLibA_51709 | 48 | -0.70051703 | TRNP1 | TMF1-regulated nuclear protein 1 [Source:HGNC Symbol;Acc:HGNC:34348] |
| ENSG00000254585 | HGLibA_27993 | 48 | -0.70051703 | MAGEL2 | MAGE family member L2 [Source:HGNC Symbol;Acc:HGNC:6814] |
| ENSG00000258713 | HGLibA_05987 | 48 | -0.70051703 | C20orf141 | chromosome 20 open reading frame 141 [Source:HGNC Symbol;Acc:HGNC:16134] |
| ENSG00000261052 | HGLibA_47737 | 48 | -0.70051703 | SULT1A3 | sulfotransferase family 1A member 3 [Source:HGNC Symbol;Acc:HGNC:11455] |
| ENSG00000268350 | HGLibA_16185 | 48 | -0.70051703 | FAM156A | family with sequence similarity 156 member A [Source:HGNC Symbol;Acc:HGNC:30114] |
| ENSG00000273111 | HGLibA_27723 | 48 | -0.70051703 | LYPD4 | LY6/PLAUR domain containing 4 [Source:HGNC Symbol;Acc:HGNC:28659] |
| ENSG00000273841 | HGLibA_48262 | 48 | -0.70051703 | TAF9 | TATA-box binding protein associated factor 9 [Source:HGNC Symbol;Acc:HGNC:11542] |
| ENSG00000276747 | HGLibA_34981 | 48 | -0.70051703 | PADI6 | peptidyl arginine deiminase 6 [Source:HGNC Symbol;Acc:HGNC:20449] |
| FAM101A | HGLibA_15973 | 48 | -0.70051703 | FAM101A | - |
| FDX1L | HGLibA_17261 | 48 | -0.70051703 | FDX1L | - |
| FOXD4L2 | HGLibA_17844 | 48 | -0.70051703 | FOXD4L2 | - |
| GTPBP6_Y | HGLibA_20620 | 48 | -0.70051703 | GTPBP6_Y | - |
| H2AFX | HGLibA_20761 | 48 | -0.70051703 | H2AFX | - |
| HNRPDL | HGLibA_21854 | 48 | -0.70051703 | HNRPDL | - |
| hsa-mir-1205 | HGLibA_57278 | 48 | -0.70051703 | hsa-mir-1205 | - |
| hsa-mir-214 | HGLibA_58247 | 48 | -0.70051703 | hsa-mir-214 | - |
| hsa-mir-3144 | HGLibA_58633 | 48 | -0.70051703 | hsa-mir-3144 | - |
| hsa-mir-3198-1 | HGLibA_58890 | 48 | -0.70051703 | hsa-mir-3198-1 | - |
| hsa-mir-3198-1 | HGLibA_58891 | 48 | -0.70051703 | hsa-mir-3198-1 | - |
| hsa-mir-3682 | HGLibA_59282 | 48 | -0.70051703 | hsa-mir-3682 | - |
| hsa-mir-3940 | HGLibA_59619 | 48 | -0.70051703 | hsa-mir-3940 | - |
| hsa-mir-3945 | HGLibA_59638 | 48 | -0.70051703 | hsa-mir-3945 | - |
| hsa-mir-4491 | HGLibA_60382 | 48 | -0.70051703 | hsa-mir-4491 | - |
| hsa-mir-4524b | HGLibA_60556 | 48 | -0.70051703 | hsa-mir-4524b | - |
| hsa-mir-4683 | HGLibA_60842 | 48 | -0.70051703 | hsa-mir-4683 | - |
| hsa-mir-4706 | HGLibA_60923 | 48 | -0.70051703 | hsa-mir-4706 | - |
| hsa-mir-4732 | HGLibA_61024 | 48 | -0.70051703 | hsa-mir-4732 | - |
| hsa-mir-5186 | HGLibA_61637 | 48 | -0.70051703 | hsa-mir-5186 | - |
| hsa-mir-5191 | HGLibA_61682 | 48 | -0.70051703 | hsa-mir-5191 | - |
| hsa-mir-5196 | HGLibA_61703 | 48 | -0.70051703 | hsa-mir-5196 | - |
| hsa-mir-520h | HGLibA_61761 | 48 | -0.70051703 | hsa-mir-520h | - |
| hsa-mir-550b-2 | HGLibA_62115 | 48 | -0.70051703 | hsa-mir-550b-2 | - |
| hsa-mir-5703 | HGLibA_62354 | 48 | -0.70051703 | hsa-mir-5703 | - |
| hsa-mir-625 | HGLibA_62741 | 48 | -0.70051703 | hsa-mir-625 | - |
| hsa-mir-6749 | HGLibA_63151 | 48 | -0.70051703 | hsa-mir-6749 | - |
| hsa-mir-6755 | HGLibA_63177 | 48 | -0.70051703 | hsa-mir-6755 | - |
| hsa-mir-6793 | HGLibA_63350 | 48 | -0.70051703 | hsa-mir-6793 | - |
| hsa-mir-6856 | HGLibA_63597 | 48 | -0.70051703 | hsa-mir-6856 | - |
| hsa-mir-6862-2 | HGLibA_63631 | 48 | -0.70051703 | hsa-mir-6862-2 | - |
| hsa-mir-7151 | HGLibA_63831 | 48 | -0.70051703 | hsa-mir-7151 | - |
| hsa-mir-934 | HGLibA_64309 | 48 | -0.70051703 | hsa-mir-934 | - |
| hsa-mir-99b | HGLibA_64382 | 48 | -0.70051703 | hsa-mir-99b | - |
| IFLTD1 | HGLibA_22654 | 48 | -0.70051703 | IFLTD1 | - |
| LOC100288332 | HGLibA_26831 | 48 | -0.70051703 | LOC100288332 | - |
| MUM1 | HGLibA_30564 | 48 | -0.70051703 | MUM1 | - |
| NHP2L1 | HGLibA_31912 | 48 | -0.70051703 | NHP2L1 | - |
| PPIAL4B | HGLibA_37899 | 48 | -0.70051703 | PPIAL4B | - |
| TMEM136 | HGLibA_49953 | 48 | -0.70051703 | TMEM136 | - |
| TSTA3 | HGLibA_52028 | 48 | -0.70051703 | TSTA3 | - |
| AGXT2L1 | HGLibA_01277 | 47 | -0.709562373 | AGXT2L1 | - |
| C2orf48 | HGLibA_06138 | 47 | -0.709562373 | C2orf48 | - |
| C2orf48 | HGLibA_06136 | 47 | -0.709562373 | C2orf48 | - |
| C9orf91 | HGLibA_06776 | 47 | -0.709562373 | C9orf91 | - |
| CCDC151 | HGLibA_07645 | 47 | -0.709562373 | CCDC151 | - |
| CCDC64 | HGLibA_07827 | 47 | -0.709562373 | CCDC64 | - |
| CXorf21 | HGLibA_11901 | 47 | -0.709562373 | CXorf21 | - |
| CXorf40A | HGLibA_11922 | 47 | -0.709562373 | CXorf40A | - |
| DIRC2 | HGLibA_13267 | 47 | -0.709562373 | DIRC2 | - |
| ENSG00000004799 | HGLibA_35903 | 47 | -0.709562373 | PDK4 | pyruvate dehydrogenase kinase 4 [Source:HGNC Symbol;Acc:HGNC:8812] |
| ENSG00000012174 | HGLibA_28537 | 47 | -0.709562373 | MBTPS2 | site 2 [Source:HGNC Symbol;Acc:HGNC:15455]" |
| ENSG00000012504 | HGLibA_32587 | 47 | -0.709562373 | NR1H4 | nuclear receptor subfamily 1 group H member 4 [Source:HGNC Symbol;Acc:HGNC:7967] |
| ENSG00000018408 | HGLibA_54456 | 47 | -0.709562373 | WWTR1 | WW domain containing transcription regulator 1 [Source:HGNC Symbol;Acc:HGNC:24042] |
| ENSG00000027001 | HGLibA_29293 | 47 | -0.709562373 | MIPEP | mitochondrial intermediate peptidase [Source:HGNC Symbol;Acc:HGNC:7104] |
| ENSG00000034677 | HGLibA_41601 | 47 | -0.709562373 | RNF19A | RBR E3 ubiquitin protein ligase [Source:HGNC Symbol;Acc:HGNC:13432]" |
| ENSG00000039560 | HGLibA_40142 | 47 | -0.709562373 | RAI14 | retinoic acid induced 14 [Source:HGNC Symbol;Acc:HGNC:14873] |
| ENSG00000042429 | HGLibA_28763 | 47 | -0.709562373 | MED17 | mediator complex subunit 17 [Source:HGNC Symbol;Acc:HGNC:2375] |
| ENSG00000048140 | HGLibA_51913 | 47 | -0.709562373 | TSPAN17 | tetraspanin 17 [Source:HGNC Symbol;Acc:HGNC:13594] |
| ENSG00000052841 | HGLibA_52059 | 47 | -0.709562373 | TTC17 | tetratricopeptide repeat domain 17 [Source:HGNC Symbol;Acc:HGNC:25596] |
| ENSG00000061987 | HGLibA_29646 | 47 | -0.709562373 | MON2 | regulator of endosome-to-Golgi trafficking [Source:HGNC Symbol;Acc:HGNC:29177]" |
| ENSG00000065000 | HGLibA_02327 | 47 | -0.709562373 | AP3D1 | adaptor related protein complex 3 subunit delta 1 [Source:HGNC Symbol;Acc:HGNC:568] |
| ENSG00000069869 | HGLibA_31603 | 47 | -0.709562373 | NEDD4 | E3 ubiquitin protein ligase [Source:HGNC Symbol;Acc:HGNC:7727]" |
| ENSG00000070882 | HGLibA_34655 | 47 | -0.709562373 | OSBPL3 | oxysterol binding protein like 3 [Source:HGNC Symbol;Acc:HGNC:16370] |
| ENSG00000070886 | HGLibA_15309 | 47 | -0.709562373 | EPHA8 | EPH receptor A8 [Source:HGNC Symbol;Acc:HGNC:3391] |
| ENSG00000071073 | HGLibA_29141 | 47 | -0.709562373 | MGAT4A | 3-mannosyl-glycoprotein 4-beta-N-acetylglucosaminyltransferase A [Source:HGNC Symbol;Acc:HGNC:7047]" |
| ENSG00000074201 | HGLibA_10091 | 47 | -0.709562373 | CLNS1A | chloride nucleotide-sensitive channel 1A [Source:HGNC Symbol;Acc:HGNC:2080] |
| ENSG00000077254 | HGLibA_53337 | 47 | -0.709562373 | USP33 | ubiquitin specific peptidase 33 [Source:HGNC Symbol;Acc:HGNC:20059] |
| ENSG00000079459 | HGLibA_17251 | 47 | -0.709562373 | FDFT1 | farnesyl-diphosphate farnesyltransferase 1 [Source:HGNC Symbol;Acc:HGNC:3629] |
| ENSG00000083312 | HGLibA_50897 | 47 | -0.709562373 | TNPO1 | transportin 1 [Source:HGNC Symbol;Acc:HGNC:6401] |
| ENSG00000084652 | HGLibA_52391 | 47 | -0.709562373 | TXLNA | taxilin alpha [Source:HGNC Symbol;Acc:HGNC:30685] |
| ENSG00000085465 | HGLibA_34797 | 47 | -0.709562373 | OVGP1 | oviductal glycoprotein 1 [Source:HGNC Symbol;Acc:HGNC:8524] |

| | | | | | |
|---|---|---|---|---|---|
| ENSG00000085511 | HGLibA_28188 | 47 | -0.709562373 | MAP3K4 | mitogen-activated protein kinase kinase kinase 4 [Source:HGNC Symbol;Acc:HGNC:6856] |
| ENSG00000085514 | HGLibA_36668 | 47 | -0.709562373 | PILRA | paired immunoglobin like type 2 receptor alpha [Source:HGNC Symbol;Acc:HGNC:20396] |
| ENSG00000086159 | HGLibA_02601 | 47 | -0.709562373 | AQP6 | aquaporin 6 [Source:HGNC Symbol;Acc:HGNC:639] |
| ENSG00000086289 | HGLibA_15275 | 47 | -0.709562373 | EPDR1 | ependymin related 1 [Source:HGNC Symbol;Acc:HGNC:17572] |
| ENSG00000087266 | HGLibA_43928 | 47 | -0.709562373 | SH3BP2 | SH3 domain binding protein 2 [Source:HGNC Symbol;Acc:HGNC:10825] |
| ENSG00000087269 | HGLibA_32303 | 47 | -0.709562373 | NOP14 | NOP14 nucleolar protein [Source:HGNC Symbol;Acc:HGNC:16821] |
| ENSG00000089847 | HGLibA_02045 | 47 | -0.709562373 | ANKRD24 | ankyrin repeat domain 24 [Source:HGNC Symbol;Acc:HGNC:29424] |
| ENSG00000091656 | HGLibA_55178 | 47 | -0.709562373 | ZFHX4 | zinc finger homeobox 4 [Source:HGNC Symbol;Acc:HGNC:30939] |
| ENSG00000091947 | HGLibA_49841 | 47 | -0.709562373 | TMEM101 | transmembrane protein 101 [Source:HGNC Symbol;Acc:HGNC:28653] |
| ENSG00000092929 | HGLibA_53001 | 47 | -0.709562373 | UNC13D | unc-13 homolog D [Source:HGNC Symbol;Acc:HGNC:23147] |
| ENSG00000097096 | HGLibA_47944 | 47 | -0.709562373 | SYDE2 | synapse defective Rho GTPase homolog 2 [Source:HGNC Symbol;Acc:HGNC:25841] |
| ENSG00000099991 | HGLibA_06840 | 47 | -0.709562373 | CABIN1 | calcineurin binding protein 1 [Source:HGNC Symbol;Acc:HGNC:24187] |
| ENSG00000100416 | HGLibA_51705 | 47 | -0.709562373 | TRMU | tRNA 5-methylaminomethyl-2-thiouridylate methyltransferase [Source:HGNC Symbol;Acc:HGNC:25481] |
| ENSG00000100644 | HGLibA_21296 | 47 | -0.709562373 | HIF1A | hypoxia inducible factor 1 subunit alpha [Source:HGNC Symbol;Acc:HGNC:4910] |
| ENSG00000100884 | HGLibA_10967 | 47 | -0.709562373 | CPNE6 | copine 6 [Source:HGNC Symbol;Acc:HGNC:2319] |
| ENSG00000101639 | HGLibA_09125 | 47 | -0.709562373 | CEP192 | centrosomal protein 192 [Source:HGNC Symbol;Acc:HGNC:25515] |
| ENSG00000102401 | HGLibA_03045 | 47 | -0.709562373 | ARMCX3 | armadillo repeat containing X-linked 3 [Source:HGNC Symbol;Acc:HGNC:24065] |
| ENSG00000102606 | HGLibA_02876 | 47 | -0.709562373 | ARHGEF7 | Rho guanine nucleotide exchange factor 7 [Source:HGNC Symbol;Acc:HGNC:15607] |
| ENSG00000103226 | HGLibA_32296 | 47 | -0.709562373 | NOMO3 | NODAL modulator 3 [Source:HGNC Symbol;Acc:HGNC:25242] |
| ENSG00000104852 | HGLibA_45959 | 47 | -0.709562373 | SNRNP70 | small nuclear ribonucleoprotein U1 subunit 70 [Source:HGNC Symbol;Acc:HGNC:11150] |
| ENSG00000104903 | HGLibA_27704 | 47 | -0.709562373 | LYL1 | basic helix-loop-helix family member [Source:HGNC Symbol;Acc:HGNC:6734]" |
| ENSG00000104918 | HGLibA_40876 | 47 | -0.709562373 | RETN | resistin [Source:HGNC Symbol;Acc:HGNC:20389] |
| ENSG00000105656 | HGLibA_14922 | 47 | -0.709562373 | ELL | elongation factor for RNA polymerase II [Source:HGNC Symbol;Acc:HGNC:23114] |
| ENSG00000106246 | HGLibA_39260 | 47 | -0.709562373 | PTCD1 | pentatricopeptide repeat domain 1 [Source:HGNC Symbol;Acc:HGNC:22198] |
| ENSG00000106628 | HGLibA_37496 | 47 | -0.709562373 | POLD2 | accessory subunit [Source:HGNC Symbol;Acc:HGNC:9176]" |
| ENSG00000106780 | HGLibA_28859 | 47 | -0.709562373 | MEGF9 | multiple EGF like domains 9 [Source:HGNC Symbol;Acc:HGNC:3234] |
| ENSG00000107249 | HGLibA_19276 | 47 | -0.709562373 | GLIS3 | GLIS family zinc finger 3 [Source:HGNC Symbol;Acc:HGNC:28510] |
| ENSG00000107262 | HGLibA_04075 | 47 | -0.709562373 | BAG1 | BCL2 associated athanogene 1 [Source:HGNC Symbol;Acc:HGNC:937] |
| ENSG00000108784 | HGLibA_31069 | 47 | -0.709562373 | NAGLU | N-acetyl-alpha-glucosaminidase [Source:HGNC Symbol;Acc:HGNC:7632] |
| ENSG00000110328 | HGLibA_18552 | 47 | -0.709562373 | GALNT18 | polypeptide N-acetylgalactosaminyltransferase 18 [Source:HGNC Symbol;Acc:HGNC:30488] |
| ENSG00000110888 | HGLibA_07194 | 47 | -0.709562373 | CAPRIN2 | caprin family member 2 [Source:HGNC Symbol;Acc:HGNC:21259] |
| ENSG00000111186 | HGLibA_54377 | 47 | -0.709562373 | WNT5B | Wnt family member 5B [Source:HGNC Symbol;Acc:HGNC:16265] |
| ENSG00000111358 | HGLibA_20556 | 47 | -0.709562373 | GTF2H3 | general transcription factor IIH subunit 3 [Source:HGNC Symbol;Acc:HGNC:4657] |
| ENSG00000112078 | HGLibA_24446 | 47 | -0.709562373 | KCTD20 | potassium channel tetramerization domain containing 20 [Source:HGNC Symbol;Acc:HGNC:21052] |
| ENSG00000112977 | HGLibA_12315 | 47 | -0.709562373 | DAP | death associated protein [Source:HGNC Symbol;Acc:HGNC:2672] |
| ENSG00000113282 | HGLibA_10040 | 47 | -0.709562373 | CLINT1 | clathrin interactor 1 [Source:HGNC Symbol;Acc:HGNC:23186] |
| ENSG00000113368 | HGLibA_26697 | 47 | -0.709562373 | LMNB1 | lamin B1 [Source:HGNC Symbol;Acc:HGNC:6637] |
| ENSG00000115425 | HGLibA_36020 | 47 | -0.709562373 | PECR | peroxisomal trans-2-enoyl-CoA reductase [Source:HGNC Symbol;Acc:HGNC:18281] |
| ENSG00000115568 | HGLibA_55488 | 47 | -0.709562373 | ZNF142 | zinc finger protein 142 [Source:HGNC Symbol;Acc:HGNC:12927] |
| ENSG00000115594 | HGLibA_23046 | 47 | -0.709562373 | IL1R1 | interleukin 1 receptor type 1 [Source:HGNC Symbol;Acc:HGNC:5993] |
| ENSG00000115762 | HGLibA_37090 | 47 | -0.709562373 | PLEKHB2 | pleckstrin homology domain containing B2 [Source:HGNC Symbol;Acc:HGNC:19236] |
| ENSG00000116584 | HGLibA_02834 | 47 | -0.709562373 | ARHGEF2 | Rho/Rac guanine nucleotide exchange factor 2 [Source:HGNC Symbol;Acc:HGNC:682] |
| ENSG00000116649 | HGLibA_46995 | 47 | -0.709562373 | SRM | spermidine synthase [Source:HGNC Symbol;Acc:HGNC:11296] |
| ENSG00000117632 | HGLibA_47515 | 47 | -0.709562373 | STMN1 | stathmin 1 [Source:HGNC Symbol;Acc:HGNC:6510] |
| ENSG00000117724 | HGLibA_09053 | 47 | -0.709562373 | CENPF | centromere protein F [Source:HGNC Symbol;Acc:HGNC:1857] |
| ENSG00000117906 | HGLibA_40705 | 47 | -0.709562373 | RCN2 | reticulocalbin 2 [Source:HGNC Symbol;Acc:HGNC:9935] |
| ENSG00000119414 | HGLibA_38219 | 47 | -0.709562373 | PPP6C | protein phosphatase 6 catalytic subunit [Source:HGNC Symbol;Acc:HGNC:9323] |
| ENSG00000119508 | HGLibA_32631 | 47 | -0.709562373 | NR4A3 | nuclear receptor subfamily 4 group A member 3 [Source:HGNC Symbol;Acc:HGNC:7982] |
| ENSG00000119929 | HGLibA_11800 | 47 | -0.709562373 | CUTC | cutC copper transporter [Source:HGNC Symbol;Acc:HGNC:24271] |
| ENSG00000120242 | HGLibA_22693 | 47 | -0.709562373 | IFNA8 | interferon alpha 8 [Source:HGNC Symbol;Acc:HGNC:5429] |
| ENSG00000120885 | HGLibA_10156 | 47 | -0.709562373 | CLU | clusterin [Source:HGNC Symbol;Acc:HGNC:2095] |
| ENSG00000121318 | HGLibA_48367 | 47 | -0.709562373 | TAS2R10 | taste 2 receptor member 10 [Source:HGNC Symbol;Acc:HGNC:14918] |
| ENSG00000122176 | HGLibA_17718 | 47 | -0.709562373 | FMOD | fibromodulin [Source:HGNC Symbol;Acc:HGNC:3774] |
| ENSG00000123349 | HGLibA_36147 | 47 | -0.709562373 | PFDN5 | prefoldin subunit 5 [Source:HGNC Symbol;Acc:HGNC:8869] |
| ENSG00000123636 | HGLibA_04193 | 47 | -0.709562373 | BAZ2B | bromodomain adjacent to zinc finger domain 2B [Source:HGNC Symbol;Acc:HGNC:963] |
| ENSG00000124003 | HGLibA_29623 | 47 | -0.709562373 | MOGAT1 | monoacylglycerol O-acyltransferase 1 [Source:HGNC Symbol;Acc:HGNC:18210] |
| ENSG00000124217 | HGLibA_29619 | 47 | -0.709562373 | MOCS3 | molybdenum cofactor synthesis 3 [Source:HGNC Symbol;Acc:HGNC:15765] |
| ENSG00000124587 | HGLibA_36122 | 47 | -0.709562373 | PEX6 | peroxisomal biogenesis factor 6 [Source:HGNC Symbol;Acc:HGNC:8859] |
| ENSG00000125633 | HGLibA_07930 | 47 | -0.709562373 | CCDC93 | coiled-coil domain containing 93 [Source:HGNC Symbol;Acc:HGNC:25611] |
| ENSG00000125970 | HGLibA_40175 | 47 | -0.709562373 | RALY | RALY heterogeneous nuclear ribonucleoprotein [Source:HGNC Symbol;Acc:HGNC:15921] |
| ENSG00000126970 | HGLibA_55001 | 47 | -0.709562373 | ZC4H2 | zinc finger C4H2-type containing [Source:HGNC Symbol;Acc:HGNC:24931] |
| ENSG00000127928 | HGLibA_19538 | 47 | -0.709562373 | GNGT1 | G protein subunit gamma transducin 1 [Source:HGNC Symbol;Acc:HGNC:4411] |
| ENSG00000129451 | HGLibA_25234 | 47 | -0.709562373 | KLK10 | kallikrein related peptidase 10 [Source:HGNC Symbol;Acc:HGNC:6358] |
| ENSG00000129667 | HGLibA_41135 | 47 | -0.709562373 | RHBDF2 | rhomboid 5 homolog 2 [Source:HGNC Symbol;Acc:HGNC:20788] |
| ENSG00000130700 | HGLibA_18676 | 47 | -0.709562373 | GATA5 | GATA binding protein 5 [Source:HGNC Symbol;Acc:HGNC:15802] |
| ENSG00000130766 | HGLibA_43623 | 47 | -0.709562373 | SESN2 | sestrin 2 [Source:HGNC Symbol;Acc:HGNC:20746] |
| ENSG00000130779 | HGLibA_10042 | 47 | -0.709562373 | CLIP1 | CAP-Gly domain containing linker protein 1 [Source:HGNC Symbol;Acc:HGNC:10461] |
| ENSG00000130818 | HGLibA_55965 | 47 | -0.709562373 | ZNF426 | zinc finger protein 426 [Source:HGNC Symbol;Acc:HGNC:20725] |
| ENSG00000132635 | HGLibA_35582 | 47 | -0.709562373 | PCED1A | PC-esterase domain containing 1A [Source:HGNC Symbol;Acc:HGNC:16212] |
| ENSG00000134759 | HGLibA_14977 | 47 | -0.709562373 | ELP2 | elongator acetyltransferase complex subunit 2 [Source:HGNC Symbol;Acc:HGNC:18248] |
| ENSG00000134779 | HGLibA_51125 | 47 | -0.709562373 | TPGS2 | tubulin polyglutamylase complex subunit 2 [Source:HGNC Symbol;Acc:HGNC:24561] |
| ENSG00000134817 | HGLibA_02438 | 47 | -0.709562373 | APLNR | apelin receptor [Source:HGNC Symbol;Acc:HGNC:339] |
| ENSG00000135119 | HGLibA_41706 | 47 | -0.709562373 | RNFT2 | transmembrane 2 [Source:HGNC Symbol;Acc:HGNC:25905]" |
| ENSG00000135577 | HGLibA_32145 | 47 | -0.709562373 | NMBR | neuromedin B receptor [Source:HGNC Symbol;Acc:HGNC:7843] |
| ENSG00000136068 | HGLibA_17643 | 47 | -0.709562373 | FLNB | filamin B [Source:HGNC Symbol;Acc:HGNC:3755] |
| ENSG00000136286 | HGLibA_30837 | 47 | -0.709562373 | MYO1G | myosin IG [Source:HGNC Symbol;Acc:HGNC:13880] |
| ENSG00000137817 | HGLibA_35241 | 47 | -0.709562373 | PARP6 | poly(ADP-ribose) polymerase family member 6 [Source:HGNC Symbol;Acc:HGNC:26921] |
| ENSG00000138071 | HGLibA_00657 | 47 | -0.709562373 | ACTR2 | ARP2 actin related protein 2 homolog [Source:HGNC Symbol;Acc:HGNC:169] |
| ENSG00000139610 | HGLibA_08988 | 47 | -0.709562373 | CELA1 | chymotrypsin like elastase family member 1 [Source:HGNC Symbol;Acc:HGNC:3308] |
| ENSG00000139624 | HGLibA_09212 | 47 | -0.709562373 | CERS5 | ceramide synthase 5 [Source:HGNC Symbol;Acc:HGNC:23749] |
| ENSG00000140612 | HGLibA_43182 | 47 | -0.709562373 | SEC11A | signal peptidase complex subunit [Source:HGNC Symbol;Acc:HGNC:17718]" |
| ENSG00000140873 | HGLibA_00815 | 47 | -0.709562373 | ADAMTS18 | ADAM metallopeptidase with thrombospondin type 1 motif 18 [Source:HGNC Symbol;Acc:HGNC:17110] |
| ENSG00000141527 | HGLibA_07224 | 47 | -0.709562373 | CARD14 | caspase recruitment domain family member 14 [Source:HGNC Symbol;Acc:HGNC:16446] |
| ENSG00000141655 | HGLibA_50746 | 47 | -0.709562373 | TNFRSF11A | TNF receptor superfamily member 11a [Source:HGNC Symbol;Acc:HGNC:11908] |
| ENSG00000143367 | HGLibA_52326 | 47 | -0.709562373 | TUFT1 | tuftelin 1 [Source:HGNC Symbol;Acc:HGNC:12422] |
| ENSG00000143434 | HGLibA_43370 | 47 | -0.709562373 | SEMA6C | semaphorin 6C [Source:HGNC Symbol;Acc:HGNC:10740] |
| ENSG00000143476 | HGLibA_14062 | 47 | -0.709562373 | DTL | denticleless E3 ubiquitin protein ligase homolog [Source:HGNC Symbol;Acc:HGNC:30288] |
| ENSG00000143498 | HGLibA_48212 | 47 | -0.709562373 | TAF1A | RNA polymerase I subunit A [Source:HGNC Symbol;Acc:HGNC:11532]" |
| ENSG00000143799 | HGLibA_35210 | 47 | -0.709562373 | PARP1 | poly(ADP-ribose) polymerase 1 [Source:HGNC Symbol;Acc:HGNC:270] |
| ENSG00000143889 | HGLibA_21837 | 47 | -0.709562373 | HNRNPLL | heterogeneous nuclear ribonucleoprotein L like [Source:HGNC Symbol;Acc:HGNC:25127] |
| ENSG00000143970 | HGLibA_03393 | 47 | -0.709562373 | ASXL2 | ASXL transcriptional regulator 2 [Source:HGNC Symbol;Acc:HGNC:23805] |
| ENSG00000145721 | HGLibA_26643 | 47 | -0.709562373 | LIX1 | limb and CNS expressed 1 [Source:HGNC Symbol;Acc:HGNC:18581] |
| ENSG00000145945 | HGLibA_16631 | 47 | -0.709562373 | FAM50B | family with sequence similarity 50 member B [Source:HGNC Symbol;Acc:HGNC:18789] |
| ENSG00000147065 | HGLibA_30231 | 47 | -0.709562373 | MSN | moesin [Source:HGNC Symbol;Acc:HGNC:7373] |
| ENSG00000147119 | HGLibA_09647 | 47 | -0.709562373 | CHST7 | carbohydrate sulfotransferase 7 [Source:HGNC Symbol;Acc:HGNC:13817] |
| ENSG00000148925 | HGLibA_04897 | 47 | -0.709562373 | BTBD10 | BTB domain containing 10 [Source:HGNC Symbol;Acc:HGNC:21445] |
| ENSG00000148925 | HGLibA_04896 | 47 | -0.709562373 | BTBD10 | BTB domain containing 10 [Source:HGNC Symbol;Acc:HGNC:21445] |
| ENSG00000149313 | HGLibA_00080 | 47 | -0.709562373 | AASDHPPT | aminoadipate-semialdehyde dehydrogenase-phosphopantetheinyl transferase [Source:HGNC Symbol;Acc:HGNC:14235] |
| ENSG00000151292 | HGLibA_11399 | 47 | -0.709562373 | CSNK1G3 | casein kinase 1 gamma 3 [Source:HGNC Symbol;Acc:HGNC:2456] |
| ENSG00000151572 | HGLibA_02179 | 47 | -0.709562373 | ANO4 | anoctamin 4 [Source:HGNC Symbol;Acc:HGNC:23837] |
| ENSG00000152217 | HGLibA_43635 | 47 | -0.709562373 | SETBP1 | SET binding protein 1 [Source:HGNC Symbol;Acc:HGNC:15573] |
| ENSG00000152292 | HGLibA_43908 | 47 | -0.709562373 | SH2D6 | SH2 domain containing 6 [Source:HGNC Symbol;Acc:HGNC:30439] |
| ENSG00000154305 | HGLibA_29189 | 47 | -0.709562373 | MIA3 | MIA SH3 domain ER export factor 3 [Source:HGNC Symbol;Acc:HGNC:24008] |
| ENSG00000155636 | HGLibA_40559 | 47 | -0.709562373 | RBM45 | RNA binding motif protein 45 [Source:HGNC Symbol;Acc:HGNC:24468] |
| ENSG00000157766 | HGLibA_00380 | 47 | -0.709562373 | ACAN | aggrecan [Source:HGNC Symbol;Acc:HGNC:319] |
| ENSG00000157796 | HGLibA_54042 | 47 | -0.709562373 | WDR19 | WD repeat domain 19 [Source:HGNC Symbol;Acc:HGNC:18340] |
| ENSG00000157884 | HGLibA_09691 | 47 | -0.709562373 | CIB4 | calcium and integrin binding family member 4 [Source:HGNC Symbol;Acc:HGNC:33703] |
| ENSG00000158623 | HGLibA_10707 | 47 | -0.709562373 | COPG2 | coatomer protein complex subunit gamma 2 [Source:HGNC Symbol;Acc:HGNC:2237] |
| ENSG00000160472 | HGLibA_50140 | 47 | -0.709562373 | TMEM190 | transmembrane protein 190 [Source:HGNC Symbol;Acc:HGNC:29632] |
| ENSG00000161980 | HGLibA_37635 | 47 | -0.709562373 | POLR3K | RNA polymerase III subunit K [Source:HGNC Symbol;Acc:HGNC:14121] |
| ENSG00000162365 | HGLibA_12174 | 47 | -0.709562373 | CYP4A22 | cytochrome P450 family 4 subfamily A member 22 [Source:HGNC Symbol;Acc:HGNC:20575] |
| ENSG00000162434 | HGLibA_23912 | 47 | -0.709562373 | JAK1 | Janus kinase 1 [Source:HGNC Symbol;Acc:HGNC:6190] |
| ENSG00000163701 | HGLibA_23017 | 47 | -0.709562373 | IL17RE | interleukin 17 receptor E [Source:HGNC Symbol;Acc:HGNC:18439] |
| ENSG00000163794 | HGLibA_52821 | 47 | -0.709562373 | UCN | urocortin [Source:HGNC Symbol;Acc:HGNC:12516] |
| ENSG00000164308 | HGLibA_15407 | 47 | -0.709562373 | ERAP2 | endoplasmic reticulum aminopeptidase 2 [Source:HGNC Symbol;Acc:HGNC:29499] |
| ENSG00000164362 | HGLibA_49020 | 47 | -0.709562373 | TERT | telomerase reverse transcriptase [Source:HGNC Symbol;Acc:HGNC:11730] |
| ENSG00000164744 | HGLibA_47794 | 47 | -0.709562373 | SUN3 | Sad1 and UNC84 domain containing 3 [Source:HGNC Symbol;Acc:HGNC:22429] |
| ENSG00000165105 | HGLibA_40325 | 47 | -0.709562373 | RASEF | RAS and EF-hand domain containing [Source:HGNC Symbol;Acc:HGNC:26464] |
| ENSG00000165202 | HGLibA_33709 | 47 | -0.709562373 | OR1Q1 | olfactory receptor family 1 subfamily Q member 1 [Source:HGNC Symbol;Acc:HGNC:8223] |
| ENSG00000165494 | HGLibA_35589 | 47 | -0.709562373 | PCF11 | cleavage and polyadenylation factor subunit [Source:HGNC Symbol;Acc:HGNC:30097]" |
| ENSG00000166261 | HGLibA_55572 | 47 | -0.709562373 | ZNF202 | zinc finger protein 202 [Source:HGNC Symbol;Acc:HGNC:12994] |
| ENSG00000166333 | HGLibA_23221 | 47 | -0.709562373 | ILK | integrin linked kinase [Source:HGNC Symbol;Acc:HGNC:6040] |
| ENSG00000166578 | HGLibA_23496 | 47 | -0.709562373 | IQCD | IQ motif containing D [Source:HGNC Symbol;Acc:HGNC:25168] |
| ENSG00000166825 | HGLibA_02211 | 47 | -0.709562373 | ANPEP | membrane [Source:HGNC Symbol;Acc:HGNC:500]" |
| ENSG00000167360 | HGLibA_34127 | 47 | -0.709562373 | OR51Q1 | olfactory receptor family 51 subfamily Q member 1 (gene/pseudogene) [Source:HGNC Symbol;Acc:HGNC:14851] |
| ENSG00000167779 | HGLibA_22831 | 47 | -0.709562373 | IGFBP6 | insulin like growth factor binding protein 6 [Source:HGNC Symbol;Acc:HGNC:5475] |
| ENSG00000167996 | HGLibA_18117 | 47 | -0.709562373 | FTH1 | ferritin heavy chain 1 [Source:HGNC Symbol;Acc:HGNC:3976] |
| ENSG00000168090 | HGLibA_10725 | 47 | -0.709562373 | COPS6 | COP9 signalosome subunit 6 [Source:HGNC Symbol;Acc:HGNC:21749] |
| ENSG00000168826 | HGLibA_54908 | 47 | -0.709562373 | ZBTB49 | zinc finger and BTB domain containing 49 [Source:HGNC Symbol;Acc:HGNC:19883] |
| ENSG00000169495 | HGLibA_22416 | 47 | -0.709562373 | HTRA4 | HtrA serine peptidase 4 [Source:HGNC Symbol;Acc:HGNC:26909] |
| ENSG00000169562 | HGLibA_19156 | 47 | -0.709562373 | GJB1 | gap junction protein beta 1 [Source:HGNC Symbol;Acc:HGNC:4283] |
| ENSG00000169856 | HGLibA_33391 | 47 | -0.709562373 | ONECUT1 | one cut homeobox 1 [Source:HGNC Symbol;Acc:HGNC:8138] |
| ENSG00000169925 | HGLibA_04775 | 47 | -0.709562373 | BRD3 | bromodomain containing 3 [Source:HGNC Symbol;Acc:HGNC:1104] |
| ENSG00000170236 | HGLibA_53393 | 47 | -0.709562373 | USP50 | ubiquitin specific peptidase 50 [Source:HGNC Symbol;Acc:HGNC:20079] |
| ENSG00000170296 | HGLibA_18331 | 47 | -0.709562373 | GABARAP | GABA type A receptor-associated protein [Source:HGNC Symbol;Acc:HGNC:4067] |

| | | | | | |
|---|---|---|---|---|---|
| ENSG00000170421 | HGLibA_25551 | 47 | -0.709562373 | KRT8 | keratin 8 [Source:HGNC Symbol;Acc:HGNC:6446] |
| ENSG00000170777 | HGLibA_51119 | 47 | -0.709562373 | TPD52L3 | TPD52 like 3 [Source:HGNC Symbol;Acc:HGNC:23382] |
| ENSG00000170807 | HGLibA_26720 | 47 | -0.709562373 | LMOD2 | leiomodin 2 [Source:HGNC Symbol;Acc:HGNC:6648] |
| ENSG00000170819 | HGLibA_04458 | 47 | -0.709562373 | BFSP2 | beaded filament structural protein 2 [Source:HGNC Symbol;Acc:HGNC:1041] |
| ENSG00000171711 | HGLibA_12915 | 47 | -0.709562373 | DEFB4A | defensin beta 4A [Source:HGNC Symbol;Acc:HGNC:2767] |
| ENSG00000171855 | HGLibA_22702 | 47 | -0.709562373 | IFNB1 | interferon beta 1 [Source:HGNC Symbol;Acc:HGNC:5434] |
| ENSG00000172199 | HGLibA_34555 | 47 | -0.709562373 | OR8U1 | olfactory receptor family 8 subfamily U member 1 [Source:HGNC Symbol;Acc:HGNC:19611] |
| ENSG00000172456 | HGLibA_17458 | 47 | -0.709562373 | FGGY | FGGY carbohydrate kinase domain containing [Source:HGNC Symbol;Acc:HGNC:25610] |
| ENSG00000173846 | HGLibA_37194 | 47 | -0.709562373 | PLK3 | polo like kinase 3 [Source:HGNC Symbol;Acc:HGNC:2154] |
| ENSG00000174099 | HGLibA_30248 | 47 | -0.709562373 | MSRB3 | methionine sulfoxide reductase B3 [Source:HGNC Symbol;Acc:HGNC:27375] |
| ENSG00000174306 | HGLibA_55317 | 47 | -0.709562373 | ZHX3 | zinc fingers and homeoboxes 3 [Source:HGNC Symbol;Acc:HGNC:15935] |
| ENSG00000174946 | HGLibA_19966 | 47 | -0.709562373 | GPR171 | G protein-coupled receptor 171 [Source:HGNC Symbol;Acc:HGNC:30057] |
| ENSG00000175221 | HGLibA_28760 | 47 | -0.709562373 | MED16 | mediator complex subunit 16 [Source:HGNC Symbol;Acc:HGNC:17556] |
| ENSG00000175229 | HGLibA_18504 | 47 | -0.709562373 | GAL3ST3 | galactose-3-O-sulfotransferase 3 [Source:HGNC Symbol;Acc:HGNC:24144] |
| ENSG00000176102 | HGLibA_11491 | 47 | -0.709562373 | CSTF3 | cleavage stimulation factor subunit 3 [Source:HGNC Symbol;Acc:HGNC:2485] |
| ENSG00000176108 | HGLibA_09499 | 47 | -0.709562373 | CHMP6 | charged multivesicular body protein 6 [Source:HGNC Symbol;Acc:HGNC:25675] |
| ENSG00000176222 | HGLibA_55923 | 47 | -0.709562373 | ZNF404 | zinc finger protein 404 [Source:HGNC Symbol;Acc:HGNC:19417] |
| ENSG00000178685 | HGLibA_35213 | 47 | -0.709562373 | PARP10 | poly(ADP-ribose) polymerase family member 10 [Source:HGNC Symbol;Acc:HGNC:25895] |
| ENSG00000179002 | HGLibA_48358 | 47 | -0.709562373 | TAS1R2 | taste 1 receptor member 2 [Source:HGNC Symbol;Acc:HGNC:14905] |
| ENSG00000181638 | HGLibA_55215 | 47 | -0.709562373 | ZFP41 | ZFP41 zinc finger protein [Source:HGNC Symbol;Acc:HGNC:26786] |
| ENSG00000181958 | HGLibA_33924 | 47 | -0.709562373 | OR4A15 | olfactory receptor family 4 subfamily A member 15 [Source:HGNC Symbol;Acc:HGNC:15152] |
| ENSG00000183150 | HGLibA_19994 | 47 | -0.709562373 | GPR19 | G protein-coupled receptor 19 [Source:HGNC Symbol;Acc:HGNC:4473] |
| ENSG00000183155 | HGLibA_40025 | 47 | -0.709562373 | RABIF | RAB interacting factor [Source:HGNC Symbol;Acc:HGNC:9797] |
| ENSG00000183395 | HGLibA_37293 | 47 | -0.709562373 | PMCH | pro-melanin concentrating hormone [Source:HGNC Symbol;Acc:HGNC:9109] |
| ENSG00000183397 | HGLibA_05703 | 47 | -0.709562373 | C19orf71 | chromosome 19 open reading frame 71 [Source:HGNC Symbol;Acc:HGNC:34496] |
| ENSG00000184481 | HGLibA_17927 | 47 | -0.709562373 | FOXO4 | forkhead box O4 [Source:HGNC Symbol;Acc:HGNC:7139] |
| ENSG00000185024 | HGLibA_04797 | 47 | -0.709562373 | BRF1 | RNA polymerase III transcription initiation factor subunit [Source:HGNC Symbol;Acc:HGNC:11551]" |
| ENSG00000185187 | HGLibA_44121 | 47 | -0.709562373 | SIGIRR | single Ig and TIR domain containing [Source:HGNC Symbol;Acc:HGNC:30575] |
| ENSG00000185352 | HGLibA_22149 | 47 | -0.709562373 | HS6ST3 | heparan sulfate 6-O-sulfotransferase 3 [Source:HGNC Symbol;Acc:HGNC:19134] |
| ENSG00000185689 | HGLibA_06467 | 47 | -0.709562373 | C6orf201 | chromosome 6 open reading frame 201 [Source:HGNC Symbol;Acc:HGNC:21620] |
| ENSG00000185880 | HGLibA_51593 | 47 | -0.709562373 | TRIM69 | tripartite motif containing 69 [Source:HGNC Symbol;Acc:HGNC:17857] |
| ENSG00000186526 | HGLibA_12197 | 47 | -0.709562373 | CYP4F8 | cytochrome P450 family 4 subfamily F member 8 [Source:HGNC Symbol;Acc:HGNC:2648] |
| ENSG00000186599 | HGLibA_12827 | 47 | -0.709562373 | DEFB105B | defensin beta 105B [Source:HGNC Symbol;Acc:HGNC:29930] |
| ENSG00000186970 | HGLibA_25653 | 47 | -0.709562373 | KRTAP15-1 | keratin associated protein 15-1 [Source:HGNC Symbol;Acc:HGNC:18927] |
| ENSG00000188042 | HGLibA_02970 | 47 | -0.709562373 | ARL4C | ADP ribosylation factor like GTPase 4C [Source:HGNC Symbol;Acc:HGNC:698] |
| ENSG00000188051 | HGLibA_50237 | 47 | -0.709562373 | TMEM221 | transmembrane protein 221 [Source:HGNC Symbol;Acc:HGNC:21943] |
| ENSG00000188191 | HGLibA_38524 | 47 | -0.709562373 | PRKAR1B | protein kinase cAMP-dependent type I regulatory subunit beta [Source:HGNC Symbol;Acc:HGNC:9390] |
| ENSG00000188295 | HGLibA_56497 | 47 | -0.709562373 | ZNF669 | zinc finger protein 669 [Source:HGNC Symbol;Acc:HGNC:25736] |
| ENSG00000188672 | HGLibA_41144 | 47 | -0.709562373 | RHCE | Rh blood group CcEe antigens [Source:HGNC Symbol;Acc:HGNC:10008] |
| ENSG00000196338 | HGLibA_32072 | 47 | -0.709562373 | NLGN3 | neuroligin 3 [Source:HGNC Symbol;Acc:HGNC:14289] |
| ENSG00000196345 | HGLibA_55361 | 47 | -0.709562373 | ZKSCAN7 | zinc finger with KRAB and SCAN domains 7 [Source:HGNC Symbol;Acc:HGNC:12955] |
| ENSG00000196873 | HGLibA_07436 | 47 | -0.709562373 | CBWD3 | COBW domain containing 3 [Source:HGNC Symbol;Acc:HGNC:18519] |
| ENSG00000197157 | HGLibA_45920 | 47 | -0.709562373 | SND1 | staphylococcal nuclease and tudor domain containing 1 [Source:HGNC Symbol;Acc:HGNC:30646] |
| ENSG00000197408 | HGLibA_12112 | 47 | -0.709562373 | CYP2B6 | cytochrome P450 family 2 subfamily B member 6 [Source:HGNC Symbol;Acc:HGNC:2615] |
| ENSG00000197415 | HGLibA_53623 | 47 | -0.709562373 | VEPH1 | ventricular zone expressed PH domain containing 1 [Source:HGNC Symbol;Acc:HGNC:25735] |
| ENSG00000198021 | HGLibA_46377 | 47 | -0.709562373 | SPANXA1 | family member A1 [Source:HGNC Symbol;Acc:HGNC:11218]" |
| ENSG00000198055 | HGLibA_20335 | 47 | -0.709562373 | GRK6 | G protein-coupled receptor kinase 6 [Source:HGNC Symbol;Acc:HGNC:4545] |
| ENSG00000198326 | HGLibA_50283 | 47 | -0.709562373 | TMEM239 | transmembrane protein 239 [Source:HGNC Symbol;Acc:HGNC:40044] |
| ENSG00000198400 | HGLibA_32873 | 47 | -0.709562373 | NTRK1 | neurotrophic receptor tyrosine kinase 1 [Source:HGNC Symbol;Acc:HGNC:8031] |
| ENSG00000198754 | HGLibA_34815 | 47 | -0.709562373 | OXCT2 | 3-oxoacid CoA-transferase 2 [Source:HGNC Symbol;Acc:HGNC:18606] |
| ENSG00000198826 | HGLibA_02701 | 47 | -0.709562373 | ARHGAP11A | Rho GTPase activating protein 11A [Source:HGNC Symbol;Acc:HGNC:15783] |
| ENSG00000203661 | HGLibA_33886 | 47 | -0.709562373 | OR2T5 | olfactory receptor family 2 subfamily T member 5 [Source:HGNC Symbol;Acc:HGNC:15017] |
| ENSG00000203880 | HGLibA_35631 | 47 | -0.709562373 | PCMTD2 | protein-L-isoaspartate (D-aspartate) O-methyltransferase domain containing 2 [Source:HGNC Symbol;Acc:HGNC:15882] |
| ENSG00000204381 | HGLibA_26074 | 47 | -0.709562373 | LAYN | layilin [Source:HGNC Symbol;Acc:HGNC:29471] |
| ENSG00000204977 | HGLibA_51407 | 47 | -0.709562373 | TRIM13 | tripartite motif containing 13 [Source:HGNC Symbol;Acc:HGNC:9976] |
| ENSG00000205213 | HGLibA_26408 | 47 | -0.709562373 | LGR4 | leucine rich repeat containing G protein-coupled receptor 4 [Source:HGNC Symbol;Acc:HGNC:13299] |
| ENSG00000205667 | HGLibA_03147 | 47 | -0.709562373 | ARSH | arylsulfatase family member H [Source:HGNC Symbol;Acc:HGNC:32488] |
| ENSG00000205669 | HGLibA_00486 | 47 | -0.709562373 | ACOT6 | acyl-CoA thioesterase 6 [Source:HGNC Symbol;Acc:HGNC:33159] |
| ENSG00000205835 | HGLibA_19414 | 47 | -0.709562373 | GMNC | geminin coiled-coil domain containing [Source:HGNC Symbol;Acc:HGNC:40049] |
| ENSG00000205944 | HGLibA_12356 | 47 | -0.709562373 | DAZ2 | deleted in azoospermia 2 [Source:HGNC Symbol;Acc:HGNC:15964] |
| ENSG00000206026 | HGLibA_45748 | 47 | -0.709562373 | SMIM21 | small integral membrane protein 21 [Source:HGNC Symbol;Acc:HGNC:27598] |
| ENSG00000212724 | HGLibA_25692 | 47 | -0.709562373 | KRTAP2-3 | keratin associated protein 2-3 [Source:HGNC Symbol;Acc:HGNC:18906] |
| ENSG00000213859 | HGLibA_24417 | 47 | -0.709562373 | KCTD11 | potassium channel tetramerization domain containing 11 [Source:HGNC Symbol;Acc:HGNC:21302] |
| ENSG00000215131 | HGLibA_05498 | 47 | -0.709562373 | C16orf90 | chromosome 16 open reading frame 90 [Source:HGNC Symbol;Acc:HGNC:34455] |
| ENSG00000215915 | HGLibA_03416 | 47 | -0.709562373 | ATAD3C | AAA domain containing 3C [Source:HGNC Symbol;Acc:HGNC:32151]" |
| ENSG00000233436 | HGLibA_04909 | 47 | -0.709562373 | BTBD18 | BTB domain containing 18 [Source:HGNC Symbol;Acc:HGNC:37214] |
| ENSG00000241476 | HGLibA_47198 | 47 | -0.709562373 | SSX2 | SSX family member 2 [Source:HGNC Symbol;Acc:HGNC:11336] |
| ENSG00000241685 | HGLibA_03073 | 47 | -0.709562373 | ARPC1A | actin related protein 2/3 complex subunit 1A [Source:HGNC Symbol;Acc:HGNC:703] |
| ENSG00000243056 | HGLibA_14835 | 47 | -0.709562373 | EIF4EBP3 | eukaryotic translation initiation factor 4E binding protein 3 [Source:HGNC Symbol;Acc:HGNC:3290] |
| ENSG00000244509 | HGLibA_02483 | 47 | -0.709562373 | APOBEC3C | apolipoprotein B mRNA editing enzyme catalytic subunit 3C [Source:HGNC Symbol;Acc:HGNC:17353] |
| ENSG00000244752 | HGLibA_11263 | 47 | -0.709562373 | CRYBB2 | crystallin beta B2 [Source:HGNC Symbol;Acc:HGNC:2398] |
| ENSG00000248098 | HGLibA_04279 | 47 | -0.709562373 | BCKDHA | alpha polypeptide [Source:HGNC Symbol;Acc:HGNC:986]" |
| ENSG00000250317 | HGLibA_45745 | 47 | -0.709562373 | SMIM20 | small integral membrane protein 20 [Source:HGNC Symbol;Acc:HGNC:37260] |
| ENSG00000251655 | HGLibA_38338 | 47 | -0.709562373 | PRB1 | proline rich protein BstNI subfamily 1 [Source:HGNC Symbol;Acc:HGNC:9337] |
| ENSG00000256660 | HGLibA_09930 | 47 | -0.709562373 | CLEC12B | C-type lectin domain family 12 member B [Source:HGNC Symbol;Acc:HGNC:31966] |
| ENSG00000260903 | HGLibA_54529 | 47 | -0.709562373 | XKR7 | XK related 7 [Source:HGNC Symbol;Acc:HGNC:23062] |
| ENSG00000261052 | HGLibA_47738 | 47 | -0.709562373 | SULT1A3 | sulfotransferase family 1A member 3 [Source:HGNC Symbol;Acc:HGNC:11455] |
| ENSG00000271858 | HGLibA_11987 | 47 | -0.709562373 | CYB561D2 | cytochrome b561 family member D2 [Source:NCBI gene;Acc:11068] |
| ENSG00000273841 | HGLibA_48260 | 47 | -0.709562373 | TAF9 | TATA-box binding protein associated factor 9 [Source:HGNC Symbol;Acc:HGNC:11542] |
| ENSG00000276430 | HGLibA_16556 | 47 | -0.709562373 | FAM25C | family with sequence similarity 25 member C [Source:HGNC Symbol;Acc:HGNC:23586] |
| FAIM3 | HGLibA_15970 | 47 | -0.709562373 | FAIM3 | - |
| FAM211A | HGLibA_16458 | 47 | -0.709562373 | FAM211A | - |
| FLJ44635 | HGLibA_17634 | 47 | -0.709562373 | FLJ44635 | - |
| GTPBP6_X | HGLibA_20617 | 47 | -0.709562373 | GTPBP6_X | - |
| hsa-mir-1-1 | HGLibA_57143 | 47 | -0.709562373 | hsa-mir-1-1 | - |
| hsa-mir-1267 | HGLibA_57493 | 47 | -0.709562373 | hsa-mir-1267 | - |
| hsa-mir-1281 | HGLibA_57582 | 47 | -0.709562373 | hsa-mir-1281 | - |
| hsa-mir-147a | HGLibA_57873 | 47 | -0.709562373 | hsa-mir-147a | - |
| hsa-mir-193a | HGLibA_58074 | 47 | -0.709562373 | hsa-mir-193a | - |
| hsa-mir-2052 | HGLibA_58181 | 47 | -0.709562373 | hsa-mir-2052 | - |
| hsa-mir-3065 | HGLibA_58464 | 47 | -0.709562373 | hsa-mir-3065 | - |
| hsa-mir-30c-1 | HGLibA_58479 | 47 | -0.709562373 | hsa-mir-30c-1 | - |
| hsa-mir-3119-1 | HGLibA_58525 | 47 | -0.709562373 | hsa-mir-3119-1 | - |
| hsa-mir-3154 | HGLibA_58675 | 47 | -0.709562373 | hsa-mir-3154 | - |
| hsa-mir-3157 | HGLibA_58698 | 47 | -0.709562373 | hsa-mir-3157 | - |
| hsa-mir-373 | HGLibA_59387 | 47 | -0.709562373 | hsa-mir-373 | - |
| hsa-mir-378j | HGLibA_59465 | 47 | -0.709562373 | hsa-mir-378j | - |
| hsa-mir-4255 | HGLibA_59729 | 47 | -0.709562373 | hsa-mir-4255 | - |
| hsa-mir-4257 | HGLibA_59737 | 47 | -0.709562373 | hsa-mir-4257 | - |
| hsa-mir-4283-1 | HGLibA_59835 | 47 | -0.709562373 | hsa-mir-4283-1 | - |
| hsa-mir-4432 | HGLibA_60115 | 47 | -0.709562373 | hsa-mir-4432 | - |
| hsa-mir-4432 | HGLibA_60116 | 47 | -0.709562373 | hsa-mir-4432 | - |
| hsa-mir-4433 | HGLibA_60119 | 47 | -0.709562373 | hsa-mir-4433 | - |
| hsa-mir-4441 | HGLibA_60165 | 47 | -0.709562373 | hsa-mir-4441 | - |
| hsa-mir-4447 | HGLibA_60196 | 47 | -0.709562373 | hsa-mir-4447 | - |
| hsa-mir-4482 | HGLibA_60348 | 47 | -0.709562373 | hsa-mir-4482 | - |
| hsa-mir-4502 | HGLibA_60433 | 47 | -0.709562373 | hsa-mir-4502 | - |
| hsa-mir-4520a | HGLibA_60533 | 47 | -0.709562373 | hsa-mir-4520a | - |
| hsa-mir-4642 | HGLibA_60674 | 47 | -0.709562373 | hsa-mir-4642 | - |
| hsa-mir-4676 | HGLibA_60822 | 47 | -0.709562373 | hsa-mir-4676 | - |
| hsa-mir-4682 | HGLibA_60840 | 47 | -0.709562373 | hsa-mir-4682 | - |
| hsa-mir-4777 | HGLibA_61217 | 47 | -0.709562373 | hsa-mir-4777 | - |
| hsa-mir-4781 | HGLibA_61231 | 47 | -0.709562373 | hsa-mir-4781 | - |
| hsa-mir-5004 | HGLibA_61425 | 47 | -0.709562373 | hsa-mir-5004 | - |
| hsa-mir-548d-2 | HGLibA_61966 | 47 | -0.709562373 | hsa-mir-548d-2 | - |
| hsa-mir-562 | HGLibA_62210 | 47 | -0.709562373 | hsa-mir-562 | - |
| hsa-mir-6089-1 | HGLibA_62615 | 47 | -0.709562373 | hsa-mir-6089-1 | - |
| hsa-mir-671 | HGLibA_63013 | 47 | -0.709562373 | hsa-mir-671 | - |
| hsa-mir-6715a | HGLibA_63018 | 47 | -0.709562373 | hsa-mir-6715a | - |
| hsa-mir-6779 | HGLibA_63290 | 47 | -0.709562373 | hsa-mir-6779 | - |
| hsa-mir-6836 | HGLibA_63520 | 47 | -0.709562373 | hsa-mir-6836 | - |
| hsa-mir-6885 | HGLibA_63726 | 47 | -0.709562373 | hsa-mir-6885 | - |
| hsa-mir-892a | HGLibA_64251 | 47 | -0.709562373 | hsa-mir-892a | - |
| KIAA1024L | HGLibA_24683 | 47 | -0.709562373 | KIAA1024L | - |
| KIAA1407 | HGLibA_24747 | 47 | -0.709562373 | KIAA1407 | - |
| LOR | HGLibA_27030 | 47 | -0.709562373 | LOR | - |
| LPHN1 | HGLibA_27086 | 47 | -0.709562373 | LPHN1 | - |
| MUT | HGLibA_30584 | 47 | -0.709562373 | MUT | - |
| PRHOXNB | HGLibA_38467 | 47 | -0.709562373 | PRHOXNB | - |
| VIMP | HGLibA_53658 | 47 | -0.709562373 | VIMP | - |
| ACPT | HGLibA_00527 | 46 | -0.718607716 | ACPT | - |
| ADRBK1 | HGLibA_01063 | 46 | -0.718607716 | ADRBK1 | - |
| ASMT_Y | HGLibA_03324 | 46 | -0.718607716 | ASMT_Y | - |
| C10orf2 | HGLibA_05061 | 46 | -0.718607716 | C10orf2 | - |
| C7orf69 | HGLibA_06581 | 46 | -0.718607716 | C7orf69 | - |

| | | | | | |
|---|---|---|---|---|---|
| CXorf27 | HGLibA_11911 | 46 | -0.718607716 | CXorf27 | - |
| ENSG00000007312 | HGLibA_08450 | 46 | -0.718607716 | CD79B | CD79b molecule [Source:HGNC Symbol;Acc:HGNC:1699] |
| ENSG00000011405 | HGLibA_36622 | 46 | -0.718607716 | PIK3C2A | phosphatidylinositol-4-phosphate 3-kinase catalytic subunit type 2 alpha [Source:HGNC Symbol;Acc:HGNC:8971] |
| ENSG00000013573 | HGLibA_12653 | 46 | -0.718607716 | DDX11 | DEAD/H-box helicase 11 [Source:HGNC Symbol;Acc:HGNC:2736] |
| ENSG00000020426 | HGLibA_29561 | 46 | -0.718607716 | MNAT1 | CDK activating kinase assembly factor [Source:HGNC Symbol;Acc:HGNC:7181]" |
| ENSG00000034239 | HGLibA_14519 | 46 | -0.718607716 | EFCAB1 | EF-hand calcium binding domain 1 [Source:HGNC Symbol;Acc:HGNC:25678] |
| ENSG00000038382 | HGLibA_51628 | 46 | -0.718607716 | TRIO | trio Rho guanine nucleotide exchange factor [Source:HGNC Symbol;Acc:HGNC:12303] |
| ENSG00000047410 | HGLibA_51173 | 46 | -0.718607716 | TPR | nuclear basket protein [Source:HGNC Symbol;Acc:HGNC:12017]" |
| ENSG00000048342 | HGLibA_07481 | 46 | -0.718607716 | CC2D2A | coiled-coil and C2 domain containing 2A [Source:HGNC Symbol;Acc:HGNC:29253] |
| ENSG00000063245 | HGLibA_15342 | 46 | -0.718607716 | EPN1 | epsin 1 [Source:HGNC Symbol;Acc:HGNC:21604] |
| ENSG00000069535 | HGLibA_28110 | 46 | -0.718607716 | MAOB | monoamine oxidase B [Source:HGNC Symbol;Acc:HGNC:6834] |
| ENSG00000069764 | HGLibA_36867 | 46 | -0.718607716 | PLA2G10 | phospholipase A2 group X [Source:HGNC Symbol;Acc:HGNC:9029] |
| ENSG00000075223 | HGLibA_43325 | 46 | -0.718607716 | SEMA3C | semaphorin 3C [Source:HGNC Symbol;Acc:HGNC:10725] |
| ENSG00000075240 | HGLibA_20210 | 46 | -0.718607716 | GRAMD4 | GRAM domain containing 4 [Source:HGNC Symbol;Acc:HGNC:29113] |
| ENSG00000075391 | HGLibA_40314 | 46 | -0.718607716 | RASAL2 | RAS protein activator like 2 [Source:HGNC Symbol;Acc:HGNC:9874] |
| ENSG00000082701 | HGLibA_20432 | 46 | -0.718607716 | GSK3B | glycogen synthase kinase 3 beta [Source:HGNC Symbol;Acc:HGNC:4617] |
| ENSG00000085978 | HGLibA_03473 | 46 | -0.718607716 | ATG16L1 | autophagy related 16 like 1 [Source:HGNC Symbol;Acc:HGNC:21498] |
| ENSG00000087884 | HGLibA_00053 | 46 | -0.718607716 | AAMDC | adipogenesis associated Mth938 domain containing [Source:HGNC Symbol;Acc:HGNC:30205] |
| ENSG00000088826 | HGLibA_45799 | 46 | -0.718607716 | SMOX | spermine oxidase [Source:HGNC Symbol;Acc:HGNC:15862] |
| ENSG00000092009 | HGLibA_10177 | 46 | -0.718607716 | CMA1 | chymase 1 [Source:HGNC Symbol;Acc:HGNC:2097] |
| ENSG00000094796 | HGLibA_25475 | 46 | -0.718607716 | KRT31 | keratin 31 [Source:HGNC Symbol;Acc:HGNC:6448] |
| ENSG00000100065 | HGLibA_07216 | 46 | -0.718607716 | CARD10 | caspase recruitment domain family member 10 [Source:HGNC Symbol;Acc:HGNC:16422] |
| ENSG00000100614 | HGLibA_37962 | 46 | -0.718607716 | PPM1A | Mg2+/Mn2+ dependent 1A [Source:HGNC Symbol;Acc:HGNC:9275]" |
| ENSG00000101203 | HGLibA_10536 | 46 | -0.718607716 | COL20A1 | collagen type XX alpha 1 chain [Source:HGNC Symbol;Acc:HGNC:14670] |
| ENSG00000101464 | HGLibA_36590 | 46 | -0.718607716 | PIGU | phosphatidylinositol glycan anchor biosynthesis class U [Source:HGNC Symbol;Acc:HGNC:15791] |
| ENSG00000102158 | HGLibA_28012 | 46 | -0.718607716 | MAGT1 | magnesium transporter 1 [Source:HGNC Symbol;Acc:HGNC:28880] |
| ENSG00000103051 | HGLibA_10479 | 46 | -0.718607716 | COG4 | component of oligomeric golgi complex 4 [Source:HGNC Symbol;Acc:HGNC:18620] |
| ENSG00000104723 | HGLibA_52348 | 46 | -0.718607716 | TUSC3 | tumor suppressor candidate 3 [Source:HGNC Symbol;Acc:HGNC:30242] |
| ENSG00000104872 | HGLibA_36609 | 46 | -0.718607716 | PIH1D1 | PIH1 domain containing 1 [Source:HGNC Symbol;Acc:HGNC:26075] |
| ENSG00000105967 | HGLibA_49166 | 46 | -0.718607716 | TFEC | transcription factor EC [Source:HGNC Symbol;Acc:HGNC:11754] |
| ENSG00000108479 | HGLibA_18516 | 46 | -0.718607716 | GALK1 | galactokinase 1 [Source:HGNC Symbol;Acc:HGNC:4118] |
| ENSG00000108588 | HGLibA_07790 | 46 | -0.718607716 | CCDC47 | coiled-coil domain containing 47 [Source:HGNC Symbol;Acc:HGNC:24856] |
| ENSG00000112893 | HGLibA_28070 | 46 | -0.718607716 | MAN2A1 | mannosidase alpha class 2A member 1 [Source:HGNC Symbol;Acc:HGNC:6824] |
| ENSG00000112964 | HGLibA_19040 | 46 | -0.718607716 | GHR | growth hormone receptor [Source:HGNC Symbol;Acc:HGNC:4263] |
| ENSG00000113448 | HGLibA_35795 | 46 | -0.718607716 | PDE4D | phosphodiesterase 4D [Source:HGNC Symbol;Acc:HGNC:8783] |
| ENSG00000115368 | HGLibA_54175 | 46 | -0.718607716 | WDR75 | WD repeat domain 75 [Source:HGNC Symbol;Acc:HGNC:25725] |
| ENSG00000116574 | HGLibA_41207 | 46 | -0.718607716 | RHOU | ras homolog family member U [Source:HGNC Symbol;Acc:HGNC:17794] |
| ENSG00000116985 | HGLibA_04622 | 46 | -0.718607716 | BMP8B | bone morphogenetic protein 8b [Source:HGNC Symbol;Acc:HGNC:1075] |
| ENSG00000117143 | HGLibA_52506 | 46 | -0.718607716 | UAP1 | UDP-N-acetylglucosamine pyrophosphorylase 1 [Source:HGNC Symbol;Acc:HGNC:12457] |
| ENSG00000118402 | HGLibA_14967 | 46 | -0.718607716 | ELOVL4 | ELOVL fatty acid elongase 4 [Source:HGNC Symbol;Acc:HGNC:14415] |
| ENSG00000119682 | HGLibA_02644 | 46 | -0.718607716 | AREL1 | apoptosis resistant E3 ubiquitin protein ligase 1 [Source:HGNC Symbol;Acc:HGNC:20363] |
| ENSG00000120690 | HGLibA_14890 | 46 | -0.718607716 | ELF1 | E74 like ETS transcription factor 1 [Source:HGNC Symbol;Acc:HGNC:3316] |
| ENSG00000123584 | HGLibA_27922 | 46 | -0.718607716 | MAGEA9 | MAGE family member A9 [Source:HGNC Symbol;Acc:HGNC:6807] |
| ENSG00000124391 | HGLibA_22996 | 46 | -0.718607716 | IL17C | interleukin 17C [Source:HGNC Symbol;Acc:HGNC:5983] |
| ENSG00000124406 | HGLibA_03774 | 46 | -0.718607716 | ATP8A1 | ATPase phospholipid transporting 8A1 [Source:HGNC Symbol;Acc:HGNC:13531] |
| ENSG00000125398 | HGLibA_46280 | 46 | -0.718607716 | SOX9 | SRY-box 9 [Source:HGNC Symbol;Acc:HGNC:11204] |
| ENSG00000125912 | HGLibA_31330 | 46 | -0.718607716 | NCLN | nicalin [Source:HGNC Symbol;Acc:HGNC:26923] |
| ENSG00000126010 | HGLibA_20376 | 46 | -0.718607716 | GRPR | gastrin releasing peptide receptor [Source:HGNC Symbol;Acc:HGNC:4609] |
| ENSG00000127993 | HGLibA_40570 | 46 | -0.718607716 | RBM48 | RNA binding motif protein 48 [Source:HGNC Symbol;Acc:HGNC:21785] |
| ENSG00000129824 | HGLibA_42145 | 46 | -0.718607716 | RPS4Y1 | ribosomal protein S4 Y-linked 1 [Source:HGNC Symbol;Acc:HGNC:10425] |
| ENSG00000130299 | HGLibA_20606 | 46 | -0.718607716 | GTPBP3 | mitochondrial [Source:HGNC Symbol;Acc:HGNC:14880]" |
| ENSG00000131013 | HGLibA_37948 | 46 | -0.718607716 | PPIL4 | peptidylprolyl isomerase like 4 [Source:HGNC Symbol;Acc:HGNC:15702] |
| ENSG00000131507 | HGLibA_31393 | 46 | -0.718607716 | NDFIP1 | Nedd4 family interacting protein 1 [Source:HGNC Symbol;Acc:HGNC:17592] |
| ENSG00000131844 | HGLibA_28567 | 46 | -0.718607716 | MCCC2 | methylcrotonoyl-CoA carboxylase 2 [Source:HGNC Symbol;Acc:HGNC:6937] |
| ENSG00000132388 | HGLibA_52606 | 46 | -0.718607716 | UBE2G1 | ubiquitin conjugating enzyme E2 G1 [Source:HGNC Symbol;Acc:HGNC:12482] |
| ENSG00000132554 | HGLibA_41089 | 46 | -0.718607716 | RGS22 | regulator of G protein signaling 22 [Source:HGNC Symbol;Acc:HGNC:24499] |
| ENSG00000132570 | HGLibA_35365 | 46 | -0.718607716 | PCBD2 | pterin-4 alpha-carbinolamine dehydratase 2 [Source:HGNC Symbol;Acc:HGNC:24474] |
| ENSG00000132840 | HGLibA_04488 | 46 | -0.718607716 | BHMT2 | betaine--homocysteine S-methyltransferase 2 [Source:HGNC Symbol;Acc:HGNC:1048] |
| ENSG00000133131 | HGLibA_29657 | 46 | -0.718607716 | MORC4 | MORC family CW-type zinc finger 4 [Source:HGNC Symbol;Acc:HGNC:23485] |
| ENSG00000134775 | HGLibA_17495 | 46 | -0.718607716 | FHOD3 | formin homology 2 domain containing 3 [Source:HGNC Symbol;Acc:HGNC:26178] |
| ENSG00000134780 | HGLibA_12294 | 46 | -0.718607716 | DAGLA | diacylglycerol lipase alpha [Source:HGNC Symbol;Acc:HGNC:1165] |
| ENSG00000135164 | HGLibA_13443 | 46 | -0.718607716 | DMTF1 | cyclin D binding myb like transcription factor 1 [Source:HGNC Symbol;Acc:HGNC:14603] |
| ENSG00000135472 | HGLibA_15967 | 46 | -0.718607716 | FAIM2 | Fas apoptotic inhibitory molecule 2 [Source:HGNC Symbol;Acc:HGNC:17067] |
| ENSG00000136546 | HGLibA_43000 | 46 | -0.718607716 | SCN7A | sodium voltage-gated channel alpha subunit 7 [Source:HGNC Symbol;Acc:HGNC:10594] |
| ENSG00000137218 | HGLibA_18051 | 46 | -0.718607716 | FRS3 | fibroblast growth factor receptor substrate 3 [Source:HGNC Symbol;Acc:HGNC:16970] |
| ENSG00000138400 | HGLibA_28686 | 46 | -0.718607716 | MDH1B | malate dehydrogenase 1B [Source:HGNC Symbol;Acc:HGNC:17836] |
| ENSG00000139197 | HGLibA_36116 | 46 | -0.718607716 | PEX5 | peroxisomal biogenesis factor 5 [Source:HGNC Symbol;Acc:HGNC:9719] |
| ENSG00000139579 | HGLibA_31028 | 46 | -0.718607716 | NABP2 | nucleic acid binding protein 2 [Source:HGNC Symbol;Acc:HGNC:28412] |
| ENSG00000140839 | HGLibA_09945 | 46 | -0.718607716 | CLEC18B | C-type lectin domain family 18 member B [Source:HGNC Symbol;Acc:HGNC:33849] |
| ENSG00000141371 | HGLibA_05558 | 46 | -0.718607716 | C17orf64 | chromosome 17 open reading frame 64 [Source:HGNC Symbol;Acc:HGNC:26990] |
| ENSG00000141425 | HGLibA_42045 | 46 | -0.718607716 | RPRD1A | regulation of nuclear pre-mRNA domain containing 1A [Source:HGNC Symbol;Acc:HGNC:25560] |
| ENSG00000142864 | HGLibA_43465 | 46 | -0.718607716 | SERBP1 | SERPINE1 mRNA binding protein 1 [Source:HGNC Symbol;Acc:HGNC:17860] |
| ENSG00000143398 | HGLibA_36714 | 46 | -0.718607716 | PIP5K1A | phosphatidylinositol-4-phosphate 5-kinase type 1 alpha [Source:HGNC Symbol;Acc:HGNC:8994] |
| ENSG00000143537 | HGLibA_00736 | 46 | -0.718607716 | ADAM15 | ADAM metallopeptidase domain 15 [Source:HGNC Symbol;Acc:HGNC:193] |
| ENSG00000143595 | HGLibA_02582 | 46 | -0.718607716 | AQP10 | aquaporin 10 [Source:HGNC Symbol;Acc:HGNC:16029] |
| ENSG00000145287 | HGLibA_36942 | 46 | -0.718607716 | PLAC8 | placenta specific 8 [Source:HGNC Symbol;Acc:HGNC:19254] |
| ENSG00000146909 | HGLibA_32287 | 46 | -0.718607716 | NOM1 | nucleolar protein with MIF4G domain 1 [Source:HGNC Symbol;Acc:HGNC:13244] |
| ENSG00000147166 | HGLibA_23775 | 46 | -0.718607716 | ITGB1BP2 | integrin subunit beta 1 binding protein 2 [Source:HGNC Symbol;Acc:HGNC:6154] |
| ENSG00000147364 | HGLibA_17054 | 46 | -0.718607716 | FBXO25 | F-box protein 25 [Source:HGNC Symbol;Acc:HGNC:13596] |
| ENSG00000147443 | HGLibA_13771 | 46 | -0.718607716 | DOK2 | docking protein 2 [Source:HGNC Symbol;Acc:HGNC:2991] |
| ENSG00000147872 | HGLibA_37172 | 46 | -0.718607716 | PLIN2 | perilipin 2 [Source:HGNC Symbol;Acc:HGNC:248] |
| ENSG00000148296 | HGLibA_47841 | 46 | -0.718607716 | SURF6 | surfeit 6 [Source:HGNC Symbol;Acc:HGNC:11478] |
| ENSG00000150773 | HGLibA_36613 | 46 | -0.718607716 | PIH1D2 | PIH1 domain containing 2 [Source:HGNC Symbol;Acc:HGNC:25210] |
| ENSG00000150783 | HGLibA_49056 | 46 | -0.718607716 | TEX12 | testis expressed 12 [Source:HGNC Symbol;Acc:HGNC:11734] |
| ENSG00000151376 | HGLibA_28714 | 46 | -0.718607716 | ME3 | malic enzyme 3 [Source:HGNC Symbol;Acc:HGNC:6985] |
| ENSG00000151914 | HGLibA_14043 | 46 | -0.718607716 | DST | dystonin [Source:HGNC Symbol;Acc:HGNC:1090] |
| ENSG00000154380 | HGLibA_15103 | 46 | -0.718607716 | ENAH | actin regulator [Source:HGNC Symbol;Acc:HGNC:18271]" |
| ENSG00000155016 | HGLibA_12144 | 46 | -0.718607716 | CYP2U1 | cytochrome P450 family 2 subfamily U member 1 [Source:HGNC Symbol;Acc:HGNC:20582] |
| ENSG00000155833 | HGLibA_12045 | 46 | -0.718607716 | CYLC2 | cylicin 2 [Source:HGNC Symbol;Acc:HGNC:2583] |
| ENSG00000156253 | HGLibA_42486 | 46 | -0.718607716 | RWDD2B | RWD domain containing 2B [Source:HGNC Symbol;Acc:HGNC:1302] |
| ENSG00000156467 | HGLibA_53109 | 46 | -0.718607716 | UQCRB | ubiquinol-cytochrome c reductase binding protein [Source:HGNC Symbol;Acc:HGNC:12582] |
| ENSG00000156587 | HGLibA_52629 | 46 | -0.718607716 | UBE2L6 | ubiquitin conjugating enzyme E2 L6 [Source:HGNC Symbol;Acc:HGNC:12490] |
| ENSG00000157470 | HGLibA_16734 | 46 | -0.718607716 | FAM81A | family with sequence similarity 81 member A [Source:HGNC Symbol;Acc:HGNC:28379] |
| ENSG00000157500 | HGLibA_02565 | 46 | -0.718607716 | APPL1 | phosphotyrosine interacting with PH domain and leucine zipper 1 [Source:HGNC Symbol;Acc:HGNC:24035]" |
| ENSG00000158683 | HGLibA_36793 | 46 | -0.718607716 | PKD1L1 | transient receptor potential channel interacting [Source:HGNC Symbol;Acc:HGNC:18053]" |
| ENSG00000161013 | HGLibA_29143 | 46 | -0.718607716 | MGAT4B | 3-mannosyl-glycoprotein 4-beta-N-acetylglucosaminyltransferase B [Source:HGNC Symbol;Acc:HGNC:7048]" |
| ENSG00000162066 | HGLibA_01763 | 46 | -0.718607716 | AMDHD2 | amidohydrolase domain containing 2 [Source:HGNC Symbol;Acc:HGNC:24262] |
| ENSG00000162594 | HGLibA_23100 | 46 | -0.718607716 | IL23R | interleukin 23 receptor [Source:HGNC Symbol;Acc:HGNC:19100] |
| ENSG00000162607 | HGLibA_53192 | 46 | -0.718607716 | USP1 | ubiquitin specific peptidase 1 [Source:HGNC Symbol;Acc:HGNC:12607] |
| ENSG00000162639 | HGLibA_21136 | 46 | -0.718607716 | HENMT1 | HEN methyltransferase 1 [Source:HGNC Symbol;Acc:HGNC:26400] |
| ENSG00000162877 | HGLibA_37284 | 46 | -0.718607716 | PM20D1 | peptidase M20 domain containing 1 [Source:HGNC Symbol;Acc:HGNC:26518] |
| ENSG00000163162 | HGLibA_41541 | 46 | -0.718607716 | RNF149 | ring finger protein 149 [Source:HGNC Symbol;Acc:HGNC:23137] |
| ENSG00000163516 | HGLibA_02165 | 46 | -0.718607716 | ANKZF1 | ankyrin repeat and zinc finger domain containing 1 [Source:HGNC Symbol;Acc:HGNC:25527] |
| ENSG00000164484 | HGLibA_50170 | 46 | -0.718607716 | TMEM200A | transmembrane protein 200A [Source:HGNC Symbol;Acc:HGNC:21075] |
| ENSG00000165949 | HGLibA_22596 | 46 | -0.718607716 | IFI27 | interferon alpha inducible protein 27 [Source:HGNC Symbol;Acc:HGNC:5397] |
| ENSG00000166111 | HGLibA_47892 | 46 | -0.718607716 | SVOP | SV2 related protein [Source:HGNC Symbol;Acc:HGNC:25417] |
| ENSG00000166199 | HGLibA_01653 | 46 | -0.718607716 | ALKBH3 | alpha-ketoglutaratedependent dioxygenase [Source:HGNC Symbol;Acc:HGNC:30141]" |
| ENSG00000166452 | HGLibA_01455 | 46 | -0.718607716 | AKIP1 | A-kinase interacting protein 1 [Source:HGNC Symbol;Acc:HGNC:1170] |
| ENSG00000166532 | HGLibA_41273 | 46 | -0.718607716 | RIMKLB | ribosomal modification protein rimK like family member B [Source:HGNC Symbol;Acc:HGNC:29228] |
| ENSG00000166707 | HGLibA_55023 | 46 | -0.718607716 | ZCCHC18 | zinc finger CCHC-type containing 18 [Source:HGNC Symbol;Acc:HGNC:32459] |
| ENSG00000166743 | HGLibA_00569 | 46 | -0.718607716 | ACSM1 | acyl-CoA synthetase medium chain family member 1 [Source:HGNC Symbol;Acc:HGNC:18049] |
| ENSG00000166965 | HGLibA_40689 | 46 | -0.718607716 | RCCD1 | RCC1 domain containing 1 [Source:HGNC Symbol;Acc:HGNC:30457] |
| ENSG00000167625 | HGLibA_56165 | 46 | -0.718607716 | ZNF526 | zinc finger protein 526 [Source:HGNC Symbol;Acc:HGNC:29415] |
| ENSG00000167792 | HGLibA_31568 | 46 | -0.718607716 | NDUFV1 | NADH:ubiquinone oxidoreductase core subunit V1 [Source:HGNC Symbol;Acc:HGNC:7716] |
| ENSG00000168159 | HGLibA_41598 | 46 | -0.718607716 | RNF187 | ring finger protein 187 [Source:HGNC Symbol;Acc:HGNC:27146] |
| ENSG00000168658 | HGLibA_53906 | 46 | -0.718607716 | VWA3B | von Willebrand factor A domain containing 3B [Source:HGNC Symbol;Acc:HGNC:28385] |
| ENSG00000168824 | HGLibA_32756 | 46 | -0.718607716 | NSG1 | neuronal vesicle trafficking associated 1 [Source:HGNC Symbol;Acc:HGNC:18790] |
| ENSG00000169696 | HGLibA_03366 | 46 | -0.718607716 | ASPSCR1 | UBX domain containing tether for SLC2A4 [Source:HGNC Symbol;Acc:HGNC:13825]" |
| ENSG00000170180 | HGLibA_20698 | 46 | -0.718607716 | GYPA | glycophorin A (MNS blood group) [Source:HGNC Symbol;Acc:HGNC:4702] |
| ENSG00000170775 | HGLibA_20032 | 46 | -0.718607716 | GPR37 | G protein-coupled receptor 37 [Source:HGNC Symbol;Acc:HGNC:4494] |
| ENSG00000170953 | HGLibA_34492 | 46 | -0.718607716 | OR8B12 | olfactory receptor family 8 subfamily B member 12 [Source:HGNC Symbol;Acc:HGNC:15307] |
| ENSG00000170961 | HGLibA_20865 | 46 | -0.718607716 | HAS2 | hyaluronan synthase 2 [Source:HGNC Symbol;Acc:HGNC:4819] |
| ENSG00000171451 | HGLibA_14018 | 46 | -0.718607716 | DSEL | dermatan sulfate epimerase like [Source:HGNC Symbol;Acc:HGNC:18144] |
| ENSG00000171497 | HGLibA_37923 | 46 | -0.718607716 | PPID | peptidylprolyl isomerase D [Source:HGNC Symbol;Acc:HGNC:9257] |
| ENSG00000171824 | HGLibA_15772 | 46 | -0.718607716 | EXOSC10 | exosome component 10 [Source:HGNC Symbol;Acc:HGNC:9138] |
| ENSG00000172007 | HGLibA_39883 | 46 | -0.718607716 | RAB33B | member RAS oncogene family [Source:HGNC Symbol;Acc:HGNC:16075]" |
| ENSG00000172059 | HGLibA_25044 | 46 | -0.718607716 | KLF11 | Kruppel like factor 11 [Source:HGNC Symbol;Acc:HGNC:11811] |
| ENSG00000173163 | HGLibA_10650 | 46 | -0.718607716 | COMMD1 | copper metabolism domain containing 1 [Source:HGNC Symbol;Acc:HGNC:23024] |
| ENSG00000173531 | HGLibA_30252 | 46 | -0.718607716 | MST1 | macrophage stimulating 1 [Source:HGNC Symbol;Acc:HGNC:7380] |
| ENSG00000174373 | HGLibA_40157 | 46 | -0.718607716 | RALGAPA1 | Ral GTPase activating protein catalytic alpha subunit 1 [Source:HGNC Symbol;Acc:HGNC:17770] |
| ENSG00000174446 | HGLibA_45903 | 46 | -0.718607716 | SNAPC5 | small nuclear RNA activating complex polypeptide 5 [Source:HGNC Symbol;Acc:HGNC:15484] |
| ENSG00000176253 | HGLibA_34020 | 46 | -0.718607716 | OR4K13 | olfactory receptor family 4 subfamily K member 13 [Source:HGNC Symbol;Acc:HGNC:15351] |
| ENSG00000176798 | HGLibA_34122 | 46 | -0.718607716 | OR51L1 | olfactory receptor family 51 subfamily L member 1 [Source:HGNC Symbol;Acc:HGNC:14759] |
| ENSG00000177575 | HGLibA_08242 | 46 | -0.718607716 | CD163 | CD163 molecule [Source:HGNC Symbol;Acc:HGNC:1631] |
| ENSG00000178473 | HGLibA_52827 | 46 | -0.718607716 | UCN3 | urocortin 3 [Source:HGNC Symbol;Acc:HGNC:17781] |

| | | | | | |
|---|---|---|---|---|---|
| ENSG00000178966 | HGLibA_41373 | 46 | -0.718607716 | RMI1 | RecQ mediated genome instability 1 [Source:HGNC Symbol;Acc:HGNC:25764] |
| ENSG00000179335 | HGLibA_10059 | 46 | -0.718607716 | CLK3 | CDC like kinase 3 [Source:HGNC Symbol;Acc:HGNC:2071] |
| ENSG00000179921 | HGLibA_19752 | 46 | -0.718607716 | GPBAR1 | G protein-coupled bile acid receptor 1 [Source:HGNC Symbol;Acc:HGNC:19680] |
| ENSG00000182053 | HGLibA_51526 | 46 | -0.718607716 | TRIM49B | tripartite motif containing 49B [Source:HGNC Symbol;Acc:HGNC:42955] |
| ENSG00000182087 | HGLibA_50345 | 46 | -0.718607716 | TMEM259 | transmembrane protein 259 [Source:HGNC Symbol;Acc:HGNC:17039] |
| ENSG00000182111 | HGLibA_56622 | 46 | -0.718607716 | ZNF716 | zinc finger protein 716 [Source:HGNC Symbol;Acc:HGNC:32458] |
| ENSG00000182197 | HGLibA_15802 | 46 | -0.718607716 | EXT1 | exostosin glycosyltransferase 1 [Source:HGNC Symbol;Acc:HGNC:3512] |
| ENSG00000183814 | HGLibA_26583 | 46 | -0.718607716 | LIN9 | lin-9 DREAM MuvB core complex component [Source:HGNC Symbol;Acc:HGNC:30830] |
| ENSG00000183831 | HGLibA_02104 | 46 | -0.718607716 | ANKRD45 | ankyrin repeat domain 45 [Source:HGNC Symbol;Acc:HGNC:24786] |
| ENSG00000183891 | HGLibA_52113 | 46 | -0.718607716 | TTC32 | tetratricopeptide repeat domain 32 [Source:HGNC Symbol;Acc:HGNC:32954] |
| ENSG00000184047 | HGLibA_13213 | 46 | -0.718607716 | DIABLO | diablo IAP-binding mitochondrial protein [Source:HGNC Symbol;Acc:HGNC:21528] |
| ENSG00000184060 | HGLibA_00865 | 46 | -0.718607716 | ADAP2 | ArfGAP with dual PH domains 2 [Source:HGNC Symbol;Acc:HGNC:16487] |
| ENSG00000184076 | HGLibA_53104 | 46 | -0.718607716 | UQCR10 | complex III subunit X [Source:HGNC Symbol;Acc:HGNC:30863]" |
| ENSG00000184402 | HGLibA_47104 | 46 | -0.718607716 | SS18L1 | nBAF chromatin remodeling complex subunit [Source:HGNC Symbol;Acc:HGNC:15592]" |
| ENSG00000184752 | HGLibA_31445 | 46 | -0.718607716 | NDUFA12 | NADH:ubiquinone oxidoreductase subunit A12 [Source:HGNC Symbol;Acc:HGNC:23987] |
| ENSG00000184787 | HGLibA_52609 | 46 | -0.718607716 | UBE2G2 | ubiquitin conjugating enzyme E2 G2 [Source:HGNC Symbol;Acc:HGNC:12483] |
| ENSG00000185164 | HGLibA_32291 | 46 | -0.718607716 | NOMO2 | NODAL modulator 2 [Source:HGNC Symbol;Acc:HGNC:22652] |
| ENSG00000185269 | HGLibA_32359 | 46 | -0.718607716 | NOTUM | palmitoleoyl-protein carboxylesterase [Source:HGNC Symbol;Acc:HGNC:27106]" |
| ENSG00000185567 | HGLibA_01305 | 46 | -0.718607716 | AHNAK2 | AHNAK nucleoprotein 2 [Source:HGNC Symbol;Acc:HGNC:20125] |
| ENSG00000185615 | HGLibA_35873 | 46 | -0.718607716 | PDIA2 | protein disulfide isomerase family A member 2 [Source:HGNC Symbol;Acc:HGNC:14180] |
| ENSG00000186376 | HGLibA_56679 | 46 | -0.718607716 | ZNF75D | zinc finger protein 75D [Source:HGNC Symbol;Acc:HGNC:13145] |
| ENSG00000186487 | HGLibA_30927 | 46 | -0.718607716 | MYT1L | myelin transcription factor 1 like [Source:HGNC Symbol;Acc:HGNC:7623] |
| ENSG00000187792 | HGLibA_56573 | 46 | -0.718607716 | ZNF70 | zinc finger protein 70 [Source:HGNC Symbol;Acc:HGNC:13140] |
| ENSG00000187955 | HGLibA_10513 | 46 | -0.718607716 | COL14A1 | collagen type XIV alpha 1 chain [Source:HGNC Symbol;Acc:HGNC:2191] |
| ENSG00000188266 | HGLibA_22454 | 46 | -0.718607716 | HYKK | hydroxylysine kinase [Source:HGNC Symbol;Acc:HGNC:34403] |
| ENSG00000188763 | HGLibA_18287 | 46 | -0.718607716 | FZD9 | frizzled class receptor 9 [Source:HGNC Symbol;Acc:HGNC:4047] |
| ENSG00000189043 | HGLibA_31458 | 46 | -0.718607716 | NDUFA4 | mitochondrial complex associated [Source:HGNC Symbol;Acc:HGNC:7687]" |
| ENSG00000196240 | HGLibA_33864 | 46 | -0.718607716 | OR2T2 | olfactory receptor family 2 subfamily T member 2 [Source:HGNC Symbol;Acc:HGNC:14725] |
| ENSG00000196693 | HGLibA_55833 | 46 | -0.718607716 | ZNF33B | zinc finger protein 33B [Source:HGNC Symbol;Acc:HGNC:13097] |
| ENSG00000196776 | HGLibA_08395 | 46 | -0.718607716 | CD47 | CD47 molecule [Source:HGNC Symbol;Acc:HGNC:1682] |
| ENSG00000196865 | HGLibA_31898 | 46 | -0.718607716 | NHLRC2 | NHL repeat containing 2 [Source:HGNC Symbol;Acc:HGNC:24731] |
| ENSG00000197734 | HGLibA_05345 | 46 | -0.718607716 | C14orf178 | chromosome 14 open reading frame 178 [Source:HGNC Symbol;Acc:HGNC:26385] |
| ENSG00000197849 | HGLibA_34516 | 46 | -0.718607716 | OR8G1 | olfactory receptor family 8 subfamily G member 1 (gene/pseudogene) [Source:HGNC Symbol;Acc:HGNC:8484] |
| ENSG00000197935 | HGLibA_55780 | 46 | -0.718607716 | ZNF311 | zinc finger protein 311 [Source:HGNC Symbol;Acc:HGNC:13847] |
| ENSG00000198488 | HGLibA_03995 | 46 | -0.718607716 | B3GNT6 | 3-N-acetylglucosaminyltransferase 6 [Source:HGNC Symbol;Acc:HGNC:24141]" |
| ENSG00000198522 | HGLibA_19824 | 46 | -0.718607716 | GPN1 | GPN-loop GTPase 1 [Source:HGNC Symbol;Acc:HGNC:17030] |
| ENSG00000198732 | HGLibA_45794 | 46 | -0.718607716 | SMOC1 | SPARC related modular calcium binding 1 [Source:HGNC Symbol;Acc:HGNC:20318] |
| ENSG00000198822 | HGLibA_20347 | 46 | -0.718607716 | GRM3 | glutamate metabotropic receptor 3 [Source:HGNC Symbol;Acc:HGNC:4595] |
| ENSG00000204540 | HGLibA_39217 | 46 | -0.718607716 | PSORS1C1 | psoriasis susceptibility 1 candidate 1 [Source:HGNC Symbol;Acc:HGNC:17202] |
| ENSG00000205213 | HGLibA_26409 | 46 | -0.718607716 | LGR4 | leucine rich repeat containing G protein-coupled receptor 4 [Source:HGNC Symbol;Acc:HGNC:13299] |
| ENSG00000206150 | HGLibA_41400 | 46 | -0.718607716 | RNASE13 | ribonuclease A family member 13 (inactive) [Source:HGNC Symbol;Acc:HGNC:25285] |
| ENSG00000212124 | HGLibA_48378 | 46 | -0.718607716 | TAS2R19 | taste 2 receptor member 19 [Source:HGNC Symbol;Acc:HGNC:19108] |
| ENSG00000213030 | HGLibA_09314 | 46 | -0.718607716 | CGB8 | chorionic gonadotropin subunit beta 8 [Source:HGNC Symbol;Acc:HGNC:16453] |
| ENSG00000213413 | HGLibA_39618 | 46 | -0.718607716 | PVRIG | PVR related immunoglobulin domain containing [Source:HGNC Symbol;Acc:HGNC:32190] |
| ENSG00000214943 | HGLibA_20024 | 46 | -0.718607716 | GPR33 | G protein-coupled receptor 33 (gene/pseudogene) [Source:HGNC Symbol;Acc:HGNC:4489] |
| ENSG00000221866 | HGLibA_37267 | 46 | -0.718607716 | PLXNA4 | plexin A4 [Source:HGNC Symbol;Acc:HGNC:9102] |
| ENSG00000223614 | HGLibA_56649 | 46 | -0.718607716 | ZNF735 | zinc finger protein 735 [Source:HGNC Symbol;Acc:HGNC:32466] |
| ENSG00000234444 | HGLibA_56654 | 46 | -0.718607716 | ZNF736 | zinc finger protein 736 [Source:HGNC Symbol;Acc:HGNC:32467] |
| ENSG00000234829 | HGLibA_22670 | 46 | -0.718607716 | IFNA17 | interferon alpha 17 [Source:HGNC Symbol;Acc:HGNC:5422] |
| ENSG00000235942 | HGLibA_26152 | 46 | -0.718607716 | LCE6A | late cornified envelope 6A [Source:HGNC Symbol;Acc:HGNC:31824] |
| ENSG00000250120 | HGLibA_35426 | 46 | -0.718607716 | PCDHA10 | protocadherin alpha 10 [Source:HGNC Symbol;Acc:HGNC:8664] |
| ENSG00000251201 | HGLibA_49822 | 46 | -0.718607716 | TMED7-TICAM2 | TMED7-TICAM2 readthrough [Source:HGNC Symbol;Acc:HGNC:33945] |
| ENSG00000254986 | HGLibA_13869 | 46 | -0.718607716 | DPP3 | dipeptidyl peptidase 3 [Source:HGNC Symbol;Acc:HGNC:3008] |
| ENSG00000259024 | HGLibA_52365 | 46 | -0.718607716 | TVP23C-CDRT4 | TVP23C-CDRT4 readthrough [Source:HGNC Symbol;Acc:HGNC:42961] |
| ENSG00000267508 | HGLibA_55746 | 46 | -0.718607716 | ZNF285 | zinc finger protein 285 [Source:HGNC Symbol;Acc:HGNC:13079] |
| ENSG00000267680 | HGLibA_55618 | 46 | -0.718607716 | ZNF224 | zinc finger protein 224 [Source:HGNC Symbol;Acc:HGNC:13017] |
| ENSG00000270617 | HGLibA_53143 | 46 | -0.718607716 | URGCP-MRPS24 | URGCP-MRPS24 readthrough [Source:HGNC Symbol;Acc:HGNC:49188] |
| ENSG00000272391 | HGLibA_37646 | 46 | -0.718607716 | POM121C | POM121 transmembrane nucleoporin C [Source:HGNC Symbol;Acc:HGNC:34005] |
| FAM122A | HGLibA_16065 | 46 | -0.718607716 | FAM122A | - |
| FAM46C | HGLibA_16603 | 46 | -0.718607716 | FAM46C | - |
| GATSL2 | HGLibA_18705 | 46 | -0.718607716 | GATSL2 | - |
| hsa-mir-1250 | HGLibA_57398 | 46 | -0.718607716 | hsa-mir-1250 | - |
| hsa-mir-181a-1 | HGLibA_57957 | 46 | -0.718607716 | hsa-mir-181a-1 | - |
| hsa-mir-27b | HGLibA_58381 | 46 | -0.718607716 | hsa-mir-27b | - |
| hsa-mir-3127 | HGLibA_58559 | 46 | -0.718607716 | hsa-mir-3127 | - |
| hsa-mir-3151 | HGLibA_58662 | 46 | -0.718607716 | hsa-mir-3151 | - |
| hsa-mir-3689b | HGLibA_59317 | 46 | -0.718607716 | hsa-mir-3689b | - |
| hsa-mir-378c | HGLibA_59433 | 46 | -0.718607716 | hsa-mir-378c | - |
| hsa-mir-378h | HGLibA_59457 | 46 | -0.718607716 | hsa-mir-378h | - |
| hsa-mir-3936 | HGLibA_59606 | 46 | -0.718607716 | hsa-mir-3936 | - |
| hsa-mir-3942 | HGLibA_59629 | 46 | -0.718607716 | hsa-mir-3942 | - |
| hsa-mir-4427 | HGLibA_60093 | 46 | -0.718607716 | hsa-mir-4427 | - |
| hsa-mir-4500 | HGLibA_60430 | 46 | -0.718607716 | hsa-mir-4500 | - |
| hsa-mir-4520b | HGLibA_60538 | 46 | -0.718607716 | hsa-mir-4520b | - |
| hsa-mir-4744 | HGLibA_61073 | 46 | -0.718607716 | hsa-mir-4744 | - |
| hsa-mir-487a | HGLibA_61345 | 46 | -0.718607716 | hsa-mir-487a | - |
| hsa-mir-5009 | HGLibA_61438 | 46 | -0.718607716 | hsa-mir-5009 | - |
| hsa-mir-520g | HGLibA_61758 | 46 | -0.718607716 | hsa-mir-520g | - |
| hsa-mir-551a | HGLibA_62117 | 46 | -0.718607716 | hsa-mir-551a | - |
| hsa-mir-6718 | HGLibA_63032 | 46 | -0.718607716 | hsa-mir-6718 | - |
| hsa-mir-6742 | HGLibA_63122 | 46 | -0.718607716 | hsa-mir-6742 | - |
| hsa-mir-6792 | HGLibA_63345 | 46 | -0.718607716 | hsa-mir-6792 | - |
| hsa-mir-6873 | HGLibA_63676 | 46 | -0.718607716 | hsa-mir-6873 | - |
| hsa-mir-8055 | HGLibA_64059 | 46 | -0.718607716 | hsa-mir-8055 | - |
| hsa-mir-8080 | HGLibA_64164 | 46 | -0.718607716 | hsa-mir-8080 | - |
| hsa-mir-8083 | HGLibA_64175 | 46 | -0.718607716 | hsa-mir-8083 | - |
| hsa-mir-940 | HGLibA_64335 | 46 | -0.718607716 | hsa-mir-940 | - |
| hsa-mir-96 | HGLibA_64368 | 46 | -0.718607716 | hsa-mir-96 | - |
| LOC100289187 | HGLibA_26839 | 46 | -0.718607716 | LOC100289187 | - |
| OR8U8 | HGLibA_34559 | 46 | -0.718607716 | OR8U8 | - |
| PLA2G16 | HGLibA_36880 | 46 | -0.718607716 | PLA2G16 | - |
| ZFP112 | HGLibA_55185 | 46 | -0.718607716 | ZFP112 | - |
| ZNRD1 | HGLibA_56935 | 46 | -0.718607716 | ZNRD1 | - |
| C11orf30 | HGLibA_05128 | 45 | -0.727653059 | C11orf30 | - |
| C12orf45 | HGLibA_05250 | 45 | -0.727653059 | C12orf45 | - |
| C15orf59 | HGLibA_05423 | 45 | -0.727653059 | C15orf59 | - |
| C16orf59 | HGLibA_05466 | 45 | -0.727653059 | C16orf59 | - |
| C1orf177 | HGLibA_05802 | 45 | -0.727653059 | C1orf177 | - |
| C9orf47 | HGLibA_06737 | 45 | -0.727653059 | C9orf47 | - |
| CARS | HGLibA_07259 | 45 | -0.727653059 | CARS | - |
| CSF2RA_Y | HGLibA_11333 | 45 | -0.727653059 | CSF2RA_Y | - |
| ENSG00000006468 | HGLibA_15648 | 45 | -0.727653059 | ETV1 | ETS variant 1 [Source:HGNC Symbol;Acc:HGNC:3490] |
| ENSG00000008130 | HGLibA_31046 | 45 | -0.727653059 | NADK | NAD kinase [Source:HGNC Symbol;Acc:HGNC:29831] |
| ENSG00000011566 | HGLibA_28217 | 45 | -0.727653059 | MAP4K3 | mitogen-activated protein kinase kinase kinase kinase 3 [Source:HGNC Symbol;Acc:HGNC:6865] |
| ENSG00000023171 | HGLibA_20196 | 45 | -0.727653059 | GRAMD1B | GRAM domain containing 1B [Source:HGNC Symbol;Acc:HGNC:29214] |
| ENSG00000028116 | HGLibA_53827 | 45 | -0.727653059 | VRK2 | VRK serine/threonine kinase 2 [Source:HGNC Symbol;Acc:HGNC:12719] |
| ENSG00000054179 | HGLibA_15199 | 45 | -0.727653059 | ENTPD2 | ectonucleoside triphosphate diphosphohydrolase 2 [Source:HGNC Symbol;Acc:HGNC:3364] |
| ENSG00000055118 | HGLibA_24214 | 45 | -0.727653059 | KCNH2 | potassium voltage-gated channel subfamily H member 2 [Source:HGNC Symbol;Acc:HGNC:6251] |
| ENSG00000060718 | HGLibA_10499 | 45 | -0.727653059 | COL11A1 | collagen type XI alpha 1 chain [Source:HGNC Symbol;Acc:HGNC:2186] |
| ENSG00000063978 | HGLibA_41677 | 45 | -0.727653059 | RNF4 | ring finger protein 4 [Source:HGNC Symbol;Acc:HGNC:10067] |
| ENSG00000064313 | HGLibA_48229 | 45 | -0.727653059 | TAF2 | TATA-box binding protein associated factor 2 [Source:HGNC Symbol;Acc:HGNC:11536] |
| ENSG00000065150 | HGLibA_23469 | 45 | -0.727653059 | IPO5 | importin 5 [Source:HGNC Symbol;Acc:HGNC:6402] |
| ENSG00000070886 | HGLibA_15308 | 45 | -0.727653059 | EPHA8 | EPH receptor A8 [Source:HGNC Symbol;Acc:HGNC:3391] |
| ENSG00000072401 | HGLibA_52581 | 45 | -0.727653059 | UBE2D1 | ubiquitin conjugating enzyme E2 D1 [Source:HGNC Symbol;Acc:HGNC:12474] |
| ENSG00000072958 | HGLibA_02290 | 45 | -0.727653059 | AP1M1 | adaptor related protein complex 1 subunit mu 1 [Source:HGNC Symbol;Acc:HGNC:13667] |
| ENSG00000073605 | HGLibA_20406 | 45 | -0.727653059 | GSDMB | gasdermin B [Source:HGNC Symbol;Acc:HGNC:23690] |
| ENSG00000073861 | HGLibA_48662 | 45 | -0.727653059 | TBX21 | T-box 21 [Source:HGNC Symbol;Acc:HGNC:11599] |
| ENSG00000074370 | HGLibA_03612 | 45 | -0.727653059 | ATP2A3 | ATPase sarcoplasmic/endoplasmic reticulum Ca2+ transporting 3 [Source:HGNC Symbol;Acc:HGNC:813] |
| ENSG00000077942 | HGLibA_16943 | 45 | -0.727653059 | FBLN1 | fibulin 1 [Source:HGNC Symbol;Acc:HGNC:3600] |
| ENSG00000080573 | HGLibA_10592 | 45 | -0.727653059 | COL5A3 | collagen type V alpha 3 chain [Source:HGNC Symbol;Acc:HGNC:14864] |
| ENSG00000081177 | HGLibA_15718 | 45 | -0.727653059 | EXD2 | exonuclease 3'-5' domain containing 2 [Source:HGNC Symbol;Acc:HGNC:20217] |
| ENSG00000083844 | HGLibA_55695 | 45 | -0.727653059 | ZNF264 | zinc finger protein 264 [Source:HGNC Symbol;Acc:HGNC:13057] |
| ENSG00000090432 | HGLibA_30561 | 45 | -0.727653059 | MUL1 | mitochondrial E3 ubiquitin protein ligase 1 [Source:HGNC Symbol;Acc:HGNC:25762] |
| ENSG00000094796 | HGLibA_25474 | 45 | -0.727653059 | KRT31 | keratin 31 [Source:HGNC Symbol;Acc:HGNC:6448] |
| ENSG00000097033 | HGLibA_43955 | 45 | -0.727653059 | SH3GLB1 | endophilin B1 [Source:HGNC Symbol;Acc:HGNC:10833]" |
| ENSG00000099399 | HGLibA_27943 | 45 | -0.727653059 | MAGEB2 | MAGE family member B2 [Source:HGNC Symbol;Acc:HGNC:6809] |
| ENSG00000099622 | HGLibA_09720 | 45 | -0.727653059 | CIRBP | cold inducible RNA binding protein [Source:HGNC Symbol;Acc:HGNC:1982] |
| ENSG00000099984 | HGLibA_20500 | 45 | -0.727653059 | GSTT2 | glutathione S-transferase theta 2 (gene/pseudogene) [Source:HGNC Symbol;Acc:HGNC:4642] |
| ENSG00000100721 | HGLibA_48814 | 45 | -0.727653059 | TCL1A | T cell leukemia/lymphoma 1A [Source:HGNC Symbol;Acc:HGNC:11648] |
| ENSG00000100902 | HGLibA_39084 | 45 | -0.727653059 | PSMA6 | proteasome subunit alpha 6 [Source:HGNC Symbol;Acc:HGNC:9535] |
| ENSG00000100926 | HGLibA_49721 | 45 | -0.727653059 | TM9SF1 | transmembrane 9 superfamily member 1 [Source:HGNC Symbol;Acc:HGNC:11864] |
| ENSG00000101251 | HGLibA_43281 | 45 | -0.727653059 | SEL1L2 | ERAD E3 ligase adaptor subunit [Source:HGNC Symbol;Acc:HGNC:15897]" |
| ENSG00000101310 | HGLibA_43222 | 45 | -0.727653059 | SEC23B | coat complex II component [Source:HGNC Symbol;Acc:HGNC:10702]" |
| ENSG00000101558 | HGLibA_53517 | 45 | -0.727653059 | VAPA | VAMP associated protein A [Source:HGNC Symbol;Acc:HGNC:12648] |
| ENSG00000102032 | HGLibA_40838 | 45 | -0.727653059 | RENBP | renin binding protein [Source:HGNC Symbol;Acc:HGNC:9959] |

| | | | | | |
|---|---|---|---|---|---|
| ENSG00000102245 | HGLibA_08387 | 45 | -0.727653059 | CD40LG | CD40 ligand [Source:HGNC Symbol;Acc:HGNC:11935] |
| ENSG00000102575 | HGLibA_00514 | 45 | -0.727653059 | ACP5 | tartrate resistant [Source:HGNC Symbol;Acc:HGNC:124]" |
| ENSG00000103202 | HGLibA_32163 | 45 | -0.727653059 | NME4 | NME/NM23 nucleoside diphosphate kinase 4 [Source:HGNC Symbol;Acc:HGNC:7852] |
| ENSG00000104499 | HGLibA_19412 | 45 | -0.727653059 | GML | glycosylphosphatidylinositol anchored molecule like [Source:HGNC Symbol;Acc:HGNC:4375] |
| ENSG00000105321 | HGLibA_07919 | 45 | -0.727653059 | CCDC9 | coiled-coil domain containing 9 [Source:HGNC Symbol;Acc:HGNC:24560] |
| ENSG00000105404 | HGLibA_39996 | 45 | -0.727653059 | RABAC1 | Rab acceptor 1 [Source:HGNC Symbol;Acc:HGNC:9794] |
| ENSG00000106609 | HGLibA_50309 | 45 | -0.727653059 | TMEM248 | transmembrane protein 248 [Source:HGNC Symbol;Acc:HGNC:25476] |
| ENSG00000106609 | HGLibA_50310 | 45 | -0.727653059 | TMEM248 | transmembrane protein 248 [Source:HGNC Symbol;Acc:HGNC:25476] |
| ENSG00000107521 | HGLibA_22045 | 45 | -0.727653059 | HPS1 | biogenesis of lysosomal organelles complex 3 subunit 1 [Source:HGNC Symbol;Acc:HGNC:5163]" |
| ENSG00000108344 | HGLibA_39169 | 45 | -0.727653059 | PSMD3 | non-ATPase 3 [Source:HGNC Symbol;Acc:HGNC:9560]" |
| ENSG00000109103 | HGLibA_52987 | 45 | -0.727653059 | UNC119 | unc-119 lipid binding chaperone [Source:HGNC Symbol;Acc:HGNC:12565] |
| ENSG00000110169 | HGLibA_22067 | 45 | -0.727653059 | HPX | hemopexin [Source:HGNC Symbol;Acc:HGNC:5171] |
| ENSG00000110200 | HGLibA_01875 | 45 | -0.727653059 | ANAPC15 | anaphase promoting complex subunit 15 [Source:HGNC Symbol;Acc:HGNC:24531] |
| ENSG00000112812 | HGLibA_38905 | 45 | -0.727653059 | PRSS16 | serine protease 16 [Source:HGNC Symbol;Acc:HGNC:9480] |
| ENSG00000112981 | HGLibA_32167 | 45 | -0.727653059 | NME5 | NME/NM23 family member 5 [Source:HGNC Symbol;Acc:HGNC:7853] |
| ENSG00000113552 | HGLibA_19564 | 45 | -0.727653059 | GNPDA1 | glucosamine-6-phosphate deaminase 1 [Source:HGNC Symbol;Acc:HGNC:4417] |
| ENSG00000116128 | HGLibA_04347 | 45 | -0.727653059 | BCL9 | B cell CLL/lymphoma 9 [Source:HGNC Symbol;Acc:HGNC:1008] |
| ENSG00000116721 | HGLibA_38277 | 45 | -0.727653059 | PRAMEF1 | PRAME family member 1 [Source:HGNC Symbol;Acc:HGNC:28840] |
| ENSG00000117525 | HGLibA_15873 | 45 | -0.727653059 | F3 | tissue factor [Source:HGNC Symbol;Acc:HGNC:3541]" |
| ENSG00000119865 | HGLibA_10381 | 45 | -0.727653059 | CNRIP1 | cannabinoid receptor interacting protein 1 [Source:HGNC Symbol;Acc:HGNC:24546] |
| ENSG00000120129 | HGLibA_14126 | 45 | -0.727653059 | DUSP1 | dual specificity phosphatase 1 [Source:HGNC Symbol;Acc:HGNC:3064] |
| ENSG00000120254 | HGLibA_30378 | 45 | -0.727653059 | MTHFD1L | methylenetetrahydrofolate dehydrogenase (NADP+ dependent) 1 like [Source:HGNC Symbol;Acc:HGNC:21055] |
| ENSG00000120686 | HGLibA_52851 | 45 | -0.727653059 | UFM1 | ubiquitin fold modifier 1 [Source:HGNC Symbol;Acc:HGNC:20597] |
| ENSG00000120733 | HGLibA_24502 | 45 | -0.727653059 | KDM3B | lysine demethylase 3B [Source:HGNC Symbol;Acc:HGNC:1337] |
| ENSG00000121068 | HGLibA_48657 | 45 | -0.727653059 | TBX2 | T-box 2 [Source:HGNC Symbol;Acc:HGNC:11597] |
| ENSG00000122952 | HGLibA_57070 | 45 | -0.727653059 | ZWINT | ZW10 interacting kinetochore protein [Source:HGNC Symbol;Acc:HGNC:13195] |
| ENSG00000122986 | HGLibA_22433 | 45 | -0.727653059 | HVCN1 | hydrogen voltage gated channel 1 [Source:HGNC Symbol;Acc:HGNC:28240] |
| ENSG00000123609 | HGLibA_32180 | 45 | -0.727653059 | NMI | N-myc and STAT interactor [Source:HGNC Symbol;Acc:HGNC:7854] |
| ENSG00000124134 | HGLibA_24385 | 45 | -0.727653059 | KCNS1 | potassium voltage-gated channel modifier subfamily S member 1 [Source:HGNC Symbol;Acc:HGNC:6300] |
| ENSG00000125901 | HGLibA_30076 | 45 | -0.727653059 | MRPS26 | mitochondrial ribosomal protein S26 [Source:HGNC Symbol;Acc:HGNC:14045] |
| ENSG00000126214 | HGLibA_25027 | 45 | -0.727653059 | KLC1 | kinesin light chain 1 [Source:HGNC Symbol;Acc:HGNC:6387] |
| ENSG00000127483 | HGLibA_22013 | 45 | -0.727653059 | HP1BP3 | heterochromatin protein 1 binding protein 3 [Source:HGNC Symbol;Acc:HGNC:24973] |
| ENSG00000127554 | HGLibA_18938 | 45 | -0.727653059 | GFER | augmenter of liver regeneration [Source:HGNC Symbol;Acc:HGNC:4236]" |
| ENSG00000128626 | HGLibA_30035 | 45 | -0.727653059 | MRPS12 | mitochondrial ribosomal protein S12 [Source:HGNC Symbol;Acc:HGNC:10380] |
| ENSG00000130829 | HGLibA_14196 | 45 | -0.727653059 | DUSP9 | dual specificity phosphatase 9 [Source:HGNC Symbol;Acc:HGNC:3076] |
| ENSG00000132122 | HGLibA_46523 | 45 | -0.727653059 | SPATA6 | spermatogenesis associated 6 [Source:HGNC Symbol;Acc:HGNC:18309] |
| ENSG00000132313 | HGLibA_29961 | 45 | -0.727653059 | MRPL35 | mitochondrial ribosomal protein L35 [Source:HGNC Symbol;Acc:HGNC:14489] |
| ENSG00000132825 | HGLibA_38107 | 45 | -0.727653059 | PPP1R3D | protein phosphatase 1 regulatory subunit 3D [Source:HGNC Symbol;Acc:HGNC:9294] |
| ENSG00000132970 | HGLibA_53959 | 45 | -0.727653059 | WASF3 | WAS protein family member 3 [Source:HGNC Symbol;Acc:HGNC:12734] |
| ENSG00000133657 | HGLibA_03572 | 45 | -0.727653059 | ATP13A3 | ATPase 13A3 [Source:HGNC Symbol;Acc:HGNC:24113] |
| ENSG00000133739 | HGLibA_27442 | 45 | -0.727653059 | LRRCC1 | leucine rich repeat and coiled-coil centrosomal protein 1 [Source:HGNC Symbol;Acc:HGNC:29373] |
| ENSG00000134339 | HGLibA_42611 | 45 | -0.727653059 | SAA2 | serum amyloid A2 [Source:HGNC Symbol;Acc:HGNC:10514] |
| ENSG00000135002 | HGLibA_40924 | 45 | -0.727653059 | RFK | riboflavin kinase [Source:HGNC Symbol;Acc:HGNC:30324] |
| ENSG00000135519 | HGLibA_24216 | 45 | -0.727653059 | KCNH3 | potassium voltage-gated channel subfamily H member 3 [Source:HGNC Symbol;Acc:HGNC:6252] |
| ENSG00000135679 | HGLibA_28697 | 45 | -0.727653059 | MDM2 | MDM2 proto-oncogene [Source:HGNC Symbol;Acc:HGNC:6973] |
| ENSG00000135999 | HGLibA_15269 | 45 | -0.727653059 | EPC2 | enhancer of polycomb homolog 2 [Source:HGNC Symbol;Acc:HGNC:24543] |
| ENSG00000136295 | HGLibA_52236 | 45 | -0.727653059 | TTYH3 | tweety family member 3 [Source:HGNC Symbol;Acc:HGNC:22222] |
| ENSG00000136824 | HGLibA_45648 | 45 | -0.727653059 | SMC2 | structural maintenance of chromosomes 2 [Source:HGNC Symbol;Acc:HGNC:14011] |
| ENSG00000137478 | HGLibA_17213 | 45 | -0.727653059 | FCHSD2 | FCH and double SH3 domains 2 [Source:HGNC Symbol;Acc:HGNC:29114] |
| ENSG00000137601 | HGLibA_31639 | 45 | -0.727653059 | NEK1 | NIMA related kinase 1 [Source:HGNC Symbol;Acc:HGNC:7744] |
| ENSG00000137818 | HGLibA_42011 | 45 | -0.727653059 | RPLP1 | ribosomal protein lateral stalk subunit P1 [Source:HGNC Symbol;Acc:HGNC:10372] |
| ENSG00000138495 | HGLibA_10818 | 45 | -0.727653059 | COX17 | cytochrome c oxidase copper chaperone COX17 [Source:HGNC Symbol;Acc:HGNC:2264] |
| ENSG00000139133 | HGLibA_01603 | 45 | -0.727653059 | ALG10 | 2-glucosyltransferase [Source:HGNC Symbol;Acc:HGNC:23162]" |
| ENSG00000139343 | HGLibA_45987 | 45 | -0.727653059 | SNRPF | small nuclear ribonucleoprotein polypeptide F [Source:HGNC Symbol;Acc:HGNC:11162] |
| ENSG00000139438 | HGLibA_16521 | 45 | -0.727653059 | FAM222A | family with sequence similarity 222 member A [Source:HGNC Symbol;Acc:HGNC:25915] |
| ENSG00000139826 | HGLibA_00244 | 45 | -0.727653059 | ABHD13 | abhydrolase domain containing 13 [Source:HGNC Symbol;Acc:HGNC:20293] |
| ENSG00000140795 | HGLibA_30791 | 45 | -0.727653059 | MYLK3 | myosin light chain kinase 3 [Source:HGNC Symbol;Acc:HGNC:29826] |
| ENSG00000141499 | HGLibA_54400 | 45 | -0.727653059 | WRAP53 | WD repeat containing antisense to TP53 [Source:HGNC Symbol;Acc:HGNC:25522] |
| ENSG00000141568 | HGLibA_17896 | 45 | -0.727653059 | FOXK2 | forkhead box K2 [Source:HGNC Symbol;Acc:HGNC:6036] |
| ENSG00000141574 | HGLibA_43272 | 45 | -0.727653059 | SECTM1 | secreted and transmembrane 1 [Source:HGNC Symbol;Acc:HGNC:10707] |
| ENSG00000143437 | HGLibA_03062 | 45 | -0.727653059 | ARNT | aryl hydrocarbon receptor nuclear translocator [Source:HGNC Symbol;Acc:HGNC:700] |
| ENSG00000143590 | HGLibA_14589 | 45 | -0.727653059 | EFNA3 | ephrin A3 [Source:HGNC Symbol;Acc:HGNC:3223] |
| ENSG00000143740 | HGLibA_45883 | 45 | -0.727653059 | SNAP47 | synaptosome associated protein 47 [Source:HGNC Symbol;Acc:HGNC:30669] |
| ENSG00000144231 | HGLibA_37574 | 45 | -0.727653059 | POLR2D | RNA polymerase II subunit D [Source:HGNC Symbol;Acc:HGNC:9191] |
| ENSG00000144468 | HGLibA_41125 | 45 | -0.727653059 | RHBDD1 | rhomboid domain containing 1 [Source:HGNC Symbol;Acc:HGNC:23081] |
| ENSG00000146856 | HGLibA_01172 | 45 | -0.727653059 | AGBL3 | ATP/GTP binding protein like 3 [Source:HGNC Symbol;Acc:HGNC:27981] |
| ENSG00000147202 | HGLibA_13221 | 45 | -0.727653059 | DIAPH2 | diaphanous related formin 2 [Source:HGNC Symbol;Acc:HGNC:2877] |
| ENSG00000147536 | HGLibA_19112 | 45 | -0.727653059 | GINS4 | GINS complex subunit 4 [Source:HGNC Symbol;Acc:HGNC:28226] |
| ENSG00000147592 | HGLibA_25921 | 45 | -0.727653059 | LACTB2 | lactamase beta 2 [Source:HGNC Symbol;Acc:HGNC:18512] |
| ENSG00000147654 | HGLibA_14345 | 45 | -0.727653059 | EBAG9 | 9 [Source:HGNC Symbol;Acc:HGNC:3123]" |
| ENSG00000147799 | HGLibA_02772 | 45 | -0.727653059 | ARHGAP39 | Rho GTPase activating protein 39 [Source:HGNC Symbol;Acc:HGNC:29351] |
| ENSG00000148584 | HGLibA_00005 | 45 | -0.727653059 | A1CF | APOBEC1 complementation factor [Source:HGNC Symbol;Acc:HGNC:24086] |
| ENSG00000149609 | HGLibA_05991 | 45 | -0.727653059 | C20orf144 | chromosome 20 open reading frame 144 [Source:HGNC Symbol;Acc:HGNC:16137] |
| ENSG00000150893 | HGLibA_17987 | 45 | -0.727653059 | FREM2 | FRAS1 related extracellular matrix protein 2 [Source:HGNC Symbol;Acc:HGNC:25396] |
| ENSG00000151320 | HGLibA_01439 | 45 | -0.727653059 | AKAP6 | A-kinase anchoring protein 6 [Source:HGNC Symbol;Acc:HGNC:376] |
| ENSG00000153066 | HGLibA_52405 | 45 | -0.727653059 | TXNDC11 | thioredoxin domain containing 11 [Source:HGNC Symbol;Acc:HGNC:28030] |
| ENSG00000154059 | HGLibA_23251 | 45 | -0.727653059 | IMPACT | impact RWD domain protein [Source:HGNC Symbol;Acc:HGNC:20387] |
| ENSG00000155465 | HGLibA_45379 | 45 | -0.727653059 | SLC7A7 | solute carrier family 7 member 7 [Source:HGNC Symbol;Acc:HGNC:11065] |
| ENSG00000155666 | HGLibA_24538 | 45 | -0.727653059 | KDM8 | lysine demethylase 8 [Source:HGNC Symbol;Acc:HGNC:25840] |
| ENSG00000156234 | HGLibA_11855 | 45 | -0.727653059 | CXCL13 | C-X-C motif chemokine ligand 13 [Source:HGNC Symbol;Acc:HGNC:10639] |
| ENSG00000156467 | HGLibA_53110 | 45 | -0.727653059 | UQCRB | ubiquinol-cytochrome c reductase binding protein [Source:HGNC Symbol;Acc:HGNC:12582] |
| ENSG00000156502 | HGLibA_47830 | 45 | -0.727653059 | SUPV3L1 | Suv3 like RNA helicase [Source:HGNC Symbol;Acc:HGNC:11471] |
| ENSG00000156642 | HGLibA_32530 | 45 | -0.727653059 | NPTN | neuroplastin [Source:HGNC Symbol;Acc:HGNC:17867] |
| ENSG00000157224 | HGLibA_09857 | 45 | -0.727653059 | CLDN12 | claudin 12 [Source:HGNC Symbol;Acc:HGNC:2034] |
| ENSG00000157554 | HGLibA_15465 | 45 | -0.727653059 | ERG | ETS transcription factor ERG [Source:HGNC Symbol;Acc:HGNC:3446] |
| ENSG00000158497 | HGLibA_21741 | 45 | -0.727653059 | HMHB1 | histocompatibility minor HB-1 [Source:HGNC Symbol;Acc:HGNC:29677] |
| ENSG00000158604 | HGLibA_49811 | 45 | -0.727653059 | TMED4 | transmembrane p24 trafficking protein 4 [Source:HGNC Symbol;Acc:HGNC:22301] |
| ENSG00000159713 | HGLibA_51172 | 45 | -0.727653059 | TPPP3 | tubulin polymerization promoting protein family member 3 [Source:HGNC Symbol;Acc:HGNC:24162] |
| ENSG00000160216 | HGLibA_01223 | 45 | -0.727653059 | AGPAT3 | 1-acylglycerol-3-phosphate O-acyltransferase 3 [Source:HGNC Symbol;Acc:HGNC:326] |
| ENSG00000162191 | HGLibA_52774 | 45 | -0.727653059 | UBXN1 | UBX domain protein 1 [Source:HGNC Symbol;Acc:HGNC:18402] |
| ENSG00000162543 | HGLibA_52776 | 45 | -0.727653059 | UBXN10 | UBX domain protein 10 [Source:HGNC Symbol;Acc:HGNC:26354] |
| ENSG00000162576 | HGLibA_30627 | 45 | -0.727653059 | MXRA8 | matrix remodeling associated 8 [Source:HGNC Symbol;Acc:HGNC:7542] |
| ENSG00000162636 | HGLibA_15982 | 45 | -0.727653059 | FAM102B | family with sequence similarity 102 member B [Source:HGNC Symbol;Acc:HGNC:27637] |
| ENSG00000162909 | HGLibA_07163 | 45 | -0.727653059 | CAPN2 | calpain 2 [Source:HGNC Symbol;Acc:HGNC:1479] |
| ENSG00000163072 | HGLibA_32337 | 45 | -0.727653059 | NOSTRIN | nitric oxide synthase trafficking [Source:HGNC Symbol;Acc:HGNC:20203] |
| ENSG00000164088 | HGLibA_37992 | 45 | -0.727653059 | PPM1M | Mg2+/Mn2+ dependent 1M [Source:HGNC Symbol;Acc:HGNC:26506]" |
| ENSG00000164093 | HGLibA_36764 | 45 | -0.727653059 | PITX2 | paired like homeodomain 2 [Source:HGNC Symbol;Acc:HGNC:9005] |
| ENSG00000164172 | HGLibA_29615 | 45 | -0.727653059 | MOCS2 | molybdenum cofactor synthesis 2 [Source:HGNC Symbol;Acc:HGNC:7193] |
| ENSG00000164327 | HGLibA_41242 | 45 | -0.727653059 | RICTOR | RPTOR independent companion of MTOR complex 2 [Source:HGNC Symbol;Acc:HGNC:28611] |
| ENSG00000164411 | HGLibA_19175 | 45 | -0.727653059 | GJB7 | gap junction protein beta 7 [Source:HGNC Symbol;Acc:HGNC:16690] |
| ENSG00000164695 | HGLibA_09494 | 45 | -0.727653059 | CHMP4C | charged multivesicular body protein 4C [Source:HGNC Symbol;Acc:HGNC:30599] |
| ENSG00000166147 | HGLibA_16956 | 45 | -0.727653059 | FBN1 | fibrillin 1 [Source:HGNC Symbol;Acc:HGNC:3603] |
| ENSG00000166295 | HGLibA_01878 | 45 | -0.727653059 | ANAPC16 | anaphase promoting complex subunit 16 [Source:HGNC Symbol;Acc:HGNC:26976] |
| ENSG00000167098 | HGLibA_47797 | 45 | -0.727653059 | SUN5 | Sad1 and UNC84 domain containing 5 [Source:HGNC Symbol;Acc:HGNC:16252] |
| ENSG00000167685 | HGLibA_56014 | 45 | -0.727653059 | ZNF444 | zinc finger protein 444 [Source:HGNC Symbol;Acc:HGNC:16052] |
| ENSG00000167699 | HGLibA_19287 | 45 | -0.727653059 | GLOD4 | glyoxalase domain containing 4 [Source:HGNC Symbol;Acc:HGNC:14111] |
| ENSG00000167785 | HGLibA_56234 | 45 | -0.727653059 | ZNF558 | zinc finger protein 558 [Source:HGNC Symbol;Acc:HGNC:26422] |
| ENSG00000167964 | HGLibA_39853 | 45 | -0.727653059 | RAB26 | member RAS oncogene family [Source:HGNC Symbol;Acc:HGNC:14259]" |
| ENSG00000168734 | HGLibA_36824 | 45 | -0.727653059 | PKIG | cAMP-dependent protein kinase inhibitor gamma [Source:HGNC Symbol;Acc:HGNC:9019] |
| ENSG00000168878 | HGLibA_43772 | 45 | -0.727653059 | SFTPB | surfactant protein B [Source:HGNC Symbol;Acc:HGNC:10801] |
| ENSG00000169064 | HGLibA_54784 | 45 | -0.727653059 | ZBBX | zinc finger B-box domain containing [Source:HGNC Symbol;Acc:HGNC:26245] |
| ENSG00000169129 | HGLibA_01102 | 45 | -0.727653059 | AFAP1L2 | actin filament associated protein 1 like 2 [Source:HGNC Symbol;Acc:HGNC:25901] |
| ENSG00000169255 | HGLibA_03947 | 45 | -0.727653059 | B3GALNT1 | 3-N-acetylgalactosaminyltransferase 1 (globoside blood group) [Source:HGNC Symbol;Acc:HGNC:918]" |
| ENSG00000170054 | HGLibA_43531 | 45 | -0.727653059 | SERPINA9 | serpin family A member 9 [Source:HGNC Symbol;Acc:HGNC:15995] |
| ENSG00000170074 | HGLibA_16167 | 45 | -0.727653059 | FAM153A | family with sequence similarity 153 member A [Source:HGNC Symbol;Acc:HGNC:29940] |
| ENSG00000170160 | HGLibA_07621 | 45 | -0.727653059 | CCDC144A | coiled-coil domain containing 144A [Source:HGNC Symbol;Acc:HGNC:29072] |
| ENSG00000170234 | HGLibA_39640 | 45 | -0.727653059 | PWWP2A | PWWP domain containing 2A [Source:HGNC Symbol;Acc:HGNC:29406] |
| ENSG00000170242 | HGLibA_53380 | 45 | -0.727653059 | USP47 | ubiquitin specific peptidase 47 [Source:HGNC Symbol;Acc:HGNC:20076] |
| ENSG00000170396 | HGLibA_56787 | 45 | -0.727653059 | ZNF804A | zinc finger protein 804A [Source:HGNC Symbol;Acc:HGNC:21711] |
| ENSG00000170412 | HGLibA_20131 | 45 | -0.727653059 | GPRC5C | G protein-coupled receptor class C group 5 member C [Source:HGNC Symbol;Acc:HGNC:13309] |
| ENSG00000170667 | HGLibA_40309 | 45 | -0.727653059 | RASA4B | RAS p21 protein activator 4B [Source:HGNC Symbol;Acc:HGNC:35202] |
| ENSG00000171130 | HGLibA_03726 | 45 | -0.727653059 | ATP6V0E2 | ATPase H+ transporting V0 subunit e2 [Source:HGNC Symbol;Acc:HGNC:21723] |
| ENSG00000171574 | HGLibA_56318 | 45 | -0.727653059 | ZNF584 | zinc finger protein 584 [Source:HGNC Symbol;Acc:HGNC:27318] |
| ENSG00000171862 | HGLibA_39295 | 45 | -0.727653059 | PTEN | phosphatase and tensin homolog [Source:HGNC Symbol;Acc:HGNC:9588] |
| ENSG00000172331 | HGLibA_04702 | 45 | -0.727653059 | BPGM | bisphosphoglycerate mutase [Source:HGNC Symbol;Acc:HGNC:1093] |
| ENSG00000173175 | HGLibA_00919 | 45 | -0.727653059 | ADCY5 | adenylate cyclase 5 [Source:HGNC Symbol;Acc:HGNC:236] |
| ENSG00000173198 | HGLibA_12227 | 45 | -0.727653059 | CYSLTR1 | cysteinyl leukotriene receptor 1 [Source:HGNC Symbol;Acc:HGNC:17451] |
| ENSG00000173432 | HGLibA_42608 | 45 | -0.727653059 | SAA1 | serum amyloid A1 [Source:HGNC Symbol;Acc:HGNC:10513] |
| ENSG00000174013 | HGLibA_17107 | 45 | -0.727653059 | FBXO45 | F-box protein 45 [Source:HGNC Symbol;Acc:HGNC:29148] |
| ENSG00000175536 | HGLibA_26636 | 45 | -0.727653059 | LIPT2 | lipoyl(octanoyl) transferase 2 [Source:HGNC Symbol;Acc:HGNC:37216] |
| ENSG00000175691 | HGLibA_56701 | 45 | -0.727653059 | ZNF77 | zinc finger protein 77 [Source:HGNC Symbol;Acc:HGNC:13150] |
| ENSG00000175820 | HGLibA_07678 | 45 | -0.727653059 | CCDC168 | coiled-coil domain containing 168 [Source:HGNC Symbol;Acc:HGNC:26851] |
| ENSG00000176108 | HGLibA_09498 | 45 | -0.727653059 | CHMP6 | charged multivesicular body protein 6 [Source:HGNC Symbol;Acc:HGNC:25675] |
| ENSG00000176148 | HGLibA_48845 | 45 | -0.727653059 | TCP11L1 | t-complex 11 like 1 [Source:HGNC Symbol;Acc:HGNC:25655] |
| ENSG00000176720 | HGLibA_04676 | 45 | -0.727653059 | BOK | BCL2 family apoptosis regulator BOK [Source:HGNC Symbol;Acc:HGNC:1087] |

| | | | | | |
|---|---|---|---|---|---|
| ENSG00000177511 | HGLibA_47294 | 45 | -0.727653059 | ST8SIA3 | 8-sialyltransferase 3 [Source:HGNC Symbol;Acc:HGNC:14269]" |
| ENSG00000178093 | HGLibA_52021 | 45 | -0.727653059 | TSSK6 | testis specific serine kinase 6 [Source:HGNC Symbol;Acc:HGNC:30410] |
| ENSG00000178252 | HGLibA_54137 | 45 | -0.727653059 | WDR6 | WD repeat domain 6 [Source:HGNC Symbol;Acc:HGNC:12758] |
| ENSG00000178773 | HGLibA_10971 | 45 | -0.727653059 | CPNE7 | copine 7 [Source:HGNC Symbol;Acc:HGNC:2320] |
| ENSG00000178852 | HGLibA_14530 | 45 | -0.727653059 | EFCAB13 | EF-hand calcium binding domain 13 [Source:HGNC Symbol;Acc:HGNC:26864] |
| ENSG00000179772 | HGLibA_17958 | 45 | -0.727653059 | FOXS1 | forkhead box S1 [Source:HGNC Symbol;Acc:HGNC:3735] |
| ENSG00000180287 | HGLibA_37047 | 45 | -0.727653059 | PLD5 | phospholipase D family member 5 [Source:HGNC Symbol;Acc:HGNC:26879] |
| ENSG00000181458 | HGLibA_50407 | 45 | -0.727653059 | TMEM45A | transmembrane protein 45A [Source:HGNC Symbol;Acc:HGNC:25480] |
| ENSG00000182512 | HGLibA_19322 | 45 | -0.727653059 | GLRX5 | glutaredoxin 5 [Source:HGNC Symbol;Acc:HGNC:20134] |
| ENSG00000182870 | HGLibA_18575 | 45 | -0.727653059 | GALNT9 | polypeptide N-acetylgalactosaminyltransferase 9 [Source:HGNC Symbol;Acc:HGNC:4131] |
| ENSG00000183421 | HGLibA_41324 | 45 | -0.727653059 | RIPK4 | receptor interacting serine/threonine kinase 4 [Source:HGNC Symbol;Acc:HGNC:496] |
| ENSG00000184226 | HGLibA_35421 | 45 | -0.727653059 | PCDH9 | protocadherin 9 [Source:HGNC Symbol;Acc:HGNC:8661] |
| ENSG00000184361 | HGLibA_46506 | 45 | -0.727653059 | SPATA32 | spermatogenesis associated 32 [Source:HGNC Symbol;Acc:HGNC:26349] |
| ENSG00000184863 | HGLibA_40532 | 45 | -0.727653059 | RBM33 | RNA binding motif protein 33 [Source:HGNC Symbol;Acc:HGNC:27223] |
| ENSG00000184983 | HGLibA_31467 | 45 | -0.727653059 | NDUFA6 | NADH:ubiquinone oxidoreductase subunit A6 [Source:HGNC Symbol;Acc:HGNC:7690] |
| ENSG00000185950 | HGLibA_23621 | 45 | -0.727653059 | IRS2 | insulin receptor substrate 2 [Source:HGNC Symbol;Acc:HGNC:6126] |
| ENSG00000185962 | HGLibA_26131 | 45 | -0.727653059 | LCE3A | late cornified envelope 3A [Source:HGNC Symbol;Acc:HGNC:29461] |
| ENSG00000186197 | HGLibA_14440 | 45 | -0.727653059 | EDARADD | EDAR associated death domain [Source:HGNC Symbol;Acc:HGNC:14341] |
| ENSG00000186567 | HGLibA_08928 | 45 | -0.727653059 | CEACAM19 | carcinoembryonic antigen related cell adhesion molecule 19 [Source:HGNC Symbol;Acc:HGNC:31951] |
| ENSG00000186918 | HGLibA_55913 | 45 | -0.727653059 | ZNF395 | zinc finger protein 395 [Source:HGNC Symbol;Acc:HGNC:18737] |
| ENSG00000187479 | HGLibA_05221 | 45 | -0.727653059 | C11orf96 | chromosome 11 open reading frame 96 [Source:HGNC Symbol;Acc:HGNC:38675] |
| ENSG00000187559 | HGLibA_17846 | 45 | -0.727653059 | FOXD4L3 | forkhead box D4 like 3 [Source:HGNC Symbol;Acc:HGNC:18523] |
| ENSG00000188501 | HGLibA_26207 | 45 | -0.727653059 | LCTL | lactase like [Source:HGNC Symbol;Acc:HGNC:15583] |
| ENSG00000188996 | HGLibA_22427 | 45 | -0.727653059 | HUS1B | HUS1 checkpoint clamp component B [Source:HGNC Symbol;Acc:HGNC:16485] |
| ENSG00000196267 | HGLibA_56843 | 45 | -0.727653059 | ZNF836 | zinc finger protein 836 [Source:HGNC Symbol;Acc:HGNC:34333] |
| ENSG00000196284 | HGLibA_47814 | 45 | -0.727653059 | SUPT3H | SAGA and STAGA complex component [Source:HGNC Symbol;Acc:HGNC:11466]" |
| ENSG00000196344 | HGLibA_00966 | 45 | -0.727653059 | ADH7 | mu or sigma polypeptide [Source:HGNC Symbol;Acc:HGNC:256]" |
| ENSG00000196504 | HGLibA_38734 | 45 | -0.727653059 | PRPF40A | pre-mRNA processing factor 40 homolog A [Source:HGNC Symbol;Acc:HGNC:16463] |
| ENSG00000196862 | HGLibA_41034 | 45 | -0.727653059 | RGPD4 | RANBP2-like and GRIP domain containing 4 [Source:HGNC Symbol;Acc:HGNC:32417] |
| ENSG00000197261 | HGLibA_06450 | 45 | -0.727653059 | C6orf141 | chromosome 6 open reading frame 141 [Source:HGNC Symbol;Acc:HGNC:21351] |
| ENSG00000197694 | HGLibA_46875 | 45 | -0.727653059 | SPTAN1 | non-erythrocytic 1 [Source:HGNC Symbol;Acc:HGNC:11273]" |
| ENSG00000197721 | HGLibA_11047 | 45 | -0.727653059 | CR1L | complement C3b/C4b receptor 1 like [Source:HGNC Symbol;Acc:HGNC:2335] |
| ENSG00000197953 | HGLibA_00033 | 45 | -0.727653059 | AADACL2 | arylacetamide deacetylase like 2 [Source:HGNC Symbol;Acc:HGNC:24427] |
| ENSG00000198393 | HGLibA_55686 | 45 | -0.727653059 | ZNF26 | zinc finger protein 26 [Source:HGNC Symbol;Acc:HGNC:13053] |
| ENSG00000198440 | HGLibA_56314 | 45 | -0.727653059 | ZNF583 | zinc finger protein 583 [Source:HGNC Symbol;Acc:HGNC:26427] |
| ENSG00000198492 | HGLibA_54733 | 45 | -0.727653059 | YTHDF2 | YTH N6-methyladenosine RNA binding protein 2 [Source:HGNC Symbol;Acc:HGNC:31675] |
| ENSG00000198746 | HGLibA_19744 | 45 | -0.727653059 | GPATCH3 | G-patch domain containing 3 [Source:HGNC Symbol;Acc:HGNC:25720] |
| ENSG00000198793 | HGLibA_30454 | 45 | -0.727653059 | MTOR | mechanistic target of rapamycin kinase [Source:HGNC Symbol;Acc:HGNC:3942] |
| ENSG00000198890 | HGLibA_38631 | 45 | -0.727653059 | PRMT6 | protein arginine methyltransferase 6 [Source:HGNC Symbol;Acc:HGNC:18241] |
| ENSG00000198901 | HGLibA_38349 | 45 | -0.727653059 | PRC1 | protein regulator of cytokinesis 1 [Source:HGNC Symbol;Acc:HGNC:9341] |
| ENSG00000198933 | HGLibA_48606 | 45 | -0.727653059 | TBKBP1 | TBK1 binding protein 1 [Source:HGNC Symbol;Acc:HGNC:30140] |
| ENSG00000204099 | HGLibA_31719 | 45 | -0.727653059 | NEU4 | neuraminidase 4 [Source:HGNC Symbol;Acc:HGNC:21328] |
| ENSG00000204612 | HGLibA_17819 | 45 | -0.727653059 | FOXB2 | forkhead box B2 [Source:HGNC Symbol;Acc:HGNC:23315] |
| ENSG00000205097 | HGLibA_17997 | 45 | -0.727653059 | FRG2 | FSHD region gene 2 [Source:HGNC Symbol;Acc:HGNC:19136] |
| ENSG00000205329 | HGLibA_34388 | 45 | -0.727653059 | OR6C3 | olfactory receptor family 6 subfamily C member 3 [Source:HGNC Symbol;Acc:HGNC:15437] |
| ENSG00000205352 | HGLibA_38775 | 45 | -0.727653059 | PRR13 | proline rich 13 [Source:HGNC Symbol;Acc:HGNC:24528] |
| ENSG00000205916 | HGLibA_12362 | 45 | -0.727653059 | DAZ4 | deleted in azoospermia 4 [Source:HGNC Symbol;Acc:HGNC:15966] |
| ENSG00000213762 | HGLibA_55469 | 45 | -0.727653059 | ZNF134 | zinc finger protein 134 [Source:HGNC Symbol;Acc:HGNC:12918] |
| ENSG00000214643 | HGLibA_12902 | 45 | -0.727653059 | DEFB133 | defensin beta 133 [Source:HGNC Symbol;Acc:HGNC:31331] |
| ENSG00000215126 | HGLibA_07442 | 45 | -0.727653059 | CBWD6 | COBW domain containing 6 [Source:HGNC Symbol;Acc:HGNC:31978] |
| ENSG00000215472 | HGLibA_41882 | 45 | -0.727653059 | RPL17-C18orf32 | RPL17-C18orf32 readthrough [Source:HGNC Symbol;Acc:HGNC:44661] |
| ENSG00000222011 | HGLibA_16332 | 45 | -0.727653059 | FAM185A | family with sequence similarity 185 member A [Source:HGNC Symbol;Acc:HGNC:22412] |
| ENSG00000223611 | HGLibA_47810 | 45 | -0.727653059 | SUPT20HL2 | SUPT20H like 2 [Source:HGNC Symbol;Acc:HGNC:31797] |
| ENSG00000227471 | HGLibA_01479 | 45 | -0.727653059 | AKR1B15 | aldo-keto reductase family 1 member B15 [Source:HGNC Symbol;Acc:HGNC:37281] |
| ENSG00000227507 | HGLibA_27582 | 45 | -0.727653059 | LTB | lymphotoxin beta [Source:HGNC Symbol;Acc:HGNC:6711] |
| ENSG00000230430 | HGLibA_53258 | 45 | -0.727653059 | USP17L25 | ubiquitin specific peptidase 17-like family member 25 [Source:HGNC Symbol;Acc:HGNC:44452] |
| ENSG00000236027 | HGLibA_35278 | 45 | -0.727653059 | PATE3 | prostate and testis expressed 3 [Source:HGNC Symbol;Acc:HGNC:35426] |
| ENSG00000240053 | HGLibA_27660 | 45 | -0.727653059 | LY6G5B | lymphocyte antigen 6 family member G5B [Source:HGNC Symbol;Acc:HGNC:13931] |
| ENSG00000240891 | HGLibA_37026 | 45 | -0.727653059 | PLCXD2 | phosphatidylinositol specific phospholipase C X domain containing 2 [Source:HGNC Symbol;Acc:HGNC:26462] |
| ENSG00000240972 | HGLibA_29261 | 45 | -0.727653059 | MIF | macrophage migration inhibitory factor [Source:HGNC Symbol;Acc:HGNC:7097] |
| ENSG00000241878 | HGLibA_36734 | 45 | -0.727653059 | PISD | phosphatidylserine decarboxylase [Source:HGNC Symbol;Acc:HGNC:8999] |
| ENSG00000247596 | HGLibA_52371 | 45 | -0.727653059 | TWF2 | twinfilin actin binding protein 2 [Source:HGNC Symbol;Acc:HGNC:9621] |
| ENSG00000249471 | HGLibA_55804 | 45 | -0.727653059 | ZNF324B | zinc finger protein 324B [Source:HGNC Symbol;Acc:HGNC:33107] |
| ENSG00000253831 | HGLibA_15657 | 45 | -0.727653059 | ETV3L | ETS variant 3 like [Source:HGNC Symbol;Acc:HGNC:33834] |
| ENSG00000254788 | HGLibA_09772 | 45 | -0.727653059 | CKLF-CMTM1 | CKLF-CMTM1 readthrough [Source:HGNC Symbol;Acc:HGNC:39977] |
| ENSG00000265972 | HGLibA_52431 | 45 | -0.727653059 | TXNIP | thioredoxin interacting protein [Source:HGNC Symbol;Acc:HGNC:16952] |
| ENSG00000266074 | HGLibA_04107 | 45 | -0.727653059 | BAHCC1 | BAH domain and coiled-coil containing 1 [Source:HGNC Symbol;Acc:HGNC:29279] |
| ENSG00000267796 | HGLibA_26564 | 45 | -0.727653059 | LIN37 | lin-37 DREAM MuvB core complex component [Source:HGNC Symbol;Acc:HGNC:33234] |
| ENSG00000269096 | HGLibA_11503 | 45 | -0.727653059 | CT45A3 | cancer/testis antigen family 45 member A3 [Source:HGNC Symbol;Acc:HGNC:33268] |
| ENSG00000273514 | HGLibA_17855 | 45 | -0.727653059 | FOXD4L6 | forkhead box D4 like 6 [Source:HGNC Symbol;Acc:HGNC:31986] |
| ENSG00000284862 | HGLibA_07771 | 45 | -0.727653059 | CCDC39 | coiled-coil domain containing 39 [Source:NCBI gene;Acc:339829] |
| GAREML | HGLibA_18629 | 45 | -0.727653059 | GAREML | - |
| H2AFJ | HGLibA_20754 | 45 | -0.727653059 | H2AFJ | - |
| HIST1H1D | HGLibA_21365 | 45 | -0.727653059 | HIST1H1D | - |
| HIST1H2AA | HGLibA_21376 | 45 | -0.727653059 | HIST1H2AA | - |
| hsa-let-7f-1 | HGLibA_57127 | 45 | -0.727653059 | hsa-let-7f-1 | - |
| hsa-mir-10b | HGLibA_57203 | 45 | -0.727653059 | hsa-mir-10b | - |
| hsa-mir-1207 | HGLibA_57282 | 45 | -0.727653059 | hsa-mir-1207 | - |
| hsa-mir-1255b-1 | HGLibA_57424 | 45 | -0.727653059 | hsa-mir-1255b-1 | - |
| hsa-mir-1277 | HGLibA_57562 | 45 | -0.727653059 | hsa-mir-1277 | - |
| hsa-mir-197 | HGLibA_58103 | 45 | -0.727653059 | hsa-mir-197 | - |
| hsa-mir-199a-2 | HGLibA_58134 | 45 | -0.727653059 | hsa-mir-199a-2 | - |
| hsa-mir-200b | HGLibA_58155 | 45 | -0.727653059 | hsa-mir-200b | - |
| hsa-mir-299 | HGLibA_58406 | 45 | -0.727653059 | hsa-mir-299 | - |
| hsa-mir-345 | HGLibA_59028 | 45 | -0.727653059 | hsa-mir-345 | - |
| hsa-mir-3681 | HGLibA_59276 | 45 | -0.727653059 | hsa-mir-3681 | - |
| hsa-mir-371a | HGLibA_59375 | 45 | -0.727653059 | hsa-mir-371a | - |
| hsa-mir-371b | HGLibA_59377 | 45 | -0.727653059 | hsa-mir-371b | - |
| hsa-mir-3942 | HGLibA_59628 | 45 | -0.727653059 | hsa-mir-3942 | - |
| hsa-mir-412 | HGLibA_59688 | 45 | -0.727653059 | hsa-mir-412 | - |
| hsa-mir-4325 | HGLibA_60022 | 45 | -0.727653059 | hsa-mir-4325 | - |
| hsa-mir-4422 | HGLibA_60075 | 45 | -0.727653059 | hsa-mir-4422 | - |
| hsa-mir-4423 | HGLibA_60079 | 45 | -0.727653059 | hsa-mir-4423 | - |
| hsa-mir-4510 | HGLibA_60483 | 45 | -0.727653059 | hsa-mir-4510 | - |
| hsa-mir-4520b | HGLibA_60539 | 45 | -0.727653059 | hsa-mir-4520b | - |
| hsa-mir-4524a | HGLibA_60552 | 45 | -0.727653059 | hsa-mir-4524a | - |
| hsa-mir-4645 | HGLibA_60685 | 45 | -0.727653059 | hsa-mir-4645 | - |
| hsa-mir-4659a | HGLibA_60744 | 45 | -0.727653059 | hsa-mir-4659a | - |
| hsa-mir-4675 | HGLibA_60815 | 45 | -0.727653059 | hsa-mir-4675 | - |
| hsa-mir-4705 | HGLibA_60920 | 45 | -0.727653059 | hsa-mir-4705 | - |
| hsa-mir-4753 | HGLibA_61111 | 45 | -0.727653059 | hsa-mir-4753 | - |
| hsa-mir-4772 | HGLibA_61188 | 45 | -0.727653059 | hsa-mir-4772 | - |
| hsa-mir-4798 | HGLibA_61297 | 45 | -0.727653059 | hsa-mir-4798 | - |
| hsa-mir-485 | HGLibA_61333 | 45 | -0.727653059 | hsa-mir-485 | - |
| hsa-mir-520b | HGLibA_61740 | 45 | -0.727653059 | hsa-mir-520b | - |
| hsa-mir-544a | HGLibA_61823 | 45 | -0.727653059 | hsa-mir-544a | - |
| hsa-mir-548l | HGLibA_62038 | 45 | -0.727653059 | hsa-mir-548l | - |
| hsa-mir-548z | HGLibA_62090 | 45 | -0.727653059 | hsa-mir-548z | - |
| hsa-mir-561 | HGLibA_62209 | 45 | -0.727653059 | hsa-mir-561 | - |
| hsa-mir-5682 | HGLibA_62247 | 45 | -0.727653059 | hsa-mir-5682 | - |
| hsa-mir-6090 | HGLibA_62627 | 45 | -0.727653059 | hsa-mir-6090 | - |
| hsa-mir-670 | HGLibA_63010 | 45 | -0.727653059 | hsa-mir-670 | - |
| hsa-mir-6768 | HGLibA_63232 | 45 | -0.727653059 | hsa-mir-6768 | - |
| hsa-mir-6805 | HGLibA_63398 | 45 | -0.727653059 | hsa-mir-6805 | - |
| hsa-mir-6814 | HGLibA_63432 | 45 | -0.727653059 | hsa-mir-6814 | - |
| hsa-mir-7851 | HGLibA_63993 | 45 | -0.727653059 | hsa-mir-7851 | - |
| hsa-mir-8067 | HGLibA_64106 | 45 | -0.727653059 | hsa-mir-8067 | - |
| hsa-mir-877 | HGLibA_64220 | 45 | -0.727653059 | hsa-mir-877 | - |
| KIAA0196 | HGLibA_24602 | 45 | -0.727653059 | KIAA0196 | - |
| KIAA1161 | HGLibA_24703 | 45 | -0.727653059 | KIAA1161 | - |
| KIAA1432 | HGLibA_24757 | 45 | -0.727653059 | KIAA1432 | - |
| LOC642799 | HGLibA_26959 | 45 | -0.727653059 | LOC642799 | - |
| LOH12CR1 | HGLibA_27012 | 45 | -0.727653059 | LOH12CR1 | - |
| LRMP | HGLibA_27193 | 45 | -0.727653059 | LRMP | - |
| NAPRT1 | HGLibA_31138 | 45 | -0.727653059 | NAPRT1 | - |
| NUTMF | HGLibA_33115 | 45 | -0.727653059 | NUTMF | - |
| PAK7 | HGLibA_35056 | 45 | -0.727653059 | PAK7 | - |
| PPAPDC2 | HGLibA_37831 | 45 | -0.727653059 | PPAPDC2 | - |
| PYCRL | HGLibA_39680 | 45 | -0.727653059 | PYCRL | - |
| RGAG4 | HGLibA_40994 | 45 | -0.727653059 | RGAG4 | - |
| SLMO2 | HGLibA_45540 | 45 | -0.727653059 | SLMO2 | - |
| TCEB3C | HGLibA_48745 | 45 | -0.727653059 | TCEB3C | - |
| ZCCHC16 | HGLibA_55017 | 45 | -0.727653059 | ZCCHC16 | - |
| 1-Dec | HGLibA_12772 | 44 | -0.736698402 | 1-Dec | - |
| ALS2CR12 | HGLibA_01728 | 44 | -0.736698402 | ALS2CR12 | - |

| | | | | | |
|---|---|---|---|---|---|
| ASMT_Y | HGLibA_03323 | 44 | -0.736698402 | ASMT_Y | - |
| BAI1 | HGLibA_04113 | 44 | -0.736698402 | BAI1 | - |
| C19orf82 | HGLibA_05718 | 44 | -0.736698402 | C19orf82 | - |
| C7orf72 | HGLibA_06587 | 44 | -0.736698402 | C7orf72 | - |
| C8orf4 | HGLibA_06618 | 44 | -0.736698402 | C8orf4 | - |
| C9orf84 | HGLibA_06763 | 44 | -0.736698402 | C9orf84 | - |
| CXorf30 | HGLibA_11915 | 44 | -0.736698402 | CXorf30 | - |
| DIEXF | HGLibA_13232 | 44 | -0.736698402 | DIEXF | - |
| ENSG00000005156 | HGLibA_26484 | 44 | -0.736698402 | LIG3 | DNA ligase 3 [Source:HGNC Symbol;Acc:HGNC:6600] |
| ENSG00000005302 | HGLibA_30217 | 44 | -0.736698402 | MSL3 | MSL complex subunit 3 [Source:HGNC Symbol;Acc:HGNC:7370] |
| ENSG00000005483 | HGLibA_25334 | 44 | -0.736698402 | KMT2E | lysine methyltransferase 2E [Source:HGNC Symbol;Acc:HGNC:18541] |
| ENSG00000010282 | HGLibA_21256 | 44 | -0.736698402 | HHATL | hedgehog acyltransferase like [Source:HGNC Symbol;Acc:HGNC:13242] |
| ENSG00000010319 | HGLibA_43338 | 44 | -0.736698402 | SEMA3G | semaphorin 3G [Source:HGNC Symbol;Acc:HGNC:30400] |
| ENSG00000010361 | HGLibA_18211 | 44 | -0.736698402 | FUZ | fuzzy planar cell polarity protein [Source:HGNC Symbol;Acc:HGNC:26219] |
| ENSG00000020256 | HGLibA_55227 | 44 | -0.736698402 | ZFP64 | ZFP64 zinc finger protein [Source:HGNC Symbol;Acc:HGNC:15940] |
| ENSG00000055211 | HGLibA_19100 | 44 | -0.736698402 | GINM1 | glycoprotein integral membrane 1 [Source:HGNC Symbol;Acc:HGNC:21074] |
| ENSG00000065717 | HGLibA_49611 | 44 | -0.736698402 | TLE2 | transducin like enhancer of split 2 [Source:HGNC Symbol;Acc:HGNC:11838] |
| ENSG00000069345 | HGLibA_13512 | 44 | -0.736698402 | DNAJA2 | DnaJ heat shock protein family (Hsp40) member A2 [Source:HGNC Symbol;Acc:HGNC:14884] |
| ENSG00000070423 | HGLibA_41496 | 44 | -0.736698402 | RNF126 | ring finger protein 126 [Source:HGNC Symbol;Acc:HGNC:21151] |
| ENSG00000075568 | HGLibA_49928 | 44 | -0.736698402 | TMEM131 | transmembrane protein 131 [Source:HGNC Symbol;Acc:HGNC:30366] |
| ENSG00000076258 | HGLibA_17711 | 44 | -0.736698402 | FMO4 | flavin containing monooxygenase 4 [Source:HGNC Symbol;Acc:HGNC:3772] |
| ENSG00000076770 | HGLibA_28515 | 44 | -0.736698402 | MBNL3 | muscleblind like splicing regulator 3 [Source:HGNC Symbol;Acc:HGNC:20564] |
| ENSG00000077782 | HGLibA_17436 | 44 | -0.736698402 | FGFR1 | fibroblast growth factor receptor 1 [Source:HGNC Symbol;Acc:HGNC:3688] |
| ENSG00000079308 | HGLibA_50921 | 44 | -0.736698402 | TNS1 | tensin 1 [Source:HGNC Symbol;Acc:HGNC:11973] |
| ENSG00000080822 | HGLibA_09915 | 44 | -0.736698402 | CLDND1 | claudin domain containing 1 [Source:HGNC Symbol;Acc:HGNC:1322] |
| ENSG00000081692 | HGLibA_23956 | 44 | -0.736698402 | JMJD4 | jumonji domain containing 4 [Source:HGNC Symbol;Acc:HGNC:25724] |
| ENSG00000085552 | HGLibA_22904 | 44 | -0.736698402 | IGSF9 | immunoglobulin superfamily member 9 [Source:HGNC Symbol;Acc:HGNC:18132] |
| ENSG00000086544 | HGLibA_23849 | 44 | -0.736698402 | ITPKC | inositol-trisphosphate 3-kinase C [Source:HGNC Symbol;Acc:HGNC:14897] |
| ENSG00000090273 | HGLibA_32919 | 44 | -0.736698402 | NUDC | dynein complex regulator [Source:HGNC Symbol;Acc:HGNC:8045]" |
| ENSG00000092096 | HGLibA_44559 | 44 | -0.736698402 | SLC22A17 | solute carrier family 22 member 17 [Source:HGNC Symbol;Acc:HGNC:23095] |
| ENSG00000093010 | HGLibA_10685 | 44 | -0.736698402 | COMT | catechol-O-methyltransferase [Source:HGNC Symbol;Acc:HGNC:2228] |
| ENSG00000095787 | HGLibA_53939 | 44 | -0.736698402 | WAC | WW domain containing adaptor with coiled-coil [Source:HGNC Symbol;Acc:HGNC:17327] |
| ENSG00000099937 | HGLibA_43573 | 44 | -0.736698402 | SERPIND1 | serpin family D member 1 [Source:HGNC Symbol;Acc:HGNC:4838] |
| ENSG00000100439 | HGLibA_00277 | 44 | -0.736698402 | ABHD4 | abhydrolase domain containing 4 [Source:HGNC Symbol;Acc:HGNC:20154] |
| ENSG00000100908 | HGLibA_15025 | 44 | -0.736698402 | EMC9 | ER membrane protein complex subunit 9 [Source:HGNC Symbol;Acc:HGNC:20273] |
| ENSG00000101191 | HGLibA_13229 | 44 | -0.736698402 | DIDO1 | death inducer-obliterator 1 [Source:HGNC Symbol;Acc:HGNC:2680] |
| ENSG00000101337 | HGLibA_49730 | 44 | -0.736698402 | TM9SF4 | transmembrane 9 superfamily member 4 [Source:HGNC Symbol;Acc:HGNC:30797] |
| ENSG00000101363 | HGLibA_28087 | 44 | -0.736698402 | MANBAL | mannosidase beta like [Source:HGNC Symbol;Acc:HGNC:15799] |
| ENSG00000102580 | HGLibA_13616 | 44 | -0.736698402 | DNAJC3 | DnaJ heat shock protein family (Hsp40) member C3 [Source:HGNC Symbol;Acc:HGNC:9439] |
| ENSG00000103005 | HGLibA_53160 | 44 | -0.736698402 | USB1 | U6 snRNA biogenesis phosphodiesterase 1 [Source:HGNC Symbol;Acc:HGNC:25792] |
| ENSG00000103021 | HGLibA_07547 | 44 | -0.736698402 | CCDC113 | coiled-coil domain containing 113 [Source:HGNC Symbol;Acc:HGNC:25002] |
| ENSG00000103044 | HGLibA_20867 | 44 | -0.736698402 | HAS3 | hyaluronan synthase 3 [Source:HGNC Symbol;Acc:HGNC:4820] |
| ENSG00000103197 | HGLibA_51815 | 44 | -0.736698402 | TSC2 | TSC complex subunit 2 [Source:HGNC Symbol;Acc:HGNC:12363] |
| ENSG00000104064 | HGLibA_18349 | 44 | -0.736698402 | GABPB1 | GA binding protein transcription factor subunit beta 1 [Source:HGNC Symbol;Acc:HGNC:4074] |
| ENSG00000104689 | HGLibA_50732 | 44 | -0.736698402 | TNFRSF10A | TNF receptor superfamily member 10a [Source:HGNC Symbol;Acc:HGNC:11904] |
| ENSG00000104691 | HGLibA_52797 | 44 | -0.736698402 | UBXN8 | UBX domain protein 8 [Source:HGNC Symbol;Acc:HGNC:30307] |
| ENSG00000104889 | HGLibA_41428 | 44 | -0.736698402 | RNASEH2A | ribonuclease H2 subunit A [Source:HGNC Symbol;Acc:HGNC:18518] |
| ENSG00000104972 | HGLibA_26508 | 44 | -0.736698402 | LILRB1 | leukocyte immunoglobulin like receptor B1 [Source:HGNC Symbol;Acc:HGNC:6605] |
| ENSG00000105202 | HGLibA_16936 | 44 | -0.736698402 | FBL | fibrillarin [Source:HGNC Symbol;Acc:HGNC:3599] |
| ENSG00000105246 | HGLibA_14361 | 44 | -0.736698402 | EBI3 | Epstein-Barr virus induced 3 [Source:HGNC Symbol;Acc:HGNC:3129] |
| ENSG00000105281 | HGLibA_44518 | 44 | -0.736698402 | SLC1A5 | solute carrier family 1 member 5 [Source:HGNC Symbol;Acc:HGNC:10943] |
| ENSG00000105499 | HGLibA_36906 | 44 | -0.736698402 | PLA2G4C | phospholipase A2 group IVC [Source:HGNC Symbol;Acc:HGNC:9037] |
| ENSG00000105641 | HGLibA_45275 | 44 | -0.736698402 | SLC5A5 | solute carrier family 5 member 5 [Source:HGNC Symbol;Acc:HGNC:11040] |
| ENSG00000105894 | HGLibA_39396 | 44 | -0.736698402 | PTN | pleiotrophin [Source:HGNC Symbol;Acc:HGNC:9630] |
| ENSG00000105991 | HGLibA_21892 | 44 | -0.736698402 | HOXA1 | homeobox A1 [Source:HGNC Symbol;Acc:HGNC:5099] |
| ENSG00000106266 | HGLibA_46108 | 44 | -0.736698402 | SNX8 | sorting nexin 8 [Source:HGNC Symbol;Acc:HGNC:14972] |
| ENSG00000106829 | HGLibA_49616 | 44 | -0.736698402 | TLE4 | transducin like enhancer of split 4 [Source:HGNC Symbol;Acc:HGNC:11840] |
| ENSG00000107020 | HGLibA_37166 | 44 | -0.736698402 | PLGRKT | plasminogen receptor with a C-terminal lysine [Source:HGNC Symbol;Acc:HGNC:23633] |
| ENSG00000107796 | HGLibA_00598 | 44 | -0.736698402 | ACTA2 | aorta [Source:HGNC Symbol;Acc:HGNC:130]" |
| ENSG00000107874 | HGLibA_11769 | 44 | -0.736698402 | CUEDC2 | CUE domain containing 2 [Source:HGNC Symbol;Acc:HGNC:28352] |
| ENSG00000108599 | HGLibA_01406 | 44 | -0.736698402 | AKAP10 | A-kinase anchoring protein 10 [Source:HGNC Symbol;Acc:HGNC:368] |
| ENSG00000109182 | HGLibA_11829 | 44 | -0.736698402 | CWH43 | cell wall biogenesis 43 C-terminal homolog [Source:HGNC Symbol;Acc:HGNC:26133] |
| ENSG00000111644 | HGLibA_00533 | 44 | -0.736698402 | ACRBP | acrosin binding protein [Source:HGNC Symbol;Acc:HGNC:17195] |
| ENSG00000112972 | HGLibA_21711 | 44 | -0.736698402 | HMGCS1 | 3-hydroxy-3-methylglutaryl-CoA synthase 1 [Source:HGNC Symbol;Acc:HGNC:5007] |
| ENSG00000114841 | HGLibA_13467 | 44 | -0.736698402 | DNAH1 | dynein axonemal heavy chain 1 [Source:HGNC Symbol;Acc:HGNC:2940] |
| ENSG00000115112 | HGLibA_49147 | 44 | -0.736698402 | TFCP2L1 | transcription factor CP2 like 1 [Source:HGNC Symbol;Acc:HGNC:17925] |
| ENSG00000115221 | HGLibA_23793 | 44 | -0.736698402 | ITGB6 | integrin subunit beta 6 [Source:HGNC Symbol;Acc:HGNC:6161] |
| ENSG00000115761 | HGLibA_32256 | 44 | -0.736698402 | NOL10 | nucleolar protein 10 [Source:HGNC Symbol;Acc:HGNC:25862] |
| ENSG00000116704 | HGLibA_44977 | 44 | -0.736698402 | SLC35D1 | solute carrier family 35 member D1 [Source:HGNC Symbol;Acc:HGNC:20800] |
| ENSG00000116962 | HGLibA_31924 | 44 | -0.736698402 | NID1 | nidogen 1 [Source:HGNC Symbol;Acc:HGNC:7821] |
| ENSG00000118058 | HGLibA_25320 | 44 | -0.736698402 | KMT2A | lysine methyltransferase 2A [Source:HGNC Symbol;Acc:HGNC:7132] |
| ENSG00000119630 | HGLibA_36234 | 44 | -0.736698402 | PGF | placental growth factor [Source:HGNC Symbol;Acc:HGNC:8893] |
| ENSG00000119950 | HGLibA_30620 | 44 | -0.736698402 | MXI1 | dimerization protein [Source:HGNC Symbol;Acc:HGNC:7534]" |
| ENSG00000120210 | HGLibA_23392 | 44 | -0.736698402 | INSL6 | insulin like 6 [Source:HGNC Symbol;Acc:HGNC:6089] |
| ENSG00000121964 | HGLibA_20514 | 44 | -0.736698402 | GTDC1 | glycosyltransferase like domain containing 1 [Source:HGNC Symbol;Acc:HGNC:20887] |
| ENSG00000122012 | HGLibA_47881 | 44 | -0.736698402 | SV2C | synaptic vesicle glycoprotein 2C [Source:HGNC Symbol;Acc:HGNC:30670] |
| ENSG00000122203 | HGLibA_24706 | 44 | -0.736698402 | KIAA1191 | KIAA1191 [Source:HGNC Symbol;Acc:HGNC:29209] |
| ENSG00000122591 | HGLibA_16081 | 44 | -0.736698402 | FAM126A | family with sequence similarity 126 member A [Source:HGNC Symbol;Acc:HGNC:24587] |
| ENSG00000123191 | HGLibA_03773 | 44 | -0.736698402 | ATP7B | ATPase copper transporting beta [Source:HGNC Symbol;Acc:HGNC:870] |
| ENSG00000123338 | HGLibA_31313 | 44 | -0.736698402 | NCKAP1L | NCK associated protein 1 like [Source:HGNC Symbol;Acc:HGNC:4862] |
| ENSG00000123505 | HGLibA_01757 | 44 | -0.736698402 | AMD1 | adenosylmethionine decarboxylase 1 [Source:HGNC Symbol;Acc:HGNC:457] |
| ENSG00000125430 | HGLibA_22131 | 44 | -0.736698402 | HS3ST3B1 | heparan sulfate-glucosamine 3-sulfotransferase 3B1 [Source:HGNC Symbol;Acc:HGNC:5198] |
| ENSG00000127399 | HGLibA_27407 | 44 | -0.736698402 | LRRC61 | leucine rich repeat containing 61 [Source:HGNC Symbol;Acc:HGNC:21704] |
| ENSG00000129194 | HGLibA_46242 | 44 | -0.736698402 | SOX15 | SRY-box 15 [Source:HGNC Symbol;Acc:HGNC:11196] |
| ENSG00000129250 | HGLibA_24888 | 44 | -0.736698402 | KIF1C | kinesin family member 1C [Source:HGNC Symbol;Acc:HGNC:6317] |
| ENSG00000129562 | HGLibA_12290 | 44 | -0.736698402 | DAD1 | defender against cell death 1 [Source:HGNC Symbol;Acc:HGNC:2664] |
| ENSG00000130751 | HGLibA_32393 | 44 | -0.736698402 | NPAS1 | neuronal PAS domain protein 1 [Source:HGNC Symbol;Acc:HGNC:7894] |
| ENSG00000131019 | HGLibA_52967 | 44 | -0.736698402 | ULBP3 | UL16 binding protein 3 [Source:HGNC Symbol;Acc:HGNC:14895] |
| ENSG00000131037 | HGLibA_15378 | 44 | -0.736698402 | EPS8L1 | EPS8 like 1 [Source:HGNC Symbol;Acc:HGNC:21295] |
| ENSG00000131620 | HGLibA_02170 | 44 | -0.736698402 | ANO1 | anoctamin 1 [Source:HGNC Symbol;Acc:HGNC:21625] |
| ENSG00000131781 | HGLibA_17713 | 44 | -0.736698402 | FMO5 | flavin containing monooxygenase 5 [Source:HGNC Symbol;Acc:HGNC:3773] |
| ENSG00000132170 | HGLibA_37843 | 44 | -0.736698402 | PPARG | peroxisome proliferator activated receptor gamma [Source:HGNC Symbol;Acc:HGNC:9236] |
| ENSG00000132207 | HGLibA_45557 | 44 | -0.736698402 | SLX1A | structure-specific endonuclease subunit [Source:HGNC Symbol;Acc:HGNC:20922]" |
| ENSG00000132670 | HGLibA_39485 | 44 | -0.736698402 | PTPRA | receptor type A [Source:HGNC Symbol;Acc:HGNC:9664]" |
| ENSG00000133454 | HGLibA_30814 | 44 | -0.736698402 | MYO18B | myosin XVIIIB [Source:HGNC Symbol;Acc:HGNC:18150] |
| ENSG00000133606 | HGLibA_29347 | 44 | -0.736698402 | MKRN1 | makorin ring finger protein 1 [Source:HGNC Symbol;Acc:HGNC:7112] |
| ENSG00000133640 | HGLibA_27454 | 44 | -0.736698402 | LRRIQ1 | leucine rich repeats and IQ motif containing 1 [Source:HGNC Symbol;Acc:HGNC:25708] |
| ENSG00000134028 | HGLibA_00788 | 44 | -0.736698402 | ADAMDEC1 | ADAM like decysin 1 [Source:HGNC Symbol;Acc:HGNC:16299] |
| ENSG00000134508 | HGLibA_06842 | 44 | -0.736698402 | CABLES1 | Cdk5 and Abl enzyme substrate 1 [Source:HGNC Symbol;Acc:HGNC:25097] |
| ENSG00000134569 | HGLibA_27221 | 44 | -0.736698402 | LRP4 | LDL receptor related protein 4 [Source:HGNC Symbol;Acc:HGNC:6696] |
| ENSG00000135912 | HGLibA_52205 | 44 | -0.736698402 | TTLL4 | tubulin tyrosine ligase like 4 [Source:HGNC Symbol;Acc:HGNC:28976] |
| ENSG00000136206 | HGLibA_46578 | 44 | -0.736698402 | SPDYE1 | speedy/RINGO cell cycle regulator family member E1 [Source:HGNC Symbol;Acc:HGNC:16408] |
| ENSG00000136807 | HGLibA_08800 | 44 | -0.736698402 | CDK9 | cyclin dependent kinase 9 [Source:HGNC Symbol;Acc:HGNC:1780] |
| ENSG00000137054 | HGLibA_37562 | 44 | -0.736698402 | POLR1E | RNA polymerase I subunit E [Source:HGNC Symbol;Acc:HGNC:17631] |
| ENSG00000137309 | HGLibA_21684 | 44 | -0.736698402 | HMGA1 | high mobility group AT-hook 1 [Source:HGNC Symbol;Acc:HGNC:5010] |
| ENSG00000138069 | HGLibA_39830 | 44 | -0.736698402 | RAB1A | member RAS oncogene family [Source:HGNC Symbol;Acc:HGNC:9758]" |
| ENSG00000138246 | HGLibA_13574 | 44 | -0.736698402 | DNAJC13 | DnaJ heat shock protein family (Hsp40) member C13 [Source:HGNC Symbol;Acc:HGNC:30343] |
| ENSG00000138600 | HGLibA_46774 | 44 | -0.736698402 | SPPL2A | signal peptide peptidase like 2A [Source:HGNC Symbol;Acc:HGNC:30227] |
| ENSG00000138617 | HGLibA_35230 | 44 | -0.736698402 | PARP16 | poly(ADP-ribose) polymerase family member 16 [Source:HGNC Symbol;Acc:HGNC:26040] |
| ENSG00000138764 | HGLibA_08095 | 44 | -0.736698402 | CCNG2 | cyclin G2 [Source:HGNC Symbol;Acc:HGNC:1593] |
| ENSG00000139579 | HGLibA_31027 | 44 | -0.736698402 | NABP2 | nucleic acid binding protein 2 [Source:HGNC Symbol;Acc:HGNC:28412] |
| ENSG00000140416 | HGLibA_51140 | 44 | -0.736698402 | TPM1 | tropomyosin 1 [Source:HGNC Symbol;Acc:HGNC:12010] |
| ENSG00000141543 | HGLibA_14809 | 44 | -0.736698402 | EIF4A3 | eukaryotic translation initiation factor 4A3 [Source:HGNC Symbol;Acc:HGNC:18683] |
| ENSG00000143125 | HGLibA_38665 | 44 | -0.736698402 | PROK1 | prokineticin 1 [Source:HGNC Symbol;Acc:HGNC:18454] |
| ENSG00000143185 | HGLibA_54497 | 44 | -0.736698402 | XCL2 | X-C motif chemokine ligand 2 [Source:HGNC Symbol;Acc:HGNC:10646] |
| ENSG00000143190 | HGLibA_37757 | 44 | -0.736698402 | POU2F1 | POU class 2 homeobox 1 [Source:HGNC Symbol;Acc:HGNC:9212] |
| ENSG00000143228 | HGLibA_32998 | 44 | -0.736698402 | NUF2 | NDC80 kinetochore complex component NUF2 [Source:HGNC Symbol;Acc:HGNC:14621] |
| ENSG00000143390 | HGLibA_40975 | 44 | -0.736698402 | RFX5 | regulatory factor X5 [Source:HGNC Symbol;Acc:HGNC:9986] |
| ENSG00000143867 | HGLibA_34699 | 44 | -0.736698402 | OSR1 | odd-skipped related transciption factor 1 [Source:HGNC Symbol;Acc:HGNC:8111] |
| ENSG00000144048 | HGLibA_14133 | 44 | -0.736698402 | DUSP11 | dual specificity phosphatase 11 [Source:HGNC Symbol;Acc:HGNC:3066] |
| ENSG00000145194 | HGLibA_14384 | 44 | -0.736698402 | ECE2 | endothelin converting enzyme 2 [Source:HGNC Symbol;Acc:HGNC:13275] |
| ENSG00000145431 | HGLibA_35846 | 44 | -0.736698402 | PDGFC | platelet derived growth factor C [Source:HGNC Symbol;Acc:HGNC:8801] |
| ENSG00000146592 | HGLibA_11104 | 44 | -0.736698402 | CREB5 | cAMP responsive element binding protein 5 [Source:HGNC Symbol;Acc:HGNC:16844] |
| ENSG00000146918 | HGLibA_31273 | 44 | -0.736698402 | NCAPG2 | non-SMC condensin II complex subunit G2 [Source:HGNC Symbol;Acc:HGNC:21904] |
| ENSG00000147416 | HGLibA_03737 | 44 | -0.736698402 | ATP6V1B2 | ATPase H+ transporting V1 subunit B2 [Source:HGNC Symbol;Acc:HGNC:854] |
| ENSG00000148719 | HGLibA_13527 | 44 | -0.736698402 | DNAJB12 | DnaJ heat shock protein family (Hsp40) member B12 [Source:HGNC Symbol;Acc:HGNC:14891] |
| ENSG00000149809 | HGLibA_49716 | 44 | -0.736698402 | TM7SF2 | transmembrane 7 superfamily member 2 [Source:HGNC Symbol;Acc:HGNC:11863] |
| ENSG00000150401 | HGLibA_12569 | 44 | -0.736698402 | DCUN1D2 | defective in cullin neddylation 1 domain containing 2 [Source:HGNC Symbol;Acc:HGNC:20328] |
| ENSG00000151849 | HGLibA_09062 | 44 | -0.736698402 | CENPJ | centromere protein J [Source:HGNC Symbol;Acc:HGNC:17272] |
| ENSG00000152147 | HGLibA_18919 | 44 | -0.736698402 | GEMIN6 | gem nuclear organelle associated protein 6 [Source:HGNC Symbol;Acc:HGNC:20044] |
| ENSG00000153317 | HGLibA_03194 | 44 | -0.736698402 | ASAP1 | ankyrin repeat and PH domain 1 [Source:HGNC Symbol;Acc:HGNC:2720]" |
| ENSG00000154237 | HGLibA_27464 | 44 | -0.736698402 | LRRK1 | leucine rich repeat kinase 1 [Source:HGNC Symbol;Acc:HGNC:18608] |
| ENSG00000154589 | HGLibA_27692 | 44 | -0.736698402 | LY96 | lymphocyte antigen 96 [Source:HGNC Symbol;Acc:HGNC:17156] |
| ENSG00000155100 | HGLibA_34773 | 44 | -0.736698402 | OTUD6B | OTU domain containing 6B [Source:HGNC Symbol;Acc:HGNC:24281] |
| ENSG00000156299 | HGLibA_49438 | 44 | -0.736698402 | TIAM1 | T cell lymphoma invasion and metastasis 1 [Source:HGNC Symbol;Acc:HGNC:11805] |

| | | | | | |
|---|---|---|---|---|---|
| ENSG00000156384 | HGLibA_43734 | 44 | -0.736698402 | SFR1 | SWI5 dependent homologous recombination repair protein 1 [Source:HGNC Symbol;Acc:HGNC:29574] |
| ENSG00000156531 | HGLibA_36383 | 44 | -0.736698402 | PHF6 | PHD finger protein 6 [Source:HGNC Symbol;Acc:HGNC:18145] |
| ENSG00000156687 | HGLibA_53027 | 44 | -0.736698402 | UNC5D | unc-5 netrin receptor D [Source:HGNC Symbol;Acc:HGNC:18634] |
| ENSG00000158042 | HGLibA_29911 | 44 | -0.736698402 | MRPL17 | mitochondrial ribosomal protein L17 [Source:HGNC Symbol;Acc:HGNC:14053] |
| ENSG00000159459 | HGLibA_52747 | 44 | -0.736698402 | UBR1 | ubiquitin protein ligase E3 component n-recognin 1 [Source:HGNC Symbol;Acc:HGNC:16808] |
| ENSG00000160688 | HGLibA_17603 | 44 | -0.736698402 | FLAD1 | flavin adenine dinucleotide synthetase 1 [Source:HGNC Symbol;Acc:HGNC:24671] |
| ENSG00000161807 | HGLibA_34482 | 44 | -0.736698402 | OR7G1 | olfactory receptor family 7 subfamily G member 1 [Source:HGNC Symbol;Acc:HGNC:8465] |
| ENSG00000162004 | HGLibA_07871 | 44 | -0.736698402 | CCDC78 | coiled-coil domain containing 78 [Source:HGNC Symbol;Acc:HGNC:14153] |
| ENSG00000162620 | HGLibA_27459 | 44 | -0.736698402 | LRRIQ3 | leucine rich repeats and IQ motif containing 3 [Source:HGNC Symbol;Acc:HGNC:28318] |
| ENSG00000162944 | HGLibA_40953 | 44 | -0.736698402 | RFTN2 | raftlin family member 2 [Source:HGNC Symbol;Acc:HGNC:26402] |
| ENSG00000163110 | HGLibA_35920 | 44 | -0.736698402 | PDLIM5 | PDZ and LIM domain 5 [Source:HGNC Symbol;Acc:HGNC:17468] |
| ENSG00000163947 | HGLibA_02846 | 44 | -0.736698402 | ARHGEF3 | Rho guanine nucleotide exchange factor 3 [Source:HGNC Symbol;Acc:HGNC:683] |
| ENSG00000164010 | HGLibA_15500 | 44 | -0.736698402 | ERMAP | erythroblast membrane associated protein (Scianna blood group) [Source:HGNC Symbol;Acc:HGNC:15743] |
| ENSG00000164181 | HGLibA_14975 | 44 | -0.736698402 | ELOVL7 | ELOVL fatty acid elongase 7 [Source:HGNC Symbol;Acc:HGNC:26292] |
| ENSG00000164338 | HGLibA_53439 | 44 | -0.736698402 | UTP15 | small subunit processome component [Source:HGNC Symbol;Acc:HGNC:25758]" |
| ENSG00000164933 | HGLibA_44709 | 44 | -0.736698402 | SLC25A32 | solute carrier family 25 member 32 [Source:HGNC Symbol;Acc:HGNC:29683] |
| ENSG00000165502 | HGLibA_41961 | 44 | -0.736698402 | RPL36AL | ribosomal protein L36a like [Source:HGNC Symbol;Acc:HGNC:10346] |
| ENSG00000165548 | HGLibA_50480 | 44 | -0.736698402 | TMEM63C | transmembrane protein 63C [Source:HGNC Symbol;Acc:HGNC:23787] |
| ENSG00000166507 | HGLibA_31428 | 44 | -0.736698402 | NDST2 | N-deacetylase and N-sulfotransferase 2 [Source:HGNC Symbol;Acc:HGNC:7681] |
| ENSG00000167470 | HGLibA_29240 | 44 | -0.736698402 | MIDN | midnolin [Source:HGNC Symbol;Acc:HGNC:16298] |
| ENSG00000168438 | HGLibA_08552 | 44 | -0.736698402 | CDC40 | cell division cycle 40 [Source:HGNC Symbol;Acc:HGNC:17350] |
| ENSG00000169221 | HGLibA_48466 | 44 | -0.736698402 | TBC1D10B | TBC1 domain family member 10B [Source:HGNC Symbol;Acc:HGNC:24510] |
| ENSG00000169223 | HGLibA_26662 | 44 | -0.736698402 | LMAN2 | mannose binding 2 [Source:HGNC Symbol;Acc:HGNC:16986]" |
| ENSG00000169247 | HGLibA_43982 | 44 | -0.736698402 | SH3TC2 | SH3 domain and tetratricopeptide repeats 2 [Source:HGNC Symbol;Acc:HGNC:29427] |
| ENSG00000169599 | HGLibA_31844 | 44 | -0.736698402 | NFU1 | NFU1 iron-sulfur cluster scaffold [Source:HGNC Symbol;Acc:HGNC:16287] |
| ENSG00000169902 | HGLibA_51203 | 44 | -0.736698402 | TPST1 | tyrosylprotein sulfotransferase 1 [Source:HGNC Symbol;Acc:HGNC:12020] |
| ENSG00000170367 | HGLibA_11458 | 44 | -0.736698402 | CST5 | cystatin D [Source:HGNC Symbol;Acc:HGNC:2477] |
| ENSG00000171495 | HGLibA_29871 | 44 | -0.736698402 | MROH2B | maestro heat like repeat family member 2B [Source:HGNC Symbol;Acc:HGNC:26857] |
| ENSG00000171557 | HGLibA_17457 | 44 | -0.736698402 | FGG | fibrinogen gamma chain [Source:HGNC Symbol;Acc:HGNC:3694] |
| ENSG00000171606 | HGLibA_55710 | 44 | -0.736698402 | ZNF274 | zinc finger protein 274 [Source:HGNC Symbol;Acc:HGNC:13068] |
| ENSG00000171791 | HGLibA_04295 | 44 | -0.736698402 | BCL2 | apoptosis regulator [Source:HGNC Symbol;Acc:HGNC:990]" |
| ENSG00000171813 | HGLibA_39642 | 44 | -0.736698402 | PWWP2B | PWWP domain containing 2B [Source:HGNC Symbol;Acc:HGNC:25150] |
| ENSG00000172421 | HGLibA_14538 | 44 | -0.736698402 | EFCAB3 | EF-hand calcium binding domain 3 [Source:HGNC Symbol;Acc:HGNC:26379] |
| ENSG00000172469 | HGLibA_28090 | 44 | -0.736698402 | MANEA | mannosidase endo-alpha [Source:HGNC Symbol;Acc:HGNC:21072] |
| ENSG00000173085 | HGLibA_10748 | 44 | -0.736698402 | COQ2 | polyprenyltransferase [Source:HGNC Symbol;Acc:HGNC:25223]" |
| ENSG00000173451 | HGLibA_49296 | 44 | -0.736698402 | THAP2 | THAP domain containing 2 [Source:HGNC Symbol;Acc:HGNC:20854] |
| ENSG00000173548 | HGLibA_46093 | 44 | -0.736698402 | SNX33 | sorting nexin 33 [Source:HGNC Symbol;Acc:HGNC:28468] |
| ENSG00000173818 | HGLibA_15122 | 44 | -0.736698402 | ENDOV | endonuclease V [Source:HGNC Symbol;Acc:HGNC:26640] |
| ENSG00000173825 | HGLibA_49469 | 44 | -0.736698402 | TIGD3 | tigger transposable element derived 3 [Source:HGNC Symbol;Acc:HGNC:18334] |
| ENSG00000174937 | HGLibA_34338 | 44 | -0.736698402 | OR5M3 | olfactory receptor family 5 subfamily M member 3 [Source:HGNC Symbol;Acc:HGNC:14806] |
| ENSG00000174937 | HGLibA_34337 | 44 | -0.736698402 | OR5M3 | olfactory receptor family 5 subfamily M member 3 [Source:HGNC Symbol;Acc:HGNC:14806] |
| ENSG00000175356 | HGLibA_43073 | 44 | -0.736698402 | SCUBE2 | CUB domain and EGF like domain containing 2 [Source:HGNC Symbol;Acc:HGNC:30425]" |
| ENSG00000175664 | HGLibA_49078 | 44 | -0.736698402 | TEX26 | testis expressed 26 [Source:HGNC Symbol;Acc:HGNC:28622] |
| ENSG00000176018 | HGLibA_27769 | 44 | -0.736698402 | LYSMD3 | LysM domain containing 3 [Source:HGNC Symbol;Acc:HGNC:26969] |
| ENSG00000176281 | HGLibA_34034 | 44 | -0.736698402 | OR4K5 | olfactory receptor family 4 subfamily K member 5 [Source:HGNC Symbol;Acc:HGNC:14745] |
| ENSG00000177854 | HGLibA_50130 | 44 | -0.736698402 | TMEM187 | transmembrane protein 187 [Source:HGNC Symbol;Acc:HGNC:13705] |
| ENSG00000177989 | HGLibA_33286 | 44 | -0.736698402 | ODF3B | outer dense fiber of sperm tails 3B [Source:HGNC Symbol;Acc:HGNC:34388] |
| ENSG00000178031 | HGLibA_00849 | 44 | -0.736698402 | ADAMTSL1 | ADAMTS like 1 [Source:HGNC Symbol;Acc:HGNC:14632] |
| ENSG00000178075 | HGLibA_20199 | 44 | -0.736698402 | GRAMD1C | GRAM domain containing 1C [Source:HGNC Symbol;Acc:HGNC:25252] |
| ENSG00000178971 | HGLibA_11593 | 44 | -0.736698402 | CTC1 | CST telomere replication complex component 1 [Source:HGNC Symbol;Acc:HGNC:26169] |
| ENSG00000179604 | HGLibA_08574 | 44 | -0.736698402 | CDC42EP4 | CDC42 effector protein 4 [Source:HGNC Symbol;Acc:HGNC:17147] |
| ENSG00000180198 | HGLibA_40684 | 44 | -0.736698402 | RCC1 | regulator of chromosome condensation 1 [Source:HGNC Symbol;Acc:HGNC:1913] |
| ENSG00000180667 | HGLibA_54704 | 44 | -0.736698402 | YOD1 | YOD1 deubiquitinase [Source:HGNC Symbol;Acc:HGNC:25035] |
| ENSG00000181444 | HGLibA_56045 | 44 | -0.736698402 | ZNF467 | zinc finger protein 467 [Source:HGNC Symbol;Acc:HGNC:23154] |
| ENSG00000181467 | HGLibA_40240 | 44 | -0.736698402 | RAP2B | member of RAS oncogene family [Source:HGNC Symbol;Acc:HGNC:9862]" |
| ENSG00000182389 | HGLibA_06925 | 44 | -0.736698402 | CACNB4 | calcium voltage-gated channel auxiliary subunit beta 4 [Source:HGNC Symbol;Acc:HGNC:1404] |
| ENSG00000182405 | HGLibA_36224 | 44 | -0.736698402 | PGBD4 | piggyBac transposable element derived 4 [Source:HGNC Symbol;Acc:HGNC:19401] |
| ENSG00000183044 | HGLibA_00091 | 44 | -0.736698402 | ABAT | 4-aminobutyrate aminotransferase [Source:HGNC Symbol;Acc:HGNC:23] |
| ENSG00000183283 | HGLibA_12366 | 44 | -0.736698402 | DAZAP2 | DAZ associated protein 2 [Source:HGNC Symbol;Acc:HGNC:2684] |
| ENSG00000184005 | HGLibA_47270 | 44 | -0.736698402 | ST6GALNAC3 | 6-sialyltransferase 3 [Source:HGNC Symbol;Acc:HGNC:19343]" |
| ENSG00000184374 | HGLibA_10633 | 44 | -0.736698402 | COLEC10 | collectin subfamily member 10 [Source:HGNC Symbol;Acc:HGNC:2220] |
| ENSG00000184489 | HGLibA_39409 | 44 | -0.736698402 | PTP4A3 | member 3 [Source:HGNC Symbol;Acc:HGNC:9636]" |
| ENSG00000184507 | HGLibA_33102 | 44 | -0.736698402 | NUTM1 | NUT midline carcinoma family member 1 [Source:HGNC Symbol;Acc:HGNC:29919] |
| ENSG00000185033 | HGLibA_43342 | 44 | -0.736698402 | SEMA4B | semaphorin 4B [Source:HGNC Symbol;Acc:HGNC:10730] |
| ENSG00000185129 | HGLibA_39587 | 44 | -0.736698402 | PURA | purine rich element binding protein A [Source:HGNC Symbol;Acc:HGNC:9701] |
| ENSG00000185164 | HGLibA_32292 | 44 | -0.736698402 | NOMO2 | NODAL modulator 2 [Source:HGNC Symbol;Acc:HGNC:22652] |
| ENSG00000185215 | HGLibA_50708 | 44 | -0.736698402 | TNFAIP2 | TNF alpha induced protein 2 [Source:HGNC Symbol;Acc:HGNC:11895] |
| ENSG00000185359 | HGLibA_21246 | 44 | -0.736698402 | HGS | hepatocyte growth factor-regulated tyrosine kinase substrate [Source:HGNC Symbol;Acc:HGNC:4897] |
| ENSG00000185482 | HGLibA_47318 | 44 | -0.736698402 | STAC3 | SH3 and cysteine rich domain 3 [Source:HGNC Symbol;Acc:HGNC:28423] |
| ENSG00000185513 | HGLibA_25896 | 44 | -0.736698402 | L3MBTL1 | histone methyl-lysine binding protein [Source:HGNC Symbol;Acc:HGNC:15905]" |
| ENSG00000185873 | HGLibA_50616 | 44 | -0.736698402 | TMPRSS11B | transmembrane serine protease 11B [Source:HGNC Symbol;Acc:HGNC:25398] |
| ENSG00000186827 | HGLibA_50785 | 44 | -0.736698402 | TNFRSF4 | TNF receptor superfamily member 4 [Source:HGNC Symbol;Acc:HGNC:11918] |
| ENSG00000187024 | HGLibA_39549 | 44 | -0.736698402 | PTRH1 | peptidyl-tRNA hydrolase 1 homolog [Source:HGNC Symbol;Acc:HGNC:27039] |
| ENSG00000187191 | HGLibA_12359 | 44 | -0.736698402 | DAZ3 | deleted in azoospermia 3 [Source:HGNC Symbol;Acc:HGNC:15965] |
| ENSG00000188162 | HGLibA_34729 | 44 | -0.736698402 | OTOG | otogelin [Source:HGNC Symbol;Acc:HGNC:8516] |
| ENSG00000189134 | HGLibA_32004 | 44 | -0.736698402 | NKAPL | NFKB activating protein like [Source:HGNC Symbol;Acc:HGNC:21584] |
| ENSG00000189157 | HGLibA_16618 | 44 | -0.736698402 | FAM47E | family with sequence similarity 47 member E [Source:HGNC Symbol;Acc:HGNC:34343] |
| ENSG00000196381 | HGLibA_56738 | 44 | -0.736698402 | ZNF781 | zinc finger protein 781 [Source:HGNC Symbol;Acc:HGNC:26745] |
| ENSG00000196466 | HGLibA_56775 | 44 | -0.736698402 | ZNF799 | zinc finger protein 799 [Source:HGNC Symbol;Acc:HGNC:28071] |
| ENSG00000197142 | HGLibA_00562 | 44 | -0.736698402 | ACSL5 | acyl-CoA synthetase long chain family member 5 [Source:HGNC Symbol;Acc:HGNC:16526] |
| ENSG00000197302 | HGLibA_56627 | 44 | -0.736698402 | ZNF720 | zinc finger protein 720 [Source:HGNC Symbol;Acc:HGNC:26987] |
| ENSG00000197451 | HGLibA_21805 | 44 | -0.736698402 | HNRNPAB | heterogeneous nuclear ribonucleoprotein A/B [Source:HGNC Symbol;Acc:HGNC:5034] |
| ENSG00000197887 | HGLibA_33717 | 44 | -0.736698402 | OR1S2 | olfactory receptor family 1 subfamily S member 2 [Source:HGNC Symbol;Acc:HGNC:15141] |
| ENSG00000198573 | HGLibA_46390 | 44 | -0.736698402 | SPANXC | SPANX family member C [Source:HGNC Symbol;Acc:HGNC:14331] |
| ENSG00000198909 | HGLibA_28185 | 44 | -0.736698402 | MAP3K3 | mitogen-activated protein kinase kinase kinase 3 [Source:HGNC Symbol;Acc:HGNC:6855] |
| ENSG00000198951 | HGLibA_31062 | 44 | -0.736698402 | NAGA | alpha-N-acetylgalactosaminidase [Source:HGNC Symbol;Acc:HGNC:7631] |
| ENSG00000203837 | HGLibA_37364 | 44 | -0.736698402 | PNLIPRP3 | pancreatic lipase related protein 3 [Source:HGNC Symbol;Acc:HGNC:23492] |
| ENSG00000203907 | HGLibA_33400 | 44 | -0.736698402 | OOEP | oocyte expressed protein [Source:HGNC Symbol;Acc:HGNC:21382] |
| ENSG00000204366 | HGLibA_54819 | 44 | -0.736698402 | ZBTB12 | zinc finger and BTB domain containing 12 [Source:HGNC Symbol;Acc:HGNC:19066] |
| ENSG00000204498 | HGLibA_31834 | 44 | -0.736698402 | NFKBIL1 | NFKB inhibitor like 1 [Source:HGNC Symbol;Acc:HGNC:7800] |
| ENSG00000204619 | HGLibA_38018 | 44 | -0.736698402 | PPP1R11 | protein phosphatase 1 regulatory inhibitor subunit 11 [Source:HGNC Symbol;Acc:HGNC:9285] |
| ENSG00000204659 | HGLibA_07472 | 44 | -0.736698402 | CBY3 | chibby family member 3 [Source:HGNC Symbol;Acc:HGNC:33278] |
| ENSG00000204688 | HGLibA_33804 | 44 | -0.736698402 | OR2H1 | olfactory receptor family 2 subfamily H member 1 [Source:HGNC Symbol;Acc:HGNC:8252] |
| ENSG00000204869 | HGLibA_22849 | 44 | -0.736698402 | IGFL4 | IGF like family member 4 [Source:HGNC Symbol;Acc:HGNC:32931] |
| ENSG00000220205 | HGLibA_53492 | 44 | -0.736698402 | VAMP2 | vesicle associated membrane protein 2 [Source:HGNC Symbol;Acc:HGNC:12643] |
| ENSG00000230178 | HGLibA_34005 | 44 | -0.736698402 | OR4F3 | olfactory receptor family 4 subfamily F member 3 [Source:HGNC Symbol;Acc:HGNC:8300] |
| ENSG00000232810 | HGLibA_50704 | 44 | -0.736698402 | TNF | tumor necrosis factor [Source:HGNC Symbol;Acc:HGNC:11892] |
| ENSG00000241343 | HGLibA_41956 | 44 | -0.736698402 | RPL36A | ribosomal protein L36a [Source:HGNC Symbol;Acc:HGNC:10359] |
| ENSG00000241598 | HGLibA_25796 | 44 | -0.736698402 | KRTAP5-4 | keratin associated protein 5-4 [Source:HGNC Symbol;Acc:HGNC:23599] |
| ENSG00000243902 | HGLibA_14909 | 44 | -0.736698402 | ELFN2 | extracellular leucine rich repeat and fibronectin type III domain containing 2 [Source:NCBI gene;Acc:114794] |
| ENSG00000249811 | HGLibA_53248 | 44 | -0.736698402 | USP17L21 | ubiquitin specific peptidase 17-like family member 21 [Source:HGNC Symbol;Acc:HGNC:44449] |
| ENSG00000256980 | HGLibA_24561 | 44 | -0.736698402 | KHDC1L | KH domain containing 1 like [Source:HGNC Symbol;Acc:HGNC:37274] |
| ENSG00000263001 | HGLibA_20566 | 44 | -0.736698402 | GTF2I | general transcription factor IIi [Source:HGNC Symbol;Acc:HGNC:4659] |
| ENSG00000275793 | HGLibA_41262 | 44 | -0.736698402 | RIMBP3 | RIMS binding protein 3 [Source:HGNC Symbol;Acc:HGNC:29344] |
| ENSG00000278129 | HGLibA_56779 | 44 | -0.736698402 | ZNF8 | zinc finger protein 8 [Source:HGNC Symbol;Acc:HGNC:13154] |
| FAM196A | HGLibA_16380 | 44 | -0.736698402 | FAM196A | - |
| GAGE2C | HGLibA_18473 | 44 | -0.736698402 | GAGE2C | - |
| HIST1H2BF | HGLibA_21425 | 44 | -0.736698402 | HIST1H2BF | - |
| hsa-mir-1208 | HGLibA_57286 | 44 | -0.736698402 | hsa-mir-1208 | - |
| hsa-mir-1258 | HGLibA_57441 | 44 | -0.736698402 | hsa-mir-1258 | - |
| hsa-mir-151b | HGLibA_57900 | 44 | -0.736698402 | hsa-mir-151b | - |
| hsa-mir-24-1 | HGLibA_58341 | 44 | -0.736698402 | hsa-mir-24-1 | - |
| hsa-mir-3180-2 | HGLibA_58807 | 44 | -0.736698402 | hsa-mir-3180-2 | - |
| hsa-mir-3617 | HGLibA_59099 | 44 | -0.736698402 | hsa-mir-3617 | - |
| hsa-mir-3670-2 | HGLibA_59238 | 44 | -0.736698402 | hsa-mir-3670-2 | - |
| hsa-mir-3690-1 | HGLibA_59344 | 44 | -0.736698402 | hsa-mir-3690-1 | - |
| hsa-mir-4317 | HGLibA_59987 | 44 | -0.736698402 | hsa-mir-4317 | - |
| hsa-mir-4444-1 | HGLibA_60179 | 44 | -0.736698402 | hsa-mir-4444-1 | - |
| hsa-mir-4444-2 | HGLibA_60183 | 44 | -0.736698402 | hsa-mir-4444-2 | - |
| hsa-mir-4486 | HGLibA_60361 | 44 | -0.736698402 | hsa-mir-4486 | - |
| hsa-mir-4520a | HGLibA_60534 | 44 | -0.736698402 | hsa-mir-4520a | - |
| hsa-mir-4523 | HGLibA_60548 | 44 | -0.736698402 | hsa-mir-4523 | - |
| hsa-mir-4659b | HGLibA_60747 | 44 | -0.736698402 | hsa-mir-4659b | - |
| hsa-mir-4666a | HGLibA_60779 | 44 | -0.736698402 | hsa-mir-4666a | - |
| hsa-mir-4756 | HGLibA_61121 | 44 | -0.736698402 | hsa-mir-4756 | - |
| hsa-mir-548c | HGLibA_61958 | 44 | -0.736698402 | hsa-mir-548c | - |
| hsa-mir-561 | HGLibA_62208 | 44 | -0.736698402 | hsa-mir-561 | - |
| hsa-mir-5681a | HGLibA_62241 | 44 | -0.736698402 | hsa-mir-5681a | - |
| hsa-mir-6073 | HGLibA_62543 | 44 | -0.736698402 | hsa-mir-6073 | - |
| hsa-mir-6082 | HGLibA_62588 | 44 | -0.736698402 | hsa-mir-6082 | - |
| hsa-mir-6088 | HGLibA_62611 | 44 | -0.736698402 | hsa-mir-6088 | - |
| hsa-mir-6134 | HGLibA_62688 | 44 | -0.736698402 | hsa-mir-6134 | - |
| hsa-mir-6515 | HGLibA_62935 | 44 | -0.736698402 | hsa-mir-6515 | - |
| hsa-mir-670 | HGLibA_63009 | 44 | -0.736698402 | hsa-mir-670 | - |
| hsa-mir-6721 | HGLibA_63047 | 44 | -0.736698402 | hsa-mir-6721 | - |

| | | | | | |
|---|---|---|---|---|---|
| hsa-mir-6796 | HGLibA_63363 | 44 | -0.736698402 | hsa-mir-6796 | - |
| hsa-mir-6873 | HGLibA_63675 | 44 | -0.736698402 | hsa-mir-6873 | - |
| hsa-mir-6892 | HGLibA_63751 | 44 | -0.736698402 | hsa-mir-6892 | - |
| hsa-mir-940 | HGLibA_64336 | 44 | -0.736698402 | hsa-mir-940 | - |
| KIAA1107 | HGLibA_24690 | 44 | -0.736698402 | KIAA1107 | - |
| KIAA1468 | HGLibA_24770 | 44 | -0.736698402 | KIAA1468 | - |
| LOC100287036 | HGLibA_26817 | 44 | -0.736698402 | LOC100287036 | - |
| LPPR5 | HGLibA_27124 | 44 | -0.736698402 | LPPR5 | - |
| NOV | HGLibA_32361 | 44 | -0.736698402 | NOV | - |
| PCNXL2 | HGLibA_35645 | 44 | -0.736698402 | PCNXL2 | - |
| 11-Sep | HGLibA_43431 | 44 | -0.736698402 | 11-Sep | - |
| ASMTL_X | HGLibA_03327 | 43 | -0.745743745 | ASMTL_X | - |
| BAGE5 | HGLibA_04104 | 43 | -0.745743745 | BAGE5 | - |
| C10orf111 | HGLibA_05031 | 43 | -0.745743745 | C10orf111 | - |
| C19orf80 | HGLibA_05712 | 43 | -0.745743745 | C19orf80 | - |
| C20orf24 | HGLibA_06018 | 43 | -0.745743745 | C20orf24 | - |
| COL4A3BP | HGLibA_10576 | 43 | -0.745743745 | COL4A3BP | - |
| ENSG00000002919 | HGLibA_46029 | 43 | -0.745743745 | SNX11 | sorting nexin 11 [Source:HGNC Symbol;Acc:HGNC:14975] |
| ENSG00000003249 | HGLibA_12391 | 43 | -0.745743745 | DBNDD1 | dysbindin domain containing 1 [Source:HGNC Symbol;Acc:HGNC:28455] |
| ENSG00000004487 | HGLibA_24487 | 43 | -0.745743745 | KDM1A | lysine demethylase 1A [Source:HGNC Symbol;Acc:HGNC:29079] |
| ENSG00000022840 | HGLibA_41454 | 43 | -0.745743745 | RNF10 | ring finger protein 10 [Source:HGNC Symbol;Acc:HGNC:10055] |
| ENSG00000023909 | HGLibA_18797 | 43 | -0.745743745 | GCLM | glutamate-cysteine ligase modifier subunit [Source:HGNC Symbol;Acc:HGNC:4312] |
| ENSG00000039068 | HGLibA_08628 | 43 | -0.745743745 | CDH1 | cadherin 1 [Source:HGNC Symbol;Acc:HGNC:1748] |
| ENSG00000049759 | HGLibA_31606 | 43 | -0.745743745 | NEDD4L | E3 ubiquitin protein ligase [Source:HGNC Symbol;Acc:HGNC:7728]" |
| ENSG00000054611 | HGLibA_48503 | 43 | -0.745743745 | TBC1D22A | TBC1 domain family member 22A [Source:HGNC Symbol;Acc:HGNC:1309] |
| ENSG00000055118 | HGLibA_24213 | 43 | -0.745743745 | KCNH2 | potassium voltage-gated channel subfamily H member 2 [Source:HGNC Symbol;Acc:HGNC:6251] |
| ENSG00000061455 | HGLibA_38394 | 43 | -0.745743745 | PRDM6 | PR/SET domain 6 [Source:HGNC Symbol;Acc:HGNC:9350] |
| ENSG00000064655 | HGLibA_15821 | 43 | -0.745743745 | EYA2 | EYA transcriptional coactivator and phosphatase 2 [Source:HGNC Symbol;Acc:HGNC:3520] |
| ENSG00000065534 | HGLibA_30783 | 43 | -0.745743745 | MYLK | myosin light chain kinase [Source:HGNC Symbol;Acc:HGNC:7590] |
| ENSG00000065911 | HGLibA_30383 | 43 | -0.745743745 | MTHFD2 | methenyltetrahydrofolate cyclohydrolase [Source:HGNC Symbol;Acc:HGNC:7434]" |
| ENSG00000067445 | HGLibA_51717 | 43 | -0.745743745 | TRO | trophinin [Source:HGNC Symbol;Acc:HGNC:12326] |
| ENSG00000069206 | HGLibA_00778 | 43 | -0.745743745 | ADAM7 | ADAM metallopeptidase domain 7 [Source:HGNC Symbol;Acc:HGNC:214] |
| ENSG00000072134 | HGLibA_15345 | 43 | -0.745743745 | EPN2 | epsin 2 [Source:HGNC Symbol;Acc:HGNC:18639] |
| ENSG00000072364 | HGLibA_01114 | 43 | -0.745743745 | AFF4 | AF4/FMR2 family member 4 [Source:HGNC Symbol;Acc:HGNC:17869] |
| ENSG00000075131 | HGLibA_49560 | 43 | -0.745743745 | TIPIN | TIMELESS interacting protein [Source:HGNC Symbol;Acc:HGNC:30750] |
| ENSG00000080007 | HGLibA_12716 | 43 | -0.745743745 | DDX43 | DEAD-box helicase 43 [Source:HGNC Symbol;Acc:HGNC:18677] |
| ENSG00000083307 | HGLibA_20251 | 43 | -0.745743745 | GRHL2 | grainyhead like transcription factor 2 [Source:HGNC Symbol;Acc:HGNC:2799] |
| ENSG00000089127 | HGLibA_33197 | 43 | -0.745743745 | OAS1 | 2'-5'-oligoadenylate synthetase 1 [Source:HGNC Symbol;Acc:HGNC:8086] |
| ENSG00000090674 | HGLibA_28638 | 43 | -0.745743745 | MCOLN1 | mucolipin 1 [Source:HGNC Symbol;Acc:HGNC:13356] |
| ENSG00000092978 | HGLibA_19738 | 43 | -0.745743745 | GPATCH2 | G-patch domain containing 2 [Source:HGNC Symbol;Acc:HGNC:25499] |
| ENSG00000096872 | HGLibA_22771 | 43 | -0.745743745 | IFT74 | intraflagellar transport 74 [Source:HGNC Symbol;Acc:HGNC:21424] |
| ENSG00000099204 | HGLibA_00308 | 43 | -0.745743745 | ABLIM1 | actin binding LIM protein 1 [Source:HGNC Symbol;Acc:HGNC:78] |
| ENSG00000099377 | HGLibA_22210 | 43 | -0.745743745 | HSD3B7 | 3 beta- and steroid delta-isomerase 7 [Source:HGNC Symbol;Acc:HGNC:18324]" |
| ENSG00000100191 | HGLibA_45271 | 43 | -0.745743745 | SLC5A4 | solute carrier family 5 member 4 [Source:HGNC Symbol;Acc:HGNC:11039] |
| ENSG00000100485 | HGLibA_46205 | 43 | -0.745743745 | SOS2 | SOS Ras/Rho guanine nucleotide exchange factor 2 [Source:HGNC Symbol;Acc:HGNC:11188] |
| ENSG00000100503 | HGLibA_31936 | 43 | -0.745743745 | NIN | ninein [Source:HGNC Symbol;Acc:HGNC:14906] |
| ENSG00000100697 | HGLibA_13226 | 43 | -0.745743745 | DICER1 | ribonuclease III [Source:HGNC Symbol;Acc:HGNC:17098]" |
| ENSG00000100997 | HGLibA_00239 | 43 | -0.745743745 | ABHD12 | abhydrolase domain containing 12 [Source:HGNC Symbol;Acc:HGNC:15868] |
| ENSG00000101019 | HGLibA_53098 | 43 | -0.745743745 | UQCC1 | ubiquinol-cytochrome c reductase complex assembly factor 1 [Source:HGNC Symbol;Acc:HGNC:15891] |
| ENSG00000101057 | HGLibA_30645 | 43 | -0.745743745 | MYBL2 | MYB proto-oncogene like 2 [Source:HGNC Symbol;Acc:HGNC:7548] |
| ENSG00000102934 | HGLibA_37203 | 43 | -0.745743745 | PLLP | plasmolipin [Source:HGNC Symbol;Acc:HGNC:18553] |
| ENSG00000103042 | HGLibA_45079 | 43 | -0.745743745 | SLC38A7 | solute carrier family 38 member 7 [Source:HGNC Symbol;Acc:HGNC:25582] |
| ENSG00000104888 | HGLibA_44476 | 43 | -0.745743745 | SLC17A7 | solute carrier family 17 member 7 [Source:HGNC Symbol;Acc:HGNC:16704] |
| ENSG00000105289 | HGLibA_49576 | 43 | -0.745743745 | TJP3 | tight junction protein 3 [Source:HGNC Symbol;Acc:HGNC:11829] |
| ENSG00000105679 | HGLibA_18616 | 43 | -0.745743745 | GAPDHS | spermatogenic [Source:HGNC Symbol;Acc:HGNC:24864]" |
| ENSG00000107537 | HGLibA_36468 | 43 | -0.745743745 | PHYH | phytanoyl-CoA 2-hydroxylase [Source:HGNC Symbol;Acc:HGNC:8940] |
| ENSG00000108039 | HGLibA_54552 | 43 | -0.745743745 | XPNPEP1 | X-prolyl aminopeptidase 1 [Source:HGNC Symbol;Acc:HGNC:12822] |
| ENSG00000108798 | HGLibA_00297 | 43 | -0.745743745 | ABI3 | ABI family member 3 [Source:HGNC Symbol;Acc:HGNC:29859] |
| ENSG00000108960 | HGLibA_29463 | 43 | -0.745743745 | MMD | monocyte to macrophage differentiation associated [Source:HGNC Symbol;Acc:HGNC:7153] |
| ENSG00000109881 | HGLibA_07759 | 43 | -0.745743745 | CCDC34 | coiled-coil domain containing 34 [Source:HGNC Symbol;Acc:HGNC:25079] |
| ENSG00000111707 | HGLibA_47712 | 43 | -0.745743745 | SUDS3 | SIN3A corepressor complex component [Source:HGNC Symbol;Acc:HGNC:29545]" |
| ENSG00000111796 | HGLibA_25281 | 43 | -0.745743745 | KLRB1 | killer cell lectin like receptor B1 [Source:HGNC Symbol;Acc:HGNC:6373] |
| ENSG00000112306 | HGLibA_42068 | 43 | -0.745743745 | RPS12 | ribosomal protein S12 [Source:HGNC Symbol;Acc:HGNC:10385] |
| ENSG00000113583 | HGLibA_06358 | 43 | -0.745743745 | C5orf15 | chromosome 5 open reading frame 15 [Source:HGNC Symbol;Acc:HGNC:20656] |
| ENSG00000114013 | HGLibA_08469 | 43 | -0.745743745 | CD86 | CD86 molecule [Source:HGNC Symbol;Acc:HGNC:1705] |
| ENSG00000114805 | HGLibA_37009 | 43 | -0.745743745 | PLCH1 | phospholipase C eta 1 [Source:HGNC Symbol;Acc:HGNC:29185] |
| ENSG00000114902 | HGLibA_46557 | 43 | -0.745743745 | SPCS1 | signal peptidase complex subunit 1 [Source:HGNC Symbol;Acc:HGNC:23401] |
| ENSG00000115207 | HGLibA_20584 | 43 | -0.745743745 | GTF3C2 | general transcription factor IIIC subunit 2 [Source:HGNC Symbol;Acc:HGNC:4665] |
| ENSG00000115598 | HGLibA_23063 | 43 | -0.745743745 | IL1RL2 | interleukin 1 receptor like 2 [Source:HGNC Symbol;Acc:HGNC:5999] |
| ENSG00000116030 | HGLibA_47775 | 43 | -0.745743745 | SUMO1 | small ubiquitin-like modifier 1 [Source:HGNC Symbol;Acc:HGNC:12502] |
| ENSG00000116128 | HGLibA_04346 | 43 | -0.745743745 | BCL9 | B cell CLL/lymphoma 9 [Source:HGNC Symbol;Acc:HGNC:1008] |
| ENSG00000117215 | HGLibA_36889 | 43 | -0.745743745 | PLA2G2D | phospholipase A2 group IID [Source:HGNC Symbol;Acc:HGNC:9033] |
| ENSG00000119650 | HGLibA_22759 | 43 | -0.745743745 | IFT43 | intraflagellar transport 43 [Source:HGNC Symbol;Acc:HGNC:29669] |
| ENSG00000119977 | HGLibA_48879 | 43 | -0.745743745 | TCTN3 | tectonic family member 3 [Source:HGNC Symbol;Acc:HGNC:24519] |
| ENSG00000121940 | HGLibA_09814 | 43 | -0.745743745 | CLCC1 | chloride channel CLIC like 1 [Source:HGNC Symbol;Acc:HGNC:29675] |
| ENSG00000122481 | HGLibA_42487 | 43 | -0.745743745 | RWDD3 | RWD domain containing 3 [Source:HGNC Symbol;Acc:HGNC:21393] |
| ENSG00000122824 | HGLibA_32934 | 43 | -0.745743745 | NUDT10 | nudix hydrolase 10 [Source:HGNC Symbol;Acc:HGNC:17621] |
| ENSG00000123154 | HGLibA_54192 | 43 | -0.745743745 | WDR83 | WD repeat domain 83 [Source:HGNC Symbol;Acc:HGNC:32672] |
| ENSG00000124226 | HGLibA_41477 | 43 | -0.745743745 | RNF114 | ring finger protein 114 [Source:HGNC Symbol;Acc:HGNC:13094] |
| ENSG00000124587 | HGLibA_36124 | 43 | -0.745743745 | PEX6 | peroxisomal biogenesis factor 6 [Source:HGNC Symbol;Acc:HGNC:8859] |
| ENSG00000127184 | HGLibA_10872 | 43 | -0.745743745 | COX7C | cytochrome c oxidase subunit 7C [Source:HGNC Symbol;Acc:HGNC:2292] |
| ENSG00000128394 | HGLibA_02487 | 43 | -0.745743745 | APOBEC3F | apolipoprotein B mRNA editing enzyme catalytic subunit 3F [Source:HGNC Symbol;Acc:HGNC:17356] |
| ENSG00000128641 | HGLibA_30822 | 43 | -0.745743745 | MYO1B | myosin IB [Source:HGNC Symbol;Acc:HGNC:7596] |
| ENSG00000129455 | HGLibA_25270 | 43 | -0.745743745 | KLK8 | kallikrein related peptidase 8 [Source:HGNC Symbol;Acc:HGNC:6369] |
| ENSG00000129465 | HGLibA_41322 | 43 | -0.745743745 | RIPK3 | receptor interacting serine/threonine kinase 3 [Source:HGNC Symbol;Acc:HGNC:10021] |
| ENSG00000130227 | HGLibA_54575 | 43 | -0.745743745 | XPO7 | exportin 7 [Source:HGNC Symbol;Acc:HGNC:14108] |
| ENSG00000130950 | HGLibA_33109 | 43 | -0.745743745 | NUTM2F | NUT family member 2F [Source:HGNC Symbol;Acc:HGNC:23450] |
| ENSG00000131203 | HGLibA_22562 | 43 | -0.745743745 | IDO1 | 3-dioxygenase 1 [Source:HGNC Symbol;Acc:HGNC:6059]" |
| ENSG00000132932 | HGLibA_03779 | 43 | -0.745743745 | ATP8A2 | ATPase phospholipid transporting 8A2 [Source:HGNC Symbol;Acc:HGNC:13533] |
| ENSG00000133962 | HGLibA_07368 | 43 | -0.745743745 | CATSPERB | cation channel sperm associated auxiliary subunit beta [Source:HGNC Symbol;Acc:HGNC:20500] |
| ENSG00000134152 | HGLibA_24083 | 43 | -0.745743745 | KATNBL1 | katanin regulatory subunit B1 like 1 [Source:HGNC Symbol;Acc:HGNC:26199] |
| ENSG00000134247 | HGLibA_39344 | 43 | -0.745743745 | PTGFRN | prostaglandin F2 receptor inhibitor [Source:HGNC Symbol;Acc:HGNC:9601] |
| ENSG00000135829 | HGLibA_13210 | 43 | -0.745743745 | DHX9 | DExH-box helicase 9 [Source:HGNC Symbol;Acc:HGNC:2750] |
| ENSG00000136828 | HGLibA_40169 | 43 | -0.745743745 | RALGPS1 | Ral GEF with PH domain and SH3 binding motif 1 [Source:HGNC Symbol;Acc:HGNC:16851] |
| ENSG00000137198 | HGLibA_19427 | 43 | -0.745743745 | GMPR | guanosine monophosphate reductase [Source:HGNC Symbol;Acc:HGNC:4376] |
| ENSG00000138472 | HGLibA_20640 | 43 | -0.745743745 | GUCA1C | guanylate cyclase activator 1C [Source:HGNC Symbol;Acc:HGNC:4680] |
| ENSG00000138663 | HGLibA_10719 | 43 | -0.745743745 | COPS4 | COP9 signalosome subunit 4 [Source:HGNC Symbol;Acc:HGNC:16702] |
| ENSG00000138813 | HGLibA_06295 | 43 | -0.745743745 | C4orf17 | chromosome 4 open reading frame 17 [Source:HGNC Symbol;Acc:HGNC:25274] |
| ENSG00000139144 | HGLibA_36628 | 43 | -0.745743745 | PIK3C2G | phosphatidylinositol-4-phosphate 3-kinase catalytic subunit type 2 gamma [Source:HGNC Symbol;Acc:HGNC:8973] |
| ENSG00000140280 | HGLibA_27767 | 43 | -0.745743745 | LYSMD2 | LysM domain containing 2 [Source:HGNC Symbol;Acc:HGNC:28571] |
| ENSG00000140416 | HGLibA_51142 | 43 | -0.745743745 | TPM1 | tropomyosin 1 [Source:HGNC Symbol;Acc:HGNC:12010] |
| ENSG00000140694 | HGLibA_35207 | 43 | -0.745743745 | PARN | poly(A)-specific ribonuclease [Source:HGNC Symbol;Acc:HGNC:8609] |
| ENSG00000140986 | HGLibA_41978 | 43 | -0.745743745 | RPL3L | ribosomal protein L3 like [Source:HGNC Symbol;Acc:HGNC:10351] |
| ENSG00000141258 | HGLibA_43862 | 43 | -0.745743745 | SGSM2 | small G protein signaling modulator 2 [Source:HGNC Symbol;Acc:HGNC:29026] |
| ENSG00000141446 | HGLibA_15556 | 43 | -0.745743745 | ESCO1 | establishment of sister chromatid cohesion N-acetyltransferase 1 [Source:HGNC Symbol;Acc:HGNC:24645] |
| ENSG00000144908 | HGLibA_01552 | 43 | -0.745743745 | ALDH1L1 | aldehyde dehydrogenase 1 family member L1 [Source:HGNC Symbol;Acc:HGNC:3978] |
| ENSG00000144962 | HGLibA_46433 | 43 | -0.745743745 | SPATA16 | spermatogenesis associated 16 [Source:HGNC Symbol;Acc:HGNC:29935] |
| ENSG00000145536 | HGLibA_00808 | 43 | -0.745743745 | ADAMTS16 | ADAM metallopeptidase with thrombospondin type 1 motif 16 [Source:HGNC Symbol;Acc:HGNC:17108] |
| ENSG00000145934 | HGLibA_48997 | 43 | -0.745743745 | TENM2 | teneurin transmembrane protein 2 [Source:HGNC Symbol;Acc:HGNC:29943] |
| ENSG00000148841 | HGLibA_23863 | 43 | -0.745743745 | ITPRIP | 5-trisphosphate receptor interacting protein [Source:HGNC Symbol;Acc:HGNC:29370]" |
| ENSG00000149970 | HGLibA_10300 | 43 | -0.745743745 | CNKSR2 | connector enhancer of kinase suppressor of Ras 2 [Source:HGNC Symbol;Acc:HGNC:19701] |
| ENSG00000150316 | HGLibA_11811 | 43 | -0.745743745 | CWC15 | CWC15 spliceosome associated protein homolog [Source:HGNC Symbol;Acc:HGNC:26939] |
| ENSG00000150676 | HGLibA_07885 | 43 | -0.745743745 | CCDC83 | coiled-coil domain containing 83 [Source:HGNC Symbol;Acc:HGNC:28535] |
| ENSG00000150787 | HGLibA_39559 | 43 | -0.745743745 | PTS | 6-pyruvoyltetrahydropterin synthase [Source:HGNC Symbol;Acc:HGNC:9689] |
| ENSG00000151176 | HGLibA_36976 | 43 | -0.745743745 | PLBD2 | phospholipase B domain containing 2 [Source:HGNC Symbol;Acc:HGNC:27283] |
| ENSG00000152430 | HGLibA_04691 | 43 | -0.745743745 | BOLL | RNA binding protein [Source:HGNC Symbol;Acc:HGNC:14273]" |
| ENSG00000152465 | HGLibA_32209 | 43 | -0.745743745 | NMT2 | N-myristoyltransferase 2 [Source:HGNC Symbol;Acc:HGNC:7858] |
| ENSG00000152669 | HGLibA_08124 | 43 | -0.745743745 | CCNO | cyclin O [Source:HGNC Symbol;Acc:HGNC:18576] |
| ENSG00000153802 | HGLibA_50618 | 43 | -0.745743745 | TMPRSS11D | transmembrane serine protease 11D [Source:HGNC Symbol;Acc:HGNC:24059] |
| ENSG00000154001 | HGLibA_38182 | 43 | -0.745743745 | PPP2R5E | protein phosphatase 2 regulatory subunit B'epsilon [Source:HGNC Symbol;Acc:HGNC:9313] |
| ENSG00000155269 | HGLibA_20087 | 43 | -0.745743745 | GPR78 | G protein-coupled receptor 78 [Source:HGNC Symbol;Acc:HGNC:4528] |
| ENSG00000155622 | HGLibA_54482 | 43 | -0.745743745 | XAGE2 | X antigen family member 2 [Source:HGNC Symbol;Acc:HGNC:4112] |
| ENSG00000155906 | HGLibA_41378 | 43 | -0.745743745 | RMND1 | required for meiotic nuclear division 1 homolog [Source:HGNC Symbol;Acc:HGNC:21176] |
| ENSG00000156304 | HGLibA_42826 | 43 | -0.745743745 | SCAF4 | SR-related CTD associated factor 4 [Source:HGNC Symbol;Acc:HGNC:19304] |
| ENSG00000156475 | HGLibA_38150 | 43 | -0.745743745 | PPP2R2B | protein phosphatase 2 regulatory subunit Bbeta [Source:HGNC Symbol;Acc:HGNC:9305] |
| ENSG00000156508 | HGLibA_14489 | 43 | -0.745743745 | EEF1A1 | eukaryotic translation elongation factor 1 alpha 1 [Source:HGNC Symbol;Acc:HGNC:3189] |
| ENSG00000156689 | HGLibA_19376 | 43 | -0.745743745 | GLYATL2 | glycine-N-acyltransferase like 2 [Source:HGNC Symbol;Acc:HGNC:24178] |
| ENSG00000156931 | HGLibA_53818 | 43 | -0.745743745 | VPS8 | CORVET complex subunit [Source:HGNC Symbol;Acc:HGNC:29122]" |
| ENSG00000157404 | HGLibA_25016 | 43 | -0.745743745 | KIT | KIT proto-oncogene receptor tyrosine kinase [Source:HGNC Symbol;Acc:HGNC:6342] |
| ENSG00000157680 | HGLibA_13076 | 43 | -0.745743745 | DGKI | diacylglycerol kinase iota [Source:HGNC Symbol;Acc:HGNC:2855] |
| ENSG00000157978 | HGLibA_26253 | 43 | -0.745743745 | LDLRAP1 | low density lipoprotein receptor adaptor protein 1 [Source:HGNC Symbol;Acc:HGNC:18640] |
| ENSG00000159147 | HGLibA_13796 | 43 | -0.745743745 | DONSON | downstream neighbor of SON [Source:HGNC Symbol;Acc:HGNC:2993] |
| ENSG00000159352 | HGLibA_39171 | 43 | -0.745743745 | PSMD4 | non-ATPase 4 [Source:HGNC Symbol;Acc:HGNC:9561]" |
| ENSG00000159423 | HGLibA_01574 | 43 | -0.745743745 | ALDH4A1 | aldehyde dehydrogenase 4 family member A1 [Source:HGNC Symbol;Acc:HGNC:406] |
| ENSG00000160799 | HGLibA_07563 | 43 | -0.745743745 | CCDC12 | coiled-coil domain containing 12 [Source:HGNC Symbol;Acc:HGNC:28332] |

| | | | | | |
|---|---|---|---|---|---|
| ENSG00000161551 | HGLibA_56296 | 43 | -0.745743745 | ZNF577 | zinc finger protein 577 [Source:HGNC Symbol;Acc:HGNC:28673] |
| ENSG00000161681 | HGLibA_43988 | 43 | -0.745743745 | SHANK1 | SH3 and multiple ankyrin repeat domains 1 [Source:HGNC Symbol;Acc:HGNC:15474] |
| ENSG00000162692 | HGLibA_53567 | 43 | -0.745743745 | VCAM1 | vascular cell adhesion molecule 1 [Source:HGNC Symbol;Acc:HGNC:12663] |
| ENSG00000162944 | HGLibA_40954 | 43 | -0.745743745 | RFTN2 | raftlin family member 2 [Source:HGNC Symbol;Acc:HGNC:26402] |
| ENSG00000162949 | HGLibA_07155 | 43 | -0.745743745 | CAPN13 | calpain 13 [Source:HGNC Symbol;Acc:HGNC:16663] |
| ENSG00000163060 | HGLibA_48981 | 43 | -0.745743745 | TEKT4 | tektin 4 [Source:HGNC Symbol;Acc:HGNC:31012] |
| ENSG00000163590 | HGLibA_37991 | 43 | -0.745743745 | PPM1L | Mg2+/Mn2+ dependent 1L [Source:HGNC Symbol;Acc:HGNC:16381]" |
| ENSG00000163624 | HGLibA_08881 | 43 | -0.745743745 | CDS1 | CDP-diacylglycerol synthase 1 [Source:HGNC Symbol;Acc:HGNC:1800] |
| ENSG00000164051 | HGLibA_07796 | 43 | -0.745743745 | CCDC51 | coiled-coil domain containing 51 [Source:HGNC Symbol;Acc:HGNC:25714] |
| ENSG00000164307 | HGLibA_15403 | 43 | -0.745743745 | ERAP1 | endoplasmic reticulum aminopeptidase 1 [Source:HGNC Symbol;Acc:HGNC:18173] |
| ENSG00000165046 | HGLibA_26335 | 43 | -0.745743745 | LETM2 | leucine zipper and EF-hand containing transmembrane protein 2 [Source:HGNC Symbol;Acc:HGNC:14648] |
| ENSG00000165462 | HGLibA_36449 | 43 | -0.745743745 | PHOX2A | paired like homeobox 2A [Source:HGNC Symbol;Acc:HGNC:691] |
| ENSG00000165623 | HGLibA_52819 | 43 | -0.745743745 | UCMA | upper zone of growth plate and cartilage matrix associated [Source:HGNC Symbol;Acc:HGNC:25205] |
| ENSG00000165953 | HGLibA_43515 | 43 | -0.745743745 | SERPINA12 | serpin family A member 12 [Source:HGNC Symbol;Acc:HGNC:18359] |
| ENSG00000166321 | HGLibA_32943 | 43 | -0.745743745 | NUDT13 | nudix hydrolase 13 [Source:HGNC Symbol;Acc:HGNC:18827] |
| ENSG00000166589 | HGLibA_08646 | 43 | -0.745743745 | CDH16 | cadherin 16 [Source:HGNC Symbol;Acc:HGNC:1755] |
| ENSG00000166707 | HGLibA_55024 | 43 | -0.745743745 | ZCCHC18 | zinc finger CCHC-type containing 18 [Source:HGNC Symbol;Acc:HGNC:32459] |
| ENSG00000167118 | HGLibA_53150 | 43 | -0.745743745 | URM1 | ubiquitin related modifier 1 [Source:HGNC Symbol;Acc:HGNC:28378] |
| ENSG00000167525 | HGLibA_38652 | 43 | -0.745743745 | PROCA1 | protein interacting with cyclin A1 [Source:HGNC Symbol;Acc:HGNC:28600] |
| ENSG00000167562 | HGLibA_56578 | 43 | -0.745743745 | ZNF701 | zinc finger protein 701 [Source:HGNC Symbol;Acc:HGNC:25597] |
| ENSG00000168476 | HGLibA_40785 | 43 | -0.745743745 | REEP4 | receptor accessory protein 4 [Source:HGNC Symbol;Acc:HGNC:26176] |
| ENSG00000169019 | HGLibA_10677 | 43 | -0.745743745 | COMMD8 | COMM domain containing 8 [Source:HGNC Symbol;Acc:HGNC:26036] |
| ENSG00000169129 | HGLibA_01103 | 43 | -0.745743745 | AFAP1L2 | actin filament associated protein 1 like 2 [Source:HGNC Symbol;Acc:HGNC:25901] |
| ENSG00000169139 | HGLibA_52670 | 43 | -0.745743745 | UBE2V2 | ubiquitin conjugating enzyme E2 V2 [Source:HGNC Symbol;Acc:HGNC:12495] |
| ENSG00000169231 | HGLibA_49329 | 43 | -0.745743745 | THBS3 | thrombospondin 3 [Source:HGNC Symbol;Acc:HGNC:11787] |
| ENSG00000169682 | HGLibA_46733 | 43 | -0.745743745 | SPNS1 | sphingolipid transporter 1 (putative) [Source:HGNC Symbol;Acc:HGNC:30621] |
| ENSG00000169744 | HGLibA_26213 | 43 | -0.745743745 | LDB2 | LIM domain binding 2 [Source:HGNC Symbol;Acc:HGNC:6533] |
| ENSG00000170265 | HGLibA_55738 | 43 | -0.745743745 | ZNF282 | zinc finger protein 282 [Source:HGNC Symbol;Acc:HGNC:13076] |
| ENSG00000170515 | HGLibA_34915 | 43 | -0.745743745 | PA2G4 | proliferation-associated 2G4 [Source:HGNC Symbol;Acc:HGNC:8550] |
| ENSG00000170790 | HGLibA_33461 | 43 | -0.745743745 | OR10A2 | olfactory receptor family 10 subfamily A member 2 [Source:HGNC Symbol;Acc:HGNC:8161] |
| ENSG00000170915 | HGLibA_35173 | 43 | -0.745743745 | PAQR8 | progestin and adipoQ receptor family member 8 [Source:HGNC Symbol;Acc:HGNC:15708] |
| ENSG00000170929 | HGLibA_33701 | 43 | -0.745743745 | OR1M1 | olfactory receptor family 1 subfamily M member 1 [Source:HGNC Symbol;Acc:HGNC:8220] |
| ENSG00000171428 | HGLibA_31165 | 43 | -0.745743745 | NAT1 | N-acetyltransferase 1 [Source:HGNC Symbol;Acc:HGNC:7645] |
| ENSG00000172137 | HGLibA_06990 | 43 | -0.745743745 | CALB2 | calbindin 2 [Source:HGNC Symbol;Acc:HGNC:1435] |
| ENSG00000172671 | HGLibA_55156 | 43 | -0.745743745 | ZFAND4 | zinc finger AN1-type containing 4 [Source:HGNC Symbol;Acc:HGNC:23504] |
| ENSG00000172673 | HGLibA_49349 | 43 | -0.745743745 | THEMIS | thymocyte selection associated [Source:HGNC Symbol;Acc:HGNC:21569] |
| ENSG00000173406 | HGLibA_12265 | 43 | -0.745743745 | DAB1 | reelin adaptor protein [Source:HGNC Symbol;Acc:HGNC:2661]" |
| ENSG00000176009 | HGLibA_03271 | 43 | -0.745743745 | ASCL3 | achaete-scute family bHLH transcription factor 3 [Source:HGNC Symbol;Acc:HGNC:740] |
| ENSG00000176165 | HGLibA_17870 | 43 | -0.745743745 | FOXG1 | forkhead box G1 [Source:HGNC Symbol;Acc:HGNC:3811] |
| ENSG00000176371 | HGLibA_56994 | 43 | -0.745743745 | ZSCAN2 | zinc finger and SCAN domain containing 2 [Source:HGNC Symbol;Acc:HGNC:20994] |
| ENSG00000176920 | HGLibA_18188 | 43 | -0.745743745 | FUT2 | fucosyltransferase 2 [Source:HGNC Symbol;Acc:HGNC:4013] |
| ENSG00000178449 | HGLibA_10810 | 43 | -0.745743745 | COX14 | cytochrome c oxidase assembly factor COX14 [Source:HGNC Symbol;Acc:HGNC:28216] |
| ENSG00000179528 | HGLibA_26087 | 43 | -0.745743745 | LBX2 | ladybird homeobox 2 [Source:HGNC Symbol;Acc:HGNC:15525] |
| ENSG00000180777 | HGLibA_02063 | 43 | -0.745743745 | ANKRD30B | ankyrin repeat domain 30B [Source:HGNC Symbol;Acc:HGNC:24165] |
| ENSG00000181023 | HGLibA_34223 | 43 | -0.745743745 | OR56B1 | olfactory receptor family 56 subfamily B member 1 [Source:HGNC Symbol;Acc:HGNC:15245] |
| ENSG00000181638 | HGLibA_55216 | 43 | -0.745743745 | ZFP41 | ZFP41 zinc finger protein [Source:HGNC Symbol;Acc:HGNC:26786] |
| ENSG00000181649 | HGLibA_36419 | 43 | -0.745743745 | PHLDA2 | pleckstrin homology like domain family A member 2 [Source:HGNC Symbol;Acc:HGNC:12385] |
| ENSG00000181803 | HGLibA_34441 | 43 | -0.745743745 | OR6S1 | olfactory receptor family 6 subfamily S member 1 [Source:HGNC Symbol;Acc:HGNC:15363] |
| ENSG00000181927 | HGLibA_34056 | 43 | -0.745743745 | OR4P4 | olfactory receptor family 4 subfamily P member 4 [Source:HGNC Symbol;Acc:HGNC:15180] |
| ENSG00000182208 | HGLibA_29591 | 43 | -0.745743745 | MOB2 | MOB kinase activator 2 [Source:HGNC Symbol;Acc:HGNC:24904] |
| ENSG00000182870 | HGLibA_18576 | 43 | -0.745743745 | GALNT9 | polypeptide N-acetylgalactosaminyltransferase 9 [Source:HGNC Symbol;Acc:HGNC:4131] |
| ENSG00000182896 | HGLibA_50564 | 43 | -0.745743745 | TMEM95 | transmembrane protein 95 [Source:HGNC Symbol;Acc:HGNC:27898] |
| ENSG00000183117 | HGLibA_11364 | 43 | -0.745743745 | CSMD1 | CUB and Sushi multiple domains 1 [Source:HGNC Symbol;Acc:HGNC:14026] |
| ENSG00000183434 | HGLibA_49156 | 43 | -0.745743745 | TFDP3 | transcription factor Dp family member 3 [Source:HGNC Symbol;Acc:HGNC:24603] |
| ENSG00000183735 | HGLibA_48601 | 43 | -0.745743745 | TBK1 | TANK binding kinase 1 [Source:HGNC Symbol;Acc:HGNC:11584] |
| ENSG00000184988 | HGLibA_49852 | 43 | -0.745743745 | TMEM106A | transmembrane protein 106A [Source:HGNC Symbol;Acc:HGNC:28288] |
| ENSG00000185818 | HGLibA_31189 | 43 | -0.745743745 | NAT8L | N-acetyltransferase 8 like [Source:HGNC Symbol;Acc:HGNC:26742] |
| ENSG00000185897 | HGLibA_17330 | 43 | -0.745743745 | FFAR3 | free fatty acid receptor 3 [Source:HGNC Symbol;Acc:HGNC:4499] |
| ENSG00000185988 | HGLibA_37199 | 43 | -0.745743745 | PLK5 | polo like kinase 5 [Source:HGNC Symbol;Acc:HGNC:27001] |
| ENSG00000187231 | HGLibA_43628 | 43 | -0.745743745 | SESTD1 | SEC14 and spectrin domain containing 1 [Source:HGNC Symbol;Acc:HGNC:18379] |
| ENSG00000187514 | HGLibA_39392 | 43 | -0.745743745 | PTMA | prothymosin alpha [Source:HGNC Symbol;Acc:HGNC:9623] |
| ENSG00000187553 | HGLibA_12090 | 43 | -0.745743745 | CYP26C1 | cytochrome P450 family 26 subfamily C member 1 [Source:HGNC Symbol;Acc:HGNC:20577] |
| ENSG00000188848 | HGLibA_04407 | 43 | -0.745743745 | BEND4 | BEN domain containing 4 [Source:HGNC Symbol;Acc:HGNC:23815] |
| ENSG00000188981 | HGLibA_30169 | 43 | -0.745743745 | MSANTD1 | Myb/SANT DNA binding domain containing 1 [Source:HGNC Symbol;Acc:HGNC:33741] |
| ENSG00000189099 | HGLibA_38951 | 43 | -0.745743745 | PRSS48 | serine protease 48 [Source:HGNC Symbol;Acc:HGNC:24635] |
| ENSG00000189120 | HGLibA_46308 | 43 | -0.745743745 | SP6 | Sp6 transcription factor [Source:HGNC Symbol;Acc:HGNC:14530] |
| ENSG00000196475 | HGLibA_19200 | 43 | -0.745743745 | GK2 | glycerol kinase 2 [Source:HGNC Symbol;Acc:HGNC:4291] |
| ENSG00000196724 | HGLibA_55952 | 43 | -0.745743745 | ZNF418 | zinc finger protein 418 [Source:HGNC Symbol;Acc:HGNC:20647] |
| ENSG00000197020 | HGLibA_55432 | 43 | -0.745743745 | ZNF100 | zinc finger protein 100 [Source:HGNC Symbol;Acc:HGNC:12880] |
| ENSG00000197540 | HGLibA_20729 | 43 | -0.745743745 | GZMM | granzyme M [Source:HGNC Symbol;Acc:HGNC:4712] |
| ENSG00000197566 | HGLibA_56416 | 43 | -0.745743745 | ZNF624 | zinc finger protein 624 [Source:HGNC Symbol;Acc:HGNC:29254] |
| ENSG00000197584 | HGLibA_24346 | 43 | -0.745743745 | KCNMB2 | potassium calcium-activated channel subfamily M regulatory beta subunit 2 [Source:HGNC Symbol;Acc:HGNC:6286] |
| ENSG00000197746 | HGLibA_39000 | 43 | -0.745743745 | PSAP | prosaposin [Source:HGNC Symbol;Acc:HGNC:9498] |
| ENSG00000197971 | HGLibA_28530 | 43 | -0.745743745 | MBP | myelin basic protein [Source:HGNC Symbol;Acc:HGNC:6925] |
| ENSG00000198056 | HGLibA_38483 | 43 | -0.745743745 | PRIM1 | DNA primase subunit 1 [Source:HGNC Symbol;Acc:HGNC:9369] |
| ENSG00000198178 | HGLibA_09981 | 43 | -0.745743745 | CLEC4C | C-type lectin domain family 4 member C [Source:HGNC Symbol;Acc:HGNC:13258] |
| ENSG00000198467 | HGLibA_51145 | 43 | -0.745743745 | TPM2 | tropomyosin 2 [Source:HGNC Symbol;Acc:HGNC:12011] |
| ENSG00000198692 | HGLibA_14715 | 43 | -0.745743745 | EIF1AY | eukaryotic translation initiation factor 1A Y-linked [Source:HGNC Symbol;Acc:HGNC:3252] |
| ENSG00000198816 | HGLibA_55870 | 43 | -0.745743745 | ZNF358 | zinc finger protein 358 [Source:HGNC Symbol;Acc:HGNC:16838] |
| ENSG00000198865 | HGLibA_07648 | 43 | -0.745743745 | CCDC152 | coiled-coil domain containing 152 [Source:HGNC Symbol;Acc:HGNC:34438] |
| ENSG00000198939 | HGLibA_55192 | 43 | -0.745743745 | ZFP2 | ZFP2 zinc finger protein [Source:HGNC Symbol;Acc:HGNC:26138] |
| ENSG00000203859 | HGLibA_22207 | 43 | -0.745743745 | HSD3B2 | 3 beta- and steroid delta-isomerase 2 [Source:HGNC Symbol;Acc:HGNC:5218]" |
| ENSG00000204469 | HGLibA_38859 | 43 | -0.745743745 | PRRC2A | proline rich coiled-coil 2A [Source:HGNC Symbol;Acc:HGNC:13918] |
| ENSG00000204520 | HGLibA_29198 | 43 | -0.745743745 | MICA | MHC class I polypeptide-related sequence A [Source:HGNC Symbol;Acc:HGNC:7090] |
| ENSG00000205409 | HGLibA_34164 | 43 | -0.745743745 | OR52E6 | olfactory receptor family 52 subfamily E member 6 [Source:HGNC Symbol;Acc:HGNC:15215] |
| ENSG00000206474 | HGLibA_33485 | 43 | -0.745743745 | OR10C1 | olfactory receptor family 10 subfamily C member 1 (gene/pseudogene) [Source:HGNC Symbol;Acc:HGNC:8165] |
| ENSG00000213853 | HGLibA_15077 | 43 | -0.745743745 | EMP2 | epithelial membrane protein 2 [Source:HGNC Symbol;Acc:HGNC:3334] |
| ENSG00000228672 | HGLibA_38646 | 43 | -0.745743745 | PROB1 | proline rich basic protein 1 [Source:HGNC Symbol;Acc:HGNC:41906] |
| ENSG00000243943 | HGLibA_56130 | 43 | -0.745743745 | ZNF512 | zinc finger protein 512 [Source:HGNC Symbol;Acc:HGNC:29380] |
| ENSG00000245680 | HGLibA_56323 | 43 | -0.745743745 | ZNF585B | zinc finger protein 585B [Source:HGNC Symbol;Acc:HGNC:30948] |
| ENSG00000249242 | HGLibA_49997 | 43 | -0.745743745 | TMEM150C | transmembrane protein 150C [Source:HGNC Symbol;Acc:HGNC:37263] |
| ENSG00000253710 | HGLibA_01609 | 43 | -0.745743745 | ALG11 | 2-mannosyltransferase [Source:HGNC Symbol;Acc:HGNC:32456]" |
| ENSG00000266173 | HGLibA_47573 | 43 | -0.745743745 | STRADA | STE20-related kinase adaptor alpha [Source:HGNC Symbol;Acc:HGNC:30172] |
| ENSG00000270800 | HGLibA_42061 | 43 | -0.745743745 | RPS10-NUDT3 | RPS10-NUDT3 readthrough [Source:HGNC Symbol;Acc:HGNC:49181] |
| ENSG00000279116 | HGLibA_34511 | 43 | -0.745743745 | OR8D2 | olfactory receptor family 8 subfamily D member 2 (gene/pseudogene) [Source:HGNC Symbol;Acc:HGNC:8482] |
| FAM134C | HGLibA_16129 | 43 | -0.745743745 | FAM134C | - |
| HIST1H2AG | HGLibA_21389 | 43 | -0.745743745 | HIST1H2AG | - |
| hsa-mir-1279 | HGLibA_57569 | 43 | -0.745743745 | hsa-mir-1279 | - |
| hsa-mir-129-1 | HGLibA_57628 | 43 | -0.745743745 | hsa-mir-129-1 | - |
| hsa-mir-132 | HGLibA_57757 | 43 | -0.745743745 | hsa-mir-132 | - |
| hsa-mir-148b | HGLibA_57883 | 43 | -0.745743745 | hsa-mir-148b | - |
| hsa-mir-182 | HGLibA_57983 | 43 | -0.745743745 | hsa-mir-182 | - |
| hsa-mir-182 | HGLibA_57982 | 43 | -0.745743745 | hsa-mir-182 | - |
| hsa-mir-199b | HGLibA_58137 | 43 | -0.745743745 | hsa-mir-199b | - |
| hsa-mir-301b | HGLibA_58432 | 43 | -0.745743745 | hsa-mir-301b | - |
| hsa-mir-3127 | HGLibA_58562 | 43 | -0.745743745 | hsa-mir-3127 | - |
| hsa-mir-3180-2 | HGLibA_58804 | 43 | -0.745743745 | hsa-mir-3180-2 | - |
| hsa-mir-3609 | HGLibA_59066 | 43 | -0.745743745 | hsa-mir-3609 | - |
| hsa-mir-3659 | HGLibA_59183 | 43 | -0.745743745 | hsa-mir-3659 | - |
| hsa-mir-424 | HGLibA_59705 | 43 | -0.745743745 | hsa-mir-424 | - |
| hsa-mir-4429 | HGLibA_60104 | 43 | -0.745743745 | hsa-mir-4429 | - |
| hsa-mir-4483 | HGLibA_60352 | 43 | -0.745743745 | hsa-mir-4483 | - |
| hsa-mir-449a | HGLibA_60418 | 43 | -0.745743745 | hsa-mir-449a | - |
| hsa-mir-4711 | HGLibA_60943 | 43 | -0.745743745 | hsa-mir-4711 | - |
| hsa-mir-4771-1 | HGLibA_61180 | 43 | -0.745743745 | hsa-mir-4771-1 | - |
| hsa-mir-4771-2 | HGLibA_61185 | 43 | -0.745743745 | hsa-mir-4771-2 | - |
| hsa-mir-5087 | HGLibA_61496 | 43 | -0.745743745 | hsa-mir-5087 | - |
| hsa-mir-510 | HGLibA_61546 | 43 | -0.745743745 | hsa-mir-510 | - |
| hsa-mir-518c | HGLibA_61663 | 43 | -0.745743745 | hsa-mir-518c | - |
| hsa-mir-542 | HGLibA_61817 | 43 | -0.745743745 | hsa-mir-542 | - |
| hsa-mir-548ao | HGLibA_61909 | 43 | -0.745743745 | hsa-mir-548ao | - |
| hsa-mir-5701-1 | HGLibA_62339 | 43 | -0.745743745 | hsa-mir-5701-1 | - |
| hsa-mir-6505 | HGLibA_62870 | 43 | -0.745743745 | hsa-mir-6505 | - |
| hsa-mir-6730 | HGLibA_63073 | 43 | -0.745743745 | hsa-mir-6730 | - |
| hsa-mir-6762 | HGLibA_63208 | 43 | -0.745743745 | hsa-mir-6762 | - |
| hsa-mir-7-2 | HGLibA_63771 | 43 | -0.745743745 | hsa-mir-7-2 | - |
| hsa-mir-892c | HGLibA_64259 | 43 | -0.745743745 | hsa-mir-892c | - |
| hsa-mir-944 | HGLibA_64362 | 43 | -0.745743745 | hsa-mir-944 | - |
| LOC100130480 | HGLibA_26790 | 43 | -0.745743745 | LOC100130480 | - |
| MAATS1 | HGLibA_27818 | 43 | -0.745743745 | MAATS1 | - |
| MKL2 | HGLibA_29336 | 43 | -0.745743745 | MKL2 | - |
| NPIP | HGLibA_32459 | 43 | -0.745743745 | NPIP | - |
| NUPL1 | HGLibA_33080 | 43 | -0.745743745 | NUPL1 | - |
| PPP2R4 | HGLibA_38166 | 43 | -0.745743745 | PPP2R4 | - |
| PRR21 | HGLibA_38815 | 43 | -0.745743745 | PRR21 | - |

| | | | | | |
|---|---|---|---|---|---|
| TMEM261 | HGLibA_50354 | 43 | -0.745743745 | TMEM261 | - |
| TWISTNB | HGLibA_52382 | 43 | -0.745743745 | TWISTNB | - |
| WDR63 | HGLibA_54148 | 43 | -0.745743745 | WDR63 | - |
| ASNA1 | HGLibA_03331 | 42 | -0.754789088 | ASNA1 | - |
| C10orf54 | HGLibA_05077 | 42 | -0.754789088 | C10orf54 | - |
| C15orf38-AP3S2 | HGLibA_05389 | 42 | -0.754789088 | C15orf38-AP3S2 | - |
| C1orf95 | HGLibA_05910 | 42 | -0.754789088 | C1orf95 | - |
| C3orf72 | HGLibA_06266 | 42 | -0.754789088 | C3orf72 | - |
| CASC4 | HGLibA_07278 | 42 | -0.754789088 | CASC4 | - |
| CCDC135 | HGLibA_07600 | 42 | -0.754789088 | CCDC135 | - |
| ENSG00000005471 | HGLibA_00140 | 42 | -0.754789088 | ABCB4 | ATP binding cassette subfamily B member 4 [Source:HGNC Symbol;Acc:HGNC:45] |
| ENSG00000005893 | HGLibA_25980 | 42 | -0.754789088 | LAMP2 | lysosomal associated membrane protein 2 [Source:HGNC Symbol;Acc:HGNC:6501] |
| ENSG00000007314 | HGLibA_42993 | 42 | -0.754789088 | SCN4A | sodium voltage-gated channel alpha subunit 4 [Source:HGNC Symbol;Acc:HGNC:10591] |
| ENSG00000008517 | HGLibA_23135 | 42 | -0.754789088 | IL32 | interleukin 32 [Source:HGNC Symbol;Acc:HGNC:16830] |
| ENSG00000009954 | HGLibA_04187 | 42 | -0.754789088 | BAZ1B | bromodomain adjacent to zinc finger domain 1B [Source:HGNC Symbol;Acc:HGNC:961] |
| ENSG00000010610 | HGLibA_08382 | 42 | -0.754789088 | CD4 | CD4 molecule [Source:HGNC Symbol;Acc:HGNC:1678] |
| ENSG00000011143 | HGLibA_29357 | 42 | -0.754789088 | MKS1 | type 1 [Source:HGNC Symbol;Acc:HGNC:7121]" |
| ENSG00000011243 | HGLibA_01447 | 42 | -0.754789088 | AKAP8L | A-kinase anchoring protein 8 like [Source:HGNC Symbol;Acc:HGNC:29857] |
| ENSG00000013364 | HGLibA_30601 | 42 | -0.754789088 | MVP | major vault protein [Source:HGNC Symbol;Acc:HGNC:7531] |
| ENSG00000019995 | HGLibA_56972 | 42 | -0.754789088 | ZRANB1 | zinc finger RANBP2-type containing 1 [Source:HGNC Symbol;Acc:HGNC:18224] |
| ENSG00000028839 | HGLibA_48625 | 42 | -0.754789088 | TBPL1 | TATA-box binding protein like 1 [Source:HGNC Symbol;Acc:HGNC:11589] |
| ENSG00000039139 | HGLibA_13489 | 42 | -0.754789088 | DNAH5 | dynein axonemal heavy chain 5 [Source:HGNC Symbol;Acc:HGNC:2950] |
| ENSG00000046889 | HGLibA_38445 | 42 | -0.754789088 | PREX2 | 5-trisphosphate dependent Rac exchange factor 2 [Source:HGNC Symbol;Acc:HGNC:22950]" |
| ENSG00000047365 | HGLibA_02627 | 42 | -0.754789088 | ARAP2 | ankyrin repeat and PH domain 2 [Source:HGNC Symbol;Acc:HGNC:16924]" |
| ENSG00000049883 | HGLibA_39263 | 42 | -0.754789088 | PTCD2 | pentatricopeptide repeat domain 2 [Source:HGNC Symbol;Acc:HGNC:25734] |
| ENSG00000050820 | HGLibA_04246 | 42 | -0.754789088 | BCAR1 | Cas family scaffold protein [Source:HGNC Symbol;Acc:HGNC:971]" |
| ENSG00000053438 | HGLibA_32219 | 42 | -0.754789088 | NNAT | neuronatin [Source:HGNC Symbol;Acc:HGNC:7860] |
| ENSG00000057468 | HGLibA_30196 | 42 | -0.754789088 | MSH4 | mutS homolog 4 [Source:HGNC Symbol;Acc:HGNC:7327] |
| ENSG00000060709 | HGLibA_41259 | 42 | -0.754789088 | RIMBP2 | RIMS binding protein 2 [Source:HGNC Symbol;Acc:HGNC:30339] |
| ENSG00000065883 | HGLibA_08733 | 42 | -0.754789088 | CDK13 | cyclin dependent kinase 13 [Source:HGNC Symbol;Acc:HGNC:1733] |
| ENSG00000073282 | HGLibA_51094 | 42 | -0.754789088 | TP63 | tumor protein p63 [Source:HGNC Symbol;Acc:HGNC:15979] |
| ENSG00000073417 | HGLibA_35828 | 42 | -0.754789088 | PDE8A | phosphodiesterase 8A [Source:HGNC Symbol;Acc:HGNC:8793] |
| ENSG00000075218 | HGLibA_20626 | 42 | -0.754789088 | GTSE1 | G2 and S-phase expressed 1 [Source:HGNC Symbol;Acc:HGNC:13698] |
| ENSG00000077147 | HGLibA_49726 | 42 | -0.754789088 | TM9SF3 | transmembrane 9 superfamily member 3 [Source:HGNC Symbol;Acc:HGNC:21529] |
| ENSG00000078549 | HGLibA_00937 | 42 | -0.754789088 | ADCYAP1R1 | ADCYAP receptor type I [Source:HGNC Symbol;Acc:HGNC:242] |
| ENSG00000082126 | HGLibA_29756 | 42 | -0.754789088 | MPP4 | membrane palmitoylated protein 4 [Source:HGNC Symbol;Acc:HGNC:13680] |
| ENSG00000087206 | HGLibA_52957 | 42 | -0.754789088 | UIMC1 | ubiquitin interaction motif containing 1 [Source:HGNC Symbol;Acc:HGNC:30298] |
| ENSG00000089597 | HGLibA_18606 | 42 | -0.754789088 | GANAB | glucosidase II alpha subunit [Source:HGNC Symbol;Acc:HGNC:4138] |
| ENSG00000091409 | HGLibA_23739 | 42 | -0.754789088 | ITGA6 | integrin subunit alpha 6 [Source:HGNC Symbol;Acc:HGNC:6142] |
| ENSG00000094661 | HGLibA_33671 | 42 | -0.754789088 | OR1I1 | olfactory receptor family 1 subfamily I member 1 [Source:HGNC Symbol;Acc:HGNC:8207] |
| ENSG00000100353 | HGLibA_14774 | 42 | -0.754789088 | EIF3D | eukaryotic translation initiation factor 3 subunit D [Source:HGNC Symbol;Acc:HGNC:3278] |
| ENSG00000101082 | HGLibA_44303 | 42 | -0.754789088 | SLA2 | Src like adaptor 2 [Source:HGNC Symbol;Acc:HGNC:17329] |
| ENSG00000101892 | HGLibA_03603 | 42 | -0.754789088 | ATP1B4 | ATPase Na+/K+ transporting family member beta 4 [Source:HGNC Symbol;Acc:HGNC:808] |
| ENSG00000102796 | HGLibA_13125 | 42 | -0.754789088 | DHRS12 | dehydrogenase/reductase 12 [Source:HGNC Symbol;Acc:HGNC:25832] |
| ENSG00000102984 | HGLibA_56817 | 42 | -0.754789088 | ZNF821 | zinc finger protein 821 [Source:HGNC Symbol;Acc:HGNC:28043] |
| ENSG00000103356 | HGLibA_14342 | 42 | -0.754789088 | EARS2 | mitochondrial [Source:HGNC Symbol;Acc:HGNC:29419]" |
| ENSG00000104147 | HGLibA_33333 | 42 | -0.754789088 | OIP5 | Opa interacting protein 5 [Source:HGNC Symbol;Acc:HGNC:20300] |
| ENSG00000104177 | HGLibA_30687 | 42 | -0.754789088 | MYEF2 | myelin expression factor 2 [Source:HGNC Symbol;Acc:HGNC:17940] |
| ENSG00000105135 | HGLibA_23225 | 42 | -0.754789088 | ILVBL | ilvB acetolactate synthase like [Source:HGNC Symbol;Acc:HGNC:6041] |
| ENSG00000105186 | HGLibA_02050 | 42 | -0.754789088 | ANKRD27 | ankyrin repeat domain 27 [Source:HGNC Symbol;Acc:HGNC:25310] |
| ENSG00000105251 | HGLibA_44019 | 42 | -0.754789088 | SHD | Src homology 2 domain containing transforming protein D [Source:HGNC Symbol;Acc:HGNC:30633] |
| ENSG00000105821 | HGLibA_13595 | 42 | -0.754789088 | DNAJC2 | DnaJ heat shock protein family (Hsp40) member C2 [Source:HGNC Symbol;Acc:HGNC:13192] |
| ENSG00000107242 | HGLibA_36717 | 42 | -0.754789088 | PIP5K1B | phosphatidylinositol-4-phosphate 5-kinase type 1 beta [Source:HGNC Symbol;Acc:HGNC:8995] |
| ENSG00000107341 | HGLibA_52653 | 42 | -0.754789088 | UBE2R2 | ubiquitin conjugating enzyme E2 R2 [Source:HGNC Symbol;Acc:HGNC:19907] |
| ENSG00000108582 | HGLibA_10912 | 42 | -0.754789088 | CPD | carboxypeptidase D [Source:HGNC Symbol;Acc:HGNC:2301] |
| ENSG00000109016 | HGLibA_13149 | 42 | -0.754789088 | DHRS7B | dehydrogenase/reductase 7B [Source:HGNC Symbol;Acc:HGNC:24547] |
| ENSG00000109705 | HGLibA_32053 | 42 | -0.754789088 | NKX3-2 | NK3 homeobox 2 [Source:HGNC Symbol;Acc:HGNC:951] |
| ENSG00000109956 | HGLibA_03973 | 42 | -0.754789088 | B3GAT1 | 3-glucuronyltransferase 1 [Source:HGNC Symbol;Acc:HGNC:921]" |
| ENSG00000110717 | HGLibA_31565 | 42 | -0.754789088 | NDUFS8 | NADH:ubiquinone oxidoreductase core subunit S8 [Source:HGNC Symbol;Acc:HGNC:7715] |
| ENSG00000110844 | HGLibA_38737 | 42 | -0.754789088 | PRPF40B | pre-mRNA processing factor 40 homolog B [Source:HGNC Symbol;Acc:HGNC:25031] |
| ENSG00000111405 | HGLibA_15118 | 42 | -0.754789088 | ENDOU | poly(U) specific [Source:HGNC Symbol;Acc:HGNC:14369]" |
| ENSG00000111816 | HGLibA_18005 | 42 | -0.754789088 | FRK | fyn related Src family tyrosine kinase [Source:HGNC Symbol;Acc:HGNC:3955] |
| ENSG00000113262 | HGLibA_20355 | 42 | -0.754789088 | GRM6 | glutamate metabotropic receptor 6 [Source:HGNC Symbol;Acc:HGNC:4598] |
| ENSG00000115009 | HGLibA_07992 | 42 | -0.754789088 | CCL20 | C-C motif chemokine ligand 20 [Source:HGNC Symbol;Acc:HGNC:10619] |
| ENSG00000115306 | HGLibA_46879 | 42 | -0.754789088 | SPTBN1 | non-erythrocytic 1 [Source:HGNC Symbol;Acc:HGNC:11275]" |
| ENSG00000115525 | HGLibA_47248 | 42 | -0.754789088 | ST3GAL5 | 3-sialyltransferase 5 [Source:HGNC Symbol;Acc:HGNC:10872]" |
| ENSG00000115977 | HGLibA_00049 | 42 | -0.754789088 | AAK1 | AP2 associated kinase 1 [Source:HGNC Symbol;Acc:HGNC:19679] |
| ENSG00000116874 | HGLibA_53946 | 42 | -0.754789088 | WARS2 | mitochondrial [Source:HGNC Symbol;Acc:HGNC:12730]" |
| ENSG00000117625 | HGLibA_40716 | 42 | -0.754789088 | RCOR3 | REST corepressor 3 [Source:HGNC Symbol;Acc:HGNC:25594] |
| ENSG00000117707 | HGLibA_38701 | 42 | -0.754789088 | PROX1 | prospero homeobox 1 [Source:HGNC Symbol;Acc:HGNC:9459] |
| ENSG00000117868 | HGLibA_15607 | 42 | -0.754789088 | ESYT2 | extended synaptotagmin 2 [Source:HGNC Symbol;Acc:HGNC:22211] |
| ENSG00000118363 | HGLibA_46562 | 42 | -0.754789088 | SPCS2 | signal peptidase complex subunit 2 [Source:HGNC Symbol;Acc:HGNC:28962] |
| ENSG00000118473 | HGLibA_43815 | 42 | -0.754789088 | SGIP1 | SH3 domain GRB2 like endophilin interacting protein 1 [Source:HGNC Symbol;Acc:HGNC:25412] |
| ENSG00000119471 | HGLibA_22217 | 42 | -0.754789088 | HSDL2 | hydroxysteroid dehydrogenase like 2 [Source:HGNC Symbol;Acc:HGNC:18572] |
| ENSG00000119608 | HGLibA_38703 | 42 | -0.754789088 | PROX2 | prospero homeobox 2 [Source:HGNC Symbol;Acc:HGNC:26715] |
| ENSG00000119737 | HGLibA_20079 | 42 | -0.754789088 | GPR75 | G protein-coupled receptor 75 [Source:HGNC Symbol;Acc:HGNC:4526] |
| ENSG00000120088 | HGLibA_11141 | 42 | -0.754789088 | CRHR1 | corticotropin releasing hormone receptor 1 [Source:HGNC Symbol;Acc:HGNC:2357] |
| ENSG00000120526 | HGLibA_32921 | 42 | -0.754789088 | NUDCD1 | NudC domain containing 1 [Source:HGNC Symbol;Acc:HGNC:24306] |
| ENSG00000120688 | HGLibA_53984 | 42 | -0.754789088 | WBP4 | WW domain binding protein 4 [Source:HGNC Symbol;Acc:HGNC:12739] |
| ENSG00000121895 | HGLibA_50012 | 42 | -0.754789088 | TMEM156 | transmembrane protein 156 [Source:HGNC Symbol;Acc:HGNC:26260] |
| ENSG00000121966 | HGLibA_11890 | 42 | -0.754789088 | CXCR4 | C-X-C motif chemokine receptor 4 [Source:HGNC Symbol;Acc:HGNC:2561] |
| ENSG00000124212 | HGLibA_39348 | 42 | -0.754789088 | PTGIS | prostaglandin I2 synthase [Source:HGNC Symbol;Acc:HGNC:9603] |
| ENSG00000124614 | HGLibA_42059 | 42 | -0.754789088 | RPS10 | ribosomal protein S10 [Source:HGNC Symbol;Acc:HGNC:10383] |
| ENSG00000124766 | HGLibA_46263 | 42 | -0.754789088 | SOX4 | SRY-box 4 [Source:HGNC Symbol;Acc:HGNC:11200] |
| ENSG00000125571 | HGLibA_23158 | 42 | -0.754789088 | IL37 | interleukin 37 [Source:HGNC Symbol;Acc:HGNC:15563] |
| ENSG00000126067 | HGLibA_39104 | 42 | -0.754789088 | PSMB2 | proteasome subunit beta 2 [Source:HGNC Symbol;Acc:HGNC:9539] |
| ENSG00000126391 | HGLibA_18029 | 42 | -0.754789088 | FRMD8 | FERM domain containing 8 [Source:HGNC Symbol;Acc:HGNC:25462] |
| ENSG00000126602 | HGLibA_51295 | 42 | -0.754789088 | TRAP1 | TNF receptor associated protein 1 [Source:HGNC Symbol;Acc:HGNC:16264] |
| ENSG00000127780 | HGLibA_33663 | 42 | -0.754789088 | OR1E2 | olfactory receptor family 1 subfamily E member 2 [Source:HGNC Symbol;Acc:HGNC:8190] |
| ENSG00000130508 | HGLibA_39649 | 42 | -0.754789088 | PXDN | peroxidasin [Source:HGNC Symbol;Acc:HGNC:14966] |
| ENSG00000131263 | HGLibA_41350 | 42 | -0.754789088 | RLIM | LIM domain interacting [Source:HGNC Symbol;Acc:HGNC:13429]" |
| ENSG00000131475 | HGLibA_53748 | 42 | -0.754789088 | VPS25 | vacuolar protein sorting 25 homolog [Source:HGNC Symbol;Acc:HGNC:28122] |
| ENSG00000131477 | HGLibA_40182 | 42 | -0.754789088 | RAMP2 | receptor activity modifying protein 2 [Source:HGNC Symbol;Acc:HGNC:9844] |
| ENSG00000133063 | HGLibA_09453 | 42 | -0.754789088 | CHIT1 | chitinase 1 [Source:HGNC Symbol;Acc:HGNC:1936] |
| ENSG00000134121 | HGLibA_09464 | 42 | -0.754789088 | CHL1 | cell adhesion molecule L1 like [Source:HGNC Symbol;Acc:HGNC:1939] |
| ENSG00000134198 | HGLibA_51921 | 42 | -0.754789088 | TSPAN2 | tetraspanin 2 [Source:HGNC Symbol;Acc:HGNC:20659] |
| ENSG00000134215 | HGLibA_53555 | 42 | -0.754789088 | VAV3 | vav guanine nucleotide exchange factor 3 [Source:HGNC Symbol;Acc:HGNC:12659] |
| ENSG00000135426 | HGLibA_49033 | 42 | -0.754789088 | TESPA1 | positive selection associated 1 [Source:HGNC Symbol;Acc:HGNC:29109]" |
| ENSG00000135587 | HGLibA_45805 | 42 | -0.754789088 | SMPD2 | sphingomyelin phosphodiesterase 2 [Source:HGNC Symbol;Acc:HGNC:11121] |
| ENSG00000136950 | HGLibA_03094 | 42 | -0.754789088 | ARPC5L | actin related protein 2/3 complex subunit 5 like [Source:HGNC Symbol;Acc:HGNC:23366] |
| ENSG00000137312 | HGLibA_17647 | 42 | -0.754789088 | FLOT1 | flotillin 1 [Source:HGNC Symbol;Acc:HGNC:3757] |
| ENSG00000137809 | HGLibA_23721 | 42 | -0.754789088 | ITGA11 | integrin subunit alpha 11 [Source:HGNC Symbol;Acc:HGNC:6136] |
| ENSG00000138433 | HGLibA_09718 | 42 | -0.754789088 | CIR1 | 1 [Source:HGNC Symbol;Acc:HGNC:24217]" |
| ENSG00000138670 | HGLibA_40331 | 42 | -0.754789088 | RASGEF1B | RasGEF domain family member 1B [Source:HGNC Symbol;Acc:HGNC:24881] |
| ENSG00000138741 | HGLibA_51730 | 42 | -0.754789088 | TRPC3 | transient receptor potential cation channel subfamily C member 3 [Source:HGNC Symbol;Acc:HGNC:12335] |
| ENSG00000139194 | HGLibA_40641 | 42 | -0.754789088 | RBP5 | retinol binding protein 5 [Source:HGNC Symbol;Acc:HGNC:15847] |
| ENSG00000140543 | HGLibA_13012 | 42 | -0.754789088 | DET1 | COP1 ubiquitin ligase partner [Source:HGNC Symbol;Acc:HGNC:25477]" |
| ENSG00000141378 | HGLibA_39553 | 42 | -0.754789088 | PTRH2 | peptidyl-tRNA hydrolase 2 [Source:HGNC Symbol;Acc:HGNC:24265] |
| ENSG00000141519 | HGLibA_07774 | 42 | -0.754789088 | CCDC40 | coiled-coil domain containing 40 [Source:HGNC Symbol;Acc:HGNC:26090] |
| ENSG00000141741 | HGLibA_29249 | 42 | -0.754789088 | MIEN1 | migration and invasion enhancer 1 [Source:HGNC Symbol;Acc:HGNC:28230] |
| ENSG00000142208 | HGLibA_01504 | 42 | -0.754789088 | AKT1 | AKT serine/threonine kinase 1 [Source:HGNC Symbol;Acc:HGNC:391] |
| ENSG00000143418 | HGLibA_09202 | 42 | -0.754789088 | CERS2 | ceramide synthase 2 [Source:HGNC Symbol;Acc:HGNC:14076] |
| ENSG00000144476 | HGLibA_00446 | 42 | -0.754789088 | ACKR3 | atypical chemokine receptor 3 [Source:HGNC Symbol;Acc:HGNC:23692] |
| ENSG00000144834 | HGLibA_48277 | 42 | -0.754789088 | TAGLN3 | transgelin 3 [Source:HGNC Symbol;Acc:HGNC:29868] |
| ENSG00000145349 | HGLibA_07073 | 42 | -0.754789088 | CAMK2D | calcium/calmodulin dependent protein kinase II delta [Source:HGNC Symbol;Acc:HGNC:1462] |
| ENSG00000145365 | HGLibA_49458 | 42 | -0.754789088 | TIFA | TRAF interacting protein with forkhead associated domain [Source:HGNC Symbol;Acc:HGNC:19075] |
| ENSG00000146828 | HGLibA_44381 | 42 | -0.754789088 | SLC12A9 | solute carrier family 12 member 9 [Source:HGNC Symbol;Acc:HGNC:17435] |
| ENSG00000147465 | HGLibA_47347 | 42 | -0.754789088 | STAR | steroidogenic acute regulatory protein [Source:HGNC Symbol;Acc:HGNC:11359] |
| ENSG00000147896 | HGLibA_22717 | 42 | -0.754789088 | IFNK | interferon kappa [Source:HGNC Symbol;Acc:HGNC:21714] |
| ENSG00000148386 | HGLibA_26187 | 42 | -0.754789088 | LCN9 | lipocalin 9 [Source:HGNC Symbol;Acc:HGNC:17442] |
| ENSG00000151532 | HGLibA_53887 | 42 | -0.754789088 | VTI1A | vesicle transport through interaction with t-SNAREs 1A [Source:HGNC Symbol;Acc:HGNC:17792] |
| ENSG00000152439 | HGLibA_56714 | 42 | -0.754789088 | ZNF773 | zinc finger protein 773 [Source:HGNC Symbol;Acc:HGNC:30487] |
| ENSG00000153339 | HGLibA_51335 | 42 | -0.754789088 | TRAPPC8 | trafficking protein particle complex 8 [Source:HGNC Symbol;Acc:HGNC:29169] |
| ENSG00000153922 | HGLibA_09383 | 42 | -0.754789088 | CHD1 | chromodomain helicase DNA binding protein 1 [Source:HGNC Symbol;Acc:HGNC:1915] |
| ENSG00000154102 | HGLibA_05480 | 42 | -0.754789088 | C16orf74 | chromosome 16 open reading frame 74 [Source:HGNC Symbol;Acc:HGNC:23362] |
| ENSG00000154328 | HGLibA_31632 | 42 | -0.754789088 | NEIL2 | nei like DNA glycosylase 2 [Source:HGNC Symbol;Acc:HGNC:18956] |
| ENSG00000154556 | HGLibA_46177 | 42 | -0.754789088 | SORBS2 | sorbin and SH3 domain containing 2 [Source:HGNC Symbol;Acc:HGNC:24098] |
| ENSG00000154781 | HGLibA_07704 | 42 | -0.754789088 | CCDC174 | coiled-coil domain containing 174 [Source:HGNC Symbol;Acc:HGNC:28033] |
| ENSG00000155886 | HGLibA_44619 | 42 | -0.754789088 | SLC24A2 | solute carrier family 24 member 2 [Source:HGNC Symbol;Acc:HGNC:10976] |
| ENSG00000156574 | HGLibA_32249 | 42 | -0.754789088 | NODAL | nodal growth differentiation factor [Source:HGNC Symbol;Acc:HGNC:7865] |
| ENSG00000156738 | HGLibA_30126 | 42 | -0.754789088 | MS4A1 | membrane spanning 4-domains A1 [Source:HGNC Symbol;Acc:HGNC:7315] |
| ENSG00000157778 | HGLibA_39210 | 42 | -0.754789088 | PSMG3 | proteasome assembly chaperone 3 [Source:HGNC Symbol;Acc:HGNC:22420] |
| ENSG00000158050 | HGLibA_14156 | 42 | -0.754789088 | DUSP2 | dual specificity phosphatase 2 [Source:HGNC Symbol;Acc:HGNC:3068] |
| ENSG00000158079 | HGLibA_39411 | 42 | -0.754789088 | PTPDC1 | protein tyrosine phosphatase domain containing 1 [Source:HGNC Symbol;Acc:HGNC:30184] |
| ENSG00000159069 | HGLibA_17149 | 42 | -0.754789088 | FBXW5 | F-box and WD repeat domain containing 5 [Source:HGNC Symbol;Acc:HGNC:13613] |

| | | | | | |
|---|---|---|---|---|---|
| ENSG00000159450 | HGLibA_48803 | 42 | -0.754789088 | TCHH | trichohyalin [Source:HGNC Symbol;Acc:HGNC:11791] |
| ENSG00000160803 | HGLibA_52740 | 42 | -0.754789088 | UBQLN4 | ubiquilin 4 [Source:HGNC Symbol;Acc:HGNC:1237] |
| ENSG00000162757 | HGLibA_05894 | 42 | -0.754789088 | C1orf74 | chromosome 1 open reading frame 74 [Source:HGNC Symbol;Acc:HGNC:26319] |
| ENSG00000163254 | HGLibA_11275 | 42 | -0.754789088 | CRYGC | crystallin gamma C [Source:HGNC Symbol;Acc:HGNC:2410] |
| ENSG00000163440 | HGLibA_35750 | 42 | -0.754789088 | PDCL2 | phosducin like 2 [Source:HGNC Symbol;Acc:HGNC:29524] |
| ENSG00000163584 | HGLibA_41902 | 42 | -0.754789088 | RPL22L1 | ribosomal protein L22 like 1 [Source:HGNC Symbol;Acc:HGNC:27610] |
| ENSG00000163586 | HGLibA_15908 | 42 | -0.754789088 | FABP1 | fatty acid binding protein 1 [Source:HGNC Symbol;Acc:HGNC:3555] |
| ENSG00000163961 | HGLibA_41564 | 42 | -0.754789088 | RNF168 | ring finger protein 168 [Source:HGNC Symbol;Acc:HGNC:26661] |
| ENSG00000164049 | HGLibA_17142 | 42 | -0.754789088 | FBXW12 | F-box and WD repeat domain containing 12 [Source:HGNC Symbol;Acc:HGNC:20729] |
| ENSG00000164756 | HGLibA_44920 | 42 | -0.754789088 | SLC30A8 | solute carrier family 30 member 8 [Source:HGNC Symbol;Acc:HGNC:20303] |
| ENSG00000165105 | HGLibA_40327 | 42 | -0.754789088 | RASEF | RAS and EF-hand domain containing [Source:HGNC Symbol;Acc:HGNC:26464] |
| ENSG00000165475 | HGLibA_11288 | 42 | -0.754789088 | CRYL1 | crystallin lambda 1 [Source:HGNC Symbol;Acc:HGNC:18246] |
| ENSG00000165630 | HGLibA_38711 | 42 | -0.754789088 | PRPF18 | pre-mRNA processing factor 18 [Source:HGNC Symbol;Acc:HGNC:17351] |
| ENSG00000165886 | HGLibA_52764 | 42 | -0.754789088 | UBTD1 | ubiquitin domain containing 1 [Source:HGNC Symbol;Acc:HGNC:25683] |
| ENSG00000166391 | HGLibA_29626 | 42 | -0.754789088 | MOGAT2 | monoacylglycerol O-acyltransferase 2 [Source:HGNC Symbol;Acc:HGNC:23248] |
| ENSG00000167196 | HGLibA_17049 | 42 | -0.754789088 | FBXO22 | F-box protein 22 [Source:HGNC Symbol;Acc:HGNC:13593] |
| ENSG00000167272 | HGLibA_37694 | 42 | -0.754789088 | POP5 | ribonuclease P/MRP subunit [Source:HGNC Symbol;Acc:HGNC:17689]" |
| ENSG00000167748 | HGLibA_25232 | 42 | -0.754789088 | KLK1 | kallikrein 1 [Source:HGNC Symbol;Acc:HGNC:6357] |
| ENSG00000168917 | HGLibA_45018 | 42 | -0.754789088 | SLC35G2 | solute carrier family 35 member G2 [Source:HGNC Symbol;Acc:HGNC:28480] |
| ENSG00000169248 | HGLibA_11847 | 42 | -0.754789088 | CXCL11 | C-X-C motif chemokine ligand 11 [Source:HGNC Symbol;Acc:HGNC:10638] |
| ENSG00000169403 | HGLibA_39244 | 42 | -0.754789088 | PTAFR | platelet activating factor receptor [Source:HGNC Symbol;Acc:HGNC:9582] |
| ENSG00000169554 | HGLibA_55137 | 42 | -0.754789088 | ZEB2 | zinc finger E-box binding homeobox 2 [Source:HGNC Symbol;Acc:HGNC:14881] |
| ENSG00000169679 | HGLibA_04989 | 42 | -0.754789088 | BUB1 | BUB1 mitotic checkpoint serine/threonine kinase [Source:HGNC Symbol;Acc:HGNC:1148] |
| ENSG00000170561 | HGLibA_23631 | 42 | -0.754789088 | IRX2 | iroquois homeobox 2 [Source:HGNC Symbol;Acc:HGNC:14359] |
| ENSG00000170632 | HGLibA_03009 | 42 | -0.754789088 | ARMC10 | armadillo repeat containing 10 [Source:HGNC Symbol;Acc:HGNC:21706] |
| ENSG00000171103 | HGLibA_51701 | 42 | -0.754789088 | TRMT61B | tRNA methyltransferase 61B [Source:HGNC Symbol;Acc:HGNC:26070] |
| ENSG00000171431 | HGLibA_25447 | 42 | -0.754789088 | KRT20 | keratin 20 [Source:HGNC Symbol;Acc:HGNC:20412] |
| ENSG00000171729 | HGLibA_50430 | 42 | -0.754789088 | TMEM51 | transmembrane protein 51 [Source:HGNC Symbol;Acc:HGNC:25488] |
| ENSG00000173013 | HGLibA_07938 | 42 | -0.754789088 | CCDC96 | coiled-coil domain containing 96 [Source:HGNC Symbol;Acc:HGNC:26900] |
| ENSG00000175065 | HGLibA_14030 | 42 | -0.754789088 | DSG4 | desmoglein 4 [Source:HGNC Symbol;Acc:HGNC:21307] |
| ENSG00000175643 | HGLibA_41376 | 42 | -0.754789088 | RMI2 | RecQ mediated genome instability 2 [Source:HGNC Symbol;Acc:HGNC:28349] |
| ENSG00000176782 | HGLibA_12818 | 42 | -0.754789088 | DEFB104A | defensin beta 104A [Source:HGNC Symbol;Acc:HGNC:18115] |
| ENSG00000177485 | HGLibA_54862 | 42 | -0.754789088 | ZBTB33 | zinc finger and BTB domain containing 33 [Source:HGNC Symbol;Acc:HGNC:16682] |
| ENSG00000177697 | HGLibA_08238 | 42 | -0.754789088 | CD151 | CD151 molecule (Raph blood group) [Source:HGNC Symbol;Acc:HGNC:1630] |
| ENSG00000178078 | HGLibA_47345 | 42 | -0.754789088 | STAP2 | signal transducing adaptor family member 2 [Source:HGNC Symbol;Acc:HGNC:30430] |
| ENSG00000180785 | HGLibA_34098 | 42 | -0.754789088 | OR51E1 | olfactory receptor family 51 subfamily E member 1 [Source:HGNC Symbol;Acc:HGNC:15194] |
| ENSG00000180828 | HGLibA_04477 | 42 | -0.754789088 | BHLHE22 | basic helix-loop-helix family member e22 [Source:HGNC Symbol;Acc:HGNC:11963] |
| ENSG00000180872 | HGLibA_12849 | 42 | -0.754789088 | DEFB112 | defensin beta 112 [Source:HGNC Symbol;Acc:HGNC:18093] |
| ENSG00000180929 | HGLibA_20064 | 42 | -0.754789088 | GPR62 | G protein-coupled receptor 62 [Source:HGNC Symbol;Acc:HGNC:13301] |
| ENSG00000182747 | HGLibA_44983 | 42 | -0.754789088 | SLC35D3 | solute carrier family 35 member D3 [Source:HGNC Symbol;Acc:HGNC:15621] |
| ENSG00000185404 | HGLibA_46295 | 42 | -0.754789088 | SP140L | SP140 nuclear body protein like [Source:HGNC Symbol;Acc:HGNC:25105] |
| ENSG00000185838 | HGLibA_19487 | 42 | -0.754789088 | GNB1L | G protein subunit beta 1 like [Source:HGNC Symbol;Acc:HGNC:4397] |
| ENSG00000185888 | HGLibA_38938 | 42 | -0.754789088 | PRSS38 | serine protease 38 [Source:HGNC Symbol;Acc:HGNC:29625] |
| ENSG00000185926 | HGLibA_33959 | 42 | -0.754789088 | OR4C46 | olfactory receptor family 4 subfamily C member 46 [Source:HGNC Symbol;Acc:HGNC:31271] |
| ENSG00000186575 | HGLibA_31762 | 42 | -0.754789088 | NF2 | neurofibromin 2 [Source:HGNC Symbol;Acc:HGNC:7773] |
| ENSG00000187118 | HGLibA_10186 | 42 | -0.754789088 | CMC1 | C-X9-C motif containing 1 [Source:HGNC Symbol;Acc:HGNC:28783] |
| ENSG00000187535 | HGLibA_22746 | 42 | -0.754789088 | IFT140 | intraflagellar transport 140 [Source:HGNC Symbol;Acc:HGNC:29077] |
| ENSG00000188529 | HGLibA_47063 | 42 | -0.754789088 | SRSF10 | serine and arginine rich splicing factor 10 [Source:HGNC Symbol;Acc:HGNC:16713] |
| ENSG00000188783 | HGLibA_38435 | 42 | -0.754789088 | PRELP | proline and arginine rich end leucine rich repeat protein [Source:HGNC Symbol;Acc:HGNC:9357] |
| ENSG00000189037 | HGLibA_14161 | 42 | -0.754789088 | DUSP21 | dual specificity phosphatase 21 [Source:HGNC Symbol;Acc:HGNC:20476] |
| ENSG00000189139 | HGLibA_18066 | 42 | -0.754789088 | FSCB | fibrous sheath CABYR binding protein [Source:HGNC Symbol;Acc:HGNC:20494] |
| ENSG00000196074 | HGLibA_47932 | 42 | -0.754789088 | SYCP2 | synaptonemal complex protein 2 [Source:HGNC Symbol;Acc:HGNC:11490] |
| ENSG00000196757 | HGLibA_56576 | 42 | -0.754789088 | ZNF700 | zinc finger protein 700 [Source:HGNC Symbol;Acc:HGNC:25292] |
| ENSG00000197786 | HGLibA_34260 | 42 | -0.754789088 | OR5B17 | olfactory receptor family 5 subfamily B member 17 [Source:HGNC Symbol;Acc:HGNC:15267] |
| ENSG00000198039 | HGLibA_55707 | 42 | -0.754789088 | ZNF273 | zinc finger protein 273 [Source:HGNC Symbol;Acc:HGNC:13067] |
| ENSG00000198851 | HGLibA_08371 | 42 | -0.754789088 | CD3E | CD3e molecule [Source:HGNC Symbol;Acc:HGNC:1674] |
| ENSG00000204611 | HGLibA_56395 | 42 | -0.754789088 | ZNF616 | zinc finger protein 616 [Source:HGNC Symbol;Acc:HGNC:28062] |
| ENSG00000204671 | HGLibA_23129 | 42 | -0.754789088 | IL31 | interleukin 31 [Source:HGNC Symbol;Acc:HGNC:19372] |
| ENSG00000206052 | HGLibA_13782 | 42 | -0.754789088 | DOK6 | docking protein 6 [Source:HGNC Symbol;Acc:HGNC:28301] |
| ENSG00000206422 | HGLibA_27309 | 42 | -0.754789088 | LRRC30 | leucine rich repeat containing 30 [Source:HGNC Symbol;Acc:HGNC:30219] |
| ENSG00000213719 | HGLibA_10021 | 42 | -0.754789088 | CLIC1 | chloride intracellular channel 1 [Source:HGNC Symbol;Acc:HGNC:2062] |
| ENSG00000214753 | HGLibA_21853 | 42 | -0.754789088 | HNRNPUL2 | heterogeneous nuclear ribonucleoprotein U like 2 [Source:HGNC Symbol;Acc:HGNC:25451] |
| ENSG00000223609 | HGLibA_20916 | 42 | -0.754789088 | HBD | hemoglobin subunit delta [Source:HGNC Symbol;Acc:HGNC:4829] |
| ENSG00000227345 | HGLibA_35192 | 42 | -0.754789088 | PARG | poly(ADP-ribose) glycohydrolase [Source:HGNC Symbol;Acc:HGNC:8605] |
| ENSG00000230031 | HGLibA_37720 | 42 | -0.754789088 | POTEB2 | POTE ankyrin domain family member B2 [Source:HGNC Symbol;Acc:HGNC:48327] |
| ENSG00000235631 | HGLibA_41539 | 42 | -0.754789088 | RNF148 | ring finger protein 148 [Source:HGNC Symbol;Acc:HGNC:22411] |
| ENSG00000236032 | HGLibA_34291 | 42 | -0.754789088 | OR5H14 | olfactory receptor family 5 subfamily H member 14 [Source:HGNC Symbol;Acc:HGNC:31286] |
| ENSG00000256525 | HGLibA_37525 | 42 | -0.754789088 | POLG2 | accessory subunit [Source:HGNC Symbol;Acc:HGNC:9180]" |
| ENSG00000257218 | HGLibA_18691 | 42 | -0.754789088 | GATC | glutamyl-tRNA amidotransferase subunit C [Source:HGNC Symbol;Acc:HGNC:25068] |
| ENSG00000258429 | HGLibA_35837 | 42 | -0.754789088 | PDF | mitochondrial [Source:HGNC Symbol;Acc:HGNC:30012]" |
| ENSG00000263002 | HGLibA_55645 | 42 | -0.754789088 | ZNF234 | zinc finger protein 234 [Source:HGNC Symbol;Acc:HGNC:13027] |
| ENSG00000268940 | HGLibA_11498 | 42 | -0.754789088 | CT45A1 | cancer/testis antigen family 45 member A1 [Source:HGNC Symbol;Acc:HGNC:33267] |
| ENSG00000271447 | HGLibA_29531 | 42 | -0.754789088 | MMP28 | matrix metallopeptidase 28 [Source:HGNC Symbol;Acc:HGNC:14366] |
| ENSG00000273611 | HGLibA_56928 | 42 | -0.754789088 | ZNHIT3 | zinc finger HIT-type containing 3 [Source:HGNC Symbol;Acc:HGNC:12309] |
| ENSG00000273820 | HGLibA_53318 | 42 | -0.754789088 | USP27X | ubiquitin specific peptidase 27 X-linked [Source:HGNC Symbol;Acc:HGNC:13486] |
| ENSG00000276231 | HGLibA_36663 | 42 | -0.754789088 | PIK3R6 | phosphoinositide-3-kinase regulatory subunit 6 [Source:HGNC Symbol;Acc:HGNC:27101] |
| FAM192A | HGLibA_16359 | 42 | -0.754789088 | FAM192A | - |
| FAM63B | HGLibA_16662 | 42 | -0.754789088 | FAM63B | - |
| HEATR2 | HGLibA_21070 | 42 | -0.754789088 | HEATR2 | - |
| hsa-mir-128-2 | HGLibA_57575 | 42 | -0.754789088 | hsa-mir-128-2 | - |
| hsa-mir-143 | HGLibA_57837 | 42 | -0.754789088 | hsa-mir-143 | - |
| hsa-mir-203b | HGLibA_58169 | 42 | -0.754789088 | hsa-mir-203b | - |
| hsa-mir-216b | HGLibA_58259 | 42 | -0.754789088 | hsa-mir-216b | - |
| hsa-mir-26a-2 | HGLibA_58368 | 42 | -0.754789088 | hsa-mir-26a-2 | - |
| hsa-mir-3117 | HGLibA_58513 | 42 | -0.754789088 | hsa-mir-3117 | - |
| hsa-mir-33b | HGLibA_59015 | 42 | -0.754789088 | hsa-mir-33b | - |
| hsa-mir-3684 | HGLibA_59290 | 42 | -0.754789088 | hsa-mir-3684 | - |
| hsa-mir-371a | HGLibA_59374 | 42 | -0.754789088 | hsa-mir-371a | - |
| hsa-mir-410 | HGLibA_59680 | 42 | -0.754789088 | hsa-mir-410 | - |
| hsa-mir-425 | HGLibA_59707 | 42 | -0.754789088 | hsa-mir-425 | - |
| hsa-mir-4254 | HGLibA_59723 | 42 | -0.754789088 | hsa-mir-4254 | - |
| hsa-mir-4434 | HGLibA_60125 | 42 | -0.754789088 | hsa-mir-4434 | - |
| hsa-mir-4450 | HGLibA_60207 | 42 | -0.754789088 | hsa-mir-4450 | - |
| hsa-mir-4464 | HGLibA_60261 | 42 | -0.754789088 | hsa-mir-4464 | - |
| hsa-mir-4474 | HGLibA_60307 | 42 | -0.754789088 | hsa-mir-4474 | - |
| hsa-mir-4477a | HGLibA_60320 | 42 | -0.754789088 | hsa-mir-4477a | - |
| hsa-mir-4683 | HGLibA_60843 | 42 | -0.754789088 | hsa-mir-4683 | - |
| hsa-mir-4714 | HGLibA_60957 | 42 | -0.754789088 | hsa-mir-4714 | - |
| hsa-mir-4802 | HGLibA_61312 | 42 | -0.754789088 | hsa-mir-4802 | - |
| hsa-mir-5008 | HGLibA_61437 | 42 | -0.754789088 | hsa-mir-5008 | - |
| hsa-mir-548ap | HGLibA_61914 | 42 | -0.754789088 | hsa-mir-548ap | - |
| hsa-mir-5690 | HGLibA_62281 | 42 | -0.754789088 | hsa-mir-5690 | - |
| hsa-mir-6126 | HGLibA_62650 | 42 | -0.754789088 | hsa-mir-6126 | - |
| hsa-mir-618 | HGLibA_62713 | 42 | -0.754789088 | hsa-mir-618 | - |
| hsa-mir-628 | HGLibA_62751 | 42 | -0.754789088 | hsa-mir-628 | - |
| hsa-mir-6814 | HGLibA_63435 | 42 | -0.754789088 | hsa-mir-6814 | - |
| hsa-mir-8056 | HGLibA_64064 | 42 | -0.754789088 | hsa-mir-8056 | - |
| KIAA1456 | HGLibA_24760 | 42 | -0.754789088 | KIAA1456 | - |
| LOC100287036 | HGLibA_26815 | 42 | -0.754789088 | LOC100287036 | - |
| LOC100288255 | HGLibA_26828 | 42 | -0.754789088 | LOC100288255 | - |
| LOC100505478 | HGLibA_26845 | 42 | -0.754789088 | LOC100505478 | - |
| LOC643037 | HGLibA_26962 | 42 | -0.754789088 | LOC643037 | - |
| MNF1 | HGLibA_29569 | 42 | -0.754789088 | MNF1 | - |
| NARG2 | HGLibA_31150 | 42 | -0.754789088 | NARG2 | - |
| PHF16 | HGLibA_36350 | 42 | -0.754789088 | PHF16 | - |
| PRMT10 | HGLibA_38620 | 42 | -0.754789088 | PRMT10 | - |
| PTPN20B | HGLibA_39454 | 42 | -0.754789088 | PTPN20B | - |
| APOBEC3A_B | HGLibA_02476 | 41 | -0.763834431 | APOBEC3A_B | - |
| ATHL1 | HGLibA_03509 | 41 | -0.763834431 | ATHL1 | - |
| ATP5G3 | HGLibA_03661 | 41 | -0.763834431 | ATP5G3 | - |
| BRE | HGLibA_04793 | 41 | -0.763834431 | BRE | - |
| C19orf77 | HGLibA_05707 | 41 | -0.763834431 | C19orf77 | - |
| C3orf27 | HGLibA_06223 | 41 | -0.763834431 | C3orf27 | - |
| C9orf169 | HGLibA_06703 | 41 | -0.763834431 | C9orf169 | - |
| C9orf84 | HGLibA_06764 | 41 | -0.763834431 | C9orf84 | - |
| DAK | HGLibA_12302 | 41 | -0.763834431 | DAK | - |
| ENSG00000005889 | HGLibA_55264 | 41 | -0.763834431 | ZFX | zinc finger protein X-linked [Source:HGNC Symbol;Acc:HGNC:12869] |
| ENSG00000010292 | HGLibA_31262 | 41 | -0.763834431 | NCAPD2 | non-SMC condensin I complex subunit D2 [Source:HGNC Symbol;Acc:HGNC:24305] |
| ENSG00000019582 | HGLibA_08445 | 41 | -0.763834431 | CD74 | CD74 molecule [Source:HGNC Symbol;Acc:HGNC:1697] |
| ENSG00000053747 | HGLibA_25947 | 41 | -0.763834431 | LAMA3 | laminin subunit alpha 3 [Source:HGNC Symbol;Acc:HGNC:6483] |
| ENSG00000065243 | HGLibA_36839 | 41 | -0.763834431 | PKN2 | protein kinase N2 [Source:HGNC Symbol;Acc:HGNC:9406] |
| ENSG00000065526 | HGLibA_46620 | 41 | -0.763834431 | SPEN | spen family transcriptional repressor [Source:HGNC Symbol;Acc:HGNC:17575] |
| ENSG00000067560 | HGLibA_41166 | 41 | -0.763834431 | RHOA | ras homolog family member A [Source:HGNC Symbol;Acc:HGNC:667] |
| ENSG00000068366 | HGLibA_00561 | 41 | -0.763834431 | ACSL4 | acyl-CoA synthetase long chain family member 4 [Source:HGNC Symbol;Acc:HGNC:3571] |
| ENSG00000069275 | HGLibA_32917 | 41 | -0.763834431 | NUCKS1 | nuclear casein kinase and cyclin dependent kinase substrate 1 [Source:HGNC Symbol;Acc:HGNC:29923] |

| | | | | | |
|---|---|---|---|---|---|
| ENSG00000069869 | HGLibA_31602 | 41 | -0.763834431 | NEDD4 | E3 ubiquitin protein ligase [Source:HGNC Symbol;Acc:HGNC:7727]" |
| ENSG00000077254 | HGLibA_53339 | 41 | -0.763834431 | USP33 | ubiquitin specific peptidase 33 [Source:HGNC Symbol;Acc:HGNC:20059] |
| ENSG00000077514 | HGLibA_37498 | 41 | -0.763834431 | POLD3 | accessory subunit [Source:HGNC Symbol;Acc:HGNC:20932]" |
| ENSG00000079385 | HGLibA_08924 | 41 | -0.763834431 | CEACAM1 | carcinoembryonic antigen related cell adhesion molecule 1 [Source:HGNC Symbol;Acc:HGNC:1814] |
| ENSG00000079819 | HGLibA_15244 | 41 | -0.763834431 | EPB41L2 | erythrocyte membrane protein band 4.1 like 2 [Source:HGNC Symbol;Acc:HGNC:3379] |
| ENSG00000087077 | HGLibA_51649 | 41 | -0.763834431 | TRIP6 | thyroid hormone receptor interactor 6 [Source:HGNC Symbol;Acc:HGNC:12311] |
| ENSG00000087087 | HGLibA_47058 | 41 | -0.763834431 | SRRT | RNA effector molecule [Source:HGNC Symbol;Acc:HGNC:24101]" |
| ENSG00000089057 | HGLibA_44608 | 41 | -0.763834431 | SLC23A2 | solute carrier family 23 member 2 [Source:HGNC Symbol;Acc:HGNC:10973] |
| ENSG00000092531 | HGLibA_45875 | 41 | -0.763834431 | SNAP23 | synaptosome associated protein 23 [Source:HGNC Symbol;Acc:HGNC:11131] |
| ENSG00000096746 | HGLibA_21827 | 41 | -0.763834431 | HNRNPH3 | heterogeneous nuclear ribonucleoprotein H3 [Source:HGNC Symbol;Acc:HGNC:5043] |
| ENSG00000099901 | HGLibA_40191 | 41 | -0.763834431 | RANBP1 | RAN binding protein 1 [Source:HGNC Symbol;Acc:HGNC:9847] |
| ENSG00000099953 | HGLibA_29484 | 41 | -0.763834431 | MMP11 | matrix metallopeptidase 11 [Source:HGNC Symbol;Acc:HGNC:7157] |
| ENSG00000100146 | HGLibA_46228 | 41 | -0.763834431 | SOX10 | SRY-box 10 [Source:HGNC Symbol;Acc:HGNC:11190] |
| ENSG00000100225 | HGLibA_17127 | 41 | -0.763834431 | FBXO7 | F-box protein 7 [Source:HGNC Symbol;Acc:HGNC:13586] |
| ENSG00000100243 | HGLibA_12008 | 41 | -0.763834431 | CYB5R3 | cytochrome b5 reductase 3 [Source:HGNC Symbol;Acc:HGNC:2873] |
| ENSG00000100362 | HGLibA_39614 | 41 | -0.763834431 | PVALB | parvalbumin [Source:HGNC Symbol;Acc:HGNC:9704] |
| ENSG00000100450 | HGLibA_20724 | 41 | -0.763834431 | GZMH | granzyme H [Source:HGNC Symbol;Acc:HGNC:4710] |
| ENSG00000100811 | HGLibA_54756 | 41 | -0.763834431 | YY1 | YY1 transcription factor [Source:HGNC Symbol;Acc:HGNC:12856] |
| ENSG00000101198 | HGLibA_31999 | 41 | -0.763834431 | NKAIN4 | sodium/potassium transporting ATPase interacting 4 [Source:HGNC Symbol;Acc:HGNC:16191] |
| ENSG00000101251 | HGLibA_43280 | 41 | -0.763834431 | SEL1L2 | ERAD E3 ligase adaptor subunit [Source:HGNC Symbol;Acc:HGNC:15897]" |
| ENSG00000101280 | HGLibA_01908 | 41 | -0.763834431 | ANGPT4 | angiopoietin 4 [Source:HGNC Symbol;Acc:HGNC:487] |
| ENSG00000101441 | HGLibA_11457 | 41 | -0.763834431 | CST4 | cystatin S [Source:HGNC Symbol;Acc:HGNC:2476] |
| ENSG00000101680 | HGLibA_25942 | 41 | -0.763834431 | LAMA1 | laminin subunit alpha 1 [Source:HGNC Symbol;Acc:HGNC:6481] |
| ENSG00000102104 | HGLibA_42293 | 41 | -0.763834431 | RS1 | retinoschisin 1 [Source:HGNC Symbol;Acc:HGNC:10457] |
| ENSG00000102172 | HGLibA_45831 | 41 | -0.763834431 | SMS | spermine synthase [Source:HGNC Symbol;Acc:HGNC:11123] |
| ENSG00000102931 | HGLibA_02961 | 41 | -0.763834431 | ARL2BP | ADP ribosylation factor like GTPase 2 binding protein [Source:HGNC Symbol;Acc:HGNC:17146] |
| ENSG00000103489 | HGLibA_54619 | 41 | -0.763834431 | XYLT1 | xylosyltransferase 1 [Source:HGNC Symbol;Acc:HGNC:15516] |
| ENSG00000103932 | HGLibA_41814 | 41 | -0.763834431 | RPAP1 | RNA polymerase II associated protein 1 [Source:HGNC Symbol;Acc:HGNC:24567] |
| ENSG00000104427 | HGLibA_54937 | 41 | -0.763834431 | ZC2HC1A | zinc finger C2HC-type containing 1A [Source:HGNC Symbol;Acc:HGNC:24277] |
| ENSG00000105053 | HGLibA_53831 | 41 | -0.763834431 | VRK3 | VRK serine/threonine kinase 3 [Source:HGNC Symbol;Acc:HGNC:18996] |
| ENSG00000105072 | HGLibA_05662 | 41 | -0.763834431 | C19orf44 | chromosome 19 open reading frame 44 [Source:HGNC Symbol;Acc:HGNC:26141] |
| ENSG00000105467 | HGLibA_47999 | 41 | -0.763834431 | SYNGR4 | synaptogyrin 4 [Source:HGNC Symbol;Acc:HGNC:11502] |
| ENSG00000106236 | HGLibA_32535 | 41 | -0.763834431 | NPTX2 | neuronal pentraxin 2 [Source:HGNC Symbol;Acc:HGNC:7953] |
| ENSG00000106803 | HGLibA_43254 | 41 | -0.763834431 | SEC61B | Sec61 translocon beta subunit [Source:HGNC Symbol;Acc:HGNC:16993] |
| ENSG00000107882 | HGLibA_47713 | 41 | -0.763834431 | SUFU | SUFU negative regulator of hedgehog signaling [Source:HGNC Symbol;Acc:HGNC:16466] |
| ENSG00000109103 | HGLibA_52986 | 41 | -0.763834431 | UNC119 | unc-119 lipid binding chaperone [Source:HGNC Symbol;Acc:HGNC:12565] |
| ENSG00000109181 | HGLibA_52913 | 41 | -0.763834431 | UGT2B10 | UDP glucuronosyltransferase family 2 member B10 [Source:HGNC Symbol;Acc:HGNC:12544] |
| ENSG00000110427 | HGLibA_24781 | 41 | -0.763834431 | KIAA1549L | KIAA1549 like [Source:HGNC Symbol;Acc:HGNC:24836] |
| ENSG00000110435 | HGLibA_35872 | 41 | -0.763834431 | PDHX | pyruvate dehydrogenase complex component X [Source:HGNC Symbol;Acc:HGNC:21350] |
| ENSG00000110880 | HGLibA_10780 | 41 | -0.763834431 | CORO1C | coronin 1C [Source:HGNC Symbol;Acc:HGNC:2254] |
| ENSG00000111596 | HGLibA_10335 | 41 | -0.763834431 | CNOT2 | CCR4-NOT transcription complex subunit 2 [Source:HGNC Symbol;Acc:HGNC:7878] |
| ENSG00000112305 | HGLibA_45597 | 41 | -0.763834431 | SMAP1 | small ArfGAP 1 [Source:HGNC Symbol;Acc:HGNC:19651] |
| ENSG00000113196 | HGLibA_20827 | 41 | -0.763834431 | HAND1 | heart and neural crest derivatives expressed 1 [Source:HGNC Symbol;Acc:HGNC:4807] |
| ENSG00000114861 | HGLibA_17930 | 41 | -0.763834431 | FOXP1 | forkhead box P1 [Source:HGNC Symbol;Acc:HGNC:3823] |
| ENSG00000115339 | HGLibA_18556 | 41 | -0.763834431 | GALNT3 | polypeptide N-acetylgalactosaminyltransferase 3 [Source:HGNC Symbol;Acc:HGNC:4125] |
| ENSG00000115484 | HGLibA_08201 | 41 | -0.763834431 | CCT4 | chaperonin containing TCP1 subunit 4 [Source:HGNC Symbol;Acc:HGNC:1617] |
| ENSG00000115539 | HGLibA_35755 | 41 | -0.763834431 | PDCL3 | phosducin like 3 [Source:HGNC Symbol;Acc:HGNC:28860] |
| ENSG00000115956 | HGLibA_37055 | 41 | -0.763834431 | PLEK | pleckstrin [Source:HGNC Symbol;Acc:HGNC:9070] |
| ENSG00000116903 | HGLibA_15763 | 41 | -0.763834431 | EXOC8 | exocyst complex component 8 [Source:HGNC Symbol;Acc:HGNC:24659] |
| ENSG00000117594 | HGLibA_22161 | 41 | -0.763834431 | HSD11B1 | hydroxysteroid 11-beta dehydrogenase 1 [Source:HGNC Symbol;Acc:HGNC:5208] |
| ENSG00000117691 | HGLibA_31691 | 41 | -0.763834431 | NENF | neudesin neurotrophic factor [Source:HGNC Symbol;Acc:HGNC:30384] |
| ENSG00000121775 | HGLibA_50388 | 41 | -0.763834431 | TMEM39B | transmembrane protein 39B [Source:HGNC Symbol;Acc:HGNC:25510] |
| ENSG00000122756 | HGLibA_10395 | 41 | -0.763834431 | CNTFR | ciliary neurotrophic factor receptor [Source:HGNC Symbol;Acc:HGNC:2170] |
| ENSG00000124233 | HGLibA_43378 | 41 | -0.763834431 | SEMG1 | semenogelin 1 [Source:HGNC Symbol;Acc:HGNC:10742] |
| ENSG00000124243 | HGLibA_04261 | 41 | -0.763834431 | BCAS4 | breast carcinoma amplified sequence 4 [Source:HGNC Symbol;Acc:HGNC:14367] |
| ENSG00000125459 | HGLibA_30269 | 41 | -0.763834431 | MSTO1 | mitochondrial distribution and morphology regulator [Source:HGNC Symbol;Acc:HGNC:29678]" |
| ENSG00000125730 | HGLibA_06203 | 41 | -0.763834431 | C3 | complement C3 [Source:HGNC Symbol;Acc:HGNC:1318] |
| ENSG00000125861 | HGLibA_18974 | 41 | -0.763834431 | GFRA4 | GDNF family receptor alpha 4 [Source:HGNC Symbol;Acc:HGNC:13821] |
| ENSG00000126249 | HGLibA_35730 | 41 | -0.763834431 | PDCD2L | programmed cell death 2 like [Source:HGNC Symbol;Acc:HGNC:28194] |
| ENSG00000126778 | HGLibA_44243 | 41 | -0.763834431 | SIX1 | SIX homeobox 1 [Source:HGNC Symbol;Acc:HGNC:10887] |
| ENSG00000130755 | HGLibA_19405 | 41 | -0.763834431 | GMFG | glia maturation factor gamma [Source:HGNC Symbol;Acc:HGNC:4374] |
| ENSG00000131183 | HGLibA_44938 | 41 | -0.763834431 | SLC34A1 | solute carrier family 34 member 1 [Source:HGNC Symbol;Acc:HGNC:11019] |
| ENSG00000132855 | HGLibA_01915 | 41 | -0.763834431 | ANGPTL3 | angiopoietin like 3 [Source:HGNC Symbol;Acc:HGNC:491] |
| ENSG00000133874 | HGLibA_41487 | 41 | -0.763834431 | RNF122 | ring finger protein 122 [Source:HGNC Symbol;Acc:HGNC:21147] |
| ENSG00000134285 | HGLibA_17556 | 41 | -0.763834431 | FKBP11 | FK506 binding protein 11 [Source:HGNC Symbol;Acc:HGNC:18624] |
| ENSG00000136682 | HGLibA_07432 | 41 | -0.763834431 | CBWD2 | COBW domain containing 2 [Source:HGNC Symbol;Acc:HGNC:17907] |
| ENSG00000136828 | HGLibA_40167 | 41 | -0.763834431 | RALGPS1 | Ral GEF with PH domain and SH3 binding motif 1 [Source:HGNC Symbol;Acc:HGNC:16851] |
| ENSG00000137124 | HGLibA_01550 | 41 | -0.763834431 | ALDH1B1 | aldehyde dehydrogenase 1 family member B1 [Source:HGNC Symbol;Acc:HGNC:407] |
| ENSG00000137337 | HGLibA_28668 | 41 | -0.763834431 | MDC1 | mediator of DNA damage checkpoint 1 [Source:HGNC Symbol;Acc:HGNC:21163] |
| ENSG00000137409 | HGLibA_30324 | 41 | -0.763834431 | MTCH1 | mitochondrial carrier 1 [Source:HGNC Symbol;Acc:HGNC:17586] |
| ENSG00000137500 | HGLibA_07922 | 41 | -0.763834431 | CCDC90B | coiled-coil domain containing 90B [Source:HGNC Symbol;Acc:HGNC:28108] |
| ENSG00000139546 | HGLibA_48333 | 41 | -0.763834431 | TARBP2 | RISC loading complex RNA binding subunit [Source:HGNC Symbol;Acc:HGNC:11569]" |
| ENSG00000139990 | HGLibA_12448 | 41 | -0.763834431 | DCAF5 | DDB1 and CUL4 associated factor 5 [Source:HGNC Symbol;Acc:HGNC:20224] |
| ENSG00000140463 | HGLibA_04218 | 41 | -0.763834431 | BBS4 | Bardet-Biedl syndrome 4 [Source:HGNC Symbol;Acc:HGNC:969] |
| ENSG00000140470 | HGLibA_00812 | 41 | -0.763834431 | ADAMTS17 | ADAM metallopeptidase with thrombospondin type 1 motif 17 [Source:HGNC Symbol;Acc:HGNC:17109] |
| ENSG00000143093 | HGLibA_47588 | 41 | -0.763834431 | STRIP1 | striatin interacting protein 1 [Source:HGNC Symbol;Acc:HGNC:25916] |
| ENSG00000143921 | HGLibA_00227 | 41 | -0.763834431 | ABCG8 | ATP binding cassette subfamily G member 8 [Source:HGNC Symbol;Acc:HGNC:13887] |
| ENSG00000144635 | HGLibA_14242 | 41 | -0.763834431 | DYNC1LI1 | dynein cytoplasmic 1 light intermediate chain 1 [Source:HGNC Symbol;Acc:HGNC:18745] |
| ENSG00000144645 | HGLibA_34644 | 41 | -0.763834431 | OSBPL10 | oxysterol binding protein like 10 [Source:HGNC Symbol;Acc:HGNC:16395] |
| ENSG00000145879 | HGLibA_46703 | 41 | -0.763834431 | SPINK7 | Kazal type 7 (putative) [Source:HGNC Symbol;Acc:HGNC:24643]" |
| ENSG00000146530 | HGLibA_53933 | 41 | -0.763834431 | VWDE | von Willebrand factor D and EGF domains [Source:HGNC Symbol;Acc:HGNC:21897] |
| ENSG00000147246 | HGLibA_22378 | 41 | -0.763834431 | HTR2C | 5-hydroxytryptamine receptor 2C [Source:HGNC Symbol;Acc:HGNC:5295] |
| ENSG00000147854 | HGLibA_52955 | 41 | -0.763834431 | UHRF2 | ubiquitin like with PHD and ring finger domains 2 [Source:HGNC Symbol;Acc:HGNC:12557] |
| ENSG00000148488 | HGLibA_47304 | 41 | -0.763834431 | ST8SIA6 | 8-sialyltransferase 6 [Source:HGNC Symbol;Acc:HGNC:23317]" |
| ENSG00000148832 | HGLibA_35117 | 41 | -0.763834431 | PAOX | polyamine oxidase [Source:HGNC Symbol;Acc:HGNC:20837] |
| ENSG00000150961 | HGLibA_43238 | 41 | -0.763834431 | SEC24D | COPII coat complex component [Source:HGNC Symbol;Acc:HGNC:10706]" |
| ENSG00000151023 | HGLibA_15138 | 41 | -0.763834431 | ENKUR | TRPC channel interacting protein [Source:HGNC Symbol;Acc:HGNC:28388]" |
| ENSG00000151360 | HGLibA_01670 | 41 | -0.763834431 | ALLC | allantoicase [Source:HGNC Symbol;Acc:HGNC:17377] |
| ENSG00000151474 | HGLibA_18014 | 41 | -0.763834431 | FRMD4A | FERM domain containing 4A [Source:HGNC Symbol;Acc:HGNC:25491] |
| ENSG00000151690 | HGLibA_29109 | 41 | -0.763834431 | MFSD6 | major facilitator superfamily domain containing 6 [Source:HGNC Symbol;Acc:HGNC:24711] |
| ENSG00000153563 | HGLibA_08472 | 41 | -0.763834431 | CD8A | CD8a molecule [Source:HGNC Symbol;Acc:HGNC:1706] |
| ENSG00000154485 | HGLibA_29512 | 41 | -0.763834431 | MMP21 | matrix metallopeptidase 21 [Source:HGNC Symbol;Acc:HGNC:14357] |
| ENSG00000154654 | HGLibA_31256 | 41 | -0.763834431 | NCAM2 | neural cell adhesion molecule 2 [Source:HGNC Symbol;Acc:HGNC:7657] |
| ENSG00000154727 | HGLibA_18347 | 41 | -0.763834431 | GABPA | GA binding protein transcription factor subunit alpha [Source:HGNC Symbol;Acc:HGNC:4071] |
| ENSG00000155256 | HGLibA_55287 | 41 | -0.763834431 | ZFYVE27 | zinc finger FYVE-type containing 27 [Source:HGNC Symbol;Acc:HGNC:26559] |
| ENSG00000155886 | HGLibA_44618 | 41 | -0.763834431 | SLC24A2 | solute carrier family 24 member 2 [Source:HGNC Symbol;Acc:HGNC:10976] |
| ENSG00000156222 | HGLibA_44832 | 41 | -0.763834431 | SLC28A1 | solute carrier family 28 member 1 [Source:HGNC Symbol;Acc:HGNC:11001] |
| ENSG00000156282 | HGLibA_09867 | 41 | -0.763834431 | CLDN17 | claudin 17 [Source:HGNC Symbol;Acc:HGNC:2038] |
| ENSG00000158270 | HGLibA_10638 | 41 | -0.763834431 | COLEC12 | collectin subfamily member 12 [Source:HGNC Symbol;Acc:HGNC:16016] |
| ENSG00000158769 | HGLibA_15847 | 41 | -0.763834431 | F11R | F11 receptor [Source:HGNC Symbol;Acc:HGNC:14685] |
| ENSG00000159082 | HGLibA_48002 | 41 | -0.763834431 | SYNJ1 | synaptojanin 1 [Source:HGNC Symbol;Acc:HGNC:11503] |
| ENSG00000159248 | HGLibA_19188 | 41 | -0.763834431 | GJD2 | gap junction protein delta 2 [Source:HGNC Symbol;Acc:HGNC:19154] |
| ENSG00000159461 | HGLibA_01780 | 41 | -0.763834431 | AMFR | autocrine motility factor receptor [Source:HGNC Symbol;Acc:HGNC:463] |
| ENSG00000161911 | HGLibA_51359 | 41 | -0.763834431 | TREML1 | triggering receptor expressed on myeloid cells like 1 [Source:HGNC Symbol;Acc:HGNC:20434] |
| ENSG00000162924 | HGLibA_40809 | 41 | -0.763834431 | REL | NF-kB subunit [Source:HGNC Symbol;Acc:HGNC:9954]" |
| ENSG00000162959 | HGLibA_28882 | 41 | -0.763834431 | MEMO1 | mediator of cell motility 1 [Source:HGNC Symbol;Acc:HGNC:14014] |
| ENSG00000163092 | HGLibA_54513 | 41 | -0.763834431 | XIRP2 | xin actin binding repeat containing 2 [Source:HGNC Symbol;Acc:HGNC:14303] |
| ENSG00000163466 | HGLibA_03080 | 41 | -0.763834431 | ARPC2 | actin related protein 2/3 complex subunit 2 [Source:HGNC Symbol;Acc:HGNC:705] |
| ENSG00000163467 | HGLibA_51807 | 41 | -0.763834431 | TSACC | TSSK6 activating cochaperone [Source:HGNC Symbol;Acc:HGNC:30636] |
| ENSG00000163867 | HGLibA_55404 | 41 | -0.763834431 | ZMYM6 | zinc finger MYM-type containing 6 [Source:HGNC Symbol;Acc:HGNC:13050] |
| ENSG00000164346 | HGLibA_32741 | 41 | -0.763834431 | NSA2 | ribosome biogenesis homolog [Source:HGNC Symbol;Acc:HGNC:30728]" |
| ENSG00000165097 | HGLibA_24490 | 41 | -0.763834431 | KDM1B | lysine demethylase 1B [Source:HGNC Symbol;Acc:HGNC:21577] |
| ENSG00000165192 | HGLibA_03208 | 41 | -0.763834431 | ASB11 | ankyrin repeat and SOCS box containing 11 [Source:HGNC Symbol;Acc:HGNC:17186] |
| ENSG00000165215 | HGLibA_09896 | 41 | -0.763834431 | CLDN3 | claudin 3 [Source:HGNC Symbol;Acc:HGNC:2045] |
| ENSG00000165819 | HGLibA_29005 | 41 | -0.763834431 | METTL3 | methyltransferase like 3 [Source:HGNC Symbol;Acc:HGNC:17563] |
| ENSG00000165821 | HGLibA_42650 | 41 | -0.763834431 | SALL2 | spalt like transcription factor 2 [Source:HGNC Symbol;Acc:HGNC:10526] |
| ENSG00000165973 | HGLibA_31683 | 41 | -0.763834431 | NELL1 | neural EGFL like 1 [Source:HGNC Symbol;Acc:HGNC:7750] |
| ENSG00000166145 | HGLibA_46712 | 41 | -0.763834431 | SPINT1 | Kunitz type 1 [Source:HGNC Symbol;Acc:HGNC:11246]" |
| ENSG00000166845 | HGLibA_05619 | 41 | -0.763834431 | C18orf54 | chromosome 18 open reading frame 54 [Source:HGNC Symbol;Acc:HGNC:13796] |
| ENSG00000166884 | HGLibA_33981 | 41 | -0.763834431 | OR4D6 | olfactory receptor family 4 subfamily D member 6 [Source:HGNC Symbol;Acc:HGNC:15175] |
| ENSG00000167642 | HGLibA_46714 | 41 | -0.763834431 | SPINT2 | Kunitz type 2 [Source:HGNC Symbol;Acc:HGNC:11247]" |
| ENSG00000168904 | HGLibA_27299 | 41 | -0.763834431 | LRRC28 | leucine rich repeat containing 28 [Source:HGNC Symbol;Acc:HGNC:28355] |
| ENSG00000170807 | HGLibA_26721 | 41 | -0.763834431 | LMOD2 | leiomodin 2 [Source:HGNC Symbol;Acc:HGNC:6648] |
| ENSG00000170892 | HGLibA_51834 | 41 | -0.763834431 | TSEN34 | tRNA splicing endonuclease subunit 34 [Source:HGNC Symbol;Acc:HGNC:15506] |
| ENSG00000171017 | HGLibA_27440 | 41 | -0.763834431 | LRRC8E | leucine rich repeat containing 8 VRAC subunit E [Source:HGNC Symbol;Acc:HGNC:26272] |
| ENSG00000171105 | HGLibA_23399 | 41 | -0.763834431 | INSR | insulin receptor [Source:HGNC Symbol;Acc:HGNC:6091] |
| ENSG00000171180 | HGLibA_33839 | 41 | -0.763834431 | OR2M4 | olfactory receptor family 2 subfamily M member 4 [Source:HGNC Symbol;Acc:HGNC:8270] |
| ENSG00000171817 | HGLibA_56187 | 41 | -0.763834431 | ZNF540 | zinc finger protein 540 [Source:HGNC Symbol;Acc:HGNC:25331] |
| ENSG00000171988 | HGLibA_23951 | 41 | -0.763834431 | JMJD1C | jumonji domain containing 1C [Source:HGNC Symbol;Acc:HGNC:12313] |
| ENSG00000172057 | HGLibA_34632 | 41 | -0.763834431 | ORMDL3 | ORMDL sphingolipid biosynthesis regulator 3 [Source:HGNC Symbol;Acc:HGNC:16038] |
| ENSG00000172172 | HGLibA_29900 | 41 | -0.763834431 | MRPL13 | mitochondrial ribosomal protein L13 [Source:HGNC Symbol;Acc:HGNC:14278] |
| ENSG00000172336 | HGLibA_37695 | 41 | -0.763834431 | POP7 | ribonuclease P/MRP subunit [Source:HGNC Symbol;Acc:HGNC:19949]" |
| ENSG00000172425 | HGLibA_52122 | 41 | -0.763834431 | TTC36 | tetratricopeptide repeat domain 36 [Source:HGNC Symbol;Acc:HGNC:33708] |
| ENSG00000173372 | HGLibA_05913 | 41 | -0.763834431 | C1QA | complement C1q A chain [Source:HGNC Symbol;Acc:HGNC:1241] |
| ENSG00000173960 | HGLibA_52782 | 41 | -0.763834431 | UBXN2A | UBX domain protein 2A [Source:HGNC Symbol;Acc:HGNC:27265] |

| | | | | | |
|---|---|---|---|---|---|
| ENSG00000174327 | HGLibA_44429 | 41 | -0.763834431 | SLC16A13 | solute carrier family 16 member 13 [Source:HGNC Symbol;Acc:HGNC:31037] |
| ENSG00000174914 | HGLibA_34569 | 41 | -0.763834431 | OR9G1 | olfactory receptor family 9 subfamily G member 1 [Source:HGNC Symbol;Acc:HGNC:15319] |
| ENSG00000175115 | HGLibA_34955 | 41 | -0.763834431 | PACS1 | phosphofurin acidic cluster sorting protein 1 [Source:HGNC Symbol;Acc:HGNC:30032] |
| ENSG00000175550 | HGLibA_13950 | 41 | -0.763834431 | DRAP1 | DR1 associated protein 1 [Source:HGNC Symbol;Acc:HGNC:3019] |
| ENSG00000175573 | HGLibA_05171 | 41 | -0.763834431 | C11orf68 | chromosome 11 open reading frame 68 [Source:HGNC Symbol;Acc:HGNC:28801] |
| ENSG00000176165 | HGLibA_17871 | 41 | -0.763834431 | FOXG1 | forkhead box G1 [Source:HGNC Symbol;Acc:HGNC:3811] |
| ENSG00000176407 | HGLibA_24121 | 41 | -0.763834431 | KCMF1 | potassium channel modulatory factor 1 [Source:HGNC Symbol;Acc:HGNC:20589] |
| ENSG00000176658 | HGLibA_30829 | 41 | -0.763834431 | MYO1D | myosin ID [Source:HGNC Symbol;Acc:HGNC:7598] |
| ENSG00000178074 | HGLibA_06167 | 41 | -0.763834431 | C2orf69 | chromosome 2 open reading frame 69 [Source:HGNC Symbol;Acc:HGNC:26799] |
| ENSG00000178896 | HGLibA_15782 | 41 | -0.763834431 | EXOSC4 | exosome component 4 [Source:HGNC Symbol;Acc:HGNC:18189] |
| ENSG00000179134 | HGLibA_42687 | 41 | -0.763834431 | SAMD4B | sterile alpha motif domain containing 4B [Source:HGNC Symbol;Acc:HGNC:25492] |
| ENSG00000179630 | HGLibA_25909 | 41 | -0.763834431 | LACC1 | laccase domain containing 1 [Source:HGNC Symbol;Acc:HGNC:26789] |
| ENSG00000180071 | HGLibA_02018 | 41 | -0.763834431 | ANKRD18A | ankyrin repeat domain 18A [Source:HGNC Symbol;Acc:HGNC:23643] |
| ENSG00000182070 | HGLibA_34140 | 41 | -0.763834431 | OR52A1 | olfactory receptor family 52 subfamily A member 1 [Source:HGNC Symbol;Acc:HGNC:8318] |
| ENSG00000182077 | HGLibA_39281 | 41 | -0.763834431 | PTCHD3 | patched domain containing 3 [Source:HGNC Symbol;Acc:HGNC:24776] |
| ENSG00000182108 | HGLibA_13016 | 41 | -0.763834431 | DEXI | Dexi homolog [Source:HGNC Symbol;Acc:HGNC:13267] |
| ENSG00000182315 | HGLibA_28479 | 41 | -0.763834431 | MBD3L3 | methyl-CpG binding domain protein 3 like 3 [Source:HGNC Symbol;Acc:HGNC:37205] |
| ENSG00000182379 | HGLibA_33170 | 41 | -0.763834431 | NXPH4 | neurexophilin 4 [Source:HGNC Symbol;Acc:HGNC:8078] |
| ENSG00000183578 | HGLibA_50729 | 41 | -0.763834431 | TNFAIP8L3 | TNF alpha induced protein 8 like 3 [Source:HGNC Symbol;Acc:HGNC:20620] |
| ENSG00000183918 | HGLibA_43883 | 41 | -0.763834431 | SH2D1A | SH2 domain containing 1A [Source:HGNC Symbol;Acc:HGNC:10820] |
| ENSG00000184209 | HGLibA_45951 | 41 | -0.763834431 | SNRNP35 | small nuclear ribonucleoprotein U11/U12 subunit 35 [Source:HGNC Symbol;Acc:HGNC:30852] |
| ENSG00000184502 | HGLibA_18662 | 41 | -0.763834431 | GAST | gastrin [Source:HGNC Symbol;Acc:HGNC:4164] |
| ENSG00000184672 | HGLibA_40177 | 41 | -0.763834431 | RALYL | RALY RNA binding protein like [Source:HGNC Symbol;Acc:HGNC:27036] |
| ENSG00000185386 | HGLibA_28254 | 41 | -0.763834431 | MAPK11 | mitogen-activated protein kinase 11 [Source:HGNC Symbol;Acc:HGNC:6873] |
| ENSG00000185760 | HGLibA_24379 | 41 | -0.763834431 | KCNQ5 | potassium voltage-gated channel subfamily Q member 5 [Source:HGNC Symbol;Acc:HGNC:6299] |
| ENSG00000186628 | HGLibA_18084 | 41 | -0.763834431 | FSD2 | fibronectin type III and SPRY domain containing 2 [Source:HGNC Symbol;Acc:HGNC:18024] |
| ENSG00000186867 | HGLibA_39740 | 41 | -0.763834431 | QRFPR | pyroglutamylated RFamide peptide receptor [Source:HGNC Symbol;Acc:HGNC:15565] |
| ENSG00000186891 | HGLibA_50767 | 41 | -0.763834431 | TNFRSF18 | TNF receptor superfamily member 18 [Source:HGNC Symbol;Acc:HGNC:11914] |
| ENSG00000187815 | HGLibA_55230 | 41 | -0.763834431 | ZFP69 | ZFP69 zinc finger protein [Source:HGNC Symbol;Acc:HGNC:24708] |
| ENSG00000188672 | HGLibA_41145 | 41 | -0.763834431 | RHCE | Rh blood group CcEe antigens [Source:HGNC Symbol;Acc:HGNC:10008] |
| ENSG00000188710 | HGLibA_39737 | 41 | -0.763834431 | QRFP | pyroglutamylated RFamide peptide [Source:HGNC Symbol;Acc:HGNC:29982] |
| ENSG00000196092 | HGLibA_35306 | 41 | -0.763834431 | PAX5 | paired box 5 [Source:HGNC Symbol;Acc:HGNC:8619] |
| ENSG00000196136 | HGLibA_43518 | 41 | -0.763834431 | SERPINA3 | serpin family A member 3 [Source:HGNC Symbol;Acc:HGNC:16] |
| ENSG00000196284 | HGLibA_47813 | 41 | -0.763834431 | SUPT3H | SAGA and STAGA complex component [Source:HGNC Symbol;Acc:HGNC:11466]" |
| ENSG00000196600 | HGLibA_44578 | 41 | -0.763834431 | SLC22A25 | solute carrier family 22 member 25 [Source:HGNC Symbol;Acc:HGNC:32935] |
| ENSG00000197150 | HGLibA_00152 | 41 | -0.763834431 | ABCB8 | ATP binding cassette subfamily B member 8 [Source:HGNC Symbol;Acc:HGNC:49] |
| ENSG00000198205 | HGLibA_57074 | 41 | -0.763834431 | ZXDA | zinc finger X-linked duplicated A [Source:HGNC Symbol;Acc:HGNC:13198] |
| ENSG00000198342 | HGLibA_56008 | 41 | -0.763834431 | ZNF442 | zinc finger protein 442 [Source:HGNC Symbol;Acc:HGNC:20877] |
| ENSG00000203730 | HGLibA_48958 | 41 | -0.763834431 | TEDDM1 | transmembrane epididymal protein 1 [Source:HGNC Symbol;Acc:HGNC:30233] |
| ENSG00000204149 | HGLibA_01155 | 41 | -0.763834431 | AGAP6 | ankyrin repeat and PH domain 6 [Source:HGNC Symbol;Acc:HGNC:23466]" |
| ENSG00000206104 | HGLibA_25704 | 41 | -0.763834431 | KRTAP20-3 | keratin associated protein 20-3 [Source:HGNC Symbol;Acc:HGNC:34001] |
| ENSG00000213160 | HGLibA_25158 | 41 | -0.763834431 | KLHL23 | kelch like family member 23 [Source:HGNC Symbol;Acc:HGNC:27506] |
| ENSG00000213199 | HGLibA_03305 | 41 | -0.763834431 | ASIC3 | acid sensing ion channel subunit 3 [Source:HGNC Symbol;Acc:HGNC:101] |
| ENSG00000213638 | HGLibA_00885 | 41 | -0.763834431 | ADAT3 | tRNA specific 3 [Source:HGNC Symbol;Acc:HGNC:25151]" |
| ENSG00000214026 | HGLibA_29934 | 41 | -0.763834431 | MRPL23 | mitochondrial ribosomal protein L23 [Source:HGNC Symbol;Acc:HGNC:10322] |
| ENSG00000214435 | HGLibA_03183 | 41 | -0.763834431 | AS3MT | arsenite methyltransferase [Source:HGNC Symbol;Acc:HGNC:17452] |
| ENSG00000215375 | HGLibA_30767 | 41 | -0.763834431 | MYL5 | myosin light chain 5 [Source:HGNC Symbol;Acc:HGNC:7586] |
| ENSG00000215475 | HGLibA_44112 | 41 | -0.763834431 | SIAH3 | siah E3 ubiquitin protein ligase family member 3 [Source:HGNC Symbol;Acc:HGNC:30553] |
| ENSG00000215853 | HGLibA_42199 | 41 | -0.763834431 | RPTN | repetin [Source:HGNC Symbol;Acc:HGNC:26809] |
| ENSG00000219607 | HGLibA_38116 | 41 | -0.763834431 | PPP1R3G | protein phosphatase 1 regulatory subunit 3G [Source:HGNC Symbol;Acc:HGNC:14945] |
| ENSG00000221882 | HGLibA_33917 | 41 | -0.763834431 | OR3A2 | olfactory receptor family 3 subfamily A member 2 [Source:HGNC Symbol;Acc:HGNC:8283] |
| ENSG00000222028 | HGLibA_39099 | 41 | -0.763834431 | PSMB11 | proteasome subunit beta 11 [Source:HGNC Symbol;Acc:HGNC:31963] |
| ENSG00000228836 | HGLibA_11511 | 41 | -0.763834431 | CT45A5 | cancer/testis antigen family 45 member A5 [Source:HGNC Symbol;Acc:HGNC:33270] |
| ENSG00000233041 | HGLibA_36397 | 41 | -0.763834431 | PHGR1 | histidine and glycine rich 1 [Source:HGNC Symbol;Acc:HGNC:37226]" |
| ENSG00000233927 | HGLibA_42130 | 41 | -0.763834431 | RPS28 | ribosomal protein S28 [Source:HGNC Symbol;Acc:HGNC:10418] |
| ENSG00000234511 | HGLibA_06412 | 41 | -0.763834431 | C5orf58 | chromosome 5 open reading frame 58 [Source:HGNC Symbol;Acc:HGNC:37272] |
| ENSG00000241258 | HGLibA_11082 | 41 | -0.763834431 | CRCP | CGRP receptor component [Source:HGNC Symbol;Acc:HGNC:17888] |
| ENSG00000243073 | HGLibA_38317 | 41 | -0.763834431 | PRAMEF4 | PRAME family member 4 [Source:HGNC Symbol;Acc:HGNC:31971] |
| ENSG00000243156 | HGLibA_29208 | 41 | -0.763834431 | MICAL3 | calponin and LIM domain containing 3 [Source:HGNC Symbol;Acc:HGNC:24694]" |
| ENSG00000243414 | HGLibA_49447 | 41 | -0.763834431 | TICAM2 | toll like receptor adaptor molecule 2 [Source:HGNC Symbol;Acc:HGNC:21354] |
| ENSG00000249459 | HGLibA_55753 | 41 | -0.763834431 | ZNF286B | zinc finger protein 286B [Source:HGNC Symbol;Acc:HGNC:33241] |
| ENSG00000249915 | HGLibA_35740 | 41 | -0.763834431 | PDCD6 | programmed cell death 6 [Source:HGNC Symbol;Acc:HGNC:8765] |
| ENSG00000255587 | HGLibA_39951 | 41 | -0.763834431 | RAB44 | member RAS oncogene family [Source:HGNC Symbol;Acc:HGNC:21068]" |
| ENSG00000255819 | HGLibA_25298 | 41 | -0.763834431 | KLRC4-KLRK1 | KLRC4-KLRK1 readthrough [Source:HGNC Symbol;Acc:HGNC:48357] |
| ENSG00000267631 | HGLibA_09303 | 41 | -0.763834431 | CGB1 | chorionic gonadotropin subunit beta 1 [Source:HGNC Symbol;Acc:HGNC:16721] |
| ERBB2IP | HGLibA_15416 | 41 | -0.763834431 | ERBB2IP | - |
| FAM57B | HGLibA_16648 | 41 | -0.763834431 | FAM57B | - |
| GPR112 | HGLibA_19852 | 41 | -0.763834431 | GPR112 | - |
| HNRPLL | HGLibA_21859 | 41 | -0.763834431 | HNRPLL | - |
| hsa-mir-1-1 | HGLibA_57141 | 41 | -0.763834431 | hsa-mir-1-1 | - |
| hsa-mir-1255b-2 | HGLibA_57427 | 41 | -0.763834431 | hsa-mir-1255b-2 | - |
| hsa-mir-129-2 | HGLibA_57632 | 41 | -0.763834431 | hsa-mir-129-2 | - |
| hsa-mir-1302-8 | HGLibA_57718 | 41 | -0.763834431 | hsa-mir-1302-8 | - |
| hsa-mir-135a-2 | HGLibA_57796 | 41 | -0.763834431 | hsa-mir-135a-2 | - |
| hsa-mir-146a | HGLibA_57855 | 41 | -0.763834431 | hsa-mir-146a | - |
| hsa-mir-200c | HGLibA_58160 | 41 | -0.763834431 | hsa-mir-200c | - |
| hsa-mir-302c | HGLibA_58445 | 41 | -0.763834431 | hsa-mir-302c | - |
| hsa-mir-30d | HGLibA_58486 | 41 | -0.763834431 | hsa-mir-30d | - |
| hsa-mir-3124 | HGLibA_58547 | 41 | -0.763834431 | hsa-mir-3124 | - |
| hsa-mir-3189 | HGLibA_58853 | 41 | -0.763834431 | hsa-mir-3189 | - |
| hsa-mir-3692 | HGLibA_59356 | 41 | -0.763834431 | hsa-mir-3692 | - |
| hsa-mir-3919 | HGLibA_59554 | 41 | -0.763834431 | hsa-mir-3919 | - |
| hsa-mir-4319 | HGLibA_59994 | 41 | -0.763834431 | hsa-mir-4319 | - |
| hsa-mir-4324 | HGLibA_60019 | 41 | -0.763834431 | hsa-mir-4324 | - |
| hsa-mir-4477b | HGLibA_60324 | 41 | -0.763834431 | hsa-mir-4477b | - |
| hsa-mir-449a | HGLibA_60415 | 41 | -0.763834431 | hsa-mir-449a | - |
| hsa-mir-4638 | HGLibA_60656 | 41 | -0.763834431 | hsa-mir-4638 | - |
| hsa-mir-4730 | HGLibA_61016 | 41 | -0.763834431 | hsa-mir-4730 | - |
| hsa-mir-4744 | HGLibA_61075 | 41 | -0.763834431 | hsa-mir-4744 | - |
| hsa-mir-491 | HGLibA_61366 | 41 | -0.763834431 | hsa-mir-491 | - |
| hsa-mir-4999 | HGLibA_61398 | 41 | -0.763834431 | hsa-mir-4999 | - |
| hsa-mir-5003 | HGLibA_61420 | 41 | -0.763834431 | hsa-mir-5003 | - |
| hsa-mir-512-1 | HGLibA_61562 | 41 | -0.763834431 | hsa-mir-512-1 | - |
| hsa-mir-516b-2 | HGLibA_61620 | 41 | -0.763834431 | hsa-mir-516b-2 | - |
| hsa-mir-539 | HGLibA_61810 | 41 | -0.763834431 | hsa-mir-539 | - |
| hsa-mir-548al | HGLibA_61899 | 41 | -0.763834431 | hsa-mir-548al | - |
| hsa-mir-548at | HGLibA_61926 | 41 | -0.763834431 | hsa-mir-548at | - |
| hsa-mir-5701-2 | HGLibA_62346 | 41 | -0.763834431 | hsa-mir-5701-2 | - |
| hsa-mir-576 | HGLibA_62398 | 41 | -0.763834431 | hsa-mir-576 | - |
| hsa-mir-639 | HGLibA_62794 | 41 | -0.763834431 | hsa-mir-639 | - |
| hsa-mir-656 | HGLibA_62958 | 41 | -0.763834431 | hsa-mir-656 | - |
| hsa-mir-6737 | HGLibA_63102 | 41 | -0.763834431 | hsa-mir-6737 | - |
| hsa-mir-6746 | HGLibA_63136 | 41 | -0.763834431 | hsa-mir-6746 | - |
| hsa-mir-6862-1 | HGLibA_63627 | 41 | -0.763834431 | hsa-mir-6862-1 | - |
| hsa-mir-7108 | HGLibA_63791 | 41 | -0.763834431 | hsa-mir-7108 | - |
| hsa-mir-7848 | HGLibA_63983 | 41 | -0.763834431 | hsa-mir-7848 | - |
| LOC100130539 | HGLibA_26791 | 41 | -0.763834431 | LOC100130539 | - |
| LOC643355 | HGLibA_26965 | 41 | -0.763834431 | LOC643355 | - |
| SRCRB4D | HGLibA_46938 | 41 | -0.763834431 | SRCRB4D | - |
| C10orf10 | HGLibA_05018 | 40 | -0.772879774 | C10orf10 | - |
| C16orf97 | HGLibA_05514 | 40 | -0.772879774 | C16orf97 | - |
| C9orf114 | HGLibA_06662 | 40 | -0.772879774 | C9orf114 | - |
| CCDC37 | HGLibA_07765 | 40 | -0.772879774 | CCDC37 | - |
| ENSG00000005022 | HGLibA_44763 | 40 | -0.772879774 | SLC25A5 | solute carrier family 25 member 5 [Source:HGNC Symbol;Acc:HGNC:10991] |
| ENSG00000010282 | HGLibA_21254 | 40 | -0.772879774 | HHATL | hedgehog acyltransferase like [Source:HGNC Symbol;Acc:HGNC:13242] |
| ENSG00000019505 | HGLibA_48058 | 40 | -0.772879774 | SYT13 | synaptotagmin 13 [Source:HGNC Symbol;Acc:HGNC:14962] |
| ENSG00000034510 | HGLibA_50656 | 40 | -0.772879774 | TMSB10 | thymosin beta 10 [Source:HGNC Symbol;Acc:HGNC:11879] |
| ENSG00000050438 | HGLibA_45229 | 40 | -0.772879774 | SLC4A8 | solute carrier family 4 member 8 [Source:HGNC Symbol;Acc:HGNC:11034] |
| ENSG00000060642 | HGLibA_36595 | 40 | -0.772879774 | PIGV | phosphatidylinositol glycan anchor biosynthesis class V [Source:HGNC Symbol;Acc:HGNC:26031] |
| ENSG00000064012 | HGLibA_07325 | 40 | -0.772879774 | CASP8 | caspase 8 [Source:HGNC Symbol;Acc:HGNC:1509] |
| ENSG00000065361 | HGLibA_15418 | 40 | -0.772879774 | ERBB3 | erb-b2 receptor tyrosine kinase 3 [Source:HGNC Symbol;Acc:HGNC:3431] |
| ENSG00000066422 | HGLibA_54818 | 40 | -0.772879774 | ZBTB11 | zinc finger and BTB domain containing 11 [Source:HGNC Symbol;Acc:HGNC:16740] |
| ENSG00000073670 | HGLibA_00732 | 40 | -0.772879774 | ADAM11 | ADAM metallopeptidase domain 11 [Source:HGNC Symbol;Acc:HGNC:189] |
| ENSG00000077152 | HGLibA_52661 | 40 | -0.772879774 | UBE2T | ubiquitin conjugating enzyme E2 T [Source:HGNC Symbol;Acc:HGNC:25009] |
| ENSG00000078114 | HGLibA_31582 | 40 | -0.772879774 | NEBL | nebulette [Source:HGNC Symbol;Acc:HGNC:16932] |
| ENSG00000079313 | HGLibA_40890 | 40 | -0.772879774 | REXO1 | RNA exonuclease 1 homolog [Source:HGNC Symbol;Acc:HGNC:24616] |
| ENSG00000080819 | HGLibA_10983 | 40 | -0.772879774 | CPOX | coproporphyrinogen oxidase [Source:HGNC Symbol;Acc:HGNC:2321] |
| ENSG00000089022 | HGLibA_28306 | 40 | -0.772879774 | MAPKAPK5 | mitogen-activated protein kinase-activated protein kinase 5 [Source:HGNC Symbol;Acc:HGNC:6889] |
| ENSG00000089289 | HGLibA_22781 | 40 | -0.772879774 | IGBP1 | immunoglobulin binding protein 1 [Source:HGNC Symbol;Acc:HGNC:5461] |
| ENSG00000092607 | HGLibA_48647 | 40 | -0.772879774 | TBX15 | T-box 15 [Source:HGNC Symbol;Acc:HGNC:11594] |
| ENSG00000099364 | HGLibA_16994 | 40 | -0.772879774 | FBXL19 | F-box and leucine rich repeat protein 19 [Source:HGNC Symbol;Acc:HGNC:25300] |
| ENSG00000100296 | HGLibA_49373 | 40 | -0.772879774 | THOC5 | THO complex 5 [Source:HGNC Symbol;Acc:HGNC:19074] |
| ENSG00000100365 | HGLibA_31302 | 40 | -0.772879774 | NCF4 | neutrophil cytosolic factor 4 [Source:HGNC Symbol;Acc:HGNC:7662] |
| ENSG00000101162 | HGLibA_52272 | 40 | -0.772879774 | TUBB1 | tubulin beta 1 class VI [Source:HGNC Symbol;Acc:HGNC:16257] |
| ENSG00000101292 | HGLibA_38674 | 40 | -0.772879774 | PROKR2 | prokineticin receptor 2 [Source:HGNC Symbol;Acc:HGNC:15836] |

| | | | | | |
|---|---|---|---|---|---|
| ENSG00000104660 | HGLibA_26331 | 40 | -0.772879774 | LEPROTL1 | leptin receptor overlapping transcript like 1 [Source:HGNC Symbol;Acc:HGNC:6555] |
| ENSG00000104731 | HGLibA_25101 | 40 | -0.772879774 | KLHDC4 | kelch domain containing 4 [Source:HGNC Symbol;Acc:HGNC:25272] |
| ENSG00000104880 | HGLibA_02828 | 40 | -0.772879774 | ARHGEF18 | Rho/Rac guanine nucleotide exchange factor 18 [Source:HGNC Symbol;Acc:HGNC:17090] |
| ENSG00000104976 | HGLibA_45892 | 40 | -0.772879774 | SNAPC2 | small nuclear RNA activating complex polypeptide 2 [Source:HGNC Symbol;Acc:HGNC:11135] |
| ENSG00000104998 | HGLibA_23116 | 40 | -0.772879774 | IL27RA | interleukin 27 receptor subunit alpha [Source:HGNC Symbol;Acc:HGNC:17290] |
| ENSG00000105127 | HGLibA_01446 | 40 | -0.772879774 | AKAP8 | A-kinase anchoring protein 8 [Source:HGNC Symbol;Acc:HGNC:378] |
| ENSG00000105221 | HGLibA_01510 | 40 | -0.772879774 | AKT2 | AKT serine/threonine kinase 2 [Source:HGNC Symbol;Acc:HGNC:392] |
| ENSG00000105278 | HGLibA_55260 | 40 | -0.772879774 | ZFR2 | zinc finger RNA binding protein 2 [Source:HGNC Symbol;Acc:HGNC:29189] |
| ENSG00000105339 | HGLibA_12948 | 40 | -0.772879774 | DENND3 | DENN domain containing 3 [Source:HGNC Symbol;Acc:HGNC:29134] |
| ENSG00000105388 | HGLibA_08944 | 40 | -0.772879774 | CEACAM5 | carcinoembryonic antigen related cell adhesion molecule 5 [Source:HGNC Symbol;Acc:HGNC:1817] |
| ENSG00000105472 | HGLibA_09924 | 40 | -0.772879774 | CLEC11A | C-type lectin domain containing 11A [Source:HGNC Symbol;Acc:HGNC:10576] |
| ENSG00000105705 | HGLibA_47717 | 40 | -0.772879774 | SUGP1 | SURP and G-patch domain containing 1 [Source:HGNC Symbol;Acc:HGNC:18643] |
| ENSG00000105707 | HGLibA_22038 | 40 | -0.772879774 | HPN | hepsin [Source:HGNC Symbol;Acc:HGNC:5155] |
| ENSG00000105778 | HGLibA_03894 | 40 | -0.772879774 | AVL9 | AVL9 cell migration associated [Source:HGNC Symbol;Acc:HGNC:28994] |
| ENSG00000106013 | HGLibA_02144 | 40 | -0.772879774 | ANKRD7 | ankyrin repeat domain 7 [Source:HGNC Symbol;Acc:HGNC:18588] |
| ENSG00000106993 | HGLibA_08548 | 40 | -0.772879774 | CDC37L1 | cell division cycle 37 like 1 [Source:HGNC Symbol;Acc:HGNC:17179] |
| ENSG00000107951 | HGLibA_30456 | 40 | -0.772879774 | MTPAP | mitochondrial poly(A) polymerase [Source:HGNC Symbol;Acc:HGNC:25532] |
| ENSG00000108509 | HGLibA_07119 | 40 | -0.772879774 | CAMTA2 | calmodulin binding transcription activator 2 [Source:HGNC Symbol;Acc:HGNC:18807] |
| ENSG00000108669 | HGLibA_12236 | 40 | -0.772879774 | CYTH1 | cytohesin 1 [Source:HGNC Symbol;Acc:HGNC:9501] |
| ENSG00000109180 | HGLibA_33245 | 40 | -0.772879774 | OCIAD1 | OCIA domain containing 1 [Source:HGNC Symbol;Acc:HGNC:16074] |
| ENSG00000109572 | HGLibA_09826 | 40 | -0.772879774 | CLCN3 | chloride voltage-gated channel 3 [Source:HGNC Symbol;Acc:HGNC:2021] |
| ENSG00000109906 | HGLibA_54827 | 40 | -0.772879774 | ZBTB16 | zinc finger and BTB domain containing 16 [Source:HGNC Symbol;Acc:HGNC:12930] |
| ENSG00000110497 | HGLibA_01753 | 40 | -0.772879774 | AMBRA1 | autophagy and beclin 1 regulator 1 [Source:HGNC Symbol;Acc:HGNC:25990] |
| ENSG00000110841 | HGLibA_37884 | 40 | -0.772879774 | PPFIBP1 | PPFIA binding protein 1 [Source:HGNC Symbol;Acc:HGNC:9249] |
| ENSG00000110876 | HGLibA_43308 | 40 | -0.772879774 | SELPLG | selectin P ligand [Source:HGNC Symbol;Acc:HGNC:10722] |
| ENSG00000111846 | HGLibA_18811 | 40 | -0.772879774 | GCNT2 | glucosaminyl (N-acetyl) transferase 2 (I blood group) [Source:HGNC Symbol;Acc:HGNC:4204] |
| ENSG00000113303 | HGLibA_04980 | 40 | -0.772879774 | BTNL8 | butyrophilin like 8 [Source:HGNC Symbol;Acc:HGNC:26131] |
| ENSG00000114686 | HGLibA_29944 | 40 | -0.772879774 | MRPL3 | mitochondrial ribosomal protein L3 [Source:HGNC Symbol;Acc:HGNC:10379] |
| ENSG00000116044 | HGLibA_31794 | 40 | -0.772879774 | NFE2L2 | erythroid 2 like 2 [Source:HGNC Symbol;Acc:HGNC:7782]" |
| ENSG00000116183 | HGLibA_35148 | 40 | -0.772879774 | PAPPA2 | pappalysin 2 [Source:HGNC Symbol;Acc:HGNC:14615] |
| ENSG00000116337 | HGLibA_01826 | 40 | -0.772879774 | AMPD2 | adenosine monophosphate deaminase 2 [Source:HGNC Symbol;Acc:HGNC:469] |
| ENSG00000117533 | HGLibA_53496 | 40 | -0.772879774 | VAMP4 | vesicle associated membrane protein 4 [Source:HGNC Symbol;Acc:HGNC:12645] |
| ENSG00000117859 | HGLibA_34670 | 40 | -0.772879774 | OSBPL9 | oxysterol binding protein like 9 [Source:HGNC Symbol;Acc:HGNC:16386] |
| ENSG00000118655 | HGLibA_12512 | 40 | -0.772879774 | DCLRE1B | DNA cross-link repair 1B [Source:HGNC Symbol;Acc:HGNC:17641] |
| ENSG00000119705 | HGLibA_45501 | 40 | -0.772879774 | SLIRP | SRA stem-loop interacting RNA binding protein [Source:HGNC Symbol;Acc:HGNC:20495] |
| ENSG00000123485 | HGLibA_21577 | 40 | -0.772879774 | HJURP | Holliday junction recognition protein [Source:HGNC Symbol;Acc:HGNC:25444] |
| ENSG00000124614 | HGLibA_42060 | 40 | -0.772879774 | RPS10 | ribosomal protein S10 [Source:HGNC Symbol;Acc:HGNC:10383] |
| ENSG00000125505 | HGLibA_28525 | 40 | -0.772879774 | MBOAT7 | membrane bound O-acyltransferase domain containing 7 [Source:HGNC Symbol;Acc:HGNC:15505] |
| ENSG00000127472 | HGLibA_36917 | 40 | -0.772879774 | PLA2G5 | phospholipase A2 group V [Source:HGNC Symbol;Acc:HGNC:9038] |
| ENSG00000127804 | HGLibA_28963 | 40 | -0.772879774 | METTL16 | methyltransferase like 16 [Source:HGNC Symbol;Acc:HGNC:28484] |
| ENSG00000127955 | HGLibA_19451 | 40 | -0.772879774 | GNAI1 | G protein subunit alpha i1 [Source:HGNC Symbol;Acc:HGNC:4384] |
| ENSG00000130150 | HGLibA_29687 | 40 | -0.772879774 | MOSPD2 | motile sperm domain containing 2 [Source:HGNC Symbol;Acc:HGNC:28381] |
| ENSG00000130165 | HGLibA_14954 | 40 | -0.772879774 | ELOF1 | elongation factor 1 homolog [Source:HGNC Symbol;Acc:HGNC:28691] |
| ENSG00000130304 | HGLibA_44813 | 40 | -0.772879774 | SLC27A1 | solute carrier family 27 member 1 [Source:HGNC Symbol;Acc:HGNC:10995] |
| ENSG00000130414 | HGLibA_31441 | 40 | -0.772879774 | NDUFA10 | NADH:ubiquinone oxidoreductase subunit A10 [Source:HGNC Symbol;Acc:HGNC:7684] |
| ENSG00000130956 | HGLibA_20795 | 40 | -0.772879774 | HABP4 | hyaluronan binding protein 4 [Source:HGNC Symbol;Acc:HGNC:17062] |
| ENSG00000131069 | HGLibA_00591 | 40 | -0.772879774 | ACSS2 | acyl-CoA synthetase short chain family member 2 [Source:HGNC Symbol;Acc:HGNC:15814] |
| ENSG00000131771 | HGLibA_38067 | 40 | -0.772879774 | PPP1R1B | protein phosphatase 1 regulatory inhibitor subunit 1B [Source:HGNC Symbol;Acc:HGNC:9287] |
| ENSG00000133169 | HGLibA_04441 | 40 | -0.772879774 | BEX1 | brain expressed X-linked 1 [Source:HGNC Symbol;Acc:HGNC:1036] |
| ENSG00000134183 | HGLibA_19476 | 40 | -0.772879774 | GNAT2 | G protein subunit alpha transducin 2 [Source:HGNC Symbol;Acc:HGNC:4394] |
| ENSG00000134245 | HGLibA_54361 | 40 | -0.772879774 | WNT2B | Wnt family member 2B [Source:HGNC Symbol;Acc:HGNC:12781] |
| ENSG00000134375 | HGLibA_49503 | 40 | -0.772879774 | TIMM17A | translocase of inner mitochondrial membrane 17A [Source:HGNC Symbol;Acc:HGNC:17315] |
| ENSG00000135144 | HGLibA_14078 | 40 | -0.772879774 | DTX1 | deltex E3 ubiquitin ligase 1 [Source:HGNC Symbol;Acc:HGNC:3060] |
| ENSG00000135312 | HGLibA_22359 | 40 | -0.772879774 | HTR1B | 5-hydroxytryptamine receptor 1B [Source:HGNC Symbol;Acc:HGNC:5287] |
| ENSG00000135346 | HGLibA_09297 | 40 | -0.772879774 | CGA | alpha polypeptide [Source:HGNC Symbol;Acc:HGNC:1885]" |
| ENSG00000136273 | HGLibA_22424 | 40 | -0.772879774 | HUS1 | HUS1 checkpoint clamp component [Source:HGNC Symbol;Acc:HGNC:5309] |
| ENSG00000136697 | HGLibA_23043 | 40 | -0.772879774 | IL1F10 | interleukin 1 family member 10 [Source:HGNC Symbol;Acc:HGNC:15552] |
| ENSG00000137033 | HGLibA_23139 | 40 | -0.772879774 | IL33 | interleukin 33 [Source:HGNC Symbol;Acc:HGNC:16028] |
| ENSG00000137409 | HGLibA_30325 | 40 | -0.772879774 | MTCH1 | mitochondrial carrier 1 [Source:HGNC Symbol;Acc:HGNC:17586] |
| ENSG00000138336 | HGLibA_49037 | 40 | -0.772879774 | TET1 | tet methylcytosine dioxygenase 1 [Source:HGNC Symbol;Acc:HGNC:29484] |
| ENSG00000139223 | HGLibA_02203 | 40 | -0.772879774 | ANP32D | acidic nuclear phosphoprotein 32 family member D [Source:HGNC Symbol;Acc:HGNC:16676] |
| ENSG00000139793 | HGLibA_28511 | 40 | -0.772879774 | MBNL2 | muscleblind like splicing regulator 2 [Source:HGNC Symbol;Acc:HGNC:16746] |
| ENSG00000140481 | HGLibA_07758 | 40 | -0.772879774 | CCDC33 | coiled-coil domain containing 33 [Source:HGNC Symbol;Acc:HGNC:26552] |
| ENSG00000140798 | HGLibA_00166 | 40 | -0.772879774 | ABCC12 | ATP binding cassette subfamily C member 12 [Source:HGNC Symbol;Acc:HGNC:14640] |
| ENSG00000141858 | HGLibA_42660 | 40 | -0.772879774 | SAMD1 | sterile alpha motif domain containing 1 [Source:HGNC Symbol;Acc:HGNC:17958] |
| ENSG00000142794 | HGLibA_31234 | 40 | -0.772879774 | NBPF3 | NBPF member 3 [Source:HGNC Symbol;Acc:HGNC:25076] |
| ENSG00000143258 | HGLibA_53301 | 40 | -0.772879774 | USP21 | ubiquitin specific peptidase 21 [Source:HGNC Symbol;Acc:HGNC:12620] |
| ENSG00000143258 | HGLibA_53302 | 40 | -0.772879774 | USP21 | ubiquitin specific peptidase 21 [Source:HGNC Symbol;Acc:HGNC:12620] |
| ENSG00000143612 | HGLibA_05858 | 40 | -0.772879774 | C1orf43 | chromosome 1 open reading frame 43 [Source:HGNC Symbol;Acc:HGNC:29876] |
| ENSG00000144010 | HGLibA_51504 | 40 | -0.772879774 | TRIM43B | tripartite motif containing 43B [Source:HGNC Symbol;Acc:HGNC:37146] |
| ENSG00000145365 | HGLibA_49456 | 40 | -0.772879774 | TIFA | TRAF interacting protein with forkhead associated domain [Source:HGNC Symbol;Acc:HGNC:19075] |
| ENSG00000145506 | HGLibA_32009 | 40 | -0.772879774 | NKD2 | naked cuticle homolog 2 [Source:HGNC Symbol;Acc:HGNC:17046] |
| ENSG00000146955 | HGLibA_39825 | 40 | -0.772879774 | RAB19 | member RAS oncogene family [Source:HGNC Symbol;Acc:HGNC:19982]" |
| ENSG00000147123 | HGLibA_31510 | 40 | -0.772879774 | NDUFB11 | NADH:ubiquinone oxidoreductase subunit B11 [Source:HGNC Symbol;Acc:HGNC:20372] |
| ENSG00000148798 | HGLibA_23267 | 40 | -0.772879774 | INA | internexin neuronal intermediate filament protein alpha [Source:HGNC Symbol;Acc:HGNC:6057] |
| ENSG00000149480 | HGLibA_30312 | 40 | -0.772879774 | MTA2 | metastasis associated 1 family member 2 [Source:HGNC Symbol;Acc:HGNC:7411] |
| ENSG00000149927 | HGLibA_13726 | 40 | -0.772879774 | DOC2A | double C2 domain alpha [Source:HGNC Symbol;Acc:HGNC:2985] |
| ENSG00000151470 | HGLibA_06324 | 40 | -0.772879774 | C4orf33 | chromosome 4 open reading frame 33 [Source:HGNC Symbol;Acc:HGNC:27025] |
| ENSG00000151687 | HGLibA_01943 | 40 | -0.772879774 | ANKAR | ankyrin and armadillo repeat containing [Source:HGNC Symbol;Acc:HGNC:26350] |
| ENSG00000152359 | HGLibA_37451 | 40 | -0.772879774 | POC5 | POC5 centriolar protein [Source:HGNC Symbol;Acc:HGNC:26658] |
| ENSG00000152822 | HGLibA_20342 | 40 | -0.772879774 | GRM1 | glutamate metabotropic receptor 1 [Source:HGNC Symbol;Acc:HGNC:4593] |
| ENSG00000152894 | HGLibA_39515 | 40 | -0.772879774 | PTPRK | receptor type K [Source:HGNC Symbol;Acc:HGNC:9674]" |
| ENSG00000154059 | HGLibA_23250 | 40 | -0.772879774 | IMPACT | impact RWD domain protein [Source:HGNC Symbol;Acc:HGNC:20387] |
| ENSG00000155380 | HGLibA_44418 | 40 | -0.772879774 | SLC16A1 | solute carrier family 16 member 1 [Source:HGNC Symbol;Acc:HGNC:10922] |
| ENSG00000157335 | HGLibA_09950 | 40 | -0.772879774 | CLEC18C | C-type lectin domain family 18 member C [Source:HGNC Symbol;Acc:HGNC:28538] |
| ENSG00000157593 | HGLibA_44963 | 40 | -0.772879774 | SLC35B2 | solute carrier family 35 member B2 [Source:HGNC Symbol;Acc:HGNC:16872] |
| ENSG00000159128 | HGLibA_22714 | 40 | -0.772879774 | IFNGR2 | interferon gamma receptor 2 [Source:HGNC Symbol;Acc:HGNC:5440] |
| ENSG00000159455 | HGLibA_26120 | 40 | -0.772879774 | LCE2B | late cornified envelope 2B [Source:HGNC Symbol;Acc:HGNC:16610] |
| ENSG00000159640 | HGLibA_00422 | 40 | -0.772879774 | ACE | angiotensin I converting enzyme [Source:HGNC Symbol;Acc:HGNC:2707] |
| ENSG00000159885 | HGLibA_55612 | 40 | -0.772879774 | ZNF222 | zinc finger protein 222 [Source:HGNC Symbol;Acc:HGNC:13015] |
| ENSG00000160213 | HGLibA_11481 | 40 | -0.772879774 | CSTB | cystatin B [Source:HGNC Symbol;Acc:HGNC:2482] |
| ENSG00000160439 | HGLibA_40740 | 40 | -0.772879774 | RDH13 | retinol dehydrogenase 13 [Source:HGNC Symbol;Acc:HGNC:19978] |
| ENSG00000160460 | HGLibA_46885 | 40 | -0.772879774 | SPTBN4 | non-erythrocytic 4 [Source:HGNC Symbol;Acc:HGNC:14896]" |
| ENSG00000162885 | HGLibA_03951 | 40 | -0.772879774 | B3GALNT2 | 3-N-acetylgalactosaminyltransferase 2 [Source:HGNC Symbol;Acc:HGNC:28596]" |
| ENSG00000163527 | HGLibA_47610 | 40 | -0.772879774 | STT3B | catalytic subunit of the oligosaccharyltransferase complex [Source:HGNC Symbol;Acc:HGNC:30611]" |
| ENSG00000163646 | HGLibA_10121 | 40 | -0.772879774 | CLRN1 | clarin 1 [Source:HGNC Symbol;Acc:HGNC:12605] |
| ENSG00000163661 | HGLibA_39572 | 40 | -0.772879774 | PTX3 | pentraxin 3 [Source:HGNC Symbol;Acc:HGNC:9692] |
| ENSG00000163932 | HGLibA_38540 | 40 | -0.772879774 | PRKCD | protein kinase C delta [Source:HGNC Symbol;Acc:HGNC:9399] |
| ENSG00000163961 | HGLibA_41565 | 40 | -0.772879774 | RNF168 | ring finger protein 168 [Source:HGNC Symbol;Acc:HGNC:26661] |
| ENSG00000164023 | HGLibA_43838 | 40 | -0.772879774 | SGMS2 | sphingomyelin synthase 2 [Source:HGNC Symbol;Acc:HGNC:28395] |
| ENSG00000164062 | HGLibA_02403 | 40 | -0.772879774 | APEH | acylaminoacyl-peptide hydrolase [Source:HGNC Symbol;Acc:HGNC:586] |
| ENSG00000164938 | HGLibA_51068 | 40 | -0.772879774 | TP53INP1 | tumor protein p53 inducible nuclear protein 1 [Source:HGNC Symbol;Acc:HGNC:18022] |
| ENSG00000165417 | HGLibA_20520 | 40 | -0.772879774 | GTF2A1 | general transcription factor IIA subunit 1 [Source:HGNC Symbol;Acc:HGNC:4646] |
| ENSG00000165630 | HGLibA_38710 | 40 | -0.772879774 | PRPF18 | pre-mRNA processing factor 18 [Source:HGNC Symbol;Acc:HGNC:17351] |
| ENSG00000165752 | HGLibA_47489 | 40 | -0.772879774 | STK32C | serine/threonine kinase 32C [Source:HGNC Symbol;Acc:HGNC:21332] |
| ENSG00000166199 | HGLibA_01651 | 40 | -0.772879774 | ALKBH3 | alpha-ketoglutaratedependent dioxygenase [Source:HGNC Symbol;Acc:HGNC:30141]" |
| ENSG00000166263 | HGLibA_47671 | 40 | -0.772879774 | STXBP4 | syntaxin binding protein 4 [Source:HGNC Symbol;Acc:HGNC:19694] |
| ENSG00000166833 | HGLibA_31198 | 40 | -0.772879774 | NAV2 | neuron navigator 2 [Source:HGNC Symbol;Acc:HGNC:15997] |
| ENSG00000167531 | HGLibA_25937 | 40 | -0.772879774 | LALBA | lactalbumin alpha [Source:HGNC Symbol;Acc:HGNC:6480] |
| ENSG00000167720 | HGLibA_47038 | 40 | -0.772879774 | SRR | serine racemase [Source:HGNC Symbol;Acc:HGNC:14398] |
| ENSG00000169087 | HGLibA_22325 | 40 | -0.772879774 | HSPBAP1 | HSPB1 associated protein 1 [Source:HGNC Symbol;Acc:HGNC:16389] |
| ENSG00000169548 | HGLibA_55723 | 40 | -0.772879774 | ZNF280A | zinc finger protein 280A [Source:HGNC Symbol;Acc:HGNC:18597] |
| ENSG00000169733 | HGLibA_40927 | 40 | -0.772879774 | RFNG | RFNG O-fucosylpeptide 3-beta-N-acetylglucosaminyltransferase [Source:HGNC Symbol;Acc:HGNC:9974] |
| ENSG00000169813 | HGLibA_21818 | 40 | -0.772879774 | HNRNPF | heterogeneous nuclear ribonucleoprotein F [Source:HGNC Symbol;Acc:HGNC:5039] |
| ENSG00000170325 | HGLibA_38366 | 40 | -0.772879774 | PRDM10 | PR/SET domain 10 [Source:HGNC Symbol;Acc:HGNC:13995] |
| ENSG00000170632 | HGLibA_03008 | 40 | -0.772879774 | ARMC10 | armadillo repeat containing 10 [Source:HGNC Symbol;Acc:HGNC:21706] |
| ENSG00000172322 | HGLibA_09929 | 40 | -0.772879774 | CLEC12A | C-type lectin domain family 12 member A [Source:HGNC Symbol;Acc:HGNC:31713] |
| ENSG00000172469 | HGLibA_28092 | 40 | -0.772879774 | MANEA | mannosidase endo-alpha [Source:HGNC Symbol;Acc:HGNC:21072] |
| ENSG00000173825 | HGLibA_49470 | 40 | -0.772879774 | TIGD3 | tigger transposable element derived 3 [Source:HGNC Symbol;Acc:HGNC:18334] |
| ENSG00000174669 | HGLibA_44843 | 40 | -0.772879774 | SLC29A2 | solute carrier family 29 member 2 [Source:HGNC Symbol;Acc:HGNC:11004] |
| ENSG00000174695 | HGLibA_50038 | 40 | -0.772879774 | TMEM167A | transmembrane protein 167A [Source:HGNC Symbol;Acc:HGNC:28330] |
| ENSG00000174915 | HGLibA_39293 | 40 | -0.772879774 | PTDSS2 | phosphatidylserine synthase 2 [Source:HGNC Symbol;Acc:HGNC:15463] |
| ENSG00000175455 | HGLibA_07614 | 40 | -0.772879774 | CCDC14 | coiled-coil domain containing 14 [Source:HGNC Symbol;Acc:HGNC:25766] |
| ENSG00000176927 | HGLibA_14546 | 40 | -0.772879774 | EFCAB5 | EF-hand calcium binding domain 5 [Source:HGNC Symbol;Acc:HGNC:24801] |
| ENSG00000176928 | HGLibA_18818 | 40 | -0.772879774 | GCNT4 | core 2 [Source:HGNC Symbol;Acc:HGNC:17973]" |
| ENSG00000177932 | HGLibA_55866 | 40 | -0.772879774 | ZNF354C | zinc finger protein 354C [Source:HGNC Symbol;Acc:HGNC:16736] |
| ENSG00000178602 | HGLibA_34747 | 40 | -0.772879774 | OTOS | otospiralin [Source:HGNC Symbol;Acc:HGNC:22644] |
| ENSG00000179057 | HGLibA_22888 | 40 | -0.772879774 | IGSF22 | immunoglobulin superfamily member 22 [Source:HGNC Symbol;Acc:HGNC:26750] |
| ENSG00000180116 | HGLibA_05238 | 40 | -0.772879774 | C12orf40 | chromosome 12 open reading frame 40 [Source:HGNC Symbol;Acc:HGNC:26846] |
| ENSG00000180530 | HGLibA_32691 | 40 | -0.772879774 | NRIP1 | nuclear receptor interacting protein 1 [Source:HGNC Symbol;Acc:HGNC:8001] |
| ENSG00000180708 | HGLibA_33534 | 40 | -0.772879774 | OR10K2 | olfactory receptor family 10 subfamily K member 2 [Source:HGNC Symbol;Acc:HGNC:14826] |
| ENSG00000180777 | HGLibA_02062 | 40 | -0.772879774 | ANKRD30B | ankyrin repeat domain 30B [Source:HGNC Symbol;Acc:HGNC:24165] |
| ENSG00000181619 | HGLibA_19894 | 40 | -0.772879774 | GPR135 | G protein-coupled receptor 135 [Source:HGNC Symbol;Acc:HGNC:19991] |
| ENSG00000181698 | HGLibA_34354 | 40 | -0.772879774 | OR5T1 | olfactory receptor family 5 subfamily T member 1 [Source:HGNC Symbol;Acc:HGNC:14821] |
| ENSG00000182534 | HGLibA_30624 | 40 | -0.772879774 | MXRA7 | matrix remodeling associated 7 [Source:HGNC Symbol;Acc:HGNC:7541] |

| | | | | | |
|---|---|---|---|---|---|
| ENSG00000182983 | HGLibA_56478 | 40 | -0.772879774 | ZNF662 | zinc finger protein 662 [Source:HGNC Symbol;Acc:HGNC:31930] |
| ENSG00000183426 | HGLibA_32462 | 40 | -0.772879774 | NPIPA1 | nuclear pore complex interacting protein family member A1 [Source:HGNC Symbol;Acc:HGNC:7909] |
| ENSG00000183624 | HGLibA_21673 | 40 | -0.772879774 | HMCES | ES cell specific [Source:HGNC Symbol;Acc:HGNC:24446]" |
| ENSG00000184076 | HGLibA_53105 | 40 | -0.772879774 | UQCR10 | complex III subunit X [Source:HGNC Symbol;Acc:HGNC:30863]" |
| ENSG00000184208 | HGLibA_06084 | 40 | -0.772879774 | C22orf46 | chromosome 22 open reading frame 46 [Source:HGNC Symbol;Acc:HGNC:26294] |
| ENSG00000184508 | HGLibA_21035 | 40 | -0.772879774 | HDDC3 | HD domain containing 3 [Source:HGNC Symbol;Acc:HGNC:30522] |
| ENSG00000184785 | HGLibA_45719 | 40 | -0.772879774 | SMIM10 | small integral membrane protein 10 [Source:HGNC Symbol;Acc:HGNC:41913] |
| ENSG00000184845 | HGLibA_13958 | 40 | -0.772879774 | DRD1 | dopamine receptor D1 [Source:HGNC Symbol;Acc:HGNC:3020] |
| ENSG00000185303 | HGLibA_43768 | 40 | -0.772879774 | SFTPA2 | surfactant protein A2 [Source:HGNC Symbol;Acc:HGNC:10799] |
| ENSG00000186501 | HGLibA_50239 | 40 | -0.772879774 | TMEM222 | transmembrane protein 222 [Source:HGNC Symbol;Acc:HGNC:25363] |
| ENSG00000186562 | HGLibA_12825 | 40 | -0.772879774 | DEFB105A | defensin beta 105A [Source:HGNC Symbol;Acc:HGNC:18087] |
| ENSG00000187792 | HGLibA_56572 | 40 | -0.772879774 | ZNF70 | zinc finger protein 70 [Source:HGNC Symbol;Acc:HGNC:13140] |
| ENSG00000188517 | HGLibA_10551 | 40 | -0.772879774 | COL25A1 | collagen type XXV alpha 1 chain [Source:HGNC Symbol;Acc:HGNC:18603] |
| ENSG00000188730 | HGLibA_53923 | 40 | -0.772879774 | VWC2 | von Willebrand factor C domain containing 2 [Source:HGNC Symbol;Acc:HGNC:30200] |
| ENSG00000196778 | HGLibA_34182 | 40 | -0.772879774 | OR52K1 | olfactory receptor family 52 subfamily K member 1 [Source:HGNC Symbol;Acc:HGNC:15222] |
| ENSG00000197081 | HGLibA_22809 | 40 | -0.772879774 | IGF2R | insulin like growth factor 2 receptor [Source:HGNC Symbol;Acc:HGNC:5467] |
| ENSG00000197430 | HGLibA_33411 | 40 | -0.772879774 | OPALIN | oligodendrocytic myelin paranodal and inner loop protein [Source:HGNC Symbol;Acc:HGNC:20707] |
| ENSG00000198464 | HGLibA_56072 | 40 | -0.772879774 | ZNF480 | zinc finger protein 480 [Source:HGNC Symbol;Acc:HGNC:23305] |
| ENSG00000198663 | HGLibA_06511 | 40 | -0.772879774 | C6orf89 | chromosome 6 open reading frame 89 [Source:HGNC Symbol;Acc:HGNC:21114] |
| ENSG00000198690 | HGLibA_16828 | 40 | -0.772879774 | FAN1 | FANCD2 and FANCI associated nuclease 1 [Source:HGNC Symbol;Acc:HGNC:29170] |
| ENSG00000198838 | HGLibA_42526 | 40 | -0.772879774 | RYR3 | ryanodine receptor 3 [Source:HGNC Symbol;Acc:HGNC:10485] |
| ENSG00000203780 | HGLibA_16863 | 40 | -0.772879774 | FANK1 | fibronectin type III and ankyrin repeat domains 1 [Source:HGNC Symbol;Acc:HGNC:23527] |
| ENSG00000204388 | HGLibA_22273 | 40 | -0.772879774 | HSPA1B | heat shock protein family A (Hsp70) member 1B [Source:HGNC Symbol;Acc:HGNC:5233] |
| ENSG00000204873 | HGLibA_25837 | 40 | -0.772879774 | KRTAP9-3 | keratin associated protein 9-3 [Source:HGNC Symbol;Acc:HGNC:16927] |
| ENSG00000205116 | HGLibA_50540 | 40 | -0.772879774 | TMEM88B | transmembrane protein 88B [Source:HGNC Symbol;Acc:HGNC:37099] |
| ENSG00000205279 | HGLibA_11761 | 40 | -0.772879774 | CTXN3 | cortexin 3 [Source:HGNC Symbol;Acc:HGNC:31110] |
| ENSG00000205643 | HGLibA_08859 | 40 | -0.772879774 | CDPF1 | cysteine rich DPF motif domain containing 1 [Source:HGNC Symbol;Acc:HGNC:33710] |
| ENSG00000205838 | HGLibA_52080 | 40 | -0.772879774 | TTC23L | tetratricopeptide repeat domain 23 like [Source:HGNC Symbol;Acc:HGNC:26355] |
| ENSG00000206422 | HGLibA_27307 | 40 | -0.772879774 | LRRC30 | leucine rich repeat containing 30 [Source:HGNC Symbol;Acc:HGNC:30219] |
| ENSG00000212122 | HGLibA_52008 | 40 | -0.772879774 | TSSK1B | testis specific serine kinase 1B [Source:HGNC Symbol;Acc:HGNC:14968] |
| ENSG00000214212 | HGLibA_05653 | 40 | -0.772879774 | C19orf38 | chromosome 19 open reading frame 38 [Source:HGNC Symbol;Acc:HGNC:34073] |
| ENSG00000221843 | HGLibA_06113 | 40 | -0.772879774 | C2orf16 | chromosome 2 open reading frame 16 [Source:HGNC Symbol;Acc:HGNC:25275] |
| ENSG00000221946 | HGLibA_18244 | 40 | -0.772879774 | FXYD7 | FXYD domain containing ion transport regulator 7 [Source:HGNC Symbol;Acc:HGNC:4034] |
| ENSG00000223802 | HGLibA_09199 | 40 | -0.772879774 | CERS1 | ceramide synthase 1 [Source:HGNC Symbol;Acc:HGNC:14253] |
| ENSG00000225899 | HGLibA_18000 | 40 | -0.772879774 | FRG2B | FSHD region gene 2 family member B [Source:HGNC Symbol;Acc:HGNC:33518] |
| ENSG00000229183 | HGLibA_36189 | 40 | -0.772879774 | PGA4 | group I (pepsinogen A) [Source:HGNC Symbol;Acc:HGNC:8886]" |
| ENSG00000229183 | HGLibA_36190 | 40 | -0.772879774 | PGA4 | group I (pepsinogen A) [Source:HGNC Symbol;Acc:HGNC:8886]" |
| ENSG00000230522 | HGLibA_28475 | 40 | -0.772879774 | MBD3L2 | methyl-CpG binding domain protein 3 like 2 [Source:HGNC Symbol;Acc:HGNC:18532] |
| ENSG00000237521 | HGLibA_34477 | 40 | -0.772879774 | OR7E24 | olfactory receptor family 7 subfamily E member 24 [Source:HGNC Symbol;Acc:HGNC:8396] |
| ENSG00000241476 | HGLibA_47197 | 40 | -0.772879774 | SSX2 | SSX family member 2 [Source:HGNC Symbol;Acc:HGNC:11336] |
| ENSG00000242612 | HGLibA_12777 | 40 | -0.772879774 | DECR2 | 4-dienoyl-CoA reductase 2 [Source:HGNC Symbol;Acc:HGNC:2754]" |
| ENSG00000243649 | HGLibA_09246 | 40 | -0.772879774 | CFB | complement factor B [Source:HGNC Symbol;Acc:HGNC:1037] |
| ENSG00000243955 | HGLibA_20451 | 40 | -0.772879774 | GSTA1 | glutathione S-transferase alpha 1 [Source:HGNC Symbol;Acc:HGNC:4626] |
| ENSG00000253873 | HGLibA_35524 | 40 | -0.772879774 | PCDHGA11 | 11 [Source:HGNC Symbol;Acc:HGNC:8698]" |
| ENSG00000257108 | HGLibA_31906 | 40 | -0.772879774 | NHLRC4 | NHL repeat containing 4 [Source:HGNC Symbol;Acc:HGNC:26700] |
| ENSG00000268940 | HGLibA_11499 | 40 | -0.772879774 | CT45A1 | cancer/testis antigen family 45 member A1 [Source:HGNC Symbol;Acc:HGNC:33267] |
| ENSG00000269067 | HGLibA_56639 | 40 | -0.772879774 | ZNF728 | zinc finger protein 728 [Source:HGNC Symbol;Acc:HGNC:32463] |
| ENSG00000272391 | HGLibA_37645 | 40 | -0.772879774 | POM121C | POM121 transmembrane nucleoporin C [Source:HGNC Symbol;Acc:HGNC:34005] |
| ENSG00000272674 | HGLibA_35490 | 40 | -0.772879774 | PCDHB16 | protocadherin beta 16 [Source:HGNC Symbol;Acc:HGNC:14546] |
| FAM92B | HGLibA_16800 | 40 | -0.772879774 | FAM92B | - |
| HRASLS2 | HGLibA_22079 | 40 | -0.772879774 | HRASLS2 | - |
| hsa-mir-1283-2 | HGLibA_57593 | 40 | -0.772879774 | hsa-mir-1283-2 | - |
| hsa-mir-186 | HGLibA_58006 | 40 | -0.772879774 | hsa-mir-186 | - |
| hsa-mir-199b | HGLibA_58136 | 40 | -0.772879774 | hsa-mir-199b | - |
| hsa-mir-3118-6 | HGLibA_58521 | 40 | -0.772879774 | hsa-mir-3118-6 | - |
| hsa-mir-3681 | HGLibA_59277 | 40 | -0.772879774 | hsa-mir-3681 | - |
| hsa-mir-373 | HGLibA_59385 | 40 | -0.772879774 | hsa-mir-373 | - |
| hsa-mir-4276 | HGLibA_59809 | 40 | -0.772879774 | hsa-mir-4276 | - |
| hsa-mir-4285 | HGLibA_59848 | 40 | -0.772879774 | hsa-mir-4285 | - |
| hsa-mir-4313 | HGLibA_59969 | 40 | -0.772879774 | hsa-mir-4313 | - |
| hsa-mir-4465 | HGLibA_60265 | 40 | -0.772879774 | hsa-mir-4465 | - |
| hsa-mir-4470 | HGLibA_60287 | 40 | -0.772879774 | hsa-mir-4470 | - |
| hsa-mir-4477b | HGLibA_60321 | 40 | -0.772879774 | hsa-mir-4477b | - |
| hsa-mir-4516 | HGLibA_60509 | 40 | -0.772879774 | hsa-mir-4516 | - |
| hsa-mir-4535 | HGLibA_60600 | 40 | -0.772879774 | hsa-mir-4535 | - |
| hsa-mir-4536-2 | HGLibA_60607 | 40 | -0.772879774 | hsa-mir-4536-2 | - |
| hsa-mir-4662b | HGLibA_60762 | 40 | -0.772879774 | hsa-mir-4662b | - |
| hsa-mir-4677 | HGLibA_60823 | 40 | -0.772879774 | hsa-mir-4677 | - |
| hsa-mir-4697 | HGLibA_60897 | 40 | -0.772879774 | hsa-mir-4697 | - |
| hsa-mir-4726 | HGLibA_61003 | 40 | -0.772879774 | hsa-mir-4726 | - |
| hsa-mir-4783 | HGLibA_61239 | 40 | -0.772879774 | hsa-mir-4783 | - |
| hsa-mir-4800 | HGLibA_61304 | 40 | -0.772879774 | hsa-mir-4800 | - |
| hsa-mir-507 | HGLibA_61488 | 40 | -0.772879774 | hsa-mir-507 | - |
| hsa-mir-5088 | HGLibA_61498 | 40 | -0.772879774 | hsa-mir-5088 | - |
| hsa-mir-509-1 | HGLibA_61506 | 40 | -0.772879774 | hsa-mir-509-1 | - |
| hsa-mir-516b-1 | HGLibA_61616 | 40 | -0.772879774 | hsa-mir-516b-1 | - |
| hsa-mir-520d | HGLibA_61748 | 40 | -0.772879774 | hsa-mir-520d | - |
| hsa-mir-548aa-2 | HGLibA_61854 | 40 | -0.772879774 | hsa-mir-548aa-2 | - |
| hsa-mir-548j | HGLibA_62027 | 40 | -0.772879774 | hsa-mir-548j | - |
| hsa-mir-548s | HGLibA_62062 | 40 | -0.772879774 | hsa-mir-548s | - |
| hsa-mir-5681a | HGLibA_62239 | 40 | -0.772879774 | hsa-mir-5681a | - |
| hsa-mir-5681b | HGLibA_62243 | 40 | -0.772879774 | hsa-mir-5681b | - |
| hsa-mir-569 | HGLibA_62277 | 40 | -0.772879774 | hsa-mir-569 | - |
| hsa-mir-5697 | HGLibA_62322 | 40 | -0.772879774 | hsa-mir-5697 | - |
| hsa-mir-6089-1 | HGLibA_62616 | 40 | -0.772879774 | hsa-mir-6089-1 | - |
| hsa-mir-646 | HGLibA_62829 | 40 | -0.772879774 | hsa-mir-646 | - |
| hsa-mir-658 | HGLibA_62964 | 40 | -0.772879774 | hsa-mir-658 | - |
| hsa-mir-676 | HGLibA_63196 | 40 | -0.772879774 | hsa-mir-676 | - |
| hsa-mir-676 | HGLibA_63198 | 40 | -0.772879774 | hsa-mir-676 | - |
| hsa-mir-6822 | HGLibA_63466 | 40 | -0.772879774 | hsa-mir-6822 | - |
| hsa-mir-6849 | HGLibA_63567 | 40 | -0.772879774 | hsa-mir-6849 | - |
| hsa-mir-6881 | HGLibA_63708 | 40 | -0.772879774 | hsa-mir-6881 | - |
| hsa-mir-708 | HGLibA_63781 | 40 | -0.772879774 | hsa-mir-708 | - |
| hsa-mir-92a-2 | HGLibA_64295 | 40 | -0.772879774 | hsa-mir-92a-2 | - |
| LOC147646 | HGLibA_26889 | 40 | -0.772879774 | LOC147646 | - |
| METTL20 | HGLibA_28972 | 40 | -0.772879774 | METTL20 | - |
| NARR | HGLibA_31152 | 40 | -0.772879774 | NARR | - |
| 9-Sep | HGLibA_43456 | 40 | -0.772879774 | 9-Sep | - |
| SMEK2 | HGLibA_45696 | 40 | -0.772879774 | SMEK2 | - |
| AGXT2L2 | HGLibA_01281 | 39 | -0.781925116 | AGXT2L2 | - |
| BAGE3 | HGLibA_04098 | 39 | -0.781925116 | BAGE3 | - |
| C16orf58 | HGLibA_05463 | 39 | -0.781925116 | C16orf58 | - |
| C3orf58 | HGLibA_06254 | 39 | -0.781925116 | C3orf58 | - |
| ENSG00000004799 | HGLibA_35904 | 39 | -0.781925116 | PDK4 | pyruvate dehydrogenase kinase 4 [Source:HGNC Symbol;Acc:HGNC:8812] |
| ENSG00000004897 | HGLibA_08539 | 39 | -0.781925116 | CDC27 | cell division cycle 27 [Source:HGNC Symbol;Acc:HGNC:1728] |
| ENSG00000005001 | HGLibA_38913 | 39 | -0.781925116 | PRSS22 | serine protease 22 [Source:HGNC Symbol;Acc:HGNC:14368] |
| ENSG00000006128 | HGLibA_48149 | 39 | -0.781925116 | TAC1 | tachykinin precursor 1 [Source:HGNC Symbol;Acc:HGNC:11517] |
| ENSG00000007314 | HGLibA_42992 | 39 | -0.781925116 | SCN4A | sodium voltage-gated channel alpha subunit 4 [Source:HGNC Symbol;Acc:HGNC:10591] |
| ENSG00000007520 | HGLibA_51999 | 39 | -0.781925116 | TSR3 | acp transferase ribosome maturation factor [Source:HGNC Symbol;Acc:HGNC:14175]" |
| ENSG00000029725 | HGLibA_40000 | 39 | -0.781925116 | RABEP1 | RAB GTPase binding effector protein 1 [Source:HGNC Symbol;Acc:HGNC:17677]" |
| ENSG00000044115 | HGLibA_11637 | 39 | -0.781925116 | CTNNA1 | catenin alpha 1 [Source:HGNC Symbol;Acc:HGNC:2509] |
| ENSG00000044574 | HGLibA_22288 | 39 | -0.781925116 | HSPA5 | heat shock protein family A (Hsp70) member 5 [Source:HGNC Symbol;Acc:HGNC:5238] |
| ENSG00000061936 | HGLibA_43749 | 39 | -0.781925116 | SFSWAP | splicing factor SWAP [Source:HGNC Symbol;Acc:HGNC:10790] |
| ENSG00000065308 | HGLibA_51288 | 39 | -0.781925116 | TRAM2 | translocation associated membrane protein 2 [Source:HGNC Symbol;Acc:HGNC:16855] |
| ENSG00000066735 | HGLibA_24917 | 39 | -0.781925116 | KIF26A | kinesin family member 26A [Source:HGNC Symbol;Acc:HGNC:20226] |
| ENSG00000066813 | HGLibA_00574 | 39 | -0.781925116 | ACSM2B | acyl-CoA synthetase medium chain family member 2B [Source:HGNC Symbol;Acc:HGNC:30931] |
| ENSG00000068724 | HGLibA_52150 | 39 | -0.781925116 | TTC7A | tetratricopeptide repeat domain 7A [Source:HGNC Symbol;Acc:HGNC:19750] |
| ENSG00000071994 | HGLibA_35727 | 39 | -0.781925116 | PDCD2 | programmed cell death 2 [Source:HGNC Symbol;Acc:HGNC:8762] |
| ENSG00000073150 | HGLibA_35113 | 39 | -0.781925116 | PANX2 | pannexin 2 [Source:HGNC Symbol;Acc:HGNC:8600] |
| ENSG00000073734 | HGLibA_00138 | 39 | -0.781925116 | ABCB11 | ATP binding cassette subfamily B member 11 [Source:HGNC Symbol;Acc:HGNC:42] |
| ENSG00000074317 | HGLibA_45914 | 39 | -0.781925116 | SNCB | synuclein beta [Source:HGNC Symbol;Acc:HGNC:11140] |
| ENSG00000080298 | HGLibA_40967 | 39 | -0.781925116 | RFX3 | regulatory factor X3 [Source:HGNC Symbol;Acc:HGNC:9984] |
| ENSG00000085491 | HGLibA_44681 | 39 | -0.781925116 | SLC25A24 | solute carrier family 25 member 24 [Source:HGNC Symbol;Acc:HGNC:20662] |
| ENSG00000087088 | HGLibA_04181 | 39 | -0.781925116 | BAX | apoptosis regulator [Source:HGNC Symbol;Acc:HGNC:959]" |
| ENSG00000089682 | HGLibA_40549 | 39 | -0.781925116 | RBM41 | RNA binding motif protein 41 [Source:HGNC Symbol;Acc:HGNC:25617] |
| ENSG00000090104 | HGLibA_41050 | 39 | -0.781925116 | RGS1 | regulator of G protein signaling 1 [Source:HGNC Symbol;Acc:HGNC:9991] |
| ENSG00000091157 | HGLibA_54159 | 39 | -0.781925116 | WDR7 | WD repeat domain 7 [Source:HGNC Symbol;Acc:HGNC:13490] |
| ENSG00000100441 | HGLibA_24578 | 39 | -0.781925116 | KHNYN | KH and NYN domain containing [Source:HGNC Symbol;Acc:HGNC:20166] |
| ENSG00000100842 | HGLibA_14611 | 39 | -0.781925116 | EFS | embryonal Fyn-associated substrate [Source:HGNC Symbol;Acc:HGNC:16898] |
| ENSG00000100982 | HGLibA_35609 | 39 | -0.781925116 | PCIF1 | PDX1 C-terminal inhibiting factor 1 [Source:HGNC Symbol;Acc:HGNC:16200] |
| ENSG00000101019 | HGLibA_53097 | 39 | -0.781925116 | UQCC1 | ubiquinol-cytochrome c reductase complex assembly factor 1 [Source:HGNC Symbol;Acc:HGNC:15891] |
| ENSG00000101160 | HGLibA_11736 | 39 | -0.781925116 | CTSZ | cathepsin Z [Source:HGNC Symbol;Acc:HGNC:2547] |
| ENSG00000101193 | HGLibA_19058 | 39 | -0.781925116 | GID8 | GID complex subunit 8 homolog [Source:HGNC Symbol;Acc:HGNC:15857] |
| ENSG00000104413 | HGLibA_15586 | 39 | -0.781925116 | ESRP1 | epithelial splicing regulatory protein 1 [Source:HGNC Symbol;Acc:HGNC:25966] |
| ENSG00000104499 | HGLibA_19413 | 39 | -0.781925116 | GML | glycosylphosphatidylinositol anchored molecule like [Source:HGNC Symbol;Acc:HGNC:4375] |

| | | | | | |
|---|---|---|---|---|---|
| ENSG00000104537 | HGLibA_02229 | 39 | -0.781925116 | ANXA13 | annexin A13 [Source:HGNC Symbol;Acc:HGNC:536] |
| ENSG00000105866 | HGLibA_46303 | 39 | -0.781925116 | SP4 | Sp4 transcription factor [Source:HGNC Symbol;Acc:HGNC:11209] |
| ENSG00000106302 | HGLibA_22447 | 39 | -0.781925116 | HYAL4 | hyaluronidase 4 [Source:HGNC Symbol;Acc:HGNC:5323] |
| ENSG00000106591 | HGLibA_29950 | 39 | -0.781925116 | MRPL32 | mitochondrial ribosomal protein L32 [Source:HGNC Symbol;Acc:HGNC:14035] |
| ENSG00000106852 | HGLibA_26461 | 39 | -0.781925116 | LHX6 | LIM homeobox 6 [Source:HGNC Symbol;Acc:HGNC:21735] |
| ENSG00000108788 | HGLibA_29439 | 39 | -0.781925116 | MLX | MAX dimerization protein [Source:HGNC Symbol;Acc:HGNC:11645]" |
| ENSG00000109445 | HGLibA_55812 | 39 | -0.781925116 | ZNF330 | zinc finger protein 330 [Source:HGNC Symbol;Acc:HGNC:15462] |
| ENSG00000112473 | HGLibA_45121 | 39 | -0.781925116 | SLC39A7 | solute carrier family 39 member 7 [Source:HGNC Symbol;Acc:HGNC:4927] |
| ENSG00000113296 | HGLibA_49330 | 39 | -0.781925116 | THBS4 | thrombospondin 4 [Source:HGNC Symbol;Acc:HGNC:11788] |
| ENSG00000113360 | HGLibA_13984 | 39 | -0.781925116 | DROSHA | drosha ribonuclease III [Source:HGNC Symbol;Acc:HGNC:17904] |
| ENSG00000113492 | HGLibA_01273 | 39 | -0.781925116 | AGXT2 | alanine--glyoxylate aminotransferase 2 [Source:HGNC Symbol;Acc:HGNC:14412] |
| ENSG00000113504 | HGLibA_44376 | 39 | -0.781925116 | SLC12A7 | solute carrier family 12 member 7 [Source:HGNC Symbol;Acc:HGNC:10915] |
| ENSG00000113763 | HGLibA_53013 | 39 | -0.781925116 | UNC5A | unc-5 netrin receptor A [Source:HGNC Symbol;Acc:HGNC:12567] |
| ENSG00000114391 | HGLibA_41911 | 39 | -0.781925116 | RPL24 | ribosomal protein L24 [Source:HGNC Symbol;Acc:HGNC:10325] |
| ENSG00000115266 | HGLibA_02393 | 39 | -0.781925116 | APC2 | WNT signaling pathway regulator [Source:HGNC Symbol;Acc:HGNC:24036]" |
| ENSG00000115641 | HGLibA_17482 | 39 | -0.781925116 | FHL2 | four and a half LIM domains 2 [Source:HGNC Symbol;Acc:HGNC:3703] |
| ENSG00000115661 | HGLibA_47458 | 39 | -0.781925116 | STK16 | serine/threonine kinase 16 [Source:HGNC Symbol;Acc:HGNC:11394] |
| ENSG00000115718 | HGLibA_38650 | 39 | -0.781925116 | PROC | inactivator of coagulation factors Va and VIIIa [Source:HGNC Symbol;Acc:HGNC:9451]" |
| ENSG00000116396 | HGLibA_24170 | 39 | -0.781925116 | KCNC4 | potassium voltage-gated channel subfamily C member 4 [Source:HGNC Symbol;Acc:HGNC:6236] |
| ENSG00000116717 | HGLibA_18415 | 39 | -0.781925116 | GADD45A | growth arrest and DNA damage inducible alpha [Source:HGNC Symbol;Acc:HGNC:4095] |
| ENSG00000117266 | HGLibA_08748 | 39 | -0.781925116 | CDK18 | cyclin dependent kinase 18 [Source:HGNC Symbol;Acc:HGNC:8751] |
| ENSG00000119283 | HGLibA_51589 | 39 | -0.781925116 | TRIM67 | tripartite motif containing 67 [Source:HGNC Symbol;Acc:HGNC:31859] |
| ENSG00000119929 | HGLibA_11799 | 39 | -0.781925116 | CUTC | cutC copper transporter [Source:HGNC Symbol;Acc:HGNC:24271] |
| ENSG00000120055 | HGLibA_05109 | 39 | -0.781925116 | C10orf95 | chromosome 10 open reading frame 95 [Source:HGNC Symbol;Acc:HGNC:25880] |
| ENSG00000120696 | HGLibA_24114 | 39 | -0.781925116 | KBTBD7 | kelch repeat and BTB domain containing 7 [Source:HGNC Symbol;Acc:HGNC:25266] |
| ENSG00000120992 | HGLibA_27736 | 39 | -0.781925116 | LYPLA1 | lysophospholipase I [Source:HGNC Symbol;Acc:HGNC:6737] |
| ENSG00000122484 | HGLibA_41818 | 39 | -0.781925116 | RPAP2 | RNA polymerase II associated protein 2 [Source:HGNC Symbol;Acc:HGNC:25791] |
| ENSG00000124155 | HGLibA_36587 | 39 | -0.781925116 | PIGT | phosphatidylinositol glycan anchor biosynthesis class T [Source:HGNC Symbol;Acc:HGNC:14938] |
| ENSG00000124721 | HGLibA_13498 | 39 | -0.781925116 | DNAH8 | dynein axonemal heavy chain 8 [Source:HGNC Symbol;Acc:HGNC:2952] |
| ENSG00000125735 | HGLibA_50815 | 39 | -0.781925116 | TNFSF14 | TNF superfamily member 14 [Source:HGNC Symbol;Acc:HGNC:11930] |
| ENSG00000125740 | HGLibA_17799 | 39 | -0.781925116 | FOSB | AP-1 transcription factor subunit [Source:HGNC Symbol;Acc:HGNC:3797]" |
| ENSG00000127318 | HGLibA_23087 | 39 | -0.781925116 | IL22 | interleukin 22 [Source:HGNC Symbol;Acc:HGNC:14900] |
| ENSG00000130193 | HGLibA_49346 | 39 | -0.781925116 | THEM6 | thioesterase superfamily member 6 [Source:HGNC Symbol;Acc:HGNC:29656] |
| ENSG00000131203 | HGLibA_22564 | 39 | -0.781925116 | IDO1 | 3-dioxygenase 1 [Source:HGNC Symbol;Acc:HGNC:6059]" |
| ENSG00000131375 | HGLibA_07175 | 39 | -0.781925116 | CAPN7 | calpain 7 [Source:HGNC Symbol;Acc:HGNC:1484] |
| ENSG00000131652 | HGLibA_49375 | 39 | -0.781925116 | THOC6 | THO complex 6 [Source:HGNC Symbol;Acc:HGNC:28369] |
| ENSG00000131788 | HGLibA_36513 | 39 | -0.781925116 | PIAS3 | protein inhibitor of activated STAT 3 [Source:HGNC Symbol;Acc:HGNC:16861] |
| ENSG00000132321 | HGLibA_23487 | 39 | -0.781925116 | IQCA1 | IQ motif containing with AAA domain 1 [Source:HGNC Symbol;Acc:HGNC:26195] |
| ENSG00000132437 | HGLibA_12600 | 39 | -0.781925116 | DDC | dopa decarboxylase [Source:HGNC Symbol;Acc:HGNC:2719] |
| ENSG00000133048 | HGLibA_09436 | 39 | -0.781925116 | CHI3L1 | chitinase 3 like 1 [Source:HGNC Symbol;Acc:HGNC:1932] |
| ENSG00000133773 | HGLibA_07807 | 39 | -0.781925116 | CCDC59 | coiled-coil domain containing 59 [Source:HGNC Symbol;Acc:HGNC:25005] |
| ENSG00000135338 | HGLibA_26090 | 39 | -0.781925116 | LCA5 | lebercilin [Source:HGNC Symbol;Acc:HGNC:31923]" |
| ENSG00000135622 | HGLibA_43353 | 39 | -0.781925116 | SEMA4F | ssemaphorin 4F [Source:HGNC Symbol;Acc:HGNC:10734] |
| ENSG00000135940 | HGLibA_10840 | 39 | -0.781925116 | COX5B | cytochrome c oxidase subunit 5B [Source:HGNC Symbol;Acc:HGNC:2269] |
| ENSG00000136531 | HGLibA_42979 | 39 | -0.781925116 | SCN2A | sodium voltage-gated channel alpha subunit 2 [Source:HGNC Symbol;Acc:HGNC:10588] |
| ENSG00000136560 | HGLibA_48302 | 39 | -0.781925116 | TANK | TRAF family member associated NFKB activator [Source:HGNC Symbol;Acc:HGNC:11562] |
| ENSG00000137547 | HGLibA_29905 | 39 | -0.781925116 | MRPL15 | mitochondrial ribosomal protein L15 [Source:HGNC Symbol;Acc:HGNC:14054] |
| ENSG00000137825 | HGLibA_23845 | 39 | -0.781925116 | ITPKA | inositol-trisphosphate 3-kinase A [Source:HGNC Symbol;Acc:HGNC:6178] |
| ENSG00000138081 | HGLibA_17027 | 39 | -0.781925116 | FBXO11 | F-box protein 11 [Source:HGNC Symbol;Acc:HGNC:13590] |
| ENSG00000138381 | HGLibA_03338 | 39 | -0.781925116 | ASNSD1 | asparagine synthetase domain containing 1 [Source:HGNC Symbol;Acc:HGNC:24910] |
| ENSG00000138483 | HGLibA_07803 | 39 | -0.781925116 | CCDC54 | coiled-coil domain containing 54 [Source:HGNC Symbol;Acc:HGNC:30703] |
| ENSG00000139648 | HGLibA_25526 | 39 | -0.781925116 | KRT71 | keratin 71 [Source:HGNC Symbol;Acc:HGNC:28927] |
| ENSG00000140092 | HGLibA_16950 | 39 | -0.781925116 | FBLN5 | fibulin 5 [Source:HGNC Symbol;Acc:HGNC:3602] |
| ENSG00000140526 | HGLibA_00271 | 39 | -0.781925116 | ABHD2 | abhydrolase domain containing 2 [Source:HGNC Symbol;Acc:HGNC:18717] |
| ENSG00000141371 | HGLibA_05557 | 39 | -0.781925116 | C17orf64 | chromosome 17 open reading frame 64 [Source:HGNC Symbol;Acc:HGNC:26990] |
| ENSG00000141556 | HGLibA_48589 | 39 | -0.781925116 | TBCD | tubulin folding cofactor D [Source:HGNC Symbol;Acc:HGNC:11581] |
| ENSG00000141858 | HGLibA_42659 | 39 | -0.781925116 | SAMD1 | sterile alpha motif domain containing 1 [Source:HGNC Symbol;Acc:HGNC:17958] |
| ENSG00000143376 | HGLibA_46076 | 39 | -0.781925116 | SNX27 | sorting nexin family member 27 [Source:HGNC Symbol;Acc:HGNC:20073] |
| ENSG00000144021 | HGLibA_09675 | 39 | -0.781925116 | CIAO1 | cytosolic iron-sulfur assembly component 1 [Source:HGNC Symbol;Acc:HGNC:14280] |
| ENSG00000144233 | HGLibA_01806 | 39 | -0.781925116 | AMMECR1L | AMMECR1 like [Source:HGNC Symbol;Acc:HGNC:28658] |
| ENSG00000146021 | HGLibA_25178 | 39 | -0.781925116 | KLHL3 | kelch like family member 3 [Source:HGNC Symbol;Acc:HGNC:6354] |
| ENSG00000146039 | HGLibA_44468 | 39 | -0.781925116 | SLC17A4 | solute carrier family 17 member 4 [Source:HGNC Symbol;Acc:HGNC:10932] |
| ENSG00000146166 | HGLibA_26418 | 39 | -0.781925116 | LGSN | lens protein with glutamine synthetase domain [Source:HGNC Symbol;Acc:HGNC:21016]" |
| ENSG00000146477 | HGLibA_44581 | 39 | -0.781925116 | SLC22A3 | solute carrier family 22 member 3 [Source:HGNC Symbol;Acc:HGNC:10967] |
| ENSG00000146858 | HGLibA_54993 | 39 | -0.781925116 | ZC3HAV1L | antiviral 1 like [Source:HGNC Symbol;Acc:HGNC:22423]" |
| ENSG00000146859 | HGLibA_49962 | 39 | -0.781925116 | TMEM140 | transmembrane protein 140 [Source:HGNC Symbol;Acc:HGNC:21870] |
| ENSG00000146872 | HGLibA_49624 | 39 | -0.781925116 | TLK2 | tousled like kinase 2 [Source:HGNC Symbol;Acc:HGNC:11842] |
| ENSG00000146918 | HGLibA_31272 | 39 | -0.781925116 | NCAPG2 | non-SMC condensin II complex subunit G2 [Source:HGNC Symbol;Acc:HGNC:21904] |
| ENSG00000147647 | HGLibA_13923 | 39 | -0.781925116 | DPYS | dihydropyrimidinase [Source:HGNC Symbol;Acc:HGNC:3013] |
| ENSG00000148057 | HGLibA_22561 | 39 | -0.781925116 | IDNK | gluconokinase [Source:HGNC Symbol;Acc:HGNC:31367]" |
| ENSG00000148429 | HGLibA_53406 | 39 | -0.781925116 | USP6NL | USP6 N-terminal like [Source:HGNC Symbol;Acc:HGNC:16858] |
| ENSG00000149418 | HGLibA_47224 | 39 | -0.781925116 | ST14 | suppression of tumorigenicity 14 [Source:HGNC Symbol;Acc:HGNC:11344] |
| ENSG00000149634 | HGLibA_46460 | 39 | -0.781925116 | SPATA25 | spermatogenesis associated 25 [Source:HGNC Symbol;Acc:HGNC:16158] |
| ENSG00000150637 | HGLibA_08301 | 39 | -0.781925116 | CD226 | CD226 molecule [Source:HGNC Symbol;Acc:HGNC:16961] |
| ENSG00000151948 | HGLibA_19332 | 39 | -0.781925116 | GLT1D1 | glycosyltransferase 1 domain containing 1 [Source:HGNC Symbol;Acc:HGNC:26483] |
| ENSG00000152910 | HGLibA_10431 | 39 | -0.781925116 | CNTNAP4 | contactin associated protein like 4 [Source:HGNC Symbol;Acc:HGNC:18747] |
| ENSG00000153165 | HGLibA_41032 | 39 | -0.781925116 | RGPD3 | RANBP2-like and GRIP domain containing 3 [Source:HGNC Symbol;Acc:HGNC:32416] |
| ENSG00000153902 | HGLibA_26401 | 39 | -0.781925116 | LGI4 | leucine rich repeat LGI family member 4 [Source:HGNC Symbol;Acc:HGNC:18712] |
| ENSG00000154040 | HGLibA_06866 | 39 | -0.781925116 | CABYR | calcium binding tyrosine phosphorylation regulated [Source:HGNC Symbol;Acc:HGNC:15569] |
| ENSG00000154114 | HGLibA_48595 | 39 | -0.781925116 | TBCEL | tubulin folding cofactor E like [Source:HGNC Symbol;Acc:HGNC:28115] |
| ENSG00000154415 | HGLibA_38097 | 39 | -0.781925116 | PPP1R3A | protein phosphatase 1 regulatory subunit 3A [Source:HGNC Symbol;Acc:HGNC:9291] |
| ENSG00000155096 | HGLibA_03938 | 39 | -0.781925116 | AZIN1 | antizyme inhibitor 1 [Source:HGNC Symbol;Acc:HGNC:16432] |
| ENSG00000155366 | HGLibA_41181 | 39 | -0.781925116 | RHOC | ras homolog family member C [Source:HGNC Symbol;Acc:HGNC:669] |
| ENSG00000156219 | HGLibA_03160 | 39 | -0.781925116 | ART3 | ADP-ribosyltransferase 3 [Source:HGNC Symbol;Acc:HGNC:725] |
| ENSG00000157045 | HGLibA_32834 | 39 | -0.781925116 | NTAN1 | N-terminal asparagine amidase [Source:HGNC Symbol;Acc:HGNC:29909] |
| ENSG00000158321 | HGLibA_03885 | 39 | -0.781925116 | AUTS2 | activator of transcription and developmental regulator [Source:HGNC Symbol;Acc:HGNC:14262]" |
| ENSG00000159248 | HGLibA_19187 | 39 | -0.781925116 | GJD2 | gap junction protein delta 2 [Source:HGNC Symbol;Acc:HGNC:19154] |
| ENSG00000159658 | HGLibA_14532 | 39 | -0.781925116 | EFCAB14 | EF-hand calcium binding domain 14 [Source:HGNC Symbol;Acc:HGNC:29051] |
| ENSG00000159884 | HGLibA_07526 | 39 | -0.781925116 | CCDC107 | coiled-coil domain containing 107 [Source:HGNC Symbol;Acc:HGNC:28465] |
| ENSG00000160218 | HGLibA_51299 | 39 | -0.781925116 | TRAPPC10 | trafficking protein particle complex 10 [Source:HGNC Symbol;Acc:HGNC:11868] |
| ENSG00000161558 | HGLibA_49967 | 39 | -0.781925116 | TMEM143 | transmembrane protein 143 [Source:HGNC Symbol;Acc:HGNC:25603] |
| ENSG00000162298 | HGLibA_48110 | 39 | -0.781925116 | SYVN1 | synoviolin 1 [Source:HGNC Symbol;Acc:HGNC:20738] |
| ENSG00000162601 | HGLibA_30921 | 39 | -0.781925116 | MYSM1 | SWIRM and MPN domains 1 [Source:HGNC Symbol;Acc:HGNC:29401]" |
| ENSG00000162804 | HGLibA_45922 | 39 | -0.781925116 | SNED1 | nidogen and EGF like domains 1 [Source:HGNC Symbol;Acc:HGNC:24696]" |
| ENSG00000162994 | HGLibA_10018 | 39 | -0.781925116 | CLHC1 | clathrin heavy chain linker domain containing 1 [Source:HGNC Symbol;Acc:HGNC:26453] |
| ENSG00000164855 | HGLibA_50110 | 39 | -0.781925116 | TMEM184A | transmembrane protein 184A [Source:HGNC Symbol;Acc:HGNC:28797] |
| ENSG00000165935 | HGLibA_45664 | 39 | -0.781925116 | SMCO2 | single-pass membrane protein with coiled-coil domains 2 [Source:HGNC Symbol;Acc:HGNC:34448] |
| ENSG00000165995 | HGLibA_06919 | 39 | -0.781925116 | CACNB2 | calcium voltage-gated channel auxiliary subunit beta 2 [Source:HGNC Symbol;Acc:HGNC:1402] |
| ENSG00000165995 | HGLibA_06921 | 39 | -0.781925116 | CACNB2 | calcium voltage-gated channel auxiliary subunit beta 2 [Source:HGNC Symbol;Acc:HGNC:1402] |
| ENSG00000166037 | HGLibA_09146 | 39 | -0.781925116 | CEP57 | centrosomal protein 57 [Source:HGNC Symbol;Acc:HGNC:30794] |
| ENSG00000166603 | HGLibA_28549 | 39 | -0.781925116 | MC4R | melanocortin 4 receptor [Source:HGNC Symbol;Acc:HGNC:6932] |
| ENSG00000167131 | HGLibA_07515 | 39 | -0.781925116 | CCDC103 | coiled-coil domain containing 103 [Source:HGNC Symbol;Acc:HGNC:32700] |
| ENSG00000167306 | HGLibA_30854 | 39 | -0.781925116 | MYO5B | myosin VB [Source:HGNC Symbol;Acc:HGNC:7603] |
| ENSG00000167615 | HGLibA_26300 | 39 | -0.781925116 | LENG8 | leukocyte receptor cluster member 8 [Source:HGNC Symbol;Acc:HGNC:15500] |
| ENSG00000167914 | HGLibA_20404 | 39 | -0.781925116 | GSDMA | gasdermin A [Source:HGNC Symbol;Acc:HGNC:13311] |
| ENSG00000167978 | HGLibA_47047 | 39 | -0.781925116 | SRRM2 | serine/arginine repetitive matrix 2 [Source:HGNC Symbol;Acc:HGNC:16639] |
| ENSG00000168454 | HGLibA_52420 | 39 | -0.781925116 | TXNDC2 | thioredoxin domain containing 2 [Source:HGNC Symbol;Acc:HGNC:16470] |
| ENSG00000168792 | HGLibA_00253 | 39 | -0.781925116 | ABHD15 | abhydrolase domain containing 15 [Source:HGNC Symbol;Acc:HGNC:26971] |
| ENSG00000168894 | HGLibA_41582 | 39 | -0.781925116 | RNF181 | ring finger protein 181 [Source:HGNC Symbol;Acc:HGNC:28037] |
| ENSG00000168993 | HGLibA_10932 | 39 | -0.781925116 | CPLX1 | complexin 1 [Source:HGNC Symbol;Acc:HGNC:2309] |
| ENSG00000169495 | HGLibA_22415 | 39 | -0.781925116 | HTRA4 | HtrA serine peptidase 4 [Source:HGNC Symbol;Acc:HGNC:26909] |
| ENSG00000169635 | HGLibA_21291 | 39 | -0.781925116 | HIC2 | HIC ZBTB transcriptional repressor 2 [Source:HGNC Symbol;Acc:HGNC:18595] |
| ENSG00000169856 | HGLibA_33392 | 39 | -0.781925116 | ONECUT1 | one cut homeobox 1 [Source:HGNC Symbol;Acc:HGNC:8138] |
| ENSG00000169857 | HGLibA_03889 | 39 | -0.781925116 | AVEN | apoptosis and caspase activation inhibitor [Source:HGNC Symbol;Acc:HGNC:13509] |
| ENSG00000169957 | HGLibA_56698 | 39 | -0.781925116 | ZNF768 | zinc finger protein 768 [Source:HGNC Symbol;Acc:HGNC:26273] |
| ENSG00000170054 | HGLibA_43533 | 39 | -0.781925116 | SERPINA9 | serpin family A member 9 [Source:HGNC Symbol;Acc:HGNC:15995] |
| ENSG00000170537 | HGLibA_49766 | 39 | -0.781925116 | TMC7 | transmembrane channel like 7 [Source:HGNC Symbol;Acc:HGNC:23000] |
| ENSG00000171509 | HGLibA_42494 | 39 | -0.781925116 | RXFP1 | relaxin family peptide receptor 1 [Source:HGNC Symbol;Acc:HGNC:19718] |
| ENSG00000172000 | HGLibA_56226 | 39 | -0.781925116 | ZNF556 | zinc finger protein 556 [Source:HGNC Symbol;Acc:HGNC:25669] |
| ENSG00000172289 | HGLibA_33550 | 39 | -0.781925116 | OR10V1 | olfactory receptor family 10 subfamily V member 1 [Source:HGNC Symbol;Acc:HGNC:15136] |
| ENSG00000173227 | HGLibA_48054 | 39 | -0.781925116 | SYT12 | synaptotagmin 12 [Source:HGNC Symbol;Acc:HGNC:18381] |
| ENSG00000173559 | HGLibA_31023 | 39 | -0.781925116 | NABP1 | nucleic acid binding protein 1 [Source:HGNC Symbol;Acc:HGNC:26232] |
| ENSG00000173960 | HGLibA_52783 | 39 | -0.781925116 | UBXN2A | UBX domain protein 2A [Source:HGNC Symbol;Acc:HGNC:27265] |
| ENSG00000174137 | HGLibA_16634 | 39 | -0.781925116 | FAM53A | family with sequence similarity 53 member A [Source:HGNC Symbol;Acc:HGNC:31860] |
| ENSG00000174586 | HGLibA_56103 | 39 | -0.781925116 | ZNF497 | zinc finger protein 497 [Source:HGNC Symbol;Acc:HGNC:23714] |
| ENSG00000175216 | HGLibA_09763 | 39 | -0.781925116 | CKAP5 | cytoskeleton associated protein 5 [Source:HGNC Symbol;Acc:HGNC:28959] |
| ENSG00000175324 | HGLibA_27517 | 39 | -0.781925116 | LSM1 | mRNA degradation associated [Source:HGNC Symbol;Acc:HGNC:20472]" |
| ENSG00000175329 | HGLibA_23690 | 39 | -0.781925116 | ISX | intestine specific homeobox [Source:HGNC Symbol;Acc:HGNC:28084] |
| ENSG00000175445 | HGLibA_27103 | 39 | -0.781925116 | LPL | lipoprotein lipase [Source:HGNC Symbol;Acc:HGNC:6677] |
| ENSG00000175567 | HGLibA_52834 | 39 | -0.781925116 | UCP2 | uncoupling protein 2 [Source:HGNC Symbol;Acc:HGNC:12518] |
| ENSG00000175602 | HGLibA_07895 | 39 | -0.781925116 | CCDC85B | coiled-coil domain containing 85B [Source:HGNC Symbol;Acc:HGNC:24926] |
| ENSG00000177519 | HGLibA_42052 | 39 | -0.781925116 | RPRM | TP53 dependent G2 arrest mediator homolog [Source:HGNC Symbol;Acc:HGNC:24201]" |
| ENSG00000178074 | HGLibA_06168 | 39 | -0.781925116 | C2orf69 | chromosome 2 open reading frame 69 [Source:HGNC Symbol;Acc:HGNC:26799] |
| ENSG00000178573 | HGLibA_27866 | 39 | -0.781925116 | MAF | MAF bZIP transcription factor [Source:HGNC Symbol;Acc:HGNC:6776] |
| ENSG00000178726 | HGLibA_49319 | 39 | -0.781925116 | THBD | thrombomodulin [Source:HGNC Symbol;Acc:HGNC:11784] |

| | | | | | |
|---|---|---|---|---|---|
| ENSG00000179919 | HGLibA_33475 | 39 | -0.781925116 | OR10A7 | olfactory receptor family 10 subfamily A member 7 [Source:HGNC Symbol;Acc:HGNC:15329] |
| ENSG00000180386 | HGLibA_25844 | 39 | -0.781925116 | KRTAP9-7 | keratin associated protein 9-7 [Source:HGNC Symbol;Acc:HGNC:18915] |
| ENSG00000180871 | HGLibA_11884 | 39 | -0.781925116 | CXCR2 | C-X-C motif chemokine receptor 2 [Source:HGNC Symbol;Acc:HGNC:6027] |
| ENSG00000181785 | HGLibA_34250 | 39 | -0.781925116 | OR5AS1 | olfactory receptor family 5 subfamily AS member 1 [Source:HGNC Symbol;Acc:HGNC:15261] |
| ENSG00000182287 | HGLibA_02298 | 39 | -0.781925116 | AP1S2 | adaptor related protein complex 1 subunit sigma 2 [Source:HGNC Symbol;Acc:HGNC:560] |
| ENSG00000182511 | HGLibA_17303 | 39 | -0.781925116 | FES | tyrosine kinase [Source:HGNC Symbol;Acc:HGNC:3657]" |
| ENSG00000182749 | HGLibA_35168 | 39 | -0.781925116 | PAQR7 | progestin and adipoQ receptor family member 7 [Source:HGNC Symbol;Acc:HGNC:23146] |
| ENSG00000182985 | HGLibA_06966 | 39 | -0.781925116 | CADM1 | cell adhesion molecule 1 [Source:HGNC Symbol;Acc:HGNC:5951] |
| ENSG00000183049 | HGLibA_07061 | 39 | -0.781925116 | CAMK1D | calcium/calmodulin dependent protein kinase ID [Source:HGNC Symbol;Acc:HGNC:19341] |
| ENSG00000183495 | HGLibA_15232 | 39 | -0.781925116 | EP400 | E1A binding protein p400 [Source:HGNC Symbol;Acc:HGNC:11958] |
| ENSG00000184792 | HGLibA_34639 | 39 | -0.781925116 | OSBP2 | oxysterol binding protein 2 [Source:HGNC Symbol;Acc:HGNC:8504] |
| ENSG00000184831 | HGLibA_02552 | 39 | -0.781925116 | APOO | apolipoprotein O [Source:HGNC Symbol;Acc:HGNC:28727] |
| ENSG00000185332 | HGLibA_49851 | 39 | -0.781925116 | TMEM105 | TMEM105 long non-coding RNA [Source:HGNC Symbol;Acc:HGNC:26794] |
| ENSG00000185669 | HGLibA_45871 | 39 | -0.781925116 | SNAI3 | snail family transcriptional repressor 3 [Source:HGNC Symbol;Acc:HGNC:18411] |
| ENSG00000187109 | HGLibA_31111 | 39 | -0.781925116 | NAP1L1 | nucleosome assembly protein 1 like 1 [Source:HGNC Symbol;Acc:HGNC:7637] |
| ENSG00000187147 | HGLibA_41641 | 39 | -0.781925116 | RNF220 | ring finger protein 220 [Source:HGNC Symbol;Acc:HGNC:25552] |
| ENSG00000187486 | HGLibA_24253 | 39 | -0.781925116 | KCNJ11 | potassium voltage-gated channel subfamily J member 11 [Source:HGNC Symbol;Acc:HGNC:6257] |
| ENSG00000187848 | HGLibA_34848 | 39 | -0.781925116 | P2RX2 | purinergic receptor P2X 2 [Source:HGNC Symbol;Acc:HGNC:15459] |
| ENSG00000187944 | HGLibA_06161 | 39 | -0.781925116 | C2orf66 | chromosome 2 open reading frame 66 [Source:HGNC Symbol;Acc:HGNC:33809] |
| ENSG00000188001 | HGLibA_51180 | 39 | -0.781925116 | TPRG1 | tumor protein p63 regulated 1 [Source:HGNC Symbol;Acc:HGNC:24759] |
| ENSG00000188089 | HGLibA_36913 | 39 | -0.781925116 | PLA2G4E | phospholipase A2 group IVE [Source:HGNC Symbol;Acc:HGNC:24791] |
| ENSG00000188306 | HGLibA_27460 | 39 | -0.781925116 | LRRIQ4 | leucine rich repeats and IQ motif containing 4 [Source:HGNC Symbol;Acc:HGNC:34298] |
| ENSG00000188818 | HGLibA_55068 | 39 | -0.781925116 | ZDHHC11 | zinc finger DHHC-type containing 11 [Source:HGNC Symbol;Acc:HGNC:19158] |
| ENSG00000189045 | HGLibA_01950 | 39 | -0.781925116 | ANKDD1B | ankyrin repeat and death domain containing 1B [Source:HGNC Symbol;Acc:HGNC:32525] |
| ENSG00000189050 | HGLibA_41704 | 39 | -0.781925116 | RNFT1 | transmembrane 1 [Source:HGNC Symbol;Acc:HGNC:30206]" |
| ENSG00000189167 | HGLibA_54781 | 39 | -0.781925116 | ZAR1L | zygote arrest 1 like [Source:HGNC Symbol;Acc:HGNC:37116] |
| ENSG00000189241 | HGLibA_51980 | 39 | -0.781925116 | TSPYL1 | TSPY like 1 [Source:HGNC Symbol;Acc:HGNC:12382] |
| ENSG00000196187 | HGLibA_50473 | 39 | -0.781925116 | TMEM63A | transmembrane protein 63A [Source:HGNC Symbol;Acc:HGNC:29118] |
| ENSG00000196368 | HGLibA_32938 | 39 | -0.781925116 | NUDT11 | nudix hydrolase 11 [Source:HGNC Symbol;Acc:HGNC:18011] |
| ENSG00000197380 | HGLibA_12286 | 39 | -0.781925116 | DACT3 | dishevelled binding antagonist of beta catenin 3 [Source:HGNC Symbol;Acc:HGNC:30745] |
| ENSG00000198130 | HGLibA_21285 | 39 | -0.781925116 | HIBCH | 3-hydroxyisobutyryl-CoA hydrolase [Source:HGNC Symbol;Acc:HGNC:4908] |
| ENSG00000198189 | HGLibA_22175 | 39 | -0.781925116 | HSD17B11 | hydroxysteroid 17-beta dehydrogenase 11 [Source:HGNC Symbol;Acc:HGNC:22960] |
| ENSG00000198598 | HGLibA_29501 | 39 | -0.781925116 | MMP17 | matrix metallopeptidase 17 [Source:HGNC Symbol;Acc:HGNC:7163] |
| ENSG00000198746 | HGLibA_19745 | 39 | -0.781925116 | GPATCH3 | G-patch domain containing 3 [Source:HGNC Symbol;Acc:HGNC:25720] |
| ENSG00000198774 | HGLibA_40399 | 39 | -0.781925116 | RASSF9 | Ras association domain family member 9 [Source:HGNC Symbol;Acc:HGNC:15739] |
| ENSG00000198961 | HGLibA_36786 | 39 | -0.781925116 | PJA2 | praja ring finger ubiquitin ligase 2 [Source:HGNC Symbol;Acc:HGNC:17481] |
| ENSG00000203734 | HGLibA_14428 | 39 | -0.781925116 | ECT2L | epithelial cell transforming 2 like [Source:HGNC Symbol;Acc:HGNC:21118] |
| ENSG00000203760 | HGLibA_09092 | 39 | -0.781925116 | CENPW | centromere protein W [Source:HGNC Symbol;Acc:HGNC:21488] |
| ENSG00000204899 | HGLibA_30941 | 39 | -0.781925116 | MZT1 | mitotic spindle organizing protein 1 [Source:HGNC Symbol;Acc:HGNC:33830] |
| ENSG00000205476 | HGLibA_07897 | 39 | -0.781925116 | CCDC85C | coiled-coil domain containing 85C [Source:HGNC Symbol;Acc:HGNC:35459] |
| ENSG00000206107 | HGLibA_25735 | 39 | -0.781925116 | KRTAP27-1 | keratin associated protein 27-1 [Source:HGNC Symbol;Acc:HGNC:33864] |
| ENSG00000211456 | HGLibA_42626 | 39 | -0.781925116 | SACM1L | SAC1 like phosphatidylinositide phosphatase [Source:HGNC Symbol;Acc:HGNC:17059] |
| ENSG00000212935 | HGLibA_25606 | 39 | -0.781925116 | KRTAP10-3 | keratin associated protein 10-3 [Source:HGNC Symbol;Acc:HGNC:22968] |
| ENSG00000213593 | HGLibA_50692 | 39 | -0.781925116 | TMX2 | thioredoxin related transmembrane protein 2 [Source:HGNC Symbol;Acc:HGNC:30739] |
| ENSG00000214929 | HGLibA_46494 | 39 | -0.781925116 | SPATA31D1 | SPATA31 subfamily D member 1 [Source:HGNC Symbol;Acc:HGNC:37283] |
| ENSG00000233276 | HGLibA_20171 | 39 | -0.781925116 | GPX1 | glutathione peroxidase 1 [Source:HGNC Symbol;Acc:HGNC:4553] |
| ENSG00000234127 | HGLibA_51444 | 39 | -0.781925116 | TRIM26 | tripartite motif containing 26 [Source:HGNC Symbol;Acc:HGNC:12962] |
| ENSG00000236311 | HGLibA_49674 | 39 | -0.781925116 | TLX1NB | TLX1 neighbor [Source:HGNC Symbol;Acc:HGNC:37183] |
| ENSG00000237330 | HGLibA_41645 | 39 | -0.781925116 | RNF223 | ring finger protein 223 [Source:HGNC Symbol;Acc:HGNC:40020] |
| ENSG00000259823 | HGLibA_27733 | 39 | -0.781925116 | LYPD8 | LY6/PLAUR domain containing 8 [Source:HGNC Symbol;Acc:HGNC:44208] |
| ENSG00000261011 | HGLibA_01140 | 39 | -0.781925116 | AGAP11 | ankyrin repeat and PH domain family (AGAP) pseudogene" |
| ENSG00000261456 | HGLibA_52295 | 39 | -0.781925116 | TUBB8 | tubulin beta 8 class VIII [Source:HGNC Symbol;Acc:HGNC:20773] |
| ENSG00000261678 | HGLibA_43058 | 39 | -0.781925116 | SCRT1 | scratch family transcriptional repressor 1 [Source:HGNC Symbol;Acc:HGNC:15950] |
| ENSG00000262655 | HGLibA_46758 | 39 | -0.781925116 | SPON1 | spondin 1 [Source:HGNC Symbol;Acc:HGNC:11252] |
| ENSG00000272333 | HGLibA_25324 | 39 | -0.781925116 | KMT2B | lysine methyltransferase 2B [Source:HGNC Symbol;Acc:HGNC:15840] |
| EPT1 | HGLibA_15389 | 39 | -0.781925116 | EPT1 | - |
| HIST2H3D | HGLibA_21545 | 39 | -0.781925116 | HIST2H3D | - |
| hsa-mir-1243 | HGLibA_57357 | 39 | -0.781925116 | hsa-mir-1243 | - |
| hsa-mir-1302-1 | HGLibA_57682 | 39 | -0.781925116 | hsa-mir-1302-1 | - |
| hsa-mir-181c | HGLibA_57975 | 39 | -0.781925116 | hsa-mir-181c | - |
| hsa-mir-1915 | HGLibA_58065 | 39 | -0.781925116 | hsa-mir-1915 | - |
| hsa-mir-194-1 | HGLibA_58080 | 39 | -0.781925116 | hsa-mir-194-1 | - |
| hsa-mir-199a-1 | HGLibA_58128 | 39 | -0.781925116 | hsa-mir-199a-1 | - |
| hsa-mir-2392 | HGLibA_58325 | 39 | -0.781925116 | hsa-mir-2392 | - |
| hsa-mir-3074 | HGLibA_58468 | 39 | -0.781925116 | hsa-mir-3074 | - |
| hsa-mir-3133 | HGLibA_58587 | 39 | -0.781925116 | hsa-mir-3133 | - |
| hsa-mir-3180-3 | HGLibA_58811 | 39 | -0.781925116 | hsa-mir-3180-3 | - |
| hsa-mir-3690-2 | HGLibA_59348 | 39 | -0.781925116 | hsa-mir-3690-2 | - |
| hsa-mir-4463 | HGLibA_60259 | 39 | -0.781925116 | hsa-mir-4463 | - |
| hsa-mir-4468 | HGLibA_60280 | 39 | -0.781925116 | hsa-mir-4468 | - |
| hsa-mir-450a-1 | HGLibA_60474 | 39 | -0.781925116 | hsa-mir-450a-1 | - |
| hsa-mir-4675 | HGLibA_60817 | 39 | -0.781925116 | hsa-mir-4675 | - |
| hsa-mir-491 | HGLibA_61363 | 39 | -0.781925116 | hsa-mir-491 | - |
| hsa-mir-518a-2 | HGLibA_61656 | 39 | -0.781925116 | hsa-mir-518a-2 | - |
| hsa-mir-518f | HGLibA_61676 | 39 | -0.781925116 | hsa-mir-518f | - |
| hsa-mir-526a-1 | HGLibA_61791 | 39 | -0.781925116 | hsa-mir-526a-1 | - |
| hsa-mir-548f-2 | HGLibA_61976 | 39 | -0.781925116 | hsa-mir-548f-2 | - |
| hsa-mir-548k | HGLibA_62031 | 39 | -0.781925116 | hsa-mir-548k | - |
| hsa-mir-5584 | HGLibA_62179 | 39 | -0.781925116 | hsa-mir-5584 | - |
| hsa-mir-624 | HGLibA_62734 | 39 | -0.781925116 | hsa-mir-624 | - |
| hsa-mir-6820 | HGLibA_63459 | 39 | -0.781925116 | hsa-mir-6820 | - |
| hsa-mir-6839 | HGLibA_63533 | 39 | -0.781925116 | hsa-mir-6839 | - |
| hsa-mir-6856 | HGLibA_63595 | 39 | -0.781925116 | hsa-mir-6856 | - |
| hsa-mir-920 | HGLibA_64276 | 39 | -0.781925116 | hsa-mir-920 | - |
| hsa-mir-935 | HGLibA_64314 | 39 | -0.781925116 | hsa-mir-935 | - |
| hsa-mir-939 | HGLibA_64332 | 39 | -0.781925116 | hsa-mir-939 | - |
| hsa-mir-99b | HGLibA_64381 | 39 | -0.781925116 | hsa-mir-99b | - |
| LUZP6 | HGLibA_27648 | 39 | -0.781925116 | LUZP6 | - |
| OR5R1 | HGLibA_34351 | 39 | -0.781925116 | OR5R1 | - |
| OR9G9 | HGLibA_34575 | 39 | -0.781925116 | OR9G9 | - |
| PCNXL2 | HGLibA_35647 | 39 | -0.781925116 | PCNXL2 | - |
| SETD8 | HGLibA_43660 | 39 | -0.781925116 | SETD8 | - |
| SLC25A6_Y | HGLibA_44776 | 39 | -0.781925116 | SLC25A6_Y | - |
| AGAP7 | HGLibA_01157 | 38 | -0.790970459 | AGAP7 | - |
| BET3L | HGLibA_04437 | 38 | -0.790970459 | BET3L | - |
| C10orf118 | HGLibA_05041 | 38 | -0.790970459 | C10orf118 | - |
| C7orf43 | HGLibA_06546 | 38 | -0.790970459 | C7orf43 | - |
| CGB | HGLibA_09299 | 38 | -0.790970459 | CGB | - |
| DFNB31 | HGLibA_13028 | 38 | -0.790970459 | DFNB31 | - |
| ENSG00000001629 | HGLibA_01969 | 38 | -0.790970459 | ANKIB1 | ankyrin repeat and IBR domain containing 1 [Source:HGNC Symbol;Acc:HGNC:22215] |
| ENSG00000010270 | HGLibA_47360 | 38 | -0.790970459 | STARD3NL | STARD3 N-terminal like [Source:HGNC Symbol;Acc:HGNC:19169] |
| ENSG00000013619 | HGLibA_28052 | 38 | -0.790970459 | MAMLD1 | mastermind like domain containing 1 [Source:HGNC Symbol;Acc:HGNC:2568] |
| ENSG00000017483 | HGLibA_45072 | 38 | -0.790970459 | SLC38A5 | solute carrier family 38 member 5 [Source:HGNC Symbol;Acc:HGNC:18070] |
| ENSG00000068878 | HGLibA_39199 | 38 | -0.790970459 | PSME4 | proteasome activator subunit 4 [Source:HGNC Symbol;Acc:HGNC:20635] |
| ENSG00000070269 | HGLibA_50350 | 38 | -0.790970459 | TMEM260 | transmembrane protein 260 [Source:HGNC Symbol;Acc:HGNC:20185] |
| ENSG00000072401 | HGLibA_52583 | 38 | -0.790970459 | UBE2D1 | ubiquitin conjugating enzyme E2 D1 [Source:HGNC Symbol;Acc:HGNC:12474] |
| ENSG00000073536 | HGLibA_32063 | 38 | -0.790970459 | NLE1 | notchless homolog 1 [Source:HGNC Symbol;Acc:HGNC:19889] |
| ENSG00000073921 | HGLibA_36523 | 38 | -0.790970459 | PICALM | phosphatidylinositol binding clathrin assembly protein [Source:HGNC Symbol;Acc:HGNC:15514] |
| ENSG00000077279 | HGLibA_12579 | 38 | -0.790970459 | DCX | doublecortin [Source:HGNC Symbol;Acc:HGNC:2714] |
| ENSG00000080819 | HGLibA_10982 | 38 | -0.790970459 | CPOX | coproporphyrinogen oxidase [Source:HGNC Symbol;Acc:HGNC:2321] |
| ENSG00000081913 | HGLibA_36435 | 38 | -0.790970459 | PHLPP1 | PH domain and leucine rich repeat protein phosphatase 1 [Source:HGNC Symbol;Acc:HGNC:20610] |
| ENSG00000085840 | HGLibA_34600 | 38 | -0.790970459 | ORC1 | origin recognition complex subunit 1 [Source:HGNC Symbol;Acc:HGNC:8487] |
| ENSG00000089048 | HGLibA_15566 | 38 | -0.790970459 | ESF1 | ESF1 nucleolar pre-rRNA processing protein homolog [Source:HGNC Symbol;Acc:HGNC:15898] |
| ENSG00000095574 | HGLibA_22942 | 38 | -0.790970459 | IKZF5 | IKAROS family zinc finger 5 [Source:HGNC Symbol;Acc:HGNC:14283] |
| ENSG00000096401 | HGLibA_08589 | 38 | -0.790970459 | CDC5L | cell division cycle 5 like [Source:HGNC Symbol;Acc:HGNC:1743] |
| ENSG00000099246 | HGLibA_39823 | 38 | -0.790970459 | RAB18 | member RAS oncogene family [Source:HGNC Symbol;Acc:HGNC:14244]" |
| ENSG00000100448 | HGLibA_11706 | 38 | -0.790970459 | CTSG | cathepsin G [Source:HGNC Symbol;Acc:HGNC:2532] |
| ENSG00000100592 | HGLibA_12260 | 38 | -0.790970459 | DAAM1 | dishevelled associated activator of morphogenesis 1 [Source:HGNC Symbol;Acc:HGNC:18142] |
| ENSG00000100934 | HGLibA_43220 | 38 | -0.790970459 | SEC23A | coat complex II component [Source:HGNC Symbol;Acc:HGNC:10701]" |
| ENSG00000103037 | HGLibA_43656 | 38 | -0.790970459 | SETD6 | SET domain containing 6 [Source:HGNC Symbol;Acc:HGNC:26116] |
| ENSG00000103150 | HGLibA_29448 | 38 | -0.790970459 | MLYCD | malonyl-CoA decarboxylase [Source:HGNC Symbol;Acc:HGNC:7150] |
| ENSG00000103241 | HGLibA_17863 | 38 | -0.790970459 | FOXF1 | forkhead box F1 [Source:HGNC Symbol;Acc:HGNC:3809] |
| ENSG00000103496 | HGLibA_47648 | 38 | -0.790970459 | STX4 | syntaxin 4 [Source:HGNC Symbol;Acc:HGNC:11439] |
| ENSG00000104219 | HGLibA_55095 | 38 | -0.790970459 | ZDHHC2 | zinc finger DHHC-type containing 2 [Source:HGNC Symbol;Acc:HGNC:18469] |
| ENSG00000105321 | HGLibA_07920 | 38 | -0.790970459 | CCDC9 | coiled-coil domain containing 9 [Source:HGNC Symbol;Acc:HGNC:24560] |
| ENSG00000105609 | HGLibA_26519 | 38 | -0.790970459 | LILRB5 | leukocyte immunoglobulin like receptor B5 [Source:HGNC Symbol;Acc:HGNC:6609] |
| ENSG00000106648 | HGLibA_18579 | 38 | -0.790970459 | GALNTL5 | polypeptide N-acetylgalactosaminyltransferase like 5 [Source:HGNC Symbol;Acc:HGNC:21725] |
| ENSG00000106714 | HGLibA_10424 | 38 | -0.790970459 | CNTNAP3 | contactin associated protein like 3 [Source:HGNC Symbol;Acc:HGNC:13834] |
| ENSG00000107104 | HGLibA_24024 | 38 | -0.790970459 | KANK1 | KN motif and ankyrin repeat domains 1 [Source:HGNC Symbol;Acc:HGNC:19309] |
| ENSG00000107159 | HGLibA_06827 | 38 | -0.790970459 | CA9 | carbonic anhydrase 9 [Source:HGNC Symbol;Acc:HGNC:1383] |
| ENSG00000107371 | HGLibA_15778 | 38 | -0.790970459 | EXOSC3 | exosome component 3 [Source:HGNC Symbol;Acc:HGNC:17944] |
| ENSG00000108001 | HGLibA_14356 | 38 | -0.790970459 | EBF3 | EBF transcription factor 3 [Source:HGNC Symbol;Acc:HGNC:19087] |
| ENSG00000109339 | HGLibA_28251 | 38 | -0.790970459 | MAPK10 | mitogen-activated protein kinase 10 [Source:HGNC Symbol;Acc:HGNC:6872] |
| ENSG00000109991 | HGLibA_34852 | 38 | -0.790970459 | P2RX3 | purinergic receptor P2X 3 [Source:HGNC Symbol;Acc:HGNC:8534] |
| ENSG00000110536 | HGLibA_39426 | 38 | -0.790970459 | PTPMT1 | mitochondrial 1 [Source:HGNC Symbol;Acc:HGNC:26965]" |

| | | | | | |
|---|---|---|---|---|---|
| ENSG00000110628 | HGLibA_44560 | 38 | -0.790970459 | SLC22A18 | solute carrier family 22 member 18 [Source:HGNC Symbol;Acc:HGNC:10964] |
| ENSG00000110917 | HGLibA_29370 | 38 | -0.790970459 | MLEC | malectin [Source:HGNC Symbol;Acc:HGNC:28973] |
| ENSG00000111241 | HGLibA_17413 | 38 | -0.790970459 | FGF6 | fibroblast growth factor 6 [Source:HGNC Symbol;Acc:HGNC:3684] |
| ENSG00000111885 | HGLibA_28059 | 38 | -0.790970459 | MAN1A1 | mannosidase alpha class 1A member 1 [Source:HGNC Symbol;Acc:HGNC:6821] |
| ENSG00000112425 | HGLibA_15336 | 38 | -0.790970459 | EPM2A | laforin glucan phosphatase [Source:HGNC Symbol;Acc:HGNC:3413]" |
| ENSG00000113319 | HGLibA_40341 | 38 | -0.790970459 | RASGRF2 | Ras protein specific guanine nucleotide releasing factor 2 [Source:HGNC Symbol;Acc:HGNC:9876] |
| ENSG00000114745 | HGLibA_19679 | 38 | -0.790970459 | GORASP1 | golgi reassembly stacking protein 1 [Source:HGNC Symbol;Acc:HGNC:16769] |
| ENSG00000114993 | HGLibA_42388 | 38 | -0.790970459 | RTKN | rhotekin [Source:HGNC Symbol;Acc:HGNC:10466] |
| ENSG00000115165 | HGLibA_12248 | 38 | -0.790970459 | CYTIP | cytohesin 1 interacting protein [Source:HGNC Symbol;Acc:HGNC:9506] |
| ENSG00000116117 | HGLibA_35180 | 38 | -0.790970459 | PARD3B | par-3 family cell polarity regulator beta [Source:HGNC Symbol;Acc:HGNC:14446] |
| ENSG00000116748 | HGLibA_01822 | 38 | -0.790970459 | AMPD1 | adenosine monophosphate deaminase 1 [Source:HGNC Symbol;Acc:HGNC:468] |
| ENSG00000118705 | HGLibA_42020 | 38 | -0.790970459 | RPN2 | ribophorin II [Source:HGNC Symbol;Acc:HGNC:10382] |
| ENSG00000119865 | HGLibA_10380 | 38 | -0.790970459 | CNRIP1 | cannabinoid receptor interacting protein 1 [Source:HGNC Symbol;Acc:HGNC:24546] |
| ENSG00000120008 | HGLibA_54025 | 38 | -0.790970459 | WDR11 | WD repeat domain 11 [Source:HGNC Symbol;Acc:HGNC:13831] |
| ENSG00000121446 | HGLibA_41118 | 38 | -0.790970459 | RGSL1 | regulator of G protein signaling like 1 [Source:HGNC Symbol;Acc:HGNC:18636] |
| ENSG00000122145 | HGLibA_48664 | 38 | -0.790970459 | TBX22 | T-box 22 [Source:HGNC Symbol;Acc:HGNC:11600] |
| ENSG00000123472 | HGLibA_03799 | 38 | -0.790970459 | ATPAF1 | ATP synthase mitochondrial F1 complex assembly factor 1 [Source:HGNC Symbol;Acc:HGNC:18803] |
| ENSG00000124216 | HGLibA_45865 | 38 | -0.790970459 | SNAI1 | snail family transcriptional repressor 1 [Source:HGNC Symbol;Acc:HGNC:11128] |
| ENSG00000125482 | HGLibA_52169 | 38 | -0.790970459 | TTF1 | transcription termination factor 1 [Source:HGNC Symbol;Acc:HGNC:12397] |
| ENSG00000125630 | HGLibA_37552 | 38 | -0.790970459 | POLR1B | RNA polymerase I subunit B [Source:HGNC Symbol;Acc:HGNC:20454] |
| ENSG00000128573 | HGLibA_17934 | 38 | -0.790970459 | FOXP2 | forkhead box P2 [Source:HGNC Symbol;Acc:HGNC:13875] |
| ENSG00000128739 | HGLibA_45995 | 38 | -0.790970459 | SNRPN | small nuclear ribonucleoprotein polypeptide N [Source:HGNC Symbol;Acc:HGNC:11164] |
| ENSG00000128989 | HGLibA_03099 | 38 | -0.790970459 | ARPP19 | cAMP regulated phosphoprotein 19 [Source:HGNC Symbol;Acc:HGNC:16967] |
| ENSG00000129595 | HGLibA_15251 | 38 | -0.790970459 | EPB41L4A | erythrocyte membrane protein band 4.1 like 4A [Source:HGNC Symbol;Acc:HGNC:13278] |
| ENSG00000131778 | HGLibA_09386 | 38 | -0.790970459 | CHD1L | chromodomain helicase DNA binding protein 1 like [Source:HGNC Symbol;Acc:HGNC:1916] |
| ENSG00000131979 | HGLibA_18783 | 38 | -0.790970459 | GCH1 | GTP cyclohydrolase 1 [Source:HGNC Symbol;Acc:HGNC:4193] |
| ENSG00000132196 | HGLibA_22199 | 38 | -0.790970459 | HSD17B7 | hydroxysteroid 17-beta dehydrogenase 7 [Source:HGNC Symbol;Acc:HGNC:5215] |
| ENSG00000132819 | HGLibA_40540 | 38 | -0.790970459 | RBM38 | RNA binding motif protein 38 [Source:HGNC Symbol;Acc:HGNC:15818] |
| ENSG00000134200 | HGLibA_51857 | 38 | -0.790970459 | TSHB | thyroid stimulating hormone beta [Source:HGNC Symbol;Acc:HGNC:12372] |
| ENSG00000134222 | HGLibA_39231 | 38 | -0.790970459 | PSRC1 | proline and serine rich coiled-coil 1 [Source:HGNC Symbol;Acc:HGNC:24472] |
| ENSG00000134308 | HGLibA_54752 | 38 | -0.790970459 | YWHAQ | tyrosine 3-monooxygenase/tryptophan 5-monooxygenase activation protein theta [Source:HGNC Symbol;Acc:HGNC:12854] |
| ENSG00000134371 | HGLibA_08599 | 38 | -0.790970459 | CDC73 | cell division cycle 73 [Source:HGNC Symbol;Acc:HGNC:16783] |
| ENSG00000134874 | HGLibA_14297 | 38 | -0.790970459 | DZIP1 | DAZ interacting zinc finger protein 1 [Source:HGNC Symbol;Acc:HGNC:20908] |
| ENSG00000134962 | HGLibA_25024 | 38 | -0.790970459 | KLB | klotho beta [Source:HGNC Symbol;Acc:HGNC:15527] |
| ENSG00000135423 | HGLibA_19328 | 38 | -0.790970459 | GLS2 | glutaminase 2 [Source:HGNC Symbol;Acc:HGNC:29570] |
| ENSG00000135441 | HGLibA_04558 | 38 | -0.790970459 | BLOC1S1 | biogenesis of lysosomal organelles complex 1 subunit 1 [Source:HGNC Symbol;Acc:HGNC:4200] |
| ENSG00000137198 | HGLibA_19426 | 38 | -0.790970459 | GMPR | guanosine monophosphate reductase [Source:HGNC Symbol;Acc:HGNC:4376] |
| ENSG00000139318 | HGLibA_14186 | 38 | -0.790970459 | DUSP6 | dual specificity phosphatase 6 [Source:HGNC Symbol;Acc:HGNC:3072] |
| ENSG00000139985 | HGLibA_00754 | 38 | -0.790970459 | ADAM21 | ADAM metallopeptidase domain 21 [Source:HGNC Symbol;Acc:HGNC:200] |
| ENSG00000140374 | HGLibA_15619 | 38 | -0.790970459 | ETFA | electron transfer flavoprotein subunit alpha [Source:HGNC Symbol;Acc:HGNC:3481] |
| ENSG00000141012 | HGLibA_18524 | 38 | -0.790970459 | GALNS | galactosamine (N-acetyl)-6-sulfatase [Source:HGNC Symbol;Acc:HGNC:4122] |
| ENSG00000141219 | HGLibA_05582 | 38 | -0.790970459 | C17orf80 | chromosome 17 open reading frame 80 [Source:HGNC Symbol;Acc:HGNC:29601] |
| ENSG00000142347 | HGLibA_30836 | 38 | -0.790970459 | MYO1F | myosin IF [Source:HGNC Symbol;Acc:HGNC:7600] |
| ENSG00000142949 | HGLibA_39503 | 38 | -0.790970459 | PTPRF | receptor type F [Source:HGNC Symbol;Acc:HGNC:9670]" |
| ENSG00000143811 | HGLibA_39677 | 38 | -0.790970459 | PYCR2 | pyrroline-5-carboxylate reductase 2 [Source:HGNC Symbol;Acc:HGNC:30262] |
| ENSG00000143845 | HGLibA_15634 | 38 | -0.790970459 | ETNK2 | ethanolamine kinase 2 [Source:HGNC Symbol;Acc:HGNC:25575] |
| ENSG00000144228 | HGLibA_46766 | 38 | -0.790970459 | SPOPL | speckle type BTB/POZ protein like [Source:HGNC Symbol;Acc:HGNC:27934] |
| ENSG00000144852 | HGLibA_32590 | 38 | -0.790970459 | NR1I2 | nuclear receptor subfamily 1 group I member 2 [Source:HGNC Symbol;Acc:HGNC:7968] |
| ENSG00000145423 | HGLibA_43739 | 38 | -0.790970459 | SFRP2 | secreted frizzled related protein 2 [Source:HGNC Symbol;Acc:HGNC:10777] |
| ENSG00000145740 | HGLibA_44911 | 38 | -0.790970459 | SLC30A5 | solute carrier family 30 member 5 [Source:HGNC Symbol;Acc:HGNC:19089] |
| ENSG00000145888 | HGLibA_19298 | 38 | -0.790970459 | GLRA1 | glycine receptor alpha 1 [Source:HGNC Symbol;Acc:HGNC:4326] |
| ENSG00000147065 | HGLibA_30233 | 38 | -0.790970459 | MSN | moesin [Source:HGNC Symbol;Acc:HGNC:7373] |
| ENSG00000147596 | HGLibA_38378 | 38 | -0.790970459 | PRDM14 | PR/SET domain 14 [Source:HGNC Symbol;Acc:HGNC:14001] |
| ENSG00000147642 | HGLibA_47909 | 38 | -0.790970459 | SYBU | syntabulin [Source:HGNC Symbol;Acc:HGNC:26011] |
| ENSG00000147955 | HGLibA_44158 | 38 | -0.790970459 | SIGMAR1 | sigma non-opioid intracellular receptor 1 [Source:HGNC Symbol;Acc:HGNC:8157] |
| ENSG00000149187 | HGLibA_09005 | 38 | -0.790970459 | CELF1 | CUGBP Elav-like family member 1 [Source:HGNC Symbol;Acc:HGNC:2549] |
| ENSG00000149639 | HGLibA_46154 | 38 | -0.790970459 | SOGA1 | autophagy associated 1 [Source:HGNC Symbol;Acc:HGNC:16111]" |
| ENSG00000152270 | HGLibA_35783 | 38 | -0.790970459 | PDE3B | phosphodiesterase 3B [Source:HGNC Symbol;Acc:HGNC:8779] |
| ENSG00000152433 | HGLibA_56203 | 38 | -0.790970459 | ZNF547 | zinc finger protein 547 [Source:HGNC Symbol;Acc:HGNC:26432] |
| ENSG00000153002 | HGLibA_10905 | 38 | -0.790970459 | CPB1 | carboxypeptidase B1 [Source:HGNC Symbol;Acc:HGNC:2299] |
| ENSG00000153157 | HGLibA_47934 | 38 | -0.790970459 | SYCP2L | synaptonemal complex protein 2 like [Source:HGNC Symbol;Acc:HGNC:21537] |
| ENSG00000159111 | HGLibA_29891 | 38 | -0.790970459 | MRPL10 | mitochondrial ribosomal protein L10 [Source:HGNC Symbol;Acc:HGNC:14055] |
| ENSG00000159556 | HGLibA_23668 | 38 | -0.790970459 | ISL2 | ISL LIM homeobox 2 [Source:HGNC Symbol;Acc:HGNC:18524] |
| ENSG00000161277 | HGLibA_49314 | 38 | -0.790970459 | THAP8 | THAP domain containing 8 [Source:HGNC Symbol;Acc:HGNC:23191] |
| ENSG00000161677 | HGLibA_23976 | 38 | -0.790970459 | JOSD2 | Josephin domain containing 2 [Source:HGNC Symbol;Acc:HGNC:28853] |
| ENSG00000162390 | HGLibA_00471 | 38 | -0.790970459 | ACOT11 | acyl-CoA thioesterase 11 [Source:HGNC Symbol;Acc:HGNC:18156] |
| ENSG00000163577 | HGLibA_14857 | 38 | -0.790970459 | EIF5A2 | eukaryotic translation initiation factor 5A2 [Source:HGNC Symbol;Acc:HGNC:3301] |
| ENSG00000163635 | HGLibA_03850 | 38 | -0.790970459 | ATXN7 | ataxin 7 [Source:HGNC Symbol;Acc:HGNC:10560] |
| ENSG00000163781 | HGLibA_51008 | 38 | -0.790970459 | TOPBP1 | DNA topoisomerase II binding protein 1 [Source:HGNC Symbol;Acc:HGNC:17008] |
| ENSG00000164039 | HGLibA_04377 | 38 | -0.790970459 | BDH2 | 3-hydroxybutyrate dehydrogenase 2 [Source:HGNC Symbol;Acc:HGNC:32389] |
| ENSG00000164045 | HGLibA_08527 | 38 | -0.790970459 | CDC25A | cell division cycle 25A [Source:HGNC Symbol;Acc:HGNC:1725] |
| ENSG00000164300 | HGLibA_43496 | 38 | -0.790970459 | SERINC5 | serine incorporator 5 [Source:HGNC Symbol;Acc:HGNC:18825] |
| ENSG00000164691 | HGLibA_48266 | 38 | -0.790970459 | TAGAP | T cell activation RhoGTPase activating protein [Source:HGNC Symbol;Acc:HGNC:15669] |
| ENSG00000165792 | HGLibA_28968 | 38 | -0.790970459 | METTL17 | methyltransferase like 17 [Source:HGNC Symbol;Acc:HGNC:19280] |
| ENSG00000166569 | HGLibA_10941 | 38 | -0.790970459 | CPLX4 | complexin 4 [Source:HGNC Symbol;Acc:HGNC:24330] |
| ENSG00000167378 | HGLibA_23615 | 38 | -0.790970459 | IRGQ | immunity related GTPase Q [Source:HGNC Symbol;Acc:HGNC:24868] |
| ENSG00000167740 | HGLibA_11998 | 38 | -0.790970459 | CYB5D2 | cytochrome b5 domain containing 2 [Source:HGNC Symbol;Acc:HGNC:28471] |
| ENSG00000168092 | HGLibA_34991 | 38 | -0.790970459 | PAFAH1B2 | platelet activating factor acetylhydrolase 1b catalytic subunit 2 [Source:HGNC Symbol;Acc:HGNC:8575] |
| ENSG00000169249 | HGLibA_56980 | 38 | -0.790970459 | ZRSR2 | RNA binding motif and serine/arginine rich 2 [Source:HGNC Symbol;Acc:HGNC:23019]" |
| ENSG00000169313 | HGLibA_34876 | 38 | -0.790970459 | P2RY12 | purinergic receptor P2Y12 [Source:HGNC Symbol;Acc:HGNC:18124] |
| ENSG00000169718 | HGLibA_14113 | 38 | -0.790970459 | DUS1L | dihydrouridine synthase 1 like [Source:HGNC Symbol;Acc:HGNC:30086] |
| ENSG00000170180 | HGLibA_20696 | 38 | -0.790970459 | GYPA | glycophorin A (MNS blood group) [Source:HGNC Symbol;Acc:HGNC:4702] |
| ENSG00000170370 | HGLibA_15095 | 38 | -0.790970459 | EMX2 | empty spiracles homeobox 2 [Source:HGNC Symbol;Acc:HGNC:3341] |
| ENSG00000170561 | HGLibA_23630 | 38 | -0.790970459 | IRX2 | iroquois homeobox 2 [Source:HGNC Symbol;Acc:HGNC:14359] |
| ENSG00000170954 | HGLibA_55941 | 38 | -0.790970459 | ZNF415 | zinc finger protein 415 [Source:HGNC Symbol;Acc:HGNC:20636] |
| ENSG00000171481 | HGLibA_33689 | 38 | -0.790970459 | OR1L3 | olfactory receptor family 1 subfamily L member 3 [Source:HGNC Symbol;Acc:HGNC:8215] |
| ENSG00000171604 | HGLibA_11975 | 38 | -0.790970459 | CXXC5 | CXXC finger protein 5 [Source:HGNC Symbol;Acc:HGNC:26943] |
| ENSG00000171766 | HGLibA_18694 | 38 | -0.790970459 | GATM | glycine amidinotransferase [Source:HGNC Symbol;Acc:HGNC:4175] |
| ENSG00000171858 | HGLibA_42108 | 38 | -0.790970459 | RPS21 | ribosomal protein S21 [Source:HGNC Symbol;Acc:HGNC:10409] |
| ENSG00000171872 | HGLibA_25063 | 38 | -0.790970459 | KLF17 | Kruppel like factor 17 [Source:HGNC Symbol;Acc:HGNC:18830] |
| ENSG00000171988 | HGLibA_23952 | 38 | -0.790970459 | JMJD1C | jumonji domain containing 1C [Source:HGNC Symbol;Acc:HGNC:12313] |
| ENSG00000172575 | HGLibA_40345 | 38 | -0.790970459 | RASGRP1 | RAS guanyl releasing protein 1 [Source:HGNC Symbol;Acc:HGNC:9878] |
| ENSG00000172932 | HGLibA_02009 | 38 | -0.790970459 | ANKRD13D | ankyrin repeat domain 13D [Source:HGNC Symbol;Acc:HGNC:27880] |
| ENSG00000173852 | HGLibA_13905 | 38 | -0.790970459 | DPY19L1 | dpy-19 like C-mannosyltransferase 1 [Source:HGNC Symbol;Acc:HGNC:22205] |
| ENSG00000174953 | HGLibA_13190 | 38 | -0.790970459 | DHX36 | DEAH-box helicase 36 [Source:HGNC Symbol;Acc:HGNC:14410] |
| ENSG00000175087 | HGLibA_35889 | 38 | -0.790970459 | PDIK1L | PDLIM1 interacting kinase 1 like [Source:HGNC Symbol;Acc:HGNC:18981] |
| ENSG00000175793 | HGLibA_43727 | 38 | -0.790970459 | SFN | stratifin [Source:HGNC Symbol;Acc:HGNC:10773] |
| ENSG00000175899 | HGLibA_00007 | 38 | -0.790970459 | A2M | alpha-2-macroglobulin [Source:HGNC Symbol;Acc:HGNC:7] |
| ENSG00000175985 | HGLibA_37091 | 38 | -0.790970459 | PLEKHD1 | pleckstrin homology and coiled-coil domain containing D1 [Source:HGNC Symbol;Acc:HGNC:20148] |
| ENSG00000176732 | HGLibA_36184 | 38 | -0.790970459 | PFN4 | profilin family member 4 [Source:HGNC Symbol;Acc:HGNC:31103] |
| ENSG00000176974 | HGLibA_44060 | 38 | -0.790970459 | SHMT1 | serine hydroxymethyltransferase 1 [Source:HGNC Symbol;Acc:HGNC:10850] |
| ENSG00000177169 | HGLibA_52969 | 38 | -0.790970459 | ULK1 | unc-51 like autophagy activating kinase 1 [Source:HGNC Symbol;Acc:HGNC:12558] |
| ENSG00000177606 | HGLibA_24002 | 38 | -0.790970459 | JUN | AP-1 transcription factor subunit [Source:HGNC Symbol;Acc:HGNC:6204]" |
| ENSG00000178826 | HGLibA_49957 | 38 | -0.790970459 | TMEM139 | transmembrane protein 139 [Source:HGNC Symbol;Acc:HGNC:22058] |
| ENSG00000179021 | HGLibA_06243 | 38 | -0.790970459 | C3orf38 | chromosome 3 open reading frame 38 [Source:HGNC Symbol;Acc:HGNC:28384] |
| ENSG00000179044 | HGLibA_15740 | 38 | -0.790970459 | EXOC3L1 | exocyst complex component 3 like 1 [Source:HGNC Symbol;Acc:HGNC:27540] |
| ENSG00000179142 | HGLibA_12058 | 38 | -0.790970459 | CYP11B2 | cytochrome P450 family 11 subfamily B member 2 [Source:HGNC Symbol;Acc:HGNC:2592] |
| ENSG00000179242 | HGLibA_08679 | 38 | -0.790970459 | CDH4 | cadherin 4 [Source:HGNC Symbol;Acc:HGNC:1763] |
| ENSG00000179627 | HGLibA_54887 | 38 | -0.790970459 | ZBTB42 | zinc finger and BTB domain containing 42 [Source:HGNC Symbol;Acc:HGNC:32550] |
| ENSG00000179639 | HGLibA_17168 | 38 | -0.790970459 | FCER1A | Fc fragment of IgE receptor Ia [Source:HGNC Symbol;Acc:HGNC:3609] |
| ENSG00000180138 | HGLibA_11385 | 38 | -0.790970459 | CSNK1A1L | casein kinase 1 alpha 1 like [Source:HGNC Symbol;Acc:HGNC:20289] |
| ENSG00000180881 | HGLibA_07198 | 38 | -0.790970459 | CAPS2 | calcyphosine 2 [Source:HGNC Symbol;Acc:HGNC:16471] |
| ENSG00000181315 | HGLibA_55798 | 38 | -0.790970459 | ZNF322 | zinc finger protein 322 [Source:HGNC Symbol;Acc:HGNC:23640] |
| ENSG00000181513 | HGLibA_00400 | 38 | -0.790970459 | ACBD4 | acyl-CoA binding domain containing 4 [Source:HGNC Symbol;Acc:HGNC:23337] |
| ENSG00000181789 | HGLibA_10703 | 38 | -0.790970459 | COPG1 | coatomer protein complex subunit gamma 1 [Source:HGNC Symbol;Acc:HGNC:2236] |
| ENSG00000183873 | HGLibA_42998 | 38 | -0.790970459 | SCN5A | sodium voltage-gated channel alpha subunit 5 [Source:HGNC Symbol;Acc:HGNC:10593] |
| ENSG00000184979 | HGLibA_53289 | 38 | -0.790970459 | USP18 | ubiquitin specific peptidase 18 [Source:HGNC Symbol;Acc:HGNC:12616] |
| ENSG00000184986 | HGLibA_49901 | 38 | -0.790970459 | TMEM121 | transmembrane protein 121 [Source:HGNC Symbol;Acc:HGNC:20511] |
| ENSG00000185437 | HGLibA_43913 | 38 | -0.790970459 | SH3BGR | SH3 domain binding glutamate rich protein [Source:HGNC Symbol;Acc:HGNC:10822] |
| ENSG00000187140 | HGLibA_17833 | 38 | -0.790970459 | FOXD3 | forkhead box D3 [Source:HGNC Symbol;Acc:HGNC:3804] |
| ENSG00000188112 | HGLibA_06443 | 38 | -0.790970459 | C6orf132 | chromosome 6 open reading frame 132 [Source:HGNC Symbol;Acc:HGNC:21288] |
| ENSG00000188234 | HGLibA_01148 | 38 | -0.790970459 | AGAP4 | ankyrin repeat and PH domain 4 [Source:HGNC Symbol;Acc:HGNC:23459]" |
| ENSG00000188487 | HGLibA_23374 | 38 | -0.790970459 | INSC | spindle orientation adaptor protein [Source:HGNC Symbol;Acc:HGNC:33116]" |
| ENSG00000189186 | HGLibA_12463 | 38 | -0.790970459 | DCAF8L2 | DDB1 and CUL4 associated factor 8 like 2 [Source:HGNC Symbol;Acc:HGNC:31811] |
| ENSG00000189357 | HGLibA_46501 | 38 | -0.790970459 | SPATA31D4 | SPATA31 subfamily D member 4 [Source:HGNC Symbol;Acc:HGNC:38601] |
| ENSG00000196104 | HGLibA_46755 | 38 | -0.790970459 | SPOCK3 | cwcv and kazal like domains proteoglycan 3 [Source:HGNC Symbol;Acc:HGNC:13565]" |
| ENSG00000196335 | HGLibA_47480 | 38 | -0.790970459 | STK31 | serine/threonine kinase 31 [Source:HGNC Symbol;Acc:HGNC:11407] |
| ENSG00000196440 | HGLibA_03048 | 38 | -0.790970459 | ARMCX4 | armadillo repeat containing X-linked 4 [Source:HGNC Symbol;Acc:HGNC:28615] |
| ENSG00000196465 | HGLibA_30771 | 38 | -0.790970459 | MYL6B | myosin light chain 6B [Source:HGNC Symbol;Acc:HGNC:29823] |
| ENSG00000197408 | HGLibA_12113 | 38 | -0.790970459 | CYP2B6 | cytochrome P450 family 2 subfamily B member 6 [Source:HGNC Symbol;Acc:HGNC:2615] |
| ENSG00000197416 | HGLibA_15910 | 38 | -0.790970459 | FABP12 | fatty acid binding protein 12 [Source:HGNC Symbol;Acc:HGNC:34524] |
| ENSG00000197935 | HGLibA_55779 | 38 | -0.790970459 | ZNF311 | zinc finger protein 311 [Source:HGNC Symbol;Acc:HGNC:13847] |
| ENSG00000198034 | HGLibA_42144 | 38 | -0.790970459 | RPS4X | ribosomal protein S4 X-linked [Source:HGNC Symbol;Acc:HGNC:10424] |
| ENSG00000198270 | HGLibA_49885 | 38 | -0.790970459 | TMEM116 | transmembrane protein 116 [Source:HGNC Symbol;Acc:HGNC:25084] |
| ENSG00000198690 | HGLibA_16826 | 38 | -0.790970459 | FAN1 | FANCD2 and FANCI associated nuclease 1 [Source:HGNC Symbol;Acc:HGNC:29170] |

| | | | | | |
|---|---|---|---|---|---|
| ENSG00000198730 | HGLibA_11674 | 38 | -0.790970459 | CTR9 | Paf1/RNA polymerase II complex component [Source:HGNC Symbol;Acc:HGNC:16850]" |
| ENSG00000198836 | HGLibA_33403 | 38 | -0.790970459 | OPA1 | mitochondrial dynamin like GTPase [Source:HGNC Symbol;Acc:HGNC:8140]" |
| ENSG00000204104 | HGLibA_51250 | 38 | -0.790970459 | TRAF3IP1 | TRAF3 interacting protein 1 [Source:HGNC Symbol;Acc:HGNC:17861] |
| ENSG00000204544 | HGLibA_30540 | 38 | -0.790970459 | MUC21 | cell surface associated [Source:HGNC Symbol;Acc:HGNC:21661]" |
| ENSG00000205108 | HGLibA_16423 | 38 | -0.790970459 | FAM205A | family with sequence similarity 205 member A [Source:HGNC Symbol;Acc:HGNC:41911] |
| ENSG00000205442 | HGLibA_23897 | 38 | -0.790970459 | IZUMO3 | IZUMO family member 3 [Source:HGNC Symbol;Acc:HGNC:31421] |
| ENSG00000212916 | HGLibA_28113 | 38 | -0.790970459 | MAP10 | microtubule associated protein 10 [Source:HGNC Symbol;Acc:HGNC:29265] |
| ENSG00000213096 | HGLibA_55676 | 38 | -0.790970459 | ZNF254 | zinc finger protein 254 [Source:HGNC Symbol;Acc:HGNC:13047] |
| ENSG00000213714 | HGLibA_16442 | 38 | -0.790970459 | FAM209B | family with sequence similarity 209 member B [Source:HGNC Symbol;Acc:HGNC:16101] |
| ENSG00000213762 | HGLibA_55468 | 38 | -0.790970459 | ZNF134 | zinc finger protein 134 [Source:HGNC Symbol;Acc:HGNC:12918] |
| ENSG00000213903 | HGLibA_27584 | 38 | -0.790970459 | LTB4R | leukotriene B4 receptor [Source:HGNC Symbol;Acc:HGNC:6713] |
| ENSG00000214711 | HGLibA_07157 | 38 | -0.790970459 | CAPN14 | calpain 14 [Source:HGNC Symbol;Acc:HGNC:16664] |
| ENSG00000215440 | HGLibA_32430 | 38 | -0.790970459 | NPEPL1 | aminopeptidase like 1 [Source:HGNC Symbol;Acc:HGNC:16244] |
| ENSG00000227057 | HGLibA_54102 | 38 | -0.790970459 | WDR46 | WD repeat domain 46 [Source:HGNC Symbol;Acc:HGNC:13923] |
| ENSG00000233013 | HGLibA_16194 | 38 | -0.790970459 | FAM157B | family with sequence similarity 157 member B [Source:HGNC Symbol;Acc:HGNC:34080] |
| ENSG00000235169 | HGLibA_45716 | 38 | -0.790970459 | SMIM1 | small integral membrane protein 1 (Vel blood group) [Source:HGNC Symbol;Acc:HGNC:44204] |
| ENSG00000239789 | HGLibA_30046 | 38 | -0.790970459 | MRPS17 | mitochondrial ribosomal protein S17 [Source:HGNC Symbol;Acc:HGNC:14047] |
| ENSG00000240771 | HGLibA_02838 | 38 | -0.790970459 | ARHGEF25 | Rho guanine nucleotide exchange factor 25 [Source:HGNC Symbol;Acc:HGNC:30275] |
| ENSG00000244038 | HGLibA_12631 | 38 | -0.790970459 | DDOST | dolichyl-diphosphooligosaccharide--protein glycosyltransferase non-catalytic subunit [Source:HGNC Symbol;Acc:HGNC:2728] |
| ENSG00000244617 | HGLibA_03362 | 38 | -0.790970459 | ASPRV1 | aspartic peptidase retroviral like 1 [Source:HGNC Symbol;Acc:HGNC:26321] |
| ENSG00000248383 | HGLibA_35462 | 38 | -0.790970459 | PCDHAC1 | 1 [Source:HGNC Symbol;Acc:HGNC:8676]" |
| ENSG00000254852 | HGLibA_32465 | 38 | -0.790970459 | NPIPA2 | nuclear pore complex interacting protein family member A2 [Source:HGNC Symbol;Acc:HGNC:41979] |
| ENSG00000256222 | HGLibA_30478 | 38 | -0.790970459 | MTRNR2L3 | MT-RNR2 like 3 [Source:HGNC Symbol;Acc:HGNC:37157] |
| ENSG00000256713 | HGLibA_36193 | 38 | -0.790970459 | PGA5 | group I (pepsinogen A) [Source:HGNC Symbol;Acc:HGNC:8887]" |
| ENSG00000257529 | HGLibA_41959 | 38 | -0.790970459 | RPL36A-HNRNPH2 | RPL36A-HNRNPH2 readthrough [Source:HGNC Symbol;Acc:HGNC:48349] |
| ENSG00000258588 | HGLibA_51562 | 38 | -0.790970459 | TRIM6-TRIM34 | TRIM6-TRIM34 readthrough [Source:HGNC Symbol;Acc:HGNC:33440] |
| ENSG00000261796 | HGLibA_23697 | 38 | -0.790970459 | ISY1-RAB43 | ISY1-RAB43 readthrough [Source:HGNC Symbol;Acc:HGNC:42969] |
| ENSG00000279263 | HGLibA_33829 | 38 | -0.790970459 | OR2L8 | olfactory receptor family 2 subfamily L member 8 (gene/pseudogene) [Source:HGNC Symbol;Acc:HGNC:15014] |
| ENSG00000279270 | HGLibA_34204 | 38 | -0.790970459 | OR52R1 | olfactory receptor family 52 subfamily R member 1 (gene/pseudogene) [Source:HGNC Symbol;Acc:HGNC:15235] |
| FAM195A | HGLibA_16376 | 38 | -0.790970459 | FAM195A | - |
| GTPBP6_X | HGLibA_20616 | 38 | -0.790970459 | GTPBP6_X | - |
| HIST1H2AM | HGLibA_21408 | 38 | -0.790970459 | HIST1H2AM | - |
| HIST1H2BN | HGLibA_21449 | 38 | -0.790970459 | HIST1H2BN | - |
| hsa-mir-1257 | HGLibA_57434 | 38 | -0.790970459 | hsa-mir-1257 | - |
| hsa-mir-1324 | HGLibA_57769 | 38 | -0.790970459 | hsa-mir-1324 | - |
| hsa-mir-138-1 | HGLibA_57812 | 38 | -0.790970459 | hsa-mir-138-1 | - |
| hsa-mir-154 | HGLibA_57926 | 38 | -0.790970459 | hsa-mir-154 | - |
| hsa-mir-3121 | HGLibA_58535 | 38 | -0.790970459 | hsa-mir-3121 | - |
| hsa-mir-3156-1 | HGLibA_58687 | 38 | -0.790970459 | hsa-mir-3156-1 | - |
| hsa-mir-3187 | HGLibA_58845 | 38 | -0.790970459 | hsa-mir-3187 | - |
| hsa-mir-3619 | HGLibA_59107 | 38 | -0.790970459 | hsa-mir-3619 | - |
| hsa-mir-3652 | HGLibA_59154 | 38 | -0.790970459 | hsa-mir-3652 | - |
| hsa-mir-374a | HGLibA_59389 | 38 | -0.790970459 | hsa-mir-374a | - |
| hsa-mir-3926-2 | HGLibA_59582 | 38 | -0.790970459 | hsa-mir-3926-2 | - |
| hsa-mir-4267 | HGLibA_59774 | 38 | -0.790970459 | hsa-mir-4267 | - |
| hsa-mir-4492 | HGLibA_60385 | 38 | -0.790970459 | hsa-mir-4492 | - |
| hsa-mir-4534 | HGLibA_60594 | 38 | -0.790970459 | hsa-mir-4534 | - |
| hsa-mir-4652 | HGLibA_60716 | 38 | -0.790970459 | hsa-mir-4652 | - |
| hsa-mir-4669 | HGLibA_60793 | 38 | -0.790970459 | hsa-mir-4669 | - |
| hsa-mir-4743 | HGLibA_61069 | 38 | -0.790970459 | hsa-mir-4743 | - |
| hsa-mir-4774 | HGLibA_61199 | 38 | -0.790970459 | hsa-mir-4774 | - |
| hsa-mir-4776-1 | HGLibA_61208 | 38 | -0.790970459 | hsa-mir-4776-1 | - |
| hsa-mir-505 | HGLibA_61480 | 38 | -0.790970459 | hsa-mir-505 | - |
| hsa-mir-519b | HGLibA_61719 | 38 | -0.790970459 | hsa-mir-519b | - |
| hsa-mir-519c | HGLibA_61721 | 38 | -0.790970459 | hsa-mir-519c | - |
| hsa-mir-548aw | HGLibA_61935 | 38 | -0.790970459 | hsa-mir-548aw | - |
| hsa-mir-548o | HGLibA_62050 | 38 | -0.790970459 | hsa-mir-548o | - |
| hsa-mir-607 | HGLibA_62530 | 38 | -0.790970459 | hsa-mir-607 | - |
| hsa-mir-6507 | HGLibA_62878 | 38 | -0.790970459 | hsa-mir-6507 | - |
| hsa-mir-6514 | HGLibA_62929 | 38 | -0.790970459 | hsa-mir-6514 | - |
| hsa-mir-6720 | HGLibA_63041 | 38 | -0.790970459 | hsa-mir-6720 | - |
| hsa-mir-6769a | HGLibA_63236 | 38 | -0.790970459 | hsa-mir-6769a | - |
| hsa-mir-6791 | HGLibA_63342 | 38 | -0.790970459 | hsa-mir-6791 | - |
| hsa-mir-6823 | HGLibA_63468 | 38 | -0.790970459 | hsa-mir-6823 | - |
| hsa-mir-6829 | HGLibA_63494 | 38 | -0.790970459 | hsa-mir-6829 | - |
| hsa-mir-6888 | HGLibA_63735 | 38 | -0.790970459 | hsa-mir-6888 | - |
| hsa-mir-802 | HGLibA_64041 | 38 | -0.790970459 | hsa-mir-802 | - |
| hsa-mir-8063 | HGLibA_64091 | 38 | -0.790970459 | hsa-mir-8063 | - |
| hsa-mir-8088 | HGLibA_64193 | 38 | -0.790970459 | hsa-mir-8088 | - |
| hsa-mir-887 | HGLibA_64226 | 38 | -0.790970459 | hsa-mir-887 | - |
| LOC653486 | HGLibA_26987 | 38 | -0.790970459 | LOC653486 | - |
| NDNL2 | HGLibA_31405 | 38 | -0.790970459 | NDNL2 | - |
| PAPD4 | HGLibA_35122 | 38 | -0.790970459 | PAPD4 | - |
| PTPN20A | HGLibA_39451 | 38 | -0.790970459 | PTPN20A | - |
| RAB7L1 | HGLibA_39982 | 38 | -0.790970459 | RAB7L1 | - |
| RARRES3 | HGLibA_40286 | 38 | -0.790970459 | RARRES3 | - |
| TMEM246 | HGLibA_50302 | 38 | -0.790970459 | TMEM246 | - |
| TMEM57 | HGLibA_50457 | 38 | -0.790970459 | TMEM57 | - |
| WHSC1L1 | HGLibA_54296 | 38 | -0.790970459 | WHSC1L1 | - |
| ACPP | HGLibA_00524 | 37 | -0.800015802 | ACPP | - |
| AGAP8 | HGLibA_01160 | 37 | -0.800015802 | AGAP8 | - |
| AGPHD1 | HGLibA_01237 | 37 | -0.800015802 | AGPHD1 | - |
| APOBEC3A_B | HGLibA_02477 | 37 | -0.800015802 | APOBEC3A_B | - |
| C10orf35 | HGLibA_05070 | 37 | -0.800015802 | C10orf35 | - |
| C15orf57 | HGLibA_05421 | 37 | -0.800015802 | C15orf57 | - |
| C17orf112 | HGLibA_05531 | 37 | -0.800015802 | C17orf112 | - |
| C1orf228 | HGLibA_05840 | 37 | -0.800015802 | C1orf228 | - |
| C6orf222 | HGLibA_06477 | 37 | -0.800015802 | C6orf222 | - |
| CENPC1 | HGLibA_09049 | 37 | -0.800015802 | CENPC1 | - |
| CT45A4 | HGLibA_11506 | 37 | -0.800015802 | CT45A4 | - |
| ENSG00000005022 | HGLibA_44762 | 37 | -0.800015802 | SLC25A5 | solute carrier family 25 member 5 [Source:HGNC Symbol;Acc:HGNC:10991] |
| ENSG00000005243 | HGLibA_10740 | 37 | -0.800015802 | COPZ2 | coatomer protein complex subunit zeta 2 [Source:HGNC Symbol;Acc:HGNC:19356] |
| ENSG00000010017 | HGLibA_40211 | 37 | -0.800015802 | RANBP9 | RAN binding protein 9 [Source:HGNC Symbol;Acc:HGNC:13727] |
| ENSG00000031081 | HGLibA_02755 | 37 | -0.800015802 | ARHGAP31 | Rho GTPase activating protein 31 [Source:HGNC Symbol;Acc:HGNC:29216] |
| ENSG00000042088 | HGLibA_48891 | 37 | -0.800015802 | TDP1 | tyrosyl-DNA phosphodiesterase 1 [Source:HGNC Symbol;Acc:HGNC:18884] |
| ENSG00000042088 | HGLibA_48890 | 37 | -0.800015802 | TDP1 | tyrosyl-DNA phosphodiesterase 1 [Source:HGNC Symbol;Acc:HGNC:18884] |
| ENSG00000048740 | HGLibA_09006 | 37 | -0.800015802 | CELF2 | CUGBP Elav-like family member 2 [Source:HGNC Symbol;Acc:HGNC:2550] |
| ENSG00000053371 | HGLibA_01499 | 37 | -0.800015802 | AKR7A2 | aldo-keto reductase family 7 member A2 [Source:HGNC Symbol;Acc:HGNC:389] |
| ENSG00000053501 | HGLibA_53163 | 37 | -0.800015802 | USE1 | unconventional SNARE in the ER 1 [Source:HGNC Symbol;Acc:HGNC:30882] |
| ENSG00000065717 | HGLibA_49610 | 37 | -0.800015802 | TLE2 | transducin like enhancer of split 2 [Source:HGNC Symbol;Acc:HGNC:11838] |
| ENSG00000066557 | HGLibA_27353 | 37 | -0.800015802 | LRRC40 | leucine rich repeat containing 40 [Source:HGNC Symbol;Acc:HGNC:26004] |
| ENSG00000067248 | HGLibA_13173 | 37 | -0.800015802 | DHX29 | DExH-box helicase 29 [Source:HGNC Symbol;Acc:HGNC:15815] |
| ENSG00000067334 | HGLibA_13723 | 37 | -0.800015802 | DNTTIP2 | deoxynucleotidyltransferase terminal interacting protein 2 [Source:HGNC Symbol;Acc:HGNC:24013] |
| ENSG00000068489 | HGLibA_38769 | 37 | -0.800015802 | PRR11 | proline rich 11 [Source:HGNC Symbol;Acc:HGNC:25619] |
| ENSG00000070182 | HGLibA_46878 | 37 | -0.800015802 | SPTB | erythrocytic [Source:HGNC Symbol;Acc:HGNC:11274]" |
| ENSG00000072506 | HGLibA_22171 | 37 | -0.800015802 | HSD17B10 | hydroxysteroid 17-beta dehydrogenase 10 [Source:HGNC Symbol;Acc:HGNC:4800] |
| ENSG00000073464 | HGLibA_09830 | 37 | -0.800015802 | CLCN4 | chloride voltage-gated channel 4 [Source:HGNC Symbol;Acc:HGNC:2022] |
| ENSG00000075856 | HGLibA_42758 | 37 | -0.800015802 | SART3 | U4/U6 recycling protein [Source:HGNC Symbol;Acc:HGNC:16860]" |
| ENSG00000077616 | HGLibA_31010 | 37 | -0.800015802 | NAALAD2 | N-acetylated alpha-linked acidic dipeptidase 2 [Source:HGNC Symbol;Acc:HGNC:14526] |
| ENSG00000078124 | HGLibA_00433 | 37 | -0.800015802 | ACER3 | alkaline ceramidase 3 [Source:HGNC Symbol;Acc:HGNC:16066] |
| ENSG00000078668 | HGLibA_53606 | 37 | -0.800015802 | VDAC3 | voltage dependent anion channel 3 [Source:HGNC Symbol;Acc:HGNC:12674] |
| ENSG00000079616 | HGLibA_24905 | 37 | -0.800015802 | KIF22 | kinesin family member 22 [Source:HGNC Symbol;Acc:HGNC:6391] |
| ENSG00000084463 | HGLibA_53970 | 37 | -0.800015802 | WBP11 | WW domain binding protein 11 [Source:HGNC Symbol;Acc:HGNC:16461] |
| ENSG00000088888 | HGLibA_28444 | 37 | -0.800015802 | MAVS | mitochondrial antiviral signaling protein [Source:HGNC Symbol;Acc:HGNC:29233] |
| ENSG00000089737 | HGLibA_12677 | 37 | -0.800015802 | DDX24 | DEAD-box helicase 24 [Source:HGNC Symbol;Acc:HGNC:13266] |
| ENSG00000090520 | HGLibA_13524 | 37 | -0.800015802 | DNAJB11 | DnaJ heat shock protein family (Hsp40) member B11 [Source:HGNC Symbol;Acc:HGNC:14889] |
| ENSG00000090661 | HGLibA_09209 | 37 | -0.800015802 | CERS4 | ceramide synthase 4 [Source:HGNC Symbol;Acc:HGNC:23747] |
| ENSG00000090863 | HGLibA_19243 | 37 | -0.800015802 | GLG1 | golgi glycoprotein 1 [Source:HGNC Symbol;Acc:HGNC:4316] |
| ENSG00000091039 | HGLibA_34668 | 37 | -0.800015802 | OSBPL8 | oxysterol binding protein like 8 [Source:HGNC Symbol;Acc:HGNC:16396] |
| ENSG00000092964 | HGLibA_13927 | 37 | -0.800015802 | DPYSL2 | dihydropyrimidinase like 2 [Source:HGNC Symbol;Acc:HGNC:3014] |
| ENSG00000094963 | HGLibA_17704 | 37 | -0.800015802 | FMO2 | flavin containing monooxygenase 2 [Source:HGNC Symbol;Acc:HGNC:3770] |
| ENSG00000100290 | HGLibA_04504 | 37 | -0.800015802 | BIK | BCL2 interacting killer [Source:HGNC Symbol;Acc:HGNC:1051] |
| ENSG00000101695 | HGLibA_41493 | 37 | -0.800015802 | RNF125 | ring finger protein 125 [Source:HGNC Symbol;Acc:HGNC:21150] |
| ENSG00000101977 | HGLibA_28576 | 37 | -0.800015802 | MCF2 | MCF.2 cell line derived transforming sequence [Source:HGNC Symbol;Acc:HGNC:6940] |
| ENSG00000102572 | HGLibA_47470 | 37 | -0.800015802 | STK24 | serine/threonine kinase 24 [Source:HGNC Symbol;Acc:HGNC:11403] |
| ENSG00000102970 | HGLibA_07978 | 37 | -0.800015802 | CCL17 | C-C motif chemokine ligand 17 [Source:HGNC Symbol;Acc:HGNC:10615] |
| ENSG00000104218 | HGLibA_11419 | 37 | -0.800015802 | CSPP1 | centrosome and spindle pole associated protein 1 [Source:HGNC Symbol;Acc:HGNC:26193] |
| ENSG00000104321 | HGLibA_51726 | 37 | -0.800015802 | TRPA1 | transient receptor potential cation channel subfamily A member 1 [Source:HGNC Symbol;Acc:HGNC:497] |
| ENSG00000104852 | HGLibA_45958 | 37 | -0.800015802 | SNRNP70 | small nuclear ribonucleoprotein U1 subunit 70 [Source:HGNC Symbol;Acc:HGNC:11150] |
| ENSG00000104879 | HGLibA_09776 | 37 | -0.800015802 | CKM | M-type [Source:HGNC Symbol;Acc:HGNC:1994]" |
| ENSG00000105204 | HGLibA_14273 | 37 | -0.800015802 | DYRK1B | dual specificity tyrosine phosphorylation regulated kinase 1B [Source:HGNC Symbol;Acc:HGNC:3092] |
| ENSG00000107140 | HGLibA_49029 | 37 | -0.800015802 | TESK1 | testis associated actin remodelling kinase 1 [Source:HGNC Symbol;Acc:HGNC:11731] |
| ENSG00000108590 | HGLibA_28806 | 37 | -0.800015802 | MED31 | mediator complex subunit 31 [Source:HGNC Symbol;Acc:HGNC:24260] |
| ENSG00000108669 | HGLibA_12234 | 37 | -0.800015802 | CYTH1 | cytohesin 1 [Source:HGNC Symbol;Acc:HGNC:9501] |
| ENSG00000109519 | HGLibA_20372 | 37 | -0.800015802 | GRPEL1 | mitochondrial [Source:HGNC Symbol;Acc:HGNC:19696]" |

| | | | | | |
|---|---|---|---|---|---|
| ENSG00000111203 | HGLibA_23709 | 37 | -0.800015802 | ITFG2 | integrin alpha FG-GAP repeat containing 2 [Source:HGNC Symbol;Acc:HGNC:30879] |
| ENSG00000111262 | HGLibA_24123 | 37 | -0.800015802 | KCNA1 | potassium voltage-gated channel subfamily A member 1 [Source:HGNC Symbol;Acc:HGNC:6218] |
| ENSG00000111291 | HGLibA_20135 | 37 | -0.800015802 | GPRC5D | G protein-coupled receptor class C group 5 member D [Source:HGNC Symbol;Acc:HGNC:13310] |
| ENSG00000111615 | HGLibA_25410 | 37 | -0.800015802 | KRR1 | small subunit processome component homolog [Source:HGNC Symbol;Acc:HGNC:5176]" |
| ENSG00000111725 | HGLibA_38498 | 37 | -0.800015802 | PRKAB1 | protein kinase AMP-activated non-catalytic subunit beta 1 [Source:HGNC Symbol;Acc:HGNC:9378] |
| ENSG00000112541 | HGLibA_35761 | 37 | -0.800015802 | PDE10A | phosphodiesterase 10A [Source:HGNC Symbol;Acc:HGNC:8772] |
| ENSG00000113805 | HGLibA_10408 | 37 | -0.800015802 | CNTN3 | contactin 3 [Source:HGNC Symbol;Acc:HGNC:2173] |
| ENSG00000115310 | HGLibA_42407 | 37 | -0.800015802 | RTN4 | reticulon 4 [Source:HGNC Symbol;Acc:HGNC:14085] |
| ENSG00000115317 | HGLibA_22411 | 37 | -0.800015802 | HTRA2 | HtrA serine peptidase 2 [Source:HGNC Symbol;Acc:HGNC:14348] |
| ENSG00000116406 | HGLibA_14459 | 37 | -0.800015802 | EDEM3 | ER degradation enhancing alpha-mannosidase like protein 3 [Source:HGNC Symbol;Acc:HGNC:16787] |
| ENSG00000117479 | HGLibA_44501 | 37 | -0.800015802 | SLC19A2 | solute carrier family 19 member 2 [Source:HGNC Symbol;Acc:HGNC:10938] |
| ENSG00000118971 | HGLibA_08074 | 37 | -0.800015802 | CCND2 | cyclin D2 [Source:HGNC Symbol;Acc:HGNC:1583] |
| ENSG00000119509 | HGLibA_23443 | 37 | -0.800015802 | INVS | inversin [Source:HGNC Symbol;Acc:HGNC:17870] |
| ENSG00000120053 | HGLibA_19690 | 37 | -0.800015802 | GOT1 | glutamic-oxaloacetic transaminase 1 [Source:HGNC Symbol;Acc:HGNC:4432] |
| ENSG00000120211 | HGLibA_23384 | 37 | -0.800015802 | INSL4 | insulin like 4 [Source:HGNC Symbol;Acc:HGNC:6087] |
| ENSG00000121775 | HGLibA_50386 | 37 | -0.800015802 | TMEM39B | transmembrane protein 39B [Source:HGNC Symbol;Acc:HGNC:25510] |
| ENSG00000122224 | HGLibA_27690 | 37 | -0.800015802 | LY9 | lymphocyte antigen 9 [Source:HGNC Symbol;Acc:HGNC:6730] |
| ENSG00000122859 | HGLibA_31751 | 37 | -0.800015802 | NEUROG3 | neurogenin 3 [Source:HGNC Symbol;Acc:HGNC:13806] |
| ENSG00000123091 | HGLibA_41462 | 37 | -0.800015802 | RNF11 | ring finger protein 11 [Source:HGNC Symbol;Acc:HGNC:10056] |
| ENSG00000123338 | HGLibA_31314 | 37 | -0.800015802 | NCKAP1L | NCK associated protein 1 like [Source:HGNC Symbol;Acc:HGNC:4862] |
| ENSG00000123737 | HGLibA_15797 | 37 | -0.800015802 | EXOSC9 | exosome component 9 [Source:HGNC Symbol;Acc:HGNC:9137] |
| ENSG00000124614 | HGLibA_42058 | 37 | -0.800015802 | RPS10 | ribosomal protein S10 [Source:HGNC Symbol;Acc:HGNC:10383] |
| ENSG00000124743 | HGLibA_25184 | 37 | -0.800015802 | KLHL31 | kelch like family member 31 [Source:HGNC Symbol;Acc:HGNC:21353] |
| ENSG00000124784 | HGLibA_41308 | 37 | -0.800015802 | RIOK1 | RIO kinase 1 [Source:HGNC Symbol;Acc:HGNC:18656] |
| ENSG00000125508 | HGLibA_46996 | 37 | -0.800015802 | SRMS | src-related kinase lacking C-terminal regulatory tyrosine and N-terminal myristylation sites [Source:HGNC Symbol;Acc:HGNC:11298] |
| ENSG00000125633 | HGLibA_07932 | 37 | -0.800015802 | CCDC93 | coiled-coil domain containing 93 [Source:HGNC Symbol;Acc:HGNC:25611] |
| ENSG00000125900 | HGLibA_44210 | 37 | -0.800015802 | SIRPD | signal regulatory protein delta [Source:HGNC Symbol;Acc:HGNC:16248] |
| ENSG00000126603 | HGLibA_19275 | 37 | -0.800015802 | GLIS2 | GLIS family zinc finger 2 [Source:HGNC Symbol;Acc:HGNC:29450] |
| ENSG00000126733 | HGLibA_12276 | 37 | -0.800015802 | DACH2 | dachshund family transcription factor 2 [Source:HGNC Symbol;Acc:HGNC:16814] |
| ENSG00000128191 | HGLibA_13054 | 37 | -0.800015802 | DGCR8 | microprocessor complex subunit [Source:HGNC Symbol;Acc:HGNC:2847]" |
| ENSG00000128335 | HGLibA_02530 | 37 | -0.800015802 | APOL2 | apolipoprotein L2 [Source:HGNC Symbol;Acc:HGNC:619] |
| ENSG00000128714 | HGLibA_21994 | 37 | -0.800015802 | HOXD13 | homeobox D13 [Source:HGNC Symbol;Acc:HGNC:5136] |
| ENSG00000129965 | HGLibA_23370 | 37 | -0.800015802 | INS-IGF2 | INS-IGF2 readthrough [Source:HGNC Symbol;Acc:HGNC:33527] |
| ENSG00000130348 | HGLibA_39747 | 37 | -0.800015802 | QRSL1 | glutaminyl-tRNA amidotransferase subunit A [Source:HGNC Symbol;Acc:HGNC:21020]" |
| ENSG00000130368 | HGLibA_28389 | 37 | -0.800015802 | MAS1 | G protein-coupled receptor [Source:HGNC Symbol;Acc:HGNC:6899]" |
| ENSG00000132938 | HGLibA_30509 | 37 | -0.800015802 | MTUS2 | microtubule associated scaffold protein 2 [Source:HGNC Symbol;Acc:HGNC:20595] |
| ENSG00000133835 | HGLibA_22192 | 37 | -0.800015802 | HSD17B4 | hydroxysteroid 17-beta dehydrogenase 4 [Source:HGNC Symbol;Acc:HGNC:5213] |
| ENSG00000134070 | HGLibA_23560 | 37 | -0.800015802 | IRAK2 | interleukin 1 receptor associated kinase 2 [Source:HGNC Symbol;Acc:HGNC:6113] |
| ENSG00000134490 | HGLibA_50289 | 37 | -0.800015802 | TMEM241 | transmembrane protein 241 [Source:HGNC Symbol;Acc:HGNC:31723] |
| ENSG00000134538 | HGLibA_45448 | 37 | -0.800015802 | SLCO1B1 | solute carrier organic anion transporter family member 1B1 [Source:HGNC Symbol;Acc:HGNC:10959] |
| ENSG00000134758 | HGLibA_41513 | 37 | -0.800015802 | RNF138 | ring finger protein 138 [Source:HGNC Symbol;Acc:HGNC:17765] |
| ENSG00000134825 | HGLibA_50343 | 37 | -0.800015802 | TMEM258 | transmembrane protein 258 [Source:HGNC Symbol;Acc:HGNC:1164] |
| ENSG00000134910 | HGLibA_47607 | 37 | -0.800015802 | STT3A | catalytic subunit of the oligosaccharyltransferase complex [Source:HGNC Symbol;Acc:HGNC:6172]" |
| ENSG00000134982 | HGLibA_02388 | 37 | -0.800015802 | APC | WNT signaling pathway regulator [Source:HGNC Symbol;Acc:HGNC:583]" |
| ENSG00000135185 | HGLibA_50293 | 37 | -0.800015802 | TMEM243 | transmembrane protein 243 [Source:HGNC Symbol;Acc:HGNC:21707] |
| ENSG00000135185 | HGLibA_50294 | 37 | -0.800015802 | TMEM243 | transmembrane protein 243 [Source:HGNC Symbol;Acc:HGNC:21707] |
| ENSG00000135824 | HGLibA_41108 | 37 | -0.800015802 | RGS8 | regulator of G protein signaling 8 [Source:HGNC Symbol;Acc:HGNC:16810] |
| ENSG00000135870 | HGLibA_40663 | 37 | -0.800015802 | RC3H1 | ring finger and CCCH-type domains 1 [Source:HGNC Symbol;Acc:HGNC:29434] |
| ENSG00000135905 | HGLibA_13734 | 37 | -0.800015802 | DOCK10 | dedicator of cytokinesis 10 [Source:HGNC Symbol;Acc:HGNC:23479] |
| ENSG00000136573 | HGLibA_04546 | 37 | -0.800015802 | BLK | Src family tyrosine kinase [Source:HGNC Symbol;Acc:HGNC:1057]" |
| ENSG00000137404 | HGLibA_32705 | 37 | -0.800015802 | NRM | nurim [Source:HGNC Symbol;Acc:HGNC:8003] |
| ENSG00000138079 | HGLibA_45130 | 37 | -0.800015802 | SLC3A1 | solute carrier family 3 member 1 [Source:HGNC Symbol;Acc:HGNC:11025] |
| ENSG00000138085 | HGLibA_03812 | 37 | -0.800015802 | ATRAID | all-trans retinoic acid induced differentiation factor [Source:HGNC Symbol;Acc:HGNC:24090] |
| ENSG00000138347 | HGLibA_30910 | 37 | -0.800015802 | MYPN | myopalladin [Source:HGNC Symbol;Acc:HGNC:23246] |
| ENSG00000139154 | HGLibA_01088 | 37 | -0.800015802 | AEBP2 | AE binding protein 2 [Source:HGNC Symbol;Acc:HGNC:24051] |
| ENSG00000139626 | HGLibA_23795 | 37 | -0.800015802 | ITGB7 | integrin subunit beta 7 [Source:HGNC Symbol;Acc:HGNC:6162] |
| ENSG00000140280 | HGLibA_27766 | 37 | -0.800015802 | LYSMD2 | LysM domain containing 2 [Source:HGNC Symbol;Acc:HGNC:28571] |
| ENSG00000140470 | HGLibA_00811 | 37 | -0.800015802 | ADAMTS17 | ADAM metallopeptidase with thrombospondin type 1 motif 17 [Source:HGNC Symbol;Acc:HGNC:17109] |
| ENSG00000141933 | HGLibA_51123 | 37 | -0.800015802 | TPGS1 | tubulin polyglutamylase complex subunit 1 [Source:HGNC Symbol;Acc:HGNC:25058] |
| ENSG00000143179 | HGLibA_52814 | 37 | -0.800015802 | UCK2 | uridine-cytidine kinase 2 [Source:HGNC Symbol;Acc:HGNC:12562] |
| ENSG00000143199 | HGLibA_00908 | 37 | -0.800015802 | ADCY10 | adenylate cyclase 10 [Source:HGNC Symbol;Acc:HGNC:21285] |
| ENSG00000143319 | HGLibA_23662 | 37 | -0.800015802 | ISG20L2 | interferon stimulated exonuclease gene 20 like 2 [Source:HGNC Symbol;Acc:HGNC:25745] |
| ENSG00000143469 | HGLibA_48060 | 37 | -0.800015802 | SYT14 | synaptotagmin 14 [Source:HGNC Symbol;Acc:HGNC:23143] |
| ENSG00000143643 | HGLibA_52052 | 37 | -0.800015802 | TTC13 | tetratricopeptide repeat domain 13 [Source:HGNC Symbol;Acc:HGNC:26204] |
| ENSG00000144224 | HGLibA_52788 | 37 | -0.800015802 | UBXN4 | UBX domain protein 4 [Source:HGNC Symbol;Acc:HGNC:14860] |
| ENSG00000144712 | HGLibA_07123 | 37 | -0.800015802 | CAND2 | cullin associated and neddylation dissociated 2 (putative) [Source:HGNC Symbol;Acc:HGNC:30689] |
| ENSG00000144843 | HGLibA_01024 | 37 | -0.800015802 | ADPRH | ADP-ribosylarginine hydrolase [Source:HGNC Symbol;Acc:HGNC:269] |
| ENSG00000145284 | HGLibA_42887 | 37 | -0.800015802 | SCD5 | stearoyl-CoA desaturase 5 [Source:HGNC Symbol;Acc:HGNC:21088] |
| ENSG00000145365 | HGLibA_49457 | 37 | -0.800015802 | TIFA | TRAF interacting protein with forkhead associated domain [Source:HGNC Symbol;Acc:HGNC:19075] |
| ENSG00000145423 | HGLibA_43740 | 37 | -0.800015802 | SFRP2 | secreted frizzled related protein 2 [Source:HGNC Symbol;Acc:HGNC:10777] |
| ENSG00000147044 | HGLibA_07285 | 37 | -0.800015802 | CASK | calcium/calmodulin dependent serine protein kinase [Source:HGNC Symbol;Acc:HGNC:1497] |
| ENSG00000147246 | HGLibA_22376 | 37 | -0.800015802 | HTR2C | 5-hydroxytryptamine receptor 2C [Source:HGNC Symbol;Acc:HGNC:5295] |
| ENSG00000148204 | HGLibA_11073 | 37 | -0.800015802 | CRB2 | cell polarity complex component [Source:HGNC Symbol;Acc:HGNC:18688]" |
| ENSG00000148943 | HGLibA_26578 | 37 | -0.800015802 | LIN7C | crumbs cell polarity complex component [Source:HGNC Symbol;Acc:HGNC:17789]" |
| ENSG00000149403 | HGLibA_20289 | 37 | -0.800015802 | GRIK4 | glutamate ionotropic receptor kainate type subunit 4 [Source:HGNC Symbol;Acc:HGNC:4582] |
| ENSG00000149499 | HGLibA_15061 | 37 | -0.800015802 | EML3 | echinoderm microtubule associated protein like 3 [Source:HGNC Symbol;Acc:HGNC:26666] |
| ENSG00000151458 | HGLibA_02114 | 37 | -0.800015802 | ANKRD50 | ankyrin repeat domain 50 [Source:HGNC Symbol;Acc:HGNC:29223] |
| ENSG00000151552 | HGLibA_39722 | 37 | -0.800015802 | QDPR | quinoid dihydropteridine reductase [Source:HGNC Symbol;Acc:HGNC:9752] |
| ENSG00000151914 | HGLibA_14044 | 37 | -0.800015802 | DST | dystonin [Source:HGNC Symbol;Acc:HGNC:1090] |
| ENSG00000152795 | HGLibA_21815 | 37 | -0.800015802 | HNRNPDL | heterogeneous nuclear ribonucleoprotein D like [Source:HGNC Symbol;Acc:HGNC:5037] |
| ENSG00000152942 | HGLibA_40058 | 37 | -0.800015802 | RAD17 | RAD17 checkpoint clamp loader component [Source:HGNC Symbol;Acc:HGNC:9807] |
| ENSG00000153006 | HGLibA_46958 | 37 | -0.800015802 | SREK1IP1 | SREK1 interacting protein 1 [Source:HGNC Symbol;Acc:HGNC:26716] |
| ENSG00000155115 | HGLibA_20596 | 37 | -0.800015802 | GTF3C6 | general transcription factor IIIC subunit 6 [Source:HGNC Symbol;Acc:HGNC:20872] |
| ENSG00000155366 | HGLibA_41180 | 37 | -0.800015802 | RHOC | ras homolog family member C [Source:HGNC Symbol;Acc:HGNC:669] |
| ENSG00000156535 | HGLibA_08232 | 37 | -0.800015802 | CD109 | CD109 molecule [Source:HGNC Symbol;Acc:HGNC:21685] |
| ENSG00000156858 | HGLibA_38779 | 37 | -0.800015802 | PRR14 | proline rich 14 [Source:HGNC Symbol;Acc:HGNC:28458] |
| ENSG00000157322 | HGLibA_09943 | 37 | -0.800015802 | CLEC18A | C-type lectin domain family 18 member A [Source:HGNC Symbol;Acc:HGNC:30388] |
| ENSG00000157335 | HGLibA_09949 | 37 | -0.800015802 | CLEC18C | C-type lectin domain family 18 member C [Source:HGNC Symbol;Acc:HGNC:28538] |
| ENSG00000157851 | HGLibA_13935 | 37 | -0.800015802 | DPYSL5 | dihydropyrimidinase like 5 [Source:HGNC Symbol;Acc:HGNC:20637] |
| ENSG00000157911 | HGLibA_36080 | 37 | -0.800015802 | PEX10 | peroxisomal biogenesis factor 10 [Source:HGNC Symbol;Acc:HGNC:8851] |
| ENSG00000158292 | HGLibA_19938 | 37 | -0.800015802 | GPR153 | G protein-coupled receptor 153 [Source:HGNC Symbol;Acc:HGNC:23618] |
| ENSG00000158717 | HGLibA_41558 | 37 | -0.800015802 | RNF166 | ring finger protein 166 [Source:HGNC Symbol;Acc:HGNC:28856] |
| ENSG00000159197 | HGLibA_24189 | 37 | -0.800015802 | KCNE2 | potassium voltage-gated channel subfamily E regulatory subunit 2 [Source:HGNC Symbol;Acc:HGNC:6242] |
| ENSG00000160181 | HGLibA_49172 | 37 | -0.800015802 | TFF2 | trefoil factor 2 [Source:HGNC Symbol;Acc:HGNC:11756] |
| ENSG00000160654 | HGLibA_08378 | 37 | -0.800015802 | CD3G | CD3g molecule [Source:HGNC Symbol;Acc:HGNC:1675] |
| ENSG00000162600 | HGLibA_33380 | 37 | -0.800015802 | OMA1 | OMA1 zinc metallopeptidase [Source:HGNC Symbol;Acc:HGNC:29661] |
| ENSG00000163714 | HGLibA_52502 | 37 | -0.800015802 | U2SURP | U2 snRNP associated SURP domain containing [Source:HGNC Symbol;Acc:HGNC:30855] |
| ENSG00000163939 | HGLibA_35340 | 37 | -0.800015802 | PBRM1 | polybromo 1 [Source:HGNC Symbol;Acc:HGNC:30064] |
| ENSG00000164038 | HGLibA_45435 | 37 | -0.800015802 | SLC9B2 | solute carrier family 9 member B2 [Source:HGNC Symbol;Acc:HGNC:25143] |
| ENSG00000164088 | HGLibA_37993 | 37 | -0.800015802 | PPM1M | Mg2+/Mn2+ dependent 1M [Source:HGNC Symbol;Acc:HGNC:26506]" |
| ENSG00000164099 | HGLibA_38901 | 37 | -0.800015802 | PRSS12 | serine protease 12 [Source:HGNC Symbol;Acc:HGNC:9477] |
| ENSG00000164304 | HGLibA_06984 | 37 | -0.800015802 | CAGE1 | cancer antigen 1 [Source:HGNC Symbol;Acc:HGNC:21622] |
| ENSG00000164411 | HGLibA_19176 | 37 | -0.800015802 | GJB7 | gap junction protein beta 7 [Source:HGNC Symbol;Acc:HGNC:16690] |
| ENSG00000164970 | HGLibA_16498 | 37 | -0.800015802 | FAM219A | family with sequence similarity 219 member A [Source:HGNC Symbol;Acc:HGNC:19920] |
| ENSG00000165434 | HGLibA_36267 | 37 | -0.800015802 | PGM2L1 | phosphoglucomutase 2 like 1 [Source:HGNC Symbol;Acc:HGNC:20898] |
| ENSG00000165630 | HGLibA_38709 | 37 | -0.800015802 | PRPF18 | pre-mRNA processing factor 18 [Source:HGNC Symbol;Acc:HGNC:17351] |
| ENSG00000165775 | HGLibA_18167 | 37 | -0.800015802 | FUNDC2 | FUN14 domain containing 2 [Source:HGNC Symbol;Acc:HGNC:24925] |
| ENSG00000166415 | HGLibA_54167 | 37 | -0.800015802 | WDR72 | WD repeat domain 72 [Source:HGNC Symbol;Acc:HGNC:26790] |
| ENSG00000166548 | HGLibA_49584 | 37 | -0.800015802 | TK2 | mitochondrial [Source:HGNC Symbol;Acc:HGNC:11831]" |
| ENSG00000166589 | HGLibA_08648 | 37 | -0.800015802 | CDH16 | cadherin 16 [Source:HGNC Symbol;Acc:HGNC:1755] |
| ENSG00000166669 | HGLibA_03459 | 37 | -0.800015802 | ATF7IP2 | activating transcription factor 7 interacting protein 2 [Source:HGNC Symbol;Acc:HGNC:20397] |
| ENSG00000166848 | HGLibA_49017 | 37 | -0.800015802 | TERF2IP | TERF2 interacting protein [Source:HGNC Symbol;Acc:HGNC:19246] |
| ENSG00000167925 | HGLibA_19034 | 37 | -0.800015802 | GHDC | GH3 domain containing [Source:HGNC Symbol;Acc:HGNC:24438] |
| ENSG00000168014 | HGLibA_06091 | 37 | -0.800015802 | C2CD3 | C2 calcium dependent domain containing 3 [Source:HGNC Symbol;Acc:HGNC:24564] |
| ENSG00000168495 | HGLibA_37619 | 37 | -0.800015802 | POLR3D | RNA polymerase III subunit D [Source:HGNC Symbol;Acc:HGNC:1080] |
| ENSG00000168589 | HGLibA_14261 | 37 | -0.800015802 | DYNLRB2 | dynein light chain roadblock-type 2 [Source:HGNC Symbol;Acc:HGNC:15467] |
| ENSG00000168778 | HGLibA_48874 | 37 | -0.800015802 | TCTN2 | tectonic family member 2 [Source:HGNC Symbol;Acc:HGNC:25774] |
| ENSG00000169903 | HGLibA_49704 | 37 | -0.800015802 | TM4SF4 | transmembrane 4 L six family member 4 [Source:HGNC Symbol;Acc:HGNC:11856] |
| ENSG00000170037 | HGLibA_10439 | 37 | -0.800015802 | CNTROB | centriole duplication and spindle assembly protein [Source:HGNC Symbol;Acc:HGNC:29616]" |
| ENSG00000170790 | HGLibA_33460 | 37 | -0.800015802 | OR10A2 | olfactory receptor family 10 subfamily A member 2 [Source:HGNC Symbol;Acc:HGNC:8161] |
| ENSG00000170909 | HGLibA_34672 | 37 | -0.800015802 | OSCAR | immunoglobulin-like receptor [Source:HGNC Symbol;Acc:HGNC:29960]" |
| ENSG00000171161 | HGLibA_56505 | 37 | -0.800015802 | ZNF672 | zinc finger protein 672 [Source:HGNC Symbol;Acc:HGNC:26179] |
| ENSG00000171496 | HGLibA_33698 | 37 | -0.800015802 | OR1L8 | olfactory receptor family 1 subfamily L member 8 [Source:HGNC Symbol;Acc:HGNC:15110] |
| ENSG00000171817 | HGLibA_56188 | 37 | -0.800015802 | ZNF540 | zinc finger protein 540 [Source:HGNC Symbol;Acc:HGNC:25331] |
| ENSG00000172062 | HGLibA_45782 | 37 | -0.800015802 | SMN1 | telomeric [Source:HGNC Symbol;Acc:HGNC:11117]" |
| ENSG00000172123 | HGLibA_45485 | 37 | -0.800015802 | SLFN12 | schlafen family member 12 [Source:HGNC Symbol;Acc:HGNC:25500] |
| ENSG00000172301 | HGLibA_10710 | 37 | -0.800015802 | COPRS | coordinator of PRMT5 and differentiation stimulator [Source:HGNC Symbol;Acc:HGNC:28848] |
| ENSG00000172367 | HGLibA_35977 | 37 | -0.800015802 | PDZD3 | PDZ domain containing 3 [Source:HGNC Symbol;Acc:HGNC:19891] |
| ENSG00000172940 | HGLibA_44547 | 37 | -0.800015802 | SLC22A13 | solute carrier family 22 member 13 [Source:HGNC Symbol;Acc:HGNC:8494] |
| ENSG00000173261 | HGLibA_36946 | 37 | -0.800015802 | PLAC8L1 | PLAC8 like 1 [Source:HGNC Symbol;Acc:HGNC:31746] |
| ENSG00000173559 | HGLibA_31024 | 37 | -0.800015802 | NABP1 | nucleic acid binding protein 1 [Source:HGNC Symbol;Acc:HGNC:26232] |
| ENSG00000174574 | HGLibA_01456 | 37 | -0.800015802 | AKIRIN1 | akirin 1 [Source:HGNC Symbol;Acc:HGNC:25744] |
| ENSG00000174871 | HGLibA_10287 | 37 | -0.800015802 | CNIH2 | cornichon family AMPA receptor auxiliary protein 2 [Source:HGNC Symbol;Acc:HGNC:28744] |
| ENSG00000175857 | HGLibA_18619 | 37 | -0.800015802 | GAPT | transmembrane [Source:HGNC Symbol;Acc:HGNC:26588]" |
| ENSG00000177674 | HGLibA_01268 | 37 | -0.800015802 | AGTRAP | angiotensin II receptor associated protein [Source:HGNC Symbol;Acc:HGNC:13539] |

| | | | | | |
|---|---|---|---|---|---|
| ENSG00000178718 | HGLibA_42030 | 37 | -0.800015802 | RPP25 | ribonuclease P and MRP subunit p25 [Source:HGNC Symbol;Acc:HGNC:30361] |
| ENSG00000179598 | HGLibA_37049 | 37 | -0.800015802 | PLD6 | phospholipase D family member 6 [Source:HGNC Symbol;Acc:HGNC:30447] |
| ENSG00000180008 | HGLibA_46131 | 37 | -0.800015802 | SOCS4 | suppressor of cytokine signaling 4 [Source:HGNC Symbol;Acc:HGNC:19392] |
| ENSG00000180776 | HGLibA_55100 | 37 | -0.800015802 | ZDHHC20 | zinc finger DHHC-type containing 20 [Source:HGNC Symbol;Acc:HGNC:20749] |
| ENSG00000181374 | HGLibA_07966 | 37 | -0.800015802 | CCL13 | C-C motif chemokine ligand 13 [Source:HGNC Symbol;Acc:HGNC:10611] |
| ENSG00000181625 | HGLibA_45559 | 37 | -0.800015802 | SLX1B | structure-specific endonuclease subunit [Source:HGNC Symbol;Acc:HGNC:28748]" |
| ENSG00000181754 | HGLibA_01793 | 37 | -0.800015802 | AMIGO1 | adhesion molecule with Ig like domain 1 [Source:HGNC Symbol;Acc:HGNC:20824] |
| ENSG00000182307 | HGLibA_06609 | 37 | -0.800015802 | C8orf33 | chromosome 8 open reading frame 33 [Source:HGNC Symbol;Acc:HGNC:26104] |
| ENSG00000184394 | HGLibA_34052 | 37 | -0.800015802 | OR4N5 | olfactory receptor family 4 subfamily N member 5 [Source:HGNC Symbol;Acc:HGNC:15358] |
| ENSG00000185189 | HGLibA_32659 | 37 | -0.800015802 | NRBP2 | nuclear receptor binding protein 2 [Source:HGNC Symbol;Acc:HGNC:19339] |
| ENSG00000186184 | HGLibA_37557 | 37 | -0.800015802 | POLR1D | RNA polymerase I and III subunit D [Source:HGNC Symbol;Acc:HGNC:20422] |
| ENSG00000187498 | HGLibA_10565 | 37 | -0.800015802 | COL4A1 | collagen type IV alpha 1 chain [Source:HGNC Symbol;Acc:HGNC:2202] |
| ENSG00000188056 | HGLibA_51366 | 37 | -0.800015802 | TREML4 | triggering receptor expressed on myeloid cells like 4 [Source:HGNC Symbol;Acc:HGNC:30807] |
| ENSG00000188120 | HGLibA_12353 | 37 | -0.800015802 | DAZ1 | deleted in azoospermia 1 [Source:HGNC Symbol;Acc:HGNC:2682] |
| ENSG00000188277 | HGLibA_05433 | 37 | -0.800015802 | C15orf62 | chromosome 15 open reading frame 62 [Source:HGNC Symbol;Acc:HGNC:34489] |
| ENSG00000189037 | HGLibA_14160 | 37 | -0.800015802 | DUSP21 | dual specificity phosphatase 21 [Source:HGNC Symbol;Acc:HGNC:20476] |
| ENSG00000196260 | HGLibA_43761 | 37 | -0.800015802 | SFTA2 | surfactant associated 2 [Source:HGNC Symbol;Acc:HGNC:18386] |
| ENSG00000196557 | HGLibA_06896 | 37 | -0.800015802 | CACNA1H | calcium voltage-gated channel subunit alpha1 H [Source:HGNC Symbol;Acc:HGNC:1395] |
| ENSG00000196597 | HGLibA_56741 | 37 | -0.800015802 | ZNF782 | zinc finger protein 782 [Source:HGNC Symbol;Acc:HGNC:33110] |
| ENSG00000197483 | HGLibA_56428 | 37 | -0.800015802 | ZNF628 | zinc finger protein 628 [Source:HGNC Symbol;Acc:HGNC:28054] |
| ENSG00000197568 | HGLibA_21273 | 37 | -0.800015802 | HHLA3 | HERV-H LTR-associating 3 [Source:HGNC Symbol;Acc:HGNC:4906] |
| ENSG00000198863 | HGLibA_42445 | 37 | -0.800015802 | RUNDC1 | RUN domain containing 1 [Source:HGNC Symbol;Acc:HGNC:25418] |
| ENSG00000204444 | HGLibA_02549 | 37 | -0.800015802 | APOM | apolipoprotein M [Source:HGNC Symbol;Acc:HGNC:13916] |
| ENSG00000204475 | HGLibA_31367 | 37 | -0.800015802 | NCR3 | natural cytotoxicity triggering receptor 3 [Source:HGNC Symbol;Acc:HGNC:19077] |
| ENSG00000205078 | HGLibA_47917 | 37 | -0.800015802 | SYCE1L | synaptonemal complex central element protein 1 like [Source:HGNC Symbol;Acc:HGNC:37236] |
| ENSG00000205502 | HGLibA_06097 | 37 | -0.800015802 | C2CD4B | C2 calcium dependent domain containing 4B [Source:HGNC Symbol;Acc:HGNC:33628] |
| ENSG00000206013 | HGLibA_22650 | 37 | -0.800015802 | IFITM5 | interferon induced transmembrane protein 5 [Source:HGNC Symbol;Acc:HGNC:16644] |
| ENSG00000212938 | HGLibA_25821 | 37 | -0.800015802 | KRTAP6-3 | keratin associated protein 6-3 [Source:HGNC Symbol;Acc:HGNC:18933] |
| ENSG00000213337 | HGLibA_02093 | 37 | -0.800015802 | ANKRD39 | ankyrin repeat domain 39 [Source:HGNC Symbol;Acc:HGNC:28640] |
| ENSG00000215252 | HGLibA_19644 | 37 | -0.800015802 | GOLGA8B | golgin A8 family member B [Source:HGNC Symbol;Acc:HGNC:31973] |
| ENSG00000215356 | HGLibA_56589 | 37 | -0.800015802 | ZNF705B | zinc finger protein 705B [Source:HGNC Symbol;Acc:HGNC:32284] |
| ENSG00000221823 | HGLibA_38195 | 37 | -0.800015802 | PPP3R1 | alpha [Source:HGNC Symbol;Acc:HGNC:9317]" |
| ENSG00000223569 | HGLibA_53228 | 37 | -0.800015802 | USP17L15 | ubiquitin specific peptidase 17-like family member 15 [Source:HGNC Symbol;Acc:HGNC:44443] |
| ENSG00000228474 | HGLibA_34705 | 37 | -0.800015802 | OST4 | non-catalytic [Source:HGNC Symbol;Acc:HGNC:32483]" |
| ENSG00000236362 | HGLibA_18449 | 37 | -0.800015802 | GAGE12F | G antigen 12F [Source:HGNC Symbol;Acc:HGNC:31906] |
| ENSG00000237247 | HGLibA_28484 | 37 | -0.800015802 | MBD3L5 | methyl-CpG binding domain protein 3 like 5 [Source:HGNC Symbol;Acc:HGNC:37204] |
| ENSG00000241233 | HGLibA_25809 | 37 | -0.800015802 | KRTAP5-8 | keratin associated protein 5-8 [Source:HGNC Symbol;Acc:HGNC:23603] |
| ENSG00000242372 | HGLibA_14867 | 37 | -0.800015802 | EIF6 | eukaryotic translation initiation factor 6 [Source:HGNC Symbol;Acc:HGNC:6159] |
| ENSG00000253710 | HGLibA_01611 | 37 | -0.800015802 | ALG11 | 2-mannosyltransferase [Source:HGNC Symbol;Acc:HGNC:32456]" |
| ENSG00000255181 | HGLibA_07673 | 37 | -0.800015802 | CCDC166 | coiled-coil domain containing 166 [Source:HGNC Symbol;Acc:HGNC:41910] |
| ENSG00000255223 | HGLibA_34333 | 37 | -0.800015802 | OR5M11 | olfactory receptor family 5 subfamily M member 11 [Source:HGNC Symbol;Acc:HGNC:15291] |
| ENSG00000258643 | HGLibA_04327 | 37 | -0.800015802 | BCL2L2-PABPN1 | BCL2L2-PABPN1 readthrough [Source:HGNC Symbol;Acc:HGNC:42959] |
| ENSG00000270800 | HGLibA_42062 | 37 | -0.800015802 | RPS10-NUDT3 | RPS10-NUDT3 readthrough [Source:HGNC Symbol;Acc:HGNC:49181] |
| ENSG00000278289 | HGLibA_11512 | 37 | -0.800015802 | CT45A6 | cancer/testis antigen family 45 member A6 [Source:HGNC Symbol;Acc:HGNC:33271] |
| ENSG00000279395 | HGLibA_34321 | 37 | -0.800015802 | OR5L1 | olfactory receptor family 5 subfamily L member 1 (gene/pseudogene) [Source:HGNC Symbol;Acc:HGNC:8350] |
| FAIM3 | HGLibA_15971 | 37 | -0.800015802 | FAIM3 | - |
| hsa-mir-1262 | HGLibA_57471 | 37 | -0.800015802 | hsa-mir-1262 | - |
| hsa-mir-1273g | HGLibA_57548 | 37 | -0.800015802 | hsa-mir-1273g | - |
| hsa-mir-206 | HGLibA_58191 | 37 | -0.800015802 | hsa-mir-206 | - |
| hsa-mir-2113 | HGLibA_58225 | 37 | -0.800015802 | hsa-mir-2113 | - |
| hsa-mir-2116 | HGLibA_58235 | 37 | -0.800015802 | hsa-mir-2116 | - |
| hsa-mir-300 | HGLibA_58424 | 37 | -0.800015802 | hsa-mir-300 | - |
| hsa-mir-3199-2 | HGLibA_58903 | 37 | -0.800015802 | hsa-mir-3199-2 | - |
| hsa-mir-3670-1 | HGLibA_59233 | 37 | -0.800015802 | hsa-mir-3670-1 | - |
| hsa-mir-424 | HGLibA_59702 | 37 | -0.800015802 | hsa-mir-424 | - |
| hsa-mir-4436a | HGLibA_60137 | 37 | -0.800015802 | hsa-mir-4436a | - |
| hsa-mir-4437 | HGLibA_60150 | 37 | -0.800015802 | hsa-mir-4437 | - |
| hsa-mir-4446 | HGLibA_60190 | 37 | -0.800015802 | hsa-mir-4446 | - |
| hsa-mir-4510 | HGLibA_60484 | 37 | -0.800015802 | hsa-mir-4510 | - |
| hsa-mir-4519 | HGLibA_60518 | 37 | -0.800015802 | hsa-mir-4519 | - |
| hsa-mir-4705 | HGLibA_60922 | 37 | -0.800015802 | hsa-mir-4705 | - |
| hsa-mir-568 | HGLibA_62233 | 37 | -0.800015802 | hsa-mir-568 | - |
| hsa-mir-642a | HGLibA_62806 | 37 | -0.800015802 | hsa-mir-642a | - |
| hsa-mir-6721 | HGLibA_63045 | 37 | -0.800015802 | hsa-mir-6721 | - |
| hsa-mir-6801 | HGLibA_63383 | 37 | -0.800015802 | hsa-mir-6801 | - |
| hsa-mir-6864 | HGLibA_63641 | 37 | -0.800015802 | hsa-mir-6864 | - |
| hsa-mir-6891 | HGLibA_63748 | 37 | -0.800015802 | hsa-mir-6891 | - |
| hsa-mir-744 | HGLibA_63883 | 37 | -0.800015802 | hsa-mir-744 | - |
| hsa-mir-767 | HGLibA_63932 | 37 | -0.800015802 | hsa-mir-767 | - |
| IGJ | HGLibA_22862 | 37 | -0.800015802 | IGJ | - |
| KIAA0226 | HGLibA_24605 | 37 | -0.800015802 | KIAA0226 | - |
| KIAA0754 | HGLibA_24651 | 37 | -0.800015802 | KIAA0754 | - |
| MEF2BNB-MEF2B | HGLibA_28836 | 37 | -0.800015802 | MEF2BNB-MEF2B | - |
| MRE11A | HGLibA_29823 | 37 | -0.800015802 | MRE11A | - |
| NUPR1L | HGLibA_33089 | 37 | -0.800015802 | NUPR1L | - |
| PRAMEF21 | HGLibA_38310 | 37 | -0.800015802 | PRAMEF21 | - |
| PRUNE | HGLibA_38986 | 37 | -0.800015802 | PRUNE | - |
| SCXA | HGLibA_43080 | 37 | -0.800015802 | SCXA | - |
| WDR16 | HGLibA_54035 | 37 | -0.800015802 | WDR16 | - |
| WDR92 | HGLibA_54220 | 37 | -0.800015802 | WDR92 | - |
| ZNF812 | HGLibA_56803 | 37 | -0.800015802 | ZNF812 | - |
| 9-Mar | HGLibA_28351 | 36 | -0.809061145 | 9-Mar | - |
| C16orf80 | HGLibA_05486 | 36 | -0.809061145 | C16orf80 | - |
| C7orf10 | HGLibA_06521 | 36 | -0.809061145 | C7orf10 | - |
| C8orf42 | HGLibA_06619 | 36 | -0.809061145 | C8orf42 | - |
| CARS | HGLibA_07260 | 36 | -0.809061145 | CARS | - |
| ENSG00000006047 | HGLibA_54646 | 36 | -0.809061145 | YBX2 | Y-box binding protein 2 [Source:HGNC Symbol;Acc:HGNC:17948] |
| ENSG00000006047 | HGLibA_54647 | 36 | -0.809061145 | YBX2 | Y-box binding protein 2 [Source:HGNC Symbol;Acc:HGNC:17948] |
| ENSG00000009844 | HGLibA_53882 | 36 | -0.809061145 | VTA1 | vesicle trafficking 1 [Source:HGNC Symbol;Acc:HGNC:20954] |
| ENSG00000011677 | HGLibA_18363 | 36 | -0.809061145 | GABRA3 | gamma-aminobutyric acid type A receptor alpha3 subunit [Source:HGNC Symbol;Acc:HGNC:4077] |
| ENSG00000029534 | HGLibA_01934 | 36 | -0.809061145 | ANK1 | ankyrin 1 [Source:HGNC Symbol;Acc:HGNC:492] |
| ENSG00000037749 | HGLibA_29044 | 36 | -0.809061145 | MFAP3 | microfibril associated protein 3 [Source:HGNC Symbol;Acc:HGNC:7034] |
| ENSG00000041357 | HGLibA_39080 | 36 | -0.809061145 | PSMA4 | proteasome subunit alpha 4 [Source:HGNC Symbol;Acc:HGNC:9533] |
| ENSG00000057294 | HGLibA_36854 | 36 | -0.809061145 | PKP2 | plakophilin 2 [Source:HGNC Symbol;Acc:HGNC:9024] |
| ENSG00000058063 | HGLibA_03558 | 36 | -0.809061145 | ATP11B | ATPase phospholipid transporting 11B (putative) [Source:HGNC Symbol;Acc:HGNC:13553] |
| ENSG00000066405 | HGLibA_09871 | 36 | -0.809061145 | CLDN18 | claudin 18 [Source:HGNC Symbol;Acc:HGNC:2039] |
| ENSG00000068831 | HGLibA_40346 | 36 | -0.809061145 | RASGRP2 | RAS guanyl releasing protein 2 [Source:HGNC Symbol;Acc:HGNC:9879] |
| ENSG00000073861 | HGLibA_48661 | 36 | -0.809061145 | TBX21 | T-box 21 [Source:HGNC Symbol;Acc:HGNC:11599] |
| ENSG00000074317 | HGLibA_45915 | 36 | -0.809061145 | SNCB | synuclein beta [Source:HGNC Symbol;Acc:HGNC:11140] |
| ENSG00000077420 | HGLibA_02380 | 36 | -0.809061145 | APBB1IP | amyloid beta precursor protein binding family B member 1 interacting protein [Source:HGNC Symbol;Acc:HGNC:17379] |
| ENSG00000077616 | HGLibA_31009 | 36 | -0.809061145 | NAALAD2 | N-acetylated alpha-linked acidic dipeptidase 2 [Source:HGNC Symbol;Acc:HGNC:14526] |
| ENSG00000078043 | HGLibA_36509 | 36 | -0.809061145 | PIAS2 | protein inhibitor of activated STAT 2 [Source:HGNC Symbol;Acc:HGNC:17311] |
| ENSG00000081154 | HGLibA_35636 | 36 | -0.809061145 | PCNP | PEST proteolytic signal containing nuclear protein [Source:HGNC Symbol;Acc:HGNC:30023] |
| ENSG00000084733 | HGLibA_39783 | 36 | -0.809061145 | RAB10 | member RAS oncogene family [Source:HGNC Symbol;Acc:HGNC:9759]" |
| ENSG00000087086 | HGLibA_18125 | 36 | -0.809061145 | FTL | ferritin light chain [Source:HGNC Symbol;Acc:HGNC:3999] |
| ENSG00000087157 | HGLibA_36294 | 36 | -0.809061145 | PGS1 | phosphatidylglycerophosphate synthase 1 [Source:HGNC Symbol;Acc:HGNC:30029] |
| ENSG00000087510 | HGLibA_49126 | 36 | -0.809061145 | TFAP2C | transcription factor AP-2 gamma [Source:HGNC Symbol;Acc:HGNC:11744] |
| ENSG00000089041 | HGLibA_34863 | 36 | -0.809061145 | P2RX7 | purinergic receptor P2X 7 [Source:HGNC Symbol;Acc:HGNC:8537] |
| ENSG00000089351 | HGLibA_20193 | 36 | -0.809061145 | GRAMD1A | GRAM domain containing 1A [Source:HGNC Symbol;Acc:HGNC:29305] |
| ENSG00000090487 | HGLibA_46636 | 36 | -0.809061145 | SPG21 | maspardin [Source:HGNC Symbol;Acc:HGNC:20373]" |
| ENSG00000099817 | HGLibA_37575 | 36 | -0.809061145 | POLR2E | RNA polymerase II subunit E [Source:HGNC Symbol;Acc:HGNC:9192] |
| ENSG00000100418 | HGLibA_13006 | 36 | -0.809061145 | DESI1 | desumoylating isopeptidase 1 [Source:HGNC Symbol;Acc:HGNC:24577] |
| ENSG00000101489 | HGLibA_09013 | 36 | -0.809061145 | CELF4 | CUGBP Elav-like family member 4 [Source:HGNC Symbol;Acc:HGNC:14015] |
| ENSG00000103335 | HGLibA_36533 | 36 | -0.809061145 | PIEZO1 | piezo type mechanosensitive ion channel component 1 [Source:HGNC Symbol;Acc:HGNC:28993] |
| ENSG00000104490 | HGLibA_31250 | 36 | -0.809061145 | NCALD | neurocalcin delta [Source:HGNC Symbol;Acc:HGNC:7655] |
| ENSG00000105135 | HGLibA_23226 | 36 | -0.809061145 | ILVBL | ilvB acetolactate synthase like [Source:HGNC Symbol;Acc:HGNC:6041] |
| ENSG00000105198 | HGLibA_26352 | 36 | -0.809061145 | LGALS13 | galectin 13 [Source:HGNC Symbol;Acc:HGNC:15449] |
| ENSG00000105402 | HGLibA_31125 | 36 | -0.809061145 | NAPA | NSF attachment protein alpha [Source:HGNC Symbol;Acc:HGNC:7641] |
| ENSG00000105464 | HGLibA_20308 | 36 | -0.809061145 | GRIN2D | glutamate ionotropic receptor NMDA type subunit 2D [Source:HGNC Symbol;Acc:HGNC:4588] |
| ENSG00000105819 | HGLibA_37330 | 36 | -0.809061145 | PMPCB | mitochondrial processing beta subunit [Source:HGNC Symbol;Acc:HGNC:9119]" |
| ENSG00000106692 | HGLibA_17599 | 36 | -0.809061145 | FKTN | fukutin [Source:HGNC Symbol;Acc:HGNC:3622] |
| ENSG00000107187 | HGLibA_26453 | 36 | -0.809061145 | LHX3 | LIM homeobox 3 [Source:HGNC Symbol;Acc:HGNC:6595] |
| ENSG00000107438 | HGLibA_35906 | 36 | -0.809061145 | PDLIM1 | PDZ and LIM domain 1 [Source:HGNC Symbol;Acc:HGNC:2067] |
| ENSG00000108688 | HGLibA_08040 | 36 | -0.809061145 | CCL7 | C-C motif chemokine ligand 7 [Source:HGNC Symbol;Acc:HGNC:10634] |
| ENSG00000110777 | HGLibA_37753 | 36 | -0.809061145 | POU2AF1 | POU class 2 associating factor 1 [Source:HGNC Symbol;Acc:HGNC:9211] |
| ENSG00000111196 | HGLibA_28010 | 36 | -0.809061145 | MAGOHB | exon junction complex subunit [Source:HGNC Symbol;Acc:HGNC:25504]" |
| ENSG00000111199 | HGLibA_51790 | 36 | -0.809061145 | TRPV4 | transient receptor potential cation channel subfamily V member 4 [Source:HGNC Symbol;Acc:HGNC:18083] |
| ENSG00000111450 | HGLibA_47643 | 36 | -0.809061145 | STX2 | syntaxin 2 [Source:HGNC Symbol;Acc:HGNC:3403] |
| ENSG00000112796 | HGLibA_15180 | 36 | -0.809061145 | ENPP5 | ectonucleotide pyrophosphatase/phosphodiesterase 5 (putative) [Source:HGNC Symbol;Acc:HGNC:13717] |
| ENSG00000113327 | HGLibA_18392 | 36 | -0.809061145 | GABRG2 | gamma-aminobutyric acid type A receptor gamma2 subunit [Source:HGNC Symbol;Acc:HGNC:4087] |
| ENSG00000113658 | HGLibA_45580 | 36 | -0.809061145 | SMAD5 | SMAD family member 5 [Source:HGNC Symbol;Acc:HGNC:6771] |
| ENSG00000113749 | HGLibA_22096 | 36 | -0.809061145 | HRH2 | histamine receptor H2 [Source:HGNC Symbol;Acc:HGNC:5183] |
| ENSG00000113851 | HGLibA_11077 | 36 | -0.809061145 | CRBN | cereblon [Source:HGNC Symbol;Acc:HGNC:30185] |
| ENSG00000114383 | HGLibA_52345 | 36 | -0.809061145 | TUSC2 | mitochondrial calcium regulator [Source:HGNC Symbol;Acc:HGNC:17034]" |
| ENSG00000116017 | HGLibA_02891 | 36 | -0.809061145 | ARID3A | AT-rich interaction domain 3A [Source:HGNC Symbol;Acc:HGNC:3031] |

| | | | | | |
|---|---|---|---|---|---|
| ENSG00000116194 | HGLibA_01909 | 36 | -0.809061145 | ANGPTL1 | angiopoietin like 1 [Source:HGNC Symbol;Acc:HGNC:489] |
| ENSG00000116586 | HGLibA_25991 | 36 | -0.809061145 | LAMTOR2 | MAPK and MTOR activator 2 [Source:HGNC Symbol;Acc:HGNC:29796]" |
| ENSG00000117758 | HGLibA_47620 | 36 | -0.809061145 | STX12 | syntaxin 12 [Source:HGNC Symbol;Acc:HGNC:11430] |
| ENSG00000119711 | HGLibA_01580 | 36 | -0.809061145 | ALDH6A1 | aldehyde dehydrogenase 6 family member A1 [Source:HGNC Symbol;Acc:HGNC:7179] |
| ENSG00000120008 | HGLibA_54024 | 36 | -0.809061145 | WDR11 | WD repeat domain 11 [Source:HGNC Symbol;Acc:HGNC:13831] |
| ENSG00000120217 | HGLibA_08317 | 36 | -0.809061145 | CD274 | CD274 molecule [Source:HGNC Symbol;Acc:HGNC:17635] |
| ENSG00000123066 | HGLibA_28750 | 36 | -0.809061145 | MED13L | mediator complex subunit 13 like [Source:HGNC Symbol;Acc:HGNC:22962] |
| ENSG00000124299 | HGLibA_36050 | 36 | -0.809061145 | PEPD | peptidase D [Source:HGNC Symbol;Acc:HGNC:8840] |
| ENSG00000125650 | HGLibA_39228 | 36 | -0.809061145 | PSPN | persephin [Source:HGNC Symbol;Acc:HGNC:9579] |
| ENSG00000125872 | HGLibA_27478 | 36 | -0.809061145 | LRRN4 | leucine rich repeat neuronal 4 [Source:HGNC Symbol;Acc:HGNC:16208] |
| ENSG00000126767 | HGLibA_14912 | 36 | -0.809061145 | ELK1 | ETS transcription factor [Source:HGNC Symbol;Acc:HGNC:3321]" |
| ENSG00000127184 | HGLibA_10873 | 36 | -0.809061145 | COX7C | cytochrome c oxidase subunit 7C [Source:HGNC Symbol;Acc:HGNC:2292] |
| ENSG00000127530 | HGLibA_34466 | 36 | -0.809061145 | OR7C1 | olfactory receptor family 7 subfamily C member 1 [Source:HGNC Symbol;Acc:HGNC:8373] |
| ENSG00000128218 | HGLibA_53726 | 36 | -0.809061145 | VPREB3 | V-set pre-B cell surrogate light chain 3 [Source:HGNC Symbol;Acc:HGNC:12710] |
| ENSG00000128283 | HGLibA_08566 | 36 | -0.809061145 | CDC42EP1 | CDC42 effector protein 1 [Source:HGNC Symbol;Acc:HGNC:17014] |
| ENSG00000128513 | HGLibA_37714 | 36 | -0.809061145 | POT1 | protection of telomeres 1 [Source:HGNC Symbol;Acc:HGNC:17284] |
| ENSG00000128833 | HGLibA_30856 | 36 | -0.809061145 | MYO5C | myosin VC [Source:HGNC Symbol;Acc:HGNC:7604] |
| ENSG00000129422 | HGLibA_30506 | 36 | -0.809061145 | MTUS1 | microtubule associated scaffold protein 1 [Source:HGNC Symbol;Acc:HGNC:29789] |
| ENSG00000129515 | HGLibA_46102 | 36 | -0.809061145 | SNX6 | sorting nexin 6 [Source:HGNC Symbol;Acc:HGNC:14970] |
| ENSG00000130193 | HGLibA_49345 | 36 | -0.809061145 | THEM6 | thioesterase superfamily member 6 [Source:HGNC Symbol;Acc:HGNC:29656] |
| ENSG00000130985 | HGLibA_52514 | 36 | -0.809061145 | UBA1 | ubiquitin like modifier activating enzyme 1 [Source:HGNC Symbol;Acc:HGNC:12469] |
| ENSG00000131037 | HGLibA_15376 | 36 | -0.809061145 | EPS8L1 | EPS8 like 1 [Source:HGNC Symbol;Acc:HGNC:21295] |
| ENSG00000131471 | HGLibA_02272 | 36 | -0.809061145 | AOC3 | copper containing 3 [Source:HGNC Symbol;Acc:HGNC:550]" |
| ENSG00000132005 | HGLibA_40961 | 36 | -0.809061145 | RFX1 | regulatory factor X1 [Source:HGNC Symbol;Acc:HGNC:9982] |
| ENSG00000132703 | HGLibA_02401 | 36 | -0.809061145 | APCS | serum [Source:HGNC Symbol;Acc:HGNC:584]" |
| ENSG00000133026 | HGLibA_30707 | 36 | -0.809061145 | MYH10 | myosin heavy chain 10 [Source:HGNC Symbol;Acc:HGNC:7568] |
| ENSG00000133059 | HGLibA_14049 | 36 | -0.809061145 | DSTYK | dual serine/threonine and tyrosine protein kinase [Source:HGNC Symbol;Acc:HGNC:29043] |
| ENSG00000133256 | HGLibA_35809 | 36 | -0.809061145 | PDE6B | phosphodiesterase 6B [Source:HGNC Symbol;Acc:HGNC:8786] |
| ENSG00000134183 | HGLibA_19475 | 36 | -0.809061145 | GNAT2 | G protein subunit alpha transducin 2 [Source:HGNC Symbol;Acc:HGNC:4394] |
| ENSG00000134313 | HGLibA_24848 | 36 | -0.809061145 | KIDINS220 | kinase D interacting substrate 220 [Source:HGNC Symbol;Acc:HGNC:29508] |
| ENSG00000134574 | HGLibA_12597 | 36 | -0.809061145 | DDB2 | damage specific DNA binding protein 2 [Source:HGNC Symbol;Acc:HGNC:2718] |
| ENSG00000134717 | HGLibA_04939 | 36 | -0.809061145 | BTF3L4 | basic transcription factor 3 like 4 [Source:HGNC Symbol;Acc:HGNC:30547] |
| ENSG00000134851 | HGLibA_50035 | 36 | -0.809061145 | TMEM165 | transmembrane protein 165 [Source:HGNC Symbol;Acc:HGNC:30760] |
| ENSG00000135547 | HGLibA_21220 | 36 | -0.809061145 | HEY2 | hes related family bHLH transcription factor with YRPW motif 2 [Source:HGNC Symbol;Acc:HGNC:4881] |
| ENSG00000136156 | HGLibA_23832 | 36 | -0.809061145 | ITM2B | integral membrane protein 2B [Source:HGNC Symbol;Acc:HGNC:6174] |
| ENSG00000136436 | HGLibA_07002 | 36 | -0.809061145 | CALCOCO2 | calcium binding and coiled-coil domain 2 [Source:HGNC Symbol;Acc:HGNC:29912] |
| ENSG00000136935 | HGLibA_19591 | 36 | -0.809061145 | GOLGA1 | golgin A1 [Source:HGNC Symbol;Acc:HGNC:4424] |
| ENSG00000137413 | HGLibA_48258 | 36 | -0.809061145 | TAF8 | TATA-box binding protein associated factor 8 [Source:HGNC Symbol;Acc:HGNC:17300] |
| ENSG00000137413 | HGLibA_48259 | 36 | -0.809061145 | TAF8 | TATA-box binding protein associated factor 8 [Source:HGNC Symbol;Acc:HGNC:17300] |
| ENSG00000138386 | HGLibA_31018 | 36 | -0.809061145 | NAB1 | NGFI-A binding protein 1 [Source:HGNC Symbol;Acc:HGNC:7626] |
| ENSG00000139182 | HGLibA_10140 | 36 | -0.809061145 | CLSTN3 | calsyntenin 3 [Source:HGNC Symbol;Acc:HGNC:18371] |
| ENSG00000139182 | HGLibA_10139 | 36 | -0.809061145 | CLSTN3 | calsyntenin 3 [Source:HGNC Symbol;Acc:HGNC:18371] |
| ENSG00000139549 | HGLibA_13108 | 36 | -0.809061145 | DHH | desert hedgehog [Source:HGNC Symbol;Acc:HGNC:2865] |
| ENSG00000139977 | HGLibA_30987 | 36 | -0.809061145 | NAA30 | NatC catalytic subunit [Source:HGNC Symbol;Acc:HGNC:19844]" |
| ENSG00000140153 | HGLibA_54047 | 36 | -0.809061145 | WDR20 | WD repeat domain 20 [Source:HGNC Symbol;Acc:HGNC:19667] |
| ENSG00000140259 | HGLibA_29039 | 36 | -0.809061145 | MFAP1 | microfibril associated protein 1 [Source:HGNC Symbol;Acc:HGNC:7032] |
| ENSG00000140511 | HGLibA_20846 | 36 | -0.809061145 | HAPLN3 | hyaluronan and proteoglycan link protein 3 [Source:HGNC Symbol;Acc:HGNC:21446] |
| ENSG00000140943 | HGLibA_28535 | 36 | -0.809061145 | MBTPS1 | site 1 [Source:HGNC Symbol;Acc:HGNC:15456]" |
| ENSG00000140986 | HGLibA_41980 | 36 | -0.809061145 | RPL3L | ribosomal protein L3 like [Source:HGNC Symbol;Acc:HGNC:10351] |
| ENSG00000142961 | HGLibA_29601 | 36 | -0.809061145 | MOB3C | MOB kinase activator 3C [Source:HGNC Symbol;Acc:HGNC:29800] |
| ENSG00000143367 | HGLibA_52327 | 36 | -0.809061145 | TUFT1 | tuftelin 1 [Source:HGNC Symbol;Acc:HGNC:12422] |
| ENSG00000143507 | HGLibA_14131 | 36 | -0.809061145 | DUSP10 | dual specificity phosphatase 10 [Source:HGNC Symbol;Acc:HGNC:3065] |
| ENSG00000143761 | HGLibA_02646 | 36 | -0.809061145 | ARF1 | ADP ribosylation factor 1 [Source:HGNC Symbol;Acc:HGNC:652] |
| ENSG00000144381 | HGLibA_22330 | 36 | -0.809061145 | HSPD1 | heat shock protein family D (Hsp60) member 1 [Source:HGNC Symbol;Acc:HGNC:5261] |
| ENSG00000144644 | HGLibA_18429 | 36 | -0.809061145 | GADL1 | glutamate decarboxylase like 1 [Source:HGNC Symbol;Acc:HGNC:27949] |
| ENSG00000145777 | HGLibA_51877 | 36 | -0.809061145 | TSLP | thymic stromal lymphopoietin [Source:HGNC Symbol;Acc:HGNC:30743] |
| ENSG00000146555 | HGLibA_43152 | 36 | -0.809061145 | SDK1 | sidekick cell adhesion molecule 1 [Source:HGNC Symbol;Acc:HGNC:19307] |
| ENSG00000147687 | HGLibA_48441 | 36 | -0.809061145 | TATDN1 | TatD DNase domain containing 1 [Source:HGNC Symbol;Acc:HGNC:24220] |
| ENSG00000148730 | HGLibA_14830 | 36 | -0.809061145 | EIF4EBP2 | eukaryotic translation initiation factor 4E binding protein 2 [Source:HGNC Symbol;Acc:HGNC:3289] |
| ENSG00000149187 | HGLibA_09004 | 36 | -0.809061145 | CELF1 | CUGBP Elav-like family member 1 [Source:HGNC Symbol;Acc:HGNC:2549] |
| ENSG00000149532 | HGLibA_11016 | 36 | -0.809061145 | CPSF7 | cleavage and polyadenylation specific factor 7 [Source:HGNC Symbol;Acc:HGNC:30098] |
| ENSG00000149806 | HGLibA_16925 | 36 | -0.809061145 | FAU | ubiquitin like and ribosomal protein S30 fusion [Source:HGNC Symbol;Acc:HGNC:3597]" |
| ENSG00000153904 | HGLibA_12587 | 36 | -0.809061145 | DDAH1 | dimethylarginine dimethylaminohydrolase 1 [Source:HGNC Symbol;Acc:HGNC:2715] |
| ENSG00000157653 | HGLibA_06735 | 36 | -0.809061145 | C9orf43 | chromosome 9 open reading frame 43 [Source:HGNC Symbol;Acc:HGNC:23570] |
| ENSG00000159164 | HGLibA_47875 | 36 | -0.809061145 | SV2A | synaptic vesicle glycoprotein 2A [Source:HGNC Symbol;Acc:HGNC:20566] |
| ENSG00000159202 | HGLibA_52674 | 36 | -0.809061145 | UBE2Z | ubiquitin conjugating enzyme E2 Z [Source:HGNC Symbol;Acc:HGNC:25847] |
| ENSG00000159403 | HGLibA_05969 | 36 | -0.809061145 | C1R | complement C1r [Source:HGNC Symbol;Acc:HGNC:1246] |
| ENSG00000159588 | HGLibA_07688 | 36 | -0.809061145 | CCDC17 | coiled-coil domain containing 17 [Source:HGNC Symbol;Acc:HGNC:26574] |
| ENSG00000159882 | HGLibA_55636 | 36 | -0.809061145 | ZNF230 | zinc finger protein 230 [Source:HGNC Symbol;Acc:HGNC:13024] |
| ENSG00000160688 | HGLibA_17602 | 36 | -0.809061145 | FLAD1 | flavin adenine dinucleotide synthetase 1 [Source:HGNC Symbol;Acc:HGNC:24671] |
| ENSG00000160691 | HGLibA_44001 | 36 | -0.809061145 | SHC1 | SHC adaptor protein 1 [Source:HGNC Symbol;Acc:HGNC:10840] |
| ENSG00000161958 | HGLibA_17367 | 36 | -0.809061145 | FGF11 | fibroblast growth factor 11 [Source:HGNC Symbol;Acc:HGNC:3667] |
| ENSG00000162757 | HGLibA_05893 | 36 | -0.809061145 | C1orf74 | chromosome 1 open reading frame 74 [Source:HGNC Symbol;Acc:HGNC:26319] |
| ENSG00000163040 | HGLibA_07862 | 36 | -0.809061145 | CCDC74A | coiled-coil domain containing 74A [Source:HGNC Symbol;Acc:HGNC:25197] |
| ENSG00000163156 | HGLibA_43010 | 36 | -0.809061145 | SCNM1 | sodium channel modifier 1 [Source:HGNC Symbol;Acc:HGNC:23136] |
| ENSG00000163380 | HGLibA_26724 | 36 | -0.809061145 | LMOD3 | leiomodin 3 [Source:HGNC Symbol;Acc:HGNC:6649] |
| ENSG00000163568 | HGLibA_01353 | 36 | -0.809061145 | AIM2 | absent in melanoma 2 [Source:HGNC Symbol;Acc:HGNC:357] |
| ENSG00000163825 | HGLibA_42427 | 36 | -0.809061145 | RTP3 | receptor transporter protein 3 [Source:HGNC Symbol;Acc:HGNC:15572] |
| ENSG00000163913 | HGLibA_22743 | 36 | -0.809061145 | IFT122 | intraflagellar transport 122 [Source:HGNC Symbol;Acc:HGNC:13556] |
| ENSG00000163931 | HGLibA_49587 | 36 | -0.809061145 | TKT | transketolase [Source:HGNC Symbol;Acc:HGNC:11834] |
| ENSG00000164118 | HGLibA_09140 | 36 | -0.809061145 | CEP44 | centrosomal protein 44 [Source:HGNC Symbol;Acc:HGNC:29356] |
| ENSG00000164512 | HGLibA_02127 | 36 | -0.809061145 | ANKRD55 | ankyrin repeat domain 55 [Source:HGNC Symbol;Acc:HGNC:25681] |
| ENSG00000164924 | HGLibA_54754 | 36 | -0.809061145 | YWHAZ | tyrosine 3-monooxygenase/tryptophan 5-monooxygenase activation protein zeta [Source:HGNC Symbol;Acc:HGNC:12855] |
| ENSG00000164978 | HGLibA_32967 | 36 | -0.809061145 | NUDT2 | nudix hydrolase 2 [Source:HGNC Symbol;Acc:HGNC:8049] |
| ENSG00000165584 | HGLibA_47206 | 36 | -0.809061145 | SSX3 | SSX family member 3 [Source:HGNC Symbol;Acc:HGNC:11337] |
| ENSG00000165584 | HGLibA_47208 | 36 | -0.809061145 | SSX3 | SSX family member 3 [Source:HGNC Symbol;Acc:HGNC:11337] |
| ENSG00000166090 | HGLibA_23107 | 36 | -0.809061145 | IL25 | interleukin 25 [Source:HGNC Symbol;Acc:HGNC:13765] |
| ENSG00000166479 | HGLibA_50693 | 36 | -0.809061145 | TMX3 | thioredoxin related transmembrane protein 3 [Source:HGNC Symbol;Acc:HGNC:24718] |
| ENSG00000166575 | HGLibA_49949 | 36 | -0.809061145 | TMEM135 | transmembrane protein 135 [Source:HGNC Symbol;Acc:HGNC:26167] |
| ENSG00000166743 | HGLibA_00568 | 36 | -0.809061145 | ACSM1 | acyl-CoA synthetase medium chain family member 1 [Source:HGNC Symbol;Acc:HGNC:18049] |
| ENSG00000166949 | HGLibA_45575 | 36 | -0.809061145 | SMAD3 | SMAD family member 3 [Source:HGNC Symbol;Acc:HGNC:6769] |
| ENSG00000167202 | HGLibA_48527 | 36 | -0.809061145 | TBC1D2B | TBC1 domain family member 2B [Source:HGNC Symbol;Acc:HGNC:29183] |
| ENSG00000167646 | HGLibA_13466 | 36 | -0.809061145 | DNAAF3 | dynein axonemal assembly factor 3 [Source:HGNC Symbol;Acc:HGNC:30492] |
| ENSG00000167720 | HGLibA_47039 | 36 | -0.809061145 | SRR | serine racemase [Source:HGNC Symbol;Acc:HGNC:14398] |
| ENSG00000167771 | HGLibA_40713 | 36 | -0.809061145 | RCOR2 | REST corepressor 2 [Source:HGNC Symbol;Acc:HGNC:27455] |
| ENSG00000167780 | HGLibA_46117 | 36 | -0.809061145 | SOAT2 | sterol O-acyltransferase 2 [Source:HGNC Symbol;Acc:HGNC:11178] |
| ENSG00000167861 | HGLibA_21294 | 36 | -0.809061145 | HID1 | HID1 domain containing [Source:HGNC Symbol;Acc:HGNC:15736] |
| ENSG00000168131 | HGLibA_33766 | 36 | -0.809061145 | OR2B2 | olfactory receptor family 2 subfamily B member 2 [Source:HGNC Symbol;Acc:HGNC:13966] |
| ENSG00000169385 | HGLibA_41404 | 36 | -0.809061145 | RNASE2 | ribonuclease A family member 2 [Source:HGNC Symbol;Acc:HGNC:10045] |
| ENSG00000169877 | HGLibA_01322 | 36 | -0.809061145 | AHSP | alpha hemoglobin stabilizing protein [Source:HGNC Symbol;Acc:HGNC:18075] |
| ENSG00000170011 | HGLibA_30919 | 36 | -0.809061145 | MYRIP | myosin VIIA and Rab interacting protein [Source:HGNC Symbol;Acc:HGNC:19156] |
| ENSG00000170264 | HGLibA_16215 | 36 | -0.809061145 | FAM161A | centrosomal protein [Source:HGNC Symbol;Acc:HGNC:25808]" |
| ENSG00000170271 | HGLibA_16932 | 36 | -0.809061145 | FAXDC2 | fatty acid hydroxylase domain containing 2 [Source:HGNC Symbol;Acc:HGNC:1334] |
| ENSG00000170464 | HGLibA_13588 | 36 | -0.809061145 | DNAJC18 | DnaJ heat shock protein family (Hsp40) member C18 [Source:HGNC Symbol;Acc:HGNC:28429] |
| ENSG00000170745 | HGLibA_24390 | 36 | -0.809061145 | KCNS3 | potassium voltage-gated channel modifier subfamily S member 3 [Source:HGNC Symbol;Acc:HGNC:6302] |
| ENSG00000170860 | HGLibA_27539 | 36 | -0.809061145 | LSM3 | U6 small nuclear RNA and mRNA degradation associated [Source:HGNC Symbol;Acc:HGNC:17874]" |
| ENSG00000171501 | HGLibA_33707 | 36 | -0.809061145 | OR1N2 | olfactory receptor family 1 subfamily N member 2 [Source:HGNC Symbol;Acc:HGNC:15111] |
| ENSG00000171720 | HGLibA_21008 | 36 | -0.809061145 | HDAC3 | histone deacetylase 3 [Source:HGNC Symbol;Acc:HGNC:4854] |
| ENSG00000171798 | HGLibA_25340 | 36 | -0.809061145 | KNDC1 | kinase non-catalytic C-lobe domain containing 1 [Source:HGNC Symbol;Acc:HGNC:29374] |
| ENSG00000172432 | HGLibA_20605 | 36 | -0.809061145 | GTPBP2 | GTP binding protein 2 [Source:HGNC Symbol;Acc:HGNC:4670] |
| ENSG00000172969 | HGLibA_18002 | 36 | -0.809061145 | FRG2C | FSHD region gene 2 family member C [Source:HGNC Symbol;Acc:HGNC:33626] |
| ENSG00000174456 | HGLibA_05299 | 36 | -0.809061145 | C12orf76 | chromosome 12 open reading frame 76 [Source:HGNC Symbol;Acc:HGNC:33790] |
| ENSG00000175344 | HGLibA_09583 | 36 | -0.809061145 | CHRNA7 | cholinergic receptor nicotinic alpha 7 subunit [Source:HGNC Symbol;Acc:HGNC:1960] |
| ENSG00000175787 | HGLibA_55520 | 36 | -0.809061145 | ZNF169 | zinc finger protein 169 [Source:HGNC Symbol;Acc:HGNC:12957] |
| ENSG00000176402 | HGLibA_19183 | 36 | -0.809061145 | GJC3 | gap junction protein gamma 3 [Source:HGNC Symbol;Acc:HGNC:17495] |
| ENSG00000176697 | HGLibA_04386 | 36 | -0.809061145 | BDNF | brain derived neurotrophic factor [Source:HGNC Symbol;Acc:HGNC:1033] |
| ENSG00000177125 | HGLibA_54866 | 36 | -0.809061145 | ZBTB34 | zinc finger and BTB domain containing 34 [Source:HGNC Symbol;Acc:HGNC:31446] |
| ENSG00000177483 | HGLibA_40556 | 36 | -0.809061145 | RBM44 | RNA binding motif protein 44 [Source:HGNC Symbol;Acc:HGNC:24756] |
| ENSG00000178449 | HGLibA_10809 | 36 | -0.809061145 | COX14 | cytochrome c oxidase assembly factor COX14 [Source:HGNC Symbol;Acc:HGNC:28216] |
| ENSG00000179627 | HGLibA_54885 | 36 | -0.809061145 | ZBTB42 | zinc finger and BTB domain containing 42 [Source:HGNC Symbol;Acc:HGNC:32550] |
| ENSG00000180104 | HGLibA_15736 | 36 | -0.809061145 | EXOC3 | exocyst complex component 3 [Source:HGNC Symbol;Acc:HGNC:30378] |
| ENSG00000180182 | HGLibA_28754 | 36 | -0.809061145 | MED14 | mediator complex subunit 14 [Source:HGNC Symbol;Acc:HGNC:2370] |
| ENSG00000180370 | HGLibA_35044 | 36 | -0.809061145 | PAK2 | p21 (RAC1) activated kinase 2 [Source:HGNC Symbol;Acc:HGNC:8591] |
| ENSG00000181467 | HGLibA_40239 | 36 | -0.809061145 | RAP2B | member of RAS oncogene family [Source:HGNC Symbol;Acc:HGNC:9862]" |
| ENSG00000181733 | HGLibA_33911 | 36 | -0.809061145 | OR2Z1 | olfactory receptor family 2 subfamily Z member 1 [Source:HGNC Symbol;Acc:HGNC:15391] |
| ENSG00000181915 | HGLibA_01007 | 36 | -0.809061145 | ADO | 2-aminoethanethiol dioxygenase [Source:HGNC Symbol;Acc:HGNC:23506] |
| ENSG00000182687 | HGLibA_18589 | 36 | -0.809061145 | GALR2 | galanin receptor 2 [Source:HGNC Symbol;Acc:HGNC:4133] |
| ENSG00000182901 | HGLibA_41103 | 36 | -0.809061145 | RGS7 | regulator of G protein signaling 7 [Source:HGNC Symbol;Acc:HGNC:10003] |
| ENSG00000183668 | HGLibA_39058 | 36 | -0.809061145 | PSG9 | pregnancy specific beta-1-glycoprotein 9 [Source:HGNC Symbol;Acc:HGNC:9526] |
| ENSG00000183696 | HGLibA_53087 | 36 | -0.809061145 | UPP1 | uridine phosphorylase 1 [Source:HGNC Symbol;Acc:HGNC:12576] |
| ENSG00000183775 | HGLibA_24431 | 36 | -0.809061145 | KCTD16 | potassium channel tetramerization domain containing 16 [Source:HGNC Symbol;Acc:HGNC:29244] |
| ENSG00000183780 | HGLibA_45006 | 36 | -0.809061145 | SLC35F3 | solute carrier family 35 member F3 [Source:HGNC Symbol;Acc:HGNC:23616] |
| ENSG00000184349 | HGLibA_14594 | 36 | -0.809061145 | EFNA5 | ephrin A5 [Source:HGNC Symbol;Acc:HGNC:3225] |
| ENSG00000185829 | HGLibA_02950 | 36 | -0.809061145 | ARL17A | ADP ribosylation factor like GTPase 17A [Source:HGNC Symbol;Acc:HGNC:24096] |
| ENSG00000186088 | HGLibA_20396 | 36 | -0.809061145 | GSAP | gamma-secretase activating protein [Source:HGNC Symbol;Acc:HGNC:28042] |

| | | | | | |
|---|---|---|---|---|---|
| ENSG00000186765 | HGLibA_18072 | 36 | -0.809061145 | FSCN2 | retinal [Source:HGNC Symbol;Acc:HGNC:3960]" |
| ENSG00000187701 | HGLibA_33866 | 36 | -0.809061145 | OR2T27 | olfactory receptor family 2 subfamily T member 27 [Source:HGNC Symbol;Acc:HGNC:31252] |
| ENSG00000189052 | HGLibA_09309 | 36 | -0.809061145 | CGB5 | chorionic gonadotropin subunit beta 5 [Source:HGNC Symbol;Acc:HGNC:16452] |
| ENSG00000189164 | HGLibA_56168 | 36 | -0.809061145 | ZNF527 | zinc finger protein 527 [Source:HGNC Symbol;Acc:HGNC:29385] |
| ENSG00000189221 | HGLibA_28107 | 36 | -0.809061145 | MAOA | monoamine oxidase A [Source:HGNC Symbol;Acc:HGNC:6833] |
| ENSG00000196092 | HGLibA_35308 | 36 | -0.809061145 | PAX5 | paired box 5 [Source:HGNC Symbol;Acc:HGNC:8619] |
| ENSG00000196800 | HGLibA_46687 | 36 | -0.809061145 | SPINK14 | Kazal type 14 (putative) [Source:HGNC Symbol;Acc:HGNC:33825]" |
| ENSG00000196873 | HGLibA_07435 | 36 | -0.809061145 | CBWD3 | COBW domain containing 3 [Source:HGNC Symbol;Acc:HGNC:18519] |
| ENSG00000196998 | HGLibA_54096 | 36 | -0.809061145 | WDR45 | WD repeat domain 45 [Source:HGNC Symbol;Acc:HGNC:28912] |
| ENSG00000197299 | HGLibA_04549 | 36 | -0.809061145 | BLM | BLM RecQ like helicase [Source:HGNC Symbol;Acc:HGNC:1058] |
| ENSG00000197415 | HGLibA_53622 | 36 | -0.809061145 | VEPH1 | ventricular zone expressed PH domain containing 1 [Source:HGNC Symbol;Acc:HGNC:25735] |
| ENSG00000197506 | HGLibA_44838 | 36 | -0.809061145 | SLC28A3 | solute carrier family 28 member 3 [Source:HGNC Symbol;Acc:HGNC:16484] |
| ENSG00000197728 | HGLibA_42120 | 36 | -0.809061145 | RPS26 | ribosomal protein S26 [Source:HGNC Symbol;Acc:HGNC:10414] |
| ENSG00000197905 | HGLibA_48935 | 36 | -0.809061145 | TEAD4 | TEA domain transcription factor 4 [Source:HGNC Symbol;Acc:HGNC:11717] |
| ENSG00000203857 | HGLibA_22205 | 36 | -0.809061145 | HSD3B1 | 3 beta- and steroid delta-isomerase 1 [Source:HGNC Symbol;Acc:HGNC:5217]" |
| ENSG00000205549 | HGLibA_06778 | 36 | -0.809061145 | C9orf92 | chromosome 9 open reading frame 92 [Source:HGNC Symbol;Acc:HGNC:19054] |
| ENSG00000205777 | HGLibA_18431 | 36 | -0.809061145 | GAGE1 | G antigen 1 [Source:HGNC Symbol;Acc:HGNC:4098] |
| ENSG00000212807 | HGLibA_33737 | 36 | -0.809061145 | OR2A42 | olfactory receptor family 2 subfamily A member 42 [Source:HGNC Symbol;Acc:HGNC:31230] |
| ENSG00000213096 | HGLibA_55675 | 36 | -0.809061145 | ZNF254 | zinc finger protein 254 [Source:HGNC Symbol;Acc:HGNC:13047] |
| ENSG00000213337 | HGLibA_02092 | 36 | -0.809061145 | ANKRD39 | ankyrin repeat domain 39 [Source:HGNC Symbol;Acc:HGNC:28640] |
| ENSG00000213339 | HGLibA_39760 | 36 | -0.809061145 | QTRT1 | queuine tRNA-ribosyltransferase catalytic subunit 1 [Source:HGNC Symbol;Acc:HGNC:23797] |
| ENSG00000213780 | HGLibA_20560 | 36 | -0.809061145 | GTF2H4 | general transcription factor IIH subunit 4 [Source:HGNC Symbol;Acc:HGNC:4658] |
| ENSG00000213920 | HGLibA_28706 | 36 | -0.809061145 | MDP1 | magnesium dependent phosphatase 1 [Source:HGNC Symbol;Acc:HGNC:28781] |
| ENSG00000213930 | HGLibA_18595 | 36 | -0.809061145 | GALT | galactose-1-phosphate uridylyltransferase [Source:HGNC Symbol;Acc:HGNC:4135] |
| ENSG00000214413 | HGLibA_04199 | 36 | -0.809061145 | BBIP1 | BBSome interacting protein 1 [Source:HGNC Symbol;Acc:HGNC:28093] |
| ENSG00000215717 | HGLibA_50041 | 36 | -0.809061145 | TMEM167B | transmembrane protein 167B [Source:HGNC Symbol;Acc:HGNC:30187] |
| ENSG00000215845 | HGLibA_52030 | 36 | -0.809061145 | TSTD1 | thiosulfate sulfurtransferase like domain containing 1 [Source:HGNC Symbol;Acc:HGNC:35410] |
| ENSG00000215845 | HGLibA_52031 | 36 | -0.809061145 | TSTD1 | thiosulfate sulfurtransferase like domain containing 1 [Source:HGNC Symbol;Acc:HGNC:35410] |
| ENSG00000224902 | HGLibA_18454 | 36 | -0.809061145 | GAGE12H | G antigen 12H [Source:HGNC Symbol;Acc:HGNC:31908] |
| ENSG00000225362 | HGLibA_11555 | 36 | -0.809061145 | CT62 | cancer/testis antigen 62 [Source:HGNC Symbol;Acc:HGNC:27286] |
| ENSG00000225921 | HGLibA_32274 | 36 | -0.809061145 | NOL7 | nucleolar protein 7 [Source:HGNC Symbol;Acc:HGNC:21040] |
| ENSG00000233917 | HGLibA_37717 | 36 | -0.809061145 | POTEB | POTE ankyrin domain family member B [Source:HGNC Symbol;Acc:HGNC:33734] |
| ENSG00000243284 | HGLibA_53851 | 36 | -0.809061145 | VSIG8 | V-set and immunoglobulin domain containing 8 [Source:HGNC Symbol;Acc:HGNC:32063] |
| ENSG00000249437 | HGLibA_31080 | 36 | -0.809061145 | NAIP | NLR family apoptosis inhibitory protein [Source:HGNC Symbol;Acc:HGNC:7634] |
| ENSG00000249853 | HGLibA_22136 | 36 | -0.809061145 | HS3ST5 | heparan sulfate-glucosamine 3-sulfotransferase 5 [Source:HGNC Symbol;Acc:HGNC:19419] |
| ENSG00000266524 | HGLibA_18855 | 36 | -0.809061145 | GDF10 | growth differentiation factor 10 [Source:HGNC Symbol;Acc:HGNC:4215] |
| ENSG00000268940 | HGLibA_11497 | 36 | -0.809061145 | CT45A1 | cancer/testis antigen family 45 member A1 [Source:HGNC Symbol;Acc:HGNC:33267] |
| ENSG00000271449 | HGLibA_11500 | 36 | -0.809061145 | CT45A2 | cancer/testis antigen family 45 member A2 [Source:HGNC Symbol;Acc:HGNC:28400] |
| ENSG00000285723 | HGLibA_29330 | 36 | -0.809061145 | MKKS | novel protein |
| FAM206A | HGLibA_16425 | 36 | -0.809061145 | FAM206A | - |
| HRASLS2 | HGLibA_22080 | 36 | -0.809061145 | HRASLS2 | - |
| hsa-mir-124-3 | HGLibA_57354 | 36 | -0.809061145 | hsa-mir-124-3 | - |
| hsa-mir-1271 | HGLibA_57525 | 36 | -0.809061145 | hsa-mir-1271 | - |
| hsa-mir-1302-3 | HGLibA_57701 | 36 | -0.809061145 | hsa-mir-1302-3 | - |
| hsa-mir-1914 | HGLibA_58060 | 36 | -0.809061145 | hsa-mir-1914 | - |
| hsa-mir-218-2 | HGLibA_58271 | 36 | -0.809061145 | hsa-mir-218-2 | - |
| hsa-mir-30c-2 | HGLibA_58482 | 36 | -0.809061145 | hsa-mir-30c-2 | - |
| hsa-mir-3158-2 | HGLibA_58707 | 36 | -0.809061145 | hsa-mir-3158-2 | - |
| hsa-mir-320a | HGLibA_58922 | 36 | -0.809061145 | hsa-mir-320a | - |
| hsa-mir-331 | HGLibA_58993 | 36 | -0.809061145 | hsa-mir-331 | - |
| hsa-mir-3656 | HGLibA_59170 | 36 | -0.809061145 | hsa-mir-3656 | - |
| hsa-mir-3936 | HGLibA_59605 | 36 | -0.809061145 | hsa-mir-3936 | - |
| hsa-mir-4427 | HGLibA_60095 | 36 | -0.809061145 | hsa-mir-4427 | - |
| hsa-mir-4471 | HGLibA_60290 | 36 | -0.809061145 | hsa-mir-4471 | - |
| hsa-mir-4501 | HGLibA_60432 | 36 | -0.809061145 | hsa-mir-4501 | - |
| hsa-mir-4512 | HGLibA_60492 | 36 | -0.809061145 | hsa-mir-4512 | - |
| hsa-mir-4653 | HGLibA_60719 | 36 | -0.809061145 | hsa-mir-4653 | - |
| hsa-mir-4688 | HGLibA_60864 | 36 | -0.809061145 | hsa-mir-4688 | - |
| hsa-mir-4710 | HGLibA_60939 | 36 | -0.809061145 | hsa-mir-4710 | - |
| hsa-mir-4761 | HGLibA_61142 | 36 | -0.809061145 | hsa-mir-4761 | - |
| hsa-mir-4776-2 | HGLibA_61212 | 36 | -0.809061145 | hsa-mir-4776-2 | - |
| hsa-mir-4999 | HGLibA_61396 | 36 | -0.809061145 | hsa-mir-4999 | - |
| hsa-mir-5006 | HGLibA_61429 | 36 | -0.809061145 | hsa-mir-5006 | - |
| hsa-mir-502 | HGLibA_61462 | 36 | -0.809061145 | hsa-mir-502 | - |
| hsa-mir-507 | HGLibA_61487 | 36 | -0.809061145 | hsa-mir-507 | - |
| hsa-mir-5100 | HGLibA_61550 | 36 | -0.809061145 | hsa-mir-5100 | - |
| hsa-mir-517a | HGLibA_61625 | 36 | -0.809061145 | hsa-mir-517a | - |
| hsa-mir-544a | HGLibA_61824 | 36 | -0.809061145 | hsa-mir-544a | - |
| hsa-mir-548ad | HGLibA_61866 | 36 | -0.809061145 | hsa-mir-548ad | - |
| hsa-mir-559 | HGLibA_62200 | 36 | -0.809061145 | hsa-mir-559 | - |
| hsa-mir-5694 | HGLibA_62311 | 36 | -0.809061145 | hsa-mir-5694 | - |
| hsa-mir-600 | HGLibA_62493 | 36 | -0.809061145 | hsa-mir-600 | - |
| hsa-mir-664b | HGLibA_62999 | 36 | -0.809061145 | hsa-mir-664b | - |
| hsa-mir-6731 | HGLibA_63077 | 36 | -0.809061145 | hsa-mir-6731 | - |
| hsa-mir-6747 | HGLibA_63141 | 36 | -0.809061145 | hsa-mir-6747 | - |
| hsa-mir-6830 | HGLibA_63499 | 36 | -0.809061145 | hsa-mir-6830 | - |
| hsa-mir-7706 | HGLibA_63957 | 36 | -0.809061145 | hsa-mir-7706 | - |
| hsa-mir-8069 | HGLibA_64116 | 36 | -0.809061145 | hsa-mir-8069 | - |
| hsa-mir-892c | HGLibA_64257 | 36 | -0.809061145 | hsa-mir-892c | - |
| KIAA0196 | HGLibA_24603 | 36 | -0.809061145 | KIAA0196 | - |
| KIAA0754 | HGLibA_24653 | 36 | -0.809061145 | KIAA0754 | - |
| LDOC1L | HGLibA_26259 | 36 | -0.809061145 | LDOC1L | - |
| LOC100996331 | HGLibA_26882 | 36 | -0.809061145 | LOC100996331 | - |
| LOC283710 | HGLibA_26912 | 36 | -0.809061145 | LOC283710 | - |
| PRAMEF16 | HGLibA_38297 | 36 | -0.809061145 | PRAMEF16 | - |
| RRNAD1 | HGLibA_42263 | 36 | -0.809061145 | RRNAD1 | - |
| SMIM11 | HGLibA_45722 | 36 | -0.809061145 | SMIM11 | - |
| SMIM11 | HGLibA_45723 | 36 | -0.809061145 | SMIM11 | - |
| UQCC | HGLibA_53094 | 36 | -0.809061145 | UQCC | - |
| C12orf10 | HGLibA_05223 | 35 | -0.818106488 | C12orf10 | - |
| C19orf66 | HGLibA_05693 | 35 | -0.818106488 | C19orf66 | - |
| C4orf26 | HGLibA_06309 | 35 | -0.818106488 | C4orf26 | - |
| C7orf55-LUC7L2 | HGLibA_06558 | 35 | -0.818106488 | C7orf55-LUC7L2 | - |
| ENSG00000002919 | HGLibA_46031 | 35 | -0.818106488 | SNX11 | sorting nexin 11 [Source:HGNC Symbol;Acc:HGNC:14975] |
| ENSG00000005339 | HGLibA_11109 | 35 | -0.818106488 | CREBBP | CREB binding protein [Source:HGNC Symbol;Acc:HGNC:2348] |
| ENSG00000010626 | HGLibA_27284 | 35 | -0.818106488 | LRRC23 | leucine rich repeat containing 23 [Source:HGNC Symbol;Acc:HGNC:19138] |
| ENSG00000018510 | HGLibA_01240 | 35 | -0.818106488 | AGPS | alkylglycerone phosphate synthase [Source:HGNC Symbol;Acc:HGNC:327] |
| ENSG00000023909 | HGLibA_18796 | 35 | -0.818106488 | GCLM | glutamate-cysteine ligase modifier subunit [Source:HGNC Symbol;Acc:HGNC:4312] |
| ENSG00000037042 | HGLibA_52306 | 35 | -0.818106488 | TUBG2 | tubulin gamma 2 [Source:HGNC Symbol;Acc:HGNC:12419] |
| ENSG00000037241 | HGLibA_41917 | 35 | -0.818106488 | RPL26L1 | ribosomal protein L26 like 1 [Source:HGNC Symbol;Acc:HGNC:17050] |
| ENSG00000041802 | HGLibA_27515 | 35 | -0.818106488 | LSG1 | large 60S subunit nuclear export GTPase 1 [Source:HGNC Symbol;Acc:HGNC:25652] |
| ENSG00000046774 | HGLibA_27963 | 35 | -0.818106488 | MAGEC2 | MAGE family member C2 [Source:HGNC Symbol;Acc:HGNC:13574] |
| ENSG00000051128 | HGLibA_21869 | 35 | -0.818106488 | HOMER3 | homer scaffold protein 3 [Source:HGNC Symbol;Acc:HGNC:17514] |
| ENSG00000051180 | HGLibA_40077 | 35 | -0.818106488 | RAD51 | RAD51 recombinase [Source:HGNC Symbol;Acc:HGNC:9817] |
| ENSG00000067064 | HGLibA_22555 | 35 | -0.818106488 | IDI1 | isopentenyl-diphosphate delta isomerase 1 [Source:HGNC Symbol;Acc:HGNC:5387] |
| ENSG00000068971 | HGLibA_38173 | 35 | -0.818106488 | PPP2R5B | protein phosphatase 2 regulatory subunit B'beta [Source:HGNC Symbol;Acc:HGNC:9310] |
| ENSG00000069667 | HGLibA_41775 | 35 | -0.818106488 | RORA | RAR related orphan receptor A [Source:HGNC Symbol;Acc:HGNC:10258] |
| ENSG00000070081 | HGLibA_32914 | 35 | -0.818106488 | NUCB2 | nucleobindin 2 [Source:HGNC Symbol;Acc:HGNC:8044] |
| ENSG00000070756 | HGLibA_34922 | 35 | -0.818106488 | PABPC1 | poly(A) binding protein cytoplasmic 1 [Source:HGNC Symbol;Acc:HGNC:8554] |
| ENSG00000074416 | HGLibA_29159 | 35 | -0.818106488 | MGLL | monoglyceride lipase [Source:HGNC Symbol;Acc:HGNC:17038] |
| ENSG00000079462 | HGLibA_34995 | 35 | -0.818106488 | PAFAH1B3 | platelet activating factor acetylhydrolase 1b catalytic subunit 3 [Source:HGNC Symbol;Acc:HGNC:8576] |
| ENSG00000079950 | HGLibA_47657 | 35 | -0.818106488 | STX7 | syntaxin 7 [Source:HGNC Symbol;Acc:HGNC:11442] |
| ENSG00000087470 | HGLibA_13685 | 35 | -0.818106488 | DNM1L | dynamin 1 like [Source:HGNC Symbol;Acc:HGNC:2973] |
| ENSG00000090339 | HGLibA_22495 | 35 | -0.818106488 | ICAM1 | intercellular adhesion molecule 1 [Source:HGNC Symbol;Acc:HGNC:5344] |
| ENSG00000099721 | HGLibA_01769 | 35 | -0.818106488 | AMELY | amelogenin Y-linked [Source:HGNC Symbol;Acc:HGNC:462] |
| ENSG00000099810 | HGLibA_30318 | 35 | -0.818106488 | MTAP | methylthioadenosine phosphorylase [Source:HGNC Symbol;Acc:HGNC:7413] |
| ENSG00000101193 | HGLibA_19057 | 35 | -0.818106488 | GID8 | GID complex subunit 8 homolog [Source:HGNC Symbol;Acc:HGNC:15857] |
| ENSG00000101213 | HGLibA_39385 | 35 | -0.818106488 | PTK6 | protein tyrosine kinase 6 [Source:HGNC Symbol;Acc:HGNC:9617] |
| ENSG00000102158 | HGLibA_28013 | 35 | -0.818106488 | MAGT1 | magnesium transporter 1 [Source:HGNC Symbol;Acc:HGNC:28880] |
| ENSG00000102174 | HGLibA_36327 | 35 | -0.818106488 | PHEX | phosphate regulating endopeptidase homolog X-linked [Source:HGNC Symbol;Acc:HGNC:8918] |
| ENSG00000102962 | HGLibA_07997 | 35 | -0.818106488 | CCL22 | C-C motif chemokine ligand 22 [Source:HGNC Symbol;Acc:HGNC:10621] |
| ENSG00000103249 | HGLibA_09838 | 35 | -0.818106488 | CLCN7 | chloride voltage-gated channel 7 [Source:HGNC Symbol;Acc:HGNC:2025] |
| ENSG00000103274 | HGLibA_32900 | 35 | -0.818106488 | NUBP1 | nucleotide binding protein 1 [Source:HGNC Symbol;Acc:HGNC:8041] |
| ENSG00000104635 | HGLibA_45103 | 35 | -0.818106488 | SLC39A14 | solute carrier family 39 member 14 [Source:HGNC Symbol;Acc:HGNC:20858] |
| ENSG00000104723 | HGLibA_52349 | 35 | -0.818106488 | TUSC3 | tumor suppressor candidate 3 [Source:HGNC Symbol;Acc:HGNC:30242] |
| ENSG00000104805 | HGLibA_32909 | 35 | -0.818106488 | NUCB1 | nucleobindin 1 [Source:HGNC Symbol;Acc:HGNC:8043] |
| ENSG00000105270 | HGLibA_10048 | 35 | -0.818106488 | CLIP3 | CAP-Gly domain containing linker protein 3 [Source:HGNC Symbol;Acc:HGNC:24314] |
| ENSG00000105640 | HGLibA_41889 | 35 | -0.818106488 | RPL18A | ribosomal protein L18a [Source:HGNC Symbol;Acc:HGNC:10311] |
| ENSG00000105649 | HGLibA_39906 | 35 | -0.818106488 | RAB3A | member RAS oncogene family [Source:HGNC Symbol;Acc:HGNC:9777]" |
| ENSG00000107099 | HGLibA_13758 | 35 | -0.818106488 | DOCK8 | dedicator of cytokinesis 8 [Source:HGNC Symbol;Acc:HGNC:19191] |
| ENSG00000108774 | HGLibA_39966 | 35 | -0.818106488 | RAB5C | member RAS oncogene family [Source:HGNC Symbol;Acc:HGNC:9785]" |
| ENSG00000108797 | HGLibA_10420 | 35 | -0.818106488 | CNTNAP1 | contactin associated protein 1 [Source:HGNC Symbol;Acc:HGNC:8011] |
| ENSG00000109270 | HGLibA_25996 | 35 | -0.818106488 | LAMTOR3 | MAPK and MTOR activator 3 [Source:HGNC Symbol;Acc:HGNC:15606]" |
| ENSG00000111110 | HGLibA_37982 | 35 | -0.818106488 | PPM1H | Mg2+/Mn2+ dependent 1H [Source:HGNC Symbol;Acc:HGNC:18583]" |
| ENSG00000111245 | HGLibA_30756 | 35 | -0.818106488 | MYL2 | myosin light chain 2 [Source:HGNC Symbol;Acc:HGNC:7583] |

| | | | | | |
|---|---|---|---|---|---|
| ENSG00000111605 | HGLibA_11013 | 35 | -0.818106488 | CPSF6 | cleavage and polyadenylation specific factor 6 [Source:HGNC Symbol;Acc:HGNC:13871] |
| ENSG00000112146 | HGLibA_17132 | 35 | -0.818106488 | FBXO9 | F-box protein 9 [Source:HGNC Symbol;Acc:HGNC:13588] |
| ENSG00000112210 | HGLibA_39844 | 35 | -0.818106488 | RAB23 | member RAS oncogene family [Source:HGNC Symbol;Acc:HGNC:14263]" |
| ENSG00000114374 | HGLibA_53420 | 35 | -0.818106488 | USP9Y | ubiquitin specific peptidase 9 Y-linked [Source:HGNC Symbol;Acc:HGNC:12633] |
| ENSG00000114737 | HGLibA_09737 | 35 | -0.818106488 | CISH | cytokine inducible SH2 containing protein [Source:HGNC Symbol;Acc:HGNC:1984] |
| ENSG00000115841 | HGLibA_41366 | 35 | -0.818106488 | RMDN2 | regulator of microtubule dynamics 2 [Source:HGNC Symbol;Acc:HGNC:26567] |
| ENSG00000115902 | HGLibA_44516 | 35 | -0.818106488 | SLC1A4 | solute carrier family 1 member 4 [Source:HGNC Symbol;Acc:HGNC:10942] |
| ENSG00000117360 | HGLibA_38717 | 35 | -0.818106488 | PRPF3 | pre-mRNA processing factor 3 [Source:HGNC Symbol;Acc:HGNC:17348] |
| ENSG00000117475 | HGLibA_04581 | 35 | -0.818106488 | BLZF1 | basic leucine zipper nuclear factor 1 [Source:HGNC Symbol;Acc:HGNC:1065] |
| ENSG00000118690 | HGLibA_03014 | 35 | -0.818106488 | ARMC2 | armadillo repeat containing 2 [Source:HGNC Symbol;Acc:HGNC:23045] |
| ENSG00000119630 | HGLibA_36233 | 35 | -0.818106488 | PGF | placental growth factor [Source:HGNC Symbol;Acc:HGNC:8893] |
| ENSG00000120265 | HGLibA_35625 | 35 | -0.818106488 | PCMT1 | protein-L-isoaspartate (D-aspartate) O-methyltransferase [Source:HGNC Symbol;Acc:HGNC:8728] |
| ENSG00000120471 | HGLibA_51051 | 35 | -0.818106488 | TP53AIP1 | tumor protein p53 regulated apoptosis inducing protein 1 [Source:HGNC Symbol;Acc:HGNC:29984] |
| ENSG00000121879 | HGLibA_36634 | 35 | -0.818106488 | PIK3CA | 5-bisphosphate 3-kinase catalytic subunit alpha [Source:HGNC Symbol;Acc:HGNC:8975]" |
| ENSG00000122482 | HGLibA_56447 | 35 | -0.818106488 | ZNF644 | zinc finger protein 644 [Source:HGNC Symbol;Acc:HGNC:29222] |
| ENSG00000122884 | HGLibA_34899 | 35 | -0.818106488 | P4HA1 | prolyl 4-hydroxylase subunit alpha 1 [Source:HGNC Symbol;Acc:HGNC:8546] |
| ENSG00000124422 | HGLibA_53305 | 35 | -0.818106488 | USP22 | ubiquitin specific peptidase 22 [Source:HGNC Symbol;Acc:HGNC:12621] |
| ENSG00000124602 | HGLibA_53022 | 35 | -0.818106488 | UNC5CL | unc-5 family C-terminal like [Source:HGNC Symbol;Acc:HGNC:21203] |
| ENSG00000127863 | HGLibA_50769 | 35 | -0.818106488 | TNFRSF19 | TNF receptor superfamily member 19 [Source:HGNC Symbol;Acc:HGNC:11915] |
| ENSG00000127995 | HGLibA_07282 | 35 | -0.818106488 | CASD1 | CAS1 domain containing 1 [Source:HGNC Symbol;Acc:HGNC:16014] |
| ENSG00000128606 | HGLibA_27269 | 35 | -0.818106488 | LRRC17 | leucine rich repeat containing 17 [Source:HGNC Symbol;Acc:HGNC:16895] |
| ENSG00000129873 | HGLibA_08915 | 35 | -0.818106488 | CDY2B | chromodomain Y-linked 2B [Source:HGNC Symbol;Acc:HGNC:23921] |
| ENSG00000130035 | HGLibA_18573 | 35 | -0.818106488 | GALNT8 | polypeptide N-acetylgalactosaminyltransferase 8 [Source:HGNC Symbol;Acc:HGNC:4130] |
| ENSG00000130701 | HGLibA_40444 | 35 | -0.818106488 | RBBP8NL | RBBP8 N-terminal like [Source:HGNC Symbol;Acc:HGNC:16144] |
| ENSG00000130950 | HGLibA_33107 | 35 | -0.818106488 | NUTM2F | NUT family member 2F [Source:HGNC Symbol;Acc:HGNC:23450] |
| ENSG00000131378 | HGLibA_40950 | 35 | -0.818106488 | RFTN1 | lipid raft linker 1 [Source:HGNC Symbol;Acc:HGNC:30278]" |
| ENSG00000132341 | HGLibA_40188 | 35 | -0.818106488 | RAN | member RAS oncogene family [Source:HGNC Symbol;Acc:HGNC:9846]" |
| ENSG00000132716 | HGLibA_12456 | 35 | -0.818106488 | DCAF8 | DDB1 and CUL4 associated factor 8 [Source:HGNC Symbol;Acc:HGNC:24891] |
| ENSG00000134758 | HGLibA_41514 | 35 | -0.818106488 | RNF138 | ring finger protein 138 [Source:HGNC Symbol;Acc:HGNC:17765] |
| ENSG00000135052 | HGLibA_19654 | 35 | -0.818106488 | GOLM1 | golgi membrane protein 1 [Source:HGNC Symbol;Acc:HGNC:15451] |
| ENSG00000135083 | HGLibA_08112 | 35 | -0.818106488 | CCNJL | cyclin J like [Source:HGNC Symbol;Acc:HGNC:25876] |
| ENSG00000136327 | HGLibA_32047 | 35 | -0.818106488 | NKX2-8 | NK2 homeobox 8 [Source:HGNC Symbol;Acc:HGNC:16364] |
| ENSG00000136750 | HGLibA_18414 | 35 | -0.818106488 | GAD2 | glutamate decarboxylase 2 [Source:HGNC Symbol;Acc:HGNC:4093] |
| ENSG00000136810 | HGLibA_52400 | 35 | -0.818106488 | TXN | thioredoxin [Source:HGNC Symbol;Acc:HGNC:12435] |
| ENSG00000136834 | HGLibA_33675 | 35 | -0.818106488 | OR1J1 | olfactory receptor family 1 subfamily J member 1 [Source:HGNC Symbol;Acc:HGNC:8208] |
| ENSG00000136854 | HGLibA_47662 | 35 | -0.818106488 | STXBP1 | syntaxin binding protein 1 [Source:HGNC Symbol;Acc:HGNC:11444] |
| ENSG00000136872 | HGLibA_01594 | 35 | -0.818106488 | ALDOB | fructose-bisphosphate B [Source:HGNC Symbol;Acc:HGNC:417]" |
| ENSG00000137880 | HGLibA_18786 | 35 | -0.818106488 | GCHFR | GTP cyclohydrolase I feedback regulator [Source:HGNC Symbol;Acc:HGNC:4194] |
| ENSG00000137992 | HGLibA_12406 | 35 | -0.818106488 | DBT | dihydrolipoamide branched chain transacylase E2 [Source:HGNC Symbol;Acc:HGNC:2698] |
| ENSG00000138030 | HGLibA_24576 | 35 | -0.818106488 | KHK | ketohexokinase [Source:HGNC Symbol;Acc:HGNC:6315] |
| ENSG00000138459 | HGLibA_44959 | 35 | -0.818106488 | SLC35A5 | solute carrier family 35 member A5 [Source:HGNC Symbol;Acc:HGNC:20792] |
| ENSG00000138496 | HGLibA_35248 | 35 | -0.818106488 | PARP9 | poly(ADP-ribose) polymerase family member 9 [Source:HGNC Symbol;Acc:HGNC:24118] |
| ENSG00000138670 | HGLibA_40333 | 35 | -0.818106488 | RASGEF1B | RasGEF domain family member 1B [Source:HGNC Symbol;Acc:HGNC:24881] |
| ENSG00000139193 | HGLibA_08316 | 35 | -0.818106488 | CD27 | CD27 molecule [Source:HGNC Symbol;Acc:HGNC:11922] |
| ENSG00000139679 | HGLibA_27068 | 35 | -0.818106488 | LPAR6 | lysophosphatidic acid receptor 6 [Source:HGNC Symbol;Acc:HGNC:15520] |
| ENSG00000140854 | HGLibA_24082 | 35 | -0.818106488 | KATNB1 | katanin regulatory subunit B1 [Source:HGNC Symbol;Acc:HGNC:6217] |
| ENSG00000141198 | HGLibA_50949 | 35 | -0.818106488 | TOM1L1 | target of myb1 like 1 membrane trafficking protein [Source:HGNC Symbol;Acc:HGNC:11983] |
| ENSG00000141385 | HGLibA_01117 | 35 | -0.818106488 | AFG3L2 | AFG3 like matrix AAA peptidase subunit 2 [Source:HGNC Symbol;Acc:HGNC:315] |
| ENSG00000142794 | HGLibA_31233 | 35 | -0.818106488 | NBPF3 | NBPF member 3 [Source:HGNC Symbol;Acc:HGNC:25076] |
| ENSG00000142961 | HGLibA_29599 | 35 | -0.818106488 | MOB3C | MOB kinase activator 3C [Source:HGNC Symbol;Acc:HGNC:29800] |
| ENSG00000143106 | HGLibA_39083 | 35 | -0.818106488 | PSMA5 | proteasome subunit alpha 5 [Source:HGNC Symbol;Acc:HGNC:9534] |
| ENSG00000143149 | HGLibA_01590 | 35 | -0.818106488 | ALDH9A1 | aldehyde dehydrogenase 9 family member A1 [Source:HGNC Symbol;Acc:HGNC:412] |
| ENSG00000143546 | HGLibA_42571 | 35 | -0.818106488 | S100A8 | S100 calcium binding protein A8 [Source:HGNC Symbol;Acc:HGNC:10498] |
| ENSG00000144355 | HGLibA_13371 | 35 | -0.818106488 | DLX1 | distal-less homeobox 1 [Source:HGNC Symbol;Acc:HGNC:2914] |
| ENSG00000144524 | HGLibA_10731 | 35 | -0.818106488 | COPS7B | COP9 signalosome subunit 7B [Source:HGNC Symbol;Acc:HGNC:16760] |
| ENSG00000144893 | HGLibA_28745 | 35 | -0.818106488 | MED12L | mediator complex subunit 12 like [Source:HGNC Symbol;Acc:HGNC:16050] |
| ENSG00000145908 | HGLibA_55771 | 35 | -0.818106488 | ZNF300 | zinc finger protein 300 [Source:HGNC Symbol;Acc:HGNC:13091] |
| ENSG00000146247 | HGLibA_36399 | 35 | -0.818106488 | PHIP | pleckstrin homology domain interacting protein [Source:HGNC Symbol;Acc:HGNC:15673] |
| ENSG00000146409 | HGLibA_44495 | 35 | -0.818106488 | SLC18B1 | solute carrier family 18 member B1 [Source:HGNC Symbol;Acc:HGNC:21573] |
| ENSG00000148950 | HGLibA_23228 | 35 | -0.818106488 | IMMP1L | inner mitochondrial membrane peptidase subunit 1 [Source:HGNC Symbol;Acc:HGNC:26317] |
| ENSG00000149346 | HGLibA_45567 | 35 | -0.818106488 | SLX4IP | SLX4 interacting protein [Source:HGNC Symbol;Acc:HGNC:16225] |
| ENSG00000149357 | HGLibA_25989 | 35 | -0.818106488 | LAMTOR1 | MAPK and MTOR activator 1 [Source:HGNC Symbol;Acc:HGNC:26068]" |
| ENSG00000149541 | HGLibA_03978 | 35 | -0.818106488 | B3GAT3 | 3-glucuronyltransferase 3 [Source:HGNC Symbol;Acc:HGNC:923]" |
| ENSG00000149654 | HGLibA_08666 | 35 | -0.818106488 | CDH22 | cadherin 22 [Source:HGNC Symbol;Acc:HGNC:13251] |
| ENSG00000150281 | HGLibA_11621 | 35 | -0.818106488 | CTF1 | cardiotrophin 1 [Source:HGNC Symbol;Acc:HGNC:2499] |
| ENSG00000152455 | HGLibA_47863 | 35 | -0.818106488 | SUV39H2 | suppressor of variegation 3-9 homolog 2 [Source:HGNC Symbol;Acc:HGNC:17287] |
| ENSG00000153485 | HGLibA_50319 | 35 | -0.818106488 | TMEM251 | transmembrane protein 251 [Source:HGNC Symbol;Acc:HGNC:20218] |
| ENSG00000153827 | HGLibA_51638 | 35 | -0.818106488 | TRIP12 | thyroid hormone receptor interactor 12 [Source:HGNC Symbol;Acc:HGNC:12306] |
| ENSG00000154760 | HGLibA_45490 | 35 | -0.818106488 | SLFN13 | schlafen family member 13 [Source:HGNC Symbol;Acc:HGNC:26481] |
| ENSG00000155849 | HGLibA_14930 | 35 | -0.818106488 | ELMO1 | engulfment and cell motility 1 [Source:HGNC Symbol;Acc:HGNC:16286] |
| ENSG00000156831 | HGLibA_32770 | 35 | -0.818106488 | NSMCE2 | SMC5-SMC6 complex SUMO ligase [Source:HGNC Symbol;Acc:HGNC:26513]" |
| ENSG00000159337 | HGLibA_36910 | 35 | -0.818106488 | PLA2G4D | phospholipase A2 group IVD [Source:HGNC Symbol;Acc:HGNC:30038] |
| ENSG00000159459 | HGLibA_52748 | 35 | -0.818106488 | UBR1 | ubiquitin protein ligase E3 component n-recognin 1 [Source:HGNC Symbol;Acc:HGNC:16808] |
| ENSG00000160218 | HGLibA_51300 | 35 | -0.818106488 | TRAPPC10 | trafficking protein particle complex 10 [Source:HGNC Symbol;Acc:HGNC:11868] |
| ENSG00000160781 | HGLibA_35167 | 35 | -0.818106488 | PAQR6 | progestin and adipoQ receptor family member 6 [Source:HGNC Symbol;Acc:HGNC:30132] |
| ENSG00000161929 | HGLibA_42945 | 35 | -0.818106488 | SCIMP | SLP adaptor and CSK interacting membrane protein [Source:HGNC Symbol;Acc:HGNC:33504] |
| ENSG00000162066 | HGLibA_01764 | 35 | -0.818106488 | AMDHD2 | amidohydrolase domain containing 2 [Source:HGNC Symbol;Acc:HGNC:24262] |
| ENSG00000162494 | HGLibA_27337 | 35 | -0.818106488 | LRRC38 | leucine rich repeat containing 38 [Source:HGNC Symbol;Acc:HGNC:27005] |
| ENSG00000162613 | HGLibA_18150 | 35 | -0.818106488 | FUBP1 | far upstream element binding protein 1 [Source:HGNC Symbol;Acc:HGNC:4004] |
| ENSG00000162878 | HGLibA_36807 | 35 | -0.818106488 | PKDCC | cytoplasmic [Source:HGNC Symbol;Acc:HGNC:25123]" |
| ENSG00000163216 | HGLibA_46814 | 35 | -0.818106488 | SPRR2D | small proline rich protein 2D [Source:HGNC Symbol;Acc:HGNC:11264] |
| ENSG00000163273 | HGLibA_32506 | 35 | -0.818106488 | NPPC | natriuretic peptide C [Source:HGNC Symbol;Acc:HGNC:7941] |
| ENSG00000163531 | HGLibA_31766 | 35 | -0.818106488 | NFASC | neurofascin [Source:HGNC Symbol;Acc:HGNC:29866] |
| ENSG00000163539 | HGLibA_09795 | 35 | -0.818106488 | CLASP2 | cytoplasmic linker associated protein 2 [Source:HGNC Symbol;Acc:HGNC:17078] |
| ENSG00000163568 | HGLibA_01351 | 35 | -0.818106488 | AIM2 | absent in melanoma 2 [Source:HGNC Symbol;Acc:HGNC:357] |
| ENSG00000163866 | HGLibA_45724 | 35 | -0.818106488 | SMIM12 | small integral membrane protein 12 [Source:HGNC Symbol;Acc:HGNC:25154] |
| ENSG00000164024 | HGLibA_28929 | 35 | -0.818106488 | METAP1 | methionyl aminopeptidase 1 [Source:HGNC Symbol;Acc:HGNC:15789] |
| ENSG00000164758 | HGLibA_28801 | 35 | -0.818106488 | MED30 | mediator complex subunit 30 [Source:HGNC Symbol;Acc:HGNC:23032] |
| ENSG00000165113 | HGLibA_19205 | 35 | -0.818106488 | GKAP1 | G kinase anchoring protein 1 [Source:HGNC Symbol;Acc:HGNC:17496] |
| ENSG00000165891 | HGLibA_14324 | 35 | -0.818106488 | E2F7 | E2F transcription factor 7 [Source:HGNC Symbol;Acc:HGNC:23820] |
| ENSG00000166002 | HGLibA_45672 | 35 | -0.818106488 | SMCO4 | single-pass membrane protein with coiled-coil domains 4 [Source:HGNC Symbol;Acc:HGNC:24810] |
| ENSG00000166250 | HGLibA_10072 | 35 | -0.818106488 | CLMP | CXADR like membrane protein [Source:HGNC Symbol;Acc:HGNC:24039] |
| ENSG00000166979 | HGLibA_15678 | 35 | -0.818106488 | EVA1C | eva-1 homolog C [Source:HGNC Symbol;Acc:HGNC:13239] |
| ENSG00000167098 | HGLibA_47798 | 35 | -0.818106488 | SUN5 | Sad1 and UNC84 domain containing 5 [Source:HGNC Symbol;Acc:HGNC:16252] |
| ENSG00000167157 | HGLibA_38896 | 35 | -0.818106488 | PRRX2 | paired related homeobox 2 [Source:HGNC Symbol;Acc:HGNC:21338] |
| ENSG00000167613 | HGLibA_25932 | 35 | -0.818106488 | LAIR1 | leukocyte associated immunoglobulin like receptor 1 [Source:HGNC Symbol;Acc:HGNC:6477] |
| ENSG00000167646 | HGLibA_13464 | 35 | -0.818106488 | DNAAF3 | dynein axonemal assembly factor 3 [Source:HGNC Symbol;Acc:HGNC:30492] |
| ENSG00000167778 | HGLibA_46851 | 35 | -0.818106488 | SPRYD3 | SPRY domain containing 3 [Source:HGNC Symbol;Acc:HGNC:25920] |
| ENSG00000168096 | HGLibA_02156 | 35 | -0.818106488 | ANKS3 | ankyrin repeat and sterile alpha motif domain containing 3 [Source:HGNC Symbol;Acc:HGNC:29422] |
| ENSG00000169410 | HGLibA_39480 | 35 | -0.818106488 | PTPN9 | non-receptor type 9 [Source:HGNC Symbol;Acc:HGNC:9661]" |
| ENSG00000169951 | HGLibA_56688 | 35 | -0.818106488 | ZNF764 | zinc finger protein 764 [Source:HGNC Symbol;Acc:HGNC:28200] |
| ENSG00000169955 | HGLibA_56669 | 35 | -0.818106488 | ZNF747 | zinc finger protein 747 [Source:HGNC Symbol;Acc:HGNC:28350] |
| ENSG00000170153 | HGLibA_41544 | 35 | -0.818106488 | RNF150 | ring finger protein 150 [Source:HGNC Symbol;Acc:HGNC:23138] |
| ENSG00000170271 | HGLibA_16930 | 35 | -0.818106488 | FAXDC2 | fatty acid hydroxylase domain containing 2 [Source:HGNC Symbol;Acc:HGNC:1334] |
| ENSG00000170571 | HGLibA_14999 | 35 | -0.818106488 | EMB | embigin [Source:HGNC Symbol;Acc:HGNC:30465] |
| ENSG00000171459 | HGLibA_33696 | 35 | -0.818106488 | OR1L6 | olfactory receptor family 1 subfamily L member 6 [Source:HGNC Symbol;Acc:HGNC:8218] |
| ENSG00000172199 | HGLibA_34556 | 35 | -0.818106488 | OR8U1 | olfactory receptor family 8 subfamily U member 1 [Source:HGNC Symbol;Acc:HGNC:19611] |
| ENSG00000174156 | HGLibA_20456 | 35 | -0.818106488 | GSTA3 | glutathione S-transferase alpha 3 [Source:HGNC Symbol;Acc:HGNC:4628] |
| ENSG00000174574 | HGLibA_01458 | 35 | -0.818106488 | AKIRIN1 | akirin 1 [Source:HGNC Symbol;Acc:HGNC:25744] |
| ENSG00000174948 | HGLibA_19924 | 35 | -0.818106488 | GPR149 | G protein-coupled receptor 149 [Source:HGNC Symbol;Acc:HGNC:23627] |
| ENSG00000175984 | HGLibA_12942 | 35 | -0.818106488 | DENND2C | DENN domain containing 2C [Source:HGNC Symbol;Acc:HGNC:24748] |
| ENSG00000176973 | HGLibA_16784 | 35 | -0.818106488 | FAM89B | family with sequence similarity 89 member B [Source:HGNC Symbol;Acc:HGNC:16708] |
| ENSG00000177182 | HGLibA_10166 | 35 | -0.818106488 | CLVS1 | clavesin 1 [Source:HGNC Symbol;Acc:HGNC:23139] |
| ENSG00000177465 | HGLibA_00481 | 35 | -0.818106488 | ACOT4 | acyl-CoA thioesterase 4 [Source:HGNC Symbol;Acc:HGNC:19748] |
| ENSG00000177548 | HGLibA_40002 | 35 | -0.818106488 | RABEP2 | RAB GTPase binding effector protein 2 [Source:HGNC Symbol;Acc:HGNC:24817]" |
| ENSG00000177721 | HGLibA_02233 | 35 | -0.818106488 | ANXA2R | annexin A2 receptor [Source:HGNC Symbol;Acc:HGNC:33463] |
| ENSG00000178498 | HGLibA_14085 | 35 | -0.818106488 | DTX3 | deltex E3 ubiquitin ligase 3 [Source:HGNC Symbol;Acc:HGNC:24457] |
| ENSG00000179532 | HGLibA_13677 | 35 | -0.818106488 | DNHD1 | dynein heavy chain domain 1 [Source:HGNC Symbol;Acc:HGNC:26532] |
| ENSG00000179600 | HGLibA_19801 | 35 | -0.818106488 | GPHB5 | glycoprotein hormone beta 5 [Source:HGNC Symbol;Acc:HGNC:18055] |
| ENSG00000180767 | HGLibA_09623 | 35 | -0.818106488 | CHST13 | carbohydrate sulfotransferase 13 [Source:HGNC Symbol;Acc:HGNC:21755] |
| ENSG00000180938 | HGLibA_56280 | 35 | -0.818106488 | ZNF572 | zinc finger protein 572 [Source:HGNC Symbol;Acc:HGNC:26758] |
| ENSG00000181449 | HGLibA_46252 | 35 | -0.818106488 | SOX2 | SRY-box 2 [Source:HGNC Symbol;Acc:HGNC:11195] |
| ENSG00000181847 | HGLibA_49483 | 35 | -0.818106488 | TIGIT | T cell immunoreceptor with Ig and ITIM domains [Source:HGNC Symbol;Acc:HGNC:26838] |
| ENSG00000182362 | HGLibA_54641 | 35 | -0.818106488 | YBEY | ybeY metalloendoribonuclease [Source:HGNC Symbol;Acc:HGNC:1299] |
| ENSG00000183569 | HGLibA_43480 | 35 | -0.818106488 | SERHL2 | serine hydrolase like 2 [Source:HGNC Symbol;Acc:HGNC:29446] |
| ENSG00000184194 | HGLibA_19969 | 35 | -0.818106488 | GPR173 | G protein-coupled receptor 173 [Source:HGNC Symbol;Acc:HGNC:18186] |
| ENSG00000184402 | HGLibA_47105 | 35 | -0.818106488 | SS18L1 | nBAF chromatin remodeling complex subunit [Source:HGNC Symbol;Acc:HGNC:15592]" |
| ENSG00000184911 | HGLibA_13437 | 35 | -0.818106488 | DMRTC1B | DMRT like family C1B [Source:HGNC Symbol;Acc:HGNC:31686] |
| ENSG00000185298 | HGLibA_07607 | 35 | -0.818106488 | CCDC137 | coiled-coil domain containing 137 [Source:HGNC Symbol;Acc:HGNC:33451] |
| ENSG00000185621 | HGLibA_26691 | 35 | -0.818106488 | LMLN | leishmanolysin like peptidase [Source:HGNC Symbol;Acc:HGNC:15991] |
| ENSG00000185668 | HGLibA_37766 | 35 | -0.818106488 | POU3F1 | POU class 3 homeobox 1 [Source:HGNC Symbol;Acc:HGNC:9214] |
| ENSG00000187950 | HGLibA_34793 | 35 | -0.818106488 | OVCH1 | ovochymase 1 [Source:HGNC Symbol;Acc:HGNC:23080] |
| ENSG00000188000 | HGLibA_34473 | 35 | -0.818106488 | OR7D2 | olfactory receptor family 7 subfamily D member 2 [Source:HGNC Symbol;Acc:HGNC:8378] |
| ENSG00000188219 | HGLibA_37730 | 35 | -0.818106488 | POTEE | POTE ankyrin domain family member E [Source:HGNC Symbol;Acc:HGNC:33895] |
| ENSG00000189180 | HGLibA_55831 | 35 | -0.818106488 | ZNF33A | zinc finger protein 33A [Source:HGNC Symbol;Acc:HGNC:13096] |

| | | | | | |
|---|---|---|---|---|---|
| ENSG00000196126 | HGLibA_21636 | 35 | -0.818106488 | HLA-DRB1 | DR beta 1 [Source:HGNC Symbol;Acc:HGNC:4948]" |
| ENSG00000196284 | HGLibA_47815 | 35 | -0.818106488 | SUPT3H | SAGA and STAGA complex component [Source:HGNC Symbol;Acc:HGNC:11466]" |
| ENSG00000196660 | HGLibA_44899 | 35 | -0.818106488 | SLC30A10 | solute carrier family 30 member 10 [Source:HGNC Symbol;Acc:HGNC:25355] |
| ENSG00000197355 | HGLibA_52510 | 35 | -0.818106488 | UAP1L1 | UDP-N-acetylglucosamine pyrophosphorylase 1 like 1 [Source:HGNC Symbol;Acc:HGNC:28082] |
| ENSG00000198315 | HGLibA_55364 | 35 | -0.818106488 | ZKSCAN8 | zinc finger with KRAB and SCAN domains 8 [Source:HGNC Symbol;Acc:HGNC:12983] |
| ENSG00000198440 | HGLibA_56313 | 35 | -0.818106488 | ZNF583 | zinc finger protein 583 [Source:HGNC Symbol;Acc:HGNC:26427] |
| ENSG00000198471 | HGLibA_42425 | 35 | -0.818106488 | RTP2 | receptor transporter protein 2 [Source:HGNC Symbol;Acc:HGNC:32486] |
| ENSG00000203485 | HGLibA_23280 | 35 | -0.818106488 | INF2 | FH2 and WH2 domain containing [Source:HGNC Symbol;Acc:HGNC:23791]" |
| ENSG00000204010 | HGLibA_22627 | 35 | -0.818106488 | IFIT1B | interferon induced protein with tetratricopeptide repeats 1B [Source:HGNC Symbol;Acc:HGNC:23442] |
| ENSG00000204172 | HGLibA_01162 | 35 | -0.818106488 | AGAP9 | ankyrin repeat and PH domain 9 [Source:HGNC Symbol;Acc:HGNC:23463]" |
| ENSG00000204370 | HGLibA_43149 | 35 | -0.818106488 | SDHD | succinate dehydrogenase complex subunit D [Source:HGNC Symbol;Acc:HGNC:10683] |
| ENSG00000204396 | HGLibA_53914 | 35 | -0.818106488 | VWA7 | von Willebrand factor A domain containing 7 [Source:HGNC Symbol;Acc:HGNC:13939] |
| ENSG00000205560 | HGLibA_11021 | 35 | -0.818106488 | CPT1B | carnitine palmitoyltransferase 1B [Source:HGNC Symbol;Acc:HGNC:2329] |
| ENSG00000205718 | HGLibA_28480 | 35 | -0.818106488 | MBD3L4 | methyl-CpG binding domain protein 3 like 4 [Source:HGNC Symbol;Acc:HGNC:37206] |
| ENSG00000205884 | HGLibA_12910 | 35 | -0.818106488 | DEFB136 | defensin beta 136 [Source:HGNC Symbol;Acc:HGNC:34433] |
| ENSG00000213965 | HGLibA_32963 | 35 | -0.818106488 | NUDT19 | nudix hydrolase 19 [Source:HGNC Symbol;Acc:HGNC:32036] |
| ENSG00000214193 | HGLibA_43943 | 35 | -0.818106488 | SH3D21 | SH3 domain containing 21 [Source:HGNC Symbol;Acc:HGNC:26236] |
| ENSG00000214711 | HGLibA_07156 | 35 | -0.818106488 | CAPN14 | calpain 14 [Source:HGNC Symbol;Acc:HGNC:16664] |
| ENSG00000215269 | HGLibA_18452 | 35 | -0.818106488 | GAGE12G | G antigen 12G [Source:HGNC Symbol;Acc:HGNC:31907] |
| ENSG00000215547 | HGLibA_12859 | 35 | -0.818106488 | DEFB115 | defensin beta 115 [Source:HGNC Symbol;Acc:HGNC:18096] |
| ENSG00000221914 | HGLibA_38145 | 35 | -0.818106488 | PPP2R2A | protein phosphatase 2 regulatory subunit Balpha [Source:HGNC Symbol;Acc:HGNC:9304] |
| ENSG00000221970 | HGLibA_33720 | 35 | -0.818106488 | OR2A1 | olfactory receptor family 2 subfamily A member 1 [Source:HGNC Symbol;Acc:HGNC:8229] |
| ENSG00000222047 | HGLibA_05080 | 35 | -0.818106488 | C10orf55 | chromosome 10 open reading frame 55 [Source:HGNC Symbol;Acc:HGNC:31008] |
| ENSG00000230989 | HGLibA_22151 | 35 | -0.818106488 | HSBP1 | heat shock factor binding protein 1 [Source:HGNC Symbol;Acc:HGNC:5203] |
| ENSG00000232838 | HGLibA_36075 | 35 | -0.818106488 | PET117 | PET117 homolog [Source:HGNC Symbol;Acc:HGNC:40045] |
| ENSG00000239713 | HGLibA_02492 | 35 | -0.818106488 | APOBEC3G | apolipoprotein B mRNA editing enzyme catalytic subunit 3G [Source:HGNC Symbol;Acc:HGNC:17357] |
| ENSG00000240021 | HGLibA_49100 | 35 | -0.818106488 | TEX35 | testis expressed 35 [Source:HGNC Symbol;Acc:HGNC:25366] |
| ENSG00000248405 | HGLibA_38845 | 35 | -0.818106488 | PRR5-ARHGAP8 | PRR5-ARHGAP8 readthrough [Source:HGNC Symbol;Acc:HGNC:34512] |
| ENSG00000248920 | HGLibA_53236 | 35 | -0.818106488 | USP17L19 | ubiquitin specific peptidase 17-like family member 19 [Source:HGNC Symbol;Acc:HGNC:44447] |
| ENSG00000249115 | HGLibA_20887 | 35 | -0.818106488 | HAUS5 | HAUS augmin like complex subunit 5 [Source:HGNC Symbol;Acc:HGNC:29130] |
| ENSG00000253910 | HGLibA_35552 | 35 | -0.818106488 | PCDHGB2 | 2 [Source:HGNC Symbol;Acc:HGNC:8709]" |
| ENSG00000265681 | HGLibA_41879 | 35 | -0.818106488 | RPL17 | ribosomal protein L17 [Source:HGNC Symbol;Acc:HGNC:10307] |
| FAM73A | HGLibA_16717 | 35 | -0.818106488 | FAM73A | - |
| HIST2H2AA4 | HGLibA_21524 | 35 | -0.818106488 | HIST2H2AA4 | - |
| hsa-mir-1255a | HGLibA_57421 | 35 | -0.818106488 | hsa-mir-1255a | - |
| hsa-mir-1255b-2 | HGLibA_57426 | 35 | -0.818106488 | hsa-mir-1255b-2 | - |
| hsa-mir-1290 | HGLibA_57635 | 35 | -0.818106488 | hsa-mir-1290 | - |
| hsa-mir-196b | HGLibA_58100 | 35 | -0.818106488 | hsa-mir-196b | - |
| hsa-mir-2278 | HGLibA_58317 | 35 | -0.818106488 | hsa-mir-2278 | - |
| hsa-mir-2467 | HGLibA_58347 | 35 | -0.818106488 | hsa-mir-2467 | - |
| hsa-mir-29b-1 | HGLibA_58412 | 35 | -0.818106488 | hsa-mir-29b-1 | - |
| hsa-mir-3189 | HGLibA_58855 | 35 | -0.818106488 | hsa-mir-3189 | - |
| hsa-mir-3610 | HGLibA_59072 | 35 | -0.818106488 | hsa-mir-3610 | - |
| hsa-mir-3683 | HGLibA_59287 | 35 | -0.818106488 | hsa-mir-3683 | - |
| hsa-mir-3922 | HGLibA_59564 | 35 | -0.818106488 | hsa-mir-3922 | - |
| hsa-mir-3960 | HGLibA_59642 | 35 | -0.818106488 | hsa-mir-3960 | - |
| hsa-mir-4251 | HGLibA_59712 | 35 | -0.818106488 | hsa-mir-4251 | - |
| hsa-mir-4287 | HGLibA_59855 | 35 | -0.818106488 | hsa-mir-4287 | - |
| hsa-mir-4319 | HGLibA_59995 | 35 | -0.818106488 | hsa-mir-4319 | - |
| hsa-mir-4464 | HGLibA_60263 | 35 | -0.818106488 | hsa-mir-4464 | - |
| hsa-mir-4465 | HGLibA_60266 | 35 | -0.818106488 | hsa-mir-4465 | - |
| hsa-mir-4487 | HGLibA_60366 | 35 | -0.818106488 | hsa-mir-4487 | - |
| hsa-mir-4513 | HGLibA_60497 | 35 | -0.818106488 | hsa-mir-4513 | - |
| hsa-mir-4715 | HGLibA_60959 | 35 | -0.818106488 | hsa-mir-4715 | - |
| hsa-mir-4743 | HGLibA_61070 | 35 | -0.818106488 | hsa-mir-4743 | - |
| hsa-mir-4786 | HGLibA_61253 | 35 | -0.818106488 | hsa-mir-4786 | - |
| hsa-mir-5089 | HGLibA_61504 | 35 | -0.818106488 | hsa-mir-5089 | - |
| hsa-mir-509-3 | HGLibA_61514 | 35 | -0.818106488 | hsa-mir-509-3 | - |
| hsa-mir-519a-2 | HGLibA_61716 | 35 | -0.818106488 | hsa-mir-519a-2 | - |
| hsa-mir-526a-2 | HGLibA_61794 | 35 | -0.818106488 | hsa-mir-526a-2 | - |
| hsa-mir-548ai | HGLibA_61883 | 35 | -0.818106488 | hsa-mir-548ai | - |
| hsa-mir-548d-1 | HGLibA_61962 | 35 | -0.818106488 | hsa-mir-548d-1 | - |
| hsa-mir-5582 | HGLibA_62167 | 35 | -0.818106488 | hsa-mir-5582 | - |
| hsa-mir-5583-2 | HGLibA_62175 | 35 | -0.818106488 | hsa-mir-5583-2 | - |
| hsa-mir-564 | HGLibA_62218 | 35 | -0.818106488 | hsa-mir-564 | - |
| hsa-mir-591 | HGLibA_62462 | 35 | -0.818106488 | hsa-mir-591 | - |
| hsa-mir-6088 | HGLibA_62613 | 35 | -0.818106488 | hsa-mir-6088 | - |
| hsa-mir-654 | HGLibA_62950 | 35 | -0.818106488 | hsa-mir-654 | - |
| hsa-mir-6794 | HGLibA_63355 | 35 | -0.818106488 | hsa-mir-6794 | - |
| hsa-mir-6887 | HGLibA_63732 | 35 | -0.818106488 | hsa-mir-6887 | - |
| hsa-mir-7159 | HGLibA_63864 | 35 | -0.818106488 | hsa-mir-7159 | - |
| hsa-mir-98 | HGLibA_64374 | 35 | -0.818106488 | hsa-mir-98 | - |
| KIAA1704 | HGLibA_24805 | 35 | -0.818106488 | KIAA1704 | - |
| LPPR4 | HGLibA_27121 | 35 | -0.818106488 | LPPR4 | - |
| PARK2 | HGLibA_35195 | 35 | -0.818106488 | PARK2 | - |
| PPAPDC2 | HGLibA_37832 | 35 | -0.818106488 | PPAPDC2 | - |
| AIM1 | HGLibA_01350 | 34 | -0.827151831 | AIM1 | - |
| C15orf59 | HGLibA_05422 | 34 | -0.827151831 | C15orf59 | - |
| C2orf40 | HGLibA_06122 | 34 | -0.827151831 | C2orf40 | - |
| DUS2L | HGLibA_14118 | 34 | -0.827151831 | DUS2L | - |
| ENSG00000007255 | HGLibA_51331 | 34 | -0.827151831 | TRAPPC6A | trafficking protein particle complex 6A [Source:HGNC Symbol;Acc:HGNC:23069] |
| ENSG00000007306 | HGLibA_08950 | 34 | -0.827151831 | CEACAM7 | carcinoembryonic antigen related cell adhesion molecule 7 [Source:HGNC Symbol;Acc:HGNC:1819] |
| ENSG00000008838 | HGLibA_28784 | 34 | -0.827151831 | MED24 | mediator complex subunit 24 [Source:HGNC Symbol;Acc:HGNC:22963] |
| ENSG00000011083 | HGLibA_45338 | 34 | -0.827151831 | SLC6A7 | solute carrier family 6 member 7 [Source:HGNC Symbol;Acc:HGNC:11054] |
| ENSG00000014164 | HGLibA_54973 | 34 | -0.827151831 | ZC3H3 | zinc finger CCCH-type containing 3 [Source:HGNC Symbol;Acc:HGNC:28972] |
| ENSG00000029993 | HGLibA_21697 | 34 | -0.827151831 | HMGB3 | high mobility group box 3 [Source:HGNC Symbol;Acc:HGNC:5004] |
| ENSG00000040341 | HGLibA_47408 | 34 | -0.827151831 | STAU2 | staufen double-stranded RNA binding protein 2 [Source:HGNC Symbol;Acc:HGNC:11371] |
| ENSG00000041353 | HGLibA_39858 | 34 | -0.827151831 | RAB27B | member RAS oncogene family [Source:HGNC Symbol;Acc:HGNC:9767]" |
| ENSG00000042753 | HGLibA_02316 | 34 | -0.827151831 | AP2S1 | adaptor related protein complex 2 subunit sigma 1 [Source:HGNC Symbol;Acc:HGNC:565] |
| ENSG00000048649 | HGLibA_42312 | 34 | -0.827151831 | RSF1 | remodeling and spacing factor 1 [Source:HGNC Symbol;Acc:HGNC:18118] |
| ENSG00000054148 | HGLibA_36457 | 34 | -0.827151831 | PHPT1 | phosphohistidine phosphatase 1 [Source:HGNC Symbol;Acc:HGNC:30033] |
| ENSG00000054598 | HGLibA_17822 | 34 | -0.827151831 | FOXC1 | forkhead box C1 [Source:HGNC Symbol;Acc:HGNC:3800] |
| ENSG00000058799 | HGLibA_54669 | 34 | -0.827151831 | YIPF1 | Yip1 domain family member 1 [Source:HGNC Symbol;Acc:HGNC:25231] |
| ENSG00000062524 | HGLibA_27612 | 34 | -0.827151831 | LTK | leukocyte receptor tyrosine kinase [Source:HGNC Symbol;Acc:HGNC:6721] |
| ENSG00000065621 | HGLibA_20491 | 34 | -0.827151831 | GSTO2 | glutathione S-transferase omega 2 [Source:HGNC Symbol;Acc:HGNC:23064] |
| ENSG00000070193 | HGLibA_17362 | 34 | -0.827151831 | FGF10 | fibroblast growth factor 10 [Source:HGNC Symbol;Acc:HGNC:3666] |
| ENSG00000070785 | HGLibA_14741 | 34 | -0.827151831 | EIF2B3 | eukaryotic translation initiation factor 2B subunit gamma [Source:HGNC Symbol;Acc:HGNC:3259] |
| ENSG00000072954 | HGLibA_50378 | 34 | -0.827151831 | TMEM38A | transmembrane protein 38A [Source:HGNC Symbol;Acc:HGNC:28462] |
| ENSG00000073050 | HGLibA_54583 | 34 | -0.827151831 | XRCC1 | X-ray repair cross complementing 1 [Source:HGNC Symbol;Acc:HGNC:12828] |
| ENSG00000076351 | HGLibA_45183 | 34 | -0.827151831 | SLC46A1 | solute carrier family 46 member 1 [Source:HGNC Symbol;Acc:HGNC:30521] |
| ENSG00000079739 | HGLibA_36261 | 34 | -0.827151831 | PGM1 | phosphoglucomutase 1 [Source:HGNC Symbol;Acc:HGNC:8905] |
| ENSG00000080561 | HGLibA_29238 | 34 | -0.827151831 | MID2 | midline 2 [Source:HGNC Symbol;Acc:HGNC:7096] |
| ENSG00000083845 | HGLibA_42153 | 34 | -0.827151831 | RPS5 | ribosomal protein S5 [Source:HGNC Symbol;Acc:HGNC:10426] |
| ENSG00000085465 | HGLibA_34799 | 34 | -0.827151831 | OVGP1 | oviductal glycoprotein 1 [Source:HGNC Symbol;Acc:HGNC:8524] |
| ENSG00000085788 | HGLibA_12605 | 34 | -0.827151831 | DDHD2 | DDHD domain containing 2 [Source:HGNC Symbol;Acc:HGNC:29106] |
| ENSG00000087338 | HGLibA_19392 | 34 | -0.827151831 | GMCL1 | spermatogenesis associated 1 [Source:HGNC Symbol;Acc:HGNC:23843]" |
| ENSG00000088876 | HGLibA_55842 | 34 | -0.827151831 | ZNF343 | zinc finger protein 343 [Source:HGNC Symbol;Acc:HGNC:16017] |
| ENSG00000091136 | HGLibA_25956 | 34 | -0.827151831 | LAMB1 | laminin subunit beta 1 [Source:HGNC Symbol;Acc:HGNC:6486] |
| ENSG00000095539 | HGLibA_43355 | 34 | -0.827151831 | SEMA4G | semaphorin 4G [Source:HGNC Symbol;Acc:HGNC:10735] |
| ENSG00000096654 | HGLibA_55546 | 34 | -0.827151831 | ZNF184 | zinc finger protein 184 [Source:HGNC Symbol;Acc:HGNC:12975] |
| ENSG00000100024 | HGLibA_53053 | 34 | -0.827151831 | UPB1 | beta-ureidopropionase 1 [Source:HGNC Symbol;Acc:HGNC:16297] |
| ENSG00000100364 | HGLibA_24669 | 34 | -0.827151831 | KIAA0930 | KIAA0930 [Source:HGNC Symbol;Acc:HGNC:1314] |
| ENSG00000100439 | HGLibA_00279 | 34 | -0.827151831 | ABHD4 | abhydrolase domain containing 4 [Source:HGNC Symbol;Acc:HGNC:20154] |
| ENSG00000100665 | HGLibA_43519 | 34 | -0.827151831 | SERPINA4 | serpin family A member 4 [Source:HGNC Symbol;Acc:HGNC:8948] |
| ENSG00000100784 | HGLibA_42170 | 34 | -0.827151831 | RPS6KA5 | ribosomal protein S6 kinase A5 [Source:HGNC Symbol;Acc:HGNC:10434] |
| ENSG00000101096 | HGLibA_31776 | 34 | -0.827151831 | NFATC2 | nuclear factor of activated T cells 2 [Source:HGNC Symbol;Acc:HGNC:7776] |
| ENSG00000101109 | HGLibA_47510 | 34 | -0.827151831 | STK4 | serine/threonine kinase 4 [Source:HGNC Symbol;Acc:HGNC:11408] |
| ENSG00000101849 | HGLibA_48609 | 34 | -0.827151831 | TBL1X | transducin beta like 1 X-linked [Source:HGNC Symbol;Acc:HGNC:11585] |
| ENSG00000103275 | HGLibA_52615 | 34 | -0.827151831 | UBE2I | ubiquitin conjugating enzyme E2 I [Source:HGNC Symbol;Acc:HGNC:12485] |
| ENSG00000105605 | HGLibA_06946 | 34 | -0.827151831 | CACNG7 | calcium voltage-gated channel auxiliary subunit gamma 7 [Source:HGNC Symbol;Acc:HGNC:13626] |
| ENSG00000106772 | HGLibA_38990 | 34 | -0.827151831 | PRUNE2 | prune homolog 2 [Source:HGNC Symbol;Acc:HGNC:25209] |
| ENSG00000107864 | HGLibA_10922 | 34 | -0.827151831 | CPEB3 | cytoplasmic polyadenylation element binding protein 3 [Source:HGNC Symbol;Acc:HGNC:21746] |
| ENSG00000107984 | HGLibA_13293 | 34 | -0.827151831 | DKK1 | dickkopf WNT signaling pathway inhibitor 1 [Source:HGNC Symbol;Acc:HGNC:2891] |
| ENSG00000108107 | HGLibA_41926 | 34 | -0.827151831 | RPL28 | ribosomal protein L28 [Source:HGNC Symbol;Acc:HGNC:10330] |
| ENSG00000108175 | HGLibA_55382 | 34 | -0.827151831 | ZMIZ1 | zinc finger MIZ-type containing 1 [Source:HGNC Symbol;Acc:HGNC:16493] |
| ENSG00000108179 | HGLibA_37931 | 34 | -0.827151831 | PPIF | peptidylprolyl isomerase F [Source:HGNC Symbol;Acc:HGNC:9259] |
| ENSG00000110080 | HGLibA_47246 | 34 | -0.827151831 | ST3GAL4 | 3-sialyltransferase 4 [Source:HGNC Symbol;Acc:HGNC:10864]" |
| ENSG00000110497 | HGLibA_01754 | 34 | -0.827151831 | AMBRA1 | autophagy and beclin 1 regulator 1 [Source:HGNC Symbol;Acc:HGNC:25990] |
| ENSG00000111269 | HGLibA_11111 | 34 | -0.827151831 | CREBL2 | cAMP responsive element binding protein like 2 [Source:HGNC Symbol;Acc:HGNC:2350] |
| ENSG00000111737 | HGLibA_39890 | 34 | -0.827151831 | RAB35 | member RAS oncogene family [Source:HGNC Symbol;Acc:HGNC:9774]" |
| ENSG00000113013 | HGLibA_22299 | 34 | -0.827151831 | HSPA9 | heat shock protein family A (Hsp70) member 9 [Source:HGNC Symbol;Acc:HGNC:5244] |
| ENSG00000113555 | HGLibA_35398 | 34 | -0.827151831 | PCDH12 | protocadherin 12 [Source:HGNC Symbol;Acc:HGNC:8657] |
| ENSG00000114770 | HGLibA_00179 | 34 | -0.827151831 | ABCC5 | ATP binding cassette subfamily C member 5 [Source:HGNC Symbol;Acc:HGNC:56] |
| ENSG00000115271 | HGLibA_18759 | 34 | -0.827151831 | GCA | grancalcin [Source:HGNC Symbol;Acc:HGNC:15990] |
| ENSG00000116731 | HGLibA_38387 | 34 | -0.827151831 | PRDM2 | PR/SET domain 2 [Source:HGNC Symbol;Acc:HGNC:9347] |

| | | | | | |
|---|---|---|---|---|---|
| ENSG00000116857 | HGLibA_50555 | 34 | -0.827151831 | TMEM9 | transmembrane protein 9 [Source:HGNC Symbol;Acc:HGNC:18823] |
| ENSG00000117360 | HGLibA_38716 | 34 | -0.827151831 | PRPF3 | pre-mRNA processing factor 3 [Source:HGNC Symbol;Acc:HGNC:17348] |
| ENSG00000119397 | HGLibA_10437 | 34 | -0.827151831 | CNTRL | centriolin [Source:HGNC Symbol;Acc:HGNC:1858] |
| ENSG00000119596 | HGLibA_54697 | 34 | -0.827151831 | YLPM1 | YLP motif containing 1 [Source:HGNC Symbol;Acc:HGNC:17798] |
| ENSG00000119812 | HGLibA_16809 | 34 | -0.827151831 | FAM98A | family with sequence similarity 98 member A [Source:HGNC Symbol;Acc:HGNC:24520] |
| ENSG00000122386 | HGLibA_55575 | 34 | -0.827151831 | ZNF205 | zinc finger protein 205 [Source:HGNC Symbol;Acc:HGNC:12996] |
| ENSG00000122390 | HGLibA_31002 | 34 | -0.827151831 | NAA60 | NatF catalytic subunit [Source:HGNC Symbol;Acc:HGNC:25875]" |
| ENSG00000124092 | HGLibA_11600 | 34 | -0.827151831 | CTCFL | CCCTC-binding factor like [Source:HGNC Symbol;Acc:HGNC:16234] |
| ENSG00000124440 | HGLibA_21302 | 34 | -0.827151831 | HIF3A | hypoxia inducible factor 3 subunit alpha [Source:HGNC Symbol;Acc:HGNC:15825] |
| ENSG00000124479 | HGLibA_31411 | 34 | -0.827151831 | NDP | norrin cystine knot growth factor [Source:HGNC Symbol;Acc:HGNC:7678]" |
| ENSG00000125458 | HGLibA_32798 | 34 | -0.827151831 | NT5C | cytosolic [Source:HGNC Symbol;Acc:HGNC:17144]" |
| ENSG00000126432 | HGLibA_38419 | 34 | -0.827151831 | PRDX5 | peroxiredoxin 5 [Source:HGNC Symbol;Acc:HGNC:9355] |
| ENSG00000126768 | HGLibA_49505 | 34 | -0.827151831 | TIMM17B | translocase of inner mitochondrial membrane 17B [Source:HGNC Symbol;Acc:HGNC:17310] |
| ENSG00000129351 | HGLibA_23217 | 34 | -0.827151831 | ILF3 | interleukin enhancer binding factor 3 [Source:HGNC Symbol;Acc:HGNC:6038] |
| ENSG00000130005 | HGLibA_18598 | 34 | -0.827151831 | GAMT | guanidinoacetate N-methyltransferase [Source:HGNC Symbol;Acc:HGNC:4136] |
| ENSG00000130508 | HGLibA_39650 | 34 | -0.827151831 | PXDN | peroxidasin [Source:HGNC Symbol;Acc:HGNC:14966] |
| ENSG00000130538 | HGLibA_33570 | 34 | -0.827151831 | OR11H1 | olfactory receptor family 11 subfamily H member 1 [Source:HGNC Symbol;Acc:HGNC:15404] |
| ENSG00000130699 | HGLibA_48233 | 34 | -0.827151831 | TAF4 | TATA-box binding protein associated factor 4 [Source:HGNC Symbol;Acc:HGNC:11537] |
| ENSG00000131634 | HGLibA_50188 | 34 | -0.827151831 | TMEM204 | transmembrane protein 204 [Source:HGNC Symbol;Acc:HGNC:14158] |
| ENSG00000131746 | HGLibA_50928 | 34 | -0.827151831 | TNS4 | tensin 4 [Source:HGNC Symbol;Acc:HGNC:24352] |
| ENSG00000132153 | HGLibA_13175 | 34 | -0.827151831 | DHX30 | DExH-box helicase 30 [Source:HGNC Symbol;Acc:HGNC:16716] |
| ENSG00000132196 | HGLibA_22200 | 34 | -0.827151831 | HSD17B7 | hydroxysteroid 17-beta dehydrogenase 7 [Source:HGNC Symbol;Acc:HGNC:5215] |
| ENSG00000132436 | HGLibA_17521 | 34 | -0.827151831 | FIGNL1 | fidgetin like 1 [Source:HGNC Symbol;Acc:HGNC:13286] |
| ENSG00000132773 | HGLibA_50941 | 34 | -0.827151831 | TOE1 | exonuclease [Source:HGNC Symbol;Acc:HGNC:15954]" |
| ENSG00000134056 | HGLibA_30101 | 34 | -0.827151831 | MRPS36 | mitochondrial ribosomal protein S36 [Source:HGNC Symbol;Acc:HGNC:16631] |
| ENSG00000134202 | HGLibA_20477 | 34 | -0.827151831 | GSTM3 | glutathione S-transferase mu 3 [Source:HGNC Symbol;Acc:HGNC:4635] |
| ENSG00000134258 | HGLibA_53884 | 34 | -0.827151831 | VTCN1 | V-set domain containing T cell activation inhibitor 1 [Source:HGNC Symbol;Acc:HGNC:28873] |
| ENSG00000135047 | HGLibA_11715 | 34 | -0.827151831 | CTSL | cathepsin L [Source:HGNC Symbol;Acc:HGNC:2537] |
| ENSG00000135341 | HGLibA_28195 | 34 | -0.827151831 | MAP3K7 | mitogen-activated protein kinase kinase kinase 7 [Source:HGNC Symbol;Acc:HGNC:6859] |
| ENSG00000135423 | HGLibA_19327 | 34 | -0.827151831 | GLS2 | glutaminase 2 [Source:HGNC Symbol;Acc:HGNC:29570] |
| ENSG00000136535 | HGLibA_48633 | 34 | -0.827151831 | TBR1 | brain 1 [Source:HGNC Symbol;Acc:HGNC:11590]" |
| ENSG00000137561 | HGLibA_52223 | 34 | -0.827151831 | TTPA | alpha tocopherol transfer protein [Source:HGNC Symbol;Acc:HGNC:12404] |
| ENSG00000137573 | HGLibA_47726 | 34 | -0.827151831 | SULF1 | sulfatase 1 [Source:HGNC Symbol;Acc:HGNC:20391] |
| ENSG00000138593 | HGLibA_43269 | 34 | -0.827151831 | SECISBP2L | SECIS binding protein 2 like [Source:HGNC Symbol;Acc:HGNC:28997] |
| ENSG00000139132 | HGLibA_17351 | 34 | -0.827151831 | FGD4 | RhoGEF and PH domain containing 4 [Source:HGNC Symbol;Acc:HGNC:19125]" |
| ENSG00000139767 | HGLibA_47051 | 34 | -0.827151831 | SRRM4 | serine/arginine repetitive matrix 4 [Source:HGNC Symbol;Acc:HGNC:29389] |
| ENSG00000142207 | HGLibA_53134 | 34 | -0.827151831 | URB1 | URB1 ribosome biogenesis homolog [Source:HGNC Symbol;Acc:HGNC:17344] |
| ENSG00000142207 | HGLibA_53135 | 34 | -0.827151831 | URB1 | URB1 ribosome biogenesis homolog [Source:HGNC Symbol;Acc:HGNC:17344] |
| ENSG00000142512 | HGLibA_44125 | 34 | -0.827151831 | SIGLEC10 | sialic acid binding Ig like lectin 10 [Source:HGNC Symbol;Acc:HGNC:15620] |
| ENSG00000143061 | HGLibA_22893 | 34 | -0.827151831 | IGSF3 | immunoglobulin superfamily member 3 [Source:HGNC Symbol;Acc:HGNC:5950] |
| ENSG00000143318 | HGLibA_07335 | 34 | -0.827151831 | CASQ1 | calsequestrin 1 [Source:HGNC Symbol;Acc:HGNC:1512] |
| ENSG00000143507 | HGLibA_14130 | 34 | -0.827151831 | DUSP10 | dual specificity phosphatase 10 [Source:HGNC Symbol;Acc:HGNC:3065] |
| ENSG00000143816 | HGLibA_54393 | 34 | -0.827151831 | WNT9A | Wnt family member 9A [Source:HGNC Symbol;Acc:HGNC:12778] |
| ENSG00000143971 | HGLibA_15614 | 34 | -0.827151831 | ETAA1 | ATR kinase activator [Source:HGNC Symbol;Acc:HGNC:24648]" |
| ENSG00000144792 | HGLibA_56477 | 34 | -0.827151831 | ZNF660 | zinc finger protein 660 [Source:HGNC Symbol;Acc:HGNC:26720] |
| ENSG00000145216 | HGLibA_17535 | 34 | -0.827151831 | FIP1L1 | factor interacting with PAPOLA and CPSF1 [Source:HGNC Symbol;Acc:HGNC:19124] |
| ENSG00000145824 | HGLibA_11857 | 34 | -0.827151831 | CXCL14 | C-X-C motif chemokine ligand 14 [Source:HGNC Symbol;Acc:HGNC:10640] |
| ENSG00000146242 | HGLibA_51100 | 34 | -0.827151831 | TPBG | trophoblast glycoprotein [Source:HGNC Symbol;Acc:HGNC:12004] |
| ENSG00000146426 | HGLibA_49442 | 34 | -0.827151831 | TIAM2 | T cell lymphoma invasion and metastasis 2 [Source:HGNC Symbol;Acc:HGNC:11806] |
| ENSG00000146733 | HGLibA_39225 | 34 | -0.827151831 | PSPH | phosphoserine phosphatase [Source:HGNC Symbol;Acc:HGNC:9577] |
| ENSG00000147955 | HGLibA_44159 | 34 | -0.827151831 | SIGMAR1 | sigma non-opioid intracellular receptor 1 [Source:HGNC Symbol;Acc:HGNC:8157] |
| ENSG00000148386 | HGLibA_26188 | 34 | -0.827151831 | LCN9 | lipocalin 9 [Source:HGNC Symbol;Acc:HGNC:17442] |
| ENSG00000148672 | HGLibA_19362 | 34 | -0.827151831 | GLUD1 | glutamate dehydrogenase 1 [Source:HGNC Symbol;Acc:HGNC:4335] |
| ENSG00000148798 | HGLibA_23268 | 34 | -0.827151831 | INA | internexin neuronal intermediate filament protein alpha [Source:HGNC Symbol;Acc:HGNC:6057] |
| ENSG00000151276 | HGLibA_27994 | 34 | -0.827151831 | MAGI1 | WW and PDZ domain containing 1 [Source:HGNC Symbol;Acc:HGNC:946]" |
| ENSG00000152404 | HGLibA_11828 | 34 | -0.827151831 | CWF19L2 | CWF19 like cell cycle control factor 2 [Source:HGNC Symbol;Acc:HGNC:26508] |
| ENSG00000153093 | HGLibA_00506 | 34 | -0.827151831 | ACOXL | acyl-CoA oxidase like [Source:HGNC Symbol;Acc:HGNC:25621] |
| ENSG00000154165 | HGLibA_19926 | 34 | -0.827151831 | GPR15 | G protein-coupled receptor 15 [Source:HGNC Symbol;Acc:HGNC:4469] |
| ENSG00000154310 | HGLibA_50832 | 34 | -0.827151831 | TNIK | TRAF2 and NCK interacting kinase [Source:HGNC Symbol;Acc:HGNC:30765] |
| ENSG00000154553 | HGLibA_35913 | 34 | -0.827151831 | PDLIM3 | PDZ and LIM domain 3 [Source:HGNC Symbol;Acc:HGNC:20767] |
| ENSG00000155265 | HGLibA_19637 | 34 | -0.827151831 | GOLGA7B | golgin A7 family member B [Source:HGNC Symbol;Acc:HGNC:31668] |
| ENSG00000155508 | HGLibA_10352 | 34 | -0.827151831 | CNOT8 | CCR4-NOT transcription complex subunit 8 [Source:HGNC Symbol;Acc:HGNC:9207] |
| ENSG00000156413 | HGLibA_18199 | 34 | -0.827151831 | FUT6 | fucosyltransferase 6 [Source:HGNC Symbol;Acc:HGNC:4017] |
| ENSG00000156650 | HGLibA_24064 | 34 | -0.827151831 | KAT6B | lysine acetyltransferase 6B [Source:HGNC Symbol;Acc:HGNC:17582] |
| ENSG00000156959 | HGLibA_26438 | 34 | -0.827151831 | LHFPL4 | LHFPL tetraspan subfamily member 4 [Source:HGNC Symbol;Acc:HGNC:29568] |
| ENSG00000157884 | HGLibA_09690 | 34 | -0.827151831 | CIB4 | calcium and integrin binding family member 4 [Source:HGNC Symbol;Acc:HGNC:33703] |
| ENSG00000158290 | HGLibA_11785 | 34 | -0.827151831 | CUL4B | cullin 4B [Source:HGNC Symbol;Acc:HGNC:2555] |
| ENSG00000158828 | HGLibA_36691 | 34 | -0.827151831 | PINK1 | PTEN induced kinase 1 [Source:HGNC Symbol;Acc:HGNC:14581] |
| ENSG00000159495 | HGLibA_49271 | 34 | -0.827151831 | TGM7 | transglutaminase 7 [Source:HGNC Symbol;Acc:HGNC:30790] |
| ENSG00000160799 | HGLibA_07561 | 34 | -0.827151831 | CCDC12 | coiled-coil domain containing 12 [Source:HGNC Symbol;Acc:HGNC:28332] |
| ENSG00000162367 | HGLibA_48279 | 34 | -0.827151831 | TAL1 | erythroid differentiation factor [Source:HGNC Symbol;Acc:HGNC:11556]" |
| ENSG00000162836 | HGLibA_00517 | 34 | -0.827151831 | ACP6 | lysophosphatidic [Source:HGNC Symbol;Acc:HGNC:29609]" |
| ENSG00000163827 | HGLibA_27277 | 34 | -0.827151831 | LRRC2 | leucine rich repeat containing 2 [Source:HGNC Symbol;Acc:HGNC:14676] |
| ENSG00000163900 | HGLibA_50394 | 34 | -0.827151831 | TMEM41A | transmembrane protein 41A [Source:HGNC Symbol;Acc:HGNC:30544] |
| ENSG00000164209 | HGLibA_44753 | 34 | -0.827151831 | SLC25A46 | solute carrier family 25 member 46 [Source:HGNC Symbol;Acc:HGNC:25198] |
| ENSG00000164251 | HGLibA_15865 | 34 | -0.827151831 | F2RL1 | F2R like trypsin receptor 1 [Source:HGNC Symbol;Acc:HGNC:3538] |
| ENSG00000164332 | HGLibA_52717 | 34 | -0.827151831 | UBLCP1 | ubiquitin like domain containing CTD phosphatase 1 [Source:HGNC Symbol;Acc:HGNC:28110] |
| ENSG00000164363 | HGLibA_45313 | 34 | -0.827151831 | SLC6A18 | solute carrier family 6 member 18 [Source:HGNC Symbol;Acc:HGNC:26441] |
| ENSG00000164494 | HGLibA_35953 | 34 | -0.827151831 | PDSS2 | decaprenyl diphosphate synthase subunit 2 [Source:HGNC Symbol;Acc:HGNC:23041] |
| ENSG00000164675 | HGLibA_23551 | 34 | -0.827151831 | IQUB | IQ motif and ubiquitin domain containing [Source:HGNC Symbol;Acc:HGNC:21995] |
| ENSG00000166260 | HGLibA_10806 | 34 | -0.827151831 | COX11 | cytochrome c oxidase copper chaperone COX11 [Source:HGNC Symbol;Acc:HGNC:2261] |
| ENSG00000166510 | HGLibA_07842 | 34 | -0.827151831 | CCDC68 | coiled-coil domain containing 68 [Source:HGNC Symbol;Acc:HGNC:24350] |
| ENSG00000167333 | HGLibA_51591 | 34 | -0.827151831 | TRIM68 | tripartite motif containing 68 [Source:HGNC Symbol;Acc:HGNC:21161] |
| ENSG00000167656 | HGLibA_27652 | 34 | -0.827151831 | LY6D | lymphocyte antigen 6 family member D [Source:HGNC Symbol;Acc:HGNC:13348] |
| ENSG00000168959 | HGLibA_20352 | 34 | -0.827151831 | GRM5 | glutamate metabotropic receptor 5 [Source:HGNC Symbol;Acc:HGNC:4597] |
| ENSG00000169836 | HGLibA_48176 | 34 | -0.827151831 | TACR3 | tachykinin receptor 3 [Source:HGNC Symbol;Acc:HGNC:11528] |
| ENSG00000170242 | HGLibA_53381 | 34 | -0.827151831 | USP47 | ubiquitin specific peptidase 47 [Source:HGNC Symbol;Acc:HGNC:20076] |
| ENSG00000171016 | HGLibA_39695 | 34 | -0.827151831 | PYGO1 | pygopus family PHD finger 1 [Source:HGNC Symbol;Acc:HGNC:30256] |
| ENSG00000171109 | HGLibA_29068 | 34 | -0.827151831 | MFN1 | mitofusin 1 [Source:HGNC Symbol;Acc:HGNC:18262] |
| ENSG00000171124 | HGLibA_18191 | 34 | -0.827151831 | FUT3 | fucosyltransferase 3 (Lewis blood group) [Source:HGNC Symbol;Acc:HGNC:4014] |
| ENSG00000171316 | HGLibA_09405 | 34 | -0.827151831 | CHD7 | chromodomain helicase DNA binding protein 7 [Source:HGNC Symbol;Acc:HGNC:20626] |
| ENSG00000171532 | HGLibA_31738 | 34 | -0.827151831 | NEUROD2 | neuronal differentiation 2 [Source:HGNC Symbol;Acc:HGNC:7763] |
| ENSG00000171772 | HGLibA_47914 | 34 | -0.827151831 | SYCE1 | synaptonemal complex central element protein 1 [Source:HGNC Symbol;Acc:HGNC:28852] |
| ENSG00000172167 | HGLibA_30321 | 34 | -0.827151831 | MTBP | MDM2 binding protein [Source:HGNC Symbol;Acc:HGNC:7417] |
| ENSG00000172320 | HGLibA_34229 | 34 | -0.827151831 | OR5A1 | olfactory receptor family 5 subfamily A member 1 [Source:HGNC Symbol;Acc:HGNC:8319] |
| ENSG00000172817 | HGLibA_12214 | 34 | -0.827151831 | CYP7B1 | cytochrome P450 family 7 subfamily B member 1 [Source:HGNC Symbol;Acc:HGNC:2652] |
| ENSG00000173678 | HGLibA_46584 | 34 | -0.827151831 | SPDYE2B | speedy/RINGO cell cycle regulator family member E2B [Source:HGNC Symbol;Acc:HGNC:48334] |
| ENSG00000173868 | HGLibA_36441 | 34 | -0.827151831 | PHOSPHO1 | phosphoethanolamine/phosphocholine phosphatase [Source:HGNC Symbol;Acc:HGNC:16815] |
| ENSG00000174227 | HGLibA_36555 | 34 | -0.827151831 | PIGG | phosphatidylinositol glycan anchor biosynthesis class G [Source:HGNC Symbol;Acc:HGNC:25985] |
| ENSG00000174876 | HGLibA_01845 | 34 | -0.827151831 | AMY1B | alpha 1B (salivary) [Source:HGNC Symbol;Acc:HGNC:475]" |
| ENSG00000175065 | HGLibA_14032 | 34 | -0.827151831 | DSG4 | desmoglein 4 [Source:HGNC Symbol;Acc:HGNC:21307] |
| ENSG00000175121 | HGLibA_54269 | 34 | -0.827151831 | WFDC5 | WAP four-disulfide core domain 5 [Source:HGNC Symbol;Acc:HGNC:20477] |
| ENSG00000175352 | HGLibA_32696 | 34 | -0.827151831 | NRIP3 | nuclear receptor interacting protein 3 [Source:HGNC Symbol;Acc:HGNC:1167] |
| ENSG00000175931 | HGLibA_52642 | 34 | -0.827151831 | UBE2O | ubiquitin conjugating enzyme E2 O [Source:HGNC Symbol;Acc:HGNC:29554] |
| ENSG00000176022 | HGLibA_03967 | 34 | -0.827151831 | B3GALT6 | 3-galactosyltransferase 6 [Source:HGNC Symbol;Acc:HGNC:17978]" |
| ENSG00000176390 | HGLibA_11194 | 34 | -0.827151831 | CRLF3 | cytokine receptor like factor 3 [Source:HGNC Symbol;Acc:HGNC:17177] |
| ENSG00000176635 | HGLibA_21889 | 34 | -0.827151831 | HORMAD2 | HORMA domain containing 2 [Source:HGNC Symbol;Acc:HGNC:28383] |
| ENSG00000177023 | HGLibA_12822 | 34 | -0.827151831 | DEFB104B | defensin beta 104B [Source:HGNC Symbol;Acc:HGNC:26165] |
| ENSG00000177463 | HGLibA_32599 | 34 | -0.827151831 | NR2C2 | nuclear receptor subfamily 2 group C member 2 [Source:HGNC Symbol;Acc:HGNC:7972] |
| ENSG00000177627 | HGLibA_05264 | 34 | -0.827151831 | C12orf54 | chromosome 12 open reading frame 54 [Source:HGNC Symbol;Acc:HGNC:28553] |
| ENSG00000178403 | HGLibA_31748 | 34 | -0.827151831 | NEUROG2 | neurogenin 2 [Source:HGNC Symbol;Acc:HGNC:13805] |
| ENSG00000178602 | HGLibA_34746 | 34 | -0.827151831 | OTOS | otospiralin [Source:HGNC Symbol;Acc:HGNC:22644] |
| ENSG00000178922 | HGLibA_22452 | 34 | -0.827151831 | HYI | hydroxypyruvate isomerase (putative) [Source:HGNC Symbol;Acc:HGNC:26948] |
| ENSG00000179256 | HGLibA_45668 | 34 | -0.827151831 | SMCO3 | single-pass membrane protein with coiled-coil domains 3 [Source:HGNC Symbol;Acc:HGNC:34401] |
| ENSG00000179284 | HGLibA_12308 | 34 | -0.827151831 | DAND5 | DAN domain BMP antagonist family member 5 [Source:HGNC Symbol;Acc:HGNC:26780] |
| ENSG00000179941 | HGLibA_04209 | 34 | -0.827151831 | BBS10 | Bardet-Biedl syndrome 10 [Source:HGNC Symbol;Acc:HGNC:26291] |
| ENSG00000180346 | HGLibA_49465 | 34 | -0.827151831 | TIGD2 | tigger transposable element derived 2 [Source:HGNC Symbol;Acc:HGNC:18333] |
| ENSG00000180479 | HGLibA_56279 | 34 | -0.827151831 | ZNF571 | zinc finger protein 571 [Source:HGNC Symbol;Acc:HGNC:25000] |
| ENSG00000181007 | HGLibA_55238 | 34 | -0.827151831 | ZFP82 | ZFP82 zinc finger protein [Source:HGNC Symbol;Acc:HGNC:28682] |
| ENSG00000182504 | HGLibA_09183 | 34 | -0.827151831 | CEP97 | centrosomal protein 97 [Source:HGNC Symbol;Acc:HGNC:26244] |
| ENSG00000183092 | HGLibA_04399 | 34 | -0.827151831 | BEGAIN | brain enriched guanylate kinase associated [Source:HGNC Symbol;Acc:HGNC:24163] |
| ENSG00000183463 | HGLibA_53131 | 34 | -0.827151831 | URAD | ureidoimidazoline (2-oxo-4-hydroxy-4-carboxy-5-) decarboxylase [Source:HGNC Symbol;Acc:HGNC:17785] |
| ENSG00000184992 | HGLibA_04805 | 34 | -0.827151831 | BRI3BP | BRI3 binding protein [Source:HGNC Symbol;Acc:HGNC:14251] |
| ENSG00000185823 | HGLibA_32391 | 34 | -0.827151831 | NPAP1 | nuclear pore associated protein 1 [Source:HGNC Symbol;Acc:HGNC:1190] |
| ENSG00000186092 | HGLibA_34010 | 34 | -0.827151831 | OR4F5 | olfactory receptor family 4 subfamily F member 5 [Source:HGNC Symbol;Acc:HGNC:14825] |
| ENSG00000187094 | HGLibA_07951 | 34 | -0.827151831 | CCK | cholecystokinin [Source:HGNC Symbol;Acc:HGNC:1569] |
| ENSG00000187918 | HGLibA_34117 | 34 | -0.827151831 | OR51I2 | olfactory receptor family 51 subfamily I member 2 [Source:HGNC Symbol;Acc:HGNC:15201] |
| ENSG00000188487 | HGLibA_23373 | 34 | -0.827151831 | INSC | spindle orientation adaptor protein [Source:HGNC Symbol;Acc:HGNC:33116]" |
| ENSG00000188493 | HGLibA_05681 | 34 | -0.827151831 | C19orf54 | chromosome 19 open reading frame 54 [Source:HGNC Symbol;Acc:HGNC:24758] |
| ENSG00000196415 | HGLibA_38984 | 34 | -0.827151831 | PRTN3 | proteinase 3 [Source:HGNC Symbol;Acc:HGNC:9495] |
| ENSG00000196428 | HGLibA_51820 | 34 | -0.827151831 | TSC22D2 | TSC22 domain family member 2 [Source:HGNC Symbol;Acc:HGNC:29095] |
| ENSG00000196935 | HGLibA_46967 | 34 | -0.827151831 | SRGAP1 | SLIT-ROBO Rho GTPase activating protein 1 [Source:HGNC Symbol;Acc:HGNC:17382] |
| ENSG00000196944 | HGLibA_33885 | 34 | -0.827151831 | OR2T4 | olfactory receptor family 2 subfamily T member 4 [Source:HGNC Symbol;Acc:HGNC:15016] |
| ENSG00000197217 | HGLibA_15206 | 34 | -0.827151831 | ENTPD4 | ectonucleoside triphosphate diphosphohydrolase 4 [Source:HGNC Symbol;Acc:HGNC:14573] |
| ENSG00000197561 | HGLibA_14877 | 34 | -0.827151831 | ELANE | neutrophil expressed [Source:HGNC Symbol;Acc:HGNC:3309]" |

| | | | | | |
|---|---|---|---|---|---|
| ENSG00000197653 | HGLibA_13472 | 34 | -0.827151831 | DNAH10 | dynein axonemal heavy chain 10 [Source:HGNC Symbol;Acc:HGNC:2941] |
| ENSG00000197965 | HGLibA_29794 | 34 | -0.827151831 | MPZL1 | myelin protein zero like 1 [Source:HGNC Symbol;Acc:HGNC:7226] |
| ENSG00000198010 | HGLibA_13343 | 34 | -0.827151831 | DLGAP2 | DLG associated protein 2 [Source:HGNC Symbol;Acc:HGNC:2906] |
| ENSG00000198093 | HGLibA_56459 | 34 | -0.827151831 | ZNF649 | zinc finger protein 649 [Source:HGNC Symbol;Acc:HGNC:25741] |
| ENSG00000198218 | HGLibA_39742 | 34 | -0.827151831 | QRICH1 | glutamine rich 1 [Source:HGNC Symbol;Acc:HGNC:24713] |
| ENSG00000198796 | HGLibA_01703 | 34 | -0.827151831 | ALPK2 | alpha kinase 2 [Source:HGNC Symbol;Acc:HGNC:20565] |
| ENSG00000198952 | HGLibA_45701 | 34 | -0.827151831 | SMG5 | nonsense mediated mRNA decay factor [Source:HGNC Symbol;Acc:HGNC:24644]" |
| ENSG00000204022 | HGLibA_26622 | 34 | -0.827151831 | LIPJ | lipase family member J [Source:HGNC Symbol;Acc:HGNC:21773] |
| ENSG00000204070 | HGLibA_48041 | 34 | -0.827151831 | SYS1 | golgi trafficking protein [Source:HGNC Symbol;Acc:HGNC:16162]" |
| ENSG00000205678 | HGLibA_48951 | 34 | -0.827151831 | TECRL | 3-enoyl-CoA reductase like [Source:HGNC Symbol;Acc:HGNC:27365]" |
| ENSG00000205869 | HGLibA_25783 | 34 | -0.827151831 | KRTAP5-1 | keratin associated protein 5-1 [Source:HGNC Symbol;Acc:HGNC:23596] |
| ENSG00000206052 | HGLibA_13783 | 34 | -0.827151831 | DOK6 | docking protein 6 [Source:HGNC Symbol;Acc:HGNC:28301] |
| ENSG00000213639 | HGLibA_38007 | 34 | -0.827151831 | PPP1CB | protein phosphatase 1 catalytic subunit beta [Source:HGNC Symbol;Acc:HGNC:9282] |
| ENSG00000214285 | HGLibA_32524 | 34 | -0.827151831 | NPS | neuropeptide S [Source:HGNC Symbol;Acc:HGNC:33940] |
| ENSG00000215126 | HGLibA_07443 | 34 | -0.827151831 | CBWD6 | COBW domain containing 6 [Source:HGNC Symbol;Acc:HGNC:31978] |
| ENSG00000227551 | HGLibA_53221 | 34 | -0.827151831 | USP17L12 | ubiquitin specific peptidase 17-like family member 12 [Source:HGNC Symbol;Acc:HGNC:44440] |
| ENSG00000237412 | HGLibA_38966 | 34 | -0.827151831 | PRSS56 | serine protease 56 [Source:HGNC Symbol;Acc:HGNC:39433] |
| ENSG00000239810 | HGLibA_38283 | 34 | -0.827151831 | PRAMEF11 | PRAME family member 11 [Source:HGNC Symbol;Acc:HGNC:14086] |
| ENSG00000243725 | HGLibA_52140 | 34 | -0.827151831 | TTC4 | tetratricopeptide repeat domain 4 [Source:HGNC Symbol;Acc:HGNC:12394] |
| ENSG00000249915 | HGLibA_35739 | 34 | -0.827151831 | PDCD6 | programmed cell death 6 [Source:HGNC Symbol;Acc:HGNC:8765] |
| ENSG00000255245 | HGLibA_18243 | 34 | -0.827151831 | FXYD6-FXYD2 | FXYD6-FXYD2 readthrough [Source:HGNC Symbol;Acc:HGNC:39978] |
| ENSG00000255302 | HGLibA_14692 | 34 | -0.827151831 | EID1 | EP300 interacting inhibitor of differentiation 1 [Source:HGNC Symbol;Acc:HGNC:1191] |
| ENSG00000265190 | HGLibA_02252 | 34 | -0.827151831 | ANXA8 | annexin A8 [Source:HGNC Symbol;Acc:HGNC:546] |
| ENSG00000265763 | HGLibA_56085 | 34 | -0.827151831 | ZNF488 | zinc finger protein 488 [Source:HGNC Symbol;Acc:HGNC:23535] |
| ENSG00000268009 | HGLibA_47211 | 34 | -0.827151831 | SSX4 | SSX family member 4 [Source:HGNC Symbol;Acc:HGNC:11338] |
| ENSG00000268447 | HGLibA_47200 | 34 | -0.827151831 | SSX2B | SSX family member 2B [Source:HGNC Symbol;Acc:HGNC:22263] |
| ENSG00000278289 | HGLibA_11513 | 34 | -0.827151831 | CT45A6 | cancer/testis antigen family 45 member A6 [Source:HGNC Symbol;Acc:HGNC:33271] |
| ENSG00000284733 | HGLibA_34002 | 34 | -0.827151831 | OR4F29 | olfactory receptor family 4 subfamily F member 29 [Source:HGNC Symbol;Acc:HGNC:31275] |
| FAM26F | HGLibA_16567 | 34 | -0.827151831 | FAM26F | - |
| GLTSCR2 | HGLibA_19358 | 34 | -0.827151831 | GLTSCR2 | - |
| HIST1H2AK | HGLibA_21403 | 34 | -0.827151831 | HIST1H2AK | - |
| hsa-mir-103a-1 | HGLibA_57163 | 34 | -0.827151831 | hsa-mir-103a-1 | - |
| hsa-mir-1180 | HGLibA_57212 | 34 | -0.827151831 | hsa-mir-1180 | - |
| hsa-mir-1291 | HGLibA_57639 | 34 | -0.827151831 | hsa-mir-1291 | - |
| hsa-mir-181b-2 | HGLibA_57972 | 34 | -0.827151831 | hsa-mir-181b-2 | - |
| hsa-mir-301b | HGLibA_58431 | 34 | -0.827151831 | hsa-mir-301b | - |
| hsa-mir-331 | HGLibA_58992 | 34 | -0.827151831 | hsa-mir-331 | - |
| hsa-mir-3661 | HGLibA_59195 | 34 | -0.827151831 | hsa-mir-3661 | - |
| hsa-mir-3679 | HGLibA_59265 | 34 | -0.827151831 | hsa-mir-3679 | - |
| hsa-mir-376c | HGLibA_59418 | 34 | -0.827151831 | hsa-mir-376c | - |
| hsa-mir-384 | HGLibA_59489 | 34 | -0.827151831 | hsa-mir-384 | - |
| hsa-mir-3926-2 | HGLibA_59581 | 34 | -0.827151831 | hsa-mir-3926-2 | - |
| hsa-mir-3974 | HGLibA_59657 | 34 | -0.827151831 | hsa-mir-3974 | - |
| hsa-mir-4311 | HGLibA_59961 | 34 | -0.827151831 | hsa-mir-4311 | - |
| hsa-mir-4328 | HGLibA_60035 | 34 | -0.827151831 | hsa-mir-4328 | - |
| hsa-mir-4461 | HGLibA_60249 | 34 | -0.827151831 | hsa-mir-4461 | - |
| hsa-mir-4478 | HGLibA_60327 | 34 | -0.827151831 | hsa-mir-4478 | - |
| hsa-mir-454 | HGLibA_60622 | 34 | -0.827151831 | hsa-mir-454 | - |
| hsa-mir-4645 | HGLibA_60684 | 34 | -0.827151831 | hsa-mir-4645 | - |
| hsa-mir-4661 | HGLibA_60752 | 34 | -0.827151831 | hsa-mir-4661 | - |
| hsa-mir-4770 | HGLibA_61175 | 34 | -0.827151831 | hsa-mir-4770 | - |
| hsa-mir-484 | HGLibA_61330 | 34 | -0.827151831 | hsa-mir-484 | - |
| hsa-mir-501 | HGLibA_61452 | 34 | -0.827151831 | hsa-mir-501 | - |
| hsa-mir-548aa-1 | HGLibA_61850 | 34 | -0.827151831 | hsa-mir-548aa-1 | - |
| hsa-mir-548f-5 | HGLibA_61988 | 34 | -0.827151831 | hsa-mir-548f-5 | - |
| hsa-mir-548z | HGLibA_62092 | 34 | -0.827151831 | hsa-mir-548z | - |
| hsa-mir-570 | HGLibA_62334 | 34 | -0.827151831 | hsa-mir-570 | - |
| hsa-mir-601 | HGLibA_62495 | 34 | -0.827151831 | hsa-mir-601 | - |
| hsa-mir-6504 | HGLibA_62865 | 34 | -0.827151831 | hsa-mir-6504 | - |
| hsa-mir-664b | HGLibA_62996 | 34 | -0.827151831 | hsa-mir-664b | - |
| hsa-mir-6754 | HGLibA_63175 | 34 | -0.827151831 | hsa-mir-6754 | - |
| hsa-mir-6761 | HGLibA_63207 | 34 | -0.827151831 | hsa-mir-6761 | - |
| hsa-mir-6809 | HGLibA_63415 | 34 | -0.827151831 | hsa-mir-6809 | - |
| hsa-mir-6820 | HGLibA_63458 | 34 | -0.827151831 | hsa-mir-6820 | - |
| hsa-mir-6890 | HGLibA_63745 | 34 | -0.827151831 | hsa-mir-6890 | - |
| hsa-mir-874 | HGLibA_64205 | 34 | -0.827151831 | hsa-mir-874 | - |
| LEPREL1 | HGLibA_26319 | 34 | -0.827151831 | LEPREL1 | - |
| LOC154872 | HGLibA_26897 | 34 | -0.827151831 | LOC154872 | - |
| LOC388849 | HGLibA_26928 | 34 | -0.827151831 | LOC388849 | - |
| OR8U8 | HGLibA_34558 | 34 | -0.827151831 | OR8U8 | - |
| P2RY8_X | HGLibA_34895 | 34 | -0.827151831 | P2RY8_X | - |
| TNFAIP8L2-SCNM1 | HGLibA_50727 | 34 | -0.827151831 | TNFAIP8L2-SCNM1 | - |
| AES | HGLibA_01095 | 33 | -0.836197174 | AES | - |
| ATPBD4 | HGLibA_03804 | 33 | -0.836197174 | ATPBD4 | - |
| BAGE4 | HGLibA_04101 | 33 | -0.836197174 | BAGE4 | - |
| C1orf95 | HGLibA_05908 | 33 | -0.836197174 | C1orf95 | - |
| C21orf59 | HGLibA_06043 | 33 | -0.836197174 | C21orf59 | - |
| C6orf70 | HGLibA_06510 | 33 | -0.836197174 | C6orf70 | - |
| C7orf55-LUC7L2 | HGLibA_06556 | 33 | -0.836197174 | C7orf55-LUC7L2 | - |
| CCDC176 | HGLibA_07710 | 33 | -0.836197174 | CCDC176 | - |
| CRLF2_Y | HGLibA_11191 | 33 | -0.836197174 | CRLF2_Y | - |
| DOPEY2 | HGLibA_13804 | 33 | -0.836197174 | DOPEY2 | - |
| ENSG00000003989 | HGLibA_45360 | 33 | -0.836197174 | SLC7A2 | solute carrier family 7 member 2 [Source:HGNC Symbol;Acc:HGNC:11060] |
| ENSG00000006638 | HGLibA_48681 | 33 | -0.836197174 | TBXA2R | thromboxane A2 receptor [Source:HGNC Symbol;Acc:HGNC:11608] |
| ENSG00000007080 | HGLibA_07573 | 33 | -0.836197174 | CCDC124 | coiled-coil domain containing 124 [Source:HGNC Symbol;Acc:HGNC:25171] |
| ENSG00000016082 | HGLibA_23663 | 33 | -0.836197174 | ISL1 | ISL LIM homeobox 1 [Source:HGNC Symbol;Acc:HGNC:6132] |
| ENSG00000047365 | HGLibA_02629 | 33 | -0.836197174 | ARAP2 | ankyrin repeat and PH domain 2 [Source:HGNC Symbol;Acc:HGNC:16924]" |
| ENSG00000050165 | HGLibA_13301 | 33 | -0.836197174 | DKK3 | dickkopf WNT signaling pathway inhibitor 3 [Source:HGNC Symbol;Acc:HGNC:2893] |
| ENSG00000063854 | HGLibA_20814 | 33 | -0.836197174 | HAGH | hydroxyacylglutathione hydrolase [Source:HGNC Symbol;Acc:HGNC:4805] |
| ENSG00000064201 | HGLibA_51931 | 33 | -0.836197174 | TSPAN32 | tetraspanin 32 [Source:HGNC Symbol;Acc:HGNC:13410] |
| ENSG00000065518 | HGLibA_31519 | 33 | -0.836197174 | NDUFB4 | NADH:ubiquinone oxidoreductase subunit B4 [Source:HGNC Symbol;Acc:HGNC:7699] |
| ENSG00000066926 | HGLibA_17271 | 33 | -0.836197174 | FECH | ferrochelatase [Source:HGNC Symbol;Acc:HGNC:3647] |
| ENSG00000067836 | HGLibA_41750 | 33 | -0.836197174 | ROGDI | rogdi homolog [Source:HGNC Symbol;Acc:HGNC:29478] |
| ENSG00000070476 | HGLibA_57079 | 33 | -0.836197174 | ZXDC | ZXD family zinc finger C [Source:HGNC Symbol;Acc:HGNC:28160] |
| ENSG00000075340 | HGLibA_00943 | 33 | -0.836197174 | ADD2 | adducin 2 [Source:HGNC Symbol;Acc:HGNC:244] |
| ENSG00000077348 | HGLibA_15783 | 33 | -0.836197174 | EXOSC5 | exosome component 5 [Source:HGNC Symbol;Acc:HGNC:24662] |
| ENSG00000079313 | HGLibA_40889 | 33 | -0.836197174 | REXO1 | RNA exonuclease 1 homolog [Source:HGNC Symbol;Acc:HGNC:24616] |
| ENSG00000082996 | HGLibA_41503 | 33 | -0.836197174 | RNF13 | ring finger protein 13 [Source:HGNC Symbol;Acc:HGNC:10057] |
| ENSG00000083844 | HGLibA_55696 | 33 | -0.836197174 | ZNF264 | zinc finger protein 264 [Source:HGNC Symbol;Acc:HGNC:13057] |
| ENSG00000087269 | HGLibA_32304 | 33 | -0.836197174 | NOP14 | NOP14 nucleolar protein [Source:HGNC Symbol;Acc:HGNC:16821] |
| ENSG00000090263 | HGLibA_30093 | 33 | -0.836197174 | MRPS33 | mitochondrial ribosomal protein S33 [Source:HGNC Symbol;Acc:HGNC:16634] |
| ENSG00000092148 | HGLibA_21094 | 33 | -0.836197174 | HECTD1 | HECT domain E3 ubiquitin protein ligase 1 [Source:HGNC Symbol;Acc:HGNC:20157] |
| ENSG00000094796 | HGLibA_25473 | 33 | -0.836197174 | KRT31 | keratin 31 [Source:HGNC Symbol;Acc:HGNC:6448] |
| ENSG00000095627 | HGLibA_48895 | 33 | -0.836197174 | TDRD1 | tudor domain containing 1 [Source:HGNC Symbol;Acc:HGNC:11712] |
| ENSG00000096384 | HGLibA_22254 | 33 | -0.836197174 | HSP90AB1 | heat shock protein 90 alpha family class B member 1 [Source:HGNC Symbol;Acc:HGNC:5258] |
| ENSG00000100379 | HGLibA_24436 | 33 | -0.836197174 | KCTD17 | potassium channel tetramerization domain containing 17 [Source:HGNC Symbol;Acc:HGNC:25705] |
| ENSG00000100412 | HGLibA_00464 | 33 | -0.836197174 | ACO2 | aconitase 2 [Source:HGNC Symbol;Acc:HGNC:118] |
| ENSG00000101210 | HGLibA_14492 | 33 | -0.836197174 | EEF1A2 | eukaryotic translation elongation factor 1 alpha 2 [Source:HGNC Symbol;Acc:HGNC:3192] |
| ENSG00000101574 | HGLibA_29008 | 33 | -0.836197174 | METTL4 | methyltransferase like 4 [Source:HGNC Symbol;Acc:HGNC:24726] |
| ENSG00000102038 | HGLibA_45603 | 33 | -0.836197174 | SMARCA1 | member 1 [Source:HGNC Symbol;Acc:HGNC:11097]" |
| ENSG00000103091 | HGLibA_54129 | 33 | -0.836197174 | WDR59 | WD repeat domain 59 [Source:HGNC Symbol;Acc:HGNC:25706] |
| ENSG00000103194 | HGLibA_53193 | 33 | -0.836197174 | USP10 | ubiquitin specific peptidase 10 [Source:HGNC Symbol;Acc:HGNC:12608] |
| ENSG00000103426 | HGLibA_10793 | 33 | -0.836197174 | CORO7-PAM16 | CORO7-PAM16 readthrough [Source:HGNC Symbol;Acc:HGNC:44424] |
| ENSG00000103512 | HGLibA_32288 | 33 | -0.836197174 | NOMO1 | NODAL modulator 1 [Source:HGNC Symbol;Acc:HGNC:30060] |
| ENSG00000104044 | HGLibA_33240 | 33 | -0.836197174 | OCA2 | OCA2 melanosomal transmembrane protein [Source:HGNC Symbol;Acc:HGNC:8101] |
| ENSG00000104783 | HGLibA_24364 | 33 | -0.836197174 | KCNN4 | potassium calcium-activated channel subfamily N member 4 [Source:HGNC Symbol;Acc:HGNC:6293] |
| ENSG00000104976 | HGLibA_45894 | 33 | -0.836197174 | SNAPC2 | small nuclear RNA activating complex polypeptide 2 [Source:HGNC Symbol;Acc:HGNC:11135] |
| ENSG00000105672 | HGLibA_15651 | 33 | -0.836197174 | ETV2 | ETS variant 2 [Source:HGNC Symbol;Acc:HGNC:3491] |
| ENSG00000107551 | HGLibA_40384 | 33 | -0.836197174 | RASSF4 | Ras association domain family member 4 [Source:HGNC Symbol;Acc:HGNC:20793] |
| ENSG00000108298 | HGLibA_41892 | 33 | -0.836197174 | RPL19 | ribosomal protein L19 [Source:HGNC Symbol;Acc:HGNC:10312] |
| ENSG00000108439 | HGLibA_37429 | 33 | -0.836197174 | PNPO | pyridoxamine 5'-phosphate oxidase [Source:HGNC Symbol;Acc:HGNC:30260] |
| ENSG00000108846 | HGLibA_00174 | 33 | -0.836197174 | ABCC3 | ATP binding cassette subfamily C member 3 [Source:HGNC Symbol;Acc:HGNC:54] |
| ENSG00000109118 | HGLibA_36340 | 33 | -0.836197174 | PHF12 | PHD finger protein 12 [Source:HGNC Symbol;Acc:HGNC:20816] |
| ENSG00000109920 | HGLibA_17741 | 33 | -0.836197174 | FNBP4 | formin binding protein 4 [Source:HGNC Symbol;Acc:HGNC:19752] |
| ENSG00000110075 | HGLibA_38226 | 33 | -0.836197174 | PPP6R3 | protein phosphatase 6 regulatory subunit 3 [Source:HGNC Symbol;Acc:HGNC:1173] |
| ENSG00000111846 | HGLibA_18813 | 33 | -0.836197174 | GCNT2 | glucosaminyl (N-acetyl) transferase 2 (I blood group) [Source:HGNC Symbol;Acc:HGNC:4204] |
| ENSG00000111846 | HGLibA_18812 | 33 | -0.836197174 | GCNT2 | glucosaminyl (N-acetyl) transferase 2 (I blood group) [Source:HGNC Symbol;Acc:HGNC:4204] |
| ENSG00000113384 | HGLibA_19657 | 33 | -0.836197174 | GOLPH3 | golgi phosphoprotein 3 [Source:HGNC Symbol;Acc:HGNC:15452] |
| ENSG00000114013 | HGLibA_08468 | 33 | -0.836197174 | CD86 | CD86 molecule [Source:HGNC Symbol;Acc:HGNC:1705] |
| ENSG00000114126 | HGLibA_49153 | 33 | -0.836197174 | TFDP2 | transcription factor Dp-2 [Source:HGNC Symbol;Acc:HGNC:11751] |
| ENSG00000114626 | HGLibA_00335 | 33 | -0.836197174 | ABTB1 | ankyrin repeat and BTB domain containing 1 [Source:HGNC Symbol;Acc:HGNC:18275] |
| ENSG00000115421 | HGLibA_35141 | 33 | -0.836197174 | PAPOLG | poly(A) polymerase gamma [Source:HGNC Symbol;Acc:HGNC:14982] |
| ENSG00000116205 | HGLibA_48732 | 33 | -0.836197174 | TCEANC2 | transcription elongation factor A N-terminal and central domain containing 2 [Source:HGNC Symbol;Acc:HGNC:26494] |
| ENSG00000116251 | HGLibA_41897 | 33 | -0.836197174 | RPL22 | ribosomal protein L22 [Source:HGNC Symbol;Acc:HGNC:10315] |
| ENSG00000116489 | HGLibA_07204 | 33 | -0.836197174 | CAPZA1 | capping actin protein of muscle Z-line subunit alpha 1 [Source:HGNC Symbol;Acc:HGNC:1488] |

| | | | | | |
|---|---|---|---|---|---|
| ENSG00000116679 | HGLibA_23883 | 33 | -0.836197174 | IVNS1ABP | influenza virus NS1A binding protein [Source:HGNC Symbol;Acc:HGNC:16951] |
| ENSG00000117148 | HGLibA_00632 | 33 | -0.836197174 | ACTL8 | actin like 8 [Source:HGNC Symbol;Acc:HGNC:24018] |
| ENSG00000117461 | HGLibA_36654 | 33 | -0.836197174 | PIK3R3 | phosphoinositide-3-kinase regulatory subunit 3 [Source:HGNC Symbol;Acc:HGNC:8981] |
| ENSG00000118007 | HGLibA_47320 | 33 | -0.836197174 | STAG1 | stromal antigen 1 [Source:HGNC Symbol;Acc:HGNC:11354] |
| ENSG00000118402 | HGLibA_14965 | 33 | -0.836197174 | ELOVL4 | ELOVL fatty acid elongase 4 [Source:HGNC Symbol;Acc:HGNC:14415] |
| ENSG00000118898 | HGLibA_37961 | 33 | -0.836197174 | PPL | periplakin [Source:HGNC Symbol;Acc:HGNC:9273] |
| ENSG00000119402 | HGLibA_17145 | 33 | -0.836197174 | FBXW2 | F-box and WD repeat domain containing 2 [Source:HGNC Symbol;Acc:HGNC:13608] |
| ENSG00000119638 | HGLibA_31667 | 33 | -0.836197174 | NEK9 | NIMA related kinase 9 [Source:HGNC Symbol;Acc:HGNC:18591] |
| ENSG00000119711 | HGLibA_01579 | 33 | -0.836197174 | ALDH6A1 | aldehyde dehydrogenase 6 family member A1 [Source:HGNC Symbol;Acc:HGNC:7179] |
| ENSG00000119973 | HGLibA_38603 | 33 | -0.836197174 | PRLHR | prolactin releasing hormone receptor [Source:HGNC Symbol;Acc:HGNC:4464] |
| ENSG00000121104 | HGLibA_16042 | 33 | -0.836197174 | FAM117A | family with sequence similarity 117 member A [Source:HGNC Symbol;Acc:HGNC:24179] |
| ENSG00000121895 | HGLibA_50011 | 33 | -0.836197174 | TMEM156 | transmembrane protein 156 [Source:HGNC Symbol;Acc:HGNC:26260] |
| ENSG00000121904 | HGLibA_11366 | 33 | -0.836197174 | CSMD2 | CUB and Sushi multiple domains 2 [Source:HGNC Symbol;Acc:HGNC:19290] |
| ENSG00000122033 | HGLibA_30399 | 33 | -0.836197174 | MTIF3 | mitochondrial translational initiation factor 3 [Source:HGNC Symbol;Acc:HGNC:29788] |
| ENSG00000122417 | HGLibA_33280 | 33 | -0.836197174 | ODF2L | outer dense fiber of sperm tails 2 like [Source:HGNC Symbol;Acc:HGNC:29225] |
| ENSG00000122584 | HGLibA_33161 | 33 | -0.836197174 | NXPH1 | neurexophilin 1 [Source:HGNC Symbol;Acc:HGNC:20693] |
| ENSG00000123607 | HGLibA_52072 | 33 | -0.836197174 | TTC21B | tetratricopeptide repeat domain 21B [Source:HGNC Symbol;Acc:HGNC:25660] |
| ENSG00000123908 | HGLibA_01207 | 33 | -0.836197174 | AGO2 | RISC catalytic component [Source:HGNC Symbol;Acc:HGNC:3263]" |
| ENSG00000123933 | HGLibA_30615 | 33 | -0.836197174 | MXD4 | MAX dimerization protein 4 [Source:HGNC Symbol;Acc:HGNC:13906] |
| ENSG00000124568 | HGLibA_44458 | 33 | -0.836197174 | SLC17A1 | solute carrier family 17 member 1 [Source:HGNC Symbol;Acc:HGNC:10929] |
| ENSG00000125850 | HGLibA_34805 | 33 | -0.836197174 | OVOL2 | ovo like zinc finger 2 [Source:HGNC Symbol;Acc:HGNC:15804] |
| ENSG00000126952 | HGLibA_33138 | 33 | -0.836197174 | NXF5 | nuclear RNA export factor 5 [Source:HGNC Symbol;Acc:HGNC:8075] |
| ENSG00000128294 | HGLibA_51208 | 33 | -0.836197174 | TPST2 | tyrosylprotein sulfotransferase 2 [Source:HGNC Symbol;Acc:HGNC:12021] |
| ENSG00000129214 | HGLibA_43998 | 33 | -0.836197174 | SHBG | sex hormone binding globulin [Source:HGNC Symbol;Acc:HGNC:10839] |
| ENSG00000130545 | HGLibA_11074 | 33 | -0.836197174 | CRB3 | cell polarity complex component [Source:HGNC Symbol;Acc:HGNC:20237]" |
| ENSG00000130649 | HGLibA_12129 | 33 | -0.836197174 | CYP2E1 | cytochrome P450 family 2 subfamily E member 1 [Source:HGNC Symbol;Acc:HGNC:2631] |
| ENSG00000130956 | HGLibA_20796 | 33 | -0.836197174 | HABP4 | hyaluronan binding protein 4 [Source:HGNC Symbol;Acc:HGNC:17062] |
| ENSG00000131233 | HGLibA_19153 | 33 | -0.836197174 | GJA9 | gap junction protein alpha 9 [Source:HGNC Symbol;Acc:HGNC:19155] |
| ENSG00000131379 | HGLibA_06218 | 33 | -0.836197174 | C3orf20 | chromosome 3 open reading frame 20 [Source:HGNC Symbol;Acc:HGNC:25320] |
| ENSG00000131899 | HGLibA_26647 | 33 | -0.836197174 | LLGL1 | scribble cell polarity complex component [Source:HGNC Symbol;Acc:HGNC:6628]" |
| ENSG00000131914 | HGLibA_26559 | 33 | -0.836197174 | LIN28A | lin-28 homolog A [Source:HGNC Symbol;Acc:HGNC:15986] |
| ENSG00000132199 | HGLibA_15156 | 33 | -0.836197174 | ENOSF1 | enolase superfamily member 1 [Source:HGNC Symbol;Acc:HGNC:30365] |
| ENSG00000133246 | HGLibA_38273 | 33 | -0.836197174 | PRAM1 | PML-RARA regulated adaptor molecule 1 [Source:HGNC Symbol;Acc:HGNC:30091] |
| ENSG00000134001 | HGLibA_14752 | 33 | -0.836197174 | EIF2S1 | eukaryotic translation initiation factor 2 subunit alpha [Source:HGNC Symbol;Acc:HGNC:3265] |
| ENSG00000134313 | HGLibA_24847 | 33 | -0.836197174 | KIDINS220 | kinase D interacting substrate 220 [Source:HGNC Symbol;Acc:HGNC:29508] |
| ENSG00000134461 | HGLibA_02012 | 33 | -0.836197174 | ANKRD16 | ankyrin repeat domain 16 [Source:HGNC Symbol;Acc:HGNC:23471] |
| ENSG00000135249 | HGLibA_41304 | 33 | -0.836197174 | RINT1 | RAD50 interactor 1 [Source:HGNC Symbol;Acc:HGNC:21876] |
| ENSG00000135404 | HGLibA_08427 | 33 | -0.836197174 | CD63 | CD63 molecule [Source:HGNC Symbol;Acc:HGNC:1692] |
| ENSG00000135413 | HGLibA_25914 | 33 | -0.836197174 | LACRT | lacritin [Source:HGNC Symbol;Acc:HGNC:16430] |
| ENSG00000136152 | HGLibA_10476 | 33 | -0.836197174 | COG3 | component of oligomeric golgi complex 3 [Source:HGNC Symbol;Acc:HGNC:18619] |
| ENSG00000136425 | HGLibA_09686 | 33 | -0.836197174 | CIB2 | calcium and integrin binding family member 2 [Source:HGNC Symbol;Acc:HGNC:24579] |
| ENSG00000136546 | HGLibA_43001 | 33 | -0.836197174 | SCN7A | sodium voltage-gated channel alpha subunit 7 [Source:HGNC Symbol;Acc:HGNC:10594] |
| ENSG00000136698 | HGLibA_09249 | 33 | -0.836197174 | CFC1 | cryptic family 1 [Source:HGNC Symbol;Acc:HGNC:18292]" |
| ENSG00000136816 | HGLibA_51025 | 33 | -0.836197174 | TOR1B | torsin family 1 member B [Source:HGNC Symbol;Acc:HGNC:11995] |
| ENSG00000136933 | HGLibA_40007 | 33 | -0.836197174 | RABEPK | Rab9 effector protein with kelch motifs [Source:HGNC Symbol;Acc:HGNC:16896] |
| ENSG00000137312 | HGLibA_17649 | 33 | -0.836197174 | FLOT1 | flotillin 1 [Source:HGNC Symbol;Acc:HGNC:3757] |
| ENSG00000137745 | HGLibA_29488 | 33 | -0.836197174 | MMP13 | matrix metallopeptidase 13 [Source:HGNC Symbol;Acc:HGNC:7159] |
| ENSG00000137752 | HGLibA_07294 | 33 | -0.836197174 | CASP1 | caspase 1 [Source:HGNC Symbol;Acc:HGNC:1499] |
| ENSG00000137962 | HGLibA_02749 | 33 | -0.836197174 | ARHGAP29 | Rho GTPase activating protein 29 [Source:HGNC Symbol;Acc:HGNC:30207] |
| ENSG00000138759 | HGLibA_17974 | 33 | -0.836197174 | FRAS1 | Fraser extracellular matrix complex subunit 1 [Source:HGNC Symbol;Acc:HGNC:19185] |
| ENSG00000138769 | HGLibA_08810 | 33 | -0.836197174 | CDKL2 | cyclin dependent kinase like 2 [Source:HGNC Symbol;Acc:HGNC:1782] |
| ENSG00000138794 | HGLibA_07318 | 33 | -0.836197174 | CASP6 | caspase 6 [Source:HGNC Symbol;Acc:HGNC:1507] |
| ENSG00000139291 | HGLibA_50138 | 33 | -0.836197174 | TMEM19 | transmembrane protein 19 [Source:HGNC Symbol;Acc:HGNC:25605] |
| ENSG00000139914 | HGLibA_17539 | 33 | -0.836197174 | FITM1 | fat storage inducing transmembrane protein 1 [Source:HGNC Symbol;Acc:HGNC:33714] |
| ENSG00000140030 | HGLibA_20073 | 33 | -0.836197174 | GPR65 | G protein-coupled receptor 65 [Source:HGNC Symbol;Acc:HGNC:4517] |
| ENSG00000140600 | HGLibA_43953 | 33 | -0.836197174 | SH3GL3 | endophilin A3 [Source:HGNC Symbol;Acc:HGNC:10832]" |
| ENSG00000141446 | HGLibA_15558 | 33 | -0.836197174 | ESCO1 | establishment of sister chromatid cohesion N-acetyltransferase 1 [Source:HGNC Symbol;Acc:HGNC:24645] |
| ENSG00000141873 | HGLibA_45109 | 33 | -0.836197174 | SLC39A3 | solute carrier family 39 member 3 [Source:HGNC Symbol;Acc:HGNC:17128] |
| ENSG00000143344 | HGLibA_41002 | 33 | -0.836197174 | RGL1 | ral guanine nucleotide dissociation stimulator like 1 [Source:HGNC Symbol;Acc:HGNC:30281] |
| ENSG00000143369 | HGLibA_14411 | 33 | -0.836197174 | ECM1 | extracellular matrix protein 1 [Source:HGNC Symbol;Acc:HGNC:3153] |
| ENSG00000143793 | HGLibA_05854 | 33 | -0.836197174 | C1orf35 | chromosome 1 open reading frame 35 [Source:HGNC Symbol;Acc:HGNC:19032] |
| ENSG00000143947 | HGLibA_42126 | 33 | -0.836197174 | RPS27A | ribosomal protein S27a [Source:HGNC Symbol;Acc:HGNC:10417] |
| ENSG00000145220 | HGLibA_27696 | 33 | -0.836197174 | LYAR | Ly1 antibody reactive [Source:HGNC Symbol;Acc:HGNC:26021] |
| ENSG00000149357 | HGLibA_25990 | 33 | -0.836197174 | LAMTOR1 | MAPK and MTOR activator 1 [Source:HGNC Symbol;Acc:HGNC:26068]" |
| ENSG00000149591 | HGLibA_48269 | 33 | -0.836197174 | TAGLN | transgelin [Source:HGNC Symbol;Acc:HGNC:11553] |
| ENSG00000149651 | HGLibA_46720 | 33 | -0.836197174 | SPINT4 | Kunitz type 4 [Source:HGNC Symbol;Acc:HGNC:16130]" |
| ENSG00000150337 | HGLibA_17184 | 33 | -0.836197174 | FCGR1A | Fc fragment of IgG receptor Ia [Source:HGNC Symbol;Acc:HGNC:3613] |
| ENSG00000151025 | HGLibA_19951 | 33 | -0.836197174 | GPR158 | G protein-coupled receptor 158 [Source:HGNC Symbol;Acc:HGNC:23689] |
| ENSG00000151233 | HGLibA_20682 | 33 | -0.836197174 | GXYLT1 | glucoside xylosyltransferase 1 [Source:HGNC Symbol;Acc:HGNC:27482] |
| ENSG00000151461 | HGLibA_53058 | 33 | -0.836197174 | UPF2 | regulator of nonsense mediated mRNA decay [Source:HGNC Symbol;Acc:HGNC:17854]" |
| ENSG00000152782 | HGLibA_35098 | 33 | -0.836197174 | PANK1 | pantothenate kinase 1 [Source:HGNC Symbol;Acc:HGNC:8598] |
| ENSG00000153786 | HGLibA_55126 | 33 | -0.836197174 | ZDHHC7 | zinc finger DHHC-type containing 7 [Source:HGNC Symbol;Acc:HGNC:18459] |
| ENSG00000155085 | HGLibA_01401 | 33 | -0.836197174 | AK9 | adenylate kinase 9 [Source:HGNC Symbol;Acc:HGNC:33814] |
| ENSG00000155827 | HGLibA_41611 | 33 | -0.836197174 | RNF20 | ring finger protein 20 [Source:HGNC Symbol;Acc:HGNC:10062] |
| ENSG00000156802 | HGLibA_03405 | 33 | -0.836197174 | ATAD2 | AAA domain containing 2 [Source:HGNC Symbol;Acc:HGNC:30123]" |
| ENSG00000158234 | HGLibA_15965 | 33 | -0.836197174 | FAIM | Fas apoptotic inhibitory molecule [Source:HGNC Symbol;Acc:HGNC:18703] |
| ENSG00000158748 | HGLibA_22400 | 33 | -0.836197174 | HTR6 | 5-hydroxytryptamine receptor 6 [Source:HGNC Symbol;Acc:HGNC:5301] |
| ENSG00000159023 | HGLibA_15238 | 33 | -0.836197174 | EPB41 | erythrocyte membrane protein band 4.1 [Source:HGNC Symbol;Acc:HGNC:3377] |
| ENSG00000159337 | HGLibA_36908 | 33 | -0.836197174 | PLA2G4D | phospholipase A2 group IVD [Source:HGNC Symbol;Acc:HGNC:30038] |
| ENSG00000160683 | HGLibA_11894 | 33 | -0.836197174 | CXCR5 | C-X-C motif chemokine receptor 5 [Source:HGNC Symbol;Acc:HGNC:1060] |
| ENSG00000162086 | HGLibA_56676 | 33 | -0.836197174 | ZNF75A | zinc finger protein 75a [Source:HGNC Symbol;Acc:HGNC:13146] |
| ENSG00000162999 | HGLibA_14153 | 33 | -0.836197174 | DUSP19 | dual specificity phosphatase 19 [Source:HGNC Symbol;Acc:HGNC:18894] |
| ENSG00000163209 | HGLibA_46825 | 33 | -0.836197174 | SPRR3 | small proline rich protein 3 [Source:HGNC Symbol;Acc:HGNC:11268] |
| ENSG00000163568 | HGLibA_01352 | 33 | -0.836197174 | AIM2 | absent in melanoma 2 [Source:HGNC Symbol;Acc:HGNC:357] |
| ENSG00000164164 | HGLibA_34765 | 33 | -0.836197174 | OTUD4 | OTU deubiquitinase 4 [Source:HGNC Symbol;Acc:HGNC:24949] |
| ENSG00000164287 | HGLibA_08520 | 33 | -0.836197174 | CDC20B | cell division cycle 20B [Source:HGNC Symbol;Acc:HGNC:24222] |
| ENSG00000164708 | HGLibA_36198 | 33 | -0.836197174 | PGAM2 | phosphoglycerate mutase 2 [Source:HGNC Symbol;Acc:HGNC:8889] |
| ENSG00000164919 | HGLibA_10855 | 33 | -0.836197174 | COX6C | cytochrome c oxidase subunit 6C [Source:HGNC Symbol;Acc:HGNC:2285] |
| ENSG00000164949 | HGLibA_18908 | 33 | -0.836197174 | GEM | GTP binding protein overexpressed in skeletal muscle [Source:HGNC Symbol;Acc:HGNC:4234] |
| ENSG00000165322 | HGLibA_02707 | 33 | -0.836197174 | ARHGAP12 | Rho GTPase activating protein 12 [Source:HGNC Symbol;Acc:HGNC:16348] |
| ENSG00000166342 | HGLibA_31705 | 33 | -0.836197174 | NETO1 | neuropilin and tolloid like 1 [Source:HGNC Symbol;Acc:HGNC:13823] |
| ENSG00000167615 | HGLibA_26299 | 33 | -0.836197174 | LENG8 | leukocyte receptor cluster member 8 [Source:HGNC Symbol;Acc:HGNC:15500] |
| ENSG00000168061 | HGLibA_42623 | 33 | -0.836197174 | SAC3D1 | SAC3 domain containing 1 [Source:HGNC Symbol;Acc:HGNC:30179] |
| ENSG00000168765 | HGLibA_20480 | 33 | -0.836197174 | GSTM4 | glutathione S-transferase mu 4 [Source:HGNC Symbol;Acc:HGNC:4636] |
| ENSG00000168936 | HGLibA_49922 | 33 | -0.836197174 | TMEM129 | transmembrane protein 129 [Source:HGNC Symbol;Acc:HGNC:25137] |
| ENSG00000169032 | HGLibA_28138 | 33 | -0.836197174 | MAP2K1 | mitogen-activated protein kinase kinase 1 [Source:HGNC Symbol;Acc:HGNC:6840] |
| ENSG00000169047 | HGLibA_23618 | 33 | -0.836197174 | IRS1 | insulin receptor substrate 1 [Source:HGNC Symbol;Acc:HGNC:6125] |
| ENSG00000170385 | HGLibA_44896 | 33 | -0.836197174 | SLC30A1 | solute carrier family 30 member 1 [Source:HGNC Symbol;Acc:HGNC:11012] |
| ENSG00000170540 | HGLibA_02986 | 33 | -0.836197174 | ARL6IP1 | ADP ribosylation factor like GTPase 6 interacting protein 1 [Source:HGNC Symbol;Acc:HGNC:697] |
| ENSG00000170832 | HGLibA_53335 | 33 | -0.836197174 | USP32 | ubiquitin specific peptidase 32 [Source:HGNC Symbol;Acc:HGNC:19143] |
| ENSG00000171443 | HGLibA_56161 | 33 | -0.836197174 | ZNF524 | zinc finger protein 524 [Source:HGNC Symbol;Acc:HGNC:28322] |
| ENSG00000172331 | HGLibA_04700 | 33 | -0.836197174 | BPGM | bisphosphoglycerate mutase [Source:HGNC Symbol;Acc:HGNC:1093] |
| ENSG00000172379 | HGLibA_03065 | 33 | -0.836197174 | ARNT2 | aryl hydrocarbon receptor nuclear translocator 2 [Source:HGNC Symbol;Acc:HGNC:16876] |
| ENSG00000173157 | HGLibA_00824 | 33 | -0.836197174 | ADAMTS20 | ADAM metallopeptidase with thrombospondin type 1 motif 20 [Source:HGNC Symbol;Acc:HGNC:17178] |
| ENSG00000173281 | HGLibA_38100 | 33 | -0.836197174 | PPP1R3B | protein phosphatase 1 regulatory subunit 3B [Source:HGNC Symbol;Acc:HGNC:14942] |
| ENSG00000173338 | HGLibA_24333 | 33 | -0.836197174 | KCNK7 | potassium two pore domain channel subfamily K member 7 [Source:HGNC Symbol;Acc:HGNC:6282] |
| ENSG00000174788 | HGLibA_35657 | 33 | -0.836197174 | PCP2 | Purkinje cell protein 2 [Source:HGNC Symbol;Acc:HGNC:30209] |
| ENSG00000175161 | HGLibA_06969 | 33 | -0.836197174 | CADM2 | cell adhesion molecule 2 [Source:HGNC Symbol;Acc:HGNC:29849] |
| ENSG00000177398 | HGLibA_52980 | 33 | -0.836197174 | UMODL1 | uromodulin like 1 [Source:HGNC Symbol;Acc:HGNC:12560] |
| ENSG00000177839 | HGLibA_35513 | 33 | -0.836197174 | PCDHB9 | protocadherin beta 9 [Source:HGNC Symbol;Acc:HGNC:8694] |
| ENSG00000178460 | HGLibA_28633 | 33 | -0.836197174 | MCMDC2 | minichromosome maintenance domain containing 2 [Source:HGNC Symbol;Acc:HGNC:26368] |
| ENSG00000179331 | HGLibA_39901 | 33 | -0.836197174 | RAB39A | member RAS oncogene family [Source:HGNC Symbol;Acc:HGNC:16521]" |
| ENSG00000179774 | HGLibA_03535 | 33 | -0.836197174 | ATOH7 | atonal bHLH transcription factor 7 [Source:HGNC Symbol;Acc:HGNC:13907] |
| ENSG00000180891 | HGLibA_11766 | 33 | -0.836197174 | CUEDC1 | CUE domain containing 1 [Source:HGNC Symbol;Acc:HGNC:31350] |
| ENSG00000181092 | HGLibA_00976 | 33 | -0.836197174 | ADIPOQ | C1Q and collagen domain containing [Source:HGNC Symbol;Acc:HGNC:13633]" |
| ENSG00000182415 | HGLibA_08912 | 33 | -0.836197174 | CDY2A | chromodomain Y-linked 2A [Source:HGNC Symbol;Acc:HGNC:1810] |
| ENSG00000183542 | HGLibA_25293 | 33 | -0.836197174 | KLRC4 | killer cell lectin like receptor C4 [Source:HGNC Symbol;Acc:HGNC:6377] |
| ENSG00000183778 | HGLibA_03963 | 33 | -0.836197174 | B3GALT5 | 3-galactosyltransferase 5 [Source:HGNC Symbol;Acc:HGNC:920]" |
| ENSG00000184014 | HGLibA_12959 | 33 | -0.836197174 | DENND5A | DENN domain containing 5A [Source:HGNC Symbol;Acc:HGNC:19344] |
| ENSG00000184432 | HGLibA_10697 | 33 | -0.836197174 | COPB2 | coatomer protein complex subunit beta 2 [Source:HGNC Symbol;Acc:HGNC:2232] |
| ENSG00000184515 | HGLibA_04450 | 33 | -0.836197174 | BEX5 | brain expressed X-linked 5 [Source:HGNC Symbol;Acc:HGNC:27990] |
| ENSG00000184634 | HGLibA_28742 | 33 | -0.836197174 | MED12 | mediator complex subunit 12 [Source:HGNC Symbol;Acc:HGNC:11957] |
| ENSG00000185187 | HGLibA_44120 | 33 | -0.836197174 | SIGIRR | single Ig and TIR domain containing [Source:HGNC Symbol;Acc:HGNC:30575] |
| ENSG00000185359 | HGLibA_21245 | 33 | -0.836197174 | HGS | hepatocyte growth factor-regulated tyrosine kinase substrate [Source:HGNC Symbol;Acc:HGNC:4897] |
| ENSG00000186051 | HGLibA_48283 | 33 | -0.836197174 | TAL2 | TAL bHLH transcription factor 2 [Source:HGNC Symbol;Acc:HGNC:11557] |
| ENSG00000186086 | HGLibA_31240 | 33 | -0.836197174 | NBPF6 | NBPF member 6 [Source:HGNC Symbol;Acc:HGNC:31988] |
| ENSG00000186132 | HGLibA_06183 | 33 | -0.836197174 | C2orf76 | chromosome 2 open reading frame 76 [Source:HGNC Symbol;Acc:HGNC:27017] |
| ENSG00000186314 | HGLibA_38431 | 33 | -0.836197174 | PRELID2 | PRELI domain containing 2 [Source:HGNC Symbol;Acc:HGNC:28306] |
| ENSG00000187527 | HGLibA_03578 | 33 | -0.836197174 | ATP13A5 | ATPase 13A5 [Source:HGNC Symbol;Acc:HGNC:31789] |
| ENSG00000187753 | HGLibA_06693 | 33 | -0.836197174 | C9orf153 | chromosome 9 open reading frame 153 [Source:HGNC Symbol;Acc:HGNC:31456] |
| ENSG00000188171 | HGLibA_56422 | 33 | -0.836197174 | ZNF626 | zinc finger protein 626 [Source:HGNC Symbol;Acc:HGNC:30461] |
| ENSG00000188732 | HGLibA_16516 | 33 | -0.836197174 | FAM221A | family with sequence similarity 221 member A [Source:HGNC Symbol;Acc:HGNC:27977] |
| ENSG00000189326 | HGLibA_46411 | 33 | -0.836197174 | SPANXN4 | SPANX family member N4 [Source:HGNC Symbol;Acc:HGNC:33177] |
| ENSG00000196415 | HGLibA_38982 | 33 | -0.836197174 | PRTN3 | proteinase 3 [Source:HGNC Symbol;Acc:HGNC:9495] |
| ENSG00000196569 | HGLibA_25945 | 33 | -0.836197174 | LAMA2 | laminin subunit alpha 2 [Source:HGNC Symbol;Acc:HGNC:6482] |

| | | | | | |
|---|---|---|---|---|---|
| ENSG00000196653 | HGLibA_56113 | 33 | -0.836197174 | ZNF502 | zinc finger protein 502 [Source:HGNC Symbol;Acc:HGNC:23718] |
| ENSG00000196792 | HGLibA_47596 | 33 | -0.836197174 | STRN3 | striatin 3 [Source:HGNC Symbol;Acc:HGNC:15720] |
| ENSG00000197617 | HGLibA_53706 | 33 | -0.836197174 | VN1R5 | vomeronasal 1 receptor 5 (gene/pseudogene) [Source:HGNC Symbol;Acc:HGNC:19870] |
| ENSG00000198088 | HGLibA_33065 | 33 | -0.836197174 | NUP62CL | nucleoporin 62 C-terminal like [Source:HGNC Symbol;Acc:HGNC:25960] |
| ENSG00000198835 | HGLibA_19182 | 33 | -0.836197174 | GJC2 | gap junction protein gamma 2 [Source:HGNC Symbol;Acc:HGNC:17494] |
| ENSG00000198939 | HGLibA_55193 | 33 | -0.836197174 | ZFP2 | ZFP2 zinc finger protein [Source:HGNC Symbol;Acc:HGNC:26138] |
| ENSG00000204231 | HGLibA_42508 | 33 | -0.836197174 | RXRB | retinoid X receptor beta [Source:HGNC Symbol;Acc:HGNC:10478] |
| ENSG00000204347 | HGLibA_04906 | 33 | -0.836197174 | BTBD17 | BTB domain containing 17 [Source:HGNC Symbol;Acc:HGNC:33758] |
| ENSG00000204538 | HGLibA_39220 | 33 | -0.836197174 | PSORS1C2 | psoriasis susceptibility 1 candidate 2 [Source:HGNC Symbol;Acc:HGNC:17199] |
| ENSG00000204704 | HGLibA_33903 | 33 | -0.836197174 | OR2W1 | olfactory receptor family 2 subfamily W member 1 [Source:HGNC Symbol;Acc:HGNC:8281] |
| ENSG00000205356 | HGLibA_48940 | 33 | -0.836197174 | TECPR1 | tectonin beta-propeller repeat containing 1 [Source:HGNC Symbol;Acc:HGNC:22214] |
| ENSG00000206127 | HGLibA_19645 | 33 | -0.836197174 | GOLGA8O | golgin A8 family member O [Source:HGNC Symbol;Acc:HGNC:44406] |
| ENSG00000213638 | HGLibA_00883 | 33 | -0.836197174 | ADAT3 | tRNA specific 3 [Source:HGNC Symbol;Acc:HGNC:25151]" |
| ENSG00000213648 | HGLibA_47739 | 33 | -0.836197174 | SULT1A4 | sulfotransferase family 1A member 4 [Source:HGNC Symbol;Acc:HGNC:30004] |
| ENSG00000213928 | HGLibA_23604 | 33 | -0.836197174 | IRF9 | interferon regulatory factor 9 [Source:HGNC Symbol;Acc:HGNC:6131] |
| ENSG00000214193 | HGLibA_43944 | 33 | -0.836197174 | SH3D21 | SH3 domain containing 21 [Source:HGNC Symbol;Acc:HGNC:26236] |
| ENSG00000222011 | HGLibA_16333 | 33 | -0.836197174 | FAM185A | family with sequence similarity 185 member A [Source:HGNC Symbol;Acc:HGNC:22412] |
| ENSG00000229859 | HGLibA_36187 | 33 | -0.836197174 | PGA3 | group I (pepsinogen A) [Source:HGNC Symbol;Acc:HGNC:8885]" |
| ENSG00000233954 | HGLibA_53126 | 33 | -0.836197174 | UQCRHL | ubiquinol-cytochrome c reductase hinge protein like [Source:HGNC Symbol;Acc:HGNC:51714] |
| ENSG00000236311 | HGLibA_49672 | 33 | -0.836197174 | TLX1NB | TLX1 neighbor [Source:HGNC Symbol;Acc:HGNC:37183] |
| ENSG00000241058 | HGLibA_32792 | 33 | -0.836197174 | NSUN6 | NOP2/Sun RNA methyltransferase family member 6 [Source:HGNC Symbol;Acc:HGNC:23529] |
| ENSG00000243147 | HGLibA_29954 | 33 | -0.836197174 | MRPL33 | mitochondrial ribosomal protein L33 [Source:HGNC Symbol;Acc:HGNC:14487] |
| ENSG00000244187 | HGLibA_49963 | 33 | -0.836197174 | TMEM141 | transmembrane protein 141 [Source:HGNC Symbol;Acc:HGNC:28211] |
| ENSG00000250361 | HGLibA_20700 | 33 | -0.836197174 | GYPB | glycophorin B (MNS blood group) [Source:HGNC Symbol;Acc:HGNC:4703] |
| ENSG00000256188 | HGLibA_48388 | 33 | -0.836197174 | TAS2R30 | taste 2 receptor member 30 [Source:HGNC Symbol;Acc:HGNC:19112] |
| ENSG00000257727 | HGLibA_10366 | 33 | -0.836197174 | CNPY2 | canopy FGF signaling regulator 2 [Source:HGNC Symbol;Acc:HGNC:13529] |
| ENSG00000280165 | HGLibA_35412 | 33 | -0.836197174 | PCDH20 | protocadherin 20 [Source:HGNC Symbol;Acc:HGNC:14257] |
| ENSG00000280165 | HGLibA_35411 | 33 | -0.836197174 | PCDH20 | protocadherin 20 [Source:HGNC Symbol;Acc:HGNC:14257] |
| ENSG00000282608 | HGLibA_01019 | 33 | -0.836197174 | ADORA3 | adenosine A3 receptor [Source:HGNC Symbol;Acc:HGNC:268] |
| FAM155B | HGLibA_16184 | 33 | -0.836197174 | FAM155B | - |
| FAM212A | HGLibA_16466 | 33 | -0.836197174 | FAM212A | - |
| FAM21A | HGLibA_16504 | 33 | -0.836197174 | FAM21A | - |
| FAM86C1 | HGLibA_16778 | 33 | -0.836197174 | FAM86C1 | - |
| FLJ22184 | HGLibA_17620 | 33 | -0.836197174 | FLJ22184 | - |
| HARS | HGLibA_20856 | 33 | -0.836197174 | HARS | - |
| HIST1H2BE | HGLibA_21423 | 33 | -0.836197174 | HIST1H2BE | - |
| HIST1H4I | HGLibA_21510 | 33 | -0.836197174 | HIST1H4I | - |
| HIST2H2AA3 | HGLibA_21522 | 33 | -0.836197174 | HIST2H2AA3 | - |
| hsa-mir-103b-1 | HGLibA_57170 | 33 | -0.836197174 | hsa-mir-103b-1 | - |
| hsa-mir-1179 | HGLibA_57210 | 33 | -0.836197174 | hsa-mir-1179 | - |
| hsa-mir-1184-1 | HGLibA_57231 | 33 | -0.836197174 | hsa-mir-1184-1 | - |
| hsa-mir-126 | HGLibA_57454 | 33 | -0.836197174 | hsa-mir-126 | - |
| hsa-mir-1302-9 | HGLibA_57725 | 33 | -0.836197174 | hsa-mir-1302-9 | - |
| hsa-mir-149 | HGLibA_57886 | 33 | -0.836197174 | hsa-mir-149 | - |
| hsa-mir-219a-2 | HGLibA_58282 | 33 | -0.836197174 | hsa-mir-219a-2 | - |
| hsa-mir-301b | HGLibA_58434 | 33 | -0.836197174 | hsa-mir-301b | - |
| hsa-mir-302c | HGLibA_58446 | 33 | -0.836197174 | hsa-mir-302c | - |
| hsa-mir-30d | HGLibA_58488 | 33 | -0.836197174 | hsa-mir-30d | - |
| hsa-mir-3118-4 | HGLibA_58517 | 33 | -0.836197174 | hsa-mir-3118-4 | - |
| hsa-mir-3119-1 | HGLibA_58523 | 33 | -0.836197174 | hsa-mir-3119-1 | - |
| hsa-mir-3176 | HGLibA_58777 | 33 | -0.836197174 | hsa-mir-3176 | - |
| hsa-mir-3672 | HGLibA_59243 | 33 | -0.836197174 | hsa-mir-3672 | - |
| hsa-mir-4263 | HGLibA_59759 | 33 | -0.836197174 | hsa-mir-4263 | - |
| hsa-mir-4264 | HGLibA_59762 | 33 | -0.836197174 | hsa-mir-4264 | - |
| hsa-mir-4283-1 | HGLibA_59834 | 33 | -0.836197174 | hsa-mir-4283-1 | - |
| hsa-mir-4308 | HGLibA_59945 | 33 | -0.836197174 | hsa-mir-4308 | - |
| hsa-mir-4439 | HGLibA_60158 | 33 | -0.836197174 | hsa-mir-4439 | - |
| hsa-mir-4500 | HGLibA_60429 | 33 | -0.836197174 | hsa-mir-4500 | - |
| hsa-mir-4657 | HGLibA_60735 | 33 | -0.836197174 | hsa-mir-4657 | - |
| hsa-mir-4666b | HGLibA_60782 | 33 | -0.836197174 | hsa-mir-4666b | - |
| hsa-mir-4739 | HGLibA_61053 | 33 | -0.836197174 | hsa-mir-4739 | - |
| hsa-mir-4751 | HGLibA_61102 | 33 | -0.836197174 | hsa-mir-4751 | - |
| hsa-mir-4783 | HGLibA_61238 | 33 | -0.836197174 | hsa-mir-4783 | - |
| hsa-mir-4797 | HGLibA_61294 | 33 | -0.836197174 | hsa-mir-4797 | - |
| hsa-mir-4797 | HGLibA_61293 | 33 | -0.836197174 | hsa-mir-4797 | - |
| hsa-mir-521-1 | HGLibA_61768 | 33 | -0.836197174 | hsa-mir-521-1 | - |
| hsa-mir-548t | HGLibA_62067 | 33 | -0.836197174 | hsa-mir-548t | - |
| hsa-mir-5689 | HGLibA_62273 | 33 | -0.836197174 | hsa-mir-5689 | - |
| hsa-mir-6077-2 | HGLibA_62563 | 33 | -0.836197174 | hsa-mir-6077-2 | - |
| hsa-mir-6129 | HGLibA_62663 | 33 | -0.836197174 | hsa-mir-6129 | - |
| hsa-mir-6779 | HGLibA_63289 | 33 | -0.836197174 | hsa-mir-6779 | - |
| hsa-mir-6800 | HGLibA_63376 | 33 | -0.836197174 | hsa-mir-6800 | - |
| hsa-mir-6815 | HGLibA_63437 | 33 | -0.836197174 | hsa-mir-6815 | - |
| hsa-mir-6851 | HGLibA_63575 | 33 | -0.836197174 | hsa-mir-6851 | - |
| hsa-mir-6862-2 | HGLibA_63633 | 33 | -0.836197174 | hsa-mir-6862-2 | - |
| hsa-mir-6872 | HGLibA_63671 | 33 | -0.836197174 | hsa-mir-6872 | - |
| hsa-mir-6873 | HGLibA_63677 | 33 | -0.836197174 | hsa-mir-6873 | - |
| hsa-mir-8081 | HGLibA_64165 | 33 | -0.836197174 | hsa-mir-8081 | - |
| IL8 | HGLibA_23197 | 33 | -0.836197174 | IL8 | - |
| KIAA0907 | HGLibA_24664 | 33 | -0.836197174 | KIAA0907 | - |
| LOC100287399 | HGLibA_26822 | 33 | -0.836197174 | LOC100287399 | - |
| MLLT4 | HGLibA_29420 | 33 | -0.836197174 | MLLT4 | - |
| PIH1D3 | HGLibA_36614 | 33 | -0.836197174 | PIH1D3 | - |
| SARS | HGLibA_42748 | 33 | -0.836197174 | SARS | - |
| CECR6 | HGLibA_08984 | 32 | -0.845242517 | CECR6 | - |
| CT45A4 | HGLibA_11507 | 32 | -0.845242517 | CT45A4 | - |
| ENSG00000008294 | HGLibA_46371 | 32 | -0.845242517 | SPAG9 | sperm associated antigen 9 [Source:HGNC Symbol;Acc:HGNC:14524] |
| ENSG00000011021 | HGLibA_09835 | 32 | -0.845242517 | CLCN6 | chloride voltage-gated channel 6 [Source:HGNC Symbol;Acc:HGNC:2024] |
| ENSG00000011260 | HGLibA_53444 | 32 | -0.845242517 | UTP18 | small subunit processome component [Source:HGNC Symbol;Acc:HGNC:24274]" |
| ENSG00000029639 | HGLibA_49139 | 32 | -0.845242517 | TFB1M | mitochondrial [Source:HGNC Symbol;Acc:HGNC:17037]" |
| ENSG00000049089 | HGLibA_10622 | 32 | -0.845242517 | COL9A2 | collagen type IX alpha 2 chain [Source:HGNC Symbol;Acc:HGNC:2218] |
| ENSG00000054282 | HGLibA_43117 | 32 | -0.845242517 | SDCCAG8 | serologically defined colon cancer antigen 8 [Source:HGNC Symbol;Acc:HGNC:10671] |
| ENSG00000060140 | HGLibA_47683 | 32 | -0.845242517 | STYK1 | serine/threonine/tyrosine kinase 1 [Source:HGNC Symbol;Acc:HGNC:18889] |
| ENSG00000075856 | HGLibA_42757 | 32 | -0.845242517 | SART3 | U4/U6 recycling protein [Source:HGNC Symbol;Acc:HGNC:16860]" |
| ENSG00000075945 | HGLibA_24968 | 32 | -0.845242517 | KIFAP3 | kinesin associated protein 3 [Source:HGNC Symbol;Acc:HGNC:17060] |
| ENSG00000078140 | HGLibA_52623 | 32 | -0.845242517 | UBE2K | ubiquitin conjugating enzyme E2 K [Source:HGNC Symbol;Acc:HGNC:4914] |
| ENSG00000079101 | HGLibA_10164 | 32 | -0.845242517 | CLUL1 | clusterin like 1 [Source:HGNC Symbol;Acc:HGNC:2096] |
| ENSG00000087510 | HGLibA_49128 | 32 | -0.845242517 | TFAP2C | transcription factor AP-2 gamma [Source:HGNC Symbol;Acc:HGNC:11744] |
| ENSG00000088986 | HGLibA_14254 | 32 | -0.845242517 | DYNLL1 | dynein light chain LC8-type 1 [Source:HGNC Symbol;Acc:HGNC:15476] |
| ENSG00000089057 | HGLibA_44609 | 32 | -0.845242517 | SLC23A2 | solute carrier family 23 member 2 [Source:HGNC Symbol;Acc:HGNC:10973] |
| ENSG00000089127 | HGLibA_33198 | 32 | -0.845242517 | OAS1 | 2'-5'-oligoadenylate synthetase 1 [Source:HGNC Symbol;Acc:HGNC:8086] |
| ENSG00000091483 | HGLibA_17472 | 32 | -0.845242517 | FH | fumarate hydratase [Source:HGNC Symbol;Acc:HGNC:3700] |
| ENSG00000095015 | HGLibA_28161 | 32 | -0.845242517 | MAP3K1 | mitogen-activated protein kinase kinase kinase 1 [Source:HGNC Symbol;Acc:HGNC:6848] |
| ENSG00000095585 | HGLibA_04554 | 32 | -0.845242517 | BLNK | B cell linker [Source:HGNC Symbol;Acc:HGNC:14211] |
| ENSG00000100632 | HGLibA_15476 | 32 | -0.845242517 | ERH | mRNA splicing and mitosis factor [Source:HGNC Symbol;Acc:HGNC:3447]" |
| ENSG00000100883 | HGLibA_47007 | 32 | -0.845242517 | SRP54 | signal recognition particle 54 [Source:HGNC Symbol;Acc:HGNC:11301] |
| ENSG00000101438 | HGLibA_44931 | 32 | -0.845242517 | SLC32A1 | solute carrier family 32 member 1 [Source:HGNC Symbol;Acc:HGNC:11018] |
| ENSG00000101670 | HGLibA_26611 | 32 | -0.845242517 | LIPG | endothelial type [Source:HGNC Symbol;Acc:HGNC:6623]" |
| ENSG00000102362 | HGLibA_48105 | 32 | -0.845242517 | SYTL4 | synaptotagmin like 4 [Source:HGNC Symbol;Acc:HGNC:15588] |
| ENSG00000102543 | HGLibA_08501 | 32 | -0.845242517 | CDADC1 | cytidine and dCMP deaminase domain containing 1 [Source:HGNC Symbol;Acc:HGNC:20299] |
| ENSG00000102879 | HGLibA_10774 | 32 | -0.845242517 | CORO1A | coronin 1A [Source:HGNC Symbol;Acc:HGNC:2252] |
| ENSG00000102897 | HGLibA_27746 | 32 | -0.845242517 | LYRM1 | LYR motif containing 1 [Source:HGNC Symbol;Acc:HGNC:25074] |
| ENSG00000103121 | HGLibA_10187 | 32 | -0.845242517 | CMC2 | C-X9-C motif containing 2 [Source:HGNC Symbol;Acc:HGNC:24447] |
| ENSG00000104067 | HGLibA_49570 | 32 | -0.845242517 | TJP1 | tight junction protein 1 [Source:HGNC Symbol;Acc:HGNC:11827] |
| ENSG00000104412 | HGLibA_15008 | 32 | -0.845242517 | EMC2 | ER membrane protein complex subunit 2 [Source:HGNC Symbol;Acc:HGNC:28963] |
| ENSG00000105325 | HGLibA_18290 | 32 | -0.845242517 | FZR1 | fizzy and cell division cycle 20 related 1 [Source:HGNC Symbol;Acc:HGNC:24824] |
| ENSG00000106853 | HGLibA_39353 | 32 | -0.845242517 | PTGR1 | prostaglandin reductase 1 [Source:HGNC Symbol;Acc:HGNC:18429] |
| ENSG00000107130 | HGLibA_31375 | 32 | -0.845242517 | NCS1 | neuronal calcium sensor 1 [Source:HGNC Symbol;Acc:HGNC:3953] |
| ENSG00000107745 | HGLibA_29221 | 32 | -0.845242517 | MICU1 | mitochondrial calcium uptake 1 [Source:HGNC Symbol;Acc:HGNC:1530] |
| ENSG00000109066 | HGLibA_49846 | 32 | -0.845242517 | TMEM104 | transmembrane protein 104 [Source:HGNC Symbol;Acc:HGNC:25984] |
| ENSG00000109171 | HGLibA_44310 | 32 | -0.845242517 | SLAIN2 | SLAIN motif family member 2 [Source:HGNC Symbol;Acc:HGNC:29282] |
| ENSG00000109321 | HGLibA_02640 | 32 | -0.845242517 | AREG | amphiregulin [Source:HGNC Symbol;Acc:HGNC:651] |
| ENSG00000109471 | HGLibA_23069 | 32 | -0.845242517 | IL2 | interleukin 2 [Source:HGNC Symbol;Acc:HGNC:6001] |
| ENSG00000109758 | HGLibA_21243 | 32 | -0.845242517 | HGFAC | HGF activator [Source:HGNC Symbol;Acc:HGNC:4894] |
| ENSG00000110172 | HGLibA_09515 | 32 | -0.845242517 | CHORDC1 | cysteine and histidine rich domain containing 1 [Source:HGNC Symbol;Acc:HGNC:14525] |
| ENSG00000110195 | HGLibA_17782 | 32 | -0.845242517 | FOLR1 | folate receptor 1 [Source:HGNC Symbol;Acc:HGNC:3791] |
| ENSG00000110851 | HGLibA_38390 | 32 | -0.845242517 | PRDM4 | PR/SET domain 4 [Source:HGNC Symbol;Acc:HGNC:9348] |
| ENSG00000111647 | HGLibA_52951 | 32 | -0.845242517 | UHRF1BP1L | UHRF1 binding protein 1 like [Source:HGNC Symbol;Acc:HGNC:29102] |
| ENSG00000111653 | HGLibA_23293 | 32 | -0.845242517 | ING4 | inhibitor of growth family member 4 [Source:HGNC Symbol;Acc:HGNC:19423] |
| ENSG00000111961 | HGLibA_42761 | 32 | -0.845242517 | SASH1 | SAM and SH3 domain containing 1 [Source:HGNC Symbol;Acc:HGNC:19182] |
| ENSG00000113430 | HGLibA_23637 | 32 | -0.845242517 | IRX4 | iroquois homeobox 4 [Source:HGNC Symbol;Acc:HGNC:6129] |
| ENSG00000113722 | HGLibA_08895 | 32 | -0.845242517 | CDX1 | caudal type homeobox 1 [Source:HGNC Symbol;Acc:HGNC:1805] |
| ENSG00000115665 | HGLibA_45280 | 32 | -0.845242517 | SLC5A7 | solute carrier family 5 member 7 [Source:HGNC Symbol;Acc:HGNC:14025] |
| ENSG00000115840 | HGLibA_44642 | 32 | -0.845242517 | SLC25A12 | solute carrier family 25 member 12 [Source:HGNC Symbol;Acc:HGNC:10982] |
| ENSG00000116016 | HGLibA_15235 | 32 | -0.845242517 | EPAS1 | endothelial PAS domain protein 1 [Source:HGNC Symbol;Acc:HGNC:3374] |

| | | | | | |
|---|---|---|---|---|---|
| ENSG00000116521 | HGLibA_42843 | 32 | -0.845242517 | SCAMP3 | secretory carrier membrane protein 3 [Source:HGNC Symbol;Acc:HGNC:10565] |
| ENSG00000116874 | HGLibA_53947 | 32 | -0.845242517 | WARS2 | mitochondrial [Source:HGNC Symbol;Acc:HGNC:12730]" |
| ENSG00000117090 | HGLibA_44311 | 32 | -0.845242517 | SLAMF1 | signaling lymphocytic activation molecule family member 1 [Source:HGNC Symbol;Acc:HGNC:10903] |
| ENSG00000117501 | HGLibA_29883 | 32 | -0.845242517 | MROH9 | maestro heat like repeat family member 9 [Source:HGNC Symbol;Acc:HGNC:26287] |
| ENSG00000118873 | HGLibA_39922 | 32 | -0.845242517 | RAB3GAP2 | RAB3 GTPase activating non-catalytic protein subunit 2 [Source:HGNC Symbol;Acc:HGNC:17168] |
| ENSG00000119314 | HGLibA_39256 | 32 | -0.845242517 | PTBP3 | polypyrimidine tract binding protein 3 [Source:HGNC Symbol;Acc:HGNC:10253] |
| ENSG00000124459 | HGLibA_56027 | 32 | -0.845242517 | ZNF45 | zinc finger protein 45 [Source:HGNC Symbol;Acc:HGNC:13111] |
| ENSG00000126749 | HGLibA_15041 | 32 | -0.845242517 | EMG1 | N1-specific pseudouridine methyltransferase [Source:HGNC Symbol;Acc:HGNC:16912]" |
| ENSG00000127124 | HGLibA_21573 | 32 | -0.845242517 | HIVEP3 | human immunodeficiency virus type I enhancer binding protein 3 [Source:HGNC Symbol;Acc:HGNC:13561] |
| ENSG00000128585 | HGLibA_29340 | 32 | -0.845242517 | MKLN1 | muskelin 1 [Source:HGNC Symbol;Acc:HGNC:7109] |
| ENSG00000128694 | HGLibA_34684 | 32 | -0.845242517 | OSGEPL1 | O-sialoglycoprotein endopeptidase like 1 [Source:HGNC Symbol;Acc:HGNC:23075] |
| ENSG00000128881 | HGLibA_52041 | 32 | -0.845242517 | TTBK2 | tau tubulin kinase 2 [Source:HGNC Symbol;Acc:HGNC:19141] |
| ENSG00000131067 | HGLibA_19019 | 32 | -0.845242517 | GGT7 | gamma-glutamyltransferase 7 [Source:HGNC Symbol;Acc:HGNC:4259] |
| ENSG00000133422 | HGLibA_29651 | 32 | -0.845242517 | MORC2 | MORC family CW-type zinc finger 2 [Source:HGNC Symbol;Acc:HGNC:23573] |
| ENSG00000133958 | HGLibA_53029 | 32 | -0.845242517 | UNC79 | NALCN channel complex subunit [Source:HGNC Symbol;Acc:HGNC:19966]" |
| ENSG00000134970 | HGLibA_49820 | 32 | -0.845242517 | TMED7 | transmembrane p24 trafficking protein 7 [Source:HGNC Symbol;Acc:HGNC:24253] |
| ENSG00000135441 | HGLibA_04556 | 32 | -0.845242517 | BLOC1S1 | biogenesis of lysosomal organelles complex 1 subunit 1 [Source:HGNC Symbol;Acc:HGNC:4200] |
| ENSG00000135931 | HGLibA_03034 | 32 | -0.845242517 | ARMC9 | armadillo repeat containing 9 [Source:HGNC Symbol;Acc:HGNC:20730] |
| ENSG00000136122 | HGLibA_04698 | 32 | -0.845242517 | BORA | aurora kinase A activator [Source:HGNC Symbol;Acc:HGNC:24724]" |
| ENSG00000136848 | HGLibA_12272 | 32 | -0.845242517 | DAB2IP | DAB2 interacting protein [Source:HGNC Symbol;Acc:HGNC:17294] |
| ENSG00000136982 | HGLibA_14005 | 32 | -0.845242517 | DSCC1 | DNA replication and sister chromatid cohesion 1 [Source:HGNC Symbol;Acc:HGNC:24453] |
| ENSG00000137261 | HGLibA_24621 | 32 | -0.845242517 | KIAA0319 | KIAA0319 [Source:HGNC Symbol;Acc:HGNC:21580] |
| ENSG00000137936 | HGLibA_04247 | 32 | -0.845242517 | BCAR3 | NSP family adaptor protein [Source:HGNC Symbol;Acc:HGNC:973]" |
| ENSG00000138835 | HGLibA_41092 | 32 | -0.845242517 | RGS3 | regulator of G protein signaling 3 [Source:HGNC Symbol;Acc:HGNC:9999] |
| ENSG00000139626 | HGLibA_23796 | 32 | -0.845242517 | ITGB7 | integrin subunit beta 7 [Source:HGNC Symbol;Acc:HGNC:6162] |
| ENSG00000140527 | HGLibA_54222 | 32 | -0.845242517 | WDR93 | WD repeat domain 93 [Source:HGNC Symbol;Acc:HGNC:26924] |
| ENSG00000140943 | HGLibA_28536 | 32 | -0.845242517 | MBTPS1 | site 1 [Source:HGNC Symbol;Acc:HGNC:15456]" |
| ENSG00000141646 | HGLibA_45578 | 32 | -0.845242517 | SMAD4 | SMAD family member 4 [Source:HGNC Symbol;Acc:HGNC:6770] |
| ENSG00000142694 | HGLibA_15674 | 32 | -0.845242517 | EVA1B | eva-1 homolog B [Source:HGNC Symbol;Acc:HGNC:25558] |
| ENSG00000143353 | HGLibA_27744 | 32 | -0.845242517 | LYPLAL1 | lysophospholipase like 1 [Source:HGNC Symbol;Acc:HGNC:20440] |
| ENSG00000143549 | HGLibA_51147 | 32 | -0.845242517 | TPM3 | tropomyosin 3 [Source:HGNC Symbol;Acc:HGNC:12012] |
| ENSG00000143627 | HGLibA_36827 | 32 | -0.845242517 | PKLR | pyruvate kinase L/R [Source:HGNC Symbol;Acc:HGNC:9020] |
| ENSG00000143631 | HGLibA_17608 | 32 | -0.845242517 | FLG | filaggrin [Source:HGNC Symbol;Acc:HGNC:3748] |
| ENSG00000144290 | HGLibA_45206 | 32 | -0.845242517 | SLC4A10 | solute carrier family 4 member 10 [Source:HGNC Symbol;Acc:HGNC:13811] |
| ENSG00000144827 | HGLibA_00233 | 32 | -0.845242517 | ABHD10 | abhydrolase domain containing 10 [Source:HGNC Symbol;Acc:HGNC:25656] |
| ENSG00000146910 | HGLibA_10362 | 32 | -0.845242517 | CNPY1 | canopy FGF signaling regulator 1 [Source:HGNC Symbol;Acc:HGNC:27786] |
| ENSG00000147274 | HGLibA_40596 | 32 | -0.845242517 | RBMX | RNA binding motif protein X-linked [Source:HGNC Symbol;Acc:HGNC:9910] |
| ENSG00000148291 | HGLibA_47836 | 32 | -0.845242517 | SURF2 | surfeit 2 [Source:HGNC Symbol;Acc:HGNC:11475] |
| ENSG00000148600 | HGLibA_08697 | 32 | -0.845242517 | CDHR1 | cadherin related family member 1 [Source:HGNC Symbol;Acc:HGNC:14550] |
| ENSG00000149968 | HGLibA_29535 | 32 | -0.845242517 | MMP3 | matrix metallopeptidase 3 [Source:HGNC Symbol;Acc:HGNC:7173] |
| ENSG00000151615 | HGLibA_37779 | 32 | -0.845242517 | POU4F2 | POU class 4 homeobox 2 [Source:HGNC Symbol;Acc:HGNC:9219] |
| ENSG00000152439 | HGLibA_56712 | 32 | -0.845242517 | ZNF773 | zinc finger protein 773 [Source:HGNC Symbol;Acc:HGNC:30487] |
| ENSG00000153060 | HGLibA_48982 | 32 | -0.845242517 | TEKT5 | tektin 5 [Source:HGNC Symbol;Acc:HGNC:26554] |
| ENSG00000154309 | HGLibA_13281 | 32 | -0.845242517 | DISP1 | dispatched RND transporter family member 1 [Source:HGNC Symbol;Acc:HGNC:19711] |
| ENSG00000154589 | HGLibA_27693 | 32 | -0.845242517 | LY96 | lymphocyte antigen 96 [Source:HGNC Symbol;Acc:HGNC:17156] |
| ENSG00000156970 | HGLibA_04991 | 32 | -0.845242517 | BUB1B | BUB1 mitotic checkpoint serine/threonine kinase B [Source:HGNC Symbol;Acc:HGNC:1149] |
| ENSG00000159086 | HGLibA_35323 | 32 | -0.845242517 | PAXBP1 | PAX3 and PAX7 binding protein 1 [Source:HGNC Symbol;Acc:HGNC:13579] |
| ENSG00000159496 | HGLibA_41010 | 32 | -0.845242517 | RGL4 | ral guanine nucleotide dissociation stimulator like 4 [Source:HGNC Symbol;Acc:HGNC:31911] |
| ENSG00000160471 | HGLibA_10851 | 32 | -0.845242517 | COX6B2 | cytochrome c oxidase subunit 6B2 [Source:HGNC Symbol;Acc:HGNC:24380] |
| ENSG00000160710 | HGLibA_00869 | 32 | -0.845242517 | ADAR | RNA specific [Source:HGNC Symbol;Acc:HGNC:225]" |
| ENSG00000160796 | HGLibA_31213 | 32 | -0.845242517 | NBEAL2 | neurobeachin like 2 [Source:HGNC Symbol;Acc:HGNC:31928] |
| ENSG00000162374 | HGLibA_14888 | 32 | -0.845242517 | ELAVL4 | ELAV like RNA binding protein 4 [Source:HGNC Symbol;Acc:HGNC:3315] |
| ENSG00000162441 | HGLibA_27797 | 32 | -0.845242517 | LZIC | leucine zipper and CTNNBIP1 domain containing [Source:HGNC Symbol;Acc:HGNC:17497] |
| ENSG00000163755 | HGLibA_22049 | 32 | -0.845242517 | HPS3 | biogenesis of lysosomal organelles complex 2 subunit 1 [Source:HGNC Symbol;Acc:HGNC:15597]" |
| ENSG00000164484 | HGLibA_50171 | 32 | -0.845242517 | TMEM200A | transmembrane protein 200A [Source:HGNC Symbol;Acc:HGNC:21075] |
| ENSG00000165376 | HGLibA_09877 | 32 | -0.845242517 | CLDN2 | claudin 2 [Source:HGNC Symbol;Acc:HGNC:2041] |
| ENSG00000165516 | HGLibA_25097 | 32 | -0.845242517 | KLHDC2 | kelch domain containing 2 [Source:HGNC Symbol;Acc:HGNC:20231] |
| ENSG00000165819 | HGLibA_29007 | 32 | -0.845242517 | METTL3 | methyltransferase like 3 [Source:HGNC Symbol;Acc:HGNC:17563] |
| ENSG00000165912 | HGLibA_34967 | 32 | -0.845242517 | PACSIN3 | protein kinase C and casein kinase substrate in neurons 3 [Source:HGNC Symbol;Acc:HGNC:8572] |
| ENSG00000166351 | HGLibA_37726 | 32 | -0.845242517 | POTED | POTE ankyrin domain family member D [Source:HGNC Symbol;Acc:HGNC:23822] |
| ENSG00000166971 | HGLibA_01517 | 32 | -0.845242517 | AKTIP | AKT interacting protein [Source:HGNC Symbol;Acc:HGNC:16710] |
| ENSG00000166997 | HGLibA_10370 | 32 | -0.845242517 | CNPY4 | canopy FGF signaling regulator 4 [Source:HGNC Symbol;Acc:HGNC:28631] |
| ENSG00000167105 | HGLibA_50562 | 32 | -0.845242517 | TMEM92 | transmembrane protein 92 [Source:HGNC Symbol;Acc:HGNC:26579] |
| ENSG00000167325 | HGLibA_42251 | 32 | -0.845242517 | RRM1 | ribonucleotide reductase catalytic subunit M1 [Source:HGNC Symbol;Acc:HGNC:10451] |
| ENSG00000167476 | HGLibA_23997 | 32 | -0.845242517 | JSRP1 | junctional sarcoplasmic reticulum protein 1 [Source:HGNC Symbol;Acc:HGNC:24963] |
| ENSG00000168118 | HGLibA_39954 | 32 | -0.845242517 | RAB4A | member RAS oncogene family [Source:HGNC Symbol;Acc:HGNC:9781]" |
| ENSG00000168993 | HGLibA_10933 | 32 | -0.845242517 | CPLX1 | complexin 1 [Source:HGNC Symbol;Acc:HGNC:2309] |
| ENSG00000169155 | HGLibA_54889 | 32 | -0.845242517 | ZBTB43 | zinc finger and BTB domain containing 43 [Source:HGNC Symbol;Acc:HGNC:17908] |
| ENSG00000169752 | HGLibA_32685 | 32 | -0.845242517 | NRG4 | neuregulin 4 [Source:HGNC Symbol;Acc:HGNC:29862] |
| ENSG00000169906 | HGLibA_42582 | 32 | -0.845242517 | S100G | S100 calcium binding protein G [Source:HGNC Symbol;Acc:HGNC:1436] |
| ENSG00000170145 | HGLibA_44165 | 32 | -0.845242517 | SIK2 | salt inducible kinase 2 [Source:HGNC Symbol;Acc:HGNC:21680] |
| ENSG00000170430 | HGLibA_29165 | 32 | -0.845242517 | MGMT | O-6-methylguanine-DNA methyltransferase [Source:HGNC Symbol;Acc:HGNC:7059] |
| ENSG00000170523 | HGLibA_25563 | 32 | -0.845242517 | KRT83 | keratin 83 [Source:HGNC Symbol;Acc:HGNC:6460] |
| ENSG00000171208 | HGLibA_31706 | 32 | -0.845242517 | NETO2 | neuropilin and tolloid like 2 [Source:HGNC Symbol;Acc:HGNC:14644] |
| ENSG00000171345 | HGLibA_25442 | 32 | -0.845242517 | KRT19 | keratin 19 [Source:HGNC Symbol;Acc:HGNC:6436] |
| ENSG00000171489 | HGLibA_46334 | 32 | -0.845242517 | SPACA5 | sperm acrosome associated 5 [Source:HGNC Symbol;Acc:HGNC:31353] |
| ENSG00000171566 | HGLibA_37222 | 32 | -0.845242517 | PLRG1 | pleiotropic regulator 1 [Source:HGNC Symbol;Acc:HGNC:9089] |
| ENSG00000171703 | HGLibA_48699 | 32 | -0.845242517 | TCEA2 | transcription elongation factor A2 [Source:HGNC Symbol;Acc:HGNC:11614] |
| ENSG00000171865 | HGLibA_41423 | 32 | -0.845242517 | RNASEH1 | ribonuclease H1 [Source:HGNC Symbol;Acc:HGNC:18466] |
| ENSG00000172062 | HGLibA_45783 | 32 | -0.845242517 | SMN1 | telomeric [Source:HGNC Symbol;Acc:HGNC:11117]" |
| ENSG00000172489 | HGLibA_34362 | 32 | -0.845242517 | OR5T3 | olfactory receptor family 5 subfamily T member 3 [Source:HGNC Symbol;Acc:HGNC:15297] |
| ENSG00000172785 | HGLibA_07431 | 32 | -0.845242517 | CBWD1 | COBW domain containing 1 [Source:HGNC Symbol;Acc:HGNC:17134] |
| ENSG00000174547 | HGLibA_29894 | 32 | -0.845242517 | MRPL11 | mitochondrial ribosomal protein L11 [Source:HGNC Symbol;Acc:HGNC:14042] |
| ENSG00000174989 | HGLibA_17157 | 32 | -0.845242517 | FBXW8 | F-box and WD repeat domain containing 8 [Source:HGNC Symbol;Acc:HGNC:13597] |
| ENSG00000175946 | HGLibA_25200 | 32 | -0.845242517 | KLHL38 | kelch like family member 38 [Source:HGNC Symbol;Acc:HGNC:34435] |
| ENSG00000176029 | HGLibA_05117 | 32 | -0.845242517 | C11orf16 | chromosome 11 open reading frame 16 [Source:HGNC Symbol;Acc:HGNC:1169] |
| ENSG00000176198 | HGLibA_33577 | 32 | -0.845242517 | OR11H4 | olfactory receptor family 11 subfamily H member 4 [Source:HGNC Symbol;Acc:HGNC:15347] |
| ENSG00000177239 | HGLibA_28063 | 32 | -0.845242517 | MAN1B1 | mannosidase alpha class 1B member 1 [Source:HGNC Symbol;Acc:HGNC:6823] |
| ENSG00000178021 | HGLibA_51990 | 32 | -0.845242517 | TSPYL6 | TSPY like 6 [Source:HGNC Symbol;Acc:HGNC:14521] |
| ENSG00000179021 | HGLibA_06242 | 32 | -0.845242517 | C3orf38 | chromosome 3 open reading frame 38 [Source:HGNC Symbol;Acc:HGNC:28384] |
| ENSG00000180785 | HGLibA_34096 | 32 | -0.845242517 | OR51E1 | olfactory receptor family 51 subfamily E member 1 [Source:HGNC Symbol;Acc:HGNC:15194] |
| ENSG00000180879 | HGLibA_47162 | 32 | -0.845242517 | SSR4 | signal sequence receptor subunit 4 [Source:HGNC Symbol;Acc:HGNC:11326] |
| ENSG00000180891 | HGLibA_11767 | 32 | -0.845242517 | CUEDC1 | CUE domain containing 1 [Source:HGNC Symbol;Acc:HGNC:31350] |
| ENSG00000181090 | HGLibA_14685 | 32 | -0.845242517 | EHMT1 | euchromatic histone lysine methyltransferase 1 [Source:HGNC Symbol;Acc:HGNC:24650] |
| ENSG00000181240 | HGLibA_44738 | 32 | -0.845242517 | SLC25A41 | solute carrier family 25 member 41 [Source:HGNC Symbol;Acc:HGNC:28533] |
| ENSG00000182141 | HGLibA_56603 | 32 | -0.845242517 | ZNF708 | zinc finger protein 708 [Source:HGNC Symbol;Acc:HGNC:12945] |
| ENSG00000183049 | HGLibA_07062 | 32 | -0.845242517 | CAMK1D | calcium/calmodulin dependent protein kinase ID [Source:HGNC Symbol;Acc:HGNC:19341] |
| ENSG00000184524 | HGLibA_09035 | 32 | -0.845242517 | CEND1 | cell cycle exit and neuronal differentiation 1 [Source:HGNC Symbol;Acc:HGNC:24153] |
| ENSG00000185627 | HGLibA_39160 | 32 | -0.845242517 | PSMD13 | non-ATPase 13 [Source:HGNC Symbol;Acc:HGNC:9558]" |
| ENSG00000185742 | HGLibA_05206 | 32 | -0.845242517 | C11orf87 | chromosome 11 open reading frame 87 [Source:HGNC Symbol;Acc:HGNC:33788] |
| ENSG00000186562 | HGLibA_12826 | 32 | -0.845242517 | DEFB105A | defensin beta 105A [Source:HGNC Symbol;Acc:HGNC:18087] |
| ENSG00000187546 | HGLibA_01203 | 32 | -0.845242517 | AGMO | alkylglycerol monooxygenase [Source:HGNC Symbol;Acc:HGNC:33784] |
| ENSG00000187546 | HGLibA_01202 | 32 | -0.845242517 | AGMO | alkylglycerol monooxygenase [Source:HGNC Symbol;Acc:HGNC:33784] |
| ENSG00000187860 | HGLibA_07662 | 32 | -0.845242517 | CCDC157 | coiled-coil domain containing 157 [Source:HGNC Symbol;Acc:HGNC:33854] |
| ENSG00000187908 | HGLibA_13392 | 32 | -0.845242517 | DMBT1 | deleted in malignant brain tumors 1 [Source:HGNC Symbol;Acc:HGNC:2926] |
| ENSG00000188803 | HGLibA_44044 | 32 | -0.845242517 | SHISA6 | shisa family member 6 [Source:HGNC Symbol;Acc:HGNC:34491] |
| ENSG00000188822 | HGLibA_10376 | 32 | -0.845242517 | CNR2 | cannabinoid receptor 2 [Source:HGNC Symbol;Acc:HGNC:2160] |
| ENSG00000196177 | HGLibA_00375 | 32 | -0.845242517 | ACADSB | acyl-CoA dehydrogenase short/branched chain [Source:HGNC Symbol;Acc:HGNC:91] |
| ENSG00000196684 | HGLibA_22246 | 32 | -0.845242517 | HSH2D | hematopoietic SH2 domain containing [Source:HGNC Symbol;Acc:HGNC:24920] |
| ENSG00000197565 | HGLibA_10583 | 32 | -0.845242517 | COL4A6 | collagen type IV alpha 6 chain [Source:HGNC Symbol;Acc:HGNC:2208] |
| ENSG00000197635 | HGLibA_13871 | 32 | -0.845242517 | DPP4 | dipeptidyl peptidase 4 [Source:HGNC Symbol;Acc:HGNC:3009] |
| ENSG00000197885 | HGLibA_32017 | 32 | -0.845242517 | NKIRAS1 | NFKB inhibitor interacting Ras like 1 [Source:HGNC Symbol;Acc:HGNC:17899] |
| ENSG00000198610 | HGLibA_01490 | 32 | -0.845242517 | AKR1C4 | aldo-keto reductase family 1 member C4 [Source:HGNC Symbol;Acc:HGNC:387] |
| ENSG00000198648 | HGLibA_47508 | 32 | -0.845242517 | STK39 | serine/threonine kinase 39 [Source:HGNC Symbol;Acc:HGNC:17717] |
| ENSG00000204518 | HGLibA_00039 | 32 | -0.845242517 | AADACL4 | arylacetamide deacetylase like 4 [Source:HGNC Symbol;Acc:HGNC:32038] |
| ENSG00000204764 | HGLibA_40198 | 32 | -0.845242517 | RANBP17 | RAN binding protein 17 [Source:HGNC Symbol;Acc:HGNC:14428] |
| ENSG00000204936 | HGLibA_08254 | 32 | -0.845242517 | CD177 | CD177 molecule [Source:HGNC Symbol;Acc:HGNC:30072] |
| ENSG00000205238 | HGLibA_46581 | 32 | -0.845242517 | SPDYE2 | speedy/RINGO cell cycle regulator family member E2 [Source:HGNC Symbol;Acc:HGNC:33841] |
| ENSG00000205423 | HGLibA_10256 | 32 | -0.845242517 | CNEP1R1 | CTD nuclear envelope phosphatase 1 regulatory subunit 1 [Source:HGNC Symbol;Acc:HGNC:26759] |
| ENSG00000206150 | HGLibA_41399 | 32 | -0.845242517 | RNASE13 | ribonuclease A family member 13 (inactive) [Source:HGNC Symbol;Acc:HGNC:25285] |
| ENSG00000206150 | HGLibA_41401 | 32 | -0.845242517 | RNASE13 | ribonuclease A family member 13 (inactive) [Source:HGNC Symbol;Acc:HGNC:25285] |
| ENSG00000212658 | HGLibA_25737 | 32 | -0.845242517 | KRTAP29-1 | keratin associated protein 29-1 [Source:HGNC Symbol;Acc:HGNC:34211] |
| ENSG00000213563 | HGLibA_06650 | 32 | -0.845242517 | C8orf82 | chromosome 8 open reading frame 82 [Source:HGNC Symbol;Acc:HGNC:33826] |
| ENSG00000213906 | HGLibA_27588 | 32 | -0.845242517 | LTB4R2 | leukotriene B4 receptor 2 [Source:HGNC Symbol;Acc:HGNC:19260] |
| ENSG00000221970 | HGLibA_33719 | 32 | -0.845242517 | OR2A1 | olfactory receptor family 2 subfamily A member 1 [Source:HGNC Symbol;Acc:HGNC:8229] |
| ENSG00000227488 | HGLibA_18443 | 32 | -0.845242517 | GAGE12D | G antigen 12D [Source:HGNC Symbol;Acc:HGNC:31904] |
| ENSG00000228607 | HGLibA_09893 | 32 | -0.845242517 | CLDN25 | claudin 25 [Source:HGNC Symbol;Acc:HGNC:37218] |
| ENSG00000242875 | HGLibA_40613 | 32 | -0.845242517 | RBMY1B | member B [Source:HGNC Symbol;Acc:HGNC:23914]" |
| ENSG00000242950 | HGLibA_15550 | 32 | -0.845242517 | ERVW-1 | envelope [Source:HGNC Symbol;Acc:HGNC:13525]" |
| ENSG00000254598 | HGLibA_11409 | 32 | -0.845242517 | CSNK2A3 | casein kinase 2 alpha 3 [Source:HGNC Symbol;Acc:HGNC:2458] |
| ENSG00000255223 | HGLibA_34335 | 32 | -0.845242517 | OR5M11 | olfactory receptor family 5 subfamily M member 11 [Source:HGNC Symbol;Acc:HGNC:15291] |
| ENSG00000255713 | HGLibA_33974 | 32 | -0.845242517 | OR4D2 | olfactory receptor family 4 subfamily D member 2 [Source:HGNC Symbol;Acc:HGNC:8294] |
| ENSG00000257008 | HGLibA_19912 | 32 | -0.845242517 | GPR142 | G protein-coupled receptor 142 [Source:HGNC Symbol;Acc:HGNC:20088] |
| ENSG00000260097 | HGLibA_46601 | 32 | -0.845242517 | SPDYE6 | speedy/RINGO cell cycle regulator family member E6 [Source:HGNC Symbol;Acc:HGNC:35465] |
| ENSG00000266472 | HGLibA_30062 | 32 | -0.845242517 | MRPS21 | mitochondrial ribosomal protein S21 [Source:HGNC Symbol;Acc:HGNC:14046] |

| | | | | | |
|---|---|---|---|---|---|
| ENSG00000267978 | HGLibA_27926 | 32 | -0.845242517 | MAGEA9B | MAGE family member A9B [Source:HGNC Symbol;Acc:HGNC:31909] |
| ENSG00000275700 | HGLibA_00085 | 32 | -0.845242517 | AATF | apoptosis antagonizing transcription factor [Source:HGNC Symbol;Acc:HGNC:19235] |
| FAM175B | HGLibA_16292 | 32 | -0.845242517 | FAM175B | - |
| GAGE2D | HGLibA_18476 | 32 | -0.845242517 | GAGE2D | - |
| hsa-mir-1249 | HGLibA_57396 | 32 | -0.845242517 | hsa-mir-1249 | - |
| hsa-mir-1255a | HGLibA_57423 | 32 | -0.845242517 | hsa-mir-1255a | - |
| hsa-mir-1292 | HGLibA_57646 | 32 | -0.845242517 | hsa-mir-1292 | - |
| hsa-mir-1470 | HGLibA_57863 | 32 | -0.845242517 | hsa-mir-1470 | - |
| hsa-mir-198 | HGLibA_58125 | 32 | -0.845242517 | hsa-mir-198 | - |
| hsa-mir-24-2 | HGLibA_58344 | 32 | -0.845242517 | hsa-mir-24-2 | - |
| hsa-mir-3159 | HGLibA_58713 | 32 | -0.845242517 | hsa-mir-3159 | - |
| hsa-mir-3621 | HGLibA_59116 | 32 | -0.845242517 | hsa-mir-3621 | - |
| hsa-mir-369 | HGLibA_59342 | 32 | -0.845242517 | hsa-mir-369 | - |
| hsa-mir-378b | HGLibA_59429 | 32 | -0.845242517 | hsa-mir-378b | - |
| hsa-mir-378c | HGLibA_59434 | 32 | -0.845242517 | hsa-mir-378c | - |
| hsa-mir-3977 | HGLibA_59669 | 32 | -0.845242517 | hsa-mir-3977 | - |
| hsa-mir-4421 | HGLibA_60073 | 32 | -0.845242517 | hsa-mir-4421 | - |
| hsa-mir-4456 | HGLibA_60231 | 32 | -0.845242517 | hsa-mir-4456 | - |
| hsa-mir-4457 | HGLibA_60236 | 32 | -0.845242517 | hsa-mir-4457 | - |
| hsa-mir-4508 | HGLibA_60458 | 32 | -0.845242517 | hsa-mir-4508 | - |
| hsa-mir-4643 | HGLibA_60676 | 32 | -0.845242517 | hsa-mir-4643 | - |
| hsa-mir-4763 | HGLibA_61150 | 32 | -0.845242517 | hsa-mir-4763 | - |
| hsa-mir-5189 | HGLibA_61646 | 32 | -0.845242517 | hsa-mir-5189 | - |
| hsa-mir-588 | HGLibA_62452 | 32 | -0.845242517 | hsa-mir-588 | - |
| hsa-mir-634 | HGLibA_62777 | 32 | -0.845242517 | hsa-mir-634 | - |
| hsa-mir-6511a-1 | HGLibA_62896 | 32 | -0.845242517 | hsa-mir-6511a-1 | - |
| hsa-mir-6514 | HGLibA_62931 | 32 | -0.845242517 | hsa-mir-6514 | - |
| hsa-mir-6780b | HGLibA_63296 | 32 | -0.845242517 | hsa-mir-6780b | - |
| hsa-mir-6831 | HGLibA_63501 | 32 | -0.845242517 | hsa-mir-6831 | - |
| hsa-mir-6874 | HGLibA_63681 | 32 | -0.845242517 | hsa-mir-6874 | - |
| hsa-mir-7845 | HGLibA_63969 | 32 | -0.845242517 | hsa-mir-7845 | - |
| hsa-mir-8055 | HGLibA_64058 | 32 | -0.845242517 | hsa-mir-8055 | - |
| hsa-mir-8058 | HGLibA_64069 | 32 | -0.845242517 | hsa-mir-8058 | - |
| hsa-mir-8058 | HGLibA_64072 | 32 | -0.845242517 | hsa-mir-8058 | - |
| hsa-mir-885 | HGLibA_64223 | 32 | -0.845242517 | hsa-mir-885 | - |
| IFLTD1 | HGLibA_22652 | 32 | -0.845242517 | IFLTD1 | - |
| KAL1 | HGLibA_24018 | 32 | -0.845242517 | KAL1 | - |
| LOC100127983 | HGLibA_26750 | 32 | -0.845242517 | LOC100127983 | - |
| LOC730159 | HGLibA_27001 | 32 | -0.845242517 | LOC730159 | - |
| MESDC2 | HGLibA_28914 | 32 | -0.845242517 | MESDC2 | - |
| PTPLAD1 | HGLibA_39419 | 32 | -0.845242517 | PTPLAD1 | - |
| SPRY3_Y | HGLibA_46844 | 32 | -0.845242517 | SPRY3_Y | - |
| TMEM173 | HGLibA_50062 | 32 | -0.845242517 | TMEM173 | - |
| ADCK4 | HGLibA_00899 | 31 | -0.85428786 | ADCK4 | - |
| C10orf68 | HGLibA_05087 | 31 | -0.85428786 | C10orf68 | - |
| C16orf11 | HGLibA_05439 | 31 | -0.85428786 | C16orf11 | - |
| CIRH1A | HGLibA_09724 | 31 | -0.85428786 | CIRH1A | - |
| CRLF2_X | HGLibA_11188 | 31 | -0.85428786 | CRLF2_X | - |
| DSCR6 | HGLibA_14013 | 31 | -0.85428786 | DSCR6 | - |
| ENSG00000001036 | HGLibA_18157 | 31 | -0.85428786 | FUCA2 | alpha-L-fucosidase 2 [Source:HGNC Symbol;Acc:HGNC:4008] |
| ENSG00000005206 | HGLibA_46778 | 31 | -0.85428786 | SPPL2B | signal peptide peptidase like 2B [Source:HGNC Symbol;Acc:HGNC:30627] |
| ENSG00000006744 | HGLibA_14872 | 31 | -0.85428786 | ELAC2 | elaC ribonuclease Z 2 [Source:HGNC Symbol;Acc:HGNC:14198] |
| ENSG00000009780 | HGLibA_16723 | 31 | -0.85428786 | FAM76A | family with sequence similarity 76 member A [Source:HGNC Symbol;Acc:HGNC:28530] |
| ENSG00000009844 | HGLibA_53880 | 31 | -0.85428786 | VTA1 | vesicle trafficking 1 [Source:HGNC Symbol;Acc:HGNC:20954] |
| ENSG00000010318 | HGLibA_36388 | 31 | -0.85428786 | PHF7 | PHD finger protein 7 [Source:HGNC Symbol;Acc:HGNC:18458] |
| ENSG00000014824 | HGLibA_44923 | 31 | -0.85428786 | SLC30A9 | solute carrier family 30 member 9 [Source:HGNC Symbol;Acc:HGNC:1329] |
| ENSG00000033178 | HGLibA_52527 | 31 | -0.85428786 | UBA6 | ubiquitin like modifier activating enzyme 6 [Source:HGNC Symbol;Acc:HGNC:25581] |
| ENSG00000038382 | HGLibA_51626 | 31 | -0.85428786 | TRIO | trio Rho guanine nucleotide exchange factor [Source:HGNC Symbol;Acc:HGNC:12303] |
| ENSG00000044524 | HGLibA_15294 | 31 | -0.85428786 | EPHA3 | EPH receptor A3 [Source:HGNC Symbol;Acc:HGNC:3387] |
| ENSG00000050130 | HGLibA_23948 | 31 | -0.85428786 | JKAMP | JNK1/MAPK8 associated membrane protein [Source:HGNC Symbol;Acc:HGNC:20184] |
| ENSG00000058673 | HGLibA_54949 | 31 | -0.85428786 | ZC3H11A | zinc finger CCCH-type containing 11A [Source:HGNC Symbol;Acc:HGNC:29093] |
| ENSG00000058866 | HGLibA_13071 | 31 | -0.85428786 | DGKG | diacylglycerol kinase gamma [Source:HGNC Symbol;Acc:HGNC:2853] |
| ENSG00000060491 | HGLibA_33318 | 31 | -0.85428786 | OGFR | opioid growth factor receptor [Source:HGNC Symbol;Acc:HGNC:15768] |
| ENSG00000062598 | HGLibA_14933 | 31 | -0.85428786 | ELMO2 | engulfment and cell motility 2 [Source:HGNC Symbol;Acc:HGNC:17233] |
| ENSG00000075399 | HGLibA_53822 | 31 | -0.85428786 | VPS9D1 | VPS9 domain containing 1 [Source:HGNC Symbol;Acc:HGNC:13526] |
| ENSG00000081148 | HGLibA_23264 | 31 | -0.85428786 | IMPG2 | interphotoreceptor matrix proteoglycan 2 [Source:HGNC Symbol;Acc:HGNC:18362] |
| ENSG00000083535 | HGLibA_36519 | 31 | -0.85428786 | PIBF1 | progesterone immunomodulatory binding factor 1 [Source:HGNC Symbol;Acc:HGNC:23352] |
| ENSG00000085274 | HGLibA_30800 | 31 | -0.85428786 | MYNN | myoneurin [Source:HGNC Symbol;Acc:HGNC:14955] |
| ENSG00000086696 | HGLibA_22188 | 31 | -0.85428786 | HSD17B2 | hydroxysteroid 17-beta dehydrogenase 2 [Source:HGNC Symbol;Acc:HGNC:5211] |
| ENSG00000088247 | HGLibA_24582 | 31 | -0.85428786 | KHSRP | KH-type splicing regulatory protein [Source:HGNC Symbol;Acc:HGNC:6316] |
| ENSG00000089723 | HGLibA_34756 | 31 | -0.85428786 | OTUB2 | ubiquitin aldehyde binding 2 [Source:HGNC Symbol;Acc:HGNC:20351]" |
| ENSG00000090372 | HGLibA_47601 | 31 | -0.85428786 | STRN4 | striatin 4 [Source:HGNC Symbol;Acc:HGNC:15721] |
| ENSG00000091262 | HGLibA_00183 | 31 | -0.85428786 | ABCC6 | ATP binding cassette subfamily C member 6 [Source:HGNC Symbol;Acc:HGNC:57] |
| ENSG00000092758 | HGLibA_10625 | 31 | -0.85428786 | COL9A3 | collagen type IX alpha 3 chain [Source:HGNC Symbol;Acc:HGNC:2219] |
| ENSG00000094661 | HGLibA_33672 | 31 | -0.85428786 | OR1I1 | olfactory receptor family 1 subfamily I member 1 [Source:HGNC Symbol;Acc:HGNC:8207] |
| ENSG00000100324 | HGLibA_48140 | 31 | -0.85428786 | TAB1 | TGF-beta activated kinase 1 (MAP3K7) binding protein 1 [Source:HGNC Symbol;Acc:HGNC:18157] |
| ENSG00000100393 | HGLibA_15231 | 31 | -0.85428786 | EP300 | E1A binding protein p300 [Source:HGNC Symbol;Acc:HGNC:3373] |
| ENSG00000100567 | HGLibA_39077 | 31 | -0.85428786 | PSMA3 | proteasome subunit alpha 3 [Source:HGNC Symbol;Acc:HGNC:9532] |
| ENSG00000100979 | HGLibA_37245 | 31 | -0.85428786 | PLTP | phospholipid transfer protein [Source:HGNC Symbol;Acc:HGNC:9093] |
| ENSG00000101158 | HGLibA_31678 | 31 | -0.85428786 | NELFCD | negative elongation factor complex member C/D [Source:HGNC Symbol;Acc:HGNC:15934] |
| ENSG00000101443 | HGLibA_54263 | 31 | -0.85428786 | WFDC2 | WAP four-disulfide core domain 2 [Source:HGNC Symbol;Acc:HGNC:15939] |
| ENSG00000101542 | HGLibA_08663 | 31 | -0.85428786 | CDH20 | cadherin 20 [Source:HGNC Symbol;Acc:HGNC:1760] |
| ENSG00000102265 | HGLibA_49535 | 31 | -0.85428786 | TIMP1 | TIMP metallopeptidase inhibitor 1 [Source:HGNC Symbol;Acc:HGNC:11820] |
| ENSG00000102900 | HGLibA_33076 | 31 | -0.85428786 | NUP93 | nucleoporin 93 [Source:HGNC Symbol;Acc:HGNC:28958] |
| ENSG00000103769 | HGLibA_39787 | 31 | -0.85428786 | RAB11A | member RAS oncogene family [Source:HGNC Symbol;Acc:HGNC:9760]" |
| ENSG00000105325 | HGLibA_18291 | 31 | -0.85428786 | FZR1 | fizzy and cell division cycle 20 related 1 [Source:HGNC Symbol;Acc:HGNC:24824] |
| ENSG00000105549 | HGLibA_49335 | 31 | -0.85428786 | THEG | theg spermatid protein [Source:HGNC Symbol;Acc:HGNC:13706] |
| ENSG00000106113 | HGLibA_11143 | 31 | -0.85428786 | CRHR2 | corticotropin releasing hormone receptor 2 [Source:HGNC Symbol;Acc:HGNC:2358] |
| ENSG00000106348 | HGLibA_23257 | 31 | -0.85428786 | IMPDH1 | inosine monophosphate dehydrogenase 1 [Source:HGNC Symbol;Acc:HGNC:6052] |
| ENSG00000106536 | HGLibA_37798 | 31 | -0.85428786 | POU6F2 | POU class 6 homeobox 2 [Source:HGNC Symbol;Acc:HGNC:21694] |
| ENSG00000106829 | HGLibA_49615 | 31 | -0.85428786 | TLE4 | transducin like enhancer of split 4 [Source:HGNC Symbol;Acc:HGNC:11840] |
| ENSG00000106927 | HGLibA_01752 | 31 | -0.85428786 | AMBP | alpha-1-microglobulin/bikunin precursor [Source:HGNC Symbol;Acc:HGNC:453] |
| ENSG00000107771 | HGLibA_08193 | 31 | -0.85428786 | CCSER2 | coiled-coil serine rich protein 2 [Source:HGNC Symbol;Acc:HGNC:29197] |
| ENSG00000108384 | HGLibA_40091 | 31 | -0.85428786 | RAD51C | RAD51 paralog C [Source:HGNC Symbol;Acc:HGNC:9820] |
| ENSG00000108651 | HGLibA_53455 | 31 | -0.85428786 | UTP6 | small subunit processome component [Source:HGNC Symbol;Acc:HGNC:18279]" |
| ENSG00000109107 | HGLibA_01599 | 31 | -0.85428786 | ALDOC | fructose-bisphosphate C [Source:HGNC Symbol;Acc:HGNC:418]" |
| ENSG00000109332 | HGLibA_52587 | 31 | -0.85428786 | UBE2D3 | ubiquitin conjugating enzyme E2 D3 [Source:HGNC Symbol;Acc:HGNC:12476] |
| ENSG00000109511 | HGLibA_02221 | 31 | -0.85428786 | ANXA10 | annexin A10 [Source:HGNC Symbol;Acc:HGNC:534] |
| ENSG00000110108 | HGLibA_49869 | 31 | -0.85428786 | TMEM109 | transmembrane protein 109 [Source:HGNC Symbol;Acc:HGNC:28771] |
| ENSG00000111261 | HGLibA_28101 | 31 | -0.85428786 | MANSC1 | MANSC domain containing 1 [Source:HGNC Symbol;Acc:HGNC:25505] |
| ENSG00000111732 | HGLibA_01325 | 31 | -0.85428786 | AICDA | activation induced cytidine deaminase [Source:HGNC Symbol;Acc:HGNC:13203] |
| ENSG00000112079 | HGLibA_47502 | 31 | -0.85428786 | STK38 | serine/threonine kinase 38 [Source:HGNC Symbol;Acc:HGNC:17847] |
| ENSG00000112335 | HGLibA_46082 | 31 | -0.85428786 | SNX3 | sorting nexin 3 [Source:HGNC Symbol;Acc:HGNC:11174] |
| ENSG00000112769 | HGLibA_25951 | 31 | -0.85428786 | LAMA4 | laminin subunit alpha 4 [Source:HGNC Symbol;Acc:HGNC:6484] |
| ENSG00000113360 | HGLibA_13983 | 31 | -0.85428786 | DROSHA | drosha ribonuclease III [Source:HGNC Symbol;Acc:HGNC:17904] |
| ENSG00000114933 | HGLibA_23328 | 31 | -0.85428786 | INO80D | INO80 complex subunit D [Source:HGNC Symbol;Acc:HGNC:25997] |
| ENSG00000115828 | HGLibA_39727 | 31 | -0.85428786 | QPCT | glutaminyl-peptide cyclotransferase [Source:HGNC Symbol;Acc:HGNC:9753] |
| ENSG00000115919 | HGLibA_25882 | 31 | -0.85428786 | KYNU | kynureninase [Source:HGNC Symbol;Acc:HGNC:6469] |
| ENSG00000116141 | HGLibA_28363 | 31 | -0.85428786 | MARK1 | microtubule affinity regulating kinase 1 [Source:HGNC Symbol;Acc:HGNC:6896] |
| ENSG00000117226 | HGLibA_18734 | 31 | -0.85428786 | GBP3 | guanylate binding protein 3 [Source:HGNC Symbol;Acc:HGNC:4184] |
| ENSG00000117519 | HGLibA_10311 | 31 | -0.85428786 | CNN3 | calponin 3 [Source:HGNC Symbol;Acc:HGNC:2157] |
| ENSG00000117602 | HGLibA_40675 | 31 | -0.85428786 | RCAN3 | RCAN family member 3 [Source:HGNC Symbol;Acc:HGNC:3042] |
| ENSG00000118707 | HGLibA_49240 | 31 | -0.85428786 | TGIF2 | TGFB induced factor homeobox 2 [Source:HGNC Symbol;Acc:HGNC:15764] |
| ENSG00000118900 | HGLibA_52719 | 31 | -0.85428786 | UBN1 | ubinuclein 1 [Source:HGNC Symbol;Acc:HGNC:12506] |
| ENSG00000119778 | HGLibA_03408 | 31 | -0.85428786 | ATAD2B | AAA domain containing 2B [Source:HGNC Symbol;Acc:HGNC:29230]" |
| ENSG00000121481 | HGLibA_41608 | 31 | -0.85428786 | RNF2 | ring finger protein 2 [Source:HGNC Symbol;Acc:HGNC:10061] |
| ENSG00000122025 | HGLibA_17666 | 31 | -0.85428786 | FLT3 | fms related tyrosine kinase 3 [Source:HGNC Symbol;Acc:HGNC:3765] |
| ENSG00000122643 | HGLibA_32815 | 31 | -0.85428786 | NT5C3A | cytosolic IIIA [Source:HGNC Symbol;Acc:HGNC:17820]" |
| ENSG00000122870 | HGLibA_04490 | 31 | -0.85428786 | BICC1 | BicC family RNA binding protein 1 [Source:HGNC Symbol;Acc:HGNC:19351] |
| ENSG00000122970 | HGLibA_22775 | 31 | -0.85428786 | IFT81 | intraflagellar transport 81 [Source:HGNC Symbol;Acc:HGNC:14313] |
| ENSG00000123165 | HGLibA_00678 | 31 | -0.85428786 | ACTRT1 | actin related protein T1 [Source:HGNC Symbol;Acc:HGNC:24027] |
| ENSG00000123473 | HGLibA_47439 | 31 | -0.85428786 | STIL | centriolar assembly protein [Source:HGNC Symbol;Acc:HGNC:10879]" |
| ENSG00000123728 | HGLibA_40243 | 31 | -0.85428786 | RAP2C | member of RAS oncogene family [Source:HGNC Symbol;Acc:HGNC:21165]" |
| ENSG00000123810 | HGLibA_04047 | 31 | -0.85428786 | B9D2 | B9 domain containing 2 [Source:HGNC Symbol;Acc:HGNC:28636] |
| ENSG00000124191 | HGLibA_51039 | 31 | -0.85428786 | TOX2 | TOX high mobility group box family member 2 [Source:HGNC Symbol;Acc:HGNC:16095] |
| ENSG00000124678 | HGLibA_48841 | 31 | -0.85428786 | TCP11 | t-complex 11 [Source:HGNC Symbol;Acc:HGNC:11658] |
| ENSG00000125351 | HGLibA_53065 | 31 | -0.85428786 | UPF3B | regulator of nonsense mediated mRNA decay [Source:HGNC Symbol;Acc:HGNC:20439]" |
| ENSG00000125356 | HGLibA_31436 | 31 | -0.85428786 | NDUFA1 | NADH:ubiquinone oxidoreductase subunit A1 [Source:HGNC Symbol;Acc:HGNC:7683] |
| ENSG00000126215 | HGLibA_54588 | 31 | -0.85428786 | XRCC3 | X-ray repair cross complementing 3 [Source:HGNC Symbol;Acc:HGNC:12830] |
| ENSG00000127445 | HGLibA_36683 | 31 | -0.85428786 | PIN1 | NIMA-interacting 1 [Source:HGNC Symbol;Acc:HGNC:8988]" |
| ENSG00000129048 | HGLibA_00448 | 31 | -0.85428786 | ACKR4 | atypical chemokine receptor 4 [Source:HGNC Symbol;Acc:HGNC:1611] |
| ENSG00000130479 | HGLibA_28134 | 31 | -0.85428786 | MAP1S | microtubule associated protein 1S [Source:HGNC Symbol;Acc:HGNC:15715] |
| ENSG00000131094 | HGLibA_05923 | 31 | -0.85428786 | C1QL1 | complement C1q like 1 [Source:HGNC Symbol;Acc:HGNC:24182] |
| ENSG00000131171 | HGLibA_43915 | 31 | -0.85428786 | SH3BGRL | SH3 domain binding glutamate rich protein like [Source:HGNC Symbol;Acc:HGNC:10823] |
| ENSG00000131351 | HGLibA_20895 | 31 | -0.85428786 | HAUS8 | HAUS augmin like complex subunit 8 [Source:HGNC Symbol;Acc:HGNC:30532] |

| | | | | | |
|---|---|---|---|---|---|
| ENSG00000131374 | HGLibA_48559 | 31 | -0.85428786 | TBC1D5 | TBC1 domain family member 5 [Source:HGNC Symbol;Acc:HGNC:19166] |
| ENSG00000131697 | HGLibA_32452 | 31 | -0.85428786 | NPHP4 | nephrocystin 4 [Source:HGNC Symbol;Acc:HGNC:19104] |
| ENSG00000132570 | HGLibA_35363 | 31 | -0.85428786 | PCBD2 | pterin-4 alpha-carbinolamine dehydratase 2 [Source:HGNC Symbol;Acc:HGNC:24474] |
| ENSG00000132570 | HGLibA_35364 | 31 | -0.85428786 | PCBD2 | pterin-4 alpha-carbinolamine dehydratase 2 [Source:HGNC Symbol;Acc:HGNC:24474] |
| ENSG00000133055 | HGLibA_30659 | 31 | -0.85428786 | MYBPH | myosin binding protein H [Source:HGNC Symbol;Acc:HGNC:7552] |
| ENSG00000134046 | HGLibA_28465 | 31 | -0.85428786 | MBD2 | methyl-CpG binding domain protein 2 [Source:HGNC Symbol;Acc:HGNC:6917] |
| ENSG00000135334 | HGLibA_01459 | 31 | -0.85428786 | AKIRIN2 | akirin 2 [Source:HGNC Symbol;Acc:HGNC:21407] |
| ENSG00000135336 | HGLibA_34607 | 31 | -0.85428786 | ORC3 | origin recognition complex subunit 3 [Source:HGNC Symbol;Acc:HGNC:8489] |
| ENSG00000135457 | HGLibA_49144 | 31 | -0.85428786 | TFCP2 | transcription factor CP2 [Source:HGNC Symbol;Acc:HGNC:11748] |
| ENSG00000135828 | HGLibA_41440 | 31 | -0.85428786 | RNASEL | ribonuclease L [Source:HGNC Symbol;Acc:HGNC:10050] |
| ENSG00000135924 | HGLibA_13537 | 31 | -0.85428786 | DNAJB2 | DnaJ heat shock protein family (Hsp40) member B2 [Source:HGNC Symbol;Acc:HGNC:5228] |
| ENSG00000135931 | HGLibA_03036 | 31 | -0.85428786 | ARMC9 | armadillo repeat containing 9 [Source:HGNC Symbol;Acc:HGNC:20730] |
| ENSG00000136122 | HGLibA_04697 | 31 | -0.85428786 | BORA | aurora kinase A activator [Source:HGNC Symbol;Acc:HGNC:24724]" |
| ENSG00000136143 | HGLibA_47696 | 31 | -0.85428786 | SUCLA2 | succinate-CoA ligase ADP-forming beta subunit [Source:HGNC Symbol;Acc:HGNC:11448] |
| ENSG00000136682 | HGLibA_07434 | 31 | -0.85428786 | CBWD2 | COBW domain containing 2 [Source:HGNC Symbol;Acc:HGNC:17907] |
| ENSG00000136859 | HGLibA_01912 | 31 | -0.85428786 | ANGPTL2 | angiopoietin like 2 [Source:HGNC Symbol;Acc:HGNC:490] |
| ENSG00000136937 | HGLibA_31282 | 31 | -0.85428786 | NCBP1 | nuclear cap binding protein subunit 1 [Source:HGNC Symbol;Acc:HGNC:7658] |
| ENSG00000136943 | HGLibA_11731 | 31 | -0.85428786 | CTSV | cathepsin V [Source:HGNC Symbol;Acc:HGNC:2538] |
| ENSG00000137713 | HGLibA_38144 | 31 | -0.85428786 | PPP2R1B | protein phosphatase 2 scaffold subunit Abeta [Source:HGNC Symbol;Acc:HGNC:9303] |
| ENSG00000139133 | HGLibA_01605 | 31 | -0.85428786 | ALG10 | 2-glucosyltransferase [Source:HGNC Symbol;Acc:HGNC:23162]" |
| ENSG00000139289 | HGLibA_36416 | 31 | -0.85428786 | PHLDA1 | pleckstrin homology like domain family A member 1 [Source:HGNC Symbol;Acc:HGNC:8933] |
| ENSG00000140057 | HGLibA_01395 | 31 | -0.85428786 | AK7 | adenylate kinase 7 [Source:HGNC Symbol;Acc:HGNC:20091] |
| ENSG00000142178 | HGLibA_44163 | 31 | -0.85428786 | SIK1 | salt inducible kinase 1 [Source:HGNC Symbol;Acc:HGNC:11142] |
| ENSG00000142405 | HGLibA_32105 | 31 | -0.85428786 | NLRP12 | NLR family pyrin domain containing 12 [Source:HGNC Symbol;Acc:HGNC:22938] |
| ENSG00000143258 | HGLibA_53303 | 31 | -0.85428786 | USP21 | ubiquitin specific peptidase 21 [Source:HGNC Symbol;Acc:HGNC:12620] |
| ENSG00000144481 | HGLibA_51774 | 31 | -0.85428786 | TRPM8 | transient receptor potential cation channel subfamily M member 8 [Source:HGNC Symbol;Acc:HGNC:17961] |
| ENSG00000144554 | HGLibA_16840 | 31 | -0.85428786 | FANCD2 | FA complementation group D2 [Source:HGNC Symbol;Acc:HGNC:3585] |
| ENSG00000144935 | HGLibA_51729 | 31 | -0.85428786 | TRPC1 | transient receptor potential cation channel subfamily C member 1 [Source:HGNC Symbol;Acc:HGNC:12333] |
| ENSG00000145348 | HGLibA_48598 | 31 | -0.85428786 | TBCK | TBC1 domain containing kinase [Source:HGNC Symbol;Acc:HGNC:28261] |
| ENSG00000146352 | HGLibA_10171 | 31 | -0.85428786 | CLVS2 | clavesin 2 [Source:HGNC Symbol;Acc:HGNC:23046] |
| ENSG00000147454 | HGLibA_44722 | 31 | -0.85428786 | SLC25A37 | solute carrier family 25 member 37 [Source:HGNC Symbol;Acc:HGNC:29786] |
| ENSG00000147526 | HGLibA_48158 | 31 | -0.85428786 | TACC1 | transforming acidic coiled-coil containing protein 1 [Source:HGNC Symbol;Acc:HGNC:11522] |
| ENSG00000147571 | HGLibA_11134 | 31 | -0.85428786 | CRH | corticotropin releasing hormone [Source:HGNC Symbol;Acc:HGNC:2355] |
| ENSG00000148297 | HGLibA_28779 | 31 | -0.85428786 | MED22 | mediator complex subunit 22 [Source:HGNC Symbol;Acc:HGNC:11477] |
| ENSG00000148334 | HGLibA_39327 | 31 | -0.85428786 | PTGES2 | prostaglandin E synthase 2 [Source:HGNC Symbol;Acc:HGNC:17822] |
| ENSG00000148735 | HGLibA_37156 | 31 | -0.85428786 | PLEKHS1 | pleckstrin homology domain containing S1 [Source:HGNC Symbol;Acc:HGNC:26285] |
| ENSG00000152093 | HGLibA_09251 | 31 | -0.85428786 | CFC1B | cryptic family 1B [Source:HGNC Symbol;Acc:HGNC:33983]" |
| ENSG00000152377 | HGLibA_46747 | 31 | -0.85428786 | SPOCK1 | cwcv and kazal like domains proteoglycan 1 [Source:HGNC Symbol;Acc:HGNC:11251]" |
| ENSG00000153046 | HGLibA_08917 | 31 | -0.85428786 | CDYL | chromodomain Y like [Source:HGNC Symbol;Acc:HGNC:1811] |
| ENSG00000153291 | HGLibA_44691 | 31 | -0.85428786 | SLC25A27 | solute carrier family 25 member 27 [Source:HGNC Symbol;Acc:HGNC:21065] |
| ENSG00000154269 | HGLibA_15172 | 31 | -0.85428786 | ENPP3 | ectonucleotide pyrophosphatase/phosphodiesterase 3 [Source:HGNC Symbol;Acc:HGNC:3358] |
| ENSG00000154822 | HGLibA_37018 | 31 | -0.85428786 | PLCL2 | phospholipase C like 2 [Source:HGNC Symbol;Acc:HGNC:9064] |
| ENSG00000156097 | HGLibA_20061 | 31 | -0.85428786 | GPR61 | G protein-coupled receptor 61 [Source:HGNC Symbol;Acc:HGNC:13300] |
| ENSG00000156232 | HGLibA_54288 | 31 | -0.85428786 | WHAMM | golgi membranes and microtubules [Source:HGNC Symbol;Acc:HGNC:30493]" |
| ENSG00000158458 | HGLibA_32680 | 31 | -0.85428786 | NRG2 | neuregulin 2 [Source:HGNC Symbol;Acc:HGNC:7998] |
| ENSG00000158769 | HGLibA_15846 | 31 | -0.85428786 | F11R | F11 receptor [Source:HGNC Symbol;Acc:HGNC:14685] |
| ENSG00000158865 | HGLibA_45259 | 31 | -0.85428786 | SLC5A11 | solute carrier family 5 member 11 [Source:HGNC Symbol;Acc:HGNC:23091] |
| ENSG00000160013 | HGLibA_39347 | 31 | -0.85428786 | PTGIR | prostaglandin I2 receptor [Source:HGNC Symbol;Acc:HGNC:9602] |
| ENSG00000160062 | HGLibA_54924 | 31 | -0.85428786 | ZBTB8A | zinc finger and BTB domain containing 8A [Source:HGNC Symbol;Acc:HGNC:24172] |
| ENSG00000160325 | HGLibA_06870 | 31 | -0.85428786 | CACFD1 | calcium channel flower domain containing 1 [Source:HGNC Symbol;Acc:HGNC:1365] |
| ENSG00000160339 | HGLibA_17220 | 31 | -0.85428786 | FCN2 | ficolin 2 [Source:HGNC Symbol;Acc:HGNC:3624] |
| ENSG00000160345 | HGLibA_06665 | 31 | -0.85428786 | C9orf116 | chromosome 9 open reading frame 116 [Source:HGNC Symbol;Acc:HGNC:28435] |
| ENSG00000160633 | HGLibA_42634 | 31 | -0.85428786 | SAFB | scaffold attachment factor B [Source:HGNC Symbol;Acc:HGNC:10520] |
| ENSG00000160767 | HGLibA_16357 | 31 | -0.85428786 | FAM189B | family with sequence similarity 189 member B [Source:HGNC Symbol;Acc:HGNC:1233] |
| ENSG00000160883 | HGLibA_21584 | 31 | -0.85428786 | HK3 | hexokinase 3 [Source:HGNC Symbol;Acc:HGNC:4925] |
| ENSG00000161204 | HGLibA_00211 | 31 | -0.85428786 | ABCF3 | ATP binding cassette subfamily F member 3 [Source:HGNC Symbol;Acc:HGNC:72] |
| ENSG00000161958 | HGLibA_17365 | 31 | -0.85428786 | FGF11 | fibroblast growth factor 11 [Source:HGNC Symbol;Acc:HGNC:3667] |
| ENSG00000163626 | HGLibA_10820 | 31 | -0.85428786 | COX18 | cytochrome c oxidase assembly factor COX18 [Source:HGNC Symbol;Acc:HGNC:26801] |
| ENSG00000163681 | HGLibA_45533 | 31 | -0.85428786 | SLMAP | sarcolemma associated protein [Source:HGNC Symbol;Acc:HGNC:16643] |
| ENSG00000163930 | HGLibA_04152 | 31 | -0.85428786 | BAP1 | BRCA1 associated protein 1 [Source:HGNC Symbol;Acc:HGNC:950] |
| ENSG00000164011 | HGLibA_56552 | 31 | -0.85428786 | ZNF691 | zinc finger protein 691 [Source:HGNC Symbol;Acc:HGNC:28028] |
| ENSG00000164037 | HGLibA_45432 | 31 | -0.85428786 | SLC9B1 | solute carrier family 9 member B1 [Source:HGNC Symbol;Acc:HGNC:24244] |
| ENSG00000164256 | HGLibA_38405 | 31 | -0.85428786 | PRDM9 | PR/SET domain 9 [Source:HGNC Symbol;Acc:HGNC:13994] |
| ENSG00000164746 | HGLibA_06559 | 31 | -0.85428786 | C7orf57 | chromosome 7 open reading frame 57 [Source:HGNC Symbol;Acc:HGNC:22247] |
| ENSG00000164751 | HGLibA_36108 | 31 | -0.85428786 | PEX2 | peroxisomal biogenesis factor 2 [Source:HGNC Symbol;Acc:HGNC:9717] |
| ENSG00000164761 | HGLibA_50749 | 31 | -0.85428786 | TNFRSF11B | TNF receptor superfamily member 11b [Source:HGNC Symbol;Acc:HGNC:11909] |
| ENSG00000166426 | HGLibA_11055 | 31 | -0.85428786 | CRABP1 | cellular retinoic acid binding protein 1 [Source:HGNC Symbol;Acc:HGNC:2338] |
| ENSG00000166855 | HGLibA_10118 | 31 | -0.85428786 | CLPX | caseinolytic mitochondrial matrix peptidase chaperone subunit [Source:HGNC Symbol;Acc:HGNC:2088] |
| ENSG00000167085 | HGLibA_36313 | 31 | -0.85428786 | PHB | prohibitin [Source:HGNC Symbol;Acc:HGNC:8912] |
| ENSG00000167216 | HGLibA_24077 | 31 | -0.85428786 | KATNAL2 | katanin catalytic subunit A1 like 2 [Source:HGNC Symbol;Acc:HGNC:25387] |
| ENSG00000167272 | HGLibA_37693 | 31 | -0.85428786 | POP5 | ribonuclease P/MRP subunit [Source:HGNC Symbol;Acc:HGNC:17689]" |
| ENSG00000167754 | HGLibA_25262 | 31 | -0.85428786 | KLK5 | kallikrein related peptidase 5 [Source:HGNC Symbol;Acc:HGNC:6366] |
| ENSG00000169242 | HGLibA_14583 | 31 | -0.85428786 | EFNA1 | ephrin A1 [Source:HGNC Symbol;Acc:HGNC:3221] |
| ENSG00000169252 | HGLibA_01059 | 31 | -0.85428786 | ADRB2 | adrenoceptor beta 2 [Source:HGNC Symbol;Acc:HGNC:286] |
| ENSG00000169562 | HGLibA_19157 | 31 | -0.85428786 | GJB1 | gap junction protein beta 1 [Source:HGNC Symbol;Acc:HGNC:4283] |
| ENSG00000170011 | HGLibA_30920 | 31 | -0.85428786 | MYRIP | myosin VIIA and Rab interacting protein [Source:HGNC Symbol;Acc:HGNC:19156] |
| ENSG00000170271 | HGLibA_16931 | 31 | -0.85428786 | FAXDC2 | fatty acid hydroxylase domain containing 2 [Source:HGNC Symbol;Acc:HGNC:1334] |
| ENSG00000170498 | HGLibA_25010 | 31 | -0.85428786 | KISS1 | KiSS-1 metastasis suppressor [Source:HGNC Symbol;Acc:HGNC:6341] |
| ENSG00000170540 | HGLibA_02988 | 31 | -0.85428786 | ARL6IP1 | ADP ribosylation factor like GTPase 6 interacting protein 1 [Source:HGNC Symbol;Acc:HGNC:697] |
| ENSG00000171475 | HGLibA_54307 | 31 | -0.85428786 | WIPF2 | WAS/WASL interacting protein family member 2 [Source:HGNC Symbol;Acc:HGNC:30923] |
| ENSG00000171612 | HGLibA_44712 | 31 | -0.85428786 | SLC25A33 | solute carrier family 25 member 33 [Source:HGNC Symbol;Acc:HGNC:29681] |
| ENSG00000172469 | HGLibA_28091 | 31 | -0.85428786 | MANEA | mannosidase endo-alpha [Source:HGNC Symbol;Acc:HGNC:21072] |
| ENSG00000172932 | HGLibA_02008 | 31 | -0.85428786 | ANKRD13D | ankyrin repeat domain 13D [Source:HGNC Symbol;Acc:HGNC:27880] |
| ENSG00000173285 | HGLibA_33530 | 31 | -0.85428786 | OR10K1 | olfactory receptor family 10 subfamily K member 1 [Source:HGNC Symbol;Acc:HGNC:14693] |
| ENSG00000173660 | HGLibA_53123 | 31 | -0.85428786 | UQCRH | ubiquinol-cytochrome c reductase hinge protein [Source:HGNC Symbol;Acc:HGNC:12590] |
| ENSG00000173705 | HGLibA_47856 | 31 | -0.85428786 | SUSD5 | sushi domain containing 5 [Source:HGNC Symbol;Acc:HGNC:29061] |
| ENSG00000173715 | HGLibA_05188 | 31 | -0.85428786 | C11orf80 | chromosome 11 open reading frame 80 [Source:HGNC Symbol;Acc:HGNC:26197] |
| ENSG00000176055 | HGLibA_28505 | 31 | -0.85428786 | MBLAC2 | metallo-beta-lactamase domain containing 2 [Source:HGNC Symbol;Acc:HGNC:33711] |
| ENSG00000176720 | HGLibA_04678 | 31 | -0.85428786 | BOK | BCL2 family apoptosis regulator BOK [Source:HGNC Symbol;Acc:HGNC:1087] |
| ENSG00000176979 | HGLibA_51565 | 31 | -0.85428786 | TRIM60 | tripartite motif containing 60 [Source:HGNC Symbol;Acc:HGNC:21162] |
| ENSG00000177151 | HGLibA_33881 | 31 | -0.85428786 | OR2T35 | olfactory receptor family 2 subfamily T member 35 [Source:HGNC Symbol;Acc:HGNC:31257] |
| ENSG00000177363 | HGLibA_27482 | 31 | -0.85428786 | LRRN4CL | LRRN4 C-terminal like [Source:HGNC Symbol;Acc:HGNC:33724] |
| ENSG00000178394 | HGLibA_22355 | 31 | -0.85428786 | HTR1A | 5-hydroxytryptamine receptor 1A [Source:HGNC Symbol;Acc:HGNC:5286] |
| ENSG00000179152 | HGLibA_48689 | 31 | -0.85428786 | TCAIM | mitochondrial [Source:HGNC Symbol;Acc:HGNC:25241]" |
| ENSG00000179335 | HGLibA_10061 | 31 | -0.85428786 | CLK3 | CDC like kinase 3 [Source:HGNC Symbol;Acc:HGNC:2071] |
| ENSG00000179774 | HGLibA_03536 | 31 | -0.85428786 | ATOH7 | atonal bHLH transcription factor 7 [Source:HGNC Symbol;Acc:HGNC:13907] |
| ENSG00000179941 | HGLibA_04210 | 31 | -0.85428786 | BBS10 | Bardet-Biedl syndrome 10 [Source:HGNC Symbol;Acc:HGNC:26291] |
| ENSG00000182533 | HGLibA_07383 | 31 | -0.85428786 | CAV3 | caveolin 3 [Source:HGNC Symbol;Acc:HGNC:1529] |
| ENSG00000182798 | HGLibA_27939 | 31 | -0.85428786 | MAGEB17 | MAGE family member B17 [Source:HGNC Symbol;Acc:HGNC:17418] |
| ENSG00000184661 | HGLibA_08603 | 31 | -0.85428786 | CDCA2 | cell division cycle associated 2 [Source:HGNC Symbol;Acc:HGNC:14623] |
| ENSG00000185294 | HGLibA_46782 | 31 | -0.85428786 | SPPL2C | signal peptide peptidase like 2C [Source:HGNC Symbol;Acc:HGNC:28902] |
| ENSG00000185361 | HGLibA_50722 | 31 | -0.85428786 | TNFAIP8L1 | TNF alpha induced protein 8 like 1 [Source:HGNC Symbol;Acc:HGNC:28279] |
| ENSG00000185630 | HGLibA_35343 | 31 | -0.85428786 | PBX1 | PBX homeobox 1 [Source:HGNC Symbol;Acc:HGNC:8632] |
| ENSG00000185924 | HGLibA_42416 | 31 | -0.85428786 | RTN4RL1 | reticulon 4 receptor like 1 [Source:HGNC Symbol;Acc:HGNC:21329] |
| ENSG00000186660 | HGLibA_55244 | 31 | -0.85428786 | ZFP91 | ZFP91 zinc finger protein [Source:HGNC Symbol;Acc:HGNC:14983] |
| ENSG00000187033 | HGLibA_42693 | 31 | -0.85428786 | SAMD7 | sterile alpha motif domain containing 7 [Source:HGNC Symbol;Acc:HGNC:25394] |
| ENSG00000187037 | HGLibA_19908 | 31 | -0.85428786 | GPR141 | G protein-coupled receptor 141 [Source:HGNC Symbol;Acc:HGNC:19997] |
| ENSG00000187741 | HGLibA_16830 | 31 | -0.85428786 | FANCA | FA complementation group A [Source:HGNC Symbol;Acc:HGNC:3582] |
| ENSG00000188266 | HGLibA_22455 | 31 | -0.85428786 | HYKK | hydroxylysine kinase [Source:HGNC Symbol;Acc:HGNC:34403] |
| ENSG00000188536 | HGLibA_20911 | 31 | -0.85428786 | HBA2 | hemoglobin subunit alpha 2 [Source:HGNC Symbol;Acc:HGNC:4824] |
| ENSG00000188763 | HGLibA_18286 | 31 | -0.85428786 | FZD9 | frizzled class receptor 9 [Source:HGNC Symbol;Acc:HGNC:4047] |
| ENSG00000189043 | HGLibA_31459 | 31 | -0.85428786 | NDUFA4 | mitochondrial complex associated [Source:HGNC Symbol;Acc:HGNC:7687]" |
| ENSG00000196247 | HGLibA_55440 | 31 | -0.85428786 | ZNF107 | zinc finger protein 107 [Source:HGNC Symbol;Acc:HGNC:12887] |
| ENSG00000197362 | HGLibA_56752 | 31 | -0.85428786 | ZNF786 | zinc finger protein 786 [Source:HGNC Symbol;Acc:HGNC:21806] |
| ENSG00000197587 | HGLibA_13396 | 31 | -0.85428786 | DMBX1 | diencephalon/mesencephalon homeobox 1 [Source:HGNC Symbol;Acc:HGNC:19026] |
| ENSG00000197653 | HGLibA_13471 | 31 | -0.85428786 | DNAH10 | dynein axonemal heavy chain 10 [Source:HGNC Symbol;Acc:HGNC:2941] |
| ENSG00000197779 | HGLibA_56799 | 31 | -0.85428786 | ZNF81 | zinc finger protein 81 [Source:HGNC Symbol;Acc:HGNC:13156] |
| ENSG00000198125 | HGLibA_28453 | 31 | -0.85428786 | MB | myoglobin [Source:HGNC Symbol;Acc:HGNC:6915] |
| ENSG00000198342 | HGLibA_56007 | 31 | -0.85428786 | ZNF442 | zinc finger protein 442 [Source:HGNC Symbol;Acc:HGNC:20877] |
| ENSG00000198498 | HGLibA_49733 | 31 | -0.85428786 | TMA16 | translation machinery associated 16 homolog [Source:HGNC Symbol;Acc:HGNC:25638] |
| ENSG00000203710 | HGLibA_11044 | 31 | -0.85428786 | CR1 | complement C3b/C4b receptor 1 (Knops blood group) [Source:HGNC Symbol;Acc:HGNC:2334] |
| ENSG00000204217 | HGLibA_04635 | 31 | -0.85428786 | BMPR2 | bone morphogenetic protein receptor type 2 [Source:HGNC Symbol;Acc:HGNC:1078] |
| ENSG00000204370 | HGLibA_43148 | 31 | -0.85428786 | SDHD | succinate dehydrogenase complex subunit D [Source:HGNC Symbol;Acc:HGNC:10683] |
| ENSG00000204449 | HGLibA_51529 | 31 | -0.85428786 | TRIM49C | tripartite motif containing 49C [Source:HGNC Symbol;Acc:HGNC:38877] |
| ENSG00000204572 | HGLibA_25784 | 31 | -0.85428786 | KRTAP5-10 | keratin associated protein 5-10 [Source:HGNC Symbol;Acc:HGNC:23605] |
| ENSG00000205030 | HGLibA_34324 | 31 | -0.85428786 | OR5L2 | olfactory receptor family 5 subfamily L member 2 [Source:HGNC Symbol;Acc:HGNC:8351] |
| ENSG00000205409 | HGLibA_34163 | 31 | -0.85428786 | OR52E6 | olfactory receptor family 52 subfamily E member 6 [Source:HGNC Symbol;Acc:HGNC:15215] |
| ENSG00000213366 | HGLibA_20476 | 31 | -0.85428786 | GSTM2 | glutathione S-transferase mu 2 [Source:HGNC Symbol;Acc:HGNC:4634] |
| ENSG00000214285 | HGLibA_32523 | 31 | -0.85428786 | NPS | neuropeptide S [Source:HGNC Symbol;Acc:HGNC:33940] |
| ENSG00000214518 | HGLibA_25690 | 31 | -0.85428786 | KRTAP2-2 | keratin associated protein 2-2 [Source:HGNC Symbol;Acc:HGNC:18905] |
| ENSG00000215305 | HGLibA_53743 | 31 | -0.85428786 | VPS16 | CORVET/HOPS core subunit [Source:HGNC Symbol;Acc:HGNC:14584]" |
| ENSG00000221813 | HGLibA_34372 | 31 | -0.85428786 | OR6B1 | olfactory receptor family 6 subfamily B member 1 [Source:HGNC Symbol;Acc:HGNC:8354] |
| ENSG00000221869 | HGLibA_08961 | 31 | -0.85428786 | CEBPD | CCAAT enhancer binding protein delta [Source:HGNC Symbol;Acc:HGNC:1835] |
| ENSG00000221882 | HGLibA_33918 | 31 | -0.85428786 | OR3A2 | olfactory receptor family 3 subfamily A member 2 [Source:HGNC Symbol;Acc:HGNC:8283] |
| ENSG00000234414 | HGLibA_40610 | 31 | -0.85428786 | RBMY1A1 | member A1 [Source:HGNC Symbol;Acc:HGNC:9912]" |

| | | | | | |
|---|---|---|---|---|---|
| ENSG00000235699 | HGLibA_11942 | 31 | -0.85428786 | CXorf51B | chromosome X open reading frame 51B [Source:HGNC Symbol;Acc:HGNC:42787] |
| ENSG00000240682 | HGLibA_23694 | 31 | -0.85428786 | ISY1 | ISY1 splicing factor homolog [Source:HGNC Symbol;Acc:HGNC:29201] |
| ENSG00000243480 | HGLibA_01851 | 31 | -0.85428786 | AMY2A | alpha 2A (pancreatic) [Source:HGNC Symbol;Acc:HGNC:477]" |
| ENSG00000244395 | HGLibA_40616 | 31 | -0.85428786 | RBMY1D | member D [Source:HGNC Symbol;Acc:HGNC:23915]" |
| ENSG00000248487 | HGLibA_00249 | 31 | -0.85428786 | ABHD14A | abhydrolase domain containing 14A [Source:HGNC Symbol;Acc:HGNC:24538] |
| ENSG00000253537 | HGLibA_35541 | 31 | -0.85428786 | PCDHGA7 | 7 [Source:HGNC Symbol;Acc:HGNC:8705]" |
| ENSG00000256870 | HGLibA_45284 | 31 | -0.85428786 | SLC5A8 | solute carrier family 5 member 8 [Source:HGNC Symbol;Acc:HGNC:19119] |
| ENSG00000258992 | HGLibA_51965 | 31 | -0.85428786 | TSPY1 | testis specific protein Y-linked 1 [Source:HGNC Symbol;Acc:HGNC:12381] |
| ENSG00000261115 | HGLibA_50083 | 31 | -0.85428786 | TMEM178B | transmembrane protein 178B [Source:HGNC Symbol;Acc:HGNC:44112] |
| ENSG00000273899 | HGLibA_32261 | 31 | -0.85428786 | NOL12 | nucleolar protein 12 [Source:HGNC Symbol;Acc:HGNC:28585] |
| ENSG00000278845 | HGLibA_29994 | 31 | -0.85428786 | MRPL45 | mitochondrial ribosomal protein L45 [Source:HGNC Symbol;Acc:HGNC:16651] |
| EPRS | HGLibA_15366 | 31 | -0.85428786 | EPRS | - |
| GIF | HGLibA_19060 | 31 | -0.85428786 | GIF | - |
| GTPBP5 | HGLibA_20612 | 31 | -0.85428786 | GTPBP5 | - |
| HIST1H2AC | HGLibA_21382 | 31 | -0.85428786 | HIST1H2AC | - |
| HIST1H2BB | HGLibA_21414 | 31 | -0.85428786 | HIST1H2BB | - |
| HIST1H2BD | HGLibA_21419 | 31 | -0.85428786 | HIST1H2BD | - |
| hsa-mir-103a-1 | HGLibA_57161 | 31 | -0.85428786 | hsa-mir-103a-1 | - |
| hsa-mir-128-1 | HGLibA_57573 | 31 | -0.85428786 | hsa-mir-128-1 | - |
| hsa-mir-17 | HGLibA_57954 | 31 | -0.85428786 | hsa-mir-17 | - |
| hsa-mir-188 | HGLibA_58015 | 31 | -0.85428786 | hsa-mir-188 | - |
| hsa-mir-196a-2 | HGLibA_58097 | 31 | -0.85428786 | hsa-mir-196a-2 | - |
| hsa-mir-223 | HGLibA_58302 | 31 | -0.85428786 | hsa-mir-223 | - |
| hsa-mir-28 | HGLibA_58384 | 31 | -0.85428786 | hsa-mir-28 | - |
| hsa-mir-3119-2 | HGLibA_58527 | 31 | -0.85428786 | hsa-mir-3119-2 | - |
| hsa-mir-3162 | HGLibA_58729 | 31 | -0.85428786 | hsa-mir-3162 | - |
| hsa-mir-3176 | HGLibA_58779 | 31 | -0.85428786 | hsa-mir-3176 | - |
| hsa-mir-337 | HGLibA_58999 | 31 | -0.85428786 | hsa-mir-337 | - |
| hsa-mir-361 | HGLibA_59068 | 31 | -0.85428786 | hsa-mir-361 | - |
| hsa-mir-3612 | HGLibA_59077 | 31 | -0.85428786 | hsa-mir-3612 | - |
| hsa-mir-3618 | HGLibA_59102 | 31 | -0.85428786 | hsa-mir-3618 | - |
| hsa-mir-377 | HGLibA_59423 | 31 | -0.85428786 | hsa-mir-377 | - |
| hsa-mir-4468 | HGLibA_60279 | 31 | -0.85428786 | hsa-mir-4468 | - |
| hsa-mir-450a-1 | HGLibA_60473 | 31 | -0.85428786 | hsa-mir-450a-1 | - |
| hsa-mir-4752 | HGLibA_61105 | 31 | -0.85428786 | hsa-mir-4752 | - |
| hsa-mir-4778 | HGLibA_61219 | 31 | -0.85428786 | hsa-mir-4778 | - |
| hsa-mir-5093 | HGLibA_61530 | 31 | -0.85428786 | hsa-mir-5093 | - |
| hsa-mir-520g | HGLibA_61757 | 31 | -0.85428786 | hsa-mir-520g | - |
| hsa-mir-554 | HGLibA_62133 | 31 | -0.85428786 | hsa-mir-554 | - |
| hsa-mir-5583-1 | HGLibA_62171 | 31 | -0.85428786 | hsa-mir-5583-1 | - |
| hsa-mir-6077-1 | HGLibA_62560 | 31 | -0.85428786 | hsa-mir-6077-1 | - |
| hsa-mir-663a | HGLibA_62985 | 31 | -0.85428786 | hsa-mir-663a | - |
| hsa-mir-6796 | HGLibA_63362 | 31 | -0.85428786 | hsa-mir-6796 | - |
| hsa-mir-6803 | HGLibA_63388 | 31 | -0.85428786 | hsa-mir-6803 | - |
| hsa-mir-6819 | HGLibA_63452 | 31 | -0.85428786 | hsa-mir-6819 | - |
| hsa-mir-877 | HGLibA_64219 | 31 | -0.85428786 | hsa-mir-877 | - |
| hsa-mir-944 | HGLibA_64363 | 31 | -0.85428786 | hsa-mir-944 | - |
| LOC100653515 | HGLibA_26877 | 31 | -0.85428786 | LOC100653515 | - |
| MTERFD3 | HGLibA_30347 | 31 | -0.85428786 | MTERFD3 | - |
| PION | HGLibA_36699 | 31 | -0.85428786 | PION | - |
| SGOL2 | HGLibA_43843 | 31 | -0.85428786 | SGOL2 | - |
| ZFP106 | HGLibA_55183 | 31 | -0.85428786 | ZFP106 | - |
| AGPHD1 | HGLibA_01239 | 30 | -0.863333203 | AGPHD1 | - |
| ATP5B | HGLibA_03641 | 30 | -0.863333203 | ATP5B | - |
| ATP5F1 | HGLibA_03653 | 30 | -0.863333203 | ATP5F1 | - |
| C10orf137 | HGLibA_05059 | 30 | -0.863333203 | C10orf137 | - |
| C17orf59 | HGLibA_05551 | 30 | -0.863333203 | C17orf59 | - |
| C1orf173 | HGLibA_05795 | 30 | -0.863333203 | C1orf173 | - |
| CSF2RA_Y | HGLibA_11332 | 30 | -0.863333203 | CSF2RA_Y | - |
| CXorf40A | HGLibA_11923 | 30 | -0.863333203 | CXorf40A | - |
| CYP3A7-CYP3AP1 | HGLibA_12166 | 30 | -0.863333203 | CYP3A7-CYP3AP1 | - |
| ENSG00000001460 | HGLibA_47557 | 30 | -0.863333203 | STPG1 | sperm tail PG-rich repeat containing 1 [Source:HGNC Symbol;Acc:HGNC:28070] |
| ENSG00000005075 | HGLibA_37590 | 30 | -0.863333203 | POLR2J | RNA polymerase II subunit J [Source:HGNC Symbol;Acc:HGNC:9197] |
| ENSG00000005884 | HGLibA_23729 | 30 | -0.863333203 | ITGA3 | integrin subunit alpha 3 [Source:HGNC Symbol;Acc:HGNC:6139] |
| ENSG00000010072 | HGLibA_46831 | 30 | -0.863333203 | SPRTN | SprT-like N-terminal domain [Source:HGNC Symbol;Acc:HGNC:25356] |
| ENSG00000011198 | HGLibA_00281 | 30 | -0.863333203 | ABHD5 | abhydrolase domain containing 5 [Source:HGNC Symbol;Acc:HGNC:21396] |
| ENSG00000014914 | HGLibA_30416 | 30 | -0.863333203 | MTMR11 | myotubularin related protein 11 [Source:HGNC Symbol;Acc:HGNC:24307] |
| ENSG00000015475 | HGLibA_04499 | 30 | -0.863333203 | BID | BH3 interacting domain death agonist [Source:HGNC Symbol;Acc:HGNC:1050] |
| ENSG00000042445 | HGLibA_40881 | 30 | -0.863333203 | RETSAT | retinol saturase [Source:HGNC Symbol;Acc:HGNC:25991] |
| ENSG00000052344 | HGLibA_38975 | 30 | -0.863333203 | PRSS8 | serine protease 8 [Source:HGNC Symbol;Acc:HGNC:9491] |
| ENSG00000054179 | HGLibA_15200 | 30 | -0.863333203 | ENTPD2 | ectonucleoside triphosphate diphosphohydrolase 2 [Source:HGNC Symbol;Acc:HGNC:3364] |
| ENSG00000058729 | HGLibA_41311 | 30 | -0.863333203 | RIOK2 | RIO kinase 2 [Source:HGNC Symbol;Acc:HGNC:18999] |
| ENSG00000065054 | HGLibA_45413 | 30 | -0.863333203 | SLC9A3R2 | SLC9A3 regulator 2 [Source:HGNC Symbol;Acc:HGNC:11076] |
| ENSG00000066027 | HGLibA_38169 | 30 | -0.863333203 | PPP2R5A | protein phosphatase 2 regulatory subunit B'alpha [Source:HGNC Symbol;Acc:HGNC:9309] |
| ENSG00000067533 | HGLibA_42271 | 30 | -0.863333203 | RRP15 | ribosomal RNA processing 15 homolog [Source:HGNC Symbol;Acc:HGNC:24255] |
| ENSG00000070915 | HGLibA_44362 | 30 | -0.863333203 | SLC12A3 | solute carrier family 12 member 3 [Source:HGNC Symbol;Acc:HGNC:10912] |
| ENSG00000070961 | HGLibA_03613 | 30 | -0.863333203 | ATP2B1 | ATPase plasma membrane Ca2+ transporting 1 [Source:HGNC Symbol;Acc:HGNC:814] |
| ENSG00000071203 | HGLibA_30134 | 30 | -0.863333203 | MS4A12 | membrane spanning 4-domains A12 [Source:HGNC Symbol;Acc:HGNC:13370] |
| ENSG00000073050 | HGLibA_54582 | 30 | -0.863333203 | XRCC1 | X-ray repair cross complementing 1 [Source:HGNC Symbol;Acc:HGNC:12828] |
| ENSG00000075407 | HGLibA_55886 | 30 | -0.863333203 | ZNF37A | zinc finger protein 37A [Source:HGNC Symbol;Acc:HGNC:13102] |
| ENSG00000077235 | HGLibA_20579 | 30 | -0.863333203 | GTF3C1 | general transcription factor IIIC subunit 1 [Source:HGNC Symbol;Acc:HGNC:4664] |
| ENSG00000078804 | HGLibA_51072 | 30 | -0.863333203 | TP53INP2 | tumor protein p53 inducible nuclear protein 2 [Source:HGNC Symbol;Acc:HGNC:16104] |
| ENSG00000079482 | HGLibA_33415 | 30 | -0.863333203 | OPHN1 | oligophrenin 1 [Source:HGNC Symbol;Acc:HGNC:8148] |
| ENSG00000080819 | HGLibA_10981 | 30 | -0.863333203 | CPOX | coproporphyrinogen oxidase [Source:HGNC Symbol;Acc:HGNC:2321] |
| ENSG00000088002 | HGLibA_47760 | 30 | -0.863333203 | SULT2B1 | sulfotransferase family 2B member 1 [Source:HGNC Symbol;Acc:HGNC:11459] |
| ENSG00000089177 | HGLibA_24869 | 30 | -0.863333203 | KIF16B | kinesin family member 16B [Source:HGNC Symbol;Acc:HGNC:15869] |
| ENSG00000089685 | HGLibA_04520 | 30 | -0.863333203 | BIRC5 | baculoviral IAP repeat containing 5 [Source:HGNC Symbol;Acc:HGNC:593] |
| ENSG00000091106 | HGLibA_32092 | 30 | -0.863333203 | NLRC4 | NLR family CARD domain containing 4 [Source:HGNC Symbol;Acc:HGNC:16412] |
| ENSG00000100024 | HGLibA_53052 | 30 | -0.863333203 | UPB1 | beta-ureidopropionase 1 [Source:HGNC Symbol;Acc:HGNC:16297] |
| ENSG00000101680 | HGLibA_25941 | 30 | -0.863333203 | LAMA1 | laminin subunit alpha 1 [Source:HGNC Symbol;Acc:HGNC:6481] |
| ENSG00000101811 | HGLibA_11485 | 30 | -0.863333203 | CSTF2 | cleavage stimulation factor subunit 2 [Source:HGNC Symbol;Acc:HGNC:2484] |
| ENSG00000101890 | HGLibA_20666 | 30 | -0.863333203 | GUCY2F | retinal [Source:HGNC Symbol;Acc:HGNC:4691]" |
| ENSG00000102218 | HGLibA_41794 | 30 | -0.863333203 | RP2 | ARL3 GTPase activating protein [Source:HGNC Symbol;Acc:HGNC:10274]" |
| ENSG00000102387 | HGLibA_48255 | 30 | -0.863333203 | TAF7L | TATA-box binding protein associated factor 7 like [Source:HGNC Symbol;Acc:HGNC:11548] |
| ENSG00000102606 | HGLibA_02877 | 30 | -0.863333203 | ARHGEF7 | Rho guanine nucleotide exchange factor 7 [Source:HGNC Symbol;Acc:HGNC:15607] |
| ENSG00000103534 | HGLibA_49760 | 30 | -0.863333203 | TMC5 | transmembrane channel like 5 [Source:HGNC Symbol;Acc:HGNC:22999] |
| ENSG00000104093 | HGLibA_13456 | 30 | -0.863333203 | DMXL2 | Dmx like 2 [Source:HGNC Symbol;Acc:HGNC:2938] |
| ENSG00000106086 | HGLibA_37082 | 30 | -0.863333203 | PLEKHA8 | pleckstrin homology domain containing A8 [Source:HGNC Symbol;Acc:HGNC:30037] |
| ENSG00000106617 | HGLibA_38514 | 30 | -0.863333203 | PRKAG2 | protein kinase AMP-activated non-catalytic subunit gamma 2 [Source:HGNC Symbol;Acc:HGNC:9386] |
| ENSG00000106809 | HGLibA_33326 | 30 | -0.863333203 | OGN | osteoglycin [Source:HGNC Symbol;Acc:HGNC:8126] |
| ENSG00000107021 | HGLibA_48475 | 30 | -0.863333203 | TBC1D13 | TBC1 domain family member 13 [Source:HGNC Symbol;Acc:HGNC:25571] |
| ENSG00000108518 | HGLibA_36173 | 30 | -0.863333203 | PFN1 | profilin 1 [Source:HGNC Symbol;Acc:HGNC:8881] |
| ENSG00000108700 | HGLibA_08041 | 30 | -0.863333203 | CCL8 | C-C motif chemokine ligand 8 [Source:HGNC Symbol;Acc:HGNC:10635] |
| ENSG00000109534 | HGLibA_18623 | 30 | -0.863333203 | GAR1 | GAR1 ribonucleoprotein [Source:HGNC Symbol;Acc:HGNC:14264] |
| ENSG00000112304 | HGLibA_00475 | 30 | -0.863333203 | ACOT13 | acyl-CoA thioesterase 13 [Source:HGNC Symbol;Acc:HGNC:20999] |
| ENSG00000113369 | HGLibA_03119 | 30 | -0.863333203 | ARRDC3 | arrestin domain containing 3 [Source:HGNC Symbol;Acc:HGNC:29263] |
| ENSG00000114670 | HGLibA_31643 | 30 | -0.863333203 | NEK11 | NIMA related kinase 11 [Source:HGNC Symbol;Acc:HGNC:18593] |
| ENSG00000115041 | HGLibA_24240 | 30 | -0.863333203 | KCNIP3 | potassium voltage-gated channel interacting protein 3 [Source:HGNC Symbol;Acc:HGNC:15523] |
| ENSG00000115392 | HGLibA_16856 | 30 | -0.863333203 | FANCL | FA complementation group L [Source:HGNC Symbol;Acc:HGNC:20748] |
| ENSG00000115904 | HGLibA_46200 | 30 | -0.863333203 | SOS1 | SOS Ras/Rac guanine nucleotide exchange factor 1 [Source:HGNC Symbol;Acc:HGNC:11187] |
| ENSG00000116285 | HGLibA_15534 | 30 | -0.863333203 | ERRFI1 | ERBB receptor feedback inhibitor 1 [Source:HGNC Symbol;Acc:HGNC:18185] |
| ENSG00000118434 | HGLibA_46323 | 30 | -0.863333203 | SPACA1 | sperm acrosome associated 1 [Source:HGNC Symbol;Acc:HGNC:14967] |
| ENSG00000119899 | HGLibA_44472 | 30 | -0.863333203 | SLC17A5 | solute carrier family 17 member 5 [Source:HGNC Symbol;Acc:HGNC:10933] |
| ENSG00000120913 | HGLibA_35909 | 30 | -0.863333203 | PDLIM2 | PDZ and LIM domain 2 [Source:HGNC Symbol;Acc:HGNC:13992] |
| ENSG00000121380 | HGLibA_04318 | 30 | -0.863333203 | BCL2L14 | BCL2 like 14 [Source:HGNC Symbol;Acc:HGNC:16657] |
| ENSG00000121454 | HGLibA_26455 | 30 | -0.863333203 | LHX4 | LIM homeobox 4 [Source:HGNC Symbol;Acc:HGNC:21734] |
| ENSG00000122140 | HGLibA_30060 | 30 | -0.863333203 | MRPS2 | mitochondrial ribosomal protein S2 [Source:HGNC Symbol;Acc:HGNC:14495] |
| ENSG00000122375 | HGLibA_33436 | 30 | -0.863333203 | OPN4 | opsin 4 [Source:HGNC Symbol;Acc:HGNC:14449] |
| ENSG00000122484 | HGLibA_41817 | 30 | -0.863333203 | RPAP2 | RNA polymerase II associated protein 2 [Source:HGNC Symbol;Acc:HGNC:25791] |
| ENSG00000123472 | HGLibA_03797 | 30 | -0.863333203 | ATPAF1 | ATP synthase mitochondrial F1 complex assembly factor 1 [Source:HGNC Symbol;Acc:HGNC:18803] |
| ENSG00000124380 | HGLibA_45946 | 30 | -0.863333203 | SNRNP27 | small nuclear ribonucleoprotein U4/U6.U5 subunit 27 [Source:HGNC Symbol;Acc:HGNC:30240] |
| ENSG00000124486 | HGLibA_53416 | 30 | -0.863333203 | USP9X | ubiquitin specific peptidase 9 X-linked [Source:HGNC Symbol;Acc:HGNC:12632] |
| ENSG00000125409 | HGLibA_48978 | 30 | -0.863333203 | TEKT3 | tektin 3 [Source:HGNC Symbol;Acc:HGNC:14293] |
| ENSG00000126215 | HGLibA_54590 | 30 | -0.863333203 | XRCC3 | X-ray repair cross complementing 3 [Source:HGNC Symbol;Acc:HGNC:12830] |
| ENSG00000126953 | HGLibA_49524 | 30 | -0.863333203 | TIMM8A | translocase of inner mitochondrial membrane 8A [Source:HGNC Symbol;Acc:HGNC:11817] |
| ENSG00000127311 | HGLibA_21111 | 30 | -0.863333203 | HELB | DNA helicase B [Source:HGNC Symbol;Acc:HGNC:17196] |
| ENSG00000127423 | HGLibA_03867 | 30 | -0.863333203 | AUNIP | aurora kinase A and ninein interacting protein [Source:HGNC Symbol;Acc:HGNC:28363] |
| ENSG00000128185 | HGLibA_13052 | 30 | -0.863333203 | DGCR6L | DiGeorge syndrome critical region gene 6 like [Source:HGNC Symbol;Acc:HGNC:18551] |
| ENSG00000129675 | HGLibA_02873 | 30 | -0.863333203 | ARHGEF6 | Rac/Cdc42 guanine nucleotide exchange factor 6 [Source:HGNC Symbol;Acc:HGNC:685] |
| ENSG00000130024 | HGLibA_36334 | 30 | -0.863333203 | PHF10 | PHD finger protein 10 [Source:HGNC Symbol;Acc:HGNC:18250] |
| ENSG00000130340 | HGLibA_46110 | 30 | -0.863333203 | SNX9 | sorting nexin 9 [Source:HGNC Symbol;Acc:HGNC:14973] |
| ENSG00000130768 | HGLibA_45819 | 30 | -0.863333203 | SMPDL3B | sphingomyelin phosphodiesterase acid like 3B [Source:HGNC Symbol;Acc:HGNC:21416] |
| ENSG00000130816 | HGLibA_13699 | 30 | -0.863333203 | DNMT1 | DNA methyltransferase 1 [Source:HGNC Symbol;Acc:HGNC:2976] |
| ENSG00000132837 | HGLibA_13406 | 30 | -0.863333203 | DMGDH | dimethylglycine dehydrogenase [Source:HGNC Symbol;Acc:HGNC:24475] |
| ENSG00000133030 | HGLibA_29777 | 30 | -0.863333203 | MPRIP | myosin phosphatase Rho interacting protein [Source:HGNC Symbol;Acc:HGNC:30321] |

| | | | | | |
|---|---|---|---|---|---|
| ENSG00000133422 | HGLibA_29650 | 30 | -0.863333203 | MORC2 | MORC family CW-type zinc finger 2 [Source:HGNC Symbol;Acc:HGNC:23573] |
| ENSG00000133574 | HGLibA_19080 | 30 | -0.863333203 | GIMAP4 | IMAP family member 4 [Source:HGNC Symbol;Acc:HGNC:21872]" |
| ENSG00000134216 | HGLibA_09441 | 30 | -0.863333203 | CHIA | acidic [Source:HGNC Symbol;Acc:HGNC:17432]" |
| ENSG00000134815 | HGLibA_13184 | 30 | -0.863333203 | DHX34 | DExH-box helicase 34 [Source:HGNC Symbol;Acc:HGNC:16719] |
| ENSG00000135336 | HGLibA_34608 | 30 | -0.863333203 | ORC3 | origin recognition complex subunit 3 [Source:HGNC Symbol;Acc:HGNC:8489] |
| ENSG00000135679 | HGLibA_28698 | 30 | -0.863333203 | MDM2 | MDM2 proto-oncogene [Source:HGNC Symbol;Acc:HGNC:6973] |
| ENSG00000135972 | HGLibA_30113 | 30 | -0.863333203 | MRPS9 | mitochondrial ribosomal protein S9 [Source:HGNC Symbol;Acc:HGNC:14501] |
| ENSG00000136152 | HGLibA_10475 | 30 | -0.863333203 | COG3 | component of oligomeric golgi complex 3 [Source:HGNC Symbol;Acc:HGNC:18619] |
| ENSG00000136205 | HGLibA_50926 | 30 | -0.863333203 | TNS3 | tensin 3 [Source:HGNC Symbol;Acc:HGNC:21616] |
| ENSG00000136710 | HGLibA_07554 | 30 | -0.863333203 | CCDC115 | coiled-coil domain containing 115 [Source:HGNC Symbol;Acc:HGNC:28178] |
| ENSG00000136933 | HGLibA_40006 | 30 | -0.863333203 | RABEPK | Rab9 effector protein with kelch motifs [Source:HGNC Symbol;Acc:HGNC:16896] |
| ENSG00000136986 | HGLibA_12995 | 30 | -0.863333203 | DERL1 | derlin 1 [Source:HGNC Symbol;Acc:HGNC:28454] |
| ENSG00000137040 | HGLibA_40209 | 30 | -0.863333203 | RANBP6 | RAN binding protein 6 [Source:HGNC Symbol;Acc:HGNC:9851] |
| ENSG00000137218 | HGLibA_18050 | 30 | -0.863333203 | FRS3 | fibroblast growth factor receptor substrate 3 [Source:HGNC Symbol;Acc:HGNC:16970] |
| ENSG00000137252 | HGLibA_20990 | 30 | -0.863333203 | HCRTR2 | hypocretin receptor 2 [Source:HGNC Symbol;Acc:HGNC:4849] |
| ENSG00000137496 | HGLibA_23024 | 30 | -0.863333203 | IL18BP | interleukin 18 binding protein [Source:HGNC Symbol;Acc:HGNC:5987] |
| ENSG00000138131 | HGLibA_27047 | 30 | -0.863333203 | LOXL4 | lysyl oxidase like 4 [Source:HGNC Symbol;Acc:HGNC:17171] |
| ENSG00000138382 | HGLibA_29013 | 30 | -0.863333203 | METTL5 | methyltransferase like 5 [Source:HGNC Symbol;Acc:HGNC:25006] |
| ENSG00000138755 | HGLibA_11878 | 30 | -0.863333203 | CXCL9 | C-X-C motif chemokine ligand 9 [Source:HGNC Symbol;Acc:HGNC:7098] |
| ENSG00000139178 | HGLibA_05973 | 30 | -0.863333203 | C1RL | complement C1r subcomponent like [Source:HGNC Symbol;Acc:HGNC:21265] |
| ENSG00000139178 | HGLibA_05971 | 30 | -0.863333203 | C1RL | complement C1r subcomponent like [Source:HGNC Symbol;Acc:HGNC:21265] |
| ENSG00000140848 | HGLibA_10957 | 30 | -0.863333203 | CPNE2 | copine 2 [Source:HGNC Symbol;Acc:HGNC:2315] |
| ENSG00000141933 | HGLibA_51124 | 30 | -0.863333203 | TPGS1 | tubulin polyglutamylase complex subunit 1 [Source:HGNC Symbol;Acc:HGNC:25058] |
| ENSG00000143105 | HGLibA_24126 | 30 | -0.863333203 | KCNA10 | potassium voltage-gated channel subfamily A member 10 [Source:HGNC Symbol;Acc:HGNC:6219] |
| ENSG00000143434 | HGLibA_43369 | 30 | -0.863333203 | SEMA6C | semaphorin 6C [Source:HGNC Symbol;Acc:HGNC:10740] |
| ENSG00000143436 | HGLibA_30025 | 30 | -0.863333203 | MRPL9 | mitochondrial ribosomal protein L9 [Source:HGNC Symbol;Acc:HGNC:14277] |
| ENSG00000143546 | HGLibA_42572 | 30 | -0.863333203 | S100A8 | S100 calcium binding protein A8 [Source:HGNC Symbol;Acc:HGNC:10498] |
| ENSG00000143578 | HGLibA_11101 | 30 | -0.863333203 | CREB3L4 | cAMP responsive element binding protein 3 like 4 [Source:HGNC Symbol;Acc:HGNC:18854] |
| ENSG00000143771 | HGLibA_10293 | 30 | -0.863333203 | CNIH4 | cornichon family AMPA receptor auxiliary protein 4 [Source:HGNC Symbol;Acc:HGNC:25013] |
| ENSG00000145244 | HGLibA_10771 | 30 | -0.863333203 | CORIN | serine peptidase [Source:HGNC Symbol;Acc:HGNC:19012]" |
| ENSG00000146112 | HGLibA_38063 | 30 | -0.863333203 | PPP1R18 | protein phosphatase 1 regulatory subunit 18 [Source:HGNC Symbol;Acc:HGNC:29413] |
| ENSG00000146587 | HGLibA_40418 | 30 | -0.863333203 | RBAK | RB associated KRAB zinc finger [Source:HGNC Symbol;Acc:HGNC:17680] |
| ENSG00000147130 | HGLibA_55396 | 30 | -0.863333203 | ZMYM3 | zinc finger MYM-type containing 3 [Source:HGNC Symbol;Acc:HGNC:13054] |
| ENSG00000148843 | HGLibA_35722 | 30 | -0.863333203 | PDCD11 | programmed cell death 11 [Source:HGNC Symbol;Acc:HGNC:13408] |
| ENSG00000149742 | HGLibA_44603 | 30 | -0.863333203 | SLC22A9 | solute carrier family 22 member 9 [Source:HGNC Symbol;Acc:HGNC:16261] |
| ENSG00000150394 | HGLibA_08693 | 30 | -0.863333203 | CDH8 | cadherin 8 [Source:HGNC Symbol;Acc:HGNC:1767] |
| ENSG00000151090 | HGLibA_49395 | 30 | -0.863333203 | THRB | thyroid hormone receptor beta [Source:HGNC Symbol;Acc:HGNC:11799] |
| ENSG00000151247 | HGLibA_14815 | 30 | -0.863333203 | EIF4E | eukaryotic translation initiation factor 4E [Source:HGNC Symbol;Acc:HGNC:3287] |
| ENSG00000151348 | HGLibA_15804 | 30 | -0.863333203 | EXT2 | exostosin glycosyltransferase 2 [Source:HGNC Symbol;Acc:HGNC:3513] |
| ENSG00000151388 | HGLibA_00797 | 30 | -0.863333203 | ADAMTS12 | ADAM metallopeptidase with thrombospondin type 1 motif 12 [Source:HGNC Symbol;Acc:HGNC:14605] |
| ENSG00000152822 | HGLibA_20341 | 30 | -0.863333203 | GRM1 | glutamate metabotropic receptor 1 [Source:HGNC Symbol;Acc:HGNC:4593] |
| ENSG00000153250 | HGLibA_40587 | 30 | -0.863333203 | RBMS1 | RNA binding motif single stranded interacting protein 1 [Source:HGNC Symbol;Acc:HGNC:9907] |
| ENSG00000154229 | HGLibA_38532 | 30 | -0.863333203 | PRKCA | protein kinase C alpha [Source:HGNC Symbol;Acc:HGNC:9393] |
| ENSG00000154229 | HGLibA_38534 | 30 | -0.863333203 | PRKCA | protein kinase C alpha [Source:HGNC Symbol;Acc:HGNC:9393] |
| ENSG00000154447 | HGLibA_43970 | 30 | -0.863333203 | SH3RF1 | SH3 domain containing ring finger 1 [Source:HGNC Symbol;Acc:HGNC:17650] |
| ENSG00000154928 | HGLibA_15311 | 30 | -0.863333203 | EPHB1 | EPH receptor B1 [Source:HGNC Symbol;Acc:HGNC:3392] |
| ENSG00000155506 | HGLibA_26027 | 30 | -0.863333203 | LARP1 | La ribonucleoprotein domain family member 1 [Source:HGNC Symbol;Acc:HGNC:29531] |
| ENSG00000155622 | HGLibA_54480 | 30 | -0.863333203 | XAGE2 | X antigen family member 2 [Source:HGNC Symbol;Acc:HGNC:4112] |
| ENSG00000155850 | HGLibA_44789 | 30 | -0.863333203 | SLC26A2 | solute carrier family 26 member 2 [Source:HGNC Symbol;Acc:HGNC:10994] |
| ENSG00000156515 | HGLibA_21578 | 30 | -0.863333203 | HK1 | hexokinase 1 [Source:HGNC Symbol;Acc:HGNC:4922] |
| ENSG00000156925 | HGLibA_55326 | 30 | -0.863333203 | ZIC3 | Zic family member 3 [Source:HGNC Symbol;Acc:HGNC:12874] |
| ENSG00000157150 | HGLibA_49544 | 30 | -0.863333203 | TIMP4 | TIMP metallopeptidase inhibitor 4 [Source:HGNC Symbol;Acc:HGNC:11823] |
| ENSG00000157193 | HGLibA_27233 | 30 | -0.863333203 | LRP8 | LDL receptor related protein 8 [Source:HGNC Symbol;Acc:HGNC:6700] |
| ENSG00000158089 | HGLibA_18542 | 30 | -0.863333203 | GALNT14 | polypeptide N-acetylgalactosaminyltransferase 14 [Source:HGNC Symbol;Acc:HGNC:22946] |
| ENSG00000158497 | HGLibA_21742 | 30 | -0.863333203 | HMHB1 | histocompatibility minor HB-1 [Source:HGNC Symbol;Acc:HGNC:29677] |
| ENSG00000158545 | HGLibA_54970 | 30 | -0.863333203 | ZC3H18 | zinc finger CCCH-type containing 18 [Source:HGNC Symbol;Acc:HGNC:25091] |
| ENSG00000158552 | HGLibA_55149 | 30 | -0.863333203 | ZFAND2B | zinc finger AN1-type containing 2B [Source:HGNC Symbol;Acc:HGNC:25206] |
| ENSG00000158786 | HGLibA_36893 | 30 | -0.863333203 | PLA2G2F | phospholipase A2 group IIF [Source:HGNC Symbol;Acc:HGNC:30040] |
| ENSG00000159173 | HGLibA_50872 | 30 | -0.863333203 | TNNI1 | slow skeletal type [Source:HGNC Symbol;Acc:HGNC:11945]" |
| ENSG00000160193 | HGLibA_54086 | 30 | -0.863333203 | WDR4 | WD repeat domain 4 [Source:HGNC Symbol;Acc:HGNC:12756] |
| ENSG00000160882 | HGLibA_12056 | 30 | -0.863333203 | CYP11B1 | cytochrome P450 family 11 subfamily B member 1 [Source:HGNC Symbol;Acc:HGNC:2591] |
| ENSG00000160959 | HGLibA_27254 | 30 | -0.863333203 | LRRC14 | leucine rich repeat containing 14 [Source:HGNC Symbol;Acc:HGNC:20419] |
| ENSG00000160959 | HGLibA_27253 | 30 | -0.863333203 | LRRC14 | leucine rich repeat containing 14 [Source:HGNC Symbol;Acc:HGNC:20419] |
| ENSG00000161791 | HGLibA_17698 | 30 | -0.863333203 | FMNL3 | formin like 3 [Source:HGNC Symbol;Acc:HGNC:23698] |
| ENSG00000161921 | HGLibA_11861 | 30 | -0.863333203 | CXCL16 | C-X-C motif chemokine ligand 16 [Source:HGNC Symbol;Acc:HGNC:16642] |
| ENSG00000162073 | HGLibA_35161 | 30 | -0.863333203 | PAQR4 | progestin and adipoQ receptor family member 4 [Source:HGNC Symbol;Acc:HGNC:26386] |
| ENSG00000162654 | HGLibA_18736 | 30 | -0.863333203 | GBP4 | guanylate binding protein 4 [Source:HGNC Symbol;Acc:HGNC:20480] |
| ENSG00000162779 | HGLibA_03917 | 30 | -0.863333203 | AXDND1 | axonemal dynein light chain domain containing 1 [Source:HGNC Symbol;Acc:HGNC:26564] |
| ENSG00000163032 | HGLibA_53854 | 30 | -0.863333203 | VSNL1 | visinin like 1 [Source:HGNC Symbol;Acc:HGNC:12722] |
| ENSG00000163125 | HGLibA_42049 | 30 | -0.863333203 | RPRD2 | regulation of nuclear pre-mRNA domain containing 2 [Source:HGNC Symbol;Acc:HGNC:29039] |
| ENSG00000163464 | HGLibA_11882 | 30 | -0.863333203 | CXCR1 | C-X-C motif chemokine receptor 1 [Source:HGNC Symbol;Acc:HGNC:6026] |
| ENSG00000164073 | HGLibA_29117 | 30 | -0.863333203 | MFSD8 | major facilitator superfamily domain containing 8 [Source:HGNC Symbol;Acc:HGNC:28486] |
| ENSG00000164091 | HGLibA_54190 | 30 | -0.863333203 | WDR82 | WD repeat domain 82 [Source:HGNC Symbol;Acc:HGNC:28826] |
| ENSG00000164128 | HGLibA_32550 | 30 | -0.863333203 | NPY1R | neuropeptide Y receptor Y1 [Source:HGNC Symbol;Acc:HGNC:7956] |
| ENSG00000164162 | HGLibA_01865 | 30 | -0.863333203 | ANAPC10 | anaphase promoting complex subunit 10 [Source:HGNC Symbol;Acc:HGNC:24077] |
| ENSG00000164291 | HGLibA_03154 | 30 | -0.863333203 | ARSK | arylsulfatase family member K [Source:HGNC Symbol;Acc:HGNC:25239] |
| ENSG00000164399 | HGLibA_23126 | 30 | -0.863333203 | IL3 | interleukin 3 [Source:HGNC Symbol;Acc:HGNC:6011] |
| ENSG00000164683 | HGLibA_21216 | 30 | -0.863333203 | HEY1 | hes related family bHLH transcription factor with YRPW motif 1 [Source:HGNC Symbol;Acc:HGNC:4880] |
| ENSG00000164880 | HGLibA_23406 | 30 | -0.863333203 | INTS1 | integrator complex subunit 1 [Source:HGNC Symbol;Acc:HGNC:24555] |
| ENSG00000165259 | HGLibA_21064 | 30 | -0.863333203 | HDX | highly divergent homeobox [Source:HGNC Symbol;Acc:HGNC:26411] |
| ENSG00000165457 | HGLibA_17786 | 30 | -0.863333203 | FOLR2 | folate receptor beta [Source:HGNC Symbol;Acc:HGNC:3793] |
| ENSG00000165643 | HGLibA_46163 | 30 | -0.863333203 | SOHLH1 | spermatogenesis and oogenesis specific basic helix-loop-helix 1 [Source:HGNC Symbol;Acc:HGNC:27845] |
| ENSG00000165807 | HGLibA_38093 | 30 | -0.863333203 | PPP1R36 | protein phosphatase 1 regulatory subunit 36 [Source:HGNC Symbol;Acc:HGNC:20097] |
| ENSG00000166136 | HGLibA_31531 | 30 | -0.863333203 | NDUFB8 | NADH:ubiquinone oxidoreductase subunit B8 [Source:HGNC Symbol;Acc:HGNC:7703] |
| ENSG00000166435 | HGLibA_54611 | 30 | -0.863333203 | XRRA1 | X-ray radiation resistance associated 1 [Source:HGNC Symbol;Acc:HGNC:18868] |
| ENSG00000166575 | HGLibA_49950 | 30 | -0.863333203 | TMEM135 | transmembrane protein 135 [Source:HGNC Symbol;Acc:HGNC:26167] |
| ENSG00000166819 | HGLibA_37171 | 30 | -0.863333203 | PLIN1 | perilipin 1 [Source:HGNC Symbol;Acc:HGNC:9076] |
| ENSG00000167766 | HGLibA_56830 | 30 | -0.863333203 | ZNF83 | zinc finger protein 83 [Source:HGNC Symbol;Acc:HGNC:13158] |
| ENSG00000168092 | HGLibA_34993 | 30 | -0.863333203 | PAFAH1B2 | platelet activating factor acetylhydrolase 1b catalytic subunit 2 [Source:HGNC Symbol;Acc:HGNC:8575] |
| ENSG00000168883 | HGLibA_53357 | 30 | -0.863333203 | USP39 | ubiquitin specific peptidase 39 [Source:HGNC Symbol;Acc:HGNC:20071] |
| ENSG00000169972 | HGLibA_39611 | 30 | -0.863333203 | PUSL1 | pseudouridine synthase like 1 [Source:HGNC Symbol;Acc:HGNC:26914] |
| ENSG00000169994 | HGLibA_30865 | 30 | -0.863333203 | MYO7B | myosin VIIB [Source:HGNC Symbol;Acc:HGNC:7607] |
| ENSG00000170889 | HGLibA_42194 | 30 | -0.863333203 | RPS9 | ribosomal protein S9 [Source:HGNC Symbol;Acc:HGNC:10442] |
| ENSG00000170921 | HGLibA_48295 | 30 | -0.863333203 | TANC2 | ankyrin repeat and coiled-coil containing 2 [Source:HGNC Symbol;Acc:HGNC:30212]" |
| ENSG00000171564 | HGLibA_17339 | 30 | -0.863333203 | FGB | fibrinogen beta chain [Source:HGNC Symbol;Acc:HGNC:3662] |
| ENSG00000171804 | HGLibA_54206 | 30 | -0.863333203 | WDR87 | WD repeat domain 87 [Source:HGNC Symbol;Acc:HGNC:29934] |
| ENSG00000172209 | HGLibA_20002 | 30 | -0.863333203 | GPR22 | G protein-coupled receptor 22 [Source:HGNC Symbol;Acc:HGNC:4477] |
| ENSG00000172260 | HGLibA_31627 | 30 | -0.863333203 | NEGR1 | neuronal growth regulator 1 [Source:HGNC Symbol;Acc:HGNC:17302] |
| ENSG00000172465 | HGLibA_48703 | 30 | -0.863333203 | TCEAL1 | transcription elongation factor A like 1 [Source:HGNC Symbol;Acc:HGNC:11616] |
| ENSG00000172738 | HGLibA_50225 | 30 | -0.863333203 | TMEM217 | transmembrane protein 217 [Source:HGNC Symbol;Acc:HGNC:21238] |
| ENSG00000173744 | HGLibA_01183 | 30 | -0.863333203 | AGFG1 | ArfGAP with FG repeats 1 [Source:HGNC Symbol;Acc:HGNC:5175] |
| ENSG00000173894 | HGLibA_07447 | 30 | -0.863333203 | CBX2 | chromobox 2 [Source:HGNC Symbol;Acc:HGNC:1552] |
| ENSG00000174136 | HGLibA_41016 | 30 | -0.863333203 | RGMB | repulsive guidance molecule BMP co-receptor b [Source:HGNC Symbol;Acc:HGNC:26896] |
| ENSG00000175054 | HGLibA_03809 | 30 | -0.863333203 | ATR | ATR serine/threonine kinase [Source:HGNC Symbol;Acc:HGNC:882] |
| ENSG00000175106 | HGLibA_52362 | 30 | -0.863333203 | TVP23C | trans-golgi network vesicle protein 23 homolog C [Source:HGNC Symbol;Acc:HGNC:30453] |
| ENSG00000175166 | HGLibA_39165 | 30 | -0.863333203 | PSMD2 | non-ATPase 2 [Source:HGNC Symbol;Acc:HGNC:9559]" |
| ENSG00000175806 | HGLibA_30239 | 30 | -0.863333203 | MSRA | methionine sulfoxide reductase A [Source:HGNC Symbol;Acc:HGNC:7377] |
| ENSG00000176208 | HGLibA_03419 | 30 | -0.863333203 | ATAD5 | AAA domain containing 5 [Source:HGNC Symbol;Acc:HGNC:25752]" |
| ENSG00000177030 | HGLibA_12768 | 30 | -0.863333203 | DEAF1 | DEAF1 transcription factor [Source:HGNC Symbol;Acc:HGNC:14677] |
| ENSG00000178257 | HGLibA_38613 | 30 | -0.863333203 | PRM3 | protamine 3 [Source:HGNC Symbol;Acc:HGNC:13732] |
| ENSG00000179010 | HGLibA_29829 | 30 | -0.863333203 | MRFAP1 | Morf4 family associated protein 1 [Source:HGNC Symbol;Acc:HGNC:24549] |
| ENSG00000179915 | HGLibA_32733 | 30 | -0.863333203 | NRXN1 | neurexin 1 [Source:HGNC Symbol;Acc:HGNC:8008] |
| ENSG00000180176 | HGLibA_49281 | 30 | -0.863333203 | TH | tyrosine hydroxylase [Source:HGNC Symbol;Acc:HGNC:11782] |
| ENSG00000180209 | HGLibA_30796 | 30 | -0.863333203 | MYLPF | fast skeletal muscle [Source:HGNC Symbol;Acc:HGNC:29824]" |
| ENSG00000180245 | HGLibA_42248 | 30 | -0.863333203 | RRH | retinal pigment epithelium-derived rhodopsin homolog [Source:HGNC Symbol;Acc:HGNC:10450] |
| ENSG00000181852 | HGLibA_41683 | 30 | -0.863333203 | RNF41 | ring finger protein 41 [Source:HGNC Symbol;Acc:HGNC:18401] |
| ENSG00000182035 | HGLibA_00973 | 30 | -0.863333203 | ADIG | adipogenin [Source:HGNC Symbol;Acc:HGNC:28606] |
| ENSG00000182256 | HGLibA_18394 | 30 | -0.863333203 | GABRG3 | gamma-aminobutyric acid type A receptor gamma3 subunit [Source:HGNC Symbol;Acc:HGNC:4088] |
| ENSG00000182645 | HGLibA_07696 | 30 | -0.863333203 | CCDC172 | coiled-coil domain containing 172 [Source:HGNC Symbol;Acc:HGNC:30524] |
| ENSG00000182670 | HGLibA_52103 | 30 | -0.863333203 | TTC3 | tetratricopeptide repeat domain 3 [Source:HGNC Symbol;Acc:HGNC:12393] |
| ENSG00000182732 | HGLibA_41100 | 30 | -0.863333203 | RGS6 | regulator of G protein signaling 6 [Source:HGNC Symbol;Acc:HGNC:10002] |
| ENSG00000182793 | HGLibA_20464 | 30 | -0.863333203 | GSTA5 | glutathione S-transferase alpha 5 [Source:HGNC Symbol;Acc:HGNC:19662] |
| ENSG00000182986 | HGLibA_55795 | 30 | -0.863333203 | ZNF320 | zinc finger protein 320 [Source:HGNC Symbol;Acc:HGNC:13842] |
| ENSG00000183718 | HGLibA_51540 | 30 | -0.863333203 | TRIM52 | tripartite motif containing 52 [Source:HGNC Symbol;Acc:HGNC:19024] |
| ENSG00000184060 | HGLibA_00866 | 30 | -0.863333203 | ADAP2 | ArfGAP with dual PH domains 2 [Source:HGNC Symbol;Acc:HGNC:16487] |
| ENSG00000184697 | HGLibA_09903 | 30 | -0.863333203 | CLDN6 | claudin 6 [Source:HGNC Symbol;Acc:HGNC:2048] |
| ENSG00000184923 | HGLibA_33104 | 30 | -0.863333203 | NUTM2A | NUT family member 2A [Source:HGNC Symbol;Acc:HGNC:23438] |
| ENSG00000185236 | HGLibA_39790 | 30 | -0.863333203 | RAB11B | member RAS oncogene family [Source:HGNC Symbol;Acc:HGNC:9761]" |
| ENSG00000185385 | HGLibA_34461 | 30 | -0.863333203 | OR7A17 | olfactory receptor family 7 subfamily A member 17 [Source:HGNC Symbol;Acc:HGNC:8363] |
| ENSG00000185483 | HGLibA_41769 | 30 | -0.863333203 | ROR1 | receptor tyrosine kinase like orphan receptor 1 [Source:HGNC Symbol;Acc:HGNC:10256] |
| ENSG00000185658 | HGLibA_04853 | 30 | -0.863333203 | BRWD1 | bromodomain and WD repeat domain containing 1 [Source:HGNC Symbol;Acc:HGNC:12760] |
| ENSG00000185880 | HGLibA_51594 | 30 | -0.863333203 | TRIM69 | tripartite motif containing 69 [Source:HGNC Symbol;Acc:HGNC:17857] |
| ENSG00000185899 | HGLibA_48423 | 30 | -0.863333203 | TAS2R60 | taste 2 receptor member 60 [Source:HGNC Symbol;Acc:HGNC:20639] |
| ENSG00000187545 | HGLibA_38281 | 30 | -0.863333203 | PRAMEF10 | PRAME family member 10 [Source:HGNC Symbol;Acc:HGNC:27997] |
| ENSG00000187764 | HGLibA_43350 | 30 | -0.863333203 | SEMA4D | semaphorin 4D [Source:HGNC Symbol;Acc:HGNC:10732] |

| | | | | | |
|---|---|---|---|---|---|
| ENSG00000188641 | HGLibA_13919 | 30 | -0.863333203 | DPYD | dihydropyrimidine dehydrogenase [Source:HGNC Symbol;Acc:HGNC:3012] |
| ENSG00000189320 | HGLibA_16311 | 30 | -0.863333203 | FAM180A | family with sequence similarity 180 member A [Source:HGNC Symbol;Acc:HGNC:33773] |
| ENSG00000197223 | HGLibA_05720 | 30 | -0.863333203 | C1D | C1D nuclear receptor corepressor [Source:HGNC Symbol;Acc:HGNC:29911] |
| ENSG00000197457 | HGLibA_47523 | 30 | -0.863333203 | STMN3 | stathmin 3 [Source:HGNC Symbol;Acc:HGNC:15926] |
| ENSG00000197937 | HGLibA_55852 | 30 | -0.863333203 | ZNF347 | zinc finger protein 347 [Source:HGNC Symbol;Acc:HGNC:16447] |
| ENSG00000198099 | HGLibA_00957 | 30 | -0.863333203 | ADH4 | pi polypeptide [Source:HGNC Symbol;Acc:HGNC:252]" |
| ENSG00000198400 | HGLibA_32874 | 30 | -0.863333203 | NTRK1 | neurotrophic receptor tyrosine kinase 1 [Source:HGNC Symbol;Acc:HGNC:8031] |
| ENSG00000203756 | HGLibA_50297 | 30 | -0.863333203 | TMEM244 | transmembrane protein 244 [Source:HGNC Symbol;Acc:HGNC:21571] |
| ENSG00000204316 | HGLibA_29969 | 30 | -0.863333203 | MRPL38 | mitochondrial ribosomal protein L38 [Source:HGNC Symbol;Acc:HGNC:14033] |
| ENSG00000204599 | HGLibA_51484 | 30 | -0.863333203 | TRIM39 | tripartite motif containing 39 [Source:HGNC Symbol;Acc:HGNC:10065] |
| ENSG00000205221 | HGLibA_53673 | 30 | -0.863333203 | VIT | vitrin [Source:HGNC Symbol;Acc:HGNC:12697] |
| ENSG00000206127 | HGLibA_19646 | 30 | -0.863333203 | GOLGA8O | golgin A8 family member O [Source:HGNC Symbol;Acc:HGNC:44406] |
| ENSG00000212126 | HGLibA_48421 | 30 | -0.863333203 | TAS2R50 | taste 2 receptor member 50 [Source:HGNC Symbol;Acc:HGNC:18882] |
| ENSG00000214681 | HGLibA_23511 | 30 | -0.863333203 | IQCF5 | IQ motif containing F5 [Source:HGNC Symbol;Acc:HGNC:35159] |
| ENSG00000226372 | HGLibA_12461 | 30 | -0.863333203 | DCAF8L1 | DDB1 and CUL4 associated factor 8 like 1 [Source:HGNC Symbol;Acc:HGNC:31810] |
| ENSG00000229859 | HGLibA_36186 | 30 | -0.863333203 | PGA3 | group I (pepsinogen A) [Source:HGNC Symbol;Acc:HGNC:8885]" |
| ENSG00000233701 | HGLibA_38828 | 30 | -0.863333203 | PRR23C | proline rich 23C [Source:HGNC Symbol;Acc:HGNC:37173] |
| ENSG00000237353 | HGLibA_35279 | 30 | -0.863333203 | PATE4 | prostate and testis expressed 4 [Source:HGNC Symbol;Acc:HGNC:35427] |
| ENSG00000242372 | HGLibA_14868 | 30 | -0.863333203 | EIF6 | eukaryotic translation initiation factor 6 [Source:HGNC Symbol;Acc:HGNC:6159] |
| ENSG00000256162 | HGLibA_45778 | 30 | -0.863333203 | SMLR1 | small leucine rich protein 1 [Source:HGNC Symbol;Acc:HGNC:44670] |
| ENSG00000256294 | HGLibA_55620 | 30 | -0.863333203 | ZNF225 | zinc finger protein 225 [Source:HGNC Symbol;Acc:HGNC:13018] |
| ENSG00000278540 | HGLibA_00348 | 30 | -0.863333203 | ACACA | acetyl-CoA carboxylase alpha [Source:HGNC Symbol;Acc:HGNC:84] |
| ENSG00000279111 | HGLibA_33556 | 30 | -0.863333203 | OR10X1 | olfactory receptor family 10 subfamily X member 1 (gene/pseudogene) [Source:HGNC Symbol;Acc:HGNC:14995] |
| FAM105A | HGLibA_15993 | 30 | -0.863333203 | FAM105A | - |
| FAM26E | HGLibA_16563 | 30 | -0.863333203 | FAM26E | - |
| GAGE12I | HGLibA_18458 | 30 | -0.863333203 | GAGE12I | - |
| GATSL1 | HGLibA_18702 | 30 | -0.863333203 | GATSL1 | - |
| GATSL2 | HGLibA_18703 | 30 | -0.863333203 | GATSL2 | - |
| hsa-mir-1183 | HGLibA_57226 | 30 | -0.863333203 | hsa-mir-1183 | - |
| hsa-mir-1270-1 | HGLibA_57514 | 30 | -0.863333203 | hsa-mir-1270-1 | - |
| hsa-mir-129-1 | HGLibA_57629 | 30 | -0.863333203 | hsa-mir-129-1 | - |
| hsa-mir-199a-1 | HGLibA_58129 | 30 | -0.863333203 | hsa-mir-199a-1 | - |
| hsa-mir-199a-2 | HGLibA_58131 | 30 | -0.863333203 | hsa-mir-199a-2 | - |
| hsa-mir-3118-5 | HGLibA_58519 | 30 | -0.863333203 | hsa-mir-3118-5 | - |
| hsa-mir-3159 | HGLibA_58710 | 30 | -0.863333203 | hsa-mir-3159 | - |
| hsa-mir-3180-1 | HGLibA_58801 | 30 | -0.863333203 | hsa-mir-3180-1 | - |
| hsa-mir-3195 | HGLibA_58876 | 30 | -0.863333203 | hsa-mir-3195 | - |
| hsa-mir-320e | HGLibA_58952 | 30 | -0.863333203 | hsa-mir-320e | - |
| hsa-mir-325 | HGLibA_58966 | 30 | -0.863333203 | hsa-mir-325 | - |
| hsa-mir-3670-2 | HGLibA_59237 | 30 | -0.863333203 | hsa-mir-3670-2 | - |
| hsa-mir-4311 | HGLibA_59958 | 30 | -0.863333203 | hsa-mir-4311 | - |
| hsa-mir-4462 | HGLibA_60253 | 30 | -0.863333203 | hsa-mir-4462 | - |
| hsa-mir-4477a | HGLibA_60317 | 30 | -0.863333203 | hsa-mir-4477a | - |
| hsa-mir-4662a | HGLibA_60757 | 30 | -0.863333203 | hsa-mir-4662a | - |
| hsa-mir-4731 | HGLibA_61020 | 30 | -0.863333203 | hsa-mir-4731 | - |
| hsa-mir-4742 | HGLibA_61064 | 30 | -0.863333203 | hsa-mir-4742 | - |
| hsa-mir-4788 | HGLibA_61258 | 30 | -0.863333203 | hsa-mir-4788 | - |
| hsa-mir-4796 | HGLibA_61290 | 30 | -0.863333203 | hsa-mir-4796 | - |
| hsa-mir-548a-3 | HGLibA_61844 | 30 | -0.863333203 | hsa-mir-548a-3 | - |
| hsa-mir-548aa-1 | HGLibA_61848 | 30 | -0.863333203 | hsa-mir-548aa-1 | - |
| hsa-mir-5589 | HGLibA_62196 | 30 | -0.863333203 | hsa-mir-5589 | - |
| hsa-mir-580 | HGLibA_62417 | 30 | -0.863333203 | hsa-mir-580 | - |
| hsa-mir-595 | HGLibA_62474 | 30 | -0.863333203 | hsa-mir-595 | - |
| hsa-mir-6077-2 | HGLibA_62564 | 30 | -0.863333203 | hsa-mir-6077-2 | - |
| hsa-mir-6083 | HGLibA_62592 | 30 | -0.863333203 | hsa-mir-6083 | - |
| hsa-mir-637 | HGLibA_62788 | 30 | -0.863333203 | hsa-mir-637 | - |
| hsa-mir-6507 | HGLibA_62879 | 30 | -0.863333203 | hsa-mir-6507 | - |
| hsa-mir-6781 | HGLibA_63300 | 30 | -0.863333203 | hsa-mir-6781 | - |
| hsa-mir-6834 | HGLibA_63514 | 30 | -0.863333203 | hsa-mir-6834 | - |
| hsa-mir-6847 | HGLibA_63562 | 30 | -0.863333203 | hsa-mir-6847 | - |
| hsa-mir-7114 | HGLibA_63824 | 30 | -0.863333203 | hsa-mir-7114 | - |
| hsa-mir-874 | HGLibA_64206 | 30 | -0.863333203 | hsa-mir-874 | - |
| hsa-mir-99a | HGLibA_64379 | 30 | -0.863333203 | hsa-mir-99a | - |
| KIAA0141 | HGLibA_24592 | 30 | -0.863333203 | KIAA0141 | - |
| LOC100506688 | HGLibA_26863 | 30 | -0.863333203 | LOC100506688 | - |
| PPAP2A | HGLibA_37816 | 30 | -0.863333203 | PPAP2A | - |
| SGK110 | HGLibA_43821 | 30 | -0.863333203 | SGK110 | - |
| SPDYE2L | HGLibA_46589 | 30 | -0.863333203 | SPDYE2L | - |
| TGIF2-C20orf24 | HGLibA_49243 | 30 | -0.863333203 | TGIF2-C20orf24 | - |
| UQCC | HGLibA_53095 | 30 | -0.863333203 | UQCC | - |
| C1orf234 | HGLibA_05849 | 29 | -0.872378546 | C1orf234 | - |
| C6orf203 | HGLibA_06471 | 29 | -0.872378546 | C6orf203 | - |
| CCDC108 | HGLibA_07529 | 29 | -0.872378546 | CCDC108 | - |
| CSB-PGBD3 | HGLibA_11309 | 29 | -0.872378546 | CSB-PGBD3 | - |
| ENSG00000005238 | HGLibA_16481 | 29 | -0.872378546 | FAM214B | family with sequence similarity 214 member B [Source:HGNC Symbol;Acc:HGNC:25666] |
| ENSG00000006638 | HGLibA_48679 | 29 | -0.872378546 | TBXA2R | thromboxane A2 receptor [Source:HGNC Symbol;Acc:HGNC:11608] |
| ENSG00000014123 | HGLibA_52849 | 29 | -0.872378546 | UFL1 | UFM1 specific ligase 1 [Source:HGNC Symbol;Acc:HGNC:23039] |
| ENSG00000023041 | HGLibA_55122 | 29 | -0.872378546 | ZDHHC6 | zinc finger DHHC-type containing 6 [Source:HGNC Symbol;Acc:HGNC:19160] |
| ENSG00000023445 | HGLibA_04519 | 29 | -0.872378546 | BIRC3 | baculoviral IAP repeat containing 3 [Source:HGNC Symbol;Acc:HGNC:591] |
| ENSG00000031691 | HGLibA_09083 | 29 | -0.872378546 | CENPQ | centromere protein Q [Source:HGNC Symbol;Acc:HGNC:21347] |
| ENSG00000042832 | HGLibA_49199 | 29 | -0.872378546 | TG | thyroglobulin [Source:HGNC Symbol;Acc:HGNC:11764] |
| ENSG00000065618 | HGLibA_10522 | 29 | -0.872378546 | COL17A1 | collagen type XVII alpha 1 chain [Source:HGNC Symbol;Acc:HGNC:2194] |
| ENSG00000066468 | HGLibA_17443 | 29 | -0.872378546 | FGFR2 | fibroblast growth factor receptor 2 [Source:HGNC Symbol;Acc:HGNC:3689] |
| ENSG00000067048 | HGLibA_12706 | 29 | -0.872378546 | DDX3Y | DEAD-box helicase 3 Y-linked [Source:HGNC Symbol;Acc:HGNC:2699] |
| ENSG00000068654 | HGLibA_37550 | 29 | -0.872378546 | POLR1A | RNA polymerase I subunit A [Source:HGNC Symbol;Acc:HGNC:17264] |
| ENSG00000069509 | HGLibA_18165 | 29 | -0.872378546 | FUNDC1 | FUN14 domain containing 1 [Source:HGNC Symbol;Acc:HGNC:28746] |
| ENSG00000070495 | HGLibA_23959 | 29 | -0.872378546 | JMJD6 | arginine demethylase and lysine hydroxylase [Source:HGNC Symbol;Acc:HGNC:19355]" |
| ENSG00000075790 | HGLibA_04240 | 29 | -0.872378546 | BCAP29 | B cell receptor associated protein 29 [Source:HGNC Symbol;Acc:HGNC:24131] |
| ENSG00000076650 | HGLibA_19736 | 29 | -0.872378546 | GPATCH1 | G-patch domain containing 1 [Source:HGNC Symbol;Acc:HGNC:24658] |
| ENSG00000076650 | HGLibA_19734 | 29 | -0.872378546 | GPATCH1 | G-patch domain containing 1 [Source:HGNC Symbol;Acc:HGNC:24658] |
| ENSG00000077935 | HGLibA_45643 | 29 | -0.872378546 | SMC1B | structural maintenance of chromosomes 1B [Source:HGNC Symbol;Acc:HGNC:11112] |
| ENSG00000081052 | HGLibA_10577 | 29 | -0.872378546 | COL4A4 | collagen type IV alpha 4 chain [Source:HGNC Symbol;Acc:HGNC:2206] |
| ENSG00000087074 | HGLibA_38047 | 29 | -0.872378546 | PPP1R15A | protein phosphatase 1 regulatory subunit 15A [Source:HGNC Symbol;Acc:HGNC:14375] |
| ENSG00000088356 | HGLibA_35941 | 29 | -0.872378546 | PDRG1 | p53 and DNA damage regulated 1 [Source:HGNC Symbol;Acc:HGNC:16119] |
| ENSG00000089289 | HGLibA_22783 | 29 | -0.872378546 | IGBP1 | immunoglobulin binding protein 1 [Source:HGNC Symbol;Acc:HGNC:5461] |
| ENSG00000092850 | HGLibA_48975 | 29 | -0.872378546 | TEKT2 | tektin 2 [Source:HGNC Symbol;Acc:HGNC:11725] |
| ENSG00000095066 | HGLibA_21880 | 29 | -0.872378546 | HOOK2 | hook microtubule tethering protein 2 [Source:HGNC Symbol;Acc:HGNC:19885] |
| ENSG00000096996 | HGLibA_22966 | 29 | -0.872378546 | IL12RB1 | interleukin 12 receptor subunit beta 1 [Source:HGNC Symbol;Acc:HGNC:5971] |
| ENSG00000100083 | HGLibA_18981 | 29 | -0.872378546 | GGA1 | ARF binding protein 1 [Source:HGNC Symbol;Acc:HGNC:17842]" |
| ENSG00000100532 | HGLibA_09328 | 29 | -0.872378546 | CGRRF1 | cell growth regulator with ring finger domain 1 [Source:HGNC Symbol;Acc:HGNC:15528] |
| ENSG00000100814 | HGLibA_08061 | 29 | -0.872378546 | CCNB1IP1 | cyclin B1 interacting protein 1 [Source:HGNC Symbol;Acc:HGNC:19437] |
| ENSG00000100883 | HGLibA_47006 | 29 | -0.872378546 | SRP54 | signal recognition particle 54 [Source:HGNC Symbol;Acc:HGNC:11301] |
| ENSG00000101280 | HGLibA_01907 | 29 | -0.872378546 | ANGPT4 | angiopoietin 4 [Source:HGNC Symbol;Acc:HGNC:487] |
| ENSG00000102243 | HGLibA_53634 | 29 | -0.872378546 | VGLL1 | vestigial like family member 1 [Source:HGNC Symbol;Acc:HGNC:20985] |
| ENSG00000104695 | HGLibA_38137 | 29 | -0.872378546 | PPP2CB | protein phosphatase 2 catalytic subunit beta [Source:HGNC Symbol;Acc:HGNC:9300] |
| ENSG00000105171 | HGLibA_37691 | 29 | -0.872378546 | POP4 | ribonuclease P/MRP subunit [Source:HGNC Symbol;Acc:HGNC:30081]" |
| ENSG00000105398 | HGLibA_47758 | 29 | -0.872378546 | SULT2A1 | sulfotransferase family 2A member 1 [Source:HGNC Symbol;Acc:HGNC:11458] |
| ENSG00000105609 | HGLibA_26520 | 29 | -0.872378546 | LILRB5 | leukocyte immunoglobulin like receptor B5 [Source:HGNC Symbol;Acc:HGNC:6609] |
| ENSG00000105613 | HGLibA_28400 | 29 | -0.872378546 | MAST1 | microtubule associated serine/threonine kinase 1 [Source:HGNC Symbol;Acc:HGNC:19034] |
| ENSG00000107731 | HGLibA_53016 | 29 | -0.872378546 | UNC5B | unc-5 netrin receptor B [Source:HGNC Symbol;Acc:HGNC:12568] |
| ENSG00000108582 | HGLibA_10911 | 29 | -0.872378546 | CPD | carboxypeptidase D [Source:HGNC Symbol;Acc:HGNC:2301] |
| ENSG00000108591 | HGLibA_13978 | 29 | -0.872378546 | DRG2 | developmentally regulated GTP binding protein 2 [Source:HGNC Symbol;Acc:HGNC:3030] |
| ENSG00000109424 | HGLibA_52831 | 29 | -0.872378546 | UCP1 | uncoupling protein 1 [Source:HGNC Symbol;Acc:HGNC:12517] |
| ENSG00000109654 | HGLibA_51426 | 29 | -0.872378546 | TRIM2 | tripartite motif containing 2 [Source:HGNC Symbol;Acc:HGNC:15974] |
| ENSG00000110484 | HGLibA_42927 | 29 | -0.872378546 | SCGB2A2 | secretoglobin family 2A member 2 [Source:HGNC Symbol;Acc:HGNC:7050] |
| ENSG00000110887 | HGLibA_12311 | 29 | -0.872378546 | DAO | D-amino acid oxidase [Source:HGNC Symbol;Acc:HGNC:2671] |
| ENSG00000111361 | HGLibA_14735 | 29 | -0.872378546 | EIF2B1 | eukaryotic translation initiation factor 2B subunit alpha [Source:HGNC Symbol;Acc:HGNC:3257] |
| ENSG00000111554 | HGLibA_28693 | 29 | -0.872378546 | MDM1 | Mdm1 nuclear protein [Source:HGNC Symbol;Acc:HGNC:29917] |
| ENSG00000112116 | HGLibA_23001 | 29 | -0.872378546 | IL17F | interleukin 17F [Source:HGNC Symbol;Acc:HGNC:16404] |
| ENSG00000112893 | HGLibA_28069 | 29 | -0.872378546 | MAN2A1 | mannosidase alpha class 2A member 1 [Source:HGNC Symbol;Acc:HGNC:6824] |
| ENSG00000114439 | HGLibA_04230 | 29 | -0.872378546 | BBX | HMG-box containing [Source:HGNC Symbol;Acc:HGNC:14422]" |
| ENSG00000115761 | HGLibA_32255 | 29 | -0.872378546 | NOL10 | nucleolar protein 10 [Source:HGNC Symbol;Acc:HGNC:25862] |
| ENSG00000116819 | HGLibA_49134 | 29 | -0.872378546 | TFAP2E | transcription factor AP-2 epsilon [Source:HGNC Symbol;Acc:HGNC:30774] |
| ENSG00000118785 | HGLibA_46768 | 29 | -0.872378546 | SPP1 | secreted phosphoprotein 1 [Source:HGNC Symbol;Acc:HGNC:11255] |
| ENSG00000120057 | HGLibA_43745 | 29 | -0.872378546 | SFRP5 | secreted frizzled related protein 5 [Source:HGNC Symbol;Acc:HGNC:10779] |
| ENSG00000121207 | HGLibA_27131 | 29 | -0.872378546 | LRAT | lecithin retinol acyltransferase [Source:HGNC Symbol;Acc:HGNC:6685] |
| ENSG00000121446 | HGLibA_41119 | 29 | -0.872378546 | RGSL1 | regulator of G protein signaling like 1 [Source:HGNC Symbol;Acc:HGNC:18636] |
| ENSG00000121594 | HGLibA_08452 | 29 | -0.872378546 | CD80 | CD80 molecule [Source:HGNC Symbol;Acc:HGNC:1700] |
| ENSG00000121903 | HGLibA_56999 | 29 | -0.872378546 | ZSCAN20 | zinc finger and SCAN domain containing 20 [Source:HGNC Symbol;Acc:HGNC:13093] |
| ENSG00000122254 | HGLibA_22123 | 29 | -0.872378546 | HS3ST2 | heparan sulfate-glucosamine 3-sulfotransferase 2 [Source:HGNC Symbol;Acc:HGNC:5195] |
| ENSG00000122359 | HGLibA_02226 | 29 | -0.872378546 | ANXA11 | annexin A11 [Source:HGNC Symbol;Acc:HGNC:535] |
| ENSG00000124171 | HGLibA_35188 | 29 | -0.872378546 | PARD6B | par-6 family cell polarity regulator beta [Source:HGNC Symbol;Acc:HGNC:16245] |
| ENSG00000124491 | HGLibA_15853 | 29 | -0.872378546 | F13A1 | coagulation factor XIII A chain [Source:HGNC Symbol;Acc:HGNC:3531] |
| ENSG00000125430 | HGLibA_22130 | 29 | -0.872378546 | HS3ST3B1 | heparan sulfate-glucosamine 3-sulfotransferase 3B1 [Source:HGNC Symbol;Acc:HGNC:5198] |
| ENSG00000125503 | HGLibA_38026 | 29 | -0.872378546 | PPP1R12C | protein phosphatase 1 regulatory subunit 12C [Source:HGNC Symbol;Acc:HGNC:14947] |

| | | | | | |
|---|---|---|---|---|---|
| ENSG00000125813 | HGLibA_35294 | 29 | -0.872378546 | PAX1 | paired box 1 [Source:HGNC Symbol;Acc:HGNC:8615] |
| ENSG00000126883 | HGLibA_33044 | 29 | -0.872378546 | NUP214 | nucleoporin 214 [Source:HGNC Symbol;Acc:HGNC:8064] |
| ENSG00000126945 | HGLibA_21826 | 29 | -0.872378546 | HNRNPH2 | heterogeneous nuclear ribonucleoprotein H2 [Source:HGNC Symbol;Acc:HGNC:5042] |
| ENSG00000128040 | HGLibA_46690 | 29 | -0.872378546 | SPINK2 | Kazal type 2 [Source:HGNC Symbol;Acc:HGNC:11245]" |
| ENSG00000128578 | HGLibA_47590 | 29 | -0.872378546 | STRIP2 | striatin interacting protein 2 [Source:HGNC Symbol;Acc:HGNC:22209] |
| ENSG00000129235 | HGLibA_52416 | 29 | -0.872378546 | TXNDC17 | thioredoxin domain containing 17 [Source:HGNC Symbol;Acc:HGNC:28218] |
| ENSG00000129636 | HGLibA_23706 | 29 | -0.872378546 | ITFG1 | integrin alpha FG-GAP repeat containing 1 [Source:HGNC Symbol;Acc:HGNC:30697] |
| ENSG00000131043 | HGLibA_00063 | 29 | -0.872378546 | AAR2 | AAR2 splicing factor homolog [Source:HGNC Symbol;Acc:HGNC:15886] |
| ENSG00000131368 | HGLibA_30074 | 29 | -0.872378546 | MRPS25 | mitochondrial ribosomal protein S25 [Source:HGNC Symbol;Acc:HGNC:14511] |
| ENSG00000131737 | HGLibA_25487 | 29 | -0.872378546 | KRT34 | keratin 34 [Source:HGNC Symbol;Acc:HGNC:6452] |
| ENSG00000132207 | HGLibA_45556 | 29 | -0.872378546 | SLX1A | structure-specific endonuclease subunit [Source:HGNC Symbol;Acc:HGNC:20922]" |
| ENSG00000132434 | HGLibA_26008 | 29 | -0.872378546 | LANCL2 | LanC like 2 [Source:HGNC Symbol;Acc:HGNC:6509] |
| ENSG00000133135 | HGLibA_41499 | 29 | -0.872378546 | RNF128 | E3 ubiquitin protein ligase [Source:HGNC Symbol;Acc:HGNC:21153]" |
| ENSG00000133422 | HGLibA_29652 | 29 | -0.872378546 | MORC2 | MORC family CW-type zinc finger 2 [Source:HGNC Symbol;Acc:HGNC:23573] |
| ENSG00000134291 | HGLibA_49858 | 29 | -0.872378546 | TMEM106C | transmembrane protein 106C [Source:HGNC Symbol;Acc:HGNC:28775] |
| ENSG00000135916 | HGLibA_23834 | 29 | -0.872378546 | ITM2C | integral membrane protein 2C [Source:HGNC Symbol;Acc:HGNC:6175] |
| ENSG00000135930 | HGLibA_14821 | 29 | -0.872378546 | EIF4E2 | eukaryotic translation initiation factor 4E family member 2 [Source:HGNC Symbol;Acc:HGNC:3293] |
| ENSG00000135968 | HGLibA_18766 | 29 | -0.872378546 | GCC2 | GRIP and coiled-coil domain containing 2 [Source:HGNC Symbol;Acc:HGNC:23218] |
| ENSG00000135974 | HGLibA_06141 | 29 | -0.872378546 | C2orf49 | chromosome 2 open reading frame 49 [Source:HGNC Symbol;Acc:HGNC:28772] |
| ENSG00000136048 | HGLibA_13944 | 29 | -0.872378546 | DRAM1 | DNA damage regulated autophagy modulator 1 [Source:HGNC Symbol;Acc:HGNC:25645] |
| ENSG00000136247 | HGLibA_55116 | 29 | -0.872378546 | ZDHHC4 | zinc finger DHHC-type containing 4 [Source:HGNC Symbol;Acc:HGNC:18471] |
| ENSG00000136709 | HGLibA_54068 | 29 | -0.872378546 | WDR33 | WD repeat domain 33 [Source:HGNC Symbol;Acc:HGNC:25651] |
| ENSG00000136881 | HGLibA_04054 | 29 | -0.872378546 | BAAT | bile acid-CoA:amino acid N-acyltransferase [Source:HGNC Symbol;Acc:HGNC:932] |
| ENSG00000136931 | HGLibA_32635 | 29 | -0.872378546 | NR5A1 | nuclear receptor subfamily 5 group A member 1 [Source:HGNC Symbol;Acc:HGNC:7983] |
| ENSG00000137976 | HGLibA_13667 | 29 | -0.872378546 | DNASE2B | deoxyribonuclease 2 beta [Source:HGNC Symbol;Acc:HGNC:28875] |
| ENSG00000138684 | HGLibA_23082 | 29 | -0.872378546 | IL21 | interleukin 21 [Source:HGNC Symbol;Acc:HGNC:6005] |
| ENSG00000138685 | HGLibA_17391 | 29 | -0.872378546 | FGF2 | fibroblast growth factor 2 [Source:HGNC Symbol;Acc:HGNC:3676] |
| ENSG00000138942 | HGLibA_41593 | 29 | -0.872378546 | RNF185 | ring finger protein 185 [Source:HGNC Symbol;Acc:HGNC:26783] |
| ENSG00000141562 | HGLibA_31142 | 29 | -0.872378546 | NARF | nuclear prelamin A recognition factor [Source:HGNC Symbol;Acc:HGNC:29916] |
| ENSG00000142534 | HGLibA_42064 | 29 | -0.872378546 | RPS11 | ribosomal protein S11 [Source:HGNC Symbol;Acc:HGNC:10384] |
| ENSG00000142541 | HGLibA_41871 | 29 | -0.872378546 | RPL13A | ribosomal protein L13a [Source:HGNC Symbol;Acc:HGNC:10304] |
| ENSG00000143157 | HGLibA_37474 | 29 | -0.872378546 | POGK | pogo transposable element derived with KRAB domain [Source:HGNC Symbol;Acc:HGNC:18800] |
| ENSG00000143641 | HGLibA_18554 | 29 | -0.872378546 | GALNT2 | polypeptide N-acetylgalactosaminyltransferase 2 [Source:HGNC Symbol;Acc:HGNC:4124] |
| ENSG00000143774 | HGLibA_20674 | 29 | -0.872378546 | GUK1 | guanylate kinase 1 [Source:HGNC Symbol;Acc:HGNC:4693] |
| ENSG00000144426 | HGLibA_31209 | 29 | -0.872378546 | NBEAL1 | neurobeachin like 1 [Source:HGNC Symbol;Acc:HGNC:20681] |
| ENSG00000145439 | HGLibA_07424 | 29 | -0.872378546 | CBR4 | carbonyl reductase 4 [Source:HGNC Symbol;Acc:HGNC:25891] |
| ENSG00000146090 | HGLibA_40334 | 29 | -0.872378546 | RASGEF1C | RasGEF domain family member 1C [Source:HGNC Symbol;Acc:HGNC:27400] |
| ENSG00000146410 | HGLibA_30368 | 29 | -0.872378546 | MTFR2 | mitochondrial fission regulator 2 [Source:HGNC Symbol;Acc:HGNC:21115] |
| ENSG00000147669 | HGLibA_37599 | 29 | -0.872378546 | POLR2K | RNA polymerase II subunit K [Source:HGNC Symbol;Acc:HGNC:9198] |
| ENSG00000147883 | HGLibA_08838 | 29 | -0.872378546 | CDKN2B | cyclin dependent kinase inhibitor 2B [Source:HGNC Symbol;Acc:HGNC:1788] |
| ENSG00000149380 | HGLibA_34907 | 29 | -0.872378546 | P4HA3 | prolyl 4-hydroxylase subunit alpha 3 [Source:HGNC Symbol;Acc:HGNC:30135] |
| ENSG00000151502 | HGLibA_53754 | 29 | -0.872378546 | VPS26B | retromer complex component B [Source:HGNC Symbol;Acc:HGNC:28119]" |
| ENSG00000153879 | HGLibA_08968 | 29 | -0.872378546 | CEBPG | CCAAT enhancer binding protein gamma [Source:HGNC Symbol;Acc:HGNC:1837] |
| ENSG00000154380 | HGLibA_15105 | 29 | -0.872378546 | ENAH | actin regulator [Source:HGNC Symbol;Acc:HGNC:18271]" |
| ENSG00000156697 | HGLibA_53434 | 29 | -0.872378546 | UTP14A | small subunit processome component [Source:HGNC Symbol;Acc:HGNC:10665]" |
| ENSG00000158315 | HGLibA_41139 | 29 | -0.872378546 | RHBDL2 | rhomboid like 2 [Source:HGNC Symbol;Acc:HGNC:16083] |
| ENSG00000158805 | HGLibA_55713 | 29 | -0.872378546 | ZNF276 | zinc finger protein 276 [Source:HGNC Symbol;Acc:HGNC:23330] |
| ENSG00000159761 | HGLibA_05488 | 29 | -0.872378546 | C16orf86 | chromosome 16 open reading frame 86 [Source:HGNC Symbol;Acc:HGNC:33755] |
| ENSG00000160200 | HGLibA_07426 | 29 | -0.872378546 | CBS | cystathionine-beta-synthase [Source:HGNC Symbol;Acc:HGNC:1550] |
| ENSG00000160888 | HGLibA_22573 | 29 | -0.872378546 | IER2 | immediate early response 2 [Source:HGNC Symbol;Acc:HGNC:28871] |
| ENSG00000162496 | HGLibA_13132 | 29 | -0.872378546 | DHRS3 | dehydrogenase/reductase 3 [Source:HGNC Symbol;Acc:HGNC:17693] |
| ENSG00000162769 | HGLibA_17675 | 29 | -0.872378546 | FLVCR1 | feline leukemia virus subgroup C cellular receptor 1 [Source:HGNC Symbol;Acc:HGNC:24682] |
| ENSG00000163554 | HGLibA_46871 | 29 | -0.872378546 | SPTA1 | erythrocytic 1 [Source:HGNC Symbol;Acc:HGNC:11272]" |
| ENSG00000163637 | HGLibA_38474 | 29 | -0.872378546 | PRICKLE2 | prickle planar cell polarity protein 2 [Source:HGNC Symbol;Acc:HGNC:20340] |
| ENSG00000163817 | HGLibA_45322 | 29 | -0.872378546 | SLC6A20 | solute carrier family 6 member 20 [Source:HGNC Symbol;Acc:HGNC:30927] |
| ENSG00000164265 | HGLibA_42936 | 29 | -0.872378546 | SCGB3A2 | secretoglobin family 3A member 2 [Source:HGNC Symbol;Acc:HGNC:18391] |
| ENSG00000164284 | HGLibA_20374 | 29 | -0.872378546 | GRPEL2 | mitochondrial [Source:HGNC Symbol;Acc:HGNC:21060]" |
| ENSG00000164366 | HGLibA_07584 | 29 | -0.872378546 | CCDC127 | coiled-coil domain containing 127 [Source:HGNC Symbol;Acc:HGNC:30520] |
| ENSG00000164398 | HGLibA_00565 | 29 | -0.872378546 | ACSL6 | acyl-CoA synthetase long chain family member 6 [Source:HGNC Symbol;Acc:HGNC:16496] |
| ENSG00000164729 | HGLibA_45021 | 29 | -0.872378546 | SLC35G3 | solute carrier family 35 member G3 [Source:HGNC Symbol;Acc:HGNC:26848] |
| ENSG00000165055 | HGLibA_29004 | 29 | -0.872378546 | METTL2B | methyltransferase like 2B [Source:HGNC Symbol;Acc:HGNC:18272] |
| ENSG00000165204 | HGLibA_33682 | 29 | -0.872378546 | OR1K1 | olfactory receptor family 1 subfamily K member 1 [Source:HGNC Symbol;Acc:HGNC:8212] |
| ENSG00000165966 | HGLibA_36001 | 29 | -0.872378546 | PDZRN4 | PDZ domain containing ring finger 4 [Source:HGNC Symbol;Acc:HGNC:30552] |
| ENSG00000166073 | HGLibA_19975 | 29 | -0.872378546 | GPR176 | G protein-coupled receptor 176 [Source:HGNC Symbol;Acc:HGNC:32370] |
| ENSG00000166165 | HGLibA_09765 | 29 | -0.872378546 | CKB | creatine kinase B [Source:HGNC Symbol;Acc:HGNC:1991] |
| ENSG00000166224 | HGLibA_43847 | 29 | -0.872378546 | SGPL1 | sphingosine-1-phosphate lyase 1 [Source:HGNC Symbol;Acc:HGNC:10817] |
| ENSG00000166619 | HGLibA_04539 | 29 | -0.872378546 | BLCAP | apoptosis inducing factor [Source:HGNC Symbol;Acc:HGNC:1055]" |
| ENSG00000166924 | HGLibA_33181 | 29 | -0.872378546 | NYAP1 | neuronal tyrosine phosphorylated phosphoinositide-3-kinase adaptor 1 [Source:HGNC Symbol;Acc:HGNC:22009] |
| ENSG00000167065 | HGLibA_14150 | 29 | -0.872378546 | DUSP18 | dual specificity phosphatase 18 [Source:HGNC Symbol;Acc:HGNC:18484] |
| ENSG00000167208 | HGLibA_46059 | 29 | -0.872378546 | SNX20 | sorting nexin 20 [Source:HGNC Symbol;Acc:HGNC:30390] |
| ENSG00000167306 | HGLibA_30852 | 29 | -0.872378546 | MYO5B | myosin VB [Source:HGNC Symbol;Acc:HGNC:7603] |
| ENSG00000167384 | HGLibA_55538 | 29 | -0.872378546 | ZNF180 | zinc finger protein 180 [Source:HGNC Symbol;Acc:HGNC:12970] |
| ENSG00000167550 | HGLibA_41156 | 29 | -0.872378546 | RHEBL1 | RHEB like 1 [Source:HGNC Symbol;Acc:HGNC:21166] |
| ENSG00000167671 | HGLibA_52793 | 29 | -0.872378546 | UBXN6 | UBX domain protein 6 [Source:HGNC Symbol;Acc:HGNC:14928] |
| ENSG00000167747 | HGLibA_05673 | 29 | -0.872378546 | C19orf48 | chromosome 19 open reading frame 48 [Source:HGNC Symbol;Acc:HGNC:29667] |
| ENSG00000167800 | HGLibA_48643 | 29 | -0.872378546 | TBX10 | T-box 10 [Source:HGNC Symbol;Acc:HGNC:11593] |
| ENSG00000168056 | HGLibA_27597 | 29 | -0.872378546 | LTBP3 | latent transforming growth factor beta binding protein 3 [Source:HGNC Symbol;Acc:HGNC:6716] |
| ENSG00000168490 | HGLibA_36474 | 29 | -0.872378546 | PHYHIP | phytanoyl-CoA 2-hydroxylase interacting protein [Source:HGNC Symbol;Acc:HGNC:16865] |
| ENSG00000168890 | HGLibA_49992 | 29 | -0.872378546 | TMEM150A | transmembrane protein 150A [Source:HGNC Symbol;Acc:HGNC:24677] |
| ENSG00000170425 | HGLibA_01015 | 29 | -0.872378546 | ADORA2B | adenosine A2b receptor [Source:HGNC Symbol;Acc:HGNC:264] |
| ENSG00000170477 | HGLibA_25505 | 29 | -0.872378546 | KRT4 | keratin 4 [Source:HGNC Symbol;Acc:HGNC:6441] |
| ENSG00000170484 | HGLibA_25534 | 29 | -0.872378546 | KRT74 | keratin 74 [Source:HGNC Symbol;Acc:HGNC:28929] |
| ENSG00000170545 | HGLibA_45593 | 29 | -0.872378546 | SMAGP | small cell adhesion glycoprotein [Source:HGNC Symbol;Acc:HGNC:26918] |
| ENSG00000170852 | HGLibA_24101 | 29 | -0.872378546 | KBTBD2 | kelch repeat and BTB domain containing 2 [Source:HGNC Symbol;Acc:HGNC:21751] |
| ENSG00000171227 | HGLibA_50375 | 29 | -0.872378546 | TMEM37 | transmembrane protein 37 [Source:HGNC Symbol;Acc:HGNC:18216] |
| ENSG00000173950 | HGLibA_54614 | 29 | -0.872378546 | XXYLT1 | xyloside xylosyltransferase 1 [Source:HGNC Symbol;Acc:HGNC:26639] |
| ENSG00000174483 | HGLibA_04205 | 29 | -0.872378546 | BBS1 | Bardet-Biedl syndrome 1 [Source:HGNC Symbol;Acc:HGNC:966] |
| ENSG00000175164 | HGLibA_00316 | 29 | -0.872378546 | ABO | alpha 1-3-N-acetylgalactosaminyltransferase and alpha 1-3-galactosyltransferase [Source:HGNC Symbol;Acc:HGNC:79]" |
| ENSG00000175764 | HGLibA_52193 | 29 | -0.872378546 | TTLL11 | tubulin tyrosine ligase like 11 [Source:HGNC Symbol;Acc:HGNC:18113] |
| ENSG00000175785 | HGLibA_38489 | 29 | -0.872378546 | PRIMA1 | proline rich membrane anchor 1 [Source:HGNC Symbol;Acc:HGNC:18319] |
| ENSG00000175879 | HGLibA_22004 | 29 | -0.872378546 | HOXD8 | homeobox D8 [Source:HGNC Symbol;Acc:HGNC:5139] |
| ENSG00000176390 | HGLibA_11196 | 29 | -0.872378546 | CRLF3 | cytokine receptor like factor 3 [Source:HGNC Symbol;Acc:HGNC:17177] |
| ENSG00000176692 | HGLibA_17826 | 29 | -0.872378546 | FOXC2 | forkhead box C2 [Source:HGNC Symbol;Acc:HGNC:3801] |
| ENSG00000176783 | HGLibA_42438 | 29 | -0.872378546 | RUFY1 | RUN and FYVE domain containing 1 [Source:HGNC Symbol;Acc:HGNC:19760] |
| ENSG00000177994 | HGLibA_06178 | 29 | -0.872378546 | C2orf73 | chromosome 2 open reading frame 73 [Source:HGNC Symbol;Acc:HGNC:26861] |
| ENSG00000178015 | HGLibA_19931 | 29 | -0.872378546 | GPR150 | G protein-coupled receptor 150 [Source:HGNC Symbol;Acc:HGNC:23628] |
| ENSG00000178750 | HGLibA_47634 | 29 | -0.872378546 | STX19 | syntaxin 19 [Source:HGNC Symbol;Acc:HGNC:19300] |
| ENSG00000178928 | HGLibA_51192 | 29 | -0.872378546 | TPRX1 | tetrapeptide repeat homeobox 1 [Source:HGNC Symbol;Acc:HGNC:32174] |
| ENSG00000179715 | HGLibA_35586 | 29 | -0.872378546 | PCED1B | PC-esterase domain containing 1B [Source:HGNC Symbol;Acc:HGNC:28255] |
| ENSG00000180938 | HGLibA_56282 | 29 | -0.872378546 | ZNF572 | zinc finger protein 572 [Source:HGNC Symbol;Acc:HGNC:26758] |
| ENSG00000181036 | HGLibA_17239 | 29 | -0.872378546 | FCRL6 | Fc receptor like 6 [Source:HGNC Symbol;Acc:HGNC:31910] |
| ENSG00000181631 | HGLibA_34878 | 29 | -0.872378546 | P2RY13 | purinergic receptor P2Y13 [Source:HGNC Symbol;Acc:HGNC:4537] |
| ENSG00000181847 | HGLibA_49484 | 29 | -0.872378546 | TIGIT | T cell immunoreceptor with Ig and ITIM domains [Source:HGNC Symbol;Acc:HGNC:26838] |
| ENSG00000182333 | HGLibA_26610 | 29 | -0.872378546 | LIPF | gastric type [Source:HGNC Symbol;Acc:HGNC:6622]" |
| ENSG00000182580 | HGLibA_15317 | 29 | -0.872378546 | EPHB3 | EPH receptor B3 [Source:HGNC Symbol;Acc:HGNC:3394] |
| ENSG00000182585 | HGLibA_15281 | 29 | -0.872378546 | EPGN | epithelial mitogen [Source:HGNC Symbol;Acc:HGNC:17470] |
| ENSG00000183640 | HGLibA_25826 | 29 | -0.872378546 | KRTAP8-1 | keratin associated protein 8-1 [Source:HGNC Symbol;Acc:HGNC:18935] |
| ENSG00000183709 | HGLibA_22723 | 29 | -0.872378546 | IFNL2 | interferon lambda 2 [Source:HGNC Symbol;Acc:HGNC:18364] |
| ENSG00000184611 | HGLibA_24228 | 29 | -0.872378546 | KCNH7 | potassium voltage-gated channel subfamily H member 7 [Source:HGNC Symbol;Acc:HGNC:18863] |
| ENSG00000185085 | HGLibA_23424 | 29 | -0.872378546 | INTS5 | integrator complex subunit 5 [Source:HGNC Symbol;Acc:HGNC:29352] |
| ENSG00000185298 | HGLibA_07606 | 29 | -0.872378546 | CCDC137 | coiled-coil domain containing 137 [Source:HGNC Symbol;Acc:HGNC:33451] |
| ENSG00000186081 | HGLibA_25511 | 29 | -0.872378546 | KRT5 | keratin 5 [Source:HGNC Symbol;Acc:HGNC:6442] |
| ENSG00000186174 | HGLibA_04350 | 29 | -0.872378546 | BCL9L | B cell CLL/lymphoma 9 like [Source:HGNC Symbol;Acc:HGNC:23688] |
| ENSG00000186440 | HGLibA_34436 | 29 | -0.872378546 | OR6P1 | olfactory receptor family 6 subfamily P member 1 [Source:HGNC Symbol;Acc:HGNC:15036] |
| ENSG00000188033 | HGLibA_56089 | 29 | -0.872378546 | ZNF490 | zinc finger protein 490 [Source:HGNC Symbol;Acc:HGNC:23705] |
| ENSG00000188175 | HGLibA_21140 | 29 | -0.872378546 | HEPACAM2 | HEPACAM family member 2 [Source:HGNC Symbol;Acc:HGNC:27364] |
| ENSG00000188283 | HGLibA_55890 | 29 | -0.872378546 | ZNF383 | zinc finger protein 383 [Source:HGNC Symbol;Acc:HGNC:18609] |
| ENSG00000188818 | HGLibA_55070 | 29 | -0.872378546 | ZDHHC11 | zinc finger DHHC-type containing 11 [Source:HGNC Symbol;Acc:HGNC:19158] |
| ENSG00000189152 | HGLibA_20217 | 29 | -0.872378546 | GRAPL | GRB2 related adaptor protein like [Source:HGNC Symbol;Acc:HGNC:37240] |
| ENSG00000196357 | HGLibA_56258 | 29 | -0.872378546 | ZNF565 | zinc finger protein 565 [Source:HGNC Symbol;Acc:HGNC:26726] |
| ENSG00000196408 | HGLibA_32384 | 29 | -0.872378546 | NOXO1 | NADPH oxidase organizer 1 [Source:HGNC Symbol;Acc:HGNC:19404] |
| ENSG00000196456 | HGLibA_56720 | 29 | -0.872378546 | ZNF775 | zinc finger protein 775 [Source:HGNC Symbol;Acc:HGNC:28501] |
| ENSG00000197043 | HGLibA_02246 | 29 | -0.872378546 | ANXA6 | annexin A6 [Source:HGNC Symbol;Acc:HGNC:544] |
| ENSG00000197128 | HGLibA_56709 | 29 | -0.872378546 | ZNF772 | zinc finger protein 772 [Source:HGNC Symbol;Acc:HGNC:33106] |
| ENSG00000198133 | HGLibA_50251 | 29 | -0.872378546 | TMEM229B | transmembrane protein 229B [Source:HGNC Symbol;Acc:HGNC:20130] |
| ENSG00000198189 | HGLibA_22174 | 29 | -0.872378546 | HSD17B11 | hydroxysteroid 17-beta dehydrogenase 11 [Source:HGNC Symbol;Acc:HGNC:22960] |
| ENSG00000198478 | HGLibA_43919 | 29 | -0.872378546 | SH3BGRL2 | SH3 domain binding glutamate rich protein like 2 [Source:HGNC Symbol;Acc:HGNC:15567] |
| ENSG00000198692 | HGLibA_14713 | 29 | -0.872378546 | EIF1AY | eukaryotic translation initiation factor 1A Y-linked [Source:HGNC Symbol;Acc:HGNC:3252] |
| ENSG00000198792 | HGLibA_50113 | 29 | -0.872378546 | TMEM184B | transmembrane protein 184B [Source:HGNC Symbol;Acc:HGNC:1310] |
| ENSG00000203926 | HGLibA_46381 | 29 | -0.872378546 | SPANXA2 | SPANX family member A2 [Source:HGNC Symbol;Acc:HGNC:14328] |
| ENSG00000204304 | HGLibA_35346 | 29 | -0.872378546 | PBX2 | PBX homeobox 2 [Source:HGNC Symbol;Acc:HGNC:8633] |
| ENSG00000204381 | HGLibA_26073 | 29 | -0.872378546 | LAYN | layilin [Source:HGNC Symbol;Acc:HGNC:29471] |
| ENSG00000204536 | HGLibA_07947 | 29 | -0.872378546 | CCHCR1 | coiled-coil alpha-helical rod protein 1 [Source:HGNC Symbol;Acc:HGNC:13930] |
| ENSG00000204613 | HGLibA_51402 | 29 | -0.872378546 | TRIM10 | tripartite motif containing 10 [Source:HGNC Symbol;Acc:HGNC:10072] |
| ENSG00000204889 | HGLibA_25507 | 29 | -0.872378546 | KRT40 | keratin 40 [Source:HGNC Symbol;Acc:HGNC:26707] |

| | | | | | |
|---|---|---|---|---|---|
| ENSG00000205649 | HGLibA_22353 | 29 | -0.872378546 | HTN3 | histatin 3 [Source:HGNC Symbol;Acc:HGNC:5284] |
| ENSG00000205978 | HGLibA_33187 | 29 | -0.872378546 | NYNRIN | NYN domain and retroviral integrase containing [Source:HGNC Symbol;Acc:HGNC:20165] |
| ENSG00000206052 | HGLibA_13781 | 29 | -0.872378546 | DOK6 | docking protein 6 [Source:HGNC Symbol;Acc:HGNC:28301] |
| ENSG00000213780 | HGLibA_20558 | 29 | -0.872378546 | GTF2H4 | general transcription factor IIH subunit 4 [Source:HGNC Symbol;Acc:HGNC:4658] |
| ENSG00000214114 | HGLibA_30666 | 29 | -0.872378546 | MYCBP | MYC binding protein [Source:HGNC Symbol;Acc:HGNC:7554] |
| ENSG00000214510 | HGLibA_46684 | 29 | -0.872378546 | SPINK13 | Kazal type 13 (putative) [Source:HGNC Symbol;Acc:HGNC:27200]" |
| ENSG00000226763 | HGLibA_47053 | 29 | -0.872378546 | SRRM5 | serine/arginine repetitive matrix 5 [Source:HGNC Symbol;Acc:HGNC:37248] |
| ENSG00000232423 | HGLibA_38322 | 29 | -0.872378546 | PRAMEF6 | PRAME family member 6 [Source:HGNC Symbol;Acc:HGNC:30583] |
| ENSG00000233276 | HGLibA_20169 | 29 | -0.872378546 | GPX1 | glutathione peroxidase 1 [Source:HGNC Symbol;Acc:HGNC:4553] |
| ENSG00000234616 | HGLibA_23991 | 29 | -0.872378546 | JRK | Jrk helix-turn-helix protein [Source:HGNC Symbol;Acc:HGNC:6199] |
| ENSG00000235034 | HGLibA_05713 | 29 | -0.872378546 | C19orf81 | chromosome 19 open reading frame 81 [Source:HGNC Symbol;Acc:HGNC:40041] |
| ENSG00000241973 | HGLibA_36499 | 29 | -0.872378546 | PI4KA | phosphatidylinositol 4-kinase alpha [Source:HGNC Symbol;Acc:HGNC:8983] |
| ENSG00000248167 | HGLibA_51487 | 29 | -0.872378546 | TRIM39-RPP21 | TRIM39-RPP21 readthrough [Source:HGNC Symbol;Acc:HGNC:38845] |
| ENSG00000253305 | HGLibA_35566 | 29 | -0.872378546 | PCDHGB6 | 6 [Source:HGNC Symbol;Acc:HGNC:8713]" |
| ENSG00000256713 | HGLibA_36192 | 29 | -0.872378546 | PGA5 | group I (pepsinogen A) [Source:HGNC Symbol;Acc:HGNC:8887]" |
| ENSG00000256977 | HGLibA_26552 | 29 | -0.872378546 | LIMS3 | LIM zinc finger domain containing 3 [Source:HGNC Symbol;Acc:HGNC:30047] |
| ENSG00000264364 | HGLibA_14257 | 29 | -0.872378546 | DYNLL2 | dynein light chain LC8-type 2 [Source:HGNC Symbol;Acc:HGNC:24596] |
| ENSG00000269096 | HGLibA_11505 | 29 | -0.872378546 | CT45A3 | cancer/testis antigen family 45 member A3 [Source:HGNC Symbol;Acc:HGNC:33268] |
| ENSG00000272414 | HGLibA_16621 | 29 | -0.872378546 | FAM47E-STBD1 | FAM47E-STBD1 readthrough [Source:HGNC Symbol;Acc:HGNC:44667] |
| ENSG00000277258 | HGLibA_35595 | 29 | -0.872378546 | PCGF2 | polycomb group ring finger 2 [Source:HGNC Symbol;Acc:HGNC:12929] |
| FAM64A | HGLibA_16664 | 29 | -0.872378546 | FAM64A | - |
| GAGE2B | HGLibA_18470 | 29 | -0.872378546 | GAGE2B | - |
| GARS | HGLibA_18634 | 29 | -0.872378546 | GARS | - |
| HIATL1 | HGLibA_21278 | 29 | -0.872378546 | HIATL1 | - |
| HIST1H2AH | HGLibA_21393 | 29 | -0.872378546 | HIST1H2AH | - |
| HIST1H2BC | HGLibA_21417 | 29 | -0.872378546 | HIST1H2BC | - |
| hsa-mir-1233-1 | HGLibA_57321 | 29 | -0.872378546 | hsa-mir-1233-1 | - |
| hsa-mir-1236 | HGLibA_57333 | 29 | -0.872378546 | hsa-mir-1236 | - |
| hsa-mir-1244-3 | HGLibA_57369 | 29 | -0.872378546 | hsa-mir-1244-3 | - |
| hsa-mir-1306 | HGLibA_57741 | 29 | -0.872378546 | hsa-mir-1306 | - |
| hsa-mir-1306 | HGLibA_57738 | 29 | -0.872378546 | hsa-mir-1306 | - |
| hsa-mir-136 | HGLibA_57803 | 29 | -0.872378546 | hsa-mir-136 | - |
| hsa-mir-153-1 | HGLibA_57909 | 29 | -0.872378546 | hsa-mir-153-1 | - |
| hsa-mir-203a | HGLibA_58166 | 29 | -0.872378546 | hsa-mir-203a | - |
| hsa-mir-2054 | HGLibA_58188 | 29 | -0.872378546 | hsa-mir-2054 | - |
| hsa-mir-20b | HGLibA_58205 | 29 | -0.872378546 | hsa-mir-20b | - |
| hsa-mir-2117 | HGLibA_58239 | 29 | -0.872378546 | hsa-mir-2117 | - |
| hsa-mir-223 | HGLibA_58300 | 29 | -0.872378546 | hsa-mir-223 | - |
| hsa-mir-3670-1 | HGLibA_59234 | 29 | -0.872378546 | hsa-mir-3670-1 | - |
| hsa-mir-376b | HGLibA_59413 | 29 | -0.872378546 | hsa-mir-376b | - |
| hsa-mir-377 | HGLibA_59422 | 29 | -0.872378546 | hsa-mir-377 | - |
| hsa-mir-3978 | HGLibA_59672 | 29 | -0.872378546 | hsa-mir-3978 | - |
| hsa-mir-4315-1 | HGLibA_59977 | 29 | -0.872378546 | hsa-mir-4315-1 | - |
| hsa-mir-4452 | HGLibA_60215 | 29 | -0.872378546 | hsa-mir-4452 | - |
| hsa-mir-4494 | HGLibA_60394 | 29 | -0.872378546 | hsa-mir-4494 | - |
| hsa-mir-4503 | HGLibA_60438 | 29 | -0.872378546 | hsa-mir-4503 | - |
| hsa-mir-451b | HGLibA_60527 | 29 | -0.872378546 | hsa-mir-451b | - |
| hsa-mir-4668 | HGLibA_60790 | 29 | -0.872378546 | hsa-mir-4668 | - |
| hsa-mir-4673 | HGLibA_60807 | 29 | -0.872378546 | hsa-mir-4673 | - |
| hsa-mir-4696 | HGLibA_60892 | 29 | -0.872378546 | hsa-mir-4696 | - |
| hsa-mir-4731 | HGLibA_61022 | 29 | -0.872378546 | hsa-mir-4731 | - |
| hsa-mir-4750 | HGLibA_61099 | 29 | -0.872378546 | hsa-mir-4750 | - |
| hsa-mir-4765 | HGLibA_61158 | 29 | -0.872378546 | hsa-mir-4765 | - |
| hsa-mir-523 | HGLibA_61777 | 29 | -0.872378546 | hsa-mir-523 | - |
| hsa-mir-5690 | HGLibA_62280 | 29 | -0.872378546 | hsa-mir-5690 | - |
| hsa-mir-5708 | HGLibA_62369 | 29 | -0.872378546 | hsa-mir-5708 | - |
| hsa-mir-6133 | HGLibA_62683 | 29 | -0.872378546 | hsa-mir-6133 | - |
| hsa-mir-6501 | HGLibA_62855 | 29 | -0.872378546 | hsa-mir-6501 | - |
| hsa-mir-652 | HGLibA_62942 | 29 | -0.872378546 | hsa-mir-652 | - |
| hsa-mir-6743 | HGLibA_63125 | 29 | -0.872378546 | hsa-mir-6743 | - |
| hsa-mir-6866 | HGLibA_63648 | 29 | -0.872378546 | hsa-mir-6866 | - |
| hsa-mir-769 | HGLibA_63935 | 29 | -0.872378546 | hsa-mir-769 | - |
| KIAA1841 | HGLibA_24823 | 29 | -0.872378546 | KIAA1841 | - |
| LOC100144595 | HGLibA_26812 | 29 | -0.872378546 | LOC100144595 | - |
| NUTMF | HGLibA_33113 | 29 | -0.872378546 | NUTMF | - |
| PVRL2 | HGLibA_39624 | 29 | -0.872378546 | PVRL2 | - |
| STRA13 | HGLibA_47564 | 29 | -0.872378546 | STRA13 | - |
| TTC40 | HGLibA_52143 | 29 | -0.872378546 | TTC40 | - |
| WDR78 | HGLibA_54184 | 29 | -0.872378546 | WDR78 | - |
| WHSC1L1 | HGLibA_54295 | 29 | -0.872378546 | WHSC1L1 | - |
| C12orf52 | HGLibA_05262 | 28 | -0.881423888 | C12orf52 | - |
| C3orf27 | HGLibA_06225 | 28 | -0.881423888 | C3orf27 | - |
| CCDC129 | HGLibA_07586 | 28 | -0.881423888 | CCDC129 | - |
| CENPC1 | HGLibA_09048 | 28 | -0.881423888 | CENPC1 | - |
| ENSG00000005381 | HGLibA_29745 | 28 | -0.881423888 | MPO | myeloperoxidase [Source:HGNC Symbol;Acc:HGNC:7218] |
| ENSG00000010327 | HGLibA_47306 | 28 | -0.881423888 | STAB1 | stabilin 1 [Source:HGNC Symbol;Acc:HGNC:18628] |
| ENSG00000013441 | HGLibA_10054 | 28 | -0.881423888 | CLK1 | CDC like kinase 1 [Source:HGNC Symbol;Acc:HGNC:2068] |
| ENSG00000023734 | HGLibA_47579 | 28 | -0.881423888 | STRAP | serine/threonine kinase receptor associated protein [Source:HGNC Symbol;Acc:HGNC:30796] |
| ENSG00000034533 | HGLibA_03375 | 28 | -0.881423888 | ASTE1 | asteroid homolog 1 [Source:HGNC Symbol;Acc:HGNC:25021] |
| ENSG00000049247 | HGLibA_53460 | 28 | -0.881423888 | UTS2 | urotensin 2 [Source:HGNC Symbol;Acc:HGNC:12636] |
| ENSG00000055917 | HGLibA_39583 | 28 | -0.881423888 | PUM2 | pumilio RNA binding family member 2 [Source:HGNC Symbol;Acc:HGNC:14958] |
| ENSG00000061938 | HGLibA_50847 | 28 | -0.881423888 | TNK2 | tyrosine kinase non receptor 2 [Source:HGNC Symbol;Acc:HGNC:19297] |
| ENSG00000061987 | HGLibA_29645 | 28 | -0.881423888 | MON2 | regulator of endosome-to-Golgi trafficking [Source:HGNC Symbol;Acc:HGNC:29177]" |
| ENSG00000067533 | HGLibA_42272 | 28 | -0.881423888 | RRP15 | ribosomal RNA processing 15 homolog [Source:HGNC Symbol;Acc:HGNC:24255] |
| ENSG00000068489 | HGLibA_38771 | 28 | -0.881423888 | PRR11 | proline rich 11 [Source:HGNC Symbol;Acc:HGNC:25619] |
| ENSG00000069399 | HGLibA_04330 | 28 | -0.881423888 | BCL3 | B cell CLL/lymphoma 3 [Source:HGNC Symbol;Acc:HGNC:998] |
| ENSG00000073350 | HGLibA_26652 | 28 | -0.881423888 | LLGL2 | scribble cell polarity complex component [Source:HGNC Symbol;Acc:HGNC:6629]" |
| ENSG00000073350 | HGLibA_26651 | 28 | -0.881423888 | LLGL2 | scribble cell polarity complex component [Source:HGNC Symbol;Acc:HGNC:6629]" |
| ENSG00000075651 | HGLibA_37036 | 28 | -0.881423888 | PLD1 | phospholipase D1 [Source:HGNC Symbol;Acc:HGNC:9067] |
| ENSG00000083123 | HGLibA_04281 | 28 | -0.881423888 | BCKDHB | branched chain keto acid dehydrogenase E1 subunit beta [Source:HGNC Symbol;Acc:HGNC:987] |
| ENSG00000083290 | HGLibA_52971 | 28 | -0.881423888 | ULK2 | unc-51 like autophagy activating kinase 2 [Source:HGNC Symbol;Acc:HGNC:13480] |
| ENSG00000088280 | HGLibA_03201 | 28 | -0.881423888 | ASAP3 | ankyrin repeat and PH domain 3 [Source:HGNC Symbol;Acc:HGNC:14987]" |
| ENSG00000091986 | HGLibA_07878 | 28 | -0.881423888 | CCDC80 | coiled-coil domain containing 80 [Source:HGNC Symbol;Acc:HGNC:30649] |
| ENSG00000099204 | HGLibA_00307 | 28 | -0.881423888 | ABLIM1 | actin binding LIM protein 1 [Source:HGNC Symbol;Acc:HGNC:78] |
| ENSG00000100030 | HGLibA_28246 | 28 | -0.881423888 | MAPK1 | mitogen-activated protein kinase 1 [Source:HGNC Symbol;Acc:HGNC:6871] |
| ENSG00000100099 | HGLibA_22052 | 28 | -0.881423888 | HPS4 | biogenesis of lysosomal organelles complex 3 subunit 2 [Source:HGNC Symbol;Acc:HGNC:15844]" |
| ENSG00000101986 | HGLibA_00192 | 28 | -0.881423888 | ABCD1 | ATP binding cassette subfamily D member 1 [Source:HGNC Symbol;Acc:HGNC:61] |
| ENSG00000102024 | HGLibA_37228 | 28 | -0.881423888 | PLS3 | plastin 3 [Source:HGNC Symbol;Acc:HGNC:9091] |
| ENSG00000102901 | HGLibA_09086 | 28 | -0.881423888 | CENPT | centromere protein T [Source:HGNC Symbol;Acc:HGNC:25787] |
| ENSG00000103154 | HGLibA_31586 | 28 | -0.881423888 | NECAB2 | N-terminal EF-hand calcium binding protein 2 [Source:HGNC Symbol;Acc:HGNC:23746] |
| ENSG00000103241 | HGLibA_17865 | 28 | -0.881423888 | FOXF1 | forkhead box F1 [Source:HGNC Symbol;Acc:HGNC:3809] |
| ENSG00000104450 | HGLibA_46343 | 28 | -0.881423888 | SPAG1 | sperm associated antigen 1 [Source:HGNC Symbol;Acc:HGNC:11212] |
| ENSG00000104497 | HGLibA_46046 | 28 | -0.881423888 | SNX16 | sorting nexin 16 [Source:HGNC Symbol;Acc:HGNC:14980] |
| ENSG00000104783 | HGLibA_24362 | 28 | -0.881423888 | KCNN4 | potassium calcium-activated channel subfamily N member 4 [Source:HGNC Symbol;Acc:HGNC:6293] |
| ENSG00000105519 | HGLibA_07197 | 28 | -0.881423888 | CAPS | calcyphosine [Source:HGNC Symbol;Acc:HGNC:1487] |
| ENSG00000106415 | HGLibA_19229 | 28 | -0.881423888 | GLCCI1 | glucocorticoid induced 1 [Source:HGNC Symbol;Acc:HGNC:18713] |
| ENSG00000106636 | HGLibA_54693 | 28 | -0.881423888 | YKT6 | YKT6 v-SNARE homolog [Source:HGNC Symbol;Acc:HGNC:16959] |
| ENSG00000106803 | HGLibA_43253 | 28 | -0.881423888 | SEC61B | Sec61 translocon beta subunit [Source:HGNC Symbol;Acc:HGNC:16993] |
| ENSG00000107130 | HGLibA_31374 | 28 | -0.881423888 | NCS1 | neuronal calcium sensor 1 [Source:HGNC Symbol;Acc:HGNC:3953] |
| ENSG00000107447 | HGLibA_13716 | 28 | -0.881423888 | DNTT | DNA nucleotidylexotransferase [Source:HGNC Symbol;Acc:HGNC:2983] |
| ENSG00000108821 | HGLibA_10529 | 28 | -0.881423888 | COL1A1 | collagen type I alpha 1 chain [Source:HGNC Symbol;Acc:HGNC:2197] |
| ENSG00000109047 | HGLibA_40721 | 28 | -0.881423888 | RCVRN | recoverin [Source:HGNC Symbol;Acc:HGNC:9937] |
| ENSG00000109099 | HGLibA_37325 | 28 | -0.881423888 | PMP22 | peripheral myelin protein 22 [Source:HGNC Symbol;Acc:HGNC:9118] |
| ENSG00000109618 | HGLibA_43423 | 28 | -0.881423888 | SEPSECS | Sep (O-phosphoserine) tRNA:Sec (selenocysteine) tRNA synthase [Source:HGNC Symbol;Acc:HGNC:30605] |
| ENSG00000111530 | HGLibA_07121 | 28 | -0.881423888 | CAND1 | cullin associated and neddylation dissociated 1 [Source:HGNC Symbol;Acc:HGNC:30688] |
| ENSG00000111647 | HGLibA_52950 | 28 | -0.881423888 | UHRF1BP1L | UHRF1 binding protein 1 like [Source:HGNC Symbol;Acc:HGNC:29102] |
| ENSG00000112333 | HGLibA_32603 | 28 | -0.881423888 | NR2E1 | nuclear receptor subfamily 2 group E member 1 [Source:HGNC Symbol;Acc:HGNC:7973] |
| ENSG00000112837 | HGLibA_48650 | 28 | -0.881423888 | TBX18 | T-box 18 [Source:HGNC Symbol;Acc:HGNC:11595] |
| ENSG00000112992 | HGLibA_32225 | 28 | -0.881423888 | NNT | nicotinamide nucleotide transhydrogenase [Source:HGNC Symbol;Acc:HGNC:7863] |
| ENSG00000113068 | HGLibA_36137 | 28 | -0.881423888 | PFDN1 | prefoldin subunit 1 [Source:HGNC Symbol;Acc:HGNC:8866] |
| ENSG00000113318 | HGLibA_30192 | 28 | -0.881423888 | MSH3 | mutS homolog 3 [Source:HGNC Symbol;Acc:HGNC:7326] |
| ENSG00000114982 | HGLibA_24046 | 28 | -0.881423888 | KANSL3 | KAT8 regulatory NSL complex subunit 3 [Source:HGNC Symbol;Acc:HGNC:25473] |
| ENSG00000115274 | HGLibA_23321 | 28 | -0.881423888 | INO80B | INO80 complex subunit B [Source:HGNC Symbol;Acc:HGNC:13324] |
| ENSG00000119321 | HGLibA_17560 | 28 | -0.881423888 | FKBP15 | FK506 binding protein 15 [Source:HGNC Symbol;Acc:HGNC:23397] |
| ENSG00000119715 | HGLibA_15595 | 28 | -0.881423888 | ESRRB | estrogen related receptor beta [Source:HGNC Symbol;Acc:HGNC:3473] |
| ENSG00000120160 | HGLibA_15399 | 28 | -0.881423888 | EQTN | equatorin [Source:HGNC Symbol;Acc:HGNC:1359] |
| ENSG00000120314 | HGLibA_54128 | 28 | -0.881423888 | WDR55 | WD repeat domain 55 [Source:HGNC Symbol;Acc:HGNC:25971] |
| ENSG00000120324 | HGLibA_35472 | 28 | -0.881423888 | PCDHB10 | protocadherin beta 10 [Source:HGNC Symbol;Acc:HGNC:8681] |
| ENSG00000120437 | HGLibA_00394 | 28 | -0.881423888 | ACAT2 | acetyl-CoA acetyltransferase 2 [Source:HGNC Symbol;Acc:HGNC:94] |
| ENSG00000121104 | HGLibA_16043 | 28 | -0.881423888 | FAM117A | family with sequence similarity 117 member A [Source:HGNC Symbol;Acc:HGNC:24179] |
| ENSG00000121542 | HGLibA_43215 | 28 | -0.881423888 | SEC22A | vesicle trafficking protein [Source:HGNC Symbol;Acc:HGNC:20260]" |
| ENSG00000121644 | HGLibA_13011 | 28 | -0.881423888 | DESI2 | desumoylating isopeptidase 2 [Source:HGNC Symbol;Acc:HGNC:24264] |
| ENSG00000122515 | HGLibA_55384 | 28 | -0.881423888 | ZMIZ2 | zinc finger MIZ-type containing 2 [Source:HGNC Symbol;Acc:HGNC:22229] |
| ENSG00000124193 | HGLibA_47084 | 28 | -0.881423888 | SRSF6 | serine and arginine rich splicing factor 6 [Source:HGNC Symbol;Acc:HGNC:10788] |
| ENSG00000124198 | HGLibA_02672 | 28 | -0.881423888 | ARFGEF2 | ADP ribosylation factor guanine nucleotide exchange factor 2 [Source:HGNC Symbol;Acc:HGNC:15853] |

| | | | | | |
|---|---|---|---|---|---|
| ENSG00000124935 | HGLibA_42918 | 28 | -0.881423888 | SCGB1D2 | secretoglobin family 1D member 2 [Source:HGNC Symbol;Acc:HGNC:18396] |
| ENSG00000125450 | HGLibA_33068 | 28 | -0.881423888 | NUP85 | nucleoporin 85 [Source:HGNC Symbol;Acc:HGNC:8734] |
| ENSG00000125772 | HGLibA_19780 | 28 | -0.881423888 | GPCPD1 | glycerophosphocholine phosphodiesterase 1 [Source:HGNC Symbol;Acc:HGNC:26957] |
| ENSG00000125827 | HGLibA_50698 | 28 | -0.881423888 | TMX4 | thioredoxin related transmembrane protein 4 [Source:HGNC Symbol;Acc:HGNC:25237] |
| ENSG00000126698 | HGLibA_13640 | 28 | -0.881423888 | DNAJC8 | DnaJ heat shock protein family (Hsp40) member C8 [Source:HGNC Symbol;Acc:HGNC:15470] |
| ENSG00000128604 | HGLibA_23591 | 28 | -0.881423888 | IRF5 | interferon regulatory factor 5 [Source:HGNC Symbol;Acc:HGNC:6120] |
| ENSG00000128881 | HGLibA_52043 | 28 | -0.881423888 | TTBK2 | tau tubulin kinase 2 [Source:HGNC Symbol;Acc:HGNC:19141] |
| ENSG00000129048 | HGLibA_00449 | 28 | -0.881423888 | ACKR4 | atypical chemokine receptor 4 [Source:HGNC Symbol;Acc:HGNC:1611] |
| ENSG00000129158 | HGLibA_43477 | 28 | -0.881423888 | SERGEF | secretion regulating guanine nucleotide exchange factor [Source:HGNC Symbol;Acc:HGNC:17499] |
| ENSG00000130299 | HGLibA_20607 | 28 | -0.881423888 | GTPBP3 | mitochondrial [Source:HGNC Symbol;Acc:HGNC:14880]" |
| ENSG00000130584 | HGLibA_54899 | 28 | -0.881423888 | ZBTB46 | zinc finger and BTB domain containing 46 [Source:HGNC Symbol;Acc:HGNC:16094] |
| ENSG00000131023 | HGLibA_26065 | 28 | -0.881423888 | LATS1 | large tumor suppressor kinase 1 [Source:HGNC Symbol;Acc:HGNC:6514] |
| ENSG00000131697 | HGLibA_32450 | 28 | -0.881423888 | NPHP4 | nephrocystin 4 [Source:HGNC Symbol;Acc:HGNC:19104] |
| ENSG00000132855 | HGLibA_01916 | 28 | -0.881423888 | ANGPTL3 | angiopoietin like 3 [Source:HGNC Symbol;Acc:HGNC:491] |
| ENSG00000133983 | HGLibA_10814 | 28 | -0.881423888 | COX16 | cytochrome c oxidase assembly factor COX16 [Source:HGNC Symbol;Acc:HGNC:20213] |
| ENSG00000134940 | HGLibA_00540 | 28 | -0.881423888 | ACRV1 | acrosomal vesicle protein 1 [Source:HGNC Symbol;Acc:HGNC:127] |
| ENSG00000135916 | HGLibA_23836 | 28 | -0.881423888 | ITM2C | integral membrane protein 2C [Source:HGNC Symbol;Acc:HGNC:6175] |
| ENSG00000135976 | HGLibA_02087 | 28 | -0.881423888 | ANKRD36 | ankyrin repeat domain 36 [Source:HGNC Symbol;Acc:HGNC:24079] |
| ENSG00000136155 | HGLibA_42890 | 28 | -0.881423888 | SCEL | sciellin [Source:HGNC Symbol;Acc:HGNC:10573] |
| ENSG00000136160 | HGLibA_14482 | 28 | -0.881423888 | EDNRB | endothelin receptor type B [Source:HGNC Symbol;Acc:HGNC:3180] |
| ENSG00000137460 | HGLibA_17474 | 28 | -0.881423888 | FHDC1 | FH2 domain containing 1 [Source:HGNC Symbol;Acc:HGNC:29363] |
| ENSG00000137727 | HGLibA_02723 | 28 | -0.881423888 | ARHGAP20 | Rho GTPase activating protein 20 [Source:HGNC Symbol;Acc:HGNC:18357] |
| ENSG00000137822 | HGLibA_52314 | 28 | -0.881423888 | TUBGCP4 | tubulin gamma complex associated protein 4 [Source:HGNC Symbol;Acc:HGNC:16691] |
| ENSG00000138592 | HGLibA_53413 | 28 | -0.881423888 | USP8 | ubiquitin specific peptidase 8 [Source:HGNC Symbol;Acc:HGNC:12631] |
| ENSG00000139219 | HGLibA_10561 | 28 | -0.881423888 | COL2A1 | collagen type II alpha 1 chain [Source:HGNC Symbol;Acc:HGNC:2200] |
| ENSG00000139874 | HGLibA_47175 | 28 | -0.881423888 | SSTR1 | somatostatin receptor 1 [Source:HGNC Symbol;Acc:HGNC:11330] |
| ENSG00000140391 | HGLibA_51925 | 28 | -0.881423888 | TSPAN3 | tetraspanin 3 [Source:HGNC Symbol;Acc:HGNC:17752] |
| ENSG00000141401 | HGLibA_23248 | 28 | -0.881423888 | IMPA2 | inositol monophosphatase 2 [Source:HGNC Symbol;Acc:HGNC:6051] |
| ENSG00000142661 | HGLibA_30896 | 28 | -0.881423888 | MYOM3 | myomesin 3 [Source:HGNC Symbol;Acc:HGNC:26679] |
| ENSG00000143222 | HGLibA_52843 | 28 | -0.881423888 | UFC1 | ubiquitin-fold modifier conjugating enzyme 1 [Source:HGNC Symbol;Acc:HGNC:26941] |
| ENSG00000145194 | HGLibA_14386 | 28 | -0.881423888 | ECE2 | endothelin converting enzyme 2 [Source:HGNC Symbol;Acc:HGNC:13275] |
| ENSG00000145220 | HGLibA_27694 | 28 | -0.881423888 | LYAR | Ly1 antibody reactive [Source:HGNC Symbol;Acc:HGNC:26021] |
| ENSG00000145391 | HGLibA_43659 | 28 | -0.881423888 | SETD7 | SET domain containing lysine methyltransferase 7 [Source:HGNC Symbol;Acc:HGNC:30412] |
| ENSG00000146038 | HGLibA_12483 | 28 | -0.881423888 | DCDC2 | doublecortin domain containing 2 [Source:HGNC Symbol;Acc:HGNC:18141] |
| ENSG00000148156 | HGLibA_00629 | 28 | -0.881423888 | ACTL7B | actin like 7B [Source:HGNC Symbol;Acc:HGNC:162] |
| ENSG00000148677 | HGLibA_01988 | 28 | -0.881423888 | ANKRD1 | ankyrin repeat domain 1 [Source:HGNC Symbol;Acc:HGNC:15819] |
| ENSG00000148840 | HGLibA_38230 | 28 | -0.881423888 | PPRC1 | coactivator-related 1 [Source:HGNC Symbol;Acc:HGNC:30025]" |
| ENSG00000149150 | HGLibA_45148 | 28 | -0.881423888 | SLC43A1 | solute carrier family 43 member 1 [Source:HGNC Symbol;Acc:HGNC:9225] |
| ENSG00000149311 | HGLibA_03523 | 28 | -0.881423888 | ATM | ATM serine/threonine kinase [Source:HGNC Symbol;Acc:HGNC:795] |
| ENSG00000150054 | HGLibA_29764 | 28 | -0.881423888 | MPP7 | membrane palmitoylated protein 7 [Source:HGNC Symbol;Acc:HGNC:26542] |
| ENSG00000150244 | HGLibA_51519 | 28 | -0.881423888 | TRIM48 | tripartite motif containing 48 [Source:HGNC Symbol;Acc:HGNC:19021] |
| ENSG00000150347 | HGLibA_02910 | 28 | -0.881423888 | ARID5B | AT-rich interaction domain 5B [Source:HGNC Symbol;Acc:HGNC:17362] |
| ENSG00000153395 | HGLibA_27071 | 28 | -0.881423888 | LPCAT1 | lysophosphatidylcholine acyltransferase 1 [Source:HGNC Symbol;Acc:HGNC:25718] |
| ENSG00000155906 | HGLibA_41379 | 28 | -0.881423888 | RMND1 | required for meiotic nuclear division 1 homolog [Source:HGNC Symbol;Acc:HGNC:21176] |
| ENSG00000155975 | HGLibA_53776 | 28 | -0.881423888 | VPS37A | ESCRT-I subunit [Source:HGNC Symbol;Acc:HGNC:24928]" |
| ENSG00000157617 | HGLibA_06087 | 28 | -0.881423888 | C2CD2 | C2 calcium dependent domain containing 2 [Source:HGNC Symbol;Acc:HGNC:1266] |
| ENSG00000160219 | HGLibA_18327 | 28 | -0.881423888 | GAB3 | GRB2 associated binding protein 3 [Source:HGNC Symbol;Acc:HGNC:17515] |
| ENSG00000161682 | HGLibA_16267 | 28 | -0.881423888 | FAM171A2 | family with sequence similarity 171 member A2 [Source:HGNC Symbol;Acc:HGNC:30480] |
| ENSG00000162772 | HGLibA_03438 | 28 | -0.881423888 | ATF3 | activating transcription factor 3 [Source:HGNC Symbol;Acc:HGNC:785] |
| ENSG00000163071 | HGLibA_46438 | 28 | -0.881423888 | SPATA18 | spermatogenesis associated 18 [Source:HGNC Symbol;Acc:HGNC:29579] |
| ENSG00000163331 | HGLibA_12332 | 28 | -0.881423888 | DAPL1 | death associated protein like 1 [Source:HGNC Symbol;Acc:HGNC:21490] |
| ENSG00000163501 | HGLibA_22909 | 28 | -0.881423888 | IHH | indian hedgehog [Source:HGNC Symbol;Acc:HGNC:5956] |
| ENSG00000164287 | HGLibA_08522 | 28 | -0.881423888 | CDC20B | cell division cycle 20B [Source:HGNC Symbol;Acc:HGNC:24222] |
| ENSG00000164989 | HGLibA_07695 | 28 | -0.881423888 | CCDC171 | coiled-coil domain containing 171 [Source:HGNC Symbol;Acc:HGNC:29828] |
| ENSG00000165370 | HGLibA_19839 | 28 | -0.881423888 | GPR101 | G protein-coupled receptor 101 [Source:HGNC Symbol;Acc:HGNC:14963] |
| ENSG00000165556 | HGLibA_08900 | 28 | -0.881423888 | CDX2 | caudal type homeobox 2 [Source:HGNC Symbol;Acc:HGNC:1806] |
| ENSG00000165943 | HGLibA_29583 | 28 | -0.881423888 | MOAP1 | modulator of apoptosis 1 [Source:HGNC Symbol;Acc:HGNC:16658] |
| ENSG00000166046 | HGLibA_48847 | 28 | -0.881423888 | TCP11L2 | t-complex 11 like 2 [Source:HGNC Symbol;Acc:HGNC:28627] |
| ENSG00000166311 | HGLibA_45802 | 28 | -0.881423888 | SMPD1 | sphingomyelin phosphodiesterase 1 [Source:HGNC Symbol;Acc:HGNC:11120] |
| ENSG00000166411 | HGLibA_22544 | 28 | -0.881423888 | IDH3A | isocitrate dehydrogenase 3 (NAD(+)) alpha [Source:HGNC Symbol;Acc:HGNC:5384] |
| ENSG00000166938 | HGLibA_13273 | 28 | -0.881423888 | DIS3L | DIS3 like exosome 3'-5' exoribonuclease [Source:HGNC Symbol;Acc:HGNC:28698] |
| ENSG00000167258 | HGLibA_08732 | 28 | -0.881423888 | CDK12 | cyclin dependent kinase 12 [Source:HGNC Symbol;Acc:HGNC:24224] |
| ENSG00000167550 | HGLibA_41157 | 28 | -0.881423888 | RHEBL1 | RHEB like 1 [Source:HGNC Symbol;Acc:HGNC:21166] |
| ENSG00000167967 | HGLibA_14332 | 28 | -0.881423888 | E4F1 | E4F transcription factor 1 [Source:HGNC Symbol;Acc:HGNC:3121] |
| ENSG00000168754 | HGLibA_16304 | 28 | -0.881423888 | FAM178B | family with sequence similarity 178 member B [Source:HGNC Symbol;Acc:HGNC:28036] |
| ENSG00000168818 | HGLibA_47629 | 28 | -0.881423888 | STX18 | syntaxin 18 [Source:HGNC Symbol;Acc:HGNC:15942] |
| ENSG00000168890 | HGLibA_49990 | 28 | -0.881423888 | TMEM150A | transmembrane protein 150A [Source:HGNC Symbol;Acc:HGNC:24677] |
| ENSG00000169064 | HGLibA_54783 | 28 | -0.881423888 | ZBBX | zinc finger B-box domain containing [Source:HGNC Symbol;Acc:HGNC:26245] |
| ENSG00000169570 | HGLibA_14077 | 28 | -0.881423888 | DTWD2 | DTW domain containing 2 [Source:HGNC Symbol;Acc:HGNC:19334] |
| ENSG00000169717 | HGLibA_00680 | 28 | -0.881423888 | ACTRT2 | actin related protein T2 [Source:HGNC Symbol;Acc:HGNC:24026] |
| ENSG00000169718 | HGLibA_14111 | 28 | -0.881423888 | DUS1L | dihydrouridine synthase 1 like [Source:HGNC Symbol;Acc:HGNC:30086] |
| ENSG00000171049 | HGLibA_17966 | 28 | -0.881423888 | FPR2 | formyl peptide receptor 2 [Source:HGNC Symbol;Acc:HGNC:3827] |
| ENSG00000171804 | HGLibA_54204 | 28 | -0.881423888 | WDR87 | WD repeat domain 87 [Source:HGNC Symbol;Acc:HGNC:29934] |
| ENSG00000172057 | HGLibA_34630 | 28 | -0.881423888 | ORMDL3 | ORMDL sphingolipid biosynthesis regulator 3 [Source:HGNC Symbol;Acc:HGNC:16038] |
| ENSG00000172732 | HGLibA_30575 | 28 | -0.881423888 | MUS81 | MUS81 structure-specific endonuclease subunit [Source:HGNC Symbol;Acc:HGNC:29814] |
| ENSG00000173166 | HGLibA_40265 | 28 | -0.881423888 | RAPH1 | Ras association (RalGDS/AF-6) and pleckstrin homology domains 1 [Source:HGNC Symbol;Acc:HGNC:14436] |
| ENSG00000173610 | HGLibA_52904 | 28 | -0.881423888 | UGT2A1 | UDP glucuronosyltransferase family 2 member A1 complex locus [Source:HGNC Symbol;Acc:HGNC:12542] |
| ENSG00000174444 | HGLibA_41981 | 28 | -0.881423888 | RPL4 | ribosomal protein L4 [Source:HGNC Symbol;Acc:HGNC:10353] |
| ENSG00000174705 | HGLibA_43966 | 28 | -0.881423888 | SH3PXD2B | SH3 and PX domains 2B [Source:HGNC Symbol;Acc:HGNC:29242] |
| ENSG00000175155 | HGLibA_54709 | 28 | -0.881423888 | YPEL2 | yippee like 2 [Source:HGNC Symbol;Acc:HGNC:18326] |
| ENSG00000176040 | HGLibA_50650 | 28 | -0.881423888 | TMPRSS7 | transmembrane serine protease 7 [Source:HGNC Symbol;Acc:HGNC:30846] |
| ENSG00000176222 | HGLibA_55924 | 28 | -0.881423888 | ZNF404 | zinc finger protein 404 [Source:HGNC Symbol;Acc:HGNC:19417] |
| ENSG00000176410 | HGLibA_13618 | 28 | -0.881423888 | DNAJC30 | DnaJ heat shock protein family (Hsp40) member C30 [Source:HGNC Symbol;Acc:HGNC:16410] |
| ENSG00000176641 | HGLibA_41551 | 28 | -0.881423888 | RNF152 | ring finger protein 152 [Source:HGNC Symbol;Acc:HGNC:26811] |
| ENSG00000177791 | HGLibA_30900 | 28 | -0.881423888 | MYOZ1 | myozenin 1 [Source:HGNC Symbol;Acc:HGNC:13752] |
| ENSG00000178053 | HGLibA_29372 | 28 | -0.881423888 | MLF1 | myeloid leukemia factor 1 [Source:HGNC Symbol;Acc:HGNC:7125] |
| ENSG00000178084 | HGLibA_22386 | 28 | -0.881423888 | HTR3C | 5-hydroxytryptamine receptor 3C [Source:HGNC Symbol;Acc:HGNC:24003] |
| ENSG00000178585 | HGLibA_11652 | 28 | -0.881423888 | CTNNBIP1 | catenin beta interacting protein 1 [Source:HGNC Symbol;Acc:HGNC:16913] |
| ENSG00000178773 | HGLibA_10972 | 28 | -0.881423888 | CPNE7 | copine 7 [Source:HGNC Symbol;Acc:HGNC:2320] |
| ENSG00000178928 | HGLibA_51193 | 28 | -0.881423888 | TPRX1 | tetrapeptide repeat homeobox 1 [Source:HGNC Symbol;Acc:HGNC:32174] |
| ENSG00000181038 | HGLibA_28990 | 28 | -0.881423888 | METTL23 | methyltransferase like 23 [Source:HGNC Symbol;Acc:HGNC:26988] |
| ENSG00000181333 | HGLibA_21146 | 28 | -0.881423888 | HEPHL1 | hephaestin like 1 [Source:HGNC Symbol;Acc:HGNC:30477] |
| ENSG00000181991 | HGLibA_30033 | 28 | -0.881423888 | MRPS11 | mitochondrial ribosomal protein S11 [Source:HGNC Symbol;Acc:HGNC:14050] |
| ENSG00000182809 | HGLibA_11152 | 28 | -0.881423888 | CRIP2 | cysteine rich protein 2 [Source:HGNC Symbol;Acc:HGNC:2361] |
| ENSG00000183709 | HGLibA_22722 | 28 | -0.881423888 | IFNL2 | interferon lambda 2 [Source:HGNC Symbol;Acc:HGNC:18364] |
| ENSG00000184281 | HGLibA_52007 | 28 | -0.881423888 | TSSC4 | tumor suppressing subtransferable candidate 4 [Source:HGNC Symbol;Acc:HGNC:12386] |
| ENSG00000184863 | HGLibA_40533 | 28 | -0.881423888 | RBM33 | RNA binding motif protein 33 [Source:HGNC Symbol;Acc:HGNC:27223] |
| ENSG00000185420 | HGLibA_45857 | 28 | -0.881423888 | SMYD3 | SET and MYND domain containing 3 [Source:HGNC Symbol;Acc:HGNC:15513] |
| ENSG00000186026 | HGLibA_55745 | 28 | -0.881423888 | ZNF284 | zinc finger protein 284 [Source:HGNC Symbol;Acc:HGNC:13078] |
| ENSG00000186212 | HGLibA_46216 | 28 | -0.881423888 | SOWAHB | sosondowah ankyrin repeat domain family member B [Source:HGNC Symbol;Acc:HGNC:32958] |
| ENSG00000186628 | HGLibA_18083 | 28 | -0.881423888 | FSD2 | fibronectin type III and SPRY domain containing 2 [Source:HGNC Symbol;Acc:HGNC:18024] |
| ENSG00000186766 | HGLibA_17879 | 28 | -0.881423888 | FOXI2 | forkhead box I2 [Source:HGNC Symbol;Acc:HGNC:32448] |
| ENSG00000188039 | HGLibA_33124 | 28 | -0.881423888 | NWD1 | NACHT and WD repeat domain containing 1 [Source:HGNC Symbol;Acc:HGNC:27619] |
| ENSG00000188624 | HGLibA_22844 | 28 | -0.881423888 | IGFL3 | IGF like family member 3 [Source:HGNC Symbol;Acc:HGNC:32930] |
| ENSG00000188641 | HGLibA_13920 | 28 | -0.881423888 | DPYD | dihydropyrimidine dehydrogenase [Source:HGNC Symbol;Acc:HGNC:3012] |
| ENSG00000196199 | HGLibA_29728 | 28 | -0.881423888 | MPHOSPH8 | M-phase phosphoprotein 8 [Source:HGNC Symbol;Acc:HGNC:29810] |
| ENSG00000196507 | HGLibA_48709 | 28 | -0.881423888 | TCEAL3 | transcription elongation factor A like 3 [Source:HGNC Symbol;Acc:HGNC:28247] |
| ENSG00000196591 | HGLibA_21006 | 28 | -0.881423888 | HDAC2 | histone deacetylase 2 [Source:HGNC Symbol;Acc:HGNC:4853] |
| ENSG00000196683 | HGLibA_50978 | 28 | -0.881423888 | TOMM7 | translocase of outer mitochondrial membrane 7 [Source:HGNC Symbol;Acc:HGNC:21648] |
| ENSG00000197084 | HGLibA_26106 | 28 | -0.881423888 | LCE1C | late cornified envelope 1C [Source:HGNC Symbol;Acc:HGNC:29464] |
| ENSG00000197157 | HGLibA_45919 | 28 | -0.881423888 | SND1 | staphylococcal nuclease and tudor domain containing 1 [Source:HGNC Symbol;Acc:HGNC:30646] |
| ENSG00000197208 | HGLibA_44588 | 28 | -0.881423888 | SLC22A4 | solute carrier family 22 member 4 [Source:HGNC Symbol;Acc:HGNC:10968] |
| ENSG00000198056 | HGLibA_38482 | 28 | -0.881423888 | PRIM1 | DNA primase subunit 1 [Source:HGNC Symbol;Acc:HGNC:9369] |
| ENSG00000198183 | HGLibA_04709 | 28 | -0.881423888 | BPIFA1 | BPI fold containing family A member 1 [Source:HGNC Symbol;Acc:HGNC:15749] |
| ENSG00000198853 | HGLibA_42469 | 28 | -0.881423888 | RUSC2 | RUN and SH3 domain containing 2 [Source:HGNC Symbol;Acc:HGNC:23625] |
| ENSG00000203908 | HGLibA_24563 | 28 | -0.881423888 | KHDC3L | subcortical maternal complex member [Source:HGNC Symbol;Acc:HGNC:33699]" |
| ENSG00000204065 | HGLibA_48717 | 28 | -0.881423888 | TCEAL5 | transcription elongation factor A like 5 [Source:HGNC Symbol;Acc:HGNC:22282] |
| ENSG00000204116 | HGLibA_09446 | 28 | -0.881423888 | CHIC1 | cysteine rich hydrophobic domain 1 [Source:HGNC Symbol;Acc:HGNC:1934] |
| ENSG00000204909 | HGLibA_46710 | 28 | -0.881423888 | SPINK9 | Kazal type 9 [Source:HGNC Symbol;Acc:HGNC:32951]" |
| ENSG00000205542 | HGLibA_50666 | 28 | -0.881423888 | TMSB4X | thymosin beta 4 X-linked [Source:HGNC Symbol;Acc:HGNC:11881] |
| ENSG00000213015 | HGLibA_56304 | 28 | -0.881423888 | ZNF580 | zinc finger protein 580 [Source:HGNC Symbol;Acc:HGNC:29473] |
| ENSG00000213901 | HGLibA_44612 | 28 | -0.881423888 | SLC23A3 | solute carrier family 23 member 3 [Source:HGNC Symbol;Acc:HGNC:20601] |
| ENSG00000214456 | HGLibA_37182 | 28 | -0.881423888 | PLIN5 | perilipin 5 [Source:HGNC Symbol;Acc:HGNC:33196] |
| ENSG00000215262 | HGLibA_24399 | 28 | -0.881423888 | KCNU1 | potassium calcium-activated channel subfamily U member 1 [Source:HGNC Symbol;Acc:HGNC:18867] |
| ENSG00000221859 | HGLibA_25594 | 28 | -0.881423888 | KRTAP10-10 | keratin associated protein 10-10 [Source:HGNC Symbol;Acc:HGNC:22972] |
| ENSG00000221864 | HGLibA_25634 | 28 | -0.881423888 | KRTAP12-2 | keratin associated protein 12-2 [Source:HGNC Symbol;Acc:HGNC:20530] |
| ENSG00000221977 | HGLibA_33987 | 28 | -0.881423888 | OR4E2 | olfactory receptor family 4 subfamily E member 2 [Source:HGNC Symbol;Acc:HGNC:8297] |
| ENSG00000224712 | HGLibA_32468 | 28 | -0.881423888 | NPIPA3 | nuclear pore complex interacting protein family member A3 [Source:HGNC Symbol;Acc:HGNC:41978] |
| ENSG00000228278 | HGLibA_34623 | 28 | -0.881423888 | ORM2 | orosomucoid 2 [Source:HGNC Symbol;Acc:HGNC:8499] |
| ENSG00000240204 | HGLibA_45776 | 28 | -0.881423888 | SMKR1 | small lysine rich protein 1 [Source:HGNC Symbol;Acc:HGNC:43561] |
| ENSG00000243902 | HGLibA_14910 | 28 | -0.881423888 | ELFN2 | extracellular leucine rich repeat and fibronectin type III domain containing 2 [Source:NCBI gene;Acc:114794] |
| ENSG00000249693 | HGLibA_49337 | 28 | -0.881423888 | THEGL | theg spermatid protein like [Source:HGNC Symbol;Acc:HGNC:43771] |
| ENSG00000250506 | HGLibA_08766 | 28 | -0.881423888 | CDK3 | cyclin dependent kinase 3 [Source:HGNC Symbol;Acc:HGNC:1772] |
| ENSG00000253598 | HGLibA_44341 | 28 | -0.881423888 | SLC10A5 | solute carrier family 10 member 5 [Source:HGNC Symbol;Acc:HGNC:22981] |
| ENSG00000270601 | HGLibA_38321 | 28 | -0.881423888 | PRAMEF5 | PRAME family member 5 [Source:HGNC Symbol;Acc:HGNC:27995] |

| | | | | | |
|---|---|---|---|---|---|
| ENSG00000277462 | HGLibA_56499 | 28 | -0.881423888 | ZNF670 | zinc finger protein 670 [Source:HGNC Symbol;Acc:HGNC:28167] |
| GATSL1 | HGLibA_18700 | 28 | -0.881423888 | GATSL1 | - |
| GRASP | HGLibA_20221 | 28 | -0.881423888 | GRASP | - |
| hsa-mir-1237 | HGLibA_57338 | 28 | -0.881423888 | hsa-mir-1237 | - |
| hsa-mir-1305 | HGLibA_57737 | 28 | -0.881423888 | hsa-mir-1305 | - |
| hsa-mir-135a-1 | HGLibA_57793 | 28 | -0.881423888 | hsa-mir-135a-1 | - |
| hsa-mir-195 | HGLibA_58090 | 28 | -0.881423888 | hsa-mir-195 | - |
| hsa-mir-302a | HGLibA_58438 | 28 | -0.881423888 | hsa-mir-302a | - |
| hsa-mir-3156-2 | HGLibA_58691 | 28 | -0.881423888 | hsa-mir-3156-2 | - |
| hsa-mir-3667 | HGLibA_59220 | 28 | -0.881423888 | hsa-mir-3667 | - |
| hsa-mir-3675 | HGLibA_59254 | 28 | -0.881423888 | hsa-mir-3675 | - |
| hsa-mir-3909 | HGLibA_59502 | 28 | -0.881423888 | hsa-mir-3909 | - |
| hsa-mir-3910-1 | HGLibA_59506 | 28 | -0.881423888 | hsa-mir-3910-1 | - |
| hsa-mir-3912 | HGLibA_59515 | 28 | -0.881423888 | hsa-mir-3912 | - |
| hsa-mir-3934 | HGLibA_59598 | 28 | -0.881423888 | hsa-mir-3934 | - |
| hsa-mir-4300 | HGLibA_59913 | 28 | -0.881423888 | hsa-mir-4300 | - |
| hsa-mir-4474 | HGLibA_60308 | 28 | -0.881423888 | hsa-mir-4474 | - |
| hsa-mir-4484 | HGLibA_60354 | 28 | -0.881423888 | hsa-mir-4484 | - |
| hsa-mir-4633 | HGLibA_60636 | 28 | -0.881423888 | hsa-mir-4633 | - |
| hsa-mir-4650-1 | HGLibA_60704 | 28 | -0.881423888 | hsa-mir-4650-1 | - |
| hsa-mir-4746 | HGLibA_61081 | 28 | -0.881423888 | hsa-mir-4746 | - |
| hsa-mir-4789 | HGLibA_61265 | 28 | -0.881423888 | hsa-mir-4789 | - |
| hsa-mir-509-2 | HGLibA_61510 | 28 | -0.881423888 | hsa-mir-509-2 | - |
| hsa-mir-5093 | HGLibA_61532 | 28 | -0.881423888 | hsa-mir-5093 | - |
| hsa-mir-514b | HGLibA_61600 | 28 | -0.881423888 | hsa-mir-514b | - |
| hsa-mir-518a-1 | HGLibA_61651 | 28 | -0.881423888 | hsa-mir-518a-1 | - |
| hsa-mir-5581 | HGLibA_62164 | 28 | -0.881423888 | hsa-mir-5581 | - |
| hsa-mir-5696 | HGLibA_62318 | 28 | -0.881423888 | hsa-mir-5696 | - |
| hsa-mir-631 | HGLibA_62765 | 28 | -0.881423888 | hsa-mir-631 | - |
| hsa-mir-7157 | HGLibA_63855 | 28 | -0.881423888 | hsa-mir-7157 | - |
| hsa-mir-8052 | HGLibA_64046 | 28 | -0.881423888 | hsa-mir-8052 | - |
| hsa-mir-891b | HGLibA_64246 | 28 | -0.881423888 | hsa-mir-891b | - |
| KIAA0226 | HGLibA_24604 | 28 | -0.881423888 | KIAA0226 | - |
| KIAA0368 | HGLibA_24629 | 28 | -0.881423888 | KIAA0368 | - |
| KIAA0391 | HGLibA_24633 | 28 | -0.881423888 | KIAA0391 | - |
| KIAA0556 | HGLibA_24644 | 28 | -0.881423888 | KIAA0556 | - |
| KIAA1377 | HGLibA_24745 | 28 | -0.881423888 | KIAA1377 | - |
| LOC152586 | HGLibA_26893 | 28 | -0.881423888 | LOC152586 | - |
| LOC729020 | HGLibA_26995 | 28 | -0.881423888 | LOC729020 | - |
| LRRC6 | HGLibA_27403 | 28 | -0.881423888 | LRRC6 | - |
| RARS | HGLibA_40291 | 28 | -0.881423888 | RARS | - |
| WBP5 | HGLibA_53987 | 28 | -0.881423888 | WBP5 | - |
| ACPP | HGLibA_00523 | 27 | -0.890469231 | ACPP | - |
| BET3L | HGLibA_04436 | 27 | -0.890469231 | BET3L | - |
| C16orf58 | HGLibA_05461 | 27 | -0.890469231 | C16orf58 | - |
| C17orf102 | HGLibA_05519 | 27 | -0.890469231 | C17orf102 | - |
| C1orf27 | HGLibA_05851 | 27 | -0.890469231 | C1orf27 | - |
| C6orf222 | HGLibA_06476 | 27 | -0.890469231 | C6orf222 | - |
| CASC5 | HGLibA_07280 | 27 | -0.890469231 | CASC5 | - |
| CYP3A7-CYP3AP1 | HGLibA_12167 | 27 | -0.890469231 | CYP3A7-CYP3AP1 | - |
| ENSG00000007312 | HGLibA_08449 | 27 | -0.890469231 | CD79B | CD79b molecule [Source:HGNC Symbol;Acc:HGNC:1699] |
| ENSG00000008197 | HGLibA_49129 | 27 | -0.890469231 | TFAP2D | transcription factor AP-2 delta [Source:HGNC Symbol;Acc:HGNC:15581] |
| ENSG00000008853 | HGLibA_41174 | 27 | -0.890469231 | RHOBTB2 | Rho related BTB domain containing 2 [Source:HGNC Symbol;Acc:HGNC:18756] |
| ENSG00000013619 | HGLibA_28051 | 27 | -0.890469231 | MAMLD1 | mastermind like domain containing 1 [Source:HGNC Symbol;Acc:HGNC:2568] |
| ENSG00000015520 | HGLibA_32421 | 27 | -0.890469231 | NPC1L1 | NPC1 like intracellular cholesterol transporter 1 [Source:HGNC Symbol;Acc:HGNC:7898] |
| ENSG00000026950 | HGLibA_04969 | 27 | -0.890469231 | BTN3A1 | butyrophilin subfamily 3 member A1 [Source:HGNC Symbol;Acc:HGNC:1138] |
| ENSG00000037965 | HGLibA_21978 | 27 | -0.890469231 | HOXC8 | homeobox C8 [Source:HGNC Symbol;Acc:HGNC:5129] |
| ENSG00000051620 | HGLibA_21088 | 27 | -0.890469231 | HEBP2 | heme binding protein 2 [Source:HGNC Symbol;Acc:HGNC:15716] |
| ENSG00000054356 | HGLibA_39521 | 27 | -0.890469231 | PTPRN | receptor type N [Source:HGNC Symbol;Acc:HGNC:9676]" |
| ENSG00000054523 | HGLibA_24886 | 27 | -0.890469231 | KIF1B | kinesin family member 1B [Source:HGNC Symbol;Acc:HGNC:16636] |
| ENSG00000058799 | HGLibA_54670 | 27 | -0.890469231 | YIPF1 | Yip1 domain family member 1 [Source:HGNC Symbol;Acc:HGNC:25231] |
| ENSG00000065000 | HGLibA_02325 | 27 | -0.890469231 | AP3D1 | adaptor related protein complex 3 subunit delta 1 [Source:HGNC Symbol;Acc:HGNC:568] |
| ENSG00000067167 | HGLibA_51281 | 27 | -0.890469231 | TRAM1 | translocation associated membrane protein 1 [Source:HGNC Symbol;Acc:HGNC:20568] |
| ENSG00000068745 | HGLibA_23447 | 27 | -0.890469231 | IP6K2 | inositol hexakisphosphate kinase 2 [Source:HGNC Symbol;Acc:HGNC:17313] |
| ENSG00000069764 | HGLibA_36868 | 27 | -0.890469231 | PLA2G10 | phospholipase A2 group X [Source:HGNC Symbol;Acc:HGNC:9029] |
| ENSG00000072818 | HGLibA_00383 | 27 | -0.890469231 | ACAP1 | ankyrin repeat and PH domains 1 [Source:HGNC Symbol;Acc:HGNC:16467]" |
| ENSG00000075975 | HGLibA_29350 | 27 | -0.890469231 | MKRN2 | makorin ring finger protein 2 [Source:HGNC Symbol;Acc:HGNC:7113] |
| ENSG00000078070 | HGLibA_28564 | 27 | -0.890469231 | MCCC1 | methylcrotonoyl-CoA carboxylase 1 [Source:HGNC Symbol;Acc:HGNC:6936] |
| ENSG00000081377 | HGLibA_08511 | 27 | -0.890469231 | CDC14B | cell division cycle 14B [Source:HGNC Symbol;Acc:HGNC:1719] |
| ENSG00000083290 | HGLibA_52972 | 27 | -0.890469231 | ULK2 | unc-51 like autophagy activating kinase 2 [Source:HGNC Symbol;Acc:HGNC:13480] |
| ENSG00000086061 | HGLibA_13511 | 27 | -0.890469231 | DNAJA1 | DnaJ heat shock protein family (Hsp40) member A1 [Source:HGNC Symbol;Acc:HGNC:5229] |
| ENSG00000087470 | HGLibA_13686 | 27 | -0.890469231 | DNM1L | dynamin 1 like [Source:HGNC Symbol;Acc:HGNC:2973] |
| ENSG00000091513 | HGLibA_49114 | 27 | -0.890469231 | TF | transferrin [Source:HGNC Symbol;Acc:HGNC:11740] |
| ENSG00000092421 | HGLibA_43364 | 27 | -0.890469231 | SEMA6A | semaphorin 6A [Source:HGNC Symbol;Acc:HGNC:10738] |
| ENSG00000099821 | HGLibA_37639 | 27 | -0.890469231 | POLRMT | RNA polymerase mitochondrial [Source:HGNC Symbol;Acc:HGNC:9200] |
| ENSG00000100003 | HGLibA_43193 | 27 | -0.890469231 | SEC14L2 | SEC14 like lipid binding 2 [Source:HGNC Symbol;Acc:HGNC:10699] |
| ENSG00000100083 | HGLibA_18979 | 27 | -0.890469231 | GGA1 | ARF binding protein 1 [Source:HGNC Symbol;Acc:HGNC:17842]" |
| ENSG00000100253 | HGLibA_29289 | 27 | -0.890469231 | MIOX | myo-inositol oxygenase [Source:HGNC Symbol;Acc:HGNC:14522] |
| ENSG00000100632 | HGLibA_15477 | 27 | -0.890469231 | ERH | mRNA splicing and mitosis factor [Source:HGNC Symbol;Acc:HGNC:3447]" |
| ENSG00000101187 | HGLibA_45469 | 27 | -0.890469231 | SLCO4A1 | solute carrier organic anion transporter family member 4A1 [Source:HGNC Symbol;Acc:HGNC:10953] |
| ENSG00000102081 | HGLibA_17720 | 27 | -0.890469231 | FMR1 | fragile X mental retardation 1 [Source:HGNC Symbol;Acc:HGNC:3775] |
| ENSG00000102230 | HGLibA_35702 | 27 | -0.890469231 | PCYT1B | beta [Source:HGNC Symbol;Acc:HGNC:8755]" |
| ENSG00000102572 | HGLibA_47472 | 27 | -0.890469231 | STK24 | serine/threonine kinase 24 [Source:HGNC Symbol;Acc:HGNC:11403] |
| ENSG00000102898 | HGLibA_33100 | 27 | -0.890469231 | NUTF2 | nuclear transport factor 2 [Source:HGNC Symbol;Acc:HGNC:13722] |
| ENSG00000103248 | HGLibA_30394 | 27 | -0.890469231 | MTHFSD | methenyltetrahydrofolate synthetase domain containing [Source:HGNC Symbol;Acc:HGNC:25778] |
| ENSG00000103275 | HGLibA_52614 | 27 | -0.890469231 | UBE2I | ubiquitin conjugating enzyme E2 I [Source:HGNC Symbol;Acc:HGNC:12485] |
| ENSG00000103723 | HGLibA_02324 | 27 | -0.890469231 | AP3B2 | adaptor related protein complex 3 subunit beta 2 [Source:HGNC Symbol;Acc:HGNC:567] |
| ENSG00000104047 | HGLibA_14074 | 27 | -0.890469231 | DTWD1 | DTW domain containing 1 [Source:HGNC Symbol;Acc:HGNC:30926] |
| ENSG00000104472 | HGLibA_09533 | 27 | -0.890469231 | CHRAC1 | chromatin accessibility complex subunit 1 [Source:HGNC Symbol;Acc:HGNC:13544] |
| ENSG00000104687 | HGLibA_20446 | 27 | -0.890469231 | GSR | glutathione-disulfide reductase [Source:HGNC Symbol;Acc:HGNC:4623] |
| ENSG00000105948 | HGLibA_52089 | 27 | -0.890469231 | TTC26 | tetratricopeptide repeat domain 26 [Source:HGNC Symbol;Acc:HGNC:21882] |
| ENSG00000106351 | HGLibA_01188 | 27 | -0.890469231 | AGFG2 | ArfGAP with FG repeats 2 [Source:HGNC Symbol;Acc:HGNC:5177] |
| ENSG00000106565 | HGLibA_50074 | 27 | -0.890469231 | TMEM176B | transmembrane protein 176B [Source:HGNC Symbol;Acc:HGNC:29596] |
| ENSG00000107447 | HGLibA_13717 | 27 | -0.890469231 | DNTT | DNA nucleotidylexotransferase [Source:HGNC Symbol;Acc:HGNC:2983] |
| ENSG00000107643 | HGLibA_28284 | 27 | -0.890469231 | MAPK8 | mitogen-activated protein kinase 8 [Source:HGNC Symbol;Acc:HGNC:6881] |
| ENSG00000108010 | HGLibA_19318 | 27 | -0.890469231 | GLRX3 | glutaredoxin 3 [Source:HGNC Symbol;Acc:HGNC:15987] |
| ENSG00000109089 | HGLibA_08868 | 27 | -0.890469231 | CDR2L | cerebellar degeneration related protein 2 like [Source:HGNC Symbol;Acc:HGNC:29999] |
| ENSG00000109220 | HGLibA_09449 | 27 | -0.890469231 | CHIC2 | cysteine rich hydrophobic domain 2 [Source:HGNC Symbol;Acc:HGNC:1935] |
| ENSG00000109436 | HGLibA_48571 | 27 | -0.890469231 | TBC1D9 | TBC1 domain family member 9 [Source:HGNC Symbol;Acc:HGNC:21710] |
| ENSG00000110195 | HGLibA_17783 | 27 | -0.890469231 | FOLR1 | folate receptor 1 [Source:HGNC Symbol;Acc:HGNC:3791] |
| ENSG00000110693 | HGLibA_46271 | 27 | -0.890469231 | SOX6 | SRY-box 6 [Source:HGNC Symbol;Acc:HGNC:16421] |
| ENSG00000111540 | HGLibA_39965 | 27 | -0.890469231 | RAB5B | member RAS oncogene family [Source:HGNC Symbol;Acc:HGNC:9784]" |
| ENSG00000112167 | HGLibA_42787 | 27 | -0.890469231 | SAYSD1 | SAYSVFN motif domain containing 1 [Source:HGNC Symbol;Acc:HGNC:21025] |
| ENSG00000116171 | HGLibA_43035 | 27 | -0.890469231 | SCP2 | sterol carrier protein 2 [Source:HGNC Symbol;Acc:HGNC:10606] |
| ENSG00000116273 | HGLibA_36343 | 27 | -0.890469231 | PHF13 | PHD finger protein 13 [Source:HGNC Symbol;Acc:HGNC:22983] |
| ENSG00000117408 | HGLibA_23464 | 27 | -0.890469231 | IPO13 | importin 13 [Source:HGNC Symbol;Acc:HGNC:16853] |
| ENSG00000119950 | HGLibA_30618 | 27 | -0.890469231 | MXI1 | dimerization protein [Source:HGNC Symbol;Acc:HGNC:7534]" |
| ENSG00000120899 | HGLibA_39383 | 27 | -0.890469231 | PTK2B | protein tyrosine kinase 2 beta [Source:HGNC Symbol;Acc:HGNC:9612] |
| ENSG00000122140 | HGLibA_30059 | 27 | -0.890469231 | MRPS2 | mitochondrial ribosomal protein S2 [Source:HGNC Symbol;Acc:HGNC:14495] |
| ENSG00000122203 | HGLibA_24705 | 27 | -0.890469231 | KIAA1191 | KIAA1191 [Source:HGNC Symbol;Acc:HGNC:29209] |
| ENSG00000123179 | HGLibA_14377 | 27 | -0.890469231 | EBPL | EBP like [Source:HGNC Symbol;Acc:HGNC:18061] |
| ENSG00000123444 | HGLibA_24108 | 27 | -0.890469231 | KBTBD4 | kelch repeat and BTB domain containing 4 [Source:HGNC Symbol;Acc:HGNC:23761] |
| ENSG00000123992 | HGLibA_13710 | 27 | -0.890469231 | DNPEP | aspartyl aminopeptidase [Source:HGNC Symbol;Acc:HGNC:2981] |
| ENSG00000124074 | HGLibA_15135 | 27 | -0.890469231 | ENKD1 | enkurin domain containing 1 [Source:HGNC Symbol;Acc:HGNC:25246] |
| ENSG00000124140 | HGLibA_44368 | 27 | -0.890469231 | SLC12A5 | solute carrier family 12 member 5 [Source:HGNC Symbol;Acc:HGNC:13818] |
| ENSG00000125409 | HGLibA_48976 | 27 | -0.890469231 | TEKT3 | tektin 3 [Source:HGNC Symbol;Acc:HGNC:14293] |
| ENSG00000125744 | HGLibA_42402 | 27 | -0.890469231 | RTN2 | reticulon 2 [Source:HGNC Symbol;Acc:HGNC:10468] |
| ENSG00000127452 | HGLibA_16972 | 27 | -0.890469231 | FBXL12 | F-box and leucine rich repeat protein 12 [Source:HGNC Symbol;Acc:HGNC:13611] |
| ENSG00000127463 | HGLibA_15002 | 27 | -0.890469231 | EMC1 | ER membrane protein complex subunit 1 [Source:HGNC Symbol;Acc:HGNC:28957] |
| ENSG00000127928 | HGLibA_19539 | 27 | -0.890469231 | GNGT1 | G protein subunit gamma transducin 1 [Source:HGNC Symbol;Acc:HGNC:4411] |
| ENSG00000129657 | HGLibA_43189 | 27 | -0.890469231 | SEC14L1 | SEC14 like lipid binding 1 [Source:HGNC Symbol;Acc:HGNC:10698] |
| ENSG00000129873 | HGLibA_08914 | 27 | -0.890469231 | CDY2B | chromodomain Y-linked 2B [Source:HGNC Symbol;Acc:HGNC:23921] |
| ENSG00000130477 | HGLibA_52994 | 27 | -0.890469231 | UNC13A | unc-13 homolog A [Source:HGNC Symbol;Acc:HGNC:23150] |
| ENSG00000130724 | HGLibA_09479 | 27 | -0.890469231 | CHMP2A | charged multivesicular body protein 2A [Source:HGNC Symbol;Acc:HGNC:30216] |
| ENSG00000130940 | HGLibA_07349 | 27 | -0.890469231 | CASZ1 | castor zinc finger 1 [Source:HGNC Symbol;Acc:HGNC:26002] |
| ENSG00000132388 | HGLibA_52607 | 27 | -0.890469231 | UBE2G1 | ubiquitin conjugating enzyme E2 G1 [Source:HGNC Symbol;Acc:HGNC:12482] |
| ENSG00000132768 | HGLibA_13838 | 27 | -0.890469231 | DPH2 | DPH2 homolog [Source:HGNC Symbol;Acc:HGNC:3004] |
| ENSG00000132872 | HGLibA_48077 | 27 | -0.890469231 | SYT4 | synaptotagmin 4 [Source:HGNC Symbol;Acc:HGNC:11512] |
| ENSG00000132912 | HGLibA_12553 | 27 | -0.890469231 | DCTN4 | dynactin subunit 4 [Source:HGNC Symbol;Acc:HGNC:15518] |
| ENSG00000133961 | HGLibA_33012 | 27 | -0.890469231 | NUMB | endocytic adaptor protein [Source:HGNC Symbol;Acc:HGNC:8060]" |
| ENSG00000134871 | HGLibA_10570 | 27 | -0.890469231 | COL4A2 | collagen type IV alpha 2 chain [Source:HGNC Symbol;Acc:HGNC:2203] |
| ENSG00000135638 | HGLibA_15093 | 27 | -0.890469231 | EMX1 | empty spiracles homeobox 1 [Source:HGNC Symbol;Acc:HGNC:3340] |
| ENSG00000135655 | HGLibA_53210 | 27 | -0.890469231 | USP15 | ubiquitin specific peptidase 15 [Source:HGNC Symbol;Acc:HGNC:12613] |
| ENSG00000136891 | HGLibA_49045 | 27 | -0.890469231 | TEX10 | testis expressed 10 [Source:HGNC Symbol;Acc:HGNC:25988] |
| ENSG00000138385 | HGLibA_47111 | 27 | -0.890469231 | SSB | Sjogren syndrome antigen B [Source:HGNC Symbol;Acc:HGNC:11316] |

| | | | | | |
|---|---|---|---|---|---|
| ENSG00000138386 | HGLibA_31019 | 27 | -0.890469231 | NAB1 | NGFI-A binding protein 1 [Source:HGNC Symbol;Acc:HGNC:7626] |
| ENSG00000138764 | HGLibA_08096 | 27 | -0.890469231 | CCNG2 | cyclin G2 [Source:HGNC Symbol;Acc:HGNC:1593] |
| ENSG00000138814 | HGLibA_38186 | 27 | -0.890469231 | PPP3CA | protein phosphatase 3 catalytic subunit alpha [Source:HGNC Symbol;Acc:HGNC:9314] |
| ENSG00000140398 | HGLibA_31628 | 27 | -0.890469231 | NEIL1 | nei like DNA glycosylase 1 [Source:HGNC Symbol;Acc:HGNC:18448] |
| ENSG00000140479 | HGLibA_35683 | 27 | -0.890469231 | PCSK6 | proprotein convertase subtilisin/kexin type 6 [Source:HGNC Symbol;Acc:HGNC:8569] |
| ENSG00000140564 | HGLibA_18174 | 27 | -0.890469231 | FURIN | paired basic amino acid cleaving enzyme [Source:HGNC Symbol;Acc:HGNC:8568]" |
| ENSG00000141434 | HGLibA_28899 | 27 | -0.890469231 | MEP1B | meprin A subunit beta [Source:HGNC Symbol;Acc:HGNC:7020] |
| ENSG00000141933 | HGLibA_51122 | 27 | -0.890469231 | TPGS1 | tubulin polyglutamylase complex subunit 1 [Source:HGNC Symbol;Acc:HGNC:25058] |
| ENSG00000142686 | HGLibA_05831 | 27 | -0.890469231 | C1orf216 | chromosome 1 open reading frame 216 [Source:HGNC Symbol;Acc:HGNC:26800] |
| ENSG00000143493 | HGLibA_23431 | 27 | -0.890469231 | INTS7 | integrator complex subunit 7 [Source:HGNC Symbol;Acc:HGNC:24484] |
| ENSG00000144057 | HGLibA_47261 | 27 | -0.890469231 | ST6GAL2 | 6-sialyltransferase 2 [Source:HGNC Symbol;Acc:HGNC:10861]" |
| ENSG00000144057 | HGLibA_47260 | 27 | -0.890469231 | ST6GAL2 | 6-sialyltransferase 2 [Source:HGNC Symbol;Acc:HGNC:10861]" |
| ENSG00000144152 | HGLibA_16951 | 27 | -0.890469231 | FBLN7 | fibulin 7 [Source:HGNC Symbol;Acc:HGNC:26740] |
| ENSG00000144182 | HGLibA_26634 | 27 | -0.890469231 | LIPT1 | lipoyltransferase 1 [Source:HGNC Symbol;Acc:HGNC:29569] |
| ENSG00000144290 | HGLibA_45205 | 27 | -0.890469231 | SLC4A10 | solute carrier family 4 member 10 [Source:HGNC Symbol;Acc:HGNC:13811] |
| ENSG00000144591 | HGLibA_19422 | 27 | -0.890469231 | GMPPA | GDP-mannose pyrophosphorylase A [Source:HGNC Symbol;Acc:HGNC:22923] |
| ENSG00000144893 | HGLibA_28746 | 27 | -0.890469231 | MED12L | mediator complex subunit 12 like [Source:HGNC Symbol;Acc:HGNC:16050] |
| ENSG00000145703 | HGLibA_23536 | 27 | -0.890469231 | IQGAP2 | IQ motif containing GTPase activating protein 2 [Source:HGNC Symbol;Acc:HGNC:6111] |
| ENSG00000145860 | HGLibA_41531 | 27 | -0.890469231 | RNF145 | ring finger protein 145 [Source:HGNC Symbol;Acc:HGNC:20853] |
| ENSG00000145863 | HGLibA_18370 | 27 | -0.890469231 | GABRA6 | gamma-aminobutyric acid type A receptor alpha6 subunit [Source:HGNC Symbol;Acc:HGNC:4080] |
| ENSG00000146066 | HGLibA_21314 | 27 | -0.890469231 | HIGD2A | HIG1 hypoxia inducible domain family member 2A [Source:HGNC Symbol;Acc:HGNC:28311] |
| ENSG00000146469 | HGLibA_53663 | 27 | -0.890469231 | VIP | vasoactive intestinal peptide [Source:HGNC Symbol;Acc:HGNC:12693] |
| ENSG00000147437 | HGLibA_19581 | 27 | -0.890469231 | GNRH1 | gonadotropin releasing hormone 1 [Source:HGNC Symbol;Acc:HGNC:4419] |
| ENSG00000148219 | HGLibA_03383 | 27 | -0.890469231 | ASTN2 | astrotactin 2 [Source:HGNC Symbol;Acc:HGNC:17021] |
| ENSG00000151379 | HGLibA_30186 | 27 | -0.890469231 | MSGN1 | mesogenin 1 [Source:HGNC Symbol;Acc:HGNC:14907] |
| ENSG00000151690 | HGLibA_29107 | 27 | -0.890469231 | MFSD6 | major facilitator superfamily domain containing 6 [Source:HGNC Symbol;Acc:HGNC:24711] |
| ENSG00000151773 | HGLibA_07570 | 27 | -0.890469231 | CCDC122 | coiled-coil domain containing 122 [Source:HGNC Symbol;Acc:HGNC:26478] |
| ENSG00000151773 | HGLibA_07571 | 27 | -0.890469231 | CCDC122 | coiled-coil domain containing 122 [Source:HGNC Symbol;Acc:HGNC:26478] |
| ENSG00000152093 | HGLibA_09250 | 27 | -0.890469231 | CFC1B | cryptic family 1B [Source:HGNC Symbol;Acc:HGNC:33983]" |
| ENSG00000152527 | HGLibA_37129 | 27 | -0.890469231 | PLEKHH2 | MyTH4 and FERM domain containing H2 [Source:HGNC Symbol;Acc:HGNC:30506]" |
| ENSG00000154134 | HGLibA_41740 | 27 | -0.890469231 | ROBO3 | roundabout guidance receptor 3 [Source:HGNC Symbol;Acc:HGNC:13433] |
| ENSG00000154144 | HGLibA_48636 | 27 | -0.890469231 | TBRG1 | transforming growth factor beta regulator 1 [Source:HGNC Symbol;Acc:HGNC:29551] |
| ENSG00000154975 | HGLibA_06787 | 27 | -0.890469231 | CA10 | carbonic anhydrase 10 [Source:HGNC Symbol;Acc:HGNC:1369] |
| ENSG00000156395 | HGLibA_46190 | 27 | -0.890469231 | SORCS3 | sortilin related VPS10 domain containing receptor 3 [Source:HGNC Symbol;Acc:HGNC:16699] |
| ENSG00000156931 | HGLibA_53819 | 27 | -0.890469231 | VPS8 | CORVET complex subunit [Source:HGNC Symbol;Acc:HGNC:29122]" |
| ENSG00000158161 | HGLibA_15824 | 27 | -0.890469231 | EYA3 | EYA transcriptional coactivator and phosphatase 3 [Source:HGNC Symbol;Acc:HGNC:3521] |
| ENSG00000159212 | HGLibA_10036 | 27 | -0.890469231 | CLIC6 | chloride intracellular channel 6 [Source:HGNC Symbol;Acc:HGNC:2065] |
| ENSG00000160868 | HGLibA_12154 | 27 | -0.890469231 | CYP3A4 | cytochrome P450 family 3 subfamily A member 4 [Source:HGNC Symbol;Acc:HGNC:2637] |
| ENSG00000160882 | HGLibA_12055 | 27 | -0.890469231 | CYP11B1 | cytochrome P450 family 11 subfamily B member 1 [Source:HGNC Symbol;Acc:HGNC:2591] |
| ENSG00000162733 | HGLibA_12636 | 27 | -0.890469231 | DDR2 | discoidin domain receptor tyrosine kinase 2 [Source:HGNC Symbol;Acc:HGNC:2731] |
| ENSG00000162877 | HGLibA_37286 | 27 | -0.890469231 | PM20D1 | peptidase M20 domain containing 1 [Source:HGNC Symbol;Acc:HGNC:26518] |
| ENSG00000163394 | HGLibA_07954 | 27 | -0.890469231 | CCKAR | cholecystokinin A receptor [Source:HGNC Symbol;Acc:HGNC:1570] |
| ENSG00000163633 | HGLibA_06327 | 27 | -0.890469231 | C4orf36 | chromosome 4 open reading frame 36 [Source:HGNC Symbol;Acc:HGNC:28386] |
| ENSG00000163918 | HGLibA_40911 | 27 | -0.890469231 | RFC4 | replication factor C subunit 4 [Source:HGNC Symbol;Acc:HGNC:9972] |
| ENSG00000165953 | HGLibA_43514 | 27 | -0.890469231 | SERPINA12 | serpin family A member 12 [Source:HGNC Symbol;Acc:HGNC:18359] |
| ENSG00000166106 | HGLibA_00806 | 27 | -0.890469231 | ADAMTS15 | ADAM metallopeptidase with thrombospondin type 1 motif 15 [Source:HGNC Symbol;Acc:HGNC:16305] |
| ENSG00000166143 | HGLibA_38043 | 27 | -0.890469231 | PPP1R14D | protein phosphatase 1 regulatory inhibitor subunit 14D [Source:HGNC Symbol;Acc:HGNC:14953] |
| ENSG00000166317 | HGLibA_48025 | 27 | -0.890469231 | SYNPO2L | synaptopodin 2 like [Source:HGNC Symbol;Acc:HGNC:23532] |
| ENSG00000166707 | HGLibA_55025 | 27 | -0.890469231 | ZCCHC18 | zinc finger CCHC-type containing 18 [Source:HGNC Symbol;Acc:HGNC:32459] |
| ENSG00000168702 | HGLibA_27209 | 27 | -0.890469231 | LRP1B | LDL receptor related protein 1B [Source:HGNC Symbol;Acc:HGNC:6693] |
| ENSG00000169129 | HGLibA_01104 | 27 | -0.890469231 | AFAP1L2 | actin filament associated protein 1 like 2 [Source:HGNC Symbol;Acc:HGNC:25901] |
| ENSG00000169410 | HGLibA_39482 | 27 | -0.890469231 | PTPN9 | non-receptor type 9 [Source:HGNC Symbol;Acc:HGNC:9661]" |
| ENSG00000169836 | HGLibA_48178 | 27 | -0.890469231 | TACR3 | tachykinin receptor 3 [Source:HGNC Symbol;Acc:HGNC:11528] |
| ENSG00000169857 | HGLibA_03887 | 27 | -0.890469231 | AVEN | apoptosis and caspase activation inhibitor [Source:HGNC Symbol;Acc:HGNC:13509] |
| ENSG00000169955 | HGLibA_56668 | 27 | -0.890469231 | ZNF747 | zinc finger protein 747 [Source:HGNC Symbol;Acc:HGNC:28350] |
| ENSG00000170364 | HGLibA_43672 | 27 | -0.890469231 | SETMAR | SET domain and mariner transposase fusion gene [Source:HGNC Symbol;Acc:HGNC:10762] |
| ENSG00000170584 | HGLibA_32925 | 27 | -0.890469231 | NUDCD2 | NudC domain containing 2 [Source:HGNC Symbol;Acc:HGNC:30535] |
| ENSG00000170703 | HGLibA_52209 | 27 | -0.890469231 | TTLL6 | tubulin tyrosine ligase like 6 [Source:HGNC Symbol;Acc:HGNC:26664] |
| ENSG00000171208 | HGLibA_31707 | 27 | -0.890469231 | NETO2 | neuropilin and tolloid like 2 [Source:HGNC Symbol;Acc:HGNC:14644] |
| ENSG00000171495 | HGLibA_29870 | 27 | -0.890469231 | MROH2B | maestro heat like repeat family member 2B [Source:HGNC Symbol;Acc:HGNC:26857] |
| ENSG00000172062 | HGLibA_45781 | 27 | -0.890469231 | SMN1 | telomeric [Source:HGNC Symbol;Acc:HGNC:11117]" |
| ENSG00000172164 | HGLibA_46001 | 27 | -0.890469231 | SNTB1 | syntrophin beta 1 [Source:HGNC Symbol;Acc:HGNC:11168] |
| ENSG00000172289 | HGLibA_33551 | 27 | -0.890469231 | OR10V1 | olfactory receptor family 10 subfamily V member 1 [Source:HGNC Symbol;Acc:HGNC:15136] |
| ENSG00000172340 | HGLibA_47702 | 27 | -0.890469231 | SUCLG2 | succinate-CoA ligase GDP-forming beta subunit [Source:HGNC Symbol;Acc:HGNC:11450] |
| ENSG00000172476 | HGLibA_39930 | 27 | -0.890469231 | RAB40A | member RAS oncogene family [Source:HGNC Symbol;Acc:HGNC:18283]" |
| ENSG00000172543 | HGLibA_11735 | 27 | -0.890469231 | CTSW | cathepsin W [Source:HGNC Symbol;Acc:HGNC:2546] |
| ENSG00000173369 | HGLibA_05915 | 27 | -0.890469231 | C1QB | complement C1q B chain [Source:HGNC Symbol;Acc:HGNC:1242] |
| ENSG00000173401 | HGLibA_19263 | 27 | -0.890469231 | GLIPR1L1 | GLIPR1 like 1 [Source:HGNC Symbol;Acc:HGNC:28392] |
| ENSG00000173540 | HGLibA_19425 | 27 | -0.890469231 | GMPPB | GDP-mannose pyrophosphorylase B [Source:HGNC Symbol;Acc:HGNC:22932] |
| ENSG00000175792 | HGLibA_42473 | 27 | -0.890469231 | RUVBL1 | RuvB like AAA ATPase 1 [Source:HGNC Symbol;Acc:HGNC:10474] |
| ENSG00000176101 | HGLibA_47144 | 27 | -0.890469231 | SSNA1 | SS nuclear autoantigen 1 [Source:HGNC Symbol;Acc:HGNC:11321] |
| ENSG00000177301 | HGLibA_24128 | 27 | -0.890469231 | KCNA2 | potassium voltage-gated channel subfamily A member 2 [Source:HGNC Symbol;Acc:HGNC:6220] |
| ENSG00000177551 | HGLibA_31894 | 27 | -0.890469231 | NHLH2 | nescient helix-loop-helix 2 [Source:HGNC Symbol;Acc:HGNC:7818] |
| ENSG00000177669 | HGLibA_28522 | 27 | -0.890469231 | MBOAT4 | membrane bound O-acyltransferase domain containing 4 [Source:HGNC Symbol;Acc:HGNC:32311] |
| ENSG00000178184 | HGLibA_35190 | 27 | -0.890469231 | PARD6G | par-6 family cell polarity regulator gamma [Source:HGNC Symbol;Acc:HGNC:16076] |
| ENSG00000179528 | HGLibA_26088 | 27 | -0.890469231 | LBX2 | ladybird homeobox 2 [Source:HGNC Symbol;Acc:HGNC:15525] |
| ENSG00000179580 | HGLibA_41548 | 27 | -0.890469231 | RNF151 | ring finger protein 151 [Source:HGNC Symbol;Acc:HGNC:23235] |
| ENSG00000181074 | HGLibA_34200 | 27 | -0.890469231 | OR52N4 | olfactory receptor family 52 subfamily N member 4 (gene/pseudogene) [Source:HGNC Symbol;Acc:HGNC:15230] |
| ENSG00000181544 | HGLibA_16834 | 27 | -0.890469231 | FANCB | FA complementation group B [Source:HGNC Symbol;Acc:HGNC:3583] |
| ENSG00000181982 | HGLibA_07636 | 27 | -0.890469231 | CCDC149 | coiled-coil domain containing 149 [Source:HGNC Symbol;Acc:HGNC:25405] |
| ENSG00000182168 | HGLibA_53020 | 27 | -0.890469231 | UNC5C | unc-5 netrin receptor C [Source:HGNC Symbol;Acc:HGNC:12569] |
| ENSG00000182287 | HGLibA_02299 | 27 | -0.890469231 | AP1S2 | adaptor related protein complex 1 subunit sigma 2 [Source:HGNC Symbol;Acc:HGNC:560] |
| ENSG00000184032 | HGLibA_25702 | 27 | -0.890469231 | KRTAP20-2 | keratin associated protein 20-2 [Source:HGNC Symbol;Acc:HGNC:18944] |
| ENSG00000184276 | HGLibA_12844 | 27 | -0.890469231 | DEFB108B | defensin beta 108B [Source:HGNC Symbol;Acc:HGNC:29966] |
| ENSG00000184500 | HGLibA_38690 | 27 | -0.890469231 | PROS1 | protein S [Source:HGNC Symbol;Acc:HGNC:9456] |
| ENSG00000184677 | HGLibA_54881 | 27 | -0.890469231 | ZBTB40 | zinc finger and BTB domain containing 40 [Source:HGNC Symbol;Acc:HGNC:29045] |
| ENSG00000185008 | HGLibA_41737 | 27 | -0.890469231 | ROBO2 | roundabout guidance receptor 2 [Source:HGNC Symbol;Acc:HGNC:10250] |
| ENSG00000185774 | HGLibA_24244 | 27 | -0.890469231 | KCNIP4 | potassium voltage-gated channel interacting protein 4 [Source:HGNC Symbol;Acc:HGNC:30083] |
| ENSG00000185897 | HGLibA_17331 | 27 | -0.890469231 | FFAR3 | free fatty acid receptor 3 [Source:HGNC Symbol;Acc:HGNC:4499] |
| ENSG00000186132 | HGLibA_06181 | 27 | -0.890469231 | C2orf76 | chromosome 2 open reading frame 76 [Source:HGNC Symbol;Acc:HGNC:27017] |
| ENSG00000186153 | HGLibA_54449 | 27 | -0.890469231 | WWOX | WW domain containing oxidoreductase [Source:HGNC Symbol;Acc:HGNC:12799] |
| ENSG00000186480 | HGLibA_23376 | 27 | -0.890469231 | INSIG1 | insulin induced gene 1 [Source:HGNC Symbol;Acc:HGNC:6083] |
| ENSG00000186844 | HGLibA_26101 | 27 | -0.890469231 | LCE1A | late cornified envelope 1A [Source:HGNC Symbol;Acc:HGNC:29459] |
| ENSG00000187098 | HGLibA_29318 | 27 | -0.890469231 | MITF | melanocyte inducing transcription factor [Source:HGNC Symbol;Acc:HGNC:7105] |
| ENSG00000187955 | HGLibA_10511 | 27 | -0.890469231 | COL14A1 | collagen type XIV alpha 1 chain [Source:HGNC Symbol;Acc:HGNC:2191] |
| ENSG00000188130 | HGLibA_28255 | 27 | -0.890469231 | MAPK12 | mitogen-activated protein kinase 12 [Source:HGNC Symbol;Acc:HGNC:6874] |
| ENSG00000188803 | HGLibA_44045 | 27 | -0.890469231 | SHISA6 | shisa family member 6 [Source:HGNC Symbol;Acc:HGNC:34491] |
| ENSG00000196110 | HGLibA_56567 | 27 | -0.890469231 | ZNF699 | zinc finger protein 699 [Source:HGNC Symbol;Acc:HGNC:24750] |
| ENSG00000196353 | HGLibA_10962 | 27 | -0.890469231 | CPNE4 | copine 4 [Source:HGNC Symbol;Acc:HGNC:2317] |
| ENSG00000196576 | HGLibA_37273 | 27 | -0.890469231 | PLXNB2 | plexin B2 [Source:HGNC Symbol;Acc:HGNC:9104] |
| ENSG00000196591 | HGLibA_21007 | 27 | -0.890469231 | HDAC2 | histone deacetylase 2 [Source:HGNC Symbol;Acc:HGNC:4853] |
| ENSG00000196935 | HGLibA_46966 | 27 | -0.890469231 | SRGAP1 | SLIT-ROBO Rho GTPase activating protein 1 [Source:HGNC Symbol;Acc:HGNC:17382] |
| ENSG00000197111 | HGLibA_35371 | 27 | -0.890469231 | PCBP2 | poly(rC) binding protein 2 [Source:HGNC Symbol;Acc:HGNC:8648] |
| ENSG00000197992 | HGLibA_10008 | 27 | -0.890469231 | CLEC9A | C-type lectin domain containing 9A [Source:HGNC Symbol;Acc:HGNC:26705] |
| ENSG00000198771 | HGLibA_40720 | 27 | -0.890469231 | RCSD1 | RCSD domain containing 1 [Source:HGNC Symbol;Acc:HGNC:28310] |
| ENSG00000198965 | HGLibA_33542 | 27 | -0.890469231 | OR10R2 | olfactory receptor family 10 subfamily R member 2 [Source:HGNC Symbol;Acc:HGNC:14820] |
| ENSG00000203710 | HGLibA_11046 | 27 | -0.890469231 | CR1 | complement C3b/C4b receptor 1 (Knops blood group) [Source:HGNC Symbol;Acc:HGNC:2334] |
| ENSG00000203757 | HGLibA_34422 | 27 | -0.890469231 | OR6K3 | olfactory receptor family 6 subfamily K member 3 [Source:HGNC Symbol;Acc:HGNC:15030] |
| ENSG00000203785 | HGLibA_46816 | 27 | -0.890469231 | SPRR2E | small proline rich protein 2E [Source:HGNC Symbol;Acc:HGNC:11265] |
| ENSG00000204361 | HGLibA_33153 | 27 | -0.890469231 | NXPE2 | neurexophilin and PC-esterase domain family member 2 [Source:HGNC Symbol;Acc:HGNC:26331] |
| ENSG00000204616 | HGLibA_51459 | 27 | -0.890469231 | TRIM31 | tripartite motif containing 31 [Source:HGNC Symbol;Acc:HGNC:16289] |
| ENSG00000204653 | HGLibA_03344 | 27 | -0.890469231 | ASPDH | aspartate dehydrogenase domain containing [Source:HGNC Symbol;Acc:HGNC:33856] |
| ENSG00000204710 | HGLibA_46576 | 27 | -0.890469231 | SPDYC | speedy/RINGO cell cycle regulator family member C [Source:HGNC Symbol;Acc:HGNC:32681] |
| ENSG00000204899 | HGLibA_30939 | 27 | -0.890469231 | MZT1 | mitotic spindle organizing protein 1 [Source:HGNC Symbol;Acc:HGNC:33830] |
| ENSG00000205085 | HGLibA_16703 | 27 | -0.890469231 | FAM71F2 | family with sequence similarity 71 member F2 [Source:HGNC Symbol;Acc:HGNC:27998] |
| ENSG00000205213 | HGLibA_26407 | 27 | -0.890469231 | LGR4 | leucine rich repeat containing G protein-coupled receptor 4 [Source:HGNC Symbol;Acc:HGNC:13299] |
| ENSG00000205495 | HGLibA_34179 | 27 | -0.890469231 | OR52J3 | olfactory receptor family 52 subfamily J member 3 [Source:HGNC Symbol;Acc:HGNC:14799] |
| ENSG00000205882 | HGLibA_12906 | 27 | -0.890469231 | DEFB134 | defensin beta 134 [Source:HGNC Symbol;Acc:HGNC:32399] |
| ENSG00000206579 | HGLibA_54524 | 27 | -0.890469231 | XKR4 | XK related 4 [Source:HGNC Symbol;Acc:HGNC:29394] |
| ENSG00000212725 | HGLibA_25687 | 27 | -0.890469231 | KRTAP2-1 | keratin associated protein 2-1 [Source:HGNC Symbol;Acc:HGNC:16775] |
| ENSG00000214253 | HGLibA_17538 | 27 | -0.890469231 | FIS1 | mitochondrial 1 [Source:HGNC Symbol;Acc:HGNC:21689]" |
| ENSG00000214943 | HGLibA_20022 | 27 | -0.890469231 | GPR33 | G protein-coupled receptor 33 (gene/pseudogene) [Source:HGNC Symbol;Acc:HGNC:4489] |
| ENSG00000215203 | HGLibA_20389 | 27 | -0.890469231 | GRXCR1 | glutaredoxin and cysteine rich domain containing 1 [Source:HGNC Symbol;Acc:HGNC:31673] |
| ENSG00000225830 | HGLibA_15446 | 27 | -0.890469231 | ERCC6 | chromatin remodeling factor [Source:HGNC Symbol;Acc:HGNC:3438]" |
| ENSG00000231396 | HGLibA_53216 | 27 | -0.890469231 | USP17L10 | ubiquitin specific peptidase 17-like family member 10 [Source:HGNC Symbol;Acc:HGNC:44438] |
| ENSG00000234068 | HGLibA_35006 | 27 | -0.890469231 | PAGE2 | PAGE family member 2 [Source:HGNC Symbol;Acc:HGNC:31804] |
| ENSG00000236398 | HGLibA_48395 | 27 | -0.890469231 | TAS2R39 | taste 2 receptor member 39 [Source:HGNC Symbol;Acc:HGNC:18886] |
| ENSG00000236737 | HGLibA_18437 | 27 | -0.890469231 | GAGE12B | G antigen 12B [Source:HGNC Symbol;Acc:HGNC:26779] |
| ENSG00000237671 | HGLibA_18440 | 27 | -0.890469231 | GAGE12C | G antigen 12C [Source:HGNC Symbol;Acc:HGNC:28402] |
| ENSG00000240542 | HGLibA_25831 | 27 | -0.890469231 | KRTAP9-1 | keratin associated protein 9-1 [Source:HGNC Symbol;Acc:HGNC:18912] |
| ENSG00000242389 | HGLibA_40619 | 27 | -0.890469231 | RBMY1E | member E [Source:HGNC Symbol;Acc:HGNC:23916]" |
| ENSG00000243708 | HGLibA_36903 | 27 | -0.890469231 | PLA2G4B | phospholipase A2 group IVB [Source:HGNC Symbol;Acc:HGNC:9036] |
| ENSG00000253626 | HGLibA_14860 | 27 | -0.890469231 | EIF5AL1 | eukaryotic translation initiation factor 5A-like 1 [Source:HGNC Symbol;Acc:HGNC:17419] |
| ENSG00000256683 | HGLibA_55859 | 27 | -0.890469231 | ZNF350 | zinc finger protein 350 [Source:HGNC Symbol;Acc:HGNC:16656] |
| ENSG00000256762 | HGLibA_47435 | 27 | -0.890469231 | STH | saitohin [Source:HGNC Symbol;Acc:HGNC:18839] |

| | | | | | |
|---|---|---|---|---|---|
| ENSG00000261150 | HGLibA_15363 | 27 | -0.890469231 | EPPK1 | epiplakin 1 [Source:HGNC Symbol;Acc:HGNC:15577] |
| ENSG00000280204 | HGLibA_33713 | 27 | -0.890469231 | OR1S1 | olfactory receptor family 1 subfamily S member 1 (gene/pseudogene) [Source:HGNC Symbol;Acc:HGNC:8227] |
| FAM179B | HGLibA_16309 | 27 | -0.890469231 | FAM179B | - |
| FAM19A1 | HGLibA_16396 | 27 | -0.890469231 | FAM19A1 | - |
| FLJ27352 | HGLibA_17626 | 27 | -0.890469231 | FLJ27352 | - |
| hsa-mir-1302-5 | HGLibA_57709 | 27 | -0.890469231 | hsa-mir-1302-5 | - |
| hsa-mir-1587 | HGLibA_57934 | 27 | -0.890469231 | hsa-mir-1587 | - |
| hsa-mir-196a-1 | HGLibA_58091 | 27 | -0.890469231 | hsa-mir-196a-1 | - |
| hsa-mir-1973 | HGLibA_58116 | 27 | -0.890469231 | hsa-mir-1973 | - |
| hsa-mir-19b-1 | HGLibA_58142 | 27 | -0.890469231 | hsa-mir-19b-1 | - |
| hsa-mir-200a | HGLibA_58151 | 27 | -0.890469231 | hsa-mir-200a | - |
| hsa-mir-3180-3 | HGLibA_58809 | 27 | -0.890469231 | hsa-mir-3180-3 | - |
| hsa-mir-3202-2 | HGLibA_58920 | 27 | -0.890469231 | hsa-mir-3202-2 | - |
| hsa-mir-365b | HGLibA_59189 | 27 | -0.890469231 | hsa-mir-365b | - |
| hsa-mir-3685 | HGLibA_59295 | 27 | -0.890469231 | hsa-mir-3685 | - |
| hsa-mir-4273 | HGLibA_59794 | 27 | -0.890469231 | hsa-mir-4273 | - |
| hsa-mir-4419b | HGLibA_60062 | 27 | -0.890469231 | hsa-mir-4419b | - |
| hsa-mir-4436b-1 | HGLibA_60142 | 27 | -0.890469231 | hsa-mir-4436b-1 | - |
| hsa-mir-4474 | HGLibA_60306 | 27 | -0.890469231 | hsa-mir-4474 | - |
| hsa-mir-451a | HGLibA_60523 | 27 | -0.890469231 | hsa-mir-451a | - |
| hsa-mir-4708 | HGLibA_60934 | 27 | -0.890469231 | hsa-mir-4708 | - |
| hsa-mir-498 | HGLibA_61392 | 27 | -0.890469231 | hsa-mir-498 | - |
| hsa-mir-5091 | HGLibA_61523 | 27 | -0.890469231 | hsa-mir-5091 | - |
| hsa-mir-518f | HGLibA_61674 | 27 | -0.890469231 | hsa-mir-518f | - |
| hsa-mir-519a-1 | HGLibA_61712 | 27 | -0.890469231 | hsa-mir-519a-1 | - |
| hsa-mir-548a-2 | HGLibA_61841 | 27 | -0.890469231 | hsa-mir-548a-2 | - |
| hsa-mir-5681a | HGLibA_62238 | 27 | -0.890469231 | hsa-mir-5681a | - |
| hsa-mir-6088 | HGLibA_62612 | 27 | -0.890469231 | hsa-mir-6088 | - |
| hsa-mir-633 | HGLibA_62772 | 27 | -0.890469231 | hsa-mir-633 | - |
| hsa-mir-6509 | HGLibA_62884 | 27 | -0.890469231 | hsa-mir-6509 | - |
| hsa-mir-6761 | HGLibA_63206 | 27 | -0.890469231 | hsa-mir-6761 | - |
| hsa-mir-6775 | HGLibA_63272 | 27 | -0.890469231 | hsa-mir-6775 | - |
| hsa-mir-6793 | HGLibA_63349 | 27 | -0.890469231 | hsa-mir-6793 | - |
| hsa-mir-7977 | HGLibA_64037 | 27 | -0.890469231 | hsa-mir-7977 | - |
| hsa-mir-887 | HGLibA_64228 | 27 | -0.890469231 | hsa-mir-887 | - |
| hsa-mir-934 | HGLibA_64312 | 27 | -0.890469231 | hsa-mir-934 | - |
| KIAA1462 | HGLibA_24764 | 27 | -0.890469231 | KIAA1462 | - |
| MESDC2 | HGLibA_28912 | 27 | -0.890469231 | MESDC2 | - |
| MLL3 | HGLibA_29400 | 27 | -0.890469231 | MLL3 | - |
| PCNXL3 | HGLibA_35650 | 27 | -0.890469231 | PCNXL3 | - |
| 6-Mar | HGLibA_28343 | 26 | -0.899514574 | 6-Mar | - |
| C12orf66 | HGLibA_05282 | 26 | -0.899514574 | C12orf66 | - |
| C14orf105 | HGLibA_05319 | 26 | -0.899514574 | C14orf105 | - |
| C14orf177 | HGLibA_05341 | 26 | -0.899514574 | C14orf177 | - |
| C9orf66 | HGLibA_06753 | 26 | -0.899514574 | C9orf66 | - |
| CCDC58 | HGLibA_07805 | 26 | -0.899514574 | CCDC58 | - |
| CD99_X | HGLibA_08490 | 26 | -0.899514574 | CD99_X | - |
| CSF2RA_X | HGLibA_11329 | 26 | -0.899514574 | CSF2RA_X | - |
| ENSG00000000460 | HGLibA_05750 | 26 | -0.899514574 | C1orf112 | chromosome 1 open reading frame 112 [Source:HGNC Symbol;Acc:HGNC:25565] |
| ENSG00000002016 | HGLibA_40096 | 26 | -0.899514574 | RAD52 | DNA repair protein [Source:HGNC Symbol;Acc:HGNC:9824]" |
| ENSG00000005175 | HGLibA_41820 | 26 | -0.899514574 | RPAP3 | RNA polymerase II associated protein 3 [Source:HGNC Symbol;Acc:HGNC:26151] |
| ENSG00000007384 | HGLibA_41132 | 26 | -0.899514574 | RHBDF1 | rhomboid 5 homolog 1 [Source:HGNC Symbol;Acc:HGNC:20561] |
| ENSG00000009724 | HGLibA_28398 | 26 | -0.899514574 | MASP2 | mannan binding lectin serine peptidase 2 [Source:HGNC Symbol;Acc:HGNC:6902] |
| ENSG00000010671 | HGLibA_04954 | 26 | -0.899514574 | BTK | Bruton tyrosine kinase [Source:HGNC Symbol;Acc:HGNC:1133] |
| ENSG00000033050 | HGLibA_00210 | 26 | -0.899514574 | ABCF2 | ATP binding cassette subfamily F member 2 [Source:HGNC Symbol;Acc:HGNC:71] |
| ENSG00000033627 | HGLibA_03703 | 26 | -0.899514574 | ATP6V0A1 | ATPase H+ transporting V0 subunit a1 [Source:HGNC Symbol;Acc:HGNC:865] |
| ENSG00000042980 | HGLibA_00763 | 26 | -0.899514574 | ADAM28 | ADAM metallopeptidase domain 28 [Source:HGNC Symbol;Acc:HGNC:206] |
| ENSG00000047188 | HGLibA_54728 | 26 | -0.899514574 | YTHDC2 | YTH domain containing 2 [Source:HGNC Symbol;Acc:HGNC:24721] |
| ENSG00000047648 | HGLibA_02785 | 26 | -0.899514574 | ARHGAP6 | Rho GTPase activating protein 6 [Source:HGNC Symbol;Acc:HGNC:676] |
| ENSG00000049860 | HGLibA_21204 | 26 | -0.899514574 | HEXB | hexosaminidase subunit beta [Source:HGNC Symbol;Acc:HGNC:4879] |
| ENSG00000049860 | HGLibA_21203 | 26 | -0.899514574 | HEXB | hexosaminidase subunit beta [Source:HGNC Symbol;Acc:HGNC:4879] |
| ENSG00000059915 | HGLibA_39013 | 26 | -0.899514574 | PSD | pleckstrin and Sec7 domain containing [Source:HGNC Symbol;Acc:HGNC:9507] |
| ENSG00000060558 | HGLibA_19447 | 26 | -0.899514574 | GNA15 | G protein subunit alpha 15 [Source:HGNC Symbol;Acc:HGNC:4383] |
| ENSG00000067365 | HGLibA_28987 | 26 | -0.899514574 | METTL22 | methyltransferase like 22 [Source:HGNC Symbol;Acc:HGNC:28368] |
| ENSG00000069399 | HGLibA_04329 | 26 | -0.899514574 | BCL3 | B cell CLL/lymphoma 3 [Source:HGNC Symbol;Acc:HGNC:998] |
| ENSG00000072210 | HGLibA_01564 | 26 | -0.899514574 | ALDH3A2 | aldehyde dehydrogenase 3 family member A2 [Source:HGNC Symbol;Acc:HGNC:403] |
| ENSG00000073578 | HGLibA_43133 | 26 | -0.899514574 | SDHA | succinate dehydrogenase complex flavoprotein subunit A [Source:HGNC Symbol;Acc:HGNC:10680] |
| ENSG00000076248 | HGLibA_53044 | 26 | -0.899514574 | UNG | uracil DNA glycosylase [Source:HGNC Symbol;Acc:HGNC:12572] |
| ENSG00000078246 | HGLibA_52336 | 26 | -0.899514574 | TULP3 | tubby like protein 3 [Source:HGNC Symbol;Acc:HGNC:12425] |
| ENSG00000078668 | HGLibA_53604 | 26 | -0.899514574 | VDAC3 | voltage dependent anion channel 3 [Source:HGNC Symbol;Acc:HGNC:12674] |
| ENSG00000085117 | HGLibA_08459 | 26 | -0.899514574 | CD82 | CD82 molecule [Source:HGNC Symbol;Acc:HGNC:6210] |
| ENSG00000087448 | HGLibA_25213 | 26 | -0.899514574 | KLHL42 | kelch like family member 42 [Source:HGNC Symbol;Acc:HGNC:29252] |
| ENSG00000090889 | HGLibA_24943 | 26 | -0.899514574 | KIF4A | kinesin family member 4A [Source:HGNC Symbol;Acc:HGNC:13339] |
| ENSG00000091181 | HGLibA_23178 | 26 | -0.899514574 | IL5RA | interleukin 5 receptor subunit alpha [Source:HGNC Symbol;Acc:HGNC:6017] |
| ENSG00000091592 | HGLibA_32097 | 26 | -0.899514574 | NLRP1 | NLR family pyrin domain containing 1 [Source:HGNC Symbol;Acc:HGNC:14374] |
| ENSG00000092445 | HGLibA_52467 | 26 | -0.899514574 | TYRO3 | TYRO3 protein tyrosine kinase [Source:HGNC Symbol;Acc:HGNC:12446] |
| ENSG00000100023 | HGLibA_37943 | 26 | -0.899514574 | PPIL2 | peptidylprolyl isomerase like 2 [Source:HGNC Symbol;Acc:HGNC:9261] |
| ENSG00000100156 | HGLibA_44453 | 26 | -0.899514574 | SLC16A8 | solute carrier family 16 member 8 [Source:HGNC Symbol;Acc:HGNC:16270] |
| ENSG00000100325 | HGLibA_03261 | 26 | -0.899514574 | ASCC2 | activating signal cointegrator 1 complex subunit 2 [Source:HGNC Symbol;Acc:HGNC:24103] |
| ENSG00000101337 | HGLibA_49731 | 26 | -0.899514574 | TM9SF4 | transmembrane 9 superfamily member 4 [Source:HGNC Symbol;Acc:HGNC:30797] |
| ENSG00000101417 | HGLibA_39662 | 26 | -0.899514574 | PXMP4 | peroxisomal membrane protein 4 [Source:HGNC Symbol;Acc:HGNC:15920] |
| ENSG00000102145 | HGLibA_18665 | 26 | -0.899514574 | GATA1 | GATA binding protein 1 [Source:HGNC Symbol;Acc:HGNC:4170] |
| ENSG00000104537 | HGLibA_02227 | 26 | -0.899514574 | ANXA13 | annexin A13 [Source:HGNC Symbol;Acc:HGNC:536] |
| ENSG00000104760 | HGLibA_17463 | 26 | -0.899514574 | FGL1 | fibrinogen like 1 [Source:HGNC Symbol;Acc:HGNC:3695] |
| ENSG00000104856 | HGLibA_40814 | 26 | -0.899514574 | RELB | NF-kB subunit [Source:HGNC Symbol;Acc:HGNC:9956]" |
| ENSG00000105438 | HGLibA_24479 | 26 | -0.899514574 | KDELR1 | KDEL endoplasmic reticulum protein retention receptor 1 [Source:HGNC Symbol;Acc:HGNC:6304] |
| ENSG00000105576 | HGLibA_50901 | 26 | -0.899514574 | TNPO2 | transportin 2 [Source:HGNC Symbol;Acc:HGNC:19998] |
| ENSG00000105996 | HGLibA_21904 | 26 | -0.899514574 | HOXA2 | homeobox A2 [Source:HGNC Symbol;Acc:HGNC:5103] |
| ENSG00000106080 | HGLibA_17559 | 26 | -0.899514574 | FKBP14 | FK506 binding protein 14 [Source:HGNC Symbol;Acc:HGNC:18625] |
| ENSG00000106460 | HGLibA_49856 | 26 | -0.899514574 | TMEM106B | transmembrane protein 106B [Source:HGNC Symbol;Acc:HGNC:22407] |
| ENSG00000107341 | HGLibA_52654 | 26 | -0.899514574 | UBE2R2 | ubiquitin conjugating enzyme E2 R2 [Source:HGNC Symbol;Acc:HGNC:19907] |
| ENSG00000108602 | HGLibA_01563 | 26 | -0.899514574 | ALDH3A1 | aldehyde dehydrogenase 3 family member A1 [Source:HGNC Symbol;Acc:HGNC:405] |
| ENSG00000108849 | HGLibA_38245 | 26 | -0.899514574 | PPY | pancreatic polypeptide [Source:HGNC Symbol;Acc:HGNC:9327] |
| ENSG00000108960 | HGLibA_29461 | 26 | -0.899514574 | MMD | monocyte to macrophage differentiation associated [Source:HGNC Symbol;Acc:HGNC:7153] |
| ENSG00000109205 | HGLibA_33269 | 26 | -0.899514574 | ODAM | ameloblast associated [Source:HGNC Symbol;Acc:HGNC:26043]" |
| ENSG00000109572 | HGLibA_09827 | 26 | -0.899514574 | CLCN3 | chloride voltage-gated channel 3 [Source:HGNC Symbol;Acc:HGNC:2021] |
| ENSG00000109756 | HGLibA_40249 | 26 | -0.899514574 | RAPGEF2 | Rap guanine nucleotide exchange factor 2 [Source:HGNC Symbol;Acc:HGNC:16854] |
| ENSG00000111424 | HGLibA_53607 | 26 | -0.899514574 | VDR | vitamin D receptor [Source:HGNC Symbol;Acc:HGNC:12679] |
| ENSG00000111678 | HGLibA_05271 | 26 | -0.899514574 | C12orf57 | chromosome 12 open reading frame 57 [Source:HGNC Symbol;Acc:HGNC:29521] |
| ENSG00000112118 | HGLibA_28607 | 26 | -0.899514574 | MCM3 | minichromosome maintenance complex component 3 [Source:HGNC Symbol;Acc:HGNC:6945] |
| ENSG00000112182 | HGLibA_04067 | 26 | -0.899514574 | BACH2 | BTB domain and CNC homolog 2 [Source:HGNC Symbol;Acc:HGNC:14078] |
| ENSG00000113716 | HGLibA_21733 | 26 | -0.899514574 | HMGXB3 | HMG-box containing 3 [Source:HGNC Symbol;Acc:HGNC:28982] |
| ENSG00000113916 | HGLibA_04332 | 26 | -0.899514574 | BCL6 | B cell CLL/lymphoma 6 [Source:HGNC Symbol;Acc:HGNC:1001] |
| ENSG00000114767 | HGLibA_42287 | 26 | -0.899514574 | RRP9 | U3 small nucleolar RNA binding protein [Source:HGNC Symbol;Acc:HGNC:16829]" |
| ENSG00000115239 | HGLibA_03237 | 26 | -0.899514574 | ASB3 | ankyrin repeat and SOCS box containing 3 [Source:HGNC Symbol;Acc:HGNC:16013] |
| ENSG00000116748 | HGLibA_01824 | 26 | -0.899514574 | AMPD1 | adenosine monophosphate deaminase 1 [Source:HGNC Symbol;Acc:HGNC:468] |
| ENSG00000117153 | HGLibA_25129 | 26 | -0.899514574 | KLHL12 | kelch like family member 12 [Source:HGNC Symbol;Acc:HGNC:19360] |
| ENSG00000117834 | HGLibA_45287 | 26 | -0.899514574 | SLC5A9 | solute carrier family 5 member 9 [Source:HGNC Symbol;Acc:HGNC:22146] |
| ENSG00000119616 | HGLibA_17176 | 26 | -0.899514574 | FCF1 | rRNA-processing protein [Source:HGNC Symbol;Acc:HGNC:20220]" |
| ENSG00000119673 | HGLibA_00479 | 26 | -0.899514574 | ACOT2 | acyl-CoA thioesterase 2 [Source:HGNC Symbol;Acc:HGNC:18431] |
| ENSG00000119973 | HGLibA_38601 | 26 | -0.899514574 | PRLHR | prolactin releasing hormone receptor [Source:HGNC Symbol;Acc:HGNC:4464] |
| ENSG00000120158 | HGLibA_40697 | 26 | -0.899514574 | RCL1 | RNA terminal phosphate cyclase like 1 [Source:HGNC Symbol;Acc:HGNC:17687] |
| ENSG00000120211 | HGLibA_23385 | 26 | -0.899514574 | INSL4 | insulin like 4 [Source:HGNC Symbol;Acc:HGNC:6087] |
| ENSG00000120279 | HGLibA_30683 | 26 | -0.899514574 | MYCT1 | MYC target 1 [Source:HGNC Symbol;Acc:HGNC:23172] |
| ENSG00000120694 | HGLibA_22343 | 26 | -0.899514574 | HSPH1 | heat shock protein family H (Hsp110) member 1 [Source:HGNC Symbol;Acc:HGNC:16969] |
| ENSG00000121552 | HGLibA_11478 | 26 | -0.899514574 | CSTA | cystatin A [Source:HGNC Symbol;Acc:HGNC:2481] |
| ENSG00000121851 | HGLibA_37631 | 26 | -0.899514574 | POLR3GL | RNA polymerase III subunit G like [Source:HGNC Symbol;Acc:HGNC:28466] |
| ENSG00000122971 | HGLibA_00372 | 26 | -0.899514574 | ACADS | acyl-CoA dehydrogenase short chain [Source:HGNC Symbol;Acc:HGNC:90] |
| ENSG00000123405 | HGLibA_31788 | 26 | -0.899514574 | NFE2 | erythroid 2 [Source:HGNC Symbol;Acc:HGNC:7780]" |
| ENSG00000123561 | HGLibA_43530 | 26 | -0.899514574 | SERPINA7 | serpin family A member 7 [Source:HGNC Symbol;Acc:HGNC:11583] |
| ENSG00000124713 | HGLibA_19559 | 26 | -0.899514574 | GNMT | glycine N-methyltransferase [Source:HGNC Symbol;Acc:HGNC:4415] |
| ENSG00000125207 | HGLibA_36772 | 26 | -0.899514574 | PIWIL1 | piwi like RNA-mediated gene silencing 1 [Source:HGNC Symbol;Acc:HGNC:9007] |
| ENSG00000125816 | HGLibA_32039 | 26 | -0.899514574 | NKX2-4 | NK2 homeobox 4 [Source:HGNC Symbol;Acc:HGNC:7837] |
| ENSG00000125968 | HGLibA_22524 | 26 | -0.899514574 | ID1 | HLH protein [Source:HGNC Symbol;Acc:HGNC:5360]" |
| ENSG00000126249 | HGLibA_35729 | 26 | -0.899514574 | PDCD2L | programmed cell death 2 like [Source:HGNC Symbol;Acc:HGNC:28194] |
| ENSG00000128218 | HGLibA_53724 | 26 | -0.899514574 | VPREB3 | V-set pre-B cell surrogate light chain 3 [Source:HGNC Symbol;Acc:HGNC:12710] |
| ENSG00000130561 | HGLibA_42641 | 26 | -0.899514574 | SAG | S-antigen visual arrestin [Source:HGNC Symbol;Acc:HGNC:10521] |
| ENSG00000130821 | HGLibA_45341 | 26 | -0.899514574 | SLC6A8 | solute carrier family 6 member 8 [Source:HGNC Symbol;Acc:HGNC:11055] |
| ENSG00000130827 | HGLibA_37265 | 26 | -0.899514574 | PLXNA3 | plexin A3 [Source:HGNC Symbol;Acc:HGNC:9101] |
| ENSG00000131508 | HGLibA_52586 | 26 | -0.899514574 | UBE2D2 | ubiquitin conjugating enzyme E2 D2 [Source:HGNC Symbol;Acc:HGNC:12475] |
| ENSG00000131584 | HGLibA_00390 | 26 | -0.899514574 | ACAP3 | ankyrin repeat and PH domains 3 [Source:HGNC Symbol;Acc:HGNC:16754]" |
| ENSG00000132470 | HGLibA_23788 | 26 | -0.899514574 | ITGB4 | integrin subunit beta 4 [Source:HGNC Symbol;Acc:HGNC:6158] |
| ENSG00000133997 | HGLibA_28812 | 26 | -0.899514574 | MED6 | mediator complex subunit 6 [Source:HGNC Symbol;Acc:HGNC:19970] |
| ENSG00000135407 | HGLibA_03891 | 26 | -0.899514574 | AVIL | advillin [Source:HGNC Symbol;Acc:HGNC:14188] |
| ENSG00000135900 | HGLibA_29990 | 26 | -0.899514574 | MRPL44 | mitochondrial ribosomal protein L44 [Source:HGNC Symbol;Acc:HGNC:16650] |

| | | | | | |
|---|---|---|---|---|---|
| ENSG00000135925 | HGLibA_54347 | 26 | -0.899514574 | WNT10A | Wnt family member 10A [Source:HGNC Symbol;Acc:HGNC:13829] |
| ENSG00000135951 | HGLibA_51848 | 26 | -0.899514574 | TSGA10 | testis specific 10 [Source:HGNC Symbol;Acc:HGNC:14927] |
| ENSG00000136108 | HGLibA_09755 | 26 | -0.899514574 | CKAP2 | cytoskeleton associated protein 2 [Source:HGNC Symbol;Acc:HGNC:1990] |
| ENSG00000136877 | HGLibA_17960 | 26 | -0.899514574 | FPGS | folylpolyglutamate synthase [Source:HGNC Symbol;Acc:HGNC:3824] |
| ENSG00000137171 | HGLibA_25035 | 26 | -0.899514574 | KLC4 | kinesin light chain 4 [Source:HGNC Symbol;Acc:HGNC:21624] |
| ENSG00000137275 | HGLibA_41315 | 26 | -0.899514574 | RIPK1 | receptor interacting serine/threonine kinase 1 [Source:HGNC Symbol;Acc:HGNC:10019] |
| ENSG00000137462 | HGLibA_49647 | 26 | -0.899514574 | TLR2 | toll like receptor 2 [Source:HGNC Symbol;Acc:HGNC:11848] |
| ENSG00000137825 | HGLibA_23843 | 26 | -0.899514574 | ITPKA | inositol-trisphosphate 3-kinase A [Source:HGNC Symbol;Acc:HGNC:6178] |
| ENSG00000137877 | HGLibA_46890 | 26 | -0.899514574 | SPTBN5 | non-erythrocytic 5 [Source:HGNC Symbol;Acc:HGNC:15680]" |
| ENSG00000138411 | HGLibA_21106 | 26 | -0.899514574 | HECW2 | C2 and WW domain containing E3 ubiquitin protein ligase 2 [Source:HGNC Symbol;Acc:HGNC:29853]" |
| ENSG00000138653 | HGLibA_31435 | 26 | -0.899514574 | NDST4 | N-deacetylase and N-sulfotransferase 4 [Source:HGNC Symbol;Acc:HGNC:20779] |
| ENSG00000138675 | HGLibA_17411 | 26 | -0.899514574 | FGF5 | fibroblast growth factor 5 [Source:HGNC Symbol;Acc:HGNC:3683] |
| ENSG00000138769 | HGLibA_08808 | 26 | -0.899514574 | CDKL2 | cyclin dependent kinase like 2 [Source:HGNC Symbol;Acc:HGNC:1782] |
| ENSG00000138780 | HGLibA_20467 | 26 | -0.899514574 | GSTCD | glutathione S-transferase C-terminal domain containing [Source:HGNC Symbol;Acc:HGNC:25806] |
| ENSG00000139190 | HGLibA_53489 | 26 | -0.899514574 | VAMP1 | vesicle associated membrane protein 1 [Source:HGNC Symbol;Acc:HGNC:12642] |
| ENSG00000139684 | HGLibA_15563 | 26 | -0.899514574 | ESD | esterase D [Source:HGNC Symbol;Acc:HGNC:3465] |
| ENSG00000139910 | HGLibA_32363 | 26 | -0.899514574 | NOVA1 | NOVA alternative splicing regulator 1 [Source:HGNC Symbol;Acc:HGNC:7886] |
| ENSG00000140379 | HGLibA_04298 | 26 | -0.899514574 | BCL2A1 | BCL2 related protein A1 [Source:HGNC Symbol;Acc:HGNC:991] |
| ENSG00000141447 | HGLibA_34649 | 26 | -0.899514574 | OSBPL1A | oxysterol binding protein like 1A [Source:HGNC Symbol;Acc:HGNC:16398] |
| ENSG00000142149 | HGLibA_22422 | 26 | -0.899514574 | HUNK | hormonally up-regulated Neu-associated kinase [Source:HGNC Symbol;Acc:HGNC:13326] |
| ENSG00000142507 | HGLibA_39115 | 26 | -0.899514574 | PSMB6 | proteasome subunit beta 6 [Source:HGNC Symbol;Acc:HGNC:9543] |
| ENSG00000142538 | HGLibA_39371 | 26 | -0.899514574 | PTH2 | parathyroid hormone 2 [Source:HGNC Symbol;Acc:HGNC:30828] |
| ENSG00000142910 | HGLibA_49549 | 26 | -0.899514574 | TINAGL1 | tubulointerstitial nephritis antigen like 1 [Source:HGNC Symbol;Acc:HGNC:19168] |
| ENSG00000143167 | HGLibA_19718 | 26 | -0.899514574 | GPA33 | glycoprotein A33 [Source:HGNC Symbol;Acc:HGNC:4445] |
| ENSG00000143458 | HGLibA_18352 | 26 | -0.899514574 | GABPB2 | GA binding protein transcription factor subunit beta 2 [Source:HGNC Symbol;Acc:HGNC:28441] |
| ENSG00000143473 | HGLibA_24210 | 26 | -0.899514574 | KCNH1 | potassium voltage-gated channel subfamily H member 1 [Source:HGNC Symbol;Acc:HGNC:6250] |
| ENSG00000144021 | HGLibA_09677 | 26 | -0.899514574 | CIAO1 | cytosolic iron-sulfur assembly component 1 [Source:HGNC Symbol;Acc:HGNC:14280] |
| ENSG00000144406 | HGLibA_53031 | 26 | -0.899514574 | UNC80 | NALCN channel complex subunit [Source:HGNC Symbol;Acc:HGNC:26582]" |
| ENSG00000144810 | HGLibA_10614 | 26 | -0.899514574 | COL8A1 | collagen type VIII alpha 1 chain [Source:HGNC Symbol;Acc:HGNC:2215] |
| ENSG00000145088 | HGLibA_14338 | 26 | -0.899514574 | EAF2 | ELL associated factor 2 [Source:HGNC Symbol;Acc:HGNC:23115] |
| ENSG00000145354 | HGLibA_09730 | 26 | -0.899514574 | CISD2 | CDGSH iron sulfur domain 2 [Source:HGNC Symbol;Acc:HGNC:24212] |
| ENSG00000145362 | HGLibA_01937 | 26 | -0.899514574 | ANK2 | ankyrin 2 [Source:HGNC Symbol;Acc:HGNC:493] |
| ENSG00000145723 | HGLibA_19097 | 26 | -0.899514574 | GIN1 | gypsy retrotransposon integrase 1 [Source:HGNC Symbol;Acc:HGNC:25959] |
| ENSG00000145743 | HGLibA_16987 | 26 | -0.899514574 | FBXL17 | F-box and leucine rich repeat protein 17 [Source:HGNC Symbol;Acc:HGNC:13615] |
| ENSG00000146281 | HGLibA_37289 | 26 | -0.899514574 | PM20D2 | peptidase M20 domain containing 2 [Source:HGNC Symbol;Acc:HGNC:21408] |
| ENSG00000146469 | HGLibA_53662 | 26 | -0.899514574 | VIP | vasoactive intestinal peptide [Source:HGNC Symbol;Acc:HGNC:12693] |
| ENSG00000147689 | HGLibA_16741 | 26 | -0.899514574 | FAM83A | family with sequence similarity 83 member A [Source:HGNC Symbol;Acc:HGNC:28210] |
| ENSG00000148303 | HGLibA_41995 | 26 | -0.899514574 | RPL7A | ribosomal protein L7a [Source:HGNC Symbol;Acc:HGNC:10364] |
| ENSG00000148602 | HGLibA_27185 | 26 | -0.899514574 | LRIT1 | Ig-like and transmembrane domains 1 [Source:HGNC Symbol;Acc:HGNC:23404]" |
| ENSG00000148948 | HGLibA_27383 | 26 | -0.899514574 | LRRC4C | leucine rich repeat containing 4C [Source:HGNC Symbol;Acc:HGNC:29317] |
| ENSG00000149295 | HGLibA_13963 | 26 | -0.899514574 | DRD2 | dopamine receptor D2 [Source:HGNC Symbol;Acc:HGNC:3023] |
| ENSG00000149541 | HGLibA_03979 | 26 | -0.899514574 | B3GAT3 | 3-glucuronyltransferase 3 [Source:HGNC Symbol;Acc:HGNC:923]" |
| ENSG00000151116 | HGLibA_52841 | 26 | -0.899514574 | UEVLD | UEV and lactate/malate dehyrogenase domains [Source:HGNC Symbol;Acc:HGNC:30866] |
| ENSG00000151247 | HGLibA_14816 | 26 | -0.899514574 | EIF4E | eukaryotic translation initiation factor 4E [Source:HGNC Symbol;Acc:HGNC:3287] |
| ENSG00000153214 | HGLibA_50534 | 26 | -0.899514574 | TMEM87B | transmembrane protein 87B [Source:HGNC Symbol;Acc:HGNC:25913] |
| ENSG00000153250 | HGLibA_40588 | 26 | -0.899514574 | RBMS1 | RNA binding motif single stranded interacting protein 1 [Source:HGNC Symbol;Acc:HGNC:9907] |
| ENSG00000153283 | HGLibA_08484 | 26 | -0.899514574 | CD96 | CD96 molecule [Source:HGNC Symbol;Acc:HGNC:16892] |
| ENSG00000153551 | HGLibA_10227 | 26 | -0.899514574 | CMTM7 | CKLF like MARVEL transmembrane domain containing 7 [Source:HGNC Symbol;Acc:HGNC:19178] |
| ENSG00000153832 | HGLibA_17080 | 26 | -0.899514574 | FBXO36 | F-box protein 36 [Source:HGNC Symbol;Acc:HGNC:27020] |
| ENSG00000154710 | HGLibA_40015 | 26 | -0.899514574 | RABGEF1 | RAB guanine nucleotide exchange factor 1 [Source:HGNC Symbol;Acc:HGNC:17676] |
| ENSG00000155918 | HGLibA_40125 | 26 | -0.899514574 | RAET1L | retinoic acid early transcript 1L [Source:HGNC Symbol;Acc:HGNC:16798] |
| ENSG00000156219 | HGLibA_03161 | 26 | -0.899514574 | ART3 | ADP-ribosyltransferase 3 [Source:HGNC Symbol;Acc:HGNC:725] |
| ENSG00000156374 | HGLibA_35603 | 26 | -0.899514574 | PCGF6 | polycomb group ring finger 6 [Source:HGNC Symbol;Acc:HGNC:21156] |
| ENSG00000157005 | HGLibA_47170 | 26 | -0.899514574 | SST | somatostatin [Source:HGNC Symbol;Acc:HGNC:11329] |
| ENSG00000158467 | HGLibA_01291 | 26 | -0.899514574 | AHCYL2 | adenosylhomocysteinase like 2 [Source:HGNC Symbol;Acc:HGNC:22204] |
| ENSG00000158481 | HGLibA_08269 | 26 | -0.899514574 | CD1C | CD1c molecule [Source:HGNC Symbol;Acc:HGNC:1636] |
| ENSG00000158966 | HGLibA_06872 | 26 | -0.899514574 | CACHD1 | cache domain containing 1 [Source:HGNC Symbol;Acc:HGNC:29314] |
| ENSG00000159189 | HGLibA_05920 | 26 | -0.899514574 | C1QC | complement C1q C chain [Source:HGNC Symbol;Acc:HGNC:1245] |
| ENSG00000159409 | HGLibA_09010 | 26 | -0.899514574 | CELF3 | CUGBP Elav-like family member 3 [Source:HGNC Symbol;Acc:HGNC:11967] |
| ENSG00000159461 | HGLibA_01782 | 26 | -0.899514574 | AMFR | autocrine motility factor receptor [Source:HGNC Symbol;Acc:HGNC:463] |
| ENSG00000160207 | HGLibA_22227 | 26 | -0.899514574 | HSF2BP | heat shock transcription factor 2 binding protein [Source:HGNC Symbol;Acc:HGNC:5226] |
| ENSG00000160345 | HGLibA_06664 | 26 | -0.899514574 | C9orf116 | chromosome 9 open reading frame 116 [Source:HGNC Symbol;Acc:HGNC:28435] |
| ENSG00000161533 | HGLibA_00496 | 26 | -0.899514574 | ACOX1 | acyl-CoA oxidase 1 [Source:HGNC Symbol;Acc:HGNC:119] |
| ENSG00000161807 | HGLibA_34480 | 26 | -0.899514574 | OR7G1 | olfactory receptor family 7 subfamily G member 1 [Source:HGNC Symbol;Acc:HGNC:8465] |
| ENSG00000161914 | HGLibA_56463 | 26 | -0.899514574 | ZNF653 | zinc finger protein 653 [Source:HGNC Symbol;Acc:HGNC:25196] |
| ENSG00000162337 | HGLibA_27225 | 26 | -0.899514574 | LRP5 | LDL receptor related protein 5 [Source:HGNC Symbol;Acc:HGNC:6697] |
| ENSG00000162777 | HGLibA_12943 | 26 | -0.899514574 | DENND2D | DENN domain containing 2D [Source:HGNC Symbol;Acc:HGNC:26192] |
| ENSG00000162961 | HGLibA_13916 | 26 | -0.899514574 | DPY30 | histone methyltransferase complex regulatory subunit [Source:HGNC Symbol;Acc:HGNC:24590]" |
| ENSG00000163006 | HGLibA_07609 | 26 | -0.899514574 | CCDC138 | coiled-coil domain containing 138 [Source:HGNC Symbol;Acc:HGNC:26531] |
| ENSG00000163191 | HGLibA_42535 | 26 | -0.899514574 | S100A11 | S100 calcium binding protein A11 [Source:HGNC Symbol;Acc:HGNC:10488] |
| ENSG00000163288 | HGLibA_18375 | 26 | -0.899514574 | GABRB1 | gamma-aminobutyric acid type A receptor beta1 subunit [Source:HGNC Symbol;Acc:HGNC:4081] |
| ENSG00000163606 | HGLibA_08285 | 26 | -0.899514574 | CD200R1 | CD200 receptor 1 [Source:HGNC Symbol;Acc:HGNC:24235] |
| ENSG00000164548 | HGLibA_51223 | 26 | -0.899514574 | TRA2A | transformer 2 alpha homolog [Source:HGNC Symbol;Acc:HGNC:16645] |
| ENSG00000165675 | HGLibA_15162 | 26 | -0.899514574 | ENOX2 | ecto-NOX disulfide-thiol exchanger 2 [Source:HGNC Symbol;Acc:HGNC:2259] |
| ENSG00000165916 | HGLibA_39134 | 26 | -0.899514574 | PSMC3 | ATPase 3 [Source:HGNC Symbol;Acc:HGNC:9549]" |
| ENSG00000166188 | HGLibA_55790 | 26 | -0.899514574 | ZNF319 | zinc finger protein 319 [Source:HGNC Symbol;Acc:HGNC:13644] |
| ENSG00000168101 | HGLibA_32954 | 26 | -0.899514574 | NUDT16L1 | nudix hydrolase 16 like 1 [Source:HGNC Symbol;Acc:HGNC:28154] |
| ENSG00000168778 | HGLibA_48876 | 26 | -0.899514574 | TCTN2 | tectonic family member 2 [Source:HGNC Symbol;Acc:HGNC:25774] |
| ENSG00000168781 | HGLibA_37953 | 26 | -0.899514574 | PPIP5K1 | diphosphoinositol pentakisphosphate kinase 1 [Source:HGNC Symbol;Acc:HGNC:29023] |
| ENSG00000168807 | HGLibA_46003 | 26 | -0.899514574 | SNTB2 | syntrophin beta 2 [Source:HGNC Symbol;Acc:HGNC:11169] |
| ENSG00000168813 | HGLibA_56123 | 26 | -0.899514574 | ZNF507 | zinc finger protein 507 [Source:HGNC Symbol;Acc:HGNC:23783] |
| ENSG00000168875 | HGLibA_46239 | 26 | -0.899514574 | SOX14 | SRY-box 14 [Source:HGNC Symbol;Acc:HGNC:11193] |
| ENSG00000169116 | HGLibA_35206 | 26 | -0.899514574 | PARM1 | prostate androgen-regulated mucin-like protein 1 [Source:HGNC Symbol;Acc:HGNC:24536] |
| ENSG00000169291 | HGLibA_44021 | 26 | -0.899514574 | SHE | Src homology 2 domain containing E [Source:HGNC Symbol;Acc:HGNC:27004] |
| ENSG00000169306 | HGLibA_23056 | 26 | -0.899514574 | IL1RAPL1 | interleukin 1 receptor accessory protein like 1 [Source:HGNC Symbol;Acc:HGNC:5996] |
| ENSG00000170923 | HGLibA_34483 | 26 | -0.899514574 | OR7G2 | olfactory receptor family 7 subfamily G member 2 [Source:HGNC Symbol;Acc:HGNC:8466] |
| ENSG00000171533 | HGLibA_28226 | 26 | -0.899514574 | MAP6 | microtubule associated protein 6 [Source:HGNC Symbol;Acc:HGNC:6868] |
| ENSG00000173418 | HGLibA_30983 | 26 | -0.899514574 | NAA20 | NatB catalytic subunit [Source:HGNC Symbol;Acc:HGNC:15908]" |
| ENSG00000173726 | HGLibA_50954 | 26 | -0.899514574 | TOMM20 | translocase of outer mitochondrial membrane 20 [Source:HGNC Symbol;Acc:HGNC:20947] |
| ENSG00000175262 | HGLibA_05766 | 26 | -0.899514574 | C1orf127 | chromosome 1 open reading frame 127 [Source:HGNC Symbol;Acc:HGNC:26730] |
| ENSG00000176009 | HGLibA_03273 | 26 | -0.899514574 | ASCL3 | achaete-scute family bHLH transcription factor 3 [Source:HGNC Symbol;Acc:HGNC:740] |
| ENSG00000176563 | HGLibA_10387 | 26 | -0.899514574 | CNTD1 | cyclin N-terminal domain containing 1 [Source:HGNC Symbol;Acc:HGNC:26847] |
| ENSG00000176746 | HGLibA_27955 | 26 | -0.899514574 | MAGEB6 | MAGE family member B6 [Source:HGNC Symbol;Acc:HGNC:23796] |
| ENSG00000176774 | HGLibA_27941 | 26 | -0.899514574 | MAGEB18 | MAGE family member B18 [Source:HGNC Symbol;Acc:HGNC:28515] |
| ENSG00000178105 | HGLibA_12652 | 26 | -0.899514574 | DDX10 | DEAD-box helicase 10 [Source:HGNC Symbol;Acc:HGNC:2735] |
| ENSG00000180370 | HGLibA_35042 | 26 | -0.899514574 | PAK2 | p21 (RAC1) activated kinase 2 [Source:HGNC Symbol;Acc:HGNC:8591] |
| ENSG00000181009 | HGLibA_34203 | 26 | -0.899514574 | OR52N5 | olfactory receptor family 52 subfamily N member 5 [Source:HGNC Symbol;Acc:HGNC:15231] |
| ENSG00000181016 | HGLibA_27556 | 26 | -0.899514574 | LSMEM1 | leucine rich single-pass membrane protein 1 [Source:HGNC Symbol;Acc:HGNC:22036] |
| ENSG00000182261 | HGLibA_32100 | 26 | -0.899514574 | NLRP10 | NLR family pyrin domain containing 10 [Source:HGNC Symbol;Acc:HGNC:21464] |
| ENSG00000183960 | HGLibA_24230 | 26 | -0.899514574 | KCNH8 | potassium voltage-gated channel subfamily H member 8 [Source:HGNC Symbol;Acc:HGNC:18864] |
| ENSG00000184571 | HGLibA_36776 | 26 | -0.899514574 | PIWIL3 | piwi like RNA-mediated gene silencing 3 [Source:HGNC Symbol;Acc:HGNC:18443] |
| ENSG00000184785 | HGLibA_45720 | 26 | -0.899514574 | SMIM10 | small integral membrane protein 10 [Source:HGNC Symbol;Acc:HGNC:41913] |
| ENSG00000186009 | HGLibA_03634 | 26 | -0.899514574 | ATP4B | ATPase H+/K+ transporting subunit beta [Source:HGNC Symbol;Acc:HGNC:820] |
| ENSG00000186103 | HGLibA_02692 | 26 | -0.899514574 | ARGFX | arginine-fifty homeobox [Source:HGNC Symbol;Acc:HGNC:30146] |
| ENSG00000186469 | HGLibA_19519 | 26 | -0.899514574 | GNG2 | G protein subunit gamma 2 [Source:HGNC Symbol;Acc:HGNC:4404] |
| ENSG00000186907 | HGLibA_42419 | 26 | -0.899514574 | RTN4RL2 | reticulon 4 receptor like 2 [Source:HGNC Symbol;Acc:HGNC:23053] |
| ENSG00000187151 | HGLibA_01923 | 26 | -0.899514574 | ANGPTL5 | angiopoietin like 5 [Source:HGNC Symbol;Acc:HGNC:19705] |
| ENSG00000187239 | HGLibA_17735 | 26 | -0.899514574 | FNBP1 | formin binding protein 1 [Source:HGNC Symbol;Acc:HGNC:17069] |
| ENSG00000189319 | HGLibA_16638 | 26 | -0.899514574 | FAM53B | family with sequence similarity 53 member B [Source:HGNC Symbol;Acc:HGNC:28968] |
| ENSG00000196337 | HGLibA_09312 | 26 | -0.899514574 | CGB7 | chorionic gonadotropin subunit beta 7 [Source:HGNC Symbol;Acc:HGNC:16451] |
| ENSG00000196976 | HGLibA_25928 | 26 | -0.899514574 | LAGE3 | L antigen family member 3 [Source:HGNC Symbol;Acc:HGNC:26058] |
| ENSG00000197386 | HGLibA_22419 | 26 | -0.899514574 | HTT | huntingtin [Source:HGNC Symbol;Acc:HGNC:4851] |
| ENSG00000197863 | HGLibA_56765 | 26 | -0.899514574 | ZNF790 | zinc finger protein 790 [Source:HGNC Symbol;Acc:HGNC:33114] |
| ENSG00000198315 | HGLibA_55362 | 26 | -0.899514574 | ZKSCAN8 | zinc finger with KRAB and SCAN domains 8 [Source:HGNC Symbol;Acc:HGNC:12983] |
| ENSG00000198355 | HGLibA_36680 | 26 | -0.899514574 | PIM3 | serine/threonine kinase [Source:HGNC Symbol;Acc:HGNC:19310]" |
| ENSG00000198785 | HGLibA_20312 | 26 | -0.899514574 | GRIN3A | glutamate ionotropic receptor NMDA type subunit 3A [Source:HGNC Symbol;Acc:HGNC:16767] |
| ENSG00000198797 | HGLibA_04816 | 26 | -0.899514574 | BRINP2 | BMP/retinoic acid inducible neural specific 2 [Source:HGNC Symbol;Acc:HGNC:13746] |
| ENSG00000198887 | HGLibA_45656 | 26 | -0.899514574 | SMC5 | structural maintenance of chromosomes 5 [Source:HGNC Symbol;Acc:HGNC:20465] |
| ENSG00000203933 | HGLibA_11961 | 26 | -0.899514574 | CXorf66 | chromosome X open reading frame 66 [Source:HGNC Symbol;Acc:HGNC:33743] |
| ENSG00000204149 | HGLibA_01153 | 26 | -0.899514574 | AGAP6 | ankyrin repeat and PH domain 6 [Source:HGNC Symbol;Acc:HGNC:23466]" |
| ENSG00000204345 | HGLibA_08340 | 26 | -0.899514574 | CD300LD | CD300 molecule like family member d [Source:HGNC Symbol;Acc:HGNC:16848] |
| ENSG00000205177 | HGLibA_05212 | 26 | -0.899514574 | C11orf91 | chromosome 11 open reading frame 91 [Source:HGNC Symbol;Acc:HGNC:34444] |
| ENSG00000205937 | HGLibA_41729 | 26 | -0.899514574 | RNPS1 | RNA binding protein with serine rich domain 1 [Source:HGNC Symbol;Acc:HGNC:10080] |
| ENSG00000206172 | HGLibA_20908 | 26 | -0.899514574 | HBA1 | hemoglobin subunit alpha 1 [Source:HGNC Symbol;Acc:HGNC:4823] |
| ENSG00000214338 | HGLibA_46158 | 26 | -0.899514574 | SOGA3 | SOGA family member 3 [Source:HGNC Symbol;Acc:HGNC:21494] |
| ENSG00000217930 | HGLibA_35085 | 26 | -0.899514574 | PAM16 | presequence translocase associated motor 16 [Source:HGNC Symbol;Acc:HGNC:29679] |
| ENSG00000225781 | HGLibA_34447 | 26 | -0.899514574 | OR6V1 | olfactory receptor family 6 subfamily V member 1 [Source:HGNC Symbol;Acc:HGNC:15090] |
| ENSG00000237289 | HGLibA_09780 | 26 | -0.899514574 | CKMT1B | mitochondrial 1B [Source:HGNC Symbol;Acc:HGNC:1995]" |
| ENSG00000240720 | HGLibA_27447 | 26 | -0.899514574 | LRRD1 | leucine rich repeats and death domain containing 1 [Source:HGNC Symbol;Acc:HGNC:34300] |
| ENSG00000241343 | HGLibA_41954 | 26 | -0.899514574 | RPL36A | ribosomal protein L36a [Source:HGNC Symbol;Acc:HGNC:10359] |
| ENSG00000255713 | HGLibA_33975 | 26 | -0.899514574 | OR4D2 | olfactory receptor family 4 subfamily D member 2 [Source:HGNC Symbol;Acc:HGNC:8294] |
| ENSG00000256222 | HGLibA_30479 | 26 | -0.899514574 | MTRNR2L3 | MT-RNR2 like 3 [Source:HGNC Symbol;Acc:HGNC:37157] |
| ENSG00000260325 | HGLibA_22323 | 26 | -0.899514574 | HSPB9 | heat shock protein family B (small) member 9 [Source:HGNC Symbol;Acc:HGNC:30589] |
| ENSG00000267631 | HGLibA_09301 | 26 | -0.899514574 | CGB1 | chorionic gonadotropin subunit beta 1 [Source:HGNC Symbol;Acc:HGNC:16721] |
| ENSG00000269502 | HGLibA_13436 | 26 | -0.899514574 | DMRTC1 | DMRT like family C1 [Source:HGNC Symbol;Acc:HGNC:13910] |

| | | | | | |
|---|---|---|---|---|---|
| GAGE7 | HGLibA_18488 | 26 | -0.899514574 | GAGE7 | - |
| GATSL3 | HGLibA_18708 | 26 | -0.899514574 | GATSL3 | - |
| hsa-mir-105-1 | HGLibA_57177 | 26 | -0.899514574 | hsa-mir-105-1 | - |
| hsa-mir-1224 | HGLibA_57294 | 26 | -0.899514574 | hsa-mir-1224 | - |
| hsa-mir-196a-2 | HGLibA_58098 | 26 | -0.899514574 | hsa-mir-196a-2 | - |
| hsa-mir-2117 | HGLibA_58241 | 26 | -0.899514574 | hsa-mir-2117 | - |
| hsa-mir-24-1 | HGLibA_58340 | 26 | -0.899514574 | hsa-mir-24-1 | - |
| hsa-mir-25 | HGLibA_58352 | 26 | -0.899514574 | hsa-mir-25 | - |
| hsa-mir-29b-2 | HGLibA_58419 | 26 | -0.899514574 | hsa-mir-29b-2 | - |
| hsa-mir-302b | HGLibA_58442 | 26 | -0.899514574 | hsa-mir-302b | - |
| hsa-mir-3606 | HGLibA_59061 | 26 | -0.899514574 | hsa-mir-3606 | - |
| hsa-mir-361 | HGLibA_59067 | 26 | -0.899514574 | hsa-mir-361 | - |
| hsa-mir-3622a | HGLibA_59123 | 26 | -0.899514574 | hsa-mir-3622a | - |
| hsa-mir-3677 | HGLibA_59259 | 26 | -0.899514574 | hsa-mir-3677 | - |
| hsa-mir-378i | HGLibA_59463 | 26 | -0.899514574 | hsa-mir-378i | - |
| hsa-mir-3973 | HGLibA_59653 | 26 | -0.899514574 | hsa-mir-3973 | - |
| hsa-mir-4307 | HGLibA_59939 | 26 | -0.899514574 | hsa-mir-4307 | - |
| hsa-mir-4417 | HGLibA_60053 | 26 | -0.899514574 | hsa-mir-4417 | - |
| hsa-mir-4433b | HGLibA_60122 | 26 | -0.899514574 | hsa-mir-4433b | - |
| hsa-mir-4436a | HGLibA_60139 | 26 | -0.899514574 | hsa-mir-4436a | - |
| hsa-mir-4480 | HGLibA_60338 | 26 | -0.899514574 | hsa-mir-4480 | - |
| hsa-mir-452 | HGLibA_60531 | 26 | -0.899514574 | hsa-mir-452 | - |
| hsa-mir-4748 | HGLibA_61091 | 26 | -0.899514574 | hsa-mir-4748 | - |
| hsa-mir-4760 | HGLibA_61138 | 26 | -0.899514574 | hsa-mir-4760 | - |
| hsa-mir-519e | HGLibA_61731 | 26 | -0.899514574 | hsa-mir-519e | - |
| hsa-mir-525 | HGLibA_61785 | 26 | -0.899514574 | hsa-mir-525 | - |
| hsa-mir-5681b | HGLibA_62242 | 26 | -0.899514574 | hsa-mir-5681b | - |
| hsa-mir-5701-1 | HGLibA_62340 | 26 | -0.899514574 | hsa-mir-5701-1 | - |
| hsa-mir-591 | HGLibA_62460 | 26 | -0.899514574 | hsa-mir-591 | - |
| hsa-mir-6089-2 | HGLibA_62620 | 26 | -0.899514574 | hsa-mir-6089-2 | - |
| hsa-mir-6784 | HGLibA_63315 | 26 | -0.899514574 | hsa-mir-6784 | - |
| hsa-mir-6883 | HGLibA_63717 | 26 | -0.899514574 | hsa-mir-6883 | - |
| hsa-mir-8073 | HGLibA_64135 | 26 | -0.899514574 | hsa-mir-8073 | - |
| ICK | HGLibA_22508 | 26 | -0.899514574 | ICK | - |
| IL3RA_X | HGLibA_23161 | 26 | -0.899514574 | IL3RA_X | - |
| KIAA0141 | HGLibA_24594 | 26 | -0.899514574 | KIAA0141 | - |
| LHFP | HGLibA_26426 | 26 | -0.899514574 | LHFP | - |
| LOC100129636 | HGLibA_26768 | 26 | -0.899514574 | LOC100129636 | - |
| LOC100288814 | HGLibA_26837 | 26 | -0.899514574 | LOC100288814 | - |
| LOC284385 | HGLibA_26916 | 26 | -0.899514574 | LOC284385 | - |
| LOC284385 | HGLibA_26914 | 26 | -0.899514574 | LOC284385 | - |
| MRP63 | HGLibA_29886 | 26 | -0.899514574 | MRP63 | - |
| MST4 | HGLibA_30261 | 26 | -0.899514574 | MST4 | - |
| OCLM | HGLibA_33251 | 26 | -0.899514574 | OCLM | - |
| WBSCR28 | HGLibA_54000 | 26 | -0.899514574 | WBSCR28 | - |
| ZFYVE20 | HGLibA_55280 | 26 | -0.899514574 | ZFYVE20 | - |
| C17orf51 | HGLibA_05543 | 25 | -0.908559917 | C17orf51 | - |
| C1orf27 | HGLibA_05853 | 25 | -0.908559917 | C1orf27 | - |
| C20orf26 | HGLibA_06020 | 25 | -0.908559917 | C20orf26 | - |
| ENSG00000000005 | HGLibA_50859 | 25 | -0.908559917 | TNMD | tenomodulin [Source:HGNC Symbol;Acc:HGNC:17757] |
| ENSG00000002549 | HGLibA_26014 | 25 | -0.908559917 | LAP3 | leucine aminopeptidase 3 [Source:HGNC Symbol;Acc:HGNC:18449] |
| ENSG00000005381 | HGLibA_29744 | 25 | -0.908559917 | MPO | myeloperoxidase [Source:HGNC Symbol;Acc:HGNC:7218] |
| ENSG00000005882 | HGLibA_35898 | 25 | -0.908559917 | PDK2 | pyruvate dehydrogenase kinase 2 [Source:HGNC Symbol;Acc:HGNC:8810] |
| ENSG00000006059 | HGLibA_25480 | 25 | -0.908559917 | KRT33A | keratin 33A [Source:HGNC Symbol;Acc:HGNC:6450] |
| ENSG00000007372 | HGLibA_35310 | 25 | -0.908559917 | PAX6 | paired box 6 [Source:HGNC Symbol;Acc:HGNC:8620] |
| ENSG00000009709 | HGLibA_35313 | 25 | -0.908559917 | PAX7 | paired box 7 [Source:HGNC Symbol;Acc:HGNC:8621] |
| ENSG00000011132 | HGLibA_02375 | 25 | -0.908559917 | APBA3 | amyloid beta precursor protein binding family A member 3 [Source:HGNC Symbol;Acc:HGNC:580] |
| ENSG00000012817 | HGLibA_24530 | 25 | -0.908559917 | KDM5D | lysine demethylase 5D [Source:HGNC Symbol;Acc:HGNC:11115] |
| ENSG00000013293 | HGLibA_45358 | 25 | -0.908559917 | SLC7A14 | solute carrier family 7 member 14 [Source:HGNC Symbol;Acc:HGNC:29326] |
| ENSG00000047188 | HGLibA_54726 | 25 | -0.908559917 | YTHDC2 | YTH domain containing 2 [Source:HGNC Symbol;Acc:HGNC:24721] |
| ENSG00000047936 | HGLibA_41785 | 25 | -0.908559917 | ROS1 | receptor tyrosine kinase [Source:HGNC Symbol;Acc:HGNC:10261]" |
| ENSG00000060491 | HGLibA_33316 | 25 | -0.908559917 | OGFR | opioid growth factor receptor [Source:HGNC Symbol;Acc:HGNC:15768] |
| ENSG00000062598 | HGLibA_14932 | 25 | -0.908559917 | ELMO2 | engulfment and cell motility 2 [Source:HGNC Symbol;Acc:HGNC:17233] |
| ENSG00000068024 | HGLibA_21011 | 25 | -0.908559917 | HDAC4 | histone deacetylase 4 [Source:HGNC Symbol;Acc:HGNC:14063] |
| ENSG00000077063 | HGLibA_11744 | 25 | -0.908559917 | CTTNBP2 | cortactin binding protein 2 [Source:HGNC Symbol;Acc:HGNC:15679] |
| ENSG00000080503 | HGLibA_45604 | 25 | -0.908559917 | SMARCA2 | member 2 [Source:HGNC Symbol;Acc:HGNC:11098]" |
| ENSG00000081721 | HGLibA_14135 | 25 | -0.908559917 | DUSP12 | dual specificity phosphatase 12 [Source:HGNC Symbol;Acc:HGNC:3067] |
| ENSG00000088766 | HGLibA_11197 | 25 | -0.908559917 | CRLS1 | cardiolipin synthase 1 [Source:HGNC Symbol;Acc:HGNC:16148] |
| ENSG00000094804 | HGLibA_08592 | 25 | -0.908559917 | CDC6 | cell division cycle 6 [Source:HGNC Symbol;Acc:HGNC:1744] |
| ENSG00000096872 | HGLibA_22770 | 25 | -0.908559917 | IFT74 | intraflagellar transport 74 [Source:HGNC Symbol;Acc:HGNC:21424] |
| ENSG00000099999 | HGLibA_41628 | 25 | -0.908559917 | RNF215 | ring finger protein 215 [Source:HGNC Symbol;Acc:HGNC:33434] |
| ENSG00000100321 | HGLibA_47991 | 25 | -0.908559917 | SYNGR1 | synaptogyrin 1 [Source:HGNC Symbol;Acc:HGNC:11498] |
| ENSG00000100575 | HGLibA_49529 | 25 | -0.908559917 | TIMM9 | translocase of inner mitochondrial membrane 9 [Source:HGNC Symbol;Acc:HGNC:11819] |
| ENSG00000100580 | HGLibA_49827 | 25 | -0.908559917 | TMED8 | transmembrane p24 trafficking protein family member 8 [Source:HGNC Symbol;Acc:HGNC:18633] |
| ENSG00000101132 | HGLibA_36143 | 25 | -0.908559917 | PFDN4 | prefoldin subunit 4 [Source:HGNC Symbol;Acc:HGNC:8868] |
| ENSG00000101577 | HGLibA_27097 | 25 | -0.908559917 | LPIN2 | lipin 2 [Source:HGNC Symbol;Acc:HGNC:14450] |
| ENSG00000101596 | HGLibA_45663 | 25 | -0.908559917 | SMCHD1 | structural maintenance of chromosomes flexible hinge domain containing 1 [Source:HGNC Symbol;Acc:HGNC:29090] |
| ENSG00000101844 | HGLibA_03487 | 25 | -0.908559917 | ATG4A | autophagy related 4A cysteine peptidase [Source:HGNC Symbol;Acc:HGNC:16489] |
| ENSG00000103121 | HGLibA_10189 | 25 | -0.908559917 | CMC2 | C-X9-C motif containing 2 [Source:HGNC Symbol;Acc:HGNC:24447] |
| ENSG00000104228 | HGLibA_51471 | 25 | -0.908559917 | TRIM35 | tripartite motif containing 35 [Source:HGNC Symbol;Acc:HGNC:16285] |
| ENSG00000104450 | HGLibA_46341 | 25 | -0.908559917 | SPAG1 | sperm associated antigen 1 [Source:HGNC Symbol;Acc:HGNC:11212] |
| ENSG00000104904 | HGLibA_33212 | 25 | -0.908559917 | OAZ1 | ornithine decarboxylase antizyme 1 [Source:HGNC Symbol;Acc:HGNC:8095] |
| ENSG00000105447 | HGLibA_20385 | 25 | -0.908559917 | GRWD1 | glutamate rich WD repeat containing 1 [Source:HGNC Symbol;Acc:HGNC:21270] |
| ENSG00000106344 | HGLibA_40526 | 25 | -0.908559917 | RBM28 | RNA binding motif protein 28 [Source:HGNC Symbol;Acc:HGNC:21863] |
| ENSG00000106511 | HGLibA_28893 | 25 | -0.908559917 | MEOX2 | mesenchyme homeobox 2 [Source:HGNC Symbol;Acc:HGNC:7014] |
| ENSG00000106635 | HGLibA_04340 | 25 | -0.908559917 | BCL7B | BCL tumor suppressor 7B [Source:HGNC Symbol;Acc:HGNC:1005] |
| ENSG00000106771 | HGLibA_50300 | 25 | -0.908559917 | TMEM245 | transmembrane protein 245 [Source:HGNC Symbol;Acc:HGNC:1363] |
| ENSG00000107669 | HGLibA_03429 | 25 | -0.908559917 | ATE1 | arginyltransferase 1 [Source:HGNC Symbol;Acc:HGNC:782] |
| ENSG00000107984 | HGLibA_13294 | 25 | -0.908559917 | DKK1 | dickkopf WNT signaling pathway inhibitor 1 [Source:HGNC Symbol;Acc:HGNC:2891] |
| ENSG00000108590 | HGLibA_28805 | 25 | -0.908559917 | MED31 | mediator complex subunit 31 [Source:HGNC Symbol;Acc:HGNC:24260] |
| ENSG00000108773 | HGLibA_24052 | 25 | -0.908559917 | KAT2A | lysine acetyltransferase 2A [Source:HGNC Symbol;Acc:HGNC:4201] |
| ENSG00000109181 | HGLibA_52911 | 25 | -0.908559917 | UGT2B10 | UDP glucuronosyltransferase family 2 member B10 [Source:HGNC Symbol;Acc:HGNC:12544] |
| ENSG00000109610 | HGLibA_46149 | 25 | -0.908559917 | SOD3 | superoxide dismutase 3 [Source:HGNC Symbol;Acc:HGNC:11181] |
| ENSG00000111328 | HGLibA_08760 | 25 | -0.908559917 | CDK2AP1 | cyclin dependent kinase 2 associated protein 1 [Source:HGNC Symbol;Acc:HGNC:14002] |
| ENSG00000111536 | HGLibA_23108 | 25 | -0.908559917 | IL26 | interleukin 26 [Source:HGNC Symbol;Acc:HGNC:17119] |
| ENSG00000113231 | HGLibA_35831 | 25 | -0.908559917 | PDE8B | phosphodiesterase 8B [Source:HGNC Symbol;Acc:HGNC:8794] |
| ENSG00000113739 | HGLibA_47416 | 25 | -0.908559917 | STC2 | stanniocalcin 2 [Source:HGNC Symbol;Acc:HGNC:11374] |
| ENSG00000115084 | HGLibA_45009 | 25 | -0.908559917 | SLC35F5 | solute carrier family 35 member F5 [Source:HGNC Symbol;Acc:HGNC:23617] |
| ENSG00000115364 | HGLibA_29918 | 25 | -0.908559917 | MRPL19 | mitochondrial ribosomal protein L19 [Source:HGNC Symbol;Acc:HGNC:14052] |
| ENSG00000115760 | HGLibA_04524 | 25 | -0.908559917 | BIRC6 | baculoviral IAP repeat containing 6 [Source:HGNC Symbol;Acc:HGNC:13516] |
| ENSG00000116641 | HGLibA_13755 | 25 | -0.908559917 | DOCK7 | dedicator of cytokinesis 7 [Source:HGNC Symbol;Acc:HGNC:19190] |
| ENSG00000117682 | HGLibA_13096 | 25 | -0.908559917 | DHDDS | dehydrodolichyl diphosphate synthase subunit [Source:HGNC Symbol;Acc:HGNC:20603] |
| ENSG00000119048 | HGLibA_52575 | 25 | -0.908559917 | UBE2B | ubiquitin conjugating enzyme E2 B [Source:HGNC Symbol;Acc:HGNC:12473] |
| ENSG00000119414 | HGLibA_38218 | 25 | -0.908559917 | PPP6C | protein phosphatase 6 catalytic subunit [Source:HGNC Symbol;Acc:HGNC:9323] |
| ENSG00000119943 | HGLibA_39705 | 25 | -0.908559917 | PYROXD2 | pyridine nucleotide-disulphide oxidoreductase domain 2 [Source:HGNC Symbol;Acc:HGNC:23517] |
| ENSG00000120137 | HGLibA_35103 | 25 | -0.908559917 | PANK3 | pantothenate kinase 3 [Source:HGNC Symbol;Acc:HGNC:19365] |
| ENSG00000121579 | HGLibA_30999 | 25 | -0.908559917 | NAA50 | NatE catalytic subunit [Source:HGNC Symbol;Acc:HGNC:29533]" |
| ENSG00000121931 | HGLibA_27172 | 25 | -0.908559917 | LRIF1 | ligand dependent nuclear receptor interacting factor 1 [Source:HGNC Symbol;Acc:HGNC:30299] |
| ENSG00000122218 | HGLibA_10692 | 25 | -0.908559917 | COPA | coatomer protein complex subunit alpha [Source:HGNC Symbol;Acc:HGNC:2230] |
| ENSG00000123609 | HGLibA_32182 | 25 | -0.908559917 | NMI | N-myc and STAT interactor [Source:HGNC Symbol;Acc:HGNC:7854] |
| ENSG00000123838 | HGLibA_06290 | 25 | -0.908559917 | C4BPA | complement component 4 binding protein alpha [Source:HGNC Symbol;Acc:HGNC:1325] |
| ENSG00000124422 | HGLibA_53306 | 25 | -0.908559917 | USP22 | ubiquitin specific peptidase 22 [Source:HGNC Symbol;Acc:HGNC:12621] |
| ENSG00000124615 | HGLibA_29613 | 25 | -0.908559917 | MOCS1 | molybdenum cofactor synthesis 1 [Source:HGNC Symbol;Acc:HGNC:7190] |
| ENSG00000124812 | HGLibA_11166 | 25 | -0.908559917 | CRISP1 | cysteine rich secretory protein 1 [Source:HGNC Symbol;Acc:HGNC:304] |
| ENSG00000125245 | HGLibA_19980 | 25 | -0.908559917 | GPR18 | G protein-coupled receptor 18 [Source:HGNC Symbol;Acc:HGNC:4472] |
| ENSG00000125848 | HGLibA_17660 | 25 | -0.908559917 | FLRT3 | fibronectin leucine rich transmembrane protein 3 [Source:HGNC Symbol;Acc:HGNC:3762] |
| ENSG00000126254 | HGLibA_40552 | 25 | -0.908559917 | RBM42 | RNA binding motif protein 42 [Source:HGNC Symbol;Acc:HGNC:28117] |
| ENSG00000126267 | HGLibA_10849 | 25 | -0.908559917 | COX6B1 | cytochrome c oxidase subunit 6B1 [Source:HGNC Symbol;Acc:HGNC:2280] |
| ENSG00000126458 | HGLibA_42237 | 25 | -0.908559917 | RRAS | RAS related [Source:HGNC Symbol;Acc:HGNC:10447] |
| ENSG00000126838 | HGLibA_39714 | 25 | -0.908559917 | PZP | alpha-2-macroglobulin like [Source:HGNC Symbol;Acc:HGNC:9750]" |
| ENSG00000129460 | HGLibA_31867 | 25 | -0.908559917 | NGDN | neuroguidin [Source:HGNC Symbol;Acc:HGNC:20271] |
| ENSG00000130032 | HGLibA_38874 | 25 | -0.908559917 | PRRG3 | proline rich and Gla domain 3 [Source:HGNC Symbol;Acc:HGNC:30798] |
| ENSG00000130741 | HGLibA_14758 | 25 | -0.908559917 | EIF2S3 | eukaryotic translation initiation factor 2 subunit gamma [Source:HGNC Symbol;Acc:HGNC:3267] |
| ENSG00000130749 | HGLibA_54975 | 25 | -0.908559917 | ZC3H4 | zinc finger CCCH-type containing 4 [Source:HGNC Symbol;Acc:HGNC:17808] |
| ENSG00000131068 | HGLibA_12863 | 25 | -0.908559917 | DEFB118 | defensin beta 118 [Source:HGNC Symbol;Acc:HGNC:16196] |
| ENSG00000131149 | HGLibA_20415 | 25 | -0.908559917 | GSE1 | Gse1 coiled-coil protein [Source:HGNC Symbol;Acc:HGNC:28979] |
| ENSG00000131966 | HGLibA_00647 | 25 | -0.908559917 | ACTR10 | actin related protein 10 homolog [Source:HGNC Symbol;Acc:HGNC:17372] |
| ENSG00000132702 | HGLibA_20844 | 25 | -0.908559917 | HAPLN2 | hyaluronan and proteoglycan link protein 2 [Source:HGNC Symbol;Acc:HGNC:17410] |
| ENSG00000132906 | HGLibA_07331 | 25 | -0.908559917 | CASP9 | caspase 9 [Source:HGNC Symbol;Acc:HGNC:1511] |
| ENSG00000133937 | HGLibA_20397 | 25 | -0.908559917 | GSC | goosecoid homeobox [Source:HGNC Symbol;Acc:HGNC:4612] |
| ENSG00000134253 | HGLibA_51510 | 25 | -0.908559917 | TRIM45 | tripartite motif containing 45 [Source:HGNC Symbol;Acc:HGNC:19018] |
| ENSG00000134489 | HGLibA_22103 | 25 | -0.908559917 | HRH4 | histamine receptor H4 [Source:HGNC Symbol;Acc:HGNC:17383] |
| ENSG00000136279 | HGLibA_12397 | 25 | -0.908559917 | DBNL | drebrin like [Source:HGNC Symbol;Acc:HGNC:2696] |
| ENSG00000136895 | HGLibA_18632 | 25 | -0.908559917 | GARNL3 | GTPase activating Rap/RanGAP domain like 3 [Source:HGNC Symbol;Acc:HGNC:25425] |
| ENSG00000136925 | HGLibA_52034 | 25 | -0.908559917 | TSTD2 | thiosulfate sulfurtransferase like domain containing 2 [Source:HGNC Symbol;Acc:HGNC:30087] |

| | | | | | |
|---|---|---|---|---|---|
| ENSG00000137948 | HGLibA_04791 | 25 | -0.908559917 | BRDT | bromodomain testis associated [Source:HGNC Symbol;Acc:HGNC:1105] |
| ENSG00000140465 | HGLibA_12067 | 25 | -0.908559917 | CYP1A1 | cytochrome P450 family 1 subfamily A member 1 [Source:HGNC Symbol;Acc:HGNC:2595] |
| ENSG00000140995 | HGLibA_12789 | 25 | -0.908559917 | DEF8 | differentially expressed in FDCP 8 homolog [Source:HGNC Symbol;Acc:HGNC:25969] |
| ENSG00000143226 | HGLibA_17189 | 25 | -0.908559917 | FCGR2A | Fc fragment of IgG receptor IIa [Source:HGNC Symbol;Acc:HGNC:3616] |
| ENSG00000143228 | HGLibA_32997 | 25 | -0.908559917 | NUF2 | NDC80 kinetochore complex component NUF2 [Source:HGNC Symbol;Acc:HGNC:14621] |
| ENSG00000143924 | HGLibA_15066 | 25 | -0.908559917 | EML4 | echinoderm microtubule associated protein like 4 [Source:HGNC Symbol;Acc:HGNC:1316] |
| ENSG00000144218 | HGLibA_01112 | 25 | -0.908559917 | AFF3 | AF4/FMR2 family member 3 [Source:HGNC Symbol;Acc:HGNC:6473] |
| ENSG00000144285 | HGLibA_42975 | 25 | -0.908559917 | SCN1A | sodium voltage-gated channel alpha subunit 1 [Source:HGNC Symbol;Acc:HGNC:10585] |
| ENSG00000145107 | HGLibA_49696 | 25 | -0.908559917 | TM4SF19 | transmembrane 4 L six family member 19 [Source:HGNC Symbol;Acc:HGNC:25167] |
| ENSG00000146414 | HGLibA_44080 | 25 | -0.908559917 | SHPRH | SNF2 histone linker PHD RING helicase [Source:HGNC Symbol;Acc:HGNC:19336] |
| ENSG00000148337 | HGLibA_09752 | 25 | -0.908559917 | CIZ1 | CDKN1A interacting zinc finger protein 1 [Source:HGNC Symbol;Acc:HGNC:16744] |
| ENSG00000148734 | HGLibA_32438 | 25 | -0.908559917 | NPFFR1 | neuropeptide FF receptor 1 [Source:HGNC Symbol;Acc:HGNC:17425] |
| ENSG00000148773 | HGLibA_29323 | 25 | -0.908559917 | MKI67 | marker of proliferation Ki-67 [Source:HGNC Symbol;Acc:HGNC:7107] |
| ENSG00000148832 | HGLibA_35119 | 25 | -0.908559917 | PAOX | polyamine oxidase [Source:HGNC Symbol;Acc:HGNC:20837] |
| ENSG00000148935 | HGLibA_18643 | 25 | -0.908559917 | GAS2 | growth arrest specific 2 [Source:HGNC Symbol;Acc:HGNC:4167] |
| ENSG00000149435 | HGLibA_19022 | 25 | -0.908559917 | GGTLC1 | gamma-glutamyltransferase light chain 1 [Source:HGNC Symbol;Acc:HGNC:16437] |
| ENSG00000149609 | HGLibA_05989 | 25 | -0.908559917 | C20orf144 | chromosome 20 open reading frame 144 [Source:HGNC Symbol;Acc:HGNC:16137] |
| ENSG00000150403 | HGLibA_49788 | 25 | -0.908559917 | TMCO3 | transmembrane and coiled-coil domains 3 [Source:HGNC Symbol;Acc:HGNC:20329] |
| ENSG00000150779 | HGLibA_49526 | 25 | -0.908559917 | TIMM8B | translocase of inner mitochondrial membrane 8 homolog B [Source:HGNC Symbol;Acc:HGNC:11818] |
| ENSG00000152092 | HGLibA_03381 | 25 | -0.908559917 | ASTN1 | astrotactin 1 [Source:HGNC Symbol;Acc:HGNC:773] |
| ENSG00000153885 | HGLibA_24429 | 25 | -0.908559917 | KCTD15 | potassium channel tetramerization domain containing 15 [Source:HGNC Symbol;Acc:HGNC:23297] |
| ENSG00000153944 | HGLibA_30208 | 25 | -0.908559917 | MSI2 | musashi RNA binding protein 2 [Source:HGNC Symbol;Acc:HGNC:18585] |
| ENSG00000154118 | HGLibA_23984 | 25 | -0.908559917 | JPH3 | junctophilin 3 [Source:HGNC Symbol;Acc:HGNC:14203] |
| ENSG00000157741 | HGLibA_52722 | 25 | -0.908559917 | UBN2 | ubinuclein 2 [Source:HGNC Symbol;Acc:HGNC:21931] |
| ENSG00000157764 | HGLibA_04751 | 25 | -0.908559917 | BRAF | serine/threonine kinase [Source:HGNC Symbol;Acc:HGNC:1097]" |
| ENSG00000158423 | HGLibA_41230 | 25 | -0.908559917 | RIBC1 | RIB43A domain with coiled-coils 1 [Source:HGNC Symbol;Acc:HGNC:26537] |
| ENSG00000160013 | HGLibA_39345 | 25 | -0.908559917 | PTGIR | prostaglandin I2 receptor [Source:HGNC Symbol;Acc:HGNC:9602] |
| ENSG00000160685 | HGLibA_54920 | 25 | -0.908559917 | ZBTB7B | zinc finger and BTB domain containing 7B [Source:HGNC Symbol;Acc:HGNC:18668] |
| ENSG00000162889 | HGLibA_28302 | 25 | -0.908559917 | MAPKAPK2 | mitogen-activated protein kinase-activated protein kinase 2 [Source:HGNC Symbol;Acc:HGNC:6887] |
| ENSG00000163220 | HGLibA_42576 | 25 | -0.908559917 | S100A9 | S100 calcium binding protein A9 [Source:HGNC Symbol;Acc:HGNC:10499] |
| ENSG00000163331 | HGLibA_12331 | 25 | -0.908559917 | DAPL1 | death associated protein like 1 [Source:HGNC Symbol;Acc:HGNC:21490] |
| ENSG00000163803 | HGLibA_36970 | 25 | -0.908559917 | PLB1 | phospholipase B1 [Source:HGNC Symbol;Acc:HGNC:30041] |
| ENSG00000164885 | HGLibA_08772 | 25 | -0.908559917 | CDK5 | cyclin dependent kinase 5 [Source:HGNC Symbol;Acc:HGNC:1774] |
| ENSG00000165476 | HGLibA_40782 | 25 | -0.908559917 | REEP3 | receptor accessory protein 3 [Source:HGNC Symbol;Acc:HGNC:23711] |
| ENSG00000166948 | HGLibA_49268 | 25 | -0.908559917 | TGM6 | transglutaminase 6 [Source:HGNC Symbol;Acc:HGNC:16255] |
| ENSG00000166960 | HGLibA_07715 | 25 | -0.908559917 | CCDC178 | coiled-coil domain containing 178 [Source:HGNC Symbol;Acc:HGNC:29588] |
| ENSG00000167916 | HGLibA_25456 | 25 | -0.908559917 | KRT24 | keratin 24 [Source:HGNC Symbol;Acc:HGNC:18527] |
| ENSG00000167972 | HGLibA_00109 | 25 | -0.908559917 | ABCA3 | ATP binding cassette subfamily A member 3 [Source:HGNC Symbol;Acc:HGNC:33] |
| ENSG00000168827 | HGLibA_18948 | 25 | -0.908559917 | GFM1 | G elongation factor mitochondrial 1 [Source:HGNC Symbol;Acc:HGNC:13780] |
| ENSG00000169474 | HGLibA_46802 | 25 | -0.908559917 | SPRR1A | small proline rich protein 1A [Source:HGNC Symbol;Acc:HGNC:11259] |
| ENSG00000169607 | HGLibA_09756 | 25 | -0.908559917 | CKAP2L | cytoskeleton associated protein 2 like [Source:HGNC Symbol;Acc:HGNC:26877] |
| ENSG00000169762 | HGLibA_48328 | 25 | -0.908559917 | TAPT1 | transmembrane anterior posterior transformation 1 [Source:HGNC Symbol;Acc:HGNC:26887] |
| ENSG00000169900 | HGLibA_39683 | 25 | -0.908559917 | PYDC1 | pyrin domain containing 1 [Source:HGNC Symbol;Acc:HGNC:30261] |
| ENSG00000170615 | HGLibA_44798 | 25 | -0.908559917 | SLC26A5 | solute carrier family 26 member 5 [Source:HGNC Symbol;Acc:HGNC:9359] |
| ENSG00000170950 | HGLibA_36243 | 25 | -0.908559917 | PGK2 | phosphoglycerate kinase 2 [Source:HGNC Symbol;Acc:HGNC:8898] |
| ENSG00000170953 | HGLibA_34494 | 25 | -0.908559917 | OR8B12 | olfactory receptor family 8 subfamily B member 12 [Source:HGNC Symbol;Acc:HGNC:15307] |
| ENSG00000171202 | HGLibA_49910 | 25 | -0.908559917 | TMEM126A | transmembrane protein 126A [Source:HGNC Symbol;Acc:HGNC:25382] |
| ENSG00000171320 | HGLibA_15561 | 25 | -0.908559917 | ESCO2 | establishment of sister chromatid cohesion N-acetyltransferase 2 [Source:HGNC Symbol;Acc:HGNC:27230] |
| ENSG00000171357 | HGLibA_27633 | 25 | -0.908559917 | LURAP1 | leucine rich adaptor protein 1 [Source:HGNC Symbol;Acc:HGNC:32327] |
| ENSG00000171587 | HGLibA_13999 | 25 | -0.908559917 | DSCAM | DS cell adhesion molecule [Source:HGNC Symbol;Acc:HGNC:3039] |
| ENSG00000171612 | HGLibA_44711 | 25 | -0.908559917 | SLC25A33 | solute carrier family 25 member 33 [Source:HGNC Symbol;Acc:HGNC:29681] |
| ENSG00000172264 | HGLibA_27838 | 25 | -0.908559917 | MACROD2 | MACRO domain containing 2 [Source:HGNC Symbol;Acc:HGNC:16126] |
| ENSG00000172270 | HGLibA_04865 | 25 | -0.908559917 | BSG | basigin (Ok blood group) [Source:HGNC Symbol;Acc:HGNC:1116] |
| ENSG00000172288 | HGLibA_08904 | 25 | -0.908559917 | CDY1 | chromodomain Y-linked 1 [Source:HGNC Symbol;Acc:HGNC:1809] |
| ENSG00000172409 | HGLibA_10095 | 25 | -0.908559917 | CLP1 | cleavage and polyadenylation factor I subunit 1 [Source:HGNC Symbol;Acc:HGNC:16999] |
| ENSG00000174225 | HGLibA_02930 | 25 | -0.908559917 | ARL13A | ADP ribosylation factor like GTPase 13A [Source:HGNC Symbol;Acc:HGNC:31709] |
| ENSG00000175121 | HGLibA_54267 | 25 | -0.908559917 | WFDC5 | WAP four-disulfide core domain 5 [Source:HGNC Symbol;Acc:HGNC:20477] |
| ENSG00000175224 | HGLibA_03466 | 25 | -0.908559917 | ATG13 | autophagy related 13 [Source:HGNC Symbol;Acc:HGNC:29091] |
| ENSG00000175229 | HGLibA_18503 | 25 | -0.908559917 | GAL3ST3 | galactose-3-O-sulfotransferase 3 [Source:HGNC Symbol;Acc:HGNC:24144] |
| ENSG00000176887 | HGLibA_46232 | 25 | -0.908559917 | SOX11 | SRY-box 11 [Source:HGNC Symbol;Acc:HGNC:11191] |
| ENSG00000176928 | HGLibA_18819 | 25 | -0.908559917 | GCNT4 | core 2 [Source:HGNC Symbol;Acc:HGNC:17973]" |
| ENSG00000177243 | HGLibA_12817 | 25 | -0.908559917 | DEFB103B | defensin beta 103B [Source:HGNC Symbol;Acc:HGNC:31702] |
| ENSG00000177830 | HGLibA_09452 | 25 | -0.908559917 | CHID1 | chitinase domain containing 1 [Source:HGNC Symbol;Acc:HGNC:28474] |
| ENSG00000178732 | HGLibA_19709 | 25 | -0.908559917 | GP5 | glycoprotein V platelet [Source:HGNC Symbol;Acc:HGNC:4443] |
| ENSG00000178896 | HGLibA_15781 | 25 | -0.908559917 | EXOSC4 | exosome component 4 [Source:HGNC Symbol;Acc:HGNC:18189] |
| ENSG00000179636 | HGLibA_51168 | 25 | -0.908559917 | TPPP2 | tubulin polymerization promoting protein family member 2 [Source:HGNC Symbol;Acc:HGNC:19293] |
| ENSG00000180479 | HGLibA_56278 | 25 | -0.908559917 | ZNF571 | zinc finger protein 571 [Source:HGNC Symbol;Acc:HGNC:25000] |
| ENSG00000181061 | HGLibA_21307 | 25 | -0.908559917 | HIGD1A | HIG1 hypoxia inducible domain family member 1A [Source:HGNC Symbol;Acc:HGNC:29527] |
| ENSG00000181418 | HGLibA_12624 | 25 | -0.908559917 | DDN | dendrin [Source:HGNC Symbol;Acc:HGNC:24458] |
| ENSG00000181929 | HGLibA_38513 | 25 | -0.908559917 | PRKAG1 | protein kinase AMP-activated non-catalytic subunit gamma 1 [Source:HGNC Symbol;Acc:HGNC:9385] |
| ENSG00000182544 | HGLibA_29105 | 25 | -0.908559917 | MFSD5 | major facilitator superfamily domain containing 5 [Source:HGNC Symbol;Acc:HGNC:28156] |
| ENSG00000182944 | HGLibA_15713 | 25 | -0.908559917 | EWSR1 | EWS RNA binding protein 1 [Source:HGNC Symbol;Acc:HGNC:3508] |
| ENSG00000183137 | HGLibA_09149 | 25 | -0.908559917 | CEP57L1 | centrosomal protein 57 like 1 [Source:HGNC Symbol;Acc:HGNC:21561] |
| ENSG00000184140 | HGLibA_34012 | 25 | -0.908559917 | OR4F6 | olfactory receptor family 4 subfamily F member 6 [Source:HGNC Symbol;Acc:HGNC:15372] |
| ENSG00000184221 | HGLibA_33367 | 25 | -0.908559917 | OLIG1 | oligodendrocyte transcription factor 1 [Source:HGNC Symbol;Acc:HGNC:16983] |
| ENSG00000184635 | HGLibA_56911 | 25 | -0.908559917 | ZNF93 | zinc finger protein 93 [Source:HGNC Symbol;Acc:HGNC:13169] |
| ENSG00000184956 | HGLibA_30552 | 25 | -0.908559917 | MUC6 | oligomeric mucus/gel-forming [Source:HGNC Symbol;Acc:HGNC:7517]" |
| ENSG00000185379 | HGLibA_40092 | 25 | -0.908559917 | RAD51D | RAD51 paralog D [Source:HGNC Symbol;Acc:HGNC:9823] |
| ENSG00000186471 | HGLibA_01417 | 25 | -0.908559917 | AKAP14 | A-kinase anchoring protein 14 [Source:HGNC Symbol;Acc:HGNC:24061] |
| ENSG00000187871 | HGLibA_18976 | 25 | -0.908559917 | GFRAL | GDNF family receptor alpha like [Source:HGNC Symbol;Acc:HGNC:32789] |
| ENSG00000188175 | HGLibA_21141 | 25 | -0.908559917 | HEPACAM2 | HEPACAM family member 2 [Source:HGNC Symbol;Acc:HGNC:27364] |
| ENSG00000196459 | HGLibA_51312 | 25 | -0.908559917 | TRAPPC2 | trafficking protein particle complex 2 [Source:HGNC Symbol;Acc:HGNC:23068] |
| ENSG00000196735 | HGLibA_21622 | 25 | -0.908559917 | HLA-DQA1 | DQ alpha 1 [Source:HGNC Symbol;Acc:HGNC:4942]" |
| ENSG00000197658 | HGLibA_44577 | 25 | -0.908559917 | SLC22A24 | solute carrier family 22 member 24 [Source:HGNC Symbol;Acc:HGNC:28542] |
| ENSG00000197921 | HGLibA_21189 | 25 | -0.908559917 | HES5 | hes family bHLH transcription factor 5 [Source:HGNC Symbol;Acc:HGNC:19764] |
| ENSG00000198270 | HGLibA_49887 | 25 | -0.908559917 | TMEM116 | transmembrane protein 116 [Source:HGNC Symbol;Acc:HGNC:25084] |
| ENSG00000198677 | HGLibA_52126 | 25 | -0.908559917 | TTC37 | tetratricopeptide repeat domain 37 [Source:HGNC Symbol;Acc:HGNC:23639] |
| ENSG00000198700 | HGLibA_23479 | 25 | -0.908559917 | IPO9 | importin 9 [Source:HGNC Symbol;Acc:HGNC:19425] |
| ENSG00000198821 | HGLibA_08308 | 25 | -0.908559917 | CD247 | CD247 molecule [Source:HGNC Symbol;Acc:HGNC:1677] |
| ENSG00000204700 | HGLibA_33809 | 25 | -0.908559917 | OR2J2 | olfactory receptor family 2 subfamily J member 2 [Source:HGNC Symbol;Acc:HGNC:8260] |
| ENSG00000206177 | HGLibA_20931 | 25 | -0.908559917 | HBM | hemoglobin subunit mu [Source:HGNC Symbol;Acc:HGNC:4826] |
| ENSG00000213516 | HGLibA_40601 | 25 | -0.908559917 | RBMXL1 | RBMX like 1 [Source:HGNC Symbol;Acc:HGNC:25073] |
| ENSG00000234465 | HGLibA_36692 | 25 | -0.908559917 | PINLYP | phospholipase A2 inhibitor and LY6/PLAUR domain containing [Source:HGNC Symbol;Acc:HGNC:44206] |
| ENSG00000236334 | HGLibA_37916 | 25 | -0.908559917 | PPIAL4G | peptidylprolyl isomerase A like 4G [Source:HGNC Symbol;Acc:HGNC:33996] |
| ENSG00000244025 | HGLibA_25668 | 25 | -0.908559917 | KRTAP19-3 | keratin associated protein 19-3 [Source:HGNC Symbol;Acc:HGNC:18938] |
| ENSG00000250479 | HGLibA_09363 | 25 | -0.908559917 | CHCHD10 | coiled-coil-helix-coiled-coil-helix domain containing 10 [Source:HGNC Symbol;Acc:HGNC:15559] |
| ENSG00000256771 | HGLibA_55673 | 25 | -0.908559917 | ZNF253 | zinc finger protein 253 [Source:HGNC Symbol;Acc:HGNC:13497] |
| ENSG00000261796 | HGLibA_23698 | 25 | -0.908559917 | ISY1-RAB43 | ISY1-RAB43 readthrough [Source:HGNC Symbol;Acc:HGNC:42969] |
| ENSG00000273706 | HGLibA_26448 | 25 | -0.908559917 | LHX1 | LIM homeobox 1 [Source:HGNC Symbol;Acc:HGNC:6593] |
| ENSG00000277972 | HGLibA_09733 | 25 | -0.908559917 | CISD3 | CDGSH iron sulfur domain 3 [Source:HGNC Symbol;Acc:HGNC:27578] |
| ENSG00000279051 | HGLibA_34439 | 25 | -0.908559917 | OR6Q1 | olfactory receptor family 6 subfamily Q member 1 (gene/pseudogene) [Source:HGNC Symbol;Acc:HGNC:15302] |
| FTSJD1 | HGLibA_18142 | 25 | -0.908559917 | FTSJD1 | - |
| HIST1H4L | HGLibA_21520 | 25 | -0.908559917 | HIST1H4L | - |
| HIST2H2AC | HGLibA_21531 | 25 | -0.908559917 | HIST2H2AC | - |
| hsa-mir-100 | HGLibA_57148 | 25 | -0.908559917 | hsa-mir-100 | - |
| hsa-mir-1225 | HGLibA_57299 | 25 | -0.908559917 | hsa-mir-1225 | - |
| hsa-mir-1246 | HGLibA_57384 | 25 | -0.908559917 | hsa-mir-1246 | - |
| hsa-mir-129-2 | HGLibA_57633 | 25 | -0.908559917 | hsa-mir-129-2 | - |
| hsa-mir-133b | HGLibA_57782 | 25 | -0.908559917 | hsa-mir-133b | - |
| hsa-mir-1539 | HGLibA_57922 | 25 | -0.908559917 | hsa-mir-1539 | - |
| hsa-mir-184 | HGLibA_58000 | 25 | -0.908559917 | hsa-mir-184 | - |
| hsa-mir-1972-2 | HGLibA_58112 | 25 | -0.908559917 | hsa-mir-1972-2 | - |
| hsa-mir-3169 | HGLibA_58757 | 25 | -0.908559917 | hsa-mir-3169 | - |
| hsa-mir-3179-3 | HGLibA_58797 | 25 | -0.908559917 | hsa-mir-3179-3 | - |
| hsa-mir-3202-1 | HGLibA_58916 | 25 | -0.908559917 | hsa-mir-3202-1 | - |
| hsa-mir-320c-2 | HGLibA_58938 | 25 | -0.908559917 | hsa-mir-320c-2 | - |
| hsa-mir-342 | HGLibA_59024 | 25 | -0.908559917 | hsa-mir-342 | - |
| hsa-mir-365a | HGLibA_59187 | 25 | -0.908559917 | hsa-mir-365a | - |
| hsa-mir-3685 | HGLibA_59294 | 25 | -0.908559917 | hsa-mir-3685 | - |
| hsa-mir-377 | HGLibA_59421 | 25 | -0.908559917 | hsa-mir-377 | - |
| hsa-mir-379 | HGLibA_59469 | 25 | -0.908559917 | hsa-mir-379 | - |
| hsa-mir-4428 | HGLibA_60100 | 25 | -0.908559917 | hsa-mir-4428 | - |
| hsa-mir-4433 | HGLibA_60118 | 25 | -0.908559917 | hsa-mir-4433 | - |
| hsa-mir-4512 | HGLibA_60490 | 25 | -0.908559917 | hsa-mir-4512 | - |
| hsa-mir-4520b | HGLibA_60536 | 25 | -0.908559917 | hsa-mir-4520b | - |
| hsa-mir-4534 | HGLibA_60595 | 25 | -0.908559917 | hsa-mir-4534 | - |
| hsa-mir-4535 | HGLibA_60599 | 25 | -0.908559917 | hsa-mir-4535 | - |
| hsa-mir-4646 | HGLibA_60687 | 25 | -0.908559917 | hsa-mir-4646 | - |
| hsa-mir-4670 | HGLibA_60798 | 25 | -0.908559917 | hsa-mir-4670 | - |
| hsa-mir-4763 | HGLibA_61149 | 25 | -0.908559917 | hsa-mir-4763 | - |
| hsa-mir-4772 | HGLibA_61187 | 25 | -0.908559917 | hsa-mir-4772 | - |
| hsa-mir-4779 | HGLibA_61223 | 25 | -0.908559917 | hsa-mir-4779 | - |
| hsa-mir-5047 | HGLibA_61474 | 25 | -0.908559917 | hsa-mir-5047 | - |
| hsa-mir-517b | HGLibA_61627 | 25 | -0.908559917 | hsa-mir-517b | - |
| hsa-mir-523 | HGLibA_61779 | 25 | -0.908559917 | hsa-mir-523 | - |

| | | | | | |
|---|---|---|---|---|---|
| hsa-mir-548d-1 | HGLibA_61960 | 25 | -0.908559917 | hsa-mir-548d-1 | - |
| hsa-mir-616 | HGLibA_62698 | 25 | -0.908559917 | hsa-mir-616 | - |
| hsa-mir-6511a-2 | HGLibA_62900 | 25 | -0.908559917 | hsa-mir-6511a-2 | - |
| hsa-mir-6788 | HGLibA_63330 | 25 | -0.908559917 | hsa-mir-6788 | - |
| hsa-mir-6853 | HGLibA_63583 | 25 | -0.908559917 | hsa-mir-6853 | - |
| hsa-mir-7855 | HGLibA_64010 | 25 | -0.908559917 | hsa-mir-7855 | - |
| hsa-mir-8059 | HGLibA_64076 | 25 | -0.908559917 | hsa-mir-8059 | - |
| hsa-mir-8060 | HGLibA_64077 | 25 | -0.908559917 | hsa-mir-8060 | - |
| hsa-mir-889 | HGLibA_64235 | 25 | -0.908559917 | hsa-mir-889 | - |
| hsa-mir-937 | HGLibA_64323 | 25 | -0.908559917 | hsa-mir-937 | - |
| hsa-mir-941-1 | HGLibA_64337 | 25 | -0.908559917 | hsa-mir-941-1 | - |
| LOC100144595 | HGLibA_26813 | 25 | -0.908559917 | LOC100144595 | - |
| LSMD1 | HGLibA_27555 | 25 | -0.908559917 | LSMD1 | - |
| MB21D1 | HGLibA_28456 | 25 | -0.908559917 | MB21D1 | - |
| MNF1 | HGLibA_29570 | 25 | -0.908559917 | MNF1 | - |
| P2RY8_Y | HGLibA_34898 | 25 | -0.908559917 | P2RY8_Y | - |
| YAE1D1 | HGLibA_54625 | 25 | -0.908559917 | YAE1D1 | - |
| 3-Mar | HGLibA_28334 | 24 | -0.91760526 | 3-Mar | - |
| AKAP2 | HGLibA_01426 | 24 | -0.91760526 | AKAP2 | - |
| B3GALTL | HGLibA_03969 | 24 | -0.91760526 | B3GALTL | - |
| C17orf104 | HGLibA_05521 | 24 | -0.91760526 | C17orf104 | - |
| C4orf27 | HGLibA_06310 | 24 | -0.91760526 | C4orf27 | - |
| C9orf9 | HGLibA_06774 | 24 | -0.91760526 | C9orf9 | - |
| DGCR14 | HGLibA_13043 | 24 | -0.91760526 | DGCR14 | - |
| ENSG00000005981 | HGLibA_03239 | 24 | -0.91760526 | ASB4 | ankyrin repeat and SOCS box containing 4 [Source:HGNC Symbol;Acc:HGNC:16009] |
| ENSG00000011523 | HGLibA_09154 | 24 | -0.91760526 | CEP68 | centrosomal protein 68 [Source:HGNC Symbol;Acc:HGNC:29076] |
| ENSG00000012061 | HGLibA_15431 | 24 | -0.91760526 | ERCC1 | endonuclease non-catalytic subunit [Source:HGNC Symbol;Acc:HGNC:3433]" |
| ENSG00000012124 | HGLibA_08296 | 24 | -0.91760526 | CD22 | CD22 molecule [Source:HGNC Symbol;Acc:HGNC:1643] |
| ENSG00000025434 | HGLibA_32582 | 24 | -0.91760526 | NR1H3 | nuclear receptor subfamily 1 group H member 3 [Source:HGNC Symbol;Acc:HGNC:7966] |
| ENSG00000029559 | HGLibA_22482 | 24 | -0.91760526 | IBSP | integrin binding sialoprotein [Source:HGNC Symbol;Acc:HGNC:5341] |
| ENSG00000040608 | HGLibA_42413 | 24 | -0.91760526 | RTN4R | reticulon 4 receptor [Source:HGNC Symbol;Acc:HGNC:18601] |
| ENSG00000047315 | HGLibA_37567 | 24 | -0.91760526 | POLR2B | RNA polymerase II subunit B [Source:HGNC Symbol;Acc:HGNC:9188] |
| ENSG00000047932 | HGLibA_19672 | 24 | -0.91760526 | GOPC | golgi associated PDZ and coiled-coil motif containing [Source:HGNC Symbol;Acc:HGNC:17643] |
| ENSG00000057663 | HGLibA_03499 | 24 | -0.91760526 | ATG5 | autophagy related 5 [Source:HGNC Symbol;Acc:HGNC:589] |
| ENSG00000062282 | HGLibA_13038 | 24 | -0.91760526 | DGAT2 | diacylglycerol O-acyltransferase 2 [Source:HGNC Symbol;Acc:HGNC:16940] |
| ENSG00000065060 | HGLibA_52949 | 24 | -0.91760526 | UHRF1BP1 | UHRF1 binding protein 1 [Source:HGNC Symbol;Acc:HGNC:21216] |
| ENSG00000067057 | HGLibA_36170 | 24 | -0.91760526 | PFKP | platelet [Source:HGNC Symbol;Acc:HGNC:8878]" |
| ENSG00000067177 | HGLibA_36402 | 24 | -0.91760526 | PHKA1 | phosphorylase kinase regulatory subunit alpha 1 [Source:HGNC Symbol;Acc:HGNC:8925] |
| ENSG00000072422 | HGLibA_41172 | 24 | -0.91760526 | RHOBTB1 | Rho related BTB domain containing 1 [Source:HGNC Symbol;Acc:HGNC:18738] |
| ENSG00000073910 | HGLibA_18052 | 24 | -0.91760526 | FRY | FRY microtubule binding protein [Source:HGNC Symbol;Acc:HGNC:20367] |
| ENSG00000077943 | HGLibA_23746 | 24 | -0.91760526 | ITGA8 | integrin subunit alpha 8 [Source:HGNC Symbol;Acc:HGNC:6144] |
| ENSG00000079263 | HGLibA_46291 | 24 | -0.91760526 | SP140 | SP140 nuclear body protein [Source:HGNC Symbol;Acc:HGNC:17133] |
| ENSG00000085365 | HGLibA_42836 | 24 | -0.91760526 | SCAMP1 | secretory carrier membrane protein 1 [Source:HGNC Symbol;Acc:HGNC:10563] |
| ENSG00000089057 | HGLibA_44610 | 24 | -0.91760526 | SLC23A2 | solute carrier family 23 member 2 [Source:HGNC Symbol;Acc:HGNC:10973] |
| ENSG00000090989 | HGLibA_15732 | 24 | -0.91760526 | EXOC1 | exocyst complex component 1 [Source:HGNC Symbol;Acc:HGNC:30380] |
| ENSG00000091583 | HGLibA_02524 | 24 | -0.91760526 | APOH | apolipoprotein H [Source:HGNC Symbol;Acc:HGNC:616] |
| ENSG00000095574 | HGLibA_22940 | 24 | -0.91760526 | IKZF5 | IKAROS family zinc finger 5 [Source:HGNC Symbol;Acc:HGNC:14283] |
| ENSG00000100836 | HGLibA_34943 | 24 | -0.91760526 | PABPN1 | poly(A) binding protein nuclear 1 [Source:HGNC Symbol;Acc:HGNC:8565] |
| ENSG00000101882 | HGLibA_32000 | 24 | -0.91760526 | NKAP | NFKB activating protein [Source:HGNC Symbol;Acc:HGNC:29873] |
| ENSG00000102385 | HGLibA_13987 | 24 | -0.91760526 | DRP2 | dystrophin related protein 2 [Source:HGNC Symbol;Acc:HGNC:3032] |
| ENSG00000102710 | HGLibA_47806 | 24 | -0.91760526 | SUPT20H | SAGA complex component [Source:HGNC Symbol;Acc:HGNC:20596]" |
| ENSG00000103042 | HGLibA_45078 | 24 | -0.91760526 | SLC38A7 | solute carrier family 38 member 7 [Source:HGNC Symbol;Acc:HGNC:25582] |
| ENSG00000103043 | HGLibA_53484 | 24 | -0.91760526 | VAC14 | PIKFYVE complex component [Source:HGNC Symbol;Acc:HGNC:25507]" |
| ENSG00000103966 | HGLibA_14676 | 24 | -0.91760526 | EHD4 | EH domain containing 4 [Source:HGNC Symbol;Acc:HGNC:3245] |
| ENSG00000105397 | HGLibA_52455 | 24 | -0.91760526 | TYK2 | tyrosine kinase 2 [Source:HGNC Symbol;Acc:HGNC:12440] |
| ENSG00000105993 | HGLibA_13549 | 24 | -0.91760526 | DNAJB6 | DnaJ heat shock protein family (Hsp40) member B6 [Source:HGNC Symbol;Acc:HGNC:14888] |
| ENSG00000107551 | HGLibA_40383 | 24 | -0.91760526 | RASSF4 | Ras association domain family member 4 [Source:HGNC Symbol;Acc:HGNC:20793] |
| ENSG00000109107 | HGLibA_01597 | 24 | -0.91760526 | ALDOC | fructose-bisphosphate C [Source:HGNC Symbol;Acc:HGNC:418]" |
| ENSG00000109132 | HGLibA_36453 | 24 | -0.91760526 | PHOX2B | paired like homeobox 2B [Source:HGNC Symbol;Acc:HGNC:9143] |
| ENSG00000109321 | HGLibA_02641 | 24 | -0.91760526 | AREG | amphiregulin [Source:HGNC Symbol;Acc:HGNC:651] |
| ENSG00000109576 | HGLibA_00042 | 24 | -0.91760526 | AADAT | aminoadipate aminotransferase [Source:HGNC Symbol;Acc:HGNC:17929] |
| ENSG00000110315 | HGLibA_41523 | 24 | -0.91760526 | RNF141 | ring finger protein 141 [Source:HGNC Symbol;Acc:HGNC:21159] |
| ENSG00000110429 | HGLibA_17063 | 24 | -0.91760526 | FBXO3 | F-box protein 3 [Source:HGNC Symbol;Acc:HGNC:13582] |
| ENSG00000110651 | HGLibA_08457 | 24 | -0.91760526 | CD81 | CD81 molecule [Source:HGNC Symbol;Acc:HGNC:1701] |
| ENSG00000110693 | HGLibA_46270 | 24 | -0.91760526 | SOX6 | SRY-box 6 [Source:HGNC Symbol;Acc:HGNC:16421] |
| ENSG00000112110 | HGLibA_29916 | 24 | -0.91760526 | MRPL18 | mitochondrial ribosomal protein L18 [Source:HGNC Symbol;Acc:HGNC:14477] |
| ENSG00000112149 | HGLibA_08463 | 24 | -0.91760526 | CD83 | CD83 molecule [Source:HGNC Symbol;Acc:HGNC:1703] |
| ENSG00000113638 | HGLibA_52118 | 24 | -0.91760526 | TTC33 | tetratricopeptide repeat domain 33 [Source:HGNC Symbol;Acc:HGNC:29959] |
| ENSG00000114113 | HGLibA_40633 | 24 | -0.91760526 | RBP2 | retinol binding protein 2 [Source:HGNC Symbol;Acc:HGNC:9920] |
| ENSG00000114757 | HGLibA_36119 | 24 | -0.91760526 | PEX5L | peroxisomal biogenesis factor 5 like [Source:HGNC Symbol;Acc:HGNC:30024] |
| ENSG00000115687 | HGLibA_35267 | 24 | -0.91760526 | PASK | PAS domain containing serine/threonine kinase [Source:HGNC Symbol;Acc:HGNC:17270] |
| ENSG00000116062 | HGLibA_30201 | 24 | -0.91760526 | MSH6 | mutS homolog 6 [Source:HGNC Symbol;Acc:HGNC:7329] |
| ENSG00000120709 | HGLibA_16642 | 24 | -0.91760526 | FAM53C | family with sequence similarity 53 member C [Source:HGNC Symbol;Acc:HGNC:1336] |
| ENSG00000122224 | HGLibA_27689 | 24 | -0.91760526 | LY9 | lymphocyte antigen 9 [Source:HGNC Symbol;Acc:HGNC:6730] |
| ENSG00000122970 | HGLibA_22777 | 24 | -0.91760526 | IFT81 | intraflagellar transport 81 [Source:HGNC Symbol;Acc:HGNC:14313] |
| ENSG00000123472 | HGLibA_03798 | 24 | -0.91760526 | ATPAF1 | ATP synthase mitochondrial F1 complex assembly factor 1 [Source:HGNC Symbol;Acc:HGNC:18803] |
| ENSG00000123505 | HGLibA_01756 | 24 | -0.91760526 | AMD1 | adenosylmethionine decarboxylase 1 [Source:HGNC Symbol;Acc:HGNC:457] |
| ENSG00000123737 | HGLibA_15796 | 24 | -0.91760526 | EXOSC9 | exosome component 9 [Source:HGNC Symbol;Acc:HGNC:9137] |
| ENSG00000124406 | HGLibA_03776 | 24 | -0.91760526 | ATP8A1 | ATPase phospholipid transporting 8A1 [Source:HGNC Symbol;Acc:HGNC:13531] |
| ENSG00000124532 | HGLibA_30117 | 24 | -0.91760526 | MRS2 | magnesium transporter MRS2 [Source:HGNC Symbol;Acc:HGNC:13785] |
| ENSG00000125629 | HGLibA_23379 | 24 | -0.91760526 | INSIG2 | insulin induced gene 2 [Source:HGNC Symbol;Acc:HGNC:20452] |
| ENSG00000129292 | HGLibA_36366 | 24 | -0.91760526 | PHF20L1 | PHD finger protein 20 like 1 [Source:HGNC Symbol;Acc:HGNC:24280] |
| ENSG00000130224 | HGLibA_27141 | 24 | -0.91760526 | LRCH2 | leucine rich repeats and calponin homology domain containing 2 [Source:HGNC Symbol;Acc:HGNC:29292] |
| ENSG00000130255 | HGLibA_41952 | 24 | -0.91760526 | RPL36 | ribosomal protein L36 [Source:HGNC Symbol;Acc:HGNC:13631] |
| ENSG00000130522 | HGLibA_24010 | 24 | -0.91760526 | JUND | AP-1 transcription factor subunit [Source:HGNC Symbol;Acc:HGNC:6206]" |
| ENSG00000130724 | HGLibA_09477 | 24 | -0.91760526 | CHMP2A | charged multivesicular body protein 2A [Source:HGNC Symbol;Acc:HGNC:30216] |
| ENSG00000130749 | HGLibA_54977 | 24 | -0.91760526 | ZC3H4 | zinc finger CCCH-type containing 4 [Source:HGNC Symbol;Acc:HGNC:17808] |
| ENSG00000131408 | HGLibA_32579 | 24 | -0.91760526 | NR1H2 | nuclear receptor subfamily 1 group H member 2 [Source:HGNC Symbol;Acc:HGNC:7965] |
| ENSG00000132676 | HGLibA_12319 | 24 | -0.91760526 | DAP3 | death associated protein 3 [Source:HGNC Symbol;Acc:HGNC:2673] |
| ENSG00000134899 | HGLibA_15444 | 24 | -0.91760526 | ERCC5 | endonuclease [Source:HGNC Symbol;Acc:HGNC:3437]" |
| ENSG00000135317 | HGLibA_46040 | 24 | -0.91760526 | SNX14 | sorting nexin 14 [Source:HGNC Symbol;Acc:HGNC:14977] |
| ENSG00000135972 | HGLibA_30112 | 24 | -0.91760526 | MRPS9 | mitochondrial ribosomal protein S9 [Source:HGNC Symbol;Acc:HGNC:14501] |
| ENSG00000135999 | HGLibA_15270 | 24 | -0.91760526 | EPC2 | enhancer of polycomb homolog 2 [Source:HGNC Symbol;Acc:HGNC:24543] |
| ENSG00000136026 | HGLibA_09761 | 24 | -0.91760526 | CKAP4 | cytoskeleton associated protein 4 [Source:HGNC Symbol;Acc:HGNC:16991] |
| ENSG00000136270 | HGLibA_48639 | 24 | -0.91760526 | TBRG4 | transforming growth factor beta regulator 4 [Source:HGNC Symbol;Acc:HGNC:17443] |
| ENSG00000137168 | HGLibA_37940 | 24 | -0.91760526 | PPIL1 | peptidylprolyl isomerase like 1 [Source:HGNC Symbol;Acc:HGNC:9260] |
| ENSG00000137270 | HGLibA_18801 | 24 | -0.91760526 | GCM1 | glial cells missing homolog 1 [Source:HGNC Symbol;Acc:HGNC:4197] |
| ENSG00000138107 | HGLibA_00649 | 24 | -0.91760526 | ACTR1A | ARP1 actin related protein 1 homolog A [Source:HGNC Symbol;Acc:HGNC:167] |
| ENSG00000138750 | HGLibA_33060 | 24 | -0.91760526 | NUP54 | nucleoporin 54 [Source:HGNC Symbol;Acc:HGNC:17359] |
| ENSG00000138834 | HGLibA_28292 | 24 | -0.91760526 | MAPK8IP3 | mitogen-activated protein kinase 8 interacting protein 3 [Source:HGNC Symbol;Acc:HGNC:6884] |
| ENSG00000139174 | HGLibA_38469 | 24 | -0.91760526 | PRICKLE1 | prickle planar cell polarity protein 1 [Source:HGNC Symbol;Acc:HGNC:17019] |
| ENSG00000141994 | HGLibA_14120 | 24 | -0.91760526 | DUS3L | dihydrouridine synthase 3 like [Source:HGNC Symbol;Acc:HGNC:26920] |
| ENSG00000142544 | HGLibA_11750 | 24 | -0.91760526 | CTU1 | cytosolic thiouridylase subunit 1 [Source:HGNC Symbol;Acc:HGNC:29590] |
| ENSG00000143514 | HGLibA_51056 | 24 | -0.91760526 | TP53BP2 | tumor protein p53 binding protein 2 [Source:HGNC Symbol;Acc:HGNC:12000] |
| ENSG00000143870 | HGLibA_35887 | 24 | -0.91760526 | PDIA6 | protein disulfide isomerase family A member 6 [Source:HGNC Symbol;Acc:HGNC:30168] |
| ENSG00000144045 | HGLibA_13939 | 24 | -0.91760526 | DQX1 | DEAQ-box RNA dependent ATPase 1 [Source:HGNC Symbol;Acc:HGNC:20410] |
| ENSG00000144445 | HGLibA_24039 | 24 | -0.91760526 | KANSL1L | KAT8 regulatory NSL complex subunit 1 like [Source:HGNC Symbol;Acc:HGNC:26310] |
| ENSG00000144635 | HGLibA_14240 | 24 | -0.91760526 | DYNC1LI1 | dynein cytoplasmic 1 light intermediate chain 1 [Source:HGNC Symbol;Acc:HGNC:18745] |
| ENSG00000145506 | HGLibA_32011 | 24 | -0.91760526 | NKD2 | naked cuticle homolog 2 [Source:HGNC Symbol;Acc:HGNC:17046] |
| ENSG00000146122 | HGLibA_12261 | 24 | -0.91760526 | DAAM2 | dishevelled associated activator of morphogenesis 2 [Source:HGNC Symbol;Acc:HGNC:18143] |
| ENSG00000146592 | HGLibA_11105 | 24 | -0.91760526 | CREB5 | cAMP responsive element binding protein 5 [Source:HGNC Symbol;Acc:HGNC:16844] |
| ENSG00000147180 | HGLibA_56614 | 24 | -0.91760526 | ZNF711 | zinc finger protein 711 [Source:HGNC Symbol;Acc:HGNC:13128] |
| ENSG00000147509 | HGLibA_41081 | 24 | -0.91760526 | RGS20 | regulator of G protein signaling 20 [Source:HGNC Symbol;Acc:HGNC:14600] |
| ENSG00000149182 | HGLibA_02665 | 24 | -0.91760526 | ARFGAP2 | ADP ribosylation factor GTPase activating protein 2 [Source:HGNC Symbol;Acc:HGNC:13504] |
| ENSG00000151239 | HGLibA_52369 | 24 | -0.91760526 | TWF1 | twinfilin actin binding protein 1 [Source:HGNC Symbol;Acc:HGNC:9620] |
| ENSG00000151292 | HGLibA_11398 | 24 | -0.91760526 | CSNK1G3 | casein kinase 1 gamma 3 [Source:HGNC Symbol;Acc:HGNC:2456] |
| ENSG00000151353 | HGLibA_50093 | 24 | -0.91760526 | TMEM18 | transmembrane protein 18 [Source:HGNC Symbol;Acc:HGNC:25257] |
| ENSG00000151491 | HGLibA_15375 | 24 | -0.91760526 | EPS8 | epidermal growth factor receptor pathway substrate 8 [Source:HGNC Symbol;Acc:HGNC:3420] |
| ENSG00000153767 | HGLibA_20531 | 24 | -0.91760526 | GTF2E1 | general transcription factor IIE subunit 1 [Source:HGNC Symbol;Acc:HGNC:4650] |
| ENSG00000154001 | HGLibA_38183 | 24 | -0.91760526 | PPP2R5E | protein phosphatase 2 regulatory subunit B'epsilon [Source:HGNC Symbol;Acc:HGNC:9313] |
| ENSG00000155890 | HGLibA_51497 | 24 | -0.91760526 | TRIM42 | tripartite motif containing 42 [Source:HGNC Symbol;Acc:HGNC:19014] |
| ENSG00000156006 | HGLibA_31176 | 24 | -0.91760526 | NAT2 | N-acetyltransferase 2 [Source:HGNC Symbol;Acc:HGNC:7646] |
| ENSG00000156603 | HGLibA_28770 | 24 | -0.91760526 | MED19 | mediator complex subunit 19 [Source:HGNC Symbol;Acc:HGNC:29600] |
| ENSG00000157303 | HGLibA_47849 | 24 | -0.91760526 | SUSD3 | sushi domain containing 3 [Source:HGNC Symbol;Acc:HGNC:28391] |
| ENSG00000157510 | HGLibA_01100 | 24 | -0.91760526 | AFAP1L1 | actin filament associated protein 1 like 1 [Source:HGNC Symbol;Acc:HGNC:26714] |
| ENSG00000157680 | HGLibA_13077 | 24 | -0.91760526 | DGKI | diacylglycerol kinase iota [Source:HGNC Symbol;Acc:HGNC:2855] |
| ENSG00000159110 | HGLibA_22699 | 24 | -0.91760526 | IFNAR2 | interferon alpha and beta receptor subunit 2 [Source:HGNC Symbol;Acc:HGNC:5433] |
| ENSG00000160360 | HGLibA_20155 | 24 | -0.91760526 | GPSM1 | G protein signaling modulator 1 [Source:HGNC Symbol;Acc:HGNC:17858] |
| ENSG00000161021 | HGLibA_28042 | 24 | -0.91760526 | MAML1 | mastermind like transcriptional coactivator 1 [Source:HGNC Symbol;Acc:HGNC:13632] |
| ENSG00000161381 | HGLibA_37251 | 24 | -0.91760526 | PLXDC1 | plexin domain containing 1 [Source:HGNC Symbol;Acc:HGNC:20945] |
| ENSG00000162396 | HGLibA_35254 | 24 | -0.91760526 | PARS2 | mitochondrial [Source:HGNC Symbol;Acc:HGNC:30563]" |
| ENSG00000162738 | HGLibA_53516 | 24 | -0.91760526 | VANGL2 | VANGL planar cell polarity protein 2 [Source:HGNC Symbol;Acc:HGNC:15511] |
| ENSG00000162909 | HGLibA_07164 | 24 | -0.91760526 | CAPN2 | calpain 2 [Source:HGNC Symbol;Acc:HGNC:1479] |
| ENSG00000163399 | HGLibA_03582 | 24 | -0.91760526 | ATP1A1 | ATPase Na+/K+ transporting subunit alpha 1 [Source:HGNC Symbol;Acc:HGNC:799] |
| ENSG00000163497 | HGLibA_17308 | 24 | -0.91760526 | FEV | ETS transcription factor [Source:HGNC Symbol;Acc:HGNC:18562]" |

| | | | | | |
|---|---|---|---|---|---|
| ENSG00000164024 | HGLibA_28927 | 24 | -0.91760526 | METAP1 | methionyl aminopeptidase 1 [Source:HGNC Symbol;Acc:HGNC:15789] |
| ENSG00000164176 | HGLibA_14466 | 24 | -0.91760526 | EDIL3 | EGF like repeats and discoidin domains 3 [Source:HGNC Symbol;Acc:HGNC:3173] |
| ENSG00000164398 | HGLibA_00567 | 24 | -0.91760526 | ACSL6 | acyl-CoA synthetase long chain family member 6 [Source:HGNC Symbol;Acc:HGNC:16496] |
| ENSG00000164967 | HGLibA_42031 | 24 | -0.91760526 | RPP25L | ribonuclease P/MRP subunit p25 like [Source:HGNC Symbol;Acc:HGNC:19909] |
| ENSG00000165125 | HGLibA_51798 | 24 | -0.91760526 | TRPV6 | transient receptor potential cation channel subfamily V member 6 [Source:HGNC Symbol;Acc:HGNC:14006] |
| ENSG00000165568 | HGLibA_01495 | 24 | -0.91760526 | AKR1E2 | aldo-keto reductase family 1 member E2 [Source:HGNC Symbol;Acc:HGNC:23437] |
| ENSG00000165695 | HGLibA_01396 | 24 | -0.91760526 | AK8 | adenylate kinase 8 [Source:HGNC Symbol;Acc:HGNC:26526] |
| ENSG00000166912 | HGLibA_30413 | 24 | -0.91760526 | MTMR10 | myotubularin related protein 10 [Source:HGNC Symbol;Acc:HGNC:25999] |
| ENSG00000166987 | HGLibA_28492 | 24 | -0.91760526 | MBD6 | methyl-CpG binding domain protein 6 [Source:HGNC Symbol;Acc:HGNC:20445] |
| ENSG00000167194 | HGLibA_05500 | 24 | -0.91760526 | C16orf92 | chromosome 16 open reading frame 92 [Source:HGNC Symbol;Acc:HGNC:26346] |
| ENSG00000167733 | HGLibA_22164 | 24 | -0.91760526 | HSD11B1L | hydroxysteroid 11-beta dehydrogenase 1 like [Source:HGNC Symbol;Acc:HGNC:30419] |
| ENSG00000167799 | HGLibA_32992 | 24 | -0.91760526 | NUDT8 | nudix hydrolase 8 [Source:HGNC Symbol;Acc:HGNC:8055] |
| ENSG00000168454 | HGLibA_52421 | 24 | -0.91760526 | TXNDC2 | thioredoxin domain containing 2 [Source:HGNC Symbol;Acc:HGNC:16470] |
| ENSG00000168828 | HGLibA_33625 | 24 | -0.91760526 | OR13J1 | olfactory receptor family 13 subfamily J member 1 [Source:HGNC Symbol;Acc:HGNC:15108] |
| ENSG00000168899 | HGLibA_53501 | 24 | -0.91760526 | VAMP5 | vesicle associated membrane protein 5 [Source:HGNC Symbol;Acc:HGNC:12646] |
| ENSG00000169413 | HGLibA_41413 | 24 | -0.91760526 | RNASE6 | ribonuclease A family member k6 [Source:HGNC Symbol;Acc:HGNC:10048] |
| ENSG00000169679 | HGLibA_04988 | 24 | -0.91760526 | BUB1 | BUB1 mitotic checkpoint serine/threonine kinase [Source:HGNC Symbol;Acc:HGNC:1148] |
| ENSG00000169992 | HGLibA_32070 | 24 | -0.91760526 | NLGN2 | neuroligin 2 [Source:HGNC Symbol;Acc:HGNC:14290] |
| ENSG00000170027 | HGLibA_54745 | 24 | -0.91760526 | YWHAG | tyrosine 3-monooxygenase/tryptophan 5-monooxygenase activation protein gamma [Source:HGNC Symbol;Acc:HGNC:12852] |
| ENSG00000170085 | HGLibA_44180 | 24 | -0.91760526 | SIMC1 | SUMO interacting motifs containing 1 [Source:HGNC Symbol;Acc:HGNC:24779] |
| ENSG00000170323 | HGLibA_15919 | 24 | -0.91760526 | FABP4 | fatty acid binding protein 4 [Source:HGNC Symbol;Acc:HGNC:3559] |
| ENSG00000170890 | HGLibA_36883 | 24 | -0.91760526 | PLA2G1B | phospholipase A2 group IB [Source:HGNC Symbol;Acc:HGNC:9030] |
| ENSG00000171116 | HGLibA_22236 | 24 | -0.91760526 | HSFX1 | X-linked 1 [Source:HGNC Symbol;Acc:HGNC:29603]" |
| ENSG00000171450 | HGLibA_08779 | 24 | -0.91760526 | CDK5R2 | cyclin dependent kinase 5 regulatory subunit 2 [Source:HGNC Symbol;Acc:HGNC:1776] |
| ENSG00000171469 | HGLibA_56244 | 24 | -0.91760526 | ZNF561 | zinc finger protein 561 [Source:HGNC Symbol;Acc:HGNC:28684] |
| ENSG00000171791 | HGLibA_04296 | 24 | -0.91760526 | BCL2 | apoptosis regulator [Source:HGNC Symbol;Acc:HGNC:990]" |
| ENSG00000172382 | HGLibA_38920 | 24 | -0.91760526 | PRSS27 | serine protease 27 [Source:HGNC Symbol;Acc:HGNC:15475] |
| ENSG00000172403 | HGLibA_48020 | 24 | -0.91760526 | SYNPO2 | synaptopodin 2 [Source:HGNC Symbol;Acc:HGNC:17732] |
| ENSG00000172828 | HGLibA_09223 | 24 | -0.91760526 | CES3 | carboxylesterase 3 [Source:HGNC Symbol;Acc:HGNC:1865] |
| ENSG00000173285 | HGLibA_33529 | 24 | -0.91760526 | OR10K1 | olfactory receptor family 10 subfamily K member 1 [Source:HGNC Symbol;Acc:HGNC:14693] |
| ENSG00000173404 | HGLibA_23394 | 24 | -0.91760526 | INSM1 | INSM transcriptional repressor 1 [Source:HGNC Symbol;Acc:HGNC:6090] |
| ENSG00000173744 | HGLibA_01184 | 24 | -0.91760526 | AGFG1 | ArfGAP with FG repeats 1 [Source:HGNC Symbol;Acc:HGNC:5175] |
| ENSG00000173786 | HGLibA_10356 | 24 | -0.91760526 | CNP | 3'-cyclic nucleotide 3' phosphodiesterase [Source:HGNC Symbol;Acc:HGNC:2158]" |
| ENSG00000174885 | HGLibA_32128 | 24 | -0.91760526 | NLRP6 | NLR family pyrin domain containing 6 [Source:HGNC Symbol;Acc:HGNC:22944] |
| ENSG00000176742 | HGLibA_34137 | 24 | -0.91760526 | OR51V1 | olfactory receptor family 51 subfamily V member 1 [Source:HGNC Symbol;Acc:HGNC:19597] |
| ENSG00000177311 | HGLibA_54870 | 24 | -0.91760526 | ZBTB38 | zinc finger and BTB domain containing 38 [Source:HGNC Symbol;Acc:HGNC:26636] |
| ENSG00000177363 | HGLibA_27481 | 24 | -0.91760526 | LRRN4CL | LRRN4 C-terminal like [Source:HGNC Symbol;Acc:HGNC:33724] |
| ENSG00000178586 | HGLibA_34378 | 24 | -0.91760526 | OR6B3 | olfactory receptor family 6 subfamily B member 3 [Source:HGNC Symbol;Acc:HGNC:15042] |
| ENSG00000178602 | HGLibA_34748 | 24 | -0.91760526 | OTOS | otospiralin [Source:HGNC Symbol;Acc:HGNC:22644] |
| ENSG00000179222 | HGLibA_27969 | 24 | -0.91760526 | MAGED1 | MAGE family member D1 [Source:HGNC Symbol;Acc:HGNC:6813] |
| ENSG00000179364 | HGLibA_34956 | 24 | -0.91760526 | PACS2 | phosphofurin acidic cluster sorting protein 2 [Source:HGNC Symbol;Acc:HGNC:23794] |
| ENSG00000179580 | HGLibA_41546 | 24 | -0.91760526 | RNF151 | ring finger protein 151 [Source:HGNC Symbol;Acc:HGNC:23235] |
| ENSG00000180182 | HGLibA_28755 | 24 | -0.91760526 | MED14 | mediator complex subunit 14 [Source:HGNC Symbol;Acc:HGNC:2370] |
| ENSG00000180433 | HGLibA_34425 | 24 | -0.91760526 | OR6K6 | olfactory receptor family 6 subfamily K member 6 [Source:HGNC Symbol;Acc:HGNC:15033] |
| ENSG00000180483 | HGLibA_12868 | 24 | -0.91760526 | DEFB119 | defensin beta 119 [Source:HGNC Symbol;Acc:HGNC:18099] |
| ENSG00000181038 | HGLibA_28991 | 24 | -0.91760526 | METTL23 | methyltransferase like 23 [Source:HGNC Symbol;Acc:HGNC:26988] |
| ENSG00000181195 | HGLibA_36049 | 24 | -0.91760526 | PENK | proenkephalin [Source:HGNC Symbol;Acc:HGNC:8831] |
| ENSG00000182415 | HGLibA_08911 | 24 | -0.91760526 | CDY2A | chromodomain Y-linked 2A [Source:HGNC Symbol;Acc:HGNC:1810] |
| ENSG00000182902 | HGLibA_44659 | 24 | -0.91760526 | SLC25A18 | solute carrier family 25 member 18 [Source:HGNC Symbol;Acc:HGNC:10988] |
| ENSG00000184408 | HGLibA_24178 | 24 | -0.91760526 | KCND2 | potassium voltage-gated channel subfamily D member 2 [Source:HGNC Symbol;Acc:HGNC:6238] |
| ENSG00000184434 | HGLibA_27276 | 24 | -0.91760526 | LRRC19 | leucine rich repeat containing 19 [Source:HGNC Symbol;Acc:HGNC:23379] |
| ENSG00000185002 | HGLibA_40977 | 24 | -0.91760526 | RFX6 | regulatory factor X6 [Source:HGNC Symbol;Acc:HGNC:21478] |
| ENSG00000185101 | HGLibA_02194 | 24 | -0.91760526 | ANO9 | anoctamin 9 [Source:HGNC Symbol;Acc:HGNC:20679] |
| ENSG00000185219 | HGLibA_56016 | 24 | -0.91760526 | ZNF445 | zinc finger protein 445 [Source:HGNC Symbol;Acc:HGNC:21018] |
| ENSG00000185933 | HGLibA_07012 | 24 | -0.91760526 | CALHM1 | calcium homeostasis modulator 1 [Source:HGNC Symbol;Acc:HGNC:23494] |
| ENSG00000185963 | HGLibA_04496 | 24 | -0.91760526 | BICD2 | BICD cargo adaptor 2 [Source:HGNC Symbol;Acc:HGNC:17208] |
| ENSG00000186074 | HGLibA_08342 | 24 | -0.91760526 | CD300LF | CD300 molecule like family member f [Source:HGNC Symbol;Acc:HGNC:29883] |
| ENSG00000186272 | HGLibA_55522 | 24 | -0.91760526 | ZNF17 | zinc finger protein 17 [Source:HGNC Symbol;Acc:HGNC:12958] |
| ENSG00000187288 | HGLibA_09703 | 24 | -0.91760526 | CIDEC | cell death inducing DFFA like effector c [Source:HGNC Symbol;Acc:HGNC:24229] |
| ENSG00000196406 | HGLibA_46394 | 24 | -0.91760526 | SPANXD | SPANX family member D [Source:HGNC Symbol;Acc:HGNC:14332] |
| ENSG00000196517 | HGLibA_45342 | 24 | -0.91760526 | SLC6A9 | solute carrier family 6 member 9 [Source:HGNC Symbol;Acc:HGNC:11056] |
| ENSG00000196878 | HGLibA_25963 | 24 | -0.91760526 | LAMB3 | laminin subunit beta 3 [Source:HGNC Symbol;Acc:HGNC:6490] |
| ENSG00000197124 | HGLibA_56530 | 24 | -0.91760526 | ZNF682 | zinc finger protein 682 [Source:HGNC Symbol;Acc:HGNC:28857] |
| ENSG00000197557 | HGLibA_52104 | 24 | -0.91760526 | TTC30A | tetratricopeptide repeat domain 30A [Source:HGNC Symbol;Acc:HGNC:25853] |
| ENSG00000197616 | HGLibA_30729 | 24 | -0.91760526 | MYH6 | myosin heavy chain 6 [Source:HGNC Symbol;Acc:HGNC:7576] |
| ENSG00000198301 | HGLibA_43093 | 24 | -0.91760526 | SDAD1 | SDA1 domain containing 1 [Source:HGNC Symbol;Acc:HGNC:25537] |
| ENSG00000198363 | HGLibA_03350 | 24 | -0.91760526 | ASPH | aspartate beta-hydroxylase [Source:HGNC Symbol;Acc:HGNC:757] |
| ENSG00000198443 | HGLibA_25748 | 24 | -0.91760526 | KRTAP4-1 | keratin associated protein 4-1 [Source:HGNC Symbol;Acc:HGNC:18907] |
| ENSG00000198556 | HGLibA_56758 | 24 | -0.91760526 | ZNF789 | zinc finger protein 789 [Source:HGNC Symbol;Acc:HGNC:27801] |
| ENSG00000198798 | HGLibA_27948 | 24 | -0.91760526 | MAGEB3 | MAGE family member B3 [Source:HGNC Symbol;Acc:HGNC:6810] |
| ENSG00000203485 | HGLibA_23279 | 24 | -0.91760526 | INF2 | FH2 and WH2 domain containing [Source:HGNC Symbol;Acc:HGNC:23791]" |
| ENSG00000204882 | HGLibA_19995 | 24 | -0.91760526 | GPR20 | G protein-coupled receptor 20 [Source:HGNC Symbol;Acc:HGNC:4475] |
| ENSG00000213339 | HGLibA_39759 | 24 | -0.91760526 | QTRT1 | queuine tRNA-ribosyltransferase catalytic subunit 1 [Source:HGNC Symbol;Acc:HGNC:23797] |
| ENSG00000214029 | HGLibA_56900 | 24 | -0.91760526 | ZNF891 | zinc finger protein 891 [Source:HGNC Symbol;Acc:HGNC:38709] |
| ENSG00000214309 | HGLibA_28503 | 24 | -0.91760526 | MBLAC1 | metallo-beta-lactamase domain containing 1 [Source:HGNC Symbol;Acc:HGNC:22180] |
| ENSG00000221954 | HGLibA_33942 | 24 | -0.91760526 | OR4C12 | olfactory receptor family 4 subfamily C member 12 [Source:HGNC Symbol;Acc:HGNC:15168] |
| ENSG00000226430 | HGLibA_53284 | 24 | -0.91760526 | USP17L7 | ubiquitin specific peptidase 17-like family member 7 [Source:HGNC Symbol;Acc:HGNC:37180] |
| ENSG00000226742 | HGLibA_22155 | 24 | -0.91760526 | HSBP1L1 | heat shock factor binding protein 1 like 1 [Source:HGNC Symbol;Acc:HGNC:37243] |
| ENSG00000233932 | HGLibA_11757 | 24 | -0.91760526 | CTXN2 | cortexin 2 [Source:HGNC Symbol;Acc:HGNC:31109] |
| ENSG00000234829 | HGLibA_22672 | 24 | -0.91760526 | IFNA17 | interferon alpha 17 [Source:HGNC Symbol;Acc:HGNC:5422] |
| ENSG00000237136 | HGLibA_06345 | 24 | -0.91760526 | C4orf51 | chromosome 4 open reading frame 51 [Source:HGNC Symbol;Acc:HGNC:37264] |
| ENSG00000237330 | HGLibA_41646 | 24 | -0.91760526 | RNF223 | ring finger protein 223 [Source:HGNC Symbol;Acc:HGNC:40020] |
| ENSG00000240021 | HGLibA_49099 | 24 | -0.91760526 | TEX35 | testis expressed 35 [Source:HGNC Symbol;Acc:HGNC:25366] |
| ENSG00000240505 | HGLibA_50755 | 24 | -0.91760526 | TNFRSF13B | TNF receptor superfamily member 13B [Source:HGNC Symbol;Acc:HGNC:18153] |
| ENSG00000243509 | HGLibA_50788 | 24 | -0.91760526 | TNFRSF6B | TNF receptor superfamily member 6b [Source:HGNC Symbol;Acc:HGNC:11921] |
| ENSG00000248487 | HGLibA_00248 | 24 | -0.91760526 | ABHD14A | abhydrolase domain containing 14A [Source:HGNC Symbol;Acc:HGNC:24538] |
| ENSG00000250317 | HGLibA_45746 | 24 | -0.91760526 | SMIM20 | small integral membrane protein 20 [Source:HGNC Symbol;Acc:HGNC:37260] |
| ENSG00000255359 | HGLibA_07717 | 24 | -0.91760526 | CCDC179 | coiled-coil domain containing 179 [Source:HGNC Symbol;Acc:HGNC:44653] |
| ENSG00000271271 | HGLibA_52907 | 24 | -0.91760526 | UGT2A2 | UDP glucuronosyltransferase family 2 member A2 [Source:HGNC Symbol;Acc:HGNC:28183] |
| ENSG00000275004 | HGLibA_55727 | 24 | -0.91760526 | ZNF280B | zinc finger protein 280B [Source:HGNC Symbol;Acc:HGNC:23022] |
| ENSG00000275385 | HGLibA_07983 | 24 | -0.91760526 | CCL18 | C-C motif chemokine ligand 18 [Source:HGNC Symbol;Acc:HGNC:10616] |
| ENSG00000278057 | HGLibA_49088 | 24 | -0.91760526 | TEX28 | testis expressed 28 [Source:HGNC Symbol;Acc:HGNC:2563] |
| FAM122B | HGLibA_16068 | 24 | -0.91760526 | FAM122B | - |
| FOXD4L2 | HGLibA_17842 | 24 | -0.91760526 | FOXD4L2 | - |
| HIST1H2AE | HGLibA_21386 | 24 | -0.91760526 | HIST1H2AE | - |
| hsa-mir-1184-2 | HGLibA_57235 | 24 | -0.91760526 | hsa-mir-1184-2 | - |
| hsa-mir-1244-2 | HGLibA_57365 | 24 | -0.91760526 | hsa-mir-1244-2 | - |
| hsa-mir-1247 | HGLibA_57387 | 24 | -0.91760526 | hsa-mir-1247 | - |
| hsa-mir-1247 | HGLibA_57385 | 24 | -0.91760526 | hsa-mir-1247 | - |
| hsa-mir-1321 | HGLibA_57759 | 24 | -0.91760526 | hsa-mir-1321 | - |
| hsa-mir-15b | HGLibA_57941 | 24 | -0.91760526 | hsa-mir-15b | - |
| hsa-mir-181b-1 | HGLibA_57965 | 24 | -0.91760526 | hsa-mir-181b-1 | - |
| hsa-mir-21 | HGLibA_58210 | 24 | -0.91760526 | hsa-mir-21 | - |
| hsa-mir-224 | HGLibA_58304 | 24 | -0.91760526 | hsa-mir-224 | - |
| hsa-mir-3128 | HGLibA_58565 | 24 | -0.91760526 | hsa-mir-3128 | - |
| hsa-mir-3136 | HGLibA_58604 | 24 | -0.91760526 | hsa-mir-3136 | - |
| hsa-mir-3139 | HGLibA_58613 | 24 | -0.91760526 | hsa-mir-3139 | - |
| hsa-mir-3146 | HGLibA_58643 | 24 | -0.91760526 | hsa-mir-3146 | - |
| hsa-mir-3179-2 | HGLibA_58794 | 24 | -0.91760526 | hsa-mir-3179-2 | - |
| hsa-mir-338 | HGLibA_59005 | 24 | -0.91760526 | hsa-mir-338 | - |
| hsa-mir-3622b | HGLibA_59125 | 24 | -0.91760526 | hsa-mir-3622b | - |
| hsa-mir-3657 | HGLibA_59173 | 24 | -0.91760526 | hsa-mir-3657 | - |
| hsa-mir-376a-2 | HGLibA_59410 | 24 | -0.91760526 | hsa-mir-376a-2 | - |
| hsa-mir-4303 | HGLibA_59925 | 24 | -0.91760526 | hsa-mir-4303 | - |
| hsa-mir-4496 | HGLibA_60400 | 24 | -0.91760526 | hsa-mir-4496 | - |
| hsa-mir-4498 | HGLibA_60408 | 24 | -0.91760526 | hsa-mir-4498 | - |
| hsa-mir-4528 | HGLibA_60572 | 24 | -0.91760526 | hsa-mir-4528 | - |
| hsa-mir-4529 | HGLibA_60575 | 24 | -0.91760526 | hsa-mir-4529 | - |
| hsa-mir-4532 | HGLibA_60589 | 24 | -0.91760526 | hsa-mir-4532 | - |
| hsa-mir-4698 | HGLibA_60901 | 24 | -0.91760526 | hsa-mir-4698 | - |
| hsa-mir-5001 | HGLibA_61412 | 24 | -0.91760526 | hsa-mir-5001 | - |
| hsa-mir-5100 | HGLibA_61552 | 24 | -0.91760526 | hsa-mir-5100 | - |
| hsa-mir-548an | HGLibA_61905 | 24 | -0.91760526 | hsa-mir-548an | - |
| hsa-mir-548ar | HGLibA_61918 | 24 | -0.91760526 | hsa-mir-548ar | - |
| hsa-mir-593 | HGLibA_62469 | 24 | -0.91760526 | hsa-mir-593 | - |
| hsa-mir-6126 | HGLibA_62651 | 24 | -0.91760526 | hsa-mir-6126 | - |
| hsa-mir-6127 | HGLibA_62655 | 24 | -0.91760526 | hsa-mir-6127 | - |
| hsa-mir-6510 | HGLibA_62893 | 24 | -0.91760526 | hsa-mir-6510 | - |
| hsa-mir-6511a-3 | HGLibA_62904 | 24 | -0.91760526 | hsa-mir-6511a-3 | - |
| hsa-mir-663b | HGLibA_62990 | 24 | -0.91760526 | hsa-mir-663b | - |
| hsa-mir-6760 | HGLibA_63202 | 24 | -0.91760526 | hsa-mir-6760 | - |
| hsa-mir-6894 | HGLibA_63759 | 24 | -0.91760526 | hsa-mir-6894 | - |
| hsa-mir-96 | HGLibA_64370 | 24 | -0.91760526 | hsa-mir-96 | - |
| IKBKAP | HGLibA_22916 | 24 | -0.91760526 | IKBKAP | - |
| KIAA1239 | HGLibA_24723 | 24 | -0.91760526 | KIAA1239 | - |

| | | | | | |
|---|---|---|---|---|---|
| LOC100132247 | HGLibA_26806 | 24 | -0.91760526 | LOC100132247 | - |
| MAATS1 | HGLibA_27819 | 24 | -0.91760526 | MAATS1 | - |
| MPP6 | HGLibA_29763 | 24 | -0.91760526 | MPP6 | - |
| NUPL1 | HGLibA_33081 | 24 | -0.91760526 | NUPL1 | - |
| APOPT1 | HGLibA_02556 | 23 | -0.926650603 | APOPT1 | - |
| C15orf38-AP3S2 | HGLibA_05390 | 23 | -0.926650603 | C15orf38-AP3S2 | - |
| C4orf22 | HGLibA_06304 | 23 | -0.926650603 | C4orf22 | - |
| C6orf211 | HGLibA_06473 | 23 | -0.926650603 | C6orf211 | - |
| CD99_Y | HGLibA_08493 | 23 | -0.926650603 | CD99_Y | - |
| CXorf40B | HGLibA_11927 | 23 | -0.926650603 | CXorf40B | - |
| ENSG00000000003 | HGLibA_51942 | 23 | -0.926650603 | TSPAN6 | tetraspanin 6 [Source:HGNC Symbol;Acc:HGNC:11858] |
| ENSG00000011454 | HGLibA_40009 | 23 | -0.926650603 | RABGAP1 | RAB GTPase activating protein 1 [Source:HGNC Symbol;Acc:HGNC:17155] |
| ENSG00000021355 | HGLibA_43535 | 23 | -0.926650603 | SERPINB1 | serpin family B member 1 [Source:HGNC Symbol;Acc:HGNC:3311] |
| ENSG00000023287 | HGLibA_40415 | 23 | -0.926650603 | RB1CC1 | RB1 inducible coiled-coil 1 [Source:HGNC Symbol;Acc:HGNC:15574] |
| ENSG00000041515 | HGLibA_30809 | 23 | -0.926650603 | MYO16 | myosin XVI [Source:HGNC Symbol;Acc:HGNC:29822] |
| ENSG00000060138 | HGLibA_54650 | 23 | -0.926650603 | YBX3 | Y-box binding protein 3 [Source:HGNC Symbol;Acc:HGNC:2428] |
| ENSG00000072657 | HGLibA_51380 | 23 | -0.926650603 | TRHDE | thyrotropin releasing hormone degrading enzyme [Source:HGNC Symbol;Acc:HGNC:30748] |
| ENSG00000077063 | HGLibA_11743 | 23 | -0.926650603 | CTTNBP2 | cortactin binding protein 2 [Source:HGNC Symbol;Acc:HGNC:15679] |
| ENSG00000077063 | HGLibA_11742 | 23 | -0.926650603 | CTTNBP2 | cortactin binding protein 2 [Source:HGNC Symbol;Acc:HGNC:15679] |
| ENSG00000079246 | HGLibA_54595 | 23 | -0.926650603 | XRCC5 | X-ray repair cross complementing 5 [Source:HGNC Symbol;Acc:HGNC:12833] |
| ENSG00000080986 | HGLibA_31384 | 23 | -0.926650603 | NDC80 | kinetochore complex component [Source:HGNC Symbol;Acc:HGNC:16909]" |
| ENSG00000081026 | HGLibA_28001 | 23 | -0.926650603 | MAGI3 | WW and PDZ domain containing 3 [Source:HGNC Symbol;Acc:HGNC:29647]" |
| ENSG00000084463 | HGLibA_53972 | 23 | -0.926650603 | WBP11 | WW domain binding protein 11 [Source:HGNC Symbol;Acc:HGNC:16461] |
| ENSG00000087087 | HGLibA_47056 | 23 | -0.926650603 | SRRT | RNA effector molecule [Source:HGNC Symbol;Acc:HGNC:24101]" |
| ENSG00000089009 | HGLibA_41990 | 23 | -0.926650603 | RPL6 | ribosomal protein L6 [Source:HGNC Symbol;Acc:HGNC:10362] |
| ENSG00000092009 | HGLibA_10175 | 23 | -0.926650603 | CMA1 | chymase 1 [Source:HGNC Symbol;Acc:HGNC:2097] |
| ENSG00000093144 | HGLibA_14394 | 23 | -0.926650603 | ECHDC1 | ethylmalonyl-CoA decarboxylase 1 [Source:HGNC Symbol;Acc:HGNC:21489] |
| ENSG00000099337 | HGLibA_24330 | 23 | -0.926650603 | KCNK6 | potassium two pore domain channel subfamily K member 6 [Source:HGNC Symbol;Acc:HGNC:6281] |
| ENSG00000099338 | HGLibA_07374 | 23 | -0.926650603 | CATSPERG | cation channel sperm associated auxiliary subunit gamma [Source:HGNC Symbol;Acc:HGNC:25243] |
| ENSG00000099985 | HGLibA_34694 | 23 | -0.926650603 | OSM | oncostatin M [Source:HGNC Symbol;Acc:HGNC:8506] |
| ENSG00000100285 | HGLibA_31616 | 23 | -0.926650603 | NEFH | neurofilament heavy [Source:HGNC Symbol;Acc:HGNC:7737] |
| ENSG00000100422 | HGLibA_09194 | 23 | -0.926650603 | CERK | ceramide kinase [Source:HGNC Symbol;Acc:HGNC:19256] |
| ENSG00000100628 | HGLibA_03233 | 23 | -0.926650603 | ASB2 | ankyrin repeat and SOCS box containing 2 [Source:HGNC Symbol;Acc:HGNC:16012] |
| ENSG00000100767 | HGLibA_35132 | 23 | -0.926650603 | PAPLN | proteoglycan like sulfated glycoprotein [Source:HGNC Symbol;Acc:HGNC:19262]" |
| ENSG00000101150 | HGLibA_51116 | 23 | -0.926650603 | TPD52L2 | TPD52 like 2 [Source:HGNC Symbol;Acc:HGNC:12007] |
| ENSG00000101181 | HGLibA_30372 | 23 | -0.926650603 | MTG2 | mitochondrial ribosome associated GTPase 2 [Source:HGNC Symbol;Acc:HGNC:16239] |
| ENSG00000101346 | HGLibA_37468 | 23 | -0.926650603 | POFUT1 | protein O-fucosyltransferase 1 [Source:HGNC Symbol;Acc:HGNC:14988] |
| ENSG00000101935 | HGLibA_01803 | 23 | -0.926650603 | AMMECR1 | midface hypoplasia and elliptocytosis chromosomal region gene 1 [Source:HGNC Symbol;Acc:HGNC:467]" |
| ENSG00000103021 | HGLibA_07548 | 23 | -0.926650603 | CCDC113 | coiled-coil domain containing 113 [Source:HGNC Symbol;Acc:HGNC:25002] |
| ENSG00000105323 | HGLibA_21850 | 23 | -0.926650603 | HNRNPUL1 | heterogeneous nuclear ribonucleoprotein U like 1 [Source:HGNC Symbol;Acc:HGNC:17011] |
| ENSG00000105722 | HGLibA_15460 | 23 | -0.926650603 | ERF | ETS2 repressor factor [Source:HGNC Symbol;Acc:HGNC:3444] |
| ENSG00000105866 | HGLibA_46302 | 23 | -0.926650603 | SP4 | Sp4 transcription factor [Source:HGNC Symbol;Acc:HGNC:11209] |
| ENSG00000105929 | HGLibA_03710 | 23 | -0.926650603 | ATP6V0A4 | ATPase H+ transporting V0 subunit a4 [Source:HGNC Symbol;Acc:HGNC:866] |
| ENSG00000106789 | HGLibA_10783 | 23 | -0.926650603 | CORO2A | coronin 2A [Source:HGNC Symbol;Acc:HGNC:2255] |
| ENSG00000108381 | HGLibA_03341 | 23 | -0.926650603 | ASPA | aspartoacylase [Source:HGNC Symbol;Acc:HGNC:756] |
| ENSG00000108448 | HGLibA_51420 | 23 | -0.926650603 | TRIM16L | tripartite motif containing 16 like [Source:HGNC Symbol;Acc:HGNC:32670] |
| ENSG00000108797 | HGLibA_10418 | 23 | -0.926650603 | CNTNAP1 | contactin associated protein 1 [Source:HGNC Symbol;Acc:HGNC:8011] |
| ENSG00000109255 | HGLibA_32211 | 23 | -0.926650603 | NMU | neuromedin U [Source:HGNC Symbol;Acc:HGNC:7859] |
| ENSG00000110697 | HGLibA_36751 | 23 | -0.926650603 | PITPNM1 | phosphatidylinositol transfer protein membrane associated 1 [Source:HGNC Symbol;Acc:HGNC:9003] |
| ENSG00000110756 | HGLibA_22055 | 23 | -0.926650603 | HPS5 | biogenesis of lysosomal organelles complex 2 subunit 2 [Source:HGNC Symbol;Acc:HGNC:17022]" |
| ENSG00000110777 | HGLibA_37752 | 23 | -0.926650603 | POU2AF1 | POU class 2 associating factor 1 [Source:HGNC Symbol;Acc:HGNC:9211] |
| ENSG00000110921 | HGLibA_30599 | 23 | -0.926650603 | MVK | mevalonate kinase [Source:HGNC Symbol;Acc:HGNC:7530] |
| ENSG00000110975 | HGLibA_48048 | 23 | -0.926650603 | SYT10 | synaptotagmin 10 [Source:HGNC Symbol;Acc:HGNC:19266] |
| ENSG00000111371 | HGLibA_45056 | 23 | -0.926650603 | SLC38A1 | solute carrier family 38 member 1 [Source:HGNC Symbol;Acc:HGNC:13447] |
| ENSG00000111786 | HGLibA_47093 | 23 | -0.926650603 | SRSF9 | serine and arginine rich splicing factor 9 [Source:HGNC Symbol;Acc:HGNC:10791] |
| ENSG00000112984 | HGLibA_24891 | 23 | -0.926650603 | KIF20A | kinesin family member 20A [Source:HGNC Symbol;Acc:HGNC:9787] |
| ENSG00000113522 | HGLibA_40076 | 23 | -0.926650603 | RAD50 | RAD50 double strand break repair protein [Source:HGNC Symbol;Acc:HGNC:9816] |
| ENSG00000113946 | HGLibA_09866 | 23 | -0.926650603 | CLDN16 | claudin 16 [Source:HGNC Symbol;Acc:HGNC:2037] |
| ENSG00000117139 | HGLibA_24524 | 23 | -0.926650603 | KDM5B | lysine demethylase 5B [Source:HGNC Symbol;Acc:HGNC:18039] |
| ENSG00000117707 | HGLibA_38702 | 23 | -0.926650603 | PROX1 | prospero homeobox 1 [Source:HGNC Symbol;Acc:HGNC:9459] |
| ENSG00000118137 | HGLibA_02449 | 23 | -0.926650603 | APOA1 | apolipoprotein A1 [Source:HGNC Symbol;Acc:HGNC:600] |
| ENSG00000118402 | HGLibA_14966 | 23 | -0.926650603 | ELOVL4 | ELOVL fatty acid elongase 4 [Source:HGNC Symbol;Acc:HGNC:14415] |
| ENSG00000119397 | HGLibA_10438 | 23 | -0.926650603 | CNTRL | centriolin [Source:HGNC Symbol;Acc:HGNC:1858] |
| ENSG00000119471 | HGLibA_22216 | 23 | -0.926650603 | HSDL2 | hydroxysteroid dehydrogenase like 2 [Source:HGNC Symbol;Acc:HGNC:18572] |
| ENSG00000120254 | HGLibA_30380 | 23 | -0.926650603 | MTHFD1L | methylenetetrahydrofolate dehydrogenase (NADP+ dependent) 1 like [Source:HGNC Symbol;Acc:HGNC:21055] |
| ENSG00000121858 | HGLibA_50795 | 23 | -0.926650603 | TNFSF10 | TNF superfamily member 10 [Source:HGNC Symbol;Acc:HGNC:11925] |
| ENSG00000121892 | HGLibA_35942 | 23 | -0.926650603 | PDS5A | PDS5 cohesin associated factor A [Source:HGNC Symbol;Acc:HGNC:29088] |
| ENSG00000122912 | HGLibA_44655 | 23 | -0.926650603 | SLC25A16 | solute carrier family 25 member 16 [Source:HGNC Symbol;Acc:HGNC:10986] |
| ENSG00000123200 | HGLibA_54961 | 23 | -0.926650603 | ZC3H13 | zinc finger CCCH-type containing 13 [Source:HGNC Symbol;Acc:HGNC:20368] |
| ENSG00000124098 | HGLibA_16455 | 23 | -0.926650603 | FAM210B | family with sequence similarity 210 member B [Source:HGNC Symbol;Acc:HGNC:16102] |
| ENSG00000125207 | HGLibA_36770 | 23 | -0.926650603 | PIWIL1 | piwi like RNA-mediated gene silencing 1 [Source:HGNC Symbol;Acc:HGNC:9007] |
| ENSG00000125968 | HGLibA_22523 | 23 | -0.926650603 | ID1 | HLH protein [Source:HGNC Symbol;Acc:HGNC:5360]" |
| ENSG00000126266 | HGLibA_17325 | 23 | -0.926650603 | FFAR1 | free fatty acid receptor 1 [Source:HGNC Symbol;Acc:HGNC:4498] |
| ENSG00000126461 | HGLibA_42822 | 23 | -0.926650603 | SCAF1 | SR-related CTD associated factor 1 [Source:HGNC Symbol;Acc:HGNC:30403] |
| ENSG00000126746 | HGLibA_55894 | 23 | -0.926650603 | ZNF384 | zinc finger protein 384 [Source:HGNC Symbol;Acc:HGNC:11955] |
| ENSG00000127540 | HGLibA_53106 | 23 | -0.926650603 | UQCR11 | complex III subunit XI [Source:HGNC Symbol;Acc:HGNC:30862]" |
| ENSG00000127990 | HGLibA_43804 | 23 | -0.926650603 | SGCE | sarcoglycan epsilon [Source:HGNC Symbol;Acc:HGNC:10808] |
| ENSG00000130159 | HGLibA_14421 | 23 | -0.926650603 | ECSIT | ECSIT signalling integrator [Source:HGNC Symbol;Acc:HGNC:29548] |
| ENSG00000130204 | HGLibA_50966 | 23 | -0.926650603 | TOMM40 | translocase of outer mitochondrial membrane 40 [Source:HGNC Symbol;Acc:HGNC:18001] |
| ENSG00000130783 | HGLibA_07820 | 23 | -0.926650603 | CCDC62 | coiled-coil domain containing 62 [Source:HGNC Symbol;Acc:HGNC:30723] |
| ENSG00000131018 | HGLibA_47977 | 23 | -0.926650603 | SYNE1 | spectrin repeat containing nuclear envelope protein 1 [Source:HGNC Symbol;Acc:HGNC:17089] |
| ENSG00000131143 | HGLibA_10831 | 23 | -0.926650603 | COX4I1 | cytochrome c oxidase subunit 4I1 [Source:HGNC Symbol;Acc:HGNC:2265] |
| ENSG00000131165 | HGLibA_09472 | 23 | -0.926650603 | CHMP1A | charged multivesicular body protein 1A [Source:HGNC Symbol;Acc:HGNC:8740] |
| ENSG00000131686 | HGLibA_06818 | 23 | -0.926650603 | CA6 | carbonic anhydrase 6 [Source:HGNC Symbol;Acc:HGNC:1380] |
| ENSG00000132549 | HGLibA_53735 | 23 | -0.926650603 | VPS13B | vacuolar protein sorting 13 homolog B [Source:HGNC Symbol;Acc:HGNC:2183] |
| ENSG00000132670 | HGLibA_39484 | 23 | -0.926650603 | PTPRA | receptor type A [Source:HGNC Symbol;Acc:HGNC:9664]" |
| ENSG00000133121 | HGLibA_47354 | 23 | -0.926650603 | STARD13 | StAR related lipid transfer domain containing 13 [Source:HGNC Symbol;Acc:HGNC:19164] |
| ENSG00000133488 | HGLibA_43200 | 23 | -0.926650603 | SEC14L4 | SEC14 like lipid binding 4 [Source:HGNC Symbol;Acc:HGNC:20627] |
| ENSG00000133742 | HGLibA_06786 | 23 | -0.926650603 | CA1 | carbonic anhydrase 1 [Source:HGNC Symbol;Acc:HGNC:1368] |
| ENSG00000134146 | HGLibA_13851 | 23 | -0.926650603 | DPH6 | diphthamine biosynthesis 6 [Source:HGNC Symbol;Acc:HGNC:30543] |
| ENSG00000134193 | HGLibA_40806 | 23 | -0.926650603 | REG4 | regenerating family member 4 [Source:HGNC Symbol;Acc:HGNC:22977] |
| ENSG00000134255 | HGLibA_09184 | 23 | -0.926650603 | CEPT1 | choline/ethanolamine phosphotransferase 1 [Source:HGNC Symbol;Acc:HGNC:24289] |
| ENSG00000134323 | HGLibA_30678 | 23 | -0.926650603 | MYCN | bHLH transcription factor [Source:HGNC Symbol;Acc:HGNC:7559]" |
| ENSG00000134709 | HGLibA_21875 | 23 | -0.926650603 | HOOK1 | hook microtubule tethering protein 1 [Source:HGNC Symbol;Acc:HGNC:19884] |
| ENSG00000134760 | HGLibA_14023 | 23 | -0.926650603 | DSG1 | desmoglein 1 [Source:HGNC Symbol;Acc:HGNC:3048] |
| ENSG00000134982 | HGLibA_02390 | 23 | -0.926650603 | APC | WNT signaling pathway regulator [Source:HGNC Symbol;Acc:HGNC:583]" |
| ENSG00000135750 | HGLibA_24293 | 23 | -0.926650603 | KCNK1 | potassium two pore domain channel subfamily K member 1 [Source:HGNC Symbol;Acc:HGNC:6272] |
| ENSG00000135763 | HGLibA_53138 | 23 | -0.926650603 | URB2 | URB2 ribosome biogenesis homolog [Source:HGNC Symbol;Acc:HGNC:28967] |
| ENSG00000136688 | HGLibA_23151 | 23 | -0.926650603 | IL36G | interleukin 36 gamma [Source:HGNC Symbol;Acc:HGNC:15741] |
| ENSG00000136895 | HGLibA_18631 | 23 | -0.926650603 | GARNL3 | GTPase activating Rap/RanGAP domain like 3 [Source:HGNC Symbol;Acc:HGNC:25425] |
| ENSG00000136918 | HGLibA_54082 | 23 | -0.926650603 | WDR38 | WD repeat domain 38 [Source:HGNC Symbol;Acc:HGNC:23745] |
| ENSG00000137267 | HGLibA_52275 | 23 | -0.926650603 | TUBB2A | tubulin beta 2A class IIa [Source:HGNC Symbol;Acc:HGNC:12412] |
| ENSG00000137275 | HGLibA_41317 | 23 | -0.926650603 | RIPK1 | receptor interacting serine/threonine kinase 1 [Source:HGNC Symbol;Acc:HGNC:10019] |
| ENSG00000137752 | HGLibA_07296 | 23 | -0.926650603 | CASP1 | caspase 1 [Source:HGNC Symbol;Acc:HGNC:1499] |
| ENSG00000137770 | HGLibA_11618 | 23 | -0.926650603 | CTDSPL2 | CTD small phosphatase like 2 [Source:HGNC Symbol;Acc:HGNC:26936] |
| ENSG00000138083 | HGLibA_44248 | 23 | -0.926650603 | SIX3 | SIX homeobox 3 [Source:HGNC Symbol;Acc:HGNC:10889] |
| ENSG00000138744 | HGLibA_31007 | 23 | -0.926650603 | NAAA | N-acylethanolamine acid amidase [Source:HGNC Symbol;Acc:HGNC:736] |
| ENSG00000140403 | HGLibA_13520 | 23 | -0.926650603 | DNAJA4 | DnaJ heat shock protein family (Hsp40) member A4 [Source:HGNC Symbol;Acc:HGNC:14885] |
| ENSG00000140465 | HGLibA_12068 | 23 | -0.926650603 | CYP1A1 | cytochrome P450 family 1 subfamily A member 1 [Source:HGNC Symbol;Acc:HGNC:2595] |
| ENSG00000140600 | HGLibA_43951 | 23 | -0.926650603 | SH3GL3 | endophilin A3 [Source:HGNC Symbol;Acc:HGNC:10832]" |
| ENSG00000141971 | HGLibA_30588 | 23 | -0.926650603 | MVB12A | multivesicular body subunit 12A [Source:HGNC Symbol;Acc:HGNC:25153] |
| ENSG00000142065 | HGLibA_55188 | 23 | -0.926650603 | ZFP14 | ZFP14 zinc finger protein [Source:HGNC Symbol;Acc:HGNC:29312] |
| ENSG00000142347 | HGLibA_30834 | 23 | -0.926650603 | MYO1F | myosin IF [Source:HGNC Symbol;Acc:HGNC:7600] |
| ENSG00000142615 | HGLibA_08993 | 23 | -0.926650603 | CELA2A | chymotrypsin like elastase family member 2A [Source:HGNC Symbol;Acc:HGNC:24609] |
| ENSG00000145632 | HGLibA_37191 | 23 | -0.926650603 | PLK2 | polo like kinase 2 [Source:HGNC Symbol;Acc:HGNC:19699] |
| ENSG00000145757 | HGLibA_46533 | 23 | -0.926650603 | SPATA9 | spermatogenesis associated 9 [Source:HGNC Symbol;Acc:HGNC:22988] |
| ENSG00000145819 | HGLibA_02740 | 23 | -0.926650603 | ARHGAP26 | Rho GTPase activating protein 26 [Source:HGNC Symbol;Acc:HGNC:17073] |
| ENSG00000145949 | HGLibA_30794 | 23 | -0.926650603 | MYLK4 | myosin light chain kinase family member 4 [Source:HGNC Symbol;Acc:HGNC:27972] |
| ENSG00000145982 | HGLibA_16879 | 23 | -0.926650603 | FARS2 | mitochondrial [Source:HGNC Symbol;Acc:HGNC:21062]" |
| ENSG00000146147 | HGLibA_29387 | 23 | -0.926650603 | MLIP | muscular LMNA interacting protein [Source:HGNC Symbol;Acc:HGNC:21355] |
| ENSG00000147133 | HGLibA_48194 | 23 | -0.926650603 | TAF1 | TATA-box binding protein associated factor 1 [Source:HGNC Symbol;Acc:HGNC:11535] |
| ENSG00000149922 | HGLibA_48678 | 23 | -0.926650603 | TBX6 | T-box 6 [Source:HGNC Symbol;Acc:HGNC:11605] |
| ENSG00000150773 | HGLibA_36612 | 23 | -0.926650603 | PIH1D2 | PIH1 domain containing 2 [Source:HGNC Symbol;Acc:HGNC:25210] |
| ENSG00000151422 | HGLibA_17285 | 23 | -0.926650603 | FER | FER tyrosine kinase [Source:HGNC Symbol;Acc:HGNC:3655] |
| ENSG00000152219 | HGLibA_02939 | 23 | -0.926650603 | ARL14EP | ADP ribosylation factor like GTPase 14 effector protein [Source:HGNC Symbol;Acc:HGNC:26798] |
| ENSG00000152254 | HGLibA_18308 | 23 | -0.926650603 | G6PC2 | glucose-6-phosphatase catalytic subunit 2 [Source:HGNC Symbol;Acc:HGNC:28906] |
| ENSG00000154122 | HGLibA_01960 | 23 | -0.926650603 | ANKH | ANKH inorganic pyrophosphate transport regulator [Source:HGNC Symbol;Acc:HGNC:15492] |
| ENSG00000154645 | HGLibA_09510 | 23 | -0.926650603 | CHODL | chondrolectin [Source:HGNC Symbol;Acc:HGNC:17807] |
| ENSG00000154719 | HGLibA_29973 | 23 | -0.926650603 | MRPL39 | mitochondrial ribosomal protein L39 [Source:HGNC Symbol;Acc:HGNC:14027] |
| ENSG00000155957 | HGLibA_49743 | 23 | -0.926650603 | TMBIM4 | transmembrane BAX inhibitor motif containing 4 [Source:HGNC Symbol;Acc:HGNC:24257] |
| ENSG00000156194 | HGLibA_37870 | 23 | -0.926650603 | PPEF2 | protein phosphatase with EF-hand domain 2 [Source:HGNC Symbol;Acc:HGNC:9244] |
| ENSG00000156925 | HGLibA_55328 | 23 | -0.926650603 | ZIC3 | Zic family member 3 [Source:HGNC Symbol;Acc:HGNC:12874] |
| ENSG00000157193 | HGLibA_27234 | 23 | -0.926650603 | LRP8 | LDL receptor related protein 8 [Source:HGNC Symbol;Acc:HGNC:6700] |
| ENSG00000158106 | HGLibA_41224 | 23 | -0.926650603 | RHPN1 | rhophilin Rho GTPase binding protein 1 [Source:HGNC Symbol;Acc:HGNC:19973] |
| ENSG00000158292 | HGLibA_19940 | 23 | -0.926650603 | GPR153 | G protein-coupled receptor 153 [Source:HGNC Symbol;Acc:HGNC:23618] |

| | | | | | |
|---|---|---|---|---|---|
| ENSG00000159214 | HGLibA_07735 | 23 | -0.926650603 | CCDC24 | coiled-coil domain containing 24 [Source:HGNC Symbol;Acc:HGNC:28688] |
| ENSG00000160870 | HGLibA_12162 | 23 | -0.926650603 | CYP3A7 | cytochrome P450 family 3 subfamily A member 7 [Source:HGNC Symbol;Acc:HGNC:2640] |
| ENSG00000161179 | HGLibA_54651 | 23 | -0.926650603 | YDJC | YdjC chitooligosaccharide deacetylase homolog [Source:HGNC Symbol;Acc:HGNC:27158] |
| ENSG00000161955 | HGLibA_50809 | 23 | -0.926650603 | TNFSF13 | TNF superfamily member 13 [Source:HGNC Symbol;Acc:HGNC:11928] |
| ENSG00000162687 | HGLibA_24396 | 23 | -0.926650603 | KCNT2 | potassium sodium-activated channel subfamily T member 2 [Source:HGNC Symbol;Acc:HGNC:18866] |
| ENSG00000162772 | HGLibA_03437 | 23 | -0.926650603 | ATF3 | activating transcription factor 3 [Source:HGNC Symbol;Acc:HGNC:785] |
| ENSG00000162896 | HGLibA_36582 | 23 | -0.926650603 | PIGR | polymeric immunoglobulin receptor [Source:HGNC Symbol;Acc:HGNC:8968] |
| ENSG00000163098 | HGLibA_04530 | 23 | -0.926650603 | BIRC8 | baculoviral IAP repeat containing 8 [Source:HGNC Symbol;Acc:HGNC:14878] |
| ENSG00000163347 | HGLibA_09847 | 23 | -0.926650603 | CLDN1 | claudin 1 [Source:HGNC Symbol;Acc:HGNC:2032] |
| ENSG00000163607 | HGLibA_20621 | 23 | -0.926650603 | GTPBP8 | GTP binding protein 8 (putative) [Source:HGNC Symbol;Acc:HGNC:25007] |
| ENSG00000163875 | HGLibA_28722 | 23 | -0.926650603 | MEAF6 | MYST/Esa1 associated factor 6 [Source:HGNC Symbol;Acc:HGNC:25674] |
| ENSG00000164128 | HGLibA_32549 | 23 | -0.926650603 | NPY1R | neuropeptide Y receptor Y1 [Source:HGNC Symbol;Acc:HGNC:7956] |
| ENSG00000165983 | HGLibA_39298 | 23 | -0.926650603 | PTER | phosphotriesterase related [Source:HGNC Symbol;Acc:HGNC:9590] |
| ENSG00000166343 | HGLibA_30249 | 23 | -0.926650603 | MSS51 | MSS51 mitochondrial translational activator [Source:HGNC Symbol;Acc:HGNC:21000] |
| ENSG00000166747 | HGLibA_02285 | 23 | -0.926650603 | AP1G1 | adaptor related protein complex 1 subunit gamma 1 [Source:HGNC Symbol;Acc:HGNC:555] |
| ENSG00000167325 | HGLibA_42252 | 23 | -0.926650603 | RRM1 | ribonucleotide reductase catalytic subunit M1 [Source:HGNC Symbol;Acc:HGNC:10451] |
| ENSG00000167515 | HGLibA_51316 | 23 | -0.926650603 | TRAPPC2L | trafficking protein particle complex 2 like [Source:HGNC Symbol;Acc:HGNC:30887] |
| ENSG00000167536 | HGLibA_13128 | 23 | -0.926650603 | DHRS13 | dehydrogenase/reductase 13 [Source:HGNC Symbol;Acc:HGNC:28326] |
| ENSG00000167716 | HGLibA_54187 | 23 | -0.926650603 | WDR81 | WD repeat domain 81 [Source:HGNC Symbol;Acc:HGNC:26600] |
| ENSG00000167825 | HGLibA_34303 | 23 | -0.926650603 | OR5I1 | olfactory receptor family 5 subfamily I member 1 [Source:HGNC Symbol;Acc:HGNC:8347] |
| ENSG00000168356 | HGLibA_42972 | 23 | -0.926650603 | SCN11A | sodium voltage-gated channel alpha subunit 11 [Source:HGNC Symbol;Acc:HGNC:10583] |
| ENSG00000168803 | HGLibA_00726 | 23 | -0.926650603 | ADAL | adenosine deaminase like [Source:HGNC Symbol;Acc:HGNC:31853] |
| ENSG00000169564 | HGLibA_35368 | 23 | -0.926650603 | PCBP1 | poly(rC) binding protein 1 [Source:HGNC Symbol;Acc:HGNC:8647] |
| ENSG00000169627 | HGLibA_04687 | 23 | -0.926650603 | BOLA2B | bolA family member 2B [Source:HGNC Symbol;Acc:HGNC:32479] |
| ENSG00000170581 | HGLibA_47384 | 23 | -0.926650603 | STAT2 | signal transducer and activator of transcription 2 [Source:HGNC Symbol;Acc:HGNC:11363] |
| ENSG00000171055 | HGLibA_17315 | 23 | -0.926650603 | FEZ2 | fasciculation and elongation protein zeta 2 [Source:HGNC Symbol;Acc:HGNC:3660] |
| ENSG00000171478 | HGLibA_46337 | 23 | -0.926650603 | SPACA5B | sperm acrosome associated 5B [Source:HGNC Symbol;Acc:HGNC:19142] |
| ENSG00000171763 | HGLibA_46519 | 23 | -0.926650603 | SPATA5L1 | spermatogenesis associated 5 like 1 [Source:HGNC Symbol;Acc:HGNC:28762] |
| ENSG00000171766 | HGLibA_18696 | 23 | -0.926650603 | GATM | glycine amidinotransferase [Source:HGNC Symbol;Acc:HGNC:4175] |
| ENSG00000172377 | HGLibA_34578 | 23 | -0.926650603 | OR9I1 | olfactory receptor family 9 subfamily I member 1 [Source:HGNC Symbol;Acc:HGNC:14718] |
| ENSG00000172831 | HGLibA_09222 | 23 | -0.926650603 | CES2 | carboxylesterase 2 [Source:HGNC Symbol;Acc:HGNC:1864] |
| ENSG00000173714 | HGLibA_54282 | 23 | -0.926650603 | WFIKKN2 | kunitz and netrin domain containing 2 [Source:HGNC Symbol;Acc:HGNC:30916]" |
| ENSG00000174233 | HGLibA_00922 | 23 | -0.926650603 | ADCY6 | adenylate cyclase 6 [Source:HGNC Symbol;Acc:HGNC:237] |
| ENSG00000174348 | HGLibA_37453 | 23 | -0.926650603 | PODN | podocan [Source:HGNC Symbol;Acc:HGNC:23174] |
| ENSG00000174373 | HGLibA_40156 | 23 | -0.926650603 | RALGAPA1 | Ral GTPase activating protein catalytic alpha subunit 1 [Source:HGNC Symbol;Acc:HGNC:17770] |
| ENSG00000174483 | HGLibA_04207 | 23 | -0.926650603 | BBS1 | Bardet-Biedl syndrome 1 [Source:HGNC Symbol;Acc:HGNC:966] |
| ENSG00000175274 | HGLibA_51059 | 23 | -0.926650603 | TP53I11 | tumor protein p53 inducible protein 11 [Source:HGNC Symbol;Acc:HGNC:16842] |
| ENSG00000175893 | HGLibA_55101 | 23 | -0.926650603 | ZDHHC21 | zinc finger DHHC-type containing 21 [Source:HGNC Symbol;Acc:HGNC:20750] |
| ENSG00000177425 | HGLibA_35292 | 23 | -0.926650603 | PAWR | pro-apoptotic WT1 regulator [Source:HGNC Symbol;Acc:HGNC:8614] |
| ENSG00000178279 | HGLibA_50896 | 23 | -0.926650603 | TNP2 | transition protein 2 [Source:HGNC Symbol;Acc:HGNC:11952] |
| ENSG00000178796 | HGLibA_41246 | 23 | -0.926650603 | RIIAD1 | regulatory subunit of type II PKA R-subunit (RIIa) domain containing 1 [Source:HGNC Symbol;Acc:HGNC:26686] |
| ENSG00000179059 | HGLibA_55220 | 23 | -0.926650603 | ZFP42 | ZFP42 zinc finger protein [Source:HGNC Symbol;Acc:HGNC:30949] |
| ENSG00000179695 | HGLibA_34385 | 23 | -0.926650603 | OR6C2 | olfactory receptor family 6 subfamily C member 2 [Source:HGNC Symbol;Acc:HGNC:15436] |
| ENSG00000180957 | HGLibA_36743 | 23 | -0.926650603 | PITPNB | phosphatidylinositol transfer protein beta [Source:HGNC Symbol;Acc:HGNC:9002] |
| ENSG00000182952 | HGLibA_21726 | 23 | -0.926650603 | HMGN4 | high mobility group nucleosomal binding domain 4 [Source:HGNC Symbol;Acc:HGNC:4989] |
| ENSG00000183396 | HGLibA_50544 | 23 | -0.926650603 | TMEM89 | transmembrane protein 89 [Source:HGNC Symbol;Acc:HGNC:32372] |
| ENSG00000183479 | HGLibA_51376 | 23 | -0.926650603 | TREX2 | three prime repair exonuclease 2 [Source:HGNC Symbol;Acc:HGNC:12270] |
| ENSG00000186113 | HGLibA_34278 | 23 | -0.926650603 | OR5D14 | olfactory receptor family 5 subfamily D member 14 [Source:HGNC Symbol;Acc:HGNC:15281] |
| ENSG00000186230 | HGLibA_56671 | 23 | -0.926650603 | ZNF749 | zinc finger protein 749 [Source:HGNC Symbol;Acc:HGNC:32783] |
| ENSG00000186314 | HGLibA_38432 | 23 | -0.926650603 | PRELID2 | PRELI domain containing 2 [Source:HGNC Symbol;Acc:HGNC:28306] |
| ENSG00000186409 | HGLibA_07753 | 23 | -0.926650603 | CCDC30 | coiled-coil domain containing 30 [Source:HGNC Symbol;Acc:HGNC:26103] |
| ENSG00000187017 | HGLibA_15574 | 23 | -0.926650603 | ESPN | espin [Source:HGNC Symbol;Acc:HGNC:13281] |
| ENSG00000187123 | HGLibA_27727 | 23 | -0.926650603 | LYPD6 | LY6/PLAUR domain containing 6 [Source:HGNC Symbol;Acc:HGNC:28751] |
| ENSG00000187726 | HGLibA_13532 | 23 | -0.926650603 | DNAJB13 | DnaJ heat shock protein family (Hsp40) member B13 [Source:HGNC Symbol;Acc:HGNC:30718] |
| ENSG00000188000 | HGLibA_34472 | 23 | -0.926650603 | OR7D2 | olfactory receptor family 7 subfamily D member 2 [Source:HGNC Symbol;Acc:HGNC:8378] |
| ENSG00000188175 | HGLibA_21142 | 23 | -0.926650603 | HEPACAM2 | HEPACAM family member 2 [Source:HGNC Symbol;Acc:HGNC:27364] |
| ENSG00000188992 | HGLibA_26618 | 23 | -0.926650603 | LIPI | lipase I [Source:HGNC Symbol;Acc:HGNC:18821] |
| ENSG00000189042 | HGLibA_56262 | 23 | -0.926650603 | ZNF567 | zinc finger protein 567 [Source:HGNC Symbol;Acc:HGNC:28696] |
| ENSG00000196418 | HGLibA_55457 | 23 | -0.926650603 | ZNF124 | zinc finger protein 124 [Source:HGNC Symbol;Acc:HGNC:12907] |
| ENSG00000196437 | HGLibA_56268 | 23 | -0.926650603 | ZNF569 | zinc finger protein 569 [Source:HGNC Symbol;Acc:HGNC:24737] |
| ENSG00000196503 | HGLibA_03002 | 23 | -0.926650603 | ARL9 | ADP ribosylation factor like GTPase 9 [Source:HGNC Symbol;Acc:HGNC:23592] |
| ENSG00000197077 | HGLibA_24799 | 23 | -0.926650603 | KIAA1671 | KIAA1671 [Source:HGNC Symbol;Acc:HGNC:29345] |
| ENSG00000197410 | HGLibA_12493 | 23 | -0.926650603 | DCHS2 | dachsous cadherin-related 2 [Source:HGNC Symbol;Acc:HGNC:23111] |
| ENSG00000197566 | HGLibA_56415 | 23 | -0.926650603 | ZNF624 | zinc finger protein 624 [Source:HGNC Symbol;Acc:HGNC:29254] |
| ENSG00000198265 | HGLibA_21124 | 23 | -0.926650603 | HELZ | helicase with zinc finger [Source:HGNC Symbol;Acc:HGNC:16878] |
| ENSG00000198633 | HGLibA_56181 | 23 | -0.926650603 | ZNF534 | zinc finger protein 534 [Source:HGNC Symbol;Acc:HGNC:26337] |
| ENSG00000198890 | HGLibA_38632 | 23 | -0.926650603 | PRMT6 | protein arginine methyltransferase 6 [Source:HGNC Symbol;Acc:HGNC:18241] |
| ENSG00000203972 | HGLibA_19378 | 23 | -0.926650603 | GLYATL3 | glycine-N-acyltransferase like 3 [Source:HGNC Symbol;Acc:HGNC:21349] |
| ENSG00000204231 | HGLibA_42510 | 23 | -0.926650603 | RXRB | retinoid X receptor beta [Source:HGNC Symbol;Acc:HGNC:10478] |
| ENSG00000205155 | HGLibA_39030 | 23 | -0.926650603 | PSENEN | gamma-secretase subunit [Source:HGNC Symbol;Acc:HGNC:30100]" |
| ENSG00000206384 | HGLibA_10608 | 23 | -0.926650603 | COL6A6 | collagen type VI alpha 6 chain [Source:HGNC Symbol;Acc:HGNC:27023] |
| ENSG00000214087 | HGLibA_02949 | 23 | -0.926650603 | ARL16 | ADP ribosylation factor like GTPase 16 [Source:HGNC Symbol;Acc:HGNC:27902] |
| ENSG00000219200 | HGLibA_41435 | 23 | -0.926650603 | RNASEK | ribonuclease K [Source:HGNC Symbol;Acc:HGNC:33911] |
| ENSG00000248712 | HGLibA_07651 | 23 | -0.926650603 | CCDC153 | coiled-coil domain containing 153 [Source:HGNC Symbol;Acc:HGNC:27446] |
| ENSG00000250571 | HGLibA_19255 | 23 | -0.926650603 | GLI4 | GLI family zinc finger 4 [Source:HGNC Symbol;Acc:HGNC:4320] |
| ENSG00000256618 | HGLibA_30471 | 23 | -0.926650603 | MTRNR2L1 | MT-RNR2 like 1 [Source:HGNC Symbol;Acc:HGNC:37155] |
| ENSG00000259956 | HGLibA_40494 | 23 | -0.926650603 | RBM15B | RNA binding motif protein 15B [Source:HGNC Symbol;Acc:HGNC:24303] |
| ENSG00000260325 | HGLibA_22322 | 23 | -0.926650603 | HSPB9 | heat shock protein family B (small) member 9 [Source:HGNC Symbol;Acc:HGNC:30589] |
| ENSG00000278505 | HGLibA_05579 | 23 | -0.926650603 | C17orf78 | chromosome 17 open reading frame 78 [Source:HGNC Symbol;Acc:HGNC:26831] |
| GAGE5 | HGLibA_18482 | 23 | -0.926650603 | GAGE5 | - |
| H2AFY2 | HGLibA_20767 | 23 | -0.926650603 | H2AFY2 | - |
| hsa-mir-1184-3 | HGLibA_57236 | 23 | -0.926650603 | hsa-mir-1184-3 | - |
| hsa-mir-1273d | HGLibA_57540 | 23 | -0.926650603 | hsa-mir-1273d | - |
| hsa-mir-135a-2 | HGLibA_57798 | 23 | -0.926650603 | hsa-mir-135a-2 | - |
| hsa-mir-2278 | HGLibA_58318 | 23 | -0.926650603 | hsa-mir-2278 | - |
| hsa-mir-3074 | HGLibA_58467 | 23 | -0.926650603 | hsa-mir-3074 | - |
| hsa-mir-3199-1 | HGLibA_58899 | 23 | -0.926650603 | hsa-mir-3199-1 | - |
| hsa-mir-362 | HGLibA_59111 | 23 | -0.926650603 | hsa-mir-362 | - |
| hsa-mir-3685 | HGLibA_59293 | 23 | -0.926650603 | hsa-mir-3685 | - |
| hsa-mir-3689f | HGLibA_59338 | 23 | -0.926650603 | hsa-mir-3689f | - |
| hsa-mir-4279 | HGLibA_59819 | 23 | -0.926650603 | hsa-mir-4279 | - |
| hsa-mir-4288 | HGLibA_59859 | 23 | -0.926650603 | hsa-mir-4288 | - |
| hsa-mir-4311 | HGLibA_59960 | 23 | -0.926650603 | hsa-mir-4311 | - |
| hsa-mir-4455 | HGLibA_60228 | 23 | -0.926650603 | hsa-mir-4455 | - |
| hsa-mir-4466 | HGLibA_60269 | 23 | -0.926650603 | hsa-mir-4466 | - |
| hsa-mir-4472-1 | HGLibA_60296 | 23 | -0.926650603 | hsa-mir-4472-1 | - |
| hsa-mir-450a-2 | HGLibA_60475 | 23 | -0.926650603 | hsa-mir-450a-2 | - |
| hsa-mir-4684 | HGLibA_60847 | 23 | -0.926650603 | hsa-mir-4684 | - |
| hsa-mir-4695 | HGLibA_60888 | 23 | -0.926650603 | hsa-mir-4695 | - |
| hsa-mir-513c | HGLibA_61581 | 23 | -0.926650603 | hsa-mir-513c | - |
| hsa-mir-520c | HGLibA_61742 | 23 | -0.926650603 | hsa-mir-520c | - |
| hsa-mir-550b-1 | HGLibA_62109 | 23 | -0.926650603 | hsa-mir-550b-1 | - |
| hsa-mir-5586 | HGLibA_62184 | 23 | -0.926650603 | hsa-mir-5586 | - |
| hsa-mir-570 | HGLibA_62333 | 23 | -0.926650603 | hsa-mir-570 | - |
| hsa-mir-5701-2 | HGLibA_62344 | 23 | -0.926650603 | hsa-mir-5701-2 | - |
| hsa-mir-5739 | HGLibA_62385 | 23 | -0.926650603 | hsa-mir-5739 | - |
| hsa-mir-5787 | HGLibA_62411 | 23 | -0.926650603 | hsa-mir-5787 | - |
| hsa-mir-582 | HGLibA_62425 | 23 | -0.926650603 | hsa-mir-582 | - |
| hsa-mir-6516 | HGLibA_62936 | 23 | -0.926650603 | hsa-mir-6516 | - |
| hsa-mir-656 | HGLibA_62956 | 23 | -0.926650603 | hsa-mir-656 | - |
| hsa-mir-6716 | HGLibA_63027 | 23 | -0.926650603 | hsa-mir-6716 | - |
| hsa-mir-6753 | HGLibA_63171 | 23 | -0.926650603 | hsa-mir-6753 | - |
| hsa-mir-6833 | HGLibA_63511 | 23 | -0.926650603 | hsa-mir-6833 | - |
| hsa-mir-6846 | HGLibA_63555 | 23 | -0.926650603 | hsa-mir-6846 | - |
| hsa-mir-6866 | HGLibA_63649 | 23 | -0.926650603 | hsa-mir-6866 | - |
| hsa-mir-6871 | HGLibA_63668 | 23 | -0.926650603 | hsa-mir-6871 | - |
| IL8 | HGLibA_23196 | 23 | -0.926650603 | IL8 | - |
| KIAA1984 | HGLibA_24835 | 23 | -0.926650603 | KIAA1984 | - |
| LOC100862671 | HGLibA_26879 | 23 | -0.926650603 | LOC100862671 | - |
| RARS | HGLibA_40289 | 23 | -0.926650603 | RARS | - |
| TCP10 | HGLibA_48833 | 23 | -0.926650603 | TCP10 | - |
| WBSCR27 | HGLibA_53999 | 23 | -0.926650603 | WBSCR27 | - |
| APITD1 | HGLibA_02424 | 22 | -0.935695946 | APITD1 | - |
| BRE | HGLibA_04794 | 22 | -0.935695946 | BRE | - |
| C17orf53 | HGLibA_05546 | 22 | -0.935695946 | C17orf53 | - |
| C20orf78 | HGLibA_06027 | 22 | -0.935695946 | C20orf78 | - |
| EMR3 | HGLibA_15088 | 22 | -0.935695946 | EMR3 | - |
| ENSG00000005007 | HGLibA_53056 | 22 | -0.935695946 | UPF1 | RNA helicase and ATPase [Source:HGNC Symbol;Acc:HGNC:9962]" |
| ENSG00000005302 | HGLibA_30216 | 22 | -0.935695946 | MSL3 | MSL complex subunit 3 [Source:HGNC Symbol;Acc:HGNC:7370] |
| ENSG00000006740 | HGLibA_02780 | 22 | -0.935695946 | ARHGAP44 | Rho GTPase activating protein 44 [Source:HGNC Symbol;Acc:HGNC:29096] |
| ENSG00000008277 | HGLibA_00757 | 22 | -0.935695946 | ADAM22 | ADAM metallopeptidase domain 22 [Source:HGNC Symbol;Acc:HGNC:201] |
| ENSG00000028203 | HGLibA_53630 | 22 | -0.935695946 | VEZT | adherens junctions transmembrane protein [Source:HGNC Symbol;Acc:HGNC:18258]" |
| ENSG00000038358 | HGLibA_14446 | 22 | -0.935695946 | EDC4 | enhancer of mRNA decapping 4 [Source:HGNC Symbol;Acc:HGNC:17157] |
| ENSG00000056291 | HGLibA_32443 | 22 | -0.935695946 | NPFFR2 | neuropeptide FF receptor 2 [Source:HGNC Symbol;Acc:HGNC:4525] |
| ENSG00000062524 | HGLibA_27611 | 22 | -0.935695946 | LTK | leukocyte receptor tyrosine kinase [Source:HGNC Symbol;Acc:HGNC:6721] |

| | | | | | |
|---|---|---|---|---|---|
| ENSG00000063761 | HGLibA_00890 | 22 | -0.935695946 | ADCK1 | aarF domain containing kinase 1 [Source:HGNC Symbol;Acc:HGNC:19038] |
| ENSG00000066135 | HGLibA_24505 | 22 | -0.935695946 | KDM4A | lysine demethylase 4A [Source:HGNC Symbol;Acc:HGNC:22978] |
| ENSG00000066739 | HGLibA_03482 | 22 | -0.935695946 | ATG2B | autophagy related 2B [Source:HGNC Symbol;Acc:HGNC:20187] |
| ENSG00000068308 | HGLibA_34769 | 22 | -0.935695946 | OTUD5 | OTU deubiquitinase 5 [Source:HGNC Symbol;Acc:HGNC:25402] |
| ENSG00000070081 | HGLibA_32912 | 22 | -0.935695946 | NUCB2 | nucleobindin 2 [Source:HGNC Symbol;Acc:HGNC:8044] |
| ENSG00000070610 | HGLibA_18713 | 22 | -0.935695946 | GBA2 | glucosylceramidase beta 2 [Source:HGNC Symbol;Acc:HGNC:18986] |
| ENSG00000073598 | HGLibA_17758 | 22 | -0.935695946 | FNDC8 | fibronectin type III domain containing 8 [Source:HGNC Symbol;Acc:HGNC:25286] |
| ENSG00000074695 | HGLibA_26656 | 22 | -0.935695946 | LMAN1 | mannose binding 1 [Source:HGNC Symbol;Acc:HGNC:6631]" |
| ENSG00000075234 | HGLibA_52130 | 22 | -0.935695946 | TTC38 | tetratricopeptide repeat domain 38 [Source:HGNC Symbol;Acc:HGNC:26082] |
| ENSG00000075429 | HGLibA_06942 | 22 | -0.935695946 | CACNG5 | calcium voltage-gated channel auxiliary subunit gamma 5 [Source:HGNC Symbol;Acc:HGNC:1409] |
| ENSG00000079785 | HGLibA_12648 | 22 | -0.935695946 | DDX1 | DEAD-box helicase 1 [Source:HGNC Symbol;Acc:HGNC:2734] |
| ENSG00000080644 | HGLibA_09571 | 22 | -0.935695946 | CHRNA3 | cholinergic receptor nicotinic alpha 3 subunit [Source:HGNC Symbol;Acc:HGNC:1957] |
| ENSG00000081923 | HGLibA_03780 | 22 | -0.935695946 | ATP8B1 | ATPase phospholipid transporting 8B1 [Source:HGNC Symbol;Acc:HGNC:3706] |
| ENSG00000081985 | HGLibA_22969 | 22 | -0.935695946 | IL12RB2 | interleukin 12 receptor subunit beta 2 [Source:HGNC Symbol;Acc:HGNC:5972] |
| ENSG00000082293 | HGLibA_10528 | 22 | -0.935695946 | COL19A1 | collagen type XIX alpha 1 chain [Source:HGNC Symbol;Acc:HGNC:2196] |
| ENSG00000083457 | HGLibA_23754 | 22 | -0.935695946 | ITGAE | integrin subunit alpha E [Source:HGNC Symbol;Acc:HGNC:6147] |
| ENSG00000083635 | HGLibA_33000 | 22 | -0.935695946 | NUFIP1 | nuclear FMR1 interacting protein 1 [Source:HGNC Symbol;Acc:HGNC:8057] |
| ENSG00000085265 | HGLibA_17217 | 22 | -0.935695946 | FCN1 | ficolin 1 [Source:HGNC Symbol;Acc:HGNC:3623] |
| ENSG00000086015 | HGLibA_28404 | 22 | -0.935695946 | MAST2 | microtubule associated serine/threonine kinase 2 [Source:HGNC Symbol;Acc:HGNC:19035] |
| ENSG00000086827 | HGLibA_57064 | 22 | -0.935695946 | ZW10 | zw10 kinetochore protein [Source:HGNC Symbol;Acc:HGNC:13194] |
| ENSG00000091656 | HGLibA_55176 | 22 | -0.935695946 | ZFHX4 | zinc finger homeobox 4 [Source:HGNC Symbol;Acc:HGNC:30939] |
| ENSG00000099917 | HGLibA_28757 | 22 | -0.935695946 | MED15 | mediator complex subunit 15 [Source:HGNC Symbol;Acc:HGNC:14248] |
| ENSG00000100601 | HGLibA_01646 | 22 | -0.935695946 | ALKBH1 | histone H2A dioxygenase [Source:HGNC Symbol;Acc:HGNC:17911]" |
| ENSG00000100764 | HGLibA_39127 | 22 | -0.935695946 | PSMC1 | ATPase 1 [Source:HGNC Symbol;Acc:HGNC:9547]" |
| ENSG00000101126 | HGLibA_01002 | 22 | -0.935695946 | ADNP | activity dependent neuroprotector homeobox [Source:HGNC Symbol;Acc:HGNC:15766] |
| ENSG00000101336 | HGLibA_20966 | 22 | -0.935695946 | HCK | Src family tyrosine kinase [Source:HGNC Symbol;Acc:HGNC:4840]" |
| ENSG00000101413 | HGLibA_42048 | 22 | -0.935695946 | RPRD1B | regulation of nuclear pre-mRNA domain containing 1B [Source:HGNC Symbol;Acc:HGNC:16209] |
| ENSG00000102897 | HGLibA_27747 | 22 | -0.935695946 | LYRM1 | LYR motif containing 1 [Source:HGNC Symbol;Acc:HGNC:25074] |
| ENSG00000103154 | HGLibA_31588 | 22 | -0.935695946 | NECAB2 | N-terminal EF-hand calcium binding protein 2 [Source:HGNC Symbol;Acc:HGNC:23746] |
| ENSG00000103642 | HGLibA_25918 | 22 | -0.935695946 | LACTB | lactamase beta [Source:HGNC Symbol;Acc:HGNC:16468] |
| ENSG00000103876 | HGLibA_15953 | 22 | -0.935695946 | FAH | fumarylacetoacetate hydrolase [Source:HGNC Symbol;Acc:HGNC:3579] |
| ENSG00000104853 | HGLibA_10113 | 22 | -0.935695946 | CLPTM1 | transmembrane protein [Source:HGNC Symbol;Acc:HGNC:2087]" |
| ENSG00000104998 | HGLibA_23115 | 22 | -0.935695946 | IL27RA | interleukin 27 receptor subunit alpha [Source:HGNC Symbol;Acc:HGNC:17290] |
| ENSG00000105568 | HGLibA_38139 | 22 | -0.935695946 | PPP2R1A | protein phosphatase 2 scaffold subunit Aalpha [Source:HGNC Symbol;Acc:HGNC:9302] |
| ENSG00000105696 | HGLibA_50462 | 22 | -0.935695946 | TMEM59L | transmembrane protein 59 like [Source:HGNC Symbol;Acc:HGNC:13237] |
| ENSG00000106049 | HGLibA_21283 | 22 | -0.935695946 | HIBADH | 3-hydroxyisobutyrate dehydrogenase [Source:HGNC Symbol;Acc:HGNC:4907] |
| ENSG00000111652 | HGLibA_10728 | 22 | -0.935695946 | COPS7A | COP9 signalosome subunit 7A [Source:HGNC Symbol;Acc:HGNC:16758] |
| ENSG00000112659 | HGLibA_11794 | 22 | -0.935695946 | CUL9 | cullin 9 [Source:HGNC Symbol;Acc:HGNC:15982] |
| ENSG00000112739 | HGLibA_38741 | 22 | -0.935695946 | PRPF4B | pre-mRNA processing factor 4B [Source:HGNC Symbol;Acc:HGNC:17346] |
| ENSG00000112742 | HGLibA_52180 | 22 | -0.935695946 | TTK | TTK protein kinase [Source:HGNC Symbol;Acc:HGNC:12401] |
| ENSG00000113657 | HGLibA_13930 | 22 | -0.935695946 | DPYSL3 | dihydropyrimidinase like 3 [Source:HGNC Symbol;Acc:HGNC:3015] |
| ENSG00000114331 | HGLibA_00386 | 22 | -0.935695946 | ACAP2 | ankyrin repeat and PH domains 2 [Source:HGNC Symbol;Acc:HGNC:16469]" |
| ENSG00000114346 | HGLibA_14424 | 22 | -0.935695946 | ECT2 | epithelial cell transforming 2 [Source:HGNC Symbol;Acc:HGNC:3155] |
| ENSG00000115488 | HGLibA_31712 | 22 | -0.935695946 | NEU2 | neuraminidase 2 [Source:HGNC Symbol;Acc:HGNC:7759] |
| ENSG00000115694 | HGLibA_47474 | 22 | -0.935695946 | STK25 | serine/threonine kinase 25 [Source:HGNC Symbol;Acc:HGNC:11404] |
| ENSG00000116106 | HGLibA_15296 | 22 | -0.935695946 | EPHA4 | EPH receptor A4 [Source:HGNC Symbol;Acc:HGNC:3388] |
| ENSG00000116171 | HGLibA_43034 | 22 | -0.935695946 | SCP2 | sterol carrier protein 2 [Source:HGNC Symbol;Acc:HGNC:10606] |
| ENSG00000116690 | HGLibA_38459 | 22 | -0.935695946 | PRG4 | proteoglycan 4 [Source:HGNC Symbol;Acc:HGNC:9364] |
| ENSG00000116761 | HGLibA_11626 | 22 | -0.935695946 | CTH | cystathionine gamma-lyase [Source:HGNC Symbol;Acc:HGNC:2501] |
| ENSG00000118162 | HGLibA_25382 | 22 | -0.935695946 | KPTN | actin binding protein [Source:HGNC Symbol;Acc:HGNC:6404]" |
| ENSG00000118181 | HGLibA_42116 | 22 | -0.935695946 | RPS25 | ribosomal protein S25 [Source:HGNC Symbol;Acc:HGNC:10413] |
| ENSG00000119616 | HGLibA_17178 | 22 | -0.935695946 | FCF1 | rRNA-processing protein [Source:HGNC Symbol;Acc:HGNC:20220]" |
| ENSG00000119912 | HGLibA_22535 | 22 | -0.935695946 | IDE | insulin degrading enzyme [Source:HGNC Symbol;Acc:HGNC:5381] |
| ENSG00000120210 | HGLibA_23390 | 22 | -0.935695946 | INSL6 | insulin like 6 [Source:HGNC Symbol;Acc:HGNC:6089] |
| ENSG00000122375 | HGLibA_33438 | 22 | -0.935695946 | OPN4 | opsin 4 [Source:HGNC Symbol;Acc:HGNC:14449] |
| ENSG00000122390 | HGLibA_31004 | 22 | -0.935695946 | NAA60 | NatF catalytic subunit [Source:HGNC Symbol;Acc:HGNC:25875]" |
| ENSG00000124102 | HGLibA_36489 | 22 | -0.935695946 | PI3 | peptidase inhibitor 3 [Source:HGNC Symbol;Acc:HGNC:8947] |
| ENSG00000124786 | HGLibA_44966 | 22 | -0.935695946 | SLC35B3 | solute carrier family 35 member B3 [Source:HGNC Symbol;Acc:HGNC:21601] |
| ENSG00000125347 | HGLibA_23571 | 22 | -0.935695946 | IRF1 | interferon regulatory factor 1 [Source:HGNC Symbol;Acc:HGNC:6116] |
| ENSG00000125414 | HGLibA_30722 | 22 | -0.935695946 | MYH2 | myosin heavy chain 2 [Source:HGNC Symbol;Acc:HGNC:7572] |
| ENSG00000125744 | HGLibA_42401 | 22 | -0.935695946 | RTN2 | reticulon 2 [Source:HGNC Symbol;Acc:HGNC:10468] |
| ENSG00000125787 | HGLibA_19584 | 22 | -0.935695946 | GNRH2 | gonadotropin releasing hormone 2 [Source:HGNC Symbol;Acc:HGNC:4420] |
| ENSG00000126231 | HGLibA_38706 | 22 | -0.935695946 | PROZ | vitamin K dependent plasma glycoprotein [Source:HGNC Symbol;Acc:HGNC:9460]" |
| ENSG00000127191 | HGLibA_51243 | 22 | -0.935695946 | TRAF2 | TNF receptor associated factor 2 [Source:HGNC Symbol;Acc:HGNC:12032] |
| ENSG00000128654 | HGLibA_30515 | 22 | -0.935695946 | MTX2 | metaxin 2 [Source:HGNC Symbol;Acc:HGNC:7506] |
| ENSG00000129354 | HGLibA_02292 | 22 | -0.935695946 | AP1M2 | adaptor related protein complex 1 subunit mu 2 [Source:HGNC Symbol;Acc:HGNC:558] |
| ENSG00000130475 | HGLibA_17203 | 22 | -0.935695946 | FCHO1 | FCH domain only 1 [Source:HGNC Symbol;Acc:HGNC:29002] |
| ENSG00000130703 | HGLibA_34653 | 22 | -0.935695946 | OSBPL2 | oxysterol binding protein like 2 [Source:HGNC Symbol;Acc:HGNC:15761] |
| ENSG00000131779 | HGLibA_36086 | 22 | -0.935695946 | PEX11B | peroxisomal biogenesis factor 11 beta [Source:HGNC Symbol;Acc:HGNC:8853] |
| ENSG00000131873 | HGLibA_09654 | 22 | -0.935695946 | CHSY1 | chondroitin sulfate synthase 1 [Source:HGNC Symbol;Acc:HGNC:17198] |
| ENSG00000132446 | HGLibA_18121 | 22 | -0.935695946 | FTHL17 | ferritin heavy chain like 17 [Source:HGNC Symbol;Acc:HGNC:3987] |
| ENSG00000135452 | HGLibA_51928 | 22 | -0.935695946 | TSPAN31 | tetraspanin 31 [Source:HGNC Symbol;Acc:HGNC:10539] |
| ENSG00000135637 | HGLibA_07619 | 22 | -0.935695946 | CCDC142 | coiled-coil domain containing 142 [Source:HGNC Symbol;Acc:HGNC:25889] |
| ENSG00000136206 | HGLibA_46580 | 22 | -0.935695946 | SPDYE1 | speedy/RINGO cell cycle regulator family member E1 [Source:HGNC Symbol;Acc:HGNC:16408] |
| ENSG00000136352 | HGLibA_32032 | 22 | -0.935695946 | NKX2-1 | NK2 homeobox 1 [Source:HGNC Symbol;Acc:HGNC:11825] |
| ENSG00000136754 | HGLibA_00290 | 22 | -0.935695946 | ABI1 | abl interactor 1 [Source:HGNC Symbol;Acc:HGNC:11320] |
| ENSG00000136930 | HGLibA_39117 | 22 | -0.935695946 | PSMB7 | proteasome subunit beta 7 [Source:HGNC Symbol;Acc:HGNC:9544] |
| ENSG00000137392 | HGLibA_10103 | 22 | -0.935695946 | CLPS | colipase [Source:HGNC Symbol;Acc:HGNC:2085] |
| ENSG00000137822 | HGLibA_52316 | 22 | -0.935695946 | TUBGCP4 | tubulin gamma complex associated protein 4 [Source:HGNC Symbol;Acc:HGNC:16691] |
| ENSG00000138161 | HGLibA_11810 | 22 | -0.935695946 | CUZD1 | CUB and zona pellucida like domains 1 [Source:HGNC Symbol;Acc:HGNC:17937] |
| ENSG00000138592 | HGLibA_53412 | 22 | -0.935695946 | USP8 | ubiquitin specific peptidase 8 [Source:HGNC Symbol;Acc:HGNC:12631] |
| ENSG00000138738 | HGLibA_38391 | 22 | -0.935695946 | PRDM5 | PR/SET domain 5 [Source:HGNC Symbol;Acc:HGNC:9349] |
| ENSG00000139675 | HGLibA_21794 | 22 | -0.935695946 | HNRNPA1L2 | heterogeneous nuclear ribonucleoprotein A1-like 2 [Source:HGNC Symbol;Acc:HGNC:27067] |
| ENSG00000140350 | HGLibA_02197 | 22 | -0.935695946 | ANP32A | acidic nuclear phosphoprotein 32 family member A [Source:HGNC Symbol;Acc:HGNC:13233] |
| ENSG00000140365 | HGLibA_10665 | 22 | -0.935695946 | COMMD4 | COMM domain containing 4 [Source:HGNC Symbol;Acc:HGNC:26027] |
| ENSG00000140564 | HGLibA_18173 | 22 | -0.935695946 | FURIN | paired basic amino acid cleaving enzyme [Source:HGNC Symbol;Acc:HGNC:8568]" |
| ENSG00000140932 | HGLibA_10212 | 22 | -0.935695946 | CMTM2 | CKLF like MARVEL transmembrane domain containing 2 [Source:HGNC Symbol;Acc:HGNC:19173] |
| ENSG00000140992 | HGLibA_35932 | 22 | -0.935695946 | PDPK1 | 3-phosphoinositide dependent protein kinase 1 [Source:HGNC Symbol;Acc:HGNC:8816] |
| ENSG00000141013 | HGLibA_18659 | 22 | -0.935695946 | GAS8 | growth arrest specific 8 [Source:HGNC Symbol;Acc:HGNC:4166] |
| ENSG00000141569 | HGLibA_51584 | 22 | -0.935695946 | TRIM65 | tripartite motif containing 65 [Source:HGNC Symbol;Acc:HGNC:27316] |
| ENSG00000143278 | HGLibA_15855 | 22 | -0.935695946 | F13B | coagulation factor XIII B chain [Source:HGNC Symbol;Acc:HGNC:3534] |
| ENSG00000143374 | HGLibA_48349 | 22 | -0.935695946 | TARS2 | mitochondrial [Source:HGNC Symbol;Acc:HGNC:30740]" |
| ENSG00000144559 | HGLibA_48288 | 22 | -0.935695946 | TAMM41 | TAM41 mitochondrial translocator assembly and maintenance homolog [Source:HGNC Symbol;Acc:HGNC:25187] |
| ENSG00000144736 | HGLibA_44085 | 22 | -0.935695946 | SHQ1 | H/ACA ribonucleoprotein assembly factor [Source:HGNC Symbol;Acc:HGNC:25543]" |
| ENSG00000145284 | HGLibA_42888 | 22 | -0.935695946 | SCD5 | stearoyl-CoA desaturase 5 [Source:HGNC Symbol;Acc:HGNC:21088] |
| ENSG00000145990 | HGLibA_18953 | 22 | -0.935695946 | GFOD1 | glucose-fructose oxidoreductase domain containing 1 [Source:HGNC Symbol;Acc:HGNC:21096] |
| ENSG00000146833 | HGLibA_51489 | 22 | -0.935695946 | TRIM4 | tripartite motif containing 4 [Source:HGNC Symbol;Acc:HGNC:16275] |
| ENSG00000148468 | HGLibA_16264 | 22 | -0.935695946 | FAM171A1 | family with sequence similarity 171 member A1 [Source:HGNC Symbol;Acc:HGNC:23522] |
| ENSG00000150433 | HGLibA_50229 | 22 | -0.935695946 | TMEM218 | transmembrane protein 218 [Source:HGNC Symbol;Acc:HGNC:27344] |
| ENSG00000152192 | HGLibA_37777 | 22 | -0.935695946 | POU4F1 | POU class 4 homeobox 1 [Source:HGNC Symbol;Acc:HGNC:9218] |
| ENSG00000153815 | HGLibA_10194 | 22 | -0.935695946 | CMIP | c-Maf inducing protein [Source:HGNC Symbol;Acc:HGNC:24319] |
| ENSG00000155008 | HGLibA_02553 | 22 | -0.935695946 | APOOL | apolipoprotein O like [Source:HGNC Symbol;Acc:HGNC:24009] |
| ENSG00000155269 | HGLibA_20085 | 22 | -0.935695946 | GPR78 | G protein-coupled receptor 78 [Source:HGNC Symbol;Acc:HGNC:4528] |
| ENSG00000156026 | HGLibA_28660 | 22 | -0.935695946 | MCU | mitochondrial calcium uniporter [Source:HGNC Symbol;Acc:HGNC:23526] |
| ENSG00000157152 | HGLibA_47959 | 22 | -0.935695946 | SYN2 | synapsin II [Source:HGNC Symbol;Acc:HGNC:11495] |
| ENSG00000157259 | HGLibA_18683 | 22 | -0.935695946 | GATAD1 | GATA zinc finger domain containing 1 [Source:HGNC Symbol;Acc:HGNC:29941] |
| ENSG00000157985 | HGLibA_01135 | 22 | -0.935695946 | AGAP1 | ankyrin repeat and PH domain 1 [Source:HGNC Symbol;Acc:HGNC:16922]" |
| ENSG00000158042 | HGLibA_29913 | 22 | -0.935695946 | MRPL17 | mitochondrial ribosomal protein L17 [Source:HGNC Symbol;Acc:HGNC:14053] |
| ENSG00000159224 | HGLibA_19116 | 22 | -0.935695946 | GIP | gastric inhibitory polypeptide [Source:HGNC Symbol;Acc:HGNC:4270] |
| ENSG00000159593 | HGLibA_31058 | 22 | -0.935695946 | NAE1 | NEDD8 activating enzyme E1 subunit 1 [Source:HGNC Symbol;Acc:HGNC:621] |
| ENSG00000159708 | HGLibA_27322 | 22 | -0.935695946 | LRRC36 | leucine rich repeat containing 36 [Source:HGNC Symbol;Acc:HGNC:25615] |
| ENSG00000160199 | HGLibA_36847 | 22 | -0.935695946 | PKNOX1 | PBX/knotted 1 homeobox 1 [Source:HGNC Symbol;Acc:HGNC:9022] |
| ENSG00000160352 | HGLibA_56621 | 22 | -0.935695946 | ZNF714 | zinc finger protein 714 [Source:HGNC Symbol;Acc:HGNC:27124] |
| ENSG00000160870 | HGLibA_12163 | 22 | -0.935695946 | CYP3A7 | cytochrome P450 family 3 subfamily A member 7 [Source:HGNC Symbol;Acc:HGNC:2640] |
| ENSG00000161682 | HGLibA_16268 | 22 | -0.935695946 | FAM171A2 | family with sequence similarity 171 member A2 [Source:HGNC Symbol;Acc:HGNC:30480] |
| ENSG00000162642 | HGLibA_05866 | 22 | -0.935695946 | C1orf52 | chromosome 1 open reading frame 52 [Source:HGNC Symbol;Acc:HGNC:24871] |
| ENSG00000162813 | HGLibA_04738 | 22 | -0.935695946 | BPNT1 | 5'-bisphosphate nucleotidase 1 [Source:HGNC Symbol;Acc:HGNC:1096]" |
| ENSG00000162873 | HGLibA_25111 | 22 | -0.935695946 | KLHDC8A | kelch domain containing 8A [Source:HGNC Symbol;Acc:HGNC:25573] |
| ENSG00000163534 | HGLibA_17226 | 22 | -0.935695946 | FCRL1 | Fc receptor like 1 [Source:HGNC Symbol;Acc:HGNC:18509] |
| ENSG00000163564 | HGLibA_39700 | 22 | -0.935695946 | PYHIN1 | pyrin and HIN domain family member 1 [Source:HGNC Symbol;Acc:HGNC:28894] |
| ENSG00000164050 | HGLibA_37270 | 22 | -0.935695946 | PLXNB1 | plexin B1 [Source:HGNC Symbol;Acc:HGNC:9103] |
| ENSG00000165092 | HGLibA_01540 | 22 | -0.935695946 | ALDH1A1 | aldehyde dehydrogenase 1 family member A1 [Source:HGNC Symbol;Acc:HGNC:402] |
| ENSG00000165644 | HGLibA_10690 | 22 | -0.935695946 | COMTD1 | catechol-O-methyltransferase domain containing 1 [Source:HGNC Symbol;Acc:HGNC:26309] |
| ENSG00000165943 | HGLibA_29581 | 22 | -0.935695946 | MOAP1 | modulator of apoptosis 1 [Source:HGNC Symbol;Acc:HGNC:16658] |
| ENSG00000166118 | HGLibA_46441 | 22 | -0.935695946 | SPATA19 | spermatogenesis associated 19 [Source:HGNC Symbol;Acc:HGNC:30614] |
| ENSG00000166546 | HGLibA_04392 | 22 | -0.935695946 | BEAN1 | brain expressed associated with NEDD4 1 [Source:HGNC Symbol;Acc:HGNC:24160] |
| ENSG00000166569 | HGLibA_10940 | 22 | -0.935695946 | CPLX4 | complexin 4 [Source:HGNC Symbol;Acc:HGNC:24330] |
| ENSG00000166889 | HGLibA_35282 | 22 | -0.935695946 | PATL1 | processing body mRNA decay factor [Source:HGNC Symbol;Acc:HGNC:26721]" |
| ENSG00000167114 | HGLibA_44823 | 22 | -0.935695946 | SLC27A4 | solute carrier family 27 member 4 [Source:HGNC Symbol;Acc:HGNC:10998] |
| ENSG00000167566 | HGLibA_31319 | 22 | -0.935695946 | NCKAP5L | NCK associated protein 5 like [Source:HGNC Symbol;Acc:HGNC:29321] |
| ENSG00000167612 | HGLibA_02070 | 22 | -0.935695946 | ANKRD33 | ankyrin repeat domain 33 [Source:HGNC Symbol;Acc:HGNC:13788] |
| ENSG00000167723 | HGLibA_51788 | 22 | -0.935695946 | TRPV3 | transient receptor potential cation channel subfamily V member 3 [Source:HGNC Symbol;Acc:HGNC:18084] |
| ENSG00000167825 | HGLibA_34304 | 22 | -0.935695946 | OR5I1 | olfactory receptor family 5 subfamily I member 1 [Source:HGNC Symbol;Acc:HGNC:8347] |
| ENSG00000168288 | HGLibA_29458 | 22 | -0.935695946 | MMADHC | metabolism of cobalamin associated D [Source:HGNC Symbol;Acc:HGNC:25221] |
| ENSG00000169184 | HGLibA_29558 | 22 | -0.935695946 | MN1 | transcriptional regulator [Source:HGNC Symbol;Acc:HGNC:7180]" |

| | | | | | |
|---|---|---|---|---|---|
| ENSG00000169221 | HGLibA_48468 | 22 | -0.935695946 | TBC1D10B | TBC1 domain family member 10B [Source:HGNC Symbol;Acc:HGNC:24510] |
| ENSG00000170417 | HGLibA_50101 | 22 | -0.935695946 | TMEM182 | transmembrane protein 182 [Source:HGNC Symbol;Acc:HGNC:26391] |
| ENSG00000170627 | HGLibA_20628 | 22 | -0.935695946 | GTSF1 | gametocyte specific factor 1 [Source:HGNC Symbol;Acc:HGNC:26565] |
| ENSG00000170948 | HGLibA_28471 | 22 | -0.935695946 | MBD3L1 | methyl-CpG binding domain protein 3 like 1 [Source:HGNC Symbol;Acc:HGNC:15774] |
| ENSG00000171060 | HGLibA_06644 | 22 | -0.935695946 | C8orf74 | chromosome 8 open reading frame 74 [Source:HGNC Symbol;Acc:HGNC:32296] |
| ENSG00000171236 | HGLibA_27166 | 22 | -0.935695946 | LRG1 | leucine rich alpha-2-glycoprotein 1 [Source:HGNC Symbol;Acc:HGNC:29480] |
| ENSG00000171302 | HGLibA_07128 | 22 | -0.935695946 | CANT1 | calcium activated nucleotidase 1 [Source:HGNC Symbol;Acc:HGNC:19721] |
| ENSG00000171316 | HGLibA_09404 | 22 | -0.935695946 | CHD7 | chromodomain helicase DNA binding protein 7 [Source:HGNC Symbol;Acc:HGNC:20626] |
| ENSG00000171425 | HGLibA_56309 | 22 | -0.935695946 | ZNF581 | zinc finger protein 581 [Source:HGNC Symbol;Acc:HGNC:25017] |
| ENSG00000171759 | HGLibA_35023 | 22 | -0.935695946 | PAH | phenylalanine hydroxylase [Source:HGNC Symbol;Acc:HGNC:8582] |
| ENSG00000173153 | HGLibA_15592 | 22 | -0.935695946 | ESRRA | estrogen related receptor alpha [Source:HGNC Symbol;Acc:HGNC:3471] |
| ENSG00000173545 | HGLibA_56409 | 22 | -0.935695946 | ZNF622 | zinc finger protein 622 [Source:HGNC Symbol;Acc:HGNC:30958] |
| ENSG00000173548 | HGLibA_46094 | 22 | -0.935695946 | SNX33 | sorting nexin 33 [Source:HGNC Symbol;Acc:HGNC:28468] |
| ENSG00000173599 | HGLibA_35358 | 22 | -0.935695946 | PC | pyruvate carboxylase [Source:HGNC Symbol;Acc:HGNC:8636] |
| ENSG00000173918 | HGLibA_05935 | 22 | -0.935695946 | C1QTNF1 | C1q and TNF related 1 [Source:HGNC Symbol;Acc:HGNC:14324] |
| ENSG00000174236 | HGLibA_40841 | 22 | -0.935695946 | REP15 | RAB15 effector protein [Source:HGNC Symbol;Acc:HGNC:33748] |
| ENSG00000174306 | HGLibA_55319 | 22 | -0.935695946 | ZHX3 | zinc fingers and homeoboxes 3 [Source:HGNC Symbol;Acc:HGNC:15935] |
| ENSG00000174516 | HGLibA_36038 | 22 | -0.935695946 | PELI3 | pellino E3 ubiquitin protein ligase family member 3 [Source:HGNC Symbol;Acc:HGNC:30010] |
| ENSG00000175018 | HGLibA_49104 | 22 | -0.935695946 | TEX36 | testis expressed 36 [Source:HGNC Symbol;Acc:HGNC:31653] |
| ENSG00000175718 | HGLibA_40608 | 22 | -0.935695946 | RBMXL3 | RBMX like 3 [Source:HGNC Symbol;Acc:HGNC:26859] |
| ENSG00000176046 | HGLibA_33088 | 22 | -0.935695946 | NUPR1 | transcriptional regulator [Source:HGNC Symbol;Acc:HGNC:29990]" |
| ENSG00000176083 | HGLibA_56534 | 22 | -0.935695946 | ZNF683 | zinc finger protein 683 [Source:HGNC Symbol;Acc:HGNC:28495] |
| ENSG00000176971 | HGLibA_17500 | 22 | -0.935695946 | FIBIN | fin bud initiation factor homolog [Source:HGNC Symbol;Acc:HGNC:33747] |
| ENSG00000177169 | HGLibA_52970 | 22 | -0.935695946 | ULK1 | unc-51 like autophagy activating kinase 1 [Source:HGNC Symbol;Acc:HGNC:12558] |
| ENSG00000177174 | HGLibA_33633 | 22 | -0.935695946 | OR14C36 | olfactory receptor family 14 subfamily C member 36 [Source:HGNC Symbol;Acc:HGNC:15026] |
| ENSG00000177990 | HGLibA_13908 | 22 | -0.935695946 | DPY19L2 | dpy-19 like 2 [Source:HGNC Symbol;Acc:HGNC:19414] |
| ENSG00000178821 | HGLibA_50432 | 22 | -0.935695946 | TMEM52 | transmembrane protein 52 [Source:HGNC Symbol;Acc:HGNC:27916] |
| ENSG00000179348 | HGLibA_18667 | 22 | -0.935695946 | GATA2 | GATA binding protein 2 [Source:HGNC Symbol;Acc:HGNC:4171] |
| ENSG00000179454 | HGLibA_25171 | 22 | -0.935695946 | KLHL28 | kelch like family member 28 [Source:HGNC Symbol;Acc:HGNC:19741] |
| ENSG00000179674 | HGLibA_02937 | 22 | -0.935695946 | ARL14 | ADP ribosylation factor like GTPase 14 [Source:HGNC Symbol;Acc:HGNC:22974] |
| ENSG00000180190 | HGLibA_48922 | 22 | -0.935695946 | TDRP | testis development related protein [Source:HGNC Symbol;Acc:HGNC:26951] |
| ENSG00000181072 | HGLibA_09551 | 22 | -0.935695946 | CHRM2 | cholinergic receptor muscarinic 2 [Source:HGNC Symbol;Acc:HGNC:1951] |
| ENSG00000181690 | HGLibA_36951 | 22 | -0.935695946 | PLAG1 | PLAG1 zinc finger [Source:HGNC Symbol;Acc:HGNC:9045] |
| ENSG00000182083 | HGLibA_34375 | 22 | -0.935695946 | OR6B2 | olfactory receptor family 6 subfamily B member 2 [Source:HGNC Symbol;Acc:HGNC:15041] |
| ENSG00000182108 | HGLibA_13015 | 22 | -0.935695946 | DEXI | Dexi homolog [Source:HGNC Symbol;Acc:HGNC:13267] |
| ENSG00000182504 | HGLibA_09181 | 22 | -0.935695946 | CEP97 | centrosomal protein 97 [Source:HGNC Symbol;Acc:HGNC:26244] |
| ENSG00000182831 | HGLibA_05476 | 22 | -0.935695946 | C16orf72 | chromosome 16 open reading frame 72 [Source:HGNC Symbol;Acc:HGNC:30103] |
| ENSG00000183463 | HGLibA_53130 | 22 | -0.935695946 | URAD | ureidoimidazoline (2-oxo-4-hydroxy-4-carboxy-5-) decarboxylase [Source:HGNC Symbol;Acc:HGNC:17785] |
| ENSG00000184478 | HGLibA_34214 | 22 | -0.935695946 | OR56A3 | olfactory receptor family 56 subfamily A member 3 [Source:HGNC Symbol;Acc:HGNC:14786] |
| ENSG00000184743 | HGLibA_03520 | 22 | -0.935695946 | ATL3 | atlastin GTPase 3 [Source:HGNC Symbol;Acc:HGNC:24526] |
| ENSG00000184939 | HGLibA_55239 | 22 | -0.935695946 | ZFP90 | ZFP90 zinc finger protein [Source:HGNC Symbol;Acc:HGNC:23329] |
| ENSG00000185294 | HGLibA_46780 | 22 | -0.935695946 | SPPL2C | signal peptide peptidase like 2C [Source:HGNC Symbol;Acc:HGNC:28902] |
| ENSG00000185920 | HGLibA_39269 | 22 | -0.935695946 | PTCH1 | patched 1 [Source:HGNC Symbol;Acc:HGNC:9585] |
| ENSG00000187080 | HGLibA_33756 | 22 | -0.935695946 | OR2AK2 | olfactory receptor family 2 subfamily AK member 2 [Source:HGNC Symbol;Acc:HGNC:19569] |
| ENSG00000187240 | HGLibA_14248 | 22 | -0.935695946 | DYNC2H1 | dynein cytoplasmic 2 heavy chain 1 [Source:HGNC Symbol;Acc:HGNC:2962] |
| ENSG00000188691 | HGLibA_34221 | 22 | -0.935695946 | OR56A5 | olfactory receptor family 56 subfamily A member 5 [Source:HGNC Symbol;Acc:HGNC:14792] |
| ENSG00000189169 | HGLibA_25599 | 22 | -0.935695946 | KRTAP10-12 | keratin associated protein 10-12 [Source:HGNC Symbol;Acc:HGNC:20533] |
| ENSG00000189266 | HGLibA_37439 | 22 | -0.935695946 | PNRC2 | proline rich nuclear receptor coactivator 2 [Source:HGNC Symbol;Acc:HGNC:23158] |
| ENSG00000189401 | HGLibA_34772 | 22 | -0.935695946 | OTUD6A | OTU deubiquitinase 6A [Source:HGNC Symbol;Acc:HGNC:32312] |
| ENSG00000196924 | HGLibA_17640 | 22 | -0.935695946 | FLNA | filamin A [Source:HGNC Symbol;Acc:HGNC:3754] |
| ENSG00000197879 | HGLibA_30827 | 22 | -0.935695946 | MYO1C | myosin IC [Source:HGNC Symbol;Acc:HGNC:7597] |
| ENSG00000198075 | HGLibA_47752 | 22 | -0.935695946 | SULT1C4 | sulfotransferase family 1C member 4 [Source:HGNC Symbol;Acc:HGNC:11457] |
| ENSG00000198563 | HGLibA_12699 | 22 | -0.935695946 | DDX39B | DExD-box helicase 39B [Source:HGNC Symbol;Acc:HGNC:13917] |
| ENSG00000198590 | HGLibA_06233 | 22 | -0.935695946 | C3orf35 | chromosome 3 open reading frame 35 [Source:HGNC Symbol;Acc:HGNC:24082] |
| ENSG00000203705 | HGLibA_48447 | 22 | -0.935695946 | TATDN3 | TatD DNase domain containing 3 [Source:HGNC Symbol;Acc:HGNC:27010] |
| ENSG00000204524 | HGLibA_56794 | 22 | -0.935695946 | ZNF805 | zinc finger protein 805 [Source:HGNC Symbol;Acc:HGNC:23272] |
| ENSG00000205133 | HGLibA_51652 | 22 | -0.935695946 | TRIQK | triple QxxK/R motif containing [Source:HGNC Symbol;Acc:HGNC:27828] |
| ENSG00000205544 | HGLibA_50336 | 22 | -0.935695946 | TMEM256 | transmembrane protein 256 [Source:HGNC Symbol;Acc:HGNC:28618] |
| ENSG00000213928 | HGLibA_23603 | 22 | -0.935695946 | IRF9 | interferon regulatory factor 9 [Source:HGNC Symbol;Acc:HGNC:6131] |
| ENSG00000214827 | HGLibA_30330 | 22 | -0.935695946 | MTCP1 | mature T cell proliferation 1 [Source:HGNC Symbol;Acc:HGNC:7423] |
| ENSG00000221900 | HGLibA_37647 | 22 | -0.935695946 | POM121L12 | POM121 transmembrane nucleoporin like 12 [Source:HGNC Symbol;Acc:HGNC:25369] |
| ENSG00000221946 | HGLibA_18246 | 22 | -0.935695946 | FXYD7 | FXYD domain containing ion transport regulator 7 [Source:HGNC Symbol;Acc:HGNC:4034] |
| ENSG00000226784 | HGLibA_36200 | 22 | -0.935695946 | PGAM4 | phosphoglycerate mutase family member 4 [Source:HGNC Symbol;Acc:HGNC:21731] |
| ENSG00000226941 | HGLibA_40626 | 22 | -0.935695946 | RBMY1J | member J [Source:HGNC Symbol;Acc:HGNC:23917]" |
| ENSG00000234965 | HGLibA_44051 | 22 | -0.935695946 | SHISA8 | shisa family member 8 [Source:HGNC Symbol;Acc:HGNC:18351] |
| ENSG00000237388 | HGLibA_33928 | 22 | -0.935695946 | OR4A47 | olfactory receptor family 4 subfamily A member 47 [Source:HGNC Symbol;Acc:HGNC:31266] |
| ENSG00000239887 | HGLibA_05833 | 22 | -0.935695946 | C1orf226 | chromosome 1 open reading frame 226 [Source:HGNC Symbol;Acc:HGNC:34351] |
| ENSG00000257949 | HGLibA_48990 | 22 | -0.935695946 | TEN1 | CST complex subunit [Source:HGNC Symbol;Acc:HGNC:37242]" |
| ENSG00000258724 | HGLibA_36697 | 22 | -0.935695946 | PINX1 | PIN2 (TERF1) interacting telomerase inhibitor 1 [Source:HGNC Symbol;Acc:HGNC:30046] |
| ENSG00000261272 | HGLibA_30545 | 22 | -0.935695946 | MUC22 | mucin 22 [Source:HGNC Symbol;Acc:HGNC:39755] |
| ENSG00000261652 | HGLibA_05435 | 22 | -0.935695946 | C15orf65 | chromosome 15 open reading frame 65 [Source:HGNC Symbol;Acc:HGNC:44654] |
| ENSG00000267909 | HGLibA_07713 | 22 | -0.935695946 | CCDC177 | coiled-coil domain containing 177 [Source:HGNC Symbol;Acc:HGNC:23243] |
| ENSG00000273611 | HGLibA_56929 | 22 | -0.935695946 | ZNHIT3 | zinc finger HIT-type containing 3 [Source:HGNC Symbol;Acc:HGNC:12309] |
| ENSG00000274274 | HGLibA_18464 | 22 | -0.935695946 | GAGE13 | G antigen 13 [Source:HGNC Symbol;Acc:HGNC:29081] |
| ENSG00000275113 | HGLibA_18479 | 22 | -0.935695946 | GAGE2E | G antigen 2E [Source:HGNC Symbol;Acc:HGNC:31960] |
| ENSG00000280314 | HGLibA_34547 | 22 | -0.935695946 | OR8K3 | olfactory receptor family 8 subfamily K member 3 (gene/pseudogene) [Source:HGNC Symbol;Acc:HGNC:15313] |
| FAM194B | HGLibA_16371 | 22 | -0.935695946 | FAM194B | - |
| GRAMD2 | HGLibA_20204 | 22 | -0.935695946 | GRAMD2 | - |
| hsa-mir-101-2 | HGLibA_57158 | 22 | -0.935695946 | hsa-mir-101-2 | - |
| hsa-mir-1250 | HGLibA_57397 | 22 | -0.935695946 | hsa-mir-1250 | - |
| hsa-mir-1258 | HGLibA_57440 | 22 | -0.935695946 | hsa-mir-1258 | - |
| hsa-mir-1282 | HGLibA_57586 | 22 | -0.935695946 | hsa-mir-1282 | - |
| hsa-mir-1302-4 | HGLibA_57705 | 22 | -0.935695946 | hsa-mir-1302-4 | - |
| hsa-mir-15a | HGLibA_57938 | 22 | -0.935695946 | hsa-mir-15a | - |
| hsa-mir-24-2 | HGLibA_58346 | 22 | -0.935695946 | hsa-mir-24-2 | - |
| hsa-mir-3122 | HGLibA_58541 | 22 | -0.935695946 | hsa-mir-3122 | - |
| hsa-mir-3185 | HGLibA_58838 | 22 | -0.935695946 | hsa-mir-3185 | - |
| hsa-mir-3658 | HGLibA_59178 | 22 | -0.935695946 | hsa-mir-3658 | - |
| hsa-mir-378g | HGLibA_59452 | 22 | -0.935695946 | hsa-mir-378g | - |
| hsa-mir-381 | HGLibA_59476 | 22 | -0.935695946 | hsa-mir-381 | - |
| hsa-mir-3928 | HGLibA_59588 | 22 | -0.935695946 | hsa-mir-3928 | - |
| hsa-mir-4460 | HGLibA_60245 | 22 | -0.935695946 | hsa-mir-4460 | - |
| hsa-mir-4662b | HGLibA_60763 | 22 | -0.935695946 | hsa-mir-4662b | - |
| hsa-mir-4748 | HGLibA_61090 | 22 | -0.935695946 | hsa-mir-4748 | - |
| hsa-mir-4760 | HGLibA_61137 | 22 | -0.935695946 | hsa-mir-4760 | - |
| hsa-mir-4798 | HGLibA_61298 | 22 | -0.935695946 | hsa-mir-4798 | - |
| hsa-mir-500b | HGLibA_61449 | 22 | -0.935695946 | hsa-mir-500b | - |
| hsa-mir-512-2 | HGLibA_61566 | 22 | -0.935695946 | hsa-mir-512-2 | - |
| hsa-mir-517c | HGLibA_61633 | 22 | -0.935695946 | hsa-mir-517c | - |
| hsa-mir-517c | HGLibA_61632 | 22 | -0.935695946 | hsa-mir-517c | - |
| hsa-mir-520b | HGLibA_61739 | 22 | -0.935695946 | hsa-mir-520b | - |
| hsa-mir-555 | HGLibA_62136 | 22 | -0.935695946 | hsa-mir-555 | - |
| hsa-mir-590 | HGLibA_62458 | 22 | -0.935695946 | hsa-mir-590 | - |
| hsa-mir-611 | HGLibA_62636 | 22 | -0.935695946 | hsa-mir-611 | - |
| hsa-mir-620 | HGLibA_62718 | 22 | -0.935695946 | hsa-mir-620 | - |
| hsa-mir-642b | HGLibA_62810 | 22 | -0.935695946 | hsa-mir-642b | - |
| hsa-mir-6737 | HGLibA_63103 | 22 | -0.935695946 | hsa-mir-6737 | - |
| hsa-mir-6802 | HGLibA_63384 | 22 | -0.935695946 | hsa-mir-6802 | - |
| hsa-mir-6867 | HGLibA_63654 | 22 | -0.935695946 | hsa-mir-6867 | - |
| hsa-mir-7150 | HGLibA_63827 | 22 | -0.935695946 | hsa-mir-7150 | - |
| KIAA0146 | HGLibA_24595 | 22 | -0.935695946 | KIAA0146 | - |
| KIAA1598 | HGLibA_24789 | 22 | -0.935695946 | KIAA1598 | - |
| LOC391322 | HGLibA_26933 | 22 | -0.935695946 | LOC391322 | - |
| MFI2 | HGLibA_29067 | 22 | -0.935695946 | MFI2 | - |
| MLL2 | HGLibA_29396 | 22 | -0.935695946 | MLL2 | - |
| MUM1 | HGLibA_30566 | 22 | -0.935695946 | MUM1 | - |
| PPAP2B | HGLibA_37820 | 22 | -0.935695946 | PPAP2B | - |
| PQLC1 | HGLibA_38251 | 22 | -0.935695946 | PQLC1 | - |
| SSSCA1 | HGLibA_47169 | 22 | -0.935695946 | SSSCA1 | - |
| ST5 | HGLibA_47254 | 22 | -0.935695946 | ST5 | - |
| TBC1D29 | HGLibA_48523 | 22 | -0.935695946 | TBC1D29 | - |
| TCEB3CL2 | HGLibA_48753 | 22 | -0.935695946 | TCEB3CL2 | - |
| TTC18 | HGLibA_52064 | 22 | -0.935695946 | TTC18 | - |
| UFD1L | HGLibA_52847 | 22 | -0.935695946 | UFD1L | - |
| B3GALTL | HGLibA_03970 | 21 | -0.944741289 | B3GALTL | - |
| C10orf91 | HGLibA_05105 | 21 | -0.944741289 | C10orf91 | - |
| C11orf88 | HGLibA_05208 | 21 | -0.944741289 | C11orf88 | - |
| C2orf47 | HGLibA_06135 | 21 | -0.944741289 | C2orf47 | - |
| C2orf47 | HGLibA_06133 | 21 | -0.944741289 | C2orf47 | - |
| ENSG00000005187 | HGLibA_00579 | 21 | -0.944741289 | ACSM3 | acyl-CoA synthetase medium chain family member 3 [Source:HGNC Symbol;Acc:HGNC:10522] |
| ENSG00000005448 | HGLibA_54124 | 21 | -0.944741289 | WDR54 | WD repeat domain 54 [Source:HGNC Symbol;Acc:HGNC:25770] |
| ENSG00000007402 | HGLibA_06907 | 21 | -0.944741289 | CACNA2D2 | calcium voltage-gated channel auxiliary subunit alpha2delta 2 [Source:HGNC Symbol;Acc:HGNC:1400] |
| ENSG00000008710 | HGLibA_36788 | 21 | -0.944741289 | PKD1 | transient receptor potential channel interacting [Source:HGNC Symbol;Acc:HGNC:9008]" |
| ENSG00000011422 | HGLibA_36965 | 21 | -0.944741289 | PLAUR | urokinase receptor [Source:HGNC Symbol;Acc:HGNC:9053]" |
| ENSG00000038945 | HGLibA_30235 | 21 | -0.944741289 | MSR1 | macrophage scavenger receptor 1 [Source:HGNC Symbol;Acc:HGNC:7376] |

| | | | | | |
|---|---|---|---|---|---|
| ENSG00000049089 | HGLibA_10623 | 21 | -0.944741289 | COL9A2 | collagen type IX alpha 2 chain [Source:HGNC Symbol;Acc:HGNC:2218] |
| ENSG00000054690 | HGLibA_37124 | 21 | -0.944741289 | PLEKHH1 | MyTH4 and FERM domain containing H1 [Source:HGNC Symbol;Acc:HGNC:17733]" |
| ENSG00000055147 | HGLibA_16032 | 21 | -0.944741289 | FAM114A2 | family with sequence similarity 114 member A2 [Source:HGNC Symbol;Acc:HGNC:1333] |
| ENSG00000062524 | HGLibA_27610 | 21 | -0.944741289 | LTK | leukocyte receptor tyrosine kinase [Source:HGNC Symbol;Acc:HGNC:6721] |
| ENSG00000064652 | HGLibA_46069 | 21 | -0.944741289 | SNX24 | sorting nexin 24 [Source:HGNC Symbol;Acc:HGNC:21533] |
| ENSG00000065491 | HGLibA_48507 | 21 | -0.944741289 | TBC1D22B | TBC1 domain family member 22B [Source:HGNC Symbol;Acc:HGNC:21602] |
| ENSG00000066557 | HGLibA_27352 | 21 | -0.944741289 | LRRC40 | leucine rich repeat containing 40 [Source:HGNC Symbol;Acc:HGNC:26004] |
| ENSG00000069493 | HGLibA_09967 | 21 | -0.944741289 | CLEC2D | C-type lectin domain family 2 member D [Source:HGNC Symbol;Acc:HGNC:14351] |
| ENSG00000073146 | HGLibA_29695 | 21 | -0.944741289 | MOV10L1 | Mov10 like RISC complex RNA helicase 1 [Source:HGNC Symbol;Acc:HGNC:7201] |
| ENSG00000073712 | HGLibA_17298 | 21 | -0.944741289 | FERMT2 | fermitin family member 2 [Source:HGNC Symbol;Acc:HGNC:15767] |
| ENSG00000073910 | HGLibA_18053 | 21 | -0.944741289 | FRY | FRY microtubule binding protein [Source:HGNC Symbol;Acc:HGNC:20367] |
| ENSG00000078687 | HGLibA_50920 | 21 | -0.944741289 | TNRC6C | trinucleotide repeat containing 6C [Source:HGNC Symbol;Acc:HGNC:29318] |
| ENSG00000079432 | HGLibA_09695 | 21 | -0.944741289 | CIC | capicua transcriptional repressor [Source:HGNC Symbol;Acc:HGNC:14214] |
| ENSG00000079805 | HGLibA_13690 | 21 | -0.944741289 | DNM2 | dynamin 2 [Source:HGNC Symbol;Acc:HGNC:2974] |
| ENSG00000092330 | HGLibA_49552 | 21 | -0.944741289 | TINF2 | TERF1 interacting nuclear factor 2 [Source:HGNC Symbol;Acc:HGNC:11824] |
| ENSG00000096006 | HGLibA_11172 | 21 | -0.944741289 | CRISP3 | cysteine rich secretory protein 3 [Source:HGNC Symbol;Acc:HGNC:16904] |
| ENSG00000100068 | HGLibA_27226 | 21 | -0.944741289 | LRP5L | LDL receptor related protein 5 like [Source:HGNC Symbol;Acc:HGNC:25323] |
| ENSG00000100784 | HGLibA_42169 | 21 | -0.944741289 | RPS6KA5 | ribosomal protein S6 kinase A5 [Source:HGNC Symbol;Acc:HGNC:10434] |
| ENSG00000101132 | HGLibA_36145 | 21 | -0.944741289 | PFDN4 | prefoldin subunit 4 [Source:HGNC Symbol;Acc:HGNC:8868] |
| ENSG00000103549 | HGLibA_41678 | 21 | -0.944741289 | RNF40 | ring finger protein 40 [Source:HGNC Symbol;Acc:HGNC:16867] |
| ENSG00000103742 | HGLibA_22787 | 21 | -0.944741289 | IGDCC4 | immunoglobulin superfamily DCC subclass member 4 [Source:HGNC Symbol;Acc:HGNC:13770] |
| ENSG00000104918 | HGLibA_40875 | 21 | -0.944741289 | RETN | resistin [Source:HGNC Symbol;Acc:HGNC:20389] |
| ENSG00000104967 | HGLibA_32367 | 21 | -0.944741289 | NOVA2 | NOVA alternative splicing regulator 2 [Source:HGNC Symbol;Acc:HGNC:7887] |
| ENSG00000105193 | HGLibA_42084 | 21 | -0.944741289 | RPS16 | ribosomal protein S16 [Source:HGNC Symbol;Acc:HGNC:10396] |
| ENSG00000105388 | HGLibA_08943 | 21 | -0.944741289 | CEACAM5 | carcinoembryonic antigen related cell adhesion molecule 5 [Source:HGNC Symbol;Acc:HGNC:1817] |
| ENSG00000106012 | HGLibA_23498 | 21 | -0.944741289 | IQCE | IQ motif containing E [Source:HGNC Symbol;Acc:HGNC:29171] |
| ENSG00000106331 | HGLibA_35304 | 21 | -0.944741289 | PAX4 | paired box 4 [Source:HGNC Symbol;Acc:HGNC:8618] |
| ENSG00000106400 | HGLibA_56923 | 21 | -0.944741289 | ZNHIT1 | zinc finger HIT-type containing 1 [Source:HGNC Symbol;Acc:HGNC:21688] |
| ENSG00000108465 | HGLibA_08789 | 21 | -0.944741289 | CDK5RAP3 | CDK5 regulatory subunit associated protein 3 [Source:HGNC Symbol;Acc:HGNC:18673] |
| ENSG00000109610 | HGLibA_46150 | 21 | -0.944741289 | SOD3 | superoxide dismutase 3 [Source:HGNC Symbol;Acc:HGNC:11181] |
| ENSG00000109929 | HGLibA_42817 | 21 | -0.944741289 | SC5D | sterol-C5-desaturase [Source:HGNC Symbol;Acc:HGNC:10547] |
| ENSG00000111199 | HGLibA_51792 | 21 | -0.944741289 | TRPV4 | transient receptor potential cation channel subfamily V member 4 [Source:HGNC Symbol;Acc:HGNC:18083] |
| ENSG00000111602 | HGLibA_49489 | 21 | -0.944741289 | TIMELESS | timeless circadian regulator [Source:HGNC Symbol;Acc:HGNC:11813] |
| ENSG00000112245 | HGLibA_39404 | 21 | -0.944741289 | PTP4A1 | member 1 [Source:HGNC Symbol;Acc:HGNC:9634]" |
| ENSG00000112499 | HGLibA_44568 | 21 | -0.944741289 | SLC22A2 | solute carrier family 22 member 2 [Source:HGNC Symbol;Acc:HGNC:10966] |
| ENSG00000113013 | HGLibA_22298 | 21 | -0.944741289 | HSPA9 | heat shock protein family A (Hsp70) member 9 [Source:HGNC Symbol;Acc:HGNC:5244] |
| ENSG00000113161 | HGLibA_21707 | 21 | -0.944741289 | HMGCR | 3-hydroxy-3-methylglutaryl-CoA reductase [Source:HGNC Symbol;Acc:HGNC:5006] |
| ENSG00000113327 | HGLibA_18393 | 21 | -0.944741289 | GABRG2 | gamma-aminobutyric acid type A receptor gamma2 subunit [Source:HGNC Symbol;Acc:HGNC:4087] |
| ENSG00000114279 | HGLibA_17369 | 21 | -0.944741289 | FGF12 | fibroblast growth factor 12 [Source:HGNC Symbol;Acc:HGNC:3668] |
| ENSG00000114631 | HGLibA_37461 | 21 | -0.944741289 | PODXL2 | podocalyxin like 2 [Source:HGNC Symbol;Acc:HGNC:17936] |
| ENSG00000115073 | HGLibA_00652 | 21 | -0.944741289 | ACTR1B | ARP1 actin related protein 1 homolog B [Source:HGNC Symbol;Acc:HGNC:168] |
| ENSG00000115091 | HGLibA_00660 | 21 | -0.944741289 | ACTR3 | ARP3 actin related protein 3 homolog [Source:HGNC Symbol;Acc:HGNC:170] |
| ENSG00000115318 | HGLibA_27044 | 21 | -0.944741289 | LOXL3 | lysyl oxidase like 3 [Source:HGNC Symbol;Acc:HGNC:13869] |
| ENSG00000115325 | HGLibA_13766 | 21 | -0.944741289 | DOK1 | docking protein 1 [Source:HGNC Symbol;Acc:HGNC:2990] |
| ENSG00000116191 | HGLibA_40171 | 21 | -0.944741289 | RALGPS2 | Ral GEF with PH domain and SH3 binding motif 2 [Source:HGNC Symbol;Acc:HGNC:30279] |
| ENSG00000116774 | HGLibA_33365 | 21 | -0.944741289 | OLFML3 | olfactomedin like 3 [Source:HGNC Symbol;Acc:HGNC:24956] |
| ENSG00000120251 | HGLibA_20263 | 21 | -0.944741289 | GRIA2 | glutamate ionotropic receptor AMPA type subunit 2 [Source:HGNC Symbol;Acc:HGNC:4572] |
| ENSG00000121067 | HGLibA_46762 | 21 | -0.944741289 | SPOP | speckle type BTB/POZ protein [Source:HGNC Symbol;Acc:HGNC:11254] |
| ENSG00000121775 | HGLibA_50387 | 21 | -0.944741289 | TMEM39B | transmembrane protein 39B [Source:HGNC Symbol;Acc:HGNC:25510] |
| ENSG00000122068 | HGLibA_18258 | 21 | -0.944741289 | FYTTD1 | forty-two-three domain containing 1 [Source:HGNC Symbol;Acc:HGNC:25407] |
| ENSG00000123892 | HGLibA_39899 | 21 | -0.944741289 | RAB38 | member RAS oncogene family [Source:HGNC Symbol;Acc:HGNC:9776]" |
| ENSG00000125447 | HGLibA_18985 | 21 | -0.944741289 | GGA3 | ARF binding protein 3 [Source:HGNC Symbol;Acc:HGNC:17079]" |
| ENSG00000125810 | HGLibA_08479 | 21 | -0.944741289 | CD93 | CD93 molecule [Source:HGNC Symbol;Acc:HGNC:15855] |
| ENSG00000125871 | HGLibA_29162 | 21 | -0.944741289 | MGME1 | mitochondrial genome maintenance exonuclease 1 [Source:HGNC Symbol;Acc:HGNC:16205] |
| ENSG00000126858 | HGLibA_41202 | 21 | -0.944741289 | RHOT1 | ras homolog family member T1 [Source:HGNC Symbol;Acc:HGNC:21168] |
| ENSG00000126895 | HGLibA_03909 | 21 | -0.944741289 | AVPR2 | arginine vasopressin receptor 2 [Source:HGNC Symbol;Acc:HGNC:897] |
| ENSG00000127527 | HGLibA_15371 | 21 | -0.944741289 | EPS15L1 | epidermal growth factor receptor pathway substrate 15 like 1 [Source:HGNC Symbol;Acc:HGNC:24634] |
| ENSG00000127530 | HGLibA_34465 | 21 | -0.944741289 | OR7C1 | olfactory receptor family 7 subfamily C member 1 [Source:HGNC Symbol;Acc:HGNC:8373] |
| ENSG00000128040 | HGLibA_46691 | 21 | -0.944741289 | SPINK2 | Kazal type 2 [Source:HGNC Symbol;Acc:HGNC:11245]" |
| ENSG00000129187 | HGLibA_12541 | 21 | -0.944741289 | DCTD | dCMP deaminase [Source:HGNC Symbol;Acc:HGNC:2710] |
| ENSG00000129245 | HGLibA_18220 | 21 | -0.944741289 | FXR2 | FMR1 autosomal homolog 2 [Source:HGNC Symbol;Acc:HGNC:4024] |
| ENSG00000129562 | HGLibA_12288 | 21 | -0.944741289 | DAD1 | defender against cell death 1 [Source:HGNC Symbol;Acc:HGNC:2664] |
| ENSG00000131263 | HGLibA_41349 | 21 | -0.944741289 | RLIM | LIM domain interacting [Source:HGNC Symbol;Acc:HGNC:13429]" |
| ENSG00000132259 | HGLibA_10273 | 21 | -0.944741289 | CNGA4 | cyclic nucleotide gated channel alpha 4 [Source:HGNC Symbol;Acc:HGNC:2152] |
| ENSG00000132463 | HGLibA_20380 | 21 | -0.944741289 | GRSF1 | G-rich RNA sequence binding factor 1 [Source:HGNC Symbol;Acc:HGNC:4610] |
| ENSG00000132693 | HGLibA_11215 | 21 | -0.944741289 | CRP | C-reactive protein [Source:HGNC Symbol;Acc:HGNC:2367] |
| ENSG00000133020 | HGLibA_30738 | 21 | -0.944741289 | MYH8 | myosin heavy chain 8 [Source:HGNC Symbol;Acc:HGNC:7578] |
| ENSG00000133028 | HGLibA_43026 | 21 | -0.944741289 | SCO1 | cytochrome c oxidase assembly protein [Source:HGNC Symbol;Acc:HGNC:10603]" |
| ENSG00000134419 | HGLibA_42080 | 21 | -0.944741289 | RPS15A | ribosomal protein S15a [Source:HGNC Symbol;Acc:HGNC:10389] |
| ENSG00000135045 | HGLibA_06729 | 21 | -0.944741289 | C9orf40 | chromosome 9 open reading frame 40 [Source:HGNC Symbol;Acc:HGNC:23433] |
| ENSG00000135222 | HGLibA_11374 | 21 | -0.944741289 | CSN2 | casein beta [Source:HGNC Symbol;Acc:HGNC:2447] |
| ENSG00000135451 | HGLibA_51720 | 21 | -0.944741289 | TROAP | trophinin associated protein [Source:HGNC Symbol;Acc:HGNC:12327] |
| ENSG00000135766 | HGLibA_14639 | 21 | -0.944741289 | EGLN1 | egl-9 family hypoxia inducible factor 1 [Source:HGNC Symbol;Acc:HGNC:1232] |
| ENSG00000136169 | HGLibA_43669 | 21 | -0.944741289 | SETDB2 | SET domain bifurcated 2 [Source:HGNC Symbol;Acc:HGNC:20263] |
| ENSG00000136521 | HGLibA_31520 | 21 | -0.944741289 | NDUFB5 | NADH:ubiquinone oxidoreductase subunit B5 [Source:HGNC Symbol;Acc:HGNC:7700] |
| ENSG00000138642 | HGLibA_21167 | 21 | -0.944741289 | HERC6 | HECT and RLD domain containing E3 ubiquitin protein ligase family member 6 [Source:HGNC Symbol;Acc:HGNC:26072] |
| ENSG00000138760 | HGLibA_42872 | 21 | -0.944741289 | SCARB2 | scavenger receptor class B member 2 [Source:HGNC Symbol;Acc:HGNC:1665] |
| ENSG00000138771 | HGLibA_44092 | 21 | -0.944741289 | SHROOM3 | shroom family member 3 [Source:HGNC Symbol;Acc:HGNC:30422] |
| ENSG00000140307 | HGLibA_20527 | 21 | -0.944741289 | GTF2A2 | general transcription factor IIA subunit 2 [Source:HGNC Symbol;Acc:HGNC:4647] |
| ENSG00000141522 | HGLibA_02794 | 21 | -0.944741289 | ARHGDIA | Rho GDP dissociation inhibitor alpha [Source:HGNC Symbol;Acc:HGNC:678] |
| ENSG00000141627 | HGLibA_14227 | 21 | -0.944741289 | DYM | dymeclin [Source:HGNC Symbol;Acc:HGNC:21317] |
| ENSG00000142039 | HGLibA_07940 | 21 | -0.944741289 | CCDC97 | coiled-coil domain containing 97 [Source:HGNC Symbol;Acc:HGNC:28289] |
| ENSG00000143621 | HGLibA_23213 | 21 | -0.944741289 | ILF2 | interleukin enhancer binding factor 2 [Source:HGNC Symbol;Acc:HGNC:6037] |
| ENSG00000144407 | HGLibA_39373 | 21 | -0.944741289 | PTH2R | parathyroid hormone 2 receptor [Source:HGNC Symbol;Acc:HGNC:9609] |
| ENSG00000146038 | HGLibA_12485 | 21 | -0.944741289 | DCDC2 | doublecortin domain containing 2 [Source:HGNC Symbol;Acc:HGNC:18141] |
| ENSG00000146385 | HGLibA_48134 | 21 | -0.944741289 | TAAR8 | trace amine associated receptor 8 [Source:HGNC Symbol;Acc:HGNC:14964] |
| ENSG00000146755 | HGLibA_51537 | 21 | -0.944741289 | TRIM50 | tripartite motif containing 50 [Source:HGNC Symbol;Acc:HGNC:19017] |
| ENSG00000147437 | HGLibA_19580 | 21 | -0.944741289 | GNRH1 | gonadotropin releasing hormone 1 [Source:HGNC Symbol;Acc:HGNC:4419] |
| ENSG00000147684 | HGLibA_31534 | 21 | -0.944741289 | NDUFB9 | NADH:ubiquinone oxidoreductase subunit B9 [Source:HGNC Symbol;Acc:HGNC:7704] |
| ENSG00000150681 | HGLibA_41074 | 21 | -0.944741289 | RGS18 | regulator of G protein signaling 18 [Source:HGNC Symbol;Acc:HGNC:14261] |
| ENSG00000151150 | HGLibA_01939 | 21 | -0.944741289 | ANK3 | ankyrin 3 [Source:HGNC Symbol;Acc:HGNC:494] |
| ENSG00000151748 | HGLibA_42785 | 21 | -0.944741289 | SAV1 | salvador family WW domain containing protein 1 [Source:HGNC Symbol;Acc:HGNC:17795] |
| ENSG00000152034 | HGLibA_28592 | 21 | -0.944741289 | MCHR2 | melanin concentrating hormone receptor 2 [Source:HGNC Symbol;Acc:HGNC:20867] |
| ENSG00000152583 | HGLibA_46420 | 21 | -0.944741289 | SPARCL1 | SPARC like 1 [Source:HGNC Symbol;Acc:HGNC:11220] |
| ENSG00000153898 | HGLibA_28641 | 21 | -0.944741289 | MCOLN2 | mucolipin 2 [Source:HGNC Symbol;Acc:HGNC:13357] |
| ENSG00000154162 | HGLibA_08639 | 21 | -0.944741289 | CDH12 | cadherin 12 [Source:HGNC Symbol;Acc:HGNC:1751] |
| ENSG00000154438 | HGLibA_03399 | 21 | -0.944741289 | ASZ1 | SAM and basic leucine zipper domain containing 1 [Source:HGNC Symbol;Acc:HGNC:1350]" |
| ENSG00000155761 | HGLibA_46353 | 21 | -0.944741289 | SPAG17 | sperm associated antigen 17 [Source:HGNC Symbol;Acc:HGNC:26620] |
| ENSG00000161849 | HGLibA_25567 | 21 | -0.944741289 | KRT84 | keratin 84 [Source:HGNC Symbol;Acc:HGNC:6461] |
| ENSG00000162378 | HGLibA_57084 | 21 | -0.944741289 | ZYG11B | cell cycle regulator [Source:HGNC Symbol;Acc:HGNC:25820]" |
| ENSG00000162526 | HGLibA_52014 | 21 | -0.944741289 | TSSK3 | testis specific serine kinase 3 [Source:HGNC Symbol;Acc:HGNC:15473] |
| ENSG00000162542 | HGLibA_49791 | 21 | -0.944741289 | TMCO4 | transmembrane and coiled-coil domains 4 [Source:HGNC Symbol;Acc:HGNC:27393] |
| ENSG00000162623 | HGLibA_52487 | 21 | -0.944741289 | TYW3 | tRNA-yW synthesizing protein 3 homolog [Source:HGNC Symbol;Acc:HGNC:24757] |
| ENSG00000162650 | HGLibA_03856 | 21 | -0.944741289 | ATXN7L2 | ataxin 7 like 2 [Source:HGNC Symbol;Acc:HGNC:28713] |
| ENSG00000162779 | HGLibA_03919 | 21 | -0.944741289 | AXDND1 | axonemal dynein light chain domain containing 1 [Source:HGNC Symbol;Acc:HGNC:26564] |
| ENSG00000163132 | HGLibA_30272 | 21 | -0.944741289 | MSX1 | msh homeobox 1 [Source:HGNC Symbol;Acc:HGNC:7391] |
| ENSG00000163563 | HGLibA_29567 | 21 | -0.944741289 | MNDA | myeloid cell nuclear differentiation antigen [Source:HGNC Symbol;Acc:HGNC:7183] |
| ENSG00000163673 | HGLibA_12506 | 21 | -0.944741289 | DCLK3 | doublecortin like kinase 3 [Source:HGNC Symbol;Acc:HGNC:19005] |
| ENSG00000163762 | HGLibA_49693 | 21 | -0.944741289 | TM4SF18 | transmembrane 4 L six family member 18 [Source:HGNC Symbol;Acc:HGNC:25181] |
| ENSG00000164338 | HGLibA_53441 | 21 | -0.944741289 | UTP15 | small subunit processome component [Source:HGNC Symbol;Acc:HGNC:25758]" |
| ENSG00000165626 | HGLibA_04416 | 21 | -0.944741289 | BEND7 | BEN domain containing 7 [Source:HGNC Symbol;Acc:HGNC:23514] |
| ENSG00000166091 | HGLibA_10222 | 21 | -0.944741289 | CMTM5 | CKLF like MARVEL transmembrane domain containing 5 [Source:HGNC Symbol;Acc:HGNC:19176] |
| ENSG00000166145 | HGLibA_46711 | 21 | -0.944741289 | SPINT1 | Kunitz type 1 [Source:HGNC Symbol;Acc:HGNC:11246]" |
| ENSG00000167447 | HGLibA_45711 | 21 | -0.944741289 | SMG8 | nonsense mediated mRNA decay factor [Source:HGNC Symbol;Acc:HGNC:25551]" |
| ENSG00000167460 | HGLibA_51149 | 21 | -0.944741289 | TPM4 | tropomyosin 4 [Source:HGNC Symbol;Acc:HGNC:12013] |
| ENSG00000167716 | HGLibA_54186 | 21 | -0.944741289 | WDR81 | WD repeat domain 81 [Source:HGNC Symbol;Acc:HGNC:26600] |
| ENSG00000167770 | HGLibA_34753 | 21 | -0.944741289 | OTUB1 | ubiquitin aldehyde binding 1 [Source:HGNC Symbol;Acc:HGNC:23077]" |
| ENSG00000168092 | HGLibA_34992 | 21 | -0.944741289 | PAFAH1B2 | platelet activating factor acetylhydrolase 1b catalytic subunit 2 [Source:HGNC Symbol;Acc:HGNC:8575] |
| ENSG00000168675 | HGLibA_26251 | 21 | -0.944741289 | LDLRAD4 | low density lipoprotein receptor class A domain containing 4 [Source:HGNC Symbol;Acc:HGNC:1224] |
| ENSG00000168961 | HGLibA_26381 | 21 | -0.944741289 | LGALS9 | galectin 9 [Source:HGNC Symbol;Acc:HGNC:6570] |
| ENSG00000169598 | HGLibA_13023 | 21 | -0.944741289 | DFFB | DNA fragmentation factor subunit beta [Source:HGNC Symbol;Acc:HGNC:2773] |
| ENSG00000170855 | HGLibA_51387 | 21 | -0.944741289 | TRIAP1 | TP53 regulated inhibitor of apoptosis 1 [Source:HGNC Symbol;Acc:HGNC:26937] |
| ENSG00000171987 | HGLibA_05137 | 21 | -0.944741289 | C11orf40 | chromosome 11 open reading frame 40 [Source:HGNC Symbol;Acc:HGNC:23986] |
| ENSG00000172680 | HGLibA_29681 | 21 | -0.944741289 | MOS | serine/threonine kinase [Source:HGNC Symbol;Acc:HGNC:7199]" |
| ENSG00000172785 | HGLibA_07430 | 21 | -0.944741289 | CBWD1 | COBW domain containing 1 [Source:HGNC Symbol;Acc:HGNC:17134] |
| ENSG00000172889 | HGLibA_14626 | 21 | -0.944741289 | EGFL7 | EGF like domain multiple 7 [Source:HGNC Symbol;Acc:HGNC:20594] |
| ENSG00000173575 | HGLibA_09390 | 21 | -0.944741289 | CHD2 | chromodomain helicase DNA binding protein 2 [Source:HGNC Symbol;Acc:HGNC:1917] |
| ENSG00000174928 | HGLibA_06230 | 21 | -0.944741289 | C3orf33 | chromosome 3 open reading frame 33 [Source:HGNC Symbol;Acc:HGNC:26434] |
| ENSG00000175104 | HGLibA_51265 | 21 | -0.944741289 | TRAF6 | TNF receptor associated factor 6 [Source:HGNC Symbol;Acc:HGNC:12036] |
| ENSG00000175197 | HGLibA_12616 | 21 | -0.944741289 | DDIT3 | DNA damage inducible transcript 3 [Source:HGNC Symbol;Acc:HGNC:2726] |
| ENSG00000175265 | HGLibA_19641 | 21 | -0.944741289 | GOLGA8A | golgin A8 family member A [Source:HGNC Symbol;Acc:HGNC:31972] |
| ENSG00000175426 | HGLibA_35667 | 21 | -0.944741289 | PCSK1 | proprotein convertase subtilisin/kexin type 1 [Source:HGNC Symbol;Acc:HGNC:8743] |
| ENSG00000175906 | HGLibA_02973 | 21 | -0.944741289 | ARL4D | ADP ribosylation factor like GTPase 4D [Source:HGNC Symbol;Acc:HGNC:656] |
| ENSG00000175928 | HGLibA_27471 | 21 | -0.944741289 | LRRN1 | leucine rich repeat neuronal 1 [Source:HGNC Symbol;Acc:HGNC:20980] |
| ENSG00000176994 | HGLibA_45684 | 21 | -0.944741289 | SMCR8 | candidate 8 [Source:HGNC Symbol;Acc:HGNC:17921]" |

| | | | | | |
|---|---|---|---|---|---|
| ENSG00000179133 | HGLibA_05085 | 21 | -0.944741289 | C10orf67 | chromosome 10 open reading frame 67 [Source:HGNC Symbol;Acc:HGNC:28716] |
| ENSG00000182095 | HGLibA_50910 | 21 | -0.944741289 | TNRC18 | trinucleotide repeat containing 18 [Source:HGNC Symbol;Acc:HGNC:11962] |
| ENSG00000182950 | HGLibA_33291 | 21 | -0.944741289 | ODF3L1 | outer dense fiber of sperm tails 3 like 1 [Source:HGNC Symbol;Acc:HGNC:28735] |
| ENSG00000184148 | HGLibA_46828 | 21 | -0.944741289 | SPRR4 | small proline rich protein 4 [Source:HGNC Symbol;Acc:HGNC:23173] |
| ENSG00000185262 | HGLibA_52544 | 21 | -0.944741289 | UBALD2 | UBA like domain containing 2 [Source:HGNC Symbol;Acc:HGNC:28438] |
| ENSG00000185404 | HGLibA_46293 | 21 | -0.944741289 | SP140L | SP140 nuclear body protein like [Source:HGNC Symbol;Acc:HGNC:25105] |
| ENSG00000185905 | HGLibA_05455 | 21 | -0.944741289 | C16orf54 | chromosome 16 open reading frame 54 [Source:HGNC Symbol;Acc:HGNC:26649] |
| ENSG00000186226 | HGLibA_26111 | 21 | -0.944741289 | LCE1E | late cornified envelope 1E [Source:HGNC Symbol;Acc:HGNC:29466] |
| ENSG00000186440 | HGLibA_34435 | 21 | -0.944741289 | OR6P1 | olfactory receptor family 6 subfamily P member 1 [Source:HGNC Symbol;Acc:HGNC:15036] |
| ENSG00000186479 | HGLibA_41107 | 21 | -0.944741289 | RGS7BP | regulator of G protein signaling 7 binding protein [Source:HGNC Symbol;Acc:HGNC:23271] |
| ENSG00000186714 | HGLibA_07858 | 21 | -0.944741289 | CCDC73 | coiled-coil domain containing 73 [Source:HGNC Symbol;Acc:HGNC:23261] |
| ENSG00000186895 | HGLibA_17404 | 21 | -0.944741289 | FGF3 | fibroblast growth factor 3 [Source:HGNC Symbol;Acc:HGNC:3681] |
| ENSG00000186918 | HGLibA_55912 | 21 | -0.944741289 | ZNF395 | zinc finger protein 395 [Source:HGNC Symbol;Acc:HGNC:18737] |
| ENSG00000187122 | HGLibA_45504 | 21 | -0.944741289 | SLIT1 | slit guidance ligand 1 [Source:HGNC Symbol;Acc:HGNC:11085] |
| ENSG00000187689 | HGLibA_01839 | 21 | -0.944741289 | AMTN | amelotin [Source:HGNC Symbol;Acc:HGNC:33188] |
| ENSG00000188167 | HGLibA_50609 | 21 | -0.944741289 | TMPPE | transmembrane protein with metallophosphoesterase domain [Source:HGNC Symbol;Acc:HGNC:33865] |
| ENSG00000188243 | HGLibA_10670 | 21 | -0.944741289 | COMMD6 | COMM domain containing 6 [Source:HGNC Symbol;Acc:HGNC:24015] |
| ENSG00000189057 | HGLibA_16026 | 21 | -0.944741289 | FAM111B | family with sequence similarity 111 member B [Source:HGNC Symbol;Acc:HGNC:24200] |
| ENSG00000189079 | HGLibA_02889 | 21 | -0.944741289 | ARID2 | AT-rich interaction domain 2 [Source:HGNC Symbol;Acc:HGNC:18037] |
| ENSG00000189357 | HGLibA_46500 | 21 | -0.944741289 | SPATA31D4 | SPATA31 subfamily D member 4 [Source:HGNC Symbol;Acc:HGNC:38601] |
| ENSG00000196468 | HGLibA_17378 | 21 | -0.944741289 | FGF16 | fibroblast growth factor 16 [Source:HGNC Symbol;Acc:HGNC:3672] |
| ENSG00000198083 | HGLibA_25851 | 21 | -0.944741289 | KRTAP9-9 | keratin associated protein 9-9 [Source:HGNC Symbol;Acc:HGNC:16773] |
| ENSG00000198521 | HGLibA_55976 | 21 | -0.944741289 | ZNF43 | zinc finger protein 43 [Source:HGNC Symbol;Acc:HGNC:13109] |
| ENSG00000198690 | HGLibA_16827 | 21 | -0.944741289 | FAN1 | FANCD2 and FANCI associated nuclease 1 [Source:HGNC Symbol;Acc:HGNC:29170] |
| ENSG00000198929 | HGLibA_32325 | 21 | -0.944741289 | NOS1AP | nitric oxide synthase 1 adaptor protein [Source:HGNC Symbol;Acc:HGNC:16859] |
| ENSG00000204186 | HGLibA_55062 | 21 | -0.944741289 | ZDBF2 | zinc finger DBF-type containing 2 [Source:HGNC Symbol;Acc:HGNC:29313] |
| ENSG00000204518 | HGLibA_00038 | 21 | -0.944741289 | AADACL4 | arylacetamide deacetylase like 4 [Source:HGNC Symbol;Acc:HGNC:32038] |
| ENSG00000204539 | HGLibA_08888 | 21 | -0.944741289 | CDSN | corneodesmosin [Source:HGNC Symbol;Acc:HGNC:1802] |
| ENSG00000205922 | HGLibA_33399 | 21 | -0.944741289 | ONECUT3 | one cut homeobox 3 [Source:HGNC Symbol;Acc:HGNC:13399] |
| ENSG00000206536 | HGLibA_34316 | 21 | -0.944741289 | OR5K3 | olfactory receptor family 5 subfamily K member 3 [Source:HGNC Symbol;Acc:HGNC:31290] |
| ENSG00000213741 | HGLibA_42135 | 21 | -0.944741289 | RPS29 | ribosomal protein S29 [Source:HGNC Symbol;Acc:HGNC:10419] |
| ENSG00000215915 | HGLibA_03417 | 21 | -0.944741289 | ATAD3C | AAA domain containing 3C [Source:HGNC Symbol;Acc:HGNC:32151]" |
| ENSG00000218891 | HGLibA_56302 | 21 | -0.944741289 | ZNF579 | zinc finger protein 579 [Source:HGNC Symbol;Acc:HGNC:26646] |
| ENSG00000234284 | HGLibA_56889 | 21 | -0.944741289 | ZNF879 | zinc finger protein 879 [Source:HGNC Symbol;Acc:HGNC:37273] |
| ENSG00000239590 | HGLibA_33681 | 21 | -0.944741289 | OR1J4 | olfactory receptor family 1 subfamily J member 4 [Source:HGNC Symbol;Acc:HGNC:8211] |
| ENSG00000239672 | HGLibA_32151 | 21 | -0.944741289 | NME1 | NME/NM23 nucleoside diphosphate kinase 1 [Source:HGNC Symbol;Acc:HGNC:7849] |
| ENSG00000242875 | HGLibA_40614 | 21 | -0.944741289 | RBMY1B | member B [Source:HGNC Symbol;Acc:HGNC:23914]" |
| ENSG00000244094 | HGLibA_46819 | 21 | -0.944741289 | SPRR2F | small proline rich protein 2F [Source:HGNC Symbol;Acc:HGNC:11266] |
| ENSG00000248487 | HGLibA_00247 | 21 | -0.944741289 | ABHD14A | abhydrolase domain containing 14A [Source:HGNC Symbol;Acc:HGNC:24538] |
| ENSG00000266472 | HGLibA_30061 | 21 | -0.944741289 | MRPS21 | mitochondrial ribosomal protein S21 [Source:HGNC Symbol;Acc:HGNC:14046] |
| ENSG00000269556 | HGLibA_50119 | 21 | -0.944741289 | TMEM185A | transmembrane protein 185A [Source:HGNC Symbol;Acc:HGNC:17125] |
| ENSG00000279169 | HGLibA_38291 | 21 | -0.944741289 | PRAMEF13 | PRAME family member 13 [Source:HGNC Symbol;Acc:HGNC:13262] |
| ENSG00000282608 | HGLibA_01018 | 21 | -0.944741289 | ADORA3 | adenosine A3 receptor [Source:HGNC Symbol;Acc:HGNC:268] |
| ENSG00000285437 | HGLibA_37596 | 21 | -0.944741289 | POLR2J3 | RNA polymerase II subunit J3 [Source:HGNC Symbol;Acc:HGNC:33853] |
| GARS | HGLibA_18635 | 21 | -0.944741289 | GARS | - |
| GPR114 | HGLibA_19858 | 21 | -0.944741289 | GPR114 | - |
| hsa-let-7a-3 | HGLibA_57107 | 21 | -0.944741289 | hsa-let-7a-3 | - |
| hsa-mir-1302-6 | HGLibA_57711 | 21 | -0.944741289 | hsa-mir-1302-6 | - |
| hsa-mir-1303 | HGLibA_57729 | 21 | -0.944741289 | hsa-mir-1303 | - |
| hsa-mir-1321 | HGLibA_57761 | 21 | -0.944741289 | hsa-mir-1321 | - |
| hsa-mir-135b | HGLibA_57802 | 21 | -0.944741289 | hsa-mir-135b | - |
| hsa-mir-210 | HGLibA_58212 | 21 | -0.944741289 | hsa-mir-210 | - |
| hsa-mir-297 | HGLibA_58399 | 21 | -0.944741289 | hsa-mir-297 | - |
| hsa-mir-3610 | HGLibA_59074 | 21 | -0.944741289 | hsa-mir-3610 | - |
| hsa-mir-3689c | HGLibA_59323 | 21 | -0.944741289 | hsa-mir-3689c | - |
| hsa-mir-3926-1 | HGLibA_59578 | 21 | -0.944741289 | hsa-mir-3926-1 | - |
| hsa-mir-4269 | HGLibA_59785 | 21 | -0.944741289 | hsa-mir-4269 | - |
| hsa-mir-4275 | HGLibA_59803 | 21 | -0.944741289 | hsa-mir-4275 | - |
| hsa-mir-4278 | HGLibA_59817 | 21 | -0.944741289 | hsa-mir-4278 | - |
| hsa-mir-4285 | HGLibA_59847 | 21 | -0.944741289 | hsa-mir-4285 | - |
| hsa-mir-4324 | HGLibA_60021 | 21 | -0.944741289 | hsa-mir-4324 | - |
| hsa-mir-452 | HGLibA_60530 | 21 | -0.944741289 | hsa-mir-452 | - |
| hsa-mir-4664 | HGLibA_60771 | 21 | -0.944741289 | hsa-mir-4664 | - |
| hsa-mir-545 | HGLibA_61831 | 21 | -0.944741289 | hsa-mir-545 | - |
| hsa-mir-548au | HGLibA_61928 | 21 | -0.944741289 | hsa-mir-548au | - |
| hsa-mir-5591 | HGLibA_62205 | 21 | -0.944741289 | hsa-mir-5591 | - |
| hsa-mir-563 | HGLibA_62217 | 21 | -0.944741289 | hsa-mir-563 | - |
| hsa-mir-5688 | HGLibA_62266 | 21 | -0.944741289 | hsa-mir-5688 | - |
| hsa-mir-5708 | HGLibA_62372 | 21 | -0.944741289 | hsa-mir-5708 | - |
| hsa-mir-6071 | HGLibA_62538 | 21 | -0.944741289 | hsa-mir-6071 | - |
| hsa-mir-6074 | HGLibA_62550 | 21 | -0.944741289 | hsa-mir-6074 | - |
| hsa-mir-6077-1 | HGLibA_62559 | 21 | -0.944741289 | hsa-mir-6077-1 | - |
| hsa-mir-617 | HGLibA_62707 | 21 | -0.944741289 | hsa-mir-617 | - |
| hsa-mir-6768 | HGLibA_63233 | 21 | -0.944741289 | hsa-mir-6768 | - |
| hsa-mir-6816 | HGLibA_63441 | 21 | -0.944741289 | hsa-mir-6816 | - |
| hsa-mir-6855 | HGLibA_63591 | 21 | -0.944741289 | hsa-mir-6855 | - |
| hsa-mir-6856 | HGLibA_63598 | 21 | -0.944741289 | hsa-mir-6856 | - |
| hsa-mir-6882 | HGLibA_63714 | 21 | -0.944741289 | hsa-mir-6882 | - |
| hsa-mir-7113 | HGLibA_63822 | 21 | -0.944741289 | hsa-mir-7113 | - |
| hsa-mir-760 | HGLibA_63899 | 21 | -0.944741289 | hsa-mir-760 | - |
| hsa-mir-891b | HGLibA_64245 | 21 | -0.944741289 | hsa-mir-891b | - |
| KARS | HGLibA_24049 | 21 | -0.944741289 | KARS | - |
| KIAA1211L | HGLibA_24717 | 21 | -0.944741289 | KIAA1211L | - |
| LOC100288255 | HGLibA_26829 | 21 | -0.944741289 | LOC100288255 | - |
| LOC554223 | HGLibA_26955 | 21 | -0.944741289 | LOC554223 | - |
| MTERFD3 | HGLibA_30345 | 21 | -0.944741289 | MTERFD3 | - |
| PPIAL4B | HGLibA_37900 | 21 | -0.944741289 | PPIAL4B | - |
| SOGA2 | HGLibA_46156 | 21 | -0.944741289 | SOGA2 | - |
| SQRDL | HGLibA_46916 | 21 | -0.944741289 | SQRDL | - |
| TMEM2 | HGLibA_50168 | 21 | -0.944741289 | TMEM2 | - |
| WDR96 | HGLibA_54226 | 21 | -0.944741289 | WDR96 | - |
| C11orf74 | HGLibA_05183 | 20 | -0.953786632 | C11orf74 | - |
| C16orf45 | HGLibA_05446 | 20 | -0.953786632 | C16orf45 | - |
| C16orf93 | HGLibA_05504 | 20 | -0.953786632 | C16orf93 | - |
| C1orf64 | HGLibA_05886 | 20 | -0.953786632 | C1orf64 | - |
| C4orf6 | HGLibA_06348 | 20 | -0.953786632 | C4orf6 | - |
| CXorf57 | HGLibA_11947 | 20 | -0.953786632 | CXorf57 | - |
| DGCR14 | HGLibA_13042 | 20 | -0.953786632 | DGCR14 | - |
| DHFRL1 | HGLibA_13106 | 20 | -0.953786632 | DHFRL1 | - |
| ENSG00000039600 | HGLibA_46261 | 20 | -0.953786632 | SOX30 | SRY-box 30 [Source:HGNC Symbol;Acc:HGNC:30635] |
| ENSG00000041357 | HGLibA_39078 | 20 | -0.953786632 | PSMA4 | proteasome subunit alpha 4 [Source:HGNC Symbol;Acc:HGNC:9533] |
| ENSG00000049249 | HGLibA_50792 | 20 | -0.953786632 | TNFRSF9 | TNF receptor superfamily member 9 [Source:HGNC Symbol;Acc:HGNC:11924] |
| ENSG00000064961 | HGLibA_21680 | 20 | -0.953786632 | HMG20B | high mobility group 20B [Source:HGNC Symbol;Acc:HGNC:5002] |
| ENSG00000067191 | HGLibA_06918 | 20 | -0.953786632 | CACNB1 | calcium voltage-gated channel auxiliary subunit beta 1 [Source:HGNC Symbol;Acc:HGNC:1401] |
| ENSG00000069974 | HGLibA_39855 | 20 | -0.953786632 | RAB27A | member RAS oncogene family [Source:HGNC Symbol;Acc:HGNC:9766]" |
| ENSG00000070444 | HGLibA_29576 | 20 | -0.953786632 | MNT | MAX network transcriptional repressor [Source:HGNC Symbol;Acc:HGNC:7188] |
| ENSG00000071859 | HGLibA_16629 | 20 | -0.953786632 | FAM50A | family with sequence similarity 50 member A [Source:HGNC Symbol;Acc:HGNC:18786] |
| ENSG00000074755 | HGLibA_57092 | 20 | -0.953786632 | ZZEF1 | zinc finger ZZ-type and EF-hand domain containing 1 [Source:HGNC Symbol;Acc:HGNC:29027] |
| ENSG00000074935 | HGLibA_52301 | 20 | -0.953786632 | TUBE1 | tubulin epsilon 1 [Source:HGNC Symbol;Acc:HGNC:20775] |
| ENSG00000076924 | HGLibA_54459 | 20 | -0.953786632 | XAB2 | XPA binding protein 2 [Source:HGNC Symbol;Acc:HGNC:14089] |
| ENSG00000081853 | HGLibA_35530 | 20 | -0.953786632 | PCDHGA2 | 2 [Source:HGNC Symbol;Acc:HGNC:8700]" |
| ENSG00000086717 | HGLibA_37867 | 20 | -0.953786632 | PPEF1 | protein phosphatase with EF-hand domain 1 [Source:HGNC Symbol;Acc:HGNC:9243] |
| ENSG00000088280 | HGLibA_03200 | 20 | -0.953786632 | ASAP3 | ankyrin repeat and PH domain 3 [Source:HGNC Symbol;Acc:HGNC:14987]" |
| ENSG00000090263 | HGLibA_30091 | 20 | -0.953786632 | MRPS33 | mitochondrial ribosomal protein S33 [Source:HGNC Symbol;Acc:HGNC:16634] |
| ENSG00000094880 | HGLibA_08525 | 20 | -0.953786632 | CDC23 | cell division cycle 23 [Source:HGNC Symbol;Acc:HGNC:1724] |
| ENSG00000101144 | HGLibA_04617 | 20 | -0.953786632 | BMP7 | bone morphogenetic protein 7 [Source:HGNC Symbol;Acc:HGNC:1074] |
| ENSG00000103044 | HGLibA_20868 | 20 | -0.953786632 | HAS3 | hyaluronan synthase 3 [Source:HGNC Symbol;Acc:HGNC:4820] |
| ENSG00000104866 | HGLibA_38095 | 20 | -0.953786632 | PPP1R37 | protein phosphatase 1 regulatory subunit 37 [Source:HGNC Symbol;Acc:HGNC:27607] |
| ENSG00000106003 | HGLibA_26343 | 20 | -0.953786632 | LFNG | LFNG O-fucosylpeptide 3-beta-N-acetylglucosaminyltransferase [Source:HGNC Symbol;Acc:HGNC:6560] |
| ENSG00000106018 | HGLibA_53670 | 20 | -0.953786632 | VIPR2 | vasoactive intestinal peptide receptor 2 [Source:HGNC Symbol;Acc:HGNC:12695] |
| ENSG00000106397 | HGLibA_37212 | 20 | -0.953786632 | PLOD3 | 2-oxoglutarate 5-dioxygenase 3 [Source:HGNC Symbol;Acc:HGNC:9083]" |
| ENSG00000106789 | HGLibA_10781 | 20 | -0.953786632 | CORO2A | coronin 2A [Source:HGNC Symbol;Acc:HGNC:2255] |
| ENSG00000108702 | HGLibA_07960 | 20 | -0.953786632 | CCL1 | C-C motif chemokine ligand 1 [Source:HGNC Symbol;Acc:HGNC:10609] |
| ENSG00000109625 | HGLibA_11043 | 20 | -0.953786632 | CPZ | carboxypeptidase Z [Source:HGNC Symbol;Acc:HGNC:2333] |
| ENSG00000110025 | HGLibA_46041 | 20 | -0.953786632 | SNX15 | sorting nexin 15 [Source:HGNC Symbol;Acc:HGNC:14978] |
| ENSG00000110108 | HGLibA_49868 | 20 | -0.953786632 | TMEM109 | transmembrane protein 109 [Source:HGNC Symbol;Acc:HGNC:28771] |
| ENSG00000110375 | HGLibA_53073 | 20 | -0.953786632 | UPK2 | uroplakin 2 [Source:HGNC Symbol;Acc:HGNC:12579] |
| ENSG00000110786 | HGLibA_39472 | 20 | -0.953786632 | PTPN5 | non-receptor type 5 [Source:HGNC Symbol;Acc:HGNC:9657]" |
| ENSG00000111652 | HGLibA_10729 | 20 | -0.953786632 | COPS7A | COP9 signalosome subunit 7A [Source:HGNC Symbol;Acc:HGNC:16758] |
| ENSG00000112981 | HGLibA_32165 | 20 | -0.953786632 | NME5 | NME/NM23 family member 5 [Source:HGNC Symbol;Acc:HGNC:7853] |
| ENSG00000114030 | HGLibA_25354 | 20 | -0.953786632 | KPNA1 | karyopherin subunit alpha 1 [Source:HGNC Symbol;Acc:HGNC:6394] |
| ENSG00000114902 | HGLibA_46558 | 20 | -0.953786632 | SPCS1 | signal peptidase complex subunit 1 [Source:HGNC Symbol;Acc:HGNC:23401] |
| ENSG00000115170 | HGLibA_00686 | 20 | -0.953786632 | ACVR1 | activin A receptor type 1 [Source:HGNC Symbol;Acc:HGNC:171] |
| ENSG00000115364 | HGLibA_29919 | 20 | -0.953786632 | MRPL19 | mitochondrial ribosomal protein L19 [Source:HGNC Symbol;Acc:HGNC:14052] |
| ENSG00000116127 | HGLibA_01673 | 20 | -0.953786632 | ALMS1 | centrosome and basal body associated protein [Source:HGNC Symbol;Acc:HGNC:428]" |

| | | | | | |
|---|---|---|---|---|---|
| ENSG00000117834 | HGLibA_45285 | 20 | -0.953786632 | SLC5A9 | solute carrier family 5 member 9 [Source:HGNC Symbol;Acc:HGNC:22146] |
| ENSG00000119969 | HGLibA_21114 | 20 | -0.953786632 | HELLS | lymphoid specific [Source:HGNC Symbol;Acc:HGNC:4861]" |
| ENSG00000120332 | HGLibA_50862 | 20 | -0.953786632 | TNN | tenascin N [Source:HGNC Symbol;Acc:HGNC:22942] |
| ENSG00000120509 | HGLibA_35970 | 20 | -0.953786632 | PDZD11 | PDZ domain containing 11 [Source:HGNC Symbol;Acc:HGNC:28034] |
| ENSG00000122952 | HGLibA_57071 | 20 | -0.953786632 | ZWINT | ZW10 interacting kinetochore protein [Source:HGNC Symbol;Acc:HGNC:13195] |
| ENSG00000127903 | HGLibA_56839 | 20 | -0.953786632 | ZNF835 | zinc finger protein 835 [Source:HGNC Symbol;Acc:HGNC:34332] |
| ENSG00000127980 | HGLibA_36078 | 20 | -0.953786632 | PEX1 | peroxisomal biogenesis factor 1 [Source:HGNC Symbol;Acc:HGNC:8850] |
| ENSG00000128059 | HGLibA_37852 | 20 | -0.953786632 | PPAT | phosphoribosyl pyrophosphate amidotransferase [Source:HGNC Symbol;Acc:HGNC:9238] |
| ENSG00000129673 | HGLibA_00060 | 20 | -0.953786632 | AANAT | aralkylamine N-acetyltransferase [Source:HGNC Symbol;Acc:HGNC:19] |
| ENSG00000130783 | HGLibA_07821 | 20 | -0.953786632 | CCDC62 | coiled-coil domain containing 62 [Source:HGNC Symbol;Acc:HGNC:30723] |
| ENSG00000130822 | HGLibA_37341 | 20 | -0.953786632 | PNCK | pregnancy up-regulated nonubiquitous CaM kinase [Source:HGNC Symbol;Acc:HGNC:13415] |
| ENSG00000132275 | HGLibA_42284 | 20 | -0.953786632 | RRP8 | ribosomal RNA processing 8 [Source:HGNC Symbol;Acc:HGNC:29030] |
| ENSG00000132485 | HGLibA_56974 | 20 | -0.953786632 | ZRANB2 | zinc finger RANBP2-type containing 2 [Source:HGNC Symbol;Acc:HGNC:13058] |
| ENSG00000132510 | HGLibA_24536 | 20 | -0.953786632 | KDM6B | lysine demethylase 6B [Source:HGNC Symbol;Acc:HGNC:29012] |
| ENSG00000134318 | HGLibA_41748 | 20 | -0.953786632 | ROCK2 | Rho associated coiled-coil containing protein kinase 2 [Source:HGNC Symbol;Acc:HGNC:10252] |
| ENSG00000135077 | HGLibA_20902 | 20 | -0.953786632 | HAVCR2 | hepatitis A virus cellular receptor 2 [Source:HGNC Symbol;Acc:HGNC:18437] |
| ENSG00000135245 | HGLibA_21317 | 20 | -0.953786632 | HILPDA | hypoxia inducible lipid droplet associated [Source:HGNC Symbol;Acc:HGNC:28859] |
| ENSG00000135632 | HGLibA_45862 | 20 | -0.953786632 | SMYD5 | SMYD family member 5 [Source:HGNC Symbol;Acc:HGNC:16258] |
| ENSG00000136280 | HGLibA_08044 | 20 | -0.953786632 | CCM2 | CCM2 scaffold protein [Source:HGNC Symbol;Acc:HGNC:21708] |
| ENSG00000136819 | HGLibA_06762 | 20 | -0.953786632 | C9orf78 | chromosome 9 open reading frame 78 [Source:HGNC Symbol;Acc:HGNC:24932] |
| ENSG00000136950 | HGLibA_03095 | 20 | -0.953786632 | ARPC5L | actin related protein 2/3 complex subunit 5 like [Source:HGNC Symbol;Acc:HGNC:23366] |
| ENSG00000138363 | HGLibA_03512 | 20 | -0.953786632 | ATIC | 5-aminoimidazole-4-carboxamide ribonucleotide formyltransferase/IMP cyclohydrolase [Source:HGNC Symbol;Acc:HGNC:794] |
| ENSG00000138382 | HGLibA_29012 | 20 | -0.953786632 | METTL5 | methyltransferase like 5 [Source:HGNC Symbol;Acc:HGNC:25006] |
| ENSG00000141026 | HGLibA_28821 | 20 | -0.953786632 | MED9 | mediator complex subunit 9 [Source:HGNC Symbol;Acc:HGNC:25487] |
| ENSG00000141101 | HGLibA_32232 | 20 | -0.953786632 | NOB1 | NIN1 (RPN12) binding protein 1 homolog [Source:HGNC Symbol;Acc:HGNC:29540] |
| ENSG00000141433 | HGLibA_00934 | 20 | -0.953786632 | ADCYAP1 | adenylate cyclase activating polypeptide 1 [Source:HGNC Symbol;Acc:HGNC:241] |
| ENSG00000141753 | HGLibA_22823 | 20 | -0.953786632 | IGFBP4 | insulin like growth factor binding protein 4 [Source:HGNC Symbol;Acc:HGNC:5473] |
| ENSG00000142002 | HGLibA_13884 | 20 | -0.953786632 | DPP9 | dipeptidyl peptidase 9 [Source:HGNC Symbol;Acc:HGNC:18648] |
| ENSG00000142507 | HGLibA_39116 | 20 | -0.953786632 | PSMB6 | proteasome subunit beta 6 [Source:HGNC Symbol;Acc:HGNC:9543] |
| ENSG00000143198 | HGLibA_29179 | 20 | -0.953786632 | MGST3 | microsomal glutathione S-transferase 3 [Source:HGNC Symbol;Acc:HGNC:7064] |
| ENSG00000143947 | HGLibA_42125 | 20 | -0.953786632 | RPS27A | ribosomal protein S27a [Source:HGNC Symbol;Acc:HGNC:10417] |
| ENSG00000146054 | HGLibA_51596 | 20 | -0.953786632 | TRIM7 | tripartite motif containing 7 [Source:HGNC Symbol;Acc:HGNC:16278] |
| ENSG00000146477 | HGLibA_44582 | 20 | -0.953786632 | SLC22A3 | solute carrier family 22 member 3 [Source:HGNC Symbol;Acc:HGNC:10967] |
| ENSG00000149532 | HGLibA_11015 | 20 | -0.953786632 | CPSF7 | cleavage and polyadenylation specific factor 7 [Source:HGNC Symbol;Acc:HGNC:30098] |
| ENSG00000150991 | HGLibA_52568 | 20 | -0.953786632 | UBC | ubiquitin C [Source:HGNC Symbol;Acc:HGNC:12468] |
| ENSG00000154025 | HGLibA_45255 | 20 | -0.953786632 | SLC5A10 | solute carrier family 5 member 10 [Source:HGNC Symbol;Acc:HGNC:23155] |
| ENSG00000154845 | HGLibA_38203 | 20 | -0.953786632 | PPP4R1 | protein phosphatase 4 regulatory subunit 1 [Source:HGNC Symbol;Acc:HGNC:9320] |
| ENSG00000157020 | HGLibA_43186 | 20 | -0.953786632 | SEC13 | nuclear pore and COPII coat complex component [Source:HGNC Symbol;Acc:HGNC:10697]" |
| ENSG00000157330 | HGLibA_05780 | 20 | -0.953786632 | C1orf158 | chromosome 1 open reading frame 158 [Source:HGNC Symbol;Acc:HGNC:28567] |
| ENSG00000158796 | HGLibA_12780 | 20 | -0.953786632 | DEDD | death effector domain containing [Source:HGNC Symbol;Acc:HGNC:2755] |
| ENSG00000158966 | HGLibA_06873 | 20 | -0.953786632 | CACHD1 | cache domain containing 1 [Source:HGNC Symbol;Acc:HGNC:29314] |
| ENSG00000159596 | HGLibA_50498 | 20 | -0.953786632 | TMEM69 | transmembrane protein 69 [Source:HGNC Symbol;Acc:HGNC:28035] |
| ENSG00000160563 | HGLibA_28793 | 20 | -0.953786632 | MED27 | mediator complex subunit 27 [Source:HGNC Symbol;Acc:HGNC:2377] |
| ENSG00000161654 | HGLibA_27526 | 20 | -0.953786632 | LSM12 | LSM12 homolog [Source:HGNC Symbol;Acc:HGNC:26407] |
| ENSG00000162104 | HGLibA_00933 | 20 | -0.953786632 | ADCY9 | adenylate cyclase 9 [Source:HGNC Symbol;Acc:HGNC:240] |
| ENSG00000163216 | HGLibA_46815 | 20 | -0.953786632 | SPRR2D | small proline rich protein 2D [Source:HGNC Symbol;Acc:HGNC:11264] |
| ENSG00000163812 | HGLibA_55114 | 20 | -0.953786632 | ZDHHC3 | zinc finger DHHC-type containing 3 [Source:HGNC Symbol;Acc:HGNC:18470] |
| ENSG00000164414 | HGLibA_44946 | 20 | -0.953786632 | SLC35A1 | solute carrier family 35 member A1 [Source:HGNC Symbol;Acc:HGNC:11021] |
| ENSG00000165025 | HGLibA_47949 | 20 | -0.953786632 | SYK | spleen associated tyrosine kinase [Source:HGNC Symbol;Acc:HGNC:11491] |
| ENSG00000165186 | HGLibA_39273 | 20 | -0.953786632 | PTCHD1 | patched domain containing 1 [Source:HGNC Symbol;Acc:HGNC:26392] |
| ENSG00000165409 | HGLibA_51860 | 20 | -0.953786632 | TSHR | thyroid stimulating hormone receptor [Source:HGNC Symbol;Acc:HGNC:12373] |
| ENSG00000165669 | HGLibA_16420 | 20 | -0.953786632 | FAM204A | family with sequence similarity 204 member A [Source:HGNC Symbol;Acc:HGNC:25794] |
| ENSG00000165672 | HGLibA_38413 | 20 | -0.953786632 | PRDX3 | peroxiredoxin 3 [Source:HGNC Symbol;Acc:HGNC:9354] |
| ENSG00000165806 | HGLibA_07323 | 20 | -0.953786632 | CASP7 | caspase 7 [Source:HGNC Symbol;Acc:HGNC:1508] |
| ENSG00000166145 | HGLibA_46713 | 20 | -0.953786632 | SPINT1 | Kunitz type 1 [Source:HGNC Symbol;Acc:HGNC:11246]" |
| ENSG00000166292 | HGLibA_49839 | 20 | -0.953786632 | TMEM100 | transmembrane protein 100 [Source:HGNC Symbol;Acc:HGNC:25607] |
| ENSG00000167700 | HGLibA_29099 | 20 | -0.953786632 | MFSD3 | major facilitator superfamily domain containing 3 [Source:HGNC Symbol;Acc:HGNC:25157] |
| ENSG00000167757 | HGLibA_25238 | 20 | -0.953786632 | KLK11 | kallikrein related peptidase 11 [Source:HGNC Symbol;Acc:HGNC:6359] |
| ENSG00000168297 | HGLibA_39656 | 20 | -0.953786632 | PXK | PX domain containing serine/threonine kinase like [Source:HGNC Symbol;Acc:HGNC:23326] |
| ENSG00000168970 | HGLibA_23963 | 20 | -0.953786632 | JMJD7-PLA2G4B | JMJD7-PLA2G4B readthrough [Source:HGNC Symbol;Acc:HGNC:34449] |
| ENSG00000170017 | HGLibA_01533 | 20 | -0.953786632 | ALCAM | activated leukocyte cell adhesion molecule [Source:HGNC Symbol;Acc:HGNC:400] |
| ENSG00000170255 | HGLibA_29846 | 20 | -0.953786632 | MRGPRX1 | MAS related GPR family member X1 [Source:HGNC Symbol;Acc:HGNC:17962] |
| ENSG00000170266 | HGLibA_19217 | 20 | -0.953786632 | GLB1 | galactosidase beta 1 [Source:HGNC Symbol;Acc:HGNC:4298] |
| ENSG00000170542 | HGLibA_43569 | 20 | -0.953786632 | SERPINB9 | serpin family B member 9 [Source:HGNC Symbol;Acc:HGNC:8955] |
| ENSG00000170577 | HGLibA_44245 | 20 | -0.953786632 | SIX2 | SIX homeobox 2 [Source:HGNC Symbol;Acc:HGNC:10888] |
| ENSG00000170906 | HGLibA_31456 | 20 | -0.953786632 | NDUFA3 | NADH:ubiquinone oxidoreductase subunit A3 [Source:HGNC Symbol;Acc:HGNC:7686] |
| ENSG00000171448 | HGLibA_54854 | 20 | -0.953786632 | ZBTB26 | zinc finger and BTB domain containing 26 [Source:HGNC Symbol;Acc:HGNC:23383] |
| ENSG00000172551 | HGLibA_30559 | 20 | -0.953786632 | MUCL1 | mucin like 1 [Source:HGNC Symbol;Acc:HGNC:30588] |
| ENSG00000174562 | HGLibA_25249 | 20 | -0.953786632 | KLK15 | kallikrein related peptidase 15 [Source:HGNC Symbol;Acc:HGNC:20453] |
| ENSG00000176208 | HGLibA_03420 | 20 | -0.953786632 | ATAD5 | AAA domain containing 5 [Source:HGNC Symbol;Acc:HGNC:25752]" |
| ENSG00000176771 | HGLibA_31316 | 20 | -0.953786632 | NCKAP5 | NCK associated protein 5 [Source:HGNC Symbol;Acc:HGNC:29847] |
| ENSG00000176895 | HGLibA_34080 | 20 | -0.953786632 | OR51A7 | olfactory receptor family 51 subfamily A member 7 [Source:HGNC Symbol;Acc:HGNC:15188] |
| ENSG00000177479 | HGLibA_02914 | 20 | -0.953786632 | ARIH2 | ariadne RBR E3 ubiquitin protein ligase 2 [Source:HGNC Symbol;Acc:HGNC:690] |
| ENSG00000178342 | HGLibA_24200 | 20 | -0.953786632 | KCNG2 | potassium voltage-gated channel modifier subfamily G member 2 [Source:HGNC Symbol;Acc:HGNC:6249] |
| ENSG00000179476 | HGLibA_05359 | 20 | -0.953786632 | C14orf28 | chromosome 14 open reading frame 28 [Source:HGNC Symbol;Acc:HGNC:19834] |
| ENSG00000179869 | HGLibA_00104 | 20 | -0.953786632 | ABCA13 | ATP binding cassette subfamily A member 13 [Source:HGNC Symbol;Acc:HGNC:14638] |
| ENSG00000180104 | HGLibA_15738 | 20 | -0.953786632 | EXOC3 | exocyst complex component 3 [Source:HGNC Symbol;Acc:HGNC:30378] |
| ENSG00000180386 | HGLibA_25846 | 20 | -0.953786632 | KRTAP9-7 | keratin associated protein 9-7 [Source:HGNC Symbol;Acc:HGNC:18915] |
| ENSG00000180398 | HGLibA_28587 | 20 | -0.953786632 | MCFD2 | multiple coagulation factor deficiency 2 [Source:HGNC Symbol;Acc:HGNC:18451] |
| ENSG00000181693 | HGLibA_34526 | 20 | -0.953786632 | OR8H1 | olfactory receptor family 8 subfamily H member 1 [Source:HGNC Symbol;Acc:HGNC:14824] |
| ENSG00000181803 | HGLibA_34443 | 20 | -0.953786632 | OR6S1 | olfactory receptor family 6 subfamily S member 1 [Source:HGNC Symbol;Acc:HGNC:15363] |
| ENSG00000182545 | HGLibA_41392 | 20 | -0.953786632 | RNASE10 | ribonuclease A family member 10 (inactive) [Source:HGNC Symbol;Acc:HGNC:19275] |
| ENSG00000183035 | HGLibA_12042 | 20 | -0.953786632 | CYLC1 | cylicin 1 [Source:HGNC Symbol;Acc:HGNC:2582] |
| ENSG00000183807 | HGLibA_16225 | 20 | -0.953786632 | FAM162B | family with sequence similarity 162 member B [Source:HGNC Symbol;Acc:HGNC:21549] |
| ENSG00000184292 | HGLibA_48180 | 20 | -0.953786632 | TACSTD2 | tumor associated calcium signal transducer 2 [Source:HGNC Symbol;Acc:HGNC:11530] |
| ENSG00000184313 | HGLibA_29877 | 20 | -0.953786632 | MROH7 | maestro heat like repeat family member 7 [Source:HGNC Symbol;Acc:HGNC:24802] |
| ENSG00000184381 | HGLibA_36921 | 20 | -0.953786632 | PLA2G6 | phospholipase A2 group VI [Source:HGNC Symbol;Acc:HGNC:9039] |
| ENSG00000185271 | HGLibA_25188 | 20 | -0.953786632 | KLHL33 | kelch like family member 33 [Source:HGNC Symbol;Acc:HGNC:31952] |
| ENSG00000185551 | HGLibA_32612 | 20 | -0.953786632 | NR2F2 | nuclear receptor subfamily 2 group F member 2 [Source:HGNC Symbol;Acc:HGNC:7976] |
| ENSG00000185630 | HGLibA_35344 | 20 | -0.953786632 | PBX1 | PBX homeobox 1 [Source:HGNC Symbol;Acc:HGNC:8632] |
| ENSG00000185736 | HGLibA_00876 | 20 | -0.953786632 | ADARB2 | RNA specific B2 (inactive) [Source:HGNC Symbol;Acc:HGNC:227]" |
| ENSG00000185966 | HGLibA_26142 | 20 | -0.953786632 | LCE3E | late cornified envelope 3E [Source:HGNC Symbol;Acc:HGNC:29463] |
| ENSG00000186732 | HGLibA_29772 | 20 | -0.953786632 | MPPED1 | metallophosphoesterase domain containing 1 [Source:HGNC Symbol;Acc:HGNC:1306] |
| ENSG00000186908 | HGLibA_55087 | 20 | -0.953786632 | ZDHHC17 | zinc finger DHHC-type containing 17 [Source:HGNC Symbol;Acc:HGNC:18412] |
| ENSG00000187054 | HGLibA_50611 | 20 | -0.953786632 | TMPRSS11A | transmembrane serine protease 11A [Source:HGNC Symbol;Acc:HGNC:27954] |
| ENSG00000187147 | HGLibA_41639 | 20 | -0.953786632 | RNF220 | ring finger protein 220 [Source:HGNC Symbol;Acc:HGNC:25552] |
| ENSG00000187556 | HGLibA_31102 | 20 | -0.953786632 | NANOS3 | nanos C2HC-type zinc finger 3 [Source:HGNC Symbol;Acc:HGNC:22048] |
| ENSG00000187682 | HGLibA_15411 | 20 | -0.953786632 | ERAS | ES cell expressed Ras [Source:HGNC Symbol;Acc:HGNC:5174] |
| ENSG00000188107 | HGLibA_15829 | 20 | -0.953786632 | EYS | eyes shut homolog [Source:HGNC Symbol;Acc:HGNC:21555] |
| ENSG00000188786 | HGLibA_30349 | 20 | -0.953786632 | MTF1 | metal regulatory transcription factor 1 [Source:HGNC Symbol;Acc:HGNC:7428] |
| ENSG00000188917 | HGLibA_51686 | 20 | -0.953786632 | TRMT2B | tRNA methyltransferase 2 homolog B [Source:HGNC Symbol;Acc:HGNC:25748] |
| ENSG00000196277 | HGLibA_20358 | 20 | -0.953786632 | GRM7 | glutamate metabotropic receptor 7 [Source:HGNC Symbol;Acc:HGNC:4599] |
| ENSG00000196417 | HGLibA_56693 | 20 | -0.953786632 | ZNF765 | zinc finger protein 765 [Source:HGNC Symbol;Acc:HGNC:25092] |
| ENSG00000197245 | HGLibA_16021 | 20 | -0.953786632 | FAM110D | family with sequence similarity 110 member D [Source:HGNC Symbol;Acc:HGNC:25860] |
| ENSG00000197375 | HGLibA_44591 | 20 | -0.953786632 | SLC22A5 | solute carrier family 22 member 5 [Source:HGNC Symbol;Acc:HGNC:10969] |
| ENSG00000197647 | HGLibA_55988 | 20 | -0.953786632 | ZNF433 | zinc finger protein 433 [Source:HGNC Symbol;Acc:HGNC:20811] |
| ENSG00000197938 | HGLibA_34297 | 20 | -0.953786632 | OR5H2 | olfactory receptor family 5 subfamily H member 2 [Source:HGNC Symbol;Acc:HGNC:14752] |
| ENSG00000197951 | HGLibA_56607 | 20 | -0.953786632 | ZNF71 | zinc finger protein 71 [Source:HGNC Symbol;Acc:HGNC:13141] |
| ENSG00000198049 | HGLibA_03907 | 20 | -0.953786632 | AVPR1B | arginine vasopressin receptor 1B [Source:HGNC Symbol;Acc:HGNC:896] |
| ENSG00000198648 | HGLibA_47506 | 20 | -0.953786632 | STK39 | serine/threonine kinase 39 [Source:HGNC Symbol;Acc:HGNC:17717] |
| ENSG00000198768 | HGLibA_02397 | 20 | -0.953786632 | APCDD1L | APC down-regulated 1 like [Source:HGNC Symbol;Acc:HGNC:26892] |
| ENSG00000203668 | HGLibA_09469 | 20 | -0.953786632 | CHML | Rab escort protein 2 [Source:HGNC Symbol;Acc:HGNC:1941]" |
| ENSG00000204304 | HGLibA_35347 | 20 | -0.953786632 | PBX2 | PBX homeobox 2 [Source:HGNC Symbol;Acc:HGNC:8633] |
| ENSG00000204389 | HGLibA_22272 | 20 | -0.953786632 | HSPA1A | heat shock protein family A (Hsp70) member 1A [Source:HGNC Symbol;Acc:HGNC:5232] |
| ENSG00000204479 | HGLibA_38298 | 20 | -0.953786632 | PRAMEF17 | PRAME family member 17 [Source:HGNC Symbol;Acc:HGNC:29485] |
| ENSG00000204941 | HGLibA_39049 | 20 | -0.953786632 | PSG5 | pregnancy specific beta-1-glycoprotein 5 [Source:HGNC Symbol;Acc:HGNC:9522] |
| ENSG00000212807 | HGLibA_33738 | 20 | -0.953786632 | OR2A42 | olfactory receptor family 2 subfamily A member 42 [Source:HGNC Symbol;Acc:HGNC:31230] |
| ENSG00000213171 | HGLibA_26595 | 20 | -0.953786632 | LINGO4 | leucine rich repeat and Ig domain containing 4 [Source:HGNC Symbol;Acc:HGNC:31814] |
| ENSG00000213934 | HGLibA_20924 | 20 | -0.953786632 | HBG1 | hemoglobin subunit gamma 1 [Source:HGNC Symbol;Acc:HGNC:4831] |
| ENSG00000215454 | HGLibA_25610 | 20 | -0.953786632 | KRTAP10-4 | keratin associated protein 10-4 [Source:HGNC Symbol;Acc:HGNC:20521] |
| ENSG00000216649 | HGLibA_18446 | 20 | -0.953786632 | GAGE12E | G antigen 12E [Source:HGNC Symbol;Acc:HGNC:31905] |
| ENSG00000221869 | HGLibA_08963 | 20 | -0.953786632 | CEBPD | CCAAT enhancer binding protein delta [Source:HGNC Symbol;Acc:HGNC:1835] |
| ENSG00000229117 | HGLibA_41985 | 20 | -0.953786632 | RPL41 | ribosomal protein L41 [Source:HGNC Symbol;Acc:HGNC:10354] |
| ENSG00000237541 | HGLibA_21624 | 20 | -0.953786632 | HLA-DQA2 | DQ alpha 2 [Source:HGNC Symbol;Acc:HGNC:4943]" |
| ENSG00000237763 | HGLibA_01842 | 20 | -0.953786632 | AMY1A | alpha 1A (salivary) [Source:HGNC Symbol;Acc:HGNC:474]" |
| ENSG00000242550 | HGLibA_43539 | 20 | -0.953786632 | SERPINB10 | serpin family B member 10 [Source:HGNC Symbol;Acc:HGNC:8942] |
| ENSG00000243716 | HGLibA_32474 | 20 | -0.953786632 | NPIPB5 | nuclear pore complex interacting protein family member B5 [Source:HGNC Symbol;Acc:HGNC:37233] |
| ENSG00000243811 | HGLibA_02484 | 20 | -0.953786632 | APOBEC3D | apolipoprotein B mRNA editing enzyme catalytic subunit 3D [Source:HGNC Symbol;Acc:HGNC:17354] |
| ENSG00000244242 | HGLibA_22640 | 20 | -0.953786632 | IFITM10 | interferon induced transmembrane protein 10 [Source:HGNC Symbol;Acc:HGNC:40022] |
| ENSG00000248483 | HGLibA_37792 | 20 | -0.953786632 | POU5F2 | transcription factor 2 [Source:HGNC Symbol;Acc:HGNC:26367]" |
| ENSG00000250799 | HGLibA_38662 | 20 | -0.953786632 | PRODH2 | proline dehydrogenase 2 [Source:HGNC Symbol;Acc:HGNC:17325] |
| ENSG00000255501 | HGLibA_07231 | 20 | -0.953786632 | CARD18 | caspase recruitment domain family member 18 [Source:HGNC Symbol;Acc:HGNC:28861] |
| ENSG00000269713 | HGLibA_31244 | 20 | -0.953786632 | NBPF9 | NBPF member 9 [Source:HGNC Symbol;Acc:HGNC:31991] |
| ENSG00000271271 | HGLibA_52906 | 20 | -0.953786632 | UGT2A2 | UDP glucuronosyltransferase family 2 member A2 [Source:HGNC Symbol;Acc:HGNC:28183] |
| ENSG00000277556 | HGLibA_33606 | 20 | -0.953786632 | OR13C5 | olfactory receptor family 13 subfamily C member 5 [Source:HGNC Symbol;Acc:HGNC:15100] |

| | | | | | |
|---|---|---|---|---|---|
| ENSG00000278129 | HGLibA_56780 | 20 | -0.953786632 | ZNF8 | zinc finger protein 8 [Source:HGNC Symbol;Acc:HGNC:13154] |
| FAM46B | HGLibA_16598 | 20 | -0.953786632 | FAM46B | - |
| GSTT1 | HGLibA_20496 | 20 | -0.953786632 | GSTT1 | - |
| HIST2H3A | HGLibA_21541 | 20 | -0.953786632 | HIST2H3A | - |
| HN1 | HGLibA_21767 | 20 | -0.953786632 | HN1 | - |
| hsa-mir-105-2 | HGLibA_57182 | 20 | -0.953786632 | hsa-mir-105-2 | - |
| hsa-mir-1255a | HGLibA_57420 | 20 | -0.953786632 | hsa-mir-1255a | - |
| hsa-mir-143 | HGLibA_57836 | 20 | -0.953786632 | hsa-mir-143 | - |
| hsa-mir-1972-1 | HGLibA_58108 | 20 | -0.953786632 | hsa-mir-1972-1 | - |
| hsa-mir-210 | HGLibA_58211 | 20 | -0.953786632 | hsa-mir-210 | - |
| hsa-mir-3179-1 | HGLibA_58790 | 20 | -0.953786632 | hsa-mir-3179-1 | - |
| hsa-mir-3194 | HGLibA_58874 | 20 | -0.953786632 | hsa-mir-3194 | - |
| hsa-mir-326 | HGLibA_58970 | 20 | -0.953786632 | hsa-mir-326 | - |
| hsa-mir-424 | HGLibA_59703 | 20 | -0.953786632 | hsa-mir-424 | - |
| hsa-mir-4437 | HGLibA_60152 | 20 | -0.953786632 | hsa-mir-4437 | - |
| hsa-mir-4440 | HGLibA_60164 | 20 | -0.953786632 | hsa-mir-4440 | - |
| hsa-mir-4477a | HGLibA_60319 | 20 | -0.953786632 | hsa-mir-4477a | - |
| hsa-mir-451b | HGLibA_60529 | 20 | -0.953786632 | hsa-mir-451b | - |
| hsa-mir-4644 | HGLibA_60680 | 20 | -0.953786632 | hsa-mir-4644 | - |
| hsa-mir-4662a | HGLibA_60758 | 20 | -0.953786632 | hsa-mir-4662a | - |
| hsa-mir-4761 | HGLibA_61139 | 20 | -0.953786632 | hsa-mir-4761 | - |
| hsa-mir-4768 | HGLibA_61168 | 20 | -0.953786632 | hsa-mir-4768 | - |
| hsa-mir-497 | HGLibA_61387 | 20 | -0.953786632 | hsa-mir-497 | - |
| hsa-mir-504 | HGLibA_61470 | 20 | -0.953786632 | hsa-mir-504 | - |
| hsa-mir-590 | HGLibA_62459 | 20 | -0.953786632 | hsa-mir-590 | - |
| hsa-mir-627 | HGLibA_62749 | 20 | -0.953786632 | hsa-mir-627 | - |
| hsa-mir-6715b | HGLibA_63023 | 20 | -0.953786632 | hsa-mir-6715b | - |
| hsa-mir-8068 | HGLibA_64109 | 20 | -0.953786632 | hsa-mir-8068 | - |
| JHDM1D | HGLibA_23946 | 20 | -0.953786632 | JHDM1D | - |
| LDOC1L | HGLibA_26258 | 20 | -0.953786632 | LDOC1L | - |
| NIM1 | HGLibA_31933 | 20 | -0.953786632 | NIM1 | - |
| SELT | HGLibA_43313 | 20 | -0.953786632 | SELT | - |
| SLC35E2 | HGLibA_44990 | 20 | -0.953786632 | SLC35E2 | - |
| SSFA2 | HGLibA_47129 | 20 | -0.953786632 | SSFA2 | - |
| TMEM14E | HGLibA_49988 | 20 | -0.953786632 | TMEM14E | - |
| WDR96 | HGLibA_54227 | 20 | -0.953786632 | WDR96 | - |
| AGAP8 | HGLibA_01159 | 19 | -0.962831975 | AGAP8 | - |
| C10orf129 | HGLibA_05051 | 19 | -0.962831975 | C10orf129 | - |
| C19orf82 | HGLibA_05716 | 19 | -0.962831975 | C19orf82 | - |
| C1orf151-NBL1 | HGLibA_05776 | 19 | -0.962831975 | C1orf151-NBL1 | - |
| C1orf233 | HGLibA_05847 | 19 | -0.962831975 | C1orf233 | - |
| C6orf106 | HGLibA_06435 | 19 | -0.962831975 | C6orf106 | - |
| C7orf69 | HGLibA_06580 | 19 | -0.962831975 | C7orf69 | - |
| C8orf42 | HGLibA_06621 | 19 | -0.962831975 | C8orf42 | - |
| DHRSX_X | HGLibA_13156 | 19 | -0.962831975 | DHRSX_X | - |
| EFCAB4A | HGLibA_14542 | 19 | -0.962831975 | EFCAB4A | - |
| ENSG00000011009 | HGLibA_27740 | 19 | -0.962831975 | LYPLA2 | lysophospholipase II [Source:HGNC Symbol;Acc:HGNC:6738] |
| ENSG00000011376 | HGLibA_26050 | 19 | -0.962831975 | LARS2 | mitochondrial [Source:HGNC Symbol;Acc:HGNC:17095]" |
| ENSG00000025039 | HGLibA_42234 | 19 | -0.962831975 | RRAGD | Ras related GTP binding D [Source:HGNC Symbol;Acc:HGNC:19903] |
| ENSG00000028137 | HGLibA_50775 | 19 | -0.962831975 | TNFRSF1B | TNF receptor superfamily member 1B [Source:HGNC Symbol;Acc:HGNC:11917] |
| ENSG00000030419 | HGLibA_22933 | 19 | -0.962831975 | IKZF2 | IKAROS family zinc finger 2 [Source:HGNC Symbol;Acc:HGNC:13177] |
| ENSG00000038274 | HGLibA_28420 | 19 | -0.962831975 | MAT2B | methionine adenosyltransferase 2B [Source:HGNC Symbol;Acc:HGNC:6905] |
| ENSG00000063177 | HGLibA_41886 | 19 | -0.962831975 | RPL18 | ribosomal protein L18 [Source:HGNC Symbol;Acc:HGNC:10310] |
| ENSG00000067225 | HGLibA_36832 | 19 | -0.962831975 | PKM | pyruvate kinase M1/2 [Source:HGNC Symbol;Acc:HGNC:9021] |
| ENSG00000070610 | HGLibA_18714 | 19 | -0.962831975 | GBA2 | glucosylceramidase beta 2 [Source:HGNC Symbol;Acc:HGNC:18986] |
| ENSG00000077238 | HGLibA_23173 | 19 | -0.962831975 | IL4R | interleukin 4 receptor [Source:HGNC Symbol;Acc:HGNC:6015] |
| ENSG00000078699 | HGLibA_07385 | 19 | -0.962831975 | CBFA2T2 | CBFA2/RUNX1 translocation partner 2 [Source:HGNC Symbol;Acc:HGNC:1536] |
| ENSG00000086967 | HGLibA_30653 | 19 | -0.962831975 | MYBPC2 | fast type [Source:HGNC Symbol;Acc:HGNC:7550]" |
| ENSG00000088320 | HGLibA_40829 | 19 | -0.962831975 | REM1 | RRAD and GEM like GTPase 1 [Source:HGNC Symbol;Acc:HGNC:15922] |
| ENSG00000089163 | HGLibA_44225 | 19 | -0.962831975 | SIRT4 | sirtuin 4 [Source:HGNC Symbol;Acc:HGNC:14932] |
| ENSG00000095906 | HGLibA_32905 | 19 | -0.962831975 | NUBP2 | nucleotide binding protein 2 [Source:HGNC Symbol;Acc:HGNC:8042] |
| ENSG00000099326 | HGLibA_30937 | 19 | -0.962831975 | MZF1 | myeloid zinc finger 1 [Source:HGNC Symbol;Acc:HGNC:13108] |
| ENSG00000100941 | HGLibA_37392 | 19 | -0.962831975 | PNN | desmosome associated protein [Source:HGNC Symbol;Acc:HGNC:9162]" |
| ENSG00000101463 | HGLibA_47969 | 19 | -0.962831975 | SYNDIG1 | synapse differentiation inducing 1 [Source:HGNC Symbol;Acc:HGNC:15885] |
| ENSG00000101746 | HGLibA_32268 | 19 | -0.962831975 | NOL4 | nucleolar protein 4 [Source:HGNC Symbol;Acc:HGNC:7870] |
| ENSG00000102409 | HGLibA_04447 | 19 | -0.962831975 | BEX4 | brain expressed X-linked 4 [Source:HGNC Symbol;Acc:HGNC:25475] |
| ENSG00000103342 | HGLibA_20440 | 19 | -0.962831975 | GSPT1 | G1 to S phase transition 1 [Source:HGNC Symbol;Acc:HGNC:4621] |
| ENSG00000106290 | HGLibA_48245 | 19 | -0.962831975 | TAF6 | TATA-box binding protein associated factor 6 [Source:HGNC Symbol;Acc:HGNC:11540] |
| ENSG00000106367 | HGLibA_02295 | 19 | -0.962831975 | AP1S1 | adaptor related protein complex 1 subunit sigma 1 [Source:HGNC Symbol;Acc:HGNC:559] |
| ENSG00000106384 | HGLibA_29629 | 19 | -0.962831975 | MOGAT3 | monoacylglycerol O-acyltransferase 3 [Source:HGNC Symbol;Acc:HGNC:23249] |
| ENSG00000107223 | HGLibA_14464 | 19 | -0.962831975 | EDF1 | endothelial differentiation related factor 1 [Source:HGNC Symbol;Acc:HGNC:3164] |
| ENSG00000107566 | HGLibA_15494 | 19 | -0.962831975 | ERLIN1 | ER lipid raft associated 1 [Source:HGNC Symbol;Acc:HGNC:16947] |
| ENSG00000108823 | HGLibA_43796 | 19 | -0.962831975 | SGCA | sarcoglycan alpha [Source:HGNC Symbol;Acc:HGNC:10805] |
| ENSG00000108924 | HGLibA_21653 | 19 | -0.962831975 | HLF | PAR bZIP transcription factor [Source:HGNC Symbol;Acc:HGNC:4977]" |
| ENSG00000109220 | HGLibA_09448 | 19 | -0.962831975 | CHIC2 | cysteine rich hydrophobic domain 2 [Source:HGNC Symbol;Acc:HGNC:1935] |
| ENSG00000112511 | HGLibA_36331 | 19 | -0.962831975 | PHF1 | PHD finger protein 1 [Source:HGNC Symbol;Acc:HGNC:8919] |
| ENSG00000112541 | HGLibA_35760 | 19 | -0.962831975 | PDE10A | phosphodiesterase 10A [Source:HGNC Symbol;Acc:HGNC:8772] |
| ENSG00000114331 | HGLibA_00387 | 19 | -0.962831975 | ACAP2 | ankyrin repeat and PH domains 2 [Source:HGNC Symbol;Acc:HGNC:16469]" |
| ENSG00000115307 | HGLibA_03870 | 19 | -0.962831975 | AUP1 | lipid droplet regulating VLDL assembly factor [Source:HGNC Symbol;Acc:HGNC:891]" |
| ENSG00000115762 | HGLibA_37089 | 19 | -0.962831975 | PLEKHB2 | pleckstrin homology domain containing B2 [Source:HGNC Symbol;Acc:HGNC:19236] |
| ENSG00000117676 | HGLibA_42157 | 19 | -0.962831975 | RPS6KA1 | ribosomal protein S6 kinase A1 [Source:HGNC Symbol;Acc:HGNC:10430] |
| ENSG00000118729 | HGLibA_07338 | 19 | -0.962831975 | CASQ2 | calsequestrin 2 [Source:HGNC Symbol;Acc:HGNC:1513] |
| ENSG00000119596 | HGLibA_54696 | 19 | -0.962831975 | YLPM1 | YLP motif containing 1 [Source:HGNC Symbol;Acc:HGNC:17798] |
| ENSG00000120658 | HGLibA_15157 | 19 | -0.962831975 | ENOX1 | ecto-NOX disulfide-thiol exchanger 1 [Source:HGNC Symbol;Acc:HGNC:25474] |
| ENSG00000121552 | HGLibA_11476 | 19 | -0.962831975 | CSTA | cystatin A [Source:HGNC Symbol;Acc:HGNC:2481] |
| ENSG00000122223 | HGLibA_08306 | 19 | -0.962831975 | CD244 | CD244 molecule [Source:HGNC Symbol;Acc:HGNC:18171] |
| ENSG00000122257 | HGLibA_40435 | 19 | -0.962831975 | RBBP6 | ubiquitin ligase [Source:HGNC Symbol;Acc:HGNC:9889]" |
| ENSG00000122481 | HGLibA_42489 | 19 | -0.962831975 | RWDD3 | RWD domain containing 3 [Source:HGNC Symbol;Acc:HGNC:21393] |
| ENSG00000123307 | HGLibA_31740 | 19 | -0.962831975 | NEUROD4 | neuronal differentiation 4 [Source:HGNC Symbol;Acc:HGNC:13802] |
| ENSG00000123560 | HGLibA_37216 | 19 | -0.962831975 | PLP1 | proteolipid protein 1 [Source:HGNC Symbol;Acc:HGNC:9086] |
| ENSG00000125046 | HGLibA_47193 | 19 | -0.962831975 | SSUH2 | ssu-2 homolog (C. elegans) [Source:HGNC Symbol;Acc:HGNC:24809] |
| ENSG00000125630 | HGLibA_37553 | 19 | -0.962831975 | POLR1B | RNA polymerase I subunit B [Source:HGNC Symbol;Acc:HGNC:20454] |
| ENSG00000125835 | HGLibA_45967 | 19 | -0.962831975 | SNRPB | small nuclear ribonucleoprotein polypeptides B and B1 [Source:HGNC Symbol;Acc:HGNC:11153] |
| ENSG00000127325 | HGLibA_04425 | 19 | -0.962831975 | BEST3 | bestrophin 3 [Source:HGNC Symbol;Acc:HGNC:17105] |
| ENSG00000128283 | HGLibA_08565 | 19 | -0.962831975 | CDC42EP1 | CDC42 effector protein 1 [Source:HGNC Symbol;Acc:HGNC:17014] |
| ENSG00000129221 | HGLibA_01363 | 19 | -0.962831975 | AIPL1 | aryl hydrocarbon receptor interacting protein like 1 [Source:HGNC Symbol;Acc:HGNC:359] |
| ENSG00000129226 | HGLibA_08429 | 19 | -0.962831975 | CD68 | CD68 molecule [Source:HGNC Symbol;Acc:HGNC:1693] |
| ENSG00000130164 | HGLibA_26237 | 19 | -0.962831975 | LDLR | low density lipoprotein receptor [Source:HGNC Symbol;Acc:HGNC:6547] |
| ENSG00000130733 | HGLibA_54673 | 19 | -0.962831975 | YIPF2 | Yip1 domain family member 2 [Source:HGNC Symbol;Acc:HGNC:28476] |
| ENSG00000131408 | HGLibA_32580 | 19 | -0.962831975 | NR1H2 | nuclear receptor subfamily 1 group H member 2 [Source:HGNC Symbol;Acc:HGNC:7965] |
| ENSG00000132600 | HGLibA_38635 | 19 | -0.962831975 | PRMT7 | protein arginine methyltransferase 7 [Source:HGNC Symbol;Acc:HGNC:25557] |
| ENSG00000132846 | HGLibA_54797 | 19 | -0.962831975 | ZBED3 | zinc finger BED-type containing 3 [Source:HGNC Symbol;Acc:HGNC:20711] |
| ENSG00000133816 | HGLibA_29205 | 19 | -0.962831975 | MICAL2 | calponin and LIM domain containing 2 [Source:HGNC Symbol;Acc:HGNC:24693]" |
| ENSG00000134317 | HGLibA_20249 | 19 | -0.962831975 | GRHL1 | grainyhead like transcription factor 1 [Source:HGNC Symbol;Acc:HGNC:17923] |
| ENSG00000134389 | HGLibA_09274 | 19 | -0.962831975 | CFHR5 | complement factor H related 5 [Source:HGNC Symbol;Acc:HGNC:24668] |
| ENSG00000134480 | HGLibA_08098 | 19 | -0.962831975 | CCNH | cyclin H [Source:HGNC Symbol;Acc:HGNC:1594] |
| ENSG00000134899 | HGLibA_15442 | 19 | -0.962831975 | ERCC5 | endonuclease [Source:HGNC Symbol;Acc:HGNC:3437]" |
| ENSG00000135443 | HGLibA_25570 | 19 | -0.962831975 | KRT85 | keratin 85 [Source:HGNC Symbol;Acc:HGNC:6462] |
| ENSG00000136888 | HGLibA_03756 | 19 | -0.962831975 | ATP6V1G1 | ATPase H+ transporting V1 subunit G1 [Source:HGNC Symbol;Acc:HGNC:864] |
| ENSG00000137204 | HGLibA_44596 | 19 | -0.962831975 | SLC22A7 | solute carrier family 22 member 7 [Source:HGNC Symbol;Acc:HGNC:10971] |
| ENSG00000138190 | HGLibA_15754 | 19 | -0.962831975 | EXOC6 | exocyst complex component 6 [Source:HGNC Symbol;Acc:HGNC:23196] |
| ENSG00000138798 | HGLibA_14621 | 19 | -0.962831975 | EGF | epidermal growth factor [Source:HGNC Symbol;Acc:HGNC:3229] |
| ENSG00000139620 | HGLibA_24043 | 19 | -0.962831975 | KANSL2 | KAT8 regulatory NSL complex subunit 2 [Source:HGNC Symbol;Acc:HGNC:26024] |
| ENSG00000140961 | HGLibA_34687 | 19 | -0.962831975 | OSGIN1 | oxidative stress induced growth inhibitor 1 [Source:HGNC Symbol;Acc:HGNC:30093] |
| ENSG00000141404 | HGLibA_19461 | 19 | -0.962831975 | GNAL | G protein subunit alpha L [Source:HGNC Symbol;Acc:HGNC:4388] |
| ENSG00000141433 | HGLibA_00935 | 19 | -0.962831975 | ADCYAP1 | adenylate cyclase activating polypeptide 1 [Source:HGNC Symbol;Acc:HGNC:241] |
| ENSG00000143033 | HGLibA_30351 | 19 | -0.962831975 | MTF2 | metal response element binding transcription factor 2 [Source:HGNC Symbol;Acc:HGNC:29535] |
| ENSG00000143199 | HGLibA_00907 | 19 | -0.962831975 | ADCY10 | adenylate cyclase 10 [Source:HGNC Symbol;Acc:HGNC:21285] |
| ENSG00000143367 | HGLibA_52328 | 19 | -0.962831975 | TUFT1 | tuftelin 1 [Source:HGNC Symbol;Acc:HGNC:12422] |
| ENSG00000143457 | HGLibA_19660 | 19 | -0.962831975 | GOLPH3L | golgi phosphoprotein 3 like [Source:HGNC Symbol;Acc:HGNC:24882] |
| ENSG00000144015 | HGLibA_51502 | 19 | -0.962831975 | TRIM43 | tripartite motif containing 43 [Source:HGNC Symbol;Acc:HGNC:19015] |
| ENSG00000144061 | HGLibA_32445 | 19 | -0.962831975 | NPHP1 | nephrocystin 1 [Source:HGNC Symbol;Acc:HGNC:7905] |
| ENSG00000147421 | HGLibA_21667 | 19 | -0.962831975 | HMBOX1 | homeobox containing 1 [Source:HGNC Symbol;Acc:HGNC:26137] |
| ENSG00000148408 | HGLibA_06879 | 19 | -0.962831975 | CACNA1B | calcium voltage-gated channel subunit alpha1 B [Source:HGNC Symbol;Acc:HGNC:1389] |
| ENSG00000149634 | HGLibA_46458 | 19 | -0.962831975 | SPATA25 | spermatogenesis associated 25 [Source:HGNC Symbol;Acc:HGNC:16158] |
| ENSG00000151778 | HGLibA_43502 | 19 | -0.962831975 | SERP2 | stress associated endoplasmic reticulum protein family member 2 [Source:HGNC Symbol;Acc:HGNC:20607] |
| ENSG00000153495 | HGLibA_49091 | 19 | -0.962831975 | TEX29 | testis expressed 29 [Source:HGNC Symbol;Acc:HGNC:20370] |
| ENSG00000153922 | HGLibA_09384 | 19 | -0.962831975 | CHD1 | chromodomain helicase DNA binding protein 1 [Source:HGNC Symbol;Acc:HGNC:1915] |
| ENSG00000158764 | HGLibA_23825 | 19 | -0.962831975 | ITLN2 | intelectin 2 [Source:HGNC Symbol;Acc:HGNC:20599] |
| ENSG00000159216 | HGLibA_42456 | 19 | -0.962831975 | RUNX1 | runt related transcription factor 1 [Source:HGNC Symbol;Acc:HGNC:10471] |
| ENSG00000159399 | HGLibA_21582 | 19 | -0.962831975 | HK2 | hexokinase 2 [Source:HGNC Symbol;Acc:HGNC:4923] |
| ENSG00000159579 | HGLibA_42356 | 19 | -0.962831975 | RSPRY1 | ring finger and SPRY domain containing 1 [Source:HGNC Symbol;Acc:HGNC:29420] |
| ENSG00000160185 | HGLibA_52557 | 19 | -0.962831975 | UBASH3A | ubiquitin associated and SH3 domain containing A [Source:HGNC Symbol;Acc:HGNC:12462] |
| ENSG00000161513 | HGLibA_17266 | 19 | -0.962831975 | FDXR | ferredoxin reductase [Source:HGNC Symbol;Acc:HGNC:3642] |
| ENSG00000162592 | HGLibA_07741 | 19 | -0.962831975 | CCDC27 | coiled-coil domain containing 27 [Source:HGNC Symbol;Acc:HGNC:26546] |

| | | | | | |
|---|---|---|---|---|---|
| ENSG00000162975 | HGLibA_24194 | 19 | -0.962831975 | KCNF1 | potassium voltage-gated channel modifier subfamily F member 1 [Source:HGNC Symbol;Acc:HGNC:6246] |
| ENSG00000163141 | HGLibA_04663 | 19 | -0.962831975 | BNIPL | BCL2 interacting protein like [Source:HGNC Symbol;Acc:HGNC:16976] |
| ENSG00000163703 | HGLibA_11126 | 19 | -0.962831975 | CRELD1 | cysteine rich with EGF like domains 1 [Source:HGNC Symbol;Acc:HGNC:14630] |
| ENSG00000163704 | HGLibA_38887 | 19 | -0.962831975 | PRRT3 | proline rich transmembrane protein 3 [Source:HGNC Symbol;Acc:HGNC:26591] |
| ENSG00000163812 | HGLibA_55115 | 19 | -0.962831975 | ZDHHC3 | zinc finger DHHC-type containing 3 [Source:HGNC Symbol;Acc:HGNC:18470] |
| ENSG00000164011 | HGLibA_56551 | 19 | -0.962831975 | ZNF691 | zinc finger protein 691 [Source:HGNC Symbol;Acc:HGNC:28028] |
| ENSG00000164049 | HGLibA_17140 | 19 | -0.962831975 | FBXW12 | F-box and WD repeat domain containing 12 [Source:HGNC Symbol;Acc:HGNC:20729] |
| ENSG00000164363 | HGLibA_45312 | 19 | -0.962831975 | SLC6A18 | solute carrier family 6 member 18 [Source:HGNC Symbol;Acc:HGNC:26441] |
| ENSG00000164600 | HGLibA_31743 | 19 | -0.962831975 | NEUROD6 | neuronal differentiation 6 [Source:HGNC Symbol;Acc:HGNC:13804] |
| ENSG00000164626 | HGLibA_24326 | 19 | -0.962831975 | KCNK5 | potassium two pore domain channel subfamily K member 5 [Source:HGNC Symbol;Acc:HGNC:6280] |
| ENSG00000165209 | HGLibA_47583 | 19 | -0.962831975 | STRBP | spermatid perinuclear RNA binding protein [Source:HGNC Symbol;Acc:HGNC:16462] |
| ENSG00000165283 | HGLibA_47537 | 19 | -0.962831975 | STOML2 | stomatin like 2 [Source:HGNC Symbol;Acc:HGNC:14559] |
| ENSG00000165972 | HGLibA_07770 | 19 | -0.962831975 | CCDC38 | coiled-coil domain containing 38 [Source:HGNC Symbol;Acc:HGNC:26843] |
| ENSG00000166348 | HGLibA_53402 | 19 | -0.962831975 | USP54 | ubiquitin specific peptidase 54 [Source:HGNC Symbol;Acc:HGNC:23513] |
| ENSG00000166946 | HGLibA_08082 | 19 | -0.962831975 | CCNDBP1 | cyclin D1 binding protein 1 [Source:HGNC Symbol;Acc:HGNC:1587] |
| ENSG00000167210 | HGLibA_27034 | 19 | -0.962831975 | LOXHD1 | lipoxygenase homology domains 1 [Source:HGNC Symbol;Acc:HGNC:26521] |
| ENSG00000167280 | HGLibA_15128 | 19 | -0.962831975 | ENGASE | endo-beta-N-acetylglucosaminidase [Source:HGNC Symbol;Acc:HGNC:24622] |
| ENSG00000167550 | HGLibA_41158 | 19 | -0.962831975 | RHEBL1 | RHEB like 1 [Source:HGNC Symbol;Acc:HGNC:21166] |
| ENSG00000168517 | HGLibA_21214 | 19 | -0.962831975 | HEXIM2 | hexamethylene bisacetamide inducible 2 [Source:HGNC Symbol;Acc:HGNC:28591] |
| ENSG00000168903 | HGLibA_04978 | 19 | -0.962831975 | BTNL3 | butyrophilin like 3 [Source:HGNC Symbol;Acc:HGNC:1143] |
| ENSG00000169031 | HGLibA_10573 | 19 | -0.962831975 | COL4A3 | collagen type IV alpha 3 chain [Source:HGNC Symbol;Acc:HGNC:2204] |
| ENSG00000169245 | HGLibA_11844 | 19 | -0.962831975 | CXCL10 | C-X-C motif chemokine ligand 10 [Source:HGNC Symbol;Acc:HGNC:10637] |
| ENSG00000170262 | HGLibA_29807 | 19 | -0.962831975 | MRAP | melanocortin 2 receptor accessory protein [Source:HGNC Symbol;Acc:HGNC:1304] |
| ENSG00000170540 | HGLibA_02987 | 19 | -0.962831975 | ARL6IP1 | ADP ribosylation factor like GTPase 6 interacting protein 1 [Source:HGNC Symbol;Acc:HGNC:697] |
| ENSG00000170892 | HGLibA_51836 | 19 | -0.962831975 | TSEN34 | tRNA splicing endonuclease subunit 34 [Source:HGNC Symbol;Acc:HGNC:15506] |
| ENSG00000170946 | HGLibA_13604 | 19 | -0.962831975 | DNAJC24 | DnaJ heat shock protein family (Hsp40) member C24 [Source:HGNC Symbol;Acc:HGNC:26979] |
| ENSG00000172115 | HGLibA_12029 | 19 | -0.962831975 | CYCS | somatic [Source:HGNC Symbol;Acc:HGNC:19986]" |
| ENSG00000172379 | HGLibA_03064 | 19 | -0.962831975 | ARNT2 | aryl hydrocarbon receptor nuclear translocator 2 [Source:HGNC Symbol;Acc:HGNC:16876] |
| ENSG00000172466 | HGLibA_55656 | 19 | -0.962831975 | ZNF24 | zinc finger protein 24 [Source:HGNC Symbol;Acc:HGNC:13032] |
| ENSG00000172936 | HGLibA_30685 | 19 | -0.962831975 | MYD88 | innate immune signal transduction adaptor [Source:HGNC Symbol;Acc:HGNC:7562]" |
| ENSG00000173848 | HGLibA_31701 | 19 | -0.962831975 | NET1 | neuroepithelial cell transforming 1 [Source:HGNC Symbol;Acc:HGNC:14592] |
| ENSG00000175267 | HGLibA_53901 | 19 | -0.962831975 | VWA3A | von Willebrand factor A domain containing 3A [Source:HGNC Symbol;Acc:HGNC:27088] |
| ENSG00000175467 | HGLibA_42755 | 19 | -0.962831975 | SART1 | recruiter of U4/U6.U5 tri-snRNP [Source:HGNC Symbol;Acc:HGNC:10538]" |
| ENSG00000176087 | HGLibA_44956 | 19 | -0.962831975 | SLC35A4 | solute carrier family 35 member A4 [Source:HGNC Symbol;Acc:HGNC:20753] |
| ENSG00000177542 | HGLibA_44674 | 19 | -0.962831975 | SLC25A22 | solute carrier family 25 member 22 [Source:HGNC Symbol;Acc:HGNC:19954] |
| ENSG00000178149 | HGLibA_12305 | 19 | -0.962831975 | DALRD3 | DALR anticodon binding domain containing 3 [Source:HGNC Symbol;Acc:HGNC:25536] |
| ENSG00000179029 | HGLibA_49863 | 19 | -0.962831975 | TMEM107 | transmembrane protein 107 [Source:HGNC Symbol;Acc:HGNC:28128] |
| ENSG00000180098 | HGLibA_51706 | 19 | -0.962831975 | TRNAU1AP | tRNA selenocysteine 1 associated protein 1 [Source:HGNC Symbol;Acc:HGNC:30813] |
| ENSG00000180773 | HGLibA_45039 | 19 | -0.962831975 | SLC36A4 | solute carrier family 36 member 4 [Source:HGNC Symbol;Acc:HGNC:19660] |
| ENSG00000181090 | HGLibA_14683 | 19 | -0.962831975 | EHMT1 | euchromatic histone lysine methyltransferase 1 [Source:HGNC Symbol;Acc:HGNC:24650] |
| ENSG00000181961 | HGLibA_33925 | 19 | -0.962831975 | OR4A16 | olfactory receptor family 4 subfamily A member 16 [Source:HGNC Symbol;Acc:HGNC:15153] |
| ENSG00000182156 | HGLibA_15184 | 19 | -0.962831975 | ENPP7 | ectonucleotide pyrophosphatase/phosphodiesterase 7 [Source:HGNC Symbol;Acc:HGNC:23764] |
| ENSG00000182327 | HGLibA_19348 | 19 | -0.962831975 | GLTPD2 | glycolipid transfer protein domain containing 2 [Source:HGNC Symbol;Acc:HGNC:33756] |
| ENSG00000182379 | HGLibA_33171 | 19 | -0.962831975 | NXPH4 | neurexophilin 4 [Source:HGNC Symbol;Acc:HGNC:8078] |
| ENSG00000182606 | HGLibA_51277 | 19 | -0.962831975 | TRAK1 | trafficking kinesin protein 1 [Source:HGNC Symbol;Acc:HGNC:29947] |
| ENSG00000182606 | HGLibA_51275 | 19 | -0.962831975 | TRAK1 | trafficking kinesin protein 1 [Source:HGNC Symbol;Acc:HGNC:29947] |
| ENSG00000183379 | HGLibA_47974 | 19 | -0.962831975 | SYNDIG1L | synapse differentiation inducing 1 like [Source:HGNC Symbol;Acc:HGNC:32388] |
| ENSG00000184276 | HGLibA_12842 | 19 | -0.962831975 | DEFB108B | defensin beta 108B [Source:HGNC Symbol;Acc:HGNC:29966] |
| ENSG00000185842 | HGLibA_13481 | 19 | -0.962831975 | DNAH14 | dynein axonemal heavy chain 14 [Source:HGNC Symbol;Acc:HGNC:2945] |
| ENSG00000185982 | HGLibA_12888 | 19 | -0.962831975 | DEFB128 | defensin beta 128 [Source:HGNC Symbol;Acc:HGNC:18106] |
| ENSG00000186106 | HGLibA_02107 | 19 | -0.962831975 | ANKRD46 | ankyrin repeat domain 46 [Source:HGNC Symbol;Acc:HGNC:27229] |
| ENSG00000187997 | HGLibA_05602 | 19 | -0.962831975 | C17orf99 | chromosome 17 open reading frame 99 [Source:HGNC Symbol;Acc:HGNC:34490] |
| ENSG00000188603 | HGLibA_10074 | 19 | -0.962831975 | CLN3 | battenin [Source:HGNC Symbol;Acc:HGNC:2074]" |
| ENSG00000188611 | HGLibA_03187 | 19 | -0.962831975 | ASAH2 | N-acylsphingosine amidohydrolase 2 [Source:HGNC Symbol;Acc:HGNC:18860] |
| ENSG00000189013 | HGLibA_24988 | 19 | -0.962831975 | KIR2DL4 | two Ig domains and long cytoplasmic tail 4 [Source:HGNC Symbol;Acc:HGNC:6332]" |
| ENSG00000189299 | HGLibA_17949 | 19 | -0.962831975 | FOXR2 | forkhead box R2 [Source:HGNC Symbol;Acc:HGNC:30469] |
| ENSG00000196074 | HGLibA_47930 | 19 | -0.962831975 | SYCP2 | synaptonemal complex protein 2 [Source:HGNC Symbol;Acc:HGNC:11490] |
| ENSG00000196091 | HGLibA_30649 | 19 | -0.962831975 | MYBPC1 | slow type [Source:HGNC Symbol;Acc:HGNC:7549]" |
| ENSG00000197217 | HGLibA_15205 | 19 | -0.962831975 | ENTPD4 | ectonucleoside triphosphate diphosphohydrolase 4 [Source:HGNC Symbol;Acc:HGNC:14573] |
| ENSG00000197587 | HGLibA_13395 | 19 | -0.962831975 | DMBX1 | diencephalon/mesencephalon homeobox 1 [Source:HGNC Symbol;Acc:HGNC:19026] |
| ENSG00000197771 | HGLibA_28631 | 19 | -0.962831975 | MCMBP | minichromosome maintenance complex binding protein [Source:HGNC Symbol;Acc:HGNC:25782] |
| ENSG00000198162 | HGLibA_28061 | 19 | -0.962831975 | MAN1A2 | mannosidase alpha class 1A member 2 [Source:HGNC Symbol;Acc:HGNC:6822] |
| ENSG00000204147 | HGLibA_03192 | 19 | -0.962831975 | ASAH2B | N-acylsphingosine amidohydrolase 2B [Source:HGNC Symbol;Acc:HGNC:23456] |
| ENSG00000206043 | HGLibA_05622 | 19 | -0.962831975 | C18orf63 | chromosome 18 open reading frame 63 [Source:HGNC Symbol;Acc:HGNC:40037] |
| ENSG00000228049 | HGLibA_37594 | 19 | -0.962831975 | POLR2J2 | RNA polymerase II subunit J2 [Source:HGNC Symbol;Acc:HGNC:23208] |
| ENSG00000237763 | HGLibA_01840 | 19 | -0.962831975 | AMY1A | alpha 1A (salivary) [Source:HGNC Symbol;Acc:HGNC:474]" |
| ENSG00000244025 | HGLibA_25670 | 19 | -0.962831975 | KRTAP19-3 | keratin associated protein 19-3 [Source:HGNC Symbol;Acc:HGNC:18938] |
| ENSG00000249860 | HGLibA_30484 | 19 | -0.962831975 | MTRNR2L5 | MT-RNR2 like 5 [Source:HGNC Symbol;Acc:HGNC:37162] |
| ENSG00000251493 | HGLibA_17829 | 19 | -0.962831975 | FOXD1 | forkhead box D1 [Source:HGNC Symbol;Acc:HGNC:3802] |
| ENSG00000254858 | HGLibA_29790 | 19 | -0.962831975 | MPV17L2 | MPV17 mitochondrial inner membrane protein like 2 [Source:HGNC Symbol;Acc:HGNC:28177] |
| ENSG00000254986 | HGLibA_13870 | 19 | -0.962831975 | DPP3 | dipeptidyl peptidase 3 [Source:HGNC Symbol;Acc:HGNC:3008] |
| ENSG00000256269 | HGLibA_21669 | 19 | -0.962831975 | HMBS | hydroxymethylbilane synthase [Source:HGNC Symbol;Acc:HGNC:4982] |
| ENSG00000288596 | HGLibA_06623 | 19 | -0.962831975 | C8orf44 | - |
| HIST1H4H | HGLibA_21507 | 19 | -0.962831975 | HIST1H4H | - |
| HRASLS2 | HGLibA_22078 | 19 | -0.962831975 | HRASLS2 | - |
| hsa-mir-1207 | HGLibA_57283 | 19 | -0.962831975 | hsa-mir-1207 | - |
| hsa-mir-137 | HGLibA_57808 | 19 | -0.962831975 | hsa-mir-137 | - |
| hsa-mir-147b | HGLibA_57878 | 19 | -0.962831975 | hsa-mir-147b | - |
| hsa-mir-193b | HGLibA_58078 | 19 | -0.962831975 | hsa-mir-193b | - |
| hsa-mir-3679 | HGLibA_59264 | 19 | -0.962831975 | hsa-mir-3679 | - |
| hsa-mir-378a | HGLibA_59424 | 19 | -0.962831975 | hsa-mir-378a | - |
| hsa-mir-382 | HGLibA_59480 | 19 | -0.962831975 | hsa-mir-382 | - |
| hsa-mir-4301 | HGLibA_59915 | 19 | -0.962831975 | hsa-mir-4301 | - |
| hsa-mir-4493 | HGLibA_60390 | 19 | -0.962831975 | hsa-mir-4493 | - |
| hsa-mir-4729 | HGLibA_61015 | 19 | -0.962831975 | hsa-mir-4729 | - |
| hsa-mir-4756 | HGLibA_61120 | 19 | -0.962831975 | hsa-mir-4756 | - |
| hsa-mir-4765 | HGLibA_61157 | 19 | -0.962831975 | hsa-mir-4765 | - |
| hsa-mir-519b | HGLibA_61717 | 19 | -0.962831975 | hsa-mir-519b | - |
| hsa-mir-548ag-1 | HGLibA_61875 | 19 | -0.962831975 | hsa-mir-548ag-1 | - |
| hsa-mir-548n | HGLibA_62045 | 19 | -0.962831975 | hsa-mir-548n | - |
| hsa-mir-5700 | HGLibA_62337 | 19 | -0.962831975 | hsa-mir-5700 | - |
| hsa-mir-5739 | HGLibA_62386 | 19 | -0.962831975 | hsa-mir-5739 | - |
| hsa-mir-642a | HGLibA_62807 | 19 | -0.962831975 | hsa-mir-642a | - |
| hsa-mir-6499 | HGLibA_62843 | 19 | -0.962831975 | hsa-mir-6499 | - |
| hsa-mir-6723 | HGLibA_63055 | 19 | -0.962831975 | hsa-mir-6723 | - |
| hsa-mir-6753 | HGLibA_63168 | 19 | -0.962831975 | hsa-mir-6753 | - |
| hsa-mir-6762 | HGLibA_63209 | 19 | -0.962831975 | hsa-mir-6762 | - |
| hsa-mir-6780a | HGLibA_63295 | 19 | -0.962831975 | hsa-mir-6780a | - |
| hsa-mir-6795 | HGLibA_63356 | 19 | -0.962831975 | hsa-mir-6795 | - |
| hsa-mir-8053 | HGLibA_64050 | 19 | -0.962831975 | hsa-mir-8053 | - |
| hsa-mir-8062 | HGLibA_64085 | 19 | -0.962831975 | hsa-mir-8062 | - |
| hsa-mir-890 | HGLibA_64239 | 19 | -0.962831975 | hsa-mir-890 | - |
| KIAA0146 | HGLibA_24597 | 19 | -0.962831975 | KIAA0146 | - |
| KIAA1009 | HGLibA_24677 | 19 | -0.962831975 | KIAA1009 | - |
| KIAA1524 | HGLibA_24776 | 19 | -0.962831975 | KIAA1524 | - |
| LOC100132146 | HGLibA_26804 | 19 | -0.962831975 | LOC100132146 | - |
| LOC440563 | HGLibA_26947 | 19 | -0.962831975 | LOC440563 | - |
| OBFC1 | HGLibA_33223 | 19 | -0.962831975 | OBFC1 | - |
| PRHOXNB | HGLibA_38466 | 19 | -0.962831975 | PRHOXNB | - |
| AZI1 | HGLibA_03934 | 18 | -0.971877317 | AZI1 | - |
| C10orf12 | HGLibA_05044 | 18 | -0.971877317 | C10orf12 | - |
| C10orf137 | HGLibA_05057 | 18 | -0.971877317 | C10orf137 | - |
| CCDC132 | HGLibA_07595 | 18 | -0.971877317 | CCDC132 | - |
| ENSG00000004864 | HGLibA_44644 | 18 | -0.971877317 | SLC25A13 | solute carrier family 25 member 13 [Source:HGNC Symbol;Acc:HGNC:10983] |
| ENSG00000008056 | HGLibA_47954 | 18 | -0.971877317 | SYN1 | synapsin I [Source:HGNC Symbol;Acc:HGNC:11494] |
| ENSG00000008311 | HGLibA_00083 | 18 | -0.971877317 | AASS | aminoadipate-semialdehyde synthase [Source:HGNC Symbol;Acc:HGNC:17366] |
| ENSG00000010278 | HGLibA_08476 | 18 | -0.971877317 | CD9 | CD9 molecule [Source:HGNC Symbol;Acc:HGNC:1709] |
| ENSG00000036672 | HGLibA_53296 | 18 | -0.971877317 | USP2 | ubiquitin specific peptidase 2 [Source:HGNC Symbol;Acc:HGNC:12618] |
| ENSG00000037042 | HGLibA_52305 | 18 | -0.971877317 | TUBG2 | tubulin gamma 2 [Source:HGNC Symbol;Acc:HGNC:12419] |
| ENSG00000042429 | HGLibA_28764 | 18 | -0.971877317 | MED17 | mediator complex subunit 17 [Source:HGNC Symbol;Acc:HGNC:2375] |
| ENSG00000047597 | HGLibA_54516 | 18 | -0.971877317 | XK | X-linked Kx blood group [Source:HGNC Symbol;Acc:HGNC:12811] |
| ENSG00000048405 | HGLibA_56786 | 18 | -0.971877317 | ZNF800 | zinc finger protein 800 [Source:HGNC Symbol;Acc:HGNC:27267] |
| ENSG00000056291 | HGLibA_32441 | 18 | -0.971877317 | NPFFR2 | neuropeptide FF receptor 2 [Source:HGNC Symbol;Acc:HGNC:4525] |
| ENSG00000065243 | HGLibA_36841 | 18 | -0.971877317 | PKN2 | protein kinase N2 [Source:HGNC Symbol;Acc:HGNC:9406] |
| ENSG00000065357 | HGLibA_13058 | 18 | -0.971877317 | DGKA | diacylglycerol kinase alpha [Source:HGNC Symbol;Acc:HGNC:2849] |
| ENSG00000065413 | HGLibA_02101 | 18 | -0.971877317 | ANKRD44 | ankyrin repeat domain 44 [Source:HGNC Symbol;Acc:HGNC:25259] |
| ENSG00000067064 | HGLibA_22554 | 18 | -0.971877317 | IDI1 | isopentenyl-diphosphate delta isomerase 1 [Source:HGNC Symbol;Acc:HGNC:5387] |
| ENSG00000068971 | HGLibA_38174 | 18 | -0.971877317 | PPP2R5B | protein phosphatase 2 regulatory subunit B'beta [Source:HGNC Symbol;Acc:HGNC:9310] |
| ENSG00000072858 | HGLibA_44114 | 18 | -0.971877317 | SIDT1 | SID1 transmembrane family member 1 [Source:HGNC Symbol;Acc:HGNC:25967] |
| ENSG00000073331 | HGLibA_01699 | 18 | -0.971877317 | ALPK1 | alpha kinase 1 [Source:HGNC Symbol;Acc:HGNC:20917] |
| ENSG00000073614 | HGLibA_24522 | 18 | -0.971877317 | KDM5A | lysine demethylase 5A [Source:HGNC Symbol;Acc:HGNC:9886] |
| ENSG00000074855 | HGLibA_02193 | 18 | -0.971877317 | ANO8 | anoctamin 8 [Source:HGNC Symbol;Acc:HGNC:29329] |
| ENSG00000076344 | HGLibA_41054 | 18 | -0.971877317 | RGS11 | regulator of G protein signaling 11 [Source:HGNC Symbol;Acc:HGNC:9993] |
| ENSG00000076513 | HGLibA_02000 | 18 | -0.971877317 | ANKRD13A | ankyrin repeat domain 13A [Source:HGNC Symbol;Acc:HGNC:21268] |
| ENSG00000077800 | HGLibA_17582 | 18 | -0.971877317 | FKBP6 | FK506 binding protein 6 [Source:HGNC Symbol;Acc:HGNC:3722] |

| | | | | | |
|---|---|---|---|---|---|
| ENSG00000077935 | HGLibA_45645 | 18 | -0.971877317 | SMC1B | structural maintenance of chromosomes 1B [Source:HGNC Symbol;Acc:HGNC:11112] |
| ENSG00000083838 | HGLibA_56020 | 18 | -0.971877317 | ZNF446 | zinc finger protein 446 [Source:HGNC Symbol;Acc:HGNC:21036] |
| ENSG00000085982 | HGLibA_53362 | 18 | -0.971877317 | USP40 | ubiquitin specific peptidase 40 [Source:HGNC Symbol;Acc:HGNC:20069] |
| ENSG00000089063 | HGLibA_50256 | 18 | -0.971877317 | TMEM230 | transmembrane protein 230 [Source:HGNC Symbol;Acc:HGNC:15876] |
| ENSG00000092020 | HGLibA_38163 | 18 | -0.971877317 | PPP2R3C | protein phosphatase 2 regulatory subunit B''gamma [Source:HGNC Symbol;Acc:HGNC:17485] |
| ENSG00000095970 | HGLibA_51357 | 18 | -0.971877317 | TREM2 | triggering receptor expressed on myeloid cells 2 [Source:HGNC Symbol;Acc:HGNC:17761] |
| ENSG00000100412 | HGLibA_00463 | 18 | -0.971877317 | ACO2 | aconitase 2 [Source:HGNC Symbol;Acc:HGNC:118] |
| ENSG00000100505 | HGLibA_51619 | 18 | -0.971877317 | TRIM9 | tripartite motif containing 9 [Source:HGNC Symbol;Acc:HGNC:16288] |
| ENSG00000100764 | HGLibA_39126 | 18 | -0.971877317 | PSMC1 | ATPase 1 [Source:HGNC Symbol;Acc:HGNC:9547]" |
| ENSG00000101104 | HGLibA_34923 | 18 | -0.971877317 | PABPC1L | poly(A) binding protein cytoplasmic 1 like [Source:HGNC Symbol;Acc:HGNC:15797] |
| ENSG00000101384 | HGLibA_23905 | 18 | -0.971877317 | JAG1 | jagged 1 [Source:HGNC Symbol;Acc:HGNC:6188] |
| ENSG00000101811 | HGLibA_11487 | 18 | -0.971877317 | CSTF2 | cleavage stimulation factor subunit 2 [Source:HGNC Symbol;Acc:HGNC:2484] |
| ENSG00000102996 | HGLibA_29495 | 18 | -0.971877317 | MMP15 | matrix metallopeptidase 15 [Source:HGNC Symbol;Acc:HGNC:7161] |
| ENSG00000104812 | HGLibA_20709 | 18 | -0.971877317 | GYS1 | glycogen synthase 1 [Source:HGNC Symbol;Acc:HGNC:4706] |
| ENSG00000105538 | HGLibA_40357 | 18 | -0.971877317 | RASIP1 | Ras interacting protein 1 [Source:HGNC Symbol;Acc:HGNC:24716] |
| ENSG00000106245 | HGLibA_05002 | 18 | -0.971877317 | BUD31 | BUD31 homolog [Source:HGNC Symbol;Acc:HGNC:29629] |
| ENSG00000107331 | HGLibA_00106 | 18 | -0.971877317 | ABCA2 | ATP binding cassette subfamily A member 2 [Source:HGNC Symbol;Acc:HGNC:32] |
| ENSG00000107331 | HGLibA_00107 | 18 | -0.971877317 | ABCA2 | ATP binding cassette subfamily A member 2 [Source:HGNC Symbol;Acc:HGNC:32] |
| ENSG00000107679 | HGLibA_37063 | 18 | -0.971877317 | PLEKHA1 | pleckstrin homology domain containing A1 [Source:HGNC Symbol;Acc:HGNC:14335] |
| ENSG00000108417 | HGLibA_25494 | 18 | -0.971877317 | KRT37 | keratin 37 [Source:HGNC Symbol;Acc:HGNC:6455] |
| ENSG00000108509 | HGLibA_07118 | 18 | -0.971877317 | CAMTA2 | calmodulin binding transcription activator 2 [Source:HGNC Symbol;Acc:HGNC:18807] |
| ENSG00000108953 | HGLibA_54743 | 18 | -0.971877317 | YWHAE | tyrosine 3-monooxygenase/tryptophan 5-monooxygenase activation protein epsilon [Source:HGNC Symbol;Acc:HGNC:12851] |
| ENSG00000112186 | HGLibA_07136 | 18 | -0.971877317 | CAP2 | cyclase associated actin cytoskeleton regulatory protein 2 [Source:HGNC Symbol;Acc:HGNC:20039] |
| ENSG00000112494 | HGLibA_53036 | 18 | -0.971877317 | UNC93A | unc-93 homolog A [Source:HGNC Symbol;Acc:HGNC:12570] |
| ENSG00000113141 | HGLibA_22912 | 18 | -0.971877317 | IK | IK cytokine [Source:HGNC Symbol;Acc:HGNC:5958] |
| ENSG00000114279 | HGLibA_17370 | 18 | -0.971877317 | FGF12 | fibroblast growth factor 12 [Source:HGNC Symbol;Acc:HGNC:3668] |
| ENSG00000114982 | HGLibA_24045 | 18 | -0.971877317 | KANSL3 | KAT8 regulatory NSL complex subunit 3 [Source:HGNC Symbol;Acc:HGNC:25473] |
| ENSG00000115947 | HGLibA_34609 | 18 | -0.971877317 | ORC4 | origin recognition complex subunit 4 [Source:HGNC Symbol;Acc:HGNC:8490] |
| ENSG00000116273 | HGLibA_36341 | 18 | -0.971877317 | PHF13 | PHD finger protein 13 [Source:HGNC Symbol;Acc:HGNC:22983] |
| ENSG00000117155 | HGLibA_47204 | 18 | -0.971877317 | SSX2IP | SSX family member 2 interacting protein [Source:HGNC Symbol;Acc:HGNC:16509] |
| ENSG00000119614 | HGLibA_53877 | 18 | -0.971877317 | VSX2 | visual system homeobox 2 [Source:HGNC Symbol;Acc:HGNC:1975] |
| ENSG00000120889 | HGLibA_50737 | 18 | -0.971877317 | TNFRSF10B | TNF receptor superfamily member 10b [Source:HGNC Symbol;Acc:HGNC:11905] |
| ENSG00000121621 | HGLibA_24874 | 18 | -0.971877317 | KIF18A | kinesin family member 18A [Source:HGNC Symbol;Acc:HGNC:29441] |
| ENSG00000121774 | HGLibA_24565 | 18 | -0.971877317 | KHDRBS1 | signal transduction associated 1 [Source:HGNC Symbol;Acc:HGNC:18116]" |
| ENSG00000122641 | HGLibA_23300 | 18 | -0.971877317 | INHBA | inhibin subunit beta A [Source:HGNC Symbol;Acc:HGNC:6066] |
| ENSG00000123095 | HGLibA_04482 | 18 | -0.971877317 | BHLHE41 | basic helix-loop-helix family member e41 [Source:HGNC Symbol;Acc:HGNC:16617] |
| ENSG00000124237 | HGLibA_06029 | 18 | -0.971877317 | C20orf85 | chromosome 20 open reading frame 85 [Source:HGNC Symbol;Acc:HGNC:16216] |
| ENSG00000125107 | HGLibA_10325 | 18 | -0.971877317 | CNOT1 | CCR4-NOT transcription complex subunit 1 [Source:HGNC Symbol;Acc:HGNC:7877] |
| ENSG00000130988 | HGLibA_41019 | 18 | -0.971877317 | RGN | regucalcin [Source:HGNC Symbol;Acc:HGNC:9989] |
| ENSG00000131097 | HGLibA_21309 | 18 | -0.971877317 | HIGD1B | HIG1 hypoxia inducible domain family member 1B [Source:HGNC Symbol;Acc:HGNC:24318] |
| ENSG00000131848 | HGLibA_57028 | 18 | -0.971877317 | ZSCAN5A | zinc finger and SCAN domain containing 5A [Source:HGNC Symbol;Acc:HGNC:23710] |
| ENSG00000132182 | HGLibA_33039 | 18 | -0.971877317 | NUP210 | nucleoporin 210 [Source:HGNC Symbol;Acc:HGNC:30052] |
| ENSG00000134138 | HGLibA_28875 | 18 | -0.971877317 | MEIS2 | Meis homeobox 2 [Source:HGNC Symbol;Acc:HGNC:7001] |
| ENSG00000134200 | HGLibA_51855 | 18 | -0.971877317 | TSHB | thyroid stimulating hormone beta [Source:HGNC Symbol;Acc:HGNC:12372] |
| ENSG00000134343 | HGLibA_02178 | 18 | -0.971877317 | ANO3 | anoctamin 3 [Source:HGNC Symbol;Acc:HGNC:14004] |
| ENSG00000134765 | HGLibA_13989 | 18 | -0.971877317 | DSC1 | desmocollin 1 [Source:HGNC Symbol;Acc:HGNC:3035] |
| ENSG00000134765 | HGLibA_13988 | 18 | -0.971877317 | DSC1 | desmocollin 1 [Source:HGNC Symbol;Acc:HGNC:3035] |
| ENSG00000135069 | HGLibA_39008 | 18 | -0.971877317 | PSAT1 | phosphoserine aminotransferase 1 [Source:HGNC Symbol;Acc:HGNC:19129] |
| ENSG00000135637 | HGLibA_07620 | 18 | -0.971877317 | CCDC142 | coiled-coil domain containing 142 [Source:HGNC Symbol;Acc:HGNC:25889] |
| ENSG00000136158 | HGLibA_46837 | 18 | -0.971877317 | SPRY2 | sprouty RTK signaling antagonist 2 [Source:HGNC Symbol;Acc:HGNC:11270] |
| ENSG00000136404 | HGLibA_49709 | 18 | -0.971877317 | TM6SF1 | transmembrane 6 superfamily member 1 [Source:HGNC Symbol;Acc:HGNC:11860] |
| ENSG00000136938 | HGLibA_02202 | 18 | -0.971877317 | ANP32B | acidic nuclear phosphoprotein 32 family member B [Source:HGNC Symbol;Acc:HGNC:16677] |
| ENSG00000136940 | HGLibA_35747 | 18 | -0.971877317 | PDCL | phosducin like [Source:HGNC Symbol;Acc:HGNC:8770] |
| ENSG00000137707 | HGLibA_04951 | 18 | -0.971877317 | BTG4 | BTG anti-proliferation factor 4 [Source:HGNC Symbol;Acc:HGNC:13862] |
| ENSG00000138039 | HGLibA_26422 | 18 | -0.971877317 | LHCGR | luteinizing hormone/choriogonadotropin receptor [Source:HGNC Symbol;Acc:HGNC:6585] |
| ENSG00000138075 | HGLibA_00225 | 18 | -0.971877317 | ABCG5 | ATP binding cassette subfamily G member 5 [Source:HGNC Symbol;Acc:HGNC:13886] |
| ENSG00000138326 | HGLibA_42114 | 18 | -0.971877317 | RPS24 | ribosomal protein S24 [Source:HGNC Symbol;Acc:HGNC:10411] |
| ENSG00000141384 | HGLibA_48238 | 18 | -0.971877317 | TAF4B | TATA-box binding protein associated factor 4b [Source:HGNC Symbol;Acc:HGNC:11538] |
| ENSG00000141497 | HGLibA_55419 | 18 | -0.971877317 | ZMYND15 | zinc finger MYND-type containing 15 [Source:HGNC Symbol;Acc:HGNC:20997] |
| ENSG00000141542 | HGLibA_39936 | 18 | -0.971877317 | RAB40B | member RAS oncogene family [Source:HGNC Symbol;Acc:HGNC:18284]" |
| ENSG00000141750 | HGLibA_47314 | 18 | -0.971877317 | STAC2 | SH3 and cysteine rich domain 2 [Source:HGNC Symbol;Acc:HGNC:23990] |
| ENSG00000144524 | HGLibA_10730 | 18 | -0.971877317 | COPS7B | COP9 signalosome subunit 7B [Source:HGNC Symbol;Acc:HGNC:16760] |
| ENSG00000144935 | HGLibA_51727 | 18 | -0.971877317 | TRPC1 | transient receptor potential cation channel subfamily C member 1 [Source:HGNC Symbol;Acc:HGNC:12333] |
| ENSG00000145832 | HGLibA_44760 | 18 | -0.971877317 | SLC25A48 | solute carrier family 25 member 48 [Source:HGNC Symbol;Acc:HGNC:30451] |
| ENSG00000147118 | HGLibA_55542 | 18 | -0.971877317 | ZNF182 | zinc finger protein 182 [Source:HGNC Symbol;Acc:HGNC:13001] |
| ENSG00000147124 | HGLibA_55933 | 18 | -0.971877317 | ZNF41 | zinc finger protein 41 [Source:HGNC Symbol;Acc:HGNC:13107] |
| ENSG00000149328 | HGLibA_19222 | 18 | -0.971877317 | GLB1L2 | galactosidase beta 1 like 2 [Source:HGNC Symbol;Acc:HGNC:25129] |
| ENSG00000151414 | HGLibA_31661 | 18 | -0.971877317 | NEK7 | NIMA related kinase 7 [Source:HGNC Symbol;Acc:HGNC:13386] |
| ENSG00000152583 | HGLibA_46419 | 18 | -0.971877317 | SPARCL1 | SPARC like 1 [Source:HGNC Symbol;Acc:HGNC:11220] |
| ENSG00000152592 | HGLibA_13411 | 18 | -0.971877317 | DMP1 | dentin matrix acidic phosphoprotein 1 [Source:HGNC Symbol;Acc:HGNC:2932] |
| ENSG00000156113 | HGLibA_24339 | 18 | -0.971877317 | KCNMA1 | potassium calcium-activated channel subfamily M alpha 1 [Source:HGNC Symbol;Acc:HGNC:6284] |
| ENSG00000157315 | HGLibA_49816 | 18 | -0.971877317 | TMED6 | transmembrane p24 trafficking protein 6 [Source:HGNC Symbol;Acc:HGNC:28331] |
| ENSG00000158169 | HGLibA_16837 | 18 | -0.971877317 | FANCC | FA complementation group C [Source:HGNC Symbol;Acc:HGNC:3584] |
| ENSG00000159131 | HGLibA_18638 | 18 | -0.971877317 | GART | phosphoribosylaminoimidazole synthetase [Source:HGNC Symbol;Acc:HGNC:4163]" |
| ENSG00000159496 | HGLibA_41011 | 18 | -0.971877317 | RGL4 | ral guanine nucleotide dissociation stimulator like 4 [Source:HGNC Symbol;Acc:HGNC:31911] |
| ENSG00000160633 | HGLibA_42635 | 18 | -0.971877317 | SAFB | scaffold attachment factor B [Source:HGNC Symbol;Acc:HGNC:10520] |
| ENSG00000160951 | HGLibA_39312 | 18 | -0.971877317 | PTGER1 | prostaglandin E receptor 1 [Source:HGNC Symbol;Acc:HGNC:9593] |
| ENSG00000162624 | HGLibA_26466 | 18 | -0.971877317 | LHX8 | LIM homeobox 8 [Source:HGNC Symbol;Acc:HGNC:28838] |
| ENSG00000163093 | HGLibA_04222 | 18 | -0.971877317 | BBS5 | Bardet-Biedl syndrome 5 [Source:HGNC Symbol;Acc:HGNC:970] |
| ENSG00000163162 | HGLibA_41540 | 18 | -0.971877317 | RNF149 | ring finger protein 149 [Source:HGNC Symbol;Acc:HGNC:23137] |
| ENSG00000163462 | HGLibA_51514 | 18 | -0.971877317 | TRIM46 | tripartite motif containing 46 [Source:HGNC Symbol;Acc:HGNC:19019] |
| ENSG00000163938 | HGLibA_19550 | 18 | -0.971877317 | GNL3 | G protein nucleolar 3 [Source:HGNC Symbol;Acc:HGNC:29931] |
| ENSG00000164576 | HGLibA_42729 | 18 | -0.971877317 | SAP30L | SAP30 like [Source:HGNC Symbol;Acc:HGNC:25663] |
| ENSG00000165244 | HGLibA_55883 | 18 | -0.971877317 | ZNF367 | zinc finger protein 367 [Source:HGNC Symbol;Acc:HGNC:18320] |
| ENSG00000165583 | HGLibA_47217 | 18 | -0.971877317 | SSX5 | SSX family member 5 [Source:HGNC Symbol;Acc:HGNC:11339] |
| ENSG00000165633 | HGLibA_53869 | 18 | -0.971877317 | VSTM4 | V-set and transmembrane domain containing 4 [Source:HGNC Symbol;Acc:HGNC:26470] |
| ENSG00000165731 | HGLibA_40872 | 18 | -0.971877317 | RET | ret proto-oncogene [Source:HGNC Symbol;Acc:HGNC:9967] |
| ENSG00000166323 | HGLibA_05170 | 18 | -0.971877317 | C11orf65 | chromosome 11 open reading frame 65 [Source:HGNC Symbol;Acc:HGNC:28519] |
| ENSG00000167380 | HGLibA_55623 | 18 | -0.971877317 | ZNF226 | zinc finger protein 226 [Source:HGNC Symbol;Acc:HGNC:13019] |
| ENSG00000167461 | HGLibA_39985 | 18 | -0.971877317 | RAB8A | member RAS oncogene family [Source:HGNC Symbol;Acc:HGNC:7007]" |
| ENSG00000167705 | HGLibA_41251 | 18 | -0.971877317 | RILP | Rab interacting lysosomal protein [Source:HGNC Symbol;Acc:HGNC:30266] |
| ENSG00000168542 | HGLibA_10562 | 18 | -0.971877317 | COL3A1 | collagen type III alpha 1 chain [Source:HGNC Symbol;Acc:HGNC:2201] |
| ENSG00000168803 | HGLibA_00725 | 18 | -0.971877317 | ADAL | adenosine deaminase like [Source:HGNC Symbol;Acc:HGNC:31853] |
| ENSG00000168894 | HGLibA_41583 | 18 | -0.971877317 | RNF181 | ring finger protein 181 [Source:HGNC Symbol;Acc:HGNC:28037] |
| ENSG00000169021 | HGLibA_53120 | 18 | -0.971877317 | UQCRFS1 | Rieske iron-sulfur polypeptide 1 [Source:HGNC Symbol;Acc:HGNC:12587]" |
| ENSG00000169598 | HGLibA_13021 | 18 | -0.971877317 | DFFB | DNA fragmentation factor subunit beta [Source:HGNC Symbol;Acc:HGNC:2773] |
| ENSG00000169760 | HGLibA_32067 | 18 | -0.971877317 | NLGN1 | neuroligin 1 [Source:HGNC Symbol;Acc:HGNC:14291] |
| ENSG00000169840 | HGLibA_20507 | 18 | -0.971877317 | GSX1 | GS homeobox 1 [Source:HGNC Symbol;Acc:HGNC:20374] |
| ENSG00000172365 | HGLibA_34261 | 18 | -0.971877317 | OR5B2 | olfactory receptor family 5 subfamily B member 2 [Source:HGNC Symbol;Acc:HGNC:8323] |
| ENSG00000173575 | HGLibA_09388 | 18 | -0.971877317 | CHD2 | chromodomain helicase DNA binding protein 2 [Source:HGNC Symbol;Acc:HGNC:1917] |
| ENSG00000173610 | HGLibA_52903 | 18 | -0.971877317 | UGT2A1 | UDP glucuronosyltransferase family 2 member A1 complex locus [Source:HGNC Symbol;Acc:HGNC:12542] |
| ENSG00000173868 | HGLibA_36442 | 18 | -0.971877317 | PHOSPHO1 | phosphoethanolamine/phosphocholine phosphatase [Source:HGNC Symbol;Acc:HGNC:16815] |
| ENSG00000174132 | HGLibA_16282 | 18 | -0.971877317 | FAM174A | family with sequence similarity 174 member A [Source:HGNC Symbol;Acc:HGNC:24943] |
| ENSG00000174236 | HGLibA_40842 | 18 | -0.971877317 | REP15 | RAB15 effector protein [Source:HGNC Symbol;Acc:HGNC:33748] |
| ENSG00000174243 | HGLibA_12674 | 18 | -0.971877317 | DDX23 | DEAD-box helicase 23 [Source:HGNC Symbol;Acc:HGNC:17347] |
| ENSG00000174469 | HGLibA_10422 | 18 | -0.971877317 | CNTNAP2 | contactin associated protein like 2 [Source:HGNC Symbol;Acc:HGNC:13830] |
| ENSG00000175189 | HGLibA_23306 | 18 | -0.971877317 | INHBC | inhibin subunit beta C [Source:HGNC Symbol;Acc:HGNC:6068] |
| ENSG00000176239 | HGLibA_34091 | 18 | -0.971877317 | OR51B6 | olfactory receptor family 51 subfamily B member 6 [Source:HGNC Symbol;Acc:HGNC:19600] |
| ENSG00000176834 | HGLibA_53840 | 18 | -0.971877317 | VSIG10 | V-set and immunoglobulin domain containing 10 [Source:HGNC Symbol;Acc:HGNC:26078] |
| ENSG00000177414 | HGLibA_52662 | 18 | -0.971877317 | UBE2U | ubiquitin conjugating enzyme E2 U [Source:HGNC Symbol;Acc:HGNC:28559] |
| ENSG00000177485 | HGLibA_54861 | 18 | -0.971877317 | ZBTB33 | zinc finger and BTB domain containing 33 [Source:HGNC Symbol;Acc:HGNC:16682] |
| ENSG00000177688 | HGLibA_47785 | 18 | -0.971877317 | SUMO4 | small ubiquitin-like modifier 4 [Source:HGNC Symbol;Acc:HGNC:21181] |
| ENSG00000177946 | HGLibA_09042 | 18 | -0.971877317 | CENPBD1 | CENPB DNA-binding domain containing 1 [Source:HGNC Symbol;Acc:HGNC:28272] |
| ENSG00000178222 | HGLibA_41619 | 18 | -0.971877317 | RNF212 | ring finger protein 212 [Source:HGNC Symbol;Acc:HGNC:27729] |
| ENSG00000178591 | HGLibA_12878 | 18 | -0.971877317 | DEFB125 | defensin beta 125 [Source:HGNC Symbol;Acc:HGNC:18105] |
| ENSG00000179455 | HGLibA_29353 | 18 | -0.971877317 | MKRN3 | makorin ring finger protein 3 [Source:HGNC Symbol;Acc:HGNC:7114] |
| ENSG00000180071 | HGLibA_02017 | 18 | -0.971877317 | ANKRD18A | ankyrin repeat domain 18A [Source:HGNC Symbol;Acc:HGNC:23643] |
| ENSG00000181029 | HGLibA_51326 | 18 | -0.971877317 | TRAPPC5 | trafficking protein particle complex 5 [Source:HGNC Symbol;Acc:HGNC:23067] |
| ENSG00000181029 | HGLibA_51327 | 18 | -0.971877317 | TRAPPC5 | trafficking protein particle complex 5 [Source:HGNC Symbol;Acc:HGNC:23067] |
| ENSG00000181610 | HGLibA_30069 | 18 | -0.971877317 | MRPS23 | mitochondrial ribosomal protein S23 [Source:HGNC Symbol;Acc:HGNC:14509] |
| ENSG00000181626 | HGLibA_02134 | 18 | -0.971877317 | ANKRD62 | ankyrin repeat domain 62 [Source:HGNC Symbol;Acc:HGNC:35241] |
| ENSG00000181698 | HGLibA_34355 | 18 | -0.971877317 | OR5T1 | olfactory receptor family 5 subfamily T member 1 [Source:HGNC Symbol;Acc:HGNC:14821] |
| ENSG00000181781 | HGLibA_33294 | 18 | -0.971877317 | ODF3L2 | outer dense fiber of sperm tails 3 like 2 [Source:HGNC Symbol;Acc:HGNC:26841] |
| ENSG00000182118 | HGLibA_16781 | 18 | -0.971877317 | FAM89A | family with sequence similarity 89 member A [Source:HGNC Symbol;Acc:HGNC:25057] |
| ENSG00000182223 | HGLibA_54779 | 18 | -0.971877317 | ZAR1 | zygote arrest 1 [Source:HGNC Symbol;Acc:HGNC:20436] |
| ENSG00000182400 | HGLibA_51332 | 18 | -0.971877317 | TRAPPC6B | trafficking protein particle complex 6B [Source:HGNC Symbol;Acc:HGNC:23066] |
| ENSG00000182774 | HGLibA_42086 | 18 | -0.971877317 | RPS17 | ribosomal protein S17 [Source:HGNC Symbol;Acc:HGNC:10397] |
| ENSG00000182923 | HGLibA_09151 | 18 | -0.971877317 | CEP63 | centrosomal protein 63 [Source:HGNC Symbol;Acc:HGNC:25815] |
| ENSG00000183208 | HGLibA_18906 | 18 | -0.971877317 | GDPGP1 | GDP-D-glucose phosphorylase 1 [Source:HGNC Symbol;Acc:HGNC:34360] |
| ENSG00000183439 | HGLibA_51568 | 18 | -0.971877317 | TRIM61 | tripartite motif containing 61 [Source:HGNC Symbol;Acc:HGNC:24339] |
| ENSG00000184384 | HGLibA_28045 | 18 | -0.971877317 | MAML2 | mastermind like transcriptional coactivator 2 [Source:HGNC Symbol;Acc:HGNC:16259] |
| ENSG00000185127 | HGLibA_06440 | 18 | -0.971877317 | C6orf120 | chromosome 6 open reading frame 120 [Source:HGNC Symbol;Acc:HGNC:21247] |
| ENSG00000185940 | HGLibA_25801 | 18 | -0.971877317 | KRTAP5-5 | keratin associated protein 5-5 [Source:HGNC Symbol;Acc:HGNC:23601] |
| ENSG00000186193 | HGLibA_42734 | 18 | -0.971877317 | SAPCD2 | suppressor APC domain containing 2 [Source:HGNC Symbol;Acc:HGNC:28055] |
| ENSG00000186723 | HGLibA_33506 | 18 | -0.971877317 | OR10H1 | olfactory receptor family 10 subfamily H member 1 [Source:HGNC Symbol;Acc:HGNC:8172] |

| | | | | | |
|---|---|---|---|---|---|
| ENSG00000187005 | HGLibA_25708 | 18 | -0.971877317 | KRTAP21-1 | keratin associated protein 21-1 [Source:HGNC Symbol;Acc:HGNC:18945] |
| ENSG00000187607 | HGLibA_55750 | 18 | -0.971877317 | ZNF286A | zinc finger protein 286A [Source:HGNC Symbol;Acc:HGNC:13501] |
| ENSG00000187997 | HGLibA_05604 | 18 | -0.971877317 | C17orf99 | chromosome 17 open reading frame 99 [Source:HGNC Symbol;Acc:HGNC:34490] |
| ENSG00000188107 | HGLibA_15830 | 18 | -0.971877317 | EYS | eyes shut homolog [Source:HGNC Symbol;Acc:HGNC:21555] |
| ENSG00000188315 | HGLibA_06258 | 18 | -0.971877317 | C3orf62 | chromosome 3 open reading frame 62 [Source:HGNC Symbol;Acc:HGNC:24771] |
| ENSG00000189052 | HGLibA_09307 | 18 | -0.971877317 | CGB5 | chorionic gonadotropin subunit beta 5 [Source:HGNC Symbol;Acc:HGNC:16452] |
| ENSG00000189132 | HGLibA_16611 | 18 | -0.971877317 | FAM47B | family with sequence similarity 47 member B [Source:HGNC Symbol;Acc:HGNC:26659] |
| ENSG00000189369 | HGLibA_20441 | 18 | -0.971877317 | GSPT2 | G1 to S phase transition 2 [Source:HGNC Symbol;Acc:HGNC:4622] |
| ENSG00000196550 | HGLibA_16706 | 18 | -0.971877317 | FAM72A | family with sequence similarity 72 member A [Source:HGNC Symbol;Acc:HGNC:24044] |
| ENSG00000196639 | HGLibA_22093 | 18 | -0.971877317 | HRH1 | histamine receptor H1 [Source:HGNC Symbol;Acc:HGNC:5182] |
| ENSG00000196678 | HGLibA_15483 | 18 | -0.971877317 | ERI2 | ERI1 exoribonuclease family member 2 [Source:HGNC Symbol;Acc:HGNC:30541] |
| ENSG00000196781 | HGLibA_49608 | 18 | -0.971877317 | TLE1 | transducin like enhancer of split 1 [Source:HGNC Symbol;Acc:HGNC:11837] |
| ENSG00000197226 | HGLibA_48574 | 18 | -0.971877317 | TBC1D9B | TBC1 domain family member 9B [Source:HGNC Symbol;Acc:HGNC:29097] |
| ENSG00000197345 | HGLibA_29928 | 18 | -0.971877317 | MRPL21 | mitochondrial ribosomal protein L21 [Source:HGNC Symbol;Acc:HGNC:14479] |
| ENSG00000197713 | HGLibA_41824 | 18 | -0.971877317 | RPE | ribulose-5-phosphate-3-epimerase [Source:HGNC Symbol;Acc:HGNC:10293] |
| ENSG00000198019 | HGLibA_17187 | 18 | -0.971877317 | FCGR1B | Fc fragment of IgG receptor Ib [Source:HGNC Symbol;Acc:HGNC:3614] |
| ENSG00000198346 | HGLibA_56807 | 18 | -0.971877317 | ZNF813 | zinc finger protein 813 [Source:HGNC Symbol;Acc:HGNC:33257] |
| ENSG00000198960 | HGLibA_03055 | 18 | -0.971877317 | ARMCX6 | armadillo repeat containing X-linked 6 [Source:HGNC Symbol;Acc:HGNC:26094] |
| ENSG00000203661 | HGLibA_33888 | 18 | -0.971877317 | OR2T5 | olfactory receptor family 2 subfamily T member 5 [Source:HGNC Symbol;Acc:HGNC:15017] |
| ENSG00000213923 | HGLibA_11389 | 18 | -0.971877317 | CSNK1E | casein kinase 1 epsilon [Source:HGNC Symbol;Acc:HGNC:2453] |
| ENSG00000219626 | HGLibA_16538 | 18 | -0.971877317 | FAM228B | family with sequence similarity 228 member B [Source:HGNC Symbol;Acc:HGNC:24736] |
| ENSG00000221933 | HGLibA_33731 | 18 | -0.971877317 | OR2A25 | olfactory receptor family 2 subfamily A member 25 [Source:HGNC Symbol;Acc:HGNC:19562] |
| ENSG00000231924 | HGLibA_39033 | 18 | -0.971877317 | PSG1 | pregnancy specific beta-1-glycoprotein 1 [Source:HGNC Symbol;Acc:HGNC:9514] |
| ENSG00000234414 | HGLibA_40611 | 18 | -0.971877317 | RBMY1A1 | member A1 [Source:HGNC Symbol;Acc:HGNC:9912]" |
| ENSG00000237289 | HGLibA_09782 | 18 | -0.971877317 | CKMT1B | mitochondrial 1B [Source:HGNC Symbol;Acc:HGNC:1995]" |
| ENSG00000239672 | HGLibA_32152 | 18 | -0.971877317 | NME1 | NME/NM23 nucleoside diphosphate kinase 1 [Source:HGNC Symbol;Acc:HGNC:7849] |
| ENSG00000241404 | HGLibA_14629 | 18 | -0.971877317 | EGFL8 | EGF like domain multiple 8 [Source:HGNC Symbol;Acc:HGNC:13944] |
| ENSG00000242715 | HGLibA_07681 | 18 | -0.971877317 | CCDC169 | coiled-coil domain containing 169 [Source:HGNC Symbol;Acc:HGNC:34361] |
| ENSG00000243789 | HGLibA_23960 | 18 | -0.971877317 | JMJD7 | jumonji domain containing 7 [Source:HGNC Symbol;Acc:HGNC:34397] |
| ENSG00000250799 | HGLibA_38661 | 18 | -0.971877317 | PRODH2 | proline dehydrogenase 2 [Source:HGNC Symbol;Acc:HGNC:17325] |
| ENSG00000255012 | HGLibA_34329 | 18 | -0.971877317 | OR5M1 | olfactory receptor family 5 subfamily M member 1 [Source:HGNC Symbol;Acc:HGNC:8352] |
| ENSG00000255112 | HGLibA_09475 | 18 | -0.971877317 | CHMP1B | charged multivesicular body protein 1B [Source:HGNC Symbol;Acc:HGNC:24287] |
| ENSG00000255398 | HGLibA_20951 | 18 | -0.971877317 | HCAR3 | hydroxycarboxylic acid receptor 3 [Source:HGNC Symbol;Acc:HGNC:16824] |
| ENSG00000257594 | HGLibA_18559 | 18 | -0.971877317 | GALNT4 | polypeptide N-acetylgalactosaminyltransferase 4 [Source:HGNC Symbol;Acc:HGNC:4126] |
| ENSG00000261509 | HGLibA_51080 | 18 | -0.971877317 | TP53TG3B | TP53 target 3B [Source:HGNC Symbol;Acc:HGNC:37202] |
| ENSG00000269404 | HGLibA_46656 | 18 | -0.971877317 | SPIB | Spi-B transcription factor [Source:HGNC Symbol;Acc:HGNC:11242] |
| ENSG00000270181 | HGLibA_04537 | 18 | -0.971877317 | BIVM-ERCC5 | BIVM-ERCC5 readthrough [Source:HGNC Symbol;Acc:HGNC:43690] |
| ENSG00000273514 | HGLibA_17854 | 18 | -0.971877317 | FOXD4L6 | forkhead box D4 like 6 [Source:HGNC Symbol;Acc:HGNC:31986] |
| ENSG00000284922 | HGLibA_27505 | 18 | -0.971877317 | LRTOMT | novel protein |
| FAM84A | HGLibA_16764 | 18 | -0.971877317 | FAM84A | - |
| GLTSCR1 | HGLibA_19353 | 18 | -0.971877317 | GLTSCR1 | - |
| HIST2H2AA4 | HGLibA_21525 | 18 | -0.971877317 | HIST2H2AA4 | - |
| hsa-mir-1226 | HGLibA_57303 | 18 | -0.971877317 | hsa-mir-1226 | - |
| hsa-mir-125b-1 | HGLibA_57449 | 18 | -0.971877317 | hsa-mir-125b-1 | - |
| hsa-mir-1268a | HGLibA_57497 | 18 | -0.971877317 | hsa-mir-1268a | - |
| hsa-mir-1321 | HGLibA_57760 | 18 | -0.971877317 | hsa-mir-1321 | - |
| hsa-mir-2052 | HGLibA_58182 | 18 | -0.971877317 | hsa-mir-2052 | - |
| hsa-mir-23a | HGLibA_58329 | 18 | -0.971877317 | hsa-mir-23a | - |
| hsa-mir-25 | HGLibA_58354 | 18 | -0.971877317 | hsa-mir-25 | - |
| hsa-mir-3065 | HGLibA_58465 | 18 | -0.971877317 | hsa-mir-3065 | - |
| hsa-mir-3118-4 | HGLibA_58518 | 18 | -0.971877317 | hsa-mir-3118-4 | - |
| hsa-mir-3126 | HGLibA_58558 | 18 | -0.971877317 | hsa-mir-3126 | - |
| hsa-mir-320b-2 | HGLibA_58932 | 18 | -0.971877317 | hsa-mir-320b-2 | - |
| hsa-mir-323a | HGLibA_58955 | 18 | -0.971877317 | hsa-mir-323a | - |
| hsa-mir-346 | HGLibA_59032 | 18 | -0.971877317 | hsa-mir-346 | - |
| hsa-mir-3914-1 | HGLibA_59529 | 18 | -0.971877317 | hsa-mir-3914-1 | - |
| hsa-mir-3937 | HGLibA_59609 | 18 | -0.971877317 | hsa-mir-3937 | - |
| hsa-mir-4315-2 | HGLibA_59979 | 18 | -0.971877317 | hsa-mir-4315-2 | - |
| hsa-mir-433 | HGLibA_60045 | 18 | -0.971877317 | hsa-mir-433 | - |
| hsa-mir-4446 | HGLibA_60192 | 18 | -0.971877317 | hsa-mir-4446 | - |
| hsa-mir-4454 | HGLibA_60224 | 18 | -0.971877317 | hsa-mir-4454 | - |
| hsa-mir-4477b | HGLibA_60322 | 18 | -0.971877317 | hsa-mir-4477b | - |
| hsa-mir-4637 | HGLibA_60654 | 18 | -0.971877317 | hsa-mir-4637 | - |
| hsa-mir-4666a | HGLibA_60777 | 18 | -0.971877317 | hsa-mir-4666a | - |
| hsa-mir-4764 | HGLibA_61152 | 18 | -0.971877317 | hsa-mir-4764 | - |
| hsa-mir-4787 | HGLibA_61257 | 18 | -0.971877317 | hsa-mir-4787 | - |
| hsa-mir-517b | HGLibA_61628 | 18 | -0.971877317 | hsa-mir-517b | - |
| hsa-mir-519e | HGLibA_61729 | 18 | -0.971877317 | hsa-mir-519e | - |
| hsa-mir-548ad | HGLibA_61865 | 18 | -0.971877317 | hsa-mir-548ad | - |
| hsa-mir-548aj-1 | HGLibA_61889 | 18 | -0.971877317 | hsa-mir-548aj-1 | - |
| hsa-mir-548ap | HGLibA_61913 | 18 | -0.971877317 | hsa-mir-548ap | - |
| hsa-mir-548b | HGLibA_61950 | 18 | -0.971877317 | hsa-mir-548b | - |
| hsa-mir-548m | HGLibA_62039 | 18 | -0.971877317 | hsa-mir-548m | - |
| hsa-mir-550a-1 | HGLibA_62097 | 18 | -0.971877317 | hsa-mir-550a-1 | - |
| hsa-mir-5684 | HGLibA_62257 | 18 | -0.971877317 | hsa-mir-5684 | - |
| hsa-mir-5692c-2 | HGLibA_62302 | 18 | -0.971877317 | hsa-mir-5692c-2 | - |
| hsa-mir-599 | HGLibA_62488 | 18 | -0.971877317 | hsa-mir-599 | - |
| hsa-mir-629 | HGLibA_62756 | 18 | -0.971877317 | hsa-mir-629 | - |
| hsa-mir-641 | HGLibA_62803 | 18 | -0.971877317 | hsa-mir-641 | - |
| hsa-mir-6742 | HGLibA_63120 | 18 | -0.971877317 | hsa-mir-6742 | - |
| hsa-mir-6777 | HGLibA_63282 | 18 | -0.971877317 | hsa-mir-6777 | - |
| hsa-mir-6882 | HGLibA_63712 | 18 | -0.971877317 | hsa-mir-6882 | - |
| hsa-mir-6883 | HGLibA_63718 | 18 | -0.971877317 | hsa-mir-6883 | - |
| hsa-mir-6885 | HGLibA_63725 | 18 | -0.971877317 | hsa-mir-6885 | - |
| hsa-mir-8061 | HGLibA_64081 | 18 | -0.971877317 | hsa-mir-8061 | - |
| LACE1 | HGLibA_25910 | 18 | -0.971877317 | LACE1 | - |
| LIMS3L | HGLibA_26554 | 18 | -0.971877317 | LIMS3L | - |
| LOC100130539 | HGLibA_26793 | 18 | -0.971877317 | LOC100130539 | - |
| METTL21B | HGLibA_28978 | 18 | -0.971877317 | METTL21B | - |
| MINA | HGLibA_29272 | 18 | -0.971877317 | MINA | - |
| MTERFD1 | HGLibA_30340 | 18 | -0.971877317 | MTERFD1 | - |
| PET112 | HGLibA_36071 | 18 | -0.971877317 | PET112 | - |
| PPAPDC1A | HGLibA_37824 | 18 | -0.971877317 | PPAPDC1A | - |
| PPP2R4 | HGLibA_38168 | 18 | -0.971877317 | PPP2R4 | - |
| PPP5D1 | HGLibA_38216 | 18 | -0.971877317 | PPP5D1 | - |
| PRKRIR | HGLibA_38590 | 18 | -0.971877317 | PRKRIR | - |
| 2-Sep | HGLibA_43436 | 18 | -0.971877317 | 2-Sep | - |
| WAPAL | HGLibA_53942 | 18 | -0.971877317 | WAPAL | - |
| C15orf54 | HGLibA_05415 | 17 | -0.98092266 | C15orf54 | - |
| C17orf105 | HGLibA_05524 | 17 | -0.98092266 | C17orf105 | - |
| EFTUD1 | HGLibA_14614 | 17 | -0.98092266 | EFTUD1 | - |
| ENSG00000007171 | HGLibA_32328 | 17 | -0.98092266 | NOS2 | nitric oxide synthase 2 [Source:HGNC Symbol;Acc:HGNC:7873] |
| ENSG00000008710 | HGLibA_36789 | 17 | -0.98092266 | PKD1 | transient receptor potential channel interacting [Source:HGNC Symbol;Acc:HGNC:9008]" |
| ENSG00000014164 | HGLibA_54972 | 17 | -0.98092266 | ZC3H3 | zinc finger CCCH-type containing 3 [Source:HGNC Symbol;Acc:HGNC:28972] |
| ENSG00000015475 | HGLibA_04501 | 17 | -0.98092266 | BID | BH3 interacting domain death agonist [Source:HGNC Symbol;Acc:HGNC:1050] |
| ENSG00000022567 | HGLibA_45180 | 17 | -0.98092266 | SLC45A4 | solute carrier family 45 member 4 [Source:HGNC Symbol;Acc:HGNC:29196] |
| ENSG00000037757 | HGLibA_29857 | 17 | -0.98092266 | MRI1 | methylthioribose-1-phosphate isomerase 1 [Source:HGNC Symbol;Acc:HGNC:28469] |
| ENSG00000041515 | HGLibA_30808 | 17 | -0.98092266 | MYO16 | myosin XVI [Source:HGNC Symbol;Acc:HGNC:29822] |
| ENSG00000053328 | HGLibA_28993 | 17 | -0.98092266 | METTL24 | methyltransferase like 24 [Source:HGNC Symbol;Acc:HGNC:21566] |
| ENSG00000054983 | HGLibA_18510 | 17 | -0.98092266 | GALC | galactosylceramidase [Source:HGNC Symbol;Acc:HGNC:4115] |
| ENSG00000060237 | HGLibA_54333 | 17 | -0.98092266 | WNK1 | WNK lysine deficient protein kinase 1 [Source:HGNC Symbol;Acc:HGNC:14540] |
| ENSG00000061656 | HGLibA_46357 | 17 | -0.98092266 | SPAG4 | sperm associated antigen 4 [Source:HGNC Symbol;Acc:HGNC:11214] |
| ENSG00000066651 | HGLibA_51668 | 17 | -0.98092266 | TRMT11 | tRNA methyltransferase 11 homolog [Source:HGNC Symbol;Acc:HGNC:21080] |
| ENSG00000071203 | HGLibA_30133 | 17 | -0.98092266 | MS4A12 | membrane spanning 4-domains A12 [Source:HGNC Symbol;Acc:HGNC:13370] |
| ENSG00000072864 | HGLibA_31385 | 17 | -0.98092266 | NDE1 | nudE neurodevelopment protein 1 [Source:HGNC Symbol;Acc:HGNC:17619] |
| ENSG00000073605 | HGLibA_20408 | 17 | -0.98092266 | GSDMB | gasdermin B [Source:HGNC Symbol;Acc:HGNC:23690] |
| ENSG00000080546 | HGLibA_43619 | 17 | -0.98092266 | SESN1 | sestrin 1 [Source:HGNC Symbol;Acc:HGNC:21595] |
| ENSG00000090238 | HGLibA_54711 | 17 | -0.98092266 | YPEL3 | yippee like 3 [Source:HGNC Symbol;Acc:HGNC:18327] |
| ENSG00000090857 | HGLibA_35937 | 17 | -0.98092266 | PDPR | pyruvate dehydrogenase phosphatase regulatory subunit [Source:HGNC Symbol;Acc:HGNC:30264] |
| ENSG00000094975 | HGLibA_47708 | 17 | -0.98092266 | SUCO | SUN domain containing ossification factor [Source:HGNC Symbol;Acc:HGNC:1240] |
| ENSG00000099399 | HGLibA_27944 | 17 | -0.98092266 | MAGEB2 | MAGE family member B2 [Source:HGNC Symbol;Acc:HGNC:6809] |
| ENSG00000099942 | HGLibA_11183 | 17 | -0.98092266 | CRKL | adaptor protein [Source:HGNC Symbol;Acc:HGNC:2363]" |
| ENSG00000100583 | HGLibA_42677 | 17 | -0.98092266 | SAMD15 | sterile alpha motif domain containing 15 [Source:HGNC Symbol;Acc:HGNC:18631] |
| ENSG00000100983 | HGLibA_20447 | 17 | -0.98092266 | GSS | glutathione synthetase [Source:HGNC Symbol;Acc:HGNC:4624] |
| ENSG00000101162 | HGLibA_52274 | 17 | -0.98092266 | TUBB1 | tubulin beta 1 class VI [Source:HGNC Symbol;Acc:HGNC:16257] |
| ENSG00000101400 | HGLibA_45998 | 17 | -0.98092266 | SNTA1 | syntrophin alpha 1 [Source:HGNC Symbol;Acc:HGNC:11167] |
| ENSG00000104218 | HGLibA_11420 | 17 | -0.98092266 | CSPP1 | centrosome and spindle pole associated protein 1 [Source:HGNC Symbol;Acc:HGNC:26193] |
| ENSG00000104442 | HGLibA_03005 | 17 | -0.98092266 | ARMC1 | armadillo repeat containing 1 [Source:HGNC Symbol;Acc:HGNC:17684] |
| ENSG00000104660 | HGLibA_26330 | 17 | -0.98092266 | LEPROTL1 | leptin receptor overlapping transcript like 1 [Source:HGNC Symbol;Acc:HGNC:6555] |
| ENSG00000106211 | HGLibA_22302 | 17 | -0.98092266 | HSPB1 | heat shock protein family B (small) member 1 [Source:HGNC Symbol;Acc:HGNC:5246] |
| ENSG00000109452 | HGLibA_23339 | 17 | -0.98092266 | INPP4B | inositol polyphosphate-4-phosphatase type II B [Source:HGNC Symbol;Acc:HGNC:6075] |
| ENSG00000110090 | HGLibA_11019 | 17 | -0.98092266 | CPT1A | carnitine palmitoyltransferase 1A [Source:HGNC Symbol;Acc:HGNC:2328] |
| ENSG00000111052 | HGLibA_26572 | 17 | -0.98092266 | LIN7A | crumbs cell polarity complex component [Source:HGNC Symbol;Acc:HGNC:17787]" |
| ENSG00000112701 | HGLibA_43396 | 17 | -0.98092266 | SENP6 | SUMO specific peptidase 6 [Source:HGNC Symbol;Acc:HGNC:20944] |
| ENSG00000113845 | HGLibA_49531 | 17 | -0.98092266 | TIMMDC1 | translocase of inner mitochondrial membrane domain containing 1 [Source:HGNC Symbol;Acc:HGNC:1321] |

| | | | | | |
|---|---|---|---|---|---|
| ENSG00000115085 | HGLibA_54776 | 17 | -0.98092266 | ZAP70 | zeta chain of T cell receptor associated protein kinase 70 [Source:HGNC Symbol;Acc:HGNC:12858] |
| ENSG00000115297 | HGLibA_49675 | 17 | -0.98092266 | TLX2 | T cell leukemia homeobox 2 [Source:HGNC Symbol;Acc:HGNC:5057] |
| ENSG00000115705 | HGLibA_51155 | 17 | -0.98092266 | TPO | thyroid peroxidase [Source:HGNC Symbol;Acc:HGNC:12015] |
| ENSG00000118785 | HGLibA_46769 | 17 | -0.98092266 | SPP1 | secreted phosphoprotein 1 [Source:HGNC Symbol;Acc:HGNC:11255] |
| ENSG00000123131 | HGLibA_38416 | 17 | -0.98092266 | PRDX4 | peroxiredoxin 4 [Source:HGNC Symbol;Acc:HGNC:17169] |
| ENSG00000123562 | HGLibA_29663 | 17 | -0.98092266 | MORF4L2 | mortality factor 4 like 2 [Source:HGNC Symbol;Acc:HGNC:16849] |
| ENSG00000124134 | HGLibA_24383 | 17 | -0.98092266 | KCNS1 | potassium voltage-gated channel modifier subfamily S member 1 [Source:HGNC Symbol;Acc:HGNC:6300] |
| ENSG00000124143 | HGLibA_02776 | 17 | -0.98092266 | ARHGAP40 | Rho GTPase activating protein 40 [Source:HGNC Symbol;Acc:HGNC:16226] |
| ENSG00000124237 | HGLibA_06028 | 17 | -0.98092266 | C20orf85 | chromosome 20 open reading frame 85 [Source:HGNC Symbol;Acc:HGNC:16216] |
| ENSG00000124827 | HGLibA_18803 | 17 | -0.98092266 | GCM2 | glial cells missing homolog 2 [Source:HGNC Symbol;Acc:HGNC:4198] |
| ENSG00000125449 | HGLibA_03029 | 17 | -0.98092266 | ARMC7 | armadillo repeat containing 7 [Source:HGNC Symbol;Acc:HGNC:26168] |
| ENSG00000125843 | HGLibA_02359 | 17 | -0.98092266 | AP5S1 | adaptor related protein complex 5 subunit sigma 1 [Source:HGNC Symbol;Acc:HGNC:15875] |
| ENSG00000127423 | HGLibA_03866 | 17 | -0.98092266 | AUNIP | aurora kinase A and ninein interacting protein [Source:HGNC Symbol;Acc:HGNC:28363] |
| ENSG00000127948 | HGLibA_37704 | 17 | -0.98092266 | POR | cytochrome p450 oxidoreductase [Source:HGNC Symbol;Acc:HGNC:9208] |
| ENSG00000130589 | HGLibA_21127 | 17 | -0.98092266 | HELZ2 | helicase with zinc finger 2 [Source:HGNC Symbol;Acc:HGNC:30021] |
| ENSG00000131269 | HGLibA_00150 | 17 | -0.98092266 | ABCB7 | ATP binding cassette subfamily B member 7 [Source:HGNC Symbol;Acc:HGNC:48] |
| ENSG00000131378 | HGLibA_40951 | 17 | -0.98092266 | RFTN1 | lipid raft linker 1 [Source:HGNC Symbol;Acc:HGNC:30278]" |
| ENSG00000133116 | HGLibA_25021 | 17 | -0.98092266 | KL | klotho [Source:HGNC Symbol;Acc:HGNC:6344] |
| ENSG00000135409 | HGLibA_01787 | 17 | -0.98092266 | AMHR2 | anti-Mullerian hormone receptor type 2 [Source:HGNC Symbol;Acc:HGNC:465] |
| ENSG00000135953 | HGLibA_29119 | 17 | -0.98092266 | MFSD9 | major facilitator superfamily domain containing 9 [Source:HGNC Symbol;Acc:HGNC:28158] |
| ENSG00000136114 | HGLibA_49399 | 17 | -0.98092266 | THSD1 | thrombospondin type 1 domain containing 1 [Source:HGNC Symbol;Acc:HGNC:17754] |
| ENSG00000136888 | HGLibA_03758 | 17 | -0.98092266 | ATP6V1G1 | ATPase H+ transporting V1 subunit G1 [Source:HGNC Symbol;Acc:HGNC:864] |
| ENSG00000137133 | HGLibA_21327 | 17 | -0.98092266 | HINT2 | histidine triad nucleotide binding protein 2 [Source:HGNC Symbol;Acc:HGNC:18344] |
| ENSG00000138190 | HGLibA_15753 | 17 | -0.98092266 | EXOC6 | exocyst complex component 6 [Source:HGNC Symbol;Acc:HGNC:23196] |
| ENSG00000138668 | HGLibA_21813 | 17 | -0.98092266 | HNRNPD | heterogeneous nuclear ribonucleoprotein D [Source:HGNC Symbol;Acc:HGNC:5036] |
| ENSG00000140285 | HGLibA_17417 | 17 | -0.98092266 | FGF7 | fibroblast growth factor 7 [Source:HGNC Symbol;Acc:HGNC:3685] |
| ENSG00000140749 | HGLibA_22898 | 17 | -0.98092266 | IGSF6 | immunoglobulin superfamily member 6 [Source:HGNC Symbol;Acc:HGNC:5953] |
| ENSG00000140876 | HGLibA_32989 | 17 | -0.98092266 | NUDT7 | nudix hydrolase 7 [Source:HGNC Symbol;Acc:HGNC:8054] |
| ENSG00000141252 | HGLibA_53808 | 17 | -0.98092266 | VPS53 | GARP complex subunit [Source:HGNC Symbol;Acc:HGNC:25608]" |
| ENSG00000141338 | HGLibA_00126 | 17 | -0.98092266 | ABCA8 | ATP binding cassette subfamily A member 8 [Source:HGNC Symbol;Acc:HGNC:38] |
| ENSG00000141750 | HGLibA_47316 | 17 | -0.98092266 | STAC2 | SH3 and cysteine rich domain 2 [Source:HGNC Symbol;Acc:HGNC:23990] |
| ENSG00000143450 | HGLibA_33218 | 17 | -0.98092266 | OAZ3 | ornithine decarboxylase antizyme 3 [Source:HGNC Symbol;Acc:HGNC:8097] |
| ENSG00000144115 | HGLibA_49362 | 17 | -0.98092266 | THNSL2 | threonine synthase like 2 [Source:HGNC Symbol;Acc:HGNC:25602] |
| ENSG00000144452 | HGLibA_00101 | 17 | -0.98092266 | ABCA12 | ATP binding cassette subfamily A member 12 [Source:HGNC Symbol;Acc:HGNC:14637] |
| ENSG00000144730 | HGLibA_23014 | 17 | -0.98092266 | IL17RD | interleukin 17 receptor D [Source:HGNC Symbol;Acc:HGNC:17616] |
| ENSG00000144848 | HGLibA_03486 | 17 | -0.98092266 | ATG3 | autophagy related 3 [Source:HGNC Symbol;Acc:HGNC:20962] |
| ENSG00000145283 | HGLibA_44346 | 17 | -0.98092266 | SLC10A6 | solute carrier family 10 member 6 [Source:HGNC Symbol;Acc:HGNC:30603] |
| ENSG00000145388 | HGLibA_28957 | 17 | -0.98092266 | METTL14 | methyltransferase like 14 [Source:HGNC Symbol;Acc:HGNC:29330] |
| ENSG00000146374 | HGLibA_42350 | 17 | -0.98092266 | RSPO3 | R-spondin 3 [Source:HGNC Symbol;Acc:HGNC:20866] |
| ENSG00000146963 | HGLibA_27622 | 17 | -0.98092266 | LUC7L2 | pre-mRNA splicing factor [Source:HGNC Symbol;Acc:HGNC:21608]" |
| ENSG00000150244 | HGLibA_51520 | 17 | -0.98092266 | TRIM48 | tripartite motif containing 48 [Source:HGNC Symbol;Acc:HGNC:19021] |
| ENSG00000150636 | HGLibA_07512 | 17 | -0.98092266 | CCDC102B | coiled-coil domain containing 102B [Source:HGNC Symbol;Acc:HGNC:26295] |
| ENSG00000150753 | HGLibA_08205 | 17 | -0.98092266 | CCT5 | chaperonin containing TCP1 subunit 5 [Source:HGNC Symbol;Acc:HGNC:1618] |
| ENSG00000150787 | HGLibA_39558 | 17 | -0.98092266 | PTS | 6-pyruvoyltetrahydropterin synthase [Source:HGNC Symbol;Acc:HGNC:9689] |
| ENSG00000150907 | HGLibA_17920 | 17 | -0.98092266 | FOXO1 | forkhead box O1 [Source:HGNC Symbol;Acc:HGNC:3819] |
| ENSG00000152700 | HGLibA_42739 | 17 | -0.98092266 | SAR1B | secretion associated Ras related GTPase 1B [Source:HGNC Symbol;Acc:HGNC:10535] |
| ENSG00000153574 | HGLibA_41849 | 17 | -0.98092266 | RPIA | ribose 5-phosphate isomerase A [Source:HGNC Symbol;Acc:HGNC:10297] |
| ENSG00000153790 | HGLibA_06533 | 17 | -0.98092266 | C7orf31 | chromosome 7 open reading frame 31 [Source:HGNC Symbol;Acc:HGNC:21722] |
| ENSG00000153802 | HGLibA_50619 | 17 | -0.98092266 | TMPRSS11D | transmembrane serine protease 11D [Source:HGNC Symbol;Acc:HGNC:24059] |
| ENSG00000155561 | HGLibA_33035 | 17 | -0.98092266 | NUP205 | nucleoporin 205 [Source:HGNC Symbol;Acc:HGNC:18658] |
| ENSG00000155962 | HGLibA_10023 | 17 | -0.98092266 | CLIC2 | chloride intracellular channel 2 [Source:HGNC Symbol;Acc:HGNC:2063] |
| ENSG00000156427 | HGLibA_17385 | 17 | -0.98092266 | FGF18 | fibroblast growth factor 18 [Source:HGNC Symbol;Acc:HGNC:3674] |
| ENSG00000156885 | HGLibA_10845 | 17 | -0.98092266 | COX6A2 | cytochrome c oxidase subunit 6A2 [Source:HGNC Symbol;Acc:HGNC:2279] |
| ENSG00000158623 | HGLibA_10706 | 17 | -0.98092266 | COPG2 | coatomer protein complex subunit gamma 2 [Source:HGNC Symbol;Acc:HGNC:2237] |
| ENSG00000158711 | HGLibA_14918 | 17 | -0.98092266 | ELK4 | ETS transcription factor [Source:HGNC Symbol;Acc:HGNC:3326]" |
| ENSG00000158869 | HGLibA_17171 | 17 | -0.98092266 | FCER1G | Fc fragment of IgE receptor Ig [Source:HGNC Symbol;Acc:HGNC:3611] |
| ENSG00000159197 | HGLibA_24188 | 17 | -0.98092266 | KCNE2 | potassium voltage-gated channel subfamily E regulatory subunit 2 [Source:HGNC Symbol;Acc:HGNC:6242] |
| ENSG00000162139 | HGLibA_31716 | 17 | -0.98092266 | NEU3 | neuraminidase 3 [Source:HGNC Symbol;Acc:HGNC:7760] |
| ENSG00000163239 | HGLibA_48900 | 17 | -0.98092266 | TDRD10 | tudor domain containing 10 [Source:HGNC Symbol;Acc:HGNC:25316] |
| ENSG00000163634 | HGLibA_49378 | 17 | -0.98092266 | THOC7 | THO complex 7 [Source:HGNC Symbol;Acc:HGNC:29874] |
| ENSG00000163714 | HGLibA_52500 | 17 | -0.98092266 | U2SURP | U2 snRNP associated SURP domain containing [Source:HGNC Symbol;Acc:HGNC:30855] |
| ENSG00000164181 | HGLibA_14976 | 17 | -0.98092266 | ELOVL7 | ELOVL fatty acid elongase 7 [Source:HGNC Symbol;Acc:HGNC:26292] |
| ENSG00000164532 | HGLibA_48658 | 17 | -0.98092266 | TBX20 | T-box 20 [Source:HGNC Symbol;Acc:HGNC:11598] |
| ENSG00000164821 | HGLibA_12802 | 17 | -0.98092266 | DEFA4 | defensin alpha 4 [Source:HGNC Symbol;Acc:HGNC:2763] |
| ENSG00000165379 | HGLibA_27163 | 17 | -0.98092266 | LRFN5 | leucine rich repeat and fibronectin type III domain containing 5 [Source:HGNC Symbol;Acc:HGNC:20360] |
| ENSG00000165929 | HGLibA_48686 | 17 | -0.98092266 | TC2N | nuclear [Source:HGNC Symbol;Acc:HGNC:19859]" |
| ENSG00000166181 | HGLibA_02419 | 17 | -0.98092266 | API5 | apoptosis inhibitor 5 [Source:HGNC Symbol;Acc:HGNC:594] |
| ENSG00000166478 | HGLibA_55492 | 17 | -0.98092266 | ZNF143 | zinc finger protein 143 [Source:HGNC Symbol;Acc:HGNC:12928] |
| ENSG00000167080 | HGLibA_04015 | 17 | -0.98092266 | B4GALNT2 | 4-N-acetyl-galactosaminyltransferase 2 [Source:HGNC Symbol;Acc:HGNC:24136]" |
| ENSG00000167191 | HGLibA_20127 | 17 | -0.98092266 | GPRC5B | G protein-coupled receptor class C group 5 member B [Source:HGNC Symbol;Acc:HGNC:13308] |
| ENSG00000167508 | HGLibA_30596 | 17 | -0.98092266 | MVD | mevalonate diphosphate decarboxylase [Source:HGNC Symbol;Acc:HGNC:7529] |
| ENSG00000168269 | HGLibA_17875 | 17 | -0.98092266 | FOXI1 | forkhead box I1 [Source:HGNC Symbol;Acc:HGNC:3815] |
| ENSG00000169435 | HGLibA_40390 | 17 | -0.98092266 | RASSF6 | Ras association domain family member 6 [Source:HGNC Symbol;Acc:HGNC:20796] |
| ENSG00000169627 | HGLibA_04685 | 17 | -0.98092266 | BOLA2B | bolA family member 2B [Source:HGNC Symbol;Acc:HGNC:32479] |
| ENSG00000169992 | HGLibA_32069 | 17 | -0.98092266 | NLGN2 | neuroligin 2 [Source:HGNC Symbol;Acc:HGNC:14290] |
| ENSG00000170683 | HGLibA_33463 | 17 | -0.98092266 | OR10A3 | olfactory receptor family 10 subfamily A member 3 [Source:HGNC Symbol;Acc:HGNC:8162] |
| ENSG00000171453 | HGLibA_37554 | 17 | -0.98092266 | POLR1C | RNA polymerase I and III subunit C [Source:HGNC Symbol;Acc:HGNC:20194] |
| ENSG00000171488 | HGLibA_27435 | 17 | -0.98092266 | LRRC8C | leucine rich repeat containing 8 VRAC subunit C [Source:HGNC Symbol;Acc:HGNC:25075] |
| ENSG00000172769 | HGLibA_34268 | 17 | -0.98092266 | OR5B3 | olfactory receptor family 5 subfamily B member 3 [Source:HGNC Symbol;Acc:HGNC:8324] |
| ENSG00000174165 | HGLibA_55112 | 17 | -0.98092266 | ZDHHC24 | zinc finger DHHC-type containing 24 [Source:HGNC Symbol;Acc:HGNC:27387] |
| ENSG00000175309 | HGLibA_36479 | 17 | -0.98092266 | PHYKPL | 5-phosphohydroxy-L-lysine phospho-lyase [Source:HGNC Symbol;Acc:HGNC:28249] |
| ENSG00000177186 | HGLibA_33845 | 17 | -0.98092266 | OR2M7 | olfactory receptor family 2 subfamily M member 7 [Source:HGNC Symbol;Acc:HGNC:19594] |
| ENSG00000178038 | HGLibA_01722 | 17 | -0.98092266 | ALS2CL | ALS2 C-terminal like [Source:HGNC Symbol;Acc:HGNC:20605] |
| ENSG00000182199 | HGLibA_44062 | 17 | -0.98092266 | SHMT2 | serine hydroxymethyltransferase 2 [Source:HGNC Symbol;Acc:HGNC:10852] |
| ENSG00000182253 | HGLibA_48014 | 17 | -0.98092266 | SYNM | synemin [Source:HGNC Symbol;Acc:HGNC:24466] |
| ENSG00000182898 | HGLibA_48807 | 17 | -0.98092266 | TCHHL1 | trichohyalin like 1 [Source:HGNC Symbol;Acc:HGNC:31796] |
| ENSG00000183011 | HGLibA_30994 | 17 | -0.98092266 | NAA38 | NatC auxiliary subunit [Source:HGNC Symbol;Acc:HGNC:28212]" |
| ENSG00000184361 | HGLibA_46508 | 17 | -0.98092266 | SPATA32 | spermatogenesis associated 32 [Source:HGNC Symbol;Acc:HGNC:26349] |
| ENSG00000184659 | HGLibA_17848 | 17 | -0.98092266 | FOXD4L4 | forkhead box D4 like 4 [Source:HGNC Symbol;Acc:HGNC:23762] |
| ENSG00000185721 | HGLibA_13973 | 17 | -0.98092266 | DRG1 | developmentally regulated GTP binding protein 1 [Source:HGNC Symbol;Acc:HGNC:3029] |
| ENSG00000186197 | HGLibA_14438 | 17 | -0.98092266 | EDARADD | EDAR associated death domain [Source:HGNC Symbol;Acc:HGNC:14341] |
| ENSG00000186204 | HGLibA_12184 | 17 | -0.98092266 | CYP4F12 | cytochrome P450 family 4 subfamily F member 12 [Source:HGNC Symbol;Acc:HGNC:18857] |
| ENSG00000186687 | HGLibA_27757 | 17 | -0.98092266 | LYRM7 | LYR motif containing 7 [Source:HGNC Symbol;Acc:HGNC:28072] |
| ENSG00000188015 | HGLibA_42553 | 17 | -0.98092266 | S100A3 | S100 calcium binding protein A3 [Source:HGNC Symbol;Acc:HGNC:10493] |
| ENSG00000188610 | HGLibA_16711 | 17 | -0.98092266 | FAM72B | family with sequence similarity 72 member B [Source:HGNC Symbol;Acc:HGNC:24805] |
| ENSG00000196233 | HGLibA_26193 | 17 | -0.98092266 | LCOR | ligand dependent nuclear receptor corepressor [Source:HGNC Symbol;Acc:HGNC:29503] |
| ENSG00000196549 | HGLibA_29467 | 17 | -0.98092266 | MME | membrane metalloendopeptidase [Source:HGNC Symbol;Acc:HGNC:7154] |
| ENSG00000196570 | HGLibA_36181 | 17 | -0.98092266 | PFN3 | profilin 3 [Source:HGNC Symbol;Acc:HGNC:18627] |
| ENSG00000197467 | HGLibA_10508 | 17 | -0.98092266 | COL13A1 | collagen type XIII alpha 1 chain [Source:HGNC Symbol;Acc:HGNC:2190] |
| ENSG00000198019 | HGLibA_17186 | 17 | -0.98092266 | FCGR1B | Fc fragment of IgG receptor Ib [Source:HGNC Symbol;Acc:HGNC:3614] |
| ENSG00000198178 | HGLibA_09983 | 17 | -0.98092266 | CLEC4C | C-type lectin domain family 4 member C [Source:HGNC Symbol;Acc:HGNC:13258] |
| ENSG00000198216 | HGLibA_06888 | 17 | -0.98092266 | CACNA1E | calcium voltage-gated channel subunit alpha1 E [Source:HGNC Symbol;Acc:HGNC:1392] |
| ENSG00000203666 | HGLibA_14534 | 17 | -0.98092266 | EFCAB2 | EF-hand calcium binding domain 2 [Source:HGNC Symbol;Acc:HGNC:28166] |
| ENSG00000204278 | HGLibA_50270 | 17 | -0.98092266 | TMEM235 | transmembrane protein 235 [Source:HGNC Symbol;Acc:HGNC:27563] |
| ENSG00000204669 | HGLibA_06744 | 17 | -0.98092266 | C9orf57 | chromosome 9 open reading frame 57 [Source:HGNC Symbol;Acc:HGNC:27037] |
| ENSG00000204956 | HGLibA_35518 | 17 | -0.98092266 | PCDHGA1 | 1 [Source:HGNC Symbol;Acc:HGNC:8696]" |
| ENSG00000205496 | HGLibA_34074 | 17 | -0.98092266 | OR51A2 | olfactory receptor family 51 subfamily A member 2 [Source:HGNC Symbol;Acc:HGNC:14764] |
| ENSG00000212721 | HGLibA_25751 | 17 | -0.98092266 | KRTAP4-11 | keratin associated protein 4-11 [Source:HGNC Symbol;Acc:HGNC:18911] |
| ENSG00000213139 | HGLibA_11286 | 17 | -0.98092266 | CRYGS | crystallin gamma S [Source:HGNC Symbol;Acc:HGNC:2417] |
| ENSG00000215183 | HGLibA_30230 | 17 | -0.98092266 | MSMP | prostate associated [Source:HGNC Symbol;Acc:HGNC:29663]" |
| ENSG00000221937 | HGLibA_48401 | 17 | -0.98092266 | TAS2R40 | taste 2 receptor member 40 [Source:HGNC Symbol;Acc:HGNC:18885] |
| ENSG00000223510 | HGLibA_08876 | 17 | -0.98092266 | CDRT15 | CMT1A duplicated region transcript 15 [Source:HGNC Symbol;Acc:HGNC:14395] |
| ENSG00000235718 | HGLibA_29079 | 17 | -0.98092266 | MFRP | membrane frizzled-related protein [Source:HGNC Symbol;Acc:HGNC:18121] |
| ENSG00000236334 | HGLibA_37914 | 17 | -0.98092266 | PPIAL4G | peptidylprolyl isomerase A like 4G [Source:HGNC Symbol;Acc:HGNC:33996] |
| ENSG00000241595 | HGLibA_25838 | 17 | -0.98092266 | KRTAP9-4 | keratin associated protein 9-4 [Source:HGNC Symbol;Acc:HGNC:18902] |
| ENSG00000242252 | HGLibA_04461 | 17 | -0.98092266 | BGLAP | bone gamma-carboxyglutamate protein [Source:HGNC Symbol;Acc:HGNC:1043] |
| ENSG00000249884 | HGLibA_41460 | 17 | -0.98092266 | RNF103-CHMP3 | RNF103-CHMP3 readthrough [Source:HGNC Symbol;Acc:HGNC:38847] |
| ENSG00000250709 | HGLibA_07686 | 17 | -0.98092266 | CCDC169-SOHLH2 | CCDC169-SOHLH2 readthrough [Source:HGNC Symbol;Acc:HGNC:38866] |
| ENSG00000253457 | HGLibA_45739 | 17 | -0.98092266 | SMIM18 | small integral membrane protein 18 [Source:HGNC Symbol;Acc:HGNC:42973] |
| ENSG00000256892 | HGLibA_30488 | 17 | -0.98092266 | MTRNR2L7 | MT-RNR2 like 7 [Source:HGNC Symbol;Acc:HGNC:37164] |
| ENSG00000258724 | HGLibA_36696 | 17 | -0.98092266 | PINX1 | PIN2 (TERF1) interacting telomerase inhibitor 1 [Source:HGNC Symbol;Acc:HGNC:30046] |
| ENSG00000258890 | HGLibA_09178 | 17 | -0.98092266 | CEP95 | centrosomal protein 95 [Source:HGNC Symbol;Acc:HGNC:25141] |
| ENSG00000263353 | HGLibA_37897 | 17 | -0.98092266 | PPIAL4A | peptidylprolyl isomerase A like 4A [Source:HGNC Symbol;Acc:HGNC:24369] |
| ENSG00000273749 | HGLibA_12032 | 17 | -0.98092266 | CYFIP1 | cytoplasmic FMR1 interacting protein 1 [Source:HGNC Symbol;Acc:HGNC:13759] |
| ENSG00000274102 | HGLibA_34044 | 17 | -0.98092266 | OR4M2 | olfactory receptor family 4 subfamily M member 2 [Source:HGNC Symbol;Acc:HGNC:15373] |
| ENSG00000276119 | HGLibA_33596 | 17 | -0.98092266 | OR13C2 | olfactory receptor family 13 subfamily C member 2 [Source:HGNC Symbol;Acc:HGNC:14701] |
| FAM26D | HGLibA_16560 | 17 | -0.98092266 | FAM26D | - |
| HIST1H4K | HGLibA_21516 | 17 | -0.98092266 | HIST1H4K | - |
| hsa-mir-1185-2 | HGLibA_57244 | 17 | -0.98092266 | hsa-mir-1185-2 | - |
| hsa-mir-1260a | HGLibA_57461 | 17 | -0.98092266 | hsa-mir-1260a | - |
| hsa-mir-141 | HGLibA_57828 | 17 | -0.98092266 | hsa-mir-141 | - |
| hsa-mir-29c | HGLibA_58421 | 17 | -0.98092266 | hsa-mir-29c | - |
| hsa-mir-3123 | HGLibA_58544 | 17 | -0.98092266 | hsa-mir-3123 | - |
| hsa-mir-320b-2 | HGLibA_58933 | 17 | -0.98092266 | hsa-mir-320b-2 | - |
| hsa-mir-3622a | HGLibA_59120 | 17 | -0.98092266 | hsa-mir-3622a | - |

| | | | | | |
|---|---|---|---|---|---|
| hsa-mir-3714 | HGLibA_59369 | 17 | -0.98092266 | hsa-mir-3714 | - |
| hsa-mir-3910-2 | HGLibA_59510 | 17 | -0.98092266 | hsa-mir-3910-2 | - |
| hsa-mir-3916 | HGLibA_59539 | 17 | -0.98092266 | hsa-mir-3916 | - |
| hsa-mir-4276 | HGLibA_59808 | 17 | -0.98092266 | hsa-mir-4276 | - |
| hsa-mir-4315-1 | HGLibA_59975 | 17 | -0.98092266 | hsa-mir-4315-1 | - |
| hsa-mir-4443 | HGLibA_60176 | 17 | -0.98092266 | hsa-mir-4443 | - |
| hsa-mir-4477b | HGLibA_60323 | 17 | -0.98092266 | hsa-mir-4477b | - |
| hsa-mir-450a-2 | HGLibA_60477 | 17 | -0.98092266 | hsa-mir-450a-2 | - |
| hsa-mir-451a | HGLibA_60525 | 17 | -0.98092266 | hsa-mir-451a | - |
| hsa-mir-4723 | HGLibA_60989 | 17 | -0.98092266 | hsa-mir-4723 | - |
| hsa-mir-4726 | HGLibA_61002 | 17 | -0.98092266 | hsa-mir-4726 | - |
| hsa-mir-4781 | HGLibA_61233 | 17 | -0.98092266 | hsa-mir-4781 | - |
| hsa-mir-516b-1 | HGLibA_61617 | 17 | -0.98092266 | hsa-mir-516b-1 | - |
| hsa-mir-517a | HGLibA_61624 | 17 | -0.98092266 | hsa-mir-517a | - |
| hsa-mir-518d | HGLibA_61666 | 17 | -0.98092266 | hsa-mir-518d | - |
| hsa-mir-521-2 | HGLibA_61770 | 17 | -0.98092266 | hsa-mir-521-2 | - |
| hsa-mir-548h-4 | HGLibA_62006 | 17 | -0.98092266 | hsa-mir-548h-4 | - |
| hsa-mir-6083 | HGLibA_62593 | 17 | -0.98092266 | hsa-mir-6083 | - |
| hsa-mir-6129 | HGLibA_62665 | 17 | -0.98092266 | hsa-mir-6129 | - |
| hsa-mir-641 | HGLibA_62805 | 17 | -0.98092266 | hsa-mir-641 | - |
| hsa-mir-6499 | HGLibA_62844 | 17 | -0.98092266 | hsa-mir-6499 | - |
| hsa-mir-6512 | HGLibA_62922 | 17 | -0.98092266 | hsa-mir-6512 | - |
| hsa-mir-6715a | HGLibA_63019 | 17 | -0.98092266 | hsa-mir-6715a | - |
| hsa-mir-6718 | HGLibA_63034 | 17 | -0.98092266 | hsa-mir-6718 | - |
| hsa-mir-6765 | HGLibA_63220 | 17 | -0.98092266 | hsa-mir-6765 | - |
| hsa-mir-6770-3 | HGLibA_63254 | 17 | -0.98092266 | hsa-mir-6770-3 | - |
| hsa-mir-7976 | HGLibA_64035 | 17 | -0.98092266 | hsa-mir-7976 | - |
| KIAA1107 | HGLibA_24691 | 17 | -0.98092266 | KIAA1107 | - |
| LACE1 | HGLibA_25912 | 17 | -0.98092266 | LACE1 | - |
| RSG1 | HGLibA_42313 | 17 | -0.98092266 | RSG1 | - |
| 3-Sep | HGLibA_43439 | 17 | -0.98092266 | 3-Sep | - |
| SLC25A6_X | HGLibA_44775 | 17 | -0.98092266 | SLC25A6_X | - |
| SPATA31A4 | HGLibA_46477 | 17 | -0.98092266 | SPATA31A4 | - |
| AAED1 | HGLibA_00043 | 16 | -0.989968003 | AAED1 | - |
| ADRBK2 | HGLibA_01068 | 16 | -0.989968003 | ADRBK2 | - |
| APITD1-CORT | HGLibA_02428 | 16 | -0.989968003 | APITD1-CORT | - |
| BZRAP1 | HGLibA_05011 | 16 | -0.989968003 | BZRAP1 | - |
| C18orf42 | HGLibA_05614 | 16 | -0.989968003 | C18orf42 | - |
| C19orf70 | HGLibA_05700 | 16 | -0.989968003 | C19orf70 | - |
| C6orf7 | HGLibA_06506 | 16 | -0.989968003 | C6orf7 | - |
| CGB | HGLibA_09298 | 16 | -0.989968003 | CGB | - |
| DFNA5 | HGLibA_13024 | 16 | -0.989968003 | DFNA5 | - |
| ENSG00000001617 | HGLibA_43333 | 16 | -0.989968003 | SEMA3F | semaphorin 3F [Source:HGNC Symbol;Acc:HGNC:10728] |
| ENSG00000003400 | HGLibA_07298 | 16 | -0.989968003 | CASP10 | caspase 10 [Source:HGNC Symbol;Acc:HGNC:1500] |
| ENSG00000004866 | HGLibA_47281 | 16 | -0.989968003 | ST7 | suppression of tumorigenicity 7 [Source:HGNC Symbol;Acc:HGNC:11351] |
| ENSG00000004975 | HGLibA_14211 | 16 | -0.989968003 | DVL2 | dishevelled segment polarity protein 2 [Source:HGNC Symbol;Acc:HGNC:3086] |
| ENSG00000005075 | HGLibA_37592 | 16 | -0.989968003 | POLR2J | RNA polymerase II subunit J [Source:HGNC Symbol;Acc:HGNC:9197] |
| ENSG00000006016 | HGLibA_11185 | 16 | -0.989968003 | CRLF1 | cytokine receptor like factor 1 [Source:HGNC Symbol;Acc:HGNC:2364] |
| ENSG00000006831 | HGLibA_00982 | 16 | -0.989968003 | ADIPOR2 | adiponectin receptor 2 [Source:HGNC Symbol;Acc:HGNC:24041] |
| ENSG00000033627 | HGLibA_03704 | 16 | -0.989968003 | ATP6V0A1 | ATPase H+ transporting V0 subunit a1 [Source:HGNC Symbol;Acc:HGNC:865] |
| ENSG00000037897 | HGLibA_28944 | 16 | -0.989968003 | METTL1 | methyltransferase like 1 [Source:HGNC Symbol;Acc:HGNC:7030] |
| ENSG00000048052 | HGLibA_21028 | 16 | -0.989968003 | HDAC9 | histone deacetylase 9 [Source:HGNC Symbol;Acc:HGNC:14065] |
| ENSG00000048052 | HGLibA_21026 | 16 | -0.989968003 | HDAC9 | histone deacetylase 9 [Source:HGNC Symbol;Acc:HGNC:14065] |
| ENSG00000049167 | HGLibA_15454 | 16 | -0.989968003 | ERCC8 | CSA ubiquitin ligase complex subunit [Source:HGNC Symbol;Acc:HGNC:3439]" |
| ENSG00000053900 | HGLibA_01882 | 16 | -0.989968003 | ANAPC4 | anaphase promoting complex subunit 4 [Source:HGNC Symbol;Acc:HGNC:19990] |
| ENSG00000064419 | HGLibA_50903 | 16 | -0.989968003 | TNPO3 | transportin 3 [Source:HGNC Symbol;Acc:HGNC:17103] |
| ENSG00000065491 | HGLibA_48506 | 16 | -0.989968003 | TBC1D22B | TBC1 domain family member 22B [Source:HGNC Symbol;Acc:HGNC:21602] |
| ENSG00000066933 | HGLibA_30867 | 16 | -0.989968003 | MYO9A | myosin IXA [Source:HGNC Symbol;Acc:HGNC:7608] |
| ENSG00000068120 | HGLibA_10457 | 16 | -0.989968003 | COASY | Coenzyme A synthase [Source:HGNC Symbol;Acc:HGNC:29932] |
| ENSG00000070019 | HGLibA_20662 | 16 | -0.989968003 | GUCY2C | guanylate cyclase 2C [Source:HGNC Symbol;Acc:HGNC:4688] |
| ENSG00000071794 | HGLibA_21657 | 16 | -0.989968003 | HLTF | helicase like transcription factor [Source:HGNC Symbol;Acc:HGNC:11099] |
| ENSG00000073792 | HGLibA_22804 | 16 | -0.989968003 | IGF2BP2 | insulin like growth factor 2 mRNA binding protein 2 [Source:HGNC Symbol;Acc:HGNC:28867] |
| ENSG00000073921 | HGLibA_36522 | 16 | -0.989968003 | PICALM | phosphatidylinositol binding clathrin assembly protein [Source:HGNC Symbol;Acc:HGNC:15514] |
| ENSG00000084073 | HGLibA_55386 | 16 | -0.989968003 | ZMPSTE24 | zinc metallopeptidase STE24 [Source:HGNC Symbol;Acc:HGNC:12877] |
| ENSG00000087495 | HGLibA_36303 | 16 | -0.989968003 | PHACTR3 | phosphatase and actin regulator 3 [Source:HGNC Symbol;Acc:HGNC:15833] |
| ENSG00000090554 | HGLibA_17669 | 16 | -0.989968003 | FLT3LG | fms related tyrosine kinase 3 ligand [Source:HGNC Symbol;Acc:HGNC:3766] |
| ENSG00000090659 | HGLibA_08294 | 16 | -0.989968003 | CD209 | CD209 molecule [Source:HGNC Symbol;Acc:HGNC:1641] |
| ENSG00000091039 | HGLibA_34666 | 16 | -0.989968003 | OSBPL8 | oxysterol binding protein like 8 [Source:HGNC Symbol;Acc:HGNC:16396] |
| ENSG00000093144 | HGLibA_14395 | 16 | -0.989968003 | ECHDC1 | ethylmalonyl-CoA decarboxylase 1 [Source:HGNC Symbol;Acc:HGNC:21489] |
| ENSG00000100296 | HGLibA_49372 | 16 | -0.989968003 | THOC5 | THO complex 5 [Source:HGNC Symbol;Acc:HGNC:19074] |
| ENSG00000100722 | HGLibA_54965 | 16 | -0.989968003 | ZC3H14 | zinc finger CCCH-type containing 14 [Source:HGNC Symbol;Acc:HGNC:20509] |
| ENSG00000101197 | HGLibA_04527 | 16 | -0.989968003 | BIRC7 | baculoviral IAP repeat containing 7 [Source:HGNC Symbol;Acc:HGNC:13702] |
| ENSG00000101200 | HGLibA_03898 | 16 | -0.989968003 | AVP | arginine vasopressin [Source:HGNC Symbol;Acc:HGNC:894] |
| ENSG00000101336 | HGLibA_20968 | 16 | -0.989968003 | HCK | Src family tyrosine kinase [Source:HGNC Symbol;Acc:HGNC:4840]" |
| ENSG00000101464 | HGLibA_36591 | 16 | -0.989968003 | PIGU | phosphatidylinositol glycan anchor biosynthesis class U [Source:HGNC Symbol;Acc:HGNC:15791] |
| ENSG00000101695 | HGLibA_41492 | 16 | -0.989968003 | RNF125 | ring finger protein 125 [Source:HGNC Symbol;Acc:HGNC:21150] |
| ENSG00000102554 | HGLibA_25075 | 16 | -0.989968003 | KLF5 | Kruppel like factor 5 [Source:HGNC Symbol;Acc:HGNC:6349] |
| ENSG00000102858 | HGLibA_29171 | 16 | -0.989968003 | MGRN1 | mahogunin ring finger 1 [Source:HGNC Symbol;Acc:HGNC:20254] |
| ENSG00000102935 | HGLibA_55960 | 16 | -0.989968003 | ZNF423 | zinc finger protein 423 [Source:HGNC Symbol;Acc:HGNC:16762] |
| ENSG00000104043 | HGLibA_03790 | 16 | -0.989968003 | ATP8B4 | ATPase phospholipid transporting 8B4 (putative) [Source:HGNC Symbol;Acc:HGNC:13536] |
| ENSG00000104738 | HGLibA_28612 | 16 | -0.989968003 | MCM4 | minichromosome maintenance complex component 4 [Source:HGNC Symbol;Acc:HGNC:6947] |
| ENSG00000104880 | HGLibA_02827 | 16 | -0.989968003 | ARHGEF18 | Rho/Rac guanine nucleotide exchange factor 18 [Source:HGNC Symbol;Acc:HGNC:17090] |
| ENSG00000104894 | HGLibA_08363 | 16 | -0.989968003 | CD37 | CD37 molecule [Source:HGNC Symbol;Acc:HGNC:1666] |
| ENSG00000105254 | HGLibA_48580 | 16 | -0.989968003 | TBCB | tubulin folding cofactor B [Source:HGNC Symbol;Acc:HGNC:1989] |
| ENSG00000105613 | HGLibA_28399 | 16 | -0.989968003 | MAST1 | microtubule associated serine/threonine kinase 1 [Source:HGNC Symbol;Acc:HGNC:19034] |
| ENSG00000107077 | HGLibA_24512 | 16 | -0.989968003 | KDM4C | lysine demethylase 4C [Source:HGNC Symbol;Acc:HGNC:17071] |
| ENSG00000108256 | HGLibA_33002 | 16 | -0.989968003 | NUFIP2 | nuclear FMR1 interacting protein 2 [Source:HGNC Symbol;Acc:HGNC:17634] |
| ENSG00000108561 | HGLibA_05918 | 16 | -0.989968003 | C1QBP | complement C1q binding protein [Source:HGNC Symbol;Acc:HGNC:1243] |
| ENSG00000108861 | HGLibA_14177 | 16 | -0.989968003 | DUSP3 | dual specificity phosphatase 3 [Source:HGNC Symbol;Acc:HGNC:3069] |
| ENSG00000111199 | HGLibA_51791 | 16 | -0.989968003 | TRPV4 | transient receptor potential cation channel subfamily V member 4 [Source:HGNC Symbol;Acc:HGNC:18083] |
| ENSG00000111229 | HGLibA_03084 | 16 | -0.989968003 | ARPC3 | actin related protein 2/3 complex subunit 3 [Source:HGNC Symbol;Acc:HGNC:706] |
| ENSG00000111249 | HGLibA_11806 | 16 | -0.989968003 | CUX2 | cut like homeobox 2 [Source:HGNC Symbol;Acc:HGNC:19347] |
| ENSG00000112299 | HGLibA_53711 | 16 | -0.989968003 | VNN1 | vanin 1 [Source:HGNC Symbol;Acc:HGNC:12705] |
| ENSG00000112308 | HGLibA_06504 | 16 | -0.989968003 | C6orf62 | chromosome 6 open reading frame 62 [Source:HGNC Symbol;Acc:HGNC:20998] |
| ENSG00000113119 | HGLibA_49796 | 16 | -0.989968003 | TMCO6 | transmembrane and coiled-coil domains 6 [Source:HGNC Symbol;Acc:HGNC:28814] |
| ENSG00000113328 | HGLibA_08092 | 16 | -0.989968003 | CCNG1 | cyclin G1 [Source:HGNC Symbol;Acc:HGNC:1592] |
| ENSG00000114353 | HGLibA_19455 | 16 | -0.989968003 | GNAI2 | G protein subunit alpha i2 [Source:HGNC Symbol;Acc:HGNC:4385] |
| ENSG00000115109 | HGLibA_15258 | 16 | -0.989968003 | EPB41L5 | erythrocyte membrane protein band 4.1 like 5 [Source:HGNC Symbol;Acc:HGNC:19819] |
| ENSG00000115423 | HGLibA_13491 | 16 | -0.989968003 | DNAH6 | dynein axonemal heavy chain 6 [Source:HGNC Symbol;Acc:HGNC:2951] |
| ENSG00000115590 | HGLibA_23050 | 16 | -0.989968003 | IL1R2 | interleukin 1 receptor type 2 [Source:HGNC Symbol;Acc:HGNC:5994] |
| ENSG00000115761 | HGLibA_32257 | 16 | -0.989968003 | NOL10 | nucleolar protein 10 [Source:HGNC Symbol;Acc:HGNC:25862] |
| ENSG00000119878 | HGLibA_11162 | 16 | -0.989968003 | CRIPT | CXXC repeat containing interactor of PDZ3 domain [Source:HGNC Symbol;Acc:HGNC:14312] |
| ENSG00000120253 | HGLibA_33053 | 16 | -0.989968003 | NUP43 | nucleoporin 43 [Source:HGNC Symbol;Acc:HGNC:21182] |
| ENSG00000124831 | HGLibA_27450 | 16 | -0.989968003 | LRRFIP1 | LRR binding FLII interacting protein 1 [Source:HGNC Symbol;Acc:HGNC:6702] |
| ENSG00000125741 | HGLibA_33406 | 16 | -0.989968003 | OPA3 | outer mitochondrial membrane lipid metabolism regulator [Source:HGNC Symbol;Acc:HGNC:8142]" |
| ENSG00000127419 | HGLibA_50068 | 16 | -0.989968003 | TMEM175 | transmembrane protein 175 [Source:HGNC Symbol;Acc:HGNC:28709] |
| ENSG00000127947 | HGLibA_39436 | 16 | -0.989968003 | PTPN12 | non-receptor type 12 [Source:HGNC Symbol;Acc:HGNC:9645]" |
| ENSG00000129116 | HGLibA_35064 | 16 | -0.989968003 | PALLD | cytoskeletal associated protein [Source:HGNC Symbol;Acc:HGNC:17068]" |
| ENSG00000129538 | HGLibA_41389 | 16 | -0.989968003 | RNASE1 | pancreatic [Source:HGNC Symbol;Acc:HGNC:10044]" |
| ENSG00000129691 | HGLibA_03297 | 16 | -0.989968003 | ASH2L | histone lysine methyltransferase complex subunit [Source:HGNC Symbol;Acc:HGNC:744]" |
| ENSG00000130377 | HGLibA_00544 | 16 | -0.989968003 | ACSBG2 | acyl-CoA synthetase bubblegum family member 2 [Source:HGNC Symbol;Acc:HGNC:24174] |
| ENSG00000130962 | HGLibA_38870 | 16 | -0.989968003 | PRRG1 | proline rich and Gla domain 1 [Source:HGNC Symbol;Acc:HGNC:9469] |
| ENSG00000131126 | HGLibA_49050 | 16 | -0.989968003 | TEX101 | testis expressed 101 [Source:HGNC Symbol;Acc:HGNC:30722] |
| ENSG00000131370 | HGLibA_43933 | 16 | -0.989968003 | SH3BP5 | SH3 domain binding protein 5 [Source:HGNC Symbol;Acc:HGNC:10827] |
| ENSG00000131791 | HGLibA_38500 | 16 | -0.989968003 | PRKAB2 | protein kinase AMP-activated non-catalytic subunit beta 2 [Source:HGNC Symbol;Acc:HGNC:9379] |
| ENSG00000133250 | HGLibA_55938 | 16 | -0.989968003 | ZNF414 | zinc finger protein 414 [Source:HGNC Symbol;Acc:HGNC:20630] |
| ENSG00000133318 | HGLibA_42404 | 16 | -0.989968003 | RTN3 | reticulon 3 [Source:HGNC Symbol;Acc:HGNC:10469] |
| ENSG00000133392 | HGLibA_30709 | 16 | -0.989968003 | MYH11 | myosin heavy chain 11 [Source:HGNC Symbol;Acc:HGNC:7569] |
| ENSG00000135316 | HGLibA_47966 | 16 | -0.989968003 | SYNCRIP | synaptotagmin binding cytoplasmic RNA interacting protein [Source:HGNC Symbol;Acc:HGNC:16918] |
| ENSG00000136003 | HGLibA_23652 | 16 | -0.989968003 | ISCU | iron-sulfur cluster assembly enzyme [Source:HGNC Symbol;Acc:HGNC:29882] |
| ENSG00000136280 | HGLibA_08046 | 16 | -0.989968003 | CCM2 | CCM2 scaffold protein [Source:HGNC Symbol;Acc:HGNC:21708] |
| ENSG00000137073 | HGLibA_52551 | 16 | -0.989968003 | UBAP2 | ubiquitin associated protein 2 [Source:HGNC Symbol;Acc:HGNC:14185] |
| ENSG00000138231 | HGLibA_12404 | 16 | -0.989968003 | DBR1 | debranching RNA lariats 1 [Source:HGNC Symbol;Acc:HGNC:15594] |
| ENSG00000138347 | HGLibA_30909 | 16 | -0.989968003 | MYPN | myopalladin [Source:HGNC Symbol;Acc:HGNC:23246] |
| ENSG00000138613 | HGLibA_02416 | 16 | -0.989968003 | APH1B | gamma-secretase subunit [Source:HGNC Symbol;Acc:HGNC:24080]" |
| ENSG00000138650 | HGLibA_35389 | 16 | -0.989968003 | PCDH10 | protocadherin 10 [Source:HGNC Symbol;Acc:HGNC:13404] |
| ENSG00000140522 | HGLibA_41342 | 16 | -0.989968003 | RLBP1 | retinaldehyde binding protein 1 [Source:HGNC Symbol;Acc:HGNC:10024] |
| ENSG00000141337 | HGLibA_03144 | 16 | -0.989968003 | ARSG | arylsulfatase G [Source:HGNC Symbol;Acc:HGNC:24102] |
| ENSG00000143256 | HGLibA_36141 | 16 | -0.989968003 | PFDN2 | prefoldin subunit 2 [Source:HGNC Symbol;Acc:HGNC:8867] |
| ENSG00000143498 | HGLibA_48213 | 16 | -0.989968003 | TAF1A | RNA polymerase I subunit A [Source:HGNC Symbol;Acc:HGNC:11532]" |
| ENSG00000143933 | HGLibA_07025 | 16 | -0.989968003 | CALM2 | calmodulin 2 [Source:HGNC Symbol;Acc:HGNC:1445] |
| ENSG00000144560 | HGLibA_53644 | 16 | -0.989968003 | VGLL4 | vestigial like family member 4 [Source:HGNC Symbol;Acc:HGNC:28966] |
| ENSG00000144647 | HGLibA_37661 | 16 | -0.989968003 | POMGNT2 | 4-) [Source:HGNC Symbol;Acc:HGNC:25902]" |
| ENSG00000144843 | HGLibA_01026 | 16 | -0.989968003 | ADPRH | ADP-ribosylarginine hydrolase [Source:HGNC Symbol;Acc:HGNC:269] |
| ENSG00000147601 | HGLibA_49013 | 16 | -0.989968003 | TERF1 | telomeric repeat binding factor 1 [Source:HGNC Symbol;Acc:HGNC:11728] |

| | | | | | |
|---|---|---|---|---|---|
| ENSG00000147669 | HGLibA_37601 | 16 | -0.989968003 | POLR2K | RNA polymerase II subunit K [Source:HGNC Symbol;Acc:HGNC:9198] |
| ENSG00000148814 | HGLibA_27297 | 16 | -0.989968003 | LRRC27 | leucine rich repeat containing 27 [Source:HGNC Symbol;Acc:HGNC:29346] |
| ENSG00000149212 | HGLibA_43625 | 16 | -0.989968003 | SESN3 | sestrin 3 [Source:HGNC Symbol;Acc:HGNC:23060] |
| ENSG00000151572 | HGLibA_02181 | 16 | -0.989968003 | ANO4 | anoctamin 4 [Source:HGNC Symbol;Acc:HGNC:23837] |
| ENSG00000151726 | HGLibA_00554 | 16 | -0.989968003 | ACSL1 | acyl-CoA synthetase long chain family member 1 [Source:HGNC Symbol;Acc:HGNC:3569] |
| ENSG00000151893 | HGLibA_06955 | 16 | -0.989968003 | CACUL1 | CDK2 associated cullin domain 1 [Source:HGNC Symbol;Acc:HGNC:23727] |
| ENSG00000153707 | HGLibA_39497 | 16 | -0.989968003 | PTPRD | receptor type D [Source:HGNC Symbol;Acc:HGNC:9668]" |
| ENSG00000154080 | HGLibA_09651 | 16 | -0.989968003 | CHST9 | carbohydrate sulfotransferase 9 [Source:HGNC Symbol;Acc:HGNC:19898] |
| ENSG00000154262 | HGLibA_00118 | 16 | -0.989968003 | ABCA6 | ATP binding cassette subfamily A member 6 [Source:HGNC Symbol;Acc:HGNC:36] |
| ENSG00000155975 | HGLibA_53775 | 16 | -0.989968003 | VPS37A | ESCRT-I subunit [Source:HGNC Symbol;Acc:HGNC:24928]" |
| ENSG00000157379 | HGLibA_13117 | 16 | -0.989968003 | DHRS1 | dehydrogenase/reductase 1 [Source:HGNC Symbol;Acc:HGNC:16445] |
| ENSG00000158022 | HGLibA_51573 | 16 | -0.989968003 | TRIM63 | tripartite motif containing 63 [Source:HGNC Symbol;Acc:HGNC:16007] |
| ENSG00000158169 | HGLibA_16836 | 16 | -0.989968003 | FANCC | FA complementation group C [Source:HGNC Symbol;Acc:HGNC:3584] |
| ENSG00000161526 | HGLibA_42725 | 16 | -0.989968003 | SAP30BP | SAP30 binding protein [Source:HGNC Symbol;Acc:HGNC:30785] |
| ENSG00000161652 | HGLibA_23895 | 16 | -0.989968003 | IZUMO2 | IZUMO family member 2 [Source:HGNC Symbol;Acc:HGNC:28518] |
| ENSG00000162607 | HGLibA_53190 | 16 | -0.989968003 | USP1 | ubiquitin specific peptidase 1 [Source:HGNC Symbol;Acc:HGNC:12607] |
| ENSG00000163798 | HGLibA_45212 | 16 | -0.989968003 | SLC4A1AP | solute carrier family 4 member 1 adaptor protein [Source:HGNC Symbol;Acc:HGNC:13813] |
| ENSG00000164663 | HGLibA_53385 | 16 | -0.989968003 | USP49 | ubiquitin specific peptidase 49 [Source:HGNC Symbol;Acc:HGNC:20078] |
| ENSG00000165792 | HGLibA_28967 | 16 | -0.989968003 | METTL17 | methyltransferase like 17 [Source:HGNC Symbol;Acc:HGNC:19280] |
| ENSG00000165794 | HGLibA_45107 | 16 | -0.989968003 | SLC39A2 | solute carrier family 39 member 2 [Source:HGNC Symbol;Acc:HGNC:17127] |
| ENSG00000165973 | HGLibA_31684 | 16 | -0.989968003 | NELL1 | neural EGFL like 1 [Source:HGNC Symbol;Acc:HGNC:7750] |
| ENSG00000165983 | HGLibA_39297 | 16 | -0.989968003 | PTER | phosphotriesterase related [Source:HGNC Symbol;Acc:HGNC:9590] |
| ENSG00000166689 | HGLibA_37081 | 16 | -0.989968003 | PLEKHA7 | pleckstrin homology domain containing A7 [Source:HGNC Symbol;Acc:HGNC:27049] |
| ENSG00000166889 | HGLibA_35283 | 16 | -0.989968003 | PATL1 | processing body mRNA decay factor [Source:HGNC Symbol;Acc:HGNC:26721]" |
| ENSG00000167377 | HGLibA_55634 | 16 | -0.989968003 | ZNF23 | zinc finger protein 23 [Source:HGNC Symbol;Acc:HGNC:13023] |
| ENSG00000168421 | HGLibA_41192 | 16 | -0.989968003 | RHOH | ras homolog family member H [Source:HGNC Symbol;Acc:HGNC:686] |
| ENSG00000168944 | HGLibA_09100 | 16 | -0.989968003 | CEP120 | centrosomal protein 120 [Source:HGNC Symbol;Acc:HGNC:26690] |
| ENSG00000169583 | HGLibA_10027 | 16 | -0.989968003 | CLIC3 | chloride intracellular channel 3 [Source:HGNC Symbol;Acc:HGNC:2064] |
| ENSG00000169621 | HGLibA_02431 | 16 | -0.989968003 | APLF | aprataxin and PNKP like factor [Source:HGNC Symbol;Acc:HGNC:28724] |
| ENSG00000169641 | HGLibA_27638 | 16 | -0.989968003 | LUZP1 | leucine zipper protein 1 [Source:HGNC Symbol;Acc:HGNC:14985] |
| ENSG00000169684 | HGLibA_09577 | 16 | -0.989968003 | CHRNA5 | cholinergic receptor nicotinic alpha 5 subunit [Source:HGNC Symbol;Acc:HGNC:1959] |
| ENSG00000170145 | HGLibA_44164 | 16 | -0.989968003 | SIK2 | salt inducible kinase 2 [Source:HGNC Symbol;Acc:HGNC:21680] |
| ENSG00000170310 | HGLibA_47659 | 16 | -0.989968003 | STX8 | syntaxin 8 [Source:HGNC Symbol;Acc:HGNC:11443] |
| ENSG00000170382 | HGLibA_27472 | 16 | -0.989968003 | LRRN2 | leucine rich repeat neuronal 2 [Source:HGNC Symbol;Acc:HGNC:16914] |
| ENSG00000170498 | HGLibA_25009 | 16 | -0.989968003 | KISS1 | KiSS-1 metastasis suppressor [Source:HGNC Symbol;Acc:HGNC:6341] |
| ENSG00000170876 | HGLibA_50402 | 16 | -0.989968003 | TMEM43 | transmembrane protein 43 [Source:HGNC Symbol;Acc:HGNC:28472] |
| ENSG00000171522 | HGLibA_39322 | 16 | -0.989968003 | PTGER4 | prostaglandin E receptor 4 [Source:HGNC Symbol;Acc:HGNC:9596] |
| ENSG00000171659 | HGLibA_20027 | 16 | -0.989968003 | GPR34 | G protein-coupled receptor 34 [Source:HGNC Symbol;Acc:HGNC:4490] |
| ENSG00000171723 | HGLibA_19803 | 16 | -0.989968003 | GPHN | gephyrin [Source:HGNC Symbol;Acc:HGNC:15465] |
| ENSG00000172352 | HGLibA_08908 | 16 | -0.989968003 | CDY1B | chromodomain Y-linked 1B [Source:HGNC Symbol;Acc:HGNC:23920] |
| ENSG00000172409 | HGLibA_10094 | 16 | -0.989968003 | CLP1 | cleavage and polyadenylation factor I subunit 1 [Source:HGNC Symbol;Acc:HGNC:16999] |
| ENSG00000173065 | HGLibA_16524 | 16 | -0.989968003 | FAM222B | family with sequence similarity 222 member B [Source:HGNC Symbol;Acc:HGNC:25563] |
| ENSG00000173705 | HGLibA_47857 | 16 | -0.989968003 | SUSD5 | sushi domain containing 5 [Source:HGNC Symbol;Acc:HGNC:29061] |
| ENSG00000174827 | HGLibA_35992 | 16 | -0.989968003 | PDZK1 | PDZ domain containing 1 [Source:HGNC Symbol;Acc:HGNC:8821] |
| ENSG00000174876 | HGLibA_01844 | 16 | -0.989968003 | AMY1B | alpha 1B (salivary) [Source:HGNC Symbol;Acc:HGNC:475]" |
| ENSG00000175311 | HGLibA_02160 | 16 | -0.989968003 | ANKS4B | ankyrin repeat and sterile alpha motif domain containing 4B [Source:HGNC Symbol;Acc:HGNC:26795] |
| ENSG00000177426 | HGLibA_49238 | 16 | -0.989968003 | TGIF1 | TGFB induced factor homeobox 1 [Source:HGNC Symbol;Acc:HGNC:11776] |
| ENSG00000177889 | HGLibA_52637 | 16 | -0.989968003 | UBE2N | ubiquitin conjugating enzyme E2 N [Source:HGNC Symbol;Acc:HGNC:12492] |
| ENSG00000178645 | HGLibA_05073 | 16 | -0.989968003 | C10orf53 | chromosome 10 open reading frame 53 [Source:HGNC Symbol;Acc:HGNC:27421] |
| ENSG00000178966 | HGLibA_41372 | 16 | -0.989968003 | RMI1 | RecQ mediated genome instability 1 [Source:HGNC Symbol;Acc:HGNC:25764] |
| ENSG00000178997 | HGLibA_15716 | 16 | -0.989968003 | EXD1 | exonuclease 3'-5' domain containing 1 [Source:HGNC Symbol;Acc:HGNC:28507] |
| ENSG00000179695 | HGLibA_34384 | 16 | -0.989968003 | OR6C2 | olfactory receptor family 6 subfamily C member 2 [Source:HGNC Symbol;Acc:HGNC:15436] |
| ENSG00000180089 | HGLibA_50529 | 16 | -0.989968003 | TMEM86B | transmembrane protein 86B [Source:HGNC Symbol;Acc:HGNC:28448] |
| ENSG00000180530 | HGLibA_32692 | 16 | -0.989968003 | NRIP1 | nuclear receptor interacting protein 1 [Source:HGNC Symbol;Acc:HGNC:8001] |
| ENSG00000180626 | HGLibA_56345 | 16 | -0.989968003 | ZNF594 | zinc finger protein 594 [Source:HGNC Symbol;Acc:HGNC:29392] |
| ENSG00000180818 | HGLibA_21955 | 16 | -0.989968003 | HOXC10 | homeobox C10 [Source:HGNC Symbol;Acc:HGNC:5122] |
| ENSG00000181804 | HGLibA_45431 | 16 | -0.989968003 | SLC9A9 | solute carrier family 9 member A9 [Source:HGNC Symbol;Acc:HGNC:20653] |
| ENSG00000182150 | HGLibA_15451 | 16 | -0.989968003 | ERCC6L2 | ERCC excision repair 6 like 2 [Source:HGNC Symbol;Acc:HGNC:26922] |
| ENSG00000183010 | HGLibA_39674 | 16 | -0.989968003 | PYCR1 | pyrroline-5-carboxylate reductase 1 [Source:HGNC Symbol;Acc:HGNC:9721] |
| ENSG00000183023 | HGLibA_45389 | 16 | -0.989968003 | SLC8A1 | solute carrier family 8 member A1 [Source:HGNC Symbol;Acc:HGNC:11068] |
| ENSG00000183269 | HGLibA_34166 | 16 | -0.989968003 | OR52E8 | olfactory receptor family 52 subfamily E member 8 [Source:HGNC Symbol;Acc:HGNC:15217] |
| ENSG00000183682 | HGLibA_04620 | 16 | -0.989968003 | BMP8A | bone morphogenetic protein 8a [Source:HGNC Symbol;Acc:HGNC:21650] |
| ENSG00000184058 | HGLibA_48640 | 16 | -0.989968003 | TBX1 | T-box 1 [Source:HGNC Symbol;Acc:HGNC:11592] |
| ENSG00000185619 | HGLibA_35599 | 16 | -0.989968003 | PCGF3 | polycomb group ring finger 3 [Source:HGNC Symbol;Acc:HGNC:10066] |
| ENSG00000187733 | HGLibA_01848 | 16 | -0.989968003 | AMY1C | alpha 1C (salivary) [Source:HGNC Symbol;Acc:HGNC:476]" |
| ENSG00000187833 | HGLibA_06184 | 16 | -0.989968003 | C2orf78 | chromosome 2 open reading frame 78 [Source:HGNC Symbol;Acc:HGNC:34349] |
| ENSG00000187969 | HGLibA_55011 | 16 | -0.989968003 | ZCCHC13 | zinc finger CCHC-type containing 13 [Source:HGNC Symbol;Acc:HGNC:31749] |
| ENSG00000188277 | HGLibA_05431 | 16 | -0.989968003 | C15orf62 | chromosome 15 open reading frame 62 [Source:HGNC Symbol;Acc:HGNC:34489] |
| ENSG00000188883 | HGLibA_25311 | 16 | -0.989968003 | KLRG2 | killer cell lectin like receptor G2 [Source:HGNC Symbol;Acc:HGNC:24778] |
| ENSG00000189120 | HGLibA_46310 | 16 | -0.989968003 | SP6 | Sp6 transcription factor [Source:HGNC Symbol;Acc:HGNC:14530] |
| ENSG00000196391 | HGLibA_56716 | 16 | -0.989968003 | ZNF774 | zinc finger protein 774 [Source:HGNC Symbol;Acc:HGNC:33108] |
| ENSG00000196510 | HGLibA_01890 | 16 | -0.989968003 | ANAPC7 | anaphase promoting complex subunit 7 [Source:HGNC Symbol;Acc:HGNC:17380] |
| ENSG00000197448 | HGLibA_20470 | 16 | -0.989968003 | GSTK1 | glutathione S-transferase kappa 1 [Source:HGNC Symbol;Acc:HGNC:16906] |
| ENSG00000197782 | HGLibA_56731 | 16 | -0.989968003 | ZNF780A | zinc finger protein 780A [Source:HGNC Symbol;Acc:HGNC:27603] |
| ENSG00000198185 | HGLibA_55822 | 16 | -0.989968003 | ZNF334 | zinc finger protein 334 [Source:HGNC Symbol;Acc:HGNC:15806] |
| ENSG00000198231 | HGLibA_12713 | 16 | -0.989968003 | DDX42 | DEAD-box helicase 42 [Source:HGNC Symbol;Acc:HGNC:18676] |
| ENSG00000198242 | HGLibA_41908 | 16 | -0.989968003 | RPL23A | ribosomal protein L23a [Source:HGNC Symbol;Acc:HGNC:10317] |
| ENSG00000198759 | HGLibA_14623 | 16 | -0.989968003 | EGFL6 | EGF like domain multiple 6 [Source:HGNC Symbol;Acc:HGNC:3235] |
| ENSG00000204271 | HGLibA_46675 | 16 | -0.989968003 | SPIN3 | spindlin family member 3 [Source:HGNC Symbol;Acc:HGNC:27272] |
| ENSG00000204344 | HGLibA_47469 | 16 | -0.989968003 | STK19 | serine/threonine kinase 19 [Source:HGNC Symbol;Acc:HGNC:11398] |
| ENSG00000204371 | HGLibA_14687 | 16 | -0.989968003 | EHMT2 | euchromatic histone lysine methyltransferase 2 [Source:HGNC Symbol;Acc:HGNC:14129] |
| ENSG00000204869 | HGLibA_22848 | 16 | -0.989968003 | IGFL4 | IGF like family member 4 [Source:HGNC Symbol;Acc:HGNC:32931] |
| ENSG00000213079 | HGLibA_42830 | 16 | -0.989968003 | SCAF8 | SR-related CTD associated factor 8 [Source:HGNC Symbol;Acc:HGNC:20959] |
| ENSG00000214026 | HGLibA_29932 | 16 | -0.989968003 | MRPL23 | mitochondrial ribosomal protein L23 [Source:HGNC Symbol;Acc:HGNC:10322] |
| ENSG00000214360 | HGLibA_14555 | 16 | -0.989968003 | EFCAB9 | EF-hand calcium binding domain 9 [Source:HGNC Symbol;Acc:HGNC:34530] |
| ENSG00000214513 | HGLibA_32356 | 16 | -0.989968003 | NOTO | notochord homeobox [Source:HGNC Symbol;Acc:HGNC:31839] |
| ENSG00000215784 | HGLibA_16713 | 16 | -0.989968003 | FAM72D | family with sequence similarity 72 member D [Source:HGNC Symbol;Acc:HGNC:33593] |
| ENSG00000216937 | HGLibA_07847 | 16 | -0.989968003 | CCDC7 | coiled-coil domain containing 7 [Source:HGNC Symbol;Acc:HGNC:26533] |
| ENSG00000221818 | HGLibA_14353 | 16 | -0.989968003 | EBF2 | EBF transcription factor 2 [Source:HGNC Symbol;Acc:HGNC:19090] |
| ENSG00000221933 | HGLibA_33730 | 16 | -0.989968003 | OR2A25 | olfactory receptor family 2 subfamily A member 25 [Source:HGNC Symbol;Acc:HGNC:19562] |
| ENSG00000232382 | HGLibA_34311 | 16 | -0.989968003 | OR5K1 | olfactory receptor family 5 subfamily K member 1 [Source:HGNC Symbol;Acc:HGNC:8349] |
| ENSG00000235109 | HGLibA_57020 | 16 | -0.989968003 | ZSCAN31 | zinc finger and SCAN domain containing 31 [Source:HGNC Symbol;Acc:HGNC:14097] |
| ENSG00000240682 | HGLibA_23695 | 16 | -0.989968003 | ISY1 | ISY1 splicing factor homolog [Source:HGNC Symbol;Acc:HGNC:29201] |
| ENSG00000241258 | HGLibA_11080 | 16 | -0.989968003 | CRCP | CGRP receptor component [Source:HGNC Symbol;Acc:HGNC:17888] |
| ENSG00000243678 | HGLibA_32158 | 16 | -0.989968003 | NME2 | NME/NM23 nucleoside diphosphate kinase 2 [Source:HGNC Symbol;Acc:HGNC:7850] |
| ENSG00000255298 | HGLibA_34523 | 16 | -0.989968003 | OR8G5 | olfactory receptor family 8 subfamily G member 5 [Source:HGNC Symbol;Acc:HGNC:19622] |
| ENSG00000278299 | HGLibA_48544 | 16 | -0.989968003 | TBC1D3C | TBC1 domain family member 3C [Source:HGNC Symbol;Acc:HGNC:24889] |
| ENSG00000278889 | HGLibA_33847 | 16 | -0.989968003 | OR2S2 | olfactory receptor family 2 subfamily S member 2 (gene/pseudogene) [Source:HGNC Symbol;Acc:HGNC:8276] |
| GAGE6 | HGLibA_18485 | 16 | -0.989968003 | GAGE6 | - |
| GNB2L1 | HGLibA_19494 | 16 | -0.989968003 | GNB2L1 | - |
| GPER | HGLibA_19791 | 16 | -0.989968003 | GPER | - |
| HMP19 | HGLibA_21754 | 16 | -0.989968003 | HMP19 | - |
| hsa-mir-1228 | HGLibA_57310 | 16 | -0.989968003 | hsa-mir-1228 | - |
| hsa-mir-1270-2 | HGLibA_57518 | 16 | -0.989968003 | hsa-mir-1270-2 | - |
| hsa-mir-1301 | HGLibA_57678 | 16 | -0.989968003 | hsa-mir-1301 | - |
| hsa-mir-193b | HGLibA_58075 | 16 | -0.989968003 | hsa-mir-193b | - |
| hsa-mir-216a | HGLibA_58257 | 16 | -0.989968003 | hsa-mir-216a | - |
| hsa-mir-3185 | HGLibA_58837 | 16 | -0.989968003 | hsa-mir-3185 | - |
| hsa-mir-4493 | HGLibA_60389 | 16 | -0.989968003 | hsa-mir-4493 | - |
| hsa-mir-4517 | HGLibA_60513 | 16 | -0.989968003 | hsa-mir-4517 | - |
| hsa-mir-4714 | HGLibA_60956 | 16 | -0.989968003 | hsa-mir-4714 | - |
| hsa-mir-4747 | HGLibA_61087 | 16 | -0.989968003 | hsa-mir-4747 | - |
| hsa-mir-548ae-2 | HGLibA_61871 | 16 | -0.989968003 | hsa-mir-548ae-2 | - |
| hsa-mir-548am | HGLibA_61900 | 16 | -0.989968003 | hsa-mir-548am | - |
| hsa-mir-548ap | HGLibA_61912 | 16 | -0.989968003 | hsa-mir-548ap | - |
| hsa-mir-548as | HGLibA_61922 | 16 | -0.989968003 | hsa-mir-548as | - |
| hsa-mir-548c | HGLibA_61957 | 16 | -0.989968003 | hsa-mir-548c | - |
| hsa-mir-5571 | HGLibA_62148 | 16 | -0.989968003 | hsa-mir-5571 | - |
| hsa-mir-5571 | HGLibA_62146 | 16 | -0.989968003 | hsa-mir-5571 | - |
| hsa-mir-581 | HGLibA_62423 | 16 | -0.989968003 | hsa-mir-581 | - |
| hsa-mir-612 | HGLibA_62641 | 16 | -0.989968003 | hsa-mir-612 | - |
| hsa-mir-615 | HGLibA_62696 | 16 | -0.989968003 | hsa-mir-615 | - |
| hsa-mir-644a | HGLibA_62820 | 16 | -0.989968003 | hsa-mir-644a | - |
| hsa-mir-6780b | HGLibA_63299 | 16 | -0.989968003 | hsa-mir-6780b | - |
| hsa-mir-6787 | HGLibA_63326 | 16 | -0.989968003 | hsa-mir-6787 | - |
| hsa-mir-6880 | HGLibA_63704 | 16 | -0.989968003 | hsa-mir-6880 | - |
| hsa-mir-7977 | HGLibA_64038 | 16 | -0.989968003 | hsa-mir-7977 | - |
| hsa-mir-8053 | HGLibA_64051 | 16 | -0.989968003 | hsa-mir-8053 | - |
| hsa-mir-8053 | HGLibA_64052 | 16 | -0.989968003 | hsa-mir-8053 | - |
| hsa-mir-8089 | HGLibA_64198 | 16 | -0.989968003 | hsa-mir-8089 | - |
| MARS | HGLibA_28377 | 16 | -0.989968003 | MARS | - |
| TMEM14E | HGLibA_49989 | 16 | -0.989968003 | TMEM14E | - |
| ATP5C1 | HGLibA_03645 | 15 | -0.999013346 | ATP5C1 | - |
| C17orf77 | HGLibA_05576 | 15 | -0.999013346 | C17orf77 | - |
| C1orf228 | HGLibA_05839 | 15 | -0.999013346 | C1orf228 | - |

| | | | | | |
|---|---|---|---|---|---|
| C4orf29 | HGLibA_06315 | 15 | -0.999013346 | C4orf29 | - |
| C8orf22 | HGLibA_06602 | 15 | -0.999013346 | C8orf22 | - |
| C9orf3 | HGLibA_06723 | 15 | -0.999013346 | C9orf3 | - |
| DDX26B | HGLibA_12683 | 15 | -0.999013346 | DDX26B | - |
| ENSG00000003147 | HGLibA_22488 | 15 | -0.999013346 | ICA1 | islet cell autoantigen 1 [Source:HGNC Symbol;Acc:HGNC:5343] |
| ENSG00000013503 | HGLibA_37613 | 15 | -0.999013346 | POLR3B | RNA polymerase III subunit B [Source:HGNC Symbol;Acc:HGNC:30348] |
| ENSG00000030582 | HGLibA_20365 | 15 | -0.999013346 | GRN | granulin precursor [Source:HGNC Symbol;Acc:HGNC:4601] |
| ENSG00000040275 | HGLibA_46570 | 15 | -0.999013346 | SPDL1 | spindle apparatus coiled-coil protein 1 [Source:HGNC Symbol;Acc:HGNC:26010] |
| ENSG00000062096 | HGLibA_03139 | 15 | -0.999013346 | ARSF | arylsulfatase F [Source:HGNC Symbol;Acc:HGNC:721] |
| ENSG00000063244 | HGLibA_52498 | 15 | -0.999013346 | U2AF2 | U2 small nuclear RNA auxiliary factor 2 [Source:HGNC Symbol;Acc:HGNC:23156] |
| ENSG00000073060 | HGLibA_42868 | 15 | -0.999013346 | SCARB1 | scavenger receptor class B member 1 [Source:HGNC Symbol;Acc:HGNC:1664] |
| ENSG00000080822 | HGLibA_09917 | 15 | -0.999013346 | CLDND1 | claudin domain containing 1 [Source:HGNC Symbol;Acc:HGNC:1322] |
| ENSG00000081059 | HGLibA_48790 | 15 | -0.999013346 | TCF7 | transcription factor 7 [Source:HGNC Symbol;Acc:HGNC:11639] |
| ENSG00000081721 | HGLibA_14137 | 15 | -0.999013346 | DUSP12 | dual specificity phosphatase 12 [Source:HGNC Symbol;Acc:HGNC:3067] |
| ENSG00000081800 | HGLibA_44385 | 15 | -0.999013346 | SLC13A1 | solute carrier family 13 member 1 [Source:HGNC Symbol;Acc:HGNC:10916] |
| ENSG00000087495 | HGLibA_36302 | 15 | -0.999013346 | PHACTR3 | phosphatase and actin regulator 3 [Source:HGNC Symbol;Acc:HGNC:15833] |
| ENSG00000088538 | HGLibA_13743 | 15 | -0.999013346 | DOCK3 | dedicator of cytokinesis 3 [Source:HGNC Symbol;Acc:HGNC:2989] |
| ENSG00000088876 | HGLibA_55844 | 15 | -0.999013346 | ZNF343 | zinc finger protein 343 [Source:HGNC Symbol;Acc:HGNC:16017] |
| ENSG00000089737 | HGLibA_12679 | 15 | -0.999013346 | DDX24 | DEAD-box helicase 24 [Source:HGNC Symbol;Acc:HGNC:13266] |
| ENSG00000090534 | HGLibA_49384 | 15 | -0.999013346 | THPO | thrombopoietin [Source:HGNC Symbol;Acc:HGNC:11795] |
| ENSG00000099139 | HGLibA_35678 | 15 | -0.999013346 | PCSK5 | proprotein convertase subtilisin/kexin type 5 [Source:HGNC Symbol;Acc:HGNC:8747] |
| ENSG00000100448 | HGLibA_11707 | 15 | -0.999013346 | CTSG | cathepsin G [Source:HGNC Symbol;Acc:HGNC:2532] |
| ENSG00000101282 | HGLibA_42354 | 15 | -0.999013346 | RSPO4 | R-spondin 4 [Source:HGNC Symbol;Acc:HGNC:16175] |
| ENSG00000104427 | HGLibA_54938 | 15 | -0.999013346 | ZC2HC1A | zinc finger C2HC-type containing 1A [Source:HGNC Symbol;Acc:HGNC:24277] |
| ENSG00000104517 | HGLibA_52760 | 15 | -0.999013346 | UBR5 | ubiquitin protein ligase E3 component n-recognin 5 [Source:HGNC Symbol;Acc:HGNC:16806] |
| ENSG00000104611 | HGLibA_43898 | 15 | -0.999013346 | SH2D4A | SH2 domain containing 4A [Source:HGNC Symbol;Acc:HGNC:26102] |
| ENSG00000105619 | HGLibA_49191 | 15 | -0.999013346 | TFPT | TCF3 fusion partner [Source:HGNC Symbol;Acc:HGNC:13630] |
| ENSG00000105695 | HGLibA_27888 | 15 | -0.999013346 | MAG | myelin associated glycoprotein [Source:HGNC Symbol;Acc:HGNC:6783] |
| ENSG00000106100 | HGLibA_32243 | 15 | -0.999013346 | NOD1 | nucleotide binding oligomerization domain containing 1 [Source:HGNC Symbol;Acc:HGNC:16390] |
| ENSG00000106113 | HGLibA_11144 | 15 | -0.999013346 | CRHR2 | corticotropin releasing hormone receptor 2 [Source:HGNC Symbol;Acc:HGNC:2358] |
| ENSG00000107566 | HGLibA_15495 | 15 | -0.999013346 | ERLIN1 | ER lipid raft associated 1 [Source:HGNC Symbol;Acc:HGNC:16947] |
| ENSG00000108239 | HGLibA_48473 | 15 | -0.999013346 | TBC1D12 | TBC1 domain family member 12 [Source:HGNC Symbol;Acc:HGNC:29082] |
| ENSG00000108352 | HGLibA_40262 | 15 | -0.999013346 | RAPGEFL1 | Rap guanine nucleotide exchange factor like 1 [Source:HGNC Symbol;Acc:HGNC:17428] |
| ENSG00000111639 | HGLibA_30012 | 15 | -0.999013346 | MRPL51 | mitochondrial ribosomal protein L51 [Source:HGNC Symbol;Acc:HGNC:14044] |
| ENSG00000115257 | HGLibA_35676 | 15 | -0.999013346 | PCSK4 | proprotein convertase subtilisin/kexin type 4 [Source:HGNC Symbol;Acc:HGNC:8746] |
| ENSG00000115541 | HGLibA_22334 | 15 | -0.999013346 | HSPE1 | heat shock protein family E (Hsp10) member 1 [Source:HGNC Symbol;Acc:HGNC:5269] |
| ENSG00000116039 | HGLibA_03732 | 15 | -0.999013346 | ATP6V1B1 | ATPase H+ transporting V1 subunit B1 [Source:HGNC Symbol;Acc:HGNC:853] |
| ENSG00000116141 | HGLibA_28364 | 15 | -0.999013346 | MARK1 | microtubule affinity regulating kinase 1 [Source:HGNC Symbol;Acc:HGNC:6896] |
| ENSG00000118520 | HGLibA_02685 | 15 | -0.999013346 | ARG1 | arginase 1 [Source:HGNC Symbol;Acc:HGNC:663] |
| ENSG00000118785 | HGLibA_46770 | 15 | -0.999013346 | SPP1 | secreted phosphoprotein 1 [Source:HGNC Symbol;Acc:HGNC:11255] |
| ENSG00000119547 | HGLibA_33394 | 15 | -0.999013346 | ONECUT2 | one cut homeobox 2 [Source:HGNC Symbol;Acc:HGNC:8139] |
| ENSG00000119771 | HGLibA_25173 | 15 | -0.999013346 | KLHL29 | kelch like family member 29 [Source:HGNC Symbol;Acc:HGNC:29404] |
| ENSG00000120328 | HGLibA_35479 | 15 | -0.999013346 | PCDHB12 | protocadherin beta 12 [Source:HGNC Symbol;Acc:HGNC:8683] |
| ENSG00000120833 | HGLibA_46126 | 15 | -0.999013346 | SOCS2 | suppressor of cytokine signaling 2 [Source:HGNC Symbol;Acc:HGNC:19382] |
| ENSG00000121853 | HGLibA_19053 | 15 | -0.999013346 | GHSR | growth hormone secretagogue receptor [Source:HGNC Symbol;Acc:HGNC:4267] |
| ENSG00000123983 | HGLibA_00557 | 15 | -0.999013346 | ACSL3 | acyl-CoA synthetase long chain family member 3 [Source:HGNC Symbol;Acc:HGNC:3570] |
| ENSG00000124257 | HGLibA_31729 | 15 | -0.999013346 | NEURL2 | neuralized E3 ubiquitin protein ligase 2 [Source:HGNC Symbol;Acc:HGNC:16156] |
| ENSG00000125255 | HGLibA_44332 | 15 | -0.999013346 | SLC10A2 | solute carrier family 10 member 2 [Source:HGNC Symbol;Acc:HGNC:10906] |
| ENSG00000125633 | HGLibA_07931 | 15 | -0.999013346 | CCDC93 | coiled-coil domain containing 93 [Source:HGNC Symbol;Acc:HGNC:25611] |
| ENSG00000125877 | HGLibA_23839 | 15 | -0.999013346 | ITPA | inosine triphosphatase [Source:HGNC Symbol;Acc:HGNC:6176] |
| ENSG00000127362 | HGLibA_48383 | 15 | -0.999013346 | TAS2R3 | taste 2 receptor member 3 [Source:HGNC Symbol;Acc:HGNC:14910] |
| ENSG00000127588 | HGLibA_19516 | 15 | -0.999013346 | GNG13 | G protein subunit gamma 13 [Source:HGNC Symbol;Acc:HGNC:14131] |
| ENSG00000130414 | HGLibA_31439 | 15 | -0.999013346 | NDUFA10 | NADH:ubiquinone oxidoreductase subunit A10 [Source:HGNC Symbol;Acc:HGNC:7684] |
| ENSG00000131238 | HGLibA_38232 | 15 | -0.999013346 | PPT1 | palmitoyl-protein thioesterase 1 [Source:HGNC Symbol;Acc:HGNC:9325] |
| ENSG00000131473 | HGLibA_00451 | 15 | -0.999013346 | ACLY | ATP citrate lyase [Source:HGNC Symbol;Acc:HGNC:115] |
| ENSG00000131480 | HGLibA_02270 | 15 | -0.999013346 | AOC2 | copper containing 2 [Source:HGNC Symbol;Acc:HGNC:549]" |
| ENSG00000133107 | HGLibA_51733 | 15 | -0.999013346 | TRPC4 | transient receptor potential cation channel subfamily C member 4 [Source:HGNC Symbol;Acc:HGNC:12336] |
| ENSG00000134283 | HGLibA_37890 | 15 | -0.999013346 | PPHLN1 | periphilin 1 [Source:HGNC Symbol;Acc:HGNC:19369] |
| ENSG00000134480 | HGLibA_08099 | 15 | -0.999013346 | CCNH | cyclin H [Source:HGNC Symbol;Acc:HGNC:1594] |
| ENSG00000134825 | HGLibA_50341 | 15 | -0.999013346 | TMEM258 | transmembrane protein 258 [Source:HGNC Symbol;Acc:HGNC:1164] |
| ENSG00000134909 | HGLibA_02760 | 15 | -0.999013346 | ARHGAP32 | Rho GTPase activating protein 32 [Source:HGNC Symbol;Acc:HGNC:17399] |
| ENSG00000135763 | HGLibA_53136 | 15 | -0.999013346 | URB2 | URB2 ribosome biogenesis homolog [Source:HGNC Symbol;Acc:HGNC:28967] |
| ENSG00000135976 | HGLibA_02088 | 15 | -0.999013346 | ANKRD36 | ankyrin repeat domain 36 [Source:HGNC Symbol;Acc:HGNC:24079] |
| ENSG00000136531 | HGLibA_42980 | 15 | -0.999013346 | SCN2A | sodium voltage-gated channel alpha subunit 2 [Source:HGNC Symbol;Acc:HGNC:10588] |
| ENSG00000136631 | HGLibA_53795 | 15 | -0.999013346 | VPS45 | vacuolar protein sorting 45 homolog [Source:HGNC Symbol;Acc:HGNC:14579] |
| ENSG00000137078 | HGLibA_44238 | 15 | -0.999013346 | SIT1 | signaling threshold regulating transmembrane adaptor 1 [Source:HGNC Symbol;Acc:HGNC:17710] |
| ENSG00000137185 | HGLibA_57035 | 15 | -0.999013346 | ZSCAN9 | zinc finger and SCAN domain containing 9 [Source:HGNC Symbol;Acc:HGNC:12984] |
| ENSG00000137413 | HGLibA_48257 | 15 | -0.999013346 | TAF8 | TATA-box binding protein associated factor 8 [Source:HGNC Symbol;Acc:HGNC:17300] |
| ENSG00000138101 | HGLibA_14066 | 15 | -0.999013346 | DTNB | dystrobrevin beta [Source:HGNC Symbol;Acc:HGNC:3058] |
| ENSG00000138600 | HGLibA_46775 | 15 | -0.999013346 | SPPL2A | signal peptide peptidase like 2A [Source:HGNC Symbol;Acc:HGNC:30227] |
| ENSG00000138660 | HGLibA_02279 | 15 | -0.999013346 | AP1AR | adaptor related protein complex 1 associated regulatory protein [Source:HGNC Symbol;Acc:HGNC:28808] |
| ENSG00000138796 | HGLibA_20805 | 15 | -0.999013346 | HADH | hydroxyacyl-CoA dehydrogenase [Source:HGNC Symbol;Acc:HGNC:4799] |
| ENSG00000138814 | HGLibA_38185 | 15 | -0.999013346 | PPP3CA | protein phosphatase 3 catalytic subunit alpha [Source:HGNC Symbol;Acc:HGNC:9314] |
| ENSG00000139718 | HGLibA_43640 | 15 | -0.999013346 | SETD1B | SET domain containing 1B [Source:HGNC Symbol;Acc:HGNC:29187] |
| ENSG00000141161 | HGLibA_53009 | 15 | -0.999013346 | UNC45B | unc-45 myosin chaperone B [Source:HGNC Symbol;Acc:HGNC:14304] |
| ENSG00000143772 | HGLibA_23847 | 15 | -0.999013346 | ITPKB | inositol-trisphosphate 3-kinase B [Source:HGNC Symbol;Acc:HGNC:6179] |
| ENSG00000143850 | HGLibA_37078 | 15 | -0.999013346 | PLEKHA6 | pleckstrin homology domain containing A6 [Source:HGNC Symbol;Acc:HGNC:17053] |
| ENSG00000147183 | HGLibA_11033 | 15 | -0.999013346 | CPXCR1 | candidate 1 [Source:HGNC Symbol;Acc:HGNC:2332]" |
| ENSG00000147576 | HGLibA_00967 | 15 | -0.999013346 | ADHFE1 | iron containing 1 [Source:HGNC Symbol;Acc:HGNC:16354]" |
| ENSG00000148396 | HGLibA_43208 | 15 | -0.999013346 | SEC16A | endoplasmic reticulum export factor [Source:HGNC Symbol;Acc:HGNC:29006]" |
| ENSG00000148584 | HGLibA_00006 | 15 | -0.999013346 | A1CF | APOBEC1 complementation factor [Source:HGNC Symbol;Acc:HGNC:24086] |
| ENSG00000149212 | HGLibA_43624 | 15 | -0.999013346 | SESN3 | sestrin 3 [Source:HGNC Symbol;Acc:HGNC:23060] |
| ENSG00000149575 | HGLibA_42984 | 15 | -0.999013346 | SCN2B | sodium voltage-gated channel beta subunit 2 [Source:HGNC Symbol;Acc:HGNC:10589] |
| ENSG00000150556 | HGLibA_27731 | 15 | -0.999013346 | LYPD6B | LY6/PLAUR domain containing 6B [Source:HGNC Symbol;Acc:HGNC:27018] |
| ENSG00000151117 | HGLibA_50524 | 15 | -0.999013346 | TMEM86A | transmembrane protein 86A [Source:HGNC Symbol;Acc:HGNC:26890] |
| ENSG00000151458 | HGLibA_02113 | 15 | -0.999013346 | ANKRD50 | ankyrin repeat domain 50 [Source:HGNC Symbol;Acc:HGNC:29223] |
| ENSG00000154654 | HGLibA_31257 | 15 | -0.999013346 | NCAM2 | neural cell adhesion molecule 2 [Source:HGNC Symbol;Acc:HGNC:7657] |
| ENSG00000155087 | HGLibA_33274 | 15 | -0.999013346 | ODF1 | outer dense fiber of sperm tails 1 [Source:HGNC Symbol;Acc:HGNC:8113] |
| ENSG00000156787 | HGLibA_48536 | 15 | -0.999013346 | TBC1D31 | TBC1 domain family member 31 [Source:HGNC Symbol;Acc:HGNC:30888] |
| ENSG00000156990 | HGLibA_42212 | 15 | -0.999013346 | RPUSD3 | RNA pseudouridine synthase D3 [Source:HGNC Symbol;Acc:HGNC:28437] |
| ENSG00000157119 | HGLibA_25206 | 15 | -0.999013346 | KLHL40 | kelch like family member 40 [Source:HGNC Symbol;Acc:HGNC:30372] |
| ENSG00000157999 | HGLibA_02131 | 15 | -0.999013346 | ANKRD61 | ankyrin repeat domain 61 [Source:HGNC Symbol;Acc:HGNC:22467] |
| ENSG00000158773 | HGLibA_53166 | 15 | -0.999013346 | USF1 | upstream transcription factor 1 [Source:HGNC Symbol;Acc:HGNC:12593] |
| ENSG00000159588 | HGLibA_07689 | 15 | -0.999013346 | CCDC17 | coiled-coil domain containing 17 [Source:HGNC Symbol;Acc:HGNC:26574] |
| ENSG00000160305 | HGLibA_13246 | 15 | -0.999013346 | DIP2A | disco interacting protein 2 homolog A [Source:HGNC Symbol;Acc:HGNC:17217] |
| ENSG00000160345 | HGLibA_06666 | 15 | -0.999013346 | C9orf116 | chromosome 9 open reading frame 116 [Source:HGNC Symbol;Acc:HGNC:28435] |
| ENSG00000161011 | HGLibA_46920 | 15 | -0.999013346 | SQSTM1 | sequestosome 1 [Source:HGNC Symbol;Acc:HGNC:11280] |
| ENSG00000162650 | HGLibA_03854 | 15 | -0.999013346 | ATXN7L2 | ataxin 7 like 2 [Source:HGNC Symbol;Acc:HGNC:28713] |
| ENSG00000162664 | HGLibA_55808 | 15 | -0.999013346 | ZNF326 | zinc finger protein 326 [Source:HGNC Symbol;Acc:HGNC:14104] |
| ENSG00000162702 | HGLibA_55734 | 15 | -0.999013346 | ZNF281 | zinc finger protein 281 [Source:HGNC Symbol;Acc:HGNC:13075] |
| ENSG00000162851 | HGLibA_49143 | 15 | -0.999013346 | TFB2M | mitochondrial [Source:HGNC Symbol;Acc:HGNC:18559]" |
| ENSG00000162909 | HGLibA_07162 | 15 | -0.999013346 | CAPN2 | calpain 2 [Source:HGNC Symbol;Acc:HGNC:1479] |
| ENSG00000162992 | HGLibA_31733 | 15 | -0.999013346 | NEUROD1 | neuronal differentiation 1 [Source:HGNC Symbol;Acc:HGNC:7762] |
| ENSG00000163220 | HGLibA_42574 | 15 | -0.999013346 | S100A9 | S100 calcium binding protein A9 [Source:HGNC Symbol;Acc:HGNC:10499] |
| ENSG00000163430 | HGLibA_18102 | 15 | -0.999013346 | FSTL1 | follistatin like 1 [Source:HGNC Symbol;Acc:HGNC:3972] |
| ENSG00000164896 | HGLibA_16897 | 15 | -0.999013346 | FASTK | Fas activated serine/threonine kinase [Source:HGNC Symbol;Acc:HGNC:24676] |
| ENSG00000165272 | HGLibA_02594 | 15 | -0.999013346 | AQP3 | aquaporin 3 (Gill blood group) [Source:HGNC Symbol;Acc:HGNC:636] |
| ENSG00000165383 | HGLibA_27271 | 15 | -0.999013346 | LRRC18 | leucine rich repeat containing 18 [Source:HGNC Symbol;Acc:HGNC:23199] |
| ENSG00000165821 | HGLibA_42649 | 15 | -0.999013346 | SALL2 | spalt like transcription factor 2 [Source:HGNC Symbol;Acc:HGNC:10526] |
| ENSG00000166477 | HGLibA_26305 | 15 | -0.999013346 | LEO1 | Paf1/RNA polymerase II complex component [Source:HGNC Symbol;Acc:HGNC:30401]" |
| ENSG00000166997 | HGLibA_10371 | 15 | -0.999013346 | CNPY4 | canopy FGF signaling regulator 4 [Source:HGNC Symbol;Acc:HGNC:28631] |
| ENSG00000167034 | HGLibA_32050 | 15 | -0.999013346 | NKX3-1 | NK3 homeobox 1 [Source:HGNC Symbol;Acc:HGNC:7838] |
| ENSG00000168395 | HGLibA_23296 | 15 | -0.999013346 | ING5 | inhibitor of growth family member 5 [Source:HGNC Symbol;Acc:HGNC:19421] |
| ENSG00000169208 | HGLibA_33490 | 15 | -0.999013346 | OR10G3 | olfactory receptor family 10 subfamily G member 3 [Source:HGNC Symbol;Acc:HGNC:8171] |
| ENSG00000169223 | HGLibA_26663 | 15 | -0.999013346 | LMAN2 | mannose binding 2 [Source:HGNC Symbol;Acc:HGNC:16986]" |
| ENSG00000169688 | HGLibA_30280 | 15 | -0.999013346 | MT1B | metallothionein 1B [Source:HGNC Symbol;Acc:HGNC:7394] |
| ENSG00000170085 | HGLibA_44179 | 15 | -0.999013346 | SIMC1 | SUMO interacting motifs containing 1 [Source:HGNC Symbol;Acc:HGNC:24779] |
| ENSG00000170484 | HGLibA_25535 | 15 | -0.999013346 | KRT74 | keratin 74 [Source:HGNC Symbol;Acc:HGNC:28929] |
| ENSG00000171469 | HGLibA_56245 | 15 | -0.999013346 | ZNF561 | zinc finger protein 561 [Source:HGNC Symbol;Acc:HGNC:28684] |
| ENSG00000172201 | HGLibA_22534 | 15 | -0.999013346 | ID4 | HLH protein [Source:HGNC Symbol;Acc:HGNC:5363]" |
| ENSG00000172232 | HGLibA_03942 | 15 | -0.999013346 | AZU1 | azurocidin 1 [Source:HGNC Symbol;Acc:HGNC:913] |
| ENSG00000172667 | HGLibA_55372 | 15 | -0.999013346 | ZMAT3 | zinc finger matrin-type 3 [Source:HGNC Symbol;Acc:HGNC:29983] |
| ENSG00000172890 | HGLibA_31054 | 15 | -0.999013346 | NADSYN1 | NAD synthetase 1 [Source:HGNC Symbol;Acc:HGNC:29832] |
| ENSG00000173464 | HGLibA_41395 | 15 | -0.999013346 | RNASE11 | ribonuclease A family member 11 (inactive) [Source:HGNC Symbol;Acc:HGNC:19269] |
| ENSG00000174276 | HGLibA_56926 | 15 | -0.999013346 | ZNHIT2 | zinc finger HIT-type containing 2 [Source:HGNC Symbol;Acc:HGNC:1177] |
| ENSG00000174279 | HGLibA_15710 | 15 | -0.999013346 | EVX2 | even-skipped homeobox 2 [Source:HGNC Symbol;Acc:HGNC:3507] |
| ENSG00000174720 | HGLibA_26042 | 15 | -0.999013346 | LARP7 | La ribonucleoprotein domain family member 7 [Source:HGNC Symbol;Acc:HGNC:24912] |
| ENSG00000174807 | HGLibA_08312 | 15 | -0.999013346 | CD248 | CD248 molecule [Source:HGNC Symbol;Acc:HGNC:18219] |
| ENSG00000175063 | HGLibA_52580 | 15 | -0.999013346 | UBE2C | ubiquitin conjugating enzyme E2 C [Source:HGNC Symbol;Acc:HGNC:15937] |
| ENSG00000175087 | HGLibA_35888 | 15 | -0.999013346 | PDIK1L | PDLIM1 interacting kinase 1 like [Source:HGNC Symbol;Acc:HGNC:18981] |
| ENSG00000175294 | HGLibA_07354 | 15 | -0.999013346 | CATSPER1 | cation channel sperm associated 1 [Source:HGNC Symbol;Acc:HGNC:17116] |
| ENSG00000176797 | HGLibA_12814 | 15 | -0.999013346 | DEFB103A | defensin beta 103A [Source:HGNC Symbol;Acc:HGNC:15967] |
| ENSG00000177181 | HGLibA_41271 | 15 | -0.999013346 | RIMKLA | ribosomal modification protein rimK like family member A [Source:HGNC Symbol;Acc:HGNC:28725] |
| ENSG00000177427 | HGLibA_29245 | 15 | -0.999013346 | MIEF2 | mitochondrial elongation factor 2 [Source:HGNC Symbol;Acc:HGNC:17920] |
| ENSG00000178234 | HGLibA_18532 | 15 | -0.999013346 | GALNT11 | polypeptide N-acetylgalactosaminyltransferase 11 [Source:HGNC Symbol;Acc:HGNC:19875] |

| | | | | | |
|---|---|---|---|---|---|
| ENSG00000178386 | HGLibA_55614 | 15 | -0.999013346 | ZNF223 | zinc finger protein 223 [Source:HGNC Symbol;Acc:HGNC:13016] |
| ENSG00000179363 | HGLibA_50365 | 15 | -0.999013346 | TMEM31 | transmembrane protein 31 [Source:HGNC Symbol;Acc:HGNC:28601] |
| ENSG00000179826 | HGLibA_29853 | 15 | -0.999013346 | MRGPRX3 | MAS related GPR family member X3 [Source:HGNC Symbol;Acc:HGNC:17980] |
| ENSG00000182154 | HGLibA_29982 | 15 | -0.999013346 | MRPL41 | mitochondrial ribosomal protein L41 [Source:HGNC Symbol;Acc:HGNC:14492] |
| ENSG00000183578 | HGLibA_50730 | 15 | -0.999013346 | TNFAIP8L3 | TNF alpha induced protein 8 like 3 [Source:HGNC Symbol;Acc:HGNC:20620] |
| ENSG00000184787 | HGLibA_52608 | 15 | -0.999013346 | UBE2G2 | ubiquitin conjugating enzyme E2 G2 [Source:HGNC Symbol;Acc:HGNC:12483] |
| ENSG00000185019 | HGLibA_52727 | 15 | -0.999013346 | UBOX5 | U-box domain containing 5 [Source:HGNC Symbol;Acc:HGNC:17777] |
| ENSG00000185774 | HGLibA_24242 | 15 | -0.999013346 | KCNIP4 | potassium voltage-gated channel interacting protein 4 [Source:HGNC Symbol;Acc:HGNC:30083] |
| ENSG00000185909 | HGLibA_25115 | 15 | -0.999013346 | KLHDC8B | kelch domain containing 8B [Source:HGNC Symbol;Acc:HGNC:28557] |
| ENSG00000185933 | HGLibA_07014 | 15 | -0.999013346 | CALHM1 | calcium homeostasis modulator 1 [Source:HGNC Symbol;Acc:HGNC:23494] |
| ENSG00000186684 | HGLibA_12101 | 15 | -0.999013346 | CYP27C1 | cytochrome P450 family 27 subfamily C member 1 [Source:HGNC Symbol;Acc:HGNC:33480] |
| ENSG00000187049 | HGLibA_50222 | 15 | -0.999013346 | TMEM216 | transmembrane protein 216 [Source:HGNC Symbol;Acc:HGNC:25018] |
| ENSG00000187242 | HGLibA_25420 | 15 | -0.999013346 | KRT12 | keratin 12 [Source:HGNC Symbol;Acc:HGNC:6414] |
| ENSG00000187682 | HGLibA_15410 | 15 | -0.999013346 | ERAS | ES cell expressed Ras [Source:HGNC Symbol;Acc:HGNC:5174] |
| ENSG00000188095 | HGLibA_28918 | 15 | -0.999013346 | MESP2 | mesoderm posterior bHLH transcription factor 2 [Source:HGNC Symbol;Acc:HGNC:29659] |
| ENSG00000188234 | HGLibA_01147 | 15 | -0.999013346 | AGAP4 | ankyrin repeat and PH domain 4 [Source:HGNC Symbol;Acc:HGNC:23459]" |
| ENSG00000196876 | HGLibA_43003 | 15 | -0.999013346 | SCN8A | sodium voltage-gated channel alpha subunit 8 [Source:HGNC Symbol;Acc:HGNC:10596] |
| ENSG00000197273 | HGLibA_20643 | 15 | -0.999013346 | GUCA2A | guanylate cyclase activator 2A [Source:HGNC Symbol;Acc:HGNC:4682] |
| ENSG00000197565 | HGLibA_10584 | 15 | -0.999013346 | COL4A6 | collagen type IV alpha 6 chain [Source:HGNC Symbol;Acc:HGNC:2208] |
| ENSG00000198626 | HGLibA_42523 | 15 | -0.999013346 | RYR2 | ryanodine receptor 2 [Source:HGNC Symbol;Acc:HGNC:10484] |
| ENSG00000198650 | HGLibA_48439 | 15 | -0.999013346 | TAT | tyrosine aminotransferase [Source:HGNC Symbol;Acc:HGNC:11573] |
| ENSG00000204385 | HGLibA_45166 | 15 | -0.999013346 | SLC44A4 | solute carrier family 44 member 4 [Source:HGNC Symbol;Acc:HGNC:13941] |
| ENSG00000204569 | HGLibA_38013 | 15 | -0.999013346 | PPP1R10 | protein phosphatase 1 regulatory subunit 10 [Source:HGNC Symbol;Acc:HGNC:9284] |
| ENSG00000204655 | HGLibA_29622 | 15 | -0.999013346 | MOG | myelin oligodendrocyte glycoprotein [Source:HGNC Symbol;Acc:HGNC:7197] |
| ENSG00000204671 | HGLibA_23130 | 15 | -0.999013346 | IL31 | interleukin 31 [Source:HGNC Symbol;Acc:HGNC:19372] |
| ENSG00000214336 | HGLibA_17882 | 15 | -0.999013346 | FOXI3 | forkhead box I3 [Source:HGNC Symbol;Acc:HGNC:35123] |
| ENSG00000215021 | HGLibA_36315 | 15 | -0.999013346 | PHB2 | prohibitin 2 [Source:HGNC Symbol;Acc:HGNC:30306] |
| ENSG00000230031 | HGLibA_37721 | 15 | -0.999013346 | POTEB2 | POTE ankyrin domain family member B2 [Source:HGNC Symbol;Acc:HGNC:48327] |
| ENSG00000232859 | HGLibA_27762 | 15 | -0.999013346 | LYRM9 | LYR motif containing 9 [Source:HGNC Symbol;Acc:HGNC:27314] |
| ENSG00000243896 | HGLibA_33744 | 15 | -0.999013346 | OR2A7 | olfactory receptor family 2 subfamily A member 7 [Source:HGNC Symbol;Acc:HGNC:8234] |
| ENSG00000244694 | HGLibA_39283 | 15 | -0.999013346 | PTCHD4 | patched domain containing 4 [Source:HGNC Symbol;Acc:HGNC:21345] |
| ENSG00000248483 | HGLibA_37793 | 15 | -0.999013346 | POU5F2 | transcription factor 2 [Source:HGNC Symbol;Acc:HGNC:26367]" |
| ENSG00000249884 | HGLibA_41461 | 15 | -0.999013346 | RNF103-CHMP3 | RNF103-CHMP3 readthrough [Source:HGNC Symbol;Acc:HGNC:38847] |
| ENSG00000251369 | HGLibA_56212 | 15 | -0.999013346 | ZNF550 | zinc finger protein 550 [Source:HGNC Symbol;Acc:HGNC:28643] |
| ENSG00000256087 | HGLibA_55983 | 15 | -0.999013346 | ZNF432 | zinc finger protein 432 [Source:HGNC Symbol;Acc:HGNC:20810] |
| ENSG00000270757 | HGLibA_22337 | 15 | -0.999013346 | HSPE1-MOB4 | HSPE1-MOB4 readthrough [Source:HGNC Symbol;Acc:HGNC:49184] |
| ENSG00000284922 | HGLibA_27506 | 15 | -0.999013346 | LRTOMT | novel protein |
| ENSG00000288867 | HGLibA_37904 | 15 | -0.999013346 | PPIAL4C | - |
| FAM103A1 | HGLibA_15984 | 15 | -0.999013346 | FAM103A1 | - |
| FLJ27352 | HGLibA_17628 | 15 | -0.999013346 | FLJ27352 | - |
| HIST1H2BK | HGLibA_21440 | 15 | -0.999013346 | HIST1H2BK | - |
| HIST1H4J | HGLibA_21512 | 15 | -0.999013346 | HIST1H4J | - |
| HIST2H2AC | HGLibA_21532 | 15 | -0.999013346 | HIST2H2AC | - |
| hsa-mir-103a-2 | HGLibA_57167 | 15 | -0.999013346 | hsa-mir-103a-2 | - |
| hsa-mir-1185-1 | HGLibA_57240 | 15 | -0.999013346 | hsa-mir-1185-1 | - |
| hsa-mir-18b | HGLibA_58021 | 15 | -0.999013346 | hsa-mir-18b | - |
| hsa-mir-216a | HGLibA_58256 | 15 | -0.999013346 | hsa-mir-216a | - |
| hsa-mir-2276 | HGLibA_58307 | 15 | -0.999013346 | hsa-mir-2276 | - |
| hsa-mir-2682 | HGLibA_58361 | 15 | -0.999013346 | hsa-mir-2682 | - |
| hsa-mir-3118-6 | HGLibA_58522 | 15 | -0.999013346 | hsa-mir-3118-6 | - |
| hsa-mir-3183 | HGLibA_58831 | 15 | -0.999013346 | hsa-mir-3183 | - |
| hsa-mir-3616 | HGLibA_59094 | 15 | -0.999013346 | hsa-mir-3616 | - |
| hsa-mir-3670-1 | HGLibA_59232 | 15 | -0.999013346 | hsa-mir-3670-1 | - |
| hsa-mir-3673 | HGLibA_59247 | 15 | -0.999013346 | hsa-mir-3673 | - |
| hsa-mir-3917 | HGLibA_59545 | 15 | -0.999013346 | hsa-mir-3917 | - |
| hsa-mir-409 | HGLibA_59675 | 15 | -0.999013346 | hsa-mir-409 | - |
| hsa-mir-4289 | HGLibA_59863 | 15 | -0.999013346 | hsa-mir-4289 | - |
| hsa-mir-4509-2 | HGLibA_60467 | 15 | -0.999013346 | hsa-mir-4509-2 | - |
| hsa-mir-4509-3 | HGLibA_60470 | 15 | -0.999013346 | hsa-mir-4509-3 | - |
| hsa-mir-4520a | HGLibA_60532 | 15 | -0.999013346 | hsa-mir-4520a | - |
| hsa-mir-4651 | HGLibA_60710 | 15 | -0.999013346 | hsa-mir-4651 | - |
| hsa-mir-4673 | HGLibA_60810 | 15 | -0.999013346 | hsa-mir-4673 | - |
| hsa-mir-4677 | HGLibA_60826 | 15 | -0.999013346 | hsa-mir-4677 | - |
| hsa-mir-4781 | HGLibA_61230 | 15 | -0.999013346 | hsa-mir-4781 | - |
| hsa-mir-491 | HGLibA_61364 | 15 | -0.999013346 | hsa-mir-491 | - |
| hsa-mir-514b | HGLibA_61599 | 15 | -0.999013346 | hsa-mir-514b | - |
| hsa-mir-516a-1 | HGLibA_61608 | 15 | -0.999013346 | hsa-mir-516a-1 | - |
| hsa-mir-518a-2 | HGLibA_61655 | 15 | -0.999013346 | hsa-mir-518a-2 | - |
| hsa-mir-548t | HGLibA_62066 | 15 | -0.999013346 | hsa-mir-548t | - |
| hsa-mir-5787 | HGLibA_62412 | 15 | -0.999013346 | hsa-mir-5787 | - |
| hsa-mir-585 | HGLibA_62437 | 15 | -0.999013346 | hsa-mir-585 | - |
| hsa-mir-6081 | HGLibA_62586 | 15 | -0.999013346 | hsa-mir-6081 | - |
| hsa-mir-610 | HGLibA_62633 | 15 | -0.999013346 | hsa-mir-610 | - |
| hsa-mir-6751 | HGLibA_63163 | 15 | -0.999013346 | hsa-mir-6751 | - |
| hsa-mir-6770-1 | HGLibA_63247 | 15 | -0.999013346 | hsa-mir-6770-1 | - |
| hsa-mir-7107 | HGLibA_63787 | 15 | -0.999013346 | hsa-mir-7107 | - |
| hsa-mir-939 | HGLibA_64329 | 15 | -0.999013346 | hsa-mir-939 | - |
| METTL10 | HGLibA_28946 | 15 | -0.999013346 | METTL10 | - |
| TCEB3CL | HGLibA_48748 | 15 | -0.999013346 | TCEB3CL | - |
| AHSA2 | HGLibA_01316 | 14 | -1.008058689 | AHSA2 | - |
| C12orf5 | HGLibA_05257 | 14 | -1.008058689 | C12orf5 | - |
| C15orf53 | HGLibA_05412 | 14 | -1.008058689 | C15orf53 | - |
| C16orf52 | HGLibA_05452 | 14 | -1.008058689 | C16orf52 | - |
| C21orf2 | HGLibA_06035 | 14 | -1.008058689 | C21orf2 | - |
| CCDC37 | HGLibA_07767 | 14 | -1.008058689 | CCDC37 | - |
| CD3EAP | HGLibA_08374 | 14 | -1.008058689 | CD3EAP | - |
| ENSG00000000938 | HGLibA_17467 | 14 | -1.008058689 | FGR | Src family tyrosine kinase [Source:HGNC Symbol;Acc:HGNC:3697]" |
| ENSG00000010219 | HGLibA_14282 | 14 | -1.008058689 | DYRK4 | dual specificity tyrosine phosphorylation regulated kinase 4 [Source:HGNC Symbol;Acc:HGNC:3095] |
| ENSG00000010270 | HGLibA_47359 | 14 | -1.008058689 | STARD3NL | STARD3 N-terminal like [Source:HGNC Symbol;Acc:HGNC:19169] |
| ENSG00000012124 | HGLibA_08298 | 14 | -1.008058689 | CD22 | CD22 molecule [Source:HGNC Symbol;Acc:HGNC:1643] |
| ENSG00000013364 | HGLibA_30600 | 14 | -1.008058689 | MVP | major vault protein [Source:HGNC Symbol;Acc:HGNC:7531] |
| ENSG00000024422 | HGLibA_14670 | 14 | -1.008058689 | EHD2 | EH domain containing 2 [Source:HGNC Symbol;Acc:HGNC:3243] |
| ENSG00000033170 | HGLibA_18207 | 14 | -1.008058689 | FUT8 | fucosyltransferase 8 [Source:HGNC Symbol;Acc:HGNC:4019] |
| ENSG00000037474 | HGLibA_32781 | 14 | -1.008058689 | NSUN2 | NOP2/Sun RNA methyltransferase family member 2 [Source:HGNC Symbol;Acc:HGNC:25994] |
| ENSG00000050748 | HGLibA_28294 | 14 | -1.008058689 | MAPK9 | mitogen-activated protein kinase 9 [Source:HGNC Symbol;Acc:HGNC:6886] |
| ENSG00000054598 | HGLibA_17821 | 14 | -1.008058689 | FOXC1 | forkhead box C1 [Source:HGNC Symbol;Acc:HGNC:3800] |
| ENSG00000060339 | HGLibA_07486 | 14 | -1.008058689 | CCAR1 | cell division cycle and apoptosis regulator 1 [Source:HGNC Symbol;Acc:HGNC:24236] |
| ENSG00000060558 | HGLibA_19449 | 14 | -1.008058689 | GNA15 | G protein subunit alpha 15 [Source:HGNC Symbol;Acc:HGNC:4383] |
| ENSG00000062725 | HGLibA_02564 | 14 | -1.008058689 | APPBP2 | amyloid beta precursor protein binding protein 2 [Source:HGNC Symbol;Acc:HGNC:622] |
| ENSG00000065243 | HGLibA_36840 | 14 | -1.008058689 | PKN2 | protein kinase N2 [Source:HGNC Symbol;Acc:HGNC:9406] |
| ENSG00000065970 | HGLibA_17887 | 14 | -1.008058689 | FOXJ2 | forkhead box J2 [Source:HGNC Symbol;Acc:HGNC:24818] |
| ENSG00000070729 | HGLibA_10276 | 14 | -1.008058689 | CNGB1 | cyclic nucleotide gated channel beta 1 [Source:HGNC Symbol;Acc:HGNC:2151] |
| ENSG00000082805 | HGLibA_15426 | 14 | -1.008058689 | ERC1 | ELKS/RAB6-interacting/CAST family member 1 [Source:HGNC Symbol;Acc:HGNC:17072] |
| ENSG00000085185 | HGLibA_04365 | 14 | -1.008058689 | BCORL1 | BCL6 corepressor like 1 [Source:HGNC Symbol;Acc:HGNC:25657] |
| ENSG00000092345 | HGLibA_12369 | 14 | -1.008058689 | DAZL | deleted in azoospermia like [Source:HGNC Symbol;Acc:HGNC:2685] |
| ENSG00000095319 | HGLibA_33034 | 14 | -1.008058689 | NUP188 | nucleoporin 188 [Source:HGNC Symbol;Acc:HGNC:17859] |
| ENSG00000099817 | HGLibA_37577 | 14 | -1.008058689 | POLR2E | RNA polymerase II subunit E [Source:HGNC Symbol;Acc:HGNC:9192] |
| ENSG00000099937 | HGLibA_43575 | 14 | -1.008058689 | SERPIND1 | serpin family D member 1 [Source:HGNC Symbol;Acc:HGNC:4838] |
| ENSG00000100023 | HGLibA_37942 | 14 | -1.008058689 | PPIL2 | peptidylprolyl isomerase like 2 [Source:HGNC Symbol;Acc:HGNC:9261] |
| ENSG00000100075 | HGLibA_44634 | 14 | -1.008058689 | SLC25A1 | solute carrier family 25 member 1 [Source:HGNC Symbol;Acc:HGNC:10979] |
| ENSG00000100078 | HGLibA_36898 | 14 | -1.008058689 | PLA2G3 | phospholipase A2 group III [Source:HGNC Symbol;Acc:HGNC:17934] |
| ENSG00000100150 | HGLibA_12984 | 14 | -1.008058689 | DEPDC5 | DEP domain containing 5 [Source:HGNC Symbol;Acc:HGNC:18423] |
| ENSG00000100599 | HGLibA_41294 | 14 | -1.008058689 | RIN3 | Ras and Rab interactor 3 [Source:HGNC Symbol;Acc:HGNC:18751] |
| ENSG00000100626 | HGLibA_18548 | 14 | -1.008058689 | GALNT16 | polypeptide N-acetylgalactosaminyltransferase 16 [Source:HGNC Symbol;Acc:HGNC:23233] |
| ENSG00000101126 | HGLibA_01000 | 14 | -1.008058689 | ADNP | activity dependent neuroprotector homeobox [Source:HGNC Symbol;Acc:HGNC:15766] |
| ENSG00000102032 | HGLibA_40839 | 14 | -1.008058689 | RENBP | renin binding protein [Source:HGNC Symbol;Acc:HGNC:9959] |
| ENSG00000103932 | HGLibA_41815 | 14 | -1.008058689 | RPAP1 | RNA polymerase II associated protein 1 [Source:HGNC Symbol;Acc:HGNC:24567] |
| ENSG00000104365 | HGLibA_22919 | 14 | -1.008058689 | IKBKB | inhibitor of nuclear factor kappa B kinase subunit beta [Source:HGNC Symbol;Acc:HGNC:5960] |
| ENSG00000104980 | HGLibA_49517 | 14 | -1.008058689 | TIMM44 | translocase of inner mitochondrial membrane 44 [Source:HGNC Symbol;Acc:HGNC:17316] |
| ENSG00000105989 | HGLibA_54357 | 14 | -1.008058689 | WNT2 | Wnt family member 2 [Source:HGNC Symbol;Acc:HGNC:12780] |
| ENSG00000106069 | HGLibA_09508 | 14 | -1.008058689 | CHN2 | chimerin 2 [Source:HGNC Symbol;Acc:HGNC:1944] |
| ENSG00000106526 | HGLibA_00664 | 14 | -1.008058689 | ACTR3C | ARP3 actin related protein 3 homolog C [Source:HGNC Symbol;Acc:HGNC:37282] |
| ENSG00000108395 | HGLibA_51476 | 14 | -1.008058689 | TRIM37 | tripartite motif containing 37 [Source:HGNC Symbol;Acc:HGNC:7523] |
| ENSG00000110871 | HGLibA_10758 | 14 | -1.008058689 | COQ5 | methyltransferase [Source:HGNC Symbol;Acc:HGNC:28722]" |
| ENSG00000111664 | HGLibA_19497 | 14 | -1.008058689 | GNB3 | G protein subunit beta 3 [Source:HGNC Symbol;Acc:HGNC:4400] |
| ENSG00000112996 | HGLibA_30085 | 14 | -1.008058689 | MRPS30 | mitochondrial ribosomal protein S30 [Source:HGNC Symbol;Acc:HGNC:8769] |
| ENSG00000114302 | HGLibA_38528 | 14 | -1.008058689 | PRKAR2A | protein kinase cAMP-dependent type II regulatory subunit alpha [Source:HGNC Symbol;Acc:HGNC:9391] |
| ENSG00000115414 | HGLibA_17725 | 14 | -1.008058689 | FN1 | fibronectin 1 [Source:HGNC Symbol;Acc:HGNC:3778] |
| ENSG00000117748 | HGLibA_41802 | 14 | -1.008058689 | RPA2 | replication protein A2 [Source:HGNC Symbol;Acc:HGNC:10290] |
| ENSG00000117906 | HGLibA_40704 | 14 | -1.008058689 | RCN2 | reticulocalbin 2 [Source:HGNC Symbol;Acc:HGNC:9935] |
| ENSG00000118193 | HGLibA_24862 | 14 | -1.008058689 | KIF14 | kinesin family member 14 [Source:HGNC Symbol;Acc:HGNC:19181] |
| ENSG00000119541 | HGLibA_53800 | 14 | -1.008058689 | VPS4B | vacuolar protein sorting 4 homolog B [Source:HGNC Symbol;Acc:HGNC:10895] |
| ENSG00000120279 | HGLibA_30681 | 14 | -1.008058689 | MYCT1 | MYC target 1 [Source:HGNC Symbol;Acc:HGNC:23172] |
| ENSG00000120915 | HGLibA_15325 | 14 | -1.008058689 | EPHX2 | epoxide hydrolase 2 [Source:HGNC Symbol;Acc:HGNC:3402] |
| ENSG00000126062 | HGLibA_49882 | 14 | -1.008058689 | TMEM115 | transmembrane protein 115 [Source:HGNC Symbol;Acc:HGNC:30055] |

| | | | | | |
|---|---|---|---|---|---|
| ENSG00000126262 | HGLibA_17328 | 14 | -1.008058689 | FFAR2 | free fatty acid receptor 2 [Source:HGNC Symbol;Acc:HGNC:4501] |
| ENSG00000126561 | HGLibA_47393 | 14 | -1.008058689 | STAT5A | signal transducer and activator of transcription 5A [Source:HGNC Symbol;Acc:HGNC:11366] |
| ENSG00000129003 | HGLibA_53736 | 14 | -1.008058689 | VPS13C | vacuolar protein sorting 13 homolog C [Source:HGNC Symbol;Acc:HGNC:23594] |
| ENSG00000129437 | HGLibA_25246 | 14 | -1.008058689 | KLK14 | kallikrein related peptidase 14 [Source:HGNC Symbol;Acc:HGNC:6362] |
| ENSG00000129467 | HGLibA_00917 | 14 | -1.008058689 | ADCY4 | adenylate cyclase 4 [Source:HGNC Symbol;Acc:HGNC:235] |
| ENSG00000130433 | HGLibA_06943 | 14 | -1.008058689 | CACNG6 | calcium voltage-gated channel auxiliary subunit gamma 6 [Source:HGNC Symbol;Acc:HGNC:13625] |
| ENSG00000130821 | HGLibA_45340 | 14 | -1.008058689 | SLC6A8 | solute carrier family 6 member 8 [Source:HGNC Symbol;Acc:HGNC:11055] |
| ENSG00000131044 | HGLibA_52216 | 14 | -1.008058689 | TTLL9 | tubulin tyrosine ligase like 9 [Source:HGNC Symbol;Acc:HGNC:16118] |
| ENSG00000131050 | HGLibA_04712 | 14 | -1.008058689 | BPIFA2 | BPI fold containing family A member 2 [Source:HGNC Symbol;Acc:HGNC:16203] |
| ENSG00000132600 | HGLibA_38636 | 14 | -1.008058689 | PRMT7 | protein arginine methyltransferase 7 [Source:HGNC Symbol;Acc:HGNC:25557] |
| ENSG00000132823 | HGLibA_34678 | 14 | -1.008058689 | OSER1 | oxidative stress responsive serine rich 1 [Source:HGNC Symbol;Acc:HGNC:16105] |
| ENSG00000133195 | HGLibA_45093 | 14 | -1.008058689 | SLC39A11 | solute carrier family 39 member 11 [Source:HGNC Symbol;Acc:HGNC:14463] |
| ENSG00000133805 | HGLibA_01828 | 14 | -1.008058689 | AMPD3 | adenosine monophosphate deaminase 3 [Source:HGNC Symbol;Acc:HGNC:470] |
| ENSG00000134243 | HGLibA_46197 | 14 | -1.008058689 | SORT1 | sortilin 1 [Source:HGNC Symbol;Acc:HGNC:11186] |
| ENSG00000135164 | HGLibA_13444 | 14 | -1.008058689 | DMTF1 | cyclin D binding myb like transcription factor 1 [Source:HGNC Symbol;Acc:HGNC:14603] |
| ENSG00000136827 | HGLibA_51016 | 14 | -1.008058689 | TOR1A | torsin family 1 member A [Source:HGNC Symbol;Acc:HGNC:3098] |
| ENSG00000137103 | HGLibA_50548 | 14 | -1.008058689 | TMEM8B | transmembrane protein 8B [Source:HGNC Symbol;Acc:HGNC:21427] |
| ENSG00000137558 | HGLibA_36482 | 14 | -1.008058689 | PI15 | peptidase inhibitor 15 [Source:HGNC Symbol;Acc:HGNC:8946] |
| ENSG00000137727 | HGLibA_02724 | 14 | -1.008058689 | ARHGAP20 | Rho GTPase activating protein 20 [Source:HGNC Symbol;Acc:HGNC:18357] |
| ENSG00000137821 | HGLibA_27377 | 14 | -1.008058689 | LRRC49 | leucine rich repeat containing 49 [Source:HGNC Symbol;Acc:HGNC:25965] |
| ENSG00000139193 | HGLibA_08314 | 14 | -1.008058689 | CD27 | CD27 molecule [Source:HGNC Symbol;Acc:HGNC:11922] |
| ENSG00000140307 | HGLibA_20526 | 14 | -1.008058689 | GTF2A2 | general transcription factor IIA subunit 2 [Source:HGNC Symbol;Acc:HGNC:4647] |
| ENSG00000143110 | HGLibA_05785 | 14 | -1.008058689 | C1orf162 | chromosome 1 open reading frame 162 [Source:HGNC Symbol;Acc:HGNC:28344] |
| ENSG00000144034 | HGLibA_51185 | 14 | -1.008058689 | TPRKB | TP53RK binding protein [Source:HGNC Symbol;Acc:HGNC:24259] |
| ENSG00000145029 | HGLibA_31919 | 14 | -1.008058689 | NICN1 | nicolin 1 [Source:HGNC Symbol;Acc:HGNC:18317] |
| ENSG00000145040 | HGLibA_52824 | 14 | -1.008058689 | UCN2 | urocortin 2 [Source:HGNC Symbol;Acc:HGNC:18414] |
| ENSG00000147036 | HGLibA_26009 | 14 | -1.008058689 | LANCL3 | LanC like 3 [Source:HGNC Symbol;Acc:HGNC:24767] |
| ENSG00000147655 | HGLibA_42346 | 14 | -1.008058689 | RSPO2 | R-spondin 2 [Source:HGNC Symbol;Acc:HGNC:28583] |
| ENSG00000147874 | HGLibA_20889 | 14 | -1.008058689 | HAUS6 | HAUS augmin like complex subunit 6 [Source:HGNC Symbol;Acc:HGNC:25948] |
| ENSG00000149809 | HGLibA_49714 | 14 | -1.008058689 | TM7SF2 | transmembrane 7 superfamily member 2 [Source:HGNC Symbol;Acc:HGNC:11863] |
| ENSG00000150048 | HGLibA_09956 | 14 | -1.008058689 | CLEC1A | C-type lectin domain family 1 member A [Source:HGNC Symbol;Acc:HGNC:24355] |
| ENSG00000150687 | HGLibA_38918 | 14 | -1.008058689 | PRSS23 | serine protease 23 [Source:HGNC Symbol;Acc:HGNC:14370] |
| ENSG00000152034 | HGLibA_28591 | 14 | -1.008058689 | MCHR2 | melanin concentrating hormone receptor 2 [Source:HGNC Symbol;Acc:HGNC:20867] |
| ENSG00000153147 | HGLibA_45611 | 14 | -1.008058689 | SMARCA5 | member 5 [Source:HGNC Symbol;Acc:HGNC:11101]" |
| ENSG00000155508 | HGLibA_10354 | 14 | -1.008058689 | CNOT8 | CCR4-NOT transcription complex subunit 8 [Source:HGNC Symbol;Acc:HGNC:9207] |
| ENSG00000155629 | HGLibA_36618 | 14 | -1.008058689 | PIK3AP1 | phosphoinositide-3-kinase adaptor protein 1 [Source:HGNC Symbol;Acc:HGNC:30034] |
| ENSG00000157227 | HGLibA_29492 | 14 | -1.008058689 | MMP14 | matrix metallopeptidase 14 [Source:HGNC Symbol;Acc:HGNC:7160] |
| ENSG00000157315 | HGLibA_49817 | 14 | -1.008058689 | TMED6 | transmembrane p24 trafficking protein 6 [Source:HGNC Symbol;Acc:HGNC:28331] |
| ENSG00000159166 | HGLibA_25922 | 14 | -1.008058689 | LAD1 | ladinin 1 [Source:HGNC Symbol;Acc:HGNC:6472] |
| ENSG00000160691 | HGLibA_44000 | 14 | -1.008058689 | SHC1 | SHC adaptor protein 1 [Source:HGNC Symbol;Acc:HGNC:10840] |
| ENSG00000161204 | HGLibA_00213 | 14 | -1.008058689 | ABCF3 | ATP binding cassette subfamily F member 3 [Source:HGNC Symbol;Acc:HGNC:72] |
| ENSG00000162511 | HGLibA_26021 | 14 | -1.008058689 | LAPTM5 | lysosomal protein transmembrane 5 [Source:HGNC Symbol;Acc:HGNC:29612] |
| ENSG00000162599 | HGLibA_31799 | 14 | -1.008058689 | NFIA | nuclear factor I A [Source:HGNC Symbol;Acc:HGNC:7784] |
| ENSG00000162613 | HGLibA_18149 | 14 | -1.008058689 | FUBP1 | far upstream element binding protein 1 [Source:HGNC Symbol;Acc:HGNC:4004] |
| ENSG00000162620 | HGLibA_27457 | 14 | -1.008058689 | LRRIQ3 | leucine rich repeats and IQ motif containing 3 [Source:HGNC Symbol;Acc:HGNC:28318] |
| ENSG00000162746 | HGLibA_17245 | 14 | -1.008058689 | FCRLB | Fc receptor like B [Source:HGNC Symbol;Acc:HGNC:26431] |
| ENSG00000162819 | HGLibA_04837 | 14 | -1.008058689 | BROX | BRO1 domain and CAAX motif containing [Source:HGNC Symbol;Acc:HGNC:26512] |
| ENSG00000162961 | HGLibA_13917 | 14 | -1.008058689 | DPY30 | histone methyltransferase complex regulatory subunit [Source:HGNC Symbol;Acc:HGNC:24590]" |
| ENSG00000163788 | HGLibA_45937 | 14 | -1.008058689 | SNRK | SNF related kinase [Source:HGNC Symbol;Acc:HGNC:30598] |
| ENSG00000164048 | HGLibA_56336 | 14 | -1.008058689 | ZNF589 | zinc finger protein 589 [Source:HGNC Symbol;Acc:HGNC:16747] |
| ENSG00000164171 | HGLibA_23725 | 14 | -1.008058689 | ITGA2 | integrin subunit alpha 2 [Source:HGNC Symbol;Acc:HGNC:6137] |
| ENSG00000164414 | HGLibA_44948 | 14 | -1.008058689 | SLC35A1 | solute carrier family 35 member A1 [Source:HGNC Symbol;Acc:HGNC:11021] |
| ENSG00000165606 | HGLibA_13980 | 14 | -1.008058689 | DRGX | dorsal root ganglia homeobox [Source:HGNC Symbol;Acc:HGNC:21536] |
| ENSG00000165632 | HGLibA_48230 | 14 | -1.008058689 | TAF3 | TATA-box binding protein associated factor 3 [Source:HGNC Symbol;Acc:HGNC:17303] |
| ENSG00000165699 | HGLibA_51811 | 14 | -1.008058689 | TSC1 | TSC complex subunit 1 [Source:HGNC Symbol;Acc:HGNC:12362] |
| ENSG00000166262 | HGLibA_16530 | 14 | -1.008058689 | FAM227B | family with sequence similarity 227 member B [Source:HGNC Symbol;Acc:HGNC:26543] |
| ENSG00000166710 | HGLibA_03946 | 14 | -1.008058689 | B2M | beta-2-microglobulin [Source:HGNC Symbol;Acc:HGNC:914] |
| ENSG00000166747 | HGLibA_02284 | 14 | -1.008058689 | AP1G1 | adaptor related protein complex 1 subunit gamma 1 [Source:HGNC Symbol;Acc:HGNC:555] |
| ENSG00000167196 | HGLibA_17048 | 14 | -1.008058689 | FBXO22 | F-box protein 22 [Source:HGNC Symbol;Acc:HGNC:13593] |
| ENSG00000167414 | HGLibA_19534 | 14 | -1.008058689 | GNG8 | G protein subunit gamma 8 [Source:HGNC Symbol;Acc:HGNC:19664] |
| ENSG00000167434 | HGLibA_06809 | 14 | -1.008058689 | CA4 | carbonic anhydrase 4 [Source:HGNC Symbol;Acc:HGNC:1375] |
| ENSG00000167702 | HGLibA_24972 | 14 | -1.008058689 | KIFC2 | kinesin family member C2 [Source:HGNC Symbol;Acc:HGNC:29530] |
| ENSG00000169035 | HGLibA_25267 | 14 | -1.008058689 | KLK7 | kallikrein related peptidase 7 [Source:HGNC Symbol;Acc:HGNC:6368] |
| ENSG00000169750 | HGLibA_40048 | 14 | -1.008058689 | RAC3 | Rac family small GTPase 3 [Source:HGNC Symbol;Acc:HGNC:9803] |
| ENSG00000171180 | HGLibA_33838 | 14 | -1.008058689 | OR2M4 | olfactory receptor family 2 subfamily M member 4 [Source:HGNC Symbol;Acc:HGNC:8270] |
| ENSG00000171208 | HGLibA_31708 | 14 | -1.008058689 | NETO2 | neuropilin and tolloid like 2 [Source:HGNC Symbol;Acc:HGNC:14644] |
| ENSG00000171680 | HGLibA_37117 | 14 | -1.008058689 | PLEKHG5 | pleckstrin homology and RhoGEF domain containing G5 [Source:HGNC Symbol;Acc:HGNC:29105] |
| ENSG00000171680 | HGLibA_37116 | 14 | -1.008058689 | PLEKHG5 | pleckstrin homology and RhoGEF domain containing G5 [Source:HGNC Symbol;Acc:HGNC:29105] |
| ENSG00000171847 | HGLibA_16792 | 14 | -1.008058689 | FAM90A1 | family with sequence similarity 90 member A1 [Source:HGNC Symbol;Acc:HGNC:25526] |
| ENSG00000172869 | HGLibA_13453 | 14 | -1.008058689 | DMXL1 | Dmx like 1 [Source:HGNC Symbol;Acc:HGNC:2937] |
| ENSG00000173041 | HGLibA_56523 | 14 | -1.008058689 | ZNF680 | zinc finger protein 680 [Source:HGNC Symbol;Acc:HGNC:26897] |
| ENSG00000173275 | HGLibA_56022 | 14 | -1.008058689 | ZNF449 | zinc finger protein 449 [Source:HGNC Symbol;Acc:HGNC:21039] |
| ENSG00000173674 | HGLibA_14712 | 14 | -1.008058689 | EIF1AX | eukaryotic translation initiation factor 1A X-linked [Source:HGNC Symbol;Acc:HGNC:3250] |
| ENSG00000173812 | HGLibA_14706 | 14 | -1.008058689 | EIF1 | eukaryotic translation initiation factor 1 [Source:HGNC Symbol;Acc:HGNC:3249] |
| ENSG00000173848 | HGLibA_31700 | 14 | -1.008058689 | NET1 | neuroepithelial cell transforming 1 [Source:HGNC Symbol;Acc:HGNC:14592] |
| ENSG00000174482 | HGLibA_26588 | 14 | -1.008058689 | LINGO2 | leucine rich repeat and Ig domain containing 2 [Source:HGNC Symbol;Acc:HGNC:21207] |
| ENSG00000175197 | HGLibA_12614 | 14 | -1.008058689 | DDIT3 | DNA damage inducible transcript 3 [Source:HGNC Symbol;Acc:HGNC:2726] |
| ENSG00000175414 | HGLibA_02925 | 14 | -1.008058689 | ARL10 | ADP ribosylation factor like GTPase 10 [Source:HGNC Symbol;Acc:HGNC:22042] |
| ENSG00000175634 | HGLibA_42178 | 14 | -1.008058689 | RPS6KB2 | ribosomal protein S6 kinase B2 [Source:HGNC Symbol;Acc:HGNC:10437] |
| ENSG00000176473 | HGLibA_54053 | 14 | -1.008058689 | WDR25 | WD repeat domain 25 [Source:HGNC Symbol;Acc:HGNC:21064] |
| ENSG00000176601 | HGLibA_28177 | 14 | -1.008058689 | MAP3K19 | mitogen-activated protein kinase kinase kinase 19 [Source:HGNC Symbol;Acc:HGNC:26249] |
| ENSG00000177212 | HGLibA_33876 | 14 | -1.008058689 | OR2T33 | olfactory receptor family 2 subfamily T member 33 [Source:HGNC Symbol;Acc:HGNC:31255] |
| ENSG00000178934 | HGLibA_26376 | 14 | -1.008058689 | LGALS7B | galectin 7B [Source:HGNC Symbol;Acc:HGNC:34447] |
| ENSG00000179133 | HGLibA_05086 | 14 | -1.008058689 | C10orf67 | chromosome 10 open reading frame 67 [Source:HGNC Symbol;Acc:HGNC:28716] |
| ENSG00000179222 | HGLibA_27967 | 14 | -1.008058689 | MAGED1 | MAGE family member D1 [Source:HGNC Symbol;Acc:HGNC:6813] |
| ENSG00000179363 | HGLibA_50367 | 14 | -1.008058689 | TMEM31 | transmembrane protein 31 [Source:HGNC Symbol;Acc:HGNC:28601] |
| ENSG00000179632 | HGLibA_27869 | 14 | -1.008058689 | MAF1 | negative regulator of RNA polymerase III [Source:HGNC Symbol;Acc:HGNC:24966]" |
| ENSG00000180658 | HGLibA_33735 | 14 | -1.008058689 | OR2A4 | olfactory receptor family 2 subfamily A member 4 [Source:HGNC Symbol;Acc:HGNC:14729] |
| ENSG00000180787 | HGLibA_55199 | 14 | -1.008058689 | ZFP3 | ZFP3 zinc finger protein [Source:HGNC Symbol;Acc:HGNC:12861] |
| ENSG00000180881 | HGLibA_07200 | 14 | -1.008058689 | CAPS2 | calcyphosine 2 [Source:HGNC Symbol;Acc:HGNC:16471] |
| ENSG00000182107 | HGLibA_50363 | 14 | -1.008058689 | TMEM30B | transmembrane protein 30B [Source:HGNC Symbol;Acc:HGNC:27254] |
| ENSG00000182372 | HGLibA_10083 | 14 | -1.008058689 | CLN8 | transmembrane ER and ERGIC protein [Source:HGNC Symbol;Acc:HGNC:2079]" |
| ENSG00000182901 | HGLibA_41102 | 14 | -1.008058689 | RGS7 | regulator of G protein signaling 7 [Source:HGNC Symbol;Acc:HGNC:10003] |
| ENSG00000183283 | HGLibA_12368 | 14 | -1.008058689 | DAZAP2 | DAZ associated protein 2 [Source:HGNC Symbol;Acc:HGNC:2684] |
| ENSG00000184530 | HGLibA_06499 | 14 | -1.008058689 | C6orf58 | chromosome 6 open reading frame 58 [Source:HGNC Symbol;Acc:HGNC:20960] |
| ENSG00000184659 | HGLibA_17849 | 14 | -1.008058689 | FOXD4L4 | forkhead box D4 like 4 [Source:HGNC Symbol;Acc:HGNC:23762] |
| ENSG00000184887 | HGLibA_04917 | 14 | -1.008058689 | BTBD6 | BTB domain containing 6 [Source:HGNC Symbol;Acc:HGNC:19897] |
| ENSG00000185926 | HGLibA_33960 | 14 | -1.008058689 | OR4C46 | olfactory receptor family 4 subfamily C member 46 [Source:HGNC Symbol;Acc:HGNC:31271] |
| ENSG00000186185 | HGLibA_24877 | 14 | -1.008058689 | KIF18B | kinesin family member 18B [Source:HGNC Symbol;Acc:HGNC:27102] |
| ENSG00000186230 | HGLibA_56670 | 14 | -1.008058689 | ZNF749 | zinc finger protein 749 [Source:HGNC Symbol;Acc:HGNC:32783] |
| ENSG00000186847 | HGLibA_25425 | 14 | -1.008058689 | KRT14 | keratin 14 [Source:HGNC Symbol;Acc:HGNC:6416] |
| ENSG00000188340 | HGLibA_34433 | 14 | -1.008058689 | OR6N2 | olfactory receptor family 6 subfamily N member 2 [Source:HGNC Symbol;Acc:HGNC:15035] |
| ENSG00000188643 | HGLibA_42549 | 14 | -1.008058689 | S100A16 | S100 calcium binding protein A16 [Source:HGNC Symbol;Acc:HGNC:20441] |
| ENSG00000188710 | HGLibA_39736 | 14 | -1.008058689 | QRFP | pyroglutamylated RFamide peptide [Source:HGNC Symbol;Acc:HGNC:29982] |
| ENSG00000189157 | HGLibA_16616 | 14 | -1.008058689 | FAM47E | family with sequence similarity 47 member E [Source:HGNC Symbol;Acc:HGNC:34343] |
| ENSG00000196335 | HGLibA_47481 | 14 | -1.008058689 | STK31 | serine/threonine kinase 31 [Source:HGNC Symbol;Acc:HGNC:11407] |
| ENSG00000196352 | HGLibA_08412 | 14 | -1.008058689 | CD55 | CD55 molecule (Cromer blood group) [Source:HGNC Symbol;Acc:HGNC:2665] |
| ENSG00000196605 | HGLibA_56865 | 14 | -1.008058689 | ZNF846 | zinc finger protein 846 [Source:HGNC Symbol;Acc:HGNC:27260] |
| ENSG00000196632 | HGLibA_54338 | 14 | -1.008058689 | WNK3 | WNK lysine deficient protein kinase 3 [Source:HGNC Symbol;Acc:HGNC:14543] |
| ENSG00000197771 | HGLibA_28632 | 14 | -1.008058689 | MCMBP | minichromosome maintenance complex binding protein [Source:HGNC Symbol;Acc:HGNC:25782] |
| ENSG00000198105 | HGLibA_55660 | 14 | -1.008058689 | ZNF248 | zinc finger protein 248 [Source:HGNC Symbol;Acc:HGNC:13041] |
| ENSG00000198445 | HGLibA_08218 | 14 | -1.008058689 | CCT8L2 | chaperonin containing TCP1 subunit 8 like 2 [Source:HGNC Symbol;Acc:HGNC:15553] |
| ENSG00000203666 | HGLibA_14535 | 14 | -1.008058689 | EFCAB2 | EF-hand calcium binding domain 2 [Source:HGNC Symbol;Acc:HGNC:28166] |
| ENSG00000204033 | HGLibA_27189 | 14 | -1.008058689 | LRIT2 | Ig-like and transmembrane domains 2 [Source:HGNC Symbol;Acc:HGNC:23443]" |
| ENSG00000204052 | HGLibA_27425 | 14 | -1.008058689 | LRRC73 | leucine rich repeat containing 73 [Source:HGNC Symbol;Acc:HGNC:21375] |
| ENSG00000205571 | HGLibA_45786 | 14 | -1.008058689 | SMN2 | centromeric [Source:HGNC Symbol;Acc:HGNC:11118]" |
| ENSG00000212993 | HGLibA_37788 | 14 | -1.008058689 | POU5F1B | POU class 5 homeobox 1B [Source:HGNC Symbol;Acc:HGNC:9223] |
| ENSG00000213030 | HGLibA_09313 | 14 | -1.008058689 | CGB8 | chorionic gonadotropin subunit beta 8 [Source:HGNC Symbol;Acc:HGNC:16453] |
| ENSG00000214285 | HGLibA_32522 | 14 | -1.008058689 | NPS | neuropeptide S [Source:HGNC Symbol;Acc:HGNC:33940] |
| ENSG00000215853 | HGLibA_42201 | 14 | -1.008058689 | RPTN | repetin [Source:HGNC Symbol;Acc:HGNC:26809] |
| ENSG00000223865 | HGLibA_21619 | 14 | -1.008058689 | HLA-DPB1 | DP beta 1 [Source:HGNC Symbol;Acc:HGNC:4940]" |
| ENSG00000227345 | HGLibA_35194 | 14 | -1.008058689 | PARG | poly(ADP-ribose) glycohydrolase [Source:HGNC Symbol;Acc:HGNC:8605] |
| ENSG00000240747 | HGLibA_25393 | 14 | -1.008058689 | KRBOX1 | KRAB box domain containing 1 [Source:HGNC Symbol;Acc:HGNC:38708] |
| ENSG00000243130 | HGLibA_39036 | 14 | -1.008058689 | PSG11 | pregnancy specific beta-1-glycoprotein 11 [Source:HGNC Symbol;Acc:HGNC:9516] |
| ENSG00000259075 | HGLibA_37446 | 14 | -1.008058689 | POC1B-GALNT4 | POC1B-GALNT4 readthrough [Source:HGNC Symbol;Acc:HGNC:42957] |
| ENSG00000260287 | HGLibA_48550 | 14 | -1.008058689 | TBC1D3G | TBC1 domain family member 3G [Source:HGNC Symbol;Acc:HGNC:29860] |
| ENSG00000274226 | HGLibA_48553 | 14 | -1.008058689 | TBC1D3H | TBC1 domain family member 3H [Source:HGNC Symbol;Acc:HGNC:30708] |
| ENSG00000274529 | HGLibA_43177 | 14 | -1.008058689 | SEBOX | SEBOX homeobox [Source:HGNC Symbol;Acc:HGNC:32942] |
| HIST2H2AB | HGLibA_21529 | 14 | -1.008058689 | HIST2H2AB | - |
| hsa-mir-1225 | HGLibA_57297 | 14 | -1.008058689 | hsa-mir-1225 | - |
| hsa-mir-1285-1 | HGLibA_57599 | 14 | -1.008058689 | hsa-mir-1285-1 | - |
| hsa-mir-187 | HGLibA_58011 | 14 | -1.008058689 | hsa-mir-187 | - |
| hsa-mir-338 | HGLibA_59002 | 14 | -1.008058689 | hsa-mir-338 | - |
| hsa-mir-4260 | HGLibA_59746 | 14 | -1.008058689 | hsa-mir-4260 | - |
| hsa-mir-4296 | HGLibA_59897 | 14 | -1.008058689 | hsa-mir-4296 | - |

| | | | | | |
|---|---|---|---|---|---|
| hsa-mir-4741 | HGLibA_61062 | 14 | -1.008058689 | hsa-mir-4741 | - |
| hsa-mir-483 | HGLibA_61325 | 14 | -1.008058689 | hsa-mir-483 | - |
| hsa-mir-5089 | HGLibA_61502 | 14 | -1.008058689 | hsa-mir-5089 | - |
| hsa-mir-516a-2 | HGLibA_61612 | 14 | -1.008058689 | hsa-mir-516a-2 | - |
| hsa-mir-520c | HGLibA_61741 | 14 | -1.008058689 | hsa-mir-520c | - |
| hsa-mir-539 | HGLibA_61811 | 14 | -1.008058689 | hsa-mir-539 | - |
| hsa-mir-6069 | HGLibA_62526 | 14 | -1.008058689 | hsa-mir-6069 | - |
| hsa-mir-651 | HGLibA_62891 | 14 | -1.008058689 | hsa-mir-651 | - |
| hsa-mir-6729 | HGLibA_63069 | 14 | -1.008058689 | hsa-mir-6729 | - |
| hsa-mir-6865 | HGLibA_63646 | 14 | -1.008058689 | hsa-mir-6865 | - |
| hsa-mir-6877 | HGLibA_63692 | 14 | -1.008058689 | hsa-mir-6877 | - |
| hsa-mir-6878 | HGLibA_63695 | 14 | -1.008058689 | hsa-mir-6878 | - |
| LOC100287534 | HGLibA_26825 | 14 | -1.008058689 | LOC100287534 | - |
| LOC283403 | HGLibA_26908 | 14 | -1.008058689 | LOC283403 | - |
| MLL5 | HGLibA_29405 | 14 | -1.008058689 | MLL5 | - |
| PAPD4 | HGLibA_35121 | 14 | -1.008058689 | PAPD4 | - |
| TBC1D29 | HGLibA_48525 | 14 | -1.008058689 | TBC1D29 | - |
| TMEM110 | HGLibA_49874 | 14 | -1.008058689 | TMEM110 | - |
| AGXT2L2 | HGLibA_01279 | 13 | -1.017104032 | AGXT2L2 | - |
| ATP5H | HGLibA_03663 | 13 | -1.017104032 | ATP5H | - |
| C17orf62 | HGLibA_05556 | 13 | -1.017104032 | C17orf62 | - |
| C3orf37 | HGLibA_06240 | 13 | -1.017104032 | C3orf37 | - |
| DHRSX_Y | HGLibA_13159 | 13 | -1.017104032 | DHRSX_Y | - |
| DPCR1 | HGLibA_13815 | 13 | -1.017104032 | DPCR1 | - |
| ENSG00000003400 | HGLibA_07299 | 13 | -1.017104032 | CASP10 | caspase 10 [Source:HGNC Symbol;Acc:HGNC:1500] |
| ENSG00000006652 | HGLibA_22738 | 13 | -1.017104032 | IFRD1 | interferon related developmental regulator 1 [Source:HGNC Symbol;Acc:HGNC:5456] |
| ENSG00000007216 | HGLibA_44386 | 13 | -1.017104032 | SLC13A2 | solute carrier family 13 member 2 [Source:HGNC Symbol;Acc:HGNC:10917] |
| ENSG00000008294 | HGLibA_46372 | 13 | -1.017104032 | SPAG9 | sperm associated antigen 9 [Source:HGNC Symbol;Acc:HGNC:14524] |
| ENSG00000010361 | HGLibA_18213 | 13 | -1.017104032 | FUZ | fuzzy planar cell polarity protein [Source:HGNC Symbol;Acc:HGNC:26219] |
| ENSG00000011485 | HGLibA_38213 | 13 | -1.017104032 | PPP5C | protein phosphatase 5 catalytic subunit [Source:HGNC Symbol;Acc:HGNC:9322] |
| ENSG00000039600 | HGLibA_46262 | 13 | -1.017104032 | SOX30 | SRY-box 30 [Source:HGNC Symbol;Acc:HGNC:30635] |
| ENSG00000054983 | HGLibA_18509 | 13 | -1.017104032 | GALC | galactosylceramidase [Source:HGNC Symbol;Acc:HGNC:4115] |
| ENSG00000059573 | HGLibA_01539 | 13 | -1.017104032 | ALDH18A1 | aldehyde dehydrogenase 18 family member A1 [Source:HGNC Symbol;Acc:HGNC:9722] |
| ENSG00000063515 | HGLibA_20402 | 13 | -1.017104032 | GSC2 | goosecoid homeobox 2 [Source:HGNC Symbol;Acc:HGNC:4613] |
| ENSG00000064886 | HGLibA_09439 | 13 | -1.017104032 | CHI3L2 | chitinase 3 like 2 [Source:HGNC Symbol;Acc:HGNC:1933] |
| ENSG00000065970 | HGLibA_17889 | 13 | -1.017104032 | FOXJ2 | forkhead box J2 [Source:HGNC Symbol;Acc:HGNC:24818] |
| ENSG00000066926 | HGLibA_17270 | 13 | -1.017104032 | FECH | ferrochelatase [Source:HGNC Symbol;Acc:HGNC:3647] |
| ENSG00000068323 | HGLibA_49159 | 13 | -1.017104032 | TFE3 | transcription factor binding to IGHM enhancer 3 [Source:HGNC Symbol;Acc:HGNC:11752] |
| ENSG00000079739 | HGLibA_36260 | 13 | -1.017104032 | PGM1 | phosphoglucomutase 1 [Source:HGNC Symbol;Acc:HGNC:8905] |
| ENSG00000081870 | HGLibA_22305 | 13 | -1.017104032 | HSPB11 | heat shock protein family B (small) member 11 [Source:HGNC Symbol;Acc:HGNC:25019] |
| ENSG00000083123 | HGLibA_04282 | 13 | -1.017104032 | BCKDHB | branched chain keto acid dehydrogenase E1 subunit beta [Source:HGNC Symbol;Acc:HGNC:987] |
| ENSG00000083799 | HGLibA_12050 | 13 | -1.017104032 | CYLD | CYLD lysine 63 deubiquitinase [Source:HGNC Symbol;Acc:HGNC:2584] |
| ENSG00000086696 | HGLibA_22187 | 13 | -1.017104032 | HSD17B2 | hydroxysteroid 17-beta dehydrogenase 2 [Source:HGNC Symbol;Acc:HGNC:5211] |
| ENSG00000097096 | HGLibA_47943 | 13 | -1.017104032 | SYDE2 | synapse defective Rho GTPase homolog 2 [Source:HGNC Symbol;Acc:HGNC:25841] |
| ENSG00000099399 | HGLibA_27945 | 13 | -1.017104032 | MAGEB2 | MAGE family member B2 [Source:HGNC Symbol;Acc:HGNC:6809] |
| ENSG00000100191 | HGLibA_45272 | 13 | -1.017104032 | SLC5A4 | solute carrier family 5 member 4 [Source:HGNC Symbol;Acc:HGNC:11039] |
| ENSG00000100393 | HGLibA_15230 | 13 | -1.017104032 | EP300 | E1A binding protein p300 [Source:HGNC Symbol;Acc:HGNC:3373] |
| ENSG00000100425 | HGLibA_04770 | 13 | -1.017104032 | BRD1 | bromodomain containing 1 [Source:HGNC Symbol;Acc:HGNC:1102] |
| ENSG00000102575 | HGLibA_00515 | 13 | -1.017104032 | ACP5 | tartrate resistant [Source:HGNC Symbol;Acc:HGNC:124]" |
| ENSG00000102580 | HGLibA_13615 | 13 | -1.017104032 | DNAJC3 | DnaJ heat shock protein family (Hsp40) member C3 [Source:HGNC Symbol;Acc:HGNC:9439] |
| ENSG00000102802 | HGLibA_28823 | 13 | -1.017104032 | MEDAG | mesenteric estrogen dependent adipogenesis [Source:HGNC Symbol;Acc:HGNC:25926] |
| ENSG00000102974 | HGLibA_11595 | 13 | -1.017104032 | CTCF | CCCTC-binding factor [Source:HGNC Symbol;Acc:HGNC:13723] |
| ENSG00000102996 | HGLibA_29496 | 13 | -1.017104032 | MMP15 | matrix metallopeptidase 15 [Source:HGNC Symbol;Acc:HGNC:7161] |
| ENSG00000103642 | HGLibA_25916 | 13 | -1.017104032 | LACTB | lactamase beta [Source:HGNC Symbol;Acc:HGNC:16468] |
| ENSG00000105229 | HGLibA_36515 | 13 | -1.017104032 | PIAS4 | protein inhibitor of activated STAT 4 [Source:HGNC Symbol;Acc:HGNC:17002] |
| ENSG00000105371 | HGLibA_22503 | 13 | -1.017104032 | ICAM4 | intercellular adhesion molecule 4 (Landsteiner-Wiener blood group) [Source:HGNC Symbol;Acc:HGNC:5347] |
| ENSG00000105443 | HGLibA_12239 | 13 | -1.017104032 | CYTH2 | cytohesin 2 [Source:HGNC Symbol;Acc:HGNC:9502] |
| ENSG00000105829 | HGLibA_04430 | 13 | -1.017104032 | BET1 | Bet1 golgi vesicular membrane trafficking protein [Source:HGNC Symbol;Acc:HGNC:14562] |
| ENSG00000106554 | HGLibA_09369 | 13 | -1.017104032 | CHCHD3 | coiled-coil-helix-coiled-coil-helix domain containing 3 [Source:HGNC Symbol;Acc:HGNC:21906] |
| ENSG00000106633 | HGLibA_18787 | 13 | -1.017104032 | GCK | glucokinase [Source:HGNC Symbol;Acc:HGNC:4195] |
| ENSG00000107372 | HGLibA_55158 | 13 | -1.017104032 | ZFAND5 | zinc finger AN1-type containing 5 [Source:HGNC Symbol;Acc:HGNC:13008] |
| ENSG00000109220 | HGLibA_09447 | 13 | -1.017104032 | CHIC2 | cysteine rich hydrophobic domain 2 [Source:HGNC Symbol;Acc:HGNC:1935] |
| ENSG00000109586 | HGLibA_18570 | 13 | -1.017104032 | GALNT7 | polypeptide N-acetylgalactosaminyltransferase 7 [Source:HGNC Symbol;Acc:HGNC:4129] |
| ENSG00000109771 | HGLibA_27215 | 13 | -1.017104032 | LRP2BP | LRP2 binding protein [Source:HGNC Symbol;Acc:HGNC:25434] |
| ENSG00000109775 | HGLibA_52858 | 13 | -1.017104032 | UFSP2 | UFM1 specific peptidase 2 [Source:HGNC Symbol;Acc:HGNC:25640] |
| ENSG00000109906 | HGLibA_54825 | 13 | -1.017104032 | ZBTB16 | zinc finger and BTB domain containing 16 [Source:HGNC Symbol;Acc:HGNC:12930] |
| ENSG00000110675 | HGLibA_14940 | 13 | -1.017104032 | ELMOD1 | ELMO domain containing 1 [Source:HGNC Symbol;Acc:HGNC:25334] |
| ENSG00000111275 | HGLibA_01558 | 13 | -1.017104032 | ALDH2 | aldehyde dehydrogenase 2 family member [Source:HGNC Symbol;Acc:HGNC:404] |
| ENSG00000111783 | HGLibA_40970 | 13 | -1.017104032 | RFX4 | regulatory factor X4 [Source:HGNC Symbol;Acc:HGNC:9985] |
| ENSG00000113889 | HGLibA_25343 | 13 | -1.017104032 | KNG1 | kininogen 1 [Source:HGNC Symbol;Acc:HGNC:6383] |
| ENSG00000114098 | HGLibA_03033 | 13 | -1.017104032 | ARMC8 | armadillo repeat containing 8 [Source:HGNC Symbol;Acc:HGNC:24999] |
| ENSG00000116030 | HGLibA_47776 | 13 | -1.017104032 | SUMO1 | small ubiquitin-like modifier 1 [Source:HGNC Symbol;Acc:HGNC:12502] |
| ENSG00000116212 | HGLibA_27359 | 13 | -1.017104032 | LRRC42 | leucine rich repeat containing 42 [Source:HGNC Symbol;Acc:HGNC:28792] |
| ENSG00000116830 | HGLibA_52170 | 13 | -1.017104032 | TTF2 | transcription termination factor 2 [Source:HGNC Symbol;Acc:HGNC:12398] |
| ENSG00000117000 | HGLibA_41346 | 13 | -1.017104032 | RLF | rearranged L-myc fusion [Source:HGNC Symbol;Acc:HGNC:10025] |
| ENSG00000117322 | HGLibA_11052 | 13 | -1.017104032 | CR2 | complement C3d receptor 2 [Source:HGNC Symbol;Acc:HGNC:2336] |
| ENSG00000121897 | HGLibA_26471 | 13 | -1.017104032 | LIAS | lipoic acid synthetase [Source:HGNC Symbol;Acc:HGNC:16429] |
| ENSG00000125084 | HGLibA_54343 | 13 | -1.017104032 | WNT1 | Wnt family member 1 [Source:HGNC Symbol;Acc:HGNC:12774] |
| ENSG00000125378 | HGLibA_04609 | 13 | -1.017104032 | BMP4 | bone morphogenetic protein 4 [Source:HGNC Symbol;Acc:HGNC:1071] |
| ENSG00000126856 | HGLibA_38399 | 13 | -1.017104032 | PRDM7 | PR/SET domain 7 [Source:HGNC Symbol;Acc:HGNC:9351] |
| ENSG00000127472 | HGLibA_36919 | 13 | -1.017104032 | PLA2G5 | phospholipase A2 group V [Source:HGNC Symbol;Acc:HGNC:9038] |
| ENSG00000127564 | HGLibA_36833 | 13 | -1.017104032 | PKMYT1 | membrane associated tyrosine/threonine 1 [Source:HGNC Symbol;Acc:HGNC:29650]" |
| ENSG00000127920 | HGLibA_19511 | 13 | -1.017104032 | GNG11 | G protein subunit gamma 11 [Source:HGNC Symbol;Acc:HGNC:4403] |
| ENSG00000128973 | HGLibA_10082 | 13 | -1.017104032 | CLN6 | transmembrane ER protein [Source:HGNC Symbol;Acc:HGNC:2077]" |
| ENSG00000129749 | HGLibA_09565 | 13 | -1.017104032 | CHRNA10 | cholinergic receptor nicotinic alpha 10 subunit [Source:HGNC Symbol;Acc:HGNC:13800] |
| ENSG00000130962 | HGLibA_38869 | 13 | -1.017104032 | PRRG1 | proline rich and Gla domain 1 [Source:HGNC Symbol;Acc:HGNC:9469] |
| ENSG00000132199 | HGLibA_15154 | 13 | -1.017104032 | ENOSF1 | enolase superfamily member 1 [Source:HGNC Symbol;Acc:HGNC:30365] |
| ENSG00000132514 | HGLibA_09922 | 13 | -1.017104032 | CLEC10A | C-type lectin domain containing 10A [Source:HGNC Symbol;Acc:HGNC:16916] |
| ENSG00000134153 | HGLibA_15021 | 13 | -1.017104032 | EMC7 | ER membrane protein complex subunit 7 [Source:HGNC Symbol;Acc:HGNC:24301] |
| ENSG00000134207 | HGLibA_48083 | 13 | -1.017104032 | SYT6 | synaptotagmin 6 [Source:HGNC Symbol;Acc:HGNC:18638] |
| ENSG00000134453 | HGLibA_40496 | 13 | -1.017104032 | RBM17 | RNA binding motif protein 17 [Source:HGNC Symbol;Acc:HGNC:16944] |
| ENSG00000135506 | HGLibA_34634 | 13 | -1.017104032 | OS9 | endoplasmic reticulum lectin [Source:HGNC Symbol;Acc:HGNC:16994]" |
| ENSG00000137200 | HGLibA_10232 | 13 | -1.017104032 | CMTR1 | cap methyltransferase 1 [Source:HGNC Symbol;Acc:HGNC:21077] |
| ENSG00000137411 | HGLibA_53527 | 13 | -1.017104032 | VARS2 | mitochondrial [Source:HGNC Symbol;Acc:HGNC:21642]" |
| ENSG00000137628 | HGLibA_12763 | 13 | -1.017104032 | DDX60 | DExD/H-box helicase 60 [Source:HGNC Symbol;Acc:HGNC:25942] |
| ENSG00000137959 | HGLibA_22613 | 13 | -1.017104032 | IFI44L | interferon induced protein 44 like [Source:HGNC Symbol;Acc:HGNC:17817] |
| ENSG00000138722 | HGLibA_29545 | 13 | -1.017104032 | MMRN1 | multimerin 1 [Source:HGNC Symbol;Acc:HGNC:7178] |
| ENSG00000139734 | HGLibA_13223 | 13 | -1.017104032 | DIAPH3 | diaphanous related formin 3 [Source:HGNC Symbol;Acc:HGNC:15480] |
| ENSG00000140319 | HGLibA_46999 | 13 | -1.017104032 | SRP14 | signal recognition particle 14 [Source:HGNC Symbol;Acc:HGNC:11299] |
| ENSG00000141098 | HGLibA_18955 | 13 | -1.017104032 | GFOD2 | glucose-fructose oxidoreductase domain containing 2 [Source:HGNC Symbol;Acc:HGNC:28159] |
| ENSG00000143171 | HGLibA_42511 | 13 | -1.017104032 | RXRG | retinoid X receptor gamma [Source:HGNC Symbol;Acc:HGNC:10479] |
| ENSG00000144161 | HGLibA_54988 | 13 | -1.017104032 | ZC3H8 | zinc finger CCCH-type containing 8 [Source:HGNC Symbol;Acc:HGNC:30941] |
| ENSG00000144182 | HGLibA_26633 | 13 | -1.017104032 | LIPT1 | lipoyltransferase 1 [Source:HGNC Symbol;Acc:HGNC:29569] |
| ENSG00000144476 | HGLibA_00447 | 13 | -1.017104032 | ACKR3 | atypical chemokine receptor 3 [Source:HGNC Symbol;Acc:HGNC:23692] |
| ENSG00000145014 | HGLibA_50406 | 13 | -1.017104032 | TMEM44 | transmembrane protein 44 [Source:HGNC Symbol;Acc:HGNC:25120] |
| ENSG00000145526 | HGLibA_08654 | 13 | -1.017104032 | CDH18 | cadherin 18 [Source:HGNC Symbol;Acc:HGNC:1757] |
| ENSG00000146285 | HGLibA_42964 | 13 | -1.017104032 | SCML4 | Scm polycomb group protein like 4 [Source:HGNC Symbol;Acc:HGNC:21397] |
| ENSG00000150201 | HGLibA_18234 | 13 | -1.017104032 | FXYD4 | FXYD domain containing ion transport regulator 4 [Source:HGNC Symbol;Acc:HGNC:4028] |
| ENSG00000150477 | HGLibA_24741 | 13 | -1.017104032 | KIAA1328 | KIAA1328 [Source:HGNC Symbol;Acc:HGNC:29248] |
| ENSG00000151332 | HGLibA_28495 | 13 | -1.017104032 | MBIP | MAP3K12 binding inhibitory protein 1 [Source:HGNC Symbol;Acc:HGNC:20427] |
| ENSG00000152147 | HGLibA_18921 | 13 | -1.017104032 | GEMIN6 | gem nuclear organelle associated protein 6 [Source:HGNC Symbol;Acc:HGNC:20044] |
| ENSG00000155229 | HGLibA_29552 | 13 | -1.017104032 | MMS19 | cytosolic iron-sulfur assembly component [Source:HGNC Symbol;Acc:HGNC:13824]" |
| ENSG00000155886 | HGLibA_44617 | 13 | -1.017104032 | SLC24A2 | solute carrier family 24 member 2 [Source:HGNC Symbol;Acc:HGNC:10976] |
| ENSG00000156471 | HGLibA_39289 | 13 | -1.017104032 | PTDSS1 | phosphatidylserine synthase 1 [Source:HGNC Symbol;Acc:HGNC:9587] |
| ENSG00000159352 | HGLibA_39173 | 13 | -1.017104032 | PSMD4 | non-ATPase 4 [Source:HGNC Symbol;Acc:HGNC:9561]" |
| ENSG00000160973 | HGLibA_17874 | 13 | -1.017104032 | FOXH1 | forkhead box H1 [Source:HGNC Symbol;Acc:HGNC:3814] |
| ENSG00000161847 | HGLibA_40401 | 13 | -1.017104032 | RAVER1 | PTB binding 1 [Source:HGNC Symbol;Acc:HGNC:30296]" |
| ENSG00000163344 | HGLibA_37338 | 13 | -1.017104032 | PMVK | phosphomevalonate kinase [Source:HGNC Symbol;Acc:HGNC:9141] |
| ENSG00000163625 | HGLibA_54010 | 13 | -1.017104032 | WDFY3 | WD repeat and FYVE domain containing 3 [Source:HGNC Symbol;Acc:HGNC:20751] |
| ENSG00000163697 | HGLibA_02382 | 13 | -1.017104032 | APBB2 | amyloid beta precursor protein binding family B member 2 [Source:HGNC Symbol;Acc:HGNC:582] |
| ENSG00000164035 | HGLibA_15030 | 13 | -1.017104032 | EMCN | endomucin [Source:HGNC Symbol;Acc:HGNC:16041] |
| ENSG00000164176 | HGLibA_14465 | 13 | -1.017104032 | EDIL3 | EGF like repeats and discoidin domains 3 [Source:HGNC Symbol;Acc:HGNC:3173] |
| ENSG00000164266 | HGLibA_46682 | 13 | -1.017104032 | SPINK1 | Kazal type 1 [Source:HGNC Symbol;Acc:HGNC:11244]" |
| ENSG00000164325 | HGLibA_50065 | 13 | -1.017104032 | TMEM174 | transmembrane protein 174 [Source:HGNC Symbol;Acc:HGNC:28187] |
| ENSG00000164904 | HGLibA_01584 | 13 | -1.017104032 | ALDH7A1 | aldehyde dehydrogenase 7 family member A1 [Source:HGNC Symbol;Acc:HGNC:877] |
| ENSG00000165669 | HGLibA_16419 | 13 | -1.017104032 | FAM204A | family with sequence similarity 204 member A [Source:HGNC Symbol;Acc:HGNC:25794] |
| ENSG00000165795 | HGLibA_31417 | 13 | -1.017104032 | NDRG2 | NDRG family member 2 [Source:HGNC Symbol;Acc:HGNC:14460] |
| ENSG00000166228 | HGLibA_35361 | 13 | -1.017104032 | PCBD1 | pterin-4 alpha-carbinolamine dehydratase 1 [Source:HGNC Symbol;Acc:HGNC:8646] |
| ENSG00000166839 | HGLibA_01946 | 13 | -1.017104032 | ANKDD1A | ankyrin repeat and death domain containing 1A [Source:HGNC Symbol;Acc:HGNC:28002] |
| ENSG00000167244 | HGLibA_22796 | 13 | -1.017104032 | IGF2 | insulin like growth factor 2 [Source:HGNC Symbol;Acc:HGNC:5466] |
| ENSG00000167487 | HGLibA_25169 | 13 | -1.017104032 | KLHL26 | kelch like family member 26 [Source:HGNC Symbol;Acc:HGNC:25623] |
| ENSG00000167619 | HGLibA_49973 | 13 | -1.017104032 | TMEM145 | transmembrane protein 145 [Source:HGNC Symbol;Acc:HGNC:26912] |
| ENSG00000167767 | HGLibA_25554 | 13 | -1.017104032 | KRT80 | keratin 80 [Source:HGNC Symbol;Acc:HGNC:27056] |
| ENSG00000167840 | HGLibA_55639 | 13 | -1.017104032 | ZNF232 | zinc finger protein 232 [Source:HGNC Symbol;Acc:HGNC:13026] |
| ENSG00000168214 | HGLibA_40648 | 13 | -1.017104032 | RBPJ | recombination signal binding protein for immunoglobulin kappa J region [Source:HGNC Symbol;Acc:HGNC:5724] |

| | | | | | |
|---|---|---|---|---|---|
| ENSG00000168781 | HGLibA_37955 | 13 | -1.017104032 | PPIP5K1 | diphosphoinositol pentakisphosphate kinase 1 [Source:HGNC Symbol;Acc:HGNC:29023] |
| ENSG00000169508 | HGLibA_19991 | 13 | -1.017104032 | GPR183 | G protein-coupled receptor 183 [Source:HGNC Symbol;Acc:HGNC:3128] |
| ENSG00000169871 | HGLibA_51549 | 13 | -1.017104032 | TRIM56 | tripartite motif containing 56 [Source:HGNC Symbol;Acc:HGNC:19028] |
| ENSG00000170381 | HGLibA_43332 | 13 | -1.017104032 | SEMA3E | semaphorin 3E [Source:HGNC Symbol;Acc:HGNC:10727] |
| ENSG00000170852 | HGLibA_24102 | 13 | -1.017104032 | KBTBD2 | kelch repeat and BTB domain containing 2 [Source:HGNC Symbol;Acc:HGNC:21751] |
| ENSG00000170891 | HGLibA_12250 | 13 | -1.017104032 | CYTL1 | cytokine like 1 [Source:HGNC Symbol;Acc:HGNC:24435] |
| ENSG00000172247 | HGLibA_05945 | 13 | -1.017104032 | C1QTNF4 | C1q and TNF related 4 [Source:HGNC Symbol;Acc:HGNC:14346] |
| ENSG00000172590 | HGLibA_30014 | 13 | -1.017104032 | MRPL52 | mitochondrial ribosomal protein L52 [Source:HGNC Symbol;Acc:HGNC:16655] |
| ENSG00000172650 | HGLibA_01152 | 13 | -1.017104032 | AGAP5 | ankyrin repeat and PH domain 5 [Source:HGNC Symbol;Acc:HGNC:23467]" |
| ENSG00000172780 | HGLibA_39949 | 13 | -1.017104032 | RAB43 | member RAS oncogene family [Source:HGNC Symbol;Acc:HGNC:19983]" |
| ENSG00000173914 | HGLibA_40571 | 13 | -1.017104032 | RBM4B | RNA binding motif protein 4B [Source:HGNC Symbol;Acc:HGNC:28842] |
| ENSG00000174007 | HGLibA_09121 | 13 | -1.017104032 | CEP19 | centrosomal protein 19 [Source:HGNC Symbol;Acc:HGNC:28209] |
| ENSG00000174125 | HGLibA_49639 | 13 | -1.017104032 | TLR1 | toll like receptor 1 [Source:HGNC Symbol;Acc:HGNC:11847] |
| ENSG00000175213 | HGLibA_55929 | 13 | -1.017104032 | ZNF408 | zinc finger protein 408 [Source:HGNC Symbol;Acc:HGNC:20041] |
| ENSG00000176390 | HGLibA_11195 | 13 | -1.017104032 | CRLF3 | cytokine receptor like factor 3 [Source:HGNC Symbol;Acc:HGNC:17177] |
| ENSG00000176624 | HGLibA_29032 | 13 | -1.017104032 | MEX3C | mex-3 RNA binding family member C [Source:HGNC Symbol;Acc:HGNC:28040] |
| ENSG00000176956 | HGLibA_27674 | 13 | -1.017104032 | LY6H | lymphocyte antigen 6 family member H [Source:HGNC Symbol;Acc:HGNC:6728] |
| ENSG00000177971 | HGLibA_23239 | 13 | -1.017104032 | IMP3 | U3 small nucleolar ribonucleoprotein [Source:HGNC Symbol;Acc:HGNC:14497]" |
| ENSG00000178999 | HGLibA_03880 | 13 | -1.017104032 | AURKB | aurora kinase B [Source:HGNC Symbol;Acc:HGNC:11390] |
| ENSG00000179088 | HGLibA_05241 | 13 | -1.017104032 | C12orf42 | chromosome 12 open reading frame 42 [Source:HGNC Symbol;Acc:HGNC:24729] |
| ENSG00000180592 | HGLibA_44280 | 13 | -1.017104032 | SKIDA1 | SKI/DACH domain containing 1 [Source:HGNC Symbol;Acc:HGNC:32697] |
| ENSG00000180787 | HGLibA_55198 | 13 | -1.017104032 | ZFP3 | ZFP3 zinc finger protein [Source:HGNC Symbol;Acc:HGNC:12861] |
| ENSG00000180999 | HGLibA_05735 | 13 | -1.017104032 | C1orf105 | chromosome 1 open reading frame 105 [Source:HGNC Symbol;Acc:HGNC:29591] |
| ENSG00000182585 | HGLibA_15282 | 13 | -1.017104032 | EPGN | epithelial mitogen [Source:HGNC Symbol;Acc:HGNC:17470] |
| ENSG00000182704 | HGLibA_51873 | 13 | -1.017104032 | TSKU | small leucine rich proteoglycan [Source:HGNC Symbol;Acc:HGNC:28850]" |
| ENSG00000182712 | HGLibA_10190 | 13 | -1.017104032 | CMC4 | C-X9-C motif containing 4 [Source:HGNC Symbol;Acc:HGNC:35428] |
| ENSG00000183032 | HGLibA_44673 | 13 | -1.017104032 | SLC25A21 | solute carrier family 25 member 21 [Source:HGNC Symbol;Acc:HGNC:14411] |
| ENSG00000183246 | HGLibA_41267 | 13 | -1.017104032 | RIMBP3C | RIMS binding protein 3C [Source:HGNC Symbol;Acc:HGNC:33892] |
| ENSG00000183258 | HGLibA_12710 | 13 | -1.017104032 | DDX41 | DEAD-box helicase 41 [Source:HGNC Symbol;Acc:HGNC:18674] |
| ENSG00000183336 | HGLibA_04684 | 13 | -1.017104032 | BOLA2 | bolA family member 2 [Source:HGNC Symbol;Acc:HGNC:29488] |
| ENSG00000183850 | HGLibA_56643 | 13 | -1.017104032 | ZNF730 | zinc finger protein 730 [Source:HGNC Symbol;Acc:HGNC:32470] |
| ENSG00000184162 | HGLibA_32601 | 13 | -1.017104032 | NR2C2AP | nuclear receptor 2C2 associated protein [Source:HGNC Symbol;Acc:HGNC:30763] |
| ENSG00000184254 | HGLibA_01548 | 13 | -1.017104032 | ALDH1A3 | aldehyde dehydrogenase 1 family member A3 [Source:HGNC Symbol;Acc:HGNC:409] |
| ENSG00000184724 | HGLibA_25816 | 13 | -1.017104032 | KRTAP6-1 | keratin associated protein 6-1 [Source:HGNC Symbol;Acc:HGNC:18931] |
| ENSG00000186638 | HGLibA_24911 | 13 | -1.017104032 | KIF24 | kinesin family member 24 [Source:HGNC Symbol;Acc:HGNC:19916] |
| ENSG00000187054 | HGLibA_50613 | 13 | -1.017104032 | TMPRSS11A | transmembrane serine protease 11A [Source:HGNC Symbol;Acc:HGNC:27954] |
| ENSG00000187486 | HGLibA_24252 | 13 | -1.017104032 | KCNJ11 | potassium voltage-gated channel subfamily J member 11 [Source:HGNC Symbol;Acc:HGNC:6257] |
| ENSG00000187608 | HGLibA_23656 | 13 | -1.017104032 | ISG15 | ISG15 ubiquitin-like modifier [Source:HGNC Symbol;Acc:HGNC:4053] |
| ENSG00000188266 | HGLibA_22456 | 13 | -1.017104032 | HYKK | hydroxylysine kinase [Source:HGNC Symbol;Acc:HGNC:34403] |
| ENSG00000188508 | HGLibA_25860 | 13 | -1.017104032 | KRTDAP | keratinocyte differentiation associated protein [Source:HGNC Symbol;Acc:HGNC:16313] |
| ENSG00000198642 | HGLibA_25229 | 13 | -1.017104032 | KLHL9 | kelch like family member 9 [Source:HGNC Symbol;Acc:HGNC:18732] |
| ENSG00000198795 | HGLibA_56159 | 13 | -1.017104032 | ZNF521 | zinc finger protein 521 [Source:HGNC Symbol;Acc:HGNC:24605] |
| ENSG00000203724 | HGLibA_05871 | 13 | -1.017104032 | C1orf53 | chromosome 1 open reading frame 53 [Source:HGNC Symbol;Acc:HGNC:30003] |
| ENSG00000204655 | HGLibA_29621 | 13 | -1.017104032 | MOG | myelin oligodendrocyte glycoprotein [Source:HGNC Symbol;Acc:HGNC:7197] |
| ENSG00000204952 | HGLibA_17114 | 13 | -1.017104032 | FBXO47 | F-box protein 47 [Source:HGNC Symbol;Acc:HGNC:31969] |
| ENSG00000204979 | HGLibA_30135 | 13 | -1.017104032 | MS4A13 | membrane spanning 4-domains A13 [Source:HGNC Symbol;Acc:HGNC:16674] |
| ENSG00000206559 | HGLibA_55061 | 13 | -1.017104032 | ZCWPW2 | zinc finger CW-type and PWWP domain containing 2 [Source:HGNC Symbol;Acc:HGNC:23574] |
| ENSG00000213965 | HGLibA_32965 | 13 | -1.017104032 | NUDT19 | nudix hydrolase 19 [Source:HGNC Symbol;Acc:HGNC:32036] |
| ENSG00000230522 | HGLibA_28474 | 13 | -1.017104032 | MBD3L2 | methyl-CpG binding domain protein 3 like 2 [Source:HGNC Symbol;Acc:HGNC:18532] |
| ENSG00000240720 | HGLibA_27446 | 13 | -1.017104032 | LRRD1 | leucine rich repeats and death domain containing 1 [Source:HGNC Symbol;Acc:HGNC:34300] |
| ENSG00000244752 | HGLibA_11264 | 13 | -1.017104032 | CRYBB2 | crystallin beta B2 [Source:HGNC Symbol;Acc:HGNC:2398] |
| ENSG00000254598 | HGLibA_11408 | 13 | -1.017104032 | CSNK2A3 | casein kinase 2 alpha 3 [Source:HGNC Symbol;Acc:HGNC:2458] |
| ENSG00000257520 | HGLibA_43762 | 13 | -1.017104032 | SFTA3 | surfactant associated 3 [Source:NCBI gene;Acc:253970] |
| ENSG00000267534 | HGLibA_42595 | 13 | -1.017104032 | S1PR2 | sphingosine-1-phosphate receptor 2 [Source:HGNC Symbol;Acc:HGNC:3169] |
| ENSG00000269343 | HGLibA_56333 | 13 | -1.017104032 | ZNF587B | zinc finger protein 587B [Source:HGNC Symbol;Acc:HGNC:37142] |
| ENSG00000274808 | HGLibA_48541 | 13 | -1.017104032 | TBC1D3B | TBC1 domain family member 3B [Source:HGNC Symbol;Acc:HGNC:27011] |
| HRSP12 | HGLibA_22111 | 13 | -1.017104032 | HRSP12 | - |
| hsa-mir-100 | HGLibA_57151 | 13 | -1.017104032 | hsa-mir-100 | - |
| hsa-mir-1262 | HGLibA_57473 | 13 | -1.017104032 | hsa-mir-1262 | - |
| hsa-mir-1973 | HGLibA_58118 | 13 | -1.017104032 | hsa-mir-1973 | - |
| hsa-mir-2861 | HGLibA_58390 | 13 | -1.017104032 | hsa-mir-2861 | - |
| hsa-mir-3124 | HGLibA_58548 | 13 | -1.017104032 | hsa-mir-3124 | - |
| hsa-mir-3176 | HGLibA_58778 | 13 | -1.017104032 | hsa-mir-3176 | - |
| hsa-mir-4301 | HGLibA_59914 | 13 | -1.017104032 | hsa-mir-4301 | - |
| hsa-mir-4499 | HGLibA_60412 | 13 | -1.017104032 | hsa-mir-4499 | - |
| hsa-mir-4509-2 | HGLibA_60465 | 13 | -1.017104032 | hsa-mir-4509-2 | - |
| hsa-mir-4634 | HGLibA_60643 | 13 | -1.017104032 | hsa-mir-4634 | - |
| hsa-mir-4660 | HGLibA_60750 | 13 | -1.017104032 | hsa-mir-4660 | - |
| hsa-mir-4678 | HGLibA_60830 | 13 | -1.017104032 | hsa-mir-4678 | - |
| hsa-mir-4778 | HGLibA_61221 | 13 | -1.017104032 | hsa-mir-4778 | - |
| hsa-mir-486-2 | HGLibA_61340 | 13 | -1.017104032 | hsa-mir-486-2 | - |
| hsa-mir-516b-2 | HGLibA_61621 | 13 | -1.017104032 | hsa-mir-516b-2 | - |
| hsa-mir-520h | HGLibA_61762 | 13 | -1.017104032 | hsa-mir-520h | - |
| hsa-mir-548f-4 | HGLibA_61982 | 13 | -1.017104032 | hsa-mir-548f-4 | - |
| hsa-mir-548h-2 | HGLibA_61998 | 13 | -1.017104032 | hsa-mir-548h-2 | - |
| hsa-mir-548n | HGLibA_62044 | 13 | -1.017104032 | hsa-mir-548n | - |
| hsa-mir-5702 | HGLibA_62347 | 13 | -1.017104032 | hsa-mir-5702 | - |
| hsa-mir-6770-2 | HGLibA_63251 | 13 | -1.017104032 | hsa-mir-6770-2 | - |
| hsa-mir-6795 | HGLibA_63358 | 13 | -1.017104032 | hsa-mir-6795 | - |
| hsa-mir-6884 | HGLibA_63720 | 13 | -1.017104032 | hsa-mir-6884 | - |
| hsa-mir-7152 | HGLibA_63837 | 13 | -1.017104032 | hsa-mir-7152 | - |
| hsa-mir-762 | HGLibA_63908 | 13 | -1.017104032 | hsa-mir-762 | - |
| hsa-mir-8071-2 | HGLibA_64125 | 13 | -1.017104032 | hsa-mir-8071-2 | - |
| hsa-mir-937 | HGLibA_64321 | 13 | -1.017104032 | hsa-mir-937 | - |
| IL3RA_Y | HGLibA_23164 | 13 | -1.017104032 | IL3RA_Y | - |
| LEPREL4 | HGLibA_26324 | 13 | -1.017104032 | LEPREL4 | - |
| LOC154872 | HGLibA_26896 | 13 | -1.017104032 | LOC154872 | - |
| PPAP2A | HGLibA_37817 | 13 | -1.017104032 | PPAP2A | - |
| SKIV2L2 | HGLibA_44287 | 13 | -1.017104032 | SKIV2L2 | - |
| SPANXF1 | HGLibA_46399 | 13 | -1.017104032 | SPANXF1 | - |
| TCTEX1D1 | HGLibA_48862 | 13 | -1.017104032 | TCTEX1D1 | - |
| TCTEX1D2 | HGLibA_48865 | 13 | -1.017104032 | TCTEX1D2 | - |
| WTH3DI | HGLibA_54432 | 13 | -1.017104032 | WTH3DI | - |
| ATP5D | HGLibA_03646 | 12 | -1.026149375 | ATP5D | - |
| C2orf43 | HGLibA_06129 | 12 | -1.026149375 | C2orf43 | - |
| C7orf34 | HGLibA_06539 | 12 | -1.026149375 | C7orf34 | - |
| C8orf37 | HGLibA_06615 | 12 | -1.026149375 | C8orf37 | - |
| CCDC147 | HGLibA_07631 | 12 | -1.026149375 | CCDC147 | - |
| ENSG00000011426 | HGLibA_02167 | 12 | -1.026149375 | ANLN | anillin actin binding protein [Source:HGNC Symbol;Acc:HGNC:14082] |
| ENSG00000019102 | HGLibA_53846 | 12 | -1.026149375 | VSIG2 | V-set and immunoglobulin domain containing 2 [Source:HGNC Symbol;Acc:HGNC:17149] |
| ENSG00000021461 | HGLibA_12157 | 12 | -1.026149375 | CYP3A43 | cytochrome P450 family 3 subfamily A member 43 [Source:HGNC Symbol;Acc:HGNC:17450] |
| ENSG00000023697 | HGLibA_12992 | 12 | -1.026149375 | DERA | deoxyribose-phosphate aldolase [Source:HGNC Symbol;Acc:HGNC:24269] |
| ENSG00000038210 | HGLibA_36495 | 12 | -1.026149375 | PI4K2B | phosphatidylinositol 4-kinase type 2 beta [Source:HGNC Symbol;Acc:HGNC:18215] |
| ENSG00000040199 | HGLibA_36439 | 12 | -1.026149375 | PHLPP2 | PH domain and leucine rich repeat protein phosphatase 2 [Source:HGNC Symbol;Acc:HGNC:29149] |
| ENSG00000048392 | HGLibA_42256 | 12 | -1.026149375 | RRM2B | ribonucleotide reductase regulatory TP53 inducible subunit M2B [Source:HGNC Symbol;Acc:HGNC:17296] |
| ENSG00000050426 | HGLibA_26340 | 12 | -1.026149375 | LETMD1 | LETM1 domain containing 1 [Source:HGNC Symbol;Acc:HGNC:24241] |
| ENSG00000054793 | HGLibA_03792 | 12 | -1.026149375 | ATP9A | ATPase phospholipid transporting 9A (putative) [Source:HGNC Symbol;Acc:HGNC:13540] |
| ENSG00000055955 | HGLibA_23814 | 12 | -1.026149375 | ITIH4 | inter-alpha-trypsin inhibitor heavy chain family member 4 [Source:HGNC Symbol;Acc:HGNC:6169] |
| ENSG00000058272 | HGLibA_38019 | 12 | -1.026149375 | PPP1R12A | protein phosphatase 1 regulatory subunit 12A [Source:HGNC Symbol;Acc:HGNC:7618] |
| ENSG00000065518 | HGLibA_31517 | 12 | -1.026149375 | NDUFB4 | NADH:ubiquinone oxidoreductase subunit B4 [Source:HGNC Symbol;Acc:HGNC:7699] |
| ENSG00000070501 | HGLibA_37490 | 12 | -1.026149375 | POLB | DNA polymerase beta [Source:HGNC Symbol;Acc:HGNC:9174] |
| ENSG00000070759 | HGLibA_49032 | 12 | -1.026149375 | TESK2 | testis associated actin remodelling kinase 2 [Source:HGNC Symbol;Acc:HGNC:11732] |
| ENSG00000072080 | HGLibA_46772 | 12 | -1.026149375 | SPP2 | secreted phosphoprotein 2 [Source:HGNC Symbol;Acc:HGNC:11256] |
| ENSG00000075826 | HGLibA_43245 | 12 | -1.026149375 | SEC31B | COPII coat complex component [Source:HGNC Symbol;Acc:HGNC:23197]" |
| ENSG00000076554 | HGLibA_51112 | 12 | -1.026149375 | TPD52 | tumor protein D52 [Source:HGNC Symbol;Acc:HGNC:12005] |
| ENSG00000076864 | HGLibA_40228 | 12 | -1.026149375 | RAP1GAP | RAP1 GTPase activating protein [Source:HGNC Symbol;Acc:HGNC:9858] |
| ENSG00000080824 | HGLibA_22251 | 12 | -1.026149375 | HSP90AA1 | heat shock protein 90 alpha family class A member 1 [Source:HGNC Symbol;Acc:HGNC:5253] |
| ENSG00000081019 | HGLibA_42301 | 12 | -1.026149375 | RSBN1 | round spermatid basic protein 1 [Source:HGNC Symbol;Acc:HGNC:25642] |
| ENSG00000092054 | HGLibA_30733 | 12 | -1.026149375 | MYH7 | myosin heavy chain 7 [Source:HGNC Symbol;Acc:HGNC:7577] |
| ENSG00000094914 | HGLibA_00024 | 12 | -1.026149375 | AAAS | aladin WD repeat nucleoporin [Source:HGNC Symbol;Acc:HGNC:13666] |
| ENSG00000095637 | HGLibA_46175 | 12 | -1.026149375 | SORBS1 | sorbin and SH3 domain containing 1 [Source:HGNC Symbol;Acc:HGNC:14565] |
| ENSG00000100012 | HGLibA_43196 | 12 | -1.026149375 | SEC14L3 | SEC14 like lipid binding 3 [Source:HGNC Symbol;Acc:HGNC:18655] |
| ENSG00000100084 | HGLibA_21352 | 12 | -1.026149375 | HIRA | histone cell cycle regulator [Source:HGNC Symbol;Acc:HGNC:4916] |
| ENSG00000100122 | HGLibA_11262 | 12 | -1.026149375 | CRYBB1 | crystallin beta B1 [Source:HGNC Symbol;Acc:HGNC:2397] |
| ENSG00000100324 | HGLibA_48142 | 12 | -1.026149375 | TAB1 | TGF-beta activated kinase 1 (MAP3K7) binding protein 1 [Source:HGNC Symbol;Acc:HGNC:18157] |
| ENSG00000100532 | HGLibA_09329 | 12 | -1.026149375 | CGRRF1 | cell growth regulator with ring finger domain 1 [Source:HGNC Symbol;Acc:HGNC:15528] |
| ENSG00000101911 | HGLibA_38761 | 12 | -1.026149375 | PRPS2 | phosphoribosyl pyrophosphate synthetase 2 [Source:HGNC Symbol;Acc:HGNC:9465] |
| ENSG00000102287 | HGLibA_18385 | 12 | -1.026149375 | GABRE | gamma-aminobutyric acid type A receptor epsilon subunit [Source:HGNC Symbol;Acc:HGNC:4085] |
| ENSG00000102910 | HGLibA_27017 | 12 | -1.026149375 | LONP2 | peroxisomal [Source:HGNC Symbol;Acc:HGNC:20598]" |
| ENSG00000103966 | HGLibA_14675 | 12 | -1.026149375 | EHD4 | EH domain containing 4 [Source:HGNC Symbol;Acc:HGNC:3245] |
| ENSG00000104140 | HGLibA_41211 | 12 | -1.026149375 | RHOV | ras homolog family member V [Source:HGNC Symbol;Acc:HGNC:18313] |
| ENSG00000104886 | HGLibA_37135 | 12 | -1.026149375 | PLEKHJ1 | pleckstrin homology domain containing J1 [Source:HGNC Symbol;Acc:HGNC:18211] |
| ENSG00000105193 | HGLibA_42082 | 12 | -1.026149375 | RPS16 | ribosomal protein S16 [Source:HGNC Symbol;Acc:HGNC:10396] |
| ENSG00000105255 | HGLibA_18078 | 12 | -1.026149375 | FSD1 | fibronectin type III and SPRY domain containing 1 [Source:HGNC Symbol;Acc:HGNC:13745] |
| ENSG00000105732 | HGLibA_56287 | 12 | -1.026149375 | ZNF574 | zinc finger protein 574 [Source:HGNC Symbol;Acc:HGNC:26166] |

| | | | | | |
|---|---|---|---|---|---|
| ENSG00000106771 | HGLibA_50299 | 12 | -1.026149375 | TMEM245 | transmembrane protein 245 [Source:HGNC Symbol;Acc:HGNC:1363] |
| ENSG00000107165 | HGLibA_52475 | 12 | -1.026149375 | TYRP1 | tyrosinase related protein 1 [Source:HGNC Symbol;Acc:HGNC:12450] |
| ENSG00000107745 | HGLibA_29222 | 12 | -1.026149375 | MICU1 | mitochondrial calcium uptake 1 [Source:HGNC Symbol;Acc:HGNC:1530] |
| ENSG00000108018 | HGLibA_46183 | 12 | -1.026149375 | SORCS1 | sortilin related VPS10 domain containing receptor 1 [Source:HGNC Symbol;Acc:HGNC:16697] |
| ENSG00000108039 | HGLibA_54554 | 12 | -1.026149375 | XPNPEP1 | X-prolyl aminopeptidase 1 [Source:HGNC Symbol;Acc:HGNC:12822] |
| ENSG00000108055 | HGLibA_45650 | 12 | -1.026149375 | SMC3 | structural maintenance of chromosomes 3 [Source:HGNC Symbol;Acc:HGNC:2468] |
| ENSG00000108506 | HGLibA_23415 | 12 | -1.026149375 | INTS2 | integrator complex subunit 2 [Source:HGNC Symbol;Acc:HGNC:29241] |
| ENSG00000112576 | HGLibA_08077 | 12 | -1.026149375 | CCND3 | cyclin D3 [Source:HGNC Symbol;Acc:HGNC:1585] |
| ENSG00000112584 | HGLibA_16059 | 12 | -1.026149375 | FAM120B | family with sequence similarity 120B [Source:HGNC Symbol;Acc:HGNC:21109] |
| ENSG00000112977 | HGLibA_12317 | 12 | -1.026149375 | DAP | death associated protein [Source:HGNC Symbol;Acc:HGNC:2672] |
| ENSG00000113838 | HGLibA_48586 | 12 | -1.026149375 | TBCCD1 | TBCC domain containing 1 [Source:HGNC Symbol;Acc:HGNC:25546] |
| ENSG00000115561 | HGLibA_09485 | 12 | -1.026149375 | CHMP3 | charged multivesicular body protein 3 [Source:HGNC Symbol;Acc:HGNC:29865] |
| ENSG00000115844 | HGLibA_13375 | 12 | -1.026149375 | DLX2 | distal-less homeobox 2 [Source:HGNC Symbol;Acc:HGNC:2915] |
| ENSG00000116685 | HGLibA_24837 | 12 | -1.026149375 | KIAA2013 | KIAA2013 [Source:HGNC Symbol;Acc:HGNC:28513] |
| ENSG00000119673 | HGLibA_00480 | 12 | -1.026149375 | ACOT2 | acyl-CoA thioesterase 2 [Source:HGNC Symbol;Acc:HGNC:18431] |
| ENSG00000121211 | HGLibA_29565 | 12 | -1.026149375 | MND1 | meiotic nuclear divisions 1 [Source:HGNC Symbol;Acc:HGNC:24839] |
| ENSG00000121211 | HGLibA_29564 | 12 | -1.026149375 | MND1 | meiotic nuclear divisions 1 [Source:HGNC Symbol;Acc:HGNC:24839] |
| ENSG00000121621 | HGLibA_24873 | 12 | -1.026149375 | KIF18A | kinesin family member 18A [Source:HGNC Symbol;Acc:HGNC:29441] |
| ENSG00000122705 | HGLibA_10144 | 12 | -1.026149375 | CLTA | clathrin light chain A [Source:HGNC Symbol;Acc:HGNC:2090] |
| ENSG00000123595 | HGLibA_39990 | 12 | -1.026149375 | RAB9A | member RAS oncogene family [Source:HGNC Symbol;Acc:HGNC:9792]" |
| ENSG00000125817 | HGLibA_09039 | 12 | -1.026149375 | CENPB | centromere protein B [Source:HGNC Symbol;Acc:HGNC:1852] |
| ENSG00000127364 | HGLibA_48400 | 12 | -1.026149375 | TAS2R4 | taste 2 receptor member 4 [Source:HGNC Symbol;Acc:HGNC:14911] |
| ENSG00000128791 | HGLibA_52385 | 12 | -1.026149375 | TWSG1 | twisted gastrulation BMP signaling modulator 1 [Source:HGNC Symbol;Acc:HGNC:12429] |
| ENSG00000129465 | HGLibA_41323 | 12 | -1.026149375 | RIPK3 | receptor interacting serine/threonine kinase 3 [Source:HGNC Symbol;Acc:HGNC:10021] |
| ENSG00000129625 | HGLibA_40787 | 12 | -1.026149375 | REEP5 | receptor accessory protein 5 [Source:HGNC Symbol;Acc:HGNC:30077] |
| ENSG00000130270 | HGLibA_03787 | 12 | -1.026149375 | ATP8B3 | ATPase phospholipid transporting 8B3 [Source:HGNC Symbol;Acc:HGNC:13535] |
| ENSG00000131148 | HGLibA_15023 | 12 | -1.026149375 | EMC8 | ER membrane protein complex subunit 8 [Source:HGNC Symbol;Acc:HGNC:7864] |
| ENSG00000131153 | HGLibA_19106 | 12 | -1.026149375 | GINS2 | GINS complex subunit 2 [Source:HGNC Symbol;Acc:HGNC:24575] |
| ENSG00000132305 | HGLibA_23235 | 12 | -1.026149375 | IMMT | inner membrane mitochondrial protein [Source:HGNC Symbol;Acc:HGNC:6047] |
| ENSG00000132326 | HGLibA_36056 | 12 | -1.026149375 | PER2 | period circadian regulator 2 [Source:HGNC Symbol;Acc:HGNC:8846] |
| ENSG00000132424 | HGLibA_37346 | 12 | -1.026149375 | PNISR | PNN interacting serine and arginine rich protein [Source:HGNC Symbol;Acc:HGNC:21222] |
| ENSG00000134186 | HGLibA_38725 | 12 | -1.026149375 | PRPF38B | pre-mRNA processing factor 38B [Source:HGNC Symbol;Acc:HGNC:25512] |
| ENSG00000134532 | HGLibA_46266 | 12 | -1.026149375 | SOX5 | SRY-box 5 [Source:HGNC Symbol;Acc:HGNC:11201] |
| ENSG00000134627 | HGLibA_36781 | 12 | -1.026149375 | PIWIL4 | piwi like RNA-mediated gene silencing 4 [Source:HGNC Symbol;Acc:HGNC:18444] |
| ENSG00000134690 | HGLibA_08621 | 12 | -1.026149375 | CDCA8 | cell division cycle associated 8 [Source:HGNC Symbol;Acc:HGNC:14629] |
| ENSG00000135049 | HGLibA_01259 | 12 | -1.026149375 | AGTPBP1 | ATP/GTP binding protein 1 [Source:HGNC Symbol;Acc:HGNC:17258] |
| ENSG00000135374 | HGLibA_14904 | 12 | -1.026149375 | ELF5 | E74 like ETS transcription factor 5 [Source:HGNC Symbol;Acc:HGNC:3320] |
| ENSG00000137766 | HGLibA_52998 | 12 | -1.026149375 | UNC13C | unc-13 homolog C [Source:HGNC Symbol;Acc:HGNC:23149] |
| ENSG00000139163 | HGLibA_15632 | 12 | -1.026149375 | ETNK1 | ethanolamine kinase 1 [Source:HGNC Symbol;Acc:HGNC:24649] |
| ENSG00000140577 | HGLibA_11234 | 12 | -1.026149375 | CRTC3 | CREB regulated transcription coactivator 3 [Source:HGNC Symbol;Acc:HGNC:26148] |
| ENSG00000140830 | HGLibA_52440 | 12 | -1.026149375 | TXNL4B | thioredoxin like 4B [Source:HGNC Symbol;Acc:HGNC:26041] |
| ENSG00000142186 | HGLibA_43085 | 12 | -1.026149375 | SCYL1 | SCY1 like pseudokinase 1 [Source:HGNC Symbol;Acc:HGNC:14372] |
| ENSG00000143486 | HGLibA_14751 | 12 | -1.026149375 | EIF2D | eukaryotic translation initiation factor 2D [Source:HGNC Symbol;Acc:HGNC:6583] |
| ENSG00000143891 | HGLibA_18522 | 12 | -1.026149375 | GALM | galactose mutarotase [Source:HGNC Symbol;Acc:HGNC:24063] |
| ENSG00000144671 | HGLibA_44550 | 12 | -1.026149375 | SLC22A14 | solute carrier family 22 member 14 [Source:HGNC Symbol;Acc:HGNC:8495] |
| ENSG00000144935 | HGLibA_51728 | 12 | -1.026149375 | TRPC1 | transient receptor potential cation channel subfamily C member 1 [Source:HGNC Symbol;Acc:HGNC:12333] |
| ENSG00000146678 | HGLibA_22816 | 12 | -1.026149375 | IGFBP1 | insulin like growth factor binding protein 1 [Source:HGNC Symbol;Acc:HGNC:5469] |
| ENSG00000147874 | HGLibA_20888 | 12 | -1.026149375 | HAUS6 | HAUS augmin like complex subunit 6 [Source:HGNC Symbol;Acc:HGNC:25948] |
| ENSG00000151360 | HGLibA_01669 | 12 | -1.026149375 | ALLC | allantoicase [Source:HGNC Symbol;Acc:HGNC:17377] |
| ENSG00000151746 | HGLibA_04494 | 12 | -1.026149375 | BICD1 | BICD cargo adaptor 1 [Source:HGNC Symbol;Acc:HGNC:1049] |
| ENSG00000155363 | HGLibA_29692 | 12 | -1.026149375 | MOV10 | Mov10 RISC complex RNA helicase [Source:HGNC Symbol;Acc:HGNC:7200] |
| ENSG00000155508 | HGLibA_10353 | 12 | -1.026149375 | CNOT8 | CCR4-NOT transcription complex subunit 8 [Source:HGNC Symbol;Acc:HGNC:9207] |
| ENSG00000157131 | HGLibA_06594 | 12 | -1.026149375 | C8A | complement C8 alpha chain [Source:HGNC Symbol;Acc:HGNC:1352] |
| ENSG00000158786 | HGLibA_36894 | 12 | -1.026149375 | PLA2G2F | phospholipase A2 group IIF [Source:HGNC Symbol;Acc:HGNC:30040] |
| ENSG00000159023 | HGLibA_15239 | 12 | -1.026149375 | EPB41 | erythrocyte membrane protein band 4.1 [Source:HGNC Symbol;Acc:HGNC:3377] |
| ENSG00000161551 | HGLibA_56297 | 12 | -1.026149375 | ZNF577 | zinc finger protein 577 [Source:HGNC Symbol;Acc:HGNC:28673] |
| ENSG00000162419 | HGLibA_19398 | 12 | -1.026149375 | GMEB1 | glucocorticoid modulatory element binding protein 1 [Source:HGNC Symbol;Acc:HGNC:4370] |
| ENSG00000162461 | HGLibA_44715 | 12 | -1.026149375 | SLC25A34 | solute carrier family 25 member 34 [Source:HGNC Symbol;Acc:HGNC:27653] |
| ENSG00000162510 | HGLibA_28427 | 12 | -1.026149375 | MATN1 | matrilin 1 [Source:HGNC Symbol;Acc:HGNC:6907] |
| ENSG00000162772 | HGLibA_03436 | 12 | -1.026149375 | ATF3 | activating transcription factor 3 [Source:HGNC Symbol;Acc:HGNC:785] |
| ENSG00000163002 | HGLibA_33048 | 12 | -1.026149375 | NUP35 | nucleoporin 35 [Source:HGNC Symbol;Acc:HGNC:29797] |
| ENSG00000166091 | HGLibA_10221 | 12 | -1.026149375 | CMTM5 | CKLF like MARVEL transmembrane domain containing 5 [Source:HGNC Symbol;Acc:HGNC:19176] |
| ENSG00000166848 | HGLibA_49015 | 12 | -1.026149375 | TERF2IP | TERF2 interacting protein [Source:HGNC Symbol;Acc:HGNC:19246] |
| ENSG00000167995 | HGLibA_04419 | 12 | -1.026149375 | BEST1 | bestrophin 1 [Source:HGNC Symbol;Acc:HGNC:12703] |
| ENSG00000167996 | HGLibA_18116 | 12 | -1.026149375 | FTH1 | ferritin heavy chain 1 [Source:HGNC Symbol;Acc:HGNC:3976] |
| ENSG00000168778 | HGLibA_48875 | 12 | -1.026149375 | TCTN2 | tectonic family member 2 [Source:HGNC Symbol;Acc:HGNC:25774] |
| ENSG00000168811 | HGLibA_22959 | 12 | -1.026149375 | IL12A | interleukin 12A [Source:HGNC Symbol;Acc:HGNC:5969] |
| ENSG00000171014 | HGLibA_33976 | 12 | -1.026149375 | OR4D5 | olfactory receptor family 4 subfamily D member 5 [Source:HGNC Symbol;Acc:HGNC:14852] |
| ENSG00000171103 | HGLibA_51702 | 12 | -1.026149375 | TRMT61B | tRNA methyltransferase 61B [Source:HGNC Symbol;Acc:HGNC:26070] |
| ENSG00000171236 | HGLibA_27167 | 12 | -1.026149375 | LRG1 | leucine rich alpha-2-glycoprotein 1 [Source:HGNC Symbol;Acc:HGNC:29480] |
| ENSG00000171914 | HGLibA_49637 | 12 | -1.026149375 | TLN2 | talin 2 [Source:HGNC Symbol;Acc:HGNC:15447] |
| ENSG00000172404 | HGLibA_13551 | 12 | -1.026149375 | DNAJB7 | DnaJ heat shock protein family (Hsp40) member B7 [Source:HGNC Symbol;Acc:HGNC:24986] |
| ENSG00000172489 | HGLibA_34361 | 12 | -1.026149375 | OR5T3 | olfactory receptor family 5 subfamily T member 3 [Source:HGNC Symbol;Acc:HGNC:15297] |
| ENSG00000172845 | HGLibA_46301 | 12 | -1.026149375 | SP3 | Sp3 transcription factor [Source:HGNC Symbol;Acc:HGNC:11208] |
| ENSG00000173193 | HGLibA_35223 | 12 | -1.026149375 | PARP14 | poly(ADP-ribose) polymerase family member 14 [Source:HGNC Symbol;Acc:HGNC:29232] |
| ENSG00000173250 | HGLibA_19934 | 12 | -1.026149375 | GPR151 | G protein-coupled receptor 151 [Source:HGNC Symbol;Acc:HGNC:23624] |
| ENSG00000173418 | HGLibA_30982 | 12 | -1.026149375 | NAA20 | NatB catalytic subunit [Source:HGNC Symbol;Acc:HGNC:15908]" |
| ENSG00000174226 | HGLibA_46087 | 12 | -1.026149375 | SNX31 | sorting nexin 31 [Source:HGNC Symbol;Acc:HGNC:28605] |
| ENSG00000174238 | HGLibA_36740 | 12 | -1.026149375 | PITPNA | phosphatidylinositol transfer protein alpha [Source:HGNC Symbol;Acc:HGNC:9001] |
| ENSG00000175344 | HGLibA_09584 | 12 | -1.026149375 | CHRNA7 | cholinergic receptor nicotinic alpha 7 subunit [Source:HGNC Symbol;Acc:HGNC:1960] |
| ENSG00000177694 | HGLibA_31016 | 12 | -1.026149375 | NAALADL2 | N-acetylated alpha-linked acidic dipeptidase like 2 [Source:HGNC Symbol;Acc:HGNC:23219] |
| ENSG00000178665 | HGLibA_56617 | 12 | -1.026149375 | ZNF713 | zinc finger protein 713 [Source:HGNC Symbol;Acc:HGNC:22043] |
| ENSG00000180425 | HGLibA_05177 | 12 | -1.026149375 | C11orf71 | chromosome 11 open reading frame 71 [Source:HGNC Symbol;Acc:HGNC:25937] |
| ENSG00000182872 | HGLibA_40472 | 12 | -1.026149375 | RBM10 | RNA binding motif protein 10 [Source:HGNC Symbol;Acc:HGNC:9896] |
| ENSG00000183624 | HGLibA_21672 | 12 | -1.026149375 | HMCES | ES cell specific [Source:HGNC Symbol;Acc:HGNC:24446]" |
| ENSG00000186265 | HGLibA_04955 | 12 | -1.026149375 | BTLA | B and T lymphocyte associated [Source:HGNC Symbol;Acc:HGNC:21087] |
| ENSG00000186297 | HGLibA_18368 | 12 | -1.026149375 | GABRA5 | gamma-aminobutyric acid type A receptor alpha5 subunit [Source:HGNC Symbol;Acc:HGNC:4079] |
| ENSG00000187187 | HGLibA_56201 | 12 | -1.026149375 | ZNF546 | zinc finger protein 546 [Source:HGNC Symbol;Acc:HGNC:28671] |
| ENSG00000187260 | HGLibA_54201 | 12 | -1.026149375 | WDR86 | WD repeat domain 86 [Source:HGNC Symbol;Acc:HGNC:28020] |
| ENSG00000189001 | HGLibA_42813 | 12 | -1.026149375 | SBSN | suprabasin [Source:HGNC Symbol;Acc:HGNC:24950] |
| ENSG00000189376 | HGLibA_06647 | 12 | -1.026149375 | C8orf76 | chromosome 8 open reading frame 76 [Source:HGNC Symbol;Acc:HGNC:25924] |
| ENSG00000196275 | HGLibA_20572 | 12 | -1.026149375 | GTF2IRD2 | GTF2I repeat domain containing 2 [Source:HGNC Symbol;Acc:HGNC:30775] |
| ENSG00000196337 | HGLibA_09311 | 12 | -1.026149375 | CGB7 | chorionic gonadotropin subunit beta 7 [Source:HGNC Symbol;Acc:HGNC:16451] |
| ENSG00000196411 | HGLibA_15321 | 12 | -1.026149375 | EPHB4 | EPH receptor B4 [Source:HGNC Symbol;Acc:HGNC:3395] |
| ENSG00000196565 | HGLibA_20929 | 12 | -1.026149375 | HBG2 | hemoglobin subunit gamma 2 [Source:HGNC Symbol;Acc:HGNC:4832] |
| ENSG00000197385 | HGLibA_56877 | 12 | -1.026149375 | ZNF860 | zinc finger protein 860 [Source:HGNC Symbol;Acc:HGNC:34513] |
| ENSG00000197956 | HGLibA_42564 | 12 | -1.026149375 | S100A6 | S100 calcium binding protein A6 [Source:HGNC Symbol;Acc:HGNC:10496] |
| ENSG00000198650 | HGLibA_48437 | 12 | -1.026149375 | TAT | tyrosine aminotransferase [Source:HGNC Symbol;Acc:HGNC:11573] |
| ENSG00000198839 | HGLibA_55718 | 12 | -1.026149375 | ZNF277 | zinc finger protein 277 [Source:HGNC Symbol;Acc:HGNC:13070] |
| ENSG00000204348 | HGLibA_14218 | 12 | -1.026149375 | DXO | decapping exoribonuclease [Source:HGNC Symbol;Acc:HGNC:2992] |
| ENSG00000213588 | HGLibA_54934 | 12 | -1.026149375 | ZBTB9 | zinc finger and BTB domain containing 9 [Source:HGNC Symbol;Acc:HGNC:28323] |
| ENSG00000215271 | HGLibA_21874 | 12 | -1.026149375 | HOMEZ | homeobox and leucine zipper encoding [Source:HGNC Symbol;Acc:HGNC:20164] |
| ENSG00000215397 | HGLibA_43062 | 12 | -1.026149375 | SCRT2 | scratch family transcriptional repressor 2 [Source:HGNC Symbol;Acc:HGNC:15952] |
| ENSG00000219200 | HGLibA_41436 | 12 | -1.026149375 | RNASEK | ribonuclease K [Source:HGNC Symbol;Acc:HGNC:33911] |
| ENSG00000228049 | HGLibA_37593 | 12 | -1.026149375 | POLR2J2 | RNA polymerase II subunit J2 [Source:HGNC Symbol;Acc:HGNC:23208] |
| ENSG00000228083 | HGLibA_22664 | 12 | -1.026149375 | IFNA14 | interferon alpha 14 [Source:HGNC Symbol;Acc:HGNC:5420] |
| ENSG00000243480 | HGLibA_01850 | 12 | -1.026149375 | AMY2A | alpha 2A (pancreatic) [Source:HGNC Symbol;Acc:HGNC:477]" |
| ENSG00000244165 | HGLibA_34873 | 12 | -1.026149375 | P2RY11 | purinergic receptor P2Y11 [Source:HGNC Symbol;Acc:HGNC:8540] |
| ENSG00000253457 | HGLibA_45740 | 12 | -1.026149375 | SMIM18 | small integral membrane protein 18 [Source:HGNC Symbol;Acc:HGNC:42973] |
| ENSG00000253767 | HGLibA_35543 | 12 | -1.026149375 | PCDHGA8 | 8 [Source:HGNC Symbol;Acc:HGNC:8706]" |
| ENSG00000254726 | HGLibA_29028 | 12 | -1.026149375 | MEX3A | mex-3 RNA binding family member A [Source:HGNC Symbol;Acc:HGNC:33482] |
| ENSG00000257520 | HGLibA_43763 | 12 | -1.026149375 | SFTA3 | surfactant associated 3 [Source:NCBI gene;Acc:253970] |
| ENSG00000258223 | HGLibA_38970 | 12 | -1.026149375 | PRSS58 | serine protease 58 [Source:HGNC Symbol;Acc:HGNC:39125] |
| ENSG00000261934 | HGLibA_35548 | 12 | -1.026149375 | PCDHGA9 | 9 [Source:HGNC Symbol;Acc:HGNC:8707]" |
| ENSG00000274611 | HGLibA_48529 | 12 | -1.026149375 | TBC1D3 | TBC1 domain family member 3 [Source:HGNC Symbol;Acc:HGNC:19031] |
| ENSG00000276547 | HGLibA_35563 | 12 | -1.026149375 | PCDHGB5 | 5 [Source:HGNC Symbol;Acc:HGNC:8712]" |
| ENSG00000279263 | HGLibA_33831 | 12 | -1.026149375 | OR2L8 | olfactory receptor family 2 subfamily L member 8 (gene/pseudogene) [Source:HGNC Symbol;Acc:HGNC:15014] |
| FOXD4L2 | HGLibA_17843 | 12 | -1.026149375 | FOXD4L2 | - |
| HIST1H2BG | HGLibA_21430 | 12 | -1.026149375 | HIST1H2BG | - |
| HRASLS5 | HGLibA_22081 | 12 | -1.026149375 | HRASLS5 | - |
| hsa-let-7f-1 | HGLibA_57126 | 12 | -1.026149375 | hsa-let-7f-1 | - |
| hsa-mir-103b-2 | HGLibA_57175 | 12 | -1.026149375 | hsa-mir-103b-2 | - |
| hsa-mir-10a | HGLibA_57196 | 12 | -1.026149375 | hsa-mir-10a | - |
| hsa-mir-1275 | HGLibA_57555 | 12 | -1.026149375 | hsa-mir-1275 | - |
| hsa-mir-1537 | HGLibA_57915 | 12 | -1.026149375 | hsa-mir-1537 | - |
| hsa-mir-30a | HGLibA_58470 | 12 | -1.026149375 | hsa-mir-30a | - |
| hsa-mir-3143 | HGLibA_58630 | 12 | -1.026149375 | hsa-mir-3143 | - |
| hsa-mir-3202-2 | HGLibA_58919 | 12 | -1.026149375 | hsa-mir-3202-2 | - |
| hsa-mir-3616 | HGLibA_59092 | 12 | -1.026149375 | hsa-mir-3616 | - |
| hsa-mir-3670-2 | HGLibA_59236 | 12 | -1.026149375 | hsa-mir-3670-2 | - |
| hsa-mir-3689d-1 | HGLibA_59326 | 12 | -1.026149375 | hsa-mir-3689d-1 | - |
| hsa-mir-373 | HGLibA_59384 | 12 | -1.026149375 | hsa-mir-373 | - |
| hsa-mir-3941 | HGLibA_59623 | 12 | -1.026149375 | hsa-mir-3941 | - |
| hsa-mir-4494 | HGLibA_60396 | 12 | -1.026149375 | hsa-mir-4494 | - |
| hsa-mir-4664 | HGLibA_60769 | 12 | -1.026149375 | hsa-mir-4664 | - |

| | | | | | |
|---|---|---|---|---|---|
| hsa-mir-5007 | HGLibA_61430 | 12 | -1.026149375 | hsa-mir-5007 | - |
| hsa-mir-513a-1 | HGLibA_61569 | 12 | -1.026149375 | hsa-mir-513a-1 | - |
| hsa-mir-548aq | HGLibA_61916 | 12 | -1.026149375 | hsa-mir-548aq | - |
| hsa-mir-548f-1 | HGLibA_61972 | 12 | -1.026149375 | hsa-mir-548f-1 | - |
| hsa-mir-548z | HGLibA_62091 | 12 | -1.026149375 | hsa-mir-548z | - |
| hsa-mir-5584 | HGLibA_62176 | 12 | -1.026149375 | hsa-mir-5584 | - |
| hsa-mir-5692b | HGLibA_62295 | 12 | -1.026149375 | hsa-mir-5692b | - |
| hsa-mir-606 | HGLibA_62518 | 12 | -1.026149375 | hsa-mir-606 | - |
| hsa-mir-621 | HGLibA_62724 | 12 | -1.026149375 | hsa-mir-621 | - |
| hsa-mir-6511b-1 | HGLibA_62915 | 12 | -1.026149375 | hsa-mir-6511b-1 | - |
| hsa-mir-6842 | HGLibA_63544 | 12 | -1.026149375 | hsa-mir-6842 | - |
| hsa-mir-99b | HGLibA_64380 | 12 | -1.026149375 | hsa-mir-99b | - |
| LOC100287177 | HGLibA_26820 | 12 | -1.026149375 | LOC100287177 | - |
| LOC100287399 | HGLibA_26823 | 12 | -1.026149375 | LOC100287399 | - |
| RPS17L | HGLibA_42089 | 12 | -1.026149375 | RPS17L | - |
| 14-Sep | HGLibA_43405 | 12 | -1.026149375 | 14-Sep | - |
| SMCR7 | HGLibA_45676 | 12 | -1.026149375 | SMCR7 | - |
| WDR34 | HGLibA_54069 | 12 | -1.026149375 | WDR34 | - |
| WDR67 | HGLibA_54156 | 12 | -1.026149375 | WDR67 | - |
| AQPEP | HGLibA_02613 | 11 | -1.035194718 | AQPEP | - |
| ATP5I | HGLibA_03666 | 11 | -1.035194718 | ATP5I | - |
| C14orf169 | HGLibA_05339 | 11 | -1.035194718 | C14orf169 | - |
| C1orf95 | HGLibA_05909 | 11 | -1.035194718 | C1orf95 | - |
| C3orf79 | HGLibA_06269 | 11 | -1.035194718 | C3orf79 | - |
| CSRP2BP | HGLibA_11438 | 11 | -1.035194718 | CSRP2BP | - |
| ENSG00000005108 | HGLibA_49407 | 11 | -1.035194718 | THSD7A | thrombospondin type 1 domain containing 7A [Source:HGNC Symbol;Acc:HGNC:22207] |
| ENSG00000006042 | HGLibA_50569 | 11 | -1.035194718 | TMEM98 | transmembrane protein 98 [Source:HGNC Symbol;Acc:HGNC:24529] |
| ENSG00000008256 | HGLibA_12242 | 11 | -1.035194718 | CYTH3 | cytohesin 3 [Source:HGNC Symbol;Acc:HGNC:9504] |
| ENSG00000008311 | HGLibA_00082 | 11 | -1.035194718 | AASS | aminoadipate-semialdehyde synthase [Source:HGNC Symbol;Acc:HGNC:17366] |
| ENSG00000009954 | HGLibA_04189 | 11 | -1.035194718 | BAZ1B | bromodomain adjacent to zinc finger domain 1B [Source:HGNC Symbol;Acc:HGNC:961] |
| ENSG00000023734 | HGLibA_47580 | 11 | -1.035194718 | STRAP | serine/threonine kinase receptor associated protein [Source:HGNC Symbol;Acc:HGNC:30796] |
| ENSG00000040341 | HGLibA_47407 | 11 | -1.035194718 | STAU2 | staufen double-stranded RNA binding protein 2 [Source:HGNC Symbol;Acc:HGNC:11371] |
| ENSG00000050628 | HGLibA_39320 | 11 | -1.035194718 | PTGER3 | prostaglandin E receptor 3 [Source:HGNC Symbol;Acc:HGNC:9595] |
| ENSG00000063601 | HGLibA_30410 | 11 | -1.035194718 | MTMR1 | myotubularin related protein 1 [Source:HGNC Symbol;Acc:HGNC:7449] |
| ENSG00000064655 | HGLibA_15819 | 11 | -1.035194718 | EYA2 | EYA transcriptional coactivator and phosphatase 2 [Source:HGNC Symbol;Acc:HGNC:3520] |
| ENSG00000067248 | HGLibA_13172 | 11 | -1.035194718 | DHX29 | DExH-box helicase 29 [Source:HGNC Symbol;Acc:HGNC:15815] |
| ENSG00000072041 | HGLibA_45303 | 11 | -1.035194718 | SLC6A15 | solute carrier family 6 member 15 [Source:HGNC Symbol;Acc:HGNC:13621] |
| ENSG00000073670 | HGLibA_00731 | 11 | -1.035194718 | ADAM11 | ADAM metallopeptidase domain 11 [Source:HGNC Symbol;Acc:HGNC:189] |
| ENSG00000078246 | HGLibA_52337 | 11 | -1.035194718 | TULP3 | tubby like protein 3 [Source:HGNC Symbol;Acc:HGNC:12425] |
| ENSG00000081277 | HGLibA_36853 | 11 | -1.035194718 | PKP1 | plakophilin 1 [Source:HGNC Symbol;Acc:HGNC:9023] |
| ENSG00000081386 | HGLibA_56124 | 11 | -1.035194718 | ZNF510 | zinc finger protein 510 [Source:HGNC Symbol;Acc:HGNC:29161] |
| ENSG00000084092 | HGLibA_32228 | 11 | -1.035194718 | NOA1 | nitric oxide associated 1 [Source:HGNC Symbol;Acc:HGNC:28473] |
| ENSG00000084234 | HGLibA_02442 | 11 | -1.035194718 | APLP2 | amyloid beta precursor like protein 2 [Source:HGNC Symbol;Acc:HGNC:598] |
| ENSG00000085760 | HGLibA_30398 | 11 | -1.035194718 | MTIF2 | mitochondrial translational initiation factor 2 [Source:HGNC Symbol;Acc:HGNC:7441] |
| ENSG00000092330 | HGLibA_49554 | 11 | -1.035194718 | TINF2 | TERF1 interacting nuclear factor 2 [Source:HGNC Symbol;Acc:HGNC:11824] |
| ENSG00000099889 | HGLibA_03177 | 11 | -1.035194718 | ARVCF | delta catenin family member [Source:HGNC Symbol;Acc:HGNC:728]" |
| ENSG00000100341 | HGLibA_37416 | 11 | -1.035194718 | PNPLA5 | patatin like phospholipase domain containing 5 [Source:HGNC Symbol;Acc:HGNC:24888] |
| ENSG00000101074 | HGLibA_39780 | 11 | -1.035194718 | R3HDML | R3H domain containing like [Source:HGNC Symbol;Acc:HGNC:16249] |
| ENSG00000102309 | HGLibA_36687 | 11 | -1.035194718 | PIN4 | NIMA-interacting 4 [Source:HGNC Symbol;Acc:HGNC:8992]" |
| ENSG00000104332 | HGLibA_43737 | 11 | -1.035194718 | SFRP1 | secreted frizzled related protein 1 [Source:HGNC Symbol;Acc:HGNC:10776] |
| ENSG00000104765 | HGLibA_04660 | 11 | -1.035194718 | BNIP3L | BCL2 interacting protein 3 like [Source:HGNC Symbol;Acc:HGNC:1085] |
| ENSG00000105486 | HGLibA_26479 | 11 | -1.035194718 | LIG1 | DNA ligase 1 [Source:HGNC Symbol;Acc:HGNC:6598] |
| ENSG00000106789 | HGLibA_10782 | 11 | -1.035194718 | CORO2A | coronin 2A [Source:HGNC Symbol;Acc:HGNC:2255] |
| ENSG00000107223 | HGLibA_14462 | 11 | -1.035194718 | EDF1 | endothelial differentiation related factor 1 [Source:HGNC Symbol;Acc:HGNC:3164] |
| ENSG00000107554 | HGLibA_13695 | 11 | -1.035194718 | DNMBP | dynamin binding protein [Source:HGNC Symbol;Acc:HGNC:30373] |
| ENSG00000108518 | HGLibA_36175 | 11 | -1.035194718 | PFN1 | profilin 1 [Source:HGNC Symbol;Acc:HGNC:8881] |
| ENSG00000109133 | HGLibA_50368 | 11 | -1.035194718 | TMEM33 | transmembrane protein 33 [Source:HGNC Symbol;Acc:HGNC:25541] |
| ENSG00000109927 | HGLibA_48953 | 11 | -1.035194718 | TECTA | tectorin alpha [Source:HGNC Symbol;Acc:HGNC:11720] |
| ENSG00000112218 | HGLibA_20067 | 11 | -1.035194718 | GPR63 | G protein-coupled receptor 63 [Source:HGNC Symbol;Acc:HGNC:13302] |
| ENSG00000113361 | HGLibA_08687 | 11 | -1.035194718 | CDH6 | cadherin 6 [Source:HGNC Symbol;Acc:HGNC:1765] |
| ENSG00000115648 | HGLibA_29431 | 11 | -1.035194718 | MLPH | melanophilin [Source:HGNC Symbol;Acc:HGNC:29643] |
| ENSG00000116209 | HGLibA_50460 | 11 | -1.035194718 | TMEM59 | transmembrane protein 59 [Source:HGNC Symbol;Acc:HGNC:1239] |
| ENSG00000116497 | HGLibA_42588 | 11 | -1.035194718 | S100PBP | S100P binding protein [Source:HGNC Symbol;Acc:HGNC:25768] |
| ENSG00000119227 | HGLibA_36606 | 11 | -1.035194718 | PIGZ | phosphatidylinositol glycan anchor biosynthesis class Z [Source:HGNC Symbol;Acc:HGNC:30596] |
| ENSG00000119915 | HGLibA_14962 | 11 | -1.035194718 | ELOVL3 | ELOVL fatty acid elongase 3 [Source:HGNC Symbol;Acc:HGNC:18047] |
| ENSG00000121377 | HGLibA_48426 | 11 | -1.035194718 | TAS2R7 | taste 2 receptor member 7 [Source:HGNC Symbol;Acc:HGNC:14913] |
| ENSG00000122335 | HGLibA_43463 | 11 | -1.035194718 | SERAC1 | serine active site containing 1 [Source:HGNC Symbol;Acc:HGNC:21061] |
| ENSG00000124120 | HGLibA_52226 | 11 | -1.035194718 | TTPAL | alpha tocopherol transfer protein like [Source:HGNC Symbol;Acc:HGNC:16114] |
| ENSG00000124839 | HGLibA_39821 | 11 | -1.035194718 | RAB17 | member RAS oncogene family [Source:HGNC Symbol;Acc:HGNC:16523]" |
| ENSG00000126947 | HGLibA_03038 | 11 | -1.035194718 | ARMCX1 | armadillo repeat containing X-linked 1 [Source:HGNC Symbol;Acc:HGNC:18073] |
| ENSG00000129007 | HGLibA_07033 | 11 | -1.035194718 | CALML4 | calmodulin like 4 [Source:HGNC Symbol;Acc:HGNC:18445] |
| ENSG00000131914 | HGLibA_26557 | 11 | -1.035194718 | LIN28A | lin-28 homolog A [Source:HGNC Symbol;Acc:HGNC:15986] |
| ENSG00000132321 | HGLibA_23486 | 11 | -1.035194718 | IQCA1 | IQ motif containing with AAA domain 1 [Source:HGNC Symbol;Acc:HGNC:26195] |
| ENSG00000134539 | HGLibA_25300 | 11 | -1.035194718 | KLRD1 | killer cell lectin like receptor D1 [Source:HGNC Symbol;Acc:HGNC:6378] |
| ENSG00000135454 | HGLibA_04010 | 11 | -1.035194718 | B4GALNT1 | 4-N-acetyl-galactosaminyltransferase 1 [Source:HGNC Symbol;Acc:HGNC:4117]" |
| ENSG00000135945 | HGLibA_40885 | 11 | -1.035194718 | REV1 | DNA directed polymerase [Source:HGNC Symbol;Acc:HGNC:14060]" |
| ENSG00000136527 | HGLibA_51225 | 11 | -1.035194718 | TRA2B | transformer 2 beta homolog [Source:HGNC Symbol;Acc:HGNC:10781] |
| ENSG00000137101 | HGLibA_08441 | 11 | -1.035194718 | CD72 | CD72 molecule [Source:HGNC Symbol;Acc:HGNC:1696] |
| ENSG00000137414 | HGLibA_16789 | 11 | -1.035194718 | FAM8A1 | family with sequence similarity 8 member A1 [Source:HGNC Symbol;Acc:HGNC:16372] |
| ENSG00000140465 | HGLibA_12066 | 11 | -1.035194718 | CYP1A1 | cytochrome P450 family 1 subfamily A member 1 [Source:HGNC Symbol;Acc:HGNC:2595] |
| ENSG00000142025 | HGLibA_13442 | 11 | -1.035194718 | DMRTC2 | DMRT like family C2 [Source:HGNC Symbol;Acc:HGNC:13911] |
| ENSG00000142156 | HGLibA_10597 | 11 | -1.035194718 | COL6A1 | collagen type VI alpha 1 chain [Source:HGNC Symbol;Acc:HGNC:2211] |
| ENSG00000143954 | HGLibA_40804 | 11 | -1.035194718 | REG3G | regenerating family member 3 gamma [Source:HGNC Symbol;Acc:HGNC:29595] |
| ENSG00000144063 | HGLibA_28028 | 11 | -1.035194718 | MALL | T cell differentiation protein like [Source:HGNC Symbol;Acc:HGNC:6818]" |
| ENSG00000148950 | HGLibA_23230 | 11 | -1.035194718 | IMMP1L | inner mitochondrial membrane peptidase subunit 1 [Source:HGNC Symbol;Acc:HGNC:26317] |
| ENSG00000149488 | HGLibA_49750 | 11 | -1.035194718 | TMC2 | transmembrane channel like 2 [Source:HGNC Symbol;Acc:HGNC:16527] |
| ENSG00000150337 | HGLibA_17182 | 11 | -1.035194718 | FCGR1A | Fc fragment of IgG receptor Ia [Source:HGNC Symbol;Acc:HGNC:3613] |
| ENSG00000150991 | HGLibA_52566 | 11 | -1.035194718 | UBC | ubiquitin C [Source:HGNC Symbol;Acc:HGNC:12468] |
| ENSG00000153495 | HGLibA_49092 | 11 | -1.035194718 | TEX29 | testis expressed 29 [Source:HGNC Symbol;Acc:HGNC:20370] |
| ENSG00000154328 | HGLibA_31633 | 11 | -1.035194718 | NEIL2 | nei like DNA glycosylase 2 [Source:HGNC Symbol;Acc:HGNC:18956] |
| ENSG00000155592 | HGLibA_55347 | 11 | -1.035194718 | ZKSCAN2 | zinc finger with KRAB and SCAN domains 2 [Source:HGNC Symbol;Acc:HGNC:25677] |
| ENSG00000156261 | HGLibA_08216 | 11 | -1.035194718 | CCT8 | chaperonin containing TCP1 subunit 8 [Source:HGNC Symbol;Acc:HGNC:1623] |
| ENSG00000156414 | HGLibA_48918 | 11 | -1.035194718 | TDRD9 | tudor domain containing 9 [Source:HGNC Symbol;Acc:HGNC:20122] |
| ENSG00000159259 | HGLibA_09350 | 11 | -1.035194718 | CHAF1B | chromatin assembly factor 1 subunit B [Source:HGNC Symbol;Acc:HGNC:1911] |
| ENSG00000159261 | HGLibA_09859 | 11 | -1.035194718 | CLDN14 | claudin 14 [Source:HGNC Symbol;Acc:HGNC:2035] |
| ENSG00000159387 | HGLibA_23644 | 11 | -1.035194718 | IRX6 | iroquois homeobox 6 [Source:HGNC Symbol;Acc:HGNC:14675] |
| ENSG00000162337 | HGLibA_27223 | 11 | -1.035194718 | LRP5 | LDL receptor related protein 5 [Source:HGNC Symbol;Acc:HGNC:6697] |
| ENSG00000162692 | HGLibA_53566 | 11 | -1.035194718 | VCAM1 | vascular cell adhesion molecule 1 [Source:HGNC Symbol;Acc:HGNC:12663] |
| ENSG00000163285 | HGLibA_18389 | 11 | -1.035194718 | GABRG1 | gamma-aminobutyric acid type A receptor gamma1 subunit [Source:HGNC Symbol;Acc:HGNC:4086] |
| ENSG00000163468 | HGLibA_08199 | 11 | -1.035194718 | CCT3 | chaperonin containing TCP1 subunit 3 [Source:HGNC Symbol;Acc:HGNC:1616] |
| ENSG00000164008 | HGLibA_05860 | 11 | -1.035194718 | C1orf50 | chromosome 1 open reading frame 50 [Source:HGNC Symbol;Acc:HGNC:28795] |
| ENSG00000165379 | HGLibA_27164 | 11 | -1.035194718 | LRFN5 | leucine rich repeat and fibronectin type III domain containing 5 [Source:HGNC Symbol;Acc:HGNC:20360] |
| ENSG00000165416 | HGLibA_47722 | 11 | -1.035194718 | SUGT1 | MIS12 kinetochore complex assembly cochaperone [Source:HGNC Symbol;Acc:HGNC:16987]" |
| ENSG00000166197 | HGLibA_32282 | 11 | -1.035194718 | NOLC1 | nucleolar and coiled-body phosphoprotein 1 [Source:HGNC Symbol;Acc:HGNC:15608] |
| ENSG00000166455 | HGLibA_05450 | 11 | -1.035194718 | C16orf46 | chromosome 16 open reading frame 46 [Source:HGNC Symbol;Acc:HGNC:26525] |
| ENSG00000166578 | HGLibA_23495 | 11 | -1.035194718 | IQCD | IQ motif containing D [Source:HGNC Symbol;Acc:HGNC:25168] |
| ENSG00000166685 | HGLibA_10469 | 11 | -1.035194718 | COG1 | component of oligomeric golgi complex 1 [Source:HGNC Symbol;Acc:HGNC:6545] |
| ENSG00000167100 | HGLibA_42673 | 11 | -1.035194718 | SAMD14 | sterile alpha motif domain containing 14 [Source:HGNC Symbol;Acc:HGNC:27312] |
| ENSG00000169507 | HGLibA_45061 | 11 | -1.035194718 | SLC38A11 | solute carrier family 38 member 11 [Source:HGNC Symbol;Acc:HGNC:26836] |
| ENSG00000169760 | HGLibA_32068 | 11 | -1.035194718 | NLGN1 | neuroligin 1 [Source:HGNC Symbol;Acc:HGNC:14291] |
| ENSG00000170017 | HGLibA_01531 | 11 | -1.035194718 | ALCAM | activated leukocyte cell adhesion molecule [Source:HGNC Symbol;Acc:HGNC:400] |
| ENSG00000170113 | HGLibA_31950 | 11 | -1.035194718 | NIPA1 | NIPA magnesium transporter 1 [Source:HGNC Symbol;Acc:HGNC:17043] |
| ENSG00000171863 | HGLibA_42189 | 11 | -1.035194718 | RPS7 | ribosomal protein S7 [Source:HGNC Symbol;Acc:HGNC:10440] |
| ENSG00000172461 | HGLibA_18209 | 11 | -1.035194718 | FUT9 | fucosyltransferase 9 [Source:HGNC Symbol;Acc:HGNC:4020] |
| ENSG00000172769 | HGLibA_34267 | 11 | -1.035194718 | OR5B3 | olfactory receptor family 5 subfamily B member 3 [Source:HGNC Symbol;Acc:HGNC:8324] |
| ENSG00000173171 | HGLibA_30510 | 11 | -1.035194718 | MTX1 | metaxin 1 [Source:HGNC Symbol;Acc:HGNC:7504] |
| ENSG00000173452 | HGLibA_50158 | 11 | -1.035194718 | TMEM196 | transmembrane protein 196 [Source:HGNC Symbol;Acc:HGNC:22431] |
| ENSG00000173621 | HGLibA_27161 | 11 | -1.035194718 | LRFN4 | leucine rich repeat and fibronectin type III domain containing 4 [Source:HGNC Symbol;Acc:HGNC:28456] |
| ENSG00000174137 | HGLibA_16636 | 11 | -1.035194718 | FAM53A | family with sequence similarity 53 member A [Source:HGNC Symbol;Acc:HGNC:31860] |
| ENSG00000174996 | HGLibA_25029 | 11 | -1.035194718 | KLC2 | kinesin light chain 2 [Source:HGNC Symbol;Acc:HGNC:20716] |
| ENSG00000178397 | HGLibA_16513 | 11 | -1.035194718 | FAM220A | family with sequence similarity 220 member A [Source:HGNC Symbol;Acc:HGNC:22422] |
| ENSG00000178935 | HGLibA_56219 | 11 | -1.035194718 | ZNF552 | zinc finger protein 552 [Source:HGNC Symbol;Acc:HGNC:26135] |
| ENSG00000179142 | HGLibA_12059 | 11 | -1.035194718 | CYP11B2 | cytochrome P450 family 11 subfamily B member 2 [Source:HGNC Symbol;Acc:HGNC:2592] |
| ENSG00000180228 | HGLibA_38583 | 11 | -1.035194718 | PRKRA | protein activator of interferon induced protein kinase EIF2AK2 [Source:HGNC Symbol;Acc:HGNC:9438] |
| ENSG00000180611 | HGLibA_28460 | 11 | -1.035194718 | MB21D2 | Mab-21 domain containing 2 [Source:HGNC Symbol;Acc:HGNC:30438] |
| ENSG00000181007 | HGLibA_55236 | 11 | -1.035194718 | ZFP82 | ZFP82 zinc finger protein [Source:HGNC Symbol;Acc:HGNC:28682] |
| ENSG00000181982 | HGLibA_07637 | 11 | -1.035194718 | CCDC149 | coiled-coil domain containing 149 [Source:HGNC Symbol;Acc:HGNC:25405] |
| ENSG00000183207 | HGLibA_42476 | 11 | -1.035194718 | RUVBL2 | RuvB like AAA ATPase 2 [Source:HGNC Symbol;Acc:HGNC:10475] |
| ENSG00000183632 | HGLibA_51078 | 11 | -1.035194718 | TP53TG3 | TP53 target 3 [Source:HGNC Symbol;Acc:HGNC:30759] |
| ENSG00000184148 | HGLibA_46829 | 11 | -1.035194718 | SPRR4 | small proline rich protein 4 [Source:HGNC Symbol;Acc:HGNC:23173] |
| ENSG00000184967 | HGLibA_32240 | 11 | -1.035194718 | NOC4L | nucleolar complex associated 4 homolog [Source:HGNC Symbol;Acc:HGNC:28461] |
| ENSG00000185127 | HGLibA_06439 | 11 | -1.035194718 | C6orf120 | chromosome 6 open reading frame 120 [Source:HGNC Symbol;Acc:HGNC:21247] |
| ENSG00000185158 | HGLibA_27336 | 11 | -1.035194718 | LRRC37B | leucine rich repeat containing 37B [Source:HGNC Symbol;Acc:HGNC:29070] |
| ENSG00000185640 | HGLibA_25550 | 11 | -1.035194718 | KRT79 | keratin 79 [Source:HGNC Symbol;Acc:HGNC:28930] |
| ENSG00000185737 | HGLibA_32683 | 11 | -1.035194718 | NRG3 | neuregulin 3 [Source:HGNC Symbol;Acc:HGNC:7999] |

| | | | | | |
|---|---|---|---|---|---|
| ENSG00000188316 | HGLibA_15149 | 11 | -1.035194718 | ENO4 | enolase 4 [Source:HGNC Symbol;Acc:HGNC:31670] |
| ENSG00000188770 | HGLibA_33456 | 11 | -1.035194718 | OPTC | opticin [Source:HGNC Symbol;Acc:HGNC:8158] |
| ENSG00000189184 | HGLibA_35407 | 11 | -1.035194718 | PCDH18 | protocadherin 18 [Source:HGNC Symbol;Acc:HGNC:14268] |
| ENSG00000196159 | HGLibA_16920 | 11 | -1.035194718 | FAT4 | FAT atypical cadherin 4 [Source:HGNC Symbol;Acc:HGNC:23109] |
| ENSG00000196767 | HGLibA_37773 | 11 | -1.035194718 | POU3F4 | POU class 3 homeobox 4 [Source:HGNC Symbol;Acc:HGNC:9217] |
| ENSG00000197050 | HGLibA_55958 | 11 | -1.035194718 | ZNF420 | zinc finger protein 420 [Source:HGNC Symbol;Acc:HGNC:20649] |
| ENSG00000197406 | HGLibA_13244 | 11 | -1.035194718 | DIO3 | iodothyronine deiodinase 3 [Source:HGNC Symbol;Acc:HGNC:2885] |
| ENSG00000198574 | HGLibA_43887 | 11 | -1.035194718 | SH2D1B | SH2 domain containing 1B [Source:HGNC Symbol;Acc:HGNC:30416] |
| ENSG00000203756 | HGLibA_50298 | 11 | -1.035194718 | TMEM244 | transmembrane protein 244 [Source:HGNC Symbol;Acc:HGNC:21571] |
| ENSG00000203989 | HGLibA_41220 | 11 | -1.035194718 | RHOXF2B | Rhox homeobox family member 2B [Source:HGNC Symbol;Acc:HGNC:33519] |
| ENSG00000204070 | HGLibA_48043 | 11 | -1.035194718 | SYS1 | golgi trafficking protein [Source:HGNC Symbol;Acc:HGNC:16162]" |
| ENSG00000204209 | HGLibA_12348 | 11 | -1.035194718 | DAXX | death domain associated protein [Source:HGNC Symbol;Acc:HGNC:2681] |
| ENSG00000204866 | HGLibA_22843 | 11 | -1.035194718 | IGFL2 | IGF like family member 2 [Source:HGNC Symbol;Acc:HGNC:32929] |
| ENSG00000205279 | HGLibA_11760 | 11 | -1.035194718 | CTXN3 | cortexin 3 [Source:HGNC Symbol;Acc:HGNC:31110] |
| ENSG00000205744 | HGLibA_12935 | 11 | -1.035194718 | DENND1C | DENN domain containing 1C [Source:HGNC Symbol;Acc:HGNC:26225] |
| ENSG00000206579 | HGLibA_54522 | 11 | -1.035194718 | XKR4 | XK related 4 [Source:HGNC Symbol;Acc:HGNC:29394] |
| ENSG00000213417 | HGLibA_25695 | 11 | -1.035194718 | KRTAP2-4 | keratin associated protein 2-4 [Source:HGNC Symbol;Acc:HGNC:18891] |
| ENSG00000214042 | HGLibA_22688 | 11 | -1.035194718 | IFNA7 | interferon alpha 7 [Source:HGNC Symbol;Acc:HGNC:5428] |
| ENSG00000214102 | HGLibA_54241 | 11 | -1.035194718 | WEE2 | WEE1 homolog 2 [Source:HGNC Symbol;Acc:HGNC:19684] |
| ENSG00000217555 | HGLibA_09769 | 11 | -1.035194718 | CKLF | chemokine like factor [Source:HGNC Symbol;Acc:HGNC:13253] |
| ENSG00000230522 | HGLibA_28476 | 11 | -1.035194718 | MBD3L2 | methyl-CpG binding domain protein 3 like 2 [Source:HGNC Symbol;Acc:HGNC:18532] |
| ENSG00000233493 | HGLibA_50278 | 11 | -1.035194718 | TMEM238 | transmembrane protein 238 [Source:HGNC Symbol;Acc:HGNC:40042] |
| ENSG00000237172 | HGLibA_04004 | 11 | -1.035194718 | B3GNT9 | 3-N-acetylglucosaminyltransferase 9 [Source:HGNC Symbol;Acc:HGNC:28714]" |
| ENSG00000240583 | HGLibA_02579 | 11 | -1.035194718 | AQP1 | aquaporin 1 (Colton blood group) [Source:HGNC Symbol;Acc:HGNC:633] |
| ENSG00000240694 | HGLibA_37370 | 11 | -1.035194718 | PNMA2 | PNMA family member 2 [Source:HGNC Symbol;Acc:HGNC:9159] |
| ENSG00000276045 | HGLibA_34590 | 11 | -1.035194718 | ORAI1 | ORAI calcium release-activated calcium modulator 1 [Source:HGNC Symbol;Acc:HGNC:25896] |
| FAM5B | HGLibA_16651 | 11 | -1.035194718 | FAM5B | - |
| FTSJD2 | HGLibA_18146 | 11 | -1.035194718 | FTSJD2 | - |
| hsa-mir-1245a | HGLibA_57374 | 11 | -1.035194718 | hsa-mir-1245a | - |
| hsa-mir-1264 | HGLibA_57478 | 11 | -1.035194718 | hsa-mir-1264 | - |
| hsa-mir-1273c | HGLibA_57534 | 11 | -1.035194718 | hsa-mir-1273c | - |
| hsa-mir-1293 | HGLibA_57650 | 11 | -1.035194718 | hsa-mir-1293 | - |
| hsa-mir-2113 | HGLibA_58223 | 11 | -1.035194718 | hsa-mir-2113 | - |
| hsa-mir-3129 | HGLibA_58568 | 11 | -1.035194718 | hsa-mir-3129 | - |
| hsa-mir-3202-1 | HGLibA_58915 | 11 | -1.035194718 | hsa-mir-3202-1 | - |
| hsa-mir-324 | HGLibA_58965 | 11 | -1.035194718 | hsa-mir-324 | - |
| hsa-mir-378a | HGLibA_59425 | 11 | -1.035194718 | hsa-mir-378a | - |
| hsa-mir-3939 | HGLibA_59617 | 11 | -1.035194718 | hsa-mir-3939 | - |
| hsa-mir-4458 | HGLibA_60240 | 11 | -1.035194718 | hsa-mir-4458 | - |
| hsa-mir-4509-1 | HGLibA_60462 | 11 | -1.035194718 | hsa-mir-4509-1 | - |
| hsa-mir-4514 | HGLibA_60499 | 11 | -1.035194718 | hsa-mir-4514 | - |
| hsa-mir-4651 | HGLibA_60711 | 11 | -1.035194718 | hsa-mir-4651 | - |
| hsa-mir-4774 | HGLibA_61200 | 11 | -1.035194718 | hsa-mir-4774 | - |
| hsa-mir-486 | HGLibA_61336 | 11 | -1.035194718 | hsa-mir-486 | - |
| hsa-mir-541 | HGLibA_61814 | 11 | -1.035194718 | hsa-mir-541 | - |
| hsa-mir-548a-2 | HGLibA_61839 | 11 | -1.035194718 | hsa-mir-548a-2 | - |
| hsa-mir-577 | HGLibA_62401 | 11 | -1.035194718 | hsa-mir-577 | - |
| hsa-mir-6719 | HGLibA_63038 | 11 | -1.035194718 | hsa-mir-6719 | - |
| hsa-mir-6768 | HGLibA_63235 | 11 | -1.035194718 | hsa-mir-6768 | - |
| hsa-mir-6825 | HGLibA_63476 | 11 | -1.035194718 | hsa-mir-6825 | - |
| hsa-mir-7844 | HGLibA_63965 | 11 | -1.035194718 | hsa-mir-7844 | - |
| hsa-mir-8071-1 | HGLibA_64121 | 11 | -1.035194718 | hsa-mir-8071-1 | - |
| KIAA1045 | HGLibA_24689 | 11 | -1.035194718 | KIAA1045 | - |
| LOC728819 | HGLibA_26994 | 11 | -1.035194718 | LOC728819 | - |
| MNF1 | HGLibA_29571 | 11 | -1.035194718 | MNF1 | - |
| PVRL1 | HGLibA_39621 | 11 | -1.035194718 | PVRL1 | - |
| SLC25A6_Y | HGLibA_44778 | 11 | -1.035194718 | SLC25A6_Y | - |
| WBSCR17 | HGLibA_53993 | 11 | -1.035194718 | WBSCR17 | - |
| ZHX1-C8ORF76 | HGLibA_55312 | 11 | -1.035194718 | ZHX1-C8ORF76 | - |
| ANKRD20A4 | HGLibA_02036 | 10 | -1.044240061 | ANKRD20A4 | - |
| C11orf93 | HGLibA_05214 | 10 | -1.044240061 | C11orf93 | - |
| C16orf70 | HGLibA_05472 | 10 | -1.044240061 | C16orf70 | - |
| ENSG00000009694 | HGLibA_48994 | 10 | -1.044240061 | TENM1 | teneurin transmembrane protein 1 [Source:HGNC Symbol;Acc:HGNC:8117] |
| ENSG00000023839 | HGLibA_00169 | 10 | -1.044240061 | ABCC2 | ATP binding cassette subfamily C member 2 [Source:HGNC Symbol;Acc:HGNC:53] |
| ENSG00000025800 | HGLibA_25370 | 10 | -1.044240061 | KPNA6 | karyopherin subunit alpha 6 [Source:HGNC Symbol;Acc:HGNC:6399] |
| ENSG00000051108 | HGLibA_21170 | 10 | -1.044240061 | HERPUD1 | homocysteine inducible ER protein with ubiquitin like domain 1 [Source:HGNC Symbol;Acc:HGNC:13744] |
| ENSG00000057149 | HGLibA_43551 | 10 | -1.044240061 | SERPINB3 | serpin family B member 3 [Source:HGNC Symbol;Acc:HGNC:10569] |
| ENSG00000064201 | HGLibA_51932 | 10 | -1.044240061 | TSPAN32 | tetraspanin 32 [Source:HGNC Symbol;Acc:HGNC:13410] |
| ENSG00000069869 | HGLibA_31601 | 10 | -1.044240061 | NEDD4 | E3 ubiquitin protein ligase [Source:HGNC Symbol;Acc:HGNC:7727]" |
| ENSG00000071054 | HGLibA_28220 | 10 | -1.044240061 | MAP4K4 | mitogen-activated protein kinase kinase kinase kinase 4 [Source:HGNC Symbol;Acc:HGNC:6866] |
| ENSG00000075790 | HGLibA_04239 | 10 | -1.044240061 | BCAP29 | B cell receptor associated protein 29 [Source:HGNC Symbol;Acc:HGNC:24131] |
| ENSG00000078814 | HGLibA_30737 | 10 | -1.044240061 | MYH7B | myosin heavy chain 7B [Source:HGNC Symbol;Acc:HGNC:15906] |
| ENSG00000079432 | HGLibA_09693 | 10 | -1.044240061 | CIC | capicua transcriptional repressor [Source:HGNC Symbol;Acc:HGNC:14214] |
| ENSG00000087494 | HGLibA_39377 | 10 | -1.044240061 | PTHLH | parathyroid hormone like hormone [Source:HGNC Symbol;Acc:HGNC:9607] |
| ENSG00000089012 | HGLibA_44213 | 10 | -1.044240061 | SIRPG | signal regulatory protein gamma [Source:HGNC Symbol;Acc:HGNC:15757] |
| ENSG00000089041 | HGLibA_34864 | 10 | -1.044240061 | P2RX7 | purinergic receptor P2X 7 [Source:HGNC Symbol;Acc:HGNC:8537] |
| ENSG00000089094 | HGLibA_24497 | 10 | -1.044240061 | KDM2B | lysine demethylase 2B [Source:HGNC Symbol;Acc:HGNC:13610] |
| ENSG00000092330 | HGLibA_49553 | 10 | -1.044240061 | TINF2 | TERF1 interacting nuclear factor 2 [Source:HGNC Symbol;Acc:HGNC:11824] |
| ENSG00000093183 | HGLibA_43217 | 10 | -1.044240061 | SEC22C | vesicle trafficking protein [Source:HGNC Symbol;Acc:HGNC:16828]" |
| ENSG00000099331 | HGLibA_30870 | 10 | -1.044240061 | MYO9B | myosin IXB [Source:HGNC Symbol;Acc:HGNC:7609] |
| ENSG00000100196 | HGLibA_24484 | 10 | -1.044240061 | KDELR3 | KDEL endoplasmic reticulum protein retention receptor 3 [Source:HGNC Symbol;Acc:HGNC:6306] |
| ENSG00000100453 | HGLibA_20720 | 10 | -1.044240061 | GZMB | granzyme B [Source:HGNC Symbol;Acc:HGNC:4709] |
| ENSG00000101400 | HGLibA_45997 | 10 | -1.044240061 | SNTA1 | syntrophin alpha 1 [Source:HGNC Symbol;Acc:HGNC:11167] |
| ENSG00000102837 | HGLibA_33352 | 10 | -1.044240061 | OLFM4 | olfactomedin 4 [Source:HGNC Symbol;Acc:HGNC:17190] |
| ENSG00000102879 | HGLibA_10773 | 10 | -1.044240061 | CORO1A | coronin 1A [Source:HGNC Symbol;Acc:HGNC:2252] |
| ENSG00000102882 | HGLibA_28272 | 10 | -1.044240061 | MAPK3 | mitogen-activated protein kinase 3 [Source:HGNC Symbol;Acc:HGNC:6877] |
| ENSG00000103978 | HGLibA_50532 | 10 | -1.044240061 | TMEM87A | transmembrane protein 87A [Source:HGNC Symbol;Acc:HGNC:24522] |
| ENSG00000104904 | HGLibA_33213 | 10 | -1.044240061 | OAZ1 | ornithine decarboxylase antizyme 1 [Source:HGNC Symbol;Acc:HGNC:8095] |
| ENSG00000105261 | HGLibA_34806 | 10 | -1.044240061 | OVOL3 | ovo like zinc finger 3 [Source:HGNC Symbol;Acc:HGNC:14186] |
| ENSG00000105364 | HGLibA_29974 | 10 | -1.044240061 | MRPL4 | mitochondrial ribosomal protein L4 [Source:HGNC Symbol;Acc:HGNC:14276] |
| ENSG00000105610 | HGLibA_25038 | 10 | -1.044240061 | KLF1 | Kruppel like factor 1 [Source:HGNC Symbol;Acc:HGNC:6345] |
| ENSG00000105996 | HGLibA_21906 | 10 | -1.044240061 | HOXA2 | homeobox A2 [Source:HGNC Symbol;Acc:HGNC:5103] |
| ENSG00000106404 | HGLibA_09863 | 10 | -1.044240061 | CLDN15 | claudin 15 [Source:HGNC Symbol;Acc:HGNC:2036] |
| ENSG00000107951 | HGLibA_30457 | 10 | -1.044240061 | MTPAP | mitochondrial poly(A) polymerase [Source:HGNC Symbol;Acc:HGNC:25532] |
| ENSG00000110435 | HGLibA_35871 | 10 | -1.044240061 | PDHX | pyruvate dehydrogenase complex component X [Source:HGNC Symbol;Acc:HGNC:21350] |
| ENSG00000110881 | HGLibA_03299 | 10 | -1.044240061 | ASIC1 | acid sensing ion channel subunit 1 [Source:HGNC Symbol;Acc:HGNC:100] |
| ENSG00000111254 | HGLibA_01430 | 10 | -1.044240061 | AKAP3 | A-kinase anchoring protein 3 [Source:HGNC Symbol;Acc:HGNC:373] |
| ENSG00000111300 | HGLibA_30984 | 10 | -1.044240061 | NAA25 | NatB auxiliary subunit [Source:HGNC Symbol;Acc:HGNC:25783]" |
| ENSG00000111325 | HGLibA_33312 | 10 | -1.044240061 | OGFOD2 | 2-oxoglutarate and iron dependent oxygenase domain containing 2 [Source:HGNC Symbol;Acc:HGNC:25823] |
| ENSG00000111879 | HGLibA_16328 | 10 | -1.044240061 | FAM184A | family with sequence similarity 184 member A [Source:HGNC Symbol;Acc:HGNC:20991] |
| ENSG00000112799 | HGLibA_27685 | 10 | -1.044240061 | LY86 | lymphocyte antigen 86 [Source:HGNC Symbol;Acc:HGNC:16837] |
| ENSG00000113070 | HGLibA_20923 | 10 | -1.044240061 | HBEGF | heparin binding EGF like growth factor [Source:HGNC Symbol;Acc:HGNC:3059] |
| ENSG00000113209 | HGLibA_35501 | 10 | -1.044240061 | PCDHB5 | protocadherin beta 5 [Source:HGNC Symbol;Acc:HGNC:8690] |
| ENSG00000113441 | HGLibA_26742 | 10 | -1.044240061 | LNPEP | leucyl and cystinyl aminopeptidase [Source:HGNC Symbol;Acc:HGNC:6656] |
| ENSG00000114988 | HGLibA_26667 | 10 | -1.044240061 | LMAN2L | mannose binding 2 like [Source:HGNC Symbol;Acc:HGNC:19263]" |
| ENSG00000116096 | HGLibA_46786 | 10 | -1.044240061 | SPR | sepiapterin reductase [Source:HGNC Symbol;Acc:HGNC:11257] |
| ENSG00000116663 | HGLibA_17122 | 10 | -1.044240061 | FBXO6 | F-box protein 6 [Source:HGNC Symbol;Acc:HGNC:13585] |
| ENSG00000116701 | HGLibA_31298 | 10 | -1.044240061 | NCF2 | neutrophil cytosolic factor 2 [Source:HGNC Symbol;Acc:HGNC:7661] |
| ENSG00000117054 | HGLibA_00368 | 10 | -1.044240061 | ACADM | acyl-CoA dehydrogenase medium chain [Source:HGNC Symbol;Acc:HGNC:89] |
| ENSG00000118997 | HGLibA_13496 | 10 | -1.044240061 | DNAH7 | dynein axonemal heavy chain 7 [Source:HGNC Symbol;Acc:HGNC:18661] |
| ENSG00000119718 | HGLibA_14739 | 10 | -1.044240061 | EIF2B2 | eukaryotic translation initiation factor 2B subunit beta [Source:HGNC Symbol;Acc:HGNC:3258] |
| ENSG00000119787 | HGLibA_03517 | 10 | -1.044240061 | ATL2 | atlastin GTPase 2 [Source:HGNC Symbol;Acc:HGNC:24047] |
| ENSG00000120727 | HGLibA_35030 | 10 | -1.044240061 | PAIP2 | poly(A) binding protein interacting protein 2 [Source:HGNC Symbol;Acc:HGNC:17970] |
| ENSG00000121570 | HGLibA_13892 | 10 | -1.044240061 | DPPA4 | developmental pluripotency associated 4 [Source:HGNC Symbol;Acc:HGNC:19200] |
| ENSG00000123066 | HGLibA_28751 | 10 | -1.044240061 | MED13L | mediator complex subunit 13 like [Source:HGNC Symbol;Acc:HGNC:22962] |
| ENSG00000124787 | HGLibA_42040 | 10 | -1.044240061 | RPP40 | ribonuclease P/MRP subunit p40 [Source:HGNC Symbol;Acc:HGNC:20992] |
| ENSG00000125691 | HGLibA_41905 | 10 | -1.044240061 | RPL23 | ribosomal protein L23 [Source:HGNC Symbol;Acc:HGNC:10316] |
| ENSG00000126216 | HGLibA_52311 | 10 | -1.044240061 | TUBGCP3 | tubulin gamma complex associated protein 3 [Source:HGNC Symbol;Acc:HGNC:18598] |
| ENSG00000127990 | HGLibA_43806 | 10 | -1.044240061 | SGCE | sarcoglycan epsilon [Source:HGNC Symbol;Acc:HGNC:10808] |
| ENSG00000128276 | HGLibA_40935 | 10 | -1.044240061 | RFPL3 | ret finger protein like 3 [Source:HGNC Symbol;Acc:HGNC:9980] |
| ENSG00000128573 | HGLibA_17932 | 10 | -1.044240061 | FOXP2 | forkhead box P2 [Source:HGNC Symbol;Acc:HGNC:13875] |
| ENSG00000130538 | HGLibA_33568 | 10 | -1.044240061 | OR11H1 | olfactory receptor family 11 subfamily H member 1 [Source:HGNC Symbol;Acc:HGNC:15404] |
| ENSG00000131043 | HGLibA_00061 | 10 | -1.044240061 | AAR2 | AAR2 splicing factor homolog [Source:HGNC Symbol;Acc:HGNC:15886] |
| ENSG00000132254 | HGLibA_02679 | 10 | -1.044240061 | ARFIP2 | ADP ribosylation factor interacting protein 2 [Source:HGNC Symbol;Acc:HGNC:17160] |
| ENSG00000132300 | HGLibA_39264 | 10 | -1.044240061 | PTCD3 | pentatricopeptide repeat domain 3 [Source:HGNC Symbol;Acc:HGNC:24717] |
| ENSG00000132793 | HGLibA_27102 | 10 | -1.044240061 | LPIN3 | lipin 3 [Source:HGNC Symbol;Acc:HGNC:14451] |
| ENSG00000134072 | HGLibA_07057 | 10 | -1.044240061 | CAMK1 | calcium/calmodulin dependent protein kinase I [Source:HGNC Symbol;Acc:HGNC:1459] |
| ENSG00000134443 | HGLibA_20367 | 10 | -1.044240061 | GRP | gastrin releasing peptide [Source:HGNC Symbol;Acc:HGNC:4605] |
| ENSG00000134508 | HGLibA_06843 | 10 | -1.044240061 | CABLES1 | Cdk5 and Abl enzyme substrate 1 [Source:HGNC Symbol;Acc:HGNC:25097] |
| ENSG00000134532 | HGLibA_46268 | 10 | -1.044240061 | SOX5 | SRY-box 5 [Source:HGNC Symbol;Acc:HGNC:11201] |
| ENSG00000134871 | HGLibA_10569 | 10 | -1.044240061 | COL4A2 | collagen type IV alpha 2 chain [Source:HGNC Symbol;Acc:HGNC:2203] |
| ENSG00000135624 | HGLibA_08212 | 10 | -1.044240061 | CCT7 | chaperonin containing TCP1 subunit 7 [Source:HGNC Symbol;Acc:HGNC:1622] |
| ENSG00000135829 | HGLibA_13212 | 10 | -1.044240061 | DHX9 | DExH-box helicase 9 [Source:HGNC Symbol;Acc:HGNC:2750] |
| ENSG00000136444 | HGLibA_42297 | 10 | -1.044240061 | RSAD1 | radical S-adenosyl methionine domain containing 1 [Source:HGNC Symbol;Acc:HGNC:25634] |
| ENSG00000137098 | HGLibA_46368 | 10 | -1.044240061 | SPAG8 | sperm associated antigen 8 [Source:HGNC Symbol;Acc:HGNC:14105] |

| | | | | | |
|---|---|---|---|---|---|
| ENSG00000137960 | HGLibA_19120 | 10 | -1.044240061 | GIPC2 | GIPC PDZ domain containing family member 2 [Source:HGNC Symbol;Acc:HGNC:18177] |
| ENSG00000138413 | HGLibA_22540 | 10 | -1.044240061 | IDH1 | cytosolic [Source:HGNC Symbol;Acc:HGNC:5382]" |
| ENSG00000138768 | HGLibA_53188 | 10 | -1.044240061 | USO1 | USO1 vesicle transport factor [Source:HGNC Symbol;Acc:HGNC:30904] |
| ENSG00000141161 | HGLibA_53007 | 10 | -1.044240061 | UNC45B | unc-45 myosin chaperone B [Source:HGNC Symbol;Acc:HGNC:14304] |
| ENSG00000142408 | HGLibA_06949 | 10 | -1.044240061 | CACNG8 | calcium voltage-gated channel auxiliary subunit gamma 8 [Source:HGNC Symbol;Acc:HGNC:13628] |
| ENSG00000144468 | HGLibA_41123 | 10 | -1.044240061 | RHBDD1 | rhomboid domain containing 1 [Source:HGNC Symbol;Acc:HGNC:23081] |
| ENSG00000145332 | HGLibA_25224 | 10 | -1.044240061 | KLHL8 | kelch like family member 8 [Source:HGNC Symbol;Acc:HGNC:18644] |
| ENSG00000145476 | HGLibA_12200 | 10 | -1.044240061 | CYP4V2 | cytochrome P450 family 4 subfamily V member 2 [Source:HGNC Symbol;Acc:HGNC:23198] |
| ENSG00000145725 | HGLibA_37956 | 10 | -1.044240061 | PPIP5K2 | diphosphoinositol pentakisphosphate kinase 2 [Source:HGNC Symbol;Acc:HGNC:29035] |
| ENSG00000146143 | HGLibA_38486 | 10 | -1.044240061 | PRIM2 | DNA primase subunit 2 [Source:HGNC Symbol;Acc:HGNC:9370] |
| ENSG00000147127 | HGLibA_39943 | 10 | -1.044240061 | RAB41 | member RAS oncogene family [Source:HGNC Symbol;Acc:HGNC:18293]" |
| ENSG00000147650 | HGLibA_27207 | 10 | -1.044240061 | LRP12 | LDL receptor related protein 12 [Source:HGNC Symbol;Acc:HGNC:31708] |
| ENSG00000148187 | HGLibA_30114 | 10 | -1.044240061 | MRRF | mitochondrial ribosome recycling factor [Source:HGNC Symbol;Acc:HGNC:7234] |
| ENSG00000148204 | HGLibA_11072 | 10 | -1.044240061 | CRB2 | cell polarity complex component [Source:HGNC Symbol;Acc:HGNC:18688]" |
| ENSG00000148341 | HGLibA_43958 | 10 | -1.044240061 | SH3GLB2 | endophilin B2 [Source:HGNC Symbol;Acc:HGNC:10834]" |
| ENSG00000148948 | HGLibA_27382 | 10 | -1.044240061 | LRRC4C | leucine rich repeat containing 4C [Source:HGNC Symbol;Acc:HGNC:29317] |
| ENSG00000151779 | HGLibA_31203 | 10 | -1.044240061 | NBAS | neuroblastoma amplified sequence [Source:HGNC Symbol;Acc:HGNC:15625] |
| ENSG00000153250 | HGLibA_40586 | 10 | -1.044240061 | RBMS1 | RNA binding motif single stranded interacting protein 1 [Source:HGNC Symbol;Acc:HGNC:9907] |
| ENSG00000153774 | HGLibA_09257 | 10 | -1.044240061 | CFDP1 | craniofacial development protein 1 [Source:HGNC Symbol;Acc:HGNC:1873] |
| ENSG00000154175 | HGLibA_00300 | 10 | -1.044240061 | ABI3BP | ABI family member 3 binding protein [Source:HGNC Symbol;Acc:HGNC:17265] |
| ENSG00000154222 | HGLibA_07478 | 10 | -1.044240061 | CC2D1B | coiled-coil and C2 domain containing 1B [Source:HGNC Symbol;Acc:HGNC:29386] |
| ENSG00000155970 | HGLibA_29228 | 10 | -1.044240061 | MICU3 | mitochondrial calcium uptake family member 3 [Source:HGNC Symbol;Acc:HGNC:27820] |
| ENSG00000157764 | HGLibA_04752 | 10 | -1.044240061 | BRAF | serine/threonine kinase [Source:HGNC Symbol;Acc:HGNC:1097]" |
| ENSG00000158113 | HGLibA_27361 | 10 | -1.044240061 | LRRC43 | leucine rich repeat containing 43 [Source:HGNC Symbol;Acc:HGNC:28562] |
| ENSG00000158195 | HGLibA_53956 | 10 | -1.044240061 | WASF2 | WAS protein family member 2 [Source:HGNC Symbol;Acc:HGNC:12733] |
| ENSG00000158941 | HGLibA_07490 | 10 | -1.044240061 | CCAR2 | cell cycle and apoptosis regulator 2 [Source:HGNC Symbol;Acc:HGNC:23360] |
| ENSG00000159055 | HGLibA_29306 | 10 | -1.044240061 | MIS18A | MIS18 kinetochore protein A [Source:HGNC Symbol;Acc:HGNC:1286] |
| ENSG00000159348 | HGLibA_12002 | 10 | -1.044240061 | CYB5R1 | cytochrome b5 reductase 1 [Source:HGNC Symbol;Acc:HGNC:13397] |
| ENSG00000159713 | HGLibA_51171 | 10 | -1.044240061 | TPPP3 | tubulin polymerization promoting protein family member 3 [Source:HGNC Symbol;Acc:HGNC:24162] |
| ENSG00000165030 | HGLibA_31809 | 10 | -1.044240061 | NFIL3 | interleukin 3 regulated [Source:HGNC Symbol;Acc:HGNC:7787]" |
| ENSG00000165288 | HGLibA_04857 | 10 | -1.044240061 | BRWD3 | bromodomain and WD repeat domain containing 3 [Source:HGNC Symbol;Acc:HGNC:17342] |
| ENSG00000165471 | HGLibA_28498 | 10 | -1.044240061 | MBL2 | mannose binding lectin 2 [Source:HGNC Symbol;Acc:HGNC:6922] |
| ENSG00000165923 | HGLibA_01168 | 10 | -1.044240061 | AGBL2 | ATP/GTP binding protein like 2 [Source:HGNC Symbol;Acc:HGNC:26296] |
| ENSG00000166323 | HGLibA_05169 | 10 | -1.044240061 | C11orf65 | chromosome 11 open reading frame 65 [Source:HGNC Symbol;Acc:HGNC:28519] |
| ENSG00000166405 | HGLibA_41232 | 10 | -1.044240061 | RIC3 | RIC3 acetylcholine receptor chaperone [Source:HGNC Symbol;Acc:HGNC:30338] |
| ENSG00000170037 | HGLibA_10441 | 10 | -1.044240061 | CNTROB | centriole duplication and spindle assembly protein [Source:HGNC Symbol;Acc:HGNC:29616]" |
| ENSG00000170260 | HGLibA_55587 | 10 | -1.044240061 | ZNF212 | zinc finger protein 212 [Source:HGNC Symbol;Acc:HGNC:13004] |
| ENSG00000170873 | HGLibA_30495 | 10 | -1.044240061 | MTSS1 | I-BAR domain containing [Source:HGNC Symbol;Acc:HGNC:20443]" |
| ENSG00000171004 | HGLibA_22145 | 10 | -1.044240061 | HS6ST2 | heparan sulfate 6-O-sulfotransferase 2 [Source:HGNC Symbol;Acc:HGNC:19133] |
| ENSG00000171119 | HGLibA_32731 | 10 | -1.044240061 | NRTN | neurturin [Source:HGNC Symbol;Acc:HGNC:8007] |
| ENSG00000171160 | HGLibA_29676 | 10 | -1.044240061 | MORN4 | MORN repeat containing 4 [Source:HGNC Symbol;Acc:HGNC:24001] |
| ENSG00000171357 | HGLibA_27631 | 10 | -1.044240061 | LURAP1 | leucine rich adaptor protein 1 [Source:HGNC Symbol;Acc:HGNC:32327] |
| ENSG00000171596 | HGLibA_32215 | 10 | -1.044240061 | NMUR1 | neuromedin U receptor 1 [Source:HGNC Symbol;Acc:HGNC:4518] |
| ENSG00000172757 | HGLibA_09281 | 10 | -1.044240061 | CFL1 | cofilin 1 [Source:HGNC Symbol;Acc:HGNC:1874] |
| ENSG00000174004 | HGLibA_32722 | 10 | -1.044240061 | NRROS | negative regulator of reactive oxygen species [Source:HGNC Symbol;Acc:HGNC:24613] |
| ENSG00000174010 | HGLibA_25138 | 10 | -1.044240061 | KLHL15 | kelch like family member 15 [Source:HGNC Symbol;Acc:HGNC:29347] |
| ENSG00000174428 | HGLibA_20574 | 10 | -1.044240061 | GTF2IRD2B | GTF2I repeat domain containing 2B [Source:HGNC Symbol;Acc:HGNC:33125] |
| ENSG00000175455 | HGLibA_07613 | 10 | -1.044240061 | CCDC14 | coiled-coil domain containing 14 [Source:HGNC Symbol;Acc:HGNC:25766] |
| ENSG00000178718 | HGLibA_42029 | 10 | -1.044240061 | RPP25 | ribonuclease P and MRP subunit p25 [Source:HGNC Symbol;Acc:HGNC:30361] |
| ENSG00000179476 | HGLibA_05360 | 10 | -1.044240061 | C14orf28 | chromosome 14 open reading frame 28 [Source:HGNC Symbol;Acc:HGNC:19834] |
| ENSG00000179820 | HGLibA_30630 | 10 | -1.044240061 | MYADM | myeloid associated differentiation marker [Source:HGNC Symbol;Acc:HGNC:7544] |
| ENSG00000180090 | HGLibA_33914 | 10 | -1.044240061 | OR3A1 | olfactory receptor family 3 subfamily A member 1 [Source:HGNC Symbol;Acc:HGNC:8282] |
| ENSG00000181408 | HGLibA_53467 | 10 | -1.044240061 | UTS2R | urotensin 2 receptor [Source:HGNC Symbol;Acc:HGNC:4468] |
| ENSG00000181965 | HGLibA_31746 | 10 | -1.044240061 | NEUROG1 | neurogenin 1 [Source:HGNC Symbol;Acc:HGNC:7764] |
| ENSG00000184500 | HGLibA_38689 | 10 | -1.044240061 | PROS1 | protein S [Source:HGNC Symbol;Acc:HGNC:9456] |
| ENSG00000184544 | HGLibA_13151 | 10 | -1.044240061 | DHRS7C | dehydrogenase/reductase 7C [Source:HGNC Symbol;Acc:HGNC:32423] |
| ENSG00000184731 | HGLibA_16019 | 10 | -1.044240061 | FAM110C | family with sequence similarity 110 member C [Source:HGNC Symbol;Acc:HGNC:33340] |
| ENSG00000185479 | HGLibA_25516 | 10 | -1.044240061 | KRT6B | keratin 6B [Source:HGNC Symbol;Acc:HGNC:6444] |
| ENSG00000185972 | HGLibA_07950 | 10 | -1.044240061 | CCIN | calicin [Source:HGNC Symbol;Acc:HGNC:1568] |
| ENSG00000186810 | HGLibA_11887 | 10 | -1.044240061 | CXCR3 | C-X-C motif chemokine receptor 3 [Source:HGNC Symbol;Acc:HGNC:4540] |
| ENSG00000186818 | HGLibA_26517 | 10 | -1.044240061 | LILRB4 | leukocyte immunoglobulin like receptor B4 [Source:HGNC Symbol;Acc:HGNC:6608] |
| ENSG00000189182 | HGLibA_25543 | 10 | -1.044240061 | KRT77 | keratin 77 [Source:HGNC Symbol;Acc:HGNC:20411] |
| ENSG00000196664 | HGLibA_49660 | 10 | -1.044240061 | TLR7 | toll like receptor 7 [Source:HGNC Symbol;Acc:HGNC:15631] |
| ENSG00000196739 | HGLibA_10555 | 10 | -1.044240061 | COL27A1 | collagen type XXVII alpha 1 chain [Source:HGNC Symbol;Acc:HGNC:22986] |
| ENSG00000196772 | HGLibA_33629 | 10 | -1.044240061 | OR14A16 | olfactory receptor family 14 subfamily A member 16 [Source:HGNC Symbol;Acc:HGNC:15022] |
| ENSG00000198173 | HGLibA_16614 | 10 | -1.044240061 | FAM47C | family with sequence similarity 47 member C [Source:HGNC Symbol;Acc:HGNC:25301] |
| ENSG00000198797 | HGLibA_04814 | 10 | -1.044240061 | BRINP2 | BMP/retinoic acid inducible neural specific 2 [Source:HGNC Symbol;Acc:HGNC:13746] |
| ENSG00000203785 | HGLibA_46817 | 10 | -1.044240061 | SPRR2E | small proline rich protein 2E [Source:HGNC Symbol;Acc:HGNC:11265] |
| ENSG00000204928 | HGLibA_20393 | 10 | -1.044240061 | GRXCR2 | glutaredoxin and cysteine rich domain containing 2 [Source:HGNC Symbol;Acc:HGNC:33862] |
| ENSG00000206069 | HGLibA_50206 | 10 | -1.044240061 | TMEM211 | transmembrane protein 211 [Source:HGNC Symbol;Acc:HGNC:33725] |
| ENSG00000215568 | HGLibA_18330 | 10 | -1.044240061 | GAB4 | GRB2 associated binding protein family member 4 [Source:HGNC Symbol;Acc:HGNC:18325] |
| ENSG00000221932 | HGLibA_21150 | 10 | -1.044240061 | HEPN1 | down-regulated 1 [Source:HGNC Symbol;Acc:HGNC:34400]" |
| ENSG00000233493 | HGLibA_50280 | 10 | -1.044240061 | TMEM238 | transmembrane protein 238 [Source:HGNC Symbol;Acc:HGNC:40042] |
| ENSG00000233917 | HGLibA_37718 | 10 | -1.044240061 | POTEB | POTE ankyrin domain family member B [Source:HGNC Symbol;Acc:HGNC:33734] |
| ENSG00000240053 | HGLibA_27659 | 10 | -1.044240061 | LY6G5B | lymphocyte antigen 6 family member G5B [Source:HGNC Symbol;Acc:HGNC:13931] |
| ENSG00000244687 | HGLibA_52667 | 10 | -1.044240061 | UBE2V1 | ubiquitin conjugating enzyme E2 V1 [Source:HGNC Symbol;Acc:HGNC:12494] |
| ENSG00000253846 | HGLibA_35521 | 10 | -1.044240061 | PCDHGA10 | 10 [Source:HGNC Symbol;Acc:HGNC:8697]" |
| ENSG00000262814 | HGLibA_29898 | 10 | -1.044240061 | MRPL12 | mitochondrial ribosomal protein L12 [Source:HGNC Symbol;Acc:HGNC:10378] |
| ENSG00000266028 | HGLibA_46970 | 10 | -1.044240061 | SRGAP2 | SLIT-ROBO Rho GTPase activating protein 2 [Source:HGNC Symbol;Acc:HGNC:19751] |
| FAM127A | HGLibA_16087 | 10 | -1.044240061 | FAM127A | - |
| FAM159A | HGLibA_16199 | 10 | -1.044240061 | FAM159A | - |
| hsa-mir-1185-1 | HGLibA_57242 | 10 | -1.044240061 | hsa-mir-1185-1 | - |
| hsa-mir-1273d | HGLibA_57539 | 10 | -1.044240061 | hsa-mir-1273d | - |
| hsa-mir-1294 | HGLibA_57653 | 10 | -1.044240061 | hsa-mir-1294 | - |
| hsa-mir-200b | HGLibA_58154 | 10 | -1.044240061 | hsa-mir-200b | - |
| hsa-mir-299 | HGLibA_58407 | 10 | -1.044240061 | hsa-mir-299 | - |
| hsa-mir-3179-3 | HGLibA_58798 | 10 | -1.044240061 | hsa-mir-3179-3 | - |
| hsa-mir-320d-1 | HGLibA_58942 | 10 | -1.044240061 | hsa-mir-320d-1 | - |
| hsa-mir-3611 | HGLibA_59075 | 10 | -1.044240061 | hsa-mir-3611 | - |
| hsa-mir-3615 | HGLibA_59091 | 10 | -1.044240061 | hsa-mir-3615 | - |
| hsa-mir-3673 | HGLibA_59246 | 10 | -1.044240061 | hsa-mir-3673 | - |
| hsa-mir-4271 | HGLibA_59791 | 10 | -1.044240061 | hsa-mir-4271 | - |
| hsa-mir-4445 | HGLibA_60185 | 10 | -1.044240061 | hsa-mir-4445 | - |
| hsa-mir-4445 | HGLibA_60187 | 10 | -1.044240061 | hsa-mir-4445 | - |
| hsa-mir-4453 | HGLibA_60219 | 10 | -1.044240061 | hsa-mir-4453 | - |
| hsa-mir-4485 | HGLibA_60357 | 10 | -1.044240061 | hsa-mir-4485 | - |
| hsa-mir-519a-2 | HGLibA_61715 | 10 | -1.044240061 | hsa-mir-519a-2 | - |
| hsa-mir-548a-2 | HGLibA_61842 | 10 | -1.044240061 | hsa-mir-548a-2 | - |
| hsa-mir-548a-3 | HGLibA_61843 | 10 | -1.044240061 | hsa-mir-548a-3 | - |
| hsa-mir-548o | HGLibA_62048 | 10 | -1.044240061 | hsa-mir-548o | - |
| hsa-mir-554 | HGLibA_62134 | 10 | -1.044240061 | hsa-mir-554 | - |
| hsa-mir-5703 | HGLibA_62352 | 10 | -1.044240061 | hsa-mir-5703 | - |
| hsa-mir-6080 | HGLibA_62579 | 10 | -1.044240061 | hsa-mir-6080 | - |
| hsa-mir-6124 | HGLibA_62643 | 10 | -1.044240061 | hsa-mir-6124 | - |
| hsa-mir-629 | HGLibA_62754 | 10 | -1.044240061 | hsa-mir-629 | - |
| hsa-mir-6777 | HGLibA_63281 | 10 | -1.044240061 | hsa-mir-6777 | - |
| hsa-mir-6847 | HGLibA_63560 | 10 | -1.044240061 | hsa-mir-6847 | - |
| hsa-mir-7856 | HGLibA_64013 | 10 | -1.044240061 | hsa-mir-7856 | - |
| hsa-mir-8080 | HGLibA_64163 | 10 | -1.044240061 | hsa-mir-8080 | - |
| hsa-mir-924 | HGLibA_64287 | 10 | -1.044240061 | hsa-mir-924 | - |
| KIAA1009 | HGLibA_24675 | 10 | -1.044240061 | KIAA1009 | - |
| C5orf30 | HGLibA_06375 | 9 | -1.053285404 | C5orf30 | - |
| C9orf16 | HGLibA_06698 | 9 | -1.053285404 | C9orf16 | - |
| CXorf27 | HGLibA_11910 | 9 | -1.053285404 | CXorf27 | - |
| ENSG00000005100 | HGLibA_13182 | 9 | -1.053285404 | DHX33 | DEAH-box helicase 33 [Source:HGNC Symbol;Acc:HGNC:16718] |
| ENSG00000007314 | HGLibA_42991 | 9 | -1.053285404 | SCN4A | sodium voltage-gated channel alpha subunit 4 [Source:HGNC Symbol;Acc:HGNC:10591] |
| ENSG00000008735 | HGLibA_28288 | 9 | -1.053285404 | MAPK8IP2 | mitogen-activated protein kinase 8 interacting protein 2 [Source:HGNC Symbol;Acc:HGNC:6883] |
| ENSG00000013297 | HGLibA_09853 | 9 | -1.053285404 | CLDN11 | claudin 11 [Source:HGNC Symbol;Acc:HGNC:8514] |
| ENSG00000013306 | HGLibA_44729 | 9 | -1.053285404 | SLC25A39 | solute carrier family 25 member 39 [Source:HGNC Symbol;Acc:HGNC:24279] |
| ENSG00000036549 | HGLibA_57094 | 9 | -1.053285404 | ZZZ3 | zinc finger ZZ-type containing 3 [Source:NCBI gene;Acc:26009] |
| ENSG00000048649 | HGLibA_42311 | 9 | -1.053285404 | RSF1 | remodeling and spacing factor 1 [Source:HGNC Symbol;Acc:HGNC:18118] |
| ENSG00000052850 | HGLibA_01738 | 9 | -1.053285404 | ALX4 | ALX homeobox 4 [Source:HGNC Symbol;Acc:HGNC:450] |
| ENSG00000057019 | HGLibA_12472 | 9 | -1.053285404 | DCBLD2 | CUB and LCCL domain containing 2 [Source:HGNC Symbol;Acc:HGNC:24627]" |
| ENSG00000064270 | HGLibA_03630 | 9 | -1.053285404 | ATP2C2 | ATPase secretory pathway Ca2+ transporting 2 [Source:HGNC Symbol;Acc:HGNC:29103] |
| ENSG00000068438 | HGLibA_18135 | 9 | -1.053285404 | FTSJ1 | FtsJ RNA methyltransferase homolog 1 [Source:HGNC Symbol;Acc:HGNC:13254] |
| ENSG00000079689 | HGLibA_42939 | 9 | -1.053285404 | SCGN | EF-hand calcium binding protein [Source:HGNC Symbol;Acc:HGNC:16941]" |
| ENSG00000084463 | HGLibA_53971 | 9 | -1.053285404 | WBP11 | WW domain binding protein 11 [Source:HGNC Symbol;Acc:HGNC:16461] |
| ENSG00000087008 | HGLibA_00503 | 9 | -1.053285404 | ACOX3 | pristanoyl [Source:HGNC Symbol;Acc:HGNC:121]" |
| ENSG00000090905 | HGLibA_50912 | 9 | -1.053285404 | TNRC6A | trinucleotide repeat containing 6A [Source:HGNC Symbol;Acc:HGNC:11969] |
| ENSG00000096080 | HGLibA_30050 | 9 | -1.053285404 | MRPS18A | mitochondrial ribosomal protein S18A [Source:HGNC Symbol;Acc:HGNC:14515] |
| ENSG00000099812 | HGLibA_29313 | 9 | -1.053285404 | MISP | mitotic spindle positioning [Source:HGNC Symbol;Acc:HGNC:27000] |
| ENSG00000100028 | HGLibA_45982 | 9 | -1.053285404 | SNRPD3 | small nuclear ribonucleoprotein D3 polypeptide [Source:HGNC Symbol;Acc:HGNC:11160] |
| ENSG00000100228 | HGLibA_39891 | 9 | -1.053285404 | RAB36 | member RAS oncogene family [Source:HGNC Symbol;Acc:HGNC:9775]" |
| ENSG00000100522 | HGLibA_19570 | 9 | -1.053285404 | GNPNAT1 | glucosamine-phosphate N-acetyltransferase 1 [Source:HGNC Symbol;Acc:HGNC:19980] |
| ENSG00000102078 | HGLibA_44648 | 9 | -1.053285404 | SLC25A14 | solute carrier family 25 member 14 [Source:HGNC Symbol;Acc:HGNC:10984] |

| | | | | | |
|---|---|---|---|---|---|
| ENSG00000102606 | HGLibA_02875 | 9 | -1.053285404 | ARHGEF7 | Rho guanine nucleotide exchange factor 7 [Source:HGNC Symbol;Acc:HGNC:15607] |
| ENSG00000103510 | HGLibA_24068 | 9 | -1.053285404 | KAT8 | lysine acetyltransferase 8 [Source:HGNC Symbol;Acc:HGNC:17933] |
| ENSG00000104613 | HGLibA_23409 | 9 | -1.053285404 | INTS10 | integrator complex subunit 10 [Source:HGNC Symbol;Acc:HGNC:25548] |
| ENSG00000104941 | HGLibA_42338 | 9 | -1.053285404 | RSPH6A | radial spoke head 6 homolog A [Source:HGNC Symbol;Acc:HGNC:14241] |
| ENSG00000104953 | HGLibA_49620 | 9 | -1.053285404 | TLE6 | transducin like enhancer of split 6 [Source:HGNC Symbol;Acc:HGNC:30788] |
| ENSG00000105523 | HGLibA_16753 | 9 | -1.053285404 | FAM83E | family with sequence similarity 83 member E [Source:HGNC Symbol;Acc:HGNC:25972] |
| ENSG00000105695 | HGLibA_27887 | 9 | -1.053285404 | MAG | myelin associated glycoprotein [Source:HGNC Symbol;Acc:HGNC:6783] |
| ENSG00000107443 | HGLibA_08109 | 9 | -1.053285404 | CCNJ | cyclin J [Source:HGNC Symbol;Acc:HGNC:23434] |
| ENSG00000111276 | HGLibA_08825 | 9 | -1.053285404 | CDKN1B | cyclin dependent kinase inhibitor 1B [Source:HGNC Symbol;Acc:HGNC:1785] |
| ENSG00000111325 | HGLibA_33310 | 9 | -1.053285404 | OGFOD2 | 2-oxoglutarate and iron dependent oxygenase domain containing 2 [Source:HGNC Symbol;Acc:HGNC:25823] |
| ENSG00000112333 | HGLibA_32605 | 9 | -1.053285404 | NR2E1 | nuclear receptor subfamily 2 group E member 1 [Source:HGNC Symbol;Acc:HGNC:7973] |
| ENSG00000117262 | HGLibA_20108 | 9 | -1.053285404 | GPR89A | G protein-coupled receptor 89A [Source:HGNC Symbol;Acc:HGNC:31984] |
| ENSG00000118412 | HGLibA_07328 | 9 | -1.053285404 | CASP8AP2 | caspase 8 associated protein 2 [Source:HGNC Symbol;Acc:HGNC:1510] |
| ENSG00000119760 | HGLibA_47827 | 9 | -1.053285404 | SUPT7L | STAGA complex gamma subunit [Source:HGNC Symbol;Acc:HGNC:30632]" |
| ENSG00000120647 | HGLibA_07869 | 9 | -1.053285404 | CCDC77 | coiled-coil domain containing 77 [Source:HGNC Symbol;Acc:HGNC:28203] |
| ENSG00000121075 | HGLibA_48671 | 9 | -1.053285404 | TBX4 | T-box 4 [Source:HGNC Symbol;Acc:HGNC:11603] |
| ENSG00000122557 | HGLibA_21174 | 9 | -1.053285404 | HERPUD2 | HERPUD family member 2 [Source:HGNC Symbol;Acc:HGNC:21915] |
| ENSG00000122735 | HGLibA_13504 | 9 | -1.053285404 | DNAI1 | dynein axonemal intermediate chain 1 [Source:HGNC Symbol;Acc:HGNC:2954] |
| ENSG00000123179 | HGLibA_14376 | 9 | -1.053285404 | EBPL | EBP like [Source:HGNC Symbol;Acc:HGNC:18061] |
| ENSG00000123297 | HGLibA_51840 | 9 | -1.053285404 | TSFM | mitochondrial [Source:HGNC Symbol;Acc:HGNC:12367]" |
| ENSG00000123552 | HGLibA_53373 | 9 | -1.053285404 | USP45 | ubiquitin specific peptidase 45 [Source:HGNC Symbol;Acc:HGNC:20080] |
| ENSG00000124019 | HGLibA_16079 | 9 | -1.053285404 | FAM124B | family with sequence similarity 124 member B [Source:HGNC Symbol;Acc:HGNC:26224] |
| ENSG00000125846 | HGLibA_55464 | 9 | -1.053285404 | ZNF133 | zinc finger protein 133 [Source:HGNC Symbol;Acc:HGNC:12917] |
| ENSG00000129245 | HGLibA_18222 | 9 | -1.053285404 | FXR2 | FMR1 autosomal homolog 2 [Source:HGNC Symbol;Acc:HGNC:4024] |
| ENSG00000130656 | HGLibA_20943 | 9 | -1.053285404 | HBZ | hemoglobin subunit zeta [Source:HGNC Symbol;Acc:HGNC:4835] |
| ENSG00000130711 | HGLibA_38370 | 9 | -1.053285404 | PRDM12 | PR/SET domain 12 [Source:HGNC Symbol;Acc:HGNC:13997] |
| ENSG00000131558 | HGLibA_15749 | 9 | -1.053285404 | EXOC4 | exocyst complex component 4 [Source:HGNC Symbol;Acc:HGNC:30389] |
| ENSG00000132693 | HGLibA_11217 | 9 | -1.053285404 | CRP | C-reactive protein [Source:HGNC Symbol;Acc:HGNC:2367] |
| ENSG00000133063 | HGLibA_09454 | 9 | -1.053285404 | CHIT1 | chitinase 1 [Source:HGNC Symbol;Acc:HGNC:1936] |
| ENSG00000134398 | HGLibA_15515 | 9 | -1.053285404 | ERN2 | endoplasmic reticulum to nucleus signaling 2 [Source:HGNC Symbol;Acc:HGNC:16942] |
| ENSG00000136732 | HGLibA_20703 | 9 | -1.053285404 | GYPC | glycophorin C (Gerbich blood group) [Source:HGNC Symbol;Acc:HGNC:4704] |
| ENSG00000137103 | HGLibA_50550 | 9 | -1.053285404 | TMEM8B | transmembrane protein 8B [Source:HGNC Symbol;Acc:HGNC:21427] |
| ENSG00000137860 | HGLibA_44834 | 9 | -1.053285404 | SLC28A2 | solute carrier family 28 member 2 [Source:HGNC Symbol;Acc:HGNC:11002] |
| ENSG00000139350 | HGLibA_31598 | 9 | -1.053285404 | NEDD1 | developmentally down-regulated 1 [Source:HGNC Symbol;Acc:HGNC:7723]" |
| ENSG00000139835 | HGLibA_20384 | 9 | -1.053285404 | GRTP1 | growth hormone regulated TBC protein 1 [Source:HGNC Symbol;Acc:HGNC:20310] |
| ENSG00000139985 | HGLibA_00755 | 9 | -1.053285404 | ADAM21 | ADAM metallopeptidase domain 21 [Source:HGNC Symbol;Acc:HGNC:200] |
| ENSG00000140025 | HGLibA_14523 | 9 | -1.053285404 | EFCAB11 | EF-hand calcium binding domain 11 [Source:HGNC Symbol;Acc:HGNC:20357] |
| ENSG00000141127 | HGLibA_38768 | 9 | -1.053285404 | PRPSAP2 | phosphoribosyl pyrophosphate synthetase associated protein 2 [Source:HGNC Symbol;Acc:HGNC:9467] |
| ENSG00000141665 | HGLibA_17029 | 9 | -1.053285404 | FBXO15 | F-box protein 15 [Source:HGNC Symbol;Acc:HGNC:13617] |
| ENSG00000143171 | HGLibA_42512 | 9 | -1.053285404 | RXRG | retinoid X receptor gamma [Source:HGNC Symbol;Acc:HGNC:10479] |
| ENSG00000143321 | HGLibA_21039 | 9 | -1.053285404 | HDGF | heparin binding growth factor [Source:HGNC Symbol;Acc:HGNC:4856] |
| ENSG00000143845 | HGLibA_15636 | 9 | -1.053285404 | ETNK2 | ethanolamine kinase 2 [Source:HGNC Symbol;Acc:HGNC:25575] |
| ENSG00000144655 | HGLibA_11424 | 9 | -1.053285404 | CSRNP1 | cysteine and serine rich nuclear protein 1 [Source:HGNC Symbol;Acc:HGNC:14300] |
| ENSG00000144867 | HGLibA_47029 | 9 | -1.053285404 | SRPRB | SRP receptor subunit beta [Source:HGNC Symbol;Acc:HGNC:24085] |
| ENSG00000146063 | HGLibA_51494 | 9 | -1.053285404 | TRIM41 | tripartite motif containing 41 [Source:HGNC Symbol;Acc:HGNC:19013] |
| ENSG00000146433 | HGLibA_50100 | 9 | -1.053285404 | TMEM181 | transmembrane protein 181 [Source:HGNC Symbol;Acc:HGNC:20958] |
| ENSG00000148053 | HGLibA_32876 | 9 | -1.053285404 | NTRK2 | neurotrophic receptor tyrosine kinase 2 [Source:HGNC Symbol;Acc:HGNC:8032] |
| ENSG00000150347 | HGLibA_02908 | 9 | -1.053285404 | ARID5B | AT-rich interaction domain 5B [Source:HGNC Symbol;Acc:HGNC:17362] |
| ENSG00000151461 | HGLibA_53060 | 9 | -1.053285404 | UPF2 | regulator of nonsense mediated mRNA decay [Source:HGNC Symbol;Acc:HGNC:17854]" |
| ENSG00000152580 | HGLibA_22877 | 9 | -1.053285404 | IGSF10 | immunoglobulin superfamily member 10 [Source:HGNC Symbol;Acc:HGNC:26384] |
| ENSG00000153246 | HGLibA_36928 | 9 | -1.053285404 | PLA2R1 | phospholipase A2 receptor 1 [Source:HGNC Symbol;Acc:HGNC:9042] |
| ENSG00000154143 | HGLibA_35116 | 9 | -1.053285404 | PANX3 | pannexin 3 [Source:HGNC Symbol;Acc:HGNC:20573] |
| ENSG00000154734 | HGLibA_00790 | 9 | -1.053285404 | ADAMTS1 | ADAM metallopeptidase with thrombospondin type 1 motif 1 [Source:HGNC Symbol;Acc:HGNC:217] |
| ENSG00000155962 | HGLibA_10025 | 9 | -1.053285404 | CLIC2 | chloride intracellular channel 2 [Source:HGNC Symbol;Acc:HGNC:2063] |
| ENSG00000159650 | HGLibA_53152 | 9 | -1.053285404 | UROC1 | urocanate hydratase 1 [Source:HGNC Symbol;Acc:HGNC:26444] |
| ENSG00000160233 | HGLibA_27304 | 9 | -1.053285404 | LRRC3 | leucine rich repeat containing 3 [Source:HGNC Symbol;Acc:HGNC:14965] |
| ENSG00000162641 | HGLibA_01466 | 9 | -1.053285404 | AKNAD1 | AKNA domain containing 1 [Source:HGNC Symbol;Acc:HGNC:28398] |
| ENSG00000163191 | HGLibA_42537 | 9 | -1.053285404 | S100A11 | S100 calcium binding protein A11 [Source:HGNC Symbol;Acc:HGNC:10488] |
| ENSG00000163468 | HGLibA_08197 | 9 | -1.053285404 | CCT3 | chaperonin containing TCP1 subunit 3 [Source:HGNC Symbol;Acc:HGNC:1616] |
| ENSG00000163491 | HGLibA_31642 | 9 | -1.053285404 | NEK10 | NIMA related kinase 10 [Source:HGNC Symbol;Acc:HGNC:18592] |
| ENSG00000164197 | HGLibA_41579 | 9 | -1.053285404 | RNF180 | ring finger protein 180 [Source:HGNC Symbol;Acc:HGNC:27752] |
| ENSG00000164253 | HGLibA_54088 | 9 | -1.053285404 | WDR41 | WD repeat domain 41 [Source:HGNC Symbol;Acc:HGNC:25601] |
| ENSG00000164509 | HGLibA_23133 | 9 | -1.053285404 | IL31RA | interleukin 31 receptor A [Source:HGNC Symbol;Acc:HGNC:18969] |
| ENSG00000165140 | HGLibA_16965 | 9 | -1.053285404 | FBP1 | fructose-bisphosphatase 1 [Source:HGNC Symbol;Acc:HGNC:3606] |
| ENSG00000165548 | HGLibA_50479 | 9 | -1.053285404 | TMEM63C | transmembrane protein 63C [Source:HGNC Symbol;Acc:HGNC:23787] |
| ENSG00000166148 | HGLibA_03903 | 9 | -1.053285404 | AVPR1A | arginine vasopressin receptor 1A [Source:HGNC Symbol;Acc:HGNC:895] |
| ENSG00000166796 | HGLibA_26232 | 9 | -1.053285404 | LDHC | lactate dehydrogenase C [Source:HGNC Symbol;Acc:HGNC:6544] |
| ENSG00000166924 | HGLibA_33180 | 9 | -1.053285404 | NYAP1 | neuronal tyrosine phosphorylated phosphoinositide-3-kinase adaptor 1 [Source:HGNC Symbol;Acc:HGNC:22009] |
| ENSG00000167191 | HGLibA_20129 | 9 | -1.053285404 | GPRC5B | G protein-coupled receptor class C group 5 member B [Source:HGNC Symbol;Acc:HGNC:13308] |
| ENSG00000167487 | HGLibA_25168 | 9 | -1.053285404 | KLHL26 | kelch like family member 26 [Source:HGNC Symbol;Acc:HGNC:25623] |
| ENSG00000168243 | HGLibA_19526 | 9 | -1.053285404 | GNG4 | G protein subunit gamma 4 [Source:HGNC Symbol;Acc:HGNC:4407] |
| ENSG00000168546 | HGLibA_18967 | 9 | -1.053285404 | GFRA2 | GDNF family receptor alpha 2 [Source:HGNC Symbol;Acc:HGNC:4244] |
| ENSG00000168679 | HGLibA_44441 | 9 | -1.053285404 | SLC16A4 | solute carrier family 16 member 4 [Source:HGNC Symbol;Acc:HGNC:10925] |
| ENSG00000169241 | HGLibA_45236 | 9 | -1.053285404 | SLC50A1 | solute carrier family 50 member 1 [Source:HGNC Symbol;Acc:HGNC:30657] |
| ENSG00000169403 | HGLibA_39245 | 9 | -1.053285404 | PTAFR | platelet activating factor receptor [Source:HGNC Symbol;Acc:HGNC:9582] |
| ENSG00000169783 | HGLibA_26584 | 9 | -1.053285404 | LINGO1 | leucine rich repeat and Ig domain containing 1 [Source:HGNC Symbol;Acc:HGNC:21205] |
| ENSG00000170464 | HGLibA_13587 | 9 | -1.053285404 | DNAJC18 | DnaJ heat shock protein family (Hsp40) member C18 [Source:HGNC Symbol;Acc:HGNC:28429] |
| ENSG00000170967 | HGLibA_12610 | 9 | -1.053285404 | DDI1 | DNA damage inducible 1 homolog 1 [Source:HGNC Symbol;Acc:HGNC:18961] |
| ENSG00000171724 | HGLibA_53544 | 9 | -1.053285404 | VAT1L | vesicle amine transport 1 like [Source:HGNC Symbol;Acc:HGNC:29315] |
| ENSG00000171864 | HGLibA_38641 | 9 | -1.053285404 | PRND | prion like protein doppel [Source:HGNC Symbol;Acc:HGNC:15748] |
| ENSG00000172742 | HGLibA_33982 | 9 | -1.053285404 | OR4D9 | olfactory receptor family 4 subfamily D member 9 [Source:HGNC Symbol;Acc:HGNC:15178] |
| ENSG00000173334 | HGLibA_51391 | 9 | -1.053285404 | TRIB1 | tribbles pseudokinase 1 [Source:HGNC Symbol;Acc:HGNC:16891] |
| ENSG00000173457 | HGLibA_38038 | 9 | -1.053285404 | PPP1R14B | protein phosphatase 1 regulatory inhibitor subunit 14B [Source:HGNC Symbol;Acc:HGNC:9057] |
| ENSG00000174963 | HGLibA_55331 | 9 | -1.053285404 | ZIC4 | Zic family member 4 [Source:HGNC Symbol;Acc:HGNC:20393] |
| ENSG00000176402 | HGLibA_19185 | 9 | -1.053285404 | GJC3 | gap junction protein gamma 3 [Source:HGNC Symbol;Acc:HGNC:17495] |
| ENSG00000176842 | HGLibA_23640 | 9 | -1.053285404 | IRX5 | iroquois homeobox 5 [Source:HGNC Symbol;Acc:HGNC:14361] |
| ENSG00000178078 | HGLibA_47346 | 9 | -1.053285404 | STAP2 | signal transducing adaptor family member 2 [Source:HGNC Symbol;Acc:HGNC:30430] |
| ENSG00000178233 | HGLibA_50004 | 9 | -1.053285404 | TMEM151B | transmembrane protein 151B [Source:HGNC Symbol;Acc:HGNC:21315] |
| ENSG00000179051 | HGLibA_40687 | 9 | -1.053285404 | RCC2 | regulator of chromosome condensation 2 [Source:HGNC Symbol;Acc:HGNC:30297] |
| ENSG00000179295 | HGLibA_39434 | 9 | -1.053285404 | PTPN11 | non-receptor type 11 [Source:HGNC Symbol;Acc:HGNC:9644]" |
| ENSG00000179886 | HGLibA_49475 | 9 | -1.053285404 | TIGD5 | tigger transposable element derived 5 [Source:HGNC Symbol;Acc:HGNC:18336] |
| ENSG00000180098 | HGLibA_51707 | 9 | -1.053285404 | TRNAU1AP | tRNA selenocysteine 1 associated protein 1 [Source:HGNC Symbol;Acc:HGNC:30813] |
| ENSG00000181085 | HGLibA_28266 | 9 | -1.053285404 | MAPK15 | mitogen-activated protein kinase 15 [Source:HGNC Symbol;Acc:HGNC:24667] |
| ENSG00000181873 | HGLibA_22479 | 9 | -1.053285404 | IBA57 | iron-sulfur cluster assembly [Source:HGNC Symbol;Acc:HGNC:27302]" |
| ENSG00000183066 | HGLibA_53981 | 9 | -1.053285404 | WBP2NL | WBP2 N-terminal like [Source:HGNC Symbol;Acc:HGNC:28389] |
| ENSG00000183206 | HGLibA_37723 | 9 | -1.053285404 | POTEC | POTE ankyrin domain family member C [Source:HGNC Symbol;Acc:HGNC:33894] |
| ENSG00000183336 | HGLibA_04682 | 9 | -1.053285404 | BOLA2 | bolA family member 2 [Source:HGNC Symbol;Acc:HGNC:29488] |
| ENSG00000183826 | HGLibA_04927 | 9 | -1.053285404 | BTBD9 | BTB domain containing 9 [Source:HGNC Symbol;Acc:HGNC:21228] |
| ENSG00000185009 | HGLibA_02330 | 9 | -1.053285404 | AP3M1 | adaptor related protein complex 3 subunit mu 1 [Source:HGNC Symbol;Acc:HGNC:569] |
| ENSG00000186431 | HGLibA_17165 | 9 | -1.053285404 | FCAR | Fc fragment of IgA receptor [Source:HGNC Symbol;Acc:HGNC:3608] |
| ENSG00000186675 | HGLibA_27983 | 9 | -1.053285404 | MAGEE2 | MAGE family member E2 [Source:HGNC Symbol;Acc:HGNC:24935] |
| ENSG00000186777 | HGLibA_56647 | 9 | -1.053285404 | ZNF732 | zinc finger protein 732 [Source:HGNC Symbol;Acc:HGNC:37138] |
| ENSG00000186930 | HGLibA_25817 | 9 | -1.053285404 | KRTAP6-2 | keratin associated protein 6-2 [Source:HGNC Symbol;Acc:HGNC:18932] |
| ENSG00000187559 | HGLibA_17847 | 9 | -1.053285404 | FOXD4L3 | forkhead box D4 like 3 [Source:HGNC Symbol;Acc:HGNC:18523] |
| ENSG00000188467 | HGLibA_44627 | 9 | -1.053285404 | SLC24A5 | solute carrier family 24 member 5 [Source:HGNC Symbol;Acc:HGNC:20611] |
| ENSG00000188488 | HGLibA_43522 | 9 | -1.053285404 | SERPINA5 | serpin family A member 5 [Source:HGNC Symbol;Acc:HGNC:8723] |
| ENSG00000188984 | HGLibA_00034 | 9 | -1.053285404 | AADACL3 | arylacetamide deacetylase like 3 [Source:HGNC Symbol;Acc:HGNC:32037] |
| ENSG00000196600 | HGLibA_44580 | 9 | -1.053285404 | SLC22A25 | solute carrier family 22 member 25 [Source:HGNC Symbol;Acc:HGNC:32935] |
| ENSG00000198833 | HGLibA_52617 | 9 | -1.053285404 | UBE2J1 | ubiquitin conjugating enzyme E2 J1 [Source:HGNC Symbol;Acc:HGNC:17598] |
| ENSG00000204640 | HGLibA_32203 | 9 | -1.053285404 | NMS | neuromedin S [Source:HGNC Symbol;Acc:HGNC:32203] |
| ENSG00000204657 | HGLibA_33805 | 9 | -1.053285404 | OR2H2 | olfactory receptor family 2 subfamily H member 2 [Source:HGNC Symbol;Acc:HGNC:8253] |
| ENSG00000205029 | HGLibA_34280 | 9 | -1.053285404 | OR5D16 | olfactory receptor family 5 subfamily D member 16 [Source:HGNC Symbol;Acc:HGNC:15283] |
| ENSG00000205456 | HGLibA_51087 | 9 | -1.053285404 | TP53TG3D | TP53 target 3D [Source:HGNC Symbol;Acc:HGNC:44657] |
| ENSG00000205846 | HGLibA_10004 | 9 | -1.053285404 | CLEC6A | C-type lectin domain containing 6A [Source:HGNC Symbol;Acc:HGNC:14556] |
| ENSG00000213463 | HGLibA_48010 | 9 | -1.053285404 | SYNJ2BP | synaptojanin 2 binding protein [Source:HGNC Symbol;Acc:HGNC:18955] |
| ENSG00000214300 | HGLibA_46592 | 9 | -1.053285404 | SPDYE3 | speedy/RINGO cell cycle regulator family member E3 [Source:HGNC Symbol;Acc:HGNC:35462] |
| ENSG00000235098 | HGLibA_02137 | 9 | -1.053285404 | ANKRD65 | ankyrin repeat domain 65 [Source:HGNC Symbol;Acc:HGNC:42950] |
| ENSG00000236609 | HGLibA_56875 | 9 | -1.053285404 | ZNF853 | zinc finger protein 853 [Source:HGNC Symbol;Acc:HGNC:21767] |
| ENSG00000261308 | HGLibA_17524 | 9 | -1.053285404 | FIGNL2 | fidgetin like 2 [Source:HGNC Symbol;Acc:HGNC:13287] |
| ENSG00000269526 | HGLibA_15545 | 9 | -1.053285404 | ERVV-1 | envelope [Source:HGNC Symbol;Acc:HGNC:26501]" |
| ENSG00000270872 | HGLibA_46980 | 9 | -1.053285404 | SRGAP2D | SLIT-ROBO Rho GTPase activating protein 2D (pseudogene) [Source:HGNC Symbol;Acc:HGNC:43932] |
| ENSG00000273706 | HGLibA_26447 | 9 | -1.053285404 | LHX1 | LIM homeobox 1 [Source:HGNC Symbol;Acc:HGNC:6593] |
| ENSG00000275113 | HGLibA_18480 | 9 | -1.053285404 | GAGE2E | G antigen 2E [Source:HGNC Symbol;Acc:HGNC:31960] |
| ENSG00000277462 | HGLibA_56501 | 9 | -1.053285404 | ZNF670 | zinc finger protein 670 [Source:HGNC Symbol;Acc:HGNC:28167] |
| FAM134B | HGLibA_16125 | 9 | -1.053285404 | FAM134B | - |
| GAGE7 | HGLibA_18489 | 9 | -1.053285404 | GAGE7 | - |
| hsa-mir-136 | HGLibA_57805 | 9 | -1.053285404 | hsa-mir-136 | - |
| hsa-mir-2861 | HGLibA_58388 | 9 | -1.053285404 | hsa-mir-2861 | - |
| hsa-mir-3167 | HGLibA_58747 | 9 | -1.053285404 | hsa-mir-3167 | - |
| hsa-mir-3591 | HGLibA_59053 | 9 | -1.053285404 | hsa-mir-3591 | - |
| hsa-mir-3658 | HGLibA_59179 | 9 | -1.053285404 | hsa-mir-3658 | - |
| hsa-mir-4419b | HGLibA_60064 | 9 | -1.053285404 | hsa-mir-4419b | - |
| hsa-mir-4458 | HGLibA_60238 | 9 | -1.053285404 | hsa-mir-4458 | - |
| hsa-mir-4663 | HGLibA_60764 | 9 | -1.053285404 | hsa-mir-4663 | - |
| hsa-mir-4672 | HGLibA_60803 | 9 | -1.053285404 | hsa-mir-4672 | - |

| | | | | | |
|---|---|---|---|---|---|
| hsa-mir-4698 | HGLibA_60899 | 9 | -1.053285404 | hsa-mir-4698 | - |
| hsa-mir-4769 | HGLibA_61174 | 9 | -1.053285404 | hsa-mir-4769 | - |
| hsa-mir-522 | HGLibA_61775 | 9 | -1.053285404 | hsa-mir-522 | - |
| hsa-mir-548aj-2 | HGLibA_61892 | 9 | -1.053285404 | hsa-mir-548aj-2 | - |
| hsa-mir-548aq | HGLibA_61915 | 9 | -1.053285404 | hsa-mir-548aq | - |
| hsa-mir-548at | HGLibA_61923 | 9 | -1.053285404 | hsa-mir-548at | - |
| hsa-mir-6511b-2 | HGLibA_62918 | 9 | -1.053285404 | hsa-mir-6511b-2 | - |
| hsa-mir-6892 | HGLibA_63752 | 9 | -1.053285404 | hsa-mir-6892 | - |
| hsa-mir-6895 | HGLibA_63765 | 9 | -1.053285404 | hsa-mir-6895 | - |
| hsa-mir-7977 | HGLibA_64036 | 9 | -1.053285404 | hsa-mir-7977 | - |
| hsa-mir-924 | HGLibA_64285 | 9 | -1.053285404 | hsa-mir-924 | - |
| KIAA1199 | HGLibA_24710 | 9 | -1.053285404 | KIAA1199 | - |
| LOC100129480 | HGLibA_26761 | 9 | -1.053285404 | LOC100129480 | - |
| SPATA31A2 | HGLibA_46470 | 9 | -1.053285404 | SPATA31A2 | - |
| TMEM194A | HGLibA_50154 | 9 | -1.053285404 | TMEM194A | - |
| TWISTNB | HGLibA_52380 | 9 | -1.053285404 | TWISTNB | - |
| ATP5A1 | HGLibA_03637 | 8 | -1.062330747 | ATP5A1 | - |
| C14orf182 | HGLibA_05351 | 8 | -1.062330747 | C14orf182 | - |
| C17orf70 | HGLibA_05564 | 8 | -1.062330747 | C17orf70 | - |
| C19orf10 | HGLibA_05627 | 8 | -1.062330747 | C19orf10 | - |
| C1orf227 | HGLibA_05838 | 8 | -1.062330747 | C1orf227 | - |
| C3orf83 | HGLibA_06274 | 8 | -1.062330747 | C3orf83 | - |
| C6orf70 | HGLibA_06509 | 8 | -1.062330747 | C6orf70 | - |
| C7orf10 | HGLibA_06522 | 8 | -1.062330747 | C7orf10 | - |
| ENSG00000015592 | HGLibA_47526 | 8 | -1.062330747 | STMN4 | stathmin 4 [Source:HGNC Symbol;Acc:HGNC:16078] |
| ENSG00000048162 | HGLibA_32308 | 8 | -1.062330747 | NOP16 | NOP16 nucleolar protein [Source:HGNC Symbol;Acc:HGNC:26934] |
| ENSG00000058673 | HGLibA_54948 | 8 | -1.062330747 | ZC3H11A | zinc finger CCCH-type containing 11A [Source:HGNC Symbol;Acc:HGNC:29093] |
| ENSG00000074800 | HGLibA_15140 | 8 | -1.062330747 | ENO1 | enolase 1 [Source:HGNC Symbol;Acc:HGNC:3350] |
| ENSG00000075975 | HGLibA_29351 | 8 | -1.062330747 | MKRN2 | makorin ring finger protein 2 [Source:HGNC Symbol;Acc:HGNC:7113] |
| ENSG00000077713 | HGLibA_44744 | 8 | -1.062330747 | SLC25A43 | solute carrier family 25 member 43 [Source:HGNC Symbol;Acc:HGNC:30557] |
| ENSG00000080166 | HGLibA_12537 | 8 | -1.062330747 | DCT | dopachrome tautomerase [Source:HGNC Symbol;Acc:HGNC:2709] |
| ENSG00000081803 | HGLibA_06981 | 8 | -1.062330747 | CADPS2 | calcium dependent secretion activator 2 [Source:HGNC Symbol;Acc:HGNC:16018] |
| ENSG00000083642 | HGLibA_35946 | 8 | -1.062330747 | PDS5B | PDS5 cohesin associated factor B [Source:HGNC Symbol;Acc:HGNC:20418] |
| ENSG00000087053 | HGLibA_30425 | 8 | -1.062330747 | MTMR2 | myotubularin related protein 2 [Source:HGNC Symbol;Acc:HGNC:7450] |
| ENSG00000089063 | HGLibA_50255 | 8 | -1.062330747 | TMEM230 | transmembrane protein 230 [Source:HGNC Symbol;Acc:HGNC:15876] |
| ENSG00000089472 | HGLibA_21144 | 8 | -1.062330747 | HEPH | hephaestin [Source:HGNC Symbol;Acc:HGNC:4866] |
| ENSG00000099977 | HGLibA_12642 | 8 | -1.062330747 | DDT | D-dopachrome tautomerase [Source:HGNC Symbol;Acc:HGNC:2732] |
| ENSG00000100346 | HGLibA_06899 | 8 | -1.062330747 | CACNA1I | calcium voltage-gated channel subunit alpha1 I [Source:HGNC Symbol;Acc:HGNC:1396] |
| ENSG00000101236 | HGLibA_41651 | 8 | -1.062330747 | RNF24 | ring finger protein 24 [Source:HGNC Symbol;Acc:HGNC:13779] |
| ENSG00000101298 | HGLibA_45934 | 8 | -1.062330747 | SNPH | syntaphilin [Source:HGNC Symbol;Acc:HGNC:15931] |
| ENSG00000103978 | HGLibA_50530 | 8 | -1.062330747 | TMEM87A | transmembrane protein 87A [Source:HGNC Symbol;Acc:HGNC:24522] |
| ENSG00000104859 | HGLibA_09799 | 8 | -1.062330747 | CLASRP | CLK4 associating serine/arginine rich protein [Source:HGNC Symbol;Acc:HGNC:17731] |
| ENSG00000107831 | HGLibA_17420 | 8 | -1.062330747 | FGF8 | fibroblast growth factor 8 [Source:HGNC Symbol;Acc:HGNC:3686] |
| ENSG00000107929 | HGLibA_26038 | 8 | -1.062330747 | LARP4B | La ribonucleoprotein domain family member 4B [Source:HGNC Symbol;Acc:HGNC:28987] |
| ENSG00000108465 | HGLibA_08788 | 8 | -1.062330747 | CDK5RAP3 | CDK5 regulatory subunit associated protein 3 [Source:HGNC Symbol;Acc:HGNC:18673] |
| ENSG00000108479 | HGLibA_18515 | 8 | -1.062330747 | GALK1 | galactokinase 1 [Source:HGNC Symbol;Acc:HGNC:4118] |
| ENSG00000108510 | HGLibA_28748 | 8 | -1.062330747 | MED13 | mediator complex subunit 13 [Source:HGNC Symbol;Acc:HGNC:22474] |
| ENSG00000108587 | HGLibA_19685 | 8 | -1.062330747 | GOSR1 | golgi SNAP receptor complex member 1 [Source:HGNC Symbol;Acc:HGNC:4430] |
| ENSG00000109332 | HGLibA_52588 | 8 | -1.062330747 | UBE2D3 | ubiquitin conjugating enzyme E2 D3 [Source:HGNC Symbol;Acc:HGNC:12476] |
| ENSG00000109832 | HGLibA_12680 | 8 | -1.062330747 | DDX25 | DEAD-box helicase 25 [Source:HGNC Symbol;Acc:HGNC:18698] |
| ENSG00000111981 | HGLibA_52960 | 8 | -1.062330747 | ULBP1 | UL16 binding protein 1 [Source:HGNC Symbol;Acc:HGNC:14893] |
| ENSG00000112640 | HGLibA_38179 | 8 | -1.062330747 | PPP2R5D | protein phosphatase 2 regulatory subunit B'delta [Source:HGNC Symbol;Acc:HGNC:9312] |
| ENSG00000113638 | HGLibA_52116 | 8 | -1.062330747 | TTC33 | tetratricopeptide repeat domain 33 [Source:HGNC Symbol;Acc:HGNC:29959] |
| ENSG00000115353 | HGLibA_48171 | 8 | -1.062330747 | TACR1 | tachykinin receptor 1 [Source:HGNC Symbol;Acc:HGNC:11526] |
| ENSG00000117569 | HGLibA_39254 | 8 | -1.062330747 | PTBP2 | polypyrimidine tract binding protein 2 [Source:HGNC Symbol;Acc:HGNC:17662] |
| ENSG00000117697 | HGLibA_32760 | 8 | -1.062330747 | NSL1 | MIS12 kinetochore complex component [Source:HGNC Symbol;Acc:HGNC:24548]" |
| ENSG00000118762 | HGLibA_36799 | 8 | -1.062330747 | PKD2 | transient receptor potential cation channel [Source:HGNC Symbol;Acc:HGNC:9009]" |
| ENSG00000119042 | HGLibA_42780 | 8 | -1.062330747 | SATB2 | SATB homeobox 2 [Source:HGNC Symbol;Acc:HGNC:21637] |
| ENSG00000119042 | HGLibA_42778 | 8 | -1.062330747 | SATB2 | SATB homeobox 2 [Source:HGNC Symbol;Acc:HGNC:21637] |
| ENSG00000122008 | HGLibA_37535 | 8 | -1.062330747 | POLK | DNA polymerase kappa [Source:HGNC Symbol;Acc:HGNC:9183] |
| ENSG00000123091 | HGLibA_41464 | 8 | -1.062330747 | RNF11 | ring finger protein 11 [Source:HGNC Symbol;Acc:HGNC:10056] |
| ENSG00000130204 | HGLibA_50967 | 8 | -1.062330747 | TOMM40 | translocase of outer mitochondrial membrane 40 [Source:HGNC Symbol;Acc:HGNC:18001] |
| ENSG00000134900 | HGLibA_51163 | 8 | -1.062330747 | TPP2 | tripeptidyl peptidase 2 [Source:HGNC Symbol;Acc:HGNC:12016] |
| ENSG00000135248 | HGLibA_16700 | 8 | -1.062330747 | FAM71F1 | family with sequence similarity 71 member F1 [Source:HGNC Symbol;Acc:HGNC:30704] |
| ENSG00000137288 | HGLibA_53100 | 8 | -1.062330747 | UQCC2 | ubiquinol-cytochrome c reductase complex assembly factor 2 [Source:HGNC Symbol;Acc:HGNC:21237] |
| ENSG00000137959 | HGLibA_22614 | 8 | -1.062330747 | IFI44L | interferon induced protein 44 like [Source:HGNC Symbol;Acc:HGNC:17817] |
| ENSG00000137996 | HGLibA_42371 | 8 | -1.062330747 | RTCA | RNA 3'-terminal phosphate cyclase [Source:HGNC Symbol;Acc:HGNC:17981] |
| ENSG00000138385 | HGLibA_47112 | 8 | -1.062330747 | SSB | Sjogren syndrome antigen B [Source:HGNC Symbol;Acc:HGNC:11316] |
| ENSG00000139174 | HGLibA_38471 | 8 | -1.062330747 | PRICKLE1 | prickle planar cell polarity protein 1 [Source:HGNC Symbol;Acc:HGNC:17019] |
| ENSG00000139436 | HGLibA_19132 | 8 | -1.062330747 | GIT2 | GIT ArfGAP 2 [Source:HGNC Symbol;Acc:HGNC:4273] |
| ENSG00000141748 | HGLibA_02980 | 8 | -1.062330747 | ARL5C | ADP ribosylation factor like GTPase 5C [Source:HGNC Symbol;Acc:HGNC:31111] |
| ENSG00000143196 | HGLibA_13901 | 8 | -1.062330747 | DPT | dermatopontin [Source:HGNC Symbol;Acc:HGNC:3011] |
| ENSG00000143318 | HGLibA_07334 | 8 | -1.062330747 | CASQ1 | calsequestrin 1 [Source:HGNC Symbol;Acc:HGNC:1512] |
| ENSG00000143382 | HGLibA_00857 | 8 | -1.062330747 | ADAMTSL4 | ADAMTS like 4 [Source:HGNC Symbol;Acc:HGNC:19706] |
| ENSG00000143401 | HGLibA_02206 | 8 | -1.062330747 | ANP32E | acidic nuclear phosphoprotein 32 family member E [Source:HGNC Symbol;Acc:HGNC:16673] |
| ENSG00000143502 | HGLibA_47854 | 8 | -1.062330747 | SUSD4 | sushi domain containing 4 [Source:HGNC Symbol;Acc:HGNC:25470] |
| ENSG00000147124 | HGLibA_55932 | 8 | -1.062330747 | ZNF41 | zinc finger protein 41 [Source:HGNC Symbol;Acc:HGNC:13107] |
| ENSG00000148187 | HGLibA_30116 | 8 | -1.062330747 | MRRF | mitochondrial ribosome recycling factor [Source:HGNC Symbol;Acc:HGNC:7234] |
| ENSG00000149403 | HGLibA_20290 | 8 | -1.062330747 | GRIK4 | glutamate ionotropic receptor kainate type subunit 4 [Source:HGNC Symbol;Acc:HGNC:4582] |
| ENSG00000149792 | HGLibA_30006 | 8 | -1.062330747 | MRPL49 | mitochondrial ribosomal protein L49 [Source:HGNC Symbol;Acc:HGNC:1176] |
| ENSG00000151005 | HGLibA_49592 | 8 | -1.062330747 | TKTL2 | transketolase like 2 [Source:HGNC Symbol;Acc:HGNC:25313] |
| ENSG00000154118 | HGLibA_23986 | 8 | -1.062330747 | JPH3 | junctophilin 3 [Source:HGNC Symbol;Acc:HGNC:14203] |
| ENSG00000154265 | HGLibA_00115 | 8 | -1.062330747 | ABCA5 | ATP binding cassette subfamily A member 5 [Source:HGNC Symbol;Acc:HGNC:35] |
| ENSG00000154478 | HGLibA_20008 | 8 | -1.062330747 | GPR26 | G protein-coupled receptor 26 [Source:HGNC Symbol;Acc:HGNC:4481] |
| ENSG00000155363 | HGLibA_29694 | 8 | -1.062330747 | MOV10 | Mov10 RISC complex RNA helicase [Source:HGNC Symbol;Acc:HGNC:7200] |
| ENSG00000158571 | HGLibA_36152 | 8 | -1.062330747 | PFKFB1 | 6-biphosphatase 1 [Source:HGNC Symbol;Acc:HGNC:8872]" |
| ENSG00000159166 | HGLibA_25924 | 8 | -1.062330747 | LAD1 | ladinin 1 [Source:HGNC Symbol;Acc:HGNC:6472] |
| ENSG00000159259 | HGLibA_09349 | 8 | -1.062330747 | CHAF1B | chromatin assembly factor 1 subunit B [Source:HGNC Symbol;Acc:HGNC:1911] |
| ENSG00000159445 | HGLibA_49339 | 8 | -1.062330747 | THEM4 | thioesterase superfamily member 4 [Source:HGNC Symbol;Acc:HGNC:17947] |
| ENSG00000160131 | HGLibA_53685 | 8 | -1.062330747 | VMA21 | vacuolar ATPase assembly factor [Source:HGNC Symbol;Acc:HGNC:22082]" |
| ENSG00000160161 | HGLibA_09711 | 8 | -1.062330747 | CILP2 | cartilage intermediate layer protein 2 [Source:HGNC Symbol;Acc:HGNC:24213] |
| ENSG00000163098 | HGLibA_04529 | 8 | -1.062330747 | BIRC8 | baculoviral IAP repeat containing 8 [Source:HGNC Symbol;Acc:HGNC:14878] |
| ENSG00000163705 | HGLibA_16843 | 8 | -1.062330747 | FANCD2OS | FANCD2 opposite strand [Source:HGNC Symbol;Acc:HGNC:28623] |
| ENSG00000164051 | HGLibA_07797 | 8 | -1.062330747 | CCDC51 | coiled-coil domain containing 51 [Source:HGNC Symbol;Acc:HGNC:25714] |
| ENSG00000164307 | HGLibA_15405 | 8 | -1.062330747 | ERAP1 | endoplasmic reticulum aminopeptidase 1 [Source:HGNC Symbol;Acc:HGNC:18173] |
| ENSG00000164535 | HGLibA_12297 | 8 | -1.062330747 | DAGLB | diacylglycerol lipase beta [Source:HGNC Symbol;Acc:HGNC:28923] |
| ENSG00000164651 | HGLibA_46315 | 8 | -1.062330747 | SP8 | Sp8 transcription factor [Source:HGNC Symbol;Acc:HGNC:19196] |
| ENSG00000164695 | HGLibA_09493 | 8 | -1.062330747 | CHMP4C | charged multivesicular body protein 4C [Source:HGNC Symbol;Acc:HGNC:30599] |
| ENSG00000165283 | HGLibA_47538 | 8 | -1.062330747 | STOML2 | stomatin like 2 [Source:HGNC Symbol;Acc:HGNC:14559] |
| ENSG00000165526 | HGLibA_42215 | 8 | -1.062330747 | RPUSD4 | RNA pseudouridine synthase D4 [Source:HGNC Symbol;Acc:HGNC:25898] |
| ENSG00000166313 | HGLibA_02377 | 8 | -1.062330747 | APBB1 | amyloid beta precursor protein binding family B member 1 [Source:HGNC Symbol;Acc:HGNC:581] |
| ENSG00000166435 | HGLibA_54610 | 8 | -1.062330747 | XRRA1 | X-ray radiation resistance associated 1 [Source:HGNC Symbol;Acc:HGNC:18868] |
| ENSG00000166452 | HGLibA_01453 | 8 | -1.062330747 | AKIP1 | A-kinase interacting protein 1 [Source:HGNC Symbol;Acc:HGNC:1170] |
| ENSG00000167281 | HGLibA_40461 | 8 | -1.062330747 | RBFOX3 | RNA binding fox-1 homolog 3 [Source:HGNC Symbol;Acc:HGNC:27097] |
| ENSG00000168484 | HGLibA_43775 | 8 | -1.062330747 | SFTPC | surfactant protein C [Source:HGNC Symbol;Acc:HGNC:10802] |
| ENSG00000168566 | HGLibA_45957 | 8 | -1.062330747 | SNRNP48 | small nuclear ribonucleoprotein U11/U12 subunit 48 [Source:HGNC Symbol;Acc:HGNC:21368] |
| ENSG00000171202 | HGLibA_49909 | 8 | -1.062330747 | TMEM126A | transmembrane protein 126A [Source:HGNC Symbol;Acc:HGNC:25382] |
| ENSG00000171560 | HGLibA_17336 | 8 | -1.062330747 | FGA | fibrinogen alpha chain [Source:HGNC Symbol;Acc:HGNC:3661] |
| ENSG00000171560 | HGLibA_17337 | 8 | -1.062330747 | FGA | fibrinogen alpha chain [Source:HGNC Symbol;Acc:HGNC:3661] |
| ENSG00000171867 | HGLibA_38644 | 8 | -1.062330747 | PRNP | prion protein [Source:HGNC Symbol;Acc:HGNC:9449] |
| ENSG00000171953 | HGLibA_03802 | 8 | -1.062330747 | ATPAF2 | ATP synthase mitochondrial F1 complex assembly factor 2 [Source:HGNC Symbol;Acc:HGNC:18802] |
| ENSG00000173611 | HGLibA_42833 | 8 | -1.062330747 | SCAI | suppressor of cancer cell invasion [Source:HGNC Symbol;Acc:HGNC:26709] |
| ENSG00000174667 | HGLibA_34476 | 8 | -1.062330747 | OR7D4 | olfactory receptor family 7 subfamily D member 4 [Source:HGNC Symbol;Acc:HGNC:8380] |
| ENSG00000175073 | HGLibA_53577 | 8 | -1.062330747 | VCPIP1 | valosin containing protein interacting protein 1 [Source:HGNC Symbol;Acc:HGNC:30897] |
| ENSG00000175155 | HGLibA_54708 | 8 | -1.062330747 | YPEL2 | yippee like 2 [Source:HGNC Symbol;Acc:HGNC:18326] |
| ENSG00000175544 | HGLibA_06855 | 8 | -1.062330747 | CABP4 | calcium binding protein 4 [Source:HGNC Symbol;Acc:HGNC:1386] |
| ENSG00000176136 | HGLibA_28554 | 8 | -1.062330747 | MC5R | melanocortin 5 receptor [Source:HGNC Symbol;Acc:HGNC:6933] |
| ENSG00000177352 | HGLibA_07852 | 8 | -1.062330747 | CCDC71 | coiled-coil domain containing 71 [Source:HGNC Symbol;Acc:HGNC:25760] |
| ENSG00000177700 | HGLibA_37604 | 8 | -1.062330747 | POLR2L | RNA polymerase II subunit L [Source:HGNC Symbol;Acc:HGNC:9199] |
| ENSG00000178358 | HGLibA_33786 | 8 | -1.062330747 | OR2D3 | olfactory receptor family 2 subfamily D member 3 [Source:HGNC Symbol;Acc:HGNC:15146] |
| ENSG00000178935 | HGLibA_56218 | 8 | -1.062330747 | ZNF552 | zinc finger protein 552 [Source:HGNC Symbol;Acc:HGNC:26135] |
| ENSG00000179091 | HGLibA_12025 | 8 | -1.062330747 | CYC1 | cytochrome c1 [Source:HGNC Symbol;Acc:HGNC:2579] |
| ENSG00000181693 | HGLibA_34525 | 8 | -1.062330747 | OR8H1 | olfactory receptor family 8 subfamily H member 1 [Source:HGNC Symbol;Acc:HGNC:14824] |
| ENSG00000182774 | HGLibA_42085 | 8 | -1.062330747 | RPS17 | ribosomal protein S17 [Source:HGNC Symbol;Acc:HGNC:10397] |
| ENSG00000183434 | HGLibA_49158 | 8 | -1.062330747 | TFDP3 | transcription factor Dp family member 3 [Source:HGNC Symbol;Acc:HGNC:24603] |
| ENSG00000183741 | HGLibA_07459 | 8 | -1.062330747 | CBX6 | chromobox 6 [Source:HGNC Symbol;Acc:HGNC:1556] |
| ENSG00000186766 | HGLibA_17878 | 8 | -1.062330747 | FOXI2 | forkhead box I2 [Source:HGNC Symbol;Acc:HGNC:32448] |
| ENSG00000187026 | HGLibA_25710 | 8 | -1.062330747 | KRTAP21-2 | keratin associated protein 21-2 [Source:HGNC Symbol;Acc:HGNC:18946] |
| ENSG00000187079 | HGLibA_48925 | 8 | -1.062330747 | TEAD1 | TEA domain transcription factor 1 [Source:HGNC Symbol;Acc:HGNC:11714] |
| ENSG00000187514 | HGLibA_39391 | 8 | -1.062330747 | PTMA | prothymosin alpha [Source:HGNC Symbol;Acc:HGNC:9623] |
| ENSG00000188092 | HGLibA_20111 | 8 | -1.062330747 | GPR89B | G protein-coupled receptor 89B [Source:HGNC Symbol;Acc:HGNC:13840] |
| ENSG00000188133 | HGLibA_50220 | 8 | -1.062330747 | TMEM215 | transmembrane protein 215 [Source:HGNC Symbol;Acc:HGNC:33816] |
| ENSG00000196126 | HGLibA_21635 | 8 | -1.062330747 | HLA-DRB1 | DR beta 1 [Source:HGNC Symbol;Acc:HGNC:4948]" |
| ENSG00000196748 | HGLibA_10109 | 8 | -1.062330747 | CLPSL2 | colipase like 2 [Source:HGNC Symbol;Acc:HGNC:21250] |
| ENSG00000197471 | HGLibA_46730 | 8 | -1.062330747 | SPN | sialophorin [Source:HGNC Symbol;Acc:HGNC:11249] |

| | | | | | |
|---|---|---|---|---|---|
| ENSG00000197498 | HGLibA_41833 | 8 | -1.062330747 | RPF2 | ribosome production factor 2 homolog [Source:HGNC Symbol;Acc:HGNC:20870] |
| ENSG00000203872 | HGLibA_06456 | 8 | -1.062330747 | C6orf163 | chromosome 6 open reading frame 163 [Source:HGNC Symbol;Acc:HGNC:21403] |
| ENSG00000203908 | HGLibA_24562 | 8 | -1.062330747 | KHDC3L | subcortical maternal complex member [Source:HGNC Symbol;Acc:HGNC:33699]" |
| ENSG00000204001 | HGLibA_26184 | 8 | -1.062330747 | LCN8 | lipocalin 8 [Source:HGNC Symbol;Acc:HGNC:27038] |
| ENSG00000204946 | HGLibA_56743 | 8 | -1.062330747 | ZNF783 | zinc finger family member 783 [Source:HGNC Symbol;Acc:HGNC:27222] |
| ENSG00000205250 | HGLibA_14316 | 8 | -1.062330747 | E2F4 | E2F transcription factor 4 [Source:HGNC Symbol;Acc:HGNC:3118] |
| ENSG00000211584 | HGLibA_45198 | 8 | -1.062330747 | SLC48A1 | solute carrier family 48 member 1 [Source:HGNC Symbol;Acc:HGNC:26035] |
| ENSG00000212123 | HGLibA_38817 | 8 | -1.062330747 | PRR22 | proline rich 22 [Source:HGNC Symbol;Acc:HGNC:28354] |
| ENSG00000221858 | HGLibA_33721 | 8 | -1.062330747 | OR2A12 | olfactory receptor family 2 subfamily A member 12 [Source:HGNC Symbol;Acc:HGNC:15082] |
| ENSG00000240038 | HGLibA_01854 | 8 | -1.062330747 | AMY2B | alpha 2B (pancreatic) [Source:HGNC Symbol;Acc:HGNC:478]" |
| ENSG00000241476 | HGLibA_47199 | 8 | -1.062330747 | SSX2 | SSX family member 2 [Source:HGNC Symbol;Acc:HGNC:11336] |
| ENSG00000254550 | HGLibA_33389 | 8 | -1.062330747 | OMP | olfactory marker protein [Source:HGNC Symbol;Acc:HGNC:8136] |
| ENSG00000268447 | HGLibA_47202 | 8 | -1.062330747 | SSX2B | SSX family member 2B [Source:HGNC Symbol;Acc:HGNC:22263] |
| FAM150B | HGLibA_16158 | 8 | -1.062330747 | FAM150B | - |
| FAM92B | HGLibA_16801 | 8 | -1.062330747 | FAM92B | - |
| HIST2H2AA3 | HGLibA_21521 | 8 | -1.062330747 | HIST2H2AA3 | - |
| hsa-let-7d | HGLibA_57119 | 8 | -1.062330747 | hsa-let-7d | - |
| hsa-mir-187 | HGLibA_58010 | 8 | -1.062330747 | hsa-mir-187 | - |
| hsa-mir-26a-1 | HGLibA_58365 | 8 | -1.062330747 | hsa-mir-26a-1 | - |
| hsa-mir-26a-2 | HGLibA_58367 | 8 | -1.062330747 | hsa-mir-26a-2 | - |
| hsa-mir-3065 | HGLibA_58462 | 8 | -1.062330747 | hsa-mir-3065 | - |
| hsa-mir-3175 | HGLibA_58774 | 8 | -1.062330747 | hsa-mir-3175 | - |
| hsa-mir-4264 | HGLibA_59765 | 8 | -1.062330747 | hsa-mir-4264 | - |
| hsa-mir-4300 | HGLibA_59911 | 8 | -1.062330747 | hsa-mir-4300 | - |
| hsa-mir-4307 | HGLibA_59938 | 8 | -1.062330747 | hsa-mir-4307 | - |
| hsa-mir-4512 | HGLibA_60491 | 8 | -1.062330747 | hsa-mir-4512 | - |
| hsa-mir-4682 | HGLibA_60837 | 8 | -1.062330747 | hsa-mir-4682 | - |
| hsa-mir-5095 | HGLibA_61537 | 8 | -1.062330747 | hsa-mir-5095 | - |
| hsa-mir-513a-2 | HGLibA_61573 | 8 | -1.062330747 | hsa-mir-513a-2 | - |
| hsa-mir-520b | HGLibA_61738 | 8 | -1.062330747 | hsa-mir-520b | - |
| hsa-mir-521-2 | HGLibA_61769 | 8 | -1.062330747 | hsa-mir-521-2 | - |
| hsa-mir-548i-4 | HGLibA_62024 | 8 | -1.062330747 | hsa-mir-548i-4 | - |
| hsa-mir-5692c-1 | HGLibA_62300 | 8 | -1.062330747 | hsa-mir-5692c-1 | - |
| hsa-mir-604 | HGLibA_62507 | 8 | -1.062330747 | hsa-mir-604 | - |
| hsa-mir-6806 | HGLibA_63401 | 8 | -1.062330747 | hsa-mir-6806 | - |
| hsa-mir-7-3 | HGLibA_63778 | 8 | -1.062330747 | hsa-mir-7-3 | - |
| hsa-mir-920 | HGLibA_64274 | 8 | -1.062330747 | hsa-mir-920 | - |
| hsa-mir-938 | HGLibA_64325 | 8 | -1.062330747 | hsa-mir-938 | - |
| IL3RA_Y | HGLibA_23162 | 8 | -1.062330747 | IL3RA_Y | - |
| PRSS46 | HGLibA_38947 | 8 | -1.062330747 | PRSS46 | - |
| RPS17L | HGLibA_42088 | 8 | -1.062330747 | RPS17L | - |
| USP17L1P | HGLibA_53239 | 8 | -1.062330747 | USP17L1P | - |
| C11orf74 | HGLibA_05184 | 7 | -1.071376089 | C11orf74 | - |
| C1orf51 | HGLibA_05863 | 7 | -1.071376089 | C1orf51 | - |
| C1orf86 | HGLibA_05900 | 7 | -1.071376089 | C1orf86 | - |
| C9orf123 | HGLibA_06672 | 7 | -1.071376089 | C9orf123 | - |
| C9orf142 | HGLibA_06687 | 7 | -1.071376089 | C9orf142 | - |
| CCDC147 | HGLibA_07632 | 7 | -1.071376089 | CCDC147 | - |
| ENSG00000012817 | HGLibA_24529 | 7 | -1.071376089 | KDM5D | lysine demethylase 5D [Source:HGNC Symbol;Acc:HGNC:11115] |
| ENSG00000018699 | HGLibA_52093 | 7 | -1.071376089 | TTC27 | tetratricopeptide repeat domain 27 [Source:HGNC Symbol;Acc:HGNC:25986] |
| ENSG00000024862 | HGLibA_07746 | 7 | -1.071376089 | CCDC28A | coiled-coil domain containing 28A [Source:HGNC Symbol;Acc:HGNC:21098] |
| ENSG00000027075 | HGLibA_38550 | 7 | -1.071376089 | PRKCH | protein kinase C eta [Source:HGNC Symbol;Acc:HGNC:9403] |
| ENSG00000033867 | HGLibA_45226 | 7 | -1.071376089 | SLC4A7 | solute carrier family 4 member 7 [Source:HGNC Symbol;Acc:HGNC:11033] |
| ENSG00000039537 | HGLibA_06426 | 7 | -1.071376089 | C6 | complement C6 [Source:HGNC Symbol;Acc:HGNC:1339] |
| ENSG00000047662 | HGLibA_16329 | 7 | -1.071376089 | FAM184B | family with sequence similarity 184 member B [Source:HGNC Symbol;Acc:HGNC:29235] |
| ENSG00000058729 | HGLibA_41309 | 7 | -1.071376089 | RIOK2 | RIO kinase 2 [Source:HGNC Symbol;Acc:HGNC:18999] |
| ENSG00000066279 | HGLibA_03360 | 7 | -1.071376089 | ASPM | abnormal spindle microtubule assembly [Source:HGNC Symbol;Acc:HGNC:19048] |
| ENSG00000074966 | HGLibA_52388 | 7 | -1.071376089 | TXK | TXK tyrosine kinase [Source:HGNC Symbol;Acc:HGNC:12434] |
| ENSG00000087299 | HGLibA_25890 | 7 | -1.071376089 | L2HGDH | L-2-hydroxyglutarate dehydrogenase [Source:HGNC Symbol;Acc:HGNC:20499] |
| ENSG00000090402 | HGLibA_44100 | 7 | -1.071376089 | SI | sucrase-isomaltase [Source:HGNC Symbol;Acc:HGNC:10856] |
| ENSG00000090432 | HGLibA_30562 | 7 | -1.071376089 | MUL1 | mitochondrial E3 ubiquitin protein ligase 1 [Source:HGNC Symbol;Acc:HGNC:25762] |
| ENSG00000095787 | HGLibA_53938 | 7 | -1.071376089 | WAC | WW domain containing adaptor with coiled-coil [Source:HGNC Symbol;Acc:HGNC:17327] |
| ENSG00000096395 | HGLibA_29426 | 7 | -1.071376089 | MLN | motilin [Source:HGNC Symbol;Acc:HGNC:7141] |
| ENSG00000099949 | HGLibA_27804 | 7 | -1.071376089 | LZTR1 | leucine zipper like transcription regulator 1 [Source:HGNC Symbol;Acc:HGNC:6742] |
| ENSG00000099957 | HGLibA_34862 | 7 | -1.071376089 | P2RX6 | purinergic receptor P2X 6 [Source:HGNC Symbol;Acc:HGNC:8538] |
| ENSG00000100105 | HGLibA_35289 | 7 | -1.071376089 | PATZ1 | POZ/BTB and AT hook containing zinc finger 1 [Source:HGNC Symbol;Acc:HGNC:13071] |
| ENSG00000100505 | HGLibA_51618 | 7 | -1.071376089 | TRIM9 | tripartite motif containing 9 [Source:HGNC Symbol;Acc:HGNC:16288] |
| ENSG00000101346 | HGLibA_37469 | 7 | -1.071376089 | POFUT1 | protein O-fucosyltransferase 1 [Source:HGNC Symbol;Acc:HGNC:14988] |
| ENSG00000101745 | HGLibA_01998 | 7 | -1.071376089 | ANKRD12 | ankyrin repeat domain 12 [Source:HGNC Symbol;Acc:HGNC:29135] |
| ENSG00000102316 | HGLibA_27971 | 7 | -1.071376089 | MAGED2 | MAGE family member D2 [Source:HGNC Symbol;Acc:HGNC:16353] |
| ENSG00000103356 | HGLibA_14344 | 7 | -1.071376089 | EARS2 | mitochondrial [Source:HGNC Symbol;Acc:HGNC:29419]" |
| ENSG00000105607 | HGLibA_18769 | 7 | -1.071376089 | GCDH | glutaryl-CoA dehydrogenase [Source:HGNC Symbol;Acc:HGNC:4189] |
| ENSG00000105708 | HGLibA_55480 | 7 | -1.071376089 | ZNF14 | zinc finger protein 14 [Source:HGNC Symbol;Acc:HGNC:12924] |
| ENSG00000106327 | HGLibA_49194 | 7 | -1.071376089 | TFR2 | transferrin receptor 2 [Source:HGNC Symbol;Acc:HGNC:11762] |
| ENSG00000108551 | HGLibA_40319 | 7 | -1.071376089 | RASD1 | ras related dexamethasone induced 1 [Source:HGNC Symbol;Acc:HGNC:15828] |
| ENSG00000109066 | HGLibA_49848 | 7 | -1.071376089 | TMEM104 | transmembrane protein 104 [Source:HGNC Symbol;Acc:HGNC:25984] |
| ENSG00000109111 | HGLibA_47824 | 7 | -1.071376089 | SUPT6H | histone chaperone [Source:HGNC Symbol;Acc:HGNC:11470]" |
| ENSG00000113734 | HGLibA_04651 | 7 | -1.071376089 | BNIP1 | BCL2 interacting protein 1 [Source:HGNC Symbol;Acc:HGNC:1082] |
| ENSG00000115850 | HGLibA_26204 | 7 | -1.071376089 | LCT | lactase [Source:HGNC Symbol;Acc:HGNC:6530] |
| ENSG00000116127 | HGLibA_01672 | 7 | -1.071376089 | ALMS1 | centrosome and basal body associated protein [Source:HGNC Symbol;Acc:HGNC:428]" |
| ENSG00000117069 | HGLibA_47277 | 7 | -1.071376089 | ST6GALNAC5 | 6-sialyltransferase 5 [Source:HGNC Symbol;Acc:HGNC:19342]" |
| ENSG00000117281 | HGLibA_08239 | 7 | -1.071376089 | CD160 | CD160 molecule [Source:HGNC Symbol;Acc:HGNC:17013] |
| ENSG00000117395 | HGLibA_14370 | 7 | -1.071376089 | EBNA1BP2 | EBNA1 binding protein 2 [Source:HGNC Symbol;Acc:HGNC:15531] |
| ENSG00000120071 | HGLibA_24037 | 7 | -1.071376089 | KANSL1 | KAT8 regulatory NSL complex subunit 1 [Source:HGNC Symbol;Acc:HGNC:24565] |
| ENSG00000120337 | HGLibA_50821 | 7 | -1.071376089 | TNFSF18 | TNF superfamily member 18 [Source:HGNC Symbol;Acc:HGNC:11932] |
| ENSG00000120509 | HGLibA_35969 | 7 | -1.071376089 | PDZD11 | PDZ domain containing 11 [Source:HGNC Symbol;Acc:HGNC:28034] |
| ENSG00000120837 | HGLibA_31858 | 7 | -1.071376089 | NFYB | nuclear transcription factor Y subunit beta [Source:HGNC Symbol;Acc:HGNC:7805] |
| ENSG00000121350 | HGLibA_39702 | 7 | -1.071376089 | PYROXD1 | pyridine nucleotide-disulphide oxidoreductase domain 1 [Source:HGNC Symbol;Acc:HGNC:26162] |
| ENSG00000122970 | HGLibA_22776 | 7 | -1.071376089 | IFT81 | intraflagellar transport 81 [Source:HGNC Symbol;Acc:HGNC:14313] |
| ENSG00000123080 | HGLibA_08843 | 7 | -1.071376089 | CDKN2C | cyclin dependent kinase inhibitor 2C [Source:HGNC Symbol;Acc:HGNC:1789] |
| ENSG00000123219 | HGLibA_09064 | 7 | -1.071376089 | CENPK | centromere protein K [Source:HGNC Symbol;Acc:HGNC:29479] |
| ENSG00000124228 | HGLibA_12688 | 7 | -1.071376089 | DDX27 | DEAD-box helicase 27 [Source:HGNC Symbol;Acc:HGNC:15837] |
| ENSG00000125124 | HGLibA_04214 | 7 | -1.071376089 | BBS2 | Bardet-Biedl syndrome 2 [Source:HGNC Symbol;Acc:HGNC:967] |
| ENSG00000125831 | HGLibA_11447 | 7 | -1.071376089 | CST11 | cystatin 11 [Source:HGNC Symbol;Acc:HGNC:15959] |
| ENSG00000126790 | HGLibA_25893 | 7 | -1.071376089 | L3HYPDH | trans-L-3-hydroxyproline dehydratase [Source:HGNC Symbol;Acc:HGNC:20488] |
| ENSG00000128610 | HGLibA_17319 | 7 | -1.071376089 | FEZF1 | FEZ family zinc finger 1 [Source:HGNC Symbol;Acc:HGNC:22788] |
| ENSG00000129159 | HGLibA_24161 | 7 | -1.071376089 | KCNC1 | potassium voltage-gated channel subfamily C member 1 [Source:HGNC Symbol;Acc:HGNC:6233] |
| ENSG00000130590 | HGLibA_42663 | 7 | -1.071376089 | SAMD10 | sterile alpha motif domain containing 10 [Source:HGNC Symbol;Acc:HGNC:16129] |
| ENSG00000130939 | HGLibA_52694 | 7 | -1.071376089 | UBE4B | ubiquitination factor E4B [Source:HGNC Symbol;Acc:HGNC:12500] |
| ENSG00000134013 | HGLibA_27040 | 7 | -1.071376089 | LOXL2 | lysyl oxidase like 2 [Source:HGNC Symbol;Acc:HGNC:6666] |
| ENSG00000134216 | HGLibA_09443 | 7 | -1.071376089 | CHIA | acidic [Source:HGNC Symbol;Acc:HGNC:17432]" |
| ENSG00000136688 | HGLibA_23152 | 7 | -1.071376089 | IL36G | interleukin 36 gamma [Source:HGNC Symbol;Acc:HGNC:15741] |
| ENSG00000137274 | HGLibA_04705 | 7 | -1.071376089 | BPHL | biphenyl hydrolase like [Source:HGNC Symbol;Acc:HGNC:1094] |
| ENSG00000137747 | HGLibA_50630 | 7 | -1.071376089 | TMPRSS13 | transmembrane serine protease 13 [Source:HGNC Symbol;Acc:HGNC:29808] |
| ENSG00000138303 | HGLibA_03256 | 7 | -1.071376089 | ASCC1 | activating signal cointegrator 1 complex subunit 1 [Source:HGNC Symbol;Acc:HGNC:24268] |
| ENSG00000138311 | HGLibA_55876 | 7 | -1.071376089 | ZNF365 | zinc finger protein 365 [Source:HGNC Symbol;Acc:HGNC:18194] |
| ENSG00000139083 | HGLibA_15664 | 7 | -1.071376089 | ETV6 | ETS variant 6 [Source:HGNC Symbol;Acc:HGNC:3495] |
| ENSG00000139915 | HGLibA_28678 | 7 | -1.071376089 | MDGA2 | MAM domain containing glycosylphosphatidylinositol anchor 2 [Source:HGNC Symbol;Acc:HGNC:19835] |
| ENSG00000141027 | HGLibA_31356 | 7 | -1.071376089 | NCOR1 | nuclear receptor corepressor 1 [Source:HGNC Symbol;Acc:HGNC:7672] |
| ENSG00000142700 | HGLibA_13430 | 7 | -1.071376089 | DMRTA2 | DMRT like family A2 [Source:HGNC Symbol;Acc:HGNC:13908] |
| ENSG00000143127 | HGLibA_23717 | 7 | -1.071376089 | ITGA10 | integrin subunit alpha 10 [Source:HGNC Symbol;Acc:HGNC:6135] |
| ENSG00000143185 | HGLibA_54495 | 7 | -1.071376089 | XCL2 | X-C motif chemokine ligand 2 [Source:HGNC Symbol;Acc:HGNC:10646] |
| ENSG00000144730 | HGLibA_23012 | 7 | -1.071376089 | IL17RD | interleukin 17 receptor D [Source:HGNC Symbol;Acc:HGNC:17616] |
| ENSG00000145725 | HGLibA_37957 | 7 | -1.071376089 | PPIP5K2 | diphosphoinositol pentakisphosphate kinase 2 [Source:HGNC Symbol;Acc:HGNC:29035] |
| ENSG00000146842 | HGLibA_50203 | 7 | -1.071376089 | TMEM209 | transmembrane protein 209 [Source:HGNC Symbol;Acc:HGNC:21898] |
| ENSG00000147400 | HGLibA_09237 | 7 | -1.071376089 | CETN2 | centrin 2 [Source:HGNC Symbol;Acc:HGNC:1867] |
| ENSG00000147697 | HGLibA_20410 | 7 | -1.071376089 | GSDMC | gasdermin C [Source:HGNC Symbol;Acc:HGNC:7151] |
| ENSG00000153140 | HGLibA_09238 | 7 | -1.071376089 | CETN3 | centrin 3 [Source:HGNC Symbol;Acc:HGNC:1868] |
| ENSG00000155714 | HGLibA_35987 | 7 | -1.071376089 | PDZD9 | PDZ domain containing 9 [Source:HGNC Symbol;Acc:HGNC:28740] |
| ENSG00000156171 | HGLibA_13948 | 7 | -1.071376089 | DRAM2 | DNA damage regulated autophagy modulator 2 [Source:HGNC Symbol;Acc:HGNC:28769] |
| ENSG00000156711 | HGLibA_28260 | 7 | -1.071376089 | MAPK13 | mitogen-activated protein kinase 13 [Source:HGNC Symbol;Acc:HGNC:6875] |
| ENSG00000159256 | HGLibA_29655 | 7 | -1.071376089 | MORC3 | MORC family CW-type zinc finger 3 [Source:HGNC Symbol;Acc:HGNC:23572] |
| ENSG00000159650 | HGLibA_53151 | 7 | -1.071376089 | UROC1 | urocanate hydratase 1 [Source:HGNC Symbol;Acc:HGNC:26444] |
| ENSG00000159658 | HGLibA_14533 | 7 | -1.071376089 | EFCAB14 | EF-hand calcium binding domain 14 [Source:HGNC Symbol;Acc:HGNC:29051] |
| ENSG00000160193 | HGLibA_54084 | 7 | -1.071376089 | WDR4 | WD repeat domain 4 [Source:HGNC Symbol;Acc:HGNC:12756] |
| ENSG00000160284 | HGLibA_46541 | 7 | -1.071376089 | SPATC1L | spermatogenesis and centriole associated 1 like [Source:HGNC Symbol;Acc:HGNC:1298] |
| ENSG00000162426 | HGLibA_45172 | 7 | -1.071376089 | SLC45A1 | solute carrier family 45 member 1 [Source:HGNC Symbol;Acc:HGNC:17939] |
| ENSG00000163660 | HGLibA_08118 | 7 | -1.071376089 | CCNL1 | cyclin L1 [Source:HGNC Symbol;Acc:HGNC:20569] |
| ENSG00000163702 | HGLibA_23009 | 7 | -1.071376089 | IL17RC | interleukin 17 receptor C [Source:HGNC Symbol;Acc:HGNC:18358] |
| ENSG00000164221 | HGLibA_07545 | 7 | -1.071376089 | CCDC112 | coiled-coil domain containing 112 [Source:HGNC Symbol;Acc:HGNC:28599] |
| ENSG00000164442 | HGLibA_09745 | 7 | -1.071376089 | CITED2 | Cbp/p300 interacting transactivator with Glu/Asp rich carboxy-terminal domain 2 [Source:HGNC Symbol;Acc:HGNC:1987] |
| ENSG00000166006 | HGLibA_24164 | 7 | -1.071376089 | KCNC2 | potassium voltage-gated channel subfamily C member 2 [Source:HGNC Symbol;Acc:HGNC:6234] |
| ENSG00000166167 | HGLibA_04986 | 7 | -1.071376089 | BTRC | beta-transducin repeat containing E3 ubiquitin protein ligase [Source:HGNC Symbol;Acc:HGNC:1144] |
| ENSG00000166181 | HGLibA_02420 | 7 | -1.071376089 | API5 | apoptosis inhibitor 5 [Source:HGNC Symbol;Acc:HGNC:594] |
| ENSG00000166347 | HGLibA_11989 | 7 | -1.071376089 | CYB5A | cytochrome b5 type A [Source:HGNC Symbol;Acc:HGNC:2570] |

| | | | | | |
|---|---|---|---|---|---|
| ENSG00000166592 | HGLibA_42221 | 7 | -1.071376089 | RRAD | Ras related glycolysis inhibitor and calcium channel regulator [Source:HGNC Symbol;Acc:HGNC:10446]" |
| ENSG00000166800 | HGLibA_26222 | 7 | -1.071376089 | LDHAL6A | lactate dehydrogenase A like 6A [Source:HGNC Symbol;Acc:HGNC:28335] |
| ENSG00000167711 | HGLibA_43588 | 7 | -1.071376089 | SERPINF2 | serpin family F member 2 [Source:HGNC Symbol;Acc:HGNC:9075] |
| ENSG00000168014 | HGLibA_06092 | 7 | -1.071376089 | C2CD3 | C2 calcium dependent domain containing 3 [Source:HGNC Symbol;Acc:HGNC:24564] |
| ENSG00000168038 | HGLibA_52976 | 7 | -1.071376089 | ULK4 | unc-51 like kinase 4 [Source:HGNC Symbol;Acc:HGNC:15784] |
| ENSG00000168172 | HGLibA_21883 | 7 | -1.071376089 | HOOK3 | hook microtubule tethering protein 3 [Source:HGNC Symbol;Acc:HGNC:23576] |
| ENSG00000168906 | HGLibA_28418 | 7 | -1.071376089 | MAT2A | methionine adenosyltransferase 2A [Source:HGNC Symbol;Acc:HGNC:6904] |
| ENSG00000169989 | HGLibA_49473 | 7 | -1.071376089 | TIGD4 | tigger transposable element derived 4 [Source:HGNC Symbol;Acc:HGNC:18335] |
| ENSG00000170324 | HGLibA_18036 | 7 | -1.071376089 | FRMPD2 | FERM and PDZ domain containing 2 [Source:HGNC Symbol;Acc:HGNC:28572] |
| ENSG00000171863 | HGLibA_42187 | 7 | -1.071376089 | RPS7 | ribosomal protein S7 [Source:HGNC Symbol;Acc:HGNC:10440] |
| ENSG00000172922 | HGLibA_41434 | 7 | -1.071376089 | RNASEH2C | ribonuclease H2 subunit C [Source:HGNC Symbol;Acc:HGNC:24116] |
| ENSG00000173040 | HGLibA_15684 | 7 | -1.071376089 | EVC2 | EvC ciliary complex subunit 2 [Source:HGNC Symbol;Acc:HGNC:19747] |
| ENSG00000175505 | HGLibA_09817 | 7 | -1.071376089 | CLCF1 | cardiotrophin like cytokine factor 1 [Source:HGNC Symbol;Acc:HGNC:17412] |
| ENSG00000175548 | HGLibA_01608 | 7 | -1.071376089 | ALG10B | 2-glucosyltransferase [Source:HGNC Symbol;Acc:HGNC:31088]" |
| ENSG00000176246 | HGLibA_34037 | 7 | -1.071376089 | OR4L1 | olfactory receptor family 4 subfamily L member 1 [Source:HGNC Symbol;Acc:HGNC:15356] |
| ENSG00000181074 | HGLibA_34199 | 7 | -1.071376089 | OR52N4 | olfactory receptor family 52 subfamily N member 4 (gene/pseudogene) [Source:HGNC Symbol;Acc:HGNC:15230] |
| ENSG00000181767 | HGLibA_34528 | 7 | -1.071376089 | OR8H2 | olfactory receptor family 8 subfamily H member 2 [Source:HGNC Symbol;Acc:HGNC:15308] |
| ENSG00000182287 | HGLibA_02300 | 7 | -1.071376089 | AP1S2 | adaptor related protein complex 1 subunit sigma 2 [Source:HGNC Symbol;Acc:HGNC:560] |
| ENSG00000182944 | HGLibA_15714 | 7 | -1.071376089 | EWSR1 | EWS RNA binding protein 1 [Source:HGNC Symbol;Acc:HGNC:3508] |
| ENSG00000183091 | HGLibA_31578 | 7 | -1.071376089 | NEB | nebulin [Source:HGNC Symbol;Acc:HGNC:7720] |
| ENSG00000183643 | HGLibA_05383 | 7 | -1.071376089 | C15orf32 | chromosome 15 open reading frame 32 [Source:HGNC Symbol;Acc:HGNC:26549] |
| ENSG00000184619 | HGLibA_25389 | 7 | -1.071376089 | KRBA2 | KRAB-A domain containing 2 [Source:HGNC Symbol;Acc:HGNC:26989] |
| ENSG00000184840 | HGLibA_49830 | 7 | -1.071376089 | TMED9 | transmembrane p24 trafficking protein 9 [Source:HGNC Symbol;Acc:HGNC:24878] |
| ENSG00000184867 | HGLibA_03041 | 7 | -1.071376089 | ARMCX2 | armadillo repeat containing X-linked 2 [Source:HGNC Symbol;Acc:HGNC:16869] |
| ENSG00000185551 | HGLibA_32614 | 7 | -1.071376089 | NR2F2 | nuclear receptor subfamily 2 group F member 2 [Source:HGNC Symbol;Acc:HGNC:7976] |
| ENSG00000185627 | HGLibA_39161 | 7 | -1.071376089 | PSMD13 | non-ATPase 13 [Source:HGNC Symbol;Acc:HGNC:9558]" |
| ENSG00000186529 | HGLibA_12194 | 7 | -1.071376089 | CYP4F3 | cytochrome P450 family 4 subfamily F member 3 [Source:HGNC Symbol;Acc:HGNC:2646] |
| ENSG00000186665 | HGLibA_05550 | 7 | -1.071376089 | C17orf58 | chromosome 17 open reading frame 58 [Source:HGNC Symbol;Acc:HGNC:27568] |
| ENSG00000187175 | HGLibA_25631 | 7 | -1.071376089 | KRTAP12-1 | keratin associated protein 12-1 [Source:HGNC Symbol;Acc:HGNC:20529] |
| ENSG00000187855 | HGLibA_03276 | 7 | -1.071376089 | ASCL4 | achaete-scute family bHLH transcription factor 4 [Source:HGNC Symbol;Acc:HGNC:24311] |
| ENSG00000188811 | HGLibA_31903 | 7 | -1.071376089 | NHLRC3 | NHL repeat containing 3 [Source:HGNC Symbol;Acc:HGNC:33751] |
| ENSG00000197079 | HGLibA_25489 | 7 | -1.071376089 | KRT35 | keratin 35 [Source:HGNC Symbol;Acc:HGNC:6453] |
| ENSG00000197324 | HGLibA_27201 | 7 | -1.071376089 | LRP10 | LDL receptor related protein 10 [Source:HGNC Symbol;Acc:HGNC:14553] |
| ENSG00000197381 | HGLibA_00871 | 7 | -1.071376089 | ADARB1 | RNA specific B1 [Source:HGNC Symbol;Acc:HGNC:226]" |
| ENSG00000197757 | HGLibA_21974 | 7 | -1.071376089 | HOXC6 | homeobox C6 [Source:HGNC Symbol;Acc:HGNC:5128] |
| ENSG00000204856 | HGLibA_16482 | 7 | -1.071376089 | FAM216A | family with sequence similarity 216 member A [Source:HGNC Symbol;Acc:HGNC:30180] |
| ENSG00000205022 | HGLibA_34945 | 7 | -1.071376089 | PABPN1L | cytoplasmic [Source:HGNC Symbol;Acc:HGNC:37237]" |
| ENSG00000205030 | HGLibA_34325 | 7 | -1.071376089 | OR5L2 | olfactory receptor family 5 subfamily L member 2 [Source:HGNC Symbol;Acc:HGNC:8351] |
| ENSG00000205403 | HGLibA_09278 | 7 | -1.071376089 | CFI | complement factor I [Source:HGNC Symbol;Acc:HGNC:5394] |
| ENSG00000205457 | HGLibA_51083 | 7 | -1.071376089 | TP53TG3C | TP53 target 3C [Source:HGNC Symbol;Acc:HGNC:42962] |
| ENSG00000218336 | HGLibA_49001 | 7 | -1.071376089 | TENM3 | teneurin transmembrane protein 3 [Source:HGNC Symbol;Acc:HGNC:29944] |
| ENSG00000222036 | HGLibA_37748 | 7 | -1.071376089 | POTEM | POTE ankyrin domain family member M [Source:HGNC Symbol;Acc:HGNC:37096] |
| ENSG00000222046 | HGLibA_12488 | 7 | -1.071376089 | DCDC2B | doublecortin domain containing 2B [Source:HGNC Symbol;Acc:HGNC:32576] |
| ENSG00000227488 | HGLibA_18444 | 7 | -1.071376089 | GAGE12D | G antigen 12D [Source:HGNC Symbol;Acc:HGNC:31904] |
| ENSG00000237412 | HGLibA_38964 | 7 | -1.071376089 | PRSS56 | serine protease 56 [Source:HGNC Symbol;Acc:HGNC:39433] |
| ENSG00000239857 | HGLibA_18933 | 7 | -1.071376089 | GET4 | golgi to ER traffic protein 4 [Source:HGNC Symbol;Acc:HGNC:21690] |
| ENSG00000239900 | HGLibA_01073 | 7 | -1.071376089 | ADSL | adenylosuccinate lyase [Source:HGNC Symbol;Acc:HGNC:291] |
| ENSG00000249751 | HGLibA_14417 | 7 | -1.071376089 | ECSCR | endothelial cell surface expressed chemotaxis and apoptosis regulator [Source:HGNC Symbol;Acc:HGNC:35454] |
| ENSG00000258644 | HGLibA_48013 | 7 | -1.071376089 | SYNJ2BP-COX16 | SYNJ2BP-COX16 readthrough [Source:HGNC Symbol;Acc:HGNC:48350] |
| ENSG00000288611 | HGLibA_32411 | 7 | -1.071376089 | NPBWR1 | - |
| GAGE6 | HGLibA_18486 | 7 | -1.071376089 | GAGE6 | - |
| GLTSCR2 | HGLibA_19357 | 7 | -1.071376089 | GLTSCR2 | - |
| H2AFJ | HGLibA_20755 | 7 | -1.071376089 | H2AFJ | - |
| HN1L | HGLibA_21772 | 7 | -1.071376089 | HN1L | - |
| hsa-mir-1298 | HGLibA_57670 | 7 | -1.071376089 | hsa-mir-1298 | - |
| hsa-mir-1538 | HGLibA_57919 | 7 | -1.071376089 | hsa-mir-1538 | - |
| hsa-mir-15b | HGLibA_57943 | 7 | -1.071376089 | hsa-mir-15b | - |
| hsa-mir-199a-2 | HGLibA_58133 | 7 | -1.071376089 | hsa-mir-199a-2 | - |
| hsa-mir-19a | HGLibA_58140 | 7 | -1.071376089 | hsa-mir-19a | - |
| hsa-mir-2909 | HGLibA_58392 | 7 | -1.071376089 | hsa-mir-2909 | - |
| hsa-mir-3147 | HGLibA_58647 | 7 | -1.071376089 | hsa-mir-3147 | - |
| hsa-mir-3663 | HGLibA_59202 | 7 | -1.071376089 | hsa-mir-3663 | - |
| hsa-mir-3673 | HGLibA_59244 | 7 | -1.071376089 | hsa-mir-3673 | - |
| hsa-mir-378f | HGLibA_59450 | 7 | -1.071376089 | hsa-mir-378f | - |
| hsa-mir-3921 | HGLibA_59559 | 7 | -1.071376089 | hsa-mir-3921 | - |
| hsa-mir-429 | HGLibA_59867 | 7 | -1.071376089 | hsa-mir-429 | - |
| hsa-mir-4477a | HGLibA_60318 | 7 | -1.071376089 | hsa-mir-4477a | - |
| hsa-mir-449b | HGLibA_60420 | 7 | -1.071376089 | hsa-mir-449b | - |
| hsa-mir-4641 | HGLibA_60669 | 7 | -1.071376089 | hsa-mir-4641 | - |
| hsa-mir-4655 | HGLibA_60728 | 7 | -1.071376089 | hsa-mir-4655 | - |
| hsa-mir-4791 | HGLibA_61271 | 7 | -1.071376089 | hsa-mir-4791 | - |
| hsa-mir-493 | HGLibA_61372 | 7 | -1.071376089 | hsa-mir-493 | - |
| hsa-mir-513b | HGLibA_61577 | 7 | -1.071376089 | hsa-mir-513b | - |
| hsa-mir-518e | HGLibA_61672 | 7 | -1.071376089 | hsa-mir-518e | - |
| hsa-mir-519c | HGLibA_61724 | 7 | -1.071376089 | hsa-mir-519c | - |
| hsa-mir-5685 | HGLibA_62258 | 7 | -1.071376089 | hsa-mir-5685 | - |
| hsa-mir-6132 | HGLibA_62678 | 7 | -1.071376089 | hsa-mir-6132 | - |
| hsa-mir-647 | HGLibA_62833 | 7 | -1.071376089 | hsa-mir-647 | - |
| hsa-mir-6782 | HGLibA_63306 | 7 | -1.071376089 | hsa-mir-6782 | - |
| hsa-mir-7854 | HGLibA_64006 | 7 | -1.071376089 | hsa-mir-7854 | - |
| KIAA0947 | HGLibA_24673 | 7 | -1.071376089 | KIAA0947 | - |
| KIAA1467 | HGLibA_24766 | 7 | -1.071376089 | KIAA1467 | - |
| MLL4 | HGLibA_29402 | 7 | -1.071376089 | MLL4 | - |
| AGAP7 | HGLibA_01156 | 6 | -1.080421432 | AGAP7 | - |
| ANKRD20A2 | HGLibA_02030 | 6 | -1.080421432 | ANKRD20A2 | - |
| C2orf43 | HGLibA_06127 | 6 | -1.080421432 | C2orf43 | - |
| C9orf170 | HGLibA_06706 | 6 | -1.080421432 | C9orf170 | - |
| ENSG00000005175 | HGLibA_41819 | 6 | -1.080421432 | RPAP3 | RNA polymerase II associated protein 3 [Source:HGNC Symbol;Acc:HGNC:26151] |
| ENSG00000033122 | HGLibA_27414 | 6 | -1.080421432 | LRRC7 | leucine rich repeat containing 7 [Source:HGNC Symbol;Acc:HGNC:18531] |
| ENSG00000035720 | HGLibA_47343 | 6 | -1.080421432 | STAP1 | signal transducing adaptor family member 1 [Source:HGNC Symbol;Acc:HGNC:24133] |
| ENSG00000047346 | HGLibA_16477 | 6 | -1.080421432 | FAM214A | family with sequence similarity 214 member A [Source:HGNC Symbol;Acc:HGNC:25609] |
| ENSG00000072080 | HGLibA_46773 | 6 | -1.080421432 | SPP2 | secreted phosphoprotein 2 [Source:HGNC Symbol;Acc:HGNC:11256] |
| ENSG00000073111 | HGLibA_28604 | 6 | -1.080421432 | MCM2 | minichromosome maintenance complex component 2 [Source:HGNC Symbol;Acc:HGNC:6944] |
| ENSG00000079102 | HGLibA_42459 | 6 | -1.080421432 | RUNX1T1 | RUNX1 translocation partner 1 [Source:HGNC Symbol;Acc:HGNC:1535] |
| ENSG00000088833 | HGLibA_32755 | 6 | -1.080421432 | NSFL1C | NSFL1 cofactor [Source:HGNC Symbol;Acc:HGNC:15912] |
| ENSG00000092621 | HGLibA_36393 | 6 | -1.080421432 | PHGDH | phosphoglycerate dehydrogenase [Source:HGNC Symbol;Acc:HGNC:8923] |
| ENSG00000095739 | HGLibA_04136 | 6 | -1.080421432 | BAMBI | BMP and activin membrane bound inhibitor [Source:HGNC Symbol;Acc:HGNC:30251] |
| ENSG00000099949 | HGLibA_27803 | 6 | -1.080421432 | LZTR1 | leucine zipper like transcription regulator 1 [Source:HGNC Symbol;Acc:HGNC:6742] |
| ENSG00000100075 | HGLibA_44632 | 6 | -1.080421432 | SLC25A1 | solute carrier family 25 member 1 [Source:HGNC Symbol;Acc:HGNC:10979] |
| ENSG00000100802 | HGLibA_05375 | 6 | -1.080421432 | C14orf93 | chromosome 14 open reading frame 93 [Source:HGNC Symbol;Acc:HGNC:20162] |
| ENSG00000102409 | HGLibA_04446 | 6 | -1.080421432 | BEX4 | brain expressed X-linked 4 [Source:HGNC Symbol;Acc:HGNC:25475] |
| ENSG00000102871 | HGLibA_51238 | 6 | -1.080421432 | TRADD | TNFRSF1A associated via death domain [Source:HGNC Symbol;Acc:HGNC:12030] |
| ENSG00000103550 | HGLibA_25344 | 6 | -1.080421432 | KNOP1 | lysine rich nucleolar protein 1 [Source:HGNC Symbol;Acc:HGNC:34404] |
| ENSG00000103707 | HGLibA_30355 | 6 | -1.080421432 | MTFMT | mitochondrial methionyl-tRNA formyltransferase [Source:HGNC Symbol;Acc:HGNC:29666] |
| ENSG00000104221 | HGLibA_04801 | 6 | -1.080421432 | BRF2 | RNA polymerase III transcription initiation factor subunit [Source:HGNC Symbol;Acc:HGNC:17298]" |
| ENSG00000105398 | HGLibA_47757 | 6 | -1.080421432 | SULT2A1 | sulfotransferase family 2A member 1 [Source:HGNC Symbol;Acc:HGNC:11458] |
| ENSG00000106302 | HGLibA_22446 | 6 | -1.080421432 | HYAL4 | hyaluronidase 4 [Source:HGNC Symbol;Acc:HGNC:5323] |
| ENSG00000106331 | HGLibA_35303 | 6 | -1.080421432 | PAX4 | paired box 4 [Source:HGNC Symbol;Acc:HGNC:8618] |
| ENSG00000108312 | HGLibA_52770 | 6 | -1.080421432 | UBTF | upstream binding transcription factor [Source:HGNC Symbol;Acc:HGNC:12511] |
| ENSG00000109272 | HGLibA_36132 | 6 | -1.080421432 | PF4V1 | platelet factor 4 variant 1 [Source:HGNC Symbol;Acc:HGNC:8862] |
| ENSG00000109511 | HGLibA_02223 | 6 | -1.080421432 | ANXA10 | annexin A10 [Source:HGNC Symbol;Acc:HGNC:534] |
| ENSG00000109670 | HGLibA_17154 | 6 | -1.080421432 | FBXW7 | F-box and WD repeat domain containing 7 [Source:HGNC Symbol;Acc:HGNC:16712] |
| ENSG00000110237 | HGLibA_02826 | 6 | -1.080421432 | ARHGEF17 | Rho guanine nucleotide exchange factor 17 [Source:HGNC Symbol;Acc:HGNC:21726] |
| ENSG00000110429 | HGLibA_17064 | 6 | -1.080421432 | FBXO3 | F-box protein 3 [Source:HGNC Symbol;Acc:HGNC:13582] |
| ENSG00000111011 | HGLibA_42363 | 6 | -1.080421432 | RSRC2 | arginine and serine rich coiled-coil 2 [Source:HGNC Symbol;Acc:HGNC:30559] |
| ENSG00000115233 | HGLibA_39164 | 6 | -1.080421432 | PSMD14 | non-ATPase 14 [Source:HGNC Symbol;Acc:HGNC:16889]" |
| ENSG00000117394 | HGLibA_44851 | 6 | -1.080421432 | SLC2A1 | solute carrier family 2 member 1 [Source:HGNC Symbol;Acc:HGNC:11005] |
| ENSG00000119699 | HGLibA_49217 | 6 | -1.080421432 | TGFB3 | transforming growth factor beta 3 [Source:HGNC Symbol;Acc:HGNC:11769] |
| ENSG00000120709 | HGLibA_16640 | 6 | -1.080421432 | FAM53C | family with sequence similarity 53 member C [Source:HGNC Symbol;Acc:HGNC:1336] |
| ENSG00000121057 | HGLibA_01404 | 6 | -1.080421432 | AKAP1 | A-kinase anchoring protein 1 [Source:HGNC Symbol;Acc:HGNC:367] |
| ENSG00000121742 | HGLibA_19171 | 6 | -1.080421432 | GJB6 | gap junction protein beta 6 [Source:HGNC Symbol;Acc:HGNC:4288] |
| ENSG00000122180 | HGLibA_30887 | 6 | -1.080421432 | MYOG | myogenin [Source:HGNC Symbol;Acc:HGNC:7612] |
| ENSG00000122417 | HGLibA_33282 | 6 | -1.080421432 | ODF2L | outer dense fiber of sperm tails 2 like [Source:HGNC Symbol;Acc:HGNC:29225] |
| ENSG00000124613 | HGLibA_55905 | 6 | -1.080421432 | ZNF391 | zinc finger protein 391 [Source:HGNC Symbol;Acc:HGNC:18779] |
| ENSG00000127515 | HGLibA_34458 | 6 | -1.080421432 | OR7A10 | olfactory receptor family 7 subfamily A member 10 [Source:HGNC Symbol;Acc:HGNC:8356] |
| ENSG00000128052 | HGLibA_24541 | 6 | -1.080421432 | KDR | kinase insert domain receptor [Source:HGNC Symbol;Acc:HGNC:6307] |
| ENSG00000130529 | HGLibA_51761 | 6 | -1.080421432 | TRPM4 | transient receptor potential cation channel subfamily M member 4 [Source:HGNC Symbol;Acc:HGNC:17993] |
| ENSG00000130590 | HGLibA_42662 | 6 | -1.080421432 | SAMD10 | sterile alpha motif domain containing 10 [Source:HGNC Symbol;Acc:HGNC:16129] |
| ENSG00000130706 | HGLibA_01070 | 6 | -1.080421432 | ADRM1 | adhesion regulating molecule 1 [Source:HGNC Symbol;Acc:HGNC:15759] |
| ENSG00000132780 | HGLibA_31161 | 6 | -1.080421432 | NASP | nuclear autoantigenic sperm protein [Source:HGNC Symbol;Acc:HGNC:7644] |
| ENSG00000132915 | HGLibA_35806 | 6 | -1.080421432 | PDE6A | phosphodiesterase 6A [Source:HGNC Symbol;Acc:HGNC:8785] |
| ENSG00000133983 | HGLibA_10816 | 6 | -1.080421432 | COX16 | cytochrome c oxidase assembly factor COX16 [Source:HGNC Symbol;Acc:HGNC:20213] |
| ENSG00000135414 | HGLibA_18856 | 6 | -1.080421432 | GDF11 | growth differentiation factor 11 [Source:HGNC Symbol;Acc:HGNC:4216] |
| ENSG00000136699 | HGLibA_45811 | 6 | -1.080421432 | SMPD4 | sphingomyelin phosphodiesterase 4 [Source:HGNC Symbol;Acc:HGNC:32949] |
| ENSG00000136866 | HGLibA_55213 | 6 | -1.080421432 | ZFP37 | ZFP37 zinc finger protein [Source:HGNC Symbol;Acc:HGNC:12863] |

| | | | | | |
|---|---|---|---|---|---|
| ENSG00000137710 | HGLibA_40757 | 6 | -1.080421432 | RDX | radixin [Source:HGNC Symbol;Acc:HGNC:9944] |
| ENSG00000140199 | HGLibA_44373 | 6 | -1.080421432 | SLC12A6 | solute carrier family 12 member 6 [Source:HGNC Symbol;Acc:HGNC:10914] |
| ENSG00000140326 | HGLibA_08504 | 6 | -1.080421432 | CDAN1 | codanin 1 [Source:HGNC Symbol;Acc:HGNC:1713] |
| ENSG00000142632 | HGLibA_02831 | 6 | -1.080421432 | ARHGEF19 | Rho guanine nucleotide exchange factor 19 [Source:HGNC Symbol;Acc:HGNC:26604] |
| ENSG00000143382 | HGLibA_00856 | 6 | -1.080421432 | ADAMTSL4 | ADAMTS like 4 [Source:HGNC Symbol;Acc:HGNC:19706] |
| ENSG00000143776 | HGLibA_08557 | 6 | -1.080421432 | CDC42BPA | CDC42 binding protein kinase alpha [Source:HGNC Symbol;Acc:HGNC:1737] |
| ENSG00000143786 | HGLibA_10291 | 6 | -1.080421432 | CNIH3 | cornichon family AMPA receptor auxiliary protein 3 [Source:HGNC Symbol;Acc:HGNC:26802] |
| ENSG00000145794 | HGLibA_28848 | 6 | -1.080421432 | MEGF10 | multiple EGF like domains 10 [Source:HGNC Symbol;Acc:HGNC:29634] |
| ENSG00000145916 | HGLibA_41386 | 6 | -1.080421432 | RMND5B | required for meiotic nuclear division 5 homolog B [Source:HGNC Symbol;Acc:HGNC:26181] |
| ENSG00000146223 | HGLibA_42000 | 6 | -1.080421432 | RPL7L1 | ribosomal protein L7 like 1 [Source:HGNC Symbol;Acc:HGNC:21370] |
| ENSG00000149506 | HGLibA_56949 | 6 | -1.080421432 | ZP1 | zona pellucida glycoprotein 1 [Source:HGNC Symbol;Acc:HGNC:13187] |
| ENSG00000150867 | HGLibA_36704 | 6 | -1.080421432 | PIP4K2A | phosphatidylinositol-5-phosphate 4-kinase type 2 alpha [Source:HGNC Symbol;Acc:HGNC:8997] |
| ENSG00000152580 | HGLibA_22878 | 6 | -1.080421432 | IGSF10 | immunoglobulin superfamily member 10 [Source:HGNC Symbol;Acc:HGNC:26384] |
| ENSG00000154143 | HGLibA_35114 | 6 | -1.080421432 | PANX3 | pannexin 3 [Source:HGNC Symbol;Acc:HGNC:20573] |
| ENSG00000156110 | HGLibA_00990 | 6 | -1.080421432 | ADK | adenosine kinase [Source:HGNC Symbol;Acc:HGNC:257] |
| ENSG00000157978 | HGLibA_26252 | 6 | -1.080421432 | LDLRAP1 | low density lipoprotein receptor adaptor protein 1 [Source:HGNC Symbol;Acc:HGNC:18640] |
| ENSG00000162763 | HGLibA_27386 | 6 | -1.080421432 | LRRC52 | leucine rich repeat containing 52 [Source:HGNC Symbol;Acc:HGNC:32156] |
| ENSG00000163273 | HGLibA_32504 | 6 | -1.080421432 | NPPC | natriuretic peptide C [Source:HGNC Symbol;Acc:HGNC:7941] |
| ENSG00000163714 | HGLibA_52501 | 6 | -1.080421432 | U2SURP | U2 snRNP associated SURP domain containing [Source:HGNC Symbol;Acc:HGNC:30855] |
| ENSG00000164105 | HGLibA_42721 | 6 | -1.080421432 | SAP30 | Sin3A associated protein 30 [Source:HGNC Symbol;Acc:HGNC:10532] |
| ENSG00000166351 | HGLibA_37727 | 6 | -1.080421432 | POTED | POTE ankyrin domain family member D [Source:HGNC Symbol;Acc:HGNC:23822] |
| ENSG00000166963 | HGLibA_28116 | 6 | -1.080421432 | MAP1A | microtubule associated protein 1A [Source:HGNC Symbol;Acc:HGNC:6835] |
| ENSG00000167971 | HGLibA_07288 | 6 | -1.080421432 | CASKIN1 | CASK interacting protein 1 [Source:HGNC Symbol;Acc:HGNC:20879] |
| ENSG00000168477 | HGLibA_50931 | 6 | -1.080421432 | TNXB | tenascin XB [Source:HGNC Symbol;Acc:HGNC:11976] |
| ENSG00000168542 | HGLibA_10564 | 6 | -1.080421432 | COL3A1 | collagen type III alpha 1 chain [Source:HGNC Symbol;Acc:HGNC:2201] |
| ENSG00000170340 | HGLibA_03985 | 6 | -1.080421432 | B3GNT2 | 3-N-acetylglucosaminyltransferase 2 [Source:HGNC Symbol;Acc:HGNC:15629]" |
| ENSG00000171509 | HGLibA_42495 | 6 | -1.080421432 | RXFP1 | relaxin family peptide receptor 1 [Source:HGNC Symbol;Acc:HGNC:19718] |
| ENSG00000171596 | HGLibA_32213 | 6 | -1.080421432 | NMUR1 | neuromedin U receptor 1 [Source:HGNC Symbol;Acc:HGNC:4518] |
| ENSG00000173598 | HGLibA_32978 | 6 | -1.080421432 | NUDT4 | nudix hydrolase 4 [Source:HGNC Symbol;Acc:HGNC:8051] |
| ENSG00000174950 | HGLibA_08252 | 6 | -1.080421432 | CD164L2 | CD164 molecule like 2 [Source:HGNC Symbol;Acc:HGNC:32043] |
| ENSG00000174963 | HGLibA_55330 | 6 | -1.080421432 | ZIC4 | Zic family member 4 [Source:HGNC Symbol;Acc:HGNC:20393] |
| ENSG00000178053 | HGLibA_29371 | 6 | -1.080421432 | MLF1 | myeloid leukemia factor 1 [Source:HGNC Symbol;Acc:HGNC:7125] |
| ENSG00000178568 | HGLibA_15421 | 6 | -1.080421432 | ERBB4 | erb-b2 receptor tyrosine kinase 4 [Source:HGNC Symbol;Acc:HGNC:3432] |
| ENSG00000179085 | HGLibA_13863 | 6 | -1.080421432 | DPM3 | dolichyl-phosphate mannosyltransferase subunit 3 [Source:HGNC Symbol;Acc:HGNC:3007] |
| ENSG00000179403 | HGLibA_53897 | 6 | -1.080421432 | VWA1 | von Willebrand factor A domain containing 1 [Source:HGNC Symbol;Acc:HGNC:30910] |
| ENSG00000179526 | HGLibA_43992 | 6 | -1.080421432 | SHARPIN | SHANK associated RH domain interactor [Source:HGNC Symbol;Acc:HGNC:25321] |
| ENSG00000179593 | HGLibA_01685 | 6 | -1.080421432 | ALOX15B | type B [Source:HGNC Symbol;Acc:HGNC:434]" |
| ENSG00000180008 | HGLibA_46133 | 6 | -1.080421432 | SOCS4 | suppressor of cytokine signaling 4 [Source:HGNC Symbol;Acc:HGNC:19392] |
| ENSG00000180083 | HGLibA_54254 | 6 | -1.080421432 | WFDC11 | WAP four-disulfide core domain 11 [Source:HGNC Symbol;Acc:HGNC:20478] |
| ENSG00000180346 | HGLibA_49467 | 6 | -1.080421432 | TIGD2 | tigger transposable element derived 2 [Source:HGNC Symbol;Acc:HGNC:18333] |
| ENSG00000181163 | HGLibA_32488 | 6 | -1.080421432 | NPM1 | nucleophosmin 1 [Source:HGNC Symbol;Acc:HGNC:7910] |
| ENSG00000182899 | HGLibA_41948 | 6 | -1.080421432 | RPL35A | ribosomal protein L35a [Source:HGNC Symbol;Acc:HGNC:10345] |
| ENSG00000183024 | HGLibA_33669 | 6 | -1.080421432 | OR1G1 | olfactory receptor family 1 subfamily G member 1 [Source:HGNC Symbol;Acc:HGNC:8204] |
| ENSG00000183160 | HGLibA_49892 | 6 | -1.080421432 | TMEM119 | transmembrane protein 119 [Source:HGNC Symbol;Acc:HGNC:27884] |
| ENSG00000185049 | HGLibA_31671 | 6 | -1.080421432 | NELFA | negative elongation factor complex member A [Source:HGNC Symbol;Acc:HGNC:12768] |
| ENSG00000185666 | HGLibA_47962 | 6 | -1.080421432 | SYN3 | synapsin III [Source:HGNC Symbol;Acc:HGNC:11496] |
| ENSG00000187800 | HGLibA_36008 | 6 | -1.080421432 | PEAR1 | platelet endothelial aggregation receptor 1 [Source:HGNC Symbol;Acc:HGNC:33631] |
| ENSG00000187824 | HGLibA_50234 | 6 | -1.080421432 | TMEM220 | transmembrane protein 220 [Source:HGNC Symbol;Acc:HGNC:33757] |
| ENSG00000188167 | HGLibA_50610 | 6 | -1.080421432 | TMPPE | transmembrane protein with metallophosphoesterase domain [Source:HGNC Symbol;Acc:HGNC:33865] |
| ENSG00000188959 | HGLibA_06688 | 6 | -1.080421432 | C9orf152 | chromosome 9 open reading frame 152 [Source:HGNC Symbol;Acc:HGNC:31455] |
| ENSG00000196150 | HGLibA_55665 | 6 | -1.080421432 | ZNF250 | zinc finger protein 250 [Source:HGNC Symbol;Acc:HGNC:13044] |
| ENSG00000196814 | HGLibA_30592 | 6 | -1.080421432 | MVB12B | multivesicular body subunit 12B [Source:HGNC Symbol;Acc:HGNC:23368] |
| ENSG00000197905 | HGLibA_48936 | 6 | -1.080421432 | TEAD4 | TEA domain transcription factor 4 [Source:HGNC Symbol;Acc:HGNC:11717] |
| ENSG00000198001 | HGLibA_23565 | 6 | -1.080421432 | IRAK4 | interleukin 1 receptor associated kinase 4 [Source:HGNC Symbol;Acc:HGNC:17967] |
| ENSG00000198691 | HGLibA_00112 | 6 | -1.080421432 | ABCA4 | ATP binding cassette subfamily A member 4 [Source:HGNC Symbol;Acc:HGNC:34] |
| ENSG00000198722 | HGLibA_52995 | 6 | -1.080421432 | UNC13B | unc-13 homolog B [Source:HGNC Symbol;Acc:HGNC:12566] |
| ENSG00000204519 | HGLibA_56216 | 6 | -1.080421432 | ZNF551 | zinc finger protein 551 [Source:HGNC Symbol;Acc:HGNC:25108] |
| ENSG00000204610 | HGLibA_51415 | 6 | -1.080421432 | TRIM15 | tripartite motif containing 15 [Source:HGNC Symbol;Acc:HGNC:16284] |
| ENSG00000213934 | HGLibA_20926 | 6 | -1.080421432 | HBG1 | hemoglobin subunit gamma 1 [Source:HGNC Symbol;Acc:HGNC:4831] |
| ENSG00000214652 | HGLibA_56635 | 6 | -1.080421432 | ZNF727 | zinc finger protein 727 [Source:HGNC Symbol;Acc:HGNC:22785] |
| ENSG00000214929 | HGLibA_46496 | 6 | -1.080421432 | SPATA31D1 | SPATA31 subfamily D member 1 [Source:HGNC Symbol;Acc:HGNC:37283] |
| ENSG00000216649 | HGLibA_18447 | 6 | -1.080421432 | GAGE12E | G antigen 12E [Source:HGNC Symbol;Acc:HGNC:31905] |
| ENSG00000217555 | HGLibA_09770 | 6 | -1.080421432 | CKLF | chemokine like factor [Source:HGNC Symbol;Acc:HGNC:13253] |
| ENSG00000223510 | HGLibA_08874 | 6 | -1.080421432 | CDRT15 | CMT1A duplicated region transcript 15 [Source:HGNC Symbol;Acc:HGNC:14395] |
| ENSG00000229579 | HGLibA_53259 | 6 | -1.080421432 | USP17L26 | ubiquitin specific peptidase 17-like family member 26 [Source:HGNC Symbol;Acc:HGNC:44454] |
| ENSG00000231051 | HGLibA_53265 | 6 | -1.080421432 | USP17L28 | ubiquitin specific peptidase 17-like family member 28 [Source:HGNC Symbol;Acc:HGNC:44456] |
| ENSG00000237763 | HGLibA_01841 | 6 | -1.080421432 | AMY1A | alpha 1A (salivary) [Source:HGNC Symbol;Acc:HGNC:474]" |
| ENSG00000250298 | HGLibA_19095 | 6 | -1.080421432 | GIMD1 | GIMAP family P-loop NTPase domain containing 1 [Source:HGNC Symbol;Acc:HGNC:44141] |
| ENSG00000253159 | HGLibA_35527 | 6 | -1.080421432 | PCDHGA12 | 12 [Source:HGNC Symbol;Acc:HGNC:8699]" |
| ENSG00000274102 | HGLibA_34043 | 6 | -1.080421432 | OR4M2 | olfactory receptor family 4 subfamily M member 2 [Source:HGNC Symbol;Acc:HGNC:15373] |
| ENSG00000274274 | HGLibA_18465 | 6 | -1.080421432 | GAGE13 | G antigen 13 [Source:HGNC Symbol;Acc:HGNC:29081] |
| ENSG00000275700 | HGLibA_00086 | 6 | -1.080421432 | AATF | apoptosis antagonizing transcription factor [Source:HGNC Symbol;Acc:HGNC:19235] |
| ENSG00000284701 | HGLibA_50307 | 6 | -1.080421432 | TMEM247 | transmembrane protein 247 [Source:HGNC Symbol;Acc:HGNC:42967] |
| ENSG00000285053 | HGLibA_48593 | 6 | -1.080421432 | TBCE | tubulin folding cofactor E [Source:NCBI gene;Acc:6905] |
| FAM127C | HGLibA_16094 | 6 | -1.080421432 | FAM127C | - |
| GBAS | HGLibA_18715 | 6 | -1.080421432 | GBAS | - |
| hsa-mir-1-2 | HGLibA_57145 | 6 | -1.080421432 | hsa-mir-1-2 | - |
| hsa-mir-141 | HGLibA_57829 | 6 | -1.080421432 | hsa-mir-141 | - |
| hsa-mir-199b | HGLibA_58135 | 6 | -1.080421432 | hsa-mir-199b | - |
| hsa-mir-223 | HGLibA_58299 | 6 | -1.080421432 | hsa-mir-223 | - |
| hsa-mir-3610 | HGLibA_59073 | 6 | -1.080421432 | hsa-mir-3610 | - |
| hsa-mir-3650 | HGLibA_59144 | 6 | -1.080421432 | hsa-mir-3650 | - |
| hsa-mir-3663 | HGLibA_59204 | 6 | -1.080421432 | hsa-mir-3663 | - |
| hsa-mir-4699 | HGLibA_60903 | 6 | -1.080421432 | hsa-mir-4699 | - |
| hsa-mir-4713 | HGLibA_60953 | 6 | -1.080421432 | hsa-mir-4713 | - |
| hsa-mir-5196 | HGLibA_61704 | 6 | -1.080421432 | hsa-mir-5196 | - |
| hsa-mir-548av | HGLibA_61930 | 6 | -1.080421432 | hsa-mir-548av | - |
| hsa-mir-548o | HGLibA_62049 | 6 | -1.080421432 | hsa-mir-548o | - |
| hsa-mir-6744 | HGLibA_63131 | 6 | -1.080421432 | hsa-mir-6744 | - |
| hsa-mir-6787 | HGLibA_63325 | 6 | -1.080421432 | hsa-mir-6787 | - |
| hsa-mir-6808 | HGLibA_63411 | 6 | -1.080421432 | hsa-mir-6808 | - |
| hsa-mir-6831 | HGLibA_63502 | 6 | -1.080421432 | hsa-mir-6831 | - |
| hsa-mir-6892 | HGLibA_63753 | 6 | -1.080421432 | hsa-mir-6892 | - |
| KIAA0922 | HGLibA_24668 | 6 | -1.080421432 | KIAA0922 | - |
| KIAA1524 | HGLibA_24774 | 6 | -1.080421432 | KIAA1524 | - |
| LOC100996331 | HGLibA_26883 | 6 | -1.080421432 | LOC100996331 | - |
| LRRC6 | HGLibA_27404 | 6 | -1.080421432 | LRRC6 | - |
| RPS17L | HGLibA_42090 | 6 | -1.080421432 | RPS17L | - |
| WBSCR16 | HGLibA_53988 | 6 | -1.080421432 | WBSCR16 | - |
| ANKRD32 | HGLibA_02065 | 5 | -1.089466775 | ANKRD32 | - |
| C10orf131 | HGLibA_05055 | 5 | -1.089466775 | C10orf131 | - |
| C3orf58 | HGLibA_06253 | 5 | -1.089466775 | C3orf58 | - |
| CCDC11 | HGLibA_07534 | 5 | -1.089466775 | CCDC11 | - |
| ENSG00000005436 | HGLibA_18772 | 5 | -1.089466775 | GCFC2 | GC-rich sequence DNA-binding factor 2 [Source:HGNC Symbol;Acc:HGNC:1317] |
| ENSG00000015532 | HGLibA_54622 | 5 | -1.089466775 | XYLT2 | xylosyltransferase 2 [Source:HGNC Symbol;Acc:HGNC:15517] |
| ENSG00000020426 | HGLibA_29562 | 5 | -1.089466775 | MNAT1 | CDK activating kinase assembly factor [Source:HGNC Symbol;Acc:HGNC:7181]" |
| ENSG00000023892 | HGLibA_12785 | 5 | -1.089466775 | DEF6 | guanine nucleotide exchange factor [Source:HGNC Symbol;Acc:HGNC:2760]" |
| ENSG00000042304 | HGLibA_06193 | 5 | -1.089466775 | C2orf83 | chromosome 2 open reading frame 83 [Source:HGNC Symbol;Acc:HGNC:25344] |
| ENSG00000048707 | HGLibA_53741 | 5 | -1.089466775 | VPS13D | vacuolar protein sorting 13 homolog D [Source:HGNC Symbol;Acc:HGNC:23595] |
| ENSG00000052749 | HGLibA_42268 | 5 | -1.089466775 | RRP12 | ribosomal RNA processing 12 homolog [Source:HGNC Symbol;Acc:HGNC:29100] |
| ENSG00000055332 | HGLibA_14725 | 5 | -1.089466775 | EIF2AK2 | eukaryotic translation initiation factor 2 alpha kinase 2 [Source:HGNC Symbol;Acc:HGNC:9437] |
| ENSG00000067082 | HGLibA_25078 | 5 | -1.089466775 | KLF6 | Kruppel like factor 6 [Source:HGNC Symbol;Acc:HGNC:2235] |
| ENSG00000070669 | HGLibA_03336 | 5 | -1.089466775 | ASNS | asparagine synthetase (glutamine-hydrolyzing) [Source:HGNC Symbol;Acc:HGNC:753] |
| ENSG00000076382 | HGLibA_46361 | 5 | -1.089466775 | SPAG5 | sperm associated antigen 5 [Source:HGNC Symbol;Acc:HGNC:13452] |
| ENSG00000086062 | HGLibA_04024 | 5 | -1.089466775 | B4GALT1 | 4-galactosyltransferase 1 [Source:HGNC Symbol;Acc:HGNC:924]" |
| ENSG00000088387 | HGLibA_13762 | 5 | -1.089466775 | DOCK9 | dedicator of cytokinesis 9 [Source:HGNC Symbol;Acc:HGNC:14132] |
| ENSG00000095906 | HGLibA_32903 | 5 | -1.089466775 | NUBP2 | nucleotide binding protein 2 [Source:HGNC Symbol;Acc:HGNC:8042] |
| ENSG00000096264 | HGLibA_31364 | 5 | -1.089466775 | NCR2 | natural cytotoxicity triggering receptor 2 [Source:HGNC Symbol;Acc:HGNC:6732] |
| ENSG00000101126 | HGLibA_01001 | 5 | -1.089466775 | ADNP | activity dependent neuroprotector homeobox [Source:HGNC Symbol;Acc:HGNC:15766] |
| ENSG00000101134 | HGLibA_13779 | 5 | -1.089466775 | DOK5 | docking protein 5 [Source:HGNC Symbol;Acc:HGNC:16173] |
| ENSG00000101310 | HGLibA_43223 | 5 | -1.089466775 | SEC23B | coat complex II component [Source:HGNC Symbol;Acc:HGNC:10702]" |
| ENSG00000101350 | HGLibA_24936 | 5 | -1.089466775 | KIF3B | kinesin family member 3B [Source:HGNC Symbol;Acc:HGNC:6320] |
| ENSG00000101473 | HGLibA_00491 | 5 | -1.089466775 | ACOT8 | acyl-CoA thioesterase 8 [Source:HGNC Symbol;Acc:HGNC:15919] |
| ENSG00000103266 | HGLibA_47613 | 5 | -1.089466775 | STUB1 | STIP1 homology and U-box containing protein 1 [Source:HGNC Symbol;Acc:HGNC:11427] |
| ENSG00000104679 | HGLibA_39766 | 5 | -1.089466775 | R3HCC1 | R3H domain and coiled-coil containing 1 [Source:HGNC Symbol;Acc:HGNC:27329] |
| ENSG00000104755 | HGLibA_00749 | 5 | -1.089466775 | ADAM2 | ADAM metallopeptidase domain 2 [Source:HGNC Symbol;Acc:HGNC:198] |
| ENSG00000104866 | HGLibA_38094 | 5 | -1.089466775 | PPP1R37 | protein phosphatase 1 regulatory subunit 37 [Source:HGNC Symbol;Acc:HGNC:27607] |
| ENSG00000105810 | HGLibA_08791 | 5 | -1.089466775 | CDK6 | cyclin dependent kinase 6 [Source:HGNC Symbol;Acc:HGNC:1777] |
| ENSG00000107859 | HGLibA_36768 | 5 | -1.089466775 | PITX3 | paired like homeodomain 3 [Source:HGNC Symbol;Acc:HGNC:9006] |
| ENSG00000108592 | HGLibA_18140 | 5 | -1.089466775 | FTSJ3 | FtsJ RNA methyltransferase homolog 3 [Source:HGNC Symbol;Acc:HGNC:17136] |
| ENSG00000111145 | HGLibA_14915 | 5 | -1.089466775 | ELK3 | ETS transcription factor [Source:HGNC Symbol;Acc:HGNC:3325]" |
| ENSG00000111319 | HGLibA_43012 | 5 | -1.089466775 | SCNN1A | sodium channel epithelial 1 alpha subunit [Source:HGNC Symbol;Acc:HGNC:10599] |
| ENSG00000112514 | HGLibA_11797 | 5 | -1.089466775 | CUTA | cutA divalent cation tolerance homolog [Source:HGNC Symbol;Acc:HGNC:21101] |
| ENSG00000113088 | HGLibA_20727 | 5 | -1.089466775 | GZMK | granzyme K [Source:HGNC Symbol;Acc:HGNC:4711] |
| ENSG00000113597 | HGLibA_51309 | 5 | -1.089466775 | TRAPPC13 | trafficking protein particle complex 13 [Source:HGNC Symbol;Acc:HGNC:25828] |

| | | | | | |
|---|---|---|---|---|---|
| ENSG00000115966 | HGLibA_03434 | 5 | -1.089466775 | ATF2 | activating transcription factor 2 [Source:HGNC Symbol;Acc:HGNC:784] |
| ENSG00000116985 | HGLibA_04623 | 5 | -1.089466775 | BMP8B | bone morphogenetic protein 8b [Source:HGNC Symbol;Acc:HGNC:1075] |
| ENSG00000117318 | HGLibA_22531 | 5 | -1.089466775 | ID3 | HLH protein [Source:HGNC Symbol;Acc:HGNC:5362]" |
| ENSG00000119408 | HGLibA_31659 | 5 | -1.089466775 | NEK6 | NIMA related kinase 6 [Source:HGNC Symbol;Acc:HGNC:7749] |
| ENSG00000120341 | HGLibA_43212 | 5 | -1.089466775 | SEC16B | endoplasmic reticulum export factor [Source:HGNC Symbol;Acc:HGNC:30301]" |
| ENSG00000120798 | HGLibA_32594 | 5 | -1.089466775 | NR2C1 | nuclear receptor subfamily 2 group C member 1 [Source:HGNC Symbol;Acc:HGNC:7971] |
| ENSG00000120915 | HGLibA_15327 | 5 | -1.089466775 | EPHX2 | epoxide hydrolase 2 [Source:HGNC Symbol;Acc:HGNC:3402] |
| ENSG00000121900 | HGLibA_50442 | 5 | -1.089466775 | TMEM54 | transmembrane protein 54 [Source:HGNC Symbol;Acc:HGNC:24143] |
| ENSG00000126653 | HGLibA_32778 | 5 | -1.089466775 | NSRP1 | nuclear speckle splicing regulatory protein 1 [Source:HGNC Symbol;Acc:HGNC:25305] |
| ENSG00000129657 | HGLibA_43191 | 5 | -1.089466775 | SEC14L1 | SEC14 like lipid binding 1 [Source:HGNC Symbol;Acc:HGNC:10698] |
| ENSG00000131095 | HGLibA_18934 | 5 | -1.089466775 | GFAP | glial fibrillary acidic protein [Source:HGNC Symbol;Acc:HGNC:4235] |
| ENSG00000132357 | HGLibA_07235 | 5 | -1.089466775 | CARD6 | caspase recruitment domain family member 6 [Source:HGNC Symbol;Acc:HGNC:16394] |
| ENSG00000132676 | HGLibA_12318 | 5 | -1.089466775 | DAP3 | death associated protein 3 [Source:HGNC Symbol;Acc:HGNC:2673] |
| ENSG00000133773 | HGLibA_07808 | 5 | -1.089466775 | CCDC59 | coiled-coil domain containing 59 [Source:HGNC Symbol;Acc:HGNC:25005] |
| ENSG00000134283 | HGLibA_37891 | 5 | -1.089466775 | PPHLN1 | periphilin 1 [Source:HGNC Symbol;Acc:HGNC:19369] |
| ENSG00000137819 | HGLibA_35163 | 5 | -1.089466775 | PAQR5 | progestin and adipoQ receptor family member 5 [Source:HGNC Symbol;Acc:HGNC:29645] |
| ENSG00000138095 | HGLibA_27240 | 5 | -1.089466775 | LRPPRC | leucine rich pentatricopeptide repeat containing [Source:HGNC Symbol;Acc:HGNC:15714] |
| ENSG00000138750 | HGLibA_33059 | 5 | -1.089466775 | NUP54 | nucleoporin 54 [Source:HGNC Symbol;Acc:HGNC:17359] |
| ENSG00000139289 | HGLibA_36417 | 5 | -1.089466775 | PHLDA1 | pleckstrin homology like domain family A member 1 [Source:HGNC Symbol;Acc:HGNC:8933] |
| ENSG00000141562 | HGLibA_31143 | 5 | -1.089466775 | NARF | nuclear prelamin A recognition factor [Source:HGNC Symbol;Acc:HGNC:29916] |
| ENSG00000141665 | HGLibA_17031 | 5 | -1.089466775 | FBXO15 | F-box protein 15 [Source:HGNC Symbol;Acc:HGNC:13617] |
| ENSG00000141971 | HGLibA_30590 | 5 | -1.089466775 | MVB12A | multivesicular body subunit 12A [Source:HGNC Symbol;Acc:HGNC:25153] |
| ENSG00000142182 | HGLibA_13706 | 5 | -1.089466775 | DNMT3L | DNA methyltransferase 3 like [Source:HGNC Symbol;Acc:HGNC:2980] |
| ENSG00000145545 | HGLibA_46940 | 5 | -1.089466775 | SRD5A1 | steroid 5 alpha-reductase 1 [Source:HGNC Symbol;Acc:HGNC:11284] |
| ENSG00000145911 | HGLibA_30960 | 5 | -1.089466775 | N4BP3 | NEDD4 binding protein 3 [Source:HGNC Symbol;Acc:HGNC:29852] |
| ENSG00000145945 | HGLibA_16632 | 5 | -1.089466775 | FAM50B | family with sequence similarity 50 member B [Source:HGNC Symbol;Acc:HGNC:18789] |
| ENSG00000147378 | HGLibA_16922 | 5 | -1.089466775 | FATE1 | fetal and adult testis expressed 1 [Source:HGNC Symbol;Acc:HGNC:24683] |
| ENSG00000149182 | HGLibA_02664 | 5 | -1.089466775 | ARFGAP2 | ADP ribosylation factor GTPase activating protein 2 [Source:HGNC Symbol;Acc:HGNC:13504] |
| ENSG00000149480 | HGLibA_30314 | 5 | -1.089466775 | MTA2 | metastasis associated 1 family member 2 [Source:HGNC Symbol;Acc:HGNC:7411] |
| ENSG00000150477 | HGLibA_24742 | 5 | -1.089466775 | KIAA1328 | KIAA1328 [Source:HGNC Symbol;Acc:HGNC:29248] |
| ENSG00000152433 | HGLibA_56202 | 5 | -1.089466775 | ZNF547 | zinc finger protein 547 [Source:HGNC Symbol;Acc:HGNC:26432] |
| ENSG00000152969 | HGLibA_23921 | 5 | -1.089466775 | JAKMIP1 | janus kinase and microtubule interacting protein 1 [Source:HGNC Symbol;Acc:HGNC:26460] |
| ENSG00000153208 | HGLibA_28906 | 5 | -1.089466775 | MERTK | tyrosine kinase [Source:HGNC Symbol;Acc:HGNC:7027]" |
| ENSG00000154945 | HGLibA_02097 | 5 | -1.089466775 | ANKRD40 | ankyrin repeat domain 40 [Source:HGNC Symbol;Acc:HGNC:28233] |
| ENSG00000155275 | HGLibA_51689 | 5 | -1.089466775 | TRMT44 | tRNA methyltransferase 44 homolog [Source:HGNC Symbol;Acc:HGNC:26653] |
| ENSG00000156787 | HGLibA_48537 | 5 | -1.089466775 | TBC1D31 | TBC1 domain family member 31 [Source:HGNC Symbol;Acc:HGNC:30888] |
| ENSG00000158301 | HGLibA_20121 | 5 | -1.089466775 | GPRASP2 | G protein-coupled receptor associated sorting protein 2 [Source:HGNC Symbol;Acc:HGNC:25169] |
| ENSG00000159399 | HGLibA_21583 | 5 | -1.089466775 | HK2 | hexokinase 2 [Source:HGNC Symbol;Acc:HGNC:4923] |
| ENSG00000161091 | HGLibA_29091 | 5 | -1.089466775 | MFSD12 | major facilitator superfamily domain containing 12 [Source:HGNC Symbol;Acc:HGNC:28299] |
| ENSG00000163793 | HGLibA_13630 | 5 | -1.089466775 | DNAJC5G | DnaJ heat shock protein family (Hsp40) member C5 gamma [Source:HGNC Symbol;Acc:HGNC:24844] |
| ENSG00000163840 | HGLibA_14087 | 5 | -1.089466775 | DTX3L | deltex E3 ubiquitin ligase 3L [Source:HGNC Symbol;Acc:HGNC:30323] |
| ENSG00000163877 | HGLibA_45929 | 5 | -1.089466775 | SNIP1 | Smad nuclear interacting protein 1 [Source:HGNC Symbol;Acc:HGNC:30587] |
| ENSG00000164299 | HGLibA_46910 | 5 | -1.089466775 | SPZ1 | spermatogenic leucine zipper 1 [Source:HGNC Symbol;Acc:HGNC:30721] |
| ENSG00000164347 | HGLibA_18949 | 5 | -1.089466775 | GFM2 | G elongation factor mitochondrial 2 [Source:HGNC Symbol;Acc:HGNC:29682] |
| ENSG00000165078 | HGLibA_10898 | 5 | -1.089466775 | CPA6 | carboxypeptidase A6 [Source:HGNC Symbol;Acc:HGNC:17245] |
| ENSG00000165804 | HGLibA_55603 | 5 | -1.089466775 | ZNF219 | zinc finger protein 219 [Source:HGNC Symbol;Acc:HGNC:13011] |
| ENSG00000165914 | HGLibA_52152 | 5 | -1.089466775 | TTC7B | tetratricopeptide repeat domain 7B [Source:HGNC Symbol;Acc:HGNC:19858] |
| ENSG00000166348 | HGLibA_53401 | 5 | -1.089466775 | USP54 | ubiquitin specific peptidase 54 [Source:HGNC Symbol;Acc:HGNC:23513] |
| ENSG00000167759 | HGLibA_25244 | 5 | -1.089466775 | KLK13 | kallikrein related peptidase 13 [Source:HGNC Symbol;Acc:HGNC:6361] |
| ENSG00000168152 | HGLibA_49317 | 5 | -1.089466775 | THAP9 | THAP domain containing 9 [Source:HGNC Symbol;Acc:HGNC:23192] |
| ENSG00000169314 | HGLibA_06054 | 5 | -1.089466775 | C22orf15 | chromosome 22 open reading frame 15 [Source:HGNC Symbol;Acc:HGNC:15558] |
| ENSG00000169490 | HGLibA_49685 | 5 | -1.089466775 | TM2D2 | TM2 domain containing 2 [Source:HGNC Symbol;Acc:HGNC:24127] |
| ENSG00000171049 | HGLibA_17965 | 5 | -1.089466775 | FPR2 | formyl peptide receptor 2 [Source:HGNC Symbol;Acc:HGNC:3827] |
| ENSG00000172410 | HGLibA_23387 | 5 | -1.089466775 | INSL5 | insulin like 5 [Source:HGNC Symbol;Acc:HGNC:6088] |
| ENSG00000174876 | HGLibA_01843 | 5 | -1.089466775 | AMY1B | alpha 1B (salivary) [Source:HGNC Symbol;Acc:HGNC:475]" |
| ENSG00000175264 | HGLibA_09609 | 5 | -1.089466775 | CHST1 | carbohydrate sulfotransferase 1 [Source:HGNC Symbol;Acc:HGNC:1969] |
| ENSG00000175697 | HGLibA_19946 | 5 | -1.089466775 | GPR156 | G protein-coupled receptor 156 [Source:HGNC Symbol;Acc:HGNC:20844] |
| ENSG00000176261 | HGLibA_54931 | 5 | -1.089466775 | ZBTB8OS | zinc finger and BTB domain containing 8 opposite strand [Source:HGNC Symbol;Acc:HGNC:24094] |
| ENSG00000177873 | HGLibA_56402 | 5 | -1.089466775 | ZNF619 | zinc finger protein 619 [Source:HGNC Symbol;Acc:HGNC:26910] |
| ENSG00000178184 | HGLibA_35189 | 5 | -1.089466775 | PARD6G | par-6 family cell polarity regulator gamma [Source:HGNC Symbol;Acc:HGNC:16076] |
| ENSG00000180228 | HGLibA_38585 | 5 | -1.089466775 | PRKRA | protein activator of interferon induced protein kinase EIF2AK2 [Source:HGNC Symbol;Acc:HGNC:9438] |
| ENSG00000180638 | HGLibA_45197 | 5 | -1.089466775 | SLC47A2 | solute carrier family 47 member 2 [Source:HGNC Symbol;Acc:HGNC:26439] |
| ENSG00000182220 | HGLibA_03699 | 5 | -1.089466775 | ATP6AP2 | ATPase H+ transporting accessory protein 2 [Source:HGNC Symbol;Acc:HGNC:18305] |
| ENSG00000183337 | HGLibA_04361 | 5 | -1.089466775 | BCOR | BCL6 corepressor [Source:HGNC Symbol;Acc:HGNC:20893] |
| ENSG00000183597 | HGLibA_48298 | 5 | -1.089466775 | TANGO2 | transport and golgi organization 2 homolog [Source:HGNC Symbol;Acc:HGNC:25439] |
| ENSG00000183837 | HGLibA_37371 | 5 | -1.089466775 | PNMA3 | PNMA family member 3 [Source:HGNC Symbol;Acc:HGNC:18742] |
| ENSG00000184445 | HGLibA_25351 | 5 | -1.089466775 | KNTC1 | kinetochore associated 1 [Source:HGNC Symbol;Acc:HGNC:17255] |
| ENSG00000185013 | HGLibA_32806 | 5 | -1.089466775 | NT5C1B | cytosolic IB [Source:HGNC Symbol;Acc:HGNC:17818]" |
| ENSG00000189056 | HGLibA_40825 | 5 | -1.089466775 | RELN | reelin [Source:HGNC Symbol;Acc:HGNC:9957] |
| ENSG00000197360 | HGLibA_56914 | 5 | -1.089466775 | ZNF98 | zinc finger protein 98 [Source:HGNC Symbol;Acc:HGNC:13174] |
| ENSG00000197622 | HGLibA_08581 | 5 | -1.089466775 | CDC42SE1 | CDC42 small effector 1 [Source:HGNC Symbol;Acc:HGNC:17719] |
| ENSG00000198729 | HGLibA_38040 | 5 | -1.089466775 | PPP1R14C | protein phosphatase 1 regulatory inhibitor subunit 14C [Source:HGNC Symbol;Acc:HGNC:14952] |
| ENSG00000198911 | HGLibA_46951 | 5 | -1.089466775 | SREBF2 | sterol regulatory element binding transcription factor 2 [Source:HGNC Symbol;Acc:HGNC:11290] |
| ENSG00000205777 | HGLibA_18432 | 5 | -1.089466775 | GAGE1 | G antigen 1 [Source:HGNC Symbol;Acc:HGNC:4098] |
| ENSG00000214114 | HGLibA_30667 | 5 | -1.089466775 | MYCBP | MYC binding protein [Source:HGNC Symbol;Acc:HGNC:7554] |
| ENSG00000215029 | HGLibA_48851 | 5 | -1.089466775 | TCP11X2 | X-linked 2 [Source:HGNC Symbol;Acc:HGNC:48335]" |
| ENSG00000215269 | HGLibA_18453 | 5 | -1.089466775 | GAGE12G | G antigen 12G [Source:HGNC Symbol;Acc:HGNC:31907] |
| ENSG00000216937 | HGLibA_07848 | 5 | -1.089466775 | CCDC7 | coiled-coil domain containing 7 [Source:HGNC Symbol;Acc:HGNC:26533] |
| ENSG00000219481 | HGLibA_31220 | 5 | -1.089466775 | NBPF1 | NBPF member 1 [Source:HGNC Symbol;Acc:HGNC:26088] |
| ENSG00000221858 | HGLibA_33722 | 5 | -1.089466775 | OR2A12 | olfactory receptor family 2 subfamily A member 12 [Source:HGNC Symbol;Acc:HGNC:15082] |
| ENSG00000226887 | HGLibA_15543 | 5 | -1.089466775 | ERVMER34-1 | envelope [Source:HGNC Symbol;Acc:HGNC:42970]" |
| ENSG00000227051 | HGLibA_05325 | 5 | -1.089466775 | C14orf132 | chromosome 14 open reading frame 132 [Source:HGNC Symbol;Acc:HGNC:20346] |
| ENSG00000227551 | HGLibA_53220 | 5 | -1.089466775 | USP17L12 | ubiquitin specific peptidase 17-like family member 12 [Source:HGNC Symbol;Acc:HGNC:44440] |
| ENSG00000228856 | HGLibA_53274 | 5 | -1.089466775 | USP17L30 | ubiquitin specific peptidase 17-like family member 30 [Source:HGNC Symbol;Acc:HGNC:44458] |
| ENSG00000236737 | HGLibA_18438 | 5 | -1.089466775 | GAGE12B | G antigen 12B [Source:HGNC Symbol;Acc:HGNC:26779] |
| ENSG00000240344 | HGLibA_37944 | 5 | -1.089466775 | PPIL3 | peptidylprolyl isomerase like 3 [Source:HGNC Symbol;Acc:HGNC:9262] |
| ENSG00000249471 | HGLibA_55803 | 5 | -1.089466775 | ZNF324B | zinc finger protein 324B [Source:HGNC Symbol;Acc:HGNC:33107] |
| ENSG00000250423 | HGLibA_24713 | 5 | -1.089466775 | KIAA1210 | KIAA1210 [Source:HGNC Symbol;Acc:HGNC:29218] |
| ENSG00000251655 | HGLibA_38339 | 5 | -1.089466775 | PRB1 | proline rich protein BstNI subfamily 1 [Source:HGNC Symbol;Acc:HGNC:9337] |
| ENSG00000255526 | HGLibA_31611 | 5 | -1.089466775 | NEDD8-MDP1 | NEDD8-MDP1 readthrough [Source:HGNC Symbol;Acc:HGNC:39551] |
| ENSG00000277150 | HGLibA_15892 | 5 | -1.089466775 | F8A3 | coagulation factor VIII associated 3 [Source:HGNC Symbol;Acc:HGNC:31850] |
| ENSG00000279486 | HGLibA_33748 | 5 | -1.089466775 | OR2AG1 | olfactory receptor family 2 subfamily AG member 1 (gene/pseudogene) [Source:HGNC Symbol;Acc:HGNC:15142] |
| FAM122A | HGLibA_16067 | 5 | -1.089466775 | FAM122A | - |
| GAGE5 | HGLibA_18483 | 5 | -1.089466775 | GAGE5 | - |
| HEXDC | HGLibA_21207 | 5 | -1.089466775 | HEXDC | - |
| HIST1H2BL | HGLibA_21445 | 5 | -1.089466775 | HIST1H2BL | - |
| hsa-let-7g | HGLibA_57134 | 5 | -1.089466775 | hsa-let-7g | - |
| hsa-let-7i | HGLibA_57138 | 5 | -1.089466775 | hsa-let-7i | - |
| hsa-mir-1185-2 | HGLibA_57247 | 5 | -1.089466775 | hsa-mir-1185-2 | - |
| hsa-mir-122 | HGLibA_57292 | 5 | -1.089466775 | hsa-mir-122 | - |
| hsa-mir-1256 | HGLibA_57430 | 5 | -1.089466775 | hsa-mir-1256 | - |
| hsa-mir-3142 | HGLibA_58625 | 5 | -1.089466775 | hsa-mir-3142 | - |
| hsa-mir-320c-1 | HGLibA_58937 | 5 | -1.089466775 | hsa-mir-320c-1 | - |
| hsa-mir-3943 | HGLibA_59632 | 5 | -1.089466775 | hsa-mir-3943 | - |
| hsa-mir-4281 | HGLibA_59826 | 5 | -1.089466775 | hsa-mir-4281 | - |
| hsa-mir-4327 | HGLibA_60030 | 5 | -1.089466775 | hsa-mir-4327 | - |
| hsa-mir-4690 | HGLibA_60870 | 5 | -1.089466775 | hsa-mir-4690 | - |
| hsa-mir-4728 | HGLibA_61011 | 5 | -1.089466775 | hsa-mir-4728 | - |
| hsa-mir-4779 | HGLibA_61222 | 5 | -1.089466775 | hsa-mir-4779 | - |
| hsa-mir-4793 | HGLibA_61280 | 5 | -1.089466775 | hsa-mir-4793 | - |
| hsa-mir-548f-2 | HGLibA_61977 | 5 | -1.089466775 | hsa-mir-548f-2 | - |
| hsa-mir-564 | HGLibA_62219 | 5 | -1.089466775 | hsa-mir-564 | - |
| hsa-mir-5706 | HGLibA_62364 | 5 | -1.089466775 | hsa-mir-5706 | - |
| hsa-mir-613 | HGLibA_62666 | 5 | -1.089466775 | hsa-mir-613 | - |
| hsa-mir-6722 | HGLibA_63049 | 5 | -1.089466775 | hsa-mir-6722 | - |
| hsa-mir-8067 | HGLibA_64107 | 5 | -1.089466775 | hsa-mir-8067 | - |
| KCNE1L | HGLibA_24186 | 5 | -1.089466775 | KCNE1L | - |
| LOC100130348 | HGLibA_26777 | 5 | -1.089466775 | LOC100130348 | - |
| LOC730159 | HGLibA_27002 | 5 | -1.089466775 | LOC730159 | - |
| MB21D1 | HGLibA_28458 | 5 | -1.089466775 | MB21D1 | - |
| MLL4 | HGLibA_29403 | 5 | -1.089466775 | MLL4 | - |
| RTFDC1 | HGLibA_42385 | 5 | -1.089466775 | RTFDC1 | - |
| BAI2 | HGLibA_04117 | 4 | -1.098512118 | BAI2 | - |
| C12orf5 | HGLibA_05256 | 4 | -1.098512118 | C12orf5 | - |
| C1orf61 | HGLibA_05880 | 4 | -1.098512118 | C1orf61 | - |
| C1orf85 | HGLibA_05897 | 4 | -1.098512118 | C1orf85 | - |
| C7orf41 | HGLibA_06543 | 4 | -1.098512118 | C7orf41 | - |
| C9orf41 | HGLibA_06732 | 4 | -1.098512118 | C9orf41 | - |
| CTAGE5 | HGLibA_11576 | 4 | -1.098512118 | CTAGE5 | - |
| ENSG00000005102 | HGLibA_28890 | 4 | -1.098512118 | MEOX1 | mesenchyme homeobox 1 [Source:HGNC Symbol;Acc:HGNC:7013] |
| ENSG00000006756 | HGLibA_03135 | 4 | -1.098512118 | ARSD | arylsulfatase D [Source:HGNC Symbol;Acc:HGNC:717] |
| ENSG00000012822 | HGLibA_06999 | 4 | -1.098512118 | CALCOCO1 | calcium binding and coiled-coil domain 1 [Source:HGNC Symbol;Acc:HGNC:29306] |
| ENSG00000049656 | HGLibA_10115 | 4 | -1.098512118 | CLPTM1L | CLPTM1 like [Source:HGNC Symbol;Acc:HGNC:24308] |
| ENSG00000063322 | HGLibA_28798 | 4 | -1.098512118 | MED29 | mediator complex subunit 29 [Source:HGNC Symbol;Acc:HGNC:23074] |
| ENSG00000066739 | HGLibA_03481 | 4 | -1.098512118 | ATG2B | autophagy related 2B [Source:HGNC Symbol;Acc:HGNC:20187] |

| | | | | | |
|---|---|---|---|---|---|
| ENSG00000070087 | HGLibA_36177 | 4 | -1.098512118 | PFN2 | profilin 2 [Source:HGNC Symbol;Acc:HGNC:8882] |
| ENSG00000082212 | HGLibA_28712 | 4 | -1.098512118 | ME2 | malic enzyme 2 [Source:HGNC Symbol;Acc:HGNC:6984] |
| ENSG00000089597 | HGLibA_18604 | 4 | -1.098512118 | GANAB | glucosidase II alpha subunit [Source:HGNC Symbol;Acc:HGNC:4138] |
| ENSG00000091039 | HGLibA_34667 | 4 | -1.098512118 | OSBPL8 | oxysterol binding protein like 8 [Source:HGNC Symbol;Acc:HGNC:16396] |
| ENSG00000100836 | HGLibA_34942 | 4 | -1.098512118 | PABPN1 | poly(A) binding protein nuclear 1 [Source:HGNC Symbol;Acc:HGNC:8565] |
| ENSG00000101624 | HGLibA_09163 | 4 | -1.098512118 | CEP76 | centrosomal protein 76 [Source:HGNC Symbol;Acc:HGNC:25727] |
| ENSG00000103260 | HGLibA_28938 | 4 | -1.098512118 | METRN | glial cell differentiation regulator [Source:HGNC Symbol;Acc:HGNC:14151]" |
| ENSG00000103522 | HGLibA_23086 | 4 | -1.098512118 | IL21R | interleukin 21 receptor [Source:HGNC Symbol;Acc:HGNC:6006] |
| ENSG00000103942 | HGLibA_21867 | 4 | -1.098512118 | HOMER2 | homer scaffold protein 2 [Source:HGNC Symbol;Acc:HGNC:17513] |
| ENSG00000110060 | HGLibA_39601 | 4 | -1.098512118 | PUS3 | pseudouridine synthase 3 [Source:HGNC Symbol;Acc:HGNC:25461] |
| ENSG00000112499 | HGLibA_44566 | 4 | -1.098512118 | SLC22A2 | solute carrier family 22 member 2 [Source:HGNC Symbol;Acc:HGNC:10966] |
| ENSG00000112855 | HGLibA_20858 | 4 | -1.098512118 | HARS2 | mitochondrial [Source:HGNC Symbol;Acc:HGNC:4817]" |
| ENSG00000113555 | HGLibA_35397 | 4 | -1.098512118 | PCDH12 | protocadherin 12 [Source:HGNC Symbol;Acc:HGNC:8657] |
| ENSG00000115138 | HGLibA_37654 | 4 | -1.098512118 | POMC | proopiomelanocortin [Source:HGNC Symbol;Acc:HGNC:9201] |
| ENSG00000115211 | HGLibA_14745 | 4 | -1.098512118 | EIF2B4 | eukaryotic translation initiation factor 2B subunit delta [Source:HGNC Symbol;Acc:HGNC:3260] |
| ENSG00000116698 | HGLibA_45708 | 4 | -1.098512118 | SMG7 | nonsense mediated mRNA decay factor [Source:HGNC Symbol;Acc:HGNC:16792]" |
| ENSG00000118680 | HGLibA_30753 | 4 | -1.098512118 | MYL12B | myosin light chain 12B [Source:HGNC Symbol;Acc:HGNC:29827] |
| ENSG00000118946 | HGLibA_35404 | 4 | -1.098512118 | PCDH17 | protocadherin 17 [Source:HGNC Symbol;Acc:HGNC:14267] |
| ENSG00000122674 | HGLibA_08221 | 4 | -1.098512118 | CCZ1 | vacuolar protein trafficking and biogenesis associated [Source:HGNC Symbol;Acc:HGNC:21691]" |
| ENSG00000123415 | HGLibA_45841 | 4 | -1.098512118 | SMUG1 | single-strand-selective monofunctional uracil-DNA glycosylase 1 [Source:HGNC Symbol;Acc:HGNC:17148] |
| ENSG00000123684 | HGLibA_27084 | 4 | -1.098512118 | LPGAT1 | lysophosphatidylglycerol acyltransferase 1 [Source:HGNC Symbol;Acc:HGNC:28985] |
| ENSG00000125170 | HGLibA_13776 | 4 | -1.098512118 | DOK4 | docking protein 4 [Source:HGNC Symbol;Acc:HGNC:19868] |
| ENSG00000126838 | HGLibA_39716 | 4 | -1.098512118 | PZP | alpha-2-macroglobulin like [Source:HGNC Symbol;Acc:HGNC:9750]" |
| ENSG00000130023 | HGLibA_15503 | 4 | -1.098512118 | ERMARD | ER membrane associated RNA degradation [Source:HGNC Symbol;Acc:HGNC:21056] |
| ENSG00000130656 | HGLibA_20942 | 4 | -1.098512118 | HBZ | hemoglobin subunit zeta [Source:HGNC Symbol;Acc:HGNC:4835] |
| ENSG00000131876 | HGLibA_45965 | 4 | -1.098512118 | SNRPA1 | small nuclear ribonucleoprotein polypeptide A' [Source:HGNC Symbol;Acc:HGNC:11152] |
| ENSG00000132017 | HGLibA_12429 | 4 | -1.098512118 | DCAF15 | DDB1 and CUL4 associated factor 15 [Source:HGNC Symbol;Acc:HGNC:25095] |
| ENSG00000132026 | HGLibA_42368 | 4 | -1.098512118 | RTBDN | retbindin [Source:HGNC Symbol;Acc:HGNC:30310] |
| ENSG00000134480 | HGLibA_08100 | 4 | -1.098512118 | CCNH | cyclin H [Source:HGNC Symbol;Acc:HGNC:1594] |
| ENSG00000135070 | HGLibA_23645 | 4 | -1.098512118 | ISCA1 | iron-sulfur cluster assembly 1 [Source:HGNC Symbol;Acc:HGNC:28660] |
| ENSG00000136541 | HGLibA_15506 | 4 | -1.098512118 | ERMN | ermin [Source:HGNC Symbol;Acc:HGNC:29208] |
| ENSG00000138684 | HGLibA_23083 | 4 | -1.098512118 | IL21 | interleukin 21 [Source:HGNC Symbol;Acc:HGNC:6005] |
| ENSG00000138778 | HGLibA_09051 | 4 | -1.098512118 | CENPE | centromere protein E [Source:HGNC Symbol;Acc:HGNC:1856] |
| ENSG00000140873 | HGLibA_00816 | 4 | -1.098512118 | ADAMTS18 | ADAM metallopeptidase with thrombospondin type 1 motif 18 [Source:HGNC Symbol;Acc:HGNC:17110] |
| ENSG00000142082 | HGLibA_44222 | 4 | -1.098512118 | SIRT3 | sirtuin 3 [Source:HGNC Symbol;Acc:HGNC:14931] |
| ENSG00000142945 | HGLibA_24932 | 4 | -1.098512118 | KIF2C | kinesin family member 2C [Source:HGNC Symbol;Acc:HGNC:6393] |
| ENSG00000143324 | HGLibA_54579 | 4 | -1.098512118 | XPR1 | xenotropic and polytropic retrovirus receptor 1 [Source:HGNC Symbol;Acc:HGNC:12827] |
| ENSG00000143373 | HGLibA_56539 | 4 | -1.098512118 | ZNF687 | zinc finger protein 687 [Source:HGNC Symbol;Acc:HGNC:29277] |
| ENSG00000144401 | HGLibA_28977 | 4 | -1.098512118 | METTL21A | methyltransferase like 21A [Source:HGNC Symbol;Acc:HGNC:30476] |
| ENSG00000145730 | HGLibA_35081 | 4 | -1.098512118 | PAM | peptidylglycine alpha-amidating monooxygenase [Source:HGNC Symbol;Acc:HGNC:8596] |
| ENSG00000149925 | HGLibA_01591 | 4 | -1.098512118 | ALDOA | fructose-bisphosphate A [Source:HGNC Symbol;Acc:HGNC:414]" |
| ENSG00000154917 | HGLibA_39973 | 4 | -1.098512118 | RAB6B | member RAS oncogene family [Source:HGNC Symbol;Acc:HGNC:14902]" |
| ENSG00000156313 | HGLibA_41836 | 4 | -1.098512118 | RPGR | retinitis pigmentosa GTPase regulator [Source:HGNC Symbol;Acc:HGNC:10295] |
| ENSG00000156381 | HGLibA_02148 | 4 | -1.098512118 | ANKRD9 | ankyrin repeat domain 9 [Source:HGNC Symbol;Acc:HGNC:20096] |
| ENSG00000156687 | HGLibA_53026 | 4 | -1.098512118 | UNC5D | unc-5 netrin receptor D [Source:HGNC Symbol;Acc:HGNC:18634] |
| ENSG00000158488 | HGLibA_08276 | 4 | -1.098512118 | CD1E | CD1e molecule [Source:HGNC Symbol;Acc:HGNC:1638] |
| ENSG00000159377 | HGLibA_39108 | 4 | -1.098512118 | PSMB4 | proteasome subunit beta 4 [Source:HGNC Symbol;Acc:HGNC:9541] |
| ENSG00000159388 | HGLibA_04945 | 4 | -1.098512118 | BTG2 | BTG anti-proliferation factor 2 [Source:HGNC Symbol;Acc:HGNC:1131] |
| ENSG00000159455 | HGLibA_26122 | 4 | -1.098512118 | LCE2B | late cornified envelope 2B [Source:HGNC Symbol;Acc:HGNC:16610] |
| ENSG00000160307 | HGLibA_42577 | 4 | -1.098512118 | S100B | S100 calcium binding protein B [Source:HGNC Symbol;Acc:HGNC:10500] |
| ENSG00000160321 | HGLibA_55583 | 4 | -1.098512118 | ZNF208 | zinc finger protein 208 [Source:HGNC Symbol;Acc:HGNC:12999] |
| ENSG00000161040 | HGLibA_16976 | 4 | -1.098512118 | FBXL13 | F-box and leucine rich repeat protein 13 [Source:HGNC Symbol;Acc:HGNC:21658] |
| ENSG00000164175 | HGLibA_45174 | 4 | -1.098512118 | SLC45A2 | solute carrier family 45 member 2 [Source:HGNC Symbol;Acc:HGNC:16472] |
| ENSG00000164951 | HGLibA_35924 | 4 | -1.098512118 | PDP1 | pyruvate dehyrogenase phosphatase catalytic subunit 1 [Source:HGNC Symbol;Acc:HGNC:9279] |
| ENSG00000166743 | HGLibA_00570 | 4 | -1.098512118 | ACSM1 | acyl-CoA synthetase medium chain family member 1 [Source:HGNC Symbol;Acc:HGNC:18049] |
| ENSG00000167157 | HGLibA_38895 | 4 | -1.098512118 | PRRX2 | paired related homeobox 2 [Source:HGNC Symbol;Acc:HGNC:21338] |
| ENSG00000168843 | HGLibA_18111 | 4 | -1.098512118 | FSTL5 | follistatin like 5 [Source:HGNC Symbol;Acc:HGNC:21386] |
| ENSG00000168952 | HGLibA_47681 | 4 | -1.098512118 | STXBP6 | syntaxin binding protein 6 [Source:HGNC Symbol;Acc:HGNC:19666] |
| ENSG00000169635 | HGLibA_21292 | 4 | -1.098512118 | HIC2 | HIC ZBTB transcriptional repressor 2 [Source:HGNC Symbol;Acc:HGNC:18595] |
| ENSG00000169714 | HGLibA_10247 | 4 | -1.098512118 | CNBP | CCHC-type zinc finger nucleic acid binding protein [Source:HGNC Symbol;Acc:HGNC:13164] |
| ENSG00000170345 | HGLibA_17796 | 4 | -1.098512118 | FOS | AP-1 transcription factor subunit [Source:HGNC Symbol;Acc:HGNC:3796]" |
| ENSG00000171217 | HGLibA_09881 | 4 | -1.098512118 | CLDN20 | claudin 20 [Source:HGNC Symbol;Acc:HGNC:2042] |
| ENSG00000172037 | HGLibA_25958 | 4 | -1.098512118 | LAMB2 | laminin subunit beta 2 [Source:HGNC Symbol;Acc:HGNC:6487] |
| ENSG00000172426 | HGLibA_42340 | 4 | -1.098512118 | RSPH9 | radial spoke head 9 homolog [Source:HGNC Symbol;Acc:HGNC:21057] |
| ENSG00000172935 | HGLibA_29840 | 4 | -1.098512118 | MRGPRF | MAS related GPR family member F [Source:HGNC Symbol;Acc:HGNC:24828] |
| ENSG00000173285 | HGLibA_33531 | 4 | -1.098512118 | OR10K1 | olfactory receptor family 10 subfamily K member 1 [Source:HGNC Symbol;Acc:HGNC:14693] |
| ENSG00000173947 | HGLibA_36539 | 4 | -1.098512118 | PIFO | primary cilia formation [Source:HGNC Symbol;Acc:HGNC:27009] |
| ENSG00000175073 | HGLibA_53578 | 4 | -1.098512118 | VCPIP1 | valosin containing protein interacting protein 1 [Source:HGNC Symbol;Acc:HGNC:30897] |
| ENSG00000176020 | HGLibA_01799 | 4 | -1.098512118 | AMIGO3 | adhesion molecule with Ig like domain 3 [Source:HGNC Symbol;Acc:HGNC:24075] |
| ENSG00000176563 | HGLibA_10386 | 4 | -1.098512118 | CNTD1 | cyclin N-terminal domain containing 1 [Source:HGNC Symbol;Acc:HGNC:26847] |
| ENSG00000176769 | HGLibA_48758 | 4 | -1.098512118 | TCERG1L | transcription elongation regulator 1 like [Source:HGNC Symbol;Acc:HGNC:23533] |
| ENSG00000177462 | HGLibA_33892 | 4 | -1.098512118 | OR2T8 | olfactory receptor family 2 subfamily T member 8 [Source:HGNC Symbol;Acc:HGNC:15020] |
| ENSG00000179144 | HGLibA_19087 | 4 | -1.098512118 | GIMAP7 | IMAP family member 7 [Source:HGNC Symbol;Acc:HGNC:22404]" |
| ENSG00000181585 | HGLibA_50583 | 4 | -1.098512118 | TMIE | transmembrane inner ear [Source:HGNC Symbol;Acc:HGNC:30800] |
| ENSG00000181656 | HGLibA_20105 | 4 | -1.098512118 | GPR88 | G protein-coupled receptor 88 [Source:HGNC Symbol;Acc:HGNC:4539] |
| ENSG00000182774 | HGLibA_42087 | 4 | -1.098512118 | RPS17 | ribosomal protein S17 [Source:HGNC Symbol;Acc:HGNC:10397] |
| ENSG00000183431 | HGLibA_43698 | 4 | -1.098512118 | SF3A3 | splicing factor 3a subunit 3 [Source:HGNC Symbol;Acc:HGNC:10767] |
| ENSG00000183621 | HGLibA_55993 | 4 | -1.098512118 | ZNF438 | zinc finger protein 438 [Source:HGNC Symbol;Acc:HGNC:21029] |
| ENSG00000183715 | HGLibA_33412 | 4 | -1.098512118 | OPCML | opioid binding protein/cell adhesion molecule like [Source:HGNC Symbol;Acc:HGNC:8143] |
| ENSG00000184949 | HGLibA_16527 | 4 | -1.098512118 | FAM227A | family with sequence similarity 227 member A [Source:HGNC Symbol;Acc:HGNC:44197] |
| ENSG00000185013 | HGLibA_32805 | 4 | -1.098512118 | NT5C1B | cytosolic IB [Source:HGNC Symbol;Acc:HGNC:17818]" |
| ENSG00000186074 | HGLibA_08343 | 4 | -1.098512118 | CD300LF | CD300 molecule like family member f [Source:HGNC Symbol;Acc:HGNC:29883] |
| ENSG00000186265 | HGLibA_04956 | 4 | -1.098512118 | BTLA | B and T lymphocyte associated [Source:HGNC Symbol;Acc:HGNC:21087] |
| ENSG00000186777 | HGLibA_56646 | 4 | -1.098512118 | ZNF732 | zinc finger protein 732 [Source:HGNC Symbol;Acc:HGNC:37138] |
| ENSG00000187634 | HGLibA_42666 | 4 | -1.098512118 | SAMD11 | sterile alpha motif domain containing 11 [Source:HGNC Symbol;Acc:HGNC:28706] |
| ENSG00000189227 | HGLibA_05429 | 4 | -1.098512118 | C15orf61 | chromosome 15 open reading frame 61 [Source:HGNC Symbol;Acc:HGNC:34453] |
| ENSG00000196932 | HGLibA_50347 | 4 | -1.098512118 | TMEM26 | transmembrane protein 26 [Source:HGNC Symbol;Acc:HGNC:28550] |
| ENSG00000197385 | HGLibA_56878 | 4 | -1.098512118 | ZNF860 | zinc finger protein 860 [Source:HGNC Symbol;Acc:HGNC:34513] |
| ENSG00000197712 | HGLibA_16030 | 4 | -1.098512118 | FAM114A1 | family with sequence similarity 114 member A1 [Source:HGNC Symbol;Acc:HGNC:25087] |
| ENSG00000197746 | HGLibA_39001 | 4 | -1.098512118 | PSAP | prosaposin [Source:HGNC Symbol;Acc:HGNC:9498] |
| ENSG00000198625 | HGLibA_28699 | 4 | -1.098512118 | MDM4 | p53 regulator [Source:HGNC Symbol;Acc:HGNC:6974]" |
| ENSG00000203697 | HGLibA_07178 | 4 | -1.098512118 | CAPN8 | calpain 8 [Source:HGNC Symbol;Acc:HGNC:1485] |
| ENSG00000204219 | HGLibA_48701 | 4 | -1.098512118 | TCEA3 | transcription elongation factor A3 [Source:HGNC Symbol;Acc:HGNC:11615] |
| ENSG00000205678 | HGLibA_48950 | 4 | -1.098512118 | TECRL | 3-enoyl-CoA reductase like [Source:HGNC Symbol;Acc:HGNC:27365]" |
| ENSG00000206026 | HGLibA_45750 | 4 | -1.098512118 | SMIM21 | small integral membrane protein 21 [Source:HGNC Symbol;Acc:HGNC:27598] |
| ENSG00000206190 | HGLibA_03544 | 4 | -1.098512118 | ATP10A | ATPase phospholipid transporting 10A (putative) [Source:HGNC Symbol;Acc:HGNC:13542] |
| ENSG00000206418 | HGLibA_39807 | 4 | -1.098512118 | RAB12 | member RAS oncogene family [Source:HGNC Symbol;Acc:HGNC:31332]" |
| ENSG00000221995 | HGLibA_49432 | 4 | -1.098512118 | TIAF1 | TGFB1-induced anti-apoptotic factor 1 [Source:HGNC Symbol;Acc:HGNC:11803] |
| ENSG00000224389 | HGLibA_06285 | 4 | -1.098512118 | C4B | complement C4B (Chido blood group) [Source:HGNC Symbol;Acc:HGNC:1324] |
| ENSG00000224440 | HGLibA_11938 | 4 | -1.098512118 | CXorf51A | chromosome X open reading frame 51A [Source:HGNC Symbol;Acc:HGNC:30533] |
| ENSG00000231637 | HGLibA_53268 | 4 | -1.098512118 | USP17L29 | ubiquitin specific peptidase 17-like family member 29 [Source:HGNC Symbol;Acc:HGNC:44457] |
| ENSG00000236125 | HGLibA_53277 | 4 | -1.098512118 | USP17L4 | ubiquitin specific peptidase 17-like family member 4 [Source:HGNC Symbol;Acc:HGNC:37176] |
| ENSG00000236438 | HGLibA_16191 | 4 | -1.098512118 | FAM157A | family with sequence similarity 157 member A [Source:HGNC Symbol;Acc:HGNC:34079] |
| ENSG00000240065 | HGLibA_39125 | 4 | -1.098512118 | PSMB9 | proteasome subunit beta 9 [Source:HGNC Symbol;Acc:HGNC:9546] |
| ENSG00000242173 | HGLibA_02802 | 4 | -1.098512118 | ARHGDIG | Rho GDP dissociation inhibitor gamma [Source:HGNC Symbol;Acc:HGNC:680] |
| ENSG00000247077 | HGLibA_36203 | 4 | -1.098512118 | PGAM5 | mitochondrial serine/threonine protein phosphatase [Source:HGNC Symbol;Acc:HGNC:28763]" |
| ENSG00000248920 | HGLibA_53235 | 4 | -1.098512118 | USP17L19 | ubiquitin specific peptidase 17-like family member 19 [Source:HGNC Symbol;Acc:HGNC:44447] |
| ENSG00000250479 | HGLibA_09362 | 4 | -1.098512118 | CHCHD10 | coiled-coil-helix-coiled-coil-helix domain containing 10 [Source:HGNC Symbol;Acc:HGNC:15559] |
| ENSG00000250571 | HGLibA_19257 | 4 | -1.098512118 | GLI4 | GLI family zinc finger 4 [Source:HGNC Symbol;Acc:HGNC:4320] |
| ENSG00000256269 | HGLibA_21668 | 4 | -1.098512118 | HMBS | hydroxymethylbilane synthase [Source:HGNC Symbol;Acc:HGNC:4982] |
| ENSG00000257365 | HGLibA_17774 | 4 | -1.098512118 | FNTB | beta [Source:HGNC Symbol;Acc:HGNC:3785]" |
| ENSG00000266524 | HGLibA_18854 | 4 | -1.098512118 | GDF10 | growth differentiation factor 10 [Source:HGNC Symbol;Acc:HGNC:4215] |
| ENSG00000266919 | HGLibA_59699 | 4 | -1.098512118 | hsa-mir-423 | microRNA 3184 |
| ENSG00000275302 | HGLibA_08027 | 4 | -1.098512118 | CCL4 | C-C motif chemokine ligand 4 [Source:HGNC Symbol;Acc:HGNC:10630] |
| ENSG00000285077 | HGLibA_02706 | 4 | -1.098512118 | ARHGAP11B | Rho GTPase activating protein 11B [Source:HGNC Symbol;Acc:HGNC:15782] |
| ENSG00000288722 | HGLibA_15885 | 4 | -1.098512118 | F8A1 | - |
| FAM26F | HGLibA_16566 | 4 | -1.098512118 | FAM26F | - |
| FLJ25363 | HGLibA_17625 | 4 | -1.098512118 | FLJ25363 | - |
| GAGE2B | HGLibA_18471 | 4 | -1.098512118 | GAGE2B | - |
| GAGE2C | HGLibA_18474 | 4 | -1.098512118 | GAGE2C | - |
| H3F3C | HGLibA_20783 | 4 | -1.098512118 | H3F3C | - |
| hsa-mir-1245b | HGLibA_57378 | 4 | -1.098512118 | hsa-mir-1245b | - |
| hsa-mir-1298 | HGLibA_57672 | 4 | -1.098512118 | hsa-mir-1298 | - |
| hsa-mir-16-2 | HGLibA_57950 | 4 | -1.098512118 | hsa-mir-16-2 | - |
| hsa-mir-183 | HGLibA_57993 | 4 | -1.098512118 | hsa-mir-183 | - |
| hsa-mir-3074 | HGLibA_58469 | 4 | -1.098512118 | hsa-mir-3074 | - |
| hsa-mir-4252 | HGLibA_59716 | 4 | -1.098512118 | hsa-mir-4252 | - |
| hsa-mir-4328 | HGLibA_60034 | 4 | -1.098512118 | hsa-mir-4328 | - |
| hsa-mir-4417 | HGLibA_60050 | 4 | -1.098512118 | hsa-mir-4417 | - |
| hsa-mir-4465 | HGLibA_60267 | 4 | -1.098512118 | hsa-mir-4465 | - |
| hsa-mir-4759 | HGLibA_61132 | 4 | -1.098512118 | hsa-mir-4759 | - |
| hsa-mir-4794 | HGLibA_61282 | 4 | -1.098512118 | hsa-mir-4794 | - |
| hsa-mir-513a-2 | HGLibA_61576 | 4 | -1.098512118 | hsa-mir-513a-2 | - |
| hsa-mir-5193 | HGLibA_61690 | 4 | -1.098512118 | hsa-mir-5193 | - |
| hsa-mir-523 | HGLibA_61778 | 4 | -1.098512118 | hsa-mir-523 | - |

| | | | | | |
|---|---|---|---|---|---|
| hsa-mir-5693 | HGLibA_62307 | 4 | -1.098512118 | hsa-mir-5693 | - |
| hsa-mir-5787 | HGLibA_62409 | 4 | -1.098512118 | hsa-mir-5787 | - |
| hsa-mir-6511a-2 | HGLibA_62901 | 4 | -1.098512118 | hsa-mir-6511a-2 | - |
| hsa-mir-6769b | HGLibA_63243 | 4 | -1.098512118 | hsa-mir-6769b | - |
| hsa-mir-769 | HGLibA_63934 | 4 | -1.098512118 | hsa-mir-769 | - |
| hsa-mir-8052 | HGLibA_64045 | 4 | -1.098512118 | hsa-mir-8052 | - |
| hsa-mir-885 | HGLibA_64224 | 4 | -1.098512118 | hsa-mir-885 | - |
| IL3RA_X | HGLibA_23159 | 4 | -1.098512118 | IL3RA_X | - |
| 4-Sep | HGLibA_43442 | 4 | -1.098512118 | 4-Sep | - |
| TMEM246 | HGLibA_50304 | 4 | -1.098512118 | TMEM246 | - |
| WDR78 | HGLibA_54183 | 4 | -1.098512118 | WDR78 | - |
| YAE1D1 | HGLibA_54624 | 4 | -1.098512118 | YAE1D1 | - |
| ANKRD20A3 | HGLibA_02032 | 3 | -1.107557461 | ANKRD20A3 | - |
| BAGE3 | HGLibA_04099 | 3 | -1.107557461 | BAGE3 | - |
| C14orf2 | HGLibA_05357 | 3 | -1.107557461 | C14orf2 | - |
| C19orf21 | HGLibA_05636 | 3 | -1.107557461 | C19orf21 | - |
| C3orf72 | HGLibA_06265 | 3 | -1.107557461 | C3orf72 | - |
| C3orf83 | HGLibA_06275 | 3 | -1.107557461 | C3orf83 | - |
| CCL4L1 | HGLibA_08031 | 3 | -1.107557461 | CCL4L1 | - |
| ENSG00000001084 | HGLibA_18793 | 3 | -1.107557461 | GCLC | glutamate-cysteine ligase catalytic subunit [Source:HGNC Symbol;Acc:HGNC:4311] |
| ENSG00000001631 | HGLibA_25407 | 3 | -1.107557461 | KRIT1 | ankyrin repeat containing [Source:HGNC Symbol;Acc:HGNC:1573]" |
| ENSG00000006712 | HGLibA_34987 | 3 | -1.107557461 | PAF1 | Paf1/RNA polymerase II complex component [Source:HGNC Symbol;Acc:HGNC:25459]" |
| ENSG00000008283 | HGLibA_11976 | 3 | -1.107557461 | CYB561 | cytochrome b561 [Source:HGNC Symbol;Acc:HGNC:2571] |
| ENSG00000008294 | HGLibA_46373 | 3 | -1.107557461 | SPAG9 | sperm associated antigen 9 [Source:HGNC Symbol;Acc:HGNC:14524] |
| ENSG00000013523 | HGLibA_01896 | 3 | -1.107557461 | ANGEL1 | angel homolog 1 [Source:HGNC Symbol;Acc:HGNC:19961] |
| ENSG00000019549 | HGLibA_45868 | 3 | -1.107557461 | SNAI2 | snail family transcriptional repressor 2 [Source:HGNC Symbol;Acc:HGNC:11094] |
| ENSG00000030304 | HGLibA_30578 | 3 | -1.107557461 | MUSK | muscle associated receptor tyrosine kinase [Source:HGNC Symbol;Acc:HGNC:7525] |
| ENSG00000042445 | HGLibA_40880 | 3 | -1.107557461 | RETSAT | retinol saturase [Source:HGNC Symbol;Acc:HGNC:25991] |
| ENSG00000047365 | HGLibA_02628 | 3 | -1.107557461 | ARAP2 | ankyrin repeat and PH domain 2 [Source:HGNC Symbol;Acc:HGNC:16924]" |
| ENSG00000059588 | HGLibA_48330 | 3 | -1.107557461 | TARBP1 | TAR (HIV-1) RNA binding protein 1 [Source:HGNC Symbol;Acc:HGNC:11568] |
| ENSG00000063176 | HGLibA_46646 | 3 | -1.107557461 | SPHK2 | sphingosine kinase 2 [Source:HGNC Symbol;Acc:HGNC:18859] |
| ENSG00000064726 | HGLibA_04894 | 3 | -1.107557461 | BTBD1 | BTB domain containing 1 [Source:HGNC Symbol;Acc:HGNC:1120] |
| ENSG00000069956 | HGLibA_28276 | 3 | -1.107557461 | MAPK6 | mitogen-activated protein kinase 6 [Source:HGNC Symbol;Acc:HGNC:6879] |
| ENSG00000075188 | HGLibA_33051 | 3 | -1.107557461 | NUP37 | nucleoporin 37 [Source:HGNC Symbol;Acc:HGNC:29929] |
| ENSG00000075407 | HGLibA_55885 | 3 | -1.107557461 | ZNF37A | zinc finger protein 37A [Source:HGNC Symbol;Acc:HGNC:13102] |
| ENSG00000081051 | HGLibA_01128 | 3 | -1.107557461 | AFP | alpha fetoprotein [Source:HGNC Symbol;Acc:HGNC:317] |
| ENSG00000085871 | HGLibA_29178 | 3 | -1.107557461 | MGST2 | microsomal glutathione S-transferase 2 [Source:HGNC Symbol;Acc:HGNC:7063] |
| ENSG00000086232 | HGLibA_14724 | 3 | -1.107557461 | EIF2AK1 | eukaryotic translation initiation factor 2 alpha kinase 1 [Source:HGNC Symbol;Acc:HGNC:24921] |
| ENSG00000086967 | HGLibA_30651 | 3 | -1.107557461 | MYBPC2 | fast type [Source:HGNC Symbol;Acc:HGNC:7550]" |
| ENSG00000089022 | HGLibA_28307 | 3 | -1.107557461 | MAPKAPK5 | mitogen-activated protein kinase-activated protein kinase 5 [Source:HGNC Symbol;Acc:HGNC:6889] |
| ENSG00000089902 | HGLibA_40709 | 3 | -1.107557461 | RCOR1 | REST corepressor 1 [Source:HGNC Symbol;Acc:HGNC:17441] |
| ENSG00000090612 | HGLibA_55706 | 3 | -1.107557461 | ZNF268 | zinc finger protein 268 [Source:HGNC Symbol;Acc:HGNC:13061] |
| ENSG00000099814 | HGLibA_09120 | 3 | -1.107557461 | CEP170B | centrosomal protein 170B [Source:HGNC Symbol;Acc:HGNC:20362] |
| ENSG00000100033 | HGLibA_38660 | 3 | -1.107557461 | PRODH | proline dehydrogenase 1 [Source:HGNC Symbol;Acc:HGNC:9453] |
| ENSG00000101361 | HGLibA_32313 | 3 | -1.107557461 | NOP56 | NOP56 ribonucleoprotein [Source:HGNC Symbol;Acc:HGNC:15911] |
| ENSG00000102763 | HGLibA_53918 | 3 | -1.107557461 | VWA8 | von Willebrand factor A domain containing 8 [Source:HGNC Symbol;Acc:HGNC:29071] |
| ENSG00000103449 | HGLibA_42647 | 3 | -1.107557461 | SALL1 | spalt like transcription factor 1 [Source:HGNC Symbol;Acc:HGNC:10524] |
| ENSG00000104671 | HGLibA_12558 | 3 | -1.107557461 | DCTN6 | dynactin subunit 6 [Source:HGNC Symbol;Acc:HGNC:16964] |
| ENSG00000104921 | HGLibA_17174 | 3 | -1.107557461 | FCER2 | Fc fragment of IgE receptor II [Source:HGNC Symbol;Acc:HGNC:3612] |
| ENSG00000106615 | HGLibA_41153 | 3 | -1.107557461 | RHEB | mTORC1 binding [Source:HGNC Symbol;Acc:HGNC:10011]" |
| ENSG00000108684 | HGLibA_03303 | 3 | -1.107557461 | ASIC2 | acid sensing ion channel subunit 2 [Source:HGNC Symbol;Acc:HGNC:99] |
| ENSG00000109458 | HGLibA_18320 | 3 | -1.107557461 | GAB1 | GRB2 associated binding protein 1 [Source:HGNC Symbol;Acc:HGNC:4066] |
| ENSG00000110713 | HGLibA_33078 | 3 | -1.107557461 | NUP98 | nucleoporin 98 [Source:HGNC Symbol;Acc:HGNC:8068] |
| ENSG00000111700 | HGLibA_45452 | 3 | -1.107557461 | SLCO1B3 | solute carrier organic anion transporter family member 1B3 [Source:HGNC Symbol;Acc:HGNC:10961] |
| ENSG00000112175 | HGLibA_04611 | 3 | -1.107557461 | BMP5 | bone morphogenetic protein 5 [Source:HGNC Symbol;Acc:HGNC:1072] |
| ENSG00000112319 | HGLibA_15827 | 3 | -1.107557461 | EYA4 | EYA transcriptional coactivator and phosphatase 4 [Source:HGNC Symbol;Acc:HGNC:3522] |
| ENSG00000112936 | HGLibA_06519 | 3 | -1.107557461 | C7 | complement C7 [Source:HGNC Symbol;Acc:HGNC:1346] |
| ENSG00000113119 | HGLibA_49797 | 3 | -1.107557461 | TMCO6 | transmembrane and coiled-coil domains 6 [Source:HGNC Symbol;Acc:HGNC:28814] |
| ENSG00000113318 | HGLibA_30194 | 3 | -1.107557461 | MSH3 | mutS homolog 3 [Source:HGNC Symbol;Acc:HGNC:7326] |
| ENSG00000115234 | HGLibA_46047 | 3 | -1.107557461 | SNX17 | sorting nexin 17 [Source:HGNC Symbol;Acc:HGNC:14979] |
| ENSG00000115290 | HGLibA_20227 | 3 | -1.107557461 | GRB14 | growth factor receptor bound protein 14 [Source:HGNC Symbol;Acc:HGNC:4565] |
| ENSG00000116984 | HGLibA_30463 | 3 | -1.107557461 | MTR | 5-methyltetrahydrofolate-homocysteine methyltransferase [Source:HGNC Symbol;Acc:HGNC:7468] |
| ENSG00000118113 | HGLibA_29539 | 3 | -1.107557461 | MMP8 | matrix metallopeptidase 8 [Source:HGNC Symbol;Acc:HGNC:7175] |
| ENSG00000120925 | HGLibA_41574 | 3 | -1.107557461 | RNF170 | ring finger protein 170 [Source:HGNC Symbol;Acc:HGNC:25358] |
| ENSG00000122861 | HGLibA_36964 | 3 | -1.107557461 | PLAU | urokinase [Source:HGNC Symbol;Acc:HGNC:9052]" |
| ENSG00000123080 | HGLibA_08841 | 3 | -1.107557461 | CDKN2C | cyclin dependent kinase inhibitor 2C [Source:HGNC Symbol;Acc:HGNC:1789] |
| ENSG00000123552 | HGLibA_53375 | 3 | -1.107557461 | USP45 | ubiquitin specific peptidase 45 [Source:HGNC Symbol;Acc:HGNC:20080] |
| ENSG00000124120 | HGLibA_52225 | 3 | -1.107557461 | TTPAL | alpha tocopherol transfer protein like [Source:HGNC Symbol;Acc:HGNC:16114] |
| ENSG00000124214 | HGLibA_47404 | 3 | -1.107557461 | STAU1 | staufen double-stranded RNA binding protein 1 [Source:HGNC Symbol;Acc:HGNC:11370] |
| ENSG00000124225 | HGLibA_37300 | 3 | -1.107557461 | PMEPA1 | androgen induced 1 [Source:HGNC Symbol;Acc:HGNC:14107]" |
| ENSG00000124256 | HGLibA_54808 | 3 | -1.107557461 | ZBP1 | Z-DNA binding protein 1 [Source:HGNC Symbol;Acc:HGNC:16176] |
| ENSG00000124659 | HGLibA_48583 | 3 | -1.107557461 | TBCC | tubulin folding cofactor C [Source:HGNC Symbol;Acc:HGNC:11580] |
| ENSG00000124702 | HGLibA_25099 | 3 | -1.107557461 | KLHDC3 | kelch domain containing 3 [Source:HGNC Symbol;Acc:HGNC:20704] |
| ENSG00000124875 | HGLibA_11874 | 3 | -1.107557461 | CXCL6 | C-X-C motif chemokine ligand 6 [Source:HGNC Symbol;Acc:HGNC:10643] |
| ENSG00000125657 | HGLibA_50828 | 3 | -1.107557461 | TNFSF9 | TNF superfamily member 9 [Source:HGNC Symbol;Acc:HGNC:11939] |
| ENSG00000126787 | HGLibA_13352 | 3 | -1.107557461 | DLGAP5 | DLG associated protein 5 [Source:HGNC Symbol;Acc:HGNC:16864] |
| ENSG00000128285 | HGLibA_28588 | 3 | -1.107557461 | MCHR1 | melanin concentrating hormone receptor 1 [Source:HGNC Symbol;Acc:HGNC:4479] |
| ENSG00000130119 | HGLibA_19554 | 3 | -1.107557461 | GNL3L | G protein nucleolar 3 like [Source:HGNC Symbol;Acc:HGNC:25553] |
| ENSG00000130487 | HGLibA_25108 | 3 | -1.107557461 | KLHDC7B | kelch domain containing 7B [Source:HGNC Symbol;Acc:HGNC:25145] |
| ENSG00000131848 | HGLibA_57029 | 3 | -1.107557461 | ZSCAN5A | zinc finger and SCAN domain containing 5A [Source:HGNC Symbol;Acc:HGNC:23710] |
| ENSG00000135503 | HGLibA_00690 | 3 | -1.107557461 | ACVR1B | activin A receptor type 1B [Source:HGNC Symbol;Acc:HGNC:172] |
| ENSG00000136147 | HGLibA_36337 | 3 | -1.107557461 | PHF11 | PHD finger protein 11 [Source:HGNC Symbol;Acc:HGNC:17024] |
| ENSG00000136213 | HGLibA_09619 | 3 | -1.107557461 | CHST12 | carbohydrate sulfotransferase 12 [Source:HGNC Symbol;Acc:HGNC:17423] |
| ENSG00000137693 | HGLibA_54632 | 3 | -1.107557461 | YAP1 | Yes associated protein 1 [Source:HGNC Symbol;Acc:HGNC:16262] |
| ENSG00000137845 | HGLibA_00727 | 3 | -1.107557461 | ADAM10 | ADAM metallopeptidase domain 10 [Source:HGNC Symbol;Acc:HGNC:188] |
| ENSG00000138134 | HGLibA_47339 | 3 | -1.107557461 | STAMBPL1 | STAM binding protein like 1 [Source:HGNC Symbol;Acc:HGNC:24105] |
| ENSG00000138778 | HGLibA_09050 | 3 | -1.107557461 | CENPE | centromere protein E [Source:HGNC Symbol;Acc:HGNC:1856] |
| ENSG00000139304 | HGLibA_39528 | 3 | -1.107557461 | PTPRQ | receptor type Q [Source:HGNC Symbol;Acc:HGNC:9679]" |
| ENSG00000140015 | HGLibA_24222 | 3 | -1.107557461 | KCNH5 | potassium voltage-gated channel subfamily H member 5 [Source:HGNC Symbol;Acc:HGNC:6254] |
| ENSG00000143028 | HGLibA_48039 | 3 | -1.107557461 | SYPL2 | synaptophysin like 2 [Source:HGNC Symbol;Acc:HGNC:27638] |
| ENSG00000143106 | HGLibA_39082 | 3 | -1.107557461 | PSMA5 | proteasome subunit alpha 5 [Source:HGNC Symbol;Acc:HGNC:9534] |
| ENSG00000143772 | HGLibA_23846 | 3 | -1.107557461 | ITPKB | inositol-trisphosphate 3-kinase B [Source:HGNC Symbol;Acc:HGNC:6179] |
| ENSG00000144857 | HGLibA_04664 | 3 | -1.107557461 | BOC | oncogene regulated [Source:HGNC Symbol;Acc:HGNC:17173]" |
| ENSG00000146049 | HGLibA_24015 | 3 | -1.107557461 | KAAG1 | kidney associated antigen 1 [Source:HGNC Symbol;Acc:HGNC:21031] |
| ENSG00000147118 | HGLibA_55544 | 3 | -1.107557461 | ZNF182 | zinc finger protein 182 [Source:HGNC Symbol;Acc:HGNC:13001] |
| ENSG00000147262 | HGLibA_19867 | 3 | -1.107557461 | GPR119 | G protein-coupled receptor 119 [Source:HGNC Symbol;Acc:HGNC:19060] |
| ENSG00000148677 | HGLibA_01987 | 3 | -1.107557461 | ANKRD1 | ankyrin repeat domain 1 [Source:HGNC Symbol;Acc:HGNC:15819] |
| ENSG00000148795 | HGLibA_12061 | 3 | -1.107557461 | CYP17A1 | cytochrome P450 family 17 subfamily A member 1 [Source:HGNC Symbol;Acc:HGNC:2593] |
| ENSG00000149313 | HGLibA_00079 | 3 | -1.107557461 | AASDHPPT | aminoadipate-semialdehyde dehydrogenase-phosphopantetheinyl transferase [Source:HGNC Symbol;Acc:HGNC:14235] |
| ENSG00000149534 | HGLibA_30144 | 3 | -1.107557461 | MS4A2 | membrane spanning 4-domains A2 [Source:HGNC Symbol;Acc:HGNC:7316] |
| ENSG00000149657 | HGLibA_27534 | 3 | -1.107557461 | LSM14B | LSM family member 14B [Source:HGNC Symbol;Acc:HGNC:15887] |
| ENSG00000150455 | HGLibA_49566 | 3 | -1.107557461 | TIRAP | TIR domain containing adaptor protein [Source:HGNC Symbol;Acc:HGNC:17192] |
| ENSG00000150967 | HGLibA_00156 | 3 | -1.107557461 | ABCB9 | ATP binding cassette subfamily B member 9 [Source:HGNC Symbol;Acc:HGNC:50] |
| ENSG00000151718 | HGLibA_54441 | 3 | -1.107557461 | WWC2 | WW and C2 domain containing 2 [Source:HGNC Symbol;Acc:HGNC:24148] |
| ENSG00000152433 | HGLibA_56204 | 3 | -1.107557461 | ZNF547 | zinc finger protein 547 [Source:HGNC Symbol;Acc:HGNC:26432] |
| ENSG00000153485 | HGLibA_50318 | 3 | -1.107557461 | TMEM251 | transmembrane protein 251 [Source:HGNC Symbol;Acc:HGNC:20218] |
| ENSG00000154127 | HGLibA_52562 | 3 | -1.107557461 | UBASH3B | ubiquitin associated and SH3 domain containing B [Source:HGNC Symbol;Acc:HGNC:29884] |
| ENSG00000154553 | HGLibA_35914 | 3 | -1.107557461 | PDLIM3 | PDZ and LIM domain 3 [Source:HGNC Symbol;Acc:HGNC:20767] |
| ENSG00000154640 | HGLibA_04948 | 3 | -1.107557461 | BTG3 | BTG anti-proliferation factor 3 [Source:HGNC Symbol;Acc:HGNC:1132] |
| ENSG00000156639 | HGLibA_55152 | 3 | -1.107557461 | ZFAND3 | zinc finger AN1-type containing 3 [Source:HGNC Symbol;Acc:HGNC:18019] |
| ENSG00000159322 | HGLibA_01023 | 3 | -1.107557461 | ADPGK | ADP dependent glucokinase [Source:HGNC Symbol;Acc:HGNC:25250] |
| ENSG00000161896 | HGLibA_23452 | 3 | -1.107557461 | IP6K3 | inositol hexakisphosphate kinase 3 [Source:HGNC Symbol;Acc:HGNC:17269] |
| ENSG00000162444 | HGLibA_40645 | 3 | -1.107557461 | RBP7 | retinol binding protein 7 [Source:HGNC Symbol;Acc:HGNC:30316] |
| ENSG00000162723 | HGLibA_44325 | 3 | -1.107557461 | SLAMF9 | SLAM family member 9 [Source:HGNC Symbol;Acc:HGNC:18430] |
| ENSG00000163795 | HGLibA_56136 | 3 | -1.107557461 | ZNF513 | zinc finger protein 513 [Source:HGNC Symbol;Acc:HGNC:26498] |
| ENSG00000163923 | HGLibA_41976 | 3 | -1.107557461 | RPL39L | ribosomal protein L39 like [Source:HGNC Symbol;Acc:HGNC:17094] |
| ENSG00000163993 | HGLibA_42584 | 3 | -1.107557461 | S100P | S100 calcium binding protein P [Source:HGNC Symbol;Acc:HGNC:10504] |
| ENSG00000164168 | HGLibA_50118 | 3 | -1.107557461 | TMEM184C | transmembrane protein 184C [Source:HGNC Symbol;Acc:HGNC:25587] |
| ENSG00000164691 | HGLibA_48267 | 3 | -1.107557461 | TAGAP | T cell activation RhoGTPase activating protein [Source:HGNC Symbol;Acc:HGNC:15669] |
| ENSG00000164778 | HGLibA_15100 | 3 | -1.107557461 | EN2 | engrailed homeobox 2 [Source:HGNC Symbol;Acc:HGNC:3343] |
| ENSG00000165259 | HGLibA_21062 | 3 | -1.107557461 | HDX | highly divergent homeobox [Source:HGNC Symbol;Acc:HGNC:26411] |
| ENSG00000165929 | HGLibA_48685 | 3 | -1.107557461 | TC2N | nuclear [Source:HGNC Symbol;Acc:HGNC:19859]" |
| ENSG00000166289 | HGLibA_37096 | 3 | -1.107557461 | PLEKHF1 | pleckstrin homology and FYVE domain containing 1 [Source:HGNC Symbol;Acc:HGNC:20764] |
| ENSG00000167118 | HGLibA_53149 | 3 | -1.107557461 | URM1 | ubiquitin related modifier 1 [Source:HGNC Symbol;Acc:HGNC:28378] |
| ENSG00000167139 | HGLibA_48499 | 3 | -1.107557461 | TBC1D21 | TBC1 domain family member 21 [Source:HGNC Symbol;Acc:HGNC:28536] |
| ENSG00000167186 | HGLibA_10763 | 3 | -1.107557461 | COQ7 | hydroxylase [Source:HGNC Symbol;Acc:HGNC:2244]" |
| ENSG00000167261 | HGLibA_13821 | 3 | -1.107557461 | DPEP2 | dipeptidase 2 [Source:HGNC Symbol;Acc:HGNC:23028] |
| ENSG00000167612 | HGLibA_02068 | 3 | -1.107557461 | ANKRD33 | ankyrin repeat domain 33 [Source:HGNC Symbol;Acc:HGNC:13788] |
| ENSG00000167693 | HGLibA_33142 | 3 | -1.107557461 | NXN | nucleoredoxin [Source:HGNC Symbol;Acc:HGNC:18008] |
| ENSG00000168297 | HGLibA_39654 | 3 | -1.107557461 | PXK | PX domain containing serine/threonine kinase like [Source:HGNC Symbol;Acc:HGNC:23326] |
| ENSG00000168488 | HGLibA_03839 | 3 | -1.107557461 | ATXN2L | ataxin 2 like [Source:HGNC Symbol;Acc:HGNC:31326] |
| ENSG00000168556 | HGLibA_23286 | 3 | -1.107557461 | ING2 | inhibitor of growth family member 2 [Source:HGNC Symbol;Acc:HGNC:6063] |
| ENSG00000169006 | HGLibA_32889 | 3 | -1.107557461 | NTSR2 | neurotensin receptor 2 [Source:HGNC Symbol;Acc:HGNC:8040] |
| ENSG00000169282 | HGLibA_24146 | 3 | -1.107557461 | KCNAB1 | potassium voltage-gated channel subfamily A member regulatory beta subunit 1 [Source:HGNC Symbol;Acc:HGNC:6228] |
| ENSG00000169800 | HGLibA_40622 | 3 | -1.107557461 | RBMY1F | member F [Source:HGNC Symbol;Acc:HGNC:23974]" |

| | | | | | |
|---|---|---|---|---|---|
| ENSG00000170296 | HGLibA_18333 | 3 | -1.107557461 | GABARAP | GABA type A receptor-associated protein [Source:HGNC Symbol;Acc:HGNC:4067] |
| ENSG00000172296 | HGLibA_46897 | 3 | -1.107557461 | SPTLC3 | serine palmitoyltransferase long chain base subunit 3 [Source:HGNC Symbol;Acc:HGNC:16253] |
| ENSG00000172324 | HGLibA_34233 | 3 | -1.107557461 | OR5A2 | olfactory receptor family 5 subfamily A member 2 [Source:HGNC Symbol;Acc:HGNC:15249] |
| ENSG00000172345 | HGLibA_47366 | 3 | -1.107557461 | STARD5 | StAR related lipid transfer domain containing 5 [Source:HGNC Symbol;Acc:HGNC:18065] |
| ENSG00000172748 | HGLibA_56350 | 3 | -1.107557461 | ZNF596 | zinc finger protein 596 [Source:HGNC Symbol;Acc:HGNC:27268] |
| ENSG00000173110 | HGLibA_22291 | 3 | -1.107557461 | HSPA6 | heat shock protein family A (Hsp70) member 6 [Source:HGNC Symbol;Acc:HGNC:5239] |
| ENSG00000173376 | HGLibA_31402 | 3 | -1.107557461 | NDNF | neuron derived neurotrophic factor [Source:HGNC Symbol;Acc:HGNC:26256] |
| ENSG00000173406 | HGLibA_12264 | 3 | -1.107557461 | DAB1 | reelin adaptor protein [Source:HGNC Symbol;Acc:HGNC:2661]" |
| ENSG00000174225 | HGLibA_02931 | 3 | -1.107557461 | ARL13A | ADP ribosylation factor like GTPase 13A [Source:HGNC Symbol;Acc:HGNC:31709] |
| ENSG00000175325 | HGLibA_38685 | 3 | -1.107557461 | PROP1 | PROP paired-like homeobox 1 [Source:HGNC Symbol;Acc:HGNC:9455] |
| ENSG00000175426 | HGLibA_35666 | 3 | -1.107557461 | PCSK1 | proprotein convertase subtilisin/kexin type 1 [Source:HGNC Symbol;Acc:HGNC:8743] |
| ENSG00000175591 | HGLibA_34886 | 3 | -1.107557461 | P2RY2 | purinergic receptor P2Y2 [Source:HGNC Symbol;Acc:HGNC:8541] |
| ENSG00000177398 | HGLibA_52981 | 3 | -1.107557461 | UMODL1 | uromodulin like 1 [Source:HGNC Symbol;Acc:HGNC:12560] |
| ENSG00000177479 | HGLibA_02915 | 3 | -1.107557461 | ARIH2 | ariadne RBR E3 ubiquitin protein ligase 2 [Source:HGNC Symbol;Acc:HGNC:690] |
| ENSG00000177556 | HGLibA_03541 | 3 | -1.107557461 | ATOX1 | antioxidant 1 copper chaperone [Source:HGNC Symbol;Acc:HGNC:798] |
| ENSG00000178233 | HGLibA_50002 | 3 | -1.107557461 | TMEM151B | transmembrane protein 151B [Source:HGNC Symbol;Acc:HGNC:21315] |
| ENSG00000180113 | HGLibA_48910 | 3 | -1.107557461 | TDRD6 | tudor domain containing 6 [Source:HGNC Symbol;Acc:HGNC:21339] |
| ENSG00000180357 | HGLibA_56376 | 3 | -1.107557461 | ZNF609 | zinc finger protein 609 [Source:HGNC Symbol;Acc:HGNC:29003] |
| ENSG00000181785 | HGLibA_34251 | 3 | -1.107557461 | OR5AS1 | olfactory receptor family 5 subfamily AS member 1 [Source:HGNC Symbol;Acc:HGNC:15261] |
| ENSG00000183615 | HGLibA_16242 | 3 | -1.107557461 | FAM167B | family with sequence similarity 167 member B [Source:HGNC Symbol;Acc:HGNC:28133] |
| ENSG00000183914 | HGLibA_13483 | 3 | -1.107557461 | DNAH2 | dynein axonemal heavy chain 2 [Source:HGNC Symbol;Acc:HGNC:2948] |
| ENSG00000184502 | HGLibA_18661 | 3 | -1.107557461 | GAST | gastrin [Source:HGNC Symbol;Acc:HGNC:4164] |
| ENSG00000185313 | HGLibA_42968 | 3 | -1.107557461 | SCN10A | sodium voltage-gated channel alpha subunit 10 [Source:HGNC Symbol;Acc:HGNC:10582] |
| ENSG00000185753 | HGLibA_11921 | 3 | -1.107557461 | CXorf38 | chromosome X open reading frame 38 [Source:HGNC Symbol;Acc:HGNC:28589] |
| ENSG00000186001 | HGLibA_27143 | 3 | -1.107557461 | LRCH3 | leucine rich repeats and calponin homology domain containing 3 [Source:HGNC Symbol;Acc:HGNC:28637] |
| ENSG00000186431 | HGLibA_17166 | 3 | -1.107557461 | FCAR | Fc fragment of IgA receptor [Source:HGNC Symbol;Acc:HGNC:3608] |
| ENSG00000186575 | HGLibA_31761 | 3 | -1.107557461 | NF2 | neurofibromin 2 [Source:HGNC Symbol;Acc:HGNC:7773] |
| ENSG00000187172 | HGLibA_04096 | 3 | -1.107557461 | BAGE2 | BAGE family member 2 [Source:HGNC Symbol;Acc:HGNC:15723] |
| ENSG00000187741 | HGLibA_16829 | 3 | -1.107557461 | FANCA | FA complementation group A [Source:HGNC Symbol;Acc:HGNC:3582] |
| ENSG00000188624 | HGLibA_22845 | 3 | -1.107557461 | IGFL3 | IGF like family member 3 [Source:HGNC Symbol;Acc:HGNC:32930] |
| ENSG00000188747 | HGLibA_32383 | 3 | -1.107557461 | NOXA1 | NADPH oxidase activator 1 [Source:HGNC Symbol;Acc:HGNC:10668] |
| ENSG00000196091 | HGLibA_30648 | 3 | -1.107557461 | MYBPC1 | slow type [Source:HGNC Symbol;Acc:HGNC:7549]" |
| ENSG00000197798 | HGLibA_16052 | 3 | -1.107557461 | FAM118B | family with sequence similarity 118 member B [Source:HGNC Symbol;Acc:HGNC:26110] |
| ENSG00000197798 | HGLibA_16050 | 3 | -1.107557461 | FAM118B | family with sequence similarity 118 member B [Source:HGNC Symbol;Acc:HGNC:26110] |
| ENSG00000198331 | HGLibA_22459 | 3 | -1.107557461 | HYLS1 | centriolar and ciliogenesis associated [Source:HGNC Symbol;Acc:HGNC:26558]" |
| ENSG00000198535 | HGLibA_06096 | 3 | -1.107557461 | C2CD4A | C2 calcium dependent domain containing 4A [Source:HGNC Symbol;Acc:HGNC:33627] |
| ENSG00000198556 | HGLibA_56757 | 3 | -1.107557461 | ZNF789 | zinc finger protein 789 [Source:HGNC Symbol;Acc:HGNC:27801] |
| ENSG00000198783 | HGLibA_56833 | 3 | -1.107557461 | ZNF830 | zinc finger protein 830 [Source:HGNC Symbol;Acc:HGNC:28291] |
| ENSG00000203879 | HGLibA_18880 | 3 | -1.107557461 | GDI1 | GDP dissociation inhibitor 1 [Source:HGNC Symbol;Acc:HGNC:4226] |
| ENSG00000204613 | HGLibA_51403 | 3 | -1.107557461 | TRIM10 | tripartite motif containing 10 [Source:HGNC Symbol;Acc:HGNC:10072] |
| ENSG00000205208 | HGLibA_06334 | 3 | -1.107557461 | C4orf46 | chromosome 4 open reading frame 46 [Source:HGNC Symbol;Acc:HGNC:27320] |
| ENSG00000205323 | HGLibA_42746 | 3 | -1.107557461 | SARNP | SAP domain containing ribonucleoprotein [Source:HGNC Symbol;Acc:HGNC:24432] |
| ENSG00000212710 | HGLibA_11566 | 3 | -1.107557461 | CTAGE1 | cutaneous T cell lymphoma-associated antigen 1 [Source:HGNC Symbol;Acc:HGNC:24346] |
| ENSG00000213347 | HGLibA_30612 | 3 | -1.107557461 | MXD3 | MAX dimerization protein 3 [Source:HGNC Symbol;Acc:HGNC:14008] |
| ENSG00000213380 | HGLibA_10490 | 3 | -1.107557461 | COG8 | component of oligomeric golgi complex 8 [Source:HGNC Symbol;Acc:HGNC:18623] |
| ENSG00000213380 | HGLibA_10492 | 3 | -1.107557461 | COG8 | component of oligomeric golgi complex 8 [Source:HGNC Symbol;Acc:HGNC:18623] |
| ENSG00000213689 | HGLibA_51371 | 3 | -1.107557461 | TREX1 | three prime repair exonuclease 1 [Source:HGNC Symbol;Acc:HGNC:12269] |
| ENSG00000213920 | HGLibA_28705 | 3 | -1.107557461 | MDP1 | magnesium dependent phosphatase 1 [Source:HGNC Symbol;Acc:HGNC:28781] |
| ENSG00000214160 | HGLibA_01627 | 3 | -1.107557461 | ALG3 | 3- mannosyltransferase [Source:HGNC Symbol;Acc:HGNC:23056]" |
| ENSG00000221883 | HGLibA_02918 | 3 | -1.107557461 | ARIH2OS | ariadne RBR E3 ubiquitin protein ligase 2 opposite strand [Source:HGNC Symbol;Acc:HGNC:34425] |
| ENSG00000221995 | HGLibA_49433 | 3 | -1.107557461 | TIAF1 | TGFB1-induced anti-apoptotic factor 1 [Source:HGNC Symbol;Acc:HGNC:11803] |
| ENSG00000224659 | HGLibA_18462 | 3 | -1.107557461 | GAGE12J | G antigen 12J [Source:HGNC Symbol;Acc:HGNC:17778] |
| ENSG00000233670 | HGLibA_36731 | 3 | -1.107557461 | PIRT | phosphoinositide interacting regulator of transient receptor potential channels [Source:HGNC Symbol;Acc:HGNC:37239] |
| ENSG00000235194 | HGLibA_38111 | 3 | -1.107557461 | PPP1R3E | protein phosphatase 1 regulatory subunit 3E [Source:HGNC Symbol;Acc:HGNC:14943] |
| ENSG00000239886 | HGLibA_25833 | 3 | -1.107557461 | KRTAP9-2 | keratin associated protein 9-2 [Source:HGNC Symbol;Acc:HGNC:16926] |
| ENSG00000239998 | HGLibA_26492 | 3 | -1.107557461 | LILRA2 | leukocyte immunoglobulin like receptor A2 [Source:HGNC Symbol;Acc:HGNC:6603] |
| ENSG00000243130 | HGLibA_39037 | 3 | -1.107557461 | PSG11 | pregnancy specific beta-1-glycoprotein 11 [Source:HGNC Symbol;Acc:HGNC:9516] |
| ENSG00000250745 | HGLibA_53244 | 3 | -1.107557461 | USP17L20 | ubiquitin specific peptidase 17-like family member 20 [Source:HGNC Symbol;Acc:HGNC:44448] |
| ENSG00000271079 | HGLibA_11569 | 3 | -1.107557461 | CTAGE15 | CTAGE family member 15 [Source:HGNC Symbol;Acc:HGNC:37295] |
| ENSG00000275793 | HGLibA_41261 | 3 | -1.107557461 | RIMBP3 | RIMS binding protein 3 [Source:HGNC Symbol;Acc:HGNC:29344] |
| ENSG00000276293 | HGLibA_36707 | 3 | -1.107557461 | PIP4K2B | phosphatidylinositol-5-phosphate 4-kinase type 2 beta [Source:HGNC Symbol;Acc:HGNC:8998] |
| ENSG00000288547 | HGLibA_06236 | 3 | -1.107557461 | C3orf36 | - |
| ENSG00000288709 | HGLibA_15888 | 3 | -1.107557461 | F8A2 | - |
| FAM19A2 | HGLibA_16398 | 3 | -1.107557461 | FAM19A2 | - |
| H2AFY | HGLibA_20764 | 3 | -1.107557461 | H2AFY | - |
| HGC6.3 | HGLibA_21235 | 3 | -1.107557461 | HGC6.3 | - |
| HIST1H3C | HGLibA_21462 | 3 | -1.107557461 | HIST1H3C | - |
| hsa-mir-101-1 | HGLibA_57152 | 3 | -1.107557461 | hsa-mir-101-1 | - |
| hsa-mir-1199 | HGLibA_57259 | 3 | -1.107557461 | hsa-mir-1199 | - |
| hsa-mir-1289-1 | HGLibA_57621 | 3 | -1.107557461 | hsa-mir-1289-1 | - |
| hsa-mir-149 | HGLibA_57888 | 3 | -1.107557461 | hsa-mir-149 | - |
| hsa-mir-26a-1 | HGLibA_58364 | 3 | -1.107557461 | hsa-mir-26a-1 | - |
| hsa-mir-3142 | HGLibA_58627 | 3 | -1.107557461 | hsa-mir-3142 | - |
| hsa-mir-3182 | HGLibA_58825 | 3 | -1.107557461 | hsa-mir-3182 | - |
| hsa-mir-3182 | HGLibA_58824 | 3 | -1.107557461 | hsa-mir-3182 | - |
| hsa-mir-3675 | HGLibA_59253 | 3 | -1.107557461 | hsa-mir-3675 | - |
| hsa-mir-3913-2 | HGLibA_59526 | 3 | -1.107557461 | hsa-mir-3913-2 | - |
| hsa-mir-4439 | HGLibA_60160 | 3 | -1.107557461 | hsa-mir-4439 | - |
| hsa-mir-4478 | HGLibA_60325 | 3 | -1.107557461 | hsa-mir-4478 | - |
| hsa-mir-4771-1 | HGLibA_61179 | 3 | -1.107557461 | hsa-mir-4771-1 | - |
| hsa-mir-501 | HGLibA_61451 | 3 | -1.107557461 | hsa-mir-501 | - |
| hsa-mir-548a-1 | HGLibA_61836 | 3 | -1.107557461 | hsa-mir-548a-1 | - |
| hsa-mir-548am | HGLibA_61901 | 3 | -1.107557461 | hsa-mir-548am | - |
| hsa-mir-548i-3 | HGLibA_62022 | 3 | -1.107557461 | hsa-mir-548i-3 | - |
| hsa-mir-548s | HGLibA_62063 | 3 | -1.107557461 | hsa-mir-548s | - |
| hsa-mir-5571 | HGLibA_62147 | 3 | -1.107557461 | hsa-mir-5571 | - |
| hsa-mir-559 | HGLibA_62199 | 3 | -1.107557461 | hsa-mir-559 | - |
| hsa-mir-6511a-1 | HGLibA_62897 | 3 | -1.107557461 | hsa-mir-6511a-1 | - |
| hsa-mir-759 | HGLibA_63896 | 3 | -1.107557461 | hsa-mir-759 | - |
| KIAA1715 | HGLibA_24809 | 3 | -1.107557461 | KIAA1715 | - |
| LOC100132146 | HGLibA_26805 | 3 | -1.107557461 | LOC100132146 | - |
| LOC158434 | HGLibA_26899 | 3 | -1.107557461 | LOC158434 | - |
| LRMP | HGLibA_27195 | 3 | -1.107557461 | LRMP | - |
| LRRC48 | HGLibA_27373 | 3 | -1.107557461 | LRRC48 | - |
| 7-Sep | HGLibA_43451 | 3 | -1.107557461 | 7-Sep | - |
| SKIV2L2 | HGLibA_44288 | 3 | -1.107557461 | SKIV2L2 | - |
| ATP5J2-PTCD1 | HGLibA_03675 | 2 | -1.116602804 | ATP5J2-PTCD1 | - |
| C1QTNF9B-AS1 | HGLibA_05966 | 2 | -1.116602804 | C1QTNF9B-AS1 | - |
| C3orf27 | HGLibA_06224 | 2 | -1.116602804 | C3orf27 | - |
| C5orf28 | HGLibA_06370 | 2 | -1.116602804 | C5orf28 | - |
| C9orf69 | HGLibA_06755 | 2 | -1.116602804 | C9orf69 | - |
| CTGF | HGLibA_11623 | 2 | -1.116602804 | CTGF | - |
| ENSG00000006282 | HGLibA_46448 | 2 | -1.116602804 | SPATA20 | spermatogenesis associated 20 [Source:HGNC Symbol;Acc:HGNC:26125] |
| ENSG00000007520 | HGLibA_52000 | 2 | -1.116602804 | TSR3 | acp transferase ribosome maturation factor [Source:HGNC Symbol;Acc:HGNC:14175]" |
| ENSG00000010932 | HGLibA_17703 | 2 | -1.116602804 | FMO1 | flavin containing monooxygenase 1 [Source:HGNC Symbol;Acc:HGNC:3769] |
| ENSG00000011009 | HGLibA_27739 | 2 | -1.116602804 | LYPLA2 | lysophospholipase II [Source:HGNC Symbol;Acc:HGNC:6738] |
| ENSG00000021300 | HGLibA_37086 | 2 | -1.116602804 | PLEKHB1 | pleckstrin homology domain containing B1 [Source:HGNC Symbol;Acc:HGNC:19079] |
| ENSG00000021776 | HGLibA_02617 | 2 | -1.116602804 | AQR | aquarius intron-binding spliceosomal factor [Source:HGNC Symbol;Acc:HGNC:29513] |
| ENSG00000038002 | HGLibA_01133 | 2 | -1.116602804 | AGA | aspartylglucosaminidase [Source:HGNC Symbol;Acc:HGNC:318] |
| ENSG00000039319 | HGLibA_55273 | 2 | -1.116602804 | ZFYVE16 | zinc finger FYVE-type containing 16 [Source:HGNC Symbol;Acc:HGNC:20756] |
| ENSG00000043093 | HGLibA_12566 | 2 | -1.116602804 | DCUN1D1 | defective in cullin neddylation 1 domain containing 1 [Source:HGNC Symbol;Acc:HGNC:18184] |
| ENSG00000053702 | HGLibA_32693 | 2 | -1.116602804 | NRIP2 | nuclear receptor interacting protein 2 [Source:HGNC Symbol;Acc:HGNC:23078] |
| ENSG00000054277 | HGLibA_33433 | 2 | -1.116602804 | OPN3 | opsin 3 [Source:HGNC Symbol;Acc:HGNC:14007] |
| ENSG00000056972 | HGLibA_51253 | 2 | -1.116602804 | TRAF3IP2 | TRAF3 interacting protein 2 [Source:HGNC Symbol;Acc:HGNC:1343] |
| ENSG00000059122 | HGLibA_17681 | 2 | -1.116602804 | FLYWCH1 | FLYWCH-type zinc finger 1 [Source:HGNC Symbol;Acc:HGNC:25404] |
| ENSG00000064218 | HGLibA_13422 | 2 | -1.116602804 | DMRT3 | doublesex and mab-3 related transcription factor 3 [Source:HGNC Symbol;Acc:HGNC:13909] |
| ENSG00000065485 | HGLibA_35884 | 2 | -1.116602804 | PDIA5 | protein disulfide isomerase family A member 5 [Source:HGNC Symbol;Acc:HGNC:24811] |
| ENSG00000065534 | HGLibA_30785 | 2 | -1.116602804 | MYLK | myosin light chain kinase [Source:HGNC Symbol;Acc:HGNC:7590] |
| ENSG00000065833 | HGLibA_28710 | 2 | -1.116602804 | ME1 | malic enzyme 1 [Source:HGNC Symbol;Acc:HGNC:6983] |
| ENSG00000065882 | HGLibA_48461 | 2 | -1.116602804 | TBC1D1 | TBC1 domain family member 1 [Source:HGNC Symbol;Acc:HGNC:11578] |
| ENSG00000068097 | HGLibA_21082 | 2 | -1.116602804 | HEATR6 | HEAT repeat containing 6 [Source:HGNC Symbol;Acc:HGNC:24076] |
| ENSG00000072041 | HGLibA_45304 | 2 | -1.116602804 | SLC6A15 | solute carrier family 6 member 15 [Source:HGNC Symbol;Acc:HGNC:13621] |
| ENSG00000073711 | HGLibA_38154 | 2 | -1.116602804 | PPP2R3A | protein phosphatase 2 regulatory subunit B''alpha [Source:HGNC Symbol;Acc:HGNC:9307] |
| ENSG00000076108 | HGLibA_04191 | 2 | -1.116602804 | BAZ2A | bromodomain adjacent to zinc finger domain 2A [Source:HGNC Symbol;Acc:HGNC:962] |
| ENSG00000076685 | HGLibA_32812 | 2 | -1.116602804 | NT5C2 | cytosolic II [Source:HGNC Symbol;Acc:HGNC:8022]" |
| ENSG00000078018 | HGLibA_28136 | 2 | -1.116602804 | MAP2 | microtubule associated protein 2 [Source:HGNC Symbol;Acc:HGNC:6839] |
| ENSG00000078295 | HGLibA_00910 | 2 | -1.116602804 | ADCY2 | adenylate cyclase 2 [Source:HGNC Symbol;Acc:HGNC:233] |
| ENSG00000079112 | HGLibA_08651 | 2 | -1.116602804 | CDH17 | cadherin 17 [Source:HGNC Symbol;Acc:HGNC:1756] |
| ENSG00000079482 | HGLibA_33416 | 2 | -1.116602804 | OPHN1 | oligophrenin 1 [Source:HGNC Symbol;Acc:HGNC:8148] |
| ENSG00000080603 | HGLibA_46931 | 2 | -1.116602804 | SRCAP | Snf2 related CREBBP activator protein [Source:HGNC Symbol;Acc:HGNC:16974] |
| ENSG00000082516 | HGLibA_18918 | 2 | -1.116602804 | GEMIN5 | gem nuclear organelle associated protein 5 [Source:HGNC Symbol;Acc:HGNC:20043] |
| ENSG00000083642 | HGLibA_35945 | 2 | -1.116602804 | PDS5B | PDS5 cohesin associated factor B [Source:HGNC Symbol;Acc:HGNC:20418] |
| ENSG00000087206 | HGLibA_52956 | 2 | -1.116602804 | UIMC1 | ubiquitin interaction motif containing 1 [Source:HGNC Symbol;Acc:HGNC:30298] |
| ENSG00000087842 | HGLibA_36728 | 2 | -1.116602804 | PIR | pirin [Source:HGNC Symbol;Acc:HGNC:30048] |
| ENSG00000089250 | HGLibA_32321 | 2 | -1.116602804 | NOS1 | nitric oxide synthase 1 [Source:HGNC Symbol;Acc:HGNC:7872] |

| | | | | | |
|---|---|---|---|---|---|
| ENSG00000089558 | HGLibA_24219 | 2 | -1.116602804 | KCNH4 | potassium voltage-gated channel subfamily H member 4 [Source:HGNC Symbol;Acc:HGNC:6253] |
| ENSG00000089692 | HGLibA_25927 | 2 | -1.116602804 | LAG3 | lymphocyte activating 3 [Source:HGNC Symbol;Acc:HGNC:6476] |
| ENSG00000090905 | HGLibA_50914 | 2 | -1.116602804 | TNRC6A | trinucleotide repeat containing 6A [Source:HGNC Symbol;Acc:HGNC:11969] |
| ENSG00000092140 | HGLibA_18297 | 2 | -1.116602804 | G2E3 | G2/M-phase specific E3 ubiquitin protein ligase [Source:HGNC Symbol;Acc:HGNC:20338] |
| ENSG00000095917 | HGLibA_51198 | 2 | -1.116602804 | TPSD1 | tryptase delta 1 [Source:HGNC Symbol;Acc:HGNC:14118] |
| ENSG00000096063 | HGLibA_47019 | 2 | -1.116602804 | SRPK1 | SRSF protein kinase 1 [Source:HGNC Symbol;Acc:HGNC:11305] |
| ENSG00000099994 | HGLibA_47848 | 2 | -1.116602804 | SUSD2 | sushi domain containing 2 [Source:HGNC Symbol;Acc:HGNC:30667] |
| ENSG00000100311 | HGLibA_35843 | 2 | -1.116602804 | PDGFB | platelet derived growth factor subunit B [Source:HGNC Symbol;Acc:HGNC:8800] |
| ENSG00000100336 | HGLibA_02536 | 2 | -1.116602804 | APOL4 | apolipoprotein L4 [Source:HGNC Symbol;Acc:HGNC:14867] |
| ENSG00000100485 | HGLibA_46204 | 2 | -1.116602804 | SOS2 | SOS Ras/Rho guanine nucleotide exchange factor 2 [Source:HGNC Symbol;Acc:HGNC:11188] |
| ENSG00000100911 | HGLibA_39192 | 2 | -1.116602804 | PSME2 | proteasome activator subunit 2 [Source:HGNC Symbol;Acc:HGNC:9569] |
| ENSG00000101290 | HGLibA_08884 | 2 | -1.116602804 | CDS2 | CDP-diacylglycerol synthase 2 [Source:HGNC Symbol;Acc:HGNC:1801] |
| ENSG00000101306 | HGLibA_30788 | 2 | -1.116602804 | MYLK2 | myosin light chain kinase 2 [Source:HGNC Symbol;Acc:HGNC:16243] |
| ENSG00000101608 | HGLibA_30750 | 2 | -1.116602804 | MYL12A | myosin light chain 12A [Source:HGNC Symbol;Acc:HGNC:16701] |
| ENSG00000101981 | HGLibA_15895 | 2 | -1.116602804 | F9 | coagulation factor IX [Source:HGNC Symbol;Acc:HGNC:3551] |
| ENSG00000102096 | HGLibA_36679 | 2 | -1.116602804 | PIM2 | serine/threonine kinase [Source:HGNC Symbol;Acc:HGNC:8987]" |
| ENSG00000102738 | HGLibA_30088 | 2 | -1.116602804 | MRPS31 | mitochondrial ribosomal protein S31 [Source:HGNC Symbol;Acc:HGNC:16632] |
| ENSG00000102967 | HGLibA_13111 | 2 | -1.116602804 | DHODH | dihydroorotate dehydrogenase (quinone) [Source:HGNC Symbol;Acc:HGNC:2867] |
| ENSG00000103710 | HGLibA_40372 | 2 | -1.116602804 | RASL12 | RAS like family 12 [Source:HGNC Symbol;Acc:HGNC:30289] |
| ENSG00000103742 | HGLibA_22788 | 2 | -1.116602804 | IGDCC4 | immunoglobulin superfamily DCC subclass member 4 [Source:HGNC Symbol;Acc:HGNC:13770] |
| ENSG00000104870 | HGLibA_17200 | 2 | -1.116602804 | FCGRT | Fc fragment of IgG receptor and transporter [Source:HGNC Symbol;Acc:HGNC:3621] |
| ENSG00000105750 | HGLibA_56869 | 2 | -1.116602804 | ZNF85 | zinc finger protein 85 [Source:HGNC Symbol;Acc:HGNC:13160] |
| ENSG00000105784 | HGLibA_42453 | 2 | -1.116602804 | RUNDC3B | RUN domain containing 3B [Source:HGNC Symbol;Acc:HGNC:30286] |
| ENSG00000106346 | HGLibA_53364 | 2 | -1.116602804 | USP42 | ubiquitin specific peptidase 42 [Source:HGNC Symbol;Acc:HGNC:20068] |
| ENSG00000106723 | HGLibA_46666 | 2 | -1.116602804 | SPIN1 | spindlin 1 [Source:HGNC Symbol;Acc:HGNC:11243] |
| ENSG00000107021 | HGLibA_48476 | 2 | -1.116602804 | TBC1D13 | TBC1 domain family member 13 [Source:HGNC Symbol;Acc:HGNC:25571] |
| ENSG00000107959 | HGLibA_36760 | 2 | -1.116602804 | PITRM1 | pitrilysin metallopeptidase 1 [Source:HGNC Symbol;Acc:HGNC:17663] |
| ENSG00000108064 | HGLibA_49117 | 2 | -1.116602804 | TFAM | mitochondrial [Source:HGNC Symbol;Acc:HGNC:11741]" |
| ENSG00000110148 | HGLibA_07957 | 2 | -1.116602804 | CCKBR | cholecystokinin B receptor [Source:HGNC Symbol;Acc:HGNC:1571] |
| ENSG00000110367 | HGLibA_12760 | 2 | -1.116602804 | DDX6 | DEAD-box helicase 6 [Source:HGNC Symbol;Acc:HGNC:2747] |
| ENSG00000110888 | HGLibA_07192 | 2 | -1.116602804 | CAPRIN2 | caprin family member 2 [Source:HGNC Symbol;Acc:HGNC:21259] |
| ENSG00000110921 | HGLibA_30598 | 2 | -1.116602804 | MVK | mevalonate kinase [Source:HGNC Symbol;Acc:HGNC:7530] |
| ENSG00000112249 | HGLibA_03264 | 2 | -1.116602804 | ASCC3 | activating signal cointegrator 1 complex subunit 3 [Source:HGNC Symbol;Acc:HGNC:18697] |
| ENSG00000112357 | HGLibA_36125 | 2 | -1.116602804 | PEX7 | peroxisomal biogenesis factor 7 [Source:HGNC Symbol;Acc:HGNC:8860] |
| ENSG00000112685 | HGLibA_15734 | 2 | -1.116602804 | EXOC2 | exocyst complex component 2 [Source:HGNC Symbol;Acc:HGNC:24968] |
| ENSG00000113161 | HGLibA_21709 | 2 | -1.116602804 | HMGCR | 3-hydroxy-3-methylglutaryl-CoA reductase [Source:HGNC Symbol;Acc:HGNC:5006] |
| ENSG00000113532 | HGLibA_47298 | 2 | -1.116602804 | ST8SIA4 | 8-sialyltransferase 4 [Source:HGNC Symbol;Acc:HGNC:10871]" |
| ENSG00000114302 | HGLibA_38527 | 2 | -1.116602804 | PRKAR2A | protein kinase cAMP-dependent type II regulatory subunit alpha [Source:HGNC Symbol;Acc:HGNC:9391] |
| ENSG00000114446 | HGLibA_22766 | 2 | -1.116602804 | IFT57 | intraflagellar transport 57 [Source:HGNC Symbol;Acc:HGNC:17367] |
| ENSG00000114738 | HGLibA_28305 | 2 | -1.116602804 | MAPKAPK3 | mitogen-activated protein kinase-activated protein kinase 3 [Source:HGNC Symbol;Acc:HGNC:6888] |
| ENSG00000114744 | HGLibA_10656 | 2 | -1.116602804 | COMMD2 | COMM domain containing 2 [Source:HGNC Symbol;Acc:HGNC:24993] |
| ENSG00000114770 | HGLibA_00180 | 2 | -1.116602804 | ABCC5 | ATP binding cassette subfamily C member 5 [Source:HGNC Symbol;Acc:HGNC:56] |
| ENSG00000115216 | HGLibA_32656 | 2 | -1.116602804 | NRBP1 | nuclear receptor binding protein 1 [Source:HGNC Symbol;Acc:HGNC:7993] |
| ENSG00000115461 | HGLibA_22828 | 2 | -1.116602804 | IGFBP5 | insulin like growth factor binding protein 5 [Source:HGNC Symbol;Acc:HGNC:5474] |
| ENSG00000115963 | HGLibA_41452 | 2 | -1.116602804 | RND3 | Rho family GTPase 3 [Source:HGNC Symbol;Acc:HGNC:671] |
| ENSG00000116133 | HGLibA_13092 | 2 | -1.116602804 | DHCR24 | 24-dehydrocholesterol reductase [Source:HGNC Symbol;Acc:HGNC:2859] |
| ENSG00000116212 | HGLibA_27360 | 2 | -1.116602804 | LRRC42 | leucine rich repeat containing 42 [Source:HGNC Symbol;Acc:HGNC:28792] |
| ENSG00000116857 | HGLibA_50556 | 2 | -1.116602804 | TMEM9 | transmembrane protein 9 [Source:HGNC Symbol;Acc:HGNC:18823] |
| ENSG00000117155 | HGLibA_47203 | 2 | -1.116602804 | SSX2IP | SSX family member 2 interacting protein [Source:HGNC Symbol;Acc:HGNC:16509] |
| ENSG00000117262 | HGLibA_20106 | 2 | -1.116602804 | GPR89A | G protein-coupled receptor 89A [Source:HGNC Symbol;Acc:HGNC:31984] |
| ENSG00000118004 | HGLibA_10636 | 2 | -1.116602804 | COLEC11 | collectin subfamily member 11 [Source:HGNC Symbol;Acc:HGNC:17213] |
| ENSG00000118507 | HGLibA_01442 | 2 | -1.116602804 | AKAP7 | A-kinase anchoring protein 7 [Source:HGNC Symbol;Acc:HGNC:377] |
| ENSG00000118514 | HGLibA_01587 | 2 | -1.116602804 | ALDH8A1 | aldehyde dehydrogenase 8 family member A1 [Source:HGNC Symbol;Acc:HGNC:15471] |
| ENSG00000118702 | HGLibA_19043 | 2 | -1.116602804 | GHRH | growth hormone releasing hormone [Source:HGNC Symbol;Acc:HGNC:4265] |
| ENSG00000119227 | HGLibA_36607 | 2 | -1.116602804 | PIGZ | phosphatidylinositol glycan anchor biosynthesis class Z [Source:HGNC Symbol;Acc:HGNC:30596] |
| ENSG00000119630 | HGLibA_36235 | 2 | -1.116602804 | PGF | placental growth factor [Source:HGNC Symbol;Acc:HGNC:8893] |
| ENSG00000120685 | HGLibA_38695 | 2 | -1.116602804 | PROSER1 | proline and serine rich 1 [Source:HGNC Symbol;Acc:HGNC:20291] |
| ENSG00000122859 | HGLibA_31753 | 2 | -1.116602804 | NEUROG3 | neurogenin 3 [Source:HGNC Symbol;Acc:HGNC:13806] |
| ENSG00000124225 | HGLibA_37299 | 2 | -1.116602804 | PMEPA1 | androgen induced 1 [Source:HGNC Symbol;Acc:HGNC:14107]" |
| ENSG00000124785 | HGLibA_32710 | 2 | -1.116602804 | NRN1 | neuritin 1 [Source:HGNC Symbol;Acc:HGNC:17972] |
| ENSG00000124920 | HGLibA_30917 | 2 | -1.116602804 | MYRF | myelin regulatory factor [Source:HGNC Symbol;Acc:HGNC:1181] |
| ENSG00000125551 | HGLibA_37165 | 2 | -1.116602804 | PLGLB2 | plasminogen-like B2 [Source:HGNC Symbol;Acc:HGNC:9073] |
| ENSG00000125775 | HGLibA_43113 | 2 | -1.116602804 | SDCBP2 | syndecan binding protein 2 [Source:HGNC Symbol;Acc:HGNC:15756] |
| ENSG00000125812 | HGLibA_20716 | 2 | -1.116602804 | GZF1 | GDNF inducible zinc finger protein 1 [Source:HGNC Symbol;Acc:HGNC:15808] |
| ENSG00000126458 | HGLibA_42236 | 2 | -1.116602804 | RRAS | RAS related [Source:HGNC Symbol;Acc:HGNC:10447] |
| ENSG00000126785 | HGLibA_41195 | 2 | -1.116602804 | RHOJ | ras homolog family member J [Source:HGNC Symbol;Acc:HGNC:688] |
| ENSG00000126821 | HGLibA_43849 | 2 | -1.116602804 | SGPP1 | sphingosine-1-phosphate phosphatase 1 [Source:HGNC Symbol;Acc:HGNC:17720] |
| ENSG00000127529 | HGLibA_34468 | 2 | -1.116602804 | OR7C2 | olfactory receptor family 7 subfamily C member 2 [Source:HGNC Symbol;Acc:HGNC:8374] |
| ENSG00000128271 | HGLibA_01013 | 2 | -1.116602804 | ADORA2A | adenosine A2a receptor [Source:HGNC Symbol;Acc:HGNC:263] |
| ENSG00000128294 | HGLibA_51206 | 2 | -1.116602804 | TPST2 | tyrosylprotein sulfotransferase 2 [Source:HGNC Symbol;Acc:HGNC:12021] |
| ENSG00000130208 | HGLibA_02504 | 2 | -1.116602804 | APOC1 | apolipoprotein C1 [Source:HGNC Symbol;Acc:HGNC:607] |
| ENSG00000130720 | HGLibA_17497 | 2 | -1.116602804 | FIBCD1 | fibrinogen C domain containing 1 [Source:HGNC Symbol;Acc:HGNC:25922] |
| ENSG00000131080 | HGLibA_14434 | 2 | -1.116602804 | EDA2R | ectodysplasin A2 receptor [Source:HGNC Symbol;Acc:HGNC:17756] |
| ENSG00000131849 | HGLibA_55463 | 2 | -1.116602804 | ZNF132 | zinc finger protein 132 [Source:HGNC Symbol;Acc:HGNC:12916] |
| ENSG00000132024 | HGLibA_07476 | 2 | -1.116602804 | CC2D1A | coiled-coil and C2 domain containing 1A [Source:HGNC Symbol;Acc:HGNC:30237] |
| ENSG00000132274 | HGLibA_51431 | 2 | -1.116602804 | TRIM22 | tripartite motif containing 22 [Source:HGNC Symbol;Acc:HGNC:16379] |
| ENSG00000132376 | HGLibA_23360 | 2 | -1.116602804 | INPP5K | inositol polyphosphate-5-phosphatase K [Source:HGNC Symbol;Acc:HGNC:33882] |
| ENSG00000133027 | HGLibA_36046 | 2 | -1.116602804 | PEMT | phosphatidylethanolamine N-methyltransferase [Source:HGNC Symbol;Acc:HGNC:8830] |
| ENSG00000133055 | HGLibA_30657 | 2 | -1.116602804 | MYBPH | myosin binding protein H [Source:HGNC Symbol;Acc:HGNC:7552] |
| ENSG00000133059 | HGLibA_14050 | 2 | -1.116602804 | DSTYK | dual serine/threonine and tyrosine protein kinase [Source:HGNC Symbol;Acc:HGNC:29043] |
| ENSG00000133101 | HGLibA_08050 | 2 | -1.116602804 | CCNA1 | cyclin A1 [Source:HGNC Symbol;Acc:HGNC:1577] |
| ENSG00000134551 | HGLibA_38463 | 2 | -1.116602804 | PRH2 | proline rich protein HaeIII subfamily 2 [Source:HGNC Symbol;Acc:HGNC:9367] |
| ENSG00000134569 | HGLibA_27220 | 2 | -1.116602804 | LRP4 | LDL receptor related protein 4 [Source:HGNC Symbol;Acc:HGNC:6696] |
| ENSG00000135049 | HGLibA_01258 | 2 | -1.116602804 | AGTPBP1 | ATP/GTP binding protein 1 [Source:HGNC Symbol;Acc:HGNC:17258] |
| ENSG00000135164 | HGLibA_13445 | 2 | -1.116602804 | DMTF1 | cyclin D binding myb like transcription factor 1 [Source:HGNC Symbol;Acc:HGNC:14603] |
| ENSG00000135222 | HGLibA_11375 | 2 | -1.116602804 | CSN2 | casein beta [Source:HGNC Symbol;Acc:HGNC:2447] |
| ENSG00000135486 | HGLibA_21791 | 2 | -1.116602804 | HNRNPA1 | heterogeneous nuclear ribonucleoprotein A1 [Source:HGNC Symbol;Acc:HGNC:5031] |
| ENSG00000136059 | HGLibA_53653 | 2 | -1.116602804 | VILL | villin like [Source:HGNC Symbol;Acc:HGNC:30906] |
| ENSG00000136826 | HGLibA_25071 | 2 | -1.116602804 | KLF4 | Kruppel like factor 4 [Source:HGNC Symbol;Acc:HGNC:6348] |
| ENSG00000136877 | HGLibA_17961 | 2 | -1.116602804 | FPGS | folylpolyglutamate synthase [Source:HGNC Symbol;Acc:HGNC:3824] |
| ENSG00000137166 | HGLibA_17939 | 2 | -1.116602804 | FOXP4 | forkhead box P4 [Source:HGNC Symbol;Acc:HGNC:20842] |
| ENSG00000137757 | HGLibA_07317 | 2 | -1.116602804 | CASP5 | caspase 5 [Source:HGNC Symbol;Acc:HGNC:1506] |
| ENSG00000137770 | HGLibA_11617 | 2 | -1.116602804 | CTDSPL2 | CTD small phosphatase like 2 [Source:HGNC Symbol;Acc:HGNC:26936] |
| ENSG00000138035 | HGLibA_37431 | 2 | -1.116602804 | PNPT1 | polyribonucleotide nucleotidyltransferase 1 [Source:HGNC Symbol;Acc:HGNC:23166] |
| ENSG00000138068 | HGLibA_47767 | 2 | -1.116602804 | SULT6B1 | sulfotransferase family 6B member 1 [Source:HGNC Symbol;Acc:HGNC:33433] |
| ENSG00000138115 | HGLibA_12121 | 2 | -1.116602804 | CYP2C8 | cytochrome P450 family 2 subfamily C member 8 [Source:HGNC Symbol;Acc:HGNC:2622] |
| ENSG00000138613 | HGLibA_02415 | 2 | -1.116602804 | APH1B | gamma-secretase subunit [Source:HGNC Symbol;Acc:HGNC:24080]" |
| ENSG00000139112 | HGLibA_18335 | 2 | -1.116602804 | GABARAPL1 | GABA type A receptor associated protein like 1 [Source:HGNC Symbol;Acc:HGNC:4068] |
| ENSG00000139631 | HGLibA_11303 | 2 | -1.116602804 | CSAD | cysteine sulfinic acid decarboxylase [Source:HGNC Symbol;Acc:HGNC:18966] |
| ENSG00000140044 | HGLibA_23944 | 2 | -1.116602804 | JDP2 | Jun dimerization protein 2 [Source:HGNC Symbol;Acc:HGNC:17546] |
| ENSG00000140521 | HGLibA_37523 | 2 | -1.116602804 | POLG | catalytic subunit [Source:HGNC Symbol;Acc:HGNC:9179]" |
| ENSG00000142556 | HGLibA_56389 | 2 | -1.116602804 | ZNF614 | zinc finger protein 614 [Source:HGNC Symbol;Acc:HGNC:24722] |
| ENSG00000142864 | HGLibA_43467 | 2 | -1.116602804 | SERBP1 | SERPINE1 mRNA binding protein 1 [Source:HGNC Symbol;Acc:HGNC:17860] |
| ENSG00000143079 | HGLibA_11747 | 2 | -1.116602804 | CTTNBP2NL | CTTNBP2 N-terminal like [Source:HGNC Symbol;Acc:HGNC:25330] |
| ENSG00000143294 | HGLibA_38354 | 2 | -1.116602804 | PRCC | proline rich mitotic checkpoint control factor [Source:HGNC Symbol;Acc:HGNC:9343] |
| ENSG00000143376 | HGLibA_46074 | 2 | -1.116602804 | SNX27 | sorting nexin family member 27 [Source:HGNC Symbol;Acc:HGNC:20073] |
| ENSG00000143786 | HGLibA_10289 | 2 | -1.116602804 | CNIH3 | cornichon family AMPA receptor auxiliary protein 3 [Source:HGNC Symbol;Acc:HGNC:26802] |
| ENSG00000143858 | HGLibA_48072 | 2 | -1.116602804 | SYT2 | synaptotagmin 2 [Source:HGNC Symbol;Acc:HGNC:11510] |
| ENSG00000144891 | HGLibA_01261 | 2 | -1.116602804 | AGTR1 | angiotensin II receptor type 1 [Source:HGNC Symbol;Acc:HGNC:336] |
| ENSG00000145414 | HGLibA_31061 | 2 | -1.116602804 | NAF1 | nuclear assembly factor 1 ribonucleoprotein [Source:HGNC Symbol;Acc:HGNC:25126] |
| ENSG00000145782 | HGLibA_03464 | 2 | -1.116602804 | ATG12 | autophagy related 12 [Source:HGNC Symbol;Acc:HGNC:588] |
| ENSG00000145907 | HGLibA_18298 | 2 | -1.116602804 | G3BP1 | G3BP stress granule assembly factor 1 [Source:HGNC Symbol;Acc:HGNC:30292] |
| ENSG00000146678 | HGLibA_22814 | 2 | -1.116602804 | IGFBP1 | insulin like growth factor binding protein 1 [Source:HGNC Symbol;Acc:HGNC:5469] |
| ENSG00000148248 | HGLibA_47839 | 2 | -1.116602804 | SURF4 | surfeit 4 [Source:HGNC Symbol;Acc:HGNC:11476] |
| ENSG00000148411 | HGLibA_31041 | 2 | -1.116602804 | NACC2 | NACC family member 2 [Source:HGNC Symbol;Acc:HGNC:23846] |
| ENSG00000148824 | HGLibA_30371 | 2 | -1.116602804 | MTG1 | mitochondrial ribosome associated GTPase 1 [Source:HGNC Symbol;Acc:HGNC:32159] |
| ENSG00000148841 | HGLibA_23861 | 2 | -1.116602804 | ITPRIP | 5-trisphosphate receptor interacting protein [Source:HGNC Symbol;Acc:HGNC:29370]" |
| ENSG00000149294 | HGLibA_31254 | 2 | -1.116602804 | NCAM1 | neural cell adhesion molecule 1 [Source:HGNC Symbol;Acc:HGNC:7656] |
| ENSG00000149527 | HGLibA_37010 | 2 | -1.116602804 | PLCH2 | phospholipase C eta 2 [Source:HGNC Symbol;Acc:HGNC:29037] |
| ENSG00000150551 | HGLibA_27714 | 2 | -1.116602804 | LYPD1 | LY6/PLAUR domain containing 1 [Source:HGNC Symbol;Acc:HGNC:28431] |
| ENSG00000150783 | HGLibA_49054 | 2 | -1.116602804 | TEX12 | testis expressed 12 [Source:HGNC Symbol;Acc:HGNC:11734] |
| ENSG00000153201 | HGLibA_40201 | 2 | -1.116602804 | RANBP2 | RAN binding protein 2 [Source:HGNC Symbol;Acc:HGNC:9848] |
| ENSG00000153406 | HGLibA_32194 | 2 | -1.116602804 | NMRAL1 | NmrA like redox sensor 1 [Source:HGNC Symbol;Acc:HGNC:24987] |
| ENSG00000153820 | HGLibA_46649 | 2 | -1.116602804 | SPHKAP | AKAP domain containing [Source:HGNC Symbol;Acc:HGNC:30619]" |
| ENSG00000153898 | HGLibA_28639 | 2 | -1.116602804 | MCOLN2 | mucolipin 2 [Source:HGNC Symbol;Acc:HGNC:13357] |
| ENSG00000154065 | HGLibA_02058 | 2 | -1.116602804 | ANKRD29 | ankyrin repeat domain 29 [Source:HGNC Symbol;Acc:HGNC:27110] |
| ENSG00000154175 | HGLibA_00299 | 2 | -1.116602804 | ABI3BP | ABI family member 3 binding protein [Source:HGNC Symbol;Acc:HGNC:17265] |
| ENSG00000154229 | HGLibA_38533 | 2 | -1.116602804 | PRKCA | protein kinase C alpha [Source:HGNC Symbol;Acc:HGNC:9393] |
| ENSG00000154274 | HGLibA_06300 | 2 | -1.116602804 | C4orf19 | chromosome 4 open reading frame 19 [Source:HGNC Symbol;Acc:HGNC:25618] |
| ENSG00000154479 | HGLibA_07701 | 2 | -1.116602804 | CCDC173 | coiled-coil domain containing 173 [Source:HGNC Symbol;Acc:HGNC:25064] |
| ENSG00000155760 | HGLibA_18281 | 2 | -1.116602804 | FZD7 | frizzled class receptor 7 [Source:HGNC Symbol;Acc:HGNC:4045] |
| ENSG00000155903 | HGLibA_40300 | 2 | -1.116602804 | RASA2 | RAS p21 protein activator 2 [Source:HGNC Symbol;Acc:HGNC:9872] |
| ENSG00000156050 | HGLibA_16218 | 2 | -1.116602804 | FAM161B | centrosomal protein [Source:HGNC Symbol;Acc:HGNC:19854]" |
| ENSG00000156467 | HGLibA_53111 | 2 | -1.116602804 | UQCRB | ubiquinol-cytochrome c reductase binding protein [Source:HGNC Symbol;Acc:HGNC:12582] |
| ENSG00000157087 | HGLibA_03618 | 2 | -1.116602804 | ATP2B2 | ATPase plasma membrane Ca2+ transporting 2 [Source:HGNC Symbol;Acc:HGNC:815] |

| | | | | | |
|---|---|---|---|---|---|
| ENSG00000158555 | HGLibA_18901 | 2 | -1.116602804 | GDPD5 | glycerophosphodiester phosphodiesterase domain containing 5 [Source:HGNC Symbol;Acc:HGNC:28804] |
| ENSG00000159131 | HGLibA_18637 | 2 | -1.116602804 | GART | phosphoribosylaminoimidazole synthetase [Source:HGNC Symbol;Acc:HGNC:4163]" |
| ENSG00000159723 | HGLibA_01254 | 2 | -1.116602804 | AGRP | agouti related neuropeptide [Source:HGNC Symbol;Acc:HGNC:330] |
| ENSG00000160055 | HGLibA_50267 | 2 | -1.116602804 | TMEM234 | transmembrane protein 234 [Source:HGNC Symbol;Acc:HGNC:28837] |
| ENSG00000160075 | HGLibA_47190 | 2 | -1.116602804 | SSU72 | RNA polymerase II CTD phosphatase [Source:HGNC Symbol;Acc:HGNC:25016]" |
| ENSG00000161813 | HGLibA_26033 | 2 | -1.116602804 | LARP4 | La ribonucleoprotein domain family member 4 [Source:HGNC Symbol;Acc:HGNC:24320] |
| ENSG00000161911 | HGLibA_51361 | 2 | -1.116602804 | TREML1 | triggering receptor expressed on myeloid cells like 1 [Source:HGNC Symbol;Acc:HGNC:20434] |
| ENSG00000162517 | HGLibA_36023 | 2 | -1.116602804 | PEF1 | penta-EF-hand domain containing 1 [Source:HGNC Symbol;Acc:HGNC:30009] |
| ENSG00000162910 | HGLibA_30024 | 2 | -1.116602804 | MRPL55 | mitochondrial ribosomal protein L55 [Source:HGNC Symbol;Acc:HGNC:16686] |
| ENSG00000163017 | HGLibA_00613 | 2 | -1.116602804 | ACTG2 | enteric [Source:HGNC Symbol;Acc:HGNC:145]" |
| ENSG00000163116 | HGLibA_47562 | 2 | -1.116602804 | STPG2 | sperm tail PG-rich repeat containing 2 [Source:HGNC Symbol;Acc:HGNC:28712] |
| ENSG00000163157 | HGLibA_50602 | 2 | -1.116602804 | TMOD4 | tropomodulin 4 [Source:HGNC Symbol;Acc:HGNC:11874] |
| ENSG00000163170 | HGLibA_04690 | 2 | -1.116602804 | BOLA3 | bolA family member 3 [Source:HGNC Symbol;Acc:HGNC:24415] |
| ENSG00000163263 | HGLibA_05809 | 2 | -1.116602804 | C1orf189 | chromosome 1 open reading frame 189 [Source:HGNC Symbol;Acc:HGNC:32305] |
| ENSG00000163412 | HGLibA_14826 | 2 | -1.116602804 | EIF4E3 | eukaryotic translation initiation factor 4E family member 3 [Source:HGNC Symbol;Acc:HGNC:31837] |
| ENSG00000163521 | HGLibA_19221 | 2 | -1.116602804 | GLB1L | galactosidase beta 1 like [Source:HGNC Symbol;Acc:HGNC:28129] |
| ENSG00000163606 | HGLibA_08284 | 2 | -1.116602804 | CD200R1 | CD200 receptor 1 [Source:HGNC Symbol;Acc:HGNC:24235] |
| ENSG00000163817 | HGLibA_45323 | 2 | -1.116602804 | SLC6A20 | solute carrier family 6 member 20 [Source:HGNC Symbol;Acc:HGNC:30927] |
| ENSG00000164050 | HGLibA_37269 | 2 | -1.116602804 | PLXNB1 | plexin B1 [Source:HGNC Symbol;Acc:HGNC:9103] |
| ENSG00000164144 | HGLibA_02677 | 2 | -1.116602804 | ARFIP1 | ADP ribosylation factor interacting protein 1 [Source:HGNC Symbol;Acc:HGNC:21496] |
| ENSG00000164144 | HGLibA_02675 | 2 | -1.116602804 | ARFIP1 | ADP ribosylation factor interacting protein 1 [Source:HGNC Symbol;Acc:HGNC:21496] |
| ENSG00000164190 | HGLibA_31968 | 2 | -1.116602804 | NIPBL | cohesin loading factor [Source:HGNC Symbol;Acc:HGNC:28862]" |
| ENSG00000164399 | HGLibA_23127 | 2 | -1.116602804 | IL3 | interleukin 3 [Source:HGNC Symbol;Acc:HGNC:6011] |
| ENSG00000164403 | HGLibA_44086 | 2 | -1.116602804 | SHROOM1 | shroom family member 1 [Source:HGNC Symbol;Acc:HGNC:24084] |
| ENSG00000164405 | HGLibA_53128 | 2 | -1.116602804 | UQCRQ | ubiquinol-cytochrome c reductase complex III subunit VII [Source:HGNC Symbol;Acc:HGNC:29594] |
| ENSG00000164520 | HGLibA_40120 | 2 | -1.116602804 | RAET1E | retinoic acid early transcript 1E [Source:HGNC Symbol;Acc:HGNC:16793] |
| ENSG00000164764 | HGLibA_42815 | 2 | -1.116602804 | SBSPON | somatomedin B and thrombospondin type 1 domain containing [Source:HGNC Symbol;Acc:HGNC:30362] |
| ENSG00000164989 | HGLibA_07693 | 2 | -1.116602804 | CCDC171 | coiled-coil domain containing 171 [Source:HGNC Symbol;Acc:HGNC:29828] |
| ENSG00000165186 | HGLibA_39274 | 2 | -1.116602804 | PTCHD1 | patched domain containing 1 [Source:HGNC Symbol;Acc:HGNC:26392] |
| ENSG00000165506 | HGLibA_13462 | 2 | -1.116602804 | DNAAF2 | dynein axonemal assembly factor 2 [Source:HGNC Symbol;Acc:HGNC:20188] |
| ENSG00000165633 | HGLibA_53868 | 2 | -1.116602804 | VSTM4 | V-set and transmembrane domain containing 4 [Source:HGNC Symbol;Acc:HGNC:26470] |
| ENSG00000165669 | HGLibA_16421 | 2 | -1.116602804 | FAM204A | family with sequence similarity 204 member A [Source:HGNC Symbol;Acc:HGNC:25794] |
| ENSG00000165912 | HGLibA_34965 | 2 | -1.116602804 | PACSIN3 | protein kinase C and casein kinase substrate in neurons 3 [Source:HGNC Symbol;Acc:HGNC:8572] |
| ENSG00000165949 | HGLibA_22597 | 2 | -1.116602804 | IFI27 | interferon alpha inducible protein 27 [Source:HGNC Symbol;Acc:HGNC:5397] |
| ENSG00000166788 | HGLibA_42620 | 2 | -1.116602804 | SAAL1 | serum amyloid A like 1 [Source:HGNC Symbol;Acc:HGNC:25158] |
| ENSG00000166819 | HGLibA_37169 | 2 | -1.116602804 | PLIN1 | perilipin 1 [Source:HGNC Symbol;Acc:HGNC:9076] |
| ENSG00000167632 | HGLibA_51339 | 2 | -1.116602804 | TRAPPC9 | trafficking protein particle complex 9 [Source:HGNC Symbol;Acc:HGNC:30832] |
| ENSG00000167799 | HGLibA_32991 | 2 | -1.116602804 | NUDT8 | nudix hydrolase 8 [Source:HGNC Symbol;Acc:HGNC:8055] |
| ENSG00000168214 | HGLibA_40646 | 2 | -1.116602804 | RBPJ | recombination signal binding protein for immunoglobulin kappa J region [Source:HGNC Symbol;Acc:HGNC:5724] |
| ENSG00000168263 | HGLibA_24406 | 2 | -1.116602804 | KCNV2 | potassium voltage-gated channel modifier subfamily V member 2 [Source:HGNC Symbol;Acc:HGNC:19698] |
| ENSG00000168556 | HGLibA_23285 | 2 | -1.116602804 | ING2 | inhibitor of growth family member 2 [Source:HGNC Symbol;Acc:HGNC:6063] |
| ENSG00000168765 | HGLibA_20481 | 2 | -1.116602804 | GSTM4 | glutathione S-transferase mu 4 [Source:HGNC Symbol;Acc:HGNC:4636] |
| ENSG00000168807 | HGLibA_46004 | 2 | -1.116602804 | SNTB2 | syntrophin beta 2 [Source:HGNC Symbol;Acc:HGNC:11169] |
| ENSG00000168883 | HGLibA_53356 | 2 | -1.116602804 | USP39 | ubiquitin specific peptidase 39 [Source:HGNC Symbol;Acc:HGNC:20071] |
| ENSG00000169169 | HGLibA_11025 | 2 | -1.116602804 | CPT1C | carnitine palmitoyltransferase 1C [Source:HGNC Symbol;Acc:HGNC:18540] |
| ENSG00000169217 | HGLibA_08331 | 2 | -1.116602804 | CD2BP2 | CD2 cytoplasmic tail binding protein 2 [Source:HGNC Symbol;Acc:HGNC:1656] |
| ENSG00000169271 | HGLibA_22310 | 2 | -1.116602804 | HSPB3 | heat shock protein family B (small) member 3 [Source:HGNC Symbol;Acc:HGNC:5248] |
| ENSG00000169490 | HGLibA_49686 | 2 | -1.116602804 | TM2D2 | TM2 domain containing 2 [Source:HGNC Symbol;Acc:HGNC:24127] |
| ENSG00000169683 | HGLibA_27364 | 2 | -1.116602804 | LRRC45 | leucine rich repeat containing 45 [Source:HGNC Symbol;Acc:HGNC:28302] |
| ENSG00000169860 | HGLibA_34866 | 2 | -1.116602804 | P2RY1 | purinergic receptor P2Y1 [Source:HGNC Symbol;Acc:HGNC:8539] |
| ENSG00000169862 | HGLibA_11661 | 2 | -1.116602804 | CTNND2 | catenin delta 2 [Source:HGNC Symbol;Acc:HGNC:2516] |
| ENSG00000170185 | HGLibA_53352 | 2 | -1.116602804 | USP38 | ubiquitin specific peptidase 38 [Source:HGNC Symbol;Acc:HGNC:20067] |
| ENSG00000170502 | HGLibA_32995 | 2 | -1.116602804 | NUDT9 | nudix hydrolase 9 [Source:HGNC Symbol;Acc:HGNC:8056] |
| ENSG00000170522 | HGLibA_14972 | 2 | -1.116602804 | ELOVL6 | ELOVL fatty acid elongase 6 [Source:HGNC Symbol;Acc:HGNC:15829] |
| ENSG00000170608 | HGLibA_17814 | 2 | -1.116602804 | FOXA3 | forkhead box A3 [Source:HGNC Symbol;Acc:HGNC:5023] |
| ENSG00000170802 | HGLibA_17911 | 2 | -1.116602804 | FOXN2 | forkhead box N2 [Source:HGNC Symbol;Acc:HGNC:5281] |
| ENSG00000171201 | HGLibA_45826 | 2 | -1.116602804 | SMR3B | submaxillary gland androgen regulated protein 3B [Source:HGNC Symbol;Acc:HGNC:17326] |
| ENSG00000171433 | HGLibA_19289 | 2 | -1.116602804 | GLOD5 | glyoxalase domain containing 5 [Source:HGNC Symbol;Acc:HGNC:33358] |
| ENSG00000171806 | HGLibA_28969 | 2 | -1.116602804 | METTL18 | methyltransferase like 18 [Source:HGNC Symbol;Acc:HGNC:28793] |
| ENSG00000171988 | HGLibA_23953 | 2 | -1.116602804 | JMJD1C | jumonji domain containing 1C [Source:HGNC Symbol;Acc:HGNC:12313] |
| ENSG00000172113 | HGLibA_32169 | 2 | -1.116602804 | NME6 | NME/NM23 nucleoside diphosphate kinase 6 [Source:HGNC Symbol;Acc:HGNC:20567] |
| ENSG00000172116 | HGLibA_08475 | 2 | -1.116602804 | CD8B | CD8b molecule [Source:HGNC Symbol;Acc:HGNC:1707] |
| ENSG00000172236 | HGLibA_51194 | 2 | -1.116602804 | TPSAB1 | tryptase alpha/beta 1 [Source:HGNC Symbol;Acc:HGNC:12019] |
| ENSG00000173275 | HGLibA_56024 | 2 | -1.116602804 | ZNF449 | zinc finger protein 449 [Source:HGNC Symbol;Acc:HGNC:21039] |
| ENSG00000173801 | HGLibA_24011 | 2 | -1.116602804 | JUP | junction plakoglobin [Source:HGNC Symbol;Acc:HGNC:6207] |
| ENSG00000173875 | HGLibA_56768 | 2 | -1.116602804 | ZNF791 | zinc finger protein 791 [Source:HGNC Symbol;Acc:HGNC:26895] |
| ENSG00000174231 | HGLibA_38747 | 2 | -1.116602804 | PRPF8 | pre-mRNA processing factor 8 [Source:HGNC Symbol;Acc:HGNC:17340] |
| ENSG00000174938 | HGLibA_43686 | 2 | -1.116602804 | SEZ6L2 | seizure related 6 homolog like 2 [Source:HGNC Symbol;Acc:HGNC:30844] |
| ENSG00000175198 | HGLibA_35380 | 2 | -1.116602804 | PCCA | propionyl-CoA carboxylase subunit alpha [Source:HGNC Symbol;Acc:HGNC:8653] |
| ENSG00000175198 | HGLibA_35379 | 2 | -1.116602804 | PCCA | propionyl-CoA carboxylase subunit alpha [Source:HGNC Symbol;Acc:HGNC:8653] |
| ENSG00000176732 | HGLibA_36182 | 2 | -1.116602804 | PFN4 | profilin family member 4 [Source:HGNC Symbol;Acc:HGNC:31103] |
| ENSG00000176890 | HGLibA_52461 | 2 | -1.116602804 | TYMS | thymidylate synthetase [Source:HGNC Symbol;Acc:HGNC:12441] |
| ENSG00000177098 | HGLibA_42994 | 2 | -1.116602804 | SCN4B | sodium voltage-gated channel beta subunit 4 [Source:HGNC Symbol;Acc:HGNC:10592] |
| ENSG00000177119 | HGLibA_02185 | 2 | -1.116602804 | ANO6 | anoctamin 6 [Source:HGNC Symbol;Acc:HGNC:25240] |
| ENSG00000177943 | HGLibA_28041 | 2 | -1.116602804 | MAMDC4 | MAM domain containing 4 [Source:HGNC Symbol;Acc:HGNC:24083] |
| ENSG00000178127 | HGLibA_31572 | 2 | -1.116602804 | NDUFV2 | NADH:ubiquinone oxidoreductase core subunit V2 [Source:HGNC Symbol;Acc:HGNC:7717] |
| ENSG00000178397 | HGLibA_16514 | 2 | -1.116602804 | FAM220A | family with sequence similarity 220 member A [Source:HGNC Symbol;Acc:HGNC:22422] |
| ENSG00000178409 | HGLibA_04404 | 2 | -1.116602804 | BEND3 | BEN domain containing 3 [Source:HGNC Symbol;Acc:HGNC:23040] |
| ENSG00000179163 | HGLibA_18155 | 2 | -1.116602804 | FUCA1 | alpha-L-fucosidase 1 [Source:HGNC Symbol;Acc:HGNC:4006] |
| ENSG00000179588 | HGLibA_55252 | 2 | -1.116602804 | ZFPM1 | FOG family member 1 [Source:HGNC Symbol;Acc:HGNC:19762]" |
| ENSG00000179796 | HGLibA_27343 | 2 | -1.116602804 | LRRC3B | leucine rich repeat containing 3B [Source:HGNC Symbol;Acc:HGNC:28105] |
| ENSG00000180219 | HGLibA_16692 | 2 | -1.116602804 | FAM71C | family with sequence similarity 71 member C [Source:HGNC Symbol;Acc:HGNC:28594] |
| ENSG00000180318 | HGLibA_01732 | 2 | -1.116602804 | ALX1 | ALX homeobox 1 [Source:HGNC Symbol;Acc:HGNC:1494] |
| ENSG00000180357 | HGLibA_56378 | 2 | -1.116602804 | ZNF609 | zinc finger protein 609 [Source:HGNC Symbol;Acc:HGNC:29003] |
| ENSG00000180509 | HGLibA_24184 | 2 | -1.116602804 | KCNE1 | potassium voltage-gated channel subfamily E regulatory subunit 1 [Source:HGNC Symbol;Acc:HGNC:6240] |
| ENSG00000180694 | HGLibA_50482 | 2 | -1.116602804 | TMEM64 | transmembrane protein 64 [Source:HGNC Symbol;Acc:HGNC:25441] |
| ENSG00000180739 | HGLibA_42604 | 2 | -1.116602804 | S1PR5 | sphingosine-1-phosphate receptor 5 [Source:HGNC Symbol;Acc:HGNC:14299] |
| ENSG00000181026 | HGLibA_01092 | 2 | -1.116602804 | AEN | apoptosis enhancing nuclease [Source:HGNC Symbol;Acc:HGNC:25722] |
| ENSG00000181409 | HGLibA_00089 | 2 | -1.116602804 | AATK | apoptosis associated tyrosine kinase [Source:HGNC Symbol;Acc:HGNC:21] |
| ENSG00000181619 | HGLibA_19893 | 2 | -1.116602804 | GPR135 | G protein-coupled receptor 135 [Source:HGNC Symbol;Acc:HGNC:19991] |
| ENSG00000182173 | HGLibA_51837 | 2 | -1.116602804 | TSEN54 | tRNA splicing endonuclease subunit 54 [Source:HGNC Symbol;Acc:HGNC:27561] |
| ENSG00000182195 | HGLibA_26257 | 2 | -1.116602804 | LDOC1 | regulator of NFKB signaling [Source:HGNC Symbol;Acc:HGNC:6548]" |
| ENSG00000182240 | HGLibA_04063 | 2 | -1.116602804 | BACE2 | beta-secretase 2 [Source:HGNC Symbol;Acc:HGNC:934] |
| ENSG00000183011 | HGLibA_30993 | 2 | -1.116602804 | NAA38 | NatC auxiliary subunit [Source:HGNC Symbol;Acc:HGNC:28212]" |
| ENSG00000183160 | HGLibA_49891 | 2 | -1.116602804 | TMEM119 | transmembrane protein 119 [Source:HGNC Symbol;Acc:HGNC:27884] |
| ENSG00000183304 | HGLibA_16819 | 2 | -1.116602804 | FAM9A | family with sequence similarity 9 member A [Source:HGNC Symbol;Acc:HGNC:18403] |
| ENSG00000183628 | HGLibA_13049 | 2 | -1.116602804 | DGCR6 | DiGeorge syndrome critical region gene 6 [Source:HGNC Symbol;Acc:HGNC:2846] |
| ENSG00000183780 | HGLibA_45008 | 2 | -1.116602804 | SLC35F3 | solute carrier family 35 member F3 [Source:HGNC Symbol;Acc:HGNC:23616] |
| ENSG00000183783 | HGLibA_24466 | 2 | -1.116602804 | KCTD8 | potassium channel tetramerization domain containing 8 [Source:HGNC Symbol;Acc:HGNC:22394] |
| ENSG00000184394 | HGLibA_34051 | 2 | -1.116602804 | OR4N5 | olfactory receptor family 4 subfamily N member 5 [Source:HGNC Symbol;Acc:HGNC:15358] |
| ENSG00000184900 | HGLibA_47783 | 2 | -1.116602804 | SUMO3 | small ubiquitin-like modifier 3 [Source:HGNC Symbol;Acc:HGNC:11124] |
| ENSG00000184923 | HGLibA_33106 | 2 | -1.116602804 | NUTM2A | NUT family member 2A [Source:HGNC Symbol;Acc:HGNC:23438] |
| ENSG00000185155 | HGLibA_29322 | 2 | -1.116602804 | MIXL1 | Mix paired-like homeobox [Source:HGNC Symbol;Acc:HGNC:13363] |
| ENSG00000185527 | HGLibA_35818 | 2 | -1.116602804 | PDE6G | phosphodiesterase 6G [Source:HGNC Symbol;Acc:HGNC:8789] |
| ENSG00000185651 | HGLibA_52626 | 2 | -1.116602804 | UBE2L3 | ubiquitin conjugating enzyme E2 L3 [Source:HGNC Symbol;Acc:HGNC:12488] |
| ENSG00000185737 | HGLibA_32681 | 2 | -1.116602804 | NRG3 | neuregulin 3 [Source:HGNC Symbol;Acc:HGNC:7999] |
| ENSG00000185920 | HGLibA_39268 | 2 | -1.116602804 | PTCH1 | patched 1 [Source:HGNC Symbol;Acc:HGNC:9585] |
| ENSG00000186231 | HGLibA_25187 | 2 | -1.116602804 | KLHL32 | kelch like family member 32 [Source:HGNC Symbol;Acc:HGNC:21221] |
| ENSG00000186675 | HGLibA_27984 | 2 | -1.116602804 | MAGEE2 | MAGE family member E2 [Source:HGNC Symbol;Acc:HGNC:24935] |
| ENSG00000187068 | HGLibA_06262 | 2 | -1.116602804 | C3orf70 | chromosome 3 open reading frame 70 [Source:HGNC Symbol;Acc:HGNC:33731] |
| ENSG00000187223 | HGLibA_26126 | 2 | -1.116602804 | LCE2D | late cornified envelope 2D [Source:HGNC Symbol;Acc:HGNC:16518] |
| ENSG00000187796 | HGLibA_07242 | 2 | -1.116602804 | CARD9 | caspase recruitment domain family member 9 [Source:HGNC Symbol;Acc:HGNC:16391] |
| ENSG00000187808 | HGLibA_46222 | 2 | -1.116602804 | SOWAHD | sosondowah ankyrin repeat domain family member D [Source:HGNC Symbol;Acc:HGNC:32960] |
| ENSG00000188306 | HGLibA_27461 | 2 | -1.116602804 | LRRIQ4 | leucine rich repeats and IQ motif containing 4 [Source:HGNC Symbol;Acc:HGNC:34298] |
| ENSG00000188334 | HGLibA_04875 | 2 | -1.116602804 | BSPH1 | binder of sperm protein homolog 1 [Source:HGNC Symbol;Acc:HGNC:33906] |
| ENSG00000188404 | HGLibA_43296 | 2 | -1.116602804 | SELL | selectin L [Source:HGNC Symbol;Acc:HGNC:10720] |
| ENSG00000188649 | HGLibA_07484 | 2 | -1.116602804 | CC2D2B | coiled-coil and C2 domain containing 2B [Source:HGNC Symbol;Acc:HGNC:31666] |
| ENSG00000188784 | HGLibA_36892 | 2 | -1.116602804 | PLA2G2E | phospholipase A2 group IIE [Source:HGNC Symbol;Acc:HGNC:13414] |
| ENSG00000189299 | HGLibA_17947 | 2 | -1.116602804 | FOXR2 | forkhead box R2 [Source:HGNC Symbol;Acc:HGNC:30469] |
| ENSG00000189375 | HGLibA_48521 | 2 | -1.116602804 | TBC1D28 | TBC1 domain family member 28 [Source:HGNC Symbol;Acc:HGNC:26858] |
| ENSG00000196141 | HGLibA_46549 | 2 | -1.116602804 | SPATS2L | spermatogenesis associated serine rich 2 like [Source:HGNC Symbol;Acc:HGNC:24574] |
| ENSG00000197275 | HGLibA_40099 | 2 | -1.116602804 | RAD54B | RAD54 homolog B [Source:HGNC Symbol;Acc:HGNC:17228] |
| ENSG00000197405 | HGLibA_06352 | 2 | -1.116602804 | C5AR1 | complement C5a receptor 1 [Source:HGNC Symbol;Acc:HGNC:1338] |
| ENSG00000197417 | HGLibA_44079 | 2 | -1.116602804 | SHPK | sedoheptulokinase [Source:HGNC Symbol;Acc:HGNC:1492] |
| ENSG00000197782 | HGLibA_56730 | 2 | -1.116602804 | ZNF780A | zinc finger protein 780A [Source:HGNC Symbol;Acc:HGNC:27603] |
| ENSG00000197857 | HGLibA_55999 | 2 | -1.116602804 | ZNF44 | zinc finger protein 44 [Source:HGNC Symbol;Acc:HGNC:13110] |
| ENSG00000197959 | HGLibA_13693 | 2 | -1.116602804 | DNM3 | dynamin 3 [Source:HGNC Symbol;Acc:HGNC:29125] |
| ENSG00000198042 | HGLibA_28018 | 2 | -1.116602804 | MAK16 | MAK16 homolog [Source:HGNC Symbol;Acc:HGNC:13703] |
| ENSG00000198589 | HGLibA_27134 | 2 | -1.116602804 | LRBA | LPS responsive beige-like anchor protein [Source:HGNC Symbol;Acc:HGNC:1742] |
| ENSG00000198643 | HGLibA_16584 | 2 | -1.116602804 | FAM3D | family with sequence similarity 3 member D [Source:HGNC Symbol;Acc:HGNC:18665] |
| ENSG00000198646 | HGLibA_31349 | 2 | -1.116602804 | NCOA6 | nuclear receptor coactivator 6 [Source:HGNC Symbol;Acc:HGNC:15936] |
| ENSG00000198881 | HGLibA_03211 | 2 | -1.116602804 | ASB12 | ankyrin repeat and SOCS box containing 12 [Source:HGNC Symbol;Acc:HGNC:19763] |
| ENSG00000203923 | HGLibA_46402 | 2 | -1.116602804 | SPANXN1 | SPANX family member N1 [Source:HGNC Symbol;Acc:HGNC:33174] |
| ENSG00000204301 | HGLibA_32351 | 2 | -1.116602804 | NOTCH4 | notch 4 [Source:HGNC Symbol;Acc:HGNC:7884] |
| ENSG00000204435 | HGLibA_11412 | 2 | -1.116602804 | CSNK2B | casein kinase 2 beta [Source:HGNC Symbol;Acc:HGNC:2460] |

| | | | | | |
|---|---|---|---|---|---|
| ENSG00000204469 | HGLibA_38861 | 2 | -1.116602804 | PRRC2A | proline rich coiled-coil 2A [Source:HGNC Symbol;Acc:HGNC:13918] |
| ENSG00000204592 | HGLibA_21642 | 2 | -1.116602804 | HLA-E | E [Source:HGNC Symbol;Acc:HGNC:4962]" |
| ENSG00000204952 | HGLibA_17115 | 2 | -1.116602804 | FBXO47 | F-box protein 47 [Source:HGNC Symbol;Acc:HGNC:31969] |
| ENSG00000205133 | HGLibA_51650 | 2 | -1.116602804 | TRIQK | triple QxxK/R motif containing [Source:HGNC Symbol;Acc:HGNC:27828] |
| ENSG00000205916 | HGLibA_12361 | 2 | -1.116602804 | DAZ4 | deleted in azoospermia 4 [Source:HGNC Symbol;Acc:HGNC:15966] |
| ENSG00000212724 | HGLibA_25694 | 2 | -1.116602804 | KRTAP2-3 | keratin associated protein 2-3 [Source:HGNC Symbol;Acc:HGNC:18906] |
| ENSG00000212899 | HGLibA_25746 | 2 | -1.116602804 | KRTAP3-3 | keratin associated protein 3-3 [Source:HGNC Symbol;Acc:HGNC:18890] |
| ENSG00000213347 | HGLibA_30614 | 2 | -1.116602804 | MXD3 | MAX dimerization protein 3 [Source:HGNC Symbol;Acc:HGNC:14008] |
| ENSG00000213420 | HGLibA_19765 | 2 | -1.116602804 | GPC2 | glypican 2 [Source:HGNC Symbol;Acc:HGNC:4450] |
| ENSG00000214021 | HGLibA_52201 | 2 | -1.116602804 | TTLL3 | tubulin tyrosine ligase like 3 [Source:HGNC Symbol;Acc:HGNC:24483] |
| ENSG00000214078 | HGLibA_10952 | 2 | -1.116602804 | CPNE1 | copine 1 [Source:HGNC Symbol;Acc:HGNC:2314] |
| ENSG00000214513 | HGLibA_32354 | 2 | -1.116602804 | NOTO | notochord homeobox [Source:HGNC Symbol;Acc:HGNC:31839] |
| ENSG00000214819 | HGLibA_08877 | 2 | -1.116602804 | CDRT15L2 | CMT1A duplicated region transcript 15 like 2 [Source:HGNC Symbol;Acc:HGNC:34075] |
| ENSG00000215218 | HGLibA_52652 | 2 | -1.116602804 | UBE2QL1 | ubiquitin conjugating enzyme E2 Q family like 1 [Source:HGNC Symbol;Acc:HGNC:37269] |
| ENSG00000219073 | HGLibA_09001 | 2 | -1.116602804 | CELA3B | chymotrypsin like elastase family member 3B [Source:HGNC Symbol;Acc:HGNC:15945] |
| ENSG00000221836 | HGLibA_33741 | 2 | -1.116602804 | OR2A5 | olfactory receptor family 2 subfamily A member 5 [Source:HGNC Symbol;Acc:HGNC:8232] |
| ENSG00000222028 | HGLibA_39100 | 2 | -1.116602804 | PSMB11 | proteasome subunit beta 11 [Source:HGNC Symbol;Acc:HGNC:31963] |
| ENSG00000222028 | HGLibA_39101 | 2 | -1.116602804 | PSMB11 | proteasome subunit beta 11 [Source:HGNC Symbol;Acc:HGNC:31963] |
| ENSG00000227140 | HGLibA_53281 | 2 | -1.116602804 | USP17L5 | ubiquitin specific peptidase 17-like family member 5 [Source:HGNC Symbol;Acc:HGNC:37177] |
| ENSG00000230797 | HGLibA_54764 | 2 | -1.116602804 | YY2 | YY2 transcription factor [Source:HGNC Symbol;Acc:HGNC:31684] |
| ENSG00000230873 | HGLibA_47529 | 2 | -1.116602804 | STMND1 | stathmin domain containing 1 [Source:HGNC Symbol;Acc:HGNC:44668] |
| ENSG00000231389 | HGLibA_21616 | 2 | -1.116602804 | HLA-DPA1 | DP alpha 1 [Source:HGNC Symbol;Acc:HGNC:4938]" |
| ENSG00000233954 | HGLibA_53125 | 2 | -1.116602804 | UQCRHL | ubiquinol-cytochrome c reductase hinge protein like [Source:HGNC Symbol;Acc:HGNC:51714] |
| ENSG00000235780 | HGLibA_53262 | 2 | -1.116602804 | USP17L27 | ubiquitin specific peptidase 17-like family member 27 [Source:HGNC Symbol;Acc:HGNC:44455] |
| ENSG00000236125 | HGLibA_53278 | 2 | -1.116602804 | USP17L4 | ubiquitin specific peptidase 17-like family member 4 [Source:HGNC Symbol;Acc:HGNC:37176] |
| ENSG00000236362 | HGLibA_18450 | 2 | -1.116602804 | GAGE12F | G antigen 12F [Source:HGNC Symbol;Acc:HGNC:31906] |
| ENSG00000237671 | HGLibA_18441 | 2 | -1.116602804 | GAGE12C | G antigen 12C [Source:HGNC Symbol;Acc:HGNC:28402] |
| ENSG00000240747 | HGLibA_25394 | 2 | -1.116602804 | KRBOX1 | KRAB box domain containing 1 [Source:HGNC Symbol;Acc:HGNC:38708] |
| ENSG00000240857 | HGLibA_40744 | 2 | -1.116602804 | RDH14 | retinol dehydrogenase 14 [Source:HGNC Symbol;Acc:HGNC:19979] |
| ENSG00000242028 | HGLibA_22463 | 2 | -1.116602804 | HYPK | huntingtin interacting protein K [Source:HGNC Symbol;Acc:HGNC:18418] |
| ENSG00000244165 | HGLibA_34872 | 2 | -1.116602804 | P2RY11 | purinergic receptor P2Y11 [Source:HGNC Symbol;Acc:HGNC:8540] |
| ENSG00000244731 | HGLibA_06282 | 2 | -1.116602804 | C4A | complement C4A (Rodgers blood group) [Source:HGNC Symbol;Acc:HGNC:1323] |
| ENSG00000247746 | HGLibA_53394 | 2 | -1.116602804 | USP51 | ubiquitin specific peptidase 51 [Source:HGNC Symbol;Acc:HGNC:23086] |
| ENSG00000248933 | HGLibA_53250 | 2 | -1.116602804 | USP17L22 | ubiquitin specific peptidase 17-like family member 22 [Source:HGNC Symbol;Acc:HGNC:44450] |
| ENSG00000250844 | HGLibA_53232 | 2 | -1.116602804 | USP17L18 | ubiquitin specific peptidase 17-like family member 18 [Source:HGNC Symbol;Acc:HGNC:44446] |
| ENSG00000254598 | HGLibA_11407 | 2 | -1.116602804 | CSNK2A3 | casein kinase 2 alpha 3 [Source:HGNC Symbol;Acc:HGNC:2458] |
| ENSG00000255561 | HGLibA_17263 | 2 | -1.116602804 | FDXACB1 | ferredoxin-fold anticodon binding domain containing 1 [Source:HGNC Symbol;Acc:HGNC:25110] |
| ENSG00000258643 | HGLibA_04326 | 2 | -1.116602804 | BCL2L2-PABPN1 | BCL2L2-PABPN1 readthrough [Source:HGNC Symbol;Acc:HGNC:42959] |
| ENSG00000270872 | HGLibA_46979 | 2 | -1.116602804 | SRGAP2D | SLIT-ROBO Rho GTPase activating protein 2D (pseudogene) [Source:HGNC Symbol;Acc:HGNC:43932] |
| ENSG00000273820 | HGLibA_53317 | 2 | -1.116602804 | USP27X | ubiquitin specific peptidase 27 X-linked [Source:HGNC Symbol;Acc:HGNC:13486] |
| ENSG00000274588 | HGLibA_13079 | 2 | -1.116602804 | DGKK | diacylglycerol kinase kappa [Source:HGNC Symbol;Acc:HGNC:32395] |
| ENSG00000280109 | HGLibA_36939 | 2 | -1.116602804 | PLAC4 | placenta specific 4 [Source:HGNC Symbol;Acc:HGNC:14616] |
| ENSG00000280236 | HGLibA_33588 | 2 | -1.116602804 | OR12D2 | olfactory receptor family 12 subfamily D member 2 (gene/pseudogene) [Source:HGNC Symbol;Acc:HGNC:8178] |
| ENSG00000281887 | HGLibA_19074 | 2 | -1.116602804 | GIMAP1-GIMAP5 | GIMAP1-GIMAP5 readthrough [Source:HGNC Symbol;Acc:HGNC:51257] |
| ENSG00000283597 | HGLibA_16254 | 2 | -1.116602804 | FAM169B | family with sequence similarity 169 member B [Source:HGNC Symbol;Acc:HGNC:26835] |
| EPB49 | HGLibA_15264 | 2 | -1.116602804 | EPB49 | - |
| EPT1 | HGLibA_15388 | 2 | -1.116602804 | EPT1 | - |
| FAM134A | HGLibA_16124 | 2 | -1.116602804 | FAM134A | - |
| FAM57B | HGLibA_16646 | 2 | -1.116602804 | FAM57B | - |
| GAGE12I | HGLibA_18459 | 2 | -1.116602804 | GAGE12I | - |
| GAGE2D | HGLibA_18477 | 2 | -1.116602804 | GAGE2D | - |
| HIST1H2AM | HGLibA_21407 | 2 | -1.116602804 | HIST1H2AM | - |
| HIST2H3A | HGLibA_21539 | 2 | -1.116602804 | HIST2H3A | - |
| HIST2H3C | HGLibA_21543 | 2 | -1.116602804 | HIST2H3C | - |
| HMP19 | HGLibA_21753 | 2 | -1.116602804 | HMP19 | - |
| hsa-let-7a-2 | HGLibA_57103 | 2 | -1.116602804 | hsa-let-7a-2 | - |
| hsa-mir-1238 | HGLibA_57343 | 2 | -1.116602804 | hsa-mir-1238 | - |
| hsa-mir-1245a | HGLibA_57373 | 2 | -1.116602804 | hsa-mir-1245a | - |
| hsa-mir-1248 | HGLibA_57390 | 2 | -1.116602804 | hsa-mir-1248 | - |
| hsa-mir-1912 | HGLibA_58053 | 2 | -1.116602804 | hsa-mir-1912 | - |
| hsa-mir-202 | HGLibA_58161 | 2 | -1.116602804 | hsa-mir-202 | - |
| hsa-mir-23b | HGLibA_58332 | 2 | -1.116602804 | hsa-mir-23b | - |
| hsa-mir-24-1 | HGLibA_58342 | 2 | -1.116602804 | hsa-mir-24-1 | - |
| hsa-mir-3146 | HGLibA_58642 | 2 | -1.116602804 | hsa-mir-3146 | - |
| hsa-mir-3165 | HGLibA_58738 | 2 | -1.116602804 | hsa-mir-3165 | - |
| hsa-mir-3175 | HGLibA_58772 | 2 | -1.116602804 | hsa-mir-3175 | - |
| hsa-mir-342 | HGLibA_59025 | 2 | -1.116602804 | hsa-mir-342 | - |
| hsa-mir-3675 | HGLibA_59252 | 2 | -1.116602804 | hsa-mir-3675 | - |
| hsa-mir-3923 | HGLibA_59568 | 2 | -1.116602804 | hsa-mir-3923 | - |
| hsa-mir-3972 | HGLibA_59649 | 2 | -1.116602804 | hsa-mir-3972 | - |
| hsa-mir-4258 | HGLibA_59740 | 2 | -1.116602804 | hsa-mir-4258 | - |
| hsa-mir-4262 | HGLibA_59754 | 2 | -1.116602804 | hsa-mir-4262 | - |
| hsa-mir-4265 | HGLibA_59769 | 2 | -1.116602804 | hsa-mir-4265 | - |
| hsa-mir-4267 | HGLibA_59775 | 2 | -1.116602804 | hsa-mir-4267 | - |
| hsa-mir-4304 | HGLibA_59928 | 2 | -1.116602804 | hsa-mir-4304 | - |
| hsa-mir-4494 | HGLibA_60393 | 2 | -1.116602804 | hsa-mir-4494 | - |
| hsa-mir-4500 | HGLibA_60428 | 2 | -1.116602804 | hsa-mir-4500 | - |
| hsa-mir-4533 | HGLibA_60590 | 2 | -1.116602804 | hsa-mir-4533 | - |
| hsa-mir-4538 | HGLibA_60614 | 2 | -1.116602804 | hsa-mir-4538 | - |
| hsa-mir-4718 | HGLibA_60973 | 2 | -1.116602804 | hsa-mir-4718 | - |
| hsa-mir-4771-1 | HGLibA_61182 | 2 | -1.116602804 | hsa-mir-4771-1 | - |
| hsa-mir-4773-2 | HGLibA_61198 | 2 | -1.116602804 | hsa-mir-4773-2 | - |
| hsa-mir-507 | HGLibA_61486 | 2 | -1.116602804 | hsa-mir-507 | - |
| hsa-mir-548ag-2 | HGLibA_61880 | 2 | -1.116602804 | hsa-mir-548ag-2 | - |
| hsa-mir-548an | HGLibA_61904 | 2 | -1.116602804 | hsa-mir-548an | - |
| hsa-mir-548i-1 | HGLibA_62012 | 2 | -1.116602804 | hsa-mir-548i-1 | - |
| hsa-mir-548o | HGLibA_62047 | 2 | -1.116602804 | hsa-mir-548o | - |
| hsa-mir-549a | HGLibA_62096 | 2 | -1.116602804 | hsa-mir-549a | - |
| hsa-mir-552 | HGLibA_62125 | 2 | -1.116602804 | hsa-mir-552 | - |
| hsa-mir-5695 | HGLibA_62314 | 2 | -1.116602804 | hsa-mir-5695 | - |
| hsa-mir-6072 | HGLibA_62540 | 2 | -1.116602804 | hsa-mir-6072 | - |
| hsa-mir-6073 | HGLibA_62544 | 2 | -1.116602804 | hsa-mir-6073 | - |
| hsa-mir-6754 | HGLibA_63174 | 2 | -1.116602804 | hsa-mir-6754 | - |
| hsa-mir-6858 | HGLibA_63603 | 2 | -1.116602804 | hsa-mir-6858 | - |
| hsa-mir-7846 | HGLibA_63974 | 2 | -1.116602804 | hsa-mir-7846 | - |
| hsa-mir-8064 | HGLibA_64095 | 2 | -1.116602804 | hsa-mir-8064 | - |
| hsa-mir-936 | HGLibA_64320 | 2 | -1.116602804 | hsa-mir-936 | - |
| IL8 | HGLibA_23195 | 2 | -1.116602804 | IL8 | - |
| KIAA0368 | HGLibA_24628 | 2 | -1.116602804 | KIAA0368 | - |
| KIAA1551 | HGLibA_24784 | 2 | -1.116602804 | KIAA1551 | - |
| LOC402160 | HGLibA_26939 | 2 | -1.116602804 | LOC402160 | - |
| LOC643037 | HGLibA_26963 | 2 | -1.116602804 | LOC643037 | - |
| NADKD1 | HGLibA_31050 | 2 | -1.116602804 | NADKD1 | - |
| OBFC1 | HGLibA_33221 | 2 | -1.116602804 | OBFC1 | - |
| RQCD1 | HGLibA_42217 | 2 | -1.116602804 | RQCD1 | - |
| 9-Sep | HGLibA_43457 | 2 | -1.116602804 | 9-Sep | - |
| SF3B14 | HGLibA_43703 | 2 | -1.116602804 | SF3B14 | - |
| SPRY3_X | HGLibA_46840 | 2 | -1.116602804 | SPRY3_X | - |
| TARP | HGLibA_48342 | 2 | -1.116602804 | TARP | - |
| WAPAL | HGLibA_53940 | 2 | -1.116602804 | WAPAL | - |
| ZUFSP | HGLibA_57060 | 2 | -1.116602804 | ZUFSP | - |
| 6-Mar | HGLibA_28342 | 1 | -1.125648147 | 6-Mar | - |
| 9-Mar | HGLibA_28352 | 1 | -1.125648147 | 9-Mar | - |
| ACPL2 | HGLibA_00520 | 1 | -1.125648147 | ACPL2 | - |
| ADRBK2 | HGLibA_01066 | 1 | -1.125648147 | ADRBK2 | - |
| ATP5SL | HGLibA_03690 | 1 | -1.125648147 | ATP5SL | - |
| BZRAP1 | HGLibA_05010 | 1 | -1.125648147 | BZRAP1 | - |
| C10orf118 | HGLibA_05039 | 1 | -1.125648147 | C10orf118 | - |
| C11orf31 | HGLibA_05130 | 1 | -1.125648147 | C11orf31 | - |
| C11orf83 | HGLibA_05192 | 1 | -1.125648147 | C11orf83 | - |
| C12orf49 | HGLibA_05254 | 1 | -1.125648147 | C12orf49 | - |
| C12orf52 | HGLibA_05261 | 1 | -1.125648147 | C12orf52 | - |
| C13orf35 | HGLibA_05310 | 1 | -1.125648147 | C13orf35 | - |
| C14orf142 | HGLibA_05326 | 1 | -1.125648147 | C14orf142 | - |
| C14orf166 | HGLibA_05334 | 1 | -1.125648147 | C14orf166 | - |
| C14orf166B | HGLibA_05335 | 1 | -1.125648147 | C14orf166B | - |
| C15orf43 | HGLibA_05403 | 1 | -1.125648147 | C15orf43 | - |
| C15orf57 | HGLibA_05420 | 1 | -1.125648147 | C15orf57 | - |
| C16orf70 | HGLibA_05471 | 1 | -1.125648147 | C16orf70 | - |
| C16orf97 | HGLibA_05512 | 1 | -1.125648147 | C16orf97 | - |
| C19orf70 | HGLibA_05698 | 1 | -1.125648147 | C19orf70 | - |
| C1orf101 | HGLibA_05732 | 1 | -1.125648147 | C1orf101 | - |
| C1orf173 | HGLibA_05794 | 1 | -1.125648147 | C1orf173 | - |
| C20orf201 | HGLibA_06012 | 1 | -1.125648147 | C20orf201 | - |
| C2orf54 | HGLibA_06149 | 1 | -1.125648147 | C2orf54 | - |

| | | | | | |
|---|---|---|---|---|---|
| C2orf82 | HGLibA_06191 | 1 | -1.125648147 | C2orf82 | - |
| C4orf26 | HGLibA_06308 | 1 | -1.125648147 | C4orf26 | - |
| C5orf42 | HGLibA_06383 | 1 | -1.125648147 | C5orf42 | - |
| C6orf10 | HGLibA_06431 | 1 | -1.125648147 | C6orf10 | - |
| C6orf222 | HGLibA_06475 | 1 | -1.125648147 | C6orf222 | - |
| C6orf25 | HGLibA_06484 | 1 | -1.125648147 | C6orf25 | - |
| C7orf66 | HGLibA_06577 | 1 | -1.125648147 | C7orf66 | - |
| C8orf87 | HGLibA_06655 | 1 | -1.125648147 | C8orf87 | - |
| C9orf123 | HGLibA_06670 | 1 | -1.125648147 | C9orf123 | - |
| C9orf142 | HGLibA_06686 | 1 | -1.125648147 | C9orf142 | - |
| C9orf171 | HGLibA_06709 | 1 | -1.125648147 | C9orf171 | - |
| CCDC23 | HGLibA_07734 | 1 | -1.125648147 | CCDC23 | - |
| DARS | HGLibA_12339 | 1 | -1.125648147 | DARS | - |
| DHRSX_X | HGLibA_13157 | 1 | -1.125648147 | DHRSX_X | - |
| DUSP27 | HGLibA_14172 | 1 | -1.125648147 | DUSP27 | - |
| ENSG00000001460 | HGLibA_47559 | 1 | -1.125648147 | STPG1 | sperm tail PG-rich repeat containing 1 [Source:HGNC Symbol;Acc:HGNC:28070] |
| ENSG00000002745 | HGLibA_54355 | 1 | -1.125648147 | WNT16 | Wnt family member 16 [Source:HGNC Symbol;Acc:HGNC:16267] |
| ENSG00000003400 | HGLibA_07297 | 1 | -1.125648147 | CASP10 | caspase 10 [Source:HGNC Symbol;Acc:HGNC:1500] |
| ENSG00000004700 | HGLibA_40766 | 1 | -1.125648147 | RECQL | RecQ like helicase [Source:HGNC Symbol;Acc:HGNC:9948] |
| ENSG00000004961 | HGLibA_20955 | 1 | -1.125648147 | HCCS | holocytochrome c synthase [Source:HGNC Symbol;Acc:HGNC:4837] |
| ENSG00000006007 | HGLibA_18847 | 1 | -1.125648147 | GDE1 | glycerophosphodiester phosphodiesterase 1 [Source:HGNC Symbol;Acc:HGNC:29644] |
| ENSG00000006116 | HGLibA_06936 | 1 | -1.125648147 | CACNG3 | calcium voltage-gated channel auxiliary subunit gamma 3 [Source:HGNC Symbol;Acc:HGNC:1407] |
| ENSG00000006377 | HGLibA_13386 | 1 | -1.125648147 | DLX6 | distal-less homeobox 6 [Source:HGNC Symbol;Acc:HGNC:2919] |
| ENSG00000006451 | HGLibA_40146 | 1 | -1.125648147 | RALA | RAS like proto-oncogene A [Source:HGNC Symbol;Acc:HGNC:9839] |
| ENSG00000006534 | HGLibA_01567 | 1 | -1.125648147 | ALDH3B1 | aldehyde dehydrogenase 3 family member B1 [Source:HGNC Symbol;Acc:HGNC:410] |
| ENSG00000006715 | HGLibA_53790 | 1 | -1.125648147 | VPS41 | HOPS complex subunit [Source:HGNC Symbol;Acc:HGNC:12713]" |
| ENSG00000006788 | HGLibA_30712 | 1 | -1.125648147 | MYH13 | myosin heavy chain 13 [Source:HGNC Symbol;Acc:HGNC:7571] |
| ENSG00000007312 | HGLibA_08451 | 1 | -1.125648147 | CD79B | CD79b molecule [Source:HGNC Symbol;Acc:HGNC:1699] |
| ENSG00000007350 | HGLibA_49589 | 1 | -1.125648147 | TKTL1 | transketolase like 1 [Source:HGNC Symbol;Acc:HGNC:11835] |
| ENSG00000007908 | HGLibA_43285 | 1 | -1.125648147 | SELE | selectin E [Source:HGNC Symbol;Acc:HGNC:10718] |
| ENSG00000007908 | HGLibA_43287 | 1 | -1.125648147 | SELE | selectin E [Source:HGNC Symbol;Acc:HGNC:10718] |
| ENSG00000008226 | HGLibA_13319 | 1 | -1.125648147 | DLEC1 | cilia and flagella associated protein [Source:HGNC Symbol;Acc:HGNC:2899]" |
| ENSG00000009694 | HGLibA_48996 | 1 | -1.125648147 | TENM1 | teneurin transmembrane protein 1 [Source:HGNC Symbol;Acc:HGNC:8117] |
| ENSG00000009790 | HGLibA_51255 | 1 | -1.125648147 | TRAF3IP3 | TRAF3 interacting protein 3 [Source:HGNC Symbol;Acc:HGNC:30766] |
| ENSG00000009954 | HGLibA_04188 | 1 | -1.125648147 | BAZ1B | bromodomain adjacent to zinc finger domain 1B [Source:HGNC Symbol;Acc:HGNC:961] |
| ENSG00000010295 | HGLibA_22587 | 1 | -1.125648147 | IFFO1 | intermediate filament family orphan 1 [Source:HGNC Symbol;Acc:HGNC:24970] |
| ENSG00000010818 | HGLibA_21570 | 1 | -1.125648147 | HIVEP2 | human immunodeficiency virus type I enhancer binding protein 2 [Source:HGNC Symbol;Acc:HGNC:4921] |
| ENSG00000011105 | HGLibA_51953 | 1 | -1.125648147 | TSPAN9 | tetraspanin 9 [Source:HGNC Symbol;Acc:HGNC:21640] |
| ENSG00000011260 | HGLibA_53443 | 1 | -1.125648147 | UTP18 | small subunit processome component [Source:HGNC Symbol;Acc:HGNC:24274]" |
| ENSG00000011590 | HGLibA_54859 | 1 | -1.125648147 | ZBTB32 | zinc finger and BTB domain containing 32 [Source:HGNC Symbol;Acc:HGNC:16763] |
| ENSG00000012963 | HGLibA_52762 | 1 | -1.125648147 | UBR7 | ubiquitin protein ligase E3 component n-recognin 7 (putative) [Source:HGNC Symbol;Acc:HGNC:20344] |
| ENSG00000014123 | HGLibA_52848 | 1 | -1.125648147 | UFL1 | UFM1 specific ligase 1 [Source:HGNC Symbol;Acc:HGNC:23039] |
| ENSG00000015133 | HGLibA_07913 | 1 | -1.125648147 | CCDC88C | coiled-coil domain containing 88C [Source:HGNC Symbol;Acc:HGNC:19967] |
| ENSG00000015133 | HGLibA_07914 | 1 | -1.125648147 | CCDC88C | coiled-coil domain containing 88C [Source:HGNC Symbol;Acc:HGNC:19967] |
| ENSG00000015676 | HGLibA_32928 | 1 | -1.125648147 | NUDCD3 | NudC domain containing 3 [Source:HGNC Symbol;Acc:HGNC:22208] |
| ENSG00000019505 | HGLibA_48056 | 1 | -1.125648147 | SYT13 | synaptotagmin 13 [Source:HGNC Symbol;Acc:HGNC:14962] |
| ENSG00000021461 | HGLibA_12158 | 1 | -1.125648147 | CYP3A43 | cytochrome P450 family 3 subfamily A member 43 [Source:HGNC Symbol;Acc:HGNC:17450] |
| ENSG00000025156 | HGLibA_22224 | 1 | -1.125648147 | HSF2 | heat shock transcription factor 2 [Source:HGNC Symbol;Acc:HGNC:5225] |
| ENSG00000025796 | HGLibA_43261 | 1 | -1.125648147 | SEC63 | protein translocation regulator [Source:HGNC Symbol;Acc:HGNC:21082]" |
| ENSG00000026025 | HGLibA_53655 | 1 | -1.125648147 | VIM | vimentin [Source:HGNC Symbol;Acc:HGNC:12692] |
| ENSG00000026751 | HGLibA_44319 | 1 | -1.125648147 | SLAMF7 | SLAM family member 7 [Source:HGNC Symbol;Acc:HGNC:21394] |
| ENSG00000027697 | HGLibA_22710 | 1 | -1.125648147 | IFNGR1 | interferon gamma receptor 1 [Source:HGNC Symbol;Acc:HGNC:5439] |
| ENSG00000029363 | HGLibA_04352 | 1 | -1.125648147 | BCLAF1 | BCL2 associated transcription factor 1 [Source:HGNC Symbol;Acc:HGNC:16863] |
| ENSG00000033100 | HGLibA_09526 | 1 | -1.125648147 | CHPF2 | chondroitin polymerizing factor 2 [Source:HGNC Symbol;Acc:HGNC:29270] |
| ENSG00000034152 | HGLibA_28144 | 1 | -1.125648147 | MAP2K3 | mitogen-activated protein kinase kinase 3 [Source:HGNC Symbol;Acc:HGNC:6843] |
| ENSG00000036448 | HGLibA_30891 | 1 | -1.125648147 | MYOM2 | myomesin 2 [Source:HGNC Symbol;Acc:HGNC:7614] |
| ENSG00000037637 | HGLibA_17098 | 1 | -1.125648147 | FBXO42 | F-box protein 42 [Source:HGNC Symbol;Acc:HGNC:29249] |
| ENSG00000038532 | HGLibA_09937 | 1 | -1.125648147 | CLEC16A | C-type lectin domain containing 16A [Source:HGNC Symbol;Acc:HGNC:29013] |
| ENSG00000038532 | HGLibA_09938 | 1 | -1.125648147 | CLEC16A | C-type lectin domain containing 16A [Source:HGNC Symbol;Acc:HGNC:29013] |
| ENSG00000039650 | HGLibA_37350 | 1 | -1.125648147 | PNKP | polynucleotide kinase 3'-phosphatase [Source:HGNC Symbol;Acc:HGNC:9154] |
| ENSG00000043355 | HGLibA_55324 | 1 | -1.125648147 | ZIC2 | Zic family member 2 [Source:HGNC Symbol;Acc:HGNC:12873] |
| ENSG00000046604 | HGLibA_14025 | 1 | -1.125648147 | DSG2 | desmoglein 2 [Source:HGNC Symbol;Acc:HGNC:3049] |
| ENSG00000047579 | HGLibA_14069 | 1 | -1.125648147 | DTNBP1 | dystrobrevin binding protein 1 [Source:HGNC Symbol;Acc:HGNC:17328] |
| ENSG00000047662 | HGLibA_16330 | 1 | -1.125648147 | FAM184B | family with sequence similarity 184 member B [Source:HGNC Symbol;Acc:HGNC:29235] |
| ENSG00000050405 | HGLibA_26526 | 1 | -1.125648147 | LIMA1 | LIM domain and actin binding 1 [Source:HGNC Symbol;Acc:HGNC:24636] |
| ENSG00000051620 | HGLibA_21086 | 1 | -1.125648147 | HEBP2 | heme binding protein 2 [Source:HGNC Symbol;Acc:HGNC:15716] |
| ENSG00000052344 | HGLibA_38974 | 1 | -1.125648147 | PRSS8 | serine protease 8 [Source:HGNC Symbol;Acc:HGNC:9491] |
| ENSG00000053524 | HGLibA_28584 | 1 | -1.125648147 | MCF2L2 | MCF.2 cell line derived transforming sequence-like 2 [Source:HGNC Symbol;Acc:HGNC:30319] |
| ENSG00000054277 | HGLibA_33435 | 1 | -1.125648147 | OPN3 | opsin 3 [Source:HGNC Symbol;Acc:HGNC:14007] |
| ENSG00000055163 | HGLibA_12033 | 1 | -1.125648147 | CYFIP2 | cytoplasmic FMR1 interacting protein 2 [Source:HGNC Symbol;Acc:HGNC:13760] |
| ENSG00000055950 | HGLibA_29987 | 1 | -1.125648147 | MRPL43 | mitochondrial ribosomal protein L43 [Source:HGNC Symbol;Acc:HGNC:14517] |
| ENSG00000055957 | HGLibA_23805 | 1 | -1.125648147 | ITIH1 | inter-alpha-trypsin inhibitor heavy chain 1 [Source:HGNC Symbol;Acc:HGNC:6166] |
| ENSG00000064545 | HGLibA_50024 | 1 | -1.125648147 | TMEM161A | transmembrane protein 161A [Source:HGNC Symbol;Acc:HGNC:26020] |
| ENSG00000064547 | HGLibA_27057 | 1 | -1.125648147 | LPAR2 | lysophosphatidic acid receptor 2 [Source:HGNC Symbol;Acc:HGNC:3168] |
| ENSG00000064666 | HGLibA_10307 | 1 | -1.125648147 | CNN2 | calponin 2 [Source:HGNC Symbol;Acc:HGNC:2156] |
| ENSG00000064692 | HGLibA_45911 | 1 | -1.125648147 | SNCAIP | synuclein alpha interacting protein [Source:HGNC Symbol;Acc:HGNC:11139] |
| ENSG00000065613 | HGLibA_45532 | 1 | -1.125648147 | SLK | STE20 like kinase [Source:HGNC Symbol;Acc:HGNC:11088] |
| ENSG00000065675 | HGLibA_38558 | 1 | -1.125648147 | PRKCQ | protein kinase C theta [Source:HGNC Symbol;Acc:HGNC:9410] |
| ENSG00000066032 | HGLibA_11641 | 1 | -1.125648147 | CTNNA2 | catenin alpha 2 [Source:HGNC Symbol;Acc:HGNC:2510] |
| ENSG00000066651 | HGLibA_51669 | 1 | -1.125648147 | TRMT11 | tRNA methyltransferase 11 homolog [Source:HGNC Symbol;Acc:HGNC:21080] |
| ENSG00000066933 | HGLibA_30868 | 1 | -1.125648147 | MYO9A | myosin IXA [Source:HGNC Symbol;Acc:HGNC:7608] |
| ENSG00000067191 | HGLibA_06916 | 1 | -1.125648147 | CACNB1 | calcium voltage-gated channel auxiliary subunit beta 1 [Source:HGNC Symbol;Acc:HGNC:1401] |
| ENSG00000067208 | HGLibA_15692 | 1 | -1.125648147 | EVI5 | ecotropic viral integration site 5 [Source:HGNC Symbol;Acc:HGNC:3501] |
| ENSG00000067369 | HGLibA_51054 | 1 | -1.125648147 | TP53BP1 | tumor protein p53 binding protein 1 [Source:HGNC Symbol;Acc:HGNC:11999] |
| ENSG00000067369 | HGLibA_51055 | 1 | -1.125648147 | TP53BP1 | tumor protein p53 binding protein 1 [Source:HGNC Symbol;Acc:HGNC:11999] |
| ENSG00000067560 | HGLibA_41165 | 1 | -1.125648147 | RHOA | ras homolog family member A [Source:HGNC Symbol;Acc:HGNC:667] |
| ENSG00000067900 | HGLibA_41745 | 1 | -1.125648147 | ROCK1 | Rho associated coiled-coil containing protein kinase 1 [Source:HGNC Symbol;Acc:HGNC:10251] |
| ENSG00000068078 | HGLibA_17448 | 1 | -1.125648147 | FGFR3 | fibroblast growth factor receptor 3 [Source:HGNC Symbol;Acc:HGNC:3690] |
| ENSG00000070404 | HGLibA_18103 | 1 | -1.125648147 | FSTL3 | follistatin like 3 [Source:HGNC Symbol;Acc:HGNC:3973] |
| ENSG00000071243 | HGLibA_23289 | 1 | -1.125648147 | ING3 | inhibitor of growth family member 3 [Source:HGNC Symbol;Acc:HGNC:14587] |
| ENSG00000071794 | HGLibA_21658 | 1 | -1.125648147 | HLTF | helicase like transcription factor [Source:HGNC Symbol;Acc:HGNC:11099] |
| ENSG00000071967 | HGLibA_12023 | 1 | -1.125648147 | CYBRD1 | cytochrome b reductase 1 [Source:HGNC Symbol;Acc:HGNC:20797] |
| ENSG00000072401 | HGLibA_52582 | 1 | -1.125648147 | UBE2D1 | ubiquitin conjugating enzyme E2 D1 [Source:HGNC Symbol;Acc:HGNC:12474] |
| ENSG00000072736 | HGLibA_31782 | 1 | -1.125648147 | NFATC3 | nuclear factor of activated T cells 3 [Source:HGNC Symbol;Acc:HGNC:7777] |
| ENSG00000072832 | HGLibA_11202 | 1 | -1.125648147 | CRMP1 | collapsin response mediator protein 1 [Source:HGNC Symbol;Acc:HGNC:2365] |
| ENSG00000072849 | HGLibA_12998 | 1 | -1.125648147 | DERL2 | derlin 2 [Source:HGNC Symbol;Acc:HGNC:17943] |
| ENSG00000073050 | HGLibA_54584 | 1 | -1.125648147 | XRCC1 | X-ray repair cross complementing 1 [Source:HGNC Symbol;Acc:HGNC:12828] |
| ENSG00000073536 | HGLibA_32064 | 1 | -1.125648147 | NLE1 | notchless homolog 1 [Source:HGNC Symbol;Acc:HGNC:19889] |
| ENSG00000073803 | HGLibA_28171 | 1 | -1.125648147 | MAP3K13 | mitogen-activated protein kinase kinase kinase 13 [Source:HGNC Symbol;Acc:HGNC:6852] |
| ENSG00000074054 | HGLibA_09792 | 1 | -1.125648147 | CLASP1 | cytoplasmic linker associated protein 1 [Source:HGNC Symbol;Acc:HGNC:17088] |
| ENSG00000074803 | HGLibA_44356 | 1 | -1.125648147 | SLC12A1 | solute carrier family 12 member 1 [Source:HGNC Symbol;Acc:HGNC:10910] |
| ENSG00000075275 | HGLibA_09021 | 1 | -1.125648147 | CELSR1 | cadherin EGF LAG seven-pass G-type receptor 1 [Source:HGNC Symbol;Acc:HGNC:1850] |
| ENSG00000075891 | HGLibA_35297 | 1 | -1.125648147 | PAX2 | paired box 2 [Source:HGNC Symbol;Acc:HGNC:8616] |
| ENSG00000076716 | HGLibA_19771 | 1 | -1.125648147 | GPC4 | glypican 4 [Source:HGNC Symbol;Acc:HGNC:4452] |
| ENSG00000077782 | HGLibA_17435 | 1 | -1.125648147 | FGFR1 | fibroblast growth factor receptor 1 [Source:HGNC Symbol;Acc:HGNC:3688] |
| ENSG00000078487 | HGLibA_55057 | 1 | -1.125648147 | ZCWPW1 | zinc finger CW-type and PWWP domain containing 1 [Source:HGNC Symbol;Acc:HGNC:23486] |
| ENSG00000078579 | HGLibA_17392 | 1 | -1.125648147 | FGF20 | fibroblast growth factor 20 [Source:HGNC Symbol;Acc:HGNC:3677] |
| ENSG00000078596 | HGLibA_23829 | 1 | -1.125648147 | ITM2A | integral membrane protein 2A [Source:HGNC Symbol;Acc:HGNC:6173] |
| ENSG00000078674 | HGLibA_35621 | 1 | -1.125648147 | PCM1 | pericentriolar material 1 [Source:HGNC Symbol;Acc:HGNC:8727] |
| ENSG00000078674 | HGLibA_35622 | 1 | -1.125648147 | PCM1 | pericentriolar material 1 [Source:HGNC Symbol;Acc:HGNC:8727] |
| ENSG00000079134 | HGLibA_49365 | 1 | -1.125648147 | THOC1 | THO complex 1 [Source:HGNC Symbol;Acc:HGNC:19070] |
| ENSG00000079215 | HGLibA_44513 | 1 | -1.125648147 | SLC1A3 | solute carrier family 1 member 3 [Source:HGNC Symbol;Acc:HGNC:10941] |
| ENSG00000079246 | HGLibA_54596 | 1 | -1.125648147 | XRCC5 | X-ray repair cross complementing 5 [Source:HGNC Symbol;Acc:HGNC:12833] |
| ENSG00000079435 | HGLibA_26607 | 1 | -1.125648147 | LIPE | hormone sensitive type [Source:HGNC Symbol;Acc:HGNC:6621]" |
| ENSG00000079462 | HGLibA_34996 | 1 | -1.125648147 | PAFAH1B3 | platelet activating factor acetylhydrolase 1b catalytic subunit 3 [Source:HGNC Symbol;Acc:HGNC:8576] |
| ENSG00000079689 | HGLibA_42937 | 1 | -1.125648147 | SCGN | EF-hand calcium binding protein [Source:HGNC Symbol;Acc:HGNC:16941]" |
| ENSG00000079841 | HGLibA_41278 | 1 | -1.125648147 | RIMS1 | regulating synaptic membrane exocytosis 1 [Source:HGNC Symbol;Acc:HGNC:17282] |
| ENSG00000080031 | HGLibA_39508 | 1 | -1.125648147 | PTPRH | receptor type H [Source:HGNC Symbol;Acc:HGNC:9672]" |
| ENSG00000080293 | HGLibA_43068 | 1 | -1.125648147 | SCTR | secretin receptor [Source:HGNC Symbol;Acc:HGNC:10608] |
| ENSG00000080603 | HGLibA_46932 | 1 | -1.125648147 | SRCAP | Snf2 related CREBBP activator protein [Source:HGNC Symbol;Acc:HGNC:16974] |
| ENSG00000081721 | HGLibA_14136 | 1 | -1.125648147 | DUSP12 | dual specificity phosphatase 12 [Source:HGNC Symbol;Acc:HGNC:3067] |
| ENSG00000081870 | HGLibA_22303 | 1 | -1.125648147 | HSPB11 | heat shock protein family B (small) member 11 [Source:HGNC Symbol;Acc:HGNC:25019] |
| ENSG00000082293 | HGLibA_10526 | 1 | -1.125648147 | COL19A1 | collagen type XIX alpha 1 chain [Source:HGNC Symbol;Acc:HGNC:2196] |
| ENSG00000082556 | HGLibA_33445 | 1 | -1.125648147 | OPRK1 | opioid receptor kappa 1 [Source:HGNC Symbol;Acc:HGNC:8154] |
| ENSG00000082701 | HGLibA_20430 | 1 | -1.125648147 | GSK3B | glycogen synthase kinase 3 beta [Source:HGNC Symbol;Acc:HGNC:4617] |
| ENSG00000083807 | HGLibA_44825 | 1 | -1.125648147 | SLC27A5 | solute carrier family 27 member 5 [Source:HGNC Symbol;Acc:HGNC:10999] |
| ENSG00000083812 | HGLibA_55801 | 1 | -1.125648147 | ZNF324 | zinc finger protein 324 [Source:HGNC Symbol;Acc:HGNC:14096] |
| ENSG00000085415 | HGLibA_43275 | 1 | -1.125648147 | SEH1L | SEH1 like nucleoporin [Source:HGNC Symbol;Acc:HGNC:30379] |
| ENSG00000085449 | HGLibA_54005 | 1 | -1.125648147 | WDFY1 | WD repeat and FYVE domain containing 1 [Source:HGNC Symbol;Acc:HGNC:20451] |
| ENSG00000085644 | HGLibA_55591 | 1 | -1.125648147 | ZNF213 | zinc finger protein 213 [Source:HGNC Symbol;Acc:HGNC:13005] |
| ENSG00000085741 | HGLibA_54351 | 1 | -1.125648147 | WNT11 | Wnt family member 11 [Source:HGNC Symbol;Acc:HGNC:12776] |
| ENSG00000085978 | HGLibA_03474 | 1 | -1.125648147 | ATG16L1 | autophagy related 16 like 1 [Source:HGNC Symbol;Acc:HGNC:21498] |
| ENSG00000085998 | HGLibA_37658 | 1 | -1.125648147 | POMGNT1 | 2-) [Source:HGNC Symbol;Acc:HGNC:19139]" |
| ENSG00000086475 | HGLibA_43412 | 1 | -1.125648147 | SEPHS1 | selenophosphate synthetase 1 [Source:HGNC Symbol;Acc:HGNC:19685] |
| ENSG00000087088 | HGLibA_04183 | 1 | -1.125648147 | BAX | apoptosis regulator [Source:HGNC Symbol;Acc:HGNC:959]" |

| | | | | | |
|---|---|---|---|---|---|
| ENSG00000087152 | HGLibA_03858 | 1 | -1.125648147 | ATXN7L3 | ataxin 7 like 3 [Source:HGNC Symbol;Acc:HGNC:25416] |
| ENSG00000088812 | HGLibA_03820 | 1 | -1.125648147 | ATRN | attractin [Source:HGNC Symbol;Acc:HGNC:885] |
| ENSG00000088930 | HGLibA_54606 | 1 | -1.125648147 | XRN2 | 5'-3' exoribonuclease 2 [Source:HGNC Symbol;Acc:HGNC:12836] |
| ENSG00000089116 | HGLibA_26459 | 1 | -1.125648147 | LHX5 | LIM homeobox 5 [Source:HGNC Symbol;Acc:HGNC:14216] |
| ENSG00000089123 | HGLibA_48436 | 1 | -1.125648147 | TASP1 | taspase 1 [Source:HGNC Symbol;Acc:HGNC:15859] |
| ENSG00000089123 | HGLibA_48435 | 1 | -1.125648147 | TASP1 | taspase 1 [Source:HGNC Symbol;Acc:HGNC:15859] |
| ENSG00000089199 | HGLibA_09435 | 1 | -1.125648147 | CHGB | chromogranin B [Source:HGNC Symbol;Acc:HGNC:1930] |
| ENSG00000089472 | HGLibA_21145 | 1 | -1.125648147 | HEPH | hephaestin [Source:HGNC Symbol;Acc:HGNC:4866] |
| ENSG00000090316 | HGLibA_27859 | 1 | -1.125648147 | MAEA | macrophage erythroblast attacher [Source:HGNC Symbol;Acc:HGNC:13731] |
| ENSG00000092140 | HGLibA_18296 | 1 | -1.125648147 | G2E3 | G2/M-phase specific E3 ubiquitin protein ligase [Source:HGNC Symbol;Acc:HGNC:20338] |
| ENSG00000092148 | HGLibA_21093 | 1 | -1.125648147 | HECTD1 | HECT domain E3 ubiquitin protein ligase 1 [Source:HGNC Symbol;Acc:HGNC:20157] |
| ENSG00000093010 | HGLibA_10686 | 1 | -1.125648147 | COMT | catechol-O-methyltransferase [Source:HGNC Symbol;Acc:HGNC:2228] |
| ENSG00000093217 | HGLibA_54617 | 1 | -1.125648147 | XYLB | xylulokinase [Source:HGNC Symbol;Acc:HGNC:12839] |
| ENSG00000094916 | HGLibA_07457 | 1 | -1.125648147 | CBX5 | chromobox 5 [Source:HGNC Symbol;Acc:HGNC:1555] |
| ENSG00000095139 | HGLibA_02636 | 1 | -1.125648147 | ARCN1 | archain 1 [Source:HGNC Symbol;Acc:HGNC:649] |
| ENSG00000095383 | HGLibA_48494 | 1 | -1.125648147 | TBC1D2 | TBC1 domain family member 2 [Source:HGNC Symbol;Acc:HGNC:18026] |
| ENSG00000095539 | HGLibA_43356 | 1 | -1.125648147 | SEMA4G | semaphorin 4G [Source:HGNC Symbol;Acc:HGNC:10735] |
| ENSG00000096093 | HGLibA_14570 | 1 | -1.125648147 | EFHC1 | EF-hand domain containing 1 [Source:HGNC Symbol;Acc:HGNC:16406] |
| ENSG00000099956 | HGLibA_45619 | 1 | -1.125648147 | SMARCB1 | member 1 [Source:HGNC Symbol;Acc:HGNC:11103]" |
| ENSG00000100014 | HGLibA_46607 | 1 | -1.125648147 | SPECC1L | sperm antigen with calponin homology and coiled-coil domains 1 like [Source:HGNC Symbol;Acc:HGNC:29022] |
| ENSG00000100170 | HGLibA_45253 | 1 | -1.125648147 | SLC5A1 | solute carrier family 5 member 1 [Source:HGNC Symbol;Acc:HGNC:11036] |
| ENSG00000100216 | HGLibA_50960 | 1 | -1.125648147 | TOMM22 | translocase of outer mitochondrial membrane 22 [Source:HGNC Symbol;Acc:HGNC:18002] |
| ENSG00000100321 | HGLibA_47992 | 1 | -1.125648147 | SYNGR1 | synaptogyrin 1 [Source:HGNC Symbol;Acc:HGNC:11498] |
| ENSG00000100365 | HGLibA_31301 | 1 | -1.125648147 | NCF4 | neutrophil cytosolic factor 4 [Source:HGNC Symbol;Acc:HGNC:7662] |
| ENSG00000100372 | HGLibA_44658 | 1 | -1.125648147 | SLC25A17 | solute carrier family 25 member 17 [Source:HGNC Symbol;Acc:HGNC:10987] |
| ENSG00000100445 | HGLibA_43164 | 1 | -1.125648147 | SDR39U1 | short chain dehydrogenase/reductase family 39U member 1 [Source:HGNC Symbol;Acc:HGNC:20275] |
| ENSG00000100583 | HGLibA_42676 | 1 | -1.125648147 | SAMD15 | sterile alpha motif domain containing 15 [Source:HGNC Symbol;Acc:HGNC:18631] |
| ENSG00000100629 | HGLibA_09103 | 1 | -1.125648147 | CEP128 | centrosomal protein 128 [Source:HGNC Symbol;Acc:HGNC:20359] |
| ENSG00000100665 | HGLibA_43521 | 1 | -1.125648147 | SERPINA4 | serpin family A member 4 [Source:HGNC Symbol;Acc:HGNC:8948] |
| ENSG00000100722 | HGLibA_54963 | 1 | -1.125648147 | ZC3H14 | zinc finger CCCH-type containing 14 [Source:HGNC Symbol;Acc:HGNC:20509] |
| ENSG00000100767 | HGLibA_35133 | 1 | -1.125648147 | PAPLN | proteoglycan like sulfated glycoprotein [Source:HGNC Symbol;Acc:HGNC:19262]" |
| ENSG00000100906 | HGLibA_31820 | 1 | -1.125648147 | NFKBIA | NFKB inhibitor alpha [Source:HGNC Symbol;Acc:HGNC:7797] |
| ENSG00000100997 | HGLibA_00240 | 1 | -1.125648147 | ABHD12 | abhydrolase domain containing 12 [Source:HGNC Symbol;Acc:HGNC:15868] |
| ENSG00000101150 | HGLibA_51118 | 1 | -1.125648147 | TPD52L2 | TPD52 like 2 [Source:HGNC Symbol;Acc:HGNC:12007] |
| ENSG00000101197 | HGLibA_04526 | 1 | -1.125648147 | BIRC7 | baculoviral IAP repeat containing 7 [Source:HGNC Symbol;Acc:HGNC:13702] |
| ENSG00000101216 | HGLibA_19400 | 1 | -1.125648147 | GMEB2 | glucocorticoid modulatory element binding protein 2 [Source:HGNC Symbol;Acc:HGNC:4371] |
| ENSG00000101230 | HGLibA_23675 | 1 | -1.125648147 | ISM1 | isthmin 1 [Source:HGNC Symbol;Acc:HGNC:16213] |
| ENSG00000101282 | HGLibA_42353 | 1 | -1.125648147 | RSPO4 | R-spondin 4 [Source:HGNC Symbol;Acc:HGNC:16175] |
| ENSG00000101336 | HGLibA_20967 | 1 | -1.125648147 | HCK | Src family tyrosine kinase [Source:HGNC Symbol;Acc:HGNC:4840]" |
| ENSG00000101367 | HGLibA_28313 | 1 | -1.125648147 | MAPRE1 | microtubule associated protein RP/EB family member 1 [Source:HGNC Symbol;Acc:HGNC:6890] |
| ENSG00000101442 | HGLibA_00667 | 1 | -1.125648147 | ACTR5 | ARP5 actin related protein 5 homolog [Source:HGNC Symbol;Acc:HGNC:14671] |
| ENSG00000101452 | HGLibA_13188 | 1 | -1.125648147 | DHX35 | DEAH-box helicase 35 [Source:HGNC Symbol;Acc:HGNC:15861] |
| ENSG00000101470 | HGLibA_50869 | 1 | -1.125648147 | TNNC2 | fast skeletal type [Source:HGNC Symbol;Acc:HGNC:11944]" |
| ENSG00000101557 | HGLibA_53207 | 1 | -1.125648147 | USP14 | ubiquitin specific peptidase 14 [Source:HGNC Symbol;Acc:HGNC:12612] |
| ENSG00000101745 | HGLibA_01997 | 1 | -1.125648147 | ANKRD12 | ankyrin repeat domain 12 [Source:HGNC Symbol;Acc:HGNC:29135] |
| ENSG00000101981 | HGLibA_15894 | 1 | -1.125648147 | F9 | coagulation factor IX [Source:HGNC Symbol;Acc:HGNC:3551] |
| ENSG00000102007 | HGLibA_37220 | 1 | -1.125648147 | PLP2 | proteolipid protein 2 [Source:HGNC Symbol;Acc:HGNC:9087] |
| ENSG00000102030 | HGLibA_30971 | 1 | -1.125648147 | NAA10 | NatA catalytic subunit [Source:HGNC Symbol;Acc:HGNC:18704]" |
| ENSG00000102038 | HGLibA_45602 | 1 | -1.125648147 | SMARCA1 | member 1 [Source:HGNC Symbol;Acc:HGNC:11097]" |
| ENSG00000102054 | HGLibA_40438 | 1 | -1.125648147 | RBBP7 | chromatin remodeling factor [Source:HGNC Symbol;Acc:HGNC:9890]" |
| ENSG00000102316 | HGLibA_27972 | 1 | -1.125648147 | MAGED2 | MAGE family member D2 [Source:HGNC Symbol;Acc:HGNC:16353] |
| ENSG00000102409 | HGLibA_04445 | 1 | -1.125648147 | BEX4 | brain expressed X-linked 4 [Source:HGNC Symbol;Acc:HGNC:25475] |
| ENSG00000102780 | HGLibA_13074 | 1 | -1.125648147 | DGKH | diacylglycerol kinase eta [Source:HGNC Symbol;Acc:HGNC:2854] |
| ENSG00000102893 | HGLibA_36408 | 1 | -1.125648147 | PHKB | phosphorylase kinase regulatory subunit beta [Source:HGNC Symbol;Acc:HGNC:8927] |
| ENSG00000103044 | HGLibA_20869 | 1 | -1.125648147 | HAS3 | hyaluronan synthase 3 [Source:HGNC Symbol;Acc:HGNC:4820] |
| ENSG00000103126 | HGLibA_03920 | 1 | -1.125648147 | AXIN1 | axin 1 [Source:HGNC Symbol;Acc:HGNC:903] |
| ENSG00000103227 | HGLibA_26685 | 1 | -1.125648147 | LMF1 | lipase maturation factor 1 [Source:HGNC Symbol;Acc:HGNC:14154] |
| ENSG00000103319 | HGLibA_14510 | 1 | -1.125648147 | EEF2K | eukaryotic elongation factor 2 kinase [Source:HGNC Symbol;Acc:HGNC:24615] |
| ENSG00000103426 | HGLibA_10795 | 1 | -1.125648147 | CORO7-PAM16 | CORO7-PAM16 readthrough [Source:HGNC Symbol;Acc:HGNC:44424] |
| ENSG00000103569 | HGLibA_02610 | 1 | -1.125648147 | AQP9 | aquaporin 9 [Source:HGNC Symbol;Acc:HGNC:643] |
| ENSG00000103591 | HGLibA_00048 | 1 | -1.125648147 | AAGAB | alpha and gamma adaptin binding protein [Source:HGNC Symbol;Acc:HGNC:25662] |
| ENSG00000103740 | HGLibA_00542 | 1 | -1.125648147 | ACSBG1 | acyl-CoA synthetase bubblegum family member 1 [Source:HGNC Symbol;Acc:HGNC:29567] |
| ENSG00000103852 | HGLibA_52078 | 1 | -1.125648147 | TTC23 | tetratricopeptide repeat domain 23 [Source:HGNC Symbol;Acc:HGNC:25730] |
| ENSG00000104047 | HGLibA_14072 | 1 | -1.125648147 | DTWD1 | DTW domain containing 1 [Source:HGNC Symbol;Acc:HGNC:30926] |
| ENSG00000104081 | HGLibA_04585 | 1 | -1.125648147 | BMF | Bcl2 modifying factor [Source:HGNC Symbol;Acc:HGNC:24132] |
| ENSG00000104213 | HGLibA_35860 | 1 | -1.125648147 | PDGFRL | platelet derived growth factor receptor like [Source:HGNC Symbol;Acc:HGNC:8805] |
| ENSG00000104321 | HGLibA_51724 | 1 | -1.125648147 | TRPA1 | transient receptor potential cation channel subfamily A member 1 [Source:HGNC Symbol;Acc:HGNC:497] |
| ENSG00000104327 | HGLibA_06985 | 1 | -1.125648147 | CALB1 | calbindin 1 [Source:HGNC Symbol;Acc:HGNC:1434] |
| ENSG00000104691 | HGLibA_52799 | 1 | -1.125648147 | UBXN8 | UBX domain protein 8 [Source:HGNC Symbol;Acc:HGNC:30307] |
| ENSG00000104826 | HGLibA_26420 | 1 | -1.125648147 | LHB | luteinizing hormone beta polypeptide [Source:HGNC Symbol;Acc:HGNC:6584] |
| ENSG00000104872 | HGLibA_36610 | 1 | -1.125648147 | PIH1D1 | PIH1 domain containing 1 [Source:HGNC Symbol;Acc:HGNC:26075] |
| ENSG00000104889 | HGLibA_41426 | 1 | -1.125648147 | RNASEH2A | ribonuclease H2 subunit A [Source:HGNC Symbol;Acc:HGNC:18518] |
| ENSG00000104953 | HGLibA_49618 | 1 | -1.125648147 | TLE6 | transducin like enhancer of split 6 [Source:HGNC Symbol;Acc:HGNC:30788] |
| ENSG00000104967 | HGLibA_32366 | 1 | -1.125648147 | NOVA2 | NOVA alternative splicing regulator 2 [Source:HGNC Symbol;Acc:HGNC:7887] |
| ENSG00000105048 | HGLibA_50882 | 1 | -1.125648147 | TNNT1 | slow skeletal type [Source:HGNC Symbol;Acc:HGNC:11948]" |
| ENSG00000105251 | HGLibA_44017 | 1 | -1.125648147 | SHD | Src homology 2 domain containing transforming protein D [Source:HGNC Symbol;Acc:HGNC:30633] |
| ENSG00000105321 | HGLibA_07918 | 1 | -1.125648147 | CCDC9 | coiled-coil domain containing 9 [Source:HGNC Symbol;Acc:HGNC:24560] |
| ENSG00000105364 | HGLibA_29975 | 1 | -1.125648147 | MRPL4 | mitochondrial ribosomal protein L4 [Source:HGNC Symbol;Acc:HGNC:14276] |
| ENSG00000105472 | HGLibA_09926 | 1 | -1.125648147 | CLEC11A | C-type lectin domain containing 11A [Source:HGNC Symbol;Acc:HGNC:10576] |
| ENSG00000105552 | HGLibA_04267 | 1 | -1.125648147 | BCAT2 | branched chain amino acid transaminase 2 [Source:HGNC Symbol;Acc:HGNC:977] |
| ENSG00000105698 | HGLibA_53169 | 1 | -1.125648147 | USF2 | c-fos interacting [Source:HGNC Symbol;Acc:HGNC:12594]" |
| ENSG00000105793 | HGLibA_20600 | 1 | -1.125648147 | GTPBP10 | GTP binding protein 10 [Source:HGNC Symbol;Acc:HGNC:25106] |
| ENSG00000105929 | HGLibA_03708 | 1 | -1.125648147 | ATP6V0A4 | ATPase H+ transporting V0 subunit a4 [Source:HGNC Symbol;Acc:HGNC:866] |
| ENSG00000106031 | HGLibA_21901 | 1 | -1.125648147 | HOXA13 | homeobox A13 [Source:HGNC Symbol;Acc:HGNC:5102] |
| ENSG00000106038 | HGLibA_15708 | 1 | -1.125648147 | EVX1 | even-skipped homeobox 1 [Source:HGNC Symbol;Acc:HGNC:3506] |
| ENSG00000106086 | HGLibA_37084 | 1 | -1.125648147 | PLEKHA8 | pleckstrin homology domain containing A8 [Source:HGNC Symbol;Acc:HGNC:30037] |
| ENSG00000106211 | HGLibA_22301 | 1 | -1.125648147 | HSPB1 | heat shock protein family B (small) member 1 [Source:HGNC Symbol;Acc:HGNC:5246] |
| ENSG00000106460 | HGLibA_49857 | 1 | -1.125648147 | TMEM106B | transmembrane protein 106B [Source:HGNC Symbol;Acc:HGNC:22407] |
| ENSG00000106526 | HGLibA_00665 | 1 | -1.125648147 | ACTR3C | ARP3 actin related protein 3 homolog C [Source:HGNC Symbol;Acc:HGNC:37282] |
| ENSG00000106608 | HGLibA_53140 | 1 | -1.125648147 | URGCP | upregulator of cell proliferation [Source:HGNC Symbol;Acc:HGNC:30890] |
| ENSG00000106615 | HGLibA_41155 | 1 | -1.125648147 | RHEB | mTORC1 binding [Source:HGNC Symbol;Acc:HGNC:10011]" |
| ENSG00000106665 | HGLibA_10045 | 1 | -1.125648147 | CLIP2 | CAP-Gly domain containing linker protein 2 [Source:HGNC Symbol;Acc:HGNC:2586] |
| ENSG00000106809 | HGLibA_33327 | 1 | -1.125648147 | OGN | osteoglycin [Source:HGNC Symbol;Acc:HGNC:8126] |
| ENSG00000106952 | HGLibA_50825 | 1 | -1.125648147 | TNFSF8 | TNF superfamily member 8 [Source:HGNC Symbol;Acc:HGNC:11938] |
| ENSG00000107014 | HGLibA_41356 | 1 | -1.125648147 | RLN2 | relaxin 2 [Source:HGNC Symbol;Acc:HGNC:10027] |
| ENSG00000107130 | HGLibA_31373 | 1 | -1.125648147 | NCS1 | neuronal calcium sensor 1 [Source:HGNC Symbol;Acc:HGNC:3953] |
| ENSG00000107518 | HGLibA_03821 | 1 | -1.125648147 | ATRNL1 | attractin like 1 [Source:HGNC Symbol;Acc:HGNC:29063] |
| ENSG00000107560 | HGLibA_39795 | 1 | -1.125648147 | RAB11FIP2 | RAB11 family interacting protein 2 [Source:HGNC Symbol;Acc:HGNC:29152] |
| ENSG00000107611 | HGLibA_11764 | 1 | -1.125648147 | CUBN | cubilin [Source:HGNC Symbol;Acc:HGNC:2548] |
| ENSG00000107862 | HGLibA_18722 | 1 | -1.125648147 | GBF1 | golgi brefeldin A resistant guanine nucleotide exchange factor 1 [Source:HGNC Symbol;Acc:HGNC:4181] |
| ENSG00000107949 | HGLibA_04270 | 1 | -1.125648147 | BCCIP | BRCA2 and CDKN1A interacting protein [Source:HGNC Symbol;Acc:HGNC:978] |
| ENSG00000107968 | HGLibA_28201 | 1 | -1.125648147 | MAP3K8 | mitogen-activated protein kinase kinase kinase 8 [Source:HGNC Symbol;Acc:HGNC:6860] |
| ENSG00000108219 | HGLibA_51905 | 1 | -1.125648147 | TSPAN14 | tetraspanin 14 [Source:HGNC Symbol;Acc:HGNC:23303] |
| ENSG00000108506 | HGLibA_23416 | 1 | -1.125648147 | INTS2 | integrator complex subunit 2 [Source:HGNC Symbol;Acc:HGNC:29241] |
| ENSG00000108510 | HGLibA_28749 | 1 | -1.125648147 | MED13 | mediator complex subunit 13 [Source:HGNC Symbol;Acc:HGNC:22474] |
| ENSG00000108528 | HGLibA_44638 | 1 | -1.125648147 | SLC25A11 | solute carrier family 25 member 11 [Source:HGNC Symbol;Acc:HGNC:10981] |
| ENSG00000108591 | HGLibA_13977 | 1 | -1.125648147 | DRG2 | developmentally regulated GTP binding protein 2 [Source:HGNC Symbol;Acc:HGNC:3030] |
| ENSG00000108622 | HGLibA_22497 | 1 | -1.125648147 | ICAM2 | intercellular adhesion molecule 2 [Source:HGNC Symbol;Acc:HGNC:5345] |
| ENSG00000108759 | HGLibA_25478 | 1 | -1.125648147 | KRT32 | keratin 32 [Source:HGNC Symbol;Acc:HGNC:6449] |
| ENSG00000108828 | HGLibA_53543 | 1 | -1.125648147 | VAT1 | vesicle amine transport 1 [Source:HGNC Symbol;Acc:HGNC:16919] |
| ENSG00000108932 | HGLibA_44448 | 1 | -1.125648147 | SLC16A6 | solute carrier family 16 member 6 [Source:HGNC Symbol;Acc:HGNC:10927] |
| ENSG00000109171 | HGLibA_44309 | 1 | -1.125648147 | SLAIN2 | SLAIN motif family member 2 [Source:HGNC Symbol;Acc:HGNC:29282] |
| ENSG00000109205 | HGLibA_33268 | 1 | -1.125648147 | ODAM | ameloblast associated [Source:HGNC Symbol;Acc:HGNC:26043]" |
| ENSG00000109208 | HGLibA_45825 | 1 | -1.125648147 | SMR3A | submaxillary gland androgen regulated protein 3A [Source:HGNC Symbol;Acc:HGNC:19216] |
| ENSG00000109466 | HGLibA_25147 | 1 | -1.125648147 | KLHL2 | kelch like family member 2 [Source:HGNC Symbol;Acc:HGNC:6353] |
| ENSG00000109586 | HGLibA_18569 | 1 | -1.125648147 | GALNT7 | polypeptide N-acetylgalactosaminyltransferase 7 [Source:HGNC Symbol;Acc:HGNC:4129] |
| ENSG00000109670 | HGLibA_17153 | 1 | -1.125648147 | FBXW7 | F-box and WD repeat domain containing 7 [Source:HGNC Symbol;Acc:HGNC:16712] |
| ENSG00000109705 | HGLibA_32052 | 1 | -1.125648147 | NKX3-2 | NK3 homeobox 2 [Source:HGNC Symbol;Acc:HGNC:951] |
| ENSG00000110422 | HGLibA_21345 | 1 | -1.125648147 | HIPK3 | homeodomain interacting protein kinase 3 [Source:HGNC Symbol;Acc:HGNC:4915] |
| ENSG00000110446 | HGLibA_44411 | 1 | -1.125648147 | SLC15A3 | solute carrier family 15 member 3 [Source:HGNC Symbol;Acc:HGNC:18068] |
| ENSG00000110583 | HGLibA_30997 | 1 | -1.125648147 | NAA40 | NatD catalytic subunit [Source:HGNC Symbol;Acc:HGNC:25845]" |
| ENSG00000110917 | HGLibA_29369 | 1 | -1.125648147 | MLEC | malectin [Source:HGNC Symbol;Acc:HGNC:28973] |
| ENSG00000111057 | HGLibA_25437 | 1 | -1.125648147 | KRT18 | keratin 18 [Source:HGNC Symbol;Acc:HGNC:6430] |
| ENSG00000111196 | HGLibA_28009 | 1 | -1.125648147 | MAGOHB | exon junction complex subunit [Source:HGNC Symbol;Acc:HGNC:25504]" |
| ENSG00000111291 | HGLibA_20134 | 1 | -1.125648147 | GPRC5D | G protein-coupled receptor class C group 5 member D [Source:HGNC Symbol;Acc:HGNC:13310] |
| ENSG00000111450 | HGLibA_47642 | 1 | -1.125648147 | STX2 | syntaxin 2 [Source:HGNC Symbol;Acc:HGNC:3403] |
| ENSG00000111666 | HGLibA_09529 | 1 | -1.125648147 | CHPT1 | choline phosphotransferase 1 [Source:HGNC Symbol;Acc:HGNC:17852] |
| ENSG00000111676 | HGLibA_03531 | 1 | -1.125648147 | ATN1 | atrophin 1 [Source:HGNC Symbol;Acc:HGNC:3033] |
| ENSG00000111711 | HGLibA_19667 | 1 | -1.125648147 | GOLT1B | golgi transport 1B [Source:HGNC Symbol;Acc:HGNC:20175] |
| ENSG00000111859 | HGLibA_31614 | 1 | -1.125648147 | NEDD9 | developmentally down-regulated 9 [Source:HGNC Symbol;Acc:HGNC:7733]" |
| ENSG00000111863 | HGLibA_01082 | 1 | -1.125648147 | ADTRP | androgen dependent TFPI regulating protein [Source:HGNC Symbol;Acc:HGNC:21214] |
| ENSG00000111877 | HGLibA_28628 | 1 | -1.125648147 | MCM9 | minichromosome maintenance 9 homologous recombination repair factor [Source:HGNC Symbol;Acc:HGNC:21484] |
| ENSG00000111911 | HGLibA_21331 | 1 | -1.125648147 | HINT3 | histidine triad nucleotide binding protein 3 [Source:HGNC Symbol;Acc:HGNC:18468] |
| ENSG00000112200 | HGLibA_56030 | 1 | -1.125648147 | ZNF451 | zinc finger protein 451 [Source:HGNC Symbol;Acc:HGNC:21091] |
| ENSG00000112339 | HGLibA_20939 | 1 | -1.125648147 | HBS1L | HBS1 like translational GTPase [Source:HGNC Symbol;Acc:HGNC:4834] |

| | | | | | |
|---|---|---|---|---|---|
| ENSG00000112406 | HGLibA_21089 | 1 | -1.125648147 | HECA | cell cycle regulator [Source:HGNC Symbol;Acc:HGNC:21041]" |
| ENSG00000112406 | HGLibA_21091 | 1 | -1.125648147 | HECA | cell cycle regulator [Source:HGNC Symbol;Acc:HGNC:21041]" |
| ENSG00000112619 | HGLibA_38753 | 1 | -1.125648147 | PRPH2 | peripherin 2 [Source:HGNC Symbol;Acc:HGNC:9942] |
| ENSG00000112697 | HGLibA_50360 | 1 | -1.125648147 | TMEM30A | transmembrane protein 30A [Source:HGNC Symbol;Acc:HGNC:16667] |
| ENSG00000112796 | HGLibA_15178 | 1 | -1.125648147 | ENPP5 | ectonucleotide pyrophosphatase/phosphodiesterase 5 (putative) [Source:HGNC Symbol;Acc:HGNC:13717] |
| ENSG00000113070 | HGLibA_20922 | 1 | -1.125648147 | HBEGF | heparin binding EGF like growth factor [Source:HGNC Symbol;Acc:HGNC:3059] |
| ENSG00000113212 | HGLibA_35508 | 1 | -1.125648147 | PCDHB7 | protocadherin beta 7 [Source:HGNC Symbol;Acc:HGNC:8692] |
| ENSG00000113593 | HGLibA_38241 | 1 | -1.125648147 | PPWD1 | peptidylprolyl isomerase domain and WD repeat containing 1 [Source:HGNC Symbol;Acc:HGNC:28954] |
| ENSG00000113594 | HGLibA_26477 | 1 | -1.125648147 | LIFR | LIF receptor alpha [Source:HGNC Symbol;Acc:HGNC:6597] |
| ENSG00000113594 | HGLibA_26478 | 1 | -1.125648147 | LIFR | LIF receptor alpha [Source:HGNC Symbol;Acc:HGNC:6597] |
| ENSG00000113722 | HGLibA_08897 | 1 | -1.125648147 | CDX1 | caudal type homeobox 1 [Source:HGNC Symbol;Acc:HGNC:1805] |
| ENSG00000113761 | HGLibA_55849 | 1 | -1.125648147 | ZNF346 | zinc finger protein 346 [Source:HGNC Symbol;Acc:HGNC:16403] |
| ENSG00000114268 | HGLibA_36162 | 1 | -1.125648147 | PFKFB4 | 6-biphosphatase 4 [Source:HGNC Symbol;Acc:HGNC:8875]" |
| ENSG00000114439 | HGLibA_04229 | 1 | -1.125648147 | BBX | HMG-box containing [Source:HGNC Symbol;Acc:HGNC:14422]" |
| ENSG00000114529 | HGLibA_06248 | 1 | -1.125648147 | C3orf52 | chromosome 3 open reading frame 52 [Source:HGNC Symbol;Acc:HGNC:26255] |
| ENSG00000114529 | HGLibA_06249 | 1 | -1.125648147 | C3orf52 | chromosome 3 open reading frame 52 [Source:HGNC Symbol;Acc:HGNC:26255] |
| ENSG00000114686 | HGLibA_29946 | 1 | -1.125648147 | MRPL3 | mitochondrial ribosomal protein L3 [Source:HGNC Symbol;Acc:HGNC:10379] |
| ENSG00000114698 | HGLibA_37239 | 1 | -1.125648147 | PLSCR4 | phospholipid scramblase 4 [Source:HGNC Symbol;Acc:HGNC:16497] |
| ENSG00000114735 | HGLibA_21133 | 1 | -1.125648147 | HEMK1 | HemK methyltransferase family member 1 [Source:HGNC Symbol;Acc:HGNC:24923] |
| ENSG00000114805 | HGLibA_37007 | 1 | -1.125648147 | PLCH1 | phospholipase C eta 1 [Source:HGNC Symbol;Acc:HGNC:29185] |
| ENSG00000114841 | HGLibA_13469 | 1 | -1.125648147 | DNAH1 | dynein axonemal heavy chain 1 [Source:HGNC Symbol;Acc:HGNC:2940] |
| ENSG00000115107 | HGLibA_47429 | 1 | -1.125648147 | STEAP3 | STEAP3 metalloreductase [Source:HGNC Symbol;Acc:HGNC:24592] |
| ENSG00000115112 | HGLibA_49148 | 1 | -1.125648147 | TFCP2L1 | transcription factor CP2 like 1 [Source:HGNC Symbol;Acc:HGNC:17925] |
| ENSG00000115129 | HGLibA_51065 | 1 | -1.125648147 | TP53I3 | tumor protein p53 inducible protein 3 [Source:HGNC Symbol;Acc:HGNC:19373] |
| ENSG00000115233 | HGLibA_39163 | 1 | -1.125648147 | PSMD14 | non-ATPase 14 [Source:HGNC Symbol;Acc:HGNC:16889]" |
| ENSG00000115386 | HGLibA_40795 | 1 | -1.125648147 | REG1A | regenerating family member 1 alpha [Source:HGNC Symbol;Acc:HGNC:9951] |
| ENSG00000115421 | HGLibA_35142 | 1 | -1.125648147 | PAPOLG | poly(A) polymerase gamma [Source:HGNC Symbol;Acc:HGNC:14982] |
| ENSG00000115461 | HGLibA_22827 | 1 | -1.125648147 | IGFBP5 | insulin like growth factor binding protein 5 [Source:HGNC Symbol;Acc:HGNC:5474] |
| ENSG00000115474 | HGLibA_24259 | 1 | -1.125648147 | KCNJ13 | potassium voltage-gated channel subfamily J member 13 [Source:HGNC Symbol;Acc:HGNC:6259] |
| ENSG00000115486 | HGLibA_18995 | 1 | -1.125648147 | GGCX | gamma-glutamyl carboxylase [Source:HGNC Symbol;Acc:HGNC:4247] |
| ENSG00000115523 | HGLibA_19557 | 1 | -1.125648147 | GNLY | granulysin [Source:HGNC Symbol;Acc:HGNC:4414] |
| ENSG00000115568 | HGLibA_55490 | 1 | -1.125648147 | ZNF142 | zinc finger protein 142 [Source:HGNC Symbol;Acc:HGNC:12927] |
| ENSG00000115641 | HGLibA_17483 | 1 | -1.125648147 | FHL2 | four and a half LIM domains 2 [Source:HGNC Symbol;Acc:HGNC:3703] |
| ENSG00000115970 | HGLibA_49284 | 1 | -1.125648147 | THADA | armadillo repeat containing [Source:HGNC Symbol;Acc:HGNC:19217]" |
| ENSG00000116096 | HGLibA_46787 | 1 | -1.125648147 | SPR | sepiapterin reductase [Source:HGNC Symbol;Acc:HGNC:11257] |
| ENSG00000116117 | HGLibA_35182 | 1 | -1.125648147 | PARD3B | par-3 family cell polarity regulator beta [Source:HGNC Symbol;Acc:HGNC:14446] |
| ENSG00000116151 | HGLibA_29665 | 1 | -1.125648147 | MORN1 | MORN repeat containing 1 [Source:HGNC Symbol;Acc:HGNC:25852] |
| ENSG00000116176 | HGLibA_51200 | 1 | -1.125648147 | TPSG1 | tryptase gamma 1 [Source:HGNC Symbol;Acc:HGNC:14134] |
| ENSG00000116183 | HGLibA_35147 | 1 | -1.125648147 | PAPPA2 | pappalysin 2 [Source:HGNC Symbol;Acc:HGNC:14615] |
| ENSG00000116285 | HGLibA_15533 | 1 | -1.125648147 | ERRFI1 | ERBB receptor feedback inhibitor 1 [Source:HGNC Symbol;Acc:HGNC:18185] |
| ENSG00000116350 | HGLibA_47078 | 1 | -1.125648147 | SRSF4 | serine and arginine rich splicing factor 4 [Source:HGNC Symbol;Acc:HGNC:10786] |
| ENSG00000116455 | HGLibA_54180 | 1 | -1.125648147 | WDR77 | WD repeat domain 77 [Source:HGNC Symbol;Acc:HGNC:29652] |
| ENSG00000116819 | HGLibA_49133 | 1 | -1.125648147 | TFAP2E | transcription factor AP-2 epsilon [Source:HGNC Symbol;Acc:HGNC:30774] |
| ENSG00000116852 | HGLibA_24900 | 1 | -1.125648147 | KIF21B | kinesin family member 21B [Source:HGNC Symbol;Acc:HGNC:29442] |
| ENSG00000117069 | HGLibA_47276 | 1 | -1.125648147 | ST6GALNAC5 | 6-sialyltransferase 5 [Source:HGNC Symbol;Acc:HGNC:19342]" |
| ENSG00000117122 | HGLibA_29042 | 1 | -1.125648147 | MFAP2 | microfibril associated protein 2 [Source:HGNC Symbol;Acc:HGNC:7033] |
| ENSG00000117411 | HGLibA_04026 | 1 | -1.125648147 | B4GALT2 | 4-galactosyltransferase 2 [Source:HGNC Symbol;Acc:HGNC:925]" |
| ENSG00000117419 | HGLibA_15486 | 1 | -1.125648147 | ERI3 | ERI1 exoribonuclease family member 3 [Source:HGNC Symbol;Acc:HGNC:17276] |
| ENSG00000117448 | HGLibA_01468 | 1 | -1.125648147 | AKR1A1 | aldo-keto reductase family 1 member A1 [Source:HGNC Symbol;Acc:HGNC:380] |
| ENSG00000117533 | HGLibA_53497 | 1 | -1.125648147 | VAMP4 | vesicle associated membrane protein 4 [Source:HGNC Symbol;Acc:HGNC:12645] |
| ENSG00000117632 | HGLibA_47516 | 1 | -1.125648147 | STMN1 | stathmin 1 [Source:HGNC Symbol;Acc:HGNC:6510] |
| ENSG00000117713 | HGLibA_02882 | 1 | -1.125648147 | ARID1A | AT-rich interaction domain 1A [Source:HGNC Symbol;Acc:HGNC:11110] |
| ENSG00000118017 | HGLibA_00019 | 1 | -1.125648147 | A4GNT | 4-N-acetylglucosaminyltransferase [Source:HGNC Symbol;Acc:HGNC:17968]" |
| ENSG00000118257 | HGLibA_32719 | 1 | -1.125648147 | NRP2 | neuropilin 2 [Source:HGNC Symbol;Acc:HGNC:8005] |
| ENSG00000118271 | HGLibA_52229 | 1 | -1.125648147 | TTR | transthyretin [Source:HGNC Symbol;Acc:HGNC:12405] |
| ENSG00000118369 | HGLibA_53344 | 1 | -1.125648147 | USP35 | ubiquitin specific peptidase 35 [Source:HGNC Symbol;Acc:HGNC:20061] |
| ENSG00000118432 | HGLibA_10374 | 1 | -1.125648147 | CNR1 | cannabinoid receptor 1 [Source:HGNC Symbol;Acc:HGNC:2159] |
| ENSG00000118454 | HGLibA_02006 | 1 | -1.125648147 | ANKRD13C | ankyrin repeat domain 13C [Source:HGNC Symbol;Acc:HGNC:25374] |
| ENSG00000118508 | HGLibA_39878 | 1 | -1.125648147 | RAB32 | member RAS oncogene family [Source:HGNC Symbol;Acc:HGNC:9772]" |
| ENSG00000118702 | HGLibA_19044 | 1 | -1.125648147 | GHRH | growth hormone releasing hormone [Source:HGNC Symbol;Acc:HGNC:4265] |
| ENSG00000118960 | HGLibA_22116 | 1 | -1.125648147 | HS1BP3 | HCLS1 binding protein 3 [Source:HGNC Symbol;Acc:HGNC:24979] |
| ENSG00000118972 | HGLibA_17402 | 1 | -1.125648147 | FGF23 | fibroblast growth factor 23 [Source:HGNC Symbol;Acc:HGNC:3680] |
| ENSG00000119231 | HGLibA_43395 | 1 | -1.125648147 | SENP5 | SUMO specific peptidase 5 [Source:HGNC Symbol;Acc:HGNC:28407] |
| ENSG00000119411 | HGLibA_04879 | 1 | -1.125648147 | BSPRY | B-box and SPRY domain containing [Source:HGNC Symbol;Acc:HGNC:18232] |
| ENSG00000119446 | HGLibA_40500 | 1 | -1.125648147 | RBM18 | RNA binding motif protein 18 [Source:HGNC Symbol;Acc:HGNC:28413] |
| ENSG00000119535 | HGLibA_11341 | 1 | -1.125648147 | CSF3R | colony stimulating factor 3 receptor [Source:HGNC Symbol;Acc:HGNC:2439] |
| ENSG00000119574 | HGLibA_54895 | 1 | -1.125648147 | ZBTB45 | zinc finger and BTB domain containing 45 [Source:HGNC Symbol;Acc:HGNC:23715] |
| ENSG00000119614 | HGLibA_53879 | 1 | -1.125648147 | VSX2 | visual system homeobox 2 [Source:HGNC Symbol;Acc:HGNC:1975] |
| ENSG00000119638 | HGLibA_31668 | 1 | -1.125648147 | NEK9 | NIMA related kinase 9 [Source:HGNC Symbol;Acc:HGNC:18591] |
| ENSG00000119689 | HGLibA_13369 | 1 | -1.125648147 | DLST | dihydrolipoamide S-succinyltransferase [Source:HGNC Symbol;Acc:HGNC:2911] |
| ENSG00000119915 | HGLibA_14963 | 1 | -1.125648147 | ELOVL3 | ELOVL fatty acid elongase 3 [Source:HGNC Symbol;Acc:HGNC:18047] |
| ENSG00000119922 | HGLibA_22629 | 1 | -1.125648147 | IFIT2 | interferon induced protein with tetratricopeptide repeats 2 [Source:HGNC Symbol;Acc:HGNC:5409] |
| ENSG00000120251 | HGLibA_20262 | 1 | -1.125648147 | GRIA2 | glutamate ionotropic receptor AMPA type subunit 2 [Source:HGNC Symbol;Acc:HGNC:4572] |
| ENSG00000120370 | HGLibA_19675 | 1 | -1.125648147 | GORAB | RAB6 interacting [Source:HGNC Symbol;Acc:HGNC:25676]" |
| ENSG00000120498 | HGLibA_49052 | 1 | -1.125648147 | TEX11 | testis expressed 11 [Source:HGNC Symbol;Acc:HGNC:11733] |
| ENSG00000120675 | HGLibA_13579 | 1 | -1.125648147 | DNAJC15 | DnaJ heat shock protein family (Hsp40) member C15 [Source:HGNC Symbol;Acc:HGNC:20325] |
| ENSG00000120694 | HGLibA_22344 | 1 | -1.125648147 | HSPH1 | heat shock protein family H (Hsp110) member 1 [Source:HGNC Symbol;Acc:HGNC:16969] |
| ENSG00000120802 | HGLibA_50605 | 1 | -1.125648147 | TMPO | thymopoietin [Source:HGNC Symbol;Acc:HGNC:11875] |
| ENSG00000120952 | HGLibA_38304 | 1 | -1.125648147 | PRAMEF2 | PRAME family member 2 [Source:HGNC Symbol;Acc:HGNC:28841] |
| ENSG00000121075 | HGLibA_48672 | 1 | -1.125648147 | TBX4 | T-box 4 [Source:HGNC Symbol;Acc:HGNC:11603] |
| ENSG00000121314 | HGLibA_48429 | 1 | -1.125648147 | TAS2R8 | taste 2 receptor member 8 [Source:HGNC Symbol;Acc:HGNC:14915] |
| ENSG00000121671 | HGLibA_11242 | 1 | -1.125648147 | CRY2 | cryptochrome circadian regulator 2 [Source:HGNC Symbol;Acc:HGNC:2385] |
| ENSG00000121766 | HGLibA_55020 | 1 | -1.125648147 | ZCCHC17 | zinc finger CCHC-type containing 17 [Source:HGNC Symbol;Acc:HGNC:30246] |
| ENSG00000121897 | HGLibA_26470 | 1 | -1.125648147 | LIAS | lipoic acid synthetase [Source:HGNC Symbol;Acc:HGNC:16429] |
| ENSG00000121905 | HGLibA_22016 | 1 | -1.125648147 | HPCA | hippocalcin [Source:HGNC Symbol;Acc:HGNC:5144] |
| ENSG00000122068 | HGLibA_18257 | 1 | -1.125648147 | FYTTD1 | forty-two-three domain containing 1 [Source:HGNC Symbol;Acc:HGNC:25407] |
| ENSG00000122223 | HGLibA_08307 | 1 | -1.125648147 | CD244 | CD244 molecule [Source:HGNC Symbol;Acc:HGNC:18171] |
| ENSG00000122223 | HGLibA_08305 | 1 | -1.125648147 | CD244 | CD244 molecule [Source:HGNC Symbol;Acc:HGNC:18171] |
| ENSG00000122515 | HGLibA_55383 | 1 | -1.125648147 | ZMIZ2 | zinc finger MIZ-type containing 2 [Source:HGNC Symbol;Acc:HGNC:22229] |
| ENSG00000122778 | HGLibA_24779 | 1 | -1.125648147 | KIAA1549 | KIAA1549 [Source:HGNC Symbol;Acc:HGNC:22219] |
| ENSG00000122824 | HGLibA_32933 | 1 | -1.125648147 | NUDT10 | nudix hydrolase 10 [Source:HGNC Symbol;Acc:HGNC:17621] |
| ENSG00000123094 | HGLibA_40396 | 1 | -1.125648147 | RASSF8 | Ras association domain family member 8 [Source:HGNC Symbol;Acc:HGNC:13232] |
| ENSG00000123213 | HGLibA_32084 | 1 | -1.125648147 | NLN | neurolysin [Source:HGNC Symbol;Acc:HGNC:16058] |
| ENSG00000123338 | HGLibA_31315 | 1 | -1.125648147 | NCKAP1L | NCK associated protein 1 like [Source:HGNC Symbol;Acc:HGNC:4862] |
| ENSG00000123415 | HGLibA_45842 | 1 | -1.125648147 | SMUG1 | single-strand-selective monofunctional uracil-DNA glycosylase 1 [Source:HGNC Symbol;Acc:HGNC:17148] |
| ENSG00000123728 | HGLibA_40242 | 1 | -1.125648147 | RAP2C | member of RAS oncogene family [Source:HGNC Symbol;Acc:HGNC:21165]" |
| ENSG00000123836 | HGLibA_36155 | 1 | -1.125648147 | PFKFB2 | 6-biphosphatase 2 [Source:HGNC Symbol;Acc:HGNC:8873]" |
| ENSG00000124196 | HGLibA_20630 | 1 | -1.125648147 | GTSF1L | gametocyte specific factor 1 like [Source:HGNC Symbol;Acc:HGNC:16198] |
| ENSG00000124215 | HGLibA_08673 | 1 | -1.125648147 | CDH26 | cadherin 26 [Source:HGNC Symbol;Acc:HGNC:15902] |
| ENSG00000124216 | HGLibA_45867 | 1 | -1.125648147 | SNAI1 | snail family transcriptional repressor 1 [Source:HGNC Symbol;Acc:HGNC:11128] |
| ENSG00000124260 | HGLibA_27893 | 1 | -1.125648147 | MAGEA10 | MAGE family member A10 [Source:HGNC Symbol;Acc:HGNC:6797] |
| ENSG00000125447 | HGLibA_18987 | 1 | -1.125648147 | GGA3 | ARF binding protein 3 [Source:HGNC Symbol;Acc:HGNC:17079]" |
| ENSG00000125498 | HGLibA_24983 | 1 | -1.125648147 | KIR2DL1 | two Ig domains and long cytoplasmic tail 1 [Source:HGNC Symbol;Acc:HGNC:6329]" |
| ENSG00000125534 | HGLibA_37865 | 1 | -1.125648147 | PPDPF | pancreatic progenitor cell differentiation and proliferation factor [Source:HGNC Symbol;Acc:HGNC:16142] |
| ENSG00000125726 | HGLibA_08439 | 1 | -1.125648147 | CD70 | CD70 molecule [Source:HGNC Symbol;Acc:HGNC:11937] |
| ENSG00000125734 | HGLibA_19846 | 1 | -1.125648147 | GPR108 | G protein-coupled receptor 108 [Source:HGNC Symbol;Acc:HGNC:17829] |
| ENSG00000125818 | HGLibA_39202 | 1 | -1.125648147 | PSMF1 | proteasome inhibitor subunit 1 [Source:HGNC Symbol;Acc:HGNC:9571] |
| ENSG00000125820 | HGLibA_32033 | 1 | -1.125648147 | NKX2-2 | NK2 homeobox 2 [Source:HGNC Symbol;Acc:HGNC:7835] |
| ENSG00000125831 | HGLibA_11446 | 1 | -1.125648147 | CST11 | cystatin 11 [Source:HGNC Symbol;Acc:HGNC:15959] |
| ENSG00000125868 | HGLibA_14047 | 1 | -1.125648147 | DSTN | actin depolymerizing factor [Source:HGNC Symbol;Acc:HGNC:15750]" |
| ENSG00000125871 | HGLibA_29161 | 1 | -1.125648147 | MGME1 | mitochondrial genome maintenance exonuclease 1 [Source:HGNC Symbol;Acc:HGNC:16205] |
| ENSG00000126070 | HGLibA_01210 | 1 | -1.125648147 | AGO3 | RISC catalytic component [Source:HGNC Symbol;Acc:HGNC:18421]" |
| ENSG00000126247 | HGLibA_07185 | 1 | -1.125648147 | CAPNS1 | calpain small subunit 1 [Source:HGNC Symbol;Acc:HGNC:1481] |
| ENSG00000126259 | HGLibA_25002 | 1 | -1.125648147 | KIRREL2 | kirre like nephrin family adhesion molecule 2 [Source:HGNC Symbol;Acc:HGNC:18816] |
| ENSG00000126267 | HGLibA_10847 | 1 | -1.125648147 | COX6B1 | cytochrome c oxidase subunit 6B1 [Source:HGNC Symbol;Acc:HGNC:2280] |
| ENSG00000126353 | HGLibA_08166 | 1 | -1.125648147 | CCR7 | C-C motif chemokine receptor 7 [Source:HGNC Symbol;Acc:HGNC:1608] |
| ENSG00000126500 | HGLibA_17653 | 1 | -1.125648147 | FLRT1 | fibronectin leucine rich transmembrane protein 1 [Source:HGNC Symbol;Acc:HGNC:3760] |
| ENSG00000126756 | HGLibA_53482 | 1 | -1.125648147 | UXT | ubiquitously expressed prefoldin like chaperone [Source:HGNC Symbol;Acc:HGNC:12641] |
| ENSG00000126785 | HGLibA_41196 | 1 | -1.125648147 | RHOJ | ras homolog family member J [Source:HGNC Symbol;Acc:HGNC:688] |
| ENSG00000127152 | HGLibA_04292 | 1 | -1.125648147 | BCL11B | B cell CLL/lymphoma 11B [Source:HGNC Symbol;Acc:HGNC:13222] |
| ENSG00000127334 | HGLibA_14276 | 1 | -1.125648147 | DYRK2 | dual specificity tyrosine phosphorylation regulated kinase 2 [Source:HGNC Symbol;Acc:HGNC:3093] |
| ENSG00000127920 | HGLibA_19510 | 1 | -1.125648147 | GNG11 | G protein subunit gamma 11 [Source:HGNC Symbol;Acc:HGNC:4403] |
| ENSG00000128011 | HGLibA_27152 | 1 | -1.125648147 | LRFN1 | leucine rich repeat and fibronectin type III domain containing 1 [Source:HGNC Symbol;Acc:HGNC:29290] |
| ENSG00000128191 | HGLibA_13055 | 1 | -1.125648147 | DGCR8 | microprocessor complex subunit [Source:HGNC Symbol;Acc:HGNC:2847]" |
| ENSG00000128250 | HGLibA_40930 | 1 | -1.125648147 | RFPL1 | ret finger protein like 1 [Source:HGNC Symbol;Acc:HGNC:9977] |
| ENSG00000128274 | HGLibA_00017 | 1 | -1.125648147 | A4GALT | 4-galactosyltransferase (P blood group) [Source:HGNC Symbol;Acc:HGNC:18149]" |
| ENSG00000128309 | HGLibA_29779 | 1 | -1.125648147 | MPST | mercaptopyruvate sulfurtransferase [Source:HGNC Symbol;Acc:HGNC:7223] |
| ENSG00000128335 | HGLibA_02531 | 1 | -1.125648147 | APOL2 | apolipoprotein L2 [Source:HGNC Symbol;Acc:HGNC:619] |
| ENSG00000128340 | HGLibA_40045 | 1 | -1.125648147 | RAC2 | Rac family small GTPase 2 [Source:HGNC Symbol;Acc:HGNC:9802] |
| ENSG00000128536 | HGLibA_08705 | 1 | -1.125648147 | CDHR3 | cadherin related family member 3 [Source:HGNC Symbol;Acc:HGNC:26308] |
| ENSG00000128596 | HGLibA_07604 | 1 | -1.125648147 | CCDC136 | coiled-coil domain containing 136 [Source:HGNC Symbol;Acc:HGNC:22225] |
| ENSG00000128604 | HGLibA_23593 | 1 | -1.125648147 | IRF5 | interferon regulatory factor 5 [Source:HGNC Symbol;Acc:HGNC:6120] |
| ENSG00000128908 | HGLibA_23318 | 1 | -1.125648147 | INO80 | INO80 complex subunit [Source:HGNC Symbol;Acc:HGNC:26956] |
| ENSG00000128917 | HGLibA_13367 | 1 | -1.125648147 | DLL4 | delta like canonical Notch ligand 4 [Source:HGNC Symbol;Acc:HGNC:2910] |
| ENSG00000128965 | HGLibA_09334 | 1 | -1.125648147 | CHAC1 | ChaC glutathione specific gamma-glutamylcyclotransferase 1 [Source:HGNC Symbol;Acc:HGNC:28680] |

| | | | | | |
|---|---|---|---|---|---|
| ENSG00000129455 | HGLibA_25271 | 1 | -1.125648147 | KLK8 | kallikrein related peptidase 8 [Source:HGNC Symbol;Acc:HGNC:6369] |
| ENSG00000129472 | HGLibA_39867 | 1 | -1.125648147 | RAB2B | member RAS oncogene family [Source:HGNC Symbol;Acc:HGNC:20246]" |
| ENSG00000129484 | HGLibA_35233 | 1 | -1.125648147 | PARP2 | poly(ADP-ribose) polymerase 2 [Source:HGNC Symbol;Acc:HGNC:272] |
| ENSG00000129646 | HGLibA_39745 | 1 | -1.125648147 | QRICH2 | glutamine rich 2 [Source:HGNC Symbol;Acc:HGNC:25326] |
| ENSG00000129680 | HGLibA_28241 | 1 | -1.125648147 | MAP7D3 | MAP7 domain containing 3 [Source:HGNC Symbol;Acc:HGNC:25742] |
| ENSG00000129965 | HGLibA_23369 | 1 | -1.125648147 | INS-IGF2 | INS-IGF2 readthrough [Source:HGNC Symbol;Acc:HGNC:33527] |
| ENSG00000130055 | HGLibA_18892 | 1 | -1.125648147 | GDPD2 | glycerophosphodiester phosphodiesterase domain containing 2 [Source:HGNC Symbol;Acc:HGNC:25974] |
| ENSG00000130147 | HGLibA_43932 | 1 | -1.125648147 | SH3BP4 | SH3 domain binding protein 4 [Source:HGNC Symbol;Acc:HGNC:10826] |
| ENSG00000130193 | HGLibA_49347 | 1 | -1.125648147 | THEM6 | thioesterase superfamily member 6 [Source:HGNC Symbol;Acc:HGNC:29656] |
| ENSG00000130208 | HGLibA_02503 | 1 | -1.125648147 | APOC1 | apolipoprotein C1 [Source:HGNC Symbol;Acc:HGNC:607] |
| ENSG00000130222 | HGLibA_18423 | 1 | -1.125648147 | GADD45G | growth arrest and DNA damage inducible gamma [Source:HGNC Symbol;Acc:HGNC:4097] |
| ENSG00000130413 | HGLibA_47493 | 1 | -1.125648147 | STK33 | serine/threonine kinase 33 [Source:HGNC Symbol;Acc:HGNC:14568] |
| ENSG00000130449 | HGLibA_57052 | 1 | -1.125648147 | ZSWIM6 | zinc finger SWIM-type containing 6 [Source:HGNC Symbol;Acc:HGNC:29316] |
| ENSG00000130528 | HGLibA_22084 | 1 | -1.125648147 | HRC | histidine rich calcium binding protein [Source:HGNC Symbol;Acc:HGNC:5178] |
| ENSG00000130598 | HGLibA_50873 | 1 | -1.125648147 | TNNI2 | fast skeletal type [Source:HGNC Symbol;Acc:HGNC:11946]" |
| ENSG00000130684 | HGLibA_55827 | 1 | -1.125648147 | ZNF337 | zinc finger protein 337 [Source:HGNC Symbol;Acc:HGNC:15809] |
| ENSG00000130768 | HGLibA_45817 | 1 | -1.125648147 | SMPDL3B | sphingomyelin phosphodiesterase acid like 3B [Source:HGNC Symbol;Acc:HGNC:21416] |
| ENSG00000130768 | HGLibA_45818 | 1 | -1.125648147 | SMPDL3B | sphingomyelin phosphodiesterase acid like 3B [Source:HGNC Symbol;Acc:HGNC:21416] |
| ENSG00000130881 | HGLibA_27217 | 1 | -1.125648147 | LRP3 | LDL receptor related protein 3 [Source:HGNC Symbol;Acc:HGNC:6695] |
| ENSG00000131023 | HGLibA_26064 | 1 | -1.125648147 | LATS1 | large tumor suppressor kinase 1 [Source:HGNC Symbol;Acc:HGNC:6514] |
| ENSG00000131043 | HGLibA_00062 | 1 | -1.125648147 | AAR2 | AAR2 splicing factor homolog [Source:HGNC Symbol;Acc:HGNC:15886] |
| ENSG00000131094 | HGLibA_05925 | 1 | -1.125648147 | C1QL1 | complement C1q like 1 [Source:HGNC Symbol;Acc:HGNC:24182] |
| ENSG00000131115 | HGLibA_55628 | 1 | -1.125648147 | ZNF227 | zinc finger protein 227 [Source:HGNC Symbol;Acc:HGNC:13020] |
| ENSG00000131471 | HGLibA_02273 | 1 | -1.125648147 | AOC3 | copper containing 3 [Source:HGNC Symbol;Acc:HGNC:550]" |
| ENSG00000131910 | HGLibA_32572 | 1 | -1.125648147 | NR0B2 | nuclear receptor subfamily 0 group B member 2 [Source:HGNC Symbol;Acc:HGNC:7961] |
| ENSG00000132010 | HGLibA_55567 | 1 | -1.125648147 | ZNF20 | zinc finger protein 20 [Source:HGNC Symbol;Acc:HGNC:12992] |
| ENSG00000132128 | HGLibA_27356 | 1 | -1.125648147 | LRRC41 | leucine rich repeat containing 41 [Source:HGNC Symbol;Acc:HGNC:16917] |
| ENSG00000132141 | HGLibA_08209 | 1 | -1.125648147 | CCT6B | chaperonin containing TCP1 subunit 6B [Source:HGNC Symbol;Acc:HGNC:1621] |
| ENSG00000132313 | HGLibA_29959 | 1 | -1.125648147 | MRPL35 | mitochondrial ribosomal protein L35 [Source:HGNC Symbol;Acc:HGNC:14489] |
| ENSG00000132329 | HGLibA_40179 | 1 | -1.125648147 | RAMP1 | receptor activity modifying protein 1 [Source:HGNC Symbol;Acc:HGNC:9843] |
| ENSG00000132330 | HGLibA_42953 | 1 | -1.125648147 | SCLY | selenocysteine lyase [Source:HGNC Symbol;Acc:HGNC:18161] |
| ENSG00000132423 | HGLibA_10751 | 1 | -1.125648147 | COQ3 | methyltransferase [Source:HGNC Symbol;Acc:HGNC:18175]" |
| ENSG00000132518 | HGLibA_20664 | 1 | -1.125648147 | GUCY2D | retinal [Source:HGNC Symbol;Acc:HGNC:4689]" |
| ENSG00000132554 | HGLibA_41088 | 1 | -1.125648147 | RGS22 | regulator of G protein signaling 22 [Source:HGNC Symbol;Acc:HGNC:24499] |
| ENSG00000132681 | HGLibA_03590 | 1 | -1.125648147 | ATP1A4 | ATPase Na+/K+ transporting subunit alpha 4 [Source:HGNC Symbol;Acc:HGNC:14073] |
| ENSG00000132746 | HGLibA_01570 | 1 | -1.125648147 | ALDH3B2 | aldehyde dehydrogenase 3 family member B2 [Source:HGNC Symbol;Acc:HGNC:411] |
| ENSG00000132792 | HGLibA_11655 | 1 | -1.125648147 | CTNNBL1 | catenin beta like 1 [Source:HGNC Symbol;Acc:HGNC:15879] |
| ENSG00000132793 | HGLibA_27100 | 1 | -1.125648147 | LPIN3 | lipin 3 [Source:HGNC Symbol;Acc:HGNC:14451] |
| ENSG00000132825 | HGLibA_38106 | 1 | -1.125648147 | PPP1R3D | protein phosphatase 1 regulatory subunit 3D [Source:HGNC Symbol;Acc:HGNC:9294] |
| ENSG00000132846 | HGLibA_54795 | 1 | -1.125648147 | ZBED3 | zinc finger BED-type containing 3 [Source:HGNC Symbol;Acc:HGNC:20711] |
| ENSG00000132854 | HGLibA_24033 | 1 | -1.125648147 | KANK4 | KN motif and ankyrin repeat domains 4 [Source:HGNC Symbol;Acc:HGNC:27263] |
| ENSG00000132964 | HGLibA_08797 | 1 | -1.125648147 | CDK8 | cyclin dependent kinase 8 [Source:HGNC Symbol;Acc:HGNC:1779] |
| ENSG00000133142 | HGLibA_48713 | 1 | -1.125648147 | TCEAL4 | transcription elongation factor A like 4 [Source:HGNC Symbol;Acc:HGNC:26121] |
| ENSG00000133246 | HGLibA_38271 | 1 | -1.125648147 | PRAM1 | PML-RARA regulated adaptor molecule 1 [Source:HGNC Symbol;Acc:HGNC:30091] |
| ENSG00000133313 | HGLibA_10255 | 1 | -1.125648147 | CNDP2 | carnosine dipeptidase 2 [Source:HGNC Symbol;Acc:HGNC:24437] |
| ENSG00000133398 | HGLibA_28737 | 1 | -1.125648147 | MED10 | mediator complex subunit 10 [Source:HGNC Symbol;Acc:HGNC:28760] |
| ENSG00000133704 | HGLibA_23475 | 1 | -1.125648147 | IPO8 | importin 8 [Source:HGNC Symbol;Acc:HGNC:9853] |
| ENSG00000133704 | HGLibA_23476 | 1 | -1.125648147 | IPO8 | importin 8 [Source:HGNC Symbol;Acc:HGNC:9853] |
| ENSG00000133789 | HGLibA_47899 | 1 | -1.125648147 | SWAP70 | switching B cell complex subunit SWAP70 [Source:HGNC Symbol;Acc:HGNC:17070] |
| ENSG00000133794 | HGLibA_03068 | 1 | -1.125648147 | ARNTL | aryl hydrocarbon receptor nuclear translocator like [Source:HGNC Symbol;Acc:HGNC:701] |
| ENSG00000134007 | HGLibA_00751 | 1 | -1.125648147 | ADAM20 | ADAM metallopeptidase domain 20 [Source:HGNC Symbol;Acc:HGNC:199] |
| ENSG00000134030 | HGLibA_11631 | 1 | -1.125648147 | CTIF | cap binding complex dependent translation initiation factor [Source:HGNC Symbol;Acc:HGNC:23925] |
| ENSG00000134058 | HGLibA_08793 | 1 | -1.125648147 | CDK7 | cyclin dependent kinase 7 [Source:HGNC Symbol;Acc:HGNC:1778] |
| ENSG00000134058 | HGLibA_08795 | 1 | -1.125648147 | CDK7 | cyclin dependent kinase 7 [Source:HGNC Symbol;Acc:HGNC:1778] |
| ENSG00000134108 | HGLibA_02998 | 1 | -1.125648147 | ARL8B | ADP ribosylation factor like GTPase 8B [Source:HGNC Symbol;Acc:HGNC:25564] |
| ENSG00000134207 | HGLibA_48084 | 1 | -1.125648147 | SYT6 | synaptotagmin 6 [Source:HGNC Symbol;Acc:HGNC:18638] |
| ENSG00000134253 | HGLibA_51511 | 1 | -1.125648147 | TRIM45 | tripartite motif containing 45 [Source:HGNC Symbol;Acc:HGNC:19018] |
| ENSG00000134339 | HGLibA_42610 | 1 | -1.125648147 | SAA2 | serum amyloid A2 [Source:HGNC Symbol;Acc:HGNC:10514] |
| ENSG00000134343 | HGLibA_02176 | 1 | -1.125648147 | ANO3 | anoctamin 3 [Source:HGNC Symbol;Acc:HGNC:14004] |
| ENSG00000134389 | HGLibA_09276 | 1 | -1.125648147 | CFHR5 | complement factor H related 5 [Source:HGNC Symbol;Acc:HGNC:24668] |
| ENSG00000134755 | HGLibA_13993 | 1 | -1.125648147 | DSC2 | desmocollin 2 [Source:HGNC Symbol;Acc:HGNC:3036] |
| ENSG00000134827 | HGLibA_48820 | 1 | -1.125648147 | TCN1 | transcobalamin 1 [Source:HGNC Symbol;Acc:HGNC:11652] |
| ENSG00000134830 | HGLibA_06355 | 1 | -1.125648147 | C5AR2 | complement component 5a receptor 2 [Source:HGNC Symbol;Acc:HGNC:4527] |
| ENSG00000134874 | HGLibA_14299 | 1 | -1.125648147 | DZIP1 | DAZ interacting zinc finger protein 1 [Source:HGNC Symbol;Acc:HGNC:20908] |
| ENSG00000135069 | HGLibA_39007 | 1 | -1.125648147 | PSAT1 | phosphoserine aminotransferase 1 [Source:HGNC Symbol;Acc:HGNC:19129] |
| ENSG00000135185 | HGLibA_50295 | 1 | -1.125648147 | TMEM243 | transmembrane protein 243 [Source:HGNC Symbol;Acc:HGNC:21707] |
| ENSG00000135253 | HGLibA_24409 | 1 | -1.125648147 | KCP | kielin cysteine rich BMP regulator [Source:HGNC Symbol;Acc:HGNC:17585] |
| ENSG00000135378 | HGLibA_38879 | 1 | -1.125648147 | PRRG4 | proline rich and Gla domain 4 [Source:HGNC Symbol;Acc:HGNC:30799] |
| ENSG00000135439 | HGLibA_01141 | 1 | -1.125648147 | AGAP2 | ankyrin repeat and PH domain 2 [Source:HGNC Symbol;Acc:HGNC:16921]" |
| ENSG00000135457 | HGLibA_49145 | 1 | -1.125648147 | TFCP2 | transcription factor CP2 [Source:HGNC Symbol;Acc:HGNC:11748] |
| ENSG00000135625 | HGLibA_14656 | 1 | -1.125648147 | EGR4 | early growth response 4 [Source:HGNC Symbol;Acc:HGNC:3241] |
| ENSG00000135677 | HGLibA_19589 | 1 | -1.125648147 | GNS | glucosamine (N-acetyl)-6-sulfatase [Source:HGNC Symbol;Acc:HGNC:4422] |
| ENSG00000135702 | HGLibA_09640 | 1 | -1.125648147 | CHST5 | carbohydrate sulfotransferase 5 [Source:HGNC Symbol;Acc:HGNC:1973] |
| ENSG00000135776 | HGLibA_00133 | 1 | -1.125648147 | ABCB10 | ATP binding cassette subfamily B member 10 [Source:HGNC Symbol;Acc:HGNC:41] |
| ENSG00000135829 | HGLibA_13211 | 1 | -1.125648147 | DHX9 | DExH-box helicase 9 [Source:HGNC Symbol;Acc:HGNC:2750] |
| ENSG00000135845 | HGLibA_36550 | 1 | -1.125648147 | PIGC | phosphatidylinositol glycan anchor biosynthesis class C [Source:HGNC Symbol;Acc:HGNC:8960] |
| ENSG00000135870 | HGLibA_40661 | 1 | -1.125648147 | RC3H1 | ring finger and CCCH-type domains 1 [Source:HGNC Symbol;Acc:HGNC:29434] |
| ENSG00000135912 | HGLibA_52203 | 1 | -1.125648147 | TTLL4 | tubulin tyrosine ligase like 4 [Source:HGNC Symbol;Acc:HGNC:28976] |
| ENSG00000135917 | HGLibA_44503 | 1 | -1.125648147 | SLC19A3 | solute carrier family 19 member 3 [Source:HGNC Symbol;Acc:HGNC:16266] |
| ENSG00000135929 | HGLibA_12094 | 1 | -1.125648147 | CYP27A1 | cytochrome P450 family 27 subfamily A member 1 [Source:HGNC Symbol;Acc:HGNC:2605] |
| ENSG00000135940 | HGLibA_10839 | 1 | -1.125648147 | COX5B | cytochrome c oxidase subunit 5B [Source:HGNC Symbol;Acc:HGNC:2269] |
| ENSG00000135960 | HGLibA_14435 | 1 | -1.125648147 | EDAR | ectodysplasin A receptor [Source:HGNC Symbol;Acc:HGNC:2895] |
| ENSG00000135999 | HGLibA_15268 | 1 | -1.125648147 | EPC2 | enhancer of polycomb homolog 2 [Source:HGNC Symbol;Acc:HGNC:24543] |
| ENSG00000136444 | HGLibA_42296 | 1 | -1.125648147 | RSAD1 | radical S-adenosyl methionine domain containing 1 [Source:HGNC Symbol;Acc:HGNC:25634] |
| ENSG00000136448 | HGLibA_32205 | 1 | -1.125648147 | NMT1 | N-myristoyltransferase 1 [Source:HGNC Symbol;Acc:HGNC:7857] |
| ENSG00000136682 | HGLibA_07433 | 1 | -1.125648147 | CBWD2 | COBW domain containing 2 [Source:HGNC Symbol;Acc:HGNC:17907] |
| ENSG00000136699 | HGLibA_45813 | 1 | -1.125648147 | SMPD4 | sphingomyelin phosphodiesterase 4 [Source:HGNC Symbol;Acc:HGNC:32949] |
| ENSG00000136718 | HGLibA_23242 | 1 | -1.125648147 | IMP4 | U3 small nucleolar ribonucleoprotein [Source:HGNC Symbol;Acc:HGNC:30856]" |
| ENSG00000136783 | HGLibA_31975 | 1 | -1.125648147 | NIPSNAP3A | nipsnap homolog 3A [Source:HGNC Symbol;Acc:HGNC:23619] |
| ENSG00000136925 | HGLibA_52033 | 1 | -1.125648147 | TSTD2 | thiosulfate sulfurtransferase like domain containing 2 [Source:HGNC Symbol;Acc:HGNC:30087] |
| ENSG00000136982 | HGLibA_14003 | 1 | -1.125648147 | DSCC1 | DNA replication and sister chromatid cohesion 1 [Source:HGNC Symbol;Acc:HGNC:24453] |
| ENSG00000136997 | HGLibA_30663 | 1 | -1.125648147 | MYC | bHLH transcription factor [Source:HGNC Symbol;Acc:HGNC:7553]" |
| ENSG00000137207 | HGLibA_54675 | 1 | -1.125648147 | YIPF3 | Yip1 domain family member 3 [Source:HGNC Symbol;Acc:HGNC:21023] |
| ENSG00000137216 | HGLibA_50478 | 1 | -1.125648147 | TMEM63B | transmembrane protein 63B [Source:HGNC Symbol;Acc:HGNC:17735] |
| ENSG00000137337 | HGLibA_28667 | 1 | -1.125648147 | MDC1 | mediator of DNA damage checkpoint 1 [Source:HGNC Symbol;Acc:HGNC:21163] |
| ENSG00000137404 | HGLibA_32707 | 1 | -1.125648147 | NRM | nurim [Source:HGNC Symbol;Acc:HGNC:8003] |
| ENSG00000137497 | HGLibA_33008 | 1 | -1.125648147 | NUMA1 | nuclear mitotic apparatus protein 1 [Source:HGNC Symbol;Acc:HGNC:8059] |
| ENSG00000137693 | HGLibA_54630 | 1 | -1.125648147 | YAP1 | Yes associated protein 1 [Source:HGNC Symbol;Acc:HGNC:16262] |
| ENSG00000137726 | HGLibA_18238 | 1 | -1.125648147 | FXYD6 | FXYD domain containing ion transport regulator 6 [Source:HGNC Symbol;Acc:HGNC:4030] |
| ENSG00000137776 | HGLibA_45548 | 1 | -1.125648147 | SLTM | SAFB like transcription modulator [Source:HGNC Symbol;Acc:HGNC:20709] |
| ENSG00000137821 | HGLibA_27376 | 1 | -1.125648147 | LRRC49 | leucine rich repeat containing 49 [Source:HGNC Symbol;Acc:HGNC:25965] |
| ENSG00000137843 | HGLibA_35052 | 1 | -1.125648147 | PAK6 | p21 (RAC1) activated kinase 6 [Source:HGNC Symbol;Acc:HGNC:16061] |
| ENSG00000137941 | HGLibA_52212 | 1 | -1.125648147 | TTLL7 | tubulin tyrosine ligase like 7 [Source:HGNC Symbol;Acc:HGNC:26242] |
| ENSG00000137960 | HGLibA_19122 | 1 | -1.125648147 | GIPC2 | GIPC PDZ domain containing family member 2 [Source:HGNC Symbol;Acc:HGNC:18177] |
| ENSG00000138083 | HGLibA_44250 | 1 | -1.125648147 | SIX3 | SIX homeobox 3 [Source:HGNC Symbol;Acc:HGNC:10889] |
| ENSG00000138448 | HGLibA_23764 | 1 | -1.125648147 | ITGAV | integrin subunit alpha V [Source:HGNC Symbol;Acc:HGNC:6150] |
| ENSG00000138629 | HGLibA_52714 | 1 | -1.125648147 | UBL7 | ubiquitin like 7 [Source:HGNC Symbol;Acc:HGNC:28221] |
| ENSG00000138698 | HGLibA_40234 | 1 | -1.125648147 | RAP1GDS1 | Rap1 GTPase-GDP dissociation stimulator 1 [Source:HGNC Symbol;Acc:HGNC:9859] |
| ENSG00000138709 | HGLibA_26030 | 1 | -1.125648147 | LARP1B | La ribonucleoprotein domain family member 1B [Source:HGNC Symbol;Acc:HGNC:24704] |
| ENSG00000138771 | HGLibA_44094 | 1 | -1.125648147 | SHROOM3 | shroom family member 3 [Source:HGNC Symbol;Acc:HGNC:30422] |
| ENSG00000138777 | HGLibA_37806 | 1 | -1.125648147 | PPA2 | pyrophosphatase (inorganic) 2 [Source:HGNC Symbol;Acc:HGNC:28883] |
| ENSG00000139218 | HGLibA_42823 | 1 | -1.125648147 | SCAF11 | SR-related CTD associated factor 11 [Source:HGNC Symbol;Acc:HGNC:10784] |
| ENSG00000139278 | HGLibA_19259 | 1 | -1.125648147 | GLIPR1 | GLI pathogenesis related 1 [Source:HGNC Symbol;Acc:HGNC:17001] |
| ENSG00000139287 | HGLibA_51131 | 1 | -1.125648147 | TPH2 | tryptophan hydroxylase 2 [Source:HGNC Symbol;Acc:HGNC:20692] |
| ENSG00000139291 | HGLibA_50139 | 1 | -1.125648147 | TMEM19 | transmembrane protein 19 [Source:HGNC Symbol;Acc:HGNC:25605] |
| ENSG00000139651 | HGLibA_56661 | 1 | -1.125648147 | ZNF740 | zinc finger protein 740 [Source:HGNC Symbol;Acc:HGNC:27465] |
| ENSG00000139737 | HGLibA_44305 | 1 | -1.125648147 | SLAIN1 | SLAIN motif family member 1 [Source:HGNC Symbol;Acc:HGNC:26387] |
| ENSG00000139780 | HGLibA_28981 | 1 | -1.125648147 | METTL21C | methyltransferase like 21C [Source:HGNC Symbol;Acc:HGNC:33717] |
| ENSG00000139793 | HGLibA_28510 | 1 | -1.125648147 | MBNL2 | muscleblind like splicing regulator 2 [Source:HGNC Symbol;Acc:HGNC:16746] |
| ENSG00000139970 | HGLibA_42399 | 1 | -1.125648147 | RTN1 | reticulon 1 [Source:HGNC Symbol;Acc:HGNC:10467] |
| ENSG00000140006 | HGLibA_54210 | 1 | -1.125648147 | WDR89 | WD repeat domain 89 [Source:HGNC Symbol;Acc:HGNC:20489] |
| ENSG00000140259 | HGLibA_29040 | 1 | -1.125648147 | MFAP1 | microfibril associated protein 1 [Source:HGNC Symbol;Acc:HGNC:7032] |
| ENSG00000140320 | HGLibA_04110 | 1 | -1.125648147 | BAHD1 | bromo adjacent homology domain containing 1 [Source:HGNC Symbol;Acc:HGNC:29153] |
| ENSG00000140451 | HGLibA_36538 | 1 | -1.125648147 | PIF1 | PIF1 5'-to-3' DNA helicase [Source:HGNC Symbol;Acc:HGNC:26220] |
| ENSG00000140478 | HGLibA_19615 | 1 | -1.125648147 | GOLGA6D | golgin A6 family member D [Source:HGNC Symbol;Acc:HGNC:32204] |
| ENSG00000140505 | HGLibA_12070 | 1 | -1.125648147 | CYP1A2 | cytochrome P450 family 1 subfamily A member 2 [Source:HGNC Symbol;Acc:HGNC:2596] |
| ENSG00000140522 | HGLibA_41344 | 1 | -1.125648147 | RLBP1 | retinaldehyde binding protein 1 [Source:HGNC Symbol;Acc:HGNC:10024] |
| ENSG00000140740 | HGLibA_53115 | 1 | -1.125648147 | UQCRC2 | ubiquinol-cytochrome c reductase core protein 2 [Source:HGNC Symbol;Acc:HGNC:12586] |
| ENSG00000141052 | HGLibA_30877 | 1 | -1.125648147 | MYOCD | myocardin [Source:HGNC Symbol;Acc:HGNC:16067] |
| ENSG00000141101 | HGLibA_32231 | 1 | -1.125648147 | NOB1 | NIN1 (RPN12) binding protein 1 homolog [Source:HGNC Symbol;Acc:HGNC:29540] |
| ENSG00000141179 | HGLibA_35691 | 1 | -1.125648147 | PCTP | phosphatidylcholine transfer protein [Source:HGNC Symbol;Acc:HGNC:8752] |
| ENSG00000141200 | HGLibA_24928 | 1 | -1.125648147 | KIF2B | kinesin family member 2B [Source:HGNC Symbol;Acc:HGNC:29443] |
| ENSG00000141314 | HGLibA_41142 | 1 | -1.125648147 | RHBDL3 | rhomboid like 3 [Source:HGNC Symbol;Acc:HGNC:16502] |
| ENSG00000141316 | HGLibA_46327 | 1 | -1.125648147 | SPACA3 | sperm acrosome associated 3 [Source:HGNC Symbol;Acc:HGNC:16260] |
| ENSG00000141505 | HGLibA_03288 | 1 | -1.125648147 | ASGR1 | asialoglycoprotein receptor 1 [Source:HGNC Symbol;Acc:HGNC:742] |

| | | | | | |
|---|---|---|---|---|---|
| ENSG00000141560 | HGLibA_17731 | 1 | -1.125648147 | FN3KRP | fructosamine 3 kinase related protein [Source:HGNC Symbol;Acc:HGNC:25700] |
| ENSG00000141569 | HGLibA_51586 | 1 | -1.125648147 | TRIM65 | tripartite motif containing 65 [Source:HGNC Symbol;Acc:HGNC:27316] |
| ENSG00000141570 | HGLibA_07467 | 1 | -1.125648147 | CBX8 | chromobox 8 [Source:HGNC Symbol;Acc:HGNC:15962] |
| ENSG00000141665 | HGLibA_17030 | 1 | -1.125648147 | FBXO15 | F-box protein 15 [Source:HGNC Symbol;Acc:HGNC:13617] |
| ENSG00000141698 | HGLibA_32817 | 1 | -1.125648147 | NT5C3B | cytosolic IIIB [Source:HGNC Symbol;Acc:HGNC:28300]" |
| ENSG00000141858 | HGLibA_42658 | 1 | -1.125648147 | SAMD1 | sterile alpha motif domain containing 1 [Source:HGNC Symbol;Acc:HGNC:17958] |
| ENSG00000141867 | HGLibA_04779 | 1 | -1.125648147 | BRD4 | bromodomain containing 4 [Source:HGNC Symbol;Acc:HGNC:13575] |
| ENSG00000142166 | HGLibA_22695 | 1 | -1.125648147 | IFNAR1 | interferon alpha and beta receptor subunit 1 [Source:HGNC Symbol;Acc:HGNC:5432] |
| ENSG00000142538 | HGLibA_39370 | 1 | -1.125648147 | PTH2 | parathyroid hormone 2 [Source:HGNC Symbol;Acc:HGNC:30828] |
| ENSG00000142546 | HGLibA_32335 | 1 | -1.125648147 | NOSIP | nitric oxide synthase interacting protein [Source:HGNC Symbol;Acc:HGNC:17946] |
| ENSG00000142789 | HGLibA_08998 | 1 | -1.125648147 | CELA3A | chymotrypsin like elastase family member 3A [Source:HGNC Symbol;Acc:HGNC:15944] |
| ENSG00000142959 | HGLibA_04429 | 1 | -1.125648147 | BEST4 | bestrophin 4 [Source:HGNC Symbol;Acc:HGNC:17106] |
| ENSG00000143147 | HGLibA_19957 | 1 | -1.125648147 | GPR161 | G protein-coupled receptor 161 [Source:HGNC Symbol;Acc:HGNC:23694] |
| ENSG00000143196 | HGLibA_13902 | 1 | -1.125648147 | DPT | dermatopontin [Source:HGNC Symbol;Acc:HGNC:3011] |
| ENSG00000143252 | HGLibA_43146 | 1 | -1.125648147 | SDHC | succinate dehydrogenase complex subunit C [Source:HGNC Symbol;Acc:HGNC:10682] |
| ENSG00000143314 | HGLibA_29935 | 1 | -1.125648147 | MRPL24 | mitochondrial ribosomal protein L24 [Source:HGNC Symbol;Acc:HGNC:14037] |
| ENSG00000143355 | HGLibA_26467 | 1 | -1.125648147 | LHX9 | LIM homeobox 9 [Source:HGNC Symbol;Acc:HGNC:14222] |
| ENSG00000143416 | HGLibA_43289 | 1 | -1.125648147 | SELENBP1 | selenium binding protein 1 [Source:HGNC Symbol;Acc:HGNC:10719] |
| ENSG00000143479 | HGLibA_14281 | 1 | -1.125648147 | DYRK3 | dual specificity tyrosine phosphorylation regulated kinase 3 [Source:HGNC Symbol;Acc:HGNC:3094] |
| ENSG00000143603 | HGLibA_24360 | 1 | -1.125648147 | KCNN3 | potassium calcium-activated channel subfamily N member 3 [Source:HGNC Symbol;Acc:HGNC:6292] |
| ENSG00000143622 | HGLibA_41338 | 1 | -1.125648147 | RIT1 | Ras like without CAAX 1 [Source:HGNC Symbol;Acc:HGNC:10023] |
| ENSG00000143776 | HGLibA_08556 | 1 | -1.125648147 | CDC42BPA | CDC42 binding protein kinase alpha [Source:HGNC Symbol;Acc:HGNC:1737] |
| ENSG00000143839 | HGLibA_40837 | 1 | -1.125648147 | REN | renin [Source:HGNC Symbol;Acc:HGNC:9958] |
| ENSG00000144191 | HGLibA_10270 | 1 | -1.125648147 | CNGA3 | cyclic nucleotide gated channel alpha 3 [Source:HGNC Symbol;Acc:HGNC:2150] |
| ENSG00000144366 | HGLibA_20676 | 1 | -1.125648147 | GULP1 | GULP PTB domain containing engulfment adaptor 1 [Source:HGNC Symbol;Acc:HGNC:18649] |
| ENSG00000144566 | HGLibA_39961 | 1 | -1.125648147 | RAB5A | member RAS oncogene family [Source:HGNC Symbol;Acc:HGNC:9783]" |
| ENSG00000144644 | HGLibA_18428 | 1 | -1.125648147 | GADL1 | glutamate decarboxylase like 1 [Source:HGNC Symbol;Acc:HGNC:27949] |
| ENSG00000144677 | HGLibA_11615 | 1 | -1.125648147 | CTDSPL | CTD small phosphatase like [Source:HGNC Symbol;Acc:HGNC:16890] |
| ENSG00000144747 | HGLibA_50580 | 1 | -1.125648147 | TMF1 | TATA element modulatory factor 1 [Source:HGNC Symbol;Acc:HGNC:11870] |
| ENSG00000144821 | HGLibA_30717 | 1 | -1.125648147 | MYH15 | myosin heavy chain 15 [Source:HGNC Symbol;Acc:HGNC:31073] |
| ENSG00000144840 | HGLibA_40032 | 1 | -1.125648147 | RABL3 | member of RAS oncogene family like 3 [Source:HGNC Symbol;Acc:HGNC:18072]" |
| ENSG00000145198 | HGLibA_53911 | 1 | -1.125648147 | VWA5B2 | von Willebrand factor A domain containing 5B2 [Source:HGNC Symbol;Acc:HGNC:25144] |
| ENSG00000145248 | HGLibA_44340 | 1 | -1.125648147 | SLC10A4 | solute carrier family 10 member 4 [Source:HGNC Symbol;Acc:HGNC:22980] |
| ENSG00000145349 | HGLibA_07074 | 1 | -1.125648147 | CAMK2D | calcium/calmodulin dependent protein kinase II delta [Source:HGNC Symbol;Acc:HGNC:1462] |
| ENSG00000145358 | HGLibA_12622 | 1 | -1.125648147 | DDIT4L | DNA damage inducible transcript 4 like [Source:HGNC Symbol;Acc:HGNC:30555] |
| ENSG00000145388 | HGLibA_28959 | 1 | -1.125648147 | METTL14 | methyltransferase like 14 [Source:HGNC Symbol;Acc:HGNC:29330] |
| ENSG00000145451 | HGLibA_19304 | 1 | -1.125648147 | GLRA3 | glycine receptor alpha 3 [Source:HGNC Symbol;Acc:HGNC:4328] |
| ENSG00000145675 | HGLibA_36648 | 1 | -1.125648147 | PIK3R1 | phosphoinositide-3-kinase regulatory subunit 1 [Source:HGNC Symbol;Acc:HGNC:8979] |
| ENSG00000145723 | HGLibA_19096 | 1 | -1.125648147 | GIN1 | gypsy retrotransposon integrase 1 [Source:HGNC Symbol;Acc:HGNC:25959] |
| ENSG00000145863 | HGLibA_18372 | 1 | -1.125648147 | GABRA6 | gamma-aminobutyric acid type A receptor alpha6 subunit [Source:HGNC Symbol;Acc:HGNC:4080] |
| ENSG00000145979 | HGLibA_48564 | 1 | -1.125648147 | TBC1D7 | TBC1 domain family member 7 [Source:HGNC Symbol;Acc:HGNC:21066] |
| ENSG00000146070 | HGLibA_36925 | 1 | -1.125648147 | PLA2G7 | phospholipase A2 group VII [Source:HGNC Symbol;Acc:HGNC:9040] |
| ENSG00000146242 | HGLibA_51098 | 1 | -1.125648147 | TPBG | trophoblast glycoprotein [Source:HGNC Symbol;Acc:HGNC:12004] |
| ENSG00000146276 | HGLibA_18403 | 1 | -1.125648147 | GABRR1 | gamma-aminobutyric acid type A receptor rho1 subunit [Source:HGNC Symbol;Acc:HGNC:4090] |
| ENSG00000146378 | HGLibA_48125 | 1 | -1.125648147 | TAAR2 | trace amine associated receptor 2 (gene/pseudogene) [Source:HGNC Symbol;Acc:HGNC:4514] |
| ENSG00000146457 | HGLibA_54431 | 1 | -1.125648147 | WTAP | WT1 associated protein [Source:HGNC Symbol;Acc:HGNC:16846] |
| ENSG00000146463 | HGLibA_55398 | 1 | -1.125648147 | ZMYM4 | zinc finger MYM-type containing 4 [Source:HGNC Symbol;Acc:HGNC:13055] |
| ENSG00000146872 | HGLibA_49625 | 1 | -1.125648147 | TLK2 | tousled like kinase 2 [Source:HGNC Symbol;Acc:HGNC:11842] |
| ENSG00000147059 | HGLibA_46669 | 1 | -1.125648147 | SPIN2A | spindlin family member 2A [Source:HGNC Symbol;Acc:HGNC:20694] |
| ENSG00000147257 | HGLibA_19769 | 1 | -1.125648147 | GPC3 | glypican 3 [Source:HGNC Symbol;Acc:HGNC:4451] |
| ENSG00000147400 | HGLibA_09236 | 1 | -1.125648147 | CETN2 | centrin 2 [Source:HGNC Symbol;Acc:HGNC:1867] |
| ENSG00000147416 | HGLibA_03736 | 1 | -1.125648147 | ATP6V1B2 | ATPase H+ transporting V1 subunit B2 [Source:HGNC Symbol;Acc:HGNC:854] |
| ENSG00000147432 | HGLibA_09596 | 1 | -1.125648147 | CHRNB3 | cholinergic receptor nicotinic beta 3 subunit [Source:HGNC Symbol;Acc:HGNC:1963] |
| ENSG00000147509 | HGLibA_41082 | 1 | -1.125648147 | RGS20 | regulator of G protein signaling 20 [Source:HGNC Symbol;Acc:HGNC:14600] |
| ENSG00000147613 | HGLibA_39066 | 1 | -1.125648147 | PSKH2 | protein serine kinase H2 [Source:HGNC Symbol;Acc:HGNC:18997] |
| ENSG00000147655 | HGLibA_42348 | 1 | -1.125648147 | RSPO2 | R-spondin 2 [Source:HGNC Symbol;Acc:HGNC:28583] |
| ENSG00000147912 | HGLibA_17025 | 1 | -1.125648147 | FBXO10 | F-box protein 10 [Source:HGNC Symbol;Acc:HGNC:13589] |
| ENSG00000148300 | HGLibA_40898 | 1 | -1.125648147 | REXO4 | 3'-5' exonuclease [Source:HGNC Symbol;Acc:HGNC:12820]" |
| ENSG00000148334 | HGLibA_39328 | 1 | -1.125648147 | PTGES2 | prostaglandin E synthase 2 [Source:HGNC Symbol;Acc:HGNC:17822] |
| ENSG00000148344 | HGLibA_39325 | 1 | -1.125648147 | PTGES | prostaglandin E synthase [Source:HGNC Symbol;Acc:HGNC:9599] |
| ENSG00000148358 | HGLibA_19842 | 1 | -1.125648147 | GPR107 | G protein-coupled receptor 107 [Source:HGNC Symbol;Acc:HGNC:17830] |
| ENSG00000148386 | HGLibA_26186 | 1 | -1.125648147 | LCN9 | lipocalin 9 [Source:HGNC Symbol;Acc:HGNC:17442] |
| ENSG00000148411 | HGLibA_31043 | 1 | -1.125648147 | NACC2 | NACC family member 2 [Source:HGNC Symbol;Acc:HGNC:23846] |
| ENSG00000148702 | HGLibA_20792 | 1 | -1.125648147 | HABP2 | hyaluronan binding protein 2 [Source:HGNC Symbol;Acc:HGNC:4798] |
| ENSG00000149021 | HGLibA_42907 | 1 | -1.125648147 | SCGB1A1 | secretoglobin family 1A member 1 [Source:HGNC Symbol;Acc:HGNC:12523] |
| ENSG00000149187 | HGLibA_09003 | 1 | -1.125648147 | CELF1 | CUGBP Elav-like family member 1 [Source:HGNC Symbol;Acc:HGNC:2549] |
| ENSG00000149260 | HGLibA_07169 | 1 | -1.125648147 | CAPN5 | calpain 5 [Source:HGNC Symbol;Acc:HGNC:1482] |
| ENSG00000149294 | HGLibA_31253 | 1 | -1.125648147 | NCAM1 | neural cell adhesion molecule 1 [Source:HGNC Symbol;Acc:HGNC:7656] |
| ENSG00000149305 | HGLibA_22383 | 1 | -1.125648147 | HTR3B | 5-hydroxytryptamine receptor 3B [Source:HGNC Symbol;Acc:HGNC:5298] |
| ENSG00000149308 | HGLibA_32407 | 1 | -1.125648147 | NPAT | coactivator of histone transcription [Source:HGNC Symbol;Acc:HGNC:7896]" |
| ENSG00000149418 | HGLibA_47225 | 1 | -1.125648147 | ST14 | suppression of tumorigenicity 14 [Source:HGNC Symbol;Acc:HGNC:11344] |
| ENSG00000149547 | HGLibA_14691 | 1 | -1.125648147 | EI24 | autophagy associated transmembrane protein [Source:HGNC Symbol;Acc:HGNC:13276]" |
| ENSG00000149548 | HGLibA_07639 | 1 | -1.125648147 | CCDC15 | coiled-coil domain containing 15 [Source:HGNC Symbol;Acc:HGNC:25798] |
| ENSG00000149564 | HGLibA_15554 | 1 | -1.125648147 | ESAM | endothelial cell adhesion molecule [Source:HGNC Symbol;Acc:HGNC:17474] |
| ENSG00000149823 | HGLibA_53802 | 1 | -1.125648147 | VPS51 | GARP complex subunit [Source:HGNC Symbol;Acc:HGNC:1172]" |
| ENSG00000150275 | HGLibA_35400 | 1 | -1.125648147 | PCDH15 | protocadherin related 15 [Source:HGNC Symbol;Acc:HGNC:14674] |
| ENSG00000150990 | HGLibA_13194 | 1 | -1.125648147 | DHX37 | DEAH-box helicase 37 [Source:HGNC Symbol;Acc:HGNC:17210] |
| ENSG00000151090 | HGLibA_49394 | 1 | -1.125648147 | THRB | thyroid hormone receptor beta [Source:HGNC Symbol;Acc:HGNC:11799] |
| ENSG00000151322 | HGLibA_32400 | 1 | -1.125648147 | NPAS3 | neuronal PAS domain protein 3 [Source:HGNC Symbol;Acc:HGNC:19311] |
| ENSG00000151365 | HGLibA_49397 | 1 | -1.125648147 | THRSP | thyroid hormone responsive [Source:HGNC Symbol;Acc:HGNC:11800] |
| ENSG00000151365 | HGLibA_49398 | 1 | -1.125648147 | THRSP | thyroid hormone responsive [Source:HGNC Symbol;Acc:HGNC:11800] |
| ENSG00000151474 | HGLibA_18013 | 1 | -1.125648147 | FRMD4A | FERM domain containing 4A [Source:HGNC Symbol;Acc:HGNC:25491] |
| ENSG00000151612 | HGLibA_56823 | 1 | -1.125648147 | ZNF827 | zinc finger protein 827 [Source:HGNC Symbol;Acc:HGNC:27193] |
| ENSG00000151650 | HGLibA_53621 | 1 | -1.125648147 | VENTX | VENT homeobox [Source:HGNC Symbol;Acc:HGNC:13639] |
| ENSG00000151729 | HGLibA_44733 | 1 | -1.125648147 | SLC25A4 | solute carrier family 25 member 4 [Source:HGNC Symbol;Acc:HGNC:10990] |
| ENSG00000151748 | HGLibA_42786 | 1 | -1.125648147 | SAV1 | salvador family WW domain containing protein 1 [Source:HGNC Symbol;Acc:HGNC:17795] |
| ENSG00000151838 | HGLibA_07707 | 1 | -1.125648147 | CCDC175 | coiled-coil domain containing 175 [Source:HGNC Symbol;Acc:HGNC:19847] |
| ENSG00000151876 | HGLibA_17089 | 1 | -1.125648147 | FBXO4 | F-box protein 4 [Source:HGNC Symbol;Acc:HGNC:13583] |
| ENSG00000151883 | HGLibA_35244 | 1 | -1.125648147 | PARP8 | poly(ADP-ribose) polymerase family member 8 [Source:HGNC Symbol;Acc:HGNC:26124] |
| ENSG00000152192 | HGLibA_37778 | 1 | -1.125648147 | POU4F1 | POU class 4 homeobox 1 [Source:HGNC Symbol;Acc:HGNC:9218] |
| ENSG00000152207 | HGLibA_12229 | 1 | -1.125648147 | CYSLTR2 | cysteinyl leukotriene receptor 2 [Source:HGNC Symbol;Acc:HGNC:18274] |
| ENSG00000152402 | HGLibA_20651 | 1 | -1.125648147 | GUCY1A2 | guanylate cyclase 1 soluble subunit alpha 2 [Source:HGNC Symbol;Acc:HGNC:4684] |
| ENSG00000152443 | HGLibA_56723 | 1 | -1.125648147 | ZNF776 | zinc finger protein 776 [Source:HGNC Symbol;Acc:HGNC:26765] |
| ENSG00000152749 | HGLibA_19983 | 1 | -1.125648147 | GPR180 | G protein-coupled receptor 180 [Source:HGNC Symbol;Acc:HGNC:28899] |
| ENSG00000153107 | HGLibA_01861 | 1 | -1.125648147 | ANAPC1 | anaphase promoting complex subunit 1 [Source:HGNC Symbol;Acc:HGNC:19988] |
| ENSG00000153162 | HGLibA_04613 | 1 | -1.125648147 | BMP6 | bone morphogenetic protein 6 [Source:HGNC Symbol;Acc:HGNC:1073] |
| ENSG00000153253 | HGLibA_42985 | 1 | -1.125648147 | SCN3A | sodium voltage-gated channel alpha subunit 3 [Source:HGNC Symbol;Acc:HGNC:10590] |
| ENSG00000153896 | HGLibA_56358 | 1 | -1.125648147 | ZNF599 | zinc finger protein 599 [Source:HGNC Symbol;Acc:HGNC:26408] |
| ENSG00000153956 | HGLibA_06906 | 1 | -1.125648147 | CACNA2D1 | calcium voltage-gated channel auxiliary subunit alpha2delta 1 [Source:HGNC Symbol;Acc:HGNC:1399] |
| ENSG00000154065 | HGLibA_02057 | 1 | -1.125648147 | ANKRD29 | ankyrin repeat domain 29 [Source:HGNC Symbol;Acc:HGNC:27110] |
| ENSG00000154080 | HGLibA_09652 | 1 | -1.125648147 | CHST9 | carbohydrate sulfotransferase 9 [Source:HGNC Symbol;Acc:HGNC:19898] |
| ENSG00000154309 | HGLibA_13283 | 1 | -1.125648147 | DISP1 | dispatched RND transporter family member 1 [Source:HGNC Symbol;Acc:HGNC:19711] |
| ENSG00000154342 | HGLibA_54366 | 1 | -1.125648147 | WNT3A | Wnt family member 3A [Source:HGNC Symbol;Acc:HGNC:15983] |
| ENSG00000154359 | HGLibA_27021 | 1 | -1.125648147 | LONRF1 | LON peptidase N-terminal domain and ring finger 1 [Source:HGNC Symbol;Acc:HGNC:26302] |
| ENSG00000154655 | HGLibA_25905 | 1 | -1.125648147 | L3MBTL4 | histone methyl-lysine binding protein [Source:HGNC Symbol;Acc:HGNC:26677]" |
| ENSG00000154920 | HGLibA_15036 | 1 | -1.125648147 | EME1 | essential meiotic structure-specific endonuclease 1 [Source:HGNC Symbol;Acc:HGNC:24965] |
| ENSG00000155090 | HGLibA_25043 | 1 | -1.125648147 | KLF10 | Kruppel like factor 10 [Source:HGNC Symbol;Acc:HGNC:11810] |
| ENSG00000155093 | HGLibA_39523 | 1 | -1.125648147 | PTPRN2 | receptor type N2 [Source:HGNC Symbol;Acc:HGNC:9677]" |
| ENSG00000155249 | HGLibA_34017 | 1 | -1.125648147 | OR4K1 | olfactory receptor family 4 subfamily K member 1 [Source:HGNC Symbol;Acc:HGNC:14726] |
| ENSG00000155868 | HGLibA_28813 | 1 | -1.125648147 | MED7 | mediator complex subunit 7 [Source:HGNC Symbol;Acc:HGNC:2378] |
| ENSG00000155926 | HGLibA_44301 | 1 | -1.125648147 | SLA | Src like adaptor [Source:HGNC Symbol;Acc:HGNC:10902] |
| ENSG00000156011 | HGLibA_39019 | 1 | -1.125648147 | PSD3 | pleckstrin and Sec7 domain containing 3 [Source:HGNC Symbol;Acc:HGNC:19093] |
| ENSG00000156463 | HGLibA_43972 | 1 | -1.125648147 | SH3RF2 | SH3 domain containing ring finger 2 [Source:HGNC Symbol;Acc:HGNC:26299] |
| ENSG00000156603 | HGLibA_28768 | 1 | -1.125648147 | MED19 | mediator complex subunit 19 [Source:HGNC Symbol;Acc:HGNC:29600] |
| ENSG00000156671 | HGLibA_42694 | 1 | -1.125648147 | SAMD8 | sterile alpha motif domain containing 8 [Source:HGNC Symbol;Acc:HGNC:26320] |
| ENSG00000156711 | HGLibA_28258 | 1 | -1.125648147 | MAPK13 | mitogen-activated protein kinase 13 [Source:HGNC Symbol;Acc:HGNC:6875] |
| ENSG00000156738 | HGLibA_30127 | 1 | -1.125648147 | MS4A1 | membrane spanning 4-domains A1 [Source:HGNC Symbol;Acc:HGNC:7315] |
| ENSG00000156966 | HGLibA_04000 | 1 | -1.125648147 | B3GNT7 | 3-N-acetylglucosaminyltransferase 7 [Source:HGNC Symbol;Acc:HGNC:18811]" |
| ENSG00000156976 | HGLibA_14806 | 1 | -1.125648147 | EIF4A2 | eukaryotic translation initiation factor 4A2 [Source:HGNC Symbol;Acc:HGNC:3284] |
| ENSG00000157060 | HGLibA_44016 | 1 | -1.125648147 | SHCBP1L | SHC binding and spindle associated 1 like [Source:HGNC Symbol;Acc:HGNC:16788] |
| ENSG00000157064 | HGLibA_32186 | 1 | -1.125648147 | NMNAT2 | nicotinamide nucleotide adenylyltransferase 2 [Source:HGNC Symbol;Acc:HGNC:16789] |
| ENSG00000157240 | HGLibA_18260 | 1 | -1.125648147 | FZD1 | frizzled class receptor 1 [Source:HGNC Symbol;Acc:HGNC:4038] |
| ENSG00000157303 | HGLibA_47851 | 1 | -1.125648147 | SUSD3 | sushi domain containing 3 [Source:HGNC Symbol;Acc:HGNC:28391] |
| ENSG00000157322 | HGLibA_09942 | 1 | -1.125648147 | CLEC18A | C-type lectin domain family 18 member A [Source:HGNC Symbol;Acc:HGNC:30388] |
| ENSG00000157423 | HGLibA_22449 | 1 | -1.125648147 | HYDIN | axonemal central pair apparatus protein [Source:HGNC Symbol;Acc:HGNC:19368]" |
| ENSG00000157600 | HGLibA_50032 | 1 | -1.125648147 | TMEM164 | transmembrane protein 164 [Source:HGNC Symbol;Acc:HGNC:26217] |
| ENSG00000157916 | HGLibA_40853 | 1 | -1.125648147 | RER1 | retention in endoplasmic reticulum sorting receptor 1 [Source:HGNC Symbol;Acc:HGNC:30309] |
| ENSG00000158553 | HGLibA_37650 | 1 | -1.125648147 | POM121L2 | POM121 transmembrane nucleoporin like 2 [Source:HGNC Symbol;Acc:HGNC:13973] |
| ENSG00000159110 | HGLibA_22697 | 1 | -1.125648147 | IFNAR2 | interferon alpha and beta receptor subunit 2 [Source:HGNC Symbol;Acc:HGNC:5433] |
| ENSG00000159445 | HGLibA_49340 | 1 | -1.125648147 | THEM4 | thioesterase superfamily member 4 [Source:HGNC Symbol;Acc:HGNC:17947] |
| ENSG00000159723 | HGLibA_01252 | 1 | -1.125648147 | AGRP | agouti related neuropeptide [Source:HGNC Symbol;Acc:HGNC:330] |
| ENSG00000159733 | HGLibA_55290 | 1 | -1.125648147 | ZFYVE28 | zinc finger FYVE-type containing 28 [Source:HGNC Symbol;Acc:HGNC:29334] |
| ENSG00000159761 | HGLibA_05490 | 1 | -1.125648147 | C16orf86 | chromosome 16 open reading frame 86 [Source:HGNC Symbol;Acc:HGNC:33755] |
| ENSG00000159871 | HGLibA_27724 | 1 | -1.125648147 | LYPD5 | LY6/PLAUR domain containing 5 [Source:HGNC Symbol;Acc:HGNC:26397] |
| ENSG00000160113 | HGLibA_32617 | 1 | -1.125648147 | NR2F6 | nuclear receptor subfamily 2 group F member 6 [Source:HGNC Symbol;Acc:HGNC:7977] |

| | | | | | |
|---|---|---|---|---|---|
| ENSG00000160180 | HGLibA_49176 | 1 | -1.125648147 | TFF3 | trefoil factor 3 [Source:HGNC Symbol;Acc:HGNC:11757] |
| ENSG00000160181 | HGLibA_49173 | 1 | -1.125648147 | TFF2 | trefoil factor 2 [Source:HGNC Symbol;Acc:HGNC:11756] |
| ENSG00000160200 | HGLibA_07428 | 1 | -1.125648147 | CBS | cystathionine-beta-synthase [Source:HGNC Symbol;Acc:HGNC:1550] |
| ENSG00000160202 | HGLibA_11247 | 1 | -1.125648147 | CRYAA | crystallin alpha A [Source:HGNC Symbol;Acc:HGNC:2388] |
| ENSG00000160223 | HGLibA_22518 | 1 | -1.125648147 | ICOSLG | inducible T cell costimulator ligand [Source:HGNC Symbol;Acc:HGNC:17087] |
| ENSG00000160360 | HGLibA_20156 | 1 | -1.125648147 | GPSM1 | G protein signaling modulator 1 [Source:HGNC Symbol;Acc:HGNC:17858] |
| ENSG00000160404 | HGLibA_51026 | 1 | -1.125648147 | TOR2A | torsin family 2 member A [Source:HGNC Symbol;Acc:HGNC:11996] |
| ENSG00000160741 | HGLibA_11230 | 1 | -1.125648147 | CRTC2 | CREB regulated transcription coactivator 2 [Source:HGNC Symbol;Acc:HGNC:27301] |
| ENSG00000160877 | HGLibA_31040 | 1 | -1.125648147 | NACC1 | nucleus accumbens associated 1 [Source:HGNC Symbol;Acc:HGNC:20967] |
| ENSG00000161031 | HGLibA_36253 | 1 | -1.125648147 | PGLYRP2 | peptidoglycan recognition protein 2 [Source:HGNC Symbol;Acc:HGNC:30013] |
| ENSG00000161249 | HGLibA_13409 | 1 | -1.125648147 | DMKN | dermokine [Source:HGNC Symbol;Acc:HGNC:25063] |
| ENSG00000161395 | HGLibA_36214 | 1 | -1.125648147 | PGAP3 | post-GPI attachment to proteins 3 [Source:HGNC Symbol;Acc:HGNC:23719] |
| ENSG00000161547 | HGLibA_47071 | 1 | -1.125648147 | SRSF2 | serine and arginine rich splicing factor 2 [Source:HGNC Symbol;Acc:HGNC:10783] |
| ENSG00000161640 | HGLibA_44128 | 1 | -1.125648147 | SIGLEC11 | sialic acid binding Ig like lectin 11 [Source:HGNC Symbol;Acc:HGNC:15622] |
| ENSG00000161905 | HGLibA_01683 | 1 | -1.125648147 | ALOX15 | arachidonate 15-lipoxygenase [Source:HGNC Symbol;Acc:HGNC:433] |
| ENSG00000161958 | HGLibA_17366 | 1 | -1.125648147 | FGF11 | fibroblast growth factor 11 [Source:HGNC Symbol;Acc:HGNC:3667] |
| ENSG00000161973 | HGLibA_07782 | 1 | -1.125648147 | CCDC42 | coiled-coil domain containing 42 [Source:HGNC Symbol;Acc:HGNC:26528] |
| ENSG00000162148 | HGLibA_38085 | 1 | -1.125648147 | PPP1R32 | protein phosphatase 1 regulatory subunit 32 [Source:HGNC Symbol;Acc:HGNC:28869] |
| ENSG00000162302 | HGLibA_42166 | 1 | -1.125648147 | RPS6KA4 | ribosomal protein S6 kinase A4 [Source:HGNC Symbol;Acc:HGNC:10433] |
| ENSG00000162373 | HGLibA_04410 | 1 | -1.125648147 | BEND5 | BEN domain containing 5 [Source:HGNC Symbol;Acc:HGNC:25668] |
| ENSG00000162409 | HGLibA_38495 | 1 | -1.125648147 | PRKAA2 | protein kinase AMP-activated catalytic subunit alpha 2 [Source:HGNC Symbol;Acc:HGNC:9377] |
| ENSG00000162413 | HGLibA_25154 | 1 | -1.125648147 | KLHL21 | kelch like family member 21 [Source:HGNC Symbol;Acc:HGNC:29041] |
| ENSG00000162444 | HGLibA_40643 | 1 | -1.125648147 | RBP7 | retinol binding protein 7 [Source:HGNC Symbol;Acc:HGNC:30316] |
| ENSG00000162512 | HGLibA_43104 | 1 | -1.125648147 | SDC3 | syndecan 3 [Source:HGNC Symbol;Acc:HGNC:10660] |
| ENSG00000162616 | HGLibA_13542 | 1 | -1.125648147 | DNAJB4 | DnaJ heat shock protein family (Hsp40) member B4 [Source:HGNC Symbol;Acc:HGNC:14886] |
| ENSG00000162630 | HGLibA_03956 | 1 | -1.125648147 | B3GALT2 | 3-galactosyltransferase 2 [Source:HGNC Symbol;Acc:HGNC:917]" |
| ENSG00000162645 | HGLibA_18731 | 1 | -1.125648147 | GBP2 | guanylate binding protein 2 [Source:HGNC Symbol;Acc:HGNC:4183] |
| ENSG00000162695 | HGLibA_44916 | 1 | -1.125648147 | SLC30A7 | solute carrier family 30 member 7 [Source:HGNC Symbol;Acc:HGNC:19306] |
| ENSG00000162728 | HGLibA_24291 | 1 | -1.125648147 | KCNJ9 | potassium voltage-gated channel subfamily J member 9 [Source:HGNC Symbol;Acc:HGNC:6270] |
| ENSG00000162817 | HGLibA_05752 | 1 | -1.125648147 | C1orf115 | chromosome 1 open reading frame 115 [Source:HGNC Symbol;Acc:HGNC:25873] |
| ENSG00000162869 | HGLibA_38077 | 1 | -1.125648147 | PPP1R21 | protein phosphatase 1 regulatory subunit 21 [Source:HGNC Symbol;Acc:HGNC:30595] |
| ENSG00000162878 | HGLibA_36808 | 1 | -1.125648147 | PKDCC | cytoplasmic [Source:HGNC Symbol;Acc:HGNC:25123]" |
| ENSG00000162951 | HGLibA_27486 | 1 | -1.125648147 | LRRTM1 | leucine rich repeat transmembrane neuronal 1 [Source:HGNC Symbol;Acc:HGNC:19408] |
| ENSG00000162980 | HGLibA_02976 | 1 | -1.125648147 | ARL5A | ADP ribosylation factor like GTPase 5A [Source:HGNC Symbol;Acc:HGNC:696] |
| ENSG00000163053 | HGLibA_44431 | 1 | -1.125648147 | SLC16A14 | solute carrier family 16 member 14 [Source:HGNC Symbol;Acc:HGNC:26417] |
| ENSG00000163072 | HGLibA_32336 | 1 | -1.125648147 | NOSTRIN | nitric oxide synthase trafficking [Source:HGNC Symbol;Acc:HGNC:20203] |
| ENSG00000163293 | HGLibA_31956 | 1 | -1.125648147 | NIPAL1 | NIPA like domain containing 1 [Source:HGNC Symbol;Acc:HGNC:27194] |
| ENSG00000163328 | HGLibA_19941 | 1 | -1.125648147 | GPR155 | G protein-coupled receptor 155 [Source:HGNC Symbol;Acc:HGNC:22951] |
| ENSG00000163359 | HGLibA_10602 | 1 | -1.125648147 | COL6A3 | collagen type VI alpha 3 chain [Source:HGNC Symbol;Acc:HGNC:2213] |
| ENSG00000163395 | HGLibA_22855 | 1 | -1.125648147 | IGFN1 | immunoglobulin-like and fibronectin type III domain containing 1 [Source:HGNC Symbol;Acc:HGNC:24607] |
| ENSG00000163449 | HGLibA_50047 | 1 | -1.125648147 | TMEM169 | transmembrane protein 169 [Source:HGNC Symbol;Acc:HGNC:25130] |
| ENSG00000163508 | HGLibA_15226 | 1 | -1.125648147 | EOMES | eomesodermin [Source:HGNC Symbol;Acc:HGNC:3372] |
| ENSG00000163539 | HGLibA_09796 | 1 | -1.125648147 | CLASP2 | cytoplasmic linker associated protein 2 [Source:HGNC Symbol;Acc:HGNC:17078] |
| ENSG00000163625 | HGLibA_54011 | 1 | -1.125648147 | WDFY3 | WD repeat and FYVE domain containing 3 [Source:HGNC Symbol;Acc:HGNC:20751] |
| ENSG00000163661 | HGLibA_39570 | 1 | -1.125648147 | PTX3 | pentraxin 3 [Source:HGNC Symbol;Acc:HGNC:9692] |
| ENSG00000163666 | HGLibA_21199 | 1 | -1.125648147 | HESX1 | HESX homeobox 1 [Source:HGNC Symbol;Acc:HGNC:4877] |
| ENSG00000163781 | HGLibA_51009 | 1 | -1.125648147 | TOPBP1 | DNA topoisomerase II binding protein 1 [Source:HGNC Symbol;Acc:HGNC:17008] |
| ENSG00000163803 | HGLibA_36969 | 1 | -1.125648147 | PLB1 | phospholipase B1 [Source:HGNC Symbol;Acc:HGNC:30041] |
| ENSG00000163840 | HGLibA_14089 | 1 | -1.125648147 | DTX3L | deltex E3 ubiquitin ligase 3L [Source:HGNC Symbol;Acc:HGNC:30323] |
| ENSG00000163874 | HGLibA_54951 | 1 | -1.125648147 | ZC3H12A | zinc finger CCCH-type containing 12A [Source:HGNC Symbol;Acc:HGNC:26259] |
| ENSG00000163898 | HGLibA_26616 | 1 | -1.125648147 | LIPH | lipase H [Source:HGNC Symbol;Acc:HGNC:18483] |
| ENSG00000163961 | HGLibA_41566 | 1 | -1.125648147 | RNF168 | ring finger protein 168 [Source:HGNC Symbol;Acc:HGNC:26661] |
| ENSG00000164023 | HGLibA_43837 | 1 | -1.125648147 | SGMS2 | sphingomyelin synthase 2 [Source:HGNC Symbol;Acc:HGNC:28395] |
| ENSG00000164024 | HGLibA_28928 | 1 | -1.125648147 | METAP1 | methionyl aminopeptidase 1 [Source:HGNC Symbol;Acc:HGNC:15789] |
| ENSG00000164031 | HGLibA_13533 | 1 | -1.125648147 | DNAJB14 | DnaJ heat shock protein family (Hsp40) member B14 [Source:HGNC Symbol;Acc:HGNC:25881] |
| ENSG00000164077 | HGLibA_29640 | 1 | -1.125648147 | MON1A | secretory trafficking associated [Source:HGNC Symbol;Acc:HGNC:28207]" |
| ENSG00000164114 | HGLibA_28244 | 1 | -1.125648147 | MAP9 | microtubule associated protein 9 [Source:HGNC Symbol;Acc:HGNC:26118] |
| ENSG00000164172 | HGLibA_29614 | 1 | -1.125648147 | MOCS2 | molybdenum cofactor synthesis 2 [Source:HGNC Symbol;Acc:HGNC:7193] |
| ENSG00000164197 | HGLibA_41580 | 1 | -1.125648147 | RNF180 | ring finger protein 180 [Source:HGNC Symbol;Acc:HGNC:27752] |
| ENSG00000164300 | HGLibA_43497 | 1 | -1.125648147 | SERINC5 | serine incorporator 5 [Source:HGNC Symbol;Acc:HGNC:18825] |
| ENSG00000164330 | HGLibA_14348 | 1 | -1.125648147 | EBF1 | EBF transcription factor 1 [Source:HGNC Symbol;Acc:HGNC:3126] |
| ENSG00000164344 | HGLibA_25276 | 1 | -1.125648147 | KLKB1 | kallikrein B1 [Source:HGNC Symbol;Acc:HGNC:6371] |
| ENSG00000164347 | HGLibA_18951 | 1 | -1.125648147 | GFM2 | G elongation factor mitochondrial 2 [Source:HGNC Symbol;Acc:HGNC:29682] |
| ENSG00000164542 | HGLibA_24659 | 1 | -1.125648147 | KIAA0895 | KIAA0895 [Source:HGNC Symbol;Acc:HGNC:22206] |
| ENSG00000164684 | HGLibA_56583 | 1 | -1.125648147 | ZNF704 | zinc finger protein 704 [Source:HGNC Symbol;Acc:HGNC:32291] |
| ENSG00000164687 | HGLibA_15923 | 1 | -1.125648147 | FABP5 | fatty acid binding protein 5 [Source:HGNC Symbol;Acc:HGNC:3560] |
| ENSG00000164742 | HGLibA_00904 | 1 | -1.125648147 | ADCY1 | adenylate cyclase 1 [Source:HGNC Symbol;Acc:HGNC:232] |
| ENSG00000164764 | HGLibA_42816 | 1 | -1.125648147 | SBSPON | somatomedin B and thrombospondin type 1 domain containing [Source:HGNC Symbol;Acc:HGNC:30362] |
| ENSG00000164794 | HGLibA_24403 | 1 | -1.125648147 | KCNV1 | potassium voltage-gated channel modifier subfamily V member 1 [Source:HGNC Symbol;Acc:HGNC:18861] |
| ENSG00000164828 | HGLibA_47789 | 1 | -1.125648147 | SUN1 | Sad1 and UNC84 domain containing 1 [Source:HGNC Symbol;Acc:HGNC:18587] |
| ENSG00000164850 | HGLibA_19794 | 1 | -1.125648147 | GPER1 | G protein-coupled estrogen receptor 1 [Source:HGNC Symbol;Acc:HGNC:4485] |
| ENSG00000164879 | HGLibA_06805 | 1 | -1.125648147 | CA3 | carbonic anhydrase 3 [Source:HGNC Symbol;Acc:HGNC:1374] |
| ENSG00000164897 | HGLibA_50681 | 1 | -1.125648147 | TMUB1 | transmembrane and ubiquitin like domain containing 1 [Source:HGNC Symbol;Acc:HGNC:21709] |
| ENSG00000164902 | HGLibA_36308 | 1 | -1.125648147 | PHAX | phosphorylated adaptor for RNA export [Source:HGNC Symbol;Acc:HGNC:10241] |
| ENSG00000164929 | HGLibA_04050 | 1 | -1.125648147 | BAALC | MAP3K1 and KLF4 binding [Source:HGNC Symbol;Acc:HGNC:14333]" |
| ENSG00000164935 | HGLibA_12536 | 1 | -1.125648147 | DCSTAMP | dendrocyte expressed seven transmembrane protein [Source:HGNC Symbol;Acc:HGNC:18549] |
| ENSG00000165055 | HGLibA_29003 | 1 | -1.125648147 | METTL2B | methyltransferase like 2B [Source:HGNC Symbol;Acc:HGNC:18272] |
| ENSG00000165091 | HGLibA_49749 | 1 | -1.125648147 | TMC1 | transmembrane channel like 1 [Source:HGNC Symbol;Acc:HGNC:16513] |
| ENSG00000165185 | HGLibA_24830 | 1 | -1.125648147 | KIAA1958 | KIAA1958 [Source:HGNC Symbol;Acc:HGNC:23427] |
| ENSG00000165194 | HGLibA_35409 | 1 | -1.125648147 | PCDH19 | protocadherin 19 [Source:HGNC Symbol;Acc:HGNC:14270] |
| ENSG00000165202 | HGLibA_33710 | 1 | -1.125648147 | OR1Q1 | olfactory receptor family 1 subfamily Q member 1 [Source:HGNC Symbol;Acc:HGNC:8223] |
| ENSG00000165259 | HGLibA_21063 | 1 | -1.125648147 | HDX | highly divergent homeobox [Source:HGNC Symbol;Acc:HGNC:26411] |
| ENSG00000165389 | HGLibA_46900 | 1 | -1.125648147 | SPTSSA | serine palmitoyltransferase small subunit A [Source:HGNC Symbol;Acc:HGNC:20361] |
| ENSG00000165424 | HGLibA_55030 | 1 | -1.125648147 | ZCCHC24 | zinc finger CCHC-type containing 24 [Source:HGNC Symbol;Acc:HGNC:26911] |
| ENSG00000165591 | HGLibA_15903 | 1 | -1.125648147 | FAAH2 | fatty acid amide hydrolase 2 [Source:HGNC Symbol;Acc:HGNC:26440] |
| ENSG00000165617 | HGLibA_12279 | 1 | -1.125648147 | DACT1 | dishevelled binding antagonist of beta catenin 1 [Source:HGNC Symbol;Acc:HGNC:17748] |
| ENSG00000165617 | HGLibA_12281 | 1 | -1.125648147 | DACT1 | dishevelled binding antagonist of beta catenin 1 [Source:HGNC Symbol;Acc:HGNC:17748] |
| ENSG00000165623 | HGLibA_52818 | 1 | -1.125648147 | UCMA | upper zone of growth plate and cartilage matrix associated [Source:HGNC Symbol;Acc:HGNC:25205] |
| ENSG00000165816 | HGLibA_53898 | 1 | -1.125648147 | VWA2 | von Willebrand factor A domain containing 2 [Source:HGNC Symbol;Acc:HGNC:24709] |
| ENSG00000165917 | HGLibA_40270 | 1 | -1.125648147 | RAPSN | receptor associated protein of the synapse [Source:HGNC Symbol;Acc:HGNC:9863] |
| ENSG00000166133 | HGLibA_42208 | 1 | -1.125648147 | RPUSD2 | RNA pseudouridine synthase domain containing 2 [Source:HGNC Symbol;Acc:HGNC:24180] |
| ENSG00000166135 | HGLibA_21300 | 1 | -1.125648147 | HIF1AN | hypoxia inducible factor 1 subunit alpha inhibitor [Source:HGNC Symbol;Acc:HGNC:17113] |
| ENSG00000166199 | HGLibA_01652 | 1 | -1.125648147 | ALKBH3 | alpha-ketoglutaratedependent dioxygenase [Source:HGNC Symbol;Acc:HGNC:30141]" |
| ENSG00000166340 | HGLibA_51158 | 1 | -1.125648147 | TPP1 | tripeptidyl peptidase 1 [Source:HGNC Symbol;Acc:HGNC:2073] |
| ENSG00000166343 | HGLibA_30250 | 1 | -1.125648147 | MSS51 | MSS51 mitochondrial translational activator [Source:HGNC Symbol;Acc:HGNC:21000] |
| ENSG00000166349 | HGLibA_40131 | 1 | -1.125648147 | RAG1 | recombination activating 1 [Source:HGNC Symbol;Acc:HGNC:9831] |
| ENSG00000166411 | HGLibA_22546 | 1 | -1.125648147 | IDH3A | isocitrate dehydrogenase 3 (NAD(+)) alpha [Source:HGNC Symbol;Acc:HGNC:5384] |
| ENSG00000166428 | HGLibA_37045 | 1 | -1.125648147 | PLD4 | phospholipase D family member 4 [Source:HGNC Symbol;Acc:HGNC:23792] |
| ENSG00000166482 | HGLibA_29050 | 1 | -1.125648147 | MFAP4 | microfibril associated protein 4 [Source:HGNC Symbol;Acc:HGNC:7035] |
| ENSG00000166507 | HGLibA_31429 | 1 | -1.125648147 | NDST2 | N-deacetylase and N-sulfotransferase 2 [Source:HGNC Symbol;Acc:HGNC:7681] |
| ENSG00000166510 | HGLibA_07841 | 1 | -1.125648147 | CCDC68 | coiled-coil domain containing 68 [Source:HGNC Symbol;Acc:HGNC:24350] |
| ENSG00000166573 | HGLibA_18586 | 1 | -1.125648147 | GALR1 | galanin receptor 1 [Source:HGNC Symbol;Acc:HGNC:4132] |
| ENSG00000166887 | HGLibA_53788 | 1 | -1.125648147 | VPS39 | HOPS complex subunit [Source:HGNC Symbol;Acc:HGNC:20593]" |
| ENSG00000166960 | HGLibA_07714 | 1 | -1.125648147 | CCDC178 | coiled-coil domain containing 178 [Source:HGNC Symbol;Acc:HGNC:29588] |
| ENSG00000166961 | HGLibA_30142 | 1 | -1.125648147 | MS4A15 | membrane spanning 4-domains A15 [Source:HGNC Symbol;Acc:HGNC:28573] |
| ENSG00000167037 | HGLibA_43859 | 1 | -1.125648147 | SGSM1 | small G protein signaling modulator 1 [Source:HGNC Symbol;Acc:HGNC:29410] |
| ENSG00000167094 | HGLibA_52058 | 1 | -1.125648147 | TTC16 | tetratricopeptide repeat domain 16 [Source:HGNC Symbol;Acc:HGNC:26536] |
| ENSG00000167130 | HGLibA_13792 | 1 | -1.125648147 | DOLPP1 | dolichyldiphosphatase 1 [Source:HGNC Symbol;Acc:HGNC:29565] |
| ENSG00000167182 | HGLibA_46296 | 1 | -1.125648147 | SP2 | Sp2 transcription factor [Source:HGNC Symbol;Acc:HGNC:11207] |
| ENSG00000167183 | HGLibA_38787 | 1 | -1.125648147 | PRR15L | proline rich 15 like [Source:HGNC Symbol;Acc:HGNC:28149] |
| ENSG00000167286 | HGLibA_08368 | 1 | -1.125648147 | CD3D | CD3d molecule [Source:HGNC Symbol;Acc:HGNC:1673] |
| ENSG00000167332 | HGLibA_34100 | 1 | -1.125648147 | OR51E2 | olfactory receptor family 51 subfamily E member 2 [Source:HGNC Symbol;Acc:HGNC:15195] |
| ENSG00000167363 | HGLibA_17728 | 1 | -1.125648147 | FN3K | fructosamine 3 kinase [Source:HGNC Symbol;Acc:HGNC:24822] |
| ENSG00000167395 | HGLibA_56451 | 1 | -1.125648147 | ZNF646 | zinc finger protein 646 [Source:HGNC Symbol;Acc:HGNC:29004] |
| ENSG00000167397 | HGLibA_53677 | 1 | -1.125648147 | VKORC1 | vitamin K epoxide reductase complex subunit 1 [Source:HGNC Symbol;Acc:HGNC:23663] |
| ENSG00000167513 | HGLibA_08891 | 1 | -1.125648147 | CDT1 | chromatin licensing and DNA replication factor 1 [Source:HGNC Symbol;Acc:HGNC:24576] |
| ENSG00000167548 | HGLibA_25331 | 1 | -1.125648147 | KMT2D | lysine methyltransferase 2D [Source:HGNC Symbol;Acc:HGNC:7133] |
| ENSG00000167600 | HGLibA_12141 | 1 | -1.125648147 | CYP2S1 | cytochrome P450 family 2 subfamily S member 1 [Source:HGNC Symbol;Acc:HGNC:15654] |
| ENSG00000167601 | HGLibA_03928 | 1 | -1.125648147 | AXL | AXL receptor tyrosine kinase [Source:HGNC Symbol;Acc:HGNC:905] |
| ENSG00000167703 | HGLibA_45150 | 1 | -1.125648147 | SLC43A2 | solute carrier family 43 member 2 [Source:HGNC Symbol;Acc:HGNC:23087] |
| ENSG00000167741 | HGLibA_19015 | 1 | -1.125648147 | GGT6 | gamma-glutamyltransferase 6 [Source:HGNC Symbol;Acc:HGNC:26891] |
| ENSG00000167751 | HGLibA_25253 | 1 | -1.125648147 | KLK2 | kallikrein related peptidase 2 [Source:HGNC Symbol;Acc:HGNC:6363] |
| ENSG00000167842 | HGLibA_29304 | 1 | -1.125648147 | MIS12 | kinetochore complex component [Source:HGNC Symbol;Acc:HGNC:24967]" |
| ENSG00000167889 | HGLibA_29152 | 1 | -1.125648147 | MGAT5B | 6-mannosylglycoprotein 6-beta-N-acetylglucosaminyltransferase B [Source:HGNC Symbol;Acc:HGNC:24140]" |
| ENSG00000167904 | HGLibA_50495 | 1 | -1.125648147 | TMEM68 | transmembrane protein 68 [Source:HGNC Symbol;Acc:HGNC:26510] |
| ENSG00000167916 | HGLibA_25457 | 1 | -1.125648147 | KRT24 | keratin 24 [Source:HGNC Symbol;Acc:HGNC:18527] |
| ENSG00000168036 | HGLibA_11649 | 1 | -1.125648147 | CTNNB1 | catenin beta 1 [Source:HGNC Symbol;Acc:HGNC:2514] |
| ENSG00000168116 | HGLibA_24786 | 1 | -1.125648147 | KIAA1586 | KIAA1586 [Source:HGNC Symbol;Acc:HGNC:21360] |
| ENSG00000168314 | HGLibA_29606 | 1 | -1.125648147 | MOBP | myelin-associated oligodendrocyte basic protein [Source:HGNC Symbol;Acc:HGNC:7189] |
| ENSG00000168348 | HGLibA_23396 | 1 | -1.125648147 | INSM2 | INSM transcriptional repressor 2 [Source:HGNC Symbol;Acc:HGNC:17539] |
| ENSG00000168350 | HGLibA_12922 | 1 | -1.125648147 | DEGS2 | sphingolipid 2 [Source:HGNC Symbol;Acc:HGNC:20113]" |
| ENSG00000168386 | HGLibA_17530 | 1 | -1.125648147 | FILIP1L | filamin A interacting protein 1 like [Source:HGNC Symbol;Acc:HGNC:24589] |
| ENSG00000168412 | HGLibA_30445 | 1 | -1.125648147 | MTNR1A | melatonin receptor 1A [Source:HGNC Symbol;Acc:HGNC:7463] |
| ENSG00000168438 | HGLibA_08551 | 1 | -1.125648147 | CDC40 | cell division cycle 40 [Source:HGNC Symbol;Acc:HGNC:17350] |
| ENSG00000168439 | HGLibA_47448 | 1 | -1.125648147 | STIP1 | stress induced phosphoprotein 1 [Source:HGNC Symbol;Acc:HGNC:11387] |

| | | | | | |
|---|---|---|---|---|---|
| ENSG00000168496 | HGLibA_17281 | 1 | -1.125648147 | FEN1 | flap structure-specific endonuclease 1 [Source:HGNC Symbol;Acc:HGNC:3650] |
| ENSG00000168582 | HGLibA_11270 | 1 | -1.125648147 | CRYGA | crystallin gamma A [Source:HGNC Symbol;Acc:HGNC:2408] |
| ENSG00000168634 | HGLibA_54260 | 1 | -1.125648147 | WFDC13 | WAP four-disulfide core domain 13 [Source:HGNC Symbol;Acc:HGNC:16131] |
| ENSG00000168702 | HGLibA_27210 | 1 | -1.125648147 | LRP1B | LDL receptor related protein 1B [Source:HGNC Symbol;Acc:HGNC:6693] |
| ENSG00000168958 | HGLibA_29058 | 1 | -1.125648147 | MFF | mitochondrial fission factor [Source:HGNC Symbol;Acc:HGNC:24858] |
| ENSG00000169047 | HGLibA_23619 | 1 | -1.125648147 | IRS1 | insulin receptor substrate 1 [Source:HGNC Symbol;Acc:HGNC:6125] |
| ENSG00000169193 | HGLibA_07579 | 1 | -1.125648147 | CCDC126 | coiled-coil domain containing 126 [Source:HGNC Symbol;Acc:HGNC:22398] |
| ENSG00000169194 | HGLibA_22970 | 1 | -1.125648147 | IL13 | interleukin 13 [Source:HGNC Symbol;Acc:HGNC:5973] |
| ENSG00000169194 | HGLibA_22972 | 1 | -1.125648147 | IL13 | interleukin 13 [Source:HGNC Symbol;Acc:HGNC:5973] |
| ENSG00000169245 | HGLibA_11845 | 1 | -1.125648147 | CXCL10 | C-X-C motif chemokine ligand 10 [Source:HGNC Symbol;Acc:HGNC:10637] |
| ENSG00000169446 | HGLibA_29474 | 1 | -1.125648147 | MMGT1 | membrane magnesium transporter 1 [Source:HGNC Symbol;Acc:HGNC:28100] |
| ENSG00000169499 | HGLibA_37066 | 1 | -1.125648147 | PLEKHA2 | pleckstrin homology domain containing A2 [Source:HGNC Symbol;Acc:HGNC:14336] |
| ENSG00000169598 | HGLibA_13022 | 1 | -1.125648147 | DFFB | DNA fragmentation factor subunit beta [Source:HGNC Symbol;Acc:HGNC:2773] |
| ENSG00000169629 | HGLibA_41043 | 1 | -1.125648147 | RGPD8 | RANBP2-like and GRIP domain containing 8 [Source:HGNC Symbol;Acc:HGNC:9849] |
| ENSG00000169714 | HGLibA_10248 | 1 | -1.125648147 | CNBP | CCHC-type zinc finger nucleic acid binding protein [Source:HGNC Symbol;Acc:HGNC:13164] |
| ENSG00000169964 | HGLibA_50398 | 1 | -1.125648147 | TMEM42 | transmembrane protein 42 [Source:HGNC Symbol;Acc:HGNC:28444] |
| ENSG00000170266 | HGLibA_19218 | 1 | -1.125648147 | GLB1 | galactosidase beta 1 [Source:HGNC Symbol;Acc:HGNC:4298] |
| ENSG00000170279 | HGLibA_06535 | 1 | -1.125648147 | C7orf33 | chromosome 7 open reading frame 33 [Source:HGNC Symbol;Acc:HGNC:21724] |
| ENSG00000170293 | HGLibA_10231 | 1 | -1.125648147 | CMTM8 | CKLF like MARVEL transmembrane domain containing 8 [Source:HGNC Symbol;Acc:HGNC:19179] |
| ENSG00000170296 | HGLibA_18332 | 1 | -1.125648147 | GABARAP | GABA type A receptor-associated protein [Source:HGNC Symbol;Acc:HGNC:4067] |
| ENSG00000170373 | HGLibA_11443 | 1 | -1.125648147 | CST1 | cystatin SN [Source:HGNC Symbol;Acc:HGNC:2473] |
| ENSG00000170417 | HGLibA_50103 | 1 | -1.125648147 | TMEM182 | transmembrane protein 182 [Source:HGNC Symbol;Acc:HGNC:26391] |
| ENSG00000170448 | HGLibA_31851 | 1 | -1.125648147 | NFXL1 | X-box binding like 1 [Source:HGNC Symbol;Acc:HGNC:18726]" |
| ENSG00000170476 | HGLibA_30934 | 1 | -1.125648147 | MZB1 | marginal zone B and B1 cell specific protein [Source:HGNC Symbol;Acc:HGNC:30125] |
| ENSG00000170485 | HGLibA_32397 | 1 | -1.125648147 | NPAS2 | neuronal PAS domain protein 2 [Source:HGNC Symbol;Acc:HGNC:7895] |
| ENSG00000170619 | HGLibA_10669 | 1 | -1.125648147 | COMMD5 | COMM domain containing 5 [Source:HGNC Symbol;Acc:HGNC:17902] |
| ENSG00000170667 | HGLibA_40307 | 1 | -1.125648147 | RASA4B | RAS p21 protein activator 4B [Source:HGNC Symbol;Acc:HGNC:35202] |
| ENSG00000170684 | HGLibA_55762 | 1 | -1.125648147 | ZNF296 | zinc finger protein 296 [Source:HGNC Symbol;Acc:HGNC:15981] |
| ENSG00000170776 | HGLibA_01415 | 1 | -1.125648147 | AKAP13 | A-kinase anchoring protein 13 [Source:HGNC Symbol;Acc:HGNC:371] |
| ENSG00000170791 | HGLibA_09379 | 1 | -1.125648147 | CHCHD7 | coiled-coil-helix-coiled-coil-helix domain containing 7 [Source:HGNC Symbol;Acc:HGNC:28314] |
| ENSG00000170906 | HGLibA_31454 | 1 | -1.125648147 | NDUFA3 | NADH:ubiquinone oxidoreductase subunit A3 [Source:HGNC Symbol;Acc:HGNC:7686] |
| ENSG00000170950 | HGLibA_36242 | 1 | -1.125648147 | PGK2 | phosphoglycerate kinase 2 [Source:HGNC Symbol;Acc:HGNC:8898] |
| ENSG00000171126 | HGLibA_24205 | 1 | -1.125648147 | KCNG3 | potassium voltage-gated channel modifier subfamily G member 3 [Source:HGNC Symbol;Acc:HGNC:18306] |
| ENSG00000171155 | HGLibA_05726 | 1 | -1.125648147 | C1GALT1C1 | C1GALT1 specific chaperone 1 [Source:HGNC Symbol;Acc:HGNC:24338] |
| ENSG00000171219 | HGLibA_08563 | 1 | -1.125648147 | CDC42BPG | CDC42 binding protein kinase gamma [Source:HGNC Symbol;Acc:HGNC:29829] |
| ENSG00000171295 | HGLibA_56003 | 1 | -1.125648147 | ZNF440 | zinc finger protein 440 [Source:HGNC Symbol;Acc:HGNC:20874] |
| ENSG00000171307 | HGLibA_55084 | 1 | -1.125648147 | ZDHHC16 | zinc finger DHHC-type containing 16 [Source:HGNC Symbol;Acc:HGNC:20714] |
| ENSG00000171435 | HGLibA_25867 | 1 | -1.125648147 | KSR2 | kinase suppressor of ras 2 [Source:HGNC Symbol;Acc:HGNC:18610] |
| ENSG00000171444 | HGLibA_28563 | 1 | -1.125648147 | MCC | WNT signaling pathway regulator [Source:HGNC Symbol;Acc:HGNC:6935]" |
| ENSG00000171643 | HGLibA_42590 | 1 | -1.125648147 | S100Z | S100 calcium binding protein Z [Source:HGNC Symbol;Acc:HGNC:30367] |
| ENSG00000171793 | HGLibA_11669 | 1 | -1.125648147 | CTPS1 | CTP synthase 1 [Source:HGNC Symbol;Acc:HGNC:2519] |
| ENSG00000171931 | HGLibA_17136 | 1 | -1.125648147 | FBXW10 | F-box and WD repeat domain containing 10 [Source:HGNC Symbol;Acc:HGNC:1211] |
| ENSG00000171940 | HGLibA_55601 | 1 | -1.125648147 | ZNF217 | zinc finger protein 217 [Source:HGNC Symbol;Acc:HGNC:13009] |
| ENSG00000171992 | HGLibA_48019 | 1 | -1.125648147 | SYNPO | synaptopodin [Source:HGNC Symbol;Acc:HGNC:30672] |
| ENSG00000172058 | HGLibA_43470 | 1 | -1.125648147 | SERF1A | small EDRK-rich factor 1A [Source:HGNC Symbol;Acc:HGNC:10755] |
| ENSG00000172061 | HGLibA_27259 | 1 | -1.125648147 | LRRC15 | leucine rich repeat containing 15 [Source:HGNC Symbol;Acc:HGNC:20818] |
| ENSG00000172123 | HGLibA_45484 | 1 | -1.125648147 | SLFN12 | schlafen family member 12 [Source:HGNC Symbol;Acc:HGNC:25500] |
| ENSG00000172146 | HGLibA_33641 | 1 | -1.125648147 | OR1A1 | olfactory receptor family 1 subfamily A member 1 [Source:HGNC Symbol;Acc:HGNC:8179] |
| ENSG00000172155 | HGLibA_26108 | 1 | -1.125648147 | LCE1D | late cornified envelope 1D [Source:HGNC Symbol;Acc:HGNC:29465] |
| ENSG00000172183 | HGLibA_23659 | 1 | -1.125648147 | ISG20 | interferon stimulated exonuclease gene 20 [Source:HGNC Symbol;Acc:HGNC:6130] |
| ENSG00000172365 | HGLibA_34263 | 1 | -1.125648147 | OR5B2 | olfactory receptor family 5 subfamily B member 2 [Source:HGNC Symbol;Acc:HGNC:8323] |
| ENSG00000172457 | HGLibA_34571 | 1 | -1.125648147 | OR9G4 | olfactory receptor family 9 subfamily G member 4 [Source:HGNC Symbol;Acc:HGNC:15322] |
| ENSG00000172482 | HGLibA_01272 | 1 | -1.125648147 | AGXT | alanine--glyoxylate and serine--pyruvate aminotransferase [Source:HGNC Symbol;Acc:HGNC:341] |
| ENSG00000172769 | HGLibA_34269 | 1 | -1.125648147 | OR5B3 | olfactory receptor family 5 subfamily B member 3 [Source:HGNC Symbol;Acc:HGNC:8324] |
| ENSG00000172818 | HGLibA_34800 | 1 | -1.125648147 | OVOL1 | ovo like transcriptional repressor 1 [Source:HGNC Symbol;Acc:HGNC:8525] |
| ENSG00000172824 | HGLibA_09228 | 1 | -1.125648147 | CES4A | carboxylesterase 4A [Source:HGNC Symbol;Acc:HGNC:26741] |
| ENSG00000172888 | HGLibA_56408 | 1 | -1.125648147 | ZNF621 | zinc finger protein 621 [Source:HGNC Symbol;Acc:HGNC:24787] |
| ENSG00000172939 | HGLibA_34838 | 1 | -1.125648147 | OXSR1 | oxidative stress responsive 1 [Source:HGNC Symbol;Acc:HGNC:8508] |
| ENSG00000172977 | HGLibA_24058 | 1 | -1.125648147 | KAT5 | lysine acetyltransferase 5 [Source:HGNC Symbol;Acc:HGNC:5275] |
| ENSG00000172977 | HGLibA_24056 | 1 | -1.125648147 | KAT5 | lysine acetyltransferase 5 [Source:HGNC Symbol;Acc:HGNC:5275] |
| ENSG00000173137 | HGLibA_00901 | 1 | -1.125648147 | ADCK5 | aarF domain containing kinase 5 [Source:HGNC Symbol;Acc:HGNC:21738] |
| ENSG00000173221 | HGLibA_19314 | 1 | -1.125648147 | GLRX | glutaredoxin [Source:HGNC Symbol;Acc:HGNC:4330] |
| ENSG00000173258 | HGLibA_56075 | 1 | -1.125648147 | ZNF483 | zinc finger protein 483 [Source:HGNC Symbol;Acc:HGNC:23384] |
| ENSG00000173452 | HGLibA_50159 | 1 | -1.125648147 | TMEM196 | transmembrane protein 196 [Source:HGNC Symbol;Acc:HGNC:22431] |
| ENSG00000173486 | HGLibA_17570 | 1 | -1.125648147 | FKBP2 | FK506 binding protein 2 [Source:HGNC Symbol;Acc:HGNC:3718] |
| ENSG00000173559 | HGLibA_31025 | 1 | -1.125648147 | NABP1 | nucleic acid binding protein 1 [Source:HGNC Symbol;Acc:HGNC:26232] |
| ENSG00000173578 | HGLibA_54499 | 1 | -1.125648147 | XCR1 | X-C motif chemokine receptor 1 [Source:HGNC Symbol;Acc:HGNC:1625] |
| ENSG00000173581 | HGLibA_07522 | 1 | -1.125648147 | CCDC106 | coiled-coil domain containing 106 [Source:HGNC Symbol;Acc:HGNC:30181] |
| ENSG00000173692 | HGLibA_39149 | 1 | -1.125648147 | PSMD1 | non-ATPase 1 [Source:HGNC Symbol;Acc:HGNC:9554]" |
| ENSG00000173728 | HGLibA_05728 | 1 | -1.125648147 | C1orf100 | chromosome 1 open reading frame 100 [Source:HGNC Symbol;Acc:HGNC:30435] |
| ENSG00000173805 | HGLibA_20838 | 1 | -1.125648147 | HAP1 | huntingtin associated protein 1 [Source:HGNC Symbol;Acc:HGNC:4812] |
| ENSG00000173812 | HGLibA_14705 | 1 | -1.125648147 | EIF1 | eukaryotic translation initiation factor 1 [Source:HGNC Symbol;Acc:HGNC:3249] |
| ENSG00000174080 | HGLibA_11705 | 1 | -1.125648147 | CTSF | cathepsin F [Source:HGNC Symbol;Acc:HGNC:2531] |
| ENSG00000174151 | HGLibA_11982 | 1 | -1.125648147 | CYB561D1 | cytochrome b561 family member D1 [Source:HGNC Symbol;Acc:HGNC:26804] |
| ENSG00000174197 | HGLibA_29122 | 1 | -1.125648147 | MGA | MAX dimerization protein [Source:HGNC Symbol;Acc:HGNC:14010]" |
| ENSG00000174339 | HGLibA_33909 | 1 | -1.125648147 | OR2Y1 | olfactory receptor family 2 subfamily Y member 1 [Source:HGNC Symbol;Acc:HGNC:14837] |
| ENSG00000174564 | HGLibA_23079 | 1 | -1.125648147 | IL20RB | interleukin 20 receptor subunit beta [Source:HGNC Symbol;Acc:HGNC:6004] |
| ENSG00000174579 | HGLibA_30213 | 1 | -1.125648147 | MSL2 | MSL complex subunit 2 [Source:HGNC Symbol;Acc:HGNC:25544] |
| ENSG00000174738 | HGLibA_32577 | 1 | -1.125648147 | NR1D2 | nuclear receptor subfamily 1 group D member 2 [Source:HGNC Symbol;Acc:HGNC:7963] |
| ENSG00000174957 | HGLibA_34306 | 1 | -1.125648147 | OR5J2 | olfactory receptor family 5 subfamily J member 2 [Source:HGNC Symbol;Acc:HGNC:19612] |
| ENSG00000175097 | HGLibA_40134 | 1 | -1.125648147 | RAG2 | recombination activating 2 [Source:HGNC Symbol;Acc:HGNC:9832] |
| ENSG00000175189 | HGLibA_23307 | 1 | -1.125648147 | INHBC | inhibin subunit beta C [Source:HGNC Symbol;Acc:HGNC:6068] |
| ENSG00000175376 | HGLibA_14707 | 1 | -1.125648147 | EIF1AD | eukaryotic translation initiation factor 1A domain containing [Source:HGNC Symbol;Acc:HGNC:28147] |
| ENSG00000175395 | HGLibA_55663 | 1 | -1.125648147 | ZNF25 | zinc finger protein 25 [Source:HGNC Symbol;Acc:HGNC:13043] |
| ENSG00000175893 | HGLibA_55102 | 1 | -1.125648147 | ZDHHC21 | zinc finger DHHC-type containing 21 [Source:HGNC Symbol;Acc:HGNC:20750] |
| ENSG00000175920 | HGLibA_13784 | 1 | -1.125648147 | DOK7 | docking protein 7 [Source:HGNC Symbol;Acc:HGNC:26594] |
| ENSG00000176020 | HGLibA_01798 | 1 | -1.125648147 | AMIGO3 | adhesion molecule with Ig like domain 3 [Source:HGNC Symbol;Acc:HGNC:24075] |
| ENSG00000176049 | HGLibA_23924 | 1 | -1.125648147 | JAKMIP2 | janus kinase and microtubule interacting protein 2 [Source:HGNC Symbol;Acc:HGNC:29067] |
| ENSG00000176153 | HGLibA_20174 | 1 | -1.125648147 | GPX2 | glutathione peroxidase 2 [Source:HGNC Symbol;Acc:HGNC:4554] |
| ENSG00000176170 | HGLibA_46644 | 1 | -1.125648147 | SPHK1 | sphingosine kinase 1 [Source:HGNC Symbol;Acc:HGNC:11240] |
| ENSG00000176371 | HGLibA_56996 | 1 | -1.125648147 | ZSCAN2 | zinc finger and SCAN domain containing 2 [Source:HGNC Symbol;Acc:HGNC:20994] |
| ENSG00000176678 | HGLibA_17899 | 1 | -1.125648147 | FOXL1 | forkhead box L1 [Source:HGNC Symbol;Acc:HGNC:3817] |
| ENSG00000176715 | HGLibA_00550 | 1 | -1.125648147 | ACSF3 | acyl-CoA synthetase family member 3 [Source:HGNC Symbol;Acc:HGNC:27288] |
| ENSG00000176742 | HGLibA_34136 | 1 | -1.125648147 | OR51V1 | olfactory receptor family 51 subfamily V member 1 [Source:HGNC Symbol;Acc:HGNC:19597] |
| ENSG00000176788 | HGLibA_04170 | 1 | -1.125648147 | BASP1 | brain abundant membrane attached signal protein 1 [Source:HGNC Symbol;Acc:HGNC:957] |
| ENSG00000176928 | HGLibA_18817 | 1 | -1.125648147 | GCNT4 | core 2 [Source:HGNC Symbol;Acc:HGNC:17973]" |
| ENSG00000176974 | HGLibA_44061 | 1 | -1.125648147 | SHMT1 | serine hydroxymethyltransferase 1 [Source:HGNC Symbol;Acc:HGNC:10850] |
| ENSG00000177051 | HGLibA_17112 | 1 | -1.125648147 | FBXO46 | F-box protein 46 [Source:HGNC Symbol;Acc:HGNC:25069] |
| ENSG00000177108 | HGLibA_55106 | 1 | -1.125648147 | ZDHHC22 | zinc finger DHHC-type containing 22 [Source:HGNC Symbol;Acc:HGNC:20106] |
| ENSG00000177283 | HGLibA_18285 | 1 | -1.125648147 | FZD8 | frizzled class receptor 8 [Source:HGNC Symbol;Acc:HGNC:4046] |
| ENSG00000177383 | HGLibA_27985 | 1 | -1.125648147 | MAGEF1 | MAGE family member F1 [Source:HGNC Symbol;Acc:HGNC:29639] |
| ENSG00000177398 | HGLibA_52982 | 1 | -1.125648147 | UMODL1 | uromodulin like 1 [Source:HGNC Symbol;Acc:HGNC:12560] |
| ENSG00000177464 | HGLibA_20041 | 1 | -1.125648147 | GPR4 | G protein-coupled receptor 4 [Source:HGNC Symbol;Acc:HGNC:4497] |
| ENSG00000177706 | HGLibA_16450 | 1 | -1.125648147 | FAM20C | golgi associated secretory pathway kinase [Source:HGNC Symbol;Acc:HGNC:22140]" |
| ENSG00000177731 | HGLibA_17618 | 1 | -1.125648147 | FLII | actin remodeling protein [Source:HGNC Symbol;Acc:HGNC:3750]" |
| ENSG00000177752 | HGLibA_54687 | 1 | -1.125648147 | YIPF7 | Yip1 domain family member 7 [Source:HGNC Symbol;Acc:HGNC:26825] |
| ENSG00000177946 | HGLibA_09044 | 1 | -1.125648147 | CENPBD1 | CENPB DNA-binding domain containing 1 [Source:HGNC Symbol;Acc:HGNC:28272] |
| ENSG00000178057 | HGLibA_31487 | 1 | -1.125648147 | NDUFAF3 | NADH:ubiquinone oxidoreductase complex assembly factor 3 [Source:HGNC Symbol;Acc:HGNC:29918] |
| ENSG00000178074 | HGLibA_06166 | 1 | -1.125648147 | C2orf69 | chromosome 2 open reading frame 69 [Source:HGNC Symbol;Acc:HGNC:26799] |
| ENSG00000178096 | HGLibA_04680 | 1 | -1.125648147 | BOLA1 | bolA family member 1 [Source:HGNC Symbol;Acc:HGNC:24263] |
| ENSG00000178297 | HGLibA_50654 | 1 | -1.125648147 | TMPRSS9 | transmembrane serine protease 9 [Source:HGNC Symbol;Acc:HGNC:30079] |
| ENSG00000178531 | HGLibA_11756 | 1 | -1.125648147 | CTXN1 | cortexin 1 [Source:HGNC Symbol;Acc:HGNC:31108] |
| ENSG00000178695 | HGLibA_24420 | 1 | -1.125648147 | KCTD12 | potassium channel tetramerization domain containing 12 [Source:HGNC Symbol;Acc:HGNC:14678] |
| ENSG00000178772 | HGLibA_10951 | 1 | -1.125648147 | CPN2 | carboxypeptidase N subunit 2 [Source:HGNC Symbol;Acc:HGNC:2313] |
| ENSG00000178814 | HGLibA_33419 | 1 | -1.125648147 | OPLAH | ATP-hydrolysing [Source:HGNC Symbol;Acc:HGNC:8149]" |
| ENSG00000179010 | HGLibA_29828 | 1 | -1.125648147 | MRFAP1 | Morf4 family associated protein 1 [Source:HGNC Symbol;Acc:HGNC:24549] |
| ENSG00000179029 | HGLibA_49862 | 1 | -1.125648147 | TMEM107 | transmembrane protein 107 [Source:HGNC Symbol;Acc:HGNC:28128] |
| ENSG00000179520 | HGLibA_44479 | 1 | -1.125648147 | SLC17A8 | solute carrier family 17 member 8 [Source:HGNC Symbol;Acc:HGNC:20151] |
| ENSG00000179562 | HGLibA_18765 | 1 | -1.125648147 | GCC1 | GRIP and coiled-coil domain containing 1 [Source:HGNC Symbol;Acc:HGNC:19095] |
| ENSG00000179564 | HGLibA_27560 | 1 | -1.125648147 | LSMEM2 | leucine rich single-pass membrane protein 2 [Source:HGNC Symbol;Acc:HGNC:26781] |
| ENSG00000179695 | HGLibA_34386 | 1 | -1.125648147 | OR6C2 | olfactory receptor family 6 subfamily C member 2 [Source:HGNC Symbol;Acc:HGNC:15436] |
| ENSG00000179796 | HGLibA_27345 | 1 | -1.125648147 | LRRC3B | leucine rich repeat containing 3B [Source:HGNC Symbol;Acc:HGNC:28105] |
| ENSG00000180205 | HGLibA_54278 | 1 | -1.125648147 | WFDC9 | WAP four-disulfide core domain 9 [Source:HGNC Symbol;Acc:HGNC:20380] |
| ENSG00000180219 | HGLibA_16693 | 1 | -1.125648147 | FAM71C | family with sequence similarity 71 member C [Source:HGNC Symbol;Acc:HGNC:28594] |
| ENSG00000180305 | HGLibA_54246 | 1 | -1.125648147 | WFDC10A | WAP four-disulfide core domain 10A [Source:HGNC Symbol;Acc:HGNC:16139] |
| ENSG00000180884 | HGLibA_56771 | 1 | -1.125648147 | ZNF792 | zinc finger protein 792 [Source:HGNC Symbol;Acc:HGNC:24751] |
| ENSG00000180919 | HGLibA_34226 | 1 | -1.125648147 | OR56B4 | olfactory receptor family 56 subfamily B member 4 [Source:HGNC Symbol;Acc:HGNC:15248] |
| ENSG00000181036 | HGLibA_17241 | 1 | -1.125648147 | FCRL6 | Fc receptor like 6 [Source:HGNC Symbol;Acc:HGNC:31910] |
| ENSG00000181284 | HGLibA_49845 | 1 | -1.125648147 | TMEM102 | transmembrane protein 102 [Source:HGNC Symbol;Acc:HGNC:26722] |
| ENSG00000181284 | HGLibA_49844 | 1 | -1.125648147 | TMEM102 | transmembrane protein 102 [Source:HGNC Symbol;Acc:HGNC:26722] |
| ENSG00000181449 | HGLibA_46251 | 1 | -1.125648147 | SOX2 | SRY-box 2 [Source:HGNC Symbol;Acc:HGNC:11195] |
| ENSG00000181458 | HGLibA_50409 | 1 | -1.125648147 | TMEM45A | transmembrane protein 45A [Source:HGNC Symbol;Acc:HGNC:25480] |
| ENSG00000181626 | HGLibA_02136 | 1 | -1.125648147 | ANKRD62 | ankyrin repeat domain 62 [Source:HGNC Symbol;Acc:HGNC:35241] |
| ENSG00000181722 | HGLibA_54837 | 1 | -1.125648147 | ZBTB20 | zinc finger and BTB domain containing 20 [Source:HGNC Symbol;Acc:HGNC:13503] |
| ENSG00000181733 | HGLibA_33910 | 1 | -1.125648147 | OR2Z1 | olfactory receptor family 2 subfamily Z member 1 [Source:HGNC Symbol;Acc:HGNC:15391] |

| | | | | | |
|---|---|---|---|---|---|
| ENSG00000181788 | HGLibA_44107 | 1 | -1.125648147 | SIAH2 | siah E3 ubiquitin protein ligase 2 [Source:HGNC Symbol;Acc:HGNC:10858] |
| ENSG00000181904 | HGLibA_06367 | 1 | -1.125648147 | C5orf24 | chromosome 5 open reading frame 24 [Source:HGNC Symbol;Acc:HGNC:26746] |
| ENSG00000182035 | HGLibA_00974 | 1 | -1.125648147 | ADIG | adipogenin [Source:HGNC Symbol;Acc:HGNC:28606] |
| ENSG00000182187 | HGLibA_11273 | 1 | -1.125648147 | CRYGB | crystallin gamma B [Source:HGNC Symbol;Acc:HGNC:2409] |
| ENSG00000182263 | HGLibA_17519 | 1 | -1.125648147 | FIGN | microtubule severing factor [Source:HGNC Symbol;Acc:HGNC:13285]" |
| ENSG00000182315 | HGLibA_28478 | 1 | -1.125648147 | MBD3L3 | methyl-CpG binding domain protein 3 like 3 [Source:HGNC Symbol;Acc:HGNC:37205] |
| ENSG00000182359 | HGLibA_24104 | 1 | -1.125648147 | KBTBD3 | kelch repeat and BTB domain containing 3 [Source:HGNC Symbol;Acc:HGNC:22934] |
| ENSG00000182450 | HGLibA_24323 | 1 | -1.125648147 | KCNK4 | potassium two pore domain channel subfamily K member 4 [Source:HGNC Symbol;Acc:HGNC:6279] |
| ENSG00000182544 | HGLibA_29106 | 1 | -1.125648147 | MFSD5 | major facilitator superfamily domain containing 5 [Source:HGNC Symbol;Acc:HGNC:28156] |
| ENSG00000182628 | HGLibA_44265 | 1 | -1.125648147 | SKA2 | spindle and kinetochore associated complex subunit 2 [Source:HGNC Symbol;Acc:HGNC:28006] |
| ENSG00000182674 | HGLibA_24158 | 1 | -1.125648147 | KCNB2 | potassium voltage-gated channel subfamily B member 2 [Source:HGNC Symbol;Acc:HGNC:6232] |
| ENSG00000182768 | HGLibA_31883 | 1 | -1.125648147 | NGRN | neurite outgrowth associated [Source:HGNC Symbol;Acc:HGNC:18077]" |
| ENSG00000182795 | HGLibA_05755 | 1 | -1.125648147 | C1orf116 | chromosome 1 open reading frame 116 [Source:HGNC Symbol;Acc:HGNC:28667] |
| ENSG00000182903 | HGLibA_56629 | 1 | -1.125648147 | ZNF721 | zinc finger protein 721 [Source:HGNC Symbol;Acc:HGNC:29425] |
| ENSG00000182985 | HGLibA_06964 | 1 | -1.125648147 | CADM1 | cell adhesion molecule 1 [Source:HGNC Symbol;Acc:HGNC:5951] |
| ENSG00000183066 | HGLibA_53979 | 1 | -1.125648147 | WBP2NL | WBP2 N-terminal like [Source:HGNC Symbol;Acc:HGNC:28389] |
| ENSG00000183251 | HGLibA_34085 | 1 | -1.125648147 | OR51B4 | olfactory receptor family 51 subfamily B member 4 [Source:HGNC Symbol;Acc:HGNC:14708] |
| ENSG00000183255 | HGLibA_39566 | 1 | -1.125648147 | PTTG1IP | PTTG1 interacting protein [Source:HGNC Symbol;Acc:HGNC:13524] |
| ENSG00000183269 | HGLibA_34165 | 1 | -1.125648147 | OR52E8 | olfactory receptor family 52 subfamily E member 8 [Source:HGNC Symbol;Acc:HGNC:15217] |
| ENSG00000183379 | HGLibA_47972 | 1 | -1.125648147 | SYNDIG1L | synapse differentiation inducing 1 like [Source:HGNC Symbol;Acc:HGNC:32388] |
| ENSG00000183389 | HGLibA_34216 | 1 | -1.125648147 | OR56A4 | olfactory receptor family 56 subfamily A member 4 [Source:HGNC Symbol;Acc:HGNC:14791] |
| ENSG00000183579 | HGLibA_56943 | 1 | -1.125648147 | ZNRF3 | zinc and ring finger 3 [Source:HGNC Symbol;Acc:HGNC:18126] |
| ENSG00000183617 | HGLibA_30019 | 1 | -1.125648147 | MRPL54 | mitochondrial ribosomal protein L54 [Source:HGNC Symbol;Acc:HGNC:16685] |
| ENSG00000183691 | HGLibA_32254 | 1 | -1.125648147 | NOG | noggin [Source:HGNC Symbol;Acc:HGNC:7866] |
| ENSG00000183706 | HGLibA_34049 | 1 | -1.125648147 | OR4N4 | olfactory receptor family 4 subfamily N member 4 [Source:HGNC Symbol;Acc:HGNC:15375] |
| ENSG00000183709 | HGLibA_22721 | 1 | -1.125648147 | IFNL2 | interferon lambda 2 [Source:HGNC Symbol;Acc:HGNC:18364] |
| ENSG00000183718 | HGLibA_51539 | 1 | -1.125648147 | TRIM52 | tripartite motif containing 52 [Source:HGNC Symbol;Acc:HGNC:19024] |
| ENSG00000183801 | HGLibA_33355 | 1 | -1.125648147 | OLFML1 | olfactomedin like 1 [Source:HGNC Symbol;Acc:HGNC:24473] |
| ENSG00000183844 | HGLibA_16580 | 1 | -1.125648147 | FAM3B | family with sequence similarity 3 member B [Source:HGNC Symbol;Acc:HGNC:1253] |
| ENSG00000184110 | HGLibA_14768 | 1 | -1.125648147 | EIF3C | eukaryotic translation initiation factor 3 subunit C [Source:HGNC Symbol;Acc:HGNC:3279] |
| ENSG00000184160 | HGLibA_01052 | 1 | -1.125648147 | ADRA2C | adrenoceptor alpha 2C [Source:HGNC Symbol;Acc:HGNC:283] |
| ENSG00000184220 | HGLibA_10207 | 1 | -1.125648147 | CMSS1 | cms1 ribosomal small subunit homolog [Source:HGNC Symbol;Acc:HGNC:28666] |
| ENSG00000184254 | HGLibA_01547 | 1 | -1.125648147 | ALDH1A3 | aldehyde dehydrogenase 1 family member A3 [Source:HGNC Symbol;Acc:HGNC:409] |
| ENSG00000184277 | HGLibA_49688 | 1 | -1.125648147 | TM2D3 | TM2 domain containing 3 [Source:HGNC Symbol;Acc:HGNC:24128] |
| ENSG00000184371 | HGLibA_11321 | 1 | -1.125648147 | CSF1 | colony stimulating factor 1 [Source:HGNC Symbol;Acc:HGNC:2432] |
| ENSG00000184389 | HGLibA_00013 | 1 | -1.125648147 | A3GALT2 | 3-galactosyltransferase 2 [Source:HGNC Symbol;Acc:HGNC:30005]" |
| ENSG00000184402 | HGLibA_47106 | 1 | -1.125648147 | SS18L1 | nBAF chromatin remodeling complex subunit [Source:HGNC Symbol;Acc:HGNC:15592]" |
| ENSG00000184408 | HGLibA_24176 | 1 | -1.125648147 | KCND2 | potassium voltage-gated channel subfamily D member 2 [Source:HGNC Symbol;Acc:HGNC:6238] |
| ENSG00000184434 | HGLibA_27275 | 1 | -1.125648147 | LRRC19 | leucine rich repeat containing 19 [Source:HGNC Symbol;Acc:HGNC:23379] |
| ENSG00000184575 | HGLibA_54577 | 1 | -1.125648147 | XPOT | exportin for tRNA [Source:HGNC Symbol;Acc:HGNC:12826] |
| ENSG00000184967 | HGLibA_32242 | 1 | -1.125648147 | NOC4L | nucleolar complex associated 4 homolog [Source:HGNC Symbol;Acc:HGNC:28461] |
| ENSG00000185015 | HGLibA_06798 | 1 | -1.125648147 | CA13 | carbonic anhydrase 13 [Source:HGNC Symbol;Acc:HGNC:14914] |
| ENSG00000185022 | HGLibA_27878 | 1 | -1.125648147 | MAFF | MAF bZIP transcription factor F [Source:HGNC Symbol;Acc:HGNC:6780] |
| ENSG00000185052 | HGLibA_44622 | 1 | -1.125648147 | SLC24A3 | solute carrier family 24 member 3 [Source:HGNC Symbol;Acc:HGNC:10977] |
| ENSG00000185069 | HGLibA_25539 | 1 | -1.125648147 | KRT76 | keratin 76 [Source:HGNC Symbol;Acc:HGNC:24430] |
| ENSG00000185104 | HGLibA_15946 | 1 | -1.125648147 | FAF1 | Fas associated factor 1 [Source:HGNC Symbol;Acc:HGNC:3578] |
| ENSG00000185122 | HGLibA_22219 | 1 | -1.125648147 | HSF1 | heat shock transcription factor 1 [Source:HGNC Symbol;Acc:HGNC:5224] |
| ENSG00000185155 | HGLibA_29321 | 1 | -1.125648147 | MIXL1 | Mix paired-like homeobox [Source:HGNC Symbol;Acc:HGNC:13363] |
| ENSG00000185264 | HGLibA_49098 | 1 | -1.125648147 | TEX33 | testis expressed 33 [Source:HGNC Symbol;Acc:HGNC:28568] |
| ENSG00000185467 | HGLibA_25373 | 1 | -1.125648147 | KPNA7 | karyopherin subunit alpha 7 [Source:HGNC Symbol;Acc:HGNC:21839] |
| ENSG00000185507 | HGLibA_23598 | 1 | -1.125648147 | IRF7 | interferon regulatory factor 7 [Source:HGNC Symbol;Acc:HGNC:6122] |
| ENSG00000185513 | HGLibA_25895 | 1 | -1.125648147 | L3MBTL1 | histone methyl-lysine binding protein [Source:HGNC Symbol;Acc:HGNC:15905]" |
| ENSG00000185624 | HGLibA_34910 | 1 | -1.125648147 | P4HB | prolyl 4-hydroxylase subunit beta [Source:HGNC Symbol;Acc:HGNC:8548] |
| ENSG00000185829 | HGLibA_02951 | 1 | -1.125648147 | ARL17A | ADP ribosylation factor like GTPase 17A [Source:HGNC Symbol;Acc:HGNC:24096] |
| ENSG00000185875 | HGLibA_49359 | 1 | -1.125648147 | THNSL1 | threonine synthase like 1 [Source:HGNC Symbol;Acc:HGNC:26160] |
| ENSG00000185905 | HGLibA_05456 | 1 | -1.125648147 | C16orf54 | chromosome 16 open reading frame 54 [Source:HGNC Symbol;Acc:HGNC:26649] |
| ENSG00000186009 | HGLibA_03636 | 1 | -1.125648147 | ATP4B | ATPase H+/K+ transporting subunit beta [Source:HGNC Symbol;Acc:HGNC:820] |
| ENSG00000186017 | HGLibA_56261 | 1 | -1.125648147 | ZNF566 | zinc finger protein 566 [Source:HGNC Symbol;Acc:HGNC:25919] |
| ENSG00000186020 | HGLibA_56173 | 1 | -1.125648147 | ZNF529 | zinc finger protein 529 [Source:HGNC Symbol;Acc:HGNC:29328] |
| ENSG00000186111 | HGLibA_36721 | 1 | -1.125648147 | PIP5K1C | phosphatidylinositol-4-phosphate 5-kinase type 1 gamma [Source:HGNC Symbol;Acc:HGNC:8996] |
| ENSG00000186193 | HGLibA_42733 | 1 | -1.125648147 | SAPCD2 | suppressor APC domain containing 2 [Source:HGNC Symbol;Acc:HGNC:28055] |
| ENSG00000186297 | HGLibA_18369 | 1 | -1.125648147 | GABRA5 | gamma-aminobutyric acid type A receptor alpha5 subunit [Source:HGNC Symbol;Acc:HGNC:4079] |
| ENSG00000186340 | HGLibA_49324 | 1 | -1.125648147 | THBS2 | thrombospondin 2 [Source:HGNC Symbol;Acc:HGNC:11786] |
| ENSG00000186493 | HGLibA_06379 | 1 | -1.125648147 | C5orf38 | chromosome 5 open reading frame 38 [Source:HGNC Symbol;Acc:HGNC:24226] |
| ENSG00000186818 | HGLibA_26516 | 1 | -1.125648147 | LILRB4 | leukocyte immunoglobulin like receptor B4 [Source:HGNC Symbol;Acc:HGNC:6608] |
| ENSG00000186860 | HGLibA_25659 | 1 | -1.125648147 | KRTAP17-1 | keratin associated protein 17-1 [Source:HGNC Symbol;Acc:HGNC:18917] |
| ENSG00000186907 | HGLibA_42420 | 1 | -1.125648147 | RTN4RL2 | reticulon 4 receptor like 2 [Source:HGNC Symbol;Acc:HGNC:23053] |
| ENSG00000186919 | HGLibA_54766 | 1 | -1.125648147 | ZACN | zinc activated ion channel [Source:HGNC Symbol;Acc:HGNC:29504] |
| ENSG00000186943 | HGLibA_33608 | 1 | -1.125648147 | OR13C8 | olfactory receptor family 13 subfamily C member 8 [Source:HGNC Symbol;Acc:HGNC:15103] |
| ENSG00000187003 | HGLibA_00625 | 1 | -1.125648147 | ACTL7A | actin like 7A [Source:HGNC Symbol;Acc:HGNC:161] |
| ENSG00000187045 | HGLibA_50649 | 1 | -1.125648147 | TMPRSS6 | transmembrane serine protease 6 [Source:HGNC Symbol;Acc:HGNC:16517] |
| ENSG00000187048 | HGLibA_12171 | 1 | -1.125648147 | CYP4A11 | cytochrome P450 family 4 subfamily A member 11 [Source:HGNC Symbol;Acc:HGNC:2642] |
| ENSG00000187051 | HGLibA_42097 | 1 | -1.125648147 | RPS19BP1 | ribosomal protein S19 binding protein 1 [Source:HGNC Symbol;Acc:HGNC:28749] |
| ENSG00000187180 | HGLibA_26124 | 1 | -1.125648147 | LCE2C | late cornified envelope 2C [Source:HGNC Symbol;Acc:HGNC:29460] |
| ENSG00000187240 | HGLibA_14247 | 1 | -1.125648147 | DYNC2H1 | dynein cytoplasmic 2 heavy chain 1 [Source:HGNC Symbol;Acc:HGNC:2962] |
| ENSG00000187266 | HGLibA_15354 | 1 | -1.125648147 | EPOR | erythropoietin receptor [Source:HGNC Symbol;Acc:HGNC:3416] |
| ENSG00000187272 | HGLibA_25847 | 1 | -1.125648147 | KRTAP9-8 | keratin associated protein 9-8 [Source:HGNC Symbol;Acc:HGNC:17231] |
| ENSG00000187658 | HGLibA_06403 | 1 | -1.125648147 | C5orf52 | chromosome 5 open reading frame 52 [Source:HGNC Symbol;Acc:HGNC:35121] |
| ENSG00000187664 | HGLibA_20850 | 1 | -1.125648147 | HAPLN4 | hyaluronan and proteoglycan link protein 4 [Source:HGNC Symbol;Acc:HGNC:31357] |
| ENSG00000187857 | HGLibA_34410 | 1 | -1.125648147 | OR6C75 | olfactory receptor family 6 subfamily C member 75 [Source:HGNC Symbol;Acc:HGNC:31304] |
| ENSG00000187871 | HGLibA_18977 | 1 | -1.125648147 | GFRAL | GDNF family receptor alpha like [Source:HGNC Symbol;Acc:HGNC:32789] |
| ENSG00000187994 | HGLibA_41300 | 1 | -1.125648147 | RINL | Ras and Rab interactor like [Source:HGNC Symbol;Acc:HGNC:24795] |
| ENSG00000188032 | HGLibA_05695 | 1 | -1.125648147 | C19orf67 | chromosome 19 open reading frame 67 [Source:HGNC Symbol;Acc:HGNC:34354] |
| ENSG00000188037 | HGLibA_09821 | 1 | -1.125648147 | CLCN1 | chloride voltage-gated channel 1 [Source:HGNC Symbol;Acc:HGNC:2019] |
| ENSG00000188038 | HGLibA_32713 | 1 | -1.125648147 | NRN1L | neuritin 1 like [Source:HGNC Symbol;Acc:HGNC:29811] |
| ENSG00000188056 | HGLibA_51365 | 1 | -1.125648147 | TREML4 | triggering receptor expressed on myeloid cells like 4 [Source:HGNC Symbol;Acc:HGNC:30807] |
| ENSG00000188092 | HGLibA_20110 | 1 | -1.125648147 | GPR89B | G protein-coupled receptor 89B [Source:HGNC Symbol;Acc:HGNC:13840] |
| ENSG00000188100 | HGLibA_16553 | 1 | -1.125648147 | FAM25A | family with sequence similarity 25 member A [Source:HGNC Symbol;Acc:HGNC:23436] |
| ENSG00000188120 | HGLibA_12351 | 1 | -1.125648147 | DAZ1 | deleted in azoospermia 1 [Source:HGNC Symbol;Acc:HGNC:2682] |
| ENSG00000188130 | HGLibA_28257 | 1 | -1.125648147 | MAPK12 | mitogen-activated protein kinase 12 [Source:HGNC Symbol;Acc:HGNC:6874] |
| ENSG00000188162 | HGLibA_34728 | 1 | -1.125648147 | OTOG | otogelin [Source:HGNC Symbol;Acc:HGNC:8516] |
| ENSG00000188163 | HGLibA_16234 | 1 | -1.125648147 | FAM166A | family with sequence similarity 166 member A [Source:HGNC Symbol;Acc:HGNC:33818] |
| ENSG00000188191 | HGLibA_38523 | 1 | -1.125648147 | PRKAR1B | protein kinase cAMP-dependent type I regulatory subunit beta [Source:HGNC Symbol;Acc:HGNC:9390] |
| ENSG00000188269 | HGLibA_34462 | 1 | -1.125648147 | OR7A5 | olfactory receptor family 7 subfamily A member 5 [Source:HGNC Symbol;Acc:HGNC:8368] |
| ENSG00000188342 | HGLibA_20542 | 1 | -1.125648147 | GTF2F2 | general transcription factor IIF subunit 2 [Source:HGNC Symbol;Acc:HGNC:4653] |
| ENSG00000188558 | HGLibA_33801 | 1 | -1.125648147 | OR2G6 | olfactory receptor family 2 subfamily G member 6 [Source:HGNC Symbol;Acc:HGNC:27019] |
| ENSG00000188581 | HGLibA_25578 | 1 | -1.125648147 | KRTAP1-1 | keratin associated protein 1-1 [Source:HGNC Symbol;Acc:HGNC:16772] |
| ENSG00000188649 | HGLibA_07483 | 1 | -1.125648147 | CC2D2B | coiled-coil and C2 domain containing 2B [Source:HGNC Symbol;Acc:HGNC:31666] |
| ENSG00000188672 | HGLibA_41146 | 1 | -1.125648147 | RHCE | Rh blood group CcEe antigens [Source:HGNC Symbol;Acc:HGNC:10008] |
| ENSG00000188958 | HGLibA_53463 | 1 | -1.125648147 | UTS2B | urotensin 2B [Source:HGNC Symbol;Acc:HGNC:30894] |
| ENSG00000189079 | HGLibA_02888 | 1 | -1.125648147 | ARID2 | AT-rich interaction domain 2 [Source:HGNC Symbol;Acc:HGNC:18037] |
| ENSG00000189120 | HGLibA_46309 | 1 | -1.125648147 | SP6 | Sp6 transcription factor [Source:HGNC Symbol;Acc:HGNC:14530] |
| ENSG00000189129 | HGLibA_36949 | 1 | -1.125648147 | PLAC9 | placenta specific 9 [Source:HGNC Symbol;Acc:HGNC:19255] |
| ENSG00000189139 | HGLibA_18064 | 1 | -1.125648147 | FSCB | fibrous sheath CABYR binding protein [Source:HGNC Symbol;Acc:HGNC:20494] |
| ENSG00000189252 | HGLibA_46409 | 1 | -1.125648147 | SPANXN3 | SPANX family member N3 [Source:HGNC Symbol;Acc:HGNC:33176] |
| ENSG00000189298 | HGLibA_55351 | 1 | -1.125648147 | ZKSCAN3 | zinc finger with KRAB and SCAN domains 3 [Source:HGNC Symbol;Acc:HGNC:13853] |
| ENSG00000189306 | HGLibA_42282 | 1 | -1.125648147 | RRP7A | ribosomal RNA processing 7 homolog A [Source:HGNC Symbol;Acc:HGNC:24286] |
| ENSG00000196104 | HGLibA_46754 | 1 | -1.125648147 | SPOCK3 | cwcv and kazal like domains proteoglycan 3 [Source:HGNC Symbol;Acc:HGNC:13565]" |
| ENSG00000196154 | HGLibA_42558 | 1 | -1.125648147 | S100A4 | S100 calcium binding protein A4 [Source:HGNC Symbol;Acc:HGNC:10494] |
| ENSG00000196233 | HGLibA_26192 | 1 | -1.125648147 | LCOR | ligand dependent nuclear receptor corepressor [Source:HGNC Symbol;Acc:HGNC:29503] |
| ENSG00000196365 | HGLibA_27014 | 1 | -1.125648147 | LONP1 | mitochondrial [Source:HGNC Symbol;Acc:HGNC:9479]" |
| ENSG00000196372 | HGLibA_03214 | 1 | -1.125648147 | ASB13 | ankyrin repeat and SOCS box containing 13 [Source:HGNC Symbol;Acc:HGNC:19765] |
| ENSG00000196378 | HGLibA_55838 | 1 | -1.125648147 | ZNF34 | zinc finger protein 34 [Source:HGNC Symbol;Acc:HGNC:13098] |
| ENSG00000196387 | HGLibA_55484 | 1 | -1.125648147 | ZNF140 | zinc finger protein 140 [Source:HGNC Symbol;Acc:HGNC:12925] |
| ENSG00000196388 | HGLibA_23275 | 1 | -1.125648147 | INCA1 | cyclin A1 interacting protein 1 [Source:HGNC Symbol;Acc:HGNC:32224]" |
| ENSG00000196776 | HGLibA_08396 | 1 | -1.125648147 | CD47 | CD47 molecule [Source:HGNC Symbol;Acc:HGNC:1682] |
| ENSG00000196778 | HGLibA_34181 | 1 | -1.125648147 | OR52K1 | olfactory receptor family 52 subfamily K member 1 [Source:HGNC Symbol;Acc:HGNC:15222] |
| ENSG00000196792 | HGLibA_47597 | 1 | -1.125648147 | STRN3 | striatin 3 [Source:HGNC Symbol;Acc:HGNC:15720] |
| ENSG00000196793 | HGLibA_55654 | 1 | -1.125648147 | ZNF239 | zinc finger protein 239 [Source:HGNC Symbol;Acc:HGNC:13031] |
| ENSG00000196812 | HGLibA_56990 | 1 | -1.125648147 | ZSCAN16 | zinc finger and SCAN domain containing 16 [Source:HGNC Symbol;Acc:HGNC:20813] |
| ENSG00000196850 | HGLibA_38239 | 1 | -1.125648147 | PPTC7 | PTC7 protein phosphatase homolog [Source:HGNC Symbol;Acc:HGNC:30695] |
| ENSG00000196937 | HGLibA_16582 | 1 | -1.125648147 | FAM3C | family with sequence similarity 3 member C [Source:HGNC Symbol;Acc:HGNC:18664] |
| ENSG00000196990 | HGLibA_16230 | 1 | -1.125648147 | FAM163B | family with sequence similarity 163 member B [Source:HGNC Symbol;Acc:HGNC:33277] |
| ENSG00000197008 | HGLibA_55477 | 1 | -1.125648147 | ZNF138 | zinc finger protein 138 [Source:HGNC Symbol;Acc:HGNC:12922] |
| ENSG00000197056 | HGLibA_55390 | 1 | -1.125648147 | ZMYM1 | zinc finger MYM-type containing 1 [Source:HGNC Symbol;Acc:HGNC:26253] |
| ENSG00000197063 | HGLibA_27881 | 1 | -1.125648147 | MAFG | MAF bZIP transcription factor G [Source:HGNC Symbol;Acc:HGNC:6781] |
| ENSG00000197070 | HGLibA_03114 | 1 | -1.125648147 | ARRDC1 | arrestin domain containing 1 [Source:HGNC Symbol;Acc:HGNC:28633] |
| ENSG00000197142 | HGLibA_00564 | 1 | -1.125648147 | ACSL5 | acyl-CoA synthetase long chain family member 5 [Source:HGNC Symbol;Acc:HGNC:16526] |
| ENSG00000197241 | HGLibA_44889 | 1 | -1.125648147 | SLC2A7 | solute carrier family 2 member 7 [Source:HGNC Symbol;Acc:HGNC:13445] |
| ENSG00000197324 | HGLibA_27200 | 1 | -1.125648147 | LRP10 | LDL receptor related protein 10 [Source:HGNC Symbol;Acc:HGNC:14553] |
| ENSG00000197363 | HGLibA_56146 | 1 | -1.125648147 | ZNF517 | zinc finger protein 517 [Source:HGNC Symbol;Acc:HGNC:27984] |
| ENSG00000197565 | HGLibA_10585 | 1 | -1.125648147 | COL4A6 | collagen type IV alpha 6 chain [Source:HGNC Symbol;Acc:HGNC:2208] |
| ENSG00000197616 | HGLibA_30730 | 1 | -1.125648147 | MYH6 | myosin heavy chain 6 [Source:HGNC Symbol;Acc:HGNC:7576] |
| ENSG00000197646 | HGLibA_35724 | 1 | -1.125648147 | PDCD1LG2 | programmed cell death 1 ligand 2 [Source:HGNC Symbol;Acc:HGNC:18731] |
| ENSG00000197745 | HGLibA_42921 | 1 | -1.125648147 | SCGB1D4 | secretoglobin family 1D member 4 [Source:HGNC Symbol;Acc:HGNC:31748] |

| | | | | | |
|---|---|---|---|---|---|
| ENSG00000197786 | HGLibA_34259 | 1 | -1.125648147 | OR5B17 | olfactory receptor family 5 subfamily B member 17 [Source:HGNC Symbol;Acc:HGNC:15267] |
| ENSG00000197849 | HGLibA_34517 | 1 | -1.125648147 | OR8G1 | olfactory receptor family 8 subfamily G member 1 (gene/pseudogene) [Source:HGNC Symbol;Acc:HGNC:8484] |
| ENSG00000197978 | HGLibA_19630 | 1 | -1.125648147 | GOLGA6L9 | golgin A6 family-like 9 [Source:HGNC Symbol;Acc:HGNC:37229] |
| ENSG00000198001 | HGLibA_23566 | 1 | -1.125648147 | IRAK4 | interleukin 1 receptor associated kinase 4 [Source:HGNC Symbol;Acc:HGNC:17967] |
| ENSG00000198010 | HGLibA_13341 | 1 | -1.125648147 | DLGAP2 | DLG associated protein 2 [Source:HGNC Symbol;Acc:HGNC:2906] |
| ENSG00000198040 | HGLibA_56850 | 1 | -1.125648147 | ZNF84 | zinc finger protein 84 [Source:HGNC Symbol;Acc:HGNC:13159] |
| ENSG00000198083 | HGLibA_25850 | 1 | -1.125648147 | KRTAP9-9 | keratin associated protein 9-9 [Source:HGNC Symbol;Acc:HGNC:16773] |
| ENSG00000198168 | HGLibA_47888 | 1 | -1.125648147 | SVIP | small VCP interacting protein [Source:HGNC Symbol;Acc:HGNC:25238] |
| ENSG00000198326 | HGLibA_50281 | 1 | -1.125648147 | TMEM239 | transmembrane protein 239 [Source:HGNC Symbol;Acc:HGNC:40044] |
| ENSG00000198466 | HGLibA_56328 | 1 | -1.125648147 | ZNF587 | zinc finger protein 587 [Source:HGNC Symbol;Acc:HGNC:30955] |
| ENSG00000198553 | HGLibA_24380 | 1 | -1.125648147 | KCNRG | potassium channel regulator [Source:HGNC Symbol;Acc:HGNC:18893] |
| ENSG00000198569 | HGLibA_44943 | 1 | -1.125648147 | SLC34A3 | solute carrier family 34 member 3 [Source:HGNC Symbol;Acc:HGNC:20305] |
| ENSG00000198585 | HGLibA_32951 | 1 | -1.125648147 | NUDT16 | nudix hydrolase 16 [Source:HGNC Symbol;Acc:HGNC:26442] |
| ENSG00000198590 | HGLibA_06234 | 1 | -1.125648147 | C3orf35 | chromosome 3 open reading frame 35 [Source:HGNC Symbol;Acc:HGNC:24082] |
| ENSG00000198643 | HGLibA_16583 | 1 | -1.125648147 | FAM3D | family with sequence similarity 3 member D [Source:HGNC Symbol;Acc:HGNC:18665] |
| ENSG00000198742 | HGLibA_45844 | 1 | -1.125648147 | SMURF1 | SMAD specific E3 ubiquitin protein ligase 1 [Source:HGNC Symbol;Acc:HGNC:16807] |
| ENSG00000198898 | HGLibA_07209 | 1 | -1.125648147 | CAPZA2 | capping actin protein of muscle Z-line subunit alpha 2 [Source:HGNC Symbol;Acc:HGNC:1490] |
| ENSG00000198910 | HGLibA_25883 | 1 | -1.125648147 | L1CAM | L1 cell adhesion molecule [Source:HGNC Symbol;Acc:HGNC:6470] |
| ENSG00000203668 | HGLibA_09470 | 1 | -1.125648147 | CHML | Rab escort protein 2 [Source:HGNC Symbol;Acc:HGNC:1941]" |
| ENSG00000203778 | HGLibA_16544 | 1 | -1.125648147 | FAM229B | family with sequence similarity 229 member B [Source:HGNC Symbol;Acc:HGNC:33858] |
| ENSG00000203972 | HGLibA_19379 | 1 | -1.125648147 | GLYATL3 | glycine-N-acyltransferase like 3 [Source:HGNC Symbol;Acc:HGNC:21349] |
| ENSG00000204086 | HGLibA_41808 | 1 | -1.125648147 | RPA4 | replication protein A4 [Source:HGNC Symbol;Acc:HGNC:30305] |
| ENSG00000204335 | HGLibA_46307 | 1 | -1.125648147 | SP5 | Sp5 transcription factor [Source:HGNC Symbol;Acc:HGNC:14529] |
| ENSG00000204385 | HGLibA_45165 | 1 | -1.125648147 | SLC44A4 | solute carrier family 44 member 4 [Source:HGNC Symbol;Acc:HGNC:13941] |
| ENSG00000204498 | HGLibA_31832 | 1 | -1.125648147 | NFKBIL1 | NFKB inhibitor like 1 [Source:HGNC Symbol;Acc:HGNC:7800] |
| ENSG00000204524 | HGLibA_56793 | 1 | -1.125648147 | ZNF805 | zinc finger protein 805 [Source:HGNC Symbol;Acc:HGNC:23272] |
| ENSG00000204536 | HGLibA_07945 | 1 | -1.125648147 | CCHCR1 | coiled-coil alpha-helical rod protein 1 [Source:HGNC Symbol;Acc:HGNC:13930] |
| ENSG00000204580 | HGLibA_12634 | 1 | -1.125648147 | DDR1 | discoidin domain receptor tyrosine kinase 1 [Source:HGNC Symbol;Acc:HGNC:2730] |
| ENSG00000204644 | HGLibA_55221 | 1 | -1.125648147 | ZFP57 | ZFP57 zinc finger protein [Source:HGNC Symbol;Acc:HGNC:18791] |
| ENSG00000204687 | HGLibA_28390 | 1 | -1.125648147 | MAS1L | G protein-coupled receptor [Source:HGNC Symbol;Acc:HGNC:13961]" |
| ENSG00000204843 | HGLibA_12543 | 1 | -1.125648147 | DCTN1 | dynactin subunit 1 [Source:HGNC Symbol;Acc:HGNC:2711] |
| ENSG00000204856 | HGLibA_16484 | 1 | -1.125648147 | FAM216A | family with sequence similarity 216 member A [Source:HGNC Symbol;Acc:HGNC:30180] |
| ENSG00000204965 | HGLibA_35447 | 1 | -1.125648147 | PCDHA5 | protocadherin alpha 5 [Source:HGNC Symbol;Acc:HGNC:8671] |
| ENSG00000204991 | HGLibA_46726 | 1 | -1.125648147 | SPIRE2 | spire type actin nucleation factor 2 [Source:HGNC Symbol;Acc:HGNC:30623] |
| ENSG00000205126 | HGLibA_00415 | 1 | -1.125648147 | ACCSL | 1-aminocyclopropane-1-carboxylate synthase homolog (inactive) like [Source:HGNC Symbol;Acc:HGNC:34391] |
| ENSG00000205189 | HGLibA_54814 | 1 | -1.125648147 | ZBTB10 | zinc finger and BTB domain containing 10 [Source:HGNC Symbol;Acc:HGNC:30953] |
| ENSG00000205208 | HGLibA_06335 | 1 | -1.125648147 | C4orf46 | chromosome 4 open reading frame 46 [Source:HGNC Symbol;Acc:HGNC:27320] |
| ENSG00000205268 | HGLibA_35824 | 1 | -1.125648147 | PDE7A | phosphodiesterase 7A [Source:HGNC Symbol;Acc:HGNC:8791] |
| ENSG00000205339 | HGLibA_23471 | 1 | -1.125648147 | IPO7 | importin 7 [Source:HGNC Symbol;Acc:HGNC:9852] |
| ENSG00000205436 | HGLibA_15745 | 1 | -1.125648147 | EXOC3L4 | exocyst complex component 3 like 4 [Source:HGNC Symbol;Acc:HGNC:20120] |
| ENSG00000205531 | HGLibA_31121 | 1 | -1.125648147 | NAP1L4 | nucleosome assembly protein 1 like 4 [Source:HGNC Symbol;Acc:HGNC:7640] |
| ENSG00000205609 | HGLibA_14770 | 1 | -1.125648147 | EIF3CL | eukaryotic translation initiation factor 3 subunit C like [Source:HGNC Symbol;Acc:HGNC:26347] |
| ENSG00000205683 | HGLibA_13833 | 1 | -1.125648147 | DPF3 | double PHD fingers 3 [Source:HGNC Symbol;Acc:HGNC:17427] |
| ENSG00000205884 | HGLibA_12911 | 1 | -1.125648147 | DEFB136 | defensin beta 136 [Source:HGNC Symbol;Acc:HGNC:34433] |
| ENSG00000206026 | HGLibA_45749 | 1 | -1.125648147 | SMIM21 | small integral membrane protein 21 [Source:HGNC Symbol;Acc:HGNC:27598] |
| ENSG00000206432 | HGLibA_50176 | 1 | -1.125648147 | TMEM200C | transmembrane protein 200C [Source:HGNC Symbol;Acc:HGNC:37208] |
| ENSG00000206557 | HGLibA_51601 | 1 | -1.125648147 | TRIM71 | tripartite motif containing 71 [Source:HGNC Symbol;Acc:HGNC:32669] |
| ENSG00000206557 | HGLibA_51599 | 1 | -1.125648147 | TRIM71 | tripartite motif containing 71 [Source:HGNC Symbol;Acc:HGNC:32669] |
| ENSG00000211452 | HGLibA_13238 | 1 | -1.125648147 | DIO1 | iodothyronine deiodinase 1 [Source:HGNC Symbol;Acc:HGNC:2883] |
| ENSG00000212124 | HGLibA_48377 | 1 | -1.125648147 | TAS2R19 | taste 2 receptor member 19 [Source:HGNC Symbol;Acc:HGNC:19108] |
| ENSG00000212658 | HGLibA_25738 | 1 | -1.125648147 | KRTAP29-1 | keratin associated protein 29-1 [Source:HGNC Symbol;Acc:HGNC:34211] |
| ENSG00000212722 | HGLibA_25779 | 1 | -1.125648147 | KRTAP4-9 | keratin associated protein 4-9 [Source:HGNC Symbol;Acc:HGNC:18910] |
| ENSG00000212900 | HGLibA_25743 | 1 | -1.125648147 | KRTAP3-2 | keratin associated protein 3-2 [Source:HGNC Symbol;Acc:HGNC:16779] |
| ENSG00000213416 | HGLibA_25756 | 1 | -1.125648147 | KRTAP4-12 | keratin associated protein 4-12 [Source:HGNC Symbol;Acc:HGNC:16776] |
| ENSG00000214022 | HGLibA_40846 | 1 | -1.125648147 | REPIN1 | replication initiator 1 [Source:HGNC Symbol;Acc:HGNC:17922] |
| ENSG00000214114 | HGLibA_30668 | 1 | -1.125648147 | MYCBP | MYC binding protein [Source:HGNC Symbol;Acc:HGNC:7554] |
| ENSG00000214140 | HGLibA_38356 | 1 | -1.125648147 | PRCD | photoreceptor disc component [Source:HGNC Symbol;Acc:HGNC:32528] |
| ENSG00000214290 | HGLibA_10628 | 1 | -1.125648147 | COLCA2 | colorectal cancer associated 2 [Source:HGNC Symbol;Acc:HGNC:26978] |
| ENSG00000215217 | HGLibA_06399 | 1 | -1.125648147 | C5orf49 | chromosome 5 open reading frame 49 [Source:HGNC Symbol;Acc:HGNC:27028] |
| ENSG00000215274 | HGLibA_18434 | 1 | -1.125648147 | GAGE10 | G antigen 10 [Source:HGNC Symbol;Acc:HGNC:30968] |
| ENSG00000215301 | HGLibA_12701 | 1 | -1.125648147 | DDX3X | DEAD-box helicase 3 X-linked [Source:HGNC Symbol;Acc:HGNC:2745] |
| ENSG00000215343 | HGLibA_56593 | 1 | -1.125648147 | ZNF705D | zinc finger protein 705D [Source:HGNC Symbol;Acc:HGNC:33202] |
| ENSG00000217930 | HGLibA_35086 | 1 | -1.125648147 | PAM16 | presequence translocase associated motor 16 [Source:HGNC Symbol;Acc:HGNC:29679] |
| ENSG00000220201 | HGLibA_55304 | 1 | -1.125648147 | ZGLP1 | GATA-like protein 1 [Source:HGNC Symbol;Acc:HGNC:37245]" |
| ENSG00000220205 | HGLibA_53490 | 1 | -1.125648147 | VAMP2 | vesicle associated membrane protein 2 [Source:HGNC Symbol;Acc:HGNC:12643] |
| ENSG00000221821 | HGLibA_06482 | 1 | -1.125648147 | C6orf226 | chromosome 6 open reading frame 226 [Source:HGNC Symbol;Acc:HGNC:34431] |
| ENSG00000221837 | HGLibA_25625 | 1 | -1.125648147 | KRTAP10-9 | keratin associated protein 10-9 [Source:HGNC Symbol;Acc:HGNC:22971] |
| ENSG00000221867 | HGLibA_27912 | 1 | -1.125648147 | MAGEA3 | MAGE family member A3 [Source:HGNC Symbol;Acc:HGNC:6801] |
| ENSG00000221900 | HGLibA_37649 | 1 | -1.125648147 | POM121L12 | POM121 transmembrane nucleoporin like 12 [Source:HGNC Symbol;Acc:HGNC:25369] |
| ENSG00000221947 | HGLibA_54534 | 1 | -1.125648147 | XKR9 | XK related 9 [Source:HGNC Symbol;Acc:HGNC:20937] |
| ENSG00000221977 | HGLibA_33985 | 1 | -1.125648147 | OR4E2 | olfactory receptor family 4 subfamily E member 2 [Source:HGNC Symbol;Acc:HGNC:8297] |
| ENSG00000223572 | HGLibA_09778 | 1 | -1.125648147 | CKMT1A | mitochondrial 1A [Source:HGNC Symbol;Acc:HGNC:31736]" |
| ENSG00000224089 | HGLibA_11520 | 1 | -1.125648147 | CT47A10 | member A10 [Source:HGNC Symbol;Acc:HGNC:33291]" |
| ENSG00000225766 | HGLibA_13139 | 1 | -1.125648147 | DHRS4L1 | dehydrogenase/reductase 4 like 1 [Source:HGNC Symbol;Acc:HGNC:19732] |
| ENSG00000225950 | HGLibA_32841 | 1 | -1.125648147 | NTF4 | neurotrophin 4 [Source:HGNC Symbol;Acc:HGNC:8024] |
| ENSG00000226023 | HGLibA_11541 | 1 | -1.125648147 | CT47A6 | member A6 [Source:HGNC Symbol;Acc:HGNC:33287]" |
| ENSG00000226685 | HGLibA_11526 | 1 | -1.125648147 | CT47A12 | member A12 [Source:HGNC Symbol;Acc:HGNC:33292]" |
| ENSG00000226929 | HGLibA_11523 | 1 | -1.125648147 | CT47A11 | member A11 [Source:HGNC Symbol;Acc:HGNC:27397]" |
| ENSG00000228517 | HGLibA_11544 | 1 | -1.125648147 | CT47A7 | member A7 [Source:HGNC Symbol;Acc:HGNC:33288]" |
| ENSG00000228696 | HGLibA_02953 | 1 | -1.125648147 | ARL17B | ADP ribosylation factor like GTPase 17B [Source:HGNC Symbol;Acc:HGNC:32387] |
| ENSG00000228716 | HGLibA_13104 | 1 | -1.125648147 | DHFR | dihydrofolate reductase [Source:HGNC Symbol;Acc:HGNC:2861] |
| ENSG00000229544 | HGLibA_32029 | 1 | -1.125648147 | NKX1-2 | NK1 homeobox 2 [Source:HGNC Symbol;Acc:HGNC:31652] |
| ENSG00000230430 | HGLibA_53256 | 1 | -1.125648147 | USP17L25 | ubiquitin specific peptidase 17-like family member 25 [Source:HGNC Symbol;Acc:HGNC:44452] |
| ENSG00000230594 | HGLibA_11535 | 1 | -1.125648147 | CT47A4 | member A4 [Source:HGNC Symbol;Acc:HGNC:33285]" |
| ENSG00000231192 | HGLibA_34289 | 1 | -1.125648147 | OR5H1 | olfactory receptor family 5 subfamily H member 1 [Source:HGNC Symbol;Acc:HGNC:8346] |
| ENSG00000232264 | HGLibA_53253 | 1 | -1.125648147 | USP17L24 | ubiquitin specific peptidase 17-like family member 24 [Source:HGNC Symbol;Acc:HGNC:44453] |
| ENSG00000232399 | HGLibA_53224 | 1 | -1.125648147 | USP17L13 | ubiquitin specific peptidase 17-like family member 13 [Source:HGNC Symbol;Acc:HGNC:44441] |
| ENSG00000232629 | HGLibA_21630 | 1 | -1.125648147 | HLA-DQB2 | DQ beta 2 [Source:HGNC Symbol;Acc:HGNC:4945]" |
| ENSG00000234465 | HGLibA_36694 | 1 | -1.125648147 | PINLYP | phospholipase A2 inhibitor and LY6/PLAUR domain containing [Source:HGNC Symbol;Acc:HGNC:44206] |
| ENSG00000235109 | HGLibA_57019 | 1 | -1.125648147 | ZSCAN31 | zinc finger and SCAN domain containing 31 [Source:HGNC Symbol;Acc:HGNC:14097] |
| ENSG00000235162 | HGLibA_05297 | 1 | -1.125648147 | C12orf75 | chromosome 12 open reading frame 75 [Source:HGNC Symbol;Acc:HGNC:35164] |
| ENSG00000235568 | HGLibA_31765 | 1 | -1.125648147 | NFAM1 | NFAT activating protein with ITAM motif 1 [Source:HGNC Symbol;Acc:HGNC:29872] |
| ENSG00000235631 | HGLibA_41537 | 1 | -1.125648147 | RNF148 | ring finger protein 148 [Source:HGNC Symbol;Acc:HGNC:22411] |
| ENSG00000235699 | HGLibA_11940 | 1 | -1.125648147 | CXorf51B | chromosome X open reading frame 51B [Source:HGNC Symbol;Acc:HGNC:42787] |
| ENSG00000235961 | HGLibA_37378 | 1 | -1.125648147 | PNMA6A | PNMA family member 6A [Source:HGNC Symbol;Acc:HGNC:28248] |
| ENSG00000236126 | HGLibA_11532 | 1 | -1.125648147 | CT47A3 | member A3 [Source:HGNC Symbol;Acc:HGNC:33284]" |
| ENSG00000236371 | HGLibA_11517 | 1 | -1.125648147 | CT47A1 | member A1 [Source:HGNC Symbol;Acc:HGNC:33282]" |
| ENSG00000236446 | HGLibA_11553 | 1 | -1.125648147 | CT47B1 | member B1 [Source:HGNC Symbol;Acc:HGNC:33293]" |
| ENSG00000238083 | HGLibA_27329 | 1 | -1.125648147 | LRRC37A2 | leucine rich repeat containing 37 member A2 [Source:HGNC Symbol;Acc:HGNC:32404] |
| ENSG00000239887 | HGLibA_05834 | 1 | -1.125648147 | C1orf226 | chromosome 1 open reading frame 226 [Source:HGNC Symbol;Acc:HGNC:34351] |
| ENSG00000240184 | HGLibA_35570 | 1 | -1.125648147 | PCDHGC3 | 3 [Source:HGNC Symbol;Acc:HGNC:8716]" |
| ENSG00000240386 | HGLibA_26115 | 1 | -1.125648147 | LCE1F | late cornified envelope 1F [Source:HGNC Symbol;Acc:HGNC:29467] |
| ENSG00000240403 | HGLibA_24995 | 1 | -1.125648147 | KIR3DL2 | three Ig domains and long cytoplasmic tail 2 [Source:HGNC Symbol;Acc:HGNC:6339]" |
| ENSG00000241697 | HGLibA_49832 | 1 | -1.125648147 | TMEFF1 | transmembrane protein with EGF like and two follistatin like domains 1 [Source:HGNC Symbol;Acc:HGNC:11866] |
| ENSG00000241878 | HGLibA_36735 | 1 | -1.125648147 | PISD | phosphatidylserine decarboxylase [Source:HGNC Symbol;Acc:HGNC:8999] |
| ENSG00000242259 | HGLibA_06074 | 1 | -1.125648147 | C22orf39 | chromosome 22 open reading frame 39 [Source:HGNC Symbol;Acc:HGNC:27012] |
| ENSG00000242866 | HGLibA_47584 | 1 | -1.125648147 | STRC | stereocilin [Source:HGNC Symbol;Acc:HGNC:16035] |
| ENSG00000243130 | HGLibA_39038 | 1 | -1.125648147 | PSG11 | pregnancy specific beta-1-glycoprotein 11 [Source:HGNC Symbol;Acc:HGNC:9516] |
| ENSG00000243449 | HGLibA_06340 | 1 | -1.125648147 | C4orf48 | chromosome 4 open reading frame 48 [Source:HGNC Symbol;Acc:HGNC:34437] |
| ENSG00000243566 | HGLibA_53079 | 1 | -1.125648147 | UPK3B | uroplakin 3B [Source:HGNC Symbol;Acc:HGNC:21444] |
| ENSG00000243729 | HGLibA_34364 | 1 | -1.125648147 | OR5V1 | olfactory receptor family 5 subfamily V member 1 [Source:HGNC Symbol;Acc:HGNC:13972] |
| ENSG00000243789 | HGLibA_23961 | 1 | -1.125648147 | JMJD7 | jumonji domain containing 7 [Source:HGNC Symbol;Acc:HGNC:34397] |
| ENSG00000244537 | HGLibA_25758 | 1 | -1.125648147 | KRTAP4-2 | keratin associated protein 4-2 [Source:HGNC Symbol;Acc:HGNC:18900] |
| ENSG00000249104 | HGLibA_53230 | 1 | -1.125648147 | USP17L17 | ubiquitin specific peptidase 17-like family member 17 [Source:HGNC Symbol;Acc:HGNC:44445] |
| ENSG00000249104 | HGLibA_53229 | 1 | -1.125648147 | USP17L17 | ubiquitin specific peptidase 17-like family member 17 [Source:HGNC Symbol;Acc:HGNC:44445] |
| ENSG00000249811 | HGLibA_53247 | 1 | -1.125648147 | USP17L21 | ubiquitin specific peptidase 17-like family member 21 [Source:HGNC Symbol;Acc:HGNC:44449] |
| ENSG00000250745 | HGLibA_53245 | 1 | -1.125648147 | USP17L20 | ubiquitin specific peptidase 17-like family member 20 [Source:HGNC Symbol;Acc:HGNC:44448] |
| ENSG00000251192 | HGLibA_56509 | 1 | -1.125648147 | ZNF674 | zinc finger protein 674 [Source:HGNC Symbol;Acc:HGNC:17625] |
| ENSG00000254004 | HGLibA_55690 | 1 | -1.125648147 | ZNF260 | zinc finger protein 260 [Source:HGNC Symbol;Acc:HGNC:13499] |
| ENSG00000254827 | HGLibA_44563 | 1 | -1.125648147 | SLC22A18AS | solute carrier family 22 member 18 antisense [Source:HGNC Symbol;Acc:HGNC:10965] |
| ENSG00000254986 | HGLibA_13868 | 1 | -1.125648147 | DPP3 | dipeptidyl peptidase 3 [Source:HGNC Symbol;Acc:HGNC:3008] |
| ENSG00000255112 | HGLibA_09474 | 1 | -1.125648147 | CHMP1B | charged multivesicular body protein 1B [Source:HGNC Symbol;Acc:HGNC:24287] |
| ENSG00000255223 | HGLibA_34334 | 1 | -1.125648147 | OR5M11 | olfactory receptor family 5 subfamily M member 11 [Source:HGNC Symbol;Acc:HGNC:15291] |
| ENSG00000255245 | HGLibA_18241 | 1 | -1.125648147 | FXYD6-FXYD2 | FXYD6-FXYD2 readthrough [Source:HGNC Symbol;Acc:HGNC:39978] |
| ENSG00000255298 | HGLibA_34524 | 1 | -1.125648147 | OR8G5 | olfactory receptor family 8 subfamily G member 5 [Source:HGNC Symbol;Acc:HGNC:19622] |
| ENSG00000256045 | HGLibA_30473 | 1 | -1.125648147 | MTRNR2L10 | MT-RNR2 like 10 [Source:HGNC Symbol;Acc:HGNC:37167] |
| ENSG00000257008 | HGLibA_19913 | 1 | -1.125648147 | GPR142 | G protein-coupled receptor 142 [Source:HGNC Symbol;Acc:HGNC:20088] |
| ENSG00000259207 | HGLibA_23781 | 1 | -1.125648147 | ITGB3 | integrin subunit beta 3 [Source:HGNC Symbol;Acc:HGNC:6156] |
| ENSG00000260916 | HGLibA_08141 | 1 | -1.125648147 | CCPG1 | cell cycle progression 1 [Source:HGNC Symbol;Acc:HGNC:24227] |
| ENSG00000261115 | HGLibA_50085 | 1 | -1.125648147 | TMEM178B | transmembrane protein 178B [Source:HGNC Symbol;Acc:HGNC:44112] |
| ENSG00000265203 | HGLibA_40635 | 1 | -1.125648147 | RBP3 | retinol binding protein 3 [Source:HGNC Symbol;Acc:HGNC:9921] |
| ENSG00000267508 | HGLibA_55748 | 1 | -1.125648147 | ZNF285 | zinc finger protein 285 [Source:HGNC Symbol;Acc:HGNC:13079] |
| ENSG00000268089 | HGLibA_18400 | 1 | -1.125648147 | GABRQ | gamma-aminobutyric acid type A receptor theta subunit [Source:HGNC Symbol;Acc:HGNC:14454] |
| ENSG00000268350 | HGLibA_16187 | 1 | -1.125648147 | FAM156A | family with sequence similarity 156 member A [Source:HGNC Symbol;Acc:HGNC:30114] |
| ENSG00000269335 | HGLibA_22927 | 1 | -1.125648147 | IKBKG | inhibitor of nuclear factor kappa B kinase subunit gamma [Source:HGNC Symbol;Acc:HGNC:5961] |

| | | | | | |
|---|---|---|---|---|---|
| ENSG00000271043 | HGLibA_30477 | 1 | -1.125648147 | MTRNR2L2 | MT-RNR2 like 2 [Source:HGNC Symbol;Acc:HGNC:37156] |
| ENSG00000271447 | HGLibA_29532 | 1 | -1.125648147 | MMP28 | matrix metallopeptidase 28 [Source:HGNC Symbol;Acc:HGNC:14366] |
| ENSG00000271567 | HGLibA_37908 | 1 | -1.125648147 | PPIAL4E | peptidylprolyl isomerase A like 4E [Source:HGNC Symbol;Acc:HGNC:33997] |
| ENSG00000272968 | HGLibA_40425 | 1 | -1.125648147 | RBAK-RBAKDN | RBAK-RBAKDN readthrough [Source:HGNC Symbol;Acc:HGNC:42971] |
| ENSG00000273173 | HGLibA_46018 | 1 | -1.125648147 | SNURF | SNRPN upstream reading frame [Source:HGNC Symbol;Acc:HGNC:11171] |
| ENSG00000274226 | HGLibA_48555 | 1 | -1.125648147 | TBC1D3H | TBC1 domain family member 3H [Source:HGNC Symbol;Acc:HGNC:30708] |
| ENSG00000274286 | HGLibA_01050 | 1 | -1.125648147 | ADRA2B | adrenoceptor alpha 2B [Source:HGNC Symbol;Acc:HGNC:282] |
| ENSG00000275066 | HGLibA_48030 | 1 | -1.125648147 | SYNRG | synergin gamma [Source:HGNC Symbol;Acc:HGNC:557] |
| ENSG00000275832 | HGLibA_02731 | 1 | -1.125648147 | ARHGAP23 | Rho GTPase activating protein 23 [Source:HGNC Symbol;Acc:HGNC:29293] |
| ENSG00000276070 | HGLibA_08033 | 1 | -1.125648147 | CCL4L2 | C-C motif chemokine ligand 4 like 2 [Source:HGNC Symbol;Acc:HGNC:24066] |
| ENSG00000276234 | HGLibA_48185 | 1 | -1.125648147 | TADA2A | transcriptional adaptor 2A [Source:HGNC Symbol;Acc:HGNC:11531] |
| ENSG00000278870 | HGLibA_34109 | 1 | -1.125648147 | OR51G1 | olfactory receptor family 51 subfamily G member 1 (gene/pseudogene) [Source:HGNC Symbol;Acc:HGNC:14738] |
| ENSG00000278870 | HGLibA_34110 | 1 | -1.125648147 | OR51G1 | olfactory receptor family 51 subfamily G member 1 (gene/pseudogene) [Source:HGNC Symbol;Acc:HGNC:14738] |
| ENSG00000280094 | HGLibA_33648 | 1 | -1.125648147 | OR1B1 | olfactory receptor family 1 subfamily B member 1 (gene/pseudogene) [Source:HGNC Symbol;Acc:HGNC:8181] |
| ENSG00000280094 | HGLibA_33647 | 1 | -1.125648147 | OR1B1 | olfactory receptor family 1 subfamily B member 1 (gene/pseudogene) [Source:HGNC Symbol;Acc:HGNC:8181] |
| ENSG00000280969 | HGLibA_42148 | 1 | -1.125648147 | RPS4Y2 | ribosomal protein S4 Y-linked 2 [Source:HGNC Symbol;Acc:HGNC:18501] |
| ENSG00000283632 | HGLibA_15744 | 1 | -1.125648147 | EXOC3L2 | exocyst complex component 3 like 2 [Source:HGNC Symbol;Acc:HGNC:30162] |
| ENSG00000288784 | HGLibA_11573 | 1 | -1.125648147 | CTAGE4 | - |
| ERO1LB | HGLibA_15520 | 1 | -1.125648147 | ERO1LB | - |
| FAM103A1 | HGLibA_15985 | 1 | -1.125648147 | FAM103A1 | - |
| FAM105B | HGLibA_15997 | 1 | -1.125648147 | FAM105B | - |
| FAM127C | HGLibA_16093 | 1 | -1.125648147 | FAM127C | - |
| FAM132A | HGLibA_16115 | 1 | -1.125648147 | FAM132A | - |
| FAM160A1 | HGLibA_16205 | 1 | -1.125648147 | FAM160A1 | - |
| FAM160A2 | HGLibA_16206 | 1 | -1.125648147 | FAM160A2 | - |
| FAM175A | HGLibA_16287 | 1 | -1.125648147 | FAM175A | - |
| FAM178A | HGLibA_16300 | 1 | -1.125648147 | FAM178A | - |
| FAM179B | HGLibA_16308 | 1 | -1.125648147 | FAM179B | - |
| FAM207A | HGLibA_16429 | 1 | -1.125648147 | FAM207A | - |
| FAM64A | HGLibA_16666 | 1 | -1.125648147 | FAM64A | - |
| FAM69A | HGLibA_16677 | 1 | -1.125648147 | FAM69A | - |
| FAM96A | HGLibA_16802 | 1 | -1.125648147 | FAM96A | - |
| FGFR1OP | HGLibA_17439 | 1 | -1.125648147 | FGFR1OP | - |
| FIGF | HGLibA_17514 | 1 | -1.125648147 | FIGF | - |
| G6PC | HGLibA_18305 | 1 | -1.125648147 | G6PC | - |
| H1FOO | HGLibA_20739 | 1 | -1.125648147 | H1FOO | - |
| H2BFM | HGLibA_20773 | 1 | -1.125648147 | H2BFM | - |
| HIST1H2AI | HGLibA_21395 | 1 | -1.125648147 | HIST1H2AI | - |
| HIST1H2BH | HGLibA_21433 | 1 | -1.125648147 | HIST1H2BH | - |
| HIST1H3E | HGLibA_21469 | 1 | -1.125648147 | HIST1H3E | - |
| HIST1H4I | HGLibA_21509 | 1 | -1.125648147 | HIST1H4I | - |
| HKR1 | HGLibA_21591 | 1 | -1.125648147 | HKR1 | - |
| HRASLS | HGLibA_22075 | 1 | -1.125648147 | HRASLS | - |
| hsa-let-7e | HGLibA_57123 | 1 | -1.125648147 | hsa-let-7e | - |
| hsa-mir-100 | HGLibA_57150 | 1 | -1.125648147 | hsa-mir-100 | - |
| hsa-mir-100 | HGLibA_57149 | 1 | -1.125648147 | hsa-mir-100 | - |
| hsa-mir-1184-2 | HGLibA_57233 | 1 | -1.125648147 | hsa-mir-1184-2 | - |
| hsa-mir-1185-2 | HGLibA_57245 | 1 | -1.125648147 | hsa-mir-1185-2 | - |
| hsa-mir-122 | HGLibA_57289 | 1 | -1.125648147 | hsa-mir-122 | - |
| hsa-mir-124-1 | HGLibA_57346 | 1 | -1.125648147 | hsa-mir-124-1 | - |
| hsa-mir-1250 | HGLibA_57399 | 1 | -1.125648147 | hsa-mir-1250 | - |
| hsa-mir-1254-2 | HGLibA_57416 | 1 | -1.125648147 | hsa-mir-1254-2 | - |
| hsa-mir-1256 | HGLibA_57432 | 1 | -1.125648147 | hsa-mir-1256 | - |
| hsa-mir-1263 | HGLibA_57474 | 1 | -1.125648147 | hsa-mir-1263 | - |
| hsa-mir-1270-1 | HGLibA_57516 | 1 | -1.125648147 | hsa-mir-1270-1 | - |
| hsa-mir-1270-2 | HGLibA_57520 | 1 | -1.125648147 | hsa-mir-1270-2 | - |
| hsa-mir-1277 | HGLibA_57563 | 1 | -1.125648147 | hsa-mir-1277 | - |
| hsa-mir-1284 | HGLibA_57597 | 1 | -1.125648147 | hsa-mir-1284 | - |
| hsa-mir-1285-1 | HGLibA_57602 | 1 | -1.125648147 | hsa-mir-1285-1 | - |
| hsa-mir-1290 | HGLibA_57637 | 1 | -1.125648147 | hsa-mir-1290 | - |
| hsa-mir-1291 | HGLibA_57640 | 1 | -1.125648147 | hsa-mir-1291 | - |
| hsa-mir-145 | HGLibA_57844 | 1 | -1.125648147 | hsa-mir-145 | - |
| hsa-mir-148b | HGLibA_57885 | 1 | -1.125648147 | hsa-mir-148b | - |
| hsa-mir-184 | HGLibA_57997 | 1 | -1.125648147 | hsa-mir-184 | - |
| hsa-mir-1910 | HGLibA_58046 | 1 | -1.125648147 | hsa-mir-1910 | - |
| hsa-mir-19b-1 | HGLibA_58144 | 1 | -1.125648147 | hsa-mir-19b-1 | - |
| hsa-mir-203b | HGLibA_58170 | 1 | -1.125648147 | hsa-mir-203b | - |
| hsa-mir-20b | HGLibA_58204 | 1 | -1.125648147 | hsa-mir-20b | - |
| hsa-mir-211 | HGLibA_58215 | 1 | -1.125648147 | hsa-mir-211 | - |
| hsa-mir-2114 | HGLibA_58228 | 1 | -1.125648147 | hsa-mir-2114 | - |
| hsa-mir-218-1 | HGLibA_58269 | 1 | -1.125648147 | hsa-mir-218-1 | - |
| hsa-mir-219b | HGLibA_58283 | 1 | -1.125648147 | hsa-mir-219b | - |
| hsa-mir-2355 | HGLibA_58321 | 1 | -1.125648147 | hsa-mir-2355 | - |
| hsa-mir-25 | HGLibA_58351 | 1 | -1.125648147 | hsa-mir-25 | - |
| hsa-mir-26b | HGLibA_58374 | 1 | -1.125648147 | hsa-mir-26b | - |
| hsa-mir-30c-2 | HGLibA_58485 | 1 | -1.125648147 | hsa-mir-30c-2 | - |
| hsa-mir-3115 | HGLibA_58499 | 1 | -1.125648147 | hsa-mir-3115 | - |
| hsa-mir-3134 | HGLibA_58592 | 1 | -1.125648147 | hsa-mir-3134 | - |
| hsa-mir-3150b | HGLibA_58659 | 1 | -1.125648147 | hsa-mir-3150b | - |
| hsa-mir-3160-1 | HGLibA_58717 | 1 | -1.125648147 | hsa-mir-3160-1 | - |
| hsa-mir-3160-2 | HGLibA_58721 | 1 | -1.125648147 | hsa-mir-3160-2 | - |
| hsa-mir-3184 | HGLibA_58832 | 1 | -1.125648147 | hsa-mir-3184 | - |
| hsa-mir-3186 | HGLibA_58840 | 1 | -1.125648147 | hsa-mir-3186 | - |
| hsa-mir-3192 | HGLibA_58866 | 1 | -1.125648147 | hsa-mir-3192 | - |
| hsa-mir-320c-2 | HGLibA_58941 | 1 | -1.125648147 | hsa-mir-320c-2 | - |
| hsa-mir-324 | HGLibA_58964 | 1 | -1.125648147 | hsa-mir-324 | - |
| hsa-mir-3654 | HGLibA_59161 | 1 | -1.125648147 | hsa-mir-3654 | - |
| hsa-mir-3656 | HGLibA_59171 | 1 | -1.125648147 | hsa-mir-3656 | - |
| hsa-mir-3664 | HGLibA_59209 | 1 | -1.125648147 | hsa-mir-3664 | - |
| hsa-mir-3689d-1 | HGLibA_59325 | 1 | -1.125648147 | hsa-mir-3689d-1 | - |
| hsa-mir-3714 | HGLibA_59370 | 1 | -1.125648147 | hsa-mir-3714 | - |
| hsa-mir-371a | HGLibA_59372 | 1 | -1.125648147 | hsa-mir-371a | - |
| hsa-mir-374b | HGLibA_59392 | 1 | -1.125648147 | hsa-mir-374b | - |
| hsa-mir-376c | HGLibA_59419 | 1 | -1.125648147 | hsa-mir-376c | - |
| hsa-mir-376c | HGLibA_59417 | 1 | -1.125648147 | hsa-mir-376c | - |
| hsa-mir-378j | HGLibA_59467 | 1 | -1.125648147 | hsa-mir-378j | - |
| hsa-mir-3917 | HGLibA_59546 | 1 | -1.125648147 | hsa-mir-3917 | - |
| hsa-mir-3920 | HGLibA_59558 | 1 | -1.125648147 | hsa-mir-3920 | - |
| hsa-mir-3923 | HGLibA_59570 | 1 | -1.125648147 | hsa-mir-3923 | - |
| hsa-mir-3926-1 | HGLibA_59576 | 1 | -1.125648147 | hsa-mir-3926-1 | - |
| hsa-mir-3943 | HGLibA_59630 | 1 | -1.125648147 | hsa-mir-3943 | - |
| hsa-mir-3944 | HGLibA_59635 | 1 | -1.125648147 | hsa-mir-3944 | - |
| hsa-mir-3972 | HGLibA_59647 | 1 | -1.125648147 | hsa-mir-3972 | - |
| hsa-mir-3976 | HGLibA_59664 | 1 | -1.125648147 | hsa-mir-3976 | - |
| hsa-mir-4266 | HGLibA_59772 | 1 | -1.125648147 | hsa-mir-4266 | - |
| hsa-mir-4277 | HGLibA_59810 | 1 | -1.125648147 | hsa-mir-4277 | - |
| hsa-mir-4289 | HGLibA_59862 | 1 | -1.125648147 | hsa-mir-4289 | - |
| hsa-mir-4296 | HGLibA_59896 | 1 | -1.125648147 | hsa-mir-4296 | - |
| hsa-mir-4305 | HGLibA_59930 | 1 | -1.125648147 | hsa-mir-4305 | - |
| hsa-mir-432 | HGLibA_59999 | 1 | -1.125648147 | hsa-mir-432 | - |
| hsa-mir-4328 | HGLibA_60036 | 1 | -1.125648147 | hsa-mir-4328 | - |
| hsa-mir-4420 | HGLibA_60067 | 1 | -1.125648147 | hsa-mir-4420 | - |
| hsa-mir-4433b | HGLibA_60121 | 1 | -1.125648147 | hsa-mir-4433b | - |
| hsa-mir-4450 | HGLibA_60206 | 1 | -1.125648147 | hsa-mir-4450 | - |
| hsa-mir-4462 | HGLibA_60256 | 1 | -1.125648147 | hsa-mir-4462 | - |
| hsa-mir-4508 | HGLibA_60457 | 1 | -1.125648147 | hsa-mir-4508 | - |
| hsa-mir-4515 | HGLibA_60504 | 1 | -1.125648147 | hsa-mir-4515 | - |
| hsa-mir-4518 | HGLibA_60516 | 1 | -1.125648147 | hsa-mir-4518 | - |
| hsa-mir-4524b | HGLibA_60559 | 1 | -1.125648147 | hsa-mir-4524b | - |
| hsa-mir-4528 | HGLibA_60573 | 1 | -1.125648147 | hsa-mir-4528 | - |
| hsa-mir-4529 | HGLibA_60574 | 1 | -1.125648147 | hsa-mir-4529 | - |
| hsa-mir-4632 | HGLibA_60635 | 1 | -1.125648147 | hsa-mir-4632 | - |
| hsa-mir-4636 | HGLibA_60650 | 1 | -1.125648147 | hsa-mir-4636 | - |
| hsa-mir-4683 | HGLibA_60841 | 1 | -1.125648147 | hsa-mir-4683 | - |
| hsa-mir-4690 | HGLibA_60872 | 1 | -1.125648147 | hsa-mir-4690 | - |
| hsa-mir-4694 | HGLibA_60883 | 1 | -1.125648147 | hsa-mir-4694 | - |
| hsa-mir-4725 | HGLibA_60997 | 1 | -1.125648147 | hsa-mir-4725 | - |
| hsa-mir-4732 | HGLibA_61025 | 1 | -1.125648147 | hsa-mir-4732 | - |
| hsa-mir-4734 | HGLibA_61032 | 1 | -1.125648147 | hsa-mir-4734 | - |
| hsa-mir-4771-2 | HGLibA_61186 | 1 | -1.125648147 | hsa-mir-4771-2 | - |
| hsa-mir-4771-2 | HGLibA_61183 | 1 | -1.125648147 | hsa-mir-4771-2 | - |
| hsa-mir-4781 | HGLibA_61232 | 1 | -1.125648147 | hsa-mir-4781 | - |
| hsa-mir-4782 | HGLibA_61234 | 1 | -1.125648147 | hsa-mir-4782 | - |
| hsa-mir-4793 | HGLibA_61279 | 1 | -1.125648147 | hsa-mir-4793 | - |
| hsa-mir-4794 | HGLibA_61283 | 1 | -1.125648147 | hsa-mir-4794 | - |

| | | | | | |
|---|---|---|---|---|---|
| hsa-mir-4802 | HGLibA_61311 | 1 | -1.125648147 | hsa-mir-4802 | - |
| hsa-mir-490 | HGLibA_61360 | 1 | -1.125648147 | hsa-mir-490 | - |
| hsa-mir-498 | HGLibA_61393 | 1 | -1.125648147 | hsa-mir-498 | - |
| hsa-mir-5008 | HGLibA_61434 | 1 | -1.125648147 | hsa-mir-5008 | - |
| hsa-mir-5047 | HGLibA_61476 | 1 | -1.125648147 | hsa-mir-5047 | - |
| hsa-mir-513c | HGLibA_61582 | 1 | -1.125648147 | hsa-mir-513c | - |
| hsa-mir-515-2 | HGLibA_61606 | 1 | -1.125648147 | hsa-mir-515-2 | - |
| hsa-mir-516a-1 | HGLibA_61610 | 1 | -1.125648147 | hsa-mir-516a-1 | - |
| hsa-mir-518d | HGLibA_61667 | 1 | -1.125648147 | hsa-mir-518d | - |
| hsa-mir-5190 | HGLibA_61678 | 1 | -1.125648147 | hsa-mir-5190 | - |
| hsa-mir-519d | HGLibA_61726 | 1 | -1.125648147 | hsa-mir-519d | - |
| hsa-mir-519d | HGLibA_61727 | 1 | -1.125648147 | hsa-mir-519d | - |
| hsa-mir-522 | HGLibA_61773 | 1 | -1.125648147 | hsa-mir-522 | - |
| hsa-mir-548ae-1 | HGLibA_61870 | 1 | -1.125648147 | hsa-mir-548ae-1 | - |
| hsa-mir-548ak | HGLibA_61894 | 1 | -1.125648147 | hsa-mir-548ak | - |
| hsa-mir-548b | HGLibA_61953 | 1 | -1.125648147 | hsa-mir-548b | - |
| hsa-mir-548f-5 | HGLibA_61989 | 1 | -1.125648147 | hsa-mir-548f-5 | - |
| hsa-mir-548h-3 | HGLibA_62002 | 1 | -1.125648147 | hsa-mir-548h-3 | - |
| hsa-mir-548i-1 | HGLibA_62014 | 1 | -1.125648147 | hsa-mir-548i-1 | - |
| hsa-mir-548o-2 | HGLibA_62051 | 1 | -1.125648147 | hsa-mir-548o-2 | - |
| hsa-mir-548o-2 | HGLibA_62052 | 1 | -1.125648147 | hsa-mir-548o-2 | - |
| hsa-mir-548s | HGLibA_62064 | 1 | -1.125648147 | hsa-mir-548s | - |
| hsa-mir-550b-1 | HGLibA_62112 | 1 | -1.125648147 | hsa-mir-550b-1 | - |
| hsa-mir-557 | HGLibA_62143 | 1 | -1.125648147 | hsa-mir-557 | - |
| hsa-mir-5586 | HGLibA_62186 | 1 | -1.125648147 | hsa-mir-5586 | - |
| hsa-mir-568 | HGLibA_62232 | 1 | -1.125648147 | hsa-mir-568 | - |
| hsa-mir-5681b | HGLibA_62244 | 1 | -1.125648147 | hsa-mir-5681b | - |
| hsa-mir-5692c-2 | HGLibA_62304 | 1 | -1.125648147 | hsa-mir-5692c-2 | - |
| hsa-mir-5694 | HGLibA_62309 | 1 | -1.125648147 | hsa-mir-5694 | - |
| hsa-mir-5698 | HGLibA_62323 | 1 | -1.125648147 | hsa-mir-5698 | - |
| hsa-mir-5699 | HGLibA_62329 | 1 | -1.125648147 | hsa-mir-5699 | - |
| hsa-mir-5699 | HGLibA_62327 | 1 | -1.125648147 | hsa-mir-5699 | - |
| hsa-mir-572 | HGLibA_62379 | 1 | -1.125648147 | hsa-mir-572 | - |
| hsa-mir-5787 | HGLibA_62410 | 1 | -1.125648147 | hsa-mir-5787 | - |
| hsa-mir-589 | HGLibA_62455 | 1 | -1.125648147 | hsa-mir-589 | - |
| hsa-mir-602 | HGLibA_62500 | 1 | -1.125648147 | hsa-mir-602 | - |
| hsa-mir-6068 | HGLibA_62519 | 1 | -1.125648147 | hsa-mir-6068 | - |
| hsa-mir-6090 | HGLibA_62626 | 1 | -1.125648147 | hsa-mir-6090 | - |
| hsa-mir-6131 | HGLibA_62677 | 1 | -1.125648147 | hsa-mir-6131 | - |
| hsa-mir-614 | HGLibA_62690 | 1 | -1.125648147 | hsa-mir-614 | - |
| hsa-mir-615 | HGLibA_62697 | 1 | -1.125648147 | hsa-mir-615 | - |
| hsa-mir-617 | HGLibA_62708 | 1 | -1.125648147 | hsa-mir-617 | - |
| hsa-mir-618 | HGLibA_62711 | 1 | -1.125648147 | hsa-mir-618 | - |
| hsa-mir-640 | HGLibA_62801 | 1 | -1.125648147 | hsa-mir-640 | - |
| hsa-mir-646 | HGLibA_62827 | 1 | -1.125648147 | hsa-mir-646 | - |
| hsa-mir-649 | HGLibA_62840 | 1 | -1.125648147 | hsa-mir-649 | - |
| hsa-mir-6511a-3 | HGLibA_62905 | 1 | -1.125648147 | hsa-mir-6511a-3 | - |
| hsa-mir-6511a-4 | HGLibA_62908 | 1 | -1.125648147 | hsa-mir-6511a-4 | - |
| hsa-mir-658 | HGLibA_62966 | 1 | -1.125648147 | hsa-mir-658 | - |
| hsa-mir-662 | HGLibA_62983 | 1 | -1.125648147 | hsa-mir-662 | - |
| hsa-mir-6722 | HGLibA_63050 | 1 | -1.125648147 | hsa-mir-6722 | - |
| hsa-mir-6764 | HGLibA_63217 | 1 | -1.125648147 | hsa-mir-6764 | - |
| hsa-mir-6765 | HGLibA_63221 | 1 | -1.125648147 | hsa-mir-6765 | - |
| hsa-mir-6766 | HGLibA_63226 | 1 | -1.125648147 | hsa-mir-6766 | - |
| hsa-mir-6776 | HGLibA_63277 | 1 | -1.125648147 | hsa-mir-6776 | - |
| hsa-mir-6780a | HGLibA_63293 | 1 | -1.125648147 | hsa-mir-6780a | - |
| hsa-mir-6782 | HGLibA_63304 | 1 | -1.125648147 | hsa-mir-6782 | - |
| hsa-mir-6786 | HGLibA_63322 | 1 | -1.125648147 | hsa-mir-6786 | - |
| hsa-mir-6790 | HGLibA_63338 | 1 | -1.125648147 | hsa-mir-6790 | - |
| hsa-mir-6792 | HGLibA_63344 | 1 | -1.125648147 | hsa-mir-6792 | - |
| hsa-mir-6807 | HGLibA_63405 | 1 | -1.125648147 | hsa-mir-6807 | - |
| hsa-mir-6812 | HGLibA_63425 | 1 | -1.125648147 | hsa-mir-6812 | - |
| hsa-mir-6813 | HGLibA_63428 | 1 | -1.125648147 | hsa-mir-6813 | - |
| hsa-mir-6819 | HGLibA_63454 | 1 | -1.125648147 | hsa-mir-6819 | - |
| hsa-mir-6839 | HGLibA_63532 | 1 | -1.125648147 | hsa-mir-6839 | - |
| hsa-mir-6856 | HGLibA_63596 | 1 | -1.125648147 | hsa-mir-6856 | - |
| hsa-mir-6860 | HGLibA_63620 | 1 | -1.125648147 | hsa-mir-6860 | - |
| hsa-mir-6870 | HGLibA_63663 | 1 | -1.125648147 | hsa-mir-6870 | - |
| hsa-mir-6877 | HGLibA_63694 | 1 | -1.125648147 | hsa-mir-6877 | - |
| hsa-mir-6889 | HGLibA_63739 | 1 | -1.125648147 | hsa-mir-6889 | - |
| hsa-mir-6893 | HGLibA_63756 | 1 | -1.125648147 | hsa-mir-6893 | - |
| hsa-mir-7-3 | HGLibA_63775 | 1 | -1.125648147 | hsa-mir-7-3 | - |
| hsa-mir-7515 | HGLibA_63887 | 1 | -1.125648147 | hsa-mir-7515 | - |
| hsa-mir-758 | HGLibA_63893 | 1 | -1.125648147 | hsa-mir-758 | - |
| hsa-mir-759 | HGLibA_63898 | 1 | -1.125648147 | hsa-mir-759 | - |
| hsa-mir-766 | HGLibA_63929 | 1 | -1.125648147 | hsa-mir-766 | - |
| hsa-mir-769 | HGLibA_63937 | 1 | -1.125648147 | hsa-mir-769 | - |
| hsa-mir-770 | HGLibA_63940 | 1 | -1.125648147 | hsa-mir-770 | - |
| hsa-mir-7702 | HGLibA_63943 | 1 | -1.125648147 | hsa-mir-7702 | - |
| hsa-mir-7704 | HGLibA_63952 | 1 | -1.125648147 | hsa-mir-7704 | - |
| hsa-mir-7845 | HGLibA_63968 | 1 | -1.125648147 | hsa-mir-7845 | - |
| hsa-mir-8064 | HGLibA_64093 | 1 | -1.125648147 | hsa-mir-8064 | - |
| hsa-mir-8065 | HGLibA_64097 | 1 | -1.125648147 | hsa-mir-8065 | - |
| hsa-mir-8077 | HGLibA_64149 | 1 | -1.125648147 | hsa-mir-8077 | - |
| hsa-mir-8088 | HGLibA_64195 | 1 | -1.125648147 | hsa-mir-8088 | - |
| hsa-mir-9-1 | HGLibA_64262 | 1 | -1.125648147 | hsa-mir-9-1 | - |
| hsa-mir-9-2 | HGLibA_64268 | 1 | -1.125648147 | hsa-mir-9-2 | - |
| hsa-mir-92a-1 | HGLibA_64291 | 1 | -1.125648147 | hsa-mir-92a-1 | - |
| hsa-mir-934 | HGLibA_64311 | 1 | -1.125648147 | hsa-mir-934 | - |
| hsa-mir-939 | HGLibA_64331 | 1 | -1.125648147 | hsa-mir-939 | - |
| hsa-mir-942 | HGLibA_64353 | 1 | -1.125648147 | hsa-mir-942 | - |
| hsa-mir-98 | HGLibA_64375 | 1 | -1.125648147 | hsa-mir-98 | - |
| ICK | HGLibA_22510 | 1 | -1.125648147 | ICK | - |
| KDELC2 | HGLibA_24477 | 1 | -1.125648147 | KDELC2 | - |
| KIAA0430 | HGLibA_24637 | 1 | -1.125648147 | KIAA0430 | - |
| KIAA1211 | HGLibA_24714 | 1 | -1.125648147 | KIAA1211 | - |
| KIAA1429 | HGLibA_24752 | 1 | -1.125648147 | KIAA1429 | - |
| KIRREL | HGLibA_25000 | 1 | -1.125648147 | KIRREL | - |
| LHFP | HGLibA_26427 | 1 | -1.125648147 | LHFP | - |
| LIMS3L | HGLibA_26556 | 1 | -1.125648147 | LIMS3L | - |
| LINS | HGLibA_26597 | 1 | -1.125648147 | LINS | - |
| LOC440335 | HGLibA_26946 | 1 | -1.125648147 | LOC440335 | - |
| LOC646498 | HGLibA_26974 | 1 | -1.125648147 | LOC646498 | - |
| LOC730159 | HGLibA_27003 | 1 | -1.125648147 | LOC730159 | - |
| METTL21B | HGLibA_28979 | 1 | -1.125648147 | METTL21B | - |
| MINOS1 | HGLibA_29279 | 1 | -1.125648147 | MINOS1 | - |
| NBPF16 | HGLibA_31228 | 1 | -1.125648147 | NBPF16 | - |
| PAPD7 | HGLibA_35126 | 1 | -1.125648147 | PAPD7 | - |
| PCNXL3 | HGLibA_35648 | 1 | -1.125648147 | PCNXL3 | - |
| PHF15 | HGLibA_36347 | 1 | -1.125648147 | PHF15 | - |
| PIDD | HGLibA_36530 | 1 | -1.125648147 | PIDD | - |
| PPAP2C | HGLibA_37823 | 1 | -1.125648147 | PPAP2C | - |
| PPP5D1 | HGLibA_38214 | 1 | -1.125648147 | PPP5D1 | - |
| PTPLB | HGLibA_39425 | 1 | -1.125648147 | PTPLB | - |
| RABL5 | HGLibA_40036 | 1 | -1.125648147 | RABL5 | - |
| RBAK-LOC389458 | HGLibA_40422 | 1 | -1.125648147 | RBAK-LOC389458 | - |
| SELM | HGLibA_43299 | 1 | -1.125648147 | SELM | - |
| SEPN1 | HGLibA_43419 | 1 | -1.125648147 | SEPN1 | - |
| 10-Sep | HGLibA_43428 | 1 | -1.125648147 | 10-Sep | - |
| SEPW1 | HGLibA_43459 | 1 | -1.125648147 | SEPW1 | - |
| SKIV2L2 | HGLibA_44289 | 1 | -1.125648147 | SKIV2L2 | - |
| SLMO2 | HGLibA_45539 | 1 | -1.125648147 | SLMO2 | - |
| SPERT | HGLibA_46623 | 1 | -1.125648147 | SPERT | - |
| SSSCA1 | HGLibA_47167 | 1 | -1.125648147 | SSSCA1 | - |
| SUV420H1 | HGLibA_47864 | 1 | -1.125648147 | SUV420H1 | - |
| TCEB3C | HGLibA_48747 | 1 | -1.125648147 | TCEB3C | - |
| TCEB3CL | HGLibA_48750 | 1 | -1.125648147 | TCEB3CL | - |
| TMEM159 | HGLibA_50018 | 1 | -1.125648147 | TMEM159 | - |
| TMEM48 | HGLibA_50418 | 1 | -1.125648147 | TMEM48 | - |
| UPK3BL | HGLibA_53083 | 1 | -1.125648147 | UPK3BL | - |
| WDR60 | HGLibA_54139 | 1 | -1.125648147 | WDR60 | - |
| WISP3 | HGLibA_54326 | 1 | -1.125648147 | WISP3 | - |
| ZBED1_X | HGLibA_54788 | 1 | -1.125648147 | ZBED1_X | - |
